# Genetic Diversity of *Botrytis cinerea* Revealed by Multilocus Sequencing, and Identification of *B. cinerea* Populations Showing Genetic Isolation and Distinct Host Adaptation

**DOI:** 10.3389/fpls.2021.663027

**Published:** 2021-05-05

**Authors:** Cecilia Plesken, Patrick Pattar, Bianka Reiss, Zinnia Naoshin Noor, Lisha Zhang, Klaus Klug, Bruno Huettel, Matthias Hahn

**Affiliations:** ^1^Department of Biology, University of Kaiserslautern, Kaiserslautern, Germany; ^2^Department of Plant Physiology, RWTH Aachen University, Aachen, Germany; ^3^Center of Plant Molecular Biology, University of Tübingen, Tübingen, Germany; ^4^Max Planck Genome Centre Cologne, Max Planck Institute for Plant Breeding Research, Cologne, Germany

**Keywords:** multilocus sequence typing (MLST), indel, taxonomy, host adaptation, PacBio sequencing, bikaverin, botcinic acid

## Abstract

*Botrytis cinerea* is a world-wide occurring plant pathogen, causing pre- and post-harvest gray mold rot on a large number of fruit, vegetable, and flower crops. *B. cinerea* is closely related to *Botrytis pseudocinerea*, another broad host range species which often occurs in sympatry with *B. cinerea*, and to several host-specific species including *Botrytis fabae* and *Botrytis calthae*. *B. cinerea* populations have been shown to be genetically heterogeneous, and attempts have been made to correlate genetic markers to virulence and host adaptation. Here, we present the development of a multilocus sequence typing (MLST) scheme, with 10 genes selected for high variability and phylogenetic congruence, to evaluate the genetic diversity of *B. cinerea*, *B. fabae*, and *B. pseudocinerea*. Using PacBio-assisted simultaneous mass sequencing of PCR products, MLST analysis of about 100 strains from diverse geographical origins and years of isolation was performed, which resulted in high-resolution strain differentiation and robust species separation. Several *B. cinerea* strains formed an as yet unknown population, referred to as group B, which was well separated from all other *B. cinerea* strains. Furthermore, the gene cluster for biosynthesis of the phytotoxin botcinic acid was missing in *B. cinerea* B strains. *B. cinerea* strains from the monocot *Iris pseudacorus* were found to form a genetically distinct population, and contained an intact gene cluster for production of the red pigment bikaverin, which is usually degenerated in *B. cinerea*. Remarkably, these strains were much more aggressive on *Iris* than other *B. cinerea* strains, which is the first unequivocal example for host specialization in *B. cinerea*. Our data reveal new insights into the genetic diversity of *B. cinerea* and provide evidence for intraspecific differentiation and different degrees of host adaptation of this polyphagous necrotrophic pathogen.

## Introduction

The genus name *Botrytis*, coined by Micheli in 1729, is one of the first described genera of fungi. The major species of this genus is *Botrytis cinerea* Pers.:Fr., a ubiquitous plant pathogen causing gray mold on more than 1000 plant species, including many economically important fruit, vegetable, and ornamental flower crops (Dean et al., 2012; Elad et al., 2016). Up to now, more than 30 species of *Botrytis* have been described, which are all necrotrophic pathogens except for *Botrytis pyriformis* which appears to be a saprophytic species (Hyde et al., 2014; Zhou et al., 2014; Zhang et al., 2016; Richards et al., 2020). A first comprehensive molecular classification of *Botrytis* spp. was performed by Staats et al. (2005), using sequence comparisons of three conserved genes (*RPB2*, *G3PDH*, and *HSP60*). In later studies, *nep1* and *nep2* encoding necrosis and ethylene inducing proteins 1 and 2 have been added to provide higher resolution (Staats et al., 2007; Hyde et al., 2014). Based on the molecular classification, *Botrytis* species can be divided into two phylogenetic clades. *Botrytis* clade 1 includes species that infect mainly or exclusively dicotyledonous plants, namely, the broad host range species *B. cinerea* and *Botrytis pseudocinerea*, and the host-specific species *Botrytis fabae*, *Botrytis calthae*, *Botrytis sinoviticola*, and *Botrytis eucalyptii* (Staats et al., 2005, 2007; Hyde et al., 2014; Liu et al., 2016). The previously described species *Botrytis pelargonii* has been found to be genetically very close or indistinguishable from *B. cinerea* (Staats et al., 2005) and is therefore not included as separate species in this study. *Botrytis* clade 2 is phylogenetically more diverse and contains predominantly monocot-specific, but also several dicot-specific *Botrytis* species, usually with restricted host range (Staats et al., 2007; Hyde et al., 2014).

*Botrytis cinerea* strains show considerable morphological variability, including differences in mycelial growth, conidiation, and sclerotium formation (Kerssies, 1990; Martinez et al., 2003). Numerous studies have also documented a high degree of genetic variability in *B. cinerea* populations (Giraud et al., 1999; Fournier et al., 2003, 2005; Rowe and Kliebenstein, 2007). Early studies performed with *B. cinerea* strains from French vineyards and other host plants based on the analysis of PCR-RFLP patterns, fungicide resistance, and the detection of transposable elements indicated the existence of genetically distinct groups within *B. cinerea* (Giraud et al., 1997, 1999). In particular, the presence or absence of the long terminal repeat retrotransposon Boty (Diolez et al., 1995) and the DNA transposon Flipper (Levis et al., 1997) was used to divide strains into four transposon types called *transposa* (strains having both elements), *vacuma* (strains having neither element), *boty*, and *flipper*. Evidence was obtained that the presence of transposable elements was correlated with higher virulence (Martinez et al., 2005). This was further supported by a seminal study which revealed that *B. cinerea* releases small RNAs derived from retrotransposons that are translocated into host cells and suppress expression of plant defense genes (Weiberg et al., 2013). However, while *B. cinerea* strains differ in their content of transposon types, later studies revealed that this feature was not very useful for genetic classification. Nevertheless, a subgroup of *vacuma* strains, originally called group I, was found to be clearly distinct from all other *B. cinerea* strains (group II) on the basis of genetic markers and DNA sequence data, and later defined as a new species called *B. pseudocinerea* (Fournier et al., 2003, 2005; Walker et al., 2011). *B. pseudocinerea* is often found as a minor species in vineyards and strawberry fields in sympatry with *B. cinerea*, but infects also a variety of other plant species, and has been found occasionally as the dominating gray mold pathogen on oilseed rape, apple, cherry, broad bean, and *Caltha palustris* (Plesken et al., 2015a, b).

The enormous host range of *B. cinerea* and its phenotypic variability would be consistent with a corresponding genetic variability, and at least some degree of host adaptation of the species. Population genetic studies have provided evidence for the existence of clusters within *B. cinerea* populations from different host plants with reduced but still existing gene exchange. Investigations of *B. cinerea* populations in France revealed that they were structured by their host plants tomato and grapevine (Walker et al., 2015). Further studies including pathogenicity tests indicated different degrees of host specialization of the populations, depending on their host origin (Mercier et al., 2019). In German strawberry fields, which are frequently treated with fungicides, *B. cinerea* strains resistant to multiple fungicides have been identified. Several of these strains carried mutations in a transcription factor gene (*mrr1*) leading to increased expression of a drug efflux transporter gene (*atrB*) and partial resistance to two commonly used *Botrytis* fungicides, fludioxonil and cyprodinil (Kretschmer et al., 2009; Leroch et al., 2013). Sequencing of *mrr1* and several other genes indicated that these strains belonged to a new subgroup of *B. cinerea* called *Botrytis* group S (subsequently referred to as *B. cinerea* S), which was separated from the sequenced strains B05.10 and T4 (Leroch et al., 2013). In Germany, *B. cinerea* S strains were found to be predominating in strawberry fields, but almost absent in vineyards. Analysis of *B. cinerea* populations in vineyards of New Zealand revealed the occurrence of strains similar to *B. cinerea* S; however, these studies did not support a clear separation from other *B. cinerea* strains (Johnston et al., 2014).

Multilocus sequence typing (MLST) and multilocus sequence analysis, in the following referred to as MLST, has been established in pathogenic bacteria for studying the epidemiology and phylogenetic relationships of populations (Maiden, 2006). MLST is performed by partial sequencing of coding regions of several genes encoding conserved proteins, for genotyping or deducing phylogenetic relationships. MLST has also been applied for human pathogenic fungi, such as *Candida albicans* and *Cryptococcus* spp. (Odds and Jacobsen, 2008; Alanio et al., 2017). The use of next generation sequencing has been shown to improve the performance and speed of MLST of *Cryptococcus neoformans* (Chen et al., 2015).

The conserved genes *hsp60*, *g3pdh*, and *rpb2* have been used for differentiation between *Botrytis* species (Staats et al., 2005; Hyde et al., 2014). However, they provide poor resolution between *B. cinerea* strains. Higher inter-species variability was observed for *nep1* and *nep2*, but these genes encode host necrosis-inducing proteins and encode some amino acids that appear to be under positive selection (Staats et al., 2007). In this study, we have tested and confirmed the suitability of genes to provide high resolution, phylogenetically reliable differentiation between strains of *B. cinerea* and closely related *Botrytis* species. For this, an MLST scheme with 10 genes was developed and applied for analysis of a large number of *Botrytis* field strains from diverse geographic and host plant origins. The resulting data confirm the genetic diversity of *B. cinerea* and revealed the existence of separated group, called *B. cinerea* B, which has significantly diverged from the majority of *B. cinerea* strains. We also demonstrate that a genetically distinct *B. cinerea* population isolated from the monocot plant *Iris pseudacorus* shows a high degree of host adaptation.

## Materials and Methods

### Cultivation and DNA Isolation of *Botrytis* Strains

*Botrytis* strains were collected in different years from a variety of infected host tissues in different locations and countries. For purification, cultures were transferred several times via hyphal tips or single spores to new malt extract (ME) agar plates. As confirmed by sequencing, all isolates in this study were genetically pure, and can therefore be referred to as strains, consistent with previous reports (Giraud et al., 1999). The strains are listed in Table 1. They were cultivated on ME agar plates under black light bulb illumination to induce rapid sporulation. To induce production of the red pigment bikaverin, *B. cinerea* strains were cultivated in the dark on CD-B agar medium (Schumacher et al., 2013). Growth tests were performed on Gamborg’s B5 agar medium supplemented with 10 mM (NH_4_)H_2_PO_4_ and one of the following carbon sources: 50 mM glucose, 0.5% polygalacturonate, 0.5% pectin, 0.5% xylan, and 0.1% total leaf extract from *I. pseudacorus*.

**TABLE 1:** *Botrytis* strains analyzed by MLST and infection tests.

No.	Name	Species	Year	Host plant	Location	Source/references	Use
1	B05.10	Bcin	1994	Grapevine	Italy	Büttner et al., 1994	IT; fullseq
2	T4	Bcin	2005	Tomato	France, Vaucluse	Fournier et al., 2005	IT; fullseq
3	C12_S_E1_5	Bcin	2012	Strawberry	China, Zhejiang, Shaoxing	Z. Ma	MLST
4	C12_S_E7_2	Bcin	2012	Strawberry	China, Zhejiang, Shaoxing	Z. Ma	MLST
5	C12_S_E7_4	Bcin	2012	Strawberry	China, Zhejiang, Shaoxing	Z. Ma	MLST
6	CH14_ES_11_1	Bcin	2014	Blueberry	Chile	E. Silva	MLST
7	CH14_ES_11_5	Bcin	2014	Blueberry	Chile	E. Silva	MLST
8	D06_1_30	Bcin	2006	Grapevine	GER, Neustadt-Weinstr.	Leroch et al., 2013	MLST
9	D08_H_8_3	Bcin	2008	Strawberry	GER, Vechta	R. Weber	IT; MLST
10	D08_H_8_4	Bcin “S”*	2008	Strawberry	GER, Vechta	Leroch et al., 2013	IT; fullseq
11	D08_H_8_6	Bcin B	2008	Strawberry	GER, Vechta	Leroch et al., 2013	MLST
12	D09_Bc11	Bcin	2009	Strawberry	GER	van Kan et al., 2017	MLST
13	D09_K_2_3	Bcin	2009	Strawberry	GER, Grafschaft-Oeverich	Leroch et al., 2013	MLST
14	D09_K_4_1	Bcin “S”*	2009	Strawberry	GER, Grafschaft-Oeverich	Leroch et al., 2013	MLST
15	D09_K_4_2	Bcin B	2009	Strawberry	GER, Grafschaft-Oeverich	This work	IT; MLST
16	D10_B_F1_6	Bcin “S”*	2010	Strawberry	GER, Breisach, Buchholz	Leroch et al., 2013	MLST
17	D10_B_F3_5	Bcin “S”*	2010	Strawberry	GER, Breisach, Buchholz	Leroch et al., 2013	MLST
18	D10_B_S3_16	Bcin B	2010	Strawberry	GER, Breisach, Rombach	Leroch et al., 2013	IT; fullseq
19	D10_B_S6_1	Bcin B	2010	Strawberry	GER, Breisach, Rombach	This work	MLST
20	D10_K_S11_6	Bcin B	2010	Strawberry	GER, Grafschaft-Oeverich	This work	IT; MLST
21	D10_MR_S19	Bcin	2010	Strawberry	GER, Grafschaft-Oeverich	Rupp et al., 2017	MLST
22	D10_K_S12_13	Bcin	2010	Strawberry	GER, Koblenz, Oeverich	This work	IT
23	D11_H_R3_7	Bcin B	2011	Strawberry	GER, Hamburg	R. Weber	MLST
24	D11_KL_tax4	Bcin	2011	*Taxus* spp.	GER, Kaiserslautern	This work	MLST
25	D11_M_E01	Bcin	2011	Strawberry	GER, Meckenheim	This work	IT; MLST
26	D11_M_E07	Bcin	2011	Strawberry	GER, Meckenheim	This work	IT; MLST
27	D11_M_W04	Bcin	2011	Grapevine	GER, Meckenheim	This work	IT; MLST
28	D11_T_B09	Bcin	2011	Broad bean	GER, Trier	This work	IT; MLST
29	D11_T_B14	Bcin	2011	Broad bean	GER, Trier	This work	IT; MLST
30	D11_T_B25	Bcin B	2011	Broad bean	GER, Trier	This work	IT; MLST
31	D11_T_B45	Bcin	2011	Strawberry	GER, Trier	This work	IT; MLST
32	D11_T_E12	Bcin B	2011	Strawberry	GER, Trier	This work	MLST
33	D11_T_E15	Bcin B	2011	Strawberry	GER, Trier	This work	IT; MLST
34	D11_T_E18	Bcin B	2011	Strawberry	GER, Trier	This work	MLST
35	D11_T_WF43	Bcin	2011	Grapevine	GER, Trier	This work	MLST
36	D11_T_WH08	Bcin	2011	Grapevine	GER, Trier	This work	IT; MLST
37	D12_E_cal13	Bcin	2012	*Caltha palustris*	GER, Kaiserslautern	This work	MLST
38	D12_BH20_4	Bcin	2012	Raspberry	GER, Buxtehude	R. Weber	MLST
39	D12_H_BioH1	Bcin	2012	Raspberry	GER, Buxtehude	R. Weber	MLST
40	D12_Pepper1	Bcin	2012	Pepper	GER	J. Schumacher	MLST
41	D13_MR_S9	Bcin	2013	Strawberry	GER, Bruchsal, Lichtenau	Rupp et al., 2017	MLST
42	D13_B_KF1_25	Bcin	2013	Strawberry	GER, Bruchsal, Sanweier	This work	MLST
43	D13_MR_S11	Bcin	2013	Strawberry	GER, Bruchsal, Hirschberg	Rupp et al., 2017	MLST
44	D13_E_IL12	Bcin I	2013	*Iris pseudacorus*	GER, Kaiserslautern	This work	MLST
45	D13_E_IL4	Bcin I	2013	*Iris pseudacorus*	GER, Kaiserslautern	This work	MLST
46	D13_E_IL7	Bcin I	2013	*Iris pseudacorus*	GER, Kaiserslautern	This work	MLST
47	D13_MR_S2	Bcin	2013	Strawberry	GER, Koblenz-Herold	Rupp et al., 2017	MLST
48	D13_MR_S1	Bcin	2013	Strawberry	GER, Bruchsal, Lichtenau	Rupp et al., 2017	MLST
49	D13_MR_S4	Bcin	2013	Strawberry	GER, Bruchsal, Lichtenau	Rupp et al., 2017	MLST
50	D13_MR_S14	Bcin	2013	Strawberry	GER, Grafschaft-Eckendorf	Rupp et al., 2017	MLST
51	D13_MR_S29	Bcin	2013	Strawberry	GER, Klein Altendorf	Rupp et al., 2017	MLST
52	D14_Heid15	Bcin B	2014	Blueberry	GER, Grethem	This work	MLST
53	D14_Ki11	Bcin	2014	Cherry	GER, Jork	R. Weber	MLST
54	D14_Ki12	Bcin B	2014	Cherry	GER, Jork	R. Weber	MLST
55	G09_S04	Bcin	2009	Strawberry	Greece	Leroch et al., 2013	MLST
56	G09_S33	Bcin “S”*	2009	Strawberry	Greece	Leroch et al., 2013	IT; fullseq
57	G11_MG1_E5	Bcin	2011	Strawberry	Greece, Manolada	G. Karaoglanidis	MLST
58	G11_MG1_E7	Bcin	2011	Strawberry	Greece, Manolada	G. Karaoglanidis	MLST
59	G11_MG1_E22	Bcin	2011	Strawberry	Greece, Manolada	G. Karaoglanidis	MLST
60	G13_EBio4	Bcin	2013	Strawberry	Greece	G. Karaoglanidis	MLST
61	N11_K_W02	Bcin	2011	Grapevine	Norway, Kvelland	This work	MLST
62	N11_K_W03	Bcin B	2011	Grapevine	Norway, Kvelland	This work	MLST
63	N11_K_W06	Bcin B	2011	Grapevine	Norway, Kvelland	This work	IT; MLST
64	N11_K_W08	Bcin	2011	Grapevine	Norway, Kvelland	Leroch et al., 2013	MLST
65	N11_K_W11	Bcin “S”*	2011	Grapevine	Norway, Kvelland	Leroch et al., 2013	IT; MLST
66	N11_K_W14	Bcin	2011	Grapevine	Norway, Kvelland	This work	IT; MLST
67	N11_S_E08	Bcin “S”*	2011	Strawberry	Norway, Søgne	Leroch et al., 2013	IT; MLST
68	N11_S_E09	Bcin B	2011	Strawberry	Norway, Søgne	This work	IT; MLST
69	N11_S_E10	Bcin B	2011	Strawberry	Norway, Søgne	This work	IT; MLST
70	N11_S_E15	Bcin	2011	Strawberry	Norway, Søgne	Leroch et al., 2013	IT; MLST
71	SA12_Ro	Bcin	2012	Rooibush	South Africa	L. Mostert	MLST
72	SAS405	Bcin	<1990	Grapevine	Italy	Faretra et al., 1988	IT; MLST
73	U10_SC_BRO1	Bcin	2010	Strawberry	United States, South Carolina	G. Schnabel	MLST
74	U11_M_E1	Bcin B	2011	Strawberry	United States, North Carolina	G. Schnabel	MLST
75	U11_SC_BR02	Bcin	2011	Strawberry	United States, South Carolina	G. Schnabel	MLST
76	V1750	Bcin	?	Cucumber	Japan	Schumacher et al., 2013	IT
77	2230	*B. fabae*	?	Broad bean	France	A.S. Walker, INRA	IT; MLST
78	2235	*B. fabae*	?	Broad bean	France	A.S. Walker, INRA	IT; MLST
79	2240	*B. fabae*	?	Broad bean	France	A.S. Walker, INRA	IT; MLST
80	11001	*B. fabae*	?	Broad bean	France	A.S. Walker, INRA	IT
81	11002	*B. fabae*	?	Broad bean	France	Leroch et al., 2013	IT; fullseq
82	D12_B_B02	*B. fabae*	2012	Broad bean	GER, Bonn	M. Heupel	MLST
83	D12_B_B28	*B. fabae*	2012	Broad bean	GER, Bonn	M. Heupel	MLST
84	G12	*B. fabae*	2012	Broad bean	Greece	G. Karaoglanidis	IT; fullseq
85	G12_B03B	*B. fabae*	2012	Broad bean	Greece	G. Karaoglanidis	MLST
86	D08_H_8_15	*B. pseud*	2008	Strawberry	GER, Vechta	Leroch et al., 2013	MLST
87	D11_KL_cal2	*B. pseud*	2011	*Caltha palustris*	GER, Kaiserslautern	This work	MLST
88	D11_M_E27	*B. pseud*	2011	Strawberry	GER, Meckenheim	This work	IT; MLST
89	D11_T_B18	*B. pseud*	2011	Broad bean	GER, Trier	This work	IT; MLST
90	D11_T_E01	*B. pseud*	2011	Strawberry	GER, Trier	This work	MLST
91	D12_E_cal10	*B. pseud*	2012	*Caltha palustris*	GER, Kaiserslautern	This work	MLST
92	D13_E_IF4	*B. pseud*	2013	Iris pseudacorus	GER, Kaiserslautern	This work	MLST
93	N11_K_W15	*B. pseud*	2011	Grapevine	Norway, Kvelland	This work	IT; MLST
94	N11_S_E06	*B. pseud*	2011	Strawberry	Norway, Søgne	Leroch et al., 2013	IT; MLST
95	VD110	*B. pseud*	2007	Grapevine	France, Courteron	Walker et al., 2011	IT; fullseq
96	VD184	*B. pseud*	2007	Blackberry	France, Courteron	Walker et al., 2011	IT
97	VD256	*B. pseud*	2007	Grapevine	France, Courteron	Walker et al., 2011	MLST
98	MUCL2830	*B. calthae*	1961	*Caltha palustris*	Canada, Quebec	Leroch et al., 2013	MLST
99	Pae14	*B. paeoniae*	2013	Peony	GER, Wonsheim	This work	MLST
100	GBc_5	*B. sinovitic*.	2010	Grapevine	China, Xinjiang	Zhou et al., 2014	MLST

*Strains marked as Bcin “S” have been described to belong to “*Botrytis* group S,” based on sequencing of *mrr1*, *hsp60*, *fg1020*, *nep2*, and *ms547* (Leroch et al., 2013). ? means unknown.

Extraction of genomic DNA was performed using conidia from sporulating agar plates. Conidia were washed from a sporulating malt agar extract plate with sterile water, filtered through glass wool, centrifuged, and suspended in water at a concentration of 1–5 × 10^7^ conidia ml^–1^; 400 μl of the spore suspension was mixed with 200 μl CTAB solution (per 100 ml: 2.5 g sorbitol; 1 g N-laurylsarcosine; 0.8 g *N*-cetyl-trimethylammonium bromide; 4.7 g NaCl; 1 g Na-EDTA; and 1 g polyvinylpyrrolidone) in a 2 ml screw-capped microfuge tube and homogenized with glass beads (1 mm diameter) with FastPrep (MP Biomedicals Germany GmbH, Eschwege, Germany; 45 and 30 s at level 6, interrupted by cooling on ice). After centrifugation (14,000 rpm, 2 min), the clear supernatant was transferred into a fresh tube, and 100 μl chloroform was added. After vortexing for 5 s and centrifugation (14,000 rpm, 3 min), the upper phase was transferred into a new tube, and the DNA precipitated by the addition of an equal volume of 2-propanol. After centrifugation (14,000 rpm, 5 min), the DNA pellet was washed with 70% ethanol, air-dried, and dissolved in 15–30 μl TE buffer. Concentration and integrity of the DNA were estimated by agarose gel electrophoresis.

### PCR Assays

For genetic analysis of *Botrytis* strains, ca. 10 ng of total Botrytis DNA was used for PCR assays with diagnostic primers, using 20 μl reactions containing 0.2 mM dNTPs, 0.25 μM primers, 40 mM Tris–HCl, 0.02% Tween 20, 2 mM MgCl2, pH 8.3, and 0.5 units Taq polymerase. Amplification was performed in different thermocyclers set at 5 min 94°C, followed by 30–35 cycles with 30 s 94°C, 30 s 52–60°C, and 1 min per kb 72°C. Electrophoresis was performed with 1 × TAE buffer in 1–3% agarose gels. Annealing temperatures were adjusted to ca. 5°C below the melting temperatures of the primers. Primers used in this study are listed in Supplementary Table 1.

### Selection of Genes for Multilocus Sequence Typing

To identify genes suitable for MLST of *B. cinerea* and related *Botrytis* species, we first searched for genes in FUNYBASE, a database containing 246 families of single-copy orthologs obtained from 21 fungal genomes (Marthey, 2008^1^). Two of the most variable FUNYBASE genes, *ms547* (Bcin12g03020) and *fg1020* (Bcin04g02090), were selected. These have been used previously for differentiation of *B. pseudocinerea* and *B. cinerea* (Walker et al., 2011), and between *B. cinerea* strains (Leroch et al., 2013). No other FUNYBASE gene was found to be suitable for MLST, because of low diversity between the four tested *Botrytis* spp. and/or between *B. cinerea* strains. For identification of further genes useful for differentiation, genome sequences from six *B. cinerea* strains and one strain each of *B. fabae*, *B. pseudocinerea*, and *B. calthae* (unpublished data) were reference aligned to the sequenced genome of *B. cinerea* strain B05.10^2^, and searched for complete homologous gene models. Genes yielding the highest diversity scores between the four *Botrytis* species were tested for high resolution among the sequenced *B. cinerea* strains, conservation in other ascomycetes (preferred), predicted function (preferred), and location on different chromosomes in the *B. cinerea* B05.10 reference genome. Based on these criteria, a final list of 10 genes, including the previously used *nep2*, *ms547*, and *fg1020*, was selected for MLST (Table 2). Primers for these genes were designed to match the sequences of *B. cinerea*, *B. fabae*, *B. pseudocinerea*, and *B. calthae*; therefore they were partly degenerated, containing mixtures of variable nucleotides. For *B. calthae*, sequence comparisons of several strains (Plesken et al., 2015b) confirmed species-specific similarities for *nep2* and *ms547*, and the suitability of the primers. Primer positions were chosen to obtain genomic PCR fragments with heterogeneous size, to facilitate their differentiation on the gels.

**TABLE 2:** Genes used for MLST of *Botrytis* isolates.

Code	Gene ID (name)*	Predicted function	CDS size (bp)	Fragment sizes (bp)	
				First PCR	Second PCR
MLST1	Bcin01g07220	ATP pheromone transporter	4551	1258	1350
MLST2	Bcin05g07690	Glycoside hydrolase	1769	1100	1192
MLST3	Bcin06g01710	Aromatic amino acid decarboxylase	1695	1019	1111
MLST4*	Bcin09g03030 (*Bcdpb2*)	DNA polymerase ε subunit B	3347	1011	1103
MLST5	Bcin11g01310	Protease	1701	921	1013
MLST6	Bcin15g03910	Palmitoyl protein thioesterase	1148	971	1063
MLST7	Bcin16g03460	2OG-Fe(II) oxygenase	968	833	925
MLST8*	Bcin12g03020 (*ms547/Bcdbp7*)	ATP-dependent RNA helicase	2634	935	1027
MLST9*	Bcin02g07770 (*Bcnep2*)	Necrosis-inducing protein 2	845	842	934
MLST10*	Bcin04g02090 (*fg1020/Bcufd2*)	Ubiquitin fusion degradation protein	3788	910	1002

*Gene names were taken from http://fungi.ensembl.org/Botrytis_cinerea/, and from Marthey (2008).

Indel-PCR has been used for preliminary identification of *Botrytis* species: A 122 bp deletion upstream of the coding region in the *B. fabae* homolog of *B. cinerea* strain B05.10 gene Bcin13g02260 (Rigotti et al., 2002), a 24 bp deletion in the *B. pseudocinerea* homolog of Bcin09p02270 (Plesken et al., 2015a), and a 9 bp deletion in the *B. calthae* homolog of Bcin16g02210 (Plesken et al., 2015b). Furthermore, an 18 bpand a 21 bp indel in the *mrr1* coding region showing variable configuration in different *B. cinerea* isolates (Leroch et al., 2013) allowed differentiation within *B. cinerea* (cf. Table 4).

### Preparation of DNA for Mass Sequencing

A two-step PCR was performed, following the protocol “Procedure & Checklist -Preparing SMRTbell^TM^ Libraries using PacBio^®^ Barcoded Universal Primers for Multiplex SMRT^®^ Sequencing,” with modifications. Instead of Phusion HF polymerase, a self-prepared Taq polymerase was used. PCR conditions were as follows: The first-round PCR with gene-specific primers attached to universal forward and reverse primer sequences (30 bp each) was performed in 20 μl reactions, using ca. 1 ng of *Botrytis* DNA, 0.2 mM dNTPs, 0.25 μM primers, 1 μl Taq polymerase, and 1 × Taq buffer (20 mM Tris–HCl, pH 8.5, 16 mM ammonium sulfate, 0.01% Tween 20, and 2 mM MgCl_2_). PCR was performed with 5 min initial denaturation at 94°C, 22 cycles of 30 s at 94°C, 30 s at 55°C, and 80 s at 72°C, and a final 5 min at 72°C. Products from the first-round PCR were checked for their sizes and amounts on an agarose gel, diluted 1:200, and used as templates for the second-round PCR using barcoding primers (for each strain, a unique pair of 16 bp barcodes 5’-attached to universal forward and reverse primers) in 25 μl with 0.25 mM dNTPs, 0.3 μM primers, 1 μl Taq polymerase, 1 × Taq buffer, and 1 μl first-round PCR product, with the same thermocycling conditions.

Concentrations of the final PCR products were estimated by their gel staining intensities, and from 100 strains, 10 PCR fragments per strain (representing MLST1-10 amplicons) mixed to yield a final concentration of 1 μg ml^–1^ for each fragment. A total of 1000 fragments were sequenced in one PacBio RSII SMRT cell according to the supplier’s protocol, yielding an average sequence depth of 32x. Reads of inserts were extracted and aligned to obtain the consensus sequences.

Sequence data are found in Supplementary File 1 (*Botrytis mrr1* sequence data) and Supplementary File 2 (*Botrytis* MLST1-10 sequence data).

### Infection Tests

Infection tests were performed with detached leaves of tomato (*Lycopersicon esculentum*), faba bean (*Vicia faba*), Phaseolus bean (*Phaseolus vulgaris*), salad (*Lactuca sativa*), barley (*Hordeum vulgaris*), yellow iris (*I. pseudacorus*), placed on wet filter paper, and with apple fruit (cv. Golden Delicious). Inoculations were performed with 20 μl conidial suspensions containing 2 × 10^5^ conidia ml^–1^ in Gamborg GB5 medium supplemented with 25 mM glucose. Inoculation of *Iris* leaves was performed either with agar plugs of 1 mm thickness and 2 mm diameter inoculated 1 day before with 2000 conidia, or with conidial suspensions as described above, after wiping away cuticular waxes of the *Iris* leaves with wet gloved fingers to allow more consistent infection. Nevertheless, infection by the *B. cinerea* strains varied to some extent with the age and size of the *Iris* leaves used. Apple fruits were inoculated by pipetting 20 μl conidial suspensions into wounds created by a cork borer with 2 mm diameter. After incubation of inoculated leaves and fruits in humid chambers at ambient light and temperature conditions for the indicated times, lesion diameters were measured with a digital caliper. Mean values of at least replicates are shown in the figures, with standard deviations.

### Tree Building and Statistical Data Evaluation

Sequence alignments and tree building were performed by PhyML for maximum-likelihood phylogenetic tree reconstruction, using SeaView version 4 software (Gouy et al., 2010). Distance-based tree reconstruction was performed by BioNJ or by maximum-parsimony, which gave similar results for all trees generated. Statistical analyses were performed using the GraphPad Prism software. The detailed analysis method is depicted in the individual figure legends. Infection and growth experiments were carried out at least three times, with two or three technical replicates per sample.

## Results

### Use of Indels in *mrr1* for Preliminary Classification of *B. cinerea* Strains

Previously, genetic analysis of *B. cinerea* strains from fungicide-treated strawberry fields has revealed a high polymorphism of the *mrr1* gene, which showed up to 5% sequence divergence between the sequenced *B. cinerea* reference strains and several strains resistant to multiple fungicides from German strawberry fields. Further sequencing of the genes *fg1020*, *ms547*, and *nep2* supported a genetic separation between this strawberry population originally referred to as “group S” and isolates similar to reference strains B05.10 and T4 (Leroch et al., 2013). Because of the high variability of *mrr1*, a comparative sequence analysis was performed. An alignment of ca. 2030 bp *mrr1* segment covering the coding region was done with 26 *B. cinerea* strains, two *B. fabae* strains, four *B. pseudocinerea* strains, and one *B. calthae* strain. Several insertions-deletions (indels) are present in *mrr1*, including an 18 bp indel and a 21 bp indel in the coding region, which are useful for preliminary characterization of *Botrytis* isolates (Leroch et al., 2013). The dendrogram for *mrr1* revealed a division of strains of *B. cinerea* and other *Botrytis* species into several distinct groups, but did not show their correct phylogenetic relationships, as several *B. cinerea* strains grouped closer to other *Botrytis* species than to other *B. cinerea* strains (Supplementary Figure 1). It was therefore evident that the high diversity of *mrr1* is useful for preliminary assessment of genetic variability in *Botrytis*, but not for estimation of phylogenetic relationships.

### Identification of Genes Suitable for MLST-Based Identification and Phylogenetic Placement of *Botrytis* Strains

To establish a reliable scheme for multilocus sequencing (MLST) of *B. cinerea* and closely related (clade 1) *Botrytis* species, 10 genes were selected which provided high-resolution differentiation and phylogenetic information of the strains analyzed (see section “Materials and Methods”; Table 2). Inter-strain variability of the MLST genes was three- to fourfold higher compared to *hsp60*, and a total of 10.5% of their sequences contained variable sites (Supplementary Table 2).

The 10 concatenated MLST sequences were aligned and used for tree building. This procedure was used for differentiation of 95 *Botrytis* strains (74 *B. cinerea*, eight *B. fabae*, 10 *B. pseudocinerea*, and one each of *B. calthae*, *B. sinoviticola*, and *Botrytis paeoniae*). The *B. cinerea* strains were obtained from 12 plant species, 10 countries, and 11 different years of sampling (Table 1). Eight *B. cinerea* strains from German strawberry fields showing multiple resistance to seven major anti-*Botrytis* fungicide classes (Rupp et al., 2017) were included into the analysis. For comparison, sequences were included from eight strains of *B. cinerea* and one strain each of *B. fabae*, *B. pseudocinerea*, and *B. calthae* for which genome sequences were available.

**FIGURE 1:**
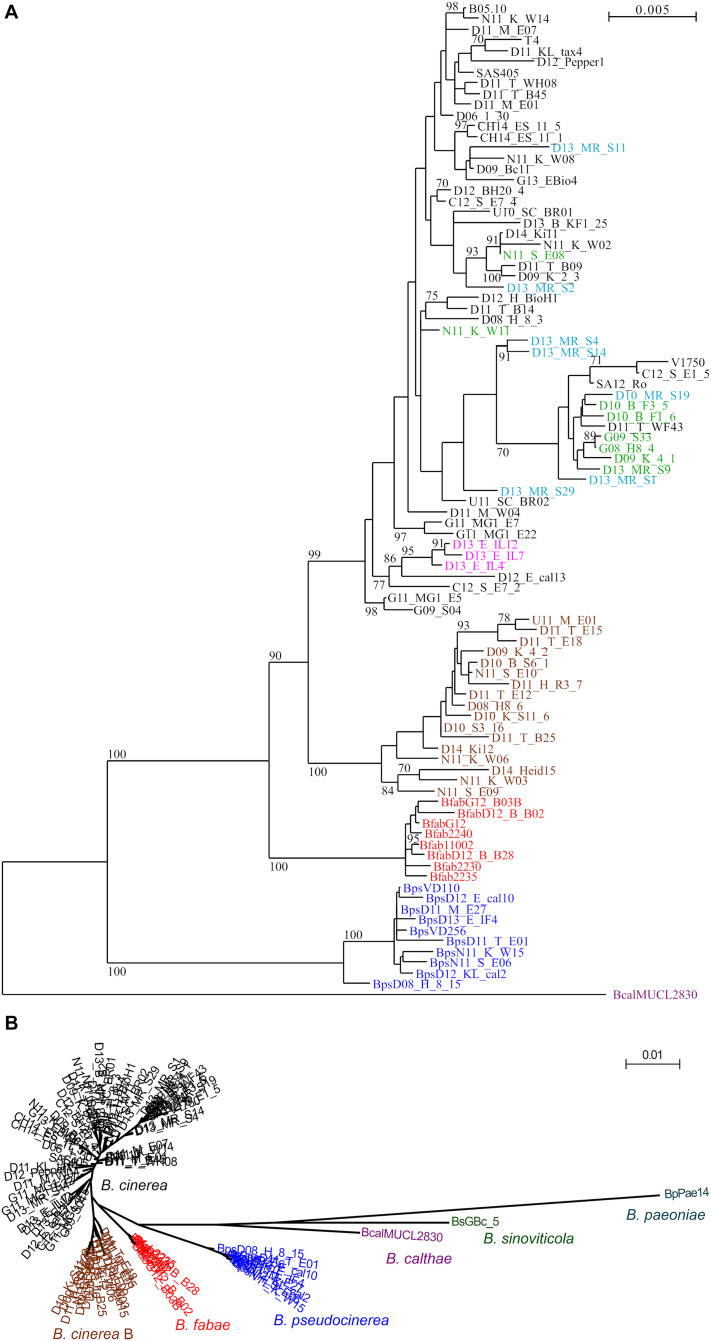
**(A)** PhyML-generated tree of 93 *Botrytis* strains, based on concatenated sequences of genes MLST1–MLST10, with 9524 nucleotides compared. Bootstrap values > 70 based on 1000 replicates are shown. Bcal (purple): *B. calthae*; Bps (blue): *B. pseudocinerea*; Bfab (red): *B. fabae*. *B. cinerea* group B (brown); *B. cinerea Iris* isolates (pink). *B. cinerea* strawberry isolates previously described as “group S” (Leroch et al., 2013) are shown in green, and *B. cinerea* strawberry isolates with multiple fungicide resistances are shown in light blue. **(B)** Circular PhyML tree including *B. paeoniae* and *B. sinoviticola*, illustrating genetic separation of all species, and of *B. cinerea* group B from other *B. cinerea* strains.

PhyML trees generated with all strains clearly supported the expected separation of the clade 1 species *B. sinoviticola*, *B. calthae*, *B. pseudocinerea*, *B. fabae*, and *B. cinerea*, and the larger phylogenetic distance of the clade 2 species *B. paeoniae* (Figures 1A,B). *B. cinerea* strains were found to form more or less distinct clusters. Most interestingly, strains in one cluster, in the following referred to as *B. cinerea* (group) B, were clearly separated from all other *B. cinerea* strains. The separation of *B. cinerea* B strains from the remaining *B. cinerea* strains was also observed with the trees generated with most of the individual MLST sequences (Supplementary Figure 2). Strains previously identified as *B. cinerea* group S, which have been found to be dominating in German strawberry fields (Leroch et al., 2013), were found to be partly clustered within the *B. cinerea* branches (Figure 1). They were found to include five or six of the seven strawberry strains with resistance to multiple fungicides, but also strains isolated in Greece, South Africa, United States, China, and Japan, several of them being collected from other host species. The two sequenced strains B05.10 and T4, which have been widely used for phenotypic and genetic studies, clustered relatively close to each other, despite their known sequence diversity and different contents of transposable elements (Amselem et al., 2011).

### *B. cinerea* B Strains Lack Biosynthesis Gene Cluster for the Phytotoxin Botcinic Acid

**FIGURE 2:**
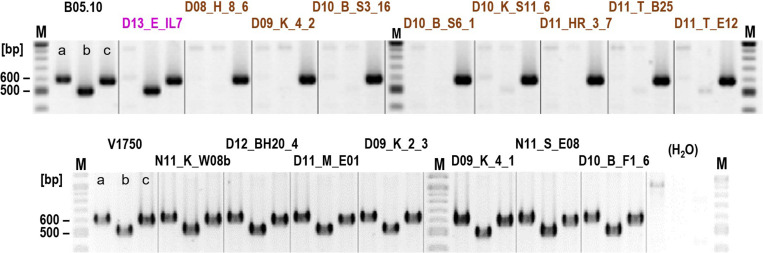
Detection of genes involved in the synthesis of botrydial and botcinic acid in *B. cinerea* strains. Agarose gel-separated PCR products for *boa17* (lanes a), *boa6* (lanes b), and *bot2* (lanes c) are shown. *B. cinerea* B strains are marked in brown and the *Iris* isolate in pink.

*Botrytis cinerea* produces two phytotoxins, the sesquiterpenoid botrydial (BOT) and the polyketide botcinic acid (BOA), which have been shown to be required together for full virulence (Dalmais et al., 2011). We tested the strains for the presence of *bot* and *boa* genes by PCR. Whereas the *bot2* gene was detected in all strains, both *boa6* (encoding a polyketide synthase) and *boa17* (the last gene of the *boa* cluster) were missing in all 16 tested *B. cinerea* B strains (Figure 2). In contrast, all other tested *B. cinerea* strains contained *boa6*, and most strains also contained *boa17*, which might not to be required for botcinic acid biosynthesis (Porquier et al., 2019). These data were consistent with genome sequence data from strain D10_S3_16, which revealed the complete absence of the *boa* cluster (not shown). Therefore, *B. cinerea* B strains apparently have lost the ability to produce botcinic acid.

### Infection Tests Reveal Different Degrees of Host Specificity of *Botrytis* Clade I Species

**FIGURE 3:**
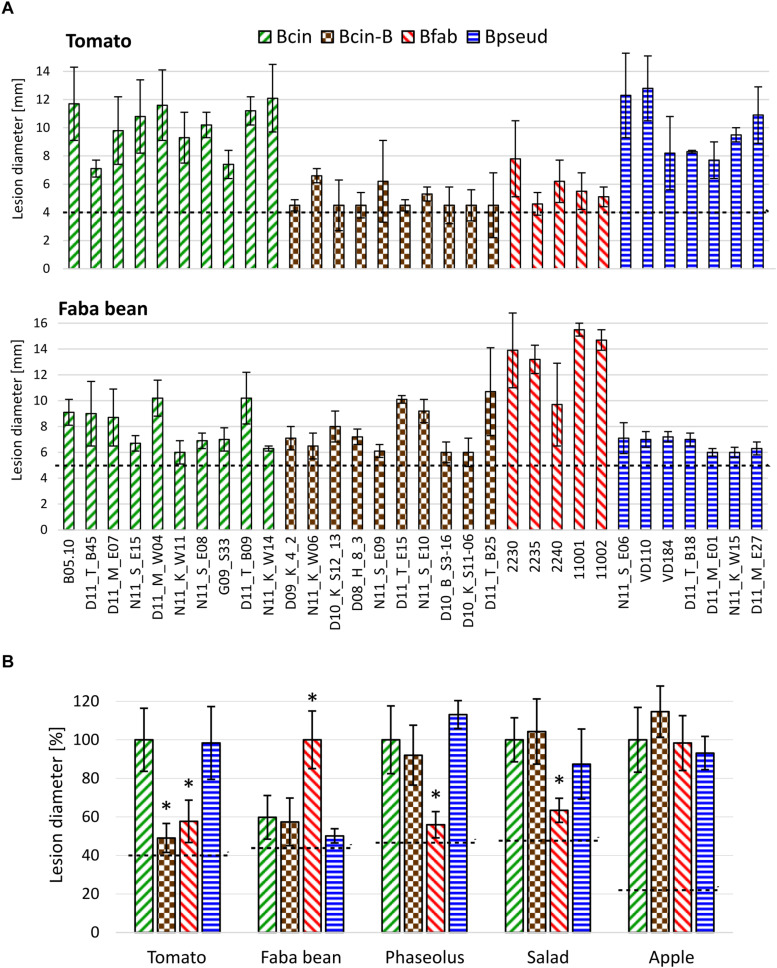
Lesion formation by strains of *B. cinerea*, *B. cinerea* B, *B. fabae*, and *B. pseudocinerea* on tissues of different plants. **(A)** Lesions caused by individual isolates on tomato and faba bean leaves. **(B)** Mean lesion sizes of strains (shown in **A** and Supplementary Figure 3) from different *Botrytis* taxa on five different plant tissues, indicated in percent lesion size produced by *B. cinerea* (for faba bean: *B. fabae*). The dotted lines indicate the average sizes of the inoculation droplets. The *p*-values by one-way ANOVA followed by Dunnett’s multiple comparisons *post hoc* test (control: *B. cinerea*) are indicated. *p < 0.001.

To compare virulence and host specificity of *B. cinerea*, *B. cinerea* B, *B. fabae*, and *B. pseudocinerea*, infection tests were performed with strains of each of these taxa on tissues of five plant species. All strains were able to form primary lesions under the inoculation droplets, but differences were observed in the size of expanding lesions (Figure 3 and Supplementary Figure 3). Overall, *B. cinerea* and *B. pseudocinerea* strains showed similar infection behavior, except for smaller lesions caused by *B. pseudocinerea* on faba bean. As expected, *B. fabae* was most aggressive on its host faba bean, but induced only small expanding lesions on leaves of tomato, *Phaseolus* bean, and salad. *B. cinerea* B strains formed significantly smaller lesions on tomato leaves compared to common *B. cinerea* strains. No significant differences were observed between *B. cinerea* and *B. cinerea* B in lesion formation on apple fruit.

### Discovery of Pink *B. cinerea* Strains From the Monocot *Iris pseudacorus*

In 2012 and 2013, five and 19 Botrytis strains, respectively, were recovered from flowers and leaves of yellow iris (*I. pseudacorus*) plants grown at two adjacent sites along a creek in the Palatine Forest in Germany. The strains showed growth characteristics and sporulation typical for *B. cinerea*. *B. convoluta*, a pathogen specific to *Iris* spp. (Whetzel and Drayton, 1932), has been reported to cause mainly rhizome infections. We did not observe infections on the rhizomes of the *Iris* plants, and were unable to obtain *Botrytis* isolates from them. DNA analysis confirmed the identity of the strains as *B. cinerea* or *B. pseudocinerea*. All isolates from 2012, and 17 isolates from 2013 were found to contain a 21 bp insertion, consistent with their initial classification as “group S” according to Leroch et al. (2013). Two isolates, obtained from flowers, were identified as *B. pseudocinerea*, based on a 24 bp deletion in Bcin09p02270 (Plesken et al., 2015a), which was confirmed for strain Bps_D12_E_IF4 by sequencing (Figure 1). With *mrr1* and concatenated MLST1-10 genes, four *B. cinerea Iris* strains were found to form a cluster within *B. cinerea* (Supplementary Figure 1). Based on the *mrr1* sequence of these strains, a primer-induced restriction analysis (PIRA-PCR) was designed (Haliassos et al., 1989), which resulted in a 72 bp PCR fragment which could be cleaved with the restriction enzyme *Acc*I with DNA of all of the *B. cinerea Iris* strains, but not with DNA from any other *B. cinerea* strains tested. Furthermore, a 6 bp indel in Bcin01g05500 was identified (see below), which was in the deleted state only in the *B. cinerea Iris* population (Table 3).

**TABLE 3:** Genetic and phenotypic differentiation of *B. cinerea Iris* strains.

Isolate	Mycel. color	*mrr1* indel	*mrr1* PIRA PCR*	Bcin01g05500 indel	Bikaverin genes				Mating type	Haplotype**
					*bcbik1*	*bcbik2*	*bcbik3*	*bcbik5*		
PCR size (bp)	–	149/128	76	85/79	679	711	1278	797	–	–
D12_E_IL1	Pink	ins	62 + 14 bp	del	yes	n.a.	n.a.	n.a.	MAT1-1	1
D12_E_IL2	Pink	ins	62 + 14 bp	del	yes	n.a.	n.a.	n.a.	MAT1-1	1
D12_E_IL4^&^	Pink	ins	62 + 14 bp	del	yes	n.a.	n.a.	n.a.	MAT1-2	5
D12_E_IL6^&^	Pink	ins	62 + 14 bp	del	yes	n.a.	n.a.	n.a.	MAT1-1	1
D12_E_IL9	Pink	ins	62 + 14 bp	del	yes	yes	yes	yes	MAT1-2	2
D13_E_IF1	Pink	ins	62 + 14 bp	del	yes	n.a.	n.a.	n.a.	MAT1-1	1
D13_E_IF5	Pink	ins	62 + 14 bp	del	yes	n.a.	n.a.	n.a.	MAT1-2	2
D13_E_IF8	Pink	ins	62 + 14 bp	del	yes	n.a.	n.a.	n.a.	MAT1-1	1
D13_E_IL1	Pink	ins	62 + 14 bp	del	yes	n.a.	n.a.	n.a.	MAT1-2	2
D13_E_IL2	Pink	ins	62 + 14 bp	del	yes	n.a.	n.a.	n.a.	MAT1-1	4
D13_E_IL3	Pink	ins	62 + 14 bp	del	yes	n.a.	n.a.	n.a.	MAT1-2	2
D13_E_IL4^$^	Gray	ins	62 + 14 bp	del	no	no	no	no	MAT1-2	3
D13_E_IL5	Pink	ins	62 + 14 bp	del	yes	n.a.	n.a.	n.a.	MAT1-2	2
D13_E_IL6	Pink	ins	62 + 14 bp	del	yes	n.a.	n.a.	n.a.	MAT1-2	2
D13_E_IL7^$^	Pink	ins	62 + 14 bp	del	yes	yes	yes	yes	MAT1-2	2
D13_E_IL8	Pink	ins	62 + 14 bp	del	yes	n.a.	n.a.	n.a.	MAT1-2	2
D13_E_IL9	Pink	ins	62 + 14 bp	del	yes	n.a.	n.a.	n.a.	MAT1-2	2
D13_E_IL1	Pink	ins	62 + 14 bp	del	yes	n.a.	n.a.	n.a.	MAT1-2	2
D13_E_IL11	Pink	ins	62 + 14 bp	del	yes	n.a.	n.a.	n.a.	MAT1-1	1
D13_E_IL12^$,&^	Pink	ins	62 + 14 bp	del	yes	yes	yes	yes	MAT1-2	5
D13_E_IL13	Pink	ins	62 + 14 bp	del	yes	n.a.	n.a.	n.a.	MAT1-2	2
BpsD13_E_IF2	Gray	del	No product	ins	n.a.	n.a.	n.a.	n.a.	n.a.	–
BpsD13_E_IF4	Gray	del	No product	ins	n.a.	n.a.	n.a.	n.a.	n.a.	–
V1750	Pink	ins	76 bp	ins	yes	yes	yes	yes	n.a.	–
B05.10	Gray	del	76 bp	ins	no	yes	yes	yes	MAT1-1	–
*B. cinerea*^®^	Gray	ins/del	76 bp	ins	no	n.a.	n.a.	n.a.	n.a.	–
*B. fabae* G12	Gray	del	76 bp	ins	no	n.a.	n.a.	n.a.	n.a.	–
*B. pseud* VD110	Gray	del	No product	ins	no	n.a.	n.a.	n.a.	n.a.	–

*Products after digestion with *Acc*I. **Based on data in the table and MLST or *mrr1* sequencing data. ^§^ Randomly chosen *B. cinerea* non-*Iris* isolates (*n* = 20). ^$^Used for MLST sequencing. ^&^Used for *mrr1* sequencing. n.a.: not analyzed.

**FIGURE 4:**
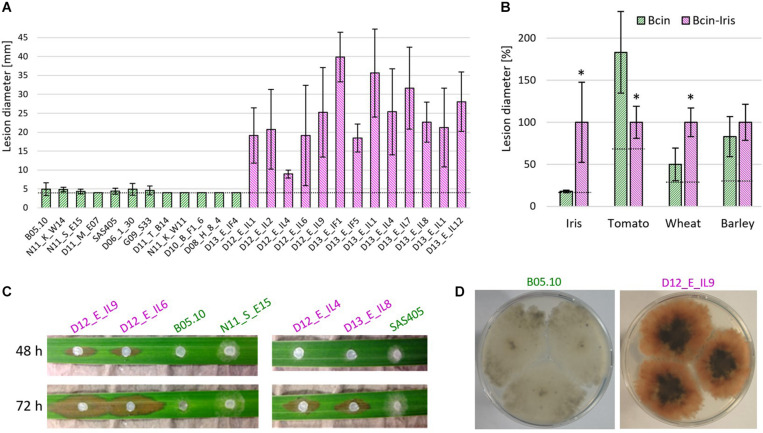
*B. cinerea Iris* strains are highly aggressive on *Iris pseudacorus* leaves and produce the red pigment bikaverin. **(A)** Lesion development on detached leaves, 5 days after inoculation with 12 *B. cinerea* and 13 *B. cinerea Iris* strains. **(B)** Mean lesion sizes induced by *B. cinerea* (green) and *B. cinerea Iris* (pink; shown in **A** and Supplementary Figure 4) on leaves of different plant species. The dotted lines indicate the average sizes of the inoculation droplets. Values for *B. cinerea* and *B. cinerea Iris* were compared by unpaired *t*-test. *p < 0.001. n.s.: not significant. **(C)** Symptoms on *I. pseudacorus* leaves induced by different *B. cinerea* strains. **(D)** Pictures of colonies of gray *B. cinerea* (left) and pink *B. cinerea Iris* (right) strains.

Remarkably, all but one of the 22 *B. cinerea Iris* strains revealed a pink color when cultured on ME agar (Figure 4D). The pink color has been observed previously in rare *B. cinerea* strains, and is caused by the production of the red pigment bikaverin. Bikaverin is produced by several *Fusarium* spp. and encoded by a cluster containing six genes including a polyketide synthase. Bikaverin producing *B. cinerea* strains have gained a complete, functional ortholog of this cluster by horizontal gene transfer (Campbell et al., 2013; Schumacher et al., 2013). Gray *B. cinerea* strains contain a degenerated gene cluster lacking *Bcbik1*, the gene for the polyketide synthase. The presence of *bcbik1* in the *B. cinerea Iris* population was confirmed by PCR with all 20 pink strains, but not for the two gray *Iris* strains. For three pink *Iris* strains and the pink control strain, V1750 (Schumacher et al., 2013), the presence of *bcbik2, bcbik3*, and *bcbik5* was confirmed by PCR. Consistent with the genome sequence, B05.10 DNA also revealed the presence of *bcbik2, bcbik3*, and *bcbik5*, whereas in the gray *Iris* strain D13_E_IL04, the four *bcbik* genes appeared to be missing (Table 3). Analysis of the mating type genes revealed the presence of both MAT1-1 and MAT1-2 genotypes, indicating that even in this small population, sexual reproduction might still occur (Table 3).

Taken together, these data demonstrate that the pink *Iris* strains, and the gray strain D13_E_IL4, represent a genetically distinct *B. cinerea* population, in the following referred to as *B. cinerea Iris*. Based on the ability to produce bikaverin, mating type, and MLST sequences, five haplotypes were distinguished among the *Iris* strains.

### *B. cinerea Iris* Strains Are Highly Aggressive on *I. pseudacorus* Leaves

Infection tests on detached leaves of different plants were performed. All of the *Iris* strains were highly aggressive on *I. pseudacorus* leaves, causing expanding lesions 5 days after inoculation. In contrast, strain B05.10 and several other *B. cinerea* strains usually developed only primary lesions, which sometimes expanded to a small extent (Figures 4A–C). Compared to common *B. cinerea* strains, the *Iris* strains showed significantly reduced lesion formation on tomato leaves. In contrast, virulence on wheat leaves was higher for the *Iris* strains than for the other *B. cinerea* strains, while no significant differences in lesion formation were observed on barley (Figure 4B and Supplementary Figure 4). Because monocot and dicot plants differ in the pectin content of their cell walls, we reasoned that the difference in virulence could be related to their cell wall degrading ability. When the growth of the isolates was tested on media containing different carbon sources, however, no significant differences were observed between two *B. cinerea* reference strains and four *Iris* strains (Supplementary Figure 5).

### Identification of New Indels for PCR-Based Identification of *B. cinerea Iris* and Group B Strains

Indel-PCR has been used for preliminary identification of *Botrytis* species, e.g., *B. fabae* and *B. pseudocinerea* (Rigotti et al., 2002; Plesken et al., 2015a). By performing blastn searches with codon sequences of predicted genes in published and self-generated genome assemblies of *B. cinerea*, *B. fabae*, *B. pseudocinerea*, and *B. calthae*, two more useful indels were identified, and flanking primers were designed which amplified the DNA from all tested clade 1 *Botrytis* species. A 6 bp indel in Bcin01g05500 was suitable for identification of *B. cinerea Iris* strains, and a 15 bp indel in Bcin11g00620 allowed identification of *B. cinerea* group B strains. The location of the indels, their specificity, and the primers used are summarized in Table 4.

**TABLE 4:** List of primers used for PCR-based differentiation of *Botrytis* species and genotypes of *B. cinerea*, depending on the insertion (ins) or deletion (del) state of diagnostic indels.

Primers	Bcal9-fw	Bps24-fw	Bfab122-fw	mrr1-18-fw	BcB15-fw	mrr1-21-fw	BcIris6-fw
	**Bcal9-rv**	**Bps24-rv**	**Bfab122-rv**	**mrr1-18-rv**	**BcB15-rv**	**mrr1-21-rv**	**BcIris6-rv**

Gene ID	Bcin16g02210	Bcin09g02270	Bcin13g02260	Bcin05g01790	Bcin11g00620	Bcin05g01790	Bcin01g05500
Indel size	9 bp	24 bp	122 bp	18 b	15 bp	21 bp	6 bp
PCR size	64/55 bp	136/112 bp	355/233 bp	200/182 bp	99/84 bp	149/128 bp	85/79 bp
*B. cin*	del	ins	ins	ins/del	ins	ins/del	ins
*B. cin B*	del	ins	ins	ins	del (39)*	ins/del	ins
*B. cin Iris*	del	ins	ins	ins	ins	ins	del (21)*
*B. fabae*	del	ins	del (31)*	ins	ins	ins	ins
*B. pseud*	del	del (69)*	ins	ins	ins	del	ins
*B. calthae*	ins (17)*	ins	ins	ins	ins	ins	ins

*Text in bold, indicating the diagnostic state of the Botrytis species or B. cinerea group to be identified. In parentheses: Number of isolates tested.*

## Discussion

In this study, the genetic diversity of a large number of *B. cinerea* strains and smaller collections of *B. pseudocinerea* and *B. fabae* was investigated. Single PCR-based genotyping of *Botrytis* strains is useful for a preliminary classification, using polymorphic sequences and indels of selected genes, as demonstrated in this and previous studies (Plesken et al., 2015a). Indels of various sizes are frequently found in fungal genomes, but most of them are located outside of the coding sequences. Because of their rare occurrence and likely stability, indels in protein coding sequences have been used as high potential phylogenetic markers (Ajawatanawong and Baldauf, 2013). In our study, all preliminary assignments of species based on indel markers that were further analyzed by sequencing could be confirmed. For robust identification of isolates with a high sensitivity for differentiation, an MLST scheme has been established, using 10 genes which were selected for high variability and phylogenetic reliability. Using PacBio sequencing, 10 PCR products each of ca. 100 isolates can be sequenced in a single run, using appropriate barcodes. The genes selected for MLST provide more sequence information and show a considerably higher diversity than *hsp60*, *g3pdh*, and *rbp2*, which have been used for species differentiation of *Botrytis* species (Staats et al., 2005). Previous studies of *B. cinerea* populations, using PCR-based markers such as RAPD, ALFP, and microsatellite or simple sequence repeats (SSR; Walker, 2016), are efficient and provide valuable informations about population structures, but no sequence data for further strain comparisons. The MLST scheme developed in this study allows differentiation of *B. cinerea* and other *Botrytis* strains with high resolution. The design of the MLST primers also allowed amplification and sequencing of genes of two clade 2 species, *B. paeoniae* and *Botrytis mali* (not shown). They are therefore likely to be useful for genotyping of strains from all *Botrytis* species, but might require sequence adjustments for optimal performance.

The MLST analysis revealed a clear separation and phylogenetic placement of all *Botrytis* species. Intraspecific diversity of *B. pseudocinerea* and *B. fabae* strains appeared to be lower than that of *B. cinerea*. This might be partially explained by their smaller sample sizes, and remains to be confirmed with more strains from similarly diverse locations as those from *B. cinerea*. The phylogenetic tree reconstructed from the MLST sequences revealed several clusters with different bootstrap support. A previously identified *B. cinerea* population, *B. cinerea* “group S” (Leroch et al., 2013), was found to be much less evident on the basis of the MLST data (Figure 2), which does not justify to maintain group S as a separate taxonomic unit. Studies on *B. cinerea* group S strains in New Zealand and France (Johnston et al., 2014; Walker, 2016) and sequencing of additional genes, as shown below, did not support such a clear separation of group S strains.

A major discovery was the identification of a population, referred to as *B. cinerea* B, which was clearly separated from all other *B. cinerea* strains. Confirmation for genetic separation of the *B. cinerea* B population came from the observation that all of them lacked probably the whole botcinic acid gene (*boa*) cluster, which is present in all other *B. cinerea* strains and the majority of sequenced *Botrytis* species (Valero-Jiménez et al., 2020). Interestingly, the *boa* cluster is located in *B. cinerea* at the left terminus of chromosome 1, directly adjacent to the telomere (van Kan et al., 2017). It seems therefore likely that parts of the left arm of chromosome 1 have been deleted in the *B. cinerea* B population. Single mutants defective in botcinic acid synthesis have been shown to be unaffected in their virulence toward bean leaves, whereas double mutants defective in both botcinic acid and botrydial formation showed reduced infection (Dalmais et al., 2011). In a recent study, however, *bot2 boa6* double mutants were not found to be significantly affected in their virulence (Leisen et al., 2020). The existence of a *B. cinerea* group which lacks botcinic acid synthesis capacity also indicates that botcinic acid has no major role for plant infection. *B. cinerea* B strains were found on a variety of host plants (strawberry, grapevine, broad bean, cherry, and blueberry) in Germany and Norway, which indicates that they are widely distributed in Europe and maybe worldwide. Similar to *B. pseudocinerea*, which exists as a minor sister species in sympatry with *B. cinerea*, *B. cinerea* B was always found as minor populations together with common *B. cinerea* isolates. The occurrence of *B. cinerea* B remains to be analyzed systematically. For this, the PCR primers that detect the 15 bp deletion in Bcin11g00620, and the loss of *boa6* will facilitate a rapid screening for *B. cinerea* B genotypes.

The genetic separation between *B. cinerea* and *B. cinerea* group B indicates that genetic exchange between these groups has been strongly reduced or even stopped. Lack of genetic exchange by sexual recombination is an essential requirement for the definition of species and subspecies (Taylor et al., 2000). However, other concepts for species definition based on differences in morphology and ecology are much less clear. Similar to *B. pseudocinerea*, *B. cinerea* B isolates are morphologically indistinghuishable from *B. cinerea* regarding mycelium growth, sporulation, and sclerotium formation. Furthermore, they have been isolated from the same hosts as *B. cinerea*, indicating that they occupy a similar ecological niche (Walker et al., 2011). Nevertheless, *B. pseudocinerea* has been found to be more abundant in the spring, and is preferentially found on dead tissue parts from grapevine rather than on living berries (Walker et al., 2011). In commercial fields and orchards, *B. pseudocinerea* disappeared rapidly after fungicide treatments, and were almost never found to acquire fungicide resistance (Plesken et al., 2015a). *B. cinerea* B strains were found to be less virulent on tomato leaves than *B. cinerea* strains, which also indicates diverse ecological adaptions. Further studies are necessary to clarify these differences between *B. cinerea* B and *B. cinerea*. While our data indicate that a sexual barrier has already been formed between them, the loss sexual compatibility should be confirmed experimentally, as reported for *B. pseudocinerea* and *B. cinerea* (Walker et al., 2011). Further studies with *B. cinerea* group B populations are required to confirm whether or not they might represent a minor subspecies of *B. cinerea*.

The isolation of a genetically homogeneous *B. cinerea* population from the wild monocot plant *I. pseudacorus* was unexpected. *B. cinerea* and other clade 1 *Botrytis* species are known to infect almost only dicotyledonous hosts (Staats et al., 2005; van Kan, 2006), although *B. cinerea* can also attack some monocot plants. *Iris* isolates were considerably more aggressive on *Iris* than common *B. cinerea* strains. In contrast, they showed lower virulence on tomato leaves. Infection assays were performed with detached leaves, which might not yield the same results as leaves attached to intact plants. Nevertheless, differences in the infection behavior of *Iris* and other *B. cinerea* strains were obvious, in particular on *Iris* leaves. Several lines of evidence confirmed that the *Iris* strains are genetically distinct. While pink variants of *B. cinerea* are very rare, all but one of the *Iris* strains were pink and contained *bcbik1* encoding the polyketide synthase for bikaverin biosynthesis. Further genetic evidences, including MLST analysis of three *Iris* strains, a single nucleotide polymorphism in *mrr1* detected by PIRA-PCR, and a 6 bp deletion in Bcin01g05500 detected by PCR, confirmed that *B. cinerea Iris* represents a distinct, possibly local population. Even this small population was found to contain different haplotypes. Both mating type loci MAT1-1 and MAT1-2 were detected in similar numbers among the *Iris* strains, indicating that sexual exchange might still occur. Surprisingly, isolate D13_E_IL4, which clustered with the other *Iris* strains and was aggressive on *Iris*, did not show a pink color. PCR analysis revealed that this isolate not only lacked *bcbik1* but also *bcbik2*, *bcbik3*, and *bcbik5*, which are retained in B05.10 and all other *B. cinerea* isolates tested. Therefore, the loss of the whole *bik* gene cluster from D13_E_IL4 must have occurred in recent history. Bikaverin has antibiotic, anticancer, and antioomycete activity (Son et al., 2008) and has been shown to play a role in antagonistic interactions with other microbes (Spraker et al., 2016). Whether the pigment provides a selective advantage for *B. cinerea* remains to be investigated.

In summary, we have shown that MLST based on 10 suitable genes is a powerful tool for differentiation and classification of *Botrytis* clade 1 and possibly also for clade 2 strains, which makes it possible to recognize populations with different degrees of genetic separation. This work might stimulate further research into the intraspecific diversity of *B. cinerea* and other *Botrytis* species. It confirms previous evidences that genetic adaptation of *B. cinerea* toward certain hosts can occur (Mercier et al., 2019). Modern genome sequencing technologies and advanced editing tools such as CRISPR/Cas (Leisen et al., 2020) will enable a comparative molecular and phenotypic analysis of *B. cinerea* isolates with different host specificity, to uncover the mechanisms determining host range and host adaptation of the gray mold fungus.

## Data Availability Statement

The original contributions presented in the study are included in the article/Supplementary Material, further inquiries can be directed to the corresponding author/s.

## Author Contributions

CP did the sampling and genetic characterization of the isolates. PP prepared the PCR fragments for PacBio sequencing. BR, ZN, LZ, and PP performed phenotypic and PCR studies with the isolates. BH performed PacBio sequencing. KK evaluated sequencing data. MH did the conception and design of the study, and wrote the manuscript. All authors contributed to the article and approved the submitted version.

## Conflict of Interest

The authors declare that the research was conducted in the absence of any commercial or financial relationships that could be construed as a potential conflict of interest.

## Footnotes

1 http://genome.jouy.inra.fr/funybase/

2 http://fungi.ensembl.org/Botrytis_cinerea/Info/Index
