# Supplementary material for: Genetic Diversity of Botrytis cinerea Revealed by Multilocus Sequencing, and Identification of B. cinerea Populations Showing Genetic Isolation and Distinct Host Adaptation
Source: Front Plant Sci. 2021 May 5;12:663027. doi: 10.3389/fpls.2021.663027 (PMC8131559; doi:10.3389/fpls.2021.663027)
Supplement: Supplementary file 1 [file Data_Sheet_1.PDF]

Supplementary Material

Supplementary Table 1: Primers used in this study.

Name	Sequence (5' – 3')	Gene, purpose	Reference
FG1020-F	GGAGGATGATATGGCAAAGTC	Bcin04g02090	Marthey et
FG1020-R	GGATTAAGAGCTTCACTACCA	(*fg1020*/*Bcufd2*)	al., 2008
MS547-F	AAGGAGGACGTTGGAAGGAT	Bcin12g03020	Walker et al.,
MS547-R	AAGTCCAGAATCTCGATGTATTTGT	(ms547/*Bcdbp7*)	2011
mrr1_PIRA_fw	GATGGCAGCAAGGAACCTGTCGA	Bcin05g01790	This work
mrr1_PIRA_rev	TTCCGTACCCCGATCTTCGGAA	(*Bcmrr1*)	
Nep2(-200)for	GAGTTTCAGGTATATTCGTTTGGTGGA	Bcin02g07770	Staats et al.,
Nep2(+1147)rev	GAACTTTGAATAGTGGGCAGTTGGG	(*Bcnep2*)	2007
Nep1(-207)for	CACCTTGTGGGAGATTGTATGGGTGGATATACATC	Bcin06g06720	Staats et al.,
Nep1(+1124)rev	GGTCACCTAATTTTGGCTTTCAGGGTC	(*Bcnep1*)	2007
Univ for	gcagtcgaacatgtagctgactcaggtcac	(sequences for	This work
Univ rev	tggatcacttgtgcaagcatcacatcgtag	1st-round PCR)	
UPMLST-1F	gcagtcgaacatgtagctgactcaggtcacCTTGYCCTCCATCTCCTACC	Bcin01g07220	This work
RPMLST-1R	tggatcacttgtgcaagcatcacatcgtagACTCGGAATCGCYATCATTG	(MLST1)	
UPMLST-2F	gcagtcgaacatgtagctgactcaggtcacCCTATGTTGCTGGTGCACTC	Bcin05g07690	This work
RPMLST-2R	tggatcacttgtgcaagcatcacatcgtagTTCGGCATTTCGCCTGATCC	(MLST2)	
UPMLST-3F	gcagtcgaacatgtagctgactcaggtcacTCTTGTAGGCCCTTGACTCC	Bcin06g01710	This work
RPMLST-3R	tggatcacttgtgcaagcatcacatcgtagATGCCTGGTCTGACACATTG	(MLST3)	
UPMLST-4Fn	gcagtcgaacatgtagctgactcaggtcacACTCCGGTACATCCTATACG	Bcin09g03030	This work
RPMLST-4R	tggatcacttgtgcaagcatcacatcgtagAAACCASGCGCTATCTTCGG	(MLST4)	
UPMLST-5F	gcagtcgaacatgtagctgactcaggtcacTTGTCGGTCCTTGTGAATGG	Bcin11g01310	This work
RPMLST-5R	tggatcacttgtgcaagcatcacatcgtagGGGACTGAGGAYATCTAAGC	(MLST5)	
UPMLST-6F	gcagtcgaacatgtagctgactcaggtcacGTCATCTGGGCTTGCTTCTC	Bcin15g03910	This work
RPMLST-6R	tggatcacttgtgcaagcatcacatcgtagCACCTTRTCCGGAAAGACTC	(MLST6)	
UPMLST-7F	gcagtcgaacatgtagctgactcaggtcacCGGTCGTCAMTCTGCTCTTC	Bcin16g03460	This work
RPMLST-7R	tggatcacttgtgcaagcatcacatcgtagAGACCAGCGTGCCATGTTTC	(MLST7)	
UPMLST-8F	gcagtcgaacatgtagctgactcaggtcacAAGGAGGACGTTGGAAGGAT	Bcin12g03020	This work
RPMLST-8R	tggatcacttgtgcaagcatcacatcgtagAAGTCCAGAATCTCGATGTATTTGT	(MLST8)	
UPMLST-9F	gcagtcgaacatgtagctgactcaggtcacTTGCCTTCTCAAAATCATTACAGC	Bcin02g07770	This work
RPMLST-9R	tggatcacttgtgcaagcatcacatcgtagTCTAGAAAGTAGCCTTCGCAAGAT	(MLST9)	
UPMLST-10F	gcagtcgaacatgtagctgactcaggtcacAGAAGCTGTGGCTCGCTTTG	Bcin04g02090	This work
RPMLST-10R	tggatcacttgtgcaagcatcacatcgtagCGTGCCACTTTAACACGTTCTG	(MLST10)	
Bfab122-fw	GAGCCTTCTCCCTTGGTTAC	Bcin13g02260	Rigotti et al.,
Bfab122-rv	ATGCATGGTGGCGTCGTTGG	(indel)	2002
Bps24-fw	GCAGATGAGGCGGATGATAG	Bcin09g02270	Plesken et al.,
Bps24-rv	TCCACCCAAGCATCATCTTC	(indel)	2015a
mrr1-18-fw	GCGACCTCATCGTTCTTTCAC	Bc05g01790	Plesken et al.,
mrr1-18-rv	GGCTCTCGATGAGCTGTTTC	(*Bcmrr1* indel)	2015a
Bcal9-fw	TCCGCAGCTTCAGTACCTTC	Bcin16g02210	Plesken et al.,
Bcal9-rv	GTAGATTTGGCAGCGGATAG	(indel)	2015b
BcB15-fw	CATTCGYGCATGAGGAGGAG	Bcin11g00620	This work
BcB15-rv	TCCTGGAGGWCCTGGAAATC	(indel)	
mrr1-21-fw	TATCGGTCTTGCAGTCCGC	Bcin05g01790	Leroch et al.,
mrr1-21-rv	TTCCGTACCCCGATCTTCGGAA	(*Bcmrr1* indel)	2013
BcIris6-fw	CGAGAAGAGTCCAACATCCATC	Bcin01g05500	This work
BcIris6-rv	GGAAGCAGAACTCGATACTG	(ms362)	
Mat1-F	GTGACCAGGAAACAGCTATGACCGGAGTGTGTTGATCGTGGAGCCGAG	MAT1-1	Amselem et al., 2011
Mat1-R	GTGACTGTAAAACGACGGCCAGTCCACACATACATCATGACGGCTCCC		
BcHMG-F	GTGACCAGGAAACAGCTATGACCGCTCCTTTCCATAAGTC	MAT1-2	

BcHMG-R	GTGACTGTAAAACGACGGCCAGTCAAGATCAGACGGAGTGCATTACCTC		
BIK1-F6	GTTCGCAGACGAGCTGAAA	*Bcbik1*	Schumacher et al., 2013
BIK1-R6	TCGATCTTTGGTCAGGCTCT		
Bik2_113_F	GTCGCGATACTCTCGAGTC	*Bcbik2*	Schumacher et al., 2013
Bik2_823_R	GGCCGAGGTTCTCAAAG		
bik3_146F	ACAAACTTCCCGAGCCTAGC	*Bcbik3*	Schumacher et al., 2013
bik3_1425R	CTACCATAAGGCGTGTTGAC		
bik5_961F	TCGCAAGGACCATGGGTCTC	*Bcbik5*	Schumacher et al., 2013
bik5_1757R	GCAGTCCCATGCYTGATCAC		
Bot2_F	GCACTTGAACCCCAGCTACACG	*Bcbot2*	This work
BOT2_R	GTACGATCGTCGAAGAGGAAC		
BOA6_F	GTTGGATATGTTGAACGCTCAGG	*Bcboa6*	This work
BOA6_R	GCAGCACCACCCAATATCTTC		
BOA17_F	TTGGCAAGGAGCGCGTCTTC	*Bcboa17*	This work
BOA17_R	TCGACCGCATCAGGTCCAACA		

Supplementary Table 2. Sequence diversity of MLST genes (*hsp60* as reference) between strains of *B. calthae*, *B. pseudocinerea*, *B. fabae* and *B. cinerea* (cf. Supplementary Figure 2).

	MLST1	MLST2	MLST3	MLST4	MLST5	MLST6	MLST7	MLST8	MLST9	MLST10	**MLST1-10**	*hsp60*
Nucleotides	1278	1025	995	997	885	938	805	903	818	876	**9520**	980
Variable sites	178	102	101	104	87	93	104	78	77	80	**1004**	29
% variable sites	13,9	10,0	10,2	10,4	9,8	9,9	12,9	8,6	9,4	9,1	**10,5**	3,0

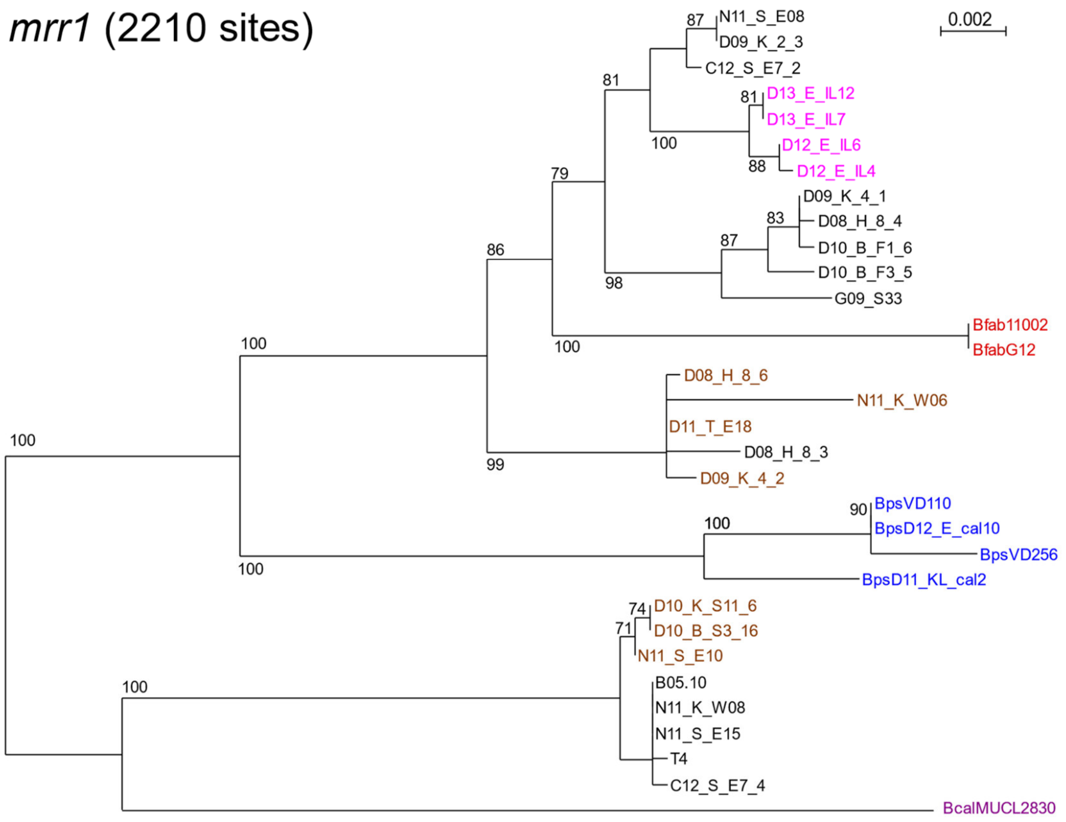

Supplementary Figure 1: PhyML-generated tree based on *mrr1* sequences, of *Botrytis* clade 1 species *B. calthae* (Bcal), *B. pseudocinerea* (Bps), *B. fabae* (Bfab) and *B. cinerea* isolates. B. cinerea B isolates are shown in brown color. Bootstrap values >70 based on 1000 replicates are shown.

MLST1 (1278 sites)

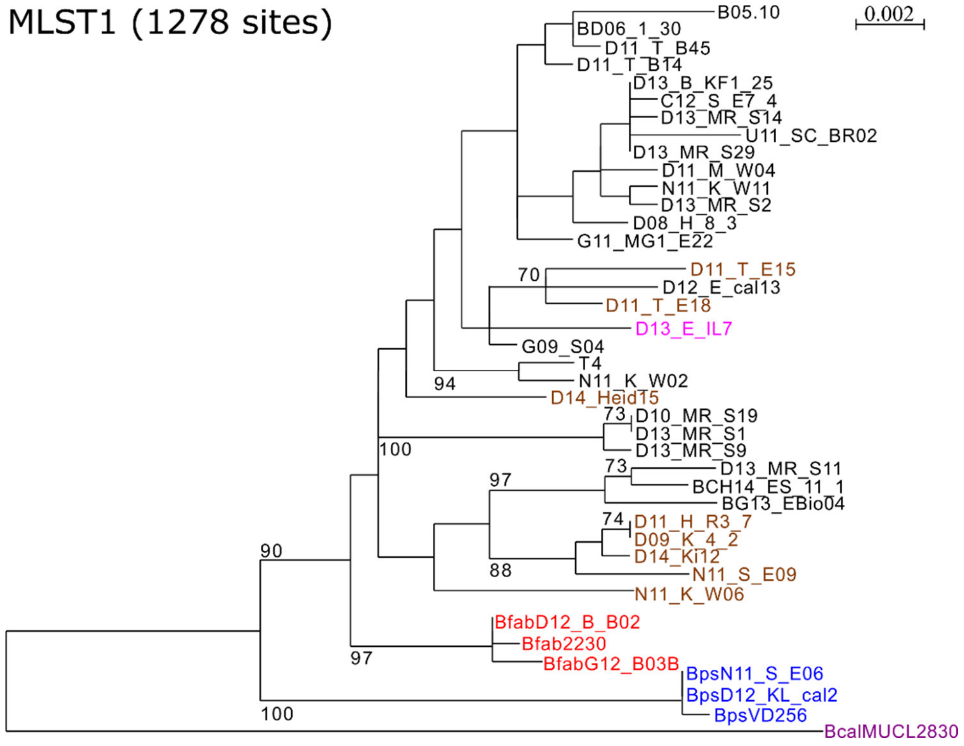

MLST2 (1025 sites)

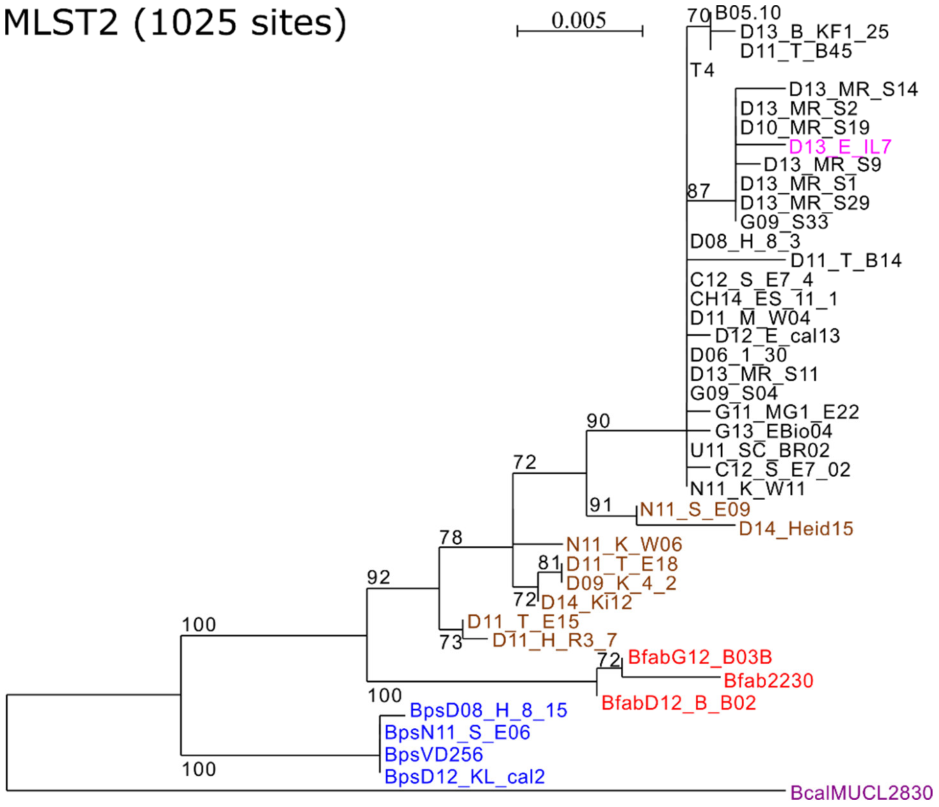

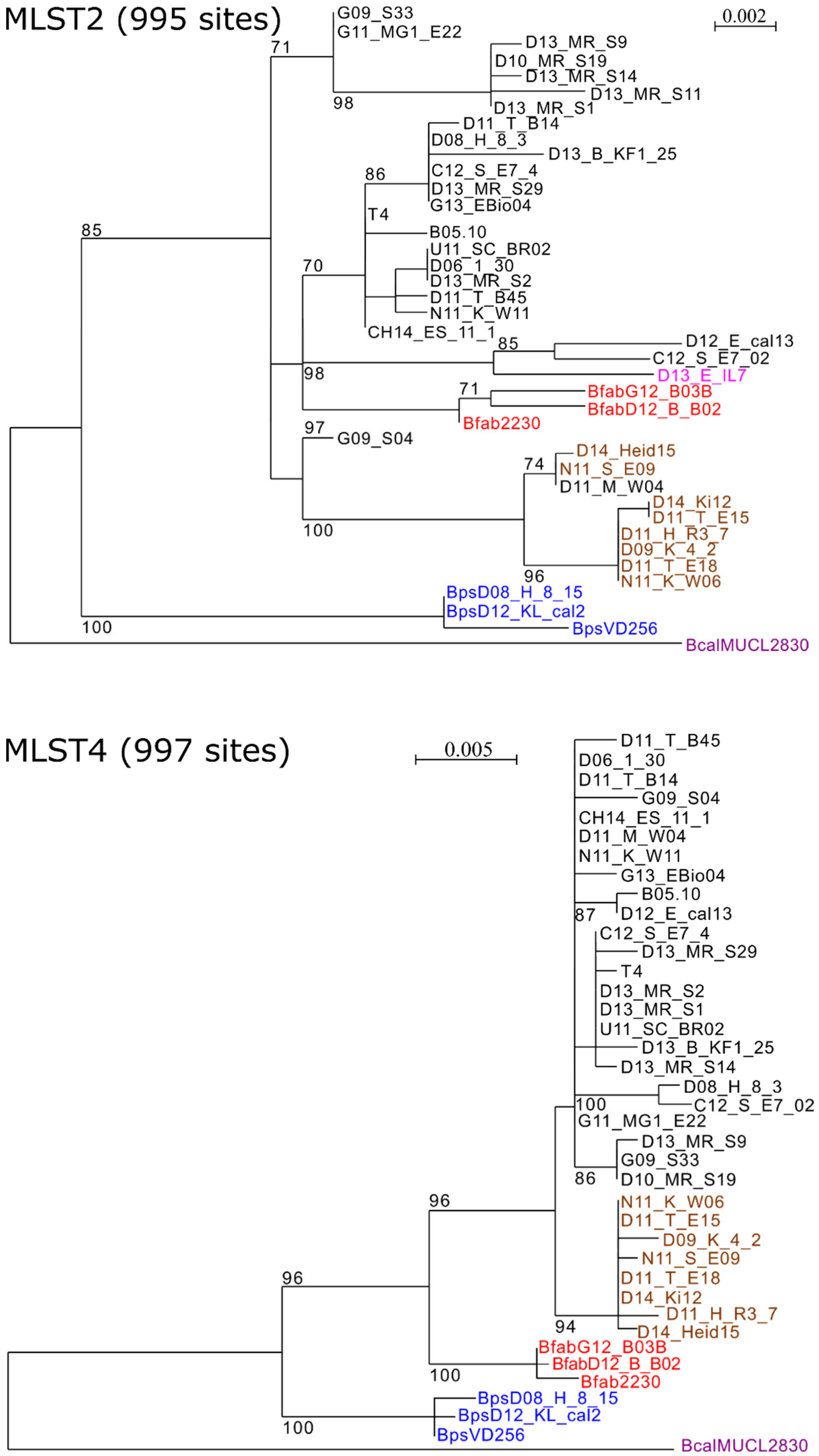
MLST2 (995 sites)
0.002
G09_S33
G11_MG1_E22
D13_MR_S9
D10_MR_S19
D13_MR_S14
D13_MR_S11
D13_MR_S1
D11_T_B14
D08_H_8_3
D13_B_KF1_25
C12_S_E7_4
D13_MR_S29
G13_EBio04
T4
B05.10
U11_SC_BR02
D06_1_30
D13_MR_S2
D11_T_B45
N11_K_W11
CH14_ES_11_1
D12_E_cal13
C12_S_E7_02
D13_E_IL7
BfabG12_B03B
BfabD12_B_B02
Bfab2230
G09_S04
D14_Heid15
N11_S_E09
D11_M_W04
D14_Ki12
D11_T_E15
D11_H_R3_7
D09_K_4_2
D11_T_E18
N11_K_W06
BpsD08_H_8_15
BpsD12_KL_cal2
BpsVD256
BcalMUCL2830
71
98
86
85
70
98
85
71
97
74
100
96
100
MLST4 (997 sites)
0.005
D11_T_B45
D06_1_30
D11_T_B14
G09_S04
CH14_ES_11_1
D11_M_W04
N11_K_W11
G13_EBio04
B05.10
D12_E_cal13
C12_S_E7_4
D13_MR_S29
T4
D13_MR_S2
D13_MR_S1
U11_SC_BR02
D13_B_KF1_25
D13_MR_S14
D08_H_8_3
C12_S_E7_02
G11_MG1_E22
D13_MR_S9
G09_S33
D10_MR_S19
N11_K_W06
D11_T_E15
D09_K_4_2
N11_S_E09
D11_T_E18
D14_Ki12
D11_H_R3_7
D14_Heid15
BfabG12_B03B
BfabD12_B_B02
Bfab2230
BpsD08_H_8_15
BpsD12_KL_cal2
BpsVD256
BcalMUCL2830
87
100
86
96
96
94
100
100

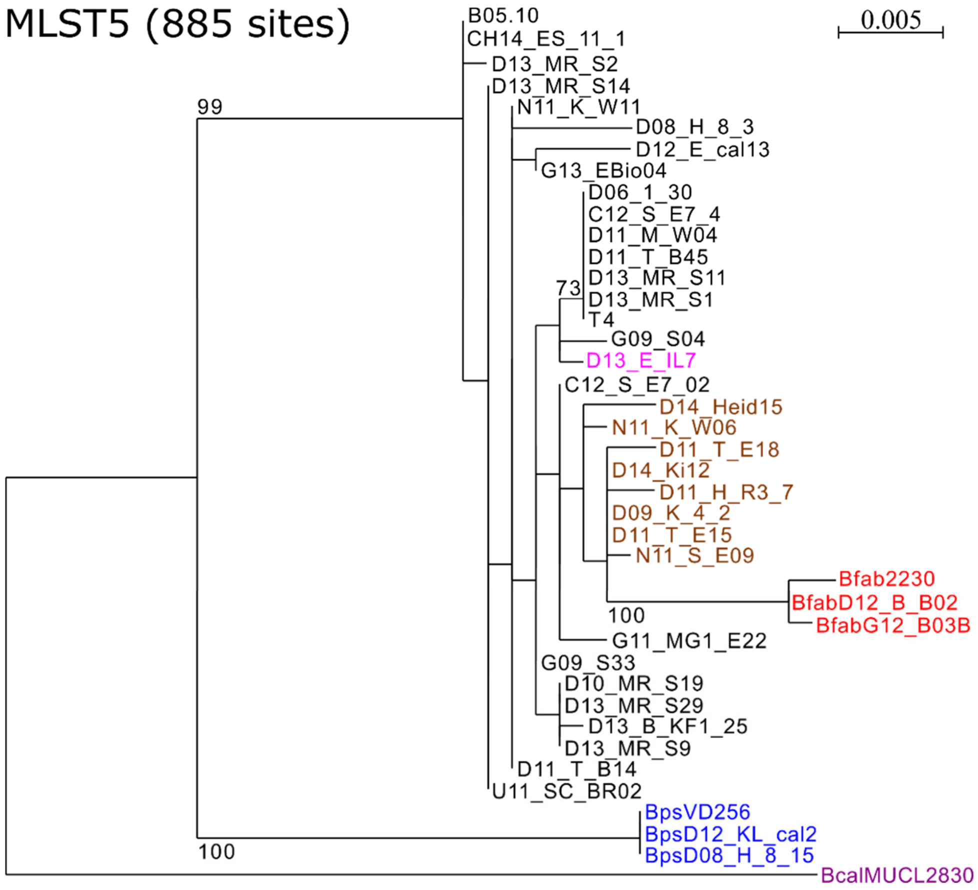
MLST5 (885 sites)
0.005
B05.10
CH14_ES_11_1
D13_MR_S2
D13_MR_S14
N11_K_W11
D08_H_8_3
D12_E_cal13
G13_EBio04
D06_1_30
C12_S_E7_4
D11_M_W04
D11_T_B45
D13_MR_S11
D13_MR_S1
T4
G09_S04
D13_E_IL7
C12_S_E7_02
D14_Heid15
N11_K_W06
D11_T_E18
D14_Ki12
D11_H_R3_7
D09_K_4_2
D11_T_E15
N11_S_E09
Bfab2230
BfabD12_B_B02
BfabG12_B03B
G11_MG1_E22
G09_S33
D10_MR_S19
D13_MR_S29
D13_B_KF1_25
D13_MR_S9
D11_T_B14
U11_SC_BR02
BpsVD256
BpsD12_KL_cal2
BpsD08_H_8_15
BcalMUCL2830
99
73
100
100

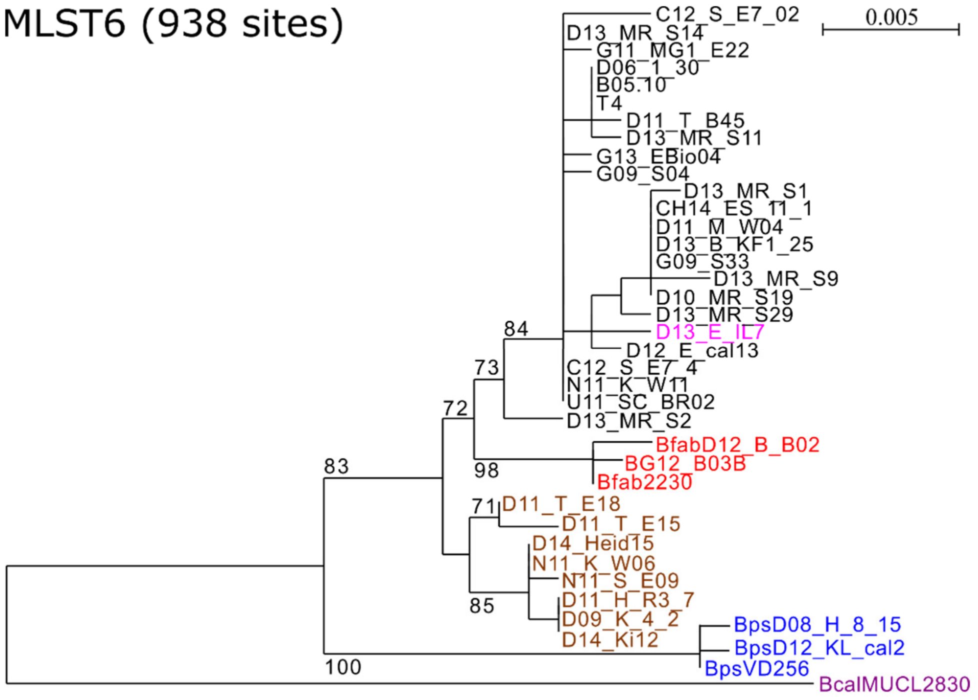
MLST6 (938 sites)
0.005
C12_S_E7_02
D13_MR_S14
G11_MG1_E22
D06_1_30
B05.10
T4
D11_T_B45
D13_MR_S11
G13_EBio04
G09_S04
D13_MR_S1
CH14_ES_11_1
D11_M_W04
D13_B_KF1_25
G09_S33
D13_MR_S9
D10_MR_S19
D13_MR_S29
D13_E_IL7
D12_E_cal13
C12_S_E7_4
N11_K_W11
U11_SC_BR02
D13_MR_S2
BfabD12_B_B02
BG12_B03B
Bfab2230
D11_T_E18
D11_T_E15
D14_Heid15
N11_K_W06
N11_S_E09
D11_H_R3_7
D09_K_4_2
D14_Ki12
BpsD08_H_8_15
BpsD12_KL_cal2
BpsVD256
BcalMUCL2830
84
73
72
83
98
71
85
100

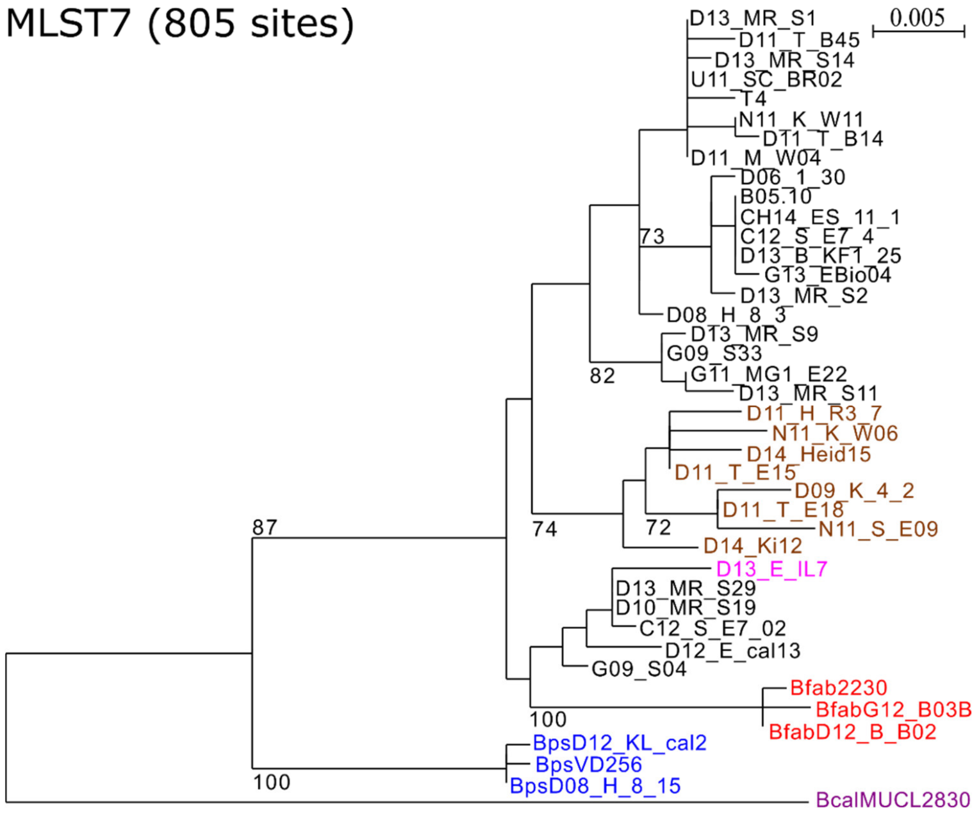

MLST7 (805 sites)
0.005
D13_MR_S1
D11_T_B45
D13_MR_S14
U11_SC_BR02
T4
N11_K_W11
D11_T_B14
D11_M_W04
D06_1_30
B05.10
CH14_ES_11_1
C12_S_E7_4
D13_B_KF1_25
G13_EBio04
D13_MR_S2
D08_H_8_3
D13_MR_S9
G09_S33
G11_MG1_E22
D13_MR_S11
D11_H_R3_7
N11_K_W06
D14_Heid15
D11_T_E15
D09_K_4_2
D11_T_E18
N11_S_E09
D14_Ki12
D13_E_IL7
D13_MR_S29
D10_MR_S19
C12_S_E7_02
D12_E_cal13
G09_S04
Bfab2230
BfabG12_B03B
BfabD12_B_B02
BpsD12_KL_cal2
BpsVD256
BpsD08_H_8_15
BcalMUCL2830
73
82
87
74
72
100
100

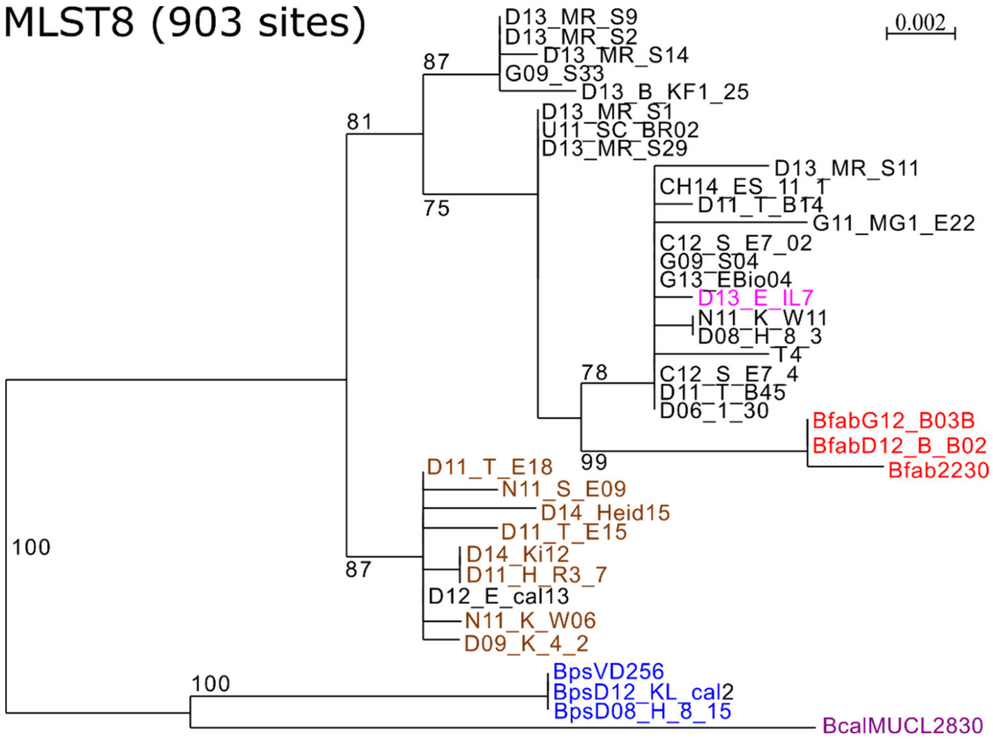

MLST8 (903 sites)
0.002
D13_MR_S9
D13_MR_S2
D13_MR_S14
G09_S33
D13_B_KF1_25
D13_MR_S1
U11_SC_BR02
D13_MR_S29
D13_MR_S11
CH14_ES_11_1
D11_T_B14
G11_MG1_E22
C12_S_E7_02
G09_S04
G13_EBio04
D13_E_IL7
N11_K_W11
D08_H_8_3
T4
C12_S_E7_4
D11_T_B45
D06_1_30
BfabG12_B03B
BfabD12_B_B02
Bfab2230
D11_T_E18
N11_S_E09
D14_Heid15
D11_T_E15
D14_Ki12
D11_H_R3_7
D12_E_cal13
N11_K_W06
D09_K_4_2
BpsVD256
BpsD12_KL_cal2
BpsD08_H_8_15
BcalMUCL2830
87
81
75
78
99
87
100
100

MLST9 (818 sites)

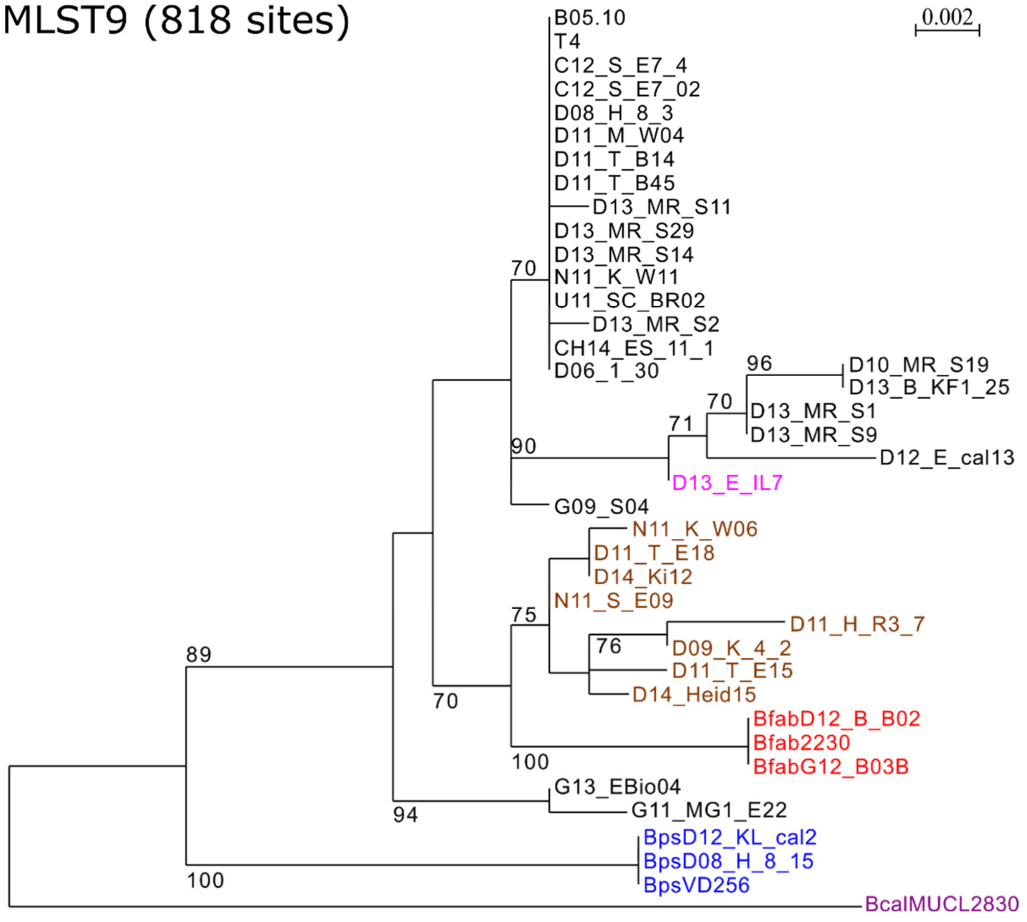

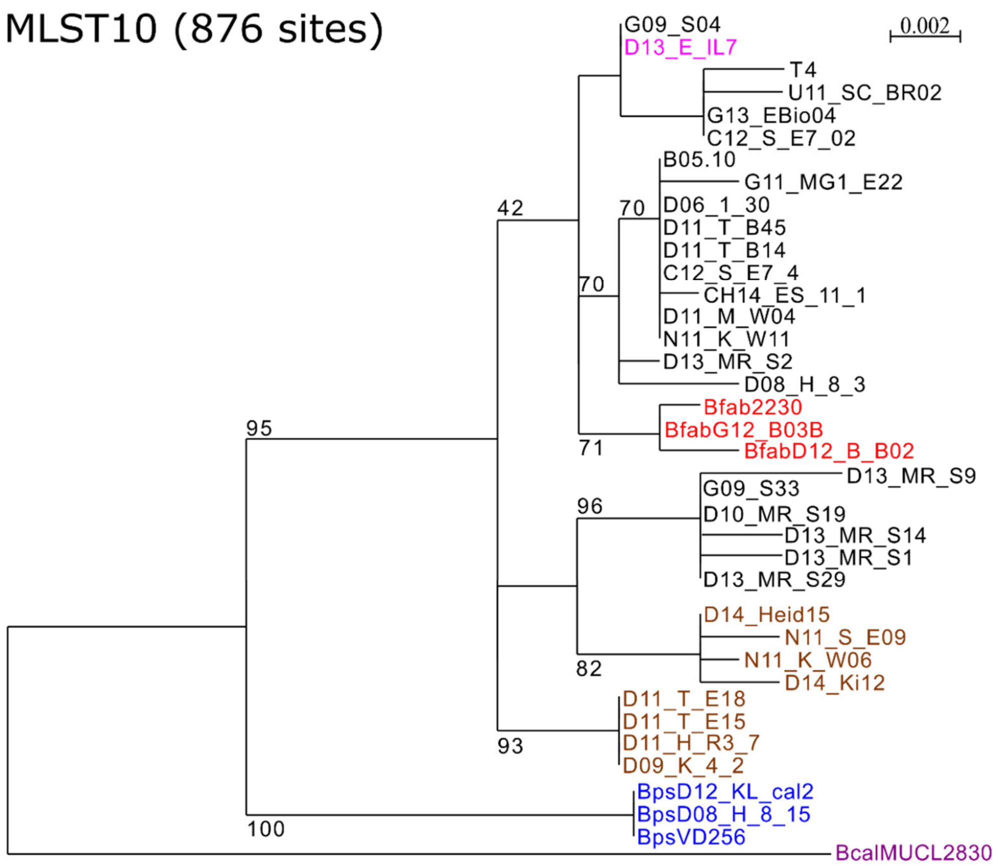

Supplementary Figure 2: PhyML-generated trees of selected *Botrytis* strains, based on the seqeunces of individual MLST genes. Bootstrap values >70 based on 500 replicates each are shown. Bcal (purple): *B. calthae*; Bps (blue): *B. pseudocinerea*; Bfab (red): *B. fabae*. *B. cinerea* group B (brown); *B. cinerea Iris* (pink).

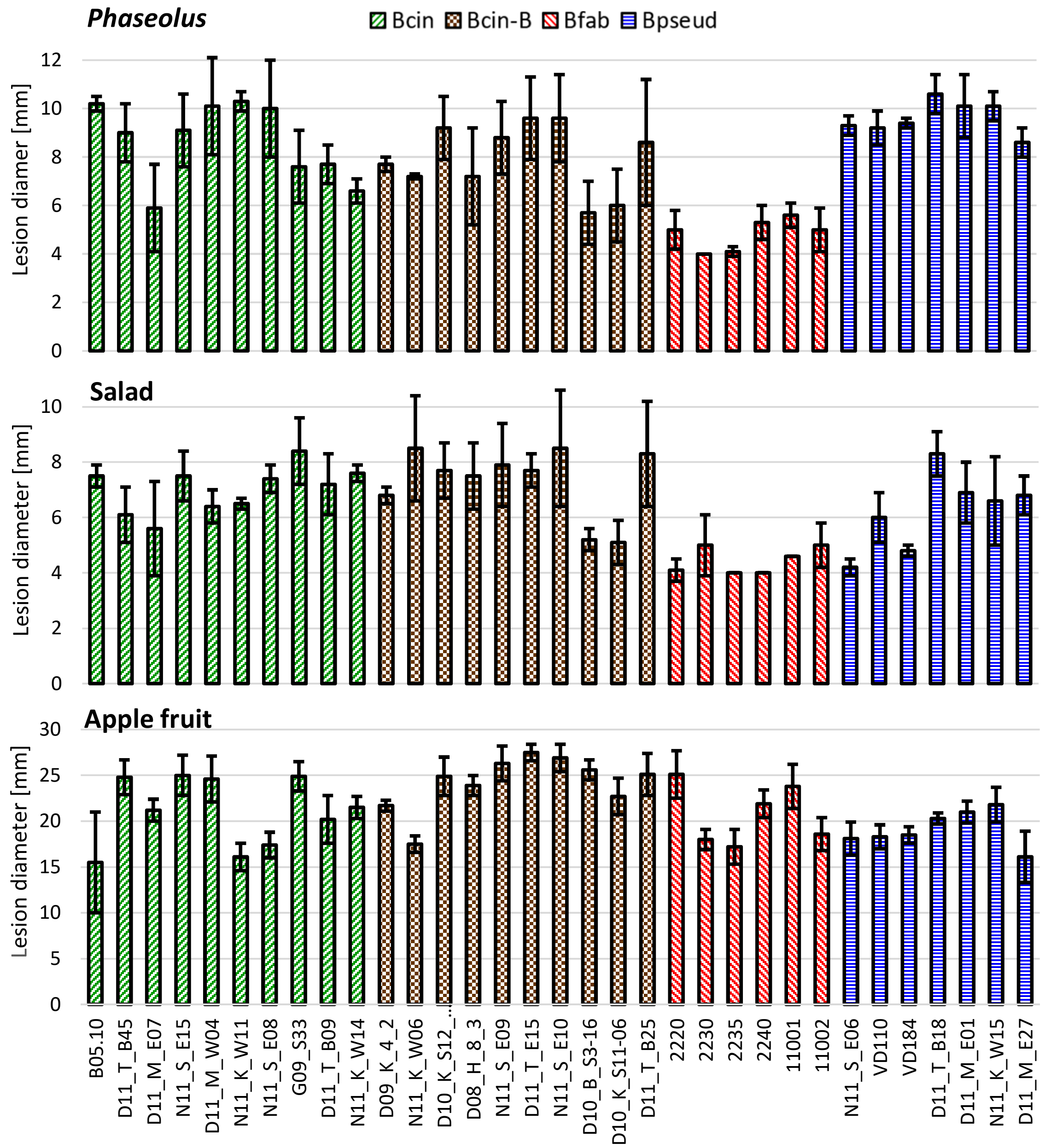

Supplementary Figure 3: Lesion formation by strains of *B. cinerea* (green), *B. cinerea* B (brown), *B. fabae* (red) and *B. pseudocinerea* (blue) on three different host tissues, three days after inoculation.

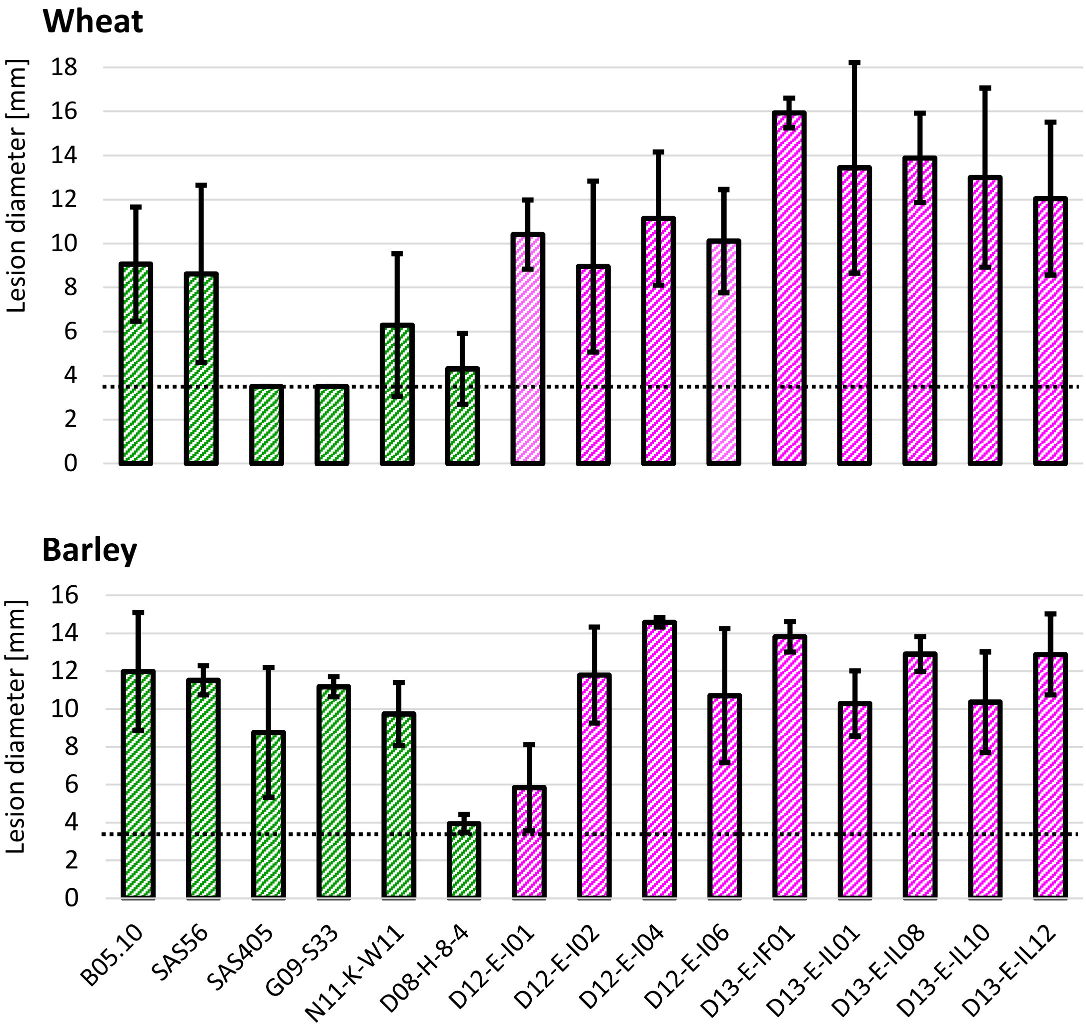

Supplementary Figure 4: Lesion formation by strains of *B. cinerea* (green) and of *B. cinerea Iris* (pink) on wheat and barley leaves, four days after inoculation.

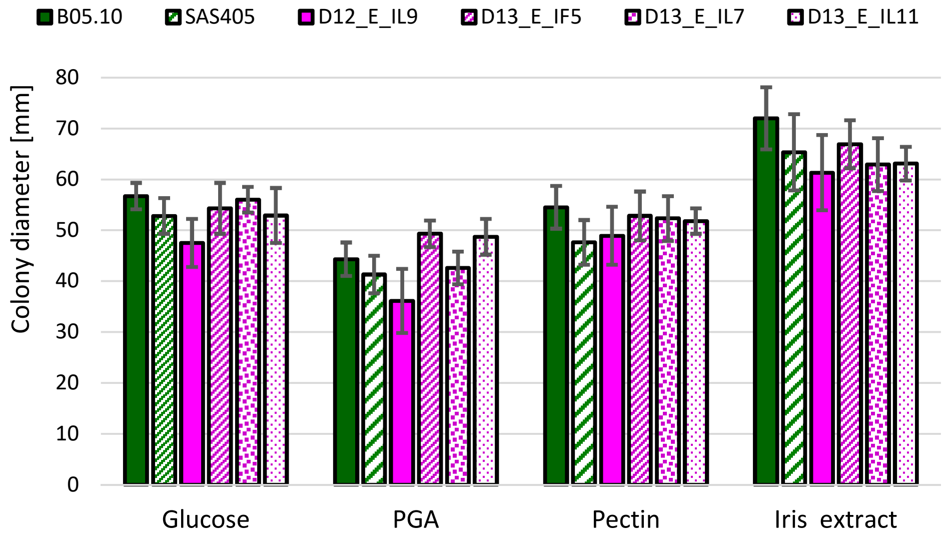

Supplementary Figure 5: Mycelium growth of two *B. cinerea* strains (green) and four *B. cinerea Iris* strains on agar containing minimal medium with glucose (50 mM), 0.5% polygalacturonic acid (PGA), 0.5% pectin, and 0.1% *Iris* total leaf extract, four days after inoculation. Data are represented as means ± standard deviation from two independent biological replicates. No significant differences between the strains were observed with one-way ANOVA, followed by Dunnett's post hoc test (control: B05.10).

frontiers
