# Supplementary material for: Genetic Diversity of Botrytis cinerea Revealed by Multilocus Sequencing, and Identification of B. cinerea Populations Showing Genetic Isolation and Distinct Host Adaptation
Source: Front Plant Sci. 2021 May 5;12:663027. doi: 10.3389/fpls.2021.663027 (PMC8131559; doi:10.3389/fpls.2021.663027)
Supplement: Supplementary file 2 [file Data_Sheet_2.pdf]

>Bcin05g01790(mrr1), partial sequence [organism=Botrytis cinerea, strain B05.10]
CAGGCATCTGGCACGGAATGTATTTTCCCTACACGTGTTCGGAACCCAAGACCGAGACAAAGCAATCCAAAGTCTCGTGATTCTGAGCTATTAA
AAAGAATCTCTCATCTCGAAAGTTTGGTGAGCAGAATTGATGCCTCAAAACTCCTTGGGGAGGATGGTCAGGTAGAATACGAGGTTCGGTCAAA
TCCGCCATCATCAGGACCGCGACCTCATCGTTCTTTCACGTCTCCAGACCATCATGAGTTTGGAATAGTACCAGGTCGTATGGCTCATCAATTT
GCTGATTTTATTAAACGCCAAGAGAACGACTCGATTTACACTCATGATGGATTTTGGACAAGACTAAGTGAAGAAATTGATGGATTGAAACAGC
TCATCGAGAGCCCCAGTGACGATGAAGATGAAAATTCTGGGTCCACTGCAACTTCGCCAGCATCCAAGTATGACTCTCCTTCACAGTTTGTCTT
TGATTCCAGGGTCACTTCCTCGAATGTCATAATTCCATATCCATCCCACGACCATACCAAAGTTTTATTCGATATTTATTTCAAAAATGTACAT
CCGATGGTCGTCACTATGCACAAGCCAACAACGTACCTTTTCACAAACCCAAATTCTGAGTTACTCGATGAGAATACTGGCCGGCACAAATTCA
AAAGTCTCGAGGCTAGCGTGTTTGCTATGTACCTGGCTGCTGTGACGAGCATGACAAATGAAGAGTGCCTTGAGCTTTTAAATGAAGAAAGAAA
TATTTTAGTAACACGGTACAAAACTGCGACTGAGATTGCTCTTCAAGAATCAGATTTTCTTAATAGTGTTGAAATTGTTACTTTACAAGCTTTA
ATAACGTATATCGTAAGTGTTTCTCTCTAAGCATCTTGGATGATGGTTTTATGATGATGATGATGATGATGTTTATGTACTTTCACAGGCAGCT
ATGCGCACTCATGATCGAAGTCGATCTATATGGGCATTTATCGGTCTTGCAGTCCGCCTTGCCCGTGGCATCGGGCTCCACAGAGATGGCAGCC
AGCAACCTTTCGATTTGGAAATGCGAAGGAGAGTGTGGTGGACATTGATTGTACTTGATACAAGAGCTTCCGAAGATCGGGGTACAGAAACCAT
GATCACTGATGGCTCTTTCGATACAAAGATGCCTGCCAACATAAACGACGAGGATATGATGATAAACTCAAAAAGTCTACCGGTCGATCGGATA
GGGATCACCAGTATGACCTTTGCTTGCATTACAATGACAGTCAGCGGGATCGGATTGAGGATGAATTTTGTACCTACGCGCCTAGACGCGCCGG
TCTTGACCACAGAACAAAAAGAGCAGATGATCAAAGGATTTACTGACAAAATCGATTCTACCTATCTCGCAGGTTCTGATCCCAATGATCCAAG
ATTGTGGTGGTATTGTCGGATATCACGACTATTATCGCTCAAGTTATGGCTGGTGACACAATATCCACTTCAACGAAGGAAATCTACAAATCGA
GTGCTTCCTCGAGGACAATCCTTGCGTACAGCTATGGCATTCCTAAATTTGGCGGAAGAAATATGGCAATATGAACCTTCAAAGGGATGGTTCT
GGTTCTTTCAAAGTTATGTGCCGTGGCATGTAATTGCGGTTGCTTTAGCTGAGCTTTGTGCGGAACCAACTGGCCCTCTTGCCGATCAAGCTTG
GGAGGGCATTGAGTTTCACTACAATAAATGGAGTGAATTCGTTGCAGATACAAAAGATGGGATGATTTGGCGACCAGTCAGGAATTTAATGAAA
AGAGCAAGAGCAGCAAGAAGGCGAGAAAGAGGTCTCAGTGAATCTCAAGCATCAACAACTCCAACGGCAATTCGCAACCCTAATACATACAGCC
CAGCTCTCAATCTAGCTGCTGGATGCTCCCCGATCATGTCGGAGAACAATAACTACTGTCAATCTTCTACAATTGGACAGGTTGACCCAATGGG
GCAGAATATTTACGGGGGAATGGGCTTCGATCTTCCCATGCCCATGGGTTCTACCCCCTCAGACACGAATATGGCATCCCCACTACCTATTCCG
GACGGCTATACAAATCCGCACTGGAACGAATTCATATTTGGTTTAGGAACAGTTTGTAGCGATGGACCCCAAAATCCAGA
>Bcin05g01790(mrr1), partial sequence [organism=Botrytis fabae, strain G12]
CAGGCATCTGGCACGGAATGCATTTTCCCTACACGTGTTCGGAACCCAAGACCGAGACAAAGCAATCCAAAGTCTCGTGATTCTGAGCTATTAA
AAAGAATCTCTCACCTCGAAAGTTTGGTGAGCAGAATTGATGCCTCAAAACTCCTTGGGGAGGATGGTCAGGTAGAAGACGAGGTTCGGTCAAA
TCCACCATCATCAGGACCGCGACCTCATCGTTCTTTCACGTCTCCAGACCACCAATACAATGACAATCATCATGAGTTCGGAATAGTACCAGGT
CGTATGACTCATCAATTTGCTGATTTTATTAAACGCCAAGAGACCGACTCGATTTACACCCATGATGGATTTTGGACAAGACTAAGTGAAGAAC
TTGATGGATTGAAACATCTCATCGAGAGCCCCAGTGACGATGAAGATGAAAATTCTGGGTCCACTGCAACTTCGCCAGCATCCAAGTATGACTC
CCCTTCACAGTTTGTCTTTGATTCCAGGGTCACTTCCTCGAATGTCACAATTCCATATCCATCCCGCGACCATACGAAAGTTTTATTCGATATT
TATTTCAAAAATATACATCCGATGATCGTCACTATGCACAAGTCAACAACGTACCTTTTCACAAACCCAAATTCTGAGTTACTCGATGAGAATA
CTGGCCGGCACAAATTCAAAAGTCTCGAGGCTAGCACATTTTCTATGTACCTGGCTGCTGTGACGAGCATGACAAATGAAGAGTGCCTTGAGCT
TTTAAATGAAGAAAGACAGATTTTAGTAACAAGGTACAAAACTGCGACTGAGATTGCTCTTCAAGAATCAGATTTTCTTAATAGTGTAGAAATT
ATTACTTTACAAGCTTTAATAATGTATATCGTAAGTATTTCTCTCTCAGCATCTTGGATGATGGTTTTATGATGATGATGATGTTTATGTACTT
TCACAGACAGCTATGCGCGCTCATGATCGAAGTCGATCTACATGGGCATTTATCGGTCTTGCAGTCCGCCTTGCCCGTGGCATCGGGCTCCACA
GGGATGGCACCGGGCTCCACAGAGATGGCAGCAAGCAACCTTTCGATGTGGAAATGCGAAGGAGAATATGGTGGACATTGATTGTACTTGATAC
AAGAGCTTCCGAAGATCGGGGCACGGAAACTATGATCACGGATGGCTCTTTCGATACAAAGATGCCTGCCAATATAAATGACGAGGATATATCG
ATAAACTCAAAAACCTCAGCGGTCGATCGGTTAGGATTCACCAGTATGACCTTTGCTTGCATTACAATGACAGCCAGCGGGATCGGATTGAAGA
TGAATTTTGTACCTACGCGCCTAGACGCGCCAGTCTTGACCACAGAACAAAAAGAGCAGATGATCAAAGGATTTACTGACAAAGTCGATTCTAC
CTATCTCACATGTTCTGATCCCAATGATCCAAGATTGTGGTGGTTTTGTCGGGTATCACGACTATTATCGCTCAAGTTATGGTTGGCGACACAA
TATCCACTTCAGCGAAGGAAATCTACAAATCGAGTGCTTCCTCGAGGACAATCCTTGCGTACAGCTATGGCATTCTTAAATTTGGCGGAAGAAA
TATGGCAATATGAACCTTCAAAGGGATGGTTCTGGTTCTTTCACAGTTATGTGCCGTGGCATGCAATTGCGGTTGCTTTAGCTGAGCTTTGCGC
GGAACCAACTGGCCCTCTTGCCGATCAAGCTTGGGGGAGTATCGAGTTTCATTACAATAAATGGAGTGAACTCGTTGCAGATACAAAAGATGGG
GTGATTTGGCGGCCAGTCAAGAATTTAATGAAAAGAGCAAAAGCAGCAAGAAGGCGAGAAAGAGGTCTCAGTGGATCTCAAGCATCAACAACTC
CAACGGCAATTCTCAACCCTAATACATACAGCCCAGCTCTCGATCTAGGTGCTGAATTCTCACCAATCATGTCGGAGAATAATAACTACCGTCA
ACCTTCTACAATTGGGCAGGTTGACCCAATGGGGCAGAATATTTACGGGGGGATGAGCTTCGATCTTCCCATGCCCATGTGTTCTACCCCCCTCA
GACACGAATATGGCATCCCCACTACCTATGCCGGACGGCTACACAAATCCGCACTGGAACGAATTCATATTTGGTTTAGGAACAGTTGGTAGTG
ATGGACTCCAAAATCCAGA
>Bcin05g01790(mrr1), partial sequence [organism=Botrytis fabae, strain 11002]
CAGGCATCTGGCACGGAATGCATTTTCCCTACACGTGTTCGGAACCCAAGACCGAGACAAAGCAATCCAAAGTCTCGTGATTCTGAGCTATTAA
AAAGAATCTCTCACCTCGAAAGTTTGGTGAGCAGAATTGATGCCTCAAAACTCCTTGGGGAGGATGGTCAGGTAGAAGACGAGGTTCGGTCAAA
TCCACCATCATCAGGACCGCGACCTCATCGTTCTTTCACGTCTCCAGACCACCAATACAATGACAATCATCATGAGTTCGGAATAGTACCAGGT
CGTATGACTCATCAATTTGCTGATTTTATTAAACGCCAAGAGACCGACTCGATTTACACCCATGATGGATTTTGGACAAGACTAAGTGAAGAAC
TTGATGGATTGAAACATCTCATCGAGAGCCCCAGTGACGATGAAGATGAAAATTCTGGGTCCACTGCAACTTCGCCAGCATCCAAGTATGACTC
CCCTTCACAGTTTGTCTTTGATTCCAGGGTCACTTCCTCGAATGTCACAATTCCATATCCATCCCGCGACCATACGAAAGTTTTATTCGATATT
TATTTCAAAAATATACATCCGATGATCGTCACTATGCACAAGTCAACAACGTACCTTTTCACAAACCCAAATTCTGAGTTACTCGATGAGAATA

CTGGCCGGCACAAATTCAAAAGTCTCGAGGCTAGCACATTTTCTATGTACCTGGCTGCTGTGACGAGCATGACAAATGAAGAGTGCCTTGAGCT
TTTAAATGAAGAAAGACAGATTTTAGTAACAAGGTACAAAACTGCGACTGAGATTGCTCTTCAAGAATCAGATTTTCTTAATAGTGTAGAAATT
ATTACTTTACAAGCTTTAATAATGTATATCGTAAGTATTTCTCTCTCAGCATCTTGGATGATGGTTTTATGATGATGATGATGTTTATGTACTT
TCACAGACAGCTATGCGCGCTCATGATCGAAGTCGATCTACATGGGCATTTATCGGTCTTGCAGTCCGCCTTGCCCGTGGCATCGGGCTCCACA
GGGATGGCACCGGGCTCCACAGAGATGGCAGCAAGCAACCTTTCGATGTGGAAATGCGAAGGAGAATATGGTGGACATTGATTGTACTTGATAC
AAGAGCTTCCGAAGATCGGGGCACGGAAACTATGATCACGGATGGCTCTTTCGATACAAAGATGCCTGCCAATATAAATGACGAGGATATATCG
ATAAACTCAAAAACCTCAGCGGTCGATCGGTTAGGATTCACCAGTATGACCTTTGCTTGCATTACAATGACAGCCAGCGGGATCGGATTGAAGA
TGAATTTTGTACCTACGCGCCTAGACGCGCCAGTCTTGACCACAGAACAAAAAGAGCAGATGATCAAAGGATTTACTGACAAAGTCGATTCTAC
CTATCTCACATGTTCTGATCCCAATGATCCAAGATTGTGGTGGTTTTGTCGGGTATCACGACTATTATCGCTCAAGTTATGGTTGGCGACACAA
TATCCACTTCAGCGAAGGAAATCTACAAATCGAGTGCTTCCTCGAGGACAATCCTTGCGTACAGCTATGGCATTCTTAAATTTGGCGGAAGAAA
TATGGCAATATGAACCTTCAAAGGGATGGTTCTGGTTCTTTCACAGTTATGTGCCGTGGCATGCAATTGCGGTTGCTTTAGCTGAGCTTTGCGC
GGAACCAACTGGCCCTCTTGCCGATCAAGCTTGGGGGAGTATCGAGTTTCATTACAATAAATGGAGTGAACTCGTTGCAGATACAAAAGATGGG
GTGATTTGGCGGCCAGTCAAGAATTTAATGAAAAGAGCAAAAGCAGCAAGAAGGCGAGAAAGAGGTCTCAGTGGATCTCAAGCATCAACAACTC
CAACGGCAATTCTCAACCCTAATACATACAGCCCAGCTCTCGATCTAGGTGCTGAATTCTCACCAATCATGTCGGAGAATAATAACTACCGTCA
ACCTTCTACAATTGGGCAGGTTGACCCAATGGGGCAGAATATTTACGGGGGGATGAGCTTCGATCTTCCCATGCCCATGTGTTCTACCCCCTCA
GACACGAATATGGCATCCCCACTACCTATGCCGGACGGCTACACAAATCCGCACTGGAACGAATTCATATTTGGTTTAGGAACAGTTGGTAGTG
ATGGACTCCAAAATCCAGA
>Bcin05g01790(mrr1), partial sequence [organism=Botrytis cinerea, strain C12_S_E7_2]
CAGGCATCTGGCACGGAATGCATTTTCCCTACACGTGTTCGGAACCCAAGACCGAGACAAAGCAATCCAAAGTCTCGTGATTCTGAGCTATTAA
AAAGAATCTCTCACCTCGAAAGTTTGGTGAGCAGAATTGATGCCTCAAAACTCCTTGGGGAGGATGGTCAGGTAGAAGACGAGGTTCGGTCAAA
TCCACCATCATCAGGACCGCGACCTCATCGTTCTTTCACGTCTCCAGACCACCAATACAATGACAATCATCATGAGTTCGGAATAGTACCAGGT
CGTATGGCTCATCAATTTGCTGATTTTATTAAACGCCAAGAGACTGACTCGATTTACACCCATGATGGATTTTGGACAAGACTAAGTGAAGAAA
TTGATGGATTGAAACAGCTCATCGAGAGCCCCAGTGACGATGAAGATGAAAATTCTGGGTCCACTGCAACTTCGCCAGCATCCAAGTATGACTC
CCCTTCACAGTTTGTCTTTGATTCCAGGGTCACTTCCTCGAATGTCACAATTCCATATCCATCCCACGACCATACGAAAGTTTTATTCGATATT
TATTTCAAAAATGTACATCCGATGGTCGTCACTATGCACAAGTCAACAACGTACCTTTTCACAAACCCAAATTCTGAGTTACTCGATGAGAATA
CTGGCCGGCACAAATTCAAAAGTCTCGAGGCTAGCGCATTTTCTATGTACCTGGCTGCTGTGACGAGCATGACAAATGAAGAGTGCCTTGAGCT
TTTAAATGAAGAAAGACAGATTTTAGTAACAAGGTACAAAACTGCGACTGAGATTGCTCTTCAAGAATCAGATTTTCTTAATAGTGTAGAAATT
GTTACTTTACAAGCTTTAATAACGTATATCGTAAGTATTTCTCTCTCAGCATCTTGGATGATGGTTTTATGATGATGATGATGATGATGTTTAT
GTACTTTCACAGGCAGCTATGCGCGCTCATGATCGAAGTCGATCTATATGGGCTTTTATCGGTCTTGCAGTCCGCCTTGCCCGTGGCATCGGGC
TCCACAGGGATGGCACCGGGCTCCACAGAGATGGCAGCAAGGAACCTTTCGATTTGGAAATGCGAAGGAGAATATGGTGGACATTGATTGTACT
TGATACAAGAGCTTCCGAAGATCGGGGTACGGAAACCATGATCACGGATGGCTCTTTCGATACAAAGATGCCTGCCAATATAAATGACGAGGAT
ATATCGATAAACTCAAAAACTCTACCGGTCGATCGGTTAGGATTCACCAGTATGTCCTTTGCTTGCATTACAATGACAGTCAGCGGGATCGGAT
TGAGGATGAATTTTGTACCTACGCGCCTAGACGCGCCGGTCTTGACCACAGAACAAAAAGAGCAGATGATCAAAGGATTTACTGACAAAGTCGA
TTCTACCTATGTCACATGTTCTGATCCCAATGATCCAAGATTGTGGTGGTTTTCTCGGGTATCACGACTATTATCGCTCAAGTTATGGTTGGCG
ACACAATATCCACTTCAGCGAAGGAAATCTACAAATCGAGTGCTTCCTCGGGGACAATCCTTGCGTACAGCTATGGCATTCTTAAATTTGGCGG
AAGAAATATGGCAATATGAACCTTCAAAGGGATGGTTCTGGTTCTTTCAAAGTTATGTGCCGTGGCATGCAATTGCGGTTGCTTTAGCTGAGCT
TTGCGCGGAACCAACTGGCCCTCTTGCCGATCAAGCTTGGGGGAGCATCGAGTTTCATTACAATAAATGGAGTGAATTCGTTGCAGATACAAAA
GATGGGATGATTTGGCGGCCAGTCAAGAATTTAATGAAAAGAGCAAGAGCAGCAAGAAGGCGAGAAAGAGGTCTCAGTGAATCTCAAGCATCAA
CAACTCCAACGGCAATTCTCAACCCTAATACATACAGCCCAGCTCTCGATCTAGGTGCTGAATTCTCACCAATCATGTCGGAGAATAATAACTA
CCGTCAACCTTCTACAATTGGGCAGGTTGACCCAATGGGGCAGAATATTTACGGGGGGATGAACTTCGATCTTCCCATGCCCATGTGTTCTACC
CCCTCAGACACGAATATGGCATCCCCACTACCTATTCCGGACGGCTATACAAATCCGCACTGGAACGAATTCATATTTGGTTTAGGAACAGTTG
GTAGCGATGGACCCCAAAATCCAGA
>Bcin05g01790(mrr1), partial sequence [organism=Botrytis cinerea, strain C12_S_E7_4]
CAGGCATCTGGCACGGAATGTATTTTCCCTACACGTGTTCGGAACCCAAGACCGAGACAAAGCAATCCAAAGTCTCGTGATTCTGAGCTATTAA
AAAGAATCTCTCATCTCGAAAGTTTGGTGAGCAGAATTGATGCCTCAAAACTCCTTGGGGAGGATGGTCAGGTAGAATACGAGGTTCGGTCAAA
TCCGCCATCATCAGGACCGCGACCTCATCGTTCTTTCACGTCTCCAGACCATCATGAGTTTGGAATAGTACCAGGTCGTATGGCTCATCAATTT
GCTGATTTTATTAAACGCCAAGAGAACGACTCGATTTACACTCATGATGGATTTTGGACAAGACTAAGTGAAGAAATTGATGGATTGAAACAGC
TCATCGAGAGCCCCAGTGACGATGAAGATGAAAATTCTGGGTCCACTGCAACTTCGCCAGCATCCAAGTATGACTCTCCTTCACAGTTTGTCTT
TGATTCCAGGGTCACTTCCTCGAATGTCATAATTCCATATCCATCCCACGACCATACCAAAGTTTTATTCGATATTTATTTCAAAAATGTACAT
CCGATGGTCGTCACTATGCACAAGCCAACAACGTACCTTTTCACAAACCCAAATTCTGAGTTACTCGATGAGAATACTGGCCGGCACAAATTCA
AAAGTCTCGAGGCTAGCGTGTTTGCTATGTACCTGGCTGCTGTGACGAGCATGACAAATGAAGAGTGCCTTGAGCTTTTAAATGAAGAAAGAAA
TATTTTAGTAACACGGTACAAAACTGCGACTGAGATTGCTCTTCAAGAATCAGATTTTCTTAATAGTGTTGAAATTGTTACTTTACAAGCTTTA
ATAACGTATATCGTAAGTGTTTCTCTCTAAGCATCTTGGATGATGGTTTTATGATGATGATGATGATGATGTTTATGTACTTTCACAGGCAGCT
ATGCGCACTCATGATCGAAGTCGATCTATATGGGCATTTATCGGTCTTGCAGTCCGCCTTGCCCGTGGCATCGGGCTCCACAGAGATGGCAGCC
AGCAACCTTTCGATTTGGAAATGCGAAGGAGAGTGTGGTGGACATTGATTGTACTTGATACAAGAGCTTCCGAAGATCGGGGTACAGAAACCAT
GATCACTGATGGCTCTTTCGATACAAAGATGCCTGCCAACATAAACGACGAGGATATGATGATAAACTCAAAAAGTCTACCGGTCGATCGGATA
GGGATCACCAGTATGACCTTTGCTTGCATTACAATGACAGTCAGCGGGATCGGATTGAGGATGAATTTTGTACCTACGCGCCTAGACGCGCCGG

TCTTGACCACAGAACAAAAAGAGCAGATGATCAAAGGATTTACTGACAAAATCGATTCTACCTATCTCGCAGGTTCTGATCCCAATGATCCAAG
ATTGTGGTGGTATTGTCGGATATCACGACTATTATCGCTCAAGTTATGGCTGGTGACACAATATCCACTTCAACGAAGGAAATCTACAAATCGA
GTGCTTCCTCGAGGACAATCCTTGCGTACAGCTATGGCATTCCTAAATTTGGCGGAAGAAATATGGCAATATGAACCTTCAAAGGGATGGTTCT
GGTTCTTTCAAAGTTATGTGCCGTGGCATGTAATTGCGGTTGCTTTAGCTGAGCTTTGTGCGGAACCAACTGGCCCTCTTGCCGATCAAGCTTG
GGAGGGCATTGAGTTTCACTACAATAAATGGAGTGAATTCGTTGCAGATACAAAAGATGGGATGATTTGGCGACCAGTCAGGAATTTAATGAAA
AGAGCAAGAGCAGCAAGAAGGCGAGAAAGAGGTCTCAGTGAATCTCAAGCATCAACAACTCCAACGGCAATTCGCAACCCTAATACATACAGCC
CAGCTCTCAATCTAGCTGCTGGATGCTCCCCGATCATGTCGGAGAACAATAACTACTGTCAATCTTCTACAATTGGACAGGTTGACCCAATGGG
GCAGAATATTTACGGGGGAATGGGCTTCGATCTTCCCATGCCCATGGGTTCTACCCCCTCAGACACGAATATGGCATCCCCACTACCTATTCCG
GACGGCTATACAAATCCGCACTGGAACGAATTCATATTTGGTTTAGGACCAGTTTGTAGCGATGGACCCCAAAATCCAGA
>Bcin05g01790(mrr1), partial sequence [organism=Botrytis cinerea, strain D08_H_8_6]
CAGGCATCTGGCACGGAATGCATTTTCCCTACACGTGTTCGGAACCCAAGACCGAGACAAAGCAATCCAAAGTCTCGTGATTCTGAGCTATTAA
AAAGAATCTCTCACCTCGAAAGTTTGGTGAGCAGAATTGATGCCTCAAAACTCCTTGGGGAGGATGGTCAGGTAGAAGACGAGGTTCGGTCAAA
TCCACCATCGTCAGGACCGCGACCTCATCGTTCTTTCACGTCTCCAGACCACCAATACAATGACAATCATCATGAGTTCGGAATAGTACCAGGT
CGTATGGCTCATCAATTTGCTGATTTTATCAAACGCCAAGAGACCGACTCGATTTACACCCATGATGGATTTTGGACAAGACTAAGTGAAGAAA
TTGATGGATTGAAACAGCTCATCGAGAGCCCCAGTGACGATGAAGATGAAAATTCTGGGTCCACTGCAACTTCGCCAGCATCCAAGTATGACTC
CCCTTCACAGTTTGTCTTTGATTCCAGGGTCACTTCCTCCAATGTCACAATTCCATATCCATCCCACGACCATACGAAAGTTTTATTCGATATT
TATTTCAAAAATGTACATCCGATGGTCGTCACTATGCACAAGCCAACAACGTACCTTTTCACAAACCCAAATTCTGAGTTACTCGATGAGAATA
CTGGCCGGCACAAATTCAAAAGTCTCGAGGCTAGCGCATTTTCTATGTACCTGGCTGCTGTGACGAGCATGACAAATGAAGAGTGCCTTGAGCT
TTTAAATGAAGAAAGACAGATTTTAGTAACAAGGTACAAAACTGCGACTGAGATTGCTCTTCAAGAATCAGATTTTCTTAATAGTGTAGGAATT
GTTACTTTACAAGCTTTGATAACGTATATCGTAAGTATTTCTCTCTCAGCATCTTGAATGATGGTTTTATGATGATGATGATGATGTTTATGTA
CTTTCACAGGCAGCTATGCGCGCTCATGATCGAAGTCGATCTACATGGACATTTATCGGTCTTGCAGTCCGCCTTGCCCGTGGCATCGGGCTCC
ACAGGGATGGCACCGGGCTCCACAGAGATGGCAGCAAGCAACCTTTCGATTTGGAAATGCGAAGGAGACTATGGTGGACATTGATTGTACTTGA
TACAAGAGCTTCCGAAGATCGGGGTACGGAAACTATGATCACTGATGGCTCTTTCGATACAAAGATGCCTGCCAATATAAATGATGAGGATATA
TCGATAAACTCAAAAACTCTACCGGTCGATCGGCTAGGATTAACCAGTATGACCTTTGCTTGCATTACAATGACAGTCAGCGGGATCGGATTGA
GGATGAATTTTGTACCTACGCGCCTAGACGCGCCGGTCTTGACCACAGAACAAAAAGAGCAGATGATCAAAGGATTTACTGACAAAATCGATTC
TACCTATGTCACATGTTCTGATCCCAATGATCCAAGATTGTGGTGGTTTTGTCGGGTATCACGACTATTATCGCTCAAGTTATGGTTGGCGACA
CAATATCCACTTCAGCGAAGGAAATCTACAAATCGAGTGCTTCCTCGAGGACAATCCTTGCGTACAGCTATGGCATTCTTAAATTTGGCGGAAG
AAATATGGCAATATGAACCTTCAAAGGGATGGTTCTGGTTCTTTCAAAGTTATGTGCCGTGGCATGCAATTGCGGTTGCTTTAGCTGAGCTTTG
TGCGGAACCAACTGGCCCTCTTGCCGATCAAGCTTGGGGGAGCATCGAGTTTCATTACAATAAATGGAGTGAATTCGTTGCAGATACAAAAGAT
GGGATGATTTGGCGGCCAGTCAAGAATTTAATGAAAAGAGCAAAAGCAGCAAGAAGGCGAGAAAGAGGTCTCAGTGAATCTCAAGCATCAACAA
CTCCAACGGCAATTCTCAACCCTAATACATACAGCCCAGCTCTCGATCTAGGTGCTGAATTCTCACCAATCATGTCGGAGAATAATAACTACCG
TCAACCTTCTACAATTGGGCAGGTTGACCCAATGGGGCAGAATATTTACGGGGGGATGAGCTTCGATCTTCCCATGCCCATGTGTTCTACCCCC
TCAGACACGAATATGGCATCCCCACTACCTATTCCGGACGGTTATACAAATCCGCACTGGAACGAATTCATATTTGGTTTAGGAACAGTTGGTA
GCGATGGACCCCAGAATCCAGA
>Bcin05g01790(mrr1), partial sequence [organism=Botrytis cinerea, strain D09_K_2_3]
CAGGCATCTGGCACGGAATGCATTTTCCCTACACGTGTTCGGAACCCAAGACCGAGACAAAGCAATCCAAAGTCTCGTGATTCTGAGCTATTAA
AAAGAATCTCTCACCTCGAAAGTTTGGTGAGCAGAATTGATGCCTCAAAACTCCTTGGGGAGGATGGTCAGGTAGAAGACGAGGTTCGGTCAAA
TCCACCATCATCAGGACCGCGACCTCATCGTTCTTTCACGTCTCCAGACCACCAATACAATGACAATCATCATGAGTTCGGAATAGTACCGGGT
CGTATGGCTCATCAATTTGCTGATTTTATTAAACGCCAAGAGACTGACTCGATTTACACCCATGATGGATTTTGGACAAGACTAAGTGAAGAAA
TTGATGGATTGAAACAGCTCATCGAGAGCCCCAGTGACGATGAAGATGAAAATTCTGGGTCCACTGCAACTTCGCCAGCATCCAAGTATGACTC
CCCTTCACAGTTTGTCTTTGATTCCAGGGTCACTTCCTCGAATGTCACAATTCCATATCCATCCCACGACCATACGAAAGTTTTATTCGATATT
TATTTCAAAAATGTACATCCGATGGTCGTCACTATGCACAAGTCAACAACGTACCTTTTCACAAACCCAAATTCTGAGTTACTCGATGAGAATA
CTGGCCGGCACAAATTCAAAAGTCTCGAGGCTAGCGCATTTTCTATGTACCTGGCTGCTGTGACGAGCATGACAAATGAAGAGTGCCTTGAGCT
TTTAAATGAAGAAAGACAGATTTTAGTAACAAGGTACAAAACTGCGACTGAGATTGCTCTTCAAGAATCAGATTTTCTTAATAGTGTAGAAATT
GTTACTTTACAAGCTTTAATAACGTATATCGCAGCTATGCGCGCTCATGATCGAAGTCGATCTATATGGGCTTTTATCGGTCTTGCAGTCCGCC
TTGCCCGTGGCATCGGGCTCCACAGGGATGGCACCGGGCTCCACAGAGATGGCAGCAAGGAACCTTTCGATTTGGAAATGCGAAGGAGAATATG
GTGGACATTGATTGTACTTGATACAAGAGCTTCCGAAGATCGGGGTACGGAAACCATGATCACGGATGGCTCTTTCGATACAAAGATGCCTGCC
AATATAAATGACGAGGATATATCGATAAACTCAAAAACTCTACCGGTCGATCGGTTAGGATTCACCAGTATGACCTTTGCTTGCATTACAATGA
CAGTCAGCGGGATCGGATTGAGGATGAATTTTGTACCTACGCGCCTAGACGCGCCGGTCTTGACCACAGAACAAAAAGAGCAGATGATCAAAGG
ATTTACTGACAAAGTCGATTCCACCTATGTCACATGTTCTGATCCCAATGATCCAAGATTGTGGTGGTTTTCTCGGGTATCACGACTATTATCG
CTCAAGTTATGGTTGGCGACACAATATCCACTTCAGCGAAGGAAATCTACAAATCGAGTGCTTCCTCGGGGACAATCCTTGCGTACAGCTATGG
CATTCTTAAATTTGGCGGAAGAAATATGGCAATATGAACCTTCAAAGGGATGGTTCTGGTTCTTTCAAAGTTATGTGCCGTGGCATGCAATTGC
GGTTGCTTTAGCTGAGCTTTGCGCGGAACCAACTGGCCCTCTTGCCGATCAAGCTTGGGGGAGCATCGAGTTTCATTACAATAAATGGAGTGAA
TTCGTTGCAGATACAAAAGATGGGATGATTTGGCGGCCAGTCAAGAATTTAATGAAAAGAGCAAGAGCAGCAAGAAGGCGAGAAAGAGGTCTCA
GTGAATCTCAAGCATCAACAACTCCAACGGCAATTCTCAACCCTAATACATACAGCCCAGCTCTCGATCTAGGTGCTGAATTCTCACCAATCAT
GTCGGAGAATAATAACTACCGTCAACCTTCTACAATTGGGCAGGTTGACCCAATGGGGCAGAATATTTACGGGGGGATGAACTTCGATCTTCCC
ATGCCCATGTGTTCTACCCCCTCAGACACGAATATGGCATCCCCACTACCTATTCCGGACGGCTATACAAATCCGCACTGGAACGAATTCATAT

```
TTGGTTTAGGAACAGTTGGTAGCGATGGACCCCAAAATCCAGA
>Bcin05g01790(mrr1), partial sequence [organism=Botrytis cinerea, strain D09_K_4_1]
CAGGCATCTGGCACGGAATGCATTTTCCCTACACGTGTTCGGAACCCAAGACCGAGACAAAGCAATCCAAAGTCTCGTGATTCTGAGCTATTAA
AAAGAATCTCTCACCTCGAAAGTTTGGTGAGCAGAATTGATGCCTCAAAACTCCTTGGGGAGGATGGTCAGGTAGAAGACGAGGTTCGGTCAAA
TCCACCATCATCAGGACCGCGACCTCATCGTTCTTTCACGTCTCCAGACCACCAATACAATGACAATCATCATGAGTTCGGAATAGTACCAGGT
CGTATGGCTCATCAATTTGCTGATTTTATTAAACGCCAAGAGACCGACTCGATTTACACCCATGATGGATTTTGGACAAGACTAAGTGAAGAAA
TTGATGGATTGAAACAGCTCATCGAGAGCCCCAGTGACGATGAAGATGAAAATTCTGGGTCCACTGCAACTTCGCCAGCATCCAAGTATGACTC
CCCTTCACAGTTTGTCTTTGATTCCAGGGTCACTTCCTCGAATGTCACAATTCCATATCCATCCCACGACCATACGAAAGTTTTATTCGATATT
TATTTCAAAAATGTACATCCGATGGTCGTCACTATGCACAAGTCAACAACGTACCTTTTCACAAACCCAAATTCTGAGTTACTCGATGAGAATA
CTGGCCGGCACAAATTCAAAAGTCTCGAGGCTAGCTCATTTTCTATGTACCTGGCTGCTGTGACGAGCATGACAAATGAAGAATGCCTTGAGCT
TTTAAATGAAGAAAGACAGATTTTAGTAACAAGGTACAAAACTGCGACTGAGATTGCTCTTCAAGAATCAGATTTTCTTAATAGTGTAGAAATT
GTTACTTTACAAGCTTTAATAACGTATATCGCAGCTATGCGCGCTCATGATCGAAGTCGATCTATATGGGCATTTATCGGTCTTGCAGTCCGCC
TTGCCCGTGGCATCGGGCTCCACAGGGATGGCACCGGGCTCCACAGAGATGGCAGCAAGGAACCTTTCGATTTGGAAATGCGAAGGAGAATATG
GTGGACATTGATTGTACTTGATACAAGAGCTTCCGAAGATCGGGGTACGGAAACTATGATCACGGATGGCTCTTTCGATACAAAGATGCCTGCC
AATATAAATGACGAGGATATAACGATAAACTCAAAAACTCTACCGGTCGATCGGTTAGGATTCACCAGTATGACCTTTGCTTGCATTACAATGA
CAGTCAGCGGGATCGGATTGAGGATGAATTTTGTACCTACGCGCCTAGACGCGCCGGTCTTGACCACAGAACAAAAAGAGCAGATGATCAAAGG
ATTTACTGACAAAGTTGATTCTACCTATGTCACATGTTCTGATCCCAATGATCCAAGATTGTGGTGGTTTTCTCGGGTATCACGACTATTATCG
CTCAAGTTATGGTTGGCGACACAATATCCACTTCAGCGAAGGAAATCTACAAATCGAGTGCTTCCTCGGGGACAATCCTTGCGTACAGCTATGG
CATTCTTAAATTTGGCGGAAGAAATATGGCAATATGAACCTTCAAAGGGATGGTCCTGGTTCTTCCAAAGTTATGTGCCGTGGCATGCAATTGC
GGTTGCTTTAGCTGAGCTTTGCGCGGAACCAACTGGCCCTCTTGCCGATCAAGCTTGGGGGAGTATCGAGTTTCATTACAATAAATGGAGTGAA
TTCGTTGCAGATACAAAAGATGGGATGATTTGGCGGCCAGTCAAGAATTTAATGAAAAGAGCAAAAGCAGCAAGAAGGCGAGAAAGAGGTCTCA
GTGAATCTCAAGCATCAACAACTCCAACGGCAATTCTCAATCCTAATACATACAGCCCAGCTCTCGGTCTAGGTGCTGAATTCTCACCAATCAT
GTCGGAGAATAATAACTACCGTCAACCTTCTACAATTGGGCAGGTTGATCCAATGGGGCAGAATATTTACGGGGGGATGAGCTTCGATCTTCCC
ATGCCCATGTGTTCTACCCCCTCAGACACGAATATGGCATCCCCACTACCTATTCCGGACGGTTATACAAATCCGCACTGGAACGAATTCATAT
TTGGTTTAGGAACAGTTGGTAGCGATGGACCCCAGAATCCAGA
>Bcin05g01790(mrr1), partial sequence [organism=Botrytis cinerea, strain D09_K_4_2]
CAGGCATCTGGCACGGAATGCATTTTCCCTACACGTGTTCGGAACCCAAGACCGAGACAAAGCAATCCAAAGTCTCGTGATTCTGAGCTATTAA
AAAGAATCTCTCACCTCGAAAGTTTGGTGAGCAGAATTGATGCCTCAAAACTCCTTGGGGAGGATGGTCAGGTAGAAGACGAGGTTCGGTCAAA
TCCACCATCGTCAGGACCGCGACCTCATCGTTCTTTCACGTCTCCAGACCACCAATACAATGACAATCATCATGAGTTCGGAATAGTACCAGGT
CGTATGGCTCATCAATTTGCTGATTTTATCAAACGCCAAGAGACCGACTCGATTTACACCCATGATGGATTTTGGACAAGACTAAGTGAAGAAA
TTGATGGATTGAAACAGCTCATCGAGAGCCCCAGTGACGATGAAGATGAAAATTCTGGGTCCACTGCAACTTCGCCAGCATCCAAGTATGACTC
CCCTTCACAGTTTGTCTTTGATTCCAGGGTCACTTCCTCCAATGTCACAATTCCATATCCATCCCACGACCATACGAAAGTTTTATTCGATATT
TATTTCAAAAATGTACATCCGATGGTCGTCACTATGCACAAGCCAACAACGTACCTTTTCACAAACCCAAATTCTGAGTTACTCGATGAGAATA
CTGGCCGGCACAAATTCAAAAGTCTCGAGGCTAGCGCATTTTCTATGTACCTGGCTGCTGTGACGAGCATGACAAATGAAGAGTGCCTTGAGCT
TTTAAATGAAGAAAGACAGATTTTAGTAACAAGGTACAAAACTGCGACTGAGATTGCTCTTCAAGAATCAGATTTTCTTAATAGTGTAGAAATT
GTTACTTTACAAGCTTTGATAACGTATATCGCAGCTATGCGCGCTCATGATCGAAGTCGATCTACATGGACATTTATCGGTCTTGCAGTCCGCC
TTGCCCGTGGCATCGGGCTCCACAGGGATGGCACCGGGCTCCACAGAGATGGCAGCAAGCAACCTTTCGATTTGGAAATGCGAAGGAGACTATG
GTGGACATTGATTGTACTTGATACAAGAGCTTCCGAAGATCGGGGTACGGAAACTATGATCACTGATGGCTCTTTCGATACAAAGATGCCTGCC
AATATAAATGATGAGGATATATCGATAAACTCAAAAACTCTACCGGTCGATCGGCTAGGATTAACCAGTATGACCTTTGCTTGCATTACAATGA
CAGTCAGCGGGATCGGATTGAGGATGAATTTTGTACCTACGCGCCTAGACGCGCCGGTCTTGACCACAGAACAAAAAGAGCAGATGATCAAAGG
ATTTACTGACAAAATCGATTCTACCTATGTCACATGTTCTGATCCCAATGATCCAAGATTGTGGTGGTTTTGTCGGGTATCACGACTATTATCG
CTCAAGTTATGGTTGGCGACACAATATCCACTTCAGCGAAGGAAATCTACAAATCGAGTGCTTCCTCGAGGACAATCCTTGCGTACAGCTATGG
CATTCTTAAATTTGGCGGAAGAAATATGGCAATATGAACCTTCAAAGGGATGGTTCCGGTTCTTTCAAAGTTATGTGCCGTGGCATGCAATTGC
GGTTGCTTTAGCTGAGCTTTGTGCGGAACCAACTGGCCCTCTTGCCGATCAAGCTTGGGGGAGCATCGAGTTTCATTACAATAAATGGAGTGAA
TTCGTTGCAGATACAAAAGATGGGATGATTTGGCGGCCAGTCAAGAATTTAATGAAAAGAGCAAAAGCAGCAAGAAGGCGAGAAAGAGGTCTCA
GTGAATCTCAAGCATCAACAACTCCAACGGCAATTCTCAACCCTAATACATACAGCCCAGCTCTCGATCTAGGTGCTGAATTCTCACCAATCAT
GTCGGAGAATAATAACTACCGTCAACCTTCTACCATTGGGCAGGTTGACCCAATGGGGCAGAATATTTACGGGGGGATGAGCTTCGATCTTCCC
ATGCCCATGTGTTCTACCCCCTCAGACACGAATATGGCATCCCCACTACCTATTCCGGACGGTTATACAAATCCGCACTGGAACGAATTCATAT
TTGGTTTAGGAACAGTTGGTAGCGATGGACCCCAGAATCCAGA
>Bcin05g01790(mrr1), partial sequence [organism=Botrytis cinerea, strain D10_B_F1_6]
CAGGCATCTGGCACGGAATGCATTTTCCCTACACGTGTTCGGAACCCAAGACCGAGACAAAGCAATCCAAAGTCTCGTGATTCTGAGCTATTAA
AAAGAATCTCTCACCTCGAAAGTTTGGTGAGCAGAATTGATGCCTCAAAACTCCTTGGGGAGGATGGTCAGGTAGAAGACGAGGTTCGGTCAAA
TCCACCATCATCAGGACCGCGACCTCATCGTTCTTTCACGTCTCCAGACCACCAATACAATGACAATCATCATGAGTTCGGAATAGTACCAGGT
CGTATGGCTCATCAATTTGCTGATTTTATTAAACGCCAAGAGACCGACTCGATTTACACCCATGATGGATTTTGGACAAGACTAAGTGAAGAAA
TTGATGGATTGAAACAGCTCATCGAGAGCCCCAGTGACGATGAAGATGAAAATTCTGGGTCCACTGCAACTTCGCCAGCATCCAAGTATGACTC
CCCTTCACAGTTTGTCTTTGATTCCAGGGTCACTTCCTCGAATGTCACAATTCCATATCCATCCCACGACCATACGAAAGTTTTATTCGATATT
TATTTCAAAAATGTACATCCGATGGTCGTCACTATGCACAAGTCAACAACGTACCTTTTCACAAACCCAAATTCTGAGTTACTCGATGAGAATA
```

CTGGCCGGCACAAATTCAAAAGTCTCGAGGCTAGCTCATTTTCTATGTACCTGGCTGCTGTGACGAGCATGACAAATGAAGAATGCCTTGAGCT
TTTAAATGAAGAAAGACAGATTTTAGTAACAAGGTACAAAACTGCGACTGAGATTGCTCTTCAAGAATCAGATTTTCTTAATAGTGTAGAAATT
GTTACTTTACAAGCTTTAATAACGTATATCTATGCGCGCTCATGATCGAAGTCGATCTATATGGGCATTTATCGGTCTTGCAGTCCGCCTTGCC
CGTGGCATCGGGCTCCACAGGGATGGCACCGGGCTCCACAGAGATGGCAGCAAGGAACCTTTCGATTTGGAAATGCGAAGGAGAATATGGTGGA
CATTGATTGTACTTGATACAAGAGCTTCCGAAGATCGGGGTACGGAAACTATGATCACGGATGGCTCTTTCGATACAAAGATGCCTGCCAATAT
AAATGACGAGGATATAACGATAAACTCAAAAACTCTACCGGTCGATCGGTTAGGATTCACCAGTATGACCTTTGCTTGCATTACAATGACAGTC
AGCGGGATCGGATTGAGGATGAATATTGTACCTACGCGCCTAGACGCGCCGGTCTTGACCACAGAACAAAAAGAGCAGATGATCAAAGGATTTA
CTGACAAAGTTGATTCTACCTATGTCACATGTTCTGATCCCAATGATCCAAGATTGTGGTGGTTTTCTCGGGTATCACGACTATTATCGCTCAA
GTTATGGTTGGCGACACAATATCCACTTCAGCGAAGGAAATCTACAAATCGAGTGCTTCCTCGGGGACAATCCTTGCGTACAGCTATGGCATTC
TTAAATTTGGCGGAAGAAATATGGCAATATGAACCTTCAAAGGGATGGTCCTGGTTCTTCCAAAGTTATGTGCCGTGGCATGCAATTGCGGTTG
CTTTAGCTGAGCTTTGCGCGGAACCAACTGGCCCTCTTGCCGATCAAGCTTGGGGGAGTATCGAGTTTCATTACAATAAATGGAGTGAATTCGT
TGCAGATACAAAAGATGGGATGATTTGGCGGCCAGTCAAGAATTTAATGAAAAGAGCAAAAGCAGCAAGAAGGCGAGAAAGAGGTCTCAGTGAA
TCTCAAGCATCAACAACTCCAACGGCAATTCTCAATCCTAATACATACAGCCCAGCTCTCGGTCTAGGTGCTGAATTCTCACCAATCATGTCGG
AGAATAATAACTACCGTCAACCTTCTACAATTGGGCAGGTTGATCCAATGGGGCAGAATATTTACGGGGGGATGAGCTTCGATCTTCCCATGCC
CATGTGTTCTACCCCCTCAGACACGAATATGGCATCCCCACTACCTATTCCGGACGGTTATACAAATCCGCACTGGAACGAATTCATATTTGGT
TTAGGAACAGTTGGTAGCGATGGACCCCAGAATCCAGA
>Bcin05g01790(mrr1), partial sequence [organism=Botrytis cinerea, strain D10_B_F3_5]
CAGGCATCTGGCACGGAATGCATTTTCCCTACACGTGTTCGGAACCCAAGACCGAGACAAAGCAATCCAAAGTCTCGTGATTCTGAGCTATTAA
AAAGAATCTCTCACCTCGAAAGTTTGGTGAGCAGAATTGATGCCTCAAAACTCCTTGGGGAGGATGGTCAGGTAGAAGACGAGGTTCGGTCAAA
TCCACCATCATCAGGACCGCGACCTCATCGTTCTTTCACGTCTCCAGACCACCAATACAATGACAATCATCATGAGTTCGGAATAGTACCAGGT
CGTATGGCTCATCAATTTGCTGATTTTATTAAACGCCAAGAGACCGACTCGATTTACACCCATGATGGATTTTGGACAAGACTAAGTGAAGAAA
TTGATGGATTGAAACAGCTCATCGAGAGCCCCAGTGACGATGAAGATGAAAATTCTGGGTCCACTGCAACTTCGCCAGCATCCAAGTATGACTC
CCCTTCACAGTTTGTCTTTGATTCCAGGGTCACTTCCTCGAATGTCACAATTCCATATCCATCCCACGACCATACGAAAGTTTTATTCGATATT
TATTTCAAAAATGTACATCCGATGGTCGTCACTATGCACAAGTCAACAACGTACCTTTTCACAAACCCAAATTCTGAGTTACTCGATGAGAATA
CTGGCCGGCACAAATTCAAAAGTCTCGAGGCTAGCTCATTTTCTATGTACCTGGCTGCTGTGACGAGCATGACAAATGAAGAATGCCTTGAGCT
TTTAAATGAAGAAAGACAGATTTTAGTAACAAGGTACAAAACTGCGACTGAGATTGCTCTTCAAGAATCAGATTTTCTTAATAGTGTAGAAATT
GTTACTTTACAAGCTTTAATAACGTATATCGCAGCTATGCGCGCTCATGATCGAAGTCGATCTATATGGGCATTTATCGGTCTTGCAGTCCGCC
TTGCCCGTGGCATCGGGCTCCACAGGGATGGCACCGGGCTCCACAGAGATGGCAGCAAGGAACCTTTCGATTTGGAAATGCGAAGGAGAATATG
GTGGACATTGATTGTACTTGATACAAGAGCTTCCGAAGATCGGGGTACGGAAACCATGATCACGGATGGCTCTTTCGATACAAAGATGCCTGCC
AATATAAATGACGAGGATATATCGATAAACTCAAAAACTCTACCGGTCGATCGGTTAGGATTCACCAGTATGACCTTTGCTTGCATTACAATGA
CAGTCAGCGGGATCGGATTGAGGATGAATTTTGTACCTACGCGCCTAGACGCGCCGGTCTTGACCACAGAACAAAAAGAGCAGATGATCAAAGG
ATTTACTGACAAAGTCGATTCTACCTATGCATGTTCTGATCCCAATGATCCAAGATTGTGGTGGTTTTCTCGGGTATCACGACTATTATCGCTC
AAGTTATGGTTGGCGACACAATATCCACTTCAGCGAAGGAAATCTACAAATCGAGTGCTTCCTCGAGGACAATCCTTGCGTACAGCTATGGCAT
TCTTAAATTTGGCGGAAGAAATATGGCAGTATGAACCTTCAAAGGGATGGTCCTGGTTCTTCCAAAGTTATGTGCCGTGGCATGCAATTGCGGT
TGCTTTAGCTGAGCTTTGCGCGGAACCAACTGGCCCTCTTGCCGATCAAGCTTGGGGGAGTATCGAGTTTCATTACAATAAATGGAGTGAATTC
GTTGCAGATACAAAAGATGGGATGATTTGGCGGCCAGTCAAGAATTTAATGAAAAGAGCAAAAGCAGCAAGAAGGCGAGAAAGAGGTCTCAGTG
AATCTCAAGCATCAACAACTCCAACGGCAATTCTCAATCCTAATACATACAGCCCAGCTCTCGGTCTAGGTGCTGAATTCTCACCAATCATGTC
GGAGAATAATAACTACCGTCAACCTTCTACAATTGGGCAGGTTGATCCAATGGGGCAGAATATTTACGGGGGGATGAGCTTCGATCTTCCCATG
CCCATGTGTTCTACCCCCTCAGACACGAATATGGCATCCCCACTACCTATTCCGGACGGTTATACAAATCCGCACTGGAACGAATTCATATTTG
GTTTAGGAACAGTTGGTAGCGATGGACCCCAGAATCCAGA
>Bcin05g01790(mrr1), partial sequence [organism=Botrytis cinerea, strain D10_B_S3_16]
CAGGCATCTGGCACGGAATGTATTTTCCCTACACGTGTTCGGAACCCAAGACCGAGACAAAGCAATCCAAAGTCTCGTGATTCTGAGCTATTAA
AAAGAATCTCTCATCTCGAAAGTTTGGTGAGCAGAATTGATGCCTCAAAACTCCTTGGGGAGGATGGTCAGGTAGAAGACGAGGTTCGGTCAAA
TCCGCCATCATCAGGACCGCGACCTCATCGTTCTTTCACGTCTCCAGACCACCAATACAATGACAATCATCATGAGTTTGGAATAGTACCAGGT
CGTATGGCTCATCAATTTGCTGATTTTATTAAACGCCAAGAGACCGACTCGATTTACACTCATGATGGATTTTGGACAAGACTAAGTGAAGAAA
TTGATGGATTGAAACAGCTCATCGAGAGCCCCAGTGACGACGAAGATGAAAATTCTGGGTCCACTGCAACTTCGCCAGCATCCAAGTATGACTC
TCCTTCACAGTTTGTCTTTGATTCCAGGGTCACTTCCTCGAATGTCATAATTCCATATCCATCCCACGACCATACCAAAGTTTTATTCGATATT
TATTTCAAAAATGTACATCCGACGGTCGTCACTATGCACAAGCCAACAACGTACCTTTTCACAAACCCAAATTCTGAGTTACTCGATGAGAATA
CTGGCCGGCACAAATTCAAAAGTCTCGAGGCTAGCGTGTTTGCTATGTACCTGGCTGCTGTGACGAGCATGACAAATGAAGAGTGCCTTGAGCT
TTTAAATGAAGAAAGAAATATTTTAGTAACACGGTACAAAACTGCGACTGAGATTGCTCTTCAAGAATCAGATTTTCTTAATAGTGTTGAAATT
GTTACTTTACAAGCTTTAATAACGTATATCGCAGCTATGCGCACTCATGATCGAAGTCGATCTATATGGGCATTTATCGGTCTTGCAGTCCGCC
TTGCCCGTGGCATCGGGCTCCACAGAGATGGCAGCCAGCAACCTTTCGATTTGGAAATGCGAAGGAGAGTGTGGTGGACATTGATTGTACTTGA
TACAAGAGCTTCCGAAGATCGGGGTACAGAAACCATGATCACTGATGGCTCTTTCGATACAAAGATGCCTGCCAACATAAACGACGAGGATATG
ATGATAAACTCAAAAAGTCTACCGGTCGATCGGATAGGGATCACCAGTATGACCTTTGCTTGCATTACAATGACAGTCAGCGGGATCGGATTGA
GGATGAATTTTGTACCTACGCGCCTAGACGCGCCGGTCTTGACCACAGAACAAAAAGAGCAGATGATCAAAGGATTTACTGACAAAATCGATTC
TACCTATCTCGCAGGTTCTGATCCCAATGATCCAAGATTGTGGTGGTATTGTCGGATATCACGACTATTATCGCTCAAGTTATGGCTGGTGACA
CAATATCCACTTCAACGAAGGAAATCTACAAATCGAGTGCTTCCTCGAGGACAATCCTTGCGTACAGCTATGGCATTCCTAAATTTGGCGGAAG

```
AAATATGGCAATATGAACCTTCAAAGGGATGGTTCTGGTTCTTTCAAAGTTATGTGCCGTGGCATGTAATTGCGGTTGCTTTAGCTGAGCTTTG
TGCGGAACCAACTGGCCCTCTTGCCGATCAAGCTTGGGAGGGCATTGAGTTTCACTACAATAAATGGAGTGAATTCGTTGCAGATACAAAAGAT
GGGATGATTTGGCGACCAGTCAGGAATTTAATGAAAAGAGCAAGAGCAGCAAGAAGGCGAGAAAGAGGTCTCAGTGAATCTCAAGCATCAACAA
CTCCAACGGCAATTCGCAACCCTAATACATACAGCCCAGCTCTCAATCTAGCTGCTGGATGCTCCCCGATCATGTCGGAGAACAATAACTACTG
TCAATCTTCTACAATTGGACAGGTTGACCCAATGGGGCAGAATATTTACGGGGGAATGGGCTTCGATCTTCCCATGCCCATGGGTTCTACCCCC
TCAGACACGAATATGGCATCCCCACTACCTATTCCGGACGGCTATACAAATCCGCACTGGAACGAATTCATATTTGGTTTAGGAACAGTTTGTA
GCGATGGACCCCAAAATCCAGA
>Bcin05g01790(mrr1), partial sequence [organism=Botrytis cinerea, strain D10_K_S11_6]
CAGGCATCTGGCACGGAATGTATTTTCCCTACACGTGTTCGGAACCCAAGACCGAGACAAAGCAATCCAAAGTCTCGTGATTCTGAGCTATTAA
AAAGAATCTCTCATCTCGAAAGTTTGGTGAGCAGAATTGATGCCTCAAAACTCCTTGGGGAGGATGGTCAGGTAGAAGACGAGGTTCGGTCAAA
TCCGCCATCATCAGGACCGCGACCTCATCGTTCTTTCACGTCTCCAGACCACCAATACAATGACAATCATCATGAGTTTGGAATAGTACCAGGT
CGTATGGCTCATCAATTTGCTGATTTTATTAAACGCCAAGAGACCGACTCGATTTACACTCATGATGGATTTTGGACAAGACTAAGTGAAGAAA
TTGATGGATTGAAACAGCTCATCGAGAGCCCCAGTGACGACGAAGATGAAAATTCTGGGTCCACTGCAACTTCGCCAGCATCCAAGTATGACTC
TCCTTCACAGTTTGTCTTTGATTCCAGGGTCACTTCCTCGAATGTCATAATTCCATATCCATCCCACGACCATACCAAAGTTTTATTCGATATT
TATTTCAAAAATGTACATCCGACGGTCGTCACTATGCACAAGCCAACAACGTACCTTTTCACAAACCCAAATTCTGAGTTACTCGATGAGAATA
CTGGCCGGCACAAATTCAAAAGTCTCGAGGCTAGCGTGTTTGCTATGTACCTGGCTGCTGTGACGAGCATGACAAATGAAGAGTGCCTTGAGCT
TTTAAATGAAGAAAGAAATATTTTAGTAACACGGTACAAAACTGCGACTGAGATTGCTCTTCAAGAATCAGATTTTCTTAATAGTGTTGAAATT
GTTACTTTACAAGCTTTAATAACGTATATCGCAGCTATGCGCACTCATGATCGAAGTCGATCTATATGGGCATTTATCGGTCTTGCAGTCCGCC
TTGCCCGTGGCATCGGGCTCCACAGAGATGGCAGCCAGCAACCTTTCGATTTGGAAATGCGAAGGAGAGTGTGGTGGACATTGATTGTACTTGA
TACAAGAGCTTCCGAAGATCGGGGTACAGAAACCATGATCACTGATGGCTCTTTCGATACAAAGATGCCTGCCAACATAAACGACGAGGATATG
ATGATAAACTCAAAAAGTCTACCGGTCGATCGGATAGGGATCACCAGTATGACCTTTGCTTGCATTACAATGACAGTCAGCGGGATCGGATTGA
GGATGAATTTTGTACCTACGCGCCTAGACGCGCCGGTCTTGACCACAGAACAAAAAGAGCAGATGATCAAAGGATTTACTGACAAAATCGATTC
TACCTATCTCGCAGGTTCTGATCCCAATGATCCAAGATTGTGGTGGTATTGTCGGATATCACGACTATTATCGCTCAAGTTATGGCTGGTGACA
CAATATCCACTTCAACGAAGGAAATCTACAAATCGAGTGCTTCCTCGAGGACAATCCTTGCGTACAGCTATGGCATTCCTAAATTTGGCGGAAG
AAATATGGCAATATGAACCTTCAAAGGGATGGTTCTGGTTCTTTCAAAGTTATGTGCCGTGGCATGTAATTGCGGTTGCTTTAGCTGAGCTTTG
TGCGGAACCAACTGGCCCTCTTGCCGATCAAGCTTGGGAGGGCATTGAGTTTCACTACAATAAATGGAGTGAATTCGTTGCAGATACAAAAGAT
GGGATGATTTGGCGACCAGTCAGGAATTTAATGAAAAGAGCAAGAGCAGCAAGAAGGCGAGAAAGAGGTCTCAGTGAATCTCAAGCATCAACAA
CTCCAACGGCAATTCGCAACCCTAATACATACAGCCCAGCTCTCAATCTAGCTGCTGGATGCTCCCCGATCATGTCGGAGAACAATAACTACTG
TCAATCTTCTACAATTGGACAGGTTGACCCAATGGGGCAGAATATTTACGGGGGAATGGGCTTCGATCTTCCCATGCCCATGGGTTCTACCCCC
TCAGACACGAATATGGCATCCCCACTACCTATTCCGGACGGCTATACAAATCCGCACTGGAACGAATTCATATTTGGTTTAGGAACAGTTTGTA
GCGATGGACCCCAAAATCCAGA
>Bcin05g01790(mrr1), partial sequence [organism=Botrytis cinerea, strain D11_T_E18]
CAGGCATCTGGCACGGAATGCATTTTCCCTACACGTGTTCGGAACCCAAGACCGAGACAAAGCAATCCAAAGTCTCGTGATTCTGAGCTATTAA
AAAGAATCTCTCACCTCGAAAGTTTGGTGAGCAGAATTGATGCCTCAAAACTCCTTGGGGAGGATGGTCAGGTAGAAGACGAGGTTCGGTCAAA
TCCACCATCGTCAGGACCGCGACCTCATCGTTCTTTCACGTCTCCAGACCACCAATACAATGACAATCATCATGAGTTCGGAATAGTACCAGGT
CGTATGGCTCATCAATTTGCTGATTTTATCAAACGCCAAGAGACCGACTCGATTTACACCCATGATGGATTTTGGACAAGACTAAGTGAAGAAA
TTGATGGATTGAAACAGCTCATCGAGAGCCCCAGTGACGATGAAGATGAAAATTCTGGGTCCACTGCAACTTCGCCAGCATCCAAGTATGACTC
CCCTTCACAGTTTGTCTTTGATTCCAGGGTCACTTCCTCCAATGTCACAATTCCATATCCATCCCACGACCATACGAAAGTTTTATTCGATATT
TATTTCAAAAATGTACATCCGATGGTCGTCACTATGCACAAGCCAACAACGTACCTTTTCACAAACCCAAATTCTGAGTTACTCGATGAGAATA
CTGGCCGGCACAAATTCAAAAGTCTCGAGGCTAGCGCATTTTCTATGTACCTGGCTGCTGTGACGAGCATGACAAATGAAGAGTGCCTTGAGCT
TTTAAATGAAGAAAGACAGATTTTAGTAACAAGGTACAAAACTGCGACTGAGATTGCTCTTCAAGAATCAGATTTTCTTAATAGTGTAGAAATT
GTTACTTTACAAGCTTTGATAACGTATATCGCAGCTATGCGCGCTCATGATCGAAGTCGATCTACATGGACATTTATCGGTCTTGCAGTCCGCC
TTGCCCGTGGCATCGGGCTCCACAGGGATGGCACCGGGCTCCACAGAGATGGCAGCAAGCAACCTTTCGATTTGGAAATGCGAAGGAGACTATG
GTGGACATTGATTGTACTTGATACAAGAGCTTCCGAAGATCGGGGTACGGAAACTATGATCACTGATGGCTCTTTCGATACAAAGATGCCTGCC
AATATAAATGATGAGGATATATCGATAAACTCAAAAACTCTACCGGTCGATCGGCTAGGATTAACCAGTATGACCTTTGCTTGCATTACAATGA
CAGTCAGCGGGATCGGATTGAGGATGAATTTTGTACCTACGCGCCTAGACGCGCCGGTCTTGACCACAGAACAAAAAGAGCAGATGATCAAAGG
ATTTACTGACAAAATCGATTCTACCTATGTCACATGTTCTGATCCCAATGATCCAAGATTGTGGTGGTTTTGTCGGGTATCACGACTATTATCG
CTCAAGTTATGGTTGGCGACACAATATCCACTTCAGCGAAGGAAATCTACAAATCGAGTGCTTCCTCGAGGACAATCCTTGCGTACAGCTATGG
CATTCTTAAATTTGGCGGAAGAAATATGGCAATATGAACCTTCAAAGGGATGGTTCTGGTTCTTTCAAAGTTATGTGCCGTGGCATGCAATTGC
GGTTGCTTTAGCTGAGCTTTGTGCGGAACCAACTGGCCCTCTTGCCGATCAAGCTTGGGGGAGCATCGAGTTTCATTACAATAAATGGAGTGAA
TTCGTTGCAGATACAAAAGATGGGATGATTTGGCGGCCAGTCAAGAATTTAATGAAAAGAGCAAAAGCAGCAAGAAGGCGAGAAAGAGGTCTCA
GTGAATCTCAAGCATCAACAACTCCAACGGCAATTCTCAACCCTAATACATACAGCCCAGCTCTCGATCTAGGTGCTGAATTCTCACCAATCAT
GTCGGAGAATAATAACTACCGTCAACCTTCTACAATTGGGCAGGTTGACCCAATGGGGCAGAATATTTACGGGGGGATGAGCTTCGATCTTCCC
ATGCCCATGTGTTCTACCCCCTCAGACACGAATATGGCATCCCCACTACCTATTCCGGACGGTTATACAAATCCGCACTGGAACGAATTCATAT
TTGGTTTAGGAACAGTTGGTAGCGATGGACCCCAGAATCCAGA
>Bcin05g01790(mrr1), partial sequence [organism=Botrytis pseudocinerea, strain BpsVD110]
CAGGCATCTGGCACGGAATGTATTTTCCCTACACGTGTTCGGAATCCAAGACCGAGACAAAGCAATCCAAAGTCTCGTGATTCTGAGCTATTGA
```

AAAGAATCTCTCATCTCGAAAGTTTGGTGAGCAGAATTGATGCCTCAAAACTCCTTGGGGAGGATGGTCAGGTAGAAGACGAGGTTCGGTCAAA
CCCACCATCATCAGGACCGCGACCTCATCGTTCTTTCACGTCTCCAGACCGCCAATACAATGACAACCACCATGAGTTTGGAATAGTACCAGGT
CGTATGGCTCATCAATTTGCTGATTTTATTAAACGCCAAGAGACCGACTCGATTTACACTCATGATGGATTTTGGACAAGATTAAGTGAAGAAA
TTGATGGATTGAAACAGCTCATCGAGAGCCCCAGTGACGATGAAGATGAAATTTCTGGGTCAACTGCAACCTCGCCAGCATCCAAGTATGACTC
CCCTTCACAGTTTGTCTTTGATTCCAGGGTCACTTCCTCGAATGTCATAATTCCATATCCATCCCACGACCATACCAAAATTTTATTCGATATT
TATTTCAAAAATGTACATCCGATGGTCGTCACTATGCACAAGCCAACAACGTACCTTTTCTCAAAACCAAATTCTGAGTTAATGGATGAGAATA
CTGGCCGGCACAAATTCAAAAGTCTCGAGGCTAGCGCATTTTCTATGTACCTGGCTGCTGTGACGAGCATGACAAATGAAGAGTGCCTTGAGCT
TTTAAATGAAGAAAGACAGATTTTAGTAACAAGGTACAAAACTGCGACTGAGATTGCTCTTCAAGAATCAGATTTTCTTAATAGTGTAGAAATT
GTTACTCTACAAGCTTTAATCACGTATATCGTAAGTGTTTCTCTCTACACATCTTAGATGATAGTTTTACGATGATGATGATGTTTATGTACTT
TCACAGGCAGCTATGCGCACTCATGATCGAAGTCGATCTACATGGGCATTTATCGGTCTTGCAGTCCGCCTTGCCCGTGGCATCGGGCTCCACA
GGGATGGCAGCACGCAATCTTTCGATTTGGAAATGCGAAGGAGAATATGGTGGACATTGATTGTACTTGATACAAGAGCCTCCGAAGATCGGGG
TACAGAAACTATGATCACTGATGGCTCTTTCGATACAAAGATGCCTGCCAATATAAATGACGAGGATATCTTGATAGACTCAAAAACTCTACCA
GTCGATCGGATAGGATTCACCAGTATGACCTTTGCTTGCATTACAATGACAGTCAGCGGGATCGGATTGAGGATGAATTTTGTACCTACGCGCC
TAGACGCGCCGGTCTTGACCACAGAACAAAAAGAGCAGATGATCAAAGGATTTACTGACAAAATCGATTCTACCTATGTCACAGGTTCTGATCC
CAATGATCCAAAATTGTGGTGGTTTTGTCGGATATCACGACTATTATCGCTCAAGTTATGGTTGGCGACACAATACCCACTTCAGCGAAGGAAA
TCTACAAATCGAGTGCTTCCTCGAGGACAATCCTTGCGTACAGCTATGGCATACTTAAATTTGGCGGAAGAAATATGGCAATATGAACCTTCAA
AGGGATGGTTCTGGTTCTTTCAAAGTTATGTGCCGTGGCATGCAATTGCGGTTGCTTTAGCTGAGCTTTGTGCGGAACCAACTGGCCCTCTTGC
CGATCACGCTTGGGGGAGCATTGAGTCTCATTACAATAAATGGAGTGAATTCGTTGCAGATACAAAAGATGGGATGATTTGGCGGCCAGTCAAG
AATTTAATGAAAAGAGCAAGAGCAGCAAGAAGGCGAGAAAGAGGTCTCAGTGAATCTCAAGCATCAACAACTCCAACGGCAATTCTCAACCCTA
ATATATACAGCCCAGCTCTCGATCTAGGTGCTGAATTCTCACCAATCATGTCGGAGAATAATAACTACCGTCAACCTTCTACAATTGGGCAGGT
TGACCCAATGGGGCAGAATATTTACGGGGGGATGAGCTTCGATCTTCCCATGCCCATGTGTTCTACCCCCTCAGACACGAATATGACATCCCCA
CTACCTATTCCGGACGGCTATACAAATCCGCACTGGAATGAATTCATATTTGGCTTAGGAACAGTTGGTAGCGATGGACCCCAAAACCCAGA
>Bcin05g01790(mrr1), partial sequence [organism=Botrytis pseudocinerea, strain BpsD11_KL_cal2]
CAGGCATCTGGCACGGAATGTATTTTCCCTACACGTGTTCGGAACCCAAGACCGAGACAAAGCAATCCAAAGTCTCGTGATTCTGAGCTATTGA
AAAGAATCTCTCACCTCGAAAGTTTGGTGAGCAGAATTGATGCCTCAAAACTCCTTGGGGAGGAGGGTCAGGTAGAAGACGAGGTTCGGTCAAA
CCCACCATCATCAGGATCGCGACCTCATCGTTCTTTCACGTCTCCAGACCGCCAATACAATGACAATCACCATGAGTTTGGAATAGTACCAGGT
CGTATGGCTCATCAATTTGCTTATTTTATTAAACGCCAAGAGGCCGACTCGATTTACACTCATGATGGATTTTGGACAAGATTAAGTGAAGAAA
TTGATGGATTGAAACAGCTCATCGAGAGCCCCAGTGACGATGAAGATGAAAATTCTGGGTCAACTGCAACTTCGCCAGCATCCAAGTATGACTC
CCCTTCACAGTTTGTCTTTGATTCCAGGGTCACTTCCTCGAATGTCATAATTCCATATCCATCCCACGACCATACCAAAGTTTTATTCGATATT
TATTTCAAAAATGTACATCCGATGGTCGTCACTATGCACAAGCCAACAACGTACCTTTTCTCAAACCCAAATTCTGAGTTAATGGATGAGAATA
CTGGCCGGCACAAATTCAAAAGTCTCGAGGCTAGCGCATTTTCTATGTACCTGGCTGCTGTGACGAGCATGACAAATGAAGAGTGCCTTGAGCT
TTTAAATGAAGAAAGACAGATTTTAGTAACAAGGTACAAAACTGCGACTGAGATTGCTCTTCAAGAATCAGATTTTCTTAATAGTGTAGAAATT
GTTACTCTACAAGCTTTAATCACGTATATCGTAAGTGTTTCTCTCTACACATCTTAGATGATAGTTTTACGATGATGATGATGTTTATGTACTT
TCACAGGCAGCTATGCGCACTCATGATCGAAGTCGATCTACATGGGCATTTATCGGTCTTGCAGTCCGCCTTGCCCGTGGCATCGGGCTCCACA
GGGATGGCAGCGGGCTCCACAGGGATGGCAGCAGGCTCAACAGAGATGGCAGCACGCAATCTTTCGATTTGGAAATGCGAAGGAGAATATGGTG
GTCATTGATTGTACTTGATACAAGAGCCTCCGAAGATCGGGGTACAGAAACTATGATCACTGATGGCTCTTTCGATACAAAGATGCCTGCCAAT
ATAAATGACGAGGATATCTTGATAGACTCAAAAACTCTACCAGTCGATCGGATAGGATTCACCAGTATGACCTTTGCTTGCATTACAATGACAG
TCAGCGGGATCGGATTGAGGATGAATTTTGTACCTACGCGCCTAGACGCGCCGGTCTTGACCACAGAACAAAAAGAGCAGATGATCAAAGGATT
TACTGACAAAATCGATTCTACCTATGTCACAGGTTCTGATCCCAATGATCCAAAATTGTGGTGGTTTTGTCGGATATCACGACTATTATCGCTC
AAGTTATGGTTGGCGACACAATACCCACTTCAGCGAAGGAAATCTACAAATCGAGTGCTTCCTCGAGGACAATCCTTGCGTACAGCTATGGCAT
TCTTAAATTTGGCGGAAGAAATATGGCAATATGAACCTTCAAAGGGATGGTTCTGGTTCTTTCAAAGTTATGTGCCGTGGCATGCAATTGCGGT
TGCTTTAGCTGAGCTTTGTGCGGAACCAACTGGCCCTCTTGCCGATCACGCTTGGGGGAGCATTGAGTTTCATTACAATAAATGGAGTGAATTC
GTTGCAGATACGAAAGATGGGATGATTTGGCGGCCAGTCAAGAATTTAATGAAAAGAGCAAGAGCAGCAAGAAGGCGAGAAAGAGGTCTCAGTG
AATCTCAAGCATCAACAACTCCAACGGCAATTCTCAACCCTAATATATACAGCCCAGCGCTCGATCTAGGTGCTGAATTCTCACCGATCATGTC
GGAGAATAATAACTACCGTCAACCTTCTACAATTGGGCAGGTTGACCCAATGGGGCAGAATATTTACGGGGGGATGAGCTTCGATCTTCCCATG
CCCATGTGTTCTACCCCCTCAGACACGAATATGGCATCCCCAATACCTATTCCGGACGGCTATACAAACCCGCACTGGAATGAATTCATATTTG
GTTTAGGAACAGTTGGTAGCGATGGACCCCAAAACCCAGA
>Bcin05g01790(mrr1), partial sequence [organism=Botrytis pseudocinerea, strain BpsD12_E_cal10]
CAGGCATCTGGCACGGAATGTATTTTCCCTACACGTGTTCGGAATCCAAGACCGAGACAAAGCAATCCAAAGTCTCGTGATTCTGAGCTATTGA
AAAGAATCTCTCATCTCGAAAGTTTGGTGAGCAGAATTGATGCCTCAAAACTCCTTGGGGAGGATGGTCAGGTAGAAGACGAGGTTCGGTCAAA
CCCACCATCATCAGGACCGCGACCTCATCGTTCTTTCACGTCTCCAGACCGCCAATACAATGACAACCACCATGAGTTTGGAATAGTACCAGGT
CGTATGGCTCATCAATTTGCTGATTTTATTAAACGCCAAGAGACCGACTCGATTTACACTCATGATGGATTTTGGACAAGATTAAGTGAAGAAA
TTGATGGATTGAAACAGCTCATCGAGAGCCCCAGTGACGATGAAGATGAAATTTCTGGGTCAACTGCAACCTCGCCAGCATCCAAGTATGACTC
CCCTTCACAGTTTGTCTTTGATTCCAGGGTCACTTCCTCGAATGTCATAATTCCATATCCATCCCACGACCATACCAAAATTTTATTCGATATT
TATTTCAAAAATGTACATCCGATGGTCGTCACTATGCACAAGCCAACAACGTACCTTTTCTCAAAACCAAATTCTGAGTTAATGGATGAGAATA
CTGGCCGGCACAAATTCAAAAGTCTCGAGGCTAGCGCATTTTCTATGTACCTGGCTGCTGTGACGAGCATGACAAATGAAGAGTGCCTTGAGCT
TTTAAATGAAGAAAGACAGATTTTAGTAACAAGGTACAAAACTGCGACTGAGATTGCTCTTCAAGAATCAGATTTTCTTAATAGTGTAGAAATT

GTTACTCTACAAGCTTTAATCACGTATATCGTAAGTGTTTCTCTCTACACATCTTAGATGATAGTTTTACGATGATGATGATGTTTATGTACTT
TCACAGGCAGCTATGCGCACTCATGATCGAAGTCGATCTACATGGGCATTTATCGGTCTTGCAGTCCGCCTTGCCCGTGGCATCGGGCTCCACA
GGGATGGCAGCACGCAATCTTTCGATTTGGAAATGCGAAGGAGAATATGGTGGACATTGATTGTACTTGATACAAGAGCCTCCGAAGATCGGGG
TACAGAAACTATGATCACTGATGGCTCTTTCGATACAAAGATGCCTGCCAATATAAATGACGAGGATATCTTGATAGACTCAAAAACTCTACCA
GTCGATCGGATAGGATTCACCAGTATGACCTTTGCTTGCATTACAATGACAGTCAGCGGGATCGGATTGAGGATGAATTTTGTACCTACGCGCC
TAGACGCGCCGGTCTTGACCACAGAACAAAAAGAGCAGATGATCAAAGGATTTACTGACAAAATCGATTCTACCTATGTCACAGGTTCTGATCC
CAATGATCCAAAATTGTGGTGGTTTTGTCGGATATCACGACTATTATCGCTCAAGTTATGGTTGGCGACACAATACCCACTTCAGCGAAGGAAA
TCTACAAATCGAGTGCTTCCTCGAGGACAATCCTTGCGTACAGCTATGGCATACTTAAATTTGGCGGAAGAAATATGGCAATATGAACCTTCAA
AGGGATGGTTCTGGTTCTTTCAAAGTTATGTGCCGTGGCATGCAATTGCGGTTGCTTTAGCTGAGCTTTGTGCGGAACCAACTGGCCCTCTTGC
CGATCACGCTTGGGGGAGCATTGAGTCTCATTACAATAAATGGAGTGAATTCGTTGCAGATACAAAAGATGGGATGATTTGGCGGCCAGTCAAG
AATTTAATGAAAAGAGCAAGAGCAGCAAGAAGGCGAGAAAGAGGTCTCAGTGAATCTCAAGCATCAACAACTCCAACGGCAATTCTCAACCCTA
ATATATACAGCCCAGCTCTCGATCTAGGTGCTGAATTCTCACCAATCATGTCGGAGAATAATAACTACCGTCAACCTTCTACAATTGGGCAGGT
TGACCCAATGGGGCAGAATATTTACGGGGGGATGAGCTTCGATCTTCCCATGCCCATGTGTTCTACCCCCTCAGACACGAATATGACATCCCCA
CTACCTATTCCGGACGGCTATACAAATCCGCACTGGAATGAATTCATATTTGGCTTAGGAACAGTTGGTAGCGATGGACCCCAAAACCCAGA
>Bcin05g01790(mrr1), partial sequence [organism=Botrytis cinerea, strain D13_E_IF04]
CAGGCATCTGGCACGGAATGCATTTTCCCTACACGTGTTCGGAACCCAAGACCGAGACAAAGCAATCCAAAGTCTCGTGATTCTGAGCTATTAA
AAAGAATCTCTCACCTCGAAAGTTTGGTGAGCAGAATTGATGCCTCAAAACTCCTTGGGGAGGATGGTCAGGTAGAAGACGAGGTTCGGTCAAA
TCCACCATCATCAGGACCGCGACCTCATCGTTCTTTCACGTCTCCAGACCACCAATACAATGATAATCATCATGAGTTCGGAATAGTACCAGGT
CGTATGGCTCATCAATTTGCTGATTTTATTAAACGCCAAGAGACTGACTCGATTTATACCCATGATGGATTTTGGACAAGACTAAGTGAAGAAA
TTGATGGATTGAAACAGCTCATCGAGAGCCCCAGTGACGATGAAGATGAAAATTTTGGGTCCACTGCAACTTCGCCAGCATCCAAGTATGACTC
CCCTTCACAGTTTGTCTTTGATTCCAGGGTCACTTCCTCGAATGTCACAATTCCATATCCATCCCACGACCATACGAAAGTTTTATTCGATATT
TATTTCAAAAATGTACATCCGATGGTCGTCACTATGCACAAGTCAACAACGTACCTTTTCACAAACCCAAATTCTGAGTTACTCGATGAGAATA
CTGGCCGGCACAAATTCAAAAGTCTCGAGGCTAGCGCATTTTCTATGTACCTGGCTGCTGTGACGAGCATGACAAATGAAGAGTGCCTTGAGCT
TTTAAATGAAGAAAGACAGATTTTAGTAACAAGGTACAAAACTGCGACTGAGATTGCTCTTCAAGAATCGGATTTTCTTAATAGTGTAGAAATT
GTTACTTTACAAGCTTTAATAACGTATATCGTAAGTATTTCTCTCTCAGCATCTTGGATGATGGTTTTATGATGATGATGATGTTTATGTACTT
TCACAGGCAGCTATGCGCGCTCATGATCGAAGTCGATCTACATGGACATTTATCGGTCTTGCAGTCCGCCTTGCCCGTGGCATCGGGCTCCACA
GGGATGGCACCGGGCTCCACAGAGATGGCAGCAAGGAACCTTTCGACTTGGAAATGCGAAGGAGAATATGGTGGACATTGATTGTACTTGATAC
AAGAGCTTCCGAAGATCGGGGTACGGAAACTATGATCACGGATGGCTCTTTCGATACAAAGATGCCTGCCAATATAAATGACGAGGATATATCG
ATAAACTCAAAAACTCTACCGGTCGATCGGTTAGGATTCACCAGTATGACCTTTGCTTGCATTACAATGACAGTCAGCGGGATCGGATTGAGGA
TGAATTTTGTACCTACGCGCCTAGACGCGCCGGTCTTGACCACAGAACAAAAAGAGCAGATGATCAAAGGATTTACTGACAAAGTCGATTCTAC
CTATGTCACATGTTCTGATCCCAATGATCCAAGATTGTGGTGGTTTTCTCGGGTATCACGACTATTATCGCTCAAGTTATGGTTGGCGACACAA
TATCCACTTCAGCGAAGGAAATCTACAAATCGAGTGCTTCCTCGGGGACAATCCTTGCGTACAGCTATGGCATTCTTAAATTTGGCGGAAGAAA
TATGGCAATATGAACCTTCAAAGGGATGGTACTGGTTCTTTCAAAGTTATGTGCCGTGGCATGCAATTGCGGTTGCTTTAGCTGAGCTTTGCGC
GGAACCAACTGGCCCTCTTGCCGATCAAGCTTGGGGGAGCATCGAGTTTCATTACAATAAATGGAGTGAATTCGTTGCAGATACAAAAGATGGG
ATGATTTGGCGGCCAGTCAAGAATTTAATGAAAAGAGCAAGAGCAGCAAGAAGGCGAGAAAGAGGTCTCAGTGAATCTCAAGCATCAACAACTC
CAACGGCAATTCTCAACCCTAATACATACAGCCCAGCTCTCGATCTAGGTGCTGAATTCTCACCAATCATGTCGGAGAATAATAACTATCGTCA
ACCTTCTACAATTGGGCAGGTTGACCCAATGGGGCAGAATATTTACGGGGGGATGAACTTCGATCTTCCCATGCCCATGTGTTCTACCCCCTCA
GACACGAATATGGCATCCCCACTACCTATTCCGGACGGCTATACAAATCCGCACTGGAACGAATTCATATTTGGTTTAGGAACAGTTGGTAGCG
ATGGACCCCAAAATCCAGA
>Bcin05g01790(mrr1), partial sequence [organism=Botrytis cinerea, strain D13_E_IL4]
CAGGCATCTGGCACGGAATGCATTTTCCCTACACGTGTTCGGAACCCAAGACCGAGACAAAGCAATCCAAAGTCTCGTGATTCTGAGCTATTAA
AAAGAATCTCTCACCTCGAAAGTTTGGTGAGCAGAATTGATGCCTCAAAACTCCTTGGGGAGGATGGTCAGGTAGAAGACGAGGTTCGGTCAAA
TCCACCATCATCAGGACCGCGACCTCATCGTTCTTTCACGTCTCCAGACCACCAATACAATGATAATCATCATGAGTTCGGAATAGTACCAGGT
CGTATGGCTCATCAATTTGCTGATTTTATTAAACGCCAAGAGACTGACTCGATTTATACCCATGATGGATTTTGGACAAGACTAAGTGAAGAAA
TTGATGGATTGAAACAGCTCATCGAGAGCCCCAGTGACGATGAAGATGAAAATTTTGGGTCCACTGCAACTTCGCCAGCATCCAAGTATGACTC
CCCTTCACAGTTTGTCTTTGATTCCAGGGTCACTTCCTCGAATGTCACAATTCCATATCCATCCCACGACCATACGAAAGTTTTATTCGATATT
TATTTCAAAAATGTACATCCGATGGTCGTCACTATGCACAAGTCAACAACGTACCTTTTCACAAACCCAAATTCTGAGTTACTCGATGAGAATA
CTGGCCGGCACAAATTCAAAAGTCTCGAGGCTAGCGCATTTTCTATGTACCTGGCTGCTGTGACGAGCATGACAAATGAAGAGTGCCTTGAGCT
TTTAAATGAAGAAAGACAGATTTTAGTAACAAGGTACAAAACTGCGACTGAGATTGCTCCTCAAGAATCGGATTTTCTTAATAGTGTAGAAATT
GTTACTTTACAAGCTTTAATAACGTATATCGTAAGTATTTCTCTCTCAGCATCTTGGATGATGGTTTTATGATGATGATGATGTTTATGTACTT
TCACAGGCAGCTATGCGCGCTCATGATCGAAGTCGATCTACATGGACATTTATCGGTCTTGCAGTCCGCCTTGCCCGTGGCATCGGGCTCCACA
GGGATGGCACCGGGCTCCACAGAGATGGCAGCAAGGAACCTTTCGACTTGGAAATGCGAAGGAGAATATGGTGGACATTGATTGTACTTGATAC
AAGAGCTTCCGAAGATCGGGGTACGGAAACTATGATCACGGATGGCTCTTTCGATACAAAGATGCCTGCCAATATAAATGACGAGGATATATCG
ATAAACTCAAAAACTCTACCGGTCGATCGGTTAGGATTCACCAGTATGACCTTTGCTTGCATTACAATGACAGTCAGCGGGATCGGATTGAGGA
TGAATTTTGTACCTACGCGCCTAGACGCGCCGGTCTTGACCACAGAACAAAAAGAGCAGATGATCAAAGGATTTACTGACAAAGTCGATTCTAC
CTATGTCACATGTTCTGATCCCAATGATCCAAGATTGTGGTGGTTTTCTCGGGTATCACGACTATTATCGCTCAAGTTATGGTTGGCGACACAA
TATCCACTTCAGCGAAGGAAATCTACAAATCGAGTGCTTCCTCGGGGACAATCCTTGCGTACAGCTATGGCATTCTTAAATTTGGCGGAAGAAA

```
TATGGCAATATGAACCTTCAAAGGGATGGTACTGGTTCTTTCAAAGTTATGTGCCGTGGCATGCAATTGCGGTTGCTTTAGCTGAGCTTTGCGC
GGAACCAACTGGCCCTCTTGCCGATCAAGCTTGGGGGAGCATCGAGTTTCATTACAATAAATGGAGTGAATTCGTTGCAGATACAAAAGATGGG
ATGATTTGGCGGCCAGTCAAGAATTTAATGAAAAGAGCAAGAGCAGCAAGAAGGCGAGAAAGAGGTCTCAGTGAATCTCAAGCATCAACAACTC
CAACGGCAATTCTCAACCCTAATACATACAGCCCAGCTCTCGATCTAGGTGCTGAATTCTCACCAATCATGTCGGAGAATAATAACTATCGTCA
ACCTTCTACAATTGGGCAGGTTGACCCAATGGGGCAGAATATTTACGGGGGGATGAACTTCGATCTTCCCATGCCCATGTGTTCTACCCCCTCA
GACACGAATATGGCATCCCCACTACCTATTCCGGACGGCTATACAAATCCGCACTGGAACGAATTCATATTTGGTTTAGGAACAGTTGGTAGCG
ATGGACCCCAAAATCCAGA
>Bcin05g01790(mrr1), partial sequence [organism=Botrytis cinerea, strain D13_E_IL7]
CAGGCATCTGGCACGGAATGCATTTTCCCTACACGTGTTCGGAACCCAAGACCGAGACAAAGCAATCCAAAGTCTCGTGATTCTGAGCTATTAA
AAAGAATCTCTCACCTCGAAAGTTTGGTGAGCAGAATTGATGCCTCAAAACTCCTTGGGGAGGATGGTCAGGTAGAAGACGAGGTTCGGTCAAA
TCCACCATCATCAGGACCGCGACCTCATCGTTCTTTCACGTCTCCAGACCACCAATACAATGATAATCATCATGAGTTCGGAATAGTACCAGGT
CGTATGGCTCATCAATTTGCTGATTTTATTAAACGCCAAGAGACTGACTCGATTTATACCCATGATGGATTTTGGACAAGACTAAGTGAAGAAA
TTGATGGATTGAAACAGCTCATCGAGAGCCCCAGTGACGATGAAGATGAAAATTCTGGGTCCACTGCAACTTCGCCAGCATCCAAGTATGACTC
CCCTTCACAGTTTGTCTTTGATTCCAGGGTCACTTCCTCGAATGTCACAATTCCATATCCATCCCACGACCATACGAAAGTTTTATTCGATATT
TATTTCAAAAATGTACATCCGATGGTCGTCACTATGCACAAGTCAACAACGTACCTTTTCACAAACCCAAATTCTGAGTTACTCGATGAGAATA
CTGGCCGGCACAAATTCAAAAGTCTCGAGGCTAGCGCATTTTCTATGTACCTGGCTGCTGTGACGAGCATGACAAATGAAGAGTGCCTTGAGCT
TTTAAATGAAGAAAGACAGATTTTAGTAACAAGGTACAAAACTGCGACTGAGATTGCTCTTCAAGAATCGGATTTTCTTAATAGTGTAGAAATT
GTTACTTTACAAGCTTTAATAACGTATATCGTAAGTATTTCTCTCTCAGCATCTTGGATGATGGTTTTATGATGATGATGATGTTTATGTACTT
TCACAGGCAGCTATGCGCGCTCATGATCGAAGTCGATCTACATGGACATTTATCGGTCTTGCAGTCCGCCTTGCCCGTGGCATCGGGCTCCACA
GGGATGGCACCGGGCTCCACAGAGATGGCAGCAAGGAACCTTTCGACTTGGAAATGCGAAGGAGAATATGGTGGACATTGATTGTACTTGATAC
AAGAGCTTCCGAAGATCGGGGTACGGAAACTATGATCACGGATGGCTCTTTCGATACAAAGATGCCTGCCAATATAAATGACGAGGATATATCG
ATAAACTCAAAAACTCTACCGGTCGATCGGTTAGGATTCACCAGTATGACCTTTGCTTGCATTACAATGACAGTCAGCGGGATCGGATTGAGGA
TGAATTTTGTACCTACGCGCCTAGACGCGCCGATCTTGACCACAGAACAAAAAGAGCAGATGATCAAAGGATTTACTGACAAAGTCGATTCTAC
CTATGTCACATGTTCTGATCCCAATGATCCAAGATTGTGGTGGTTTTCTCGGGTATCACGACTATTATCGCTCAAGTTATGGTTGGCGACACAA
TATCCACTTCAGCGAAGGAAATCTACAAATCGAGTGCTTCCTCGGGGACAATCCTTGCGTACAGCTATGGCATTCTTAAATTTGGCGGAAGAAA
TATGGCAATATGAACCTTCAAAGGGATGGTTCTGGTTCTTTCAAAGTTATGTGCCGTGGCATGCAATTGCGGTTGCTTTAGCTGAGCTTTGCGC
GGAACCAACTGGCCCTCTTGCCGATCAAGCTTGGGGGAGCATCGAGTTTCATTACAATAAATGGAGTGAATTCGTTGCAGATACAAAAGATGGG
ATGATTTGGCGGCCAGTCAAGAATTTAATGAAAAGAGCAAGAGCAGCAAGAAGGCGAGAAAGAGGTCTCAGTGAATCTCAAGCATCAACAACTC
CAACGGCAATTCTCAACCCTAATACATACAGCCCAGCTCTCGATCTAGGTGCTGAATTCTCACCAATCATGTCGGAGAATAATAACTATCGTCA
ACCTTCTACAATTGGGCAGGTTGACCCAATGGGGCAGAATATTTACGGGGGGATGAACTTCGATCTTCCCATGCCCATGTGTTCTACCCCCTCA
GACACGAATATGGCATCCCCACTACCTATTCCGGACGGCTATACAAATCCGCACTGGAACGAATTCATATTTGGTTTAGGAACAGTTGGTAGCG
ATGGACCCCAAAATCCAGA
>Bcin05g01790(mrr1), partial sequence [organism=Botrytis cinerea, strain D13_E_IL12]
CAGGCATCTGGCACGGAATGCATTTTCCCTACACGTGTTCGGAACCCAAGACCGAGACAAAGCAATCCAAAGTCTCGTGATTCTGAGCTATTAA
AAAGAATCTCTCACCTCGAAAGTTTGGTGAGCAGAATTGATGCCTCAAAACTCCTTGGGGAGGATGGTCAGGTAGAAGACGAGGTTCGGTCAAA
TCCACCATCATCAGGACCGCGACCTCATCGTTCTTTCACGTCTCCAGACCACCAATACAATGATAATCATCATGAGTTCGGAATAGTACCAGGT
CGTATGGCTCATCAATTTGCTGATTTTATTAAACGCCAAGAGACTGACTCGATTTATACCCATGATGGATTTTGGACAAGACTAAGTGAAGAAA
TTGATGGATTGAAACAGCTCATCGAGAGCCCCAGTGACGATGAAGATGAAAATTCTGGGTCCACTGCAACTTCGCCAGCATCCAAGTATGACTC
CCCTTCACAGTTTGTCTTTGATTCCAGGGTCACTTCCTCGAATGTCACAATTCCATATCCATCCCACGACCATACGAAAGTTTTATTCGATATT
TATTTCAAAAATGTACATCCGATGGTCGTCACTATGCACAAGTCAACAACGTACCTTTTCACAAACCCAAATTCTGAGTTACTCGATGAGAATA
CTGGCCGGCACAAATTCAAAAGTCTCGAGGCTAGCGCATTTTCTATGTACCTGGCTGCTGTGACGAGCATGACAAATGAAGAGTGCCTTGAGCT
TTTAAATGAAGAAAGACAGATTTTAGTAACAAGGTACAAAACTGCGACTGAGATTGCTCTTCAAGAATCGGATTTTCTTAATAGTGTAGAAATT
GTTACTTTACAAGCTTTAATAACGTATATCGTAAGTATTTCTCTCTCAGCATCTTGGATGATGGTTTTATGATGATGATGATGTTTATGTACTT
TCACAGGCAGCTATGCGCGCTCATGATCGAAGTCGATCTACATGGACATTTATCGGTCTTGCAGTCCGCCTTGCCCGTGGCATCGGGCTCCACA
GGGATGGCACCGGGCTCCACAGAGATGGCAGCAAGGAACCTTTCGACTTGGAAATGCGAAGGAGAATATGGTGGACATTGATTGTACTTGATAC
AAGAGCTTCCGAAGATCGGGGTACGGAAACTATGATCACGGATGGCTCTTTCGATACAAAGATGCCTGCCAATATAAATGACGAGGATATATCG
ATAAACTCAAAAACTCTACCGGTCGATCGGTTAGGATTCACCAGTATGACCTTTGCTTGCATTACAATGACAGTCAGCGGGATCGGATTGAGGA
TGAATTTTGTACCTACGCGCCTAGACGCGCCGATCTTGACCACAGAACAAAAAGAGCAGATGATCAAAGGATTTACTGACAAAGTCGATTCTAC
CTATGTCACATGTTCTGATCCCAATGATCCAAGATTGTGGTGGTTTTCTCGGGTATCACGACTATTATCGCTCAAGTTATGGTTGGCGACACAA
TATCCACTTCAGCGAAGGAAATCTACAAATCGAGTGCTTCCTCGGGGACAATCCTTGCGTACAGCTATGGCATTCTTAAATTTGGCGGAAGAAA
TATGGCAATATGAACCTTCAAAGGGATGGTTCTGGTTCTTTCAAAGTTATGTGCCGTGGCATGCAATTGCGGTTGCTTTAGCTGAGCTTTGCGC
GGAACCAACTGGCCCTCTTGCCGATCAAGCTTGGGGGAGCATCGAGTTTCATTACAATAAATGGAGTGAATTCGTTGCAGATACAAAAGATGGG
ATGATTTGGCGGCCAGTCAAGAATTTAATGAAAAGAGCAAGAGCAGCAAGAAGGCGAGAAAGAGGTCTCAGTGAATCTCAAGCATCAACAACTC
CAACGGCAATTCTCAACCCTAATACATACAGCCCAGCTCTCGATCTAGGTGCTGAATTCTCACCAATCATGTCGGAGAATAATAACTATCGTCA
ACCTTCTACAATTGGGCAGGTTGACCCAATGGGGCAGAATATTTACGGGGGGATGAACTTCGATCTTCCCATGCCCATGTGTTCTACCCCCTCA
GACACGAATATGGCATCCCCACTACCTATTCCGGACGGCTATACAAATCCGCACTGGAACGAATTCATATTTGGTTTAGGAACAGTTGGTAGCG
ATGGACCCCAAAATCCAGA
```

```
>Bcin05g01790(mrr1), partial sequence [organism=Botrytis cinerea, strain G09_S33]
CAGGCATCTGGCACGGAATGCATTTTCCCTACACGTGTTCGGAACCCAAGACCGAGACAAAGCAATCCAAAGTCTCGTGATTCTGAGCTATTAA
AAAGAATCTCTCACCTCGAAAGTTTGGTGAGCAGAATTGATGCCTCAAAACTCCTTGGGGAGGATGGTCAGGTGGAAGACGAGGTTCGGTCAAA
TCCACCATCATCAGGACCGCGACCTCATCGTTCTTTCACGTCTCCAGACCACCAATACAATGACAATCATCATGAGTTCGGAATAGTACCAGGT
CGTATGGCTCATCAATTTGCTGATTTTATTAAACGCCAAGAGACCAACTCGATTTACACCCATGATGGATTTTGGACAAGACTAAGTGAAGAAA
TTGATGGATTGAAACAGCTCATCGAGAGCCCCAGTGACGATGAAGATGAAAATTCTGGGTCCACTGCAACTTCGCCAGCATCCAAGTATGACTC
CCCTTCACAGTTTGTCTTTGATTCCAGGGTCACTTCCTCGAATGTCACAATTCCATATCCATCCCACGACCATATGAAAGTTTTATTCGATATT
TATTTCAAAAATGTACATCCGATGGTCGTCACTATGCACAAGTCAACAACGTACCTTTTCACAAACCCAAATTCTGAGTTACTCGATGAGAATA
CTGGCCGGCACAAATTCAAAAGTCTCGAGGCTAGCTCATTTTCTGTGTACCTGGCTGCTGTGACGAGCATGACAAATGAAGAATGCCTTGAGCT
TTTAAATGAAGAAAGACAGATTTTAGTAACAAGGTACAAAACTGCGACTGAAATTGCTCTTCAAGAATCAGATTTTCTTAATAGTGTAGAAATT
GTTACTTTACAAGCTTTAATAACGTATATCGCAGCTATGCGCGCTCATGATCGAAGTCGATCTATATGGGCATTTATCGGTCTTGCAGTCCGCC
TTGCCCGTGGCATCGGGCTCCACAGGGATGGCACCGGGCTCCACAGAGATGGCAGCAAGGAACCTTTCGATTTGGAAATGCGAAGGAGAATATG
GTGGACATTGATTGTACTTGATACAAGAGCTTCCGAAGATCGGGGTACGGAAACTATGATCACGGATGGCTCTTTCGATACAAAGATGCCTGCC
AATATAAATGACGAGGATATATCGATAAACTCAAAAACTCTACCGGTCGATCGGTTAGGATTCACCAGTATGACCTTTGCTTGCATTACAATGA
CAGTCAGCGGGATCGGATTGAGGATGAATTTTGTACCTACGCGCCTAGACGCGCCGGTCTTGACCACAGAACAAAAAGAGCAGATGATCAAAGG
ATTTACTGACAAAGTCGATTCTACCTATGTCACATGTTCTGATCCCAATGATCCAAGATTGTGGTGGTTTTCTCGGGTATCACGACTATTATCG
CTCAAGTTATGGTTGGCGACACAATATCCACTTCAGCGAAGGAAATCTACAAATCGAGTGCTTCCTCGGGGACAATCCTTGCGTACAGCTATGG
CATTCTTAAATTTGGCGGAAGAAATATGGCAATATGAACCTTCAAAGGGATGGTTCTGGTTCTTTCAAAGTTATGTGCCGTGGCATGCAATTGC
GGTTGCTTTAGCTGAGCTTTGTGCGGAACCAACTGGCCCTCTTGCCGATCAAGCTTGGGGGAGTATCGAGTTTCATTACAATAAATGGAGTGAA
TTCGTTGCAGATACAAAAGATGGGATGATTTGGCGGCCAGTCAAGAATTTAATGAAAAGAGCAAAAGCAGCAAGAAGGCGAGAAAGAGGTCTCA
GTGAATCTCAAGCATCAACAACTCCAACGGCAATTCTCAATCCTAATACATACAGCCCAGCTCTCGATCTAGGTGCTGAATTCTCACCAATCAT
GTCGGAGAATAATAACTACCGTCAACCTTCTACAATTGGGCAGGTTGATCCAATGGGGCAGAATATTTACGGGGGGATGAGCTTCGATCTTCCC
ATGCCCATGTGTTCTACCCCCTCAGACACGAATATGGCATCCCCACTACCTATTCCGGACGGTTACACAAATCCGCACTGGAACGAATTCATAT
TTGGTTTAGGAACAGTTGGTAGCGATGGACCCCAGAATCCAGA
>Bcin05g01790(mrr1), partial sequence [organism=Botrytis cinerea, strain D08_H_8_4]
CAGGCATCTGGCACGGAATGCATTTTCCCTACACGTGTTCGGAACCCAAGACCGAGACAAAGCAATCCAAAGTCTCGTGATTCTGAGCTATTAA
AAAGAATCTCTCACCTCGAAAGTTTGGTGAGCAGAATTGATGCCTCAAAACTCCTTGGGGAGGATGGTCAGGTAGAAGACGAGGTTCGGTCAAA
TCCACCATCATCAGGACCGCGACCTCATCGTTCTTTCACGTCTCCAGACCACCAATACAATGACAATCATCATGAGTTCGGAATAGTACCAGGT
CGTATGGCTCATCAATTTGCTGATTTTATTAAACGCCAAGAGACCGACTCGATTTACACCCATGATGGATTTTGGACAAGACTAAGTGAAGAAA
TTGATGGATTGAAACAGCTCATCGAGAGCCCCAGTGACGATGAAGATGAAAATTCTGGGTCCACTGCAACTTCGCCAGCATCCAAGTATGACTC
CCCTTCACAGTTTGTCTTTGATTCCAGGGTCACTTCCTCGAATGTCACAATTCCATATCCATCCCACGACCATACGAAAGTTTTATTCGATATT
TATTTCAAAAATGTACATCCGATGGTCGTCACTATGCACAAGTCAACAACGTACCTTTTCACAAACCCAAATTCTGAGTTACTCGATGAGAATA
CTGGCCGGCACAAATTCAAAAGTCTCGAGGCTAGCTCATTTTCTATGTACCTGGCTGCTGTGACGAGCATGACAAATGAAGAATGCCTTGAGCT
TTTAAATGAAGAAAGACAGATTTTAGTAACAAGGTACAAAACTGCGACTGAGATTGCTCTTCAAGAATCAGATTTTCTTAATAGTGTAGAAATT
GTTACTTTACAAGCTTTAATAACGTATATCGCAGCTATGCGCGCTCATGATCGAAGTCGATCTATATGGGCATTTATCGGTCTTGCAGTCCGCC
TTGCCCGTGGCATCGGGCTCCACAGGGATGGCACCGGGCTCCACAGAGATGGCAGCAAGGAACCTTTCGATTTGGAAATGCGAAGGAGAATATG
GTGGACATTGATTGTACTTGATACAAGAGCTTCCGAAGATCGGGGTACGGAAACTATGATCACGGATGGCTCTTTCGATACAAAGATGCCTGCC
AATATAAATGACGAGGATATAACGATAAACTCAAAAACTCTACCGGTCGATCGGTTAGGATTCACCAGTATGACCTTTGCTTGCATTACAATGA
CAGTCAGCGGGATCGGATTGAGGATGAATTTTGTACCTACGCGCCTAGACGCGCCGGTCTTGACCACAGAACAAAAAGAGCAGATGATCAAAGG
ATTTACTGACAAAGTTGATTCTACCTATGTCACATGTTCTGATCCCAATGATCCAAGATTGTGGTGGTTTTCTCGGGTATCACGACTATTATCG
CTCAAGTTATGGTTGGCGACACAATATCCACTTCAGCGAAGGAAATCTACAAATCGAGTGCTTCCTCGGGGACAATCCTTGCGTACAGCTATGG
CATTCTTAAATTTGGCGGAAGAAATATGGCAATATGAACCTTCAAAGGGATGGTCCTGGTTCTTCCAAAGTTATGTGCCGTGGCATGCAATTGC
GGTTGCTTTAGCTGAGCTTTGCGCGGAACCAACTGGCCCTCTTGCCGATCAAGCTTGGGGGAGTATCGAGTTTCATTACAATAAATGGAGTGAA
TTCGTTGCAGATACAAAAGATGGGATGATTTGGCGGCCAGTCAAGAATTTAATGAAAAGAGCAAAAGCAGCAAGAAGGCGAGAAAGAGGTCTCA
GTGAATCTCAAGCATCAACAACTCCAACGGCAATTCTCAATCCTAATACATACAGCCCAGCTCTCGGTCTAGGTGCTGAATTCTCACCAATCAT
GTCGGAGAATAATAACTACCGTCAACCTTCTACAATTGGGCAGGTTGATCCAATGGGGCAGAATATTTACGGGGGGATGAGCTTCGATCTTCCC
ATGCCCATGTGTTCTACCCCCTCAGACACGAATATGGCATCCCCACTACCTATTCCGGACGGTTACACAAATCCGCACTGGAACGAATTCATAT
TTGGTTTAGGAACAGTTGGTAGCGATGGACCCCAGAATCCAGA
>Bcin05g01790(mrr1), partial sequence [organism=Botrytis cinerea, strain N11_K_W06]
CAGGCATCTGGCACGGAATGCATTTTCCCTACACGTGTTCGGAACCCAAGACCGAGACAAAGCAATCCAAAGTCTCGTGATTCTGAGCTATTAA
AAAGAATCTCTCACCTCGAAAGTTTGGTGAGCAGAATTGATGCCTCAAAACTCCTTGGGGAGGATGGTCATGTAGAAGACGAGGTTCGGTCAAA
TCCACCATCGTCAGGACCGCGACCTCATCGTTCTTTCACGTCTCCAGACCACCAATACAATGACAATCATCATGAGTTCGGAATAGTACCAGGT
CGTATGGCTCATCAATTTGCTGATTTTATCAAACGCCAAGAGACCGACTCGATTTACACCCATGATGGATTTTGGACAAGACTAAGTGAAGAAA
TTGATGGATTGAAACAGCTCATCGAGAGCCCTAGTGACGATGAAGATGAAAATTCTGGGTCCACTGCAACTTCGCCAGCATCCAAGTATGACTC
CCCTTCACAGTTTGTCTTTGATTCCAGGGTCACTTCCTCCAATGTCACAATTCCATATCCATCCCACGACCATACGAAAGTTTTATTCGATATT
TATTTCAAAAATGTACATCCGATGGTCGTCACTATGCACAAGCCAACAACGTACCTTTTCACAAACCCAAATTCTGAGTTACTCGATGAGAATA
CTGGCCGGCACAAATTCAAAAGTCTCGAGGCTAGCGCATTTTCTATGTACCTGGCTGCTGTGACGAGCATGACAAATGAAGAGTGCCTTGAGCT
```

TTTAAATGAAGAAAGACAGATTTTAGTAACAAGGTACAAAACTGCGACTGAGATTGCTCTTCAAGAATCAGATTTTCTTAATAGTGTAGAAATT
GTTACTTTACAAGCTTTGATAACGTATATCGCAGCTATGCGCGCTCATGATCGAAGTCGATCTACATGGACATTTATCGGTCTTGCAGTCCGCC
TTGCCCGTGGCATCGGGCTCCACAGGGATGGCACCGGGCTCCACAGAGATGGCAGCAAGCAACCTTTCGATTTGGAAATGCGAAGGAGACTATG
GTGGACATTGATTGTACTTGATACAAGAGCTTCCGAAGATCGGGGTACGGAAACTATGATCACTGATGGCTCTTTCGATACAAAGATGCCTGCC
AATATAAATGATGAGGATATATCGATAAACTCAAAAACTCTACCGGTCGATCGGCTAGGATTAACCAGTATGACCTTTGCTTGCATTACAATGA
CAGTCAGCGGGATCGGATTGAGGATGAATTTTGTACCTACGCGCCTAGACGCGCCGGTCTTGACCACAGAACAAAAAGAGCAGATGATCAAAGG
ATTTACTGACAAAATCGATTCTACCTATCTCGCAGGTTCTGATCCCAATGATCCAAGATTGTGGTGGTATTGTCGGATATCACGACTATTATCG
CTCAAGTTATGGCTGGTGACACAATATCCACTTCAACGAAGGAAATCTACAAATCGAGTGCTTCCTCGAGGACAATCCTTGCGTACAGCTATGG
CATTCCTAAATTTGGCGGAAGAAATATGGCAATATGAACCTTCAAAGGGATGGTTCTGGTTCTTTCAAAGTTATGTGCCGTGGCATGCAATTGC
GGTTGCTTTAGCTGAGCTTTGTGCGGAACCAACTGGCCCTCTTGCCGATCAAGCTTGGGGGAGCATCGAGTTTCATTACAATAAATGGAGTGAA
TTCGTTGCAGATACAAAAGATGGGATGATTTGGCGGCCAGTCAAGAATTTAATGAAAAGAGCAAAAGCAGCAAGAAGGCGAGAAAGAGGTCTCA
GTGAATCTCAAGCATCAACAACTCCAACGGCAATTCTCAACCCTAATACATACAGCCCAGCTCTCGATCTAGGTGCTGAATTCTCACCAATCAT
GTCGGAGAATAATAACTACCATCAACCTTCTACAATTGGGCAGGTTGACCCAATGGGGCAGAATATTTACGGGGGGATGAGCTTCGATCTTCCC
ATGCCCATGTGTTCTACCCCCTCAGACACGAATATGGCATCCCCACTACCTATTCCGGACGGTTATACAAATCCGCACTGGAACGAATTCATAT
TTGGTTTAGGAACAGTTGGTAGCGATGGACCCCAGAATCCAGA
>Bcin05g01790(mrr1), partial sequence [organism=Botrytis cinerea, strain N11_K_W08]
CAGGCATCTGGCACGGAATGTATTTTCCCTACACGTGTTCGGAACCCAAGACCGAGACAAAGCAATCCAAAGTCTCGTGATTCTGAGCTATTAA
AAAGAATCTCTCATCTCGAAAGTTTGGTGAGCAGAATTGATGCCTCAAAACTCCTTGGGGAGGATGGTCAGGTAGAATACGAGGTTCGGTCAAA
TCCGCCATCATCAGGACCGCGACCTCATCGTTCTTTCACGTCTCCAGACCATCATGAGTTTGGAATAGTACCAGGTCGTATGGCTCATCAATTT
GCTGATTTTATTAAACGCCAAGAGAACGACTCGATTTACACTCATGATGGATTTTGGACAAGACTAAGTGAAGAAATTGATGGATTGAAACAGC
TCATCGAGAGCCCCAGTGACGATGAAGATGAAAATTCTGGGTCCACTGCAACTTCGCCAGCATCCAAGTATGACTCTCCTTCACAGTTTGTCTT
TGATTCCAGGGTCACTTCCTCGAATGTCATAATTCCATATCCATCCCACGACCATACCAAAGTTTTATTCGATATTTATTTCAAAAATGTACAT
CCGATGGTCGTCACTATGCACAAGCCAACAACGTACCTTTTCACAAACCCAAATTCTGAGTTACTCGATGAGAATACTGGCCGGCACAAATTCA
AAAGTCTCGAGGCTAGCGTGTTTGCTATGTACCTGGCTGCTGTGACGAGCATGACAAATGAAGAGTGCCTTGAGCTTTTAAATGAAGAAAGAAA
TATTTTAGTAACACGGTACAAAACTGCGACTGAGATTGCTCTTCAAGAATCAGATTTTCTTAATAGTGTTGAAATTGTTACTTTACAAGCTTTA
ATAACGTATATCGTAAGTGTTTCTCTCTAAGCATCTTGGATGATGGTTTTATGATGATGATGATGATGATGTTTATGTACTTTCACAGGCAGCT
ATGCGCACTCATGATCGAAGTCGATCTATATGGGCATTTATCGGTCTTGCAGTCCGCCTTGCCCGTGGCATCGGGCTCCACAGAGATGGCAGCC
AGCAACCTTTCGATTTGGAAATGCGAAGGAGAGTGTGGTGGACATTGATTGTACTTGATACAAGAGCTTCCGAAGATCGGGGTACAGAAACCAT
GATCACTGATGGCTCTTTCGATACAAAGATGCCTGCCAACATAAACGACGAGGATATGATGATAAACTCAAAAAGTCTACCGGTCGATCGGATA
GGGATCACCAGTATGACCTTTGCTTGCATTACAATGACAGTCAGCGGGATCGGATTGAGGATGAATTTTGTACCTACGCGCCTAGACGCGCCGG
TCTTGACCACAGAACAAAAAGAGCAGATGATCAAAGGATTTACTGACAAAATCGATTCTACCTATCTCGCAGGTTCTGATCCCAATGATCCAAG
ATTGTGGTGGTATTGTCGGATATCACGACTATTATCGCTCAAGTTATGGCTGGTGACACAATATCCACTTCAACGAAGGAAATCTACAAATCGA
GTGCTTCCTCGAGGACAATCCTTGCGTACAGCTATGGCATTCCTAAATTTGGCGGAAGAAATATGGCAATATGAACCTTCAAAGGGATGGTTCT
GGTTCTTTCAAAGTTATGTGCCGTGGCATGTAATTGCGGTTGCTTTAGCTGAGCTTTGTGCGGAACCAACTGGCCCTCTTGCCGATCAAGCTTG
GGAGGGCATTGAGTTTCACTACAATAAATGGAGTGAATTCGTTGCAGATACAAAAGATGGGATGATTTGGCGACCAGTCAGGAATTTAATGAAA
AGAGCAAGAGCAGCAAGAAGGCGAGAAAGAGGTCTCAGTGAATCTCAAGCATCAACAACTCCAACGGCAATTCGCAACCCTAATACATACAGCC
CAGCTCTCAATCTAGCTGCTGGATGCTCCCCGATCATGTCGGAGAACAATAACTACTGTCAATCTTCTACAATTGGACAGGTTGACCCAATGGG
GCAGAATATTTACGGGGGAATGGGCTTCGATCTTCCCATGCCCATGGGTTCTACCCCCTCAGACACGAATATGGCATCCCCACTACCTATTCCG
GACGGCTATACAAATCCGCACTGGAACGAATTCATATTTGGTTTAGGAACAGTTTGTAGCGATGGACCCCAAAATCCAGA
>Bcin05g01790(mrr1), partial sequence [organism=Botrytis cinerea, strain N11_S_E08]
CAGGCATCTGGCACGGAATGCATTTTCCCTACACGTGTTCGGAACCCAAGACCGAGACAAAGCAATCCAAAGTCTCGTGATTCTGAGCTATTAA
AAAGAATCTCTCACCTCGAAAGTTTGGTGAGCAGAATTGATGCCTCAAAACTCCTTGGGGAGGATGGTCAGGTAGAAGACGAGGTTCGGTCAAA
TCCACCATCATCAGGACCGCGACCTCATCGTTCTTTCACGTCTCCAGACCACCAATACAATGACAATCATCATGAGTTCGGAATAGTACCGGGT
CGTATGGCTCATCAATTTGCTGATTTTATTAAACGCCAAGAGACTGACTCGATTTACACCCATGATGGATTTTGGACAAGACTAAGTGAAGAAA
TTGATGGATTGAAACAGCTCATCGAGAGCCCCAGTGACGATGAAGATGAAAATTCTGGGTCCACTGCAACTTCGCCAGCATCCAAGTATGACTC
CCCTTCACAGTTTGTCTTTGATTCCAGGGTCACTTCCTCGAATGTCACAATTCCATATCCATCCCACGACCATACGAAAGTTTTATTCGATATT
TATTTCAAAAATGTACATCCGATGGTCGTCACTATGCACAAGTCAACAACGTACCTTTTCACAAACCCAAATTCTGAGTTACTCGATGAGAATA
CTGGCCGGCACAAATTCAAAAGTCTCGAGGCTAGCGCATTTTCTATGTACCTGGCTGCTGTGACGAGCATGACAAATGAAGAGTGCCTTGAGCT
TTTAAATGAAGAAAGACAGATTTTAGTAACAAGGTACAAAACTGCGACTGAGATTGCTCTTCAAGAATCAGATTTTCTTAATAGTGTAGAAATT
GTTACTTTACAAGCTTTAATAACGTATATCGTAAGTATTTCTCTCTCAGCATCTTGGATGATGGTTTTATGATGATGATGATGATGATGTTTAT
GTACTTTCACAGGCAGCTATGCGCGCTCATGATCGAAGTCGATCTATATGGGCTTTTATCGGTCTTGCAGTCCGCCTTGCCCGTGGCATCGGGC
TCCACAGGGATGGCACCGGGCTCCACAGAGATGGCAGCAAGGAACCTTTCGATTTGGAAATGCGAAGGAGAATATGGTGGACATTGATTGTACT
TGATACAAGAGCTTCCGAAGATCGGGGTACGGAAACCATGATCACGGATGGCTCTTTCGATACAAAGATGCCTGCCAATATAAATGACGAGGAT
ATATCGATAAACTCAAAAACTCTACCGGTCGATCGGTTAGGATTCACCAGTATGACCTTTGCTTGCATTACAATGACAGTCAGCGGGATCGGAT
TGAGGATGAATTTTGTACCTACGCGCCTAGACGCGCCGGTCTTGACCACAGAACAAAAAGAGCAGATGATCAAAGGATTTACTGACAAAGTCGA
TTCCACCTATGTCACATGTTCTGATCCCAATGATCCAAGATTGTGGTGGTTTTCTCGGGTATCACGACTATTATCGCTCAAGTTATGGTTGGCG
ACACAATATCCACTTCAGCGAAGGAAATCTACAAATCGAGTGCTTCCTCGGGGACAATCCTTGCGTACAGCTATGGCATTCTTAAATTTGGCGG

AAGAAATATGGCAATATGAACCTTCAAAGGGATGGTTCTGGTTCTTTCAAAGTTATGTGCCGTGGCATGCAATTGCGGTTGCTTTAGCTGAGCT
TTGCGCGGAACCAACTGGCCCTCTTGCCGATCAAGCTTGGGGGAGCATCGAGTTTCATTACAATAAATGGAGTGAATTCGTTGCAGATACAAAA
GATGGGATGATTTGGCGGCCAGTCAAGAATTTAATGAAAAGAGCAAGAGCAGCAAGAAGGCGAGAAAGAGGTCTCAGTGAATCTCAAGCATCAA
CAACTCCAACGGCAATTCTCAACCCTAATACATACAGCCCAGCTCTCGATCTAGGTGCTGAATTCTCACCAATCATGTCGGAGAATAATAACTA
CCGTCAACCTTCTACAATTGGGCAGGTTGACCCAATGGGGCAGAATATTTACGGGGGGATGAACTTCGATCTTCCCATGCCCATGTGTTCTACC
CCCTCAGACACGAATATGGCATCCCCACTACCTATTCCGGACGGCTATACAAATCCGCACTGGAACGAATTCATATTTGGTTTAGGAACAGTTG
GTAGCGATGGACCCCAAAATCCAGA
>Bcin05g01790(mrr1), partial sequence [organism=Botrytis cinerea, strain N11_S_E10]
CAGGCATCTGGCACGGAATGTATTTTCCCTACACGTGTTCGGAACCCAAGACCGAGACAAAGCAATCCAAAGTCTCGTGATTCTGAGCTATTAA
AAAGAATCTCTCATCTCGAAAGTTTGGTGAGCAGAATTGATGCCTCAAAACTCCTTGGGGAGGATGGTCAGGTAGAAGACGAGGTTCGGTCAAA
TCCGCCATCATCAGGACCGCGACCTCATCGTTCTTTCACGTCTCCAGACCACCAATACAATGACAATCATCATGAGTTTGGAATAGTACCAGGT
CGTATGGCTCATCAATTTGCTGATTTTATTAAACGCCAAGAGACCGACTCGATTTACACTCATGATGGATTTTGGACAAGACTAAGTGAAGAAA
TTGATGGATTGAAACAGCTCATCGAGAGCCCCAGTGACGACGAAGATGAAAATTCTGGGTCCACTGCAACTTCGCCAGCATCCAAGTATGACTC
TCCTTCACAGTTTGTCTTTGATTCCAGGGTCACTTCCTCGAATGTCATAATTCCATATCCATCCCACGACCATACCAAAGTTTTATTCGATATT
TATTTCAAAAATGTACATCCGATGGTCGTCACTATGCACAAGCCAACAACGTACCTTTTCACAAACCCAAATTCTGAGTTACTCGATGAGAATA
CTGGCCGGCACAAATTCAAAAGTCTCGAGGCTAGCGTGTTTGCTATGTACCTGGCTGCTGTGACGAGCATGACAAATGAAGAGTGCCTTGAGCT
TTTAAATGAAGAAAGAAATATTTTAGTAACACGGTACAAAACTGCGACTGAGATTGCTCTTCAAGAATCAGATTTTCTTAATAGTGTTGAAATT
GTTACTTTACAAGCTTTAATAACGTATATCGCAGCTATGCGCACTCATGATCGAAGTCGATCTATATGGGCATTTATCGGTCTTGCAGTCCGCC
TTGCCCGTGGCATCGGGCTCCACAGAGATGGCAGCCAGCAACCTTTCGATTTGGAAATGCGAAGGAGAGTGTGGTGGACATTGATTGTACTTGA
TACAAGAGCTTCCGAAGATCGGGGTACAGAAACCATGATCACTGATGGCTCTTTCGATACAAAGATGCCTGCCAACATAAACGACGAGGATATG
ATGATAAACTCAAAAAGTCTACCGGTCGATCGGATAGGGATCACCAGTATGACCTTTGCTTGCATTACAATGACAGTCAGCGGGATCGGATTGA
GGATGAATTTTGTACCTACGCGCCTAGACGCGCCGGTCTTGACCACAGAACAAAAAGAGCAGATGATCAAAGGATTTACTGACAAAATCGATTC
TACCTATCTCGCAGGTTCTGATCCCAATGATCCAAGATTGTGGTGGTATTGTCGGATATCACGACTATTATCGCTCAAGTTATGGCTGGTGACA
CAATATCCACTTCAACGAAGGAAATCTACAAATCGAGTGCTTCCTCGAGGACAATCCTTGCGTACAGCTATGGCATTCCTAAATTTGGCGGAAG
AAATATGGCAATATGAACCTTCAAAGGGATGGTTCTGGTTCTTTCAAAGTTATGTGCCGTGGCATGTAATTGCGGTTGCTTTAGCTGAGCTTTG
TGCGGAACCAACTGGCCCTCTTGCCGATCAAGCTTGGGAGGGCATTGAGTTTCACTACAATAAATGGAGTGAATTCGTTGCAGATACAAAAGAT
GGGATGATTTGGCGACCAGTCAGGAATTTAATGAAAAGAGCAAGAGCAGCAAGAAGGCGAGAAAGAGGTCTCAGTGAATCTCAAGCATCAACAA
CTCCAACGGCAATTCGCAACCCTAATACATACAGCCCAGCTCTCAATCTAGCTGCTGGATGCTCCCCGATCATGTCGGAGAACAATAACTACTG
TCAATCTTCTACAATTGGACAGGTTGACCCAATGGGGCAGAATATTTACGGGGGAATGGGCTTCGATCTTCCCATGCCCATGGGTTCTACCCCC
TCAGACACGAATATGGCATCCCCACTACCTATTCCGGACGGCTATACAAATCCGCACTGGAACGAATTCATATTTGGTTTAGGAACAGTTTGTA
GCGATGGACCCCAAAATCCAGA
>Bcin05g01790(mrr1), partial sequence [organism=Botrytis cinerea, strain N11_S_E15]
CAGGCATCTGGCACGGAATGTATTTTCCCTACACGTGTTCGGAACCCAAGACCGAGACAAAGCAATCCAAAGTCTCGTGATTCTGAGCTATTAA
AAAGAATCTCTCATCTCGAAAGTTTGGTGAGCAGAATTGATGCCTCAAAACTCCTTGGGGAGGATGGTCAGGTAGAATACGAGGTTCGGTCAAA
TCCGCCATCATCAGGACCGCGACCTCATCGTTCTTTCACGTCTCCAGACCATCATGAGTTTGGAATAGTACCAGGTCGTATGGCTCATCAATTT
GCTGATTTTATTAAACGCCAAGAGAACGACTCGATTTACACTCATGATGGATTTTGGACAAGACTAAGTGAAGAAATTGATGGATTGAAACAGC
TCATCGAGAGCCCCAGTGACGATGAAGATGAAAATTCTGGGTCCACTGCAACTTCGCCAGCATCCAAGTATGACTCTCCTTCACAGTTTGTCTT
TGATTCCAGGGTCACTTCCTCGAATGTCATAATTCCATATCCATCCCACGACCATACCAAAGTTTTATTCGATATTTATTTCAAAAATGTACAT
CCGATGGTCGTCACTATGCACAAGCCAACAACGTACCTTTTCACAAACCCAAATTCTGAGTTACTCGATGAGAATACTGGCCGGCACAAATTCA
AAAGTCTCGAGGCTAGCGTGTTTGCTATGTACCTGGCTGCTGTGACGAGCATGACAAATGAAGAGTGCCTTGAGCTTTTAAATGAAGAAAGAAA
TATTTTAGTAACACGGTACAAAACTGCGACTGAGATTGCTCTTCAAGAATCAGATTTTCTTAATAGTGTTGAAATTGTTACTTTACAAGCTTTA
ATAACGTATATCGTAAGTGTTTCTCTCTAAGCATCTTGGATGATGGTTTTATGATGATGATGATGATGATGTTTATGTACTTTCACAGGCAGCT
ATGCGCACTCATGATCGAAGTCGATCTATATGGGCATTTATCGGTCTTGCAGTCCGCCTTGCCCGTGGCATCGGGCTCCACAGAGATGGCAGCC
AGCAACCTTTCGATTTGGAAATGCGAAGGAGAGTGTGGTGGACATTGATTGTACTTGATACAAGAGCTTCCGAAGATCGGGGTACAGAAACCAT
GATCACTGATGGCTCTTTCGATACAAAGATGCCTGCCAACATAAACGACGAGGATATGATGATAAACTCAAAAAGTCTACCGGTCGATCGGATA
GGGATCACCAGTATGACCTTTGCTTGCATTACAATGACAGTCAGCGGGATCGGATTGAGGATGAATTTTGTACCTACGCGCCTAGACGCGCCGG
TCTTGACCACAGAACAAAAAGAGCAGATGATCAAAGGATTTACTGACAAAATCGATTCTACCTATCTCGCAGGTTCTGATCCCAATGATCCAAG
ATTGTGGTGGTATTGTCGGATATCACGACTATTATCGCTCAAGTTATGGCTGGTGACACAATATCCACTTCAACGAAGGAAATCTACAAATCGA
GTGCTTCCTCGAGGACAATCCTTGCGTACAGCTATGGCATTCCTAAATTTGGCGGAAGAAATATGGCAATATGAACCTTCAAAGGGATGGTTCT
GGTTCTTTCAAAGTTATGTGCCGTGGCATGTAATTGCGGTTGCTTTAGCTGAGCTTTGTGCGGAACCAACTGGCCCTCTTGCCGATCAAGCTTG
GGAGGGCATTGAGTTTCACTACAATAAATGGAGTGAATTCGTTGCAGATACAAAAGATGGGATGATTTGGCGACCAGTCAGGAATTTAATGAAA
AGAGCAAGAGCAGCAAGAAGGCGAGAAAGAGGTCTCAGTGAATCTCAAGCATCAACAACTCCAACGGCAATTCGCAACCCTAATACATACAGCC
CAGCTCTCAATCTAGCTGCTGGATGCTCCCCGATCATGTCGGAGAACAATAACTACTGTCAATCTTCTACAATTGGACAGGTTGACCCAATGGG
GCAGAATATTTACGGGGGAATGGGCTTCGATCTTCCCATGCCCATGGGTTCTACCCCCTCAGACACGAATATGGCATCCCCACTACCTATTCCG
GACGGCTATACAAATCCGCACTGGAACGAATTCATATTTGGTTTAGGAACAGTTTGTAGCGATGGACCCCAAAATCCAGA
>Bcin05g01790(mrr1), partial sequence [organism=Botrytis cinerea, strain T4]
CAGGCATCTGGCACGGAATGTATTTTCCCTACACGTGTTCGGAACCCAAGACCGAGACAAAGCAATCCAAAGTCTCGTGATTCTGAGCTATTAA

AAAGAATCTCTCATCTCGAAAGTTTGGTGAGCAGAATTGATGCCTCAAAACTCCTTGGGGAGGATGGTCAGGTAGAATACGAGGTTCGGTCAAA
TCCGCCATCATCAGGACCGCGACCTCATCGTTCTTTCACGTCTCCAGACCATCATGAGTTTGGAATAGTACCAGGTCGTATGGCTCATCAATTT
GCTGATTTTATTAAACGCCAAGAGAACGACTCGATTTACACTCATGATGGATTTTGGACAAGACTAAGTGAAGAAATTGATGGATTGAAACAGC
TCATCGAGAGCCCCAGTGACGATGAAGATGAAAATTCTGGGTCCACTGCAACTTCGCCAGCATCCAAGTATGACTCTCCTTCACAGTTTGTCTT
TGATTCCAGGGTCACTTCCTCGAATGTCATAATTCCATATCCATCCCACGACCATACCAAAGTTTTATTCGATATTTATTTCAAAAATGTACAT
CCGATGGTCGTCACTATGCACAAGCCAACAACGTACCTTTTCACAAACCCAAATTCTGAGTTACTCGATGAGAATACTGGCCGGCACAAATTCA
AAAGTCTCGAGGCTAGCGTGTTTGCTATGTACCTGGCTGCTGTGACGAGCATGACAAATGAAGAGTGCCTTGAGCTTTTAAATGAAGAAAGAAA
TATTTTAGTAACACGGTACAAAACTGCGACTGAGATTGCTCTTCAAGAATCAGATTTTCTTAATAGTGTTGAAATTGTTACTTTACAAGCTTTA
ATAACGTATATCGTAAGTGTTTCTCTCTAAGCATCTTGGATGATGGTTTTATGATGATGATGATGATGATGTTTATGTACTTTCACAGGCAGCT
ATGCGCACTCATGATCGAAGTCGATCTATATGGGCATTTATCGGTCTTGCAGTCCGCCTTGCCCGTGGCATCGGGCTCCACAGAGATGGCAGCC
AGCAACCTTTCGATTTGGAAATGCGAAGGAGAGTGTGGTGGACATTGATTGTACTTGATACAAGAGCTTCCGAAGATCGGGGTACAGAAACCAT
GATCACTGATGGCTCTTTCGATACAAAGATGCCTGCCAACATAAACGACGAGGATATGATGATAAACTCAAAAAGTCTACCGGTCGATCGGATA
GGGATCACCAGTATGACCTTTGCTTGCATTACAATGACAGTCAGCGGGATCGGATTGAGGATGAATTTTGTACCTACGCGCCTAGACGCGCCGG
TCTTGACCACAGAACAAAAAGAGCAGATGATCAAAGGATTTACTGACAAAATCGATTCTACCTATCTCGCAGGTTCTGATCCCAATGATCCAAG
ATTGTGGTGGTATTGTCGGATATCACGACTATTATCGCTCAAGTTATGGCTGGTGACACAATATCCACTTCAACGAAGGAAATCTACAAATCGA
GTGCTTCCTCGAGGACAATCCTTGCGTACAGCTATGGCATTCCTAAATTTGGCGGAAGAAATATGGCAATATGAACCTTCAAAGGGATGGTTCT
GGTTCTTTCAAAGTTATGTGCCGTGGCATGTAATTGCGGTTGCTTTAGCTGAGCTTTGTGCGGAACCAACTGGCCCTCTTGCCGATCAAGCTTG
GGAGGGCATTGAGTTTCACTACAATAAATGGAGTGAATTCGTTGCAGATACAAAAGATGGGATGATTTGGCGACCAGTCAGGAATTTAATGAAA
AGAGCAAGAGCAGCAAGAAGGCGAGAAAGAGGTCTCAGTGAATCTCAAGCATCAACAACTCCAACGGCAATTCGCAACCCTAATACATACAGCC
CAGCTCTCAATCTAGCTGCTGGATGCTCCCCGATCATGTCGGAGAACAATAACTACTGTCAATCTTCTACAATTGGACAGGTTGACCCAATGGG
GCAGAATATTTACGGGGGAATGGGCTTCGATCTTCCCATGCCCATGGGTTCTACCCCCTCAGACACGAATATGGCATCCCCACTACCTATTCCG
GACGGCTATACAAACCCGCACTGGAACGAATTCATATTTGGTTTAGGAACAGTTTGTAGCGATGGACCCCAAAATCCAGA
>Bcin05g01790(mrr1), partial sequence [organism=Botrytis calthae, strain BcalMUCL2830]
CAGGCATCTGGTACGGAATGTATCTTCCCTACACGTGTTCGGAACCCAAGACCGAGACAAAGCAATCCAAAGTCTCGTGATTCTGAGCTATTGA
AGAGAATCTCTCACCTCGAAAGTTTGGTGAGCAGAATTGATGCCTCAAAACTCCTTGGGGAGGATGGTCAGGTAGATGACGAGGTTCGGTCAAA
TACACCATCCTCAGGACCGCGACCTCATCGTTCTTTCACGTCTCCAAACCACCAATACAATGACAATCACCATGAGTTTGGAATAGTACCAGGA
CGTATGGCTCATCAATTTGCTCATTTTATTAAACGCCAAGAGACCGACTCGATTTACACTCATGATGGATTTTGGACAAGATTAAGTGAAGAAA
TTGATGGATTGAAGCAGCTCATCGAGAGCCCCAGTGATGATGAAGATGAAAATTCTGGGTCAACTGCAACTTCGCCAGCATCCAAGTATGACTC
CCCTTCACAGTTTGTCTTTGATTCCAGGGTCACTTCCTCGAATGTCATAATTCCATATCCATCCCACGACCATGTCAAAATTTTATTCGATTTT
TATTTCAAAAATGTACATCCGATGGTCGTCACTATGCACAAGCCAACAACGCACCTTTTCACAAACCCAAATTCTGAGTTATTTGATGAGAATA
CTGGCCGGCACAAATTCAAAAGTCTTGAGGCTAGCGCATTTGCTATGTACCTGGCTGCTGTGACAAGCATGACAAATGAAGAGTGCCTTAAGCT
TTTAAATGAAGAAAGACATATTTTAGTAACACGGTACAAAACTGCGACTGAGATTGCTCTTCAAGAATCAGATTTTCTTAATAGTGTAGAAATT
GTTACTTTACAAGCTTTAATAACGTATATCGTAAGTGTTTCTCTCTAAGCATCTTGGATGATGGTTTTATGATGATGATGATGTTTATGTACTA
TCACAGGCAGCTATGCGCACTCATGATCGAAGTCGATCTGTATGGGCATTTATCGGTCTTGCAGTCCGCCTTGCCCGTGGCATCGGGCTCCACA
GAGATGGCAGCCAGCAACCTTTCGATTTGGAAATGCGAAGGAGAGTATGGTGGACGTTGATTGTACTTGATACAAGAGCTTCCGAAGATCGGGG
TACAGAAACCATGATTACTGATGGCTCTTTCGATACAAAGATGCCTGCCAACGTAAACGACGAGGATTTAACGATAAACTCAAAAACTCTACCG
GCCGATCGGATAGGACTCACCAGTATGACCTTTGCTTGCATTACAATGACAGTCAGCGGGATCGGATTGAGGATGAATTTTGTACCTACGCGCC
TAGACGCGCCGGTCTTGACCACAGAACAAAAAGAGCAGATGATCAAAGGATTTACTGACAAAATCGATTCTACCTATCTCGTAGGTTCTGATCC
CAATGATCCAAGAATGTGGTGGTTTTGTCGGATATCACGACTATTATCGCTCAAGTTATGGTTGGTGATACAATATCCACTTCAGCGAAGGAAA
TCTACAAATCGAGTGCTTCCTCGAGGACAATCCTTGCGTACAGCTATGGCATTCCTAAATTTGGCGGAAGAAATATGGCAATATGAACCTTCAA
AGGGATGGTTCTGGTTCTTTCAAAGTTATGTGCCGTGGCATGCAATCGCGGTTGCTTTAGCTGAGCTTTGTGCGGAACCAACTGGCCCTCTTGC
CGATCAAGCTTGGGAGGGCATTGAGTTTCATTACAATAAATGGAGTGAATTCGTTGCAGATACAAAAGATGGGATGATTTGGCGACCAGTCAAG
AATTTAATGAAAAGAGCAAGAGCAGCAAGAAGGCGAGAAAGAGGTCTCAGTGAATCTCAAGCATCAACAACTTCAACGGCGATTCCCAACCCTA
ATACATACAGCTCAGCTCCCAATCTAGGTGCTGAATTCTCTCCAACCATGTCGGAGAATAATGTCTACCGTCAACCTTCTACAATTGTGCAGGT
TGACCCAATGGGGCAGAATATTTATGGGGGGATGGGCTTCGATCTTTCCATGCCCATGGGTTCTACCTCCTCAGACACGAATATGGCATCCCCA
CTATCTATTCCGGACGGCTATACAAATCCACACTGGAACGAATTCATATTTGGTTTAGGAACAGTTGGTAGCGATGGACCTCAAAATCCAGA
>Bcin05g01790(mrr1), partial sequence [organism=Botrytis cinerea, strain D08_H_8_3]
CAGGCATCTGGCACGGAATGCATTTTCCCTACACGTGTTCGGAACCCAAGACCGAGACAAAGCAATCCAAAGTCTCGTGATTCTGAGCTATTAA
AAAGAATCTCTCACCTCGAAAGTTTGGTGAGCAGAATTGATGCCTCAAAACTCCTTGGGGAGGATGGTCAGGTAGAAGACGAGGTTCGGTCAAA
TCCACCATCGTCAGGACCGCGACCTCATCGTTCTTTCACGTCTCCAGACCACCAATACAATGACAATCATCATGAGTTCGGAATAGTACCAGGT
CGTATGGCTCATCAATTTGCTGATTTTATCAAACGCCAAGAGACCGACTCGATTTACACCCATGATGGATTTTGGACAAGACTAAGTGAAGAAA
TTGATGGATTGAAACAGCTCATCGAGAGCCCCAGTGACGATGAAGATGAAAATTCTGGGTCCACTGCAACTTCGCCAGCATCCAAGTATGACTC
CCCTTCACAGTTTGTCTTTGATTCCAGGGTCACTTCCTCCAATGTCACAATTCCATATCCATCCCACGACCATACGAAAGTTTTATTCGATATT
GATTTCAAAAATGTACATCCGATGGTCGTCACTATGCACAAGCCAACAACGTACCTTTTCACAAACCCAAATTATGAGTTACTCGATGAGAATA
CTGGCCGGCACAAATTCAAAAGTCTCGAGGCTAGGGCATTTTCTATGTACCTGGCTGCTGTGACGAGCATGACAAATGAAGAGCGCCTTGAGCT
TTTAAATGAAGAAAGACAGATTTTAGTAACAAGGTACAAAACTGCGACTGAGATTGCTCTTCAAGAATCAGATTTTCTTAATAGTGTAGAAATT
GTTACTTTACAAGCTTTGATAACGTATATCGTAAGTATTTCTCTCTCAGCATCTTGAATGATGGTTTTATGATGATGATGATGATGTTTATGTA

CTTTCACAGGCAGCTATGCGCGCTCATGATCGAAGTCGATCTACATGGACATTTATCGGTCTTGCAGTCCGCCTTGCCCGTGGCATCGGGCTCC
ACAGGGATGGCACCGGGCTCCACAGAGATGGCAGCAAGCAACCTTTCGATTTGGAAATGCGAAGGAGACTATGGTGGACATTGATTGTACTTGA
TACAAGAGCTTCCGAAGATCGGGGTACGGAAACTATGATCACTGATGGCTCTTTCGATACAAAGATGCCTGCCAATATAAATGATGAGGATATA
TCGATAAACTCAAAAACTCTACCGGTCGATCGGCTAGGATTAACCAGTATGACCTTTGCTTGCATTACAATGACAGTCAGCGGGATCGGATTGA
GGATGAATTTTGTACCTACGCGCCTAGACGCGCCGGTCTTGACCACAGAACAAAAAGAGCAGATGATCAAAGGATTTACTGACAAAATCGATTC
TACCTATGTCACATGTTCTGATCCCAATGATCCAAGATTGTGGTGGTTTTGTCGGGTATCACGACTATTATCGCTCAAGTTATGGTTGGCGACA
CAATATCCACTTCAGCGAAGGAAATCTACAAATCGAGTGCTTCCTCGAGGACAATCCTTGCGTACAGCTATGGCATTCTTAAATTTGGCGGAAG
AAATATGGCAATATGAACCTTCAAAGGGATGGTTCTGGTTCTTTCAAAGTTATGTGCCGTGGCATGCAATTGCGGTTGCTTTAGCTGAGCTTTG
TGCGGAACCAACTGGCCCTCTTGCCGATCAAGCTTGGGGGAGCATCGAGTTTCATTACAATAAATGGAGTGAATTCGTTGCAGATACAAAAGAT
GGGATGATTTGGCGGCCAGTCAAGAGTTTAATGAAAAGAGCAAAAGCAGCAAGAAGGCGAGAAAGAGGTCTCAGTGAATCTCAAGCATCAACAA
CTCCAACGGCAATTCTCAACCCTAATACATACAGCCCAGCTCTCGATCTAGGTGCTGAATTCTCACCAATCATGTCGGAGAATAATAACTACCG
TCAACCTTCTACAATTGGGCAGGTTGACCCAATGGGGCAGAATATTTACGGGGGGATGAGCTTCGATCTTCCCATGCCCATGTGTTCTACCCCC
TCAGACACGAATATGGCATCCCCACTACCTATTCCGGACGGTTATACAAATCCGCACTGGAACGAATTCATATTTGGTTTAGGAACAGTTGGTA
GCGATGGACCCCAGAATCCAGA
>Bcin05g01790(mrr1), partial sequence [organism=Botrytis pseudocinerea, strain BpsVD256]
CAGGCATCTGGCACGGAATGTATTTTCCCTACACGTGTTCGGAATCCAAGACCGAGACAAAGCAATCCAAAGTCTCGTGATTCTGAGCTATTGA
AAAGAATCTCTCATCTCGAAAGTTTGGTGAGCAGAATTGATGCCTCAAAACTCCTTGGGGAGGATGGTCAGGTAGAAGACGAGGTTCGGTCAAA
CCCACCATCATCAGGACCGCGACCTCATCGTTCTTTCACGTCTCCAGACCGCCAATACAATGACAACCACCATGAGTTTGGAATAGTACCAGGT
CGTATGGCTCATCAATTTGCTGATTTTATTAAACGCCAAGAGACCGACTCGATTTACACTCATGATGGATTTTGGACAAGATTAAGTGAAGAAA
TTGATGGATTGAAACAGCTCATCGAGAGCCCCAGTGACGATGAAGATGAAATTTCTGGGTCAACTGCAACCTCGCCAGCATCCAAGTATGACTC
CCCTTCACAGTTTGTCTTTGATTCCAGGGTCACTTCCTCGAATGTCATAATTCCATATCCATCCCACGACCATACCAAAATTTTATTCGATATT
TATTTCAAAAATGTACATCCGATGGTCGTCACTATGCACAAGCCAACAACGTACCTTTTCTCAAAACCAAATTGTGAGTTAATGGATGAGAATG
CTGGCCGGCACAAATTCAAAAGTCTCGAGGCTAGCGCATTTTATATGTACCCGGCTGCTGTGACGAGCATGACAAATGAAGAGTGCCTTGAGCT
TTTAAATGAAGAAAGACAGATTTTAGTAACAAGGTACAAAACTGCGACTGAGATTGCTCTTCAAGAATCAGATTTTTTTAATAGTGTAGAAATT
GTTACTCTACAAGCTTTAATCACGTATATCGTAAGTGTTTCTCTCTACACATCTTAGATGATAGTTTTACGATGATGATGATGTTTATGTACTT
TCACAGGCAGCTATGCGCACTCATGATCGAAGTCGATCTACATGGGCATTTATCGGTCTTGCAGTCCGCCTTGCCCGTGGCATCGGGCTCCACA
GGGATGGCAGCACGCAATCTTTCGATTTGGAAATGCGAAGGAGAATATGGTGGACATTGATTGTACTTGATACAAGAGCCTCCGAAGATCGGGG
TACAGAAACTATGATCACTGATGGCTCTTTCGATACAAAGATGCCTGCCAATATAAATGACGAGGATATCTTGATAGACTCAAAAACTCTACCA
GTCGATCGGATAGGATTCACCAGTATGACCTTTGCTTGCATTACAATGACAGTCAGCGGGATCGGATTGAGGATGAATTTTGTACCTACGCGCC
TAGACGCGCCGGTCTTGACCACAGAACAAAAAGAGCAGATGATCAAAGGATTTACTGACAAAATCGATTCTACCTATGTCACAGGTTCTGATCC
CAATGATCCAAAATTGTGGTGGTTTTGTCGGATATCACGACTATTATCGCTCAAGTTATGGTTGGCGACACAATACCCACTTCAGCGAAGGAAA
TCTACAAATCGAGTGCTTCCTCGAGGACAATCCTTGCGTACAGCTATGGCATACTTAAATTTGGCGGAAGAAATATGGCAATATGAACCTTCAA
AGGGATGGTTCTGGTTCTTTCAAAGTTATGTGCCGTGGCATGCAATTGCGGTTGCTTTAGCTGAGCTTTGTGCGGAACCAACTGGCCCTCTTGC
CGATCACGCTTGGGGGAGCATTGAGTCTCATTACAATAAATGGAGTGAATTCGTTGCAGATACAAAAGATGGGATGATTTGGCGGCCAGTCAAG
AATTTAATGAAAAGAGCAAGAGCAGCAAGAAGGCGAGAAAGAGGTCTCAGTGAATCTCAAGCATCAACAACTCCAACGGCAATTCTCAACCCTA
ATATATACAGCCCAGCTCTCGATCTAGGTGCTGAATTCTCACCAATCATGTCGGAGAATAATAACTACCGTCAACCTTCTACAATTGGGCAGGT
TGACCCAATGGGGCAGAATATTTACGGGGGGATGAGCTTCGATCTTCCCATGCCCATGTGTTCTACCCCCCTCAGACACGAATATGACATCCCC
ACTACCTATTCCGGACGGCTATACAAATCCGCACTGGAATGAATTCATATTTGGCTTAGGGAACAGTTGGTAGCGATGGACCCCAAAACCCAGA
