# Supplementary material for: Genetic Diversity of Botrytis cinerea Revealed by Multilocus Sequencing, and Identification of B. cinerea Populations Showing Genetic Isolation and Distinct Host Adaptation
Source: Front Plant Sci. 2021 May 5;12:663027. doi: 10.3389/fpls.2021.663027 (PMC8131559; doi:10.3389/fpls.2021.663027)
Supplement: Supplementary file 3 [file Data_Sheet_3.pdf]

>Bcin01g07220(MLST1), partial sequence [organism=Botrytis calthae, strain MUCL2830]
GATGGCATATCTTGTTTCTTTTCGCATTATGCTTTAGAACGCGCTGGACAAGCTTGGGTCAGTGCTCTACGTGTAGAAGCACTAAAGAAGATTC
TCGCACAACCGAAGTCATGGTTTGAGGAATCCAGTAATTCACCTGGCCGGTTGAACGAAGTTTTGGATAGGAACTCCGAGGAAATGCGTAATCT
CGTTGGTCGCTTTGTTGGTATTGTATTCACAGCATTTTTTATGCTATTGATATCAATCATCTGGGCTTTCGTGAATACATGGAAACTGTCATTA
GTCTCAATGGCAACTGGGCCAGTTATATACGGTGTCACCAAAACCTTCAATCGCGTGAGTGGAAAATGGGAAAACAAGTGCAACTACGCATCTG
AAATGGCCACCGGCATATTTTCAGAGACTTTCTCCAACATCAAAGTGGTTCGGGCTTTTACTCTGGAAACTTACTTTGAGACAAAACACACCAA
AGCTACAGAAGAACTTTATAAAGTTGGACTAATGCGAGCAAACTACTCGGGATTACTGTGGGGACTGACAGATGCGATGTCATTCTTCATCACT
GCAACTATCTTTTATTATGCCACGGTTCTCATTACCAAGAGAGAGATCAGTATCGCGACTGCACTACAGACTGTCAATCTTTTATTATTTGGTA
TTTCTAATAGTACGAATATGCTGGCCATGATACCACAAATCAACTCTTCTCGCGTTACAGCTACGCATATGCTTGCATTAGCCAATCTCGATTC
ATCTTCCTCCCACGAAAATAAAGGAACCGAACGGCTTTCGACAATCTTTCCAATCGAATTTAACCGCCTCTCATTCACATATCCTGCTCGTCCT
GAAACACGAACGATATCATCTTTTTCTCTTTCCCTGAGTCCTAACTCAACAACTGCACTTGTCGGACCCTCCGGCTCCGGAAAATCTACAATAG
CTGCTCTGCTAATTGGTCTTTATCCGCCAGATACTTCAACACCTCCACCGTTGACATTTAATCGCGTTTCCATAAGCAACTGTCACATTCCGTC
TCTCCGGGCTTCTATCTCACTCGTCCCACAAACACCGATTCTATTTCCAGCTACTATTCTCCATAACATCATCTATGGTCTTCCAGAATCTTCT
CCTTATGCTAATCTTCCATCTGCTATTGCATCAGCCAAAGATGCTGGGATCCATGAATTTATCACGTCACTTCCACAATGTTATAATACTAT

>Bcin05g07690(MLST2), partial sequence [organism=Botrytis calthae, strain MUCL2830]
ACCACCATCACTAAGTCCTTTTCTGCACTTTCCTCACCAAAAACGAACGAGAACATCATCAATAAATACTTTGCTGAAGTAATTGGCTTTTATT
TTGGCCAGGATGCCTTCTCTCTACGACAGCAAGCCTATGATGATATGTTATGGGTAGTCCTGGGTTGGTTGGATACTGTTAAATTCATTGATTT
ACACTCTGAATTACACTATTCGAACGACTCTCAGCCAGAATGGTACGGACAACAATATAAACCTGCATTTGCACACCGAGCGCGACTGTTTTGG
GAATTGGCGTCACAAGGATGGGATACTACCCTCTGTGGTGGTGGGATGATATGGTCTCCATACCTTACTCCATACAAGAACGCAATTACCAATG
AACTCTATATCGCAGCTTCGATATCGATGTACCTCTATTTCCCTGGAGATGACAATCAATCGCCATTTATGCTTTCCAACCCTTCATACCCACC
TCGCGATCCGAAACATCTACAGGCAGCTGTTGATGCTTACAAATGGCTGAATAGTTCCAACATGACGGATTTGCAAGGATTATATGTCGACGGA
TATCATATCTCGAATCTTTCTGGCGGTGAAAACACCCATTGCGATTCTAGAAACGAGATGGTATATACCTACAATCAAGGTGTTTTGCTTACTG
GACAACGTGGTTTGTATGACGCCACCGCCGCACGATCATACCTTGTGGATGGCCACAAACTCATCGCGAATGTTATTAATGCCACAGGCTATGA
TCTGAAACACAACGTTGCCATCTCACCGCCACCCAAAGATGGTTCCGAATTGGCAAAGTGGTTTGGCCTGGGTAGGAATGGAATACTGGAAGAA
GGTTGCGATGCAAGTGCTTCGTGTTCTCAAAATGGACAGACTTTCAAAGGCATATTCTTTCATCATTTGATTGCATTCTGTAGTGATTTGCCAA
GGGAGCCTATTGCAGGGACGAAAGAAGGCCTGGAACTCGATAGAGAGTGGCATTCTGAAAAATGCTCACAGTATACAAAATG

>Bcin06g01710(MLST3), partial sequence [organism=Botrytis calthae, strain MUCL2830]
TAGGTTCTTAGGACAAACCAAATCTTTAAGCTTCGGAAGCGTCTTCCGAGAGGAATTTGCCAGTCCCGATAGTCGGTTACGAGTCCACTCTCTG
AAAACTCGTTGCGAAGATAACTTGGTGTTATGGAGAGTGCATCGATCAAATCTTTGCGTTTCTTGACATATAGACAAGAAGCGTCGAAATTTGT
AAGAAGCCATTTGTGCATGTTCATATCGAAGGAATGGAAATGTTGGAAGGACGATGTTAGATGATGGTATTCAGGGCAAACCAAAGCTGCACCT
GCATAAGCAGCATCGACGTGAACCCAGATCTCGCCTGCAACATCTGGAGGAGCATATTTGGAAAGCACTGTTGCGATAGACGCGAAGTCATCAA
CTGCGCATGTAGATGTTGTTCCCAAAGTCGAAGTTAGATAGAAGGGTTCCAATCCTTGAGATTTGCATTCTTTCAATACCTTCTCTAGATCATC
ACCCGTCATGGCGAAATCATTGGATGCGAGTACTGGAATCGATCGGAATCTAACGCCAGCTATTTGCGCTGCTTTCTGCGTGGAGCTGTGTGCC
ATTTCGCTTCCTAGTGCAACTAGCTTACTCCTCTTGTGTGCAATCGCATCCTCGAGTTCAATACCTGACAGACCTTCAGTAGTTTCACGAAGAT
ATTTATCGCGGGCAGCGACCATAACGGTAACGATAGCTTCTGAAGCTGATCCTTGAATGACACCACCACCATGAGTCGAAGACAAATAACAGTC
TGGGAGATTGAGAAGCTTGGCCAGCCAATCCATTACAACCGTCTCCAATTCTGTCACAGCAGGGGAACAGATCCAATTGAAAGCAGGAGCTGTG
AAAGCTGCTGAGTATAATTCTCCAAGCATTCCAGGGTAGGTAGATGATGCAGGGAAGAAAGCCATGAAATTAGGCGATTGCCTTGGTATATCAG
TGTCTATCTTAACATCCAAATATAAAAGTCAGACTCAC

>Bcin09g03030(MLST4), partial sequence [organism=Botrytis calthae, strain MUCL2830]
CCCTTCAATCCCACCACAGCTCCTGTACCAAAAGCGAGTATCCTACCAATTCTCCTTCCGCCTCCGACTTTAAGACCATTGGCTTTTCGCACTT
TCACAAAAAAGCATAGTTTAACATTGACGTCGTCGGCATTACAAGTGTTGGCTACCTTTATTGGAAAGCATTGCGGGACAGGATGGAGGGAAGA
AGGATTGGCAGAGAGAGTTTTAGAGGAGGTTGCAAAGAGTTGGAAGAATAGGAGTGGCGGTGTTATTGTCGAGGGCGAGGGGACGGAATTGAAG
GAGATTCTGAAAGCTCTGGAAGGGAATATGAGCGGTGGAAGGATAGTCGTAGGAAGAGAATTAAGCCGGCAGAACAGTTTAGTACTGGGGTCAT
CACAATATGGAGAGGTCAATCATACGAGACTTGGACTACGGCCTGGGAATATACCTAGAGAGGATAGTCAGTCAAGTTTGGGGATGTCAACGTT
GGAGGTCAATGACGAGGAAGATGAGGATGGCCTGATGGATCCAAGGAGGTGGTTAAAAGTCATTGATGCATTTGAGCAACCTCGACTGGTGTAT
AATGTTGCTAAGAAGCACTTTGATAGGTATGTTTTAATGATAAAAATTTCATCTGAATCGACTAACTCAGTACAGAGATACCTCCAAACCTTCA
TTGTTCCCACCTGCGTCTCATAAAACACTTCTCTTCCAAAACCGCTACAATGTTATCCATCAACGTCTCCTTCGCAATGAATCTTTTCAAACGC
CCACTTTTCAAGGTGGCAAAGCTTCCCTTCAACGCAGCACGTCCACTATTACCACCCAGCAACATTCATACAAATTAACGCCGATAGCTAATCT
TCTTGGCCGCAATCGCAGCTCTCATATGCTTCTCGGCCTCCTCAGTATTTCACCCACTGGTACCCTCGCCATTAATGACCTGACGGGCAGCATC
GCTCTCGACCTTACACACGCAACATCCAT

>Bcin11g01310(MLST5), partial sequence [organism=Botrytis calthae, strain MUCL2830]
CCACTGACATGGACCTCATGTGGAACTGGTGTAGAATGCGCAACGCTTGAAGTTCCGCTCGAATATGGCGACGCGACGTCAACAGCAAAAGCCA
GTGTTGCGCTTGCTCGTTATCCTGCCACTGTTGCCGCGAGCAAGAAGCTCGGGTCTCTCTTGATAAATCCCGGTGGACCTGGTGCCAGTGGTGT

TGGATTTGTGCAGTCTGGGGCCGGTGCCGCGGTCTCAACACTGAGTGGTGGATTATATGATATCATCGGATGGGATCCACGTGGAACCGGTGCT
TCGGCTCCTATTTTGGAATGTTTTCCAAATGCCACTGCGGAGTATGATTTTAACAACGCGTTTCCATCTGCTCCAAATCTCTGGCTCGGACAAT
TTTCGAATGCCAGCGCAGATTCTGCTGTTAGCTCTGCTATCACATCTTTCGACTCTTCTGTCGCTGCTCTTGCAGAAGCTTGTGTGGCTCAGAA
TTCTCCCGCTCTGTACACCTCAACAGCAGCATATGTTGCTCGAGACATGGCAGCGATAGTCGATGCATTGGATGGGACCTCTGCAAAACTCAAC
TACTGGGGTTTCTCATATGGAACCATTTTCCTCGCCGAGTTTATCCAAACTTTCCCAGGTCGCGTGGGAAGAATTCTTGCCGATGGTGTTTTTG
ACGCAGAGGCAAATGCCCTCACTTACGTTAGCCAACTTCCCAACGATCAACTCAGTGTTCGTGCTTCGTTAAACGATTTTGCAGCTTTCTGCAC
CACCGCCGGTAGTGGAGGTTGCTCTTTTGCCACCGCTCCTGCTGGAACCACAGGTACTGTTGCCACTAGACTGGACAACATAATGGAGGATATG
TTCCTCAATCCTATTGTTGCTTCGGGCTTAAGCATCA

>Bcin15g03910(MLST6), partial sequence [organism=Botrytis calthae, strain MUCL2830]
GCCAAAGCACAAAATCTTCTAACGATGAAGATGATACTCCACTGCCCTTGATCATCTGGCATGGACTCGGCGATAATTATAAAGCGGATGGTCT
TGCGCAGGTTGGGAAATTAGCTGAAGCTATTCATCCCGGAATTTTTGTCTATAATATTCATGTAGATGAGGACGCATCTGCAGATAGGACGGCT
ACCTTCTTTGGAAATCTCACTCGTGAGTACATCCCCTAGTTTTCCTTCAAATACCATACTAACTCTCTTACCAAGTTCAAATTGAAAAGGTCTG
CGAAGACCTCGCCTCCCATCCTATTCTTTCTACCGCGCCCGCTGTCGACGCAATCGGATTCTCCCAAGGCGGCCAATTCTTGCGCGGTTACATA
TCCCGCTGCAATTCTCCACCCATCCGCTCTCTCCTGACCTTCGGCTCCCAACACAACGGCATTTCTGCCTTCCAAGCCTGTGGTCCTACCGATT
TCCTCTGTCGCGGTGCTCAAACCCTTTTGCGATCCAACACCTGGTCAACCTTTGTCCAATCTCGTCTCGTACCCGCTCAATACTTCAGAGATCC
GGAAAACCTAGACTCTTACCTTGAACATTCCAATTTCCTCGCCGACATCAACAATGAGCGCGTTCTCAAGAACGAAACATATAAATCCAACATG
GAAAAATTGGAACGATTCGTAATGTACGTCTTTGAAGACGACACAACCGTCATTCCTAAGGAAAGTGGATGGTGGGCTGAAGTCAACGGTACGG
AAGTTACACCACTGAAAGAAAGAGCCATTTATAAAGAAGATTGGGTAGGATTGAAGACATTGGATGAGGCTGGAAAATTAGTTTTCGAAACCAT
TCCAGGGGGTCATATGACGTTAGGAGAAGAAATGCTAGAGAAGGCTTTCAAAGAGTACTTTGGTCCAGCAGGGAAGAAATTTGGG

>Bcin16g03460(MLST7), partial sequence [organism=Botrytis calthae, strain MUCL2830]
ATGAACCTCTTAATTTGAACTCTTCATTAATTGCAAGTGATGAACCCCTATTTTGTCCAGAAGATAGTTACAAGACGTACATCATTAGTCGAGA
ACCACTCATGATATATATTGAAGGATTTTTGAAAGCAAATGAAAGTAAACATTTGGTTGATGTTAGGTATGTTATTTATTCTGATGAGATGAAC
AAGAGAAACTGATGAGATAGTGAGCCGCTTTATGAACCATCTACTGTTTCTCATGGACAGGAAGTTACCATTGATACTTCGGTTCGAAATTCCG
AAGTGGCGGTTTTAGAGAGGGATGAGGTGGTCAGGTGTATTGAGCATAGAGCGAGAGCATTTCAGGGGTGGAGGGGGGAGATGGGGATTGAGAA
GTTGAGGACGCAGAGGTATGGGGTTGGAGGACATTATGGGATGCACTTGTAAGTTTTGAGGGATTGACGAAGGCTTTTGTCTATTTTCTATCAA
TACGGATCTTTAAAGAGAGATTTCATGAACACGTGGGCTAATGACCAGGAAATAGTGATTGGAGCGGAGGCAAACGTGGCATAGACCGATTCAG
TACTTTCATGGTCTATGTCGACGTATCCTCTGATATCGAAGGTGGAGGAACGGAATTCCCACGTATCGTGGGGCCAAAAGGAGGAAGGTGGGAG
GAATTCCTGGATACTACCGAAGCATTAGATCCAAGAACTGGAAAAAATGTAACAGTAGAAGGGGTGACATTCAAACCTATCAAGGGAAATGCCG
TATTCTGGGAAAATACTGACAAGGACGGGAGGGGATATGAT

>Bcin12g03020(MLST8), partial sequence [organism=Botrytis calthae, strain MUCL2830]
CGATTGGCTGCGAAGAAAACTGCGCAGCACCGAGCCACTAAAAGCACGAATAAACCATCGGCAAGAGAAATATTCTCTGAACCACAACATGACA
CGAGCGCGGAAGAATATATCGGGCGAGGTGCTTCATCAAGAGCACCAAAGCGACAACGAGTCGATGATAATTACAACTCTTACGGTGGAAGAAA
TGAAAATACAGCAGCTTATGCTTCCGGAAAGCTTCCATCTGGAAGTATAAATGTTGGTGGAGGTAGGAAGACCACTTTTCAAGAAGAACCTCGA
ACGGCATTTGTCGCTGGCAAGTTGCCCCCTGGAAGTATCAACATCGGTGGAAAGAAGGCTACCCAAATCGAGGACGAAGGAAGAGCAGCTTATG
CTTCCGGGAAATTGCCCCCAGGAAGTATAAACTCTGGCGCAAAGAAGGCCATTTCATTCCAAGATGAAACGAGACCGGCTTATGTCTCAGGAAA
GCTCCCACATGGTAATATTGATGGTATGCGAAACCGCGAAATGGCTGCCGTCCACCGCGAAATTGCTGAGGGTGGGAGGAAACCAGGTCAGGTT
GTCTCTTCTCTATTCACATTCAATCCTACTTCAAAGAAAACGTTCGATGAACCCGAAGAACAAGCGGAGCCGGCAAAACCATCCAATGCGCCTT
TGACAGAGGAAATGGAAACATTCACCAATCTAGGATTATCGAGAAGGCTTGCAGCCCATCTATCGACTAAACTCGATATGAAAGCTCCGACCGC
CATTCAAAAAGCATCTGTGCAGCAGTTGGTATCGGACGATAGCGATGCTTTCATACAAGCAGAGACTGGATCTGGAAAAACCTTGGCATATCTA
CTACCTATAGTCGAGCGGATATTAGCATTGAGTGAGAATGGCGTACAAA

>Bcin02g07770(MLST9), partial sequence [organism=Botrytis calthae, strain MUCL2830]
CAGCTTTCCCTTTCGGTCTTGGCATCTACAGTCATTGCCATCCCTACACCATCACAACTTGAGTCTCGGGCCGTTATCAGCTCCGATGCCGTTG
TAGGATTTGCCGAAGCTGTTCCCAGTGGGACCGTAGGAACAGTTTATGAGGCATATAAACCATTCCTTAAAGTCGTAAATGGATGCGTACCATT
CCCTGCCGTCGATGCATCGGGTAACACAGGGTATGTCCTTATATCTTTCTCTTCCACACGATTTCTATTGAGTCTCTAACATATTTTAGTGGTG
GTTTGTCACCAACTGGCAGTAGCAATGGTGGTTGCAGCAGCAGTACCGGTCAAGTATATGTTCGAGGAGGACAAAGCGGATCAAAGTACGCTAT
CATGTACTCCTGGTAAGTTCTTTCTAAACTTTTCGTTATAGATCTAATCTAACAAAATCTCAGGTACATGCCAAAAGACGAGCCCTCAACCGGT
ATTGGTCACCGTCACGATTGGGAAGGTGTAATTGTCTGGCTCTCCAGCGCCACCGCCACAACTGCCGACAACATCTTAGCCGTTTGTCCTTCCG
CCCACGGAGGCTGGGATTGTTCCACCGATGGTTATTCCCTTTCTGGTACCCGCCCTCTTATCAAGTACGAAAGTATCTGGCCCATCGATCATTC
AATGGGTCTCACTAGTACTGTTGGTGGAAATCAACCTATGATTGCTTGGGAATCTTTAACTACTGCTGCTCAAACTGCTCTTGAGAACACTGAT
TTCGGTGCTGCGAATGTTCCATTCATTCCAGCTGCTTTCACTTCTAAT

>Bcin04g02090(MLST10), partial sequence [organism=Botrytis calthae, strain MUCL2830]
GGAGGATGATATGGCAAAGTCTATGATCACCAAAGCATTTGTGGGCATGAGTAGTAAACTCTCGAATATGACTATGAATGATGTTTACAAGCCC

TATATCCATGTAAGAAATGTAGAATGGAAGATCAATAACTGGAACTAATGTTGTTTGTAGGCTTTCAAATTACTTACACAGTTCAACCCAATCA
CTACAGCTATTGCCGAATCCCCACTATTTCAAATGGCTGTCTCAGCAAATACCATCGAAAAGTACACACTGCTAGGTCCTTTCTTCAGAATATC
TCCTTTGCAACAGGAAGTTACCAGGGAATACTTCAGTGCACCAAAGACGATAGATAGGCGACATATTGCCACATCTCAAGATGCGTTACGATTG
ACCTTACAAACCCATCAAAAAGATTTACTTGATATCATCAACCACTTTGTTCGAGCAAGTCCAATCGCCAAAAGCAAAACCTTGGATTGGTTCG
CCTACATCGTGAATCAAAATCACAAGCGCCGAGCACTTCAGGTAGACCCGAAAGAAGTATCTTCTGATGGTTTCATGCATAATGTCACTGTCGT
TCTGGATGGTCTCTGTGAGCCATTCATGGATACCACATTCTCGAAGATTTCAAAGATTGATATTGATTATTTAAGGCGTGCGCCTCGTGTAGAT
ATCAAGGATGAGACCAAGTTGAACGCTGATGAGAAGGCTTCCGAGAAGTATTATGAGGACACTGTTCCTGGCACTTCTAATTTCATCTCTGAGG
TCTTCTTTCTGACATTGGCTGCTCATCATTATGGTAGTGAAGCTCTTAATGCCACGCATAAGAGTCTGGAGAAAGACATCAAATATATTCAAAA
GCAATTGACTGCCGTTGAAGCA

>Bcin01g07220(MLST1), partial sequence [organism=Botrytis cinerea, strain C12_S_E7_02]
CATAGTATCATAACCTTGTGGAAGCGATGTGATAAATTCATGGATCCCAGCATCTTTTGCTGATGCAATAGCAGATGGAAGACTAGCACAAGGA
GAAGATTCTGGGAGACCATAAATGATGTTATGGAGAATGGTAGCTGGAAATAGAATCGGTATTTGTGGAACGAGTGAGAGAGAAGCCCGGAGAG
ACGGAATGTGACAGTTACTTATGGAGACGCGATTGAACGTCAACGGTGGAGGTGTTGAAGTATCTGGCGGATAGAGACCAATGAGCAGAGCAGC
TATTGTAGATTTTCCGGAGCCGGAGGGTCCGACAAGTGCAGTTGTTGAGTTAGGAATCAGGGAAAGAGAAAAGGATGATATCGTTCGTTTTTCA
GGACGAGTAGGGTATGTGAATGAAAGACGGTTGAATTTGATTGGAAAGATTGTCGAAAGCCGTTCGGTTCCTTTATTTTCGTGGGAGGAAGATG
AATCGAGATTGGCTAATGCAAGCATATGCGTAGCTGTAACGCGAGAAGAGTTGATTTGTGGTATCATGGCCAGCATATTCGTACTATTAGAAAT
ACCAAATAATAGAAGATTGACAGTCTGTAGTGCAGTCGCGATACTGATCTCTCTCTTGGTAATGAGAACCGTGGCATAATAAAAGATAGTTGCA
GTGATGAAGAACGACATCGCATCTGTCAATCCCCACAGCAATCCCGAGTAGTTTGCTCGTATTAGTCCAACTTTATAAAGTTCTTCTGTAGCTT
TGGTGTGTTTTGTCTCAAAGTAAGTTTCCAGAGTAAAAGCCCGAACCACTTTGATGTTGGAGAAAGTCTCTGAAAATATGCCAGTGGTCATTTC
AGAGGCGTAGTTGCACTTGTTTTCCCATTTTCCACTCACGCGATTGAATGTTTTGGTGACAGCGTATATAACTGGCCCAGTTGCCATCGAGACT
AATGTCAGTTTCCATGTATTCACAAAAGCCCAAATGATTGATATCAATAGCATAGAAAATGCTGTGAATACAATACCAGCAAAGCGGCCAACGA
GATTACGCATTTCCTCAGAGTTCCTATCCAAAACTTCGTTCAGCCGGCCAGGTGAATTCCTGGATTCCTCAAACCATGACTTCGGTTGTGCGAG
AATCTTCTTTAGTGCTTCTACACGCAGAGCACTAACCCAAGCTTGTCCAGCACGTTCCAAAGCATAATGCGAAAAGAAACAAGATATGCCATC

>Bcin05g07690(MLST2), partial sequence [organism=Botrytis cinerea, strain C12_S_E7_02]
ACCACTATCACCAAGTCCTTTTCTGCACTTTCCTCATCAAAAACGAACGAGAACATCATCAATAAATACTTTGCTGAAGTAATTGGGTTTTATT
TTGGCCAGGATGCCTTCTCTCTACGACAGCAAGCCTTCGATGATATGTTGTGGGTAGTTCTTGGCTGGCTGGATACTGTCAAATTCATTGATTT
ACATTCTGAATTGCACTATTCAAACGACTCTCAGCCAGAATGGTACGGACAACAATATAAACCTGCATTTGCACATCGAGCGCGACTATTTTGG
GAATTGGCTTCACAAGGATGGGATACTACTCTCTGTGGTGGTGGGATGATATGGTCACCATACCTTACTCCATACAAGAATGCAATTACCAATG
AACTCTATATCGCAGCTTCGATATCGATGTACCTATATTTCCCCGGAGATGACAATCAATCCCCATTTATGCTTTCCAACCCTTCATATCCACC
TCACGATCCGAAATATCTACAGGCAGCTGTTGATGCTTACAAATGGCTGAATGGTTCCAACATGACGGATTTACAAGGATTATATGTCGACGGG
TACCATATCTCGAATCTTTCTGGCGGTGAAAACACCCATTGCGATTCTAGAAATGAGATGGTATATACCTACAATCAAGGTGTTTTGCTTACTG
GACAACGTGGTTTGTATGACGCAACCGCCGCACGATCATACCTTGTAGATGGCCACAAACTCATCGCGAATGTTATTAATGCCACAGGCTATGA
CCTGAAACACAATGTTGTCATCTCACCGCCACCCAAAGATGGTTCCGCATTGGCAAAGTGGTTTGGCCTGGGTAGGAATGGAATACTGGAAGAA
GGATGCGATTCAAGTGCTTCGTGTTCTCAAAATGGACAAACTTTCAAAGGCATATTCTTTCATCACTTGATTGCGTTCTGTAGTGATTTGCCAG
GGGAGCCTATTGCAGGGACGAAGGAAAGCTTAGAACTCGACAGAGTGTGGCATTCTGACAAATGCTCACAGTATACAAAATG

>Bcin06g01710(MLST3), partial sequence [organism=Botrytis cinerea, strain C12_S_E7_02]
GGTGAGTCTGACTTTTTTATTTGAGCATTAAGATAGACACTGATATACCAAGGCAATCACCTAATTTCATGGCGTTCTTCCCTGCATCATCTAC
CTACCCTGGAATGCTGGGAGAATTATACTCAGCAGCTCTCACAGCACCTGCTTTCAATTGGATCTGTTCCCCTGCTGTGACAGAATTGGAGACG
GTTGTAATGGATTGGCTGGCCAAGCTTCTCAATCTCCCAGACTGTTATTTGTCTTCGACTCATGGTGGTGGTGTTATCCAAGGATCAGCCTCGG
AAGCTGTCGTTACCGTTATGGTTGCCGCCCGCGATAAATATCTTCGTGAAACTACTGAAGGTCTGTCGGGCATTGAACTTGAGAATGCGATTGC
ATATAAGAGGAGTAAGCTAGTTGCACTAGGAAGCGAAATGGCACACAGTTCCACGCAGAAAGCAGCGCAGATAGCTGGCGTTAGATTCCGATCG
ATTCCAGTACTCGCATCCAATGATTTTGCCATGACGGGTGATGATTTAGAGAAGGTATTGAAAGAATGCAAATCTCAAGGATTGGAACCCTTCT
ATCTAACTTCGACTTTGGGAACAACATCTACATGCGCAGTTGACGACTTCGCATCTATTACAACAGTACTTTCAAAATATGCACCTCCAGATGT
TGCAGGCGAGATCTGGGTTCACGTCGATGCTGCTTATGCAGGTGCAGCTTTGGTTTGCCCTGAATACCATCATCTAACATCGTCCTTCCAGCAT
TTCCATTCCTTCGATATGAACATGCACAAATGGCTTCTGACAAATTTCGACGCTTCTTGTCTATATGTCAAGAAACGCAAAGATCTGATCGATG
CACTCTCCATAACGCCAAGTTATCTTCGCAACGAGTTTTCAGAGAGTGGACTCGTAACCGACTATCGGGACTGGCAAATTCCTCTCGGAAGACG
CTTCCGAAGCTTAAAGATTTGGTTTGTCCTCAGAACCTAC

>Bcin09g03030(MLST4), partial sequence [organism=Botrytis cinerea, strain C12_S_E7_02]
ACCCTTCAACCCCACCGCAGCTCCTATACCGAAAGCGAGTATCCTACCAATTCTCCTTCCACCGCGACTTTAAGACCATTGGCTTTCCGCACTT
TCACAAAAAAGCATAGTTTAACATTGACGTCGTCGGCATTACAAGTGTTGGCTACTTTTATTGGAAAGTATTGTGGGACAGGATGGAGGGAAGA
AGGACTGGCAGAGAGAGTCTTGGAGGAGGTCGCCAAGAGTTGGAAGAATAGGAGTGGCGGTGTCATTGTCGAGGGCGAGGGAACGGAATTGAAG
GAGATTCTGAAAGCTTTGGAAGGGAATATGAGTGGTGGAAGGATAGTCATAGGAAGAGAGCTAAGCCGGCAGAATAGTTTAGTACTGGGATCAT
CACAATATGGAGAGGTCAATCATACAAGACTTGGGCTACGGCCAGGGAATATACCCAGAGAGGATAGTCAGTCAAGTTTGGGAATGTCAACGTT

```
GGAGGTCAATGACGAAGAAGATGAGGATGGCCTGATGGATCCAAGAAGGTGGTTAAAAGTCATTGATGCATTTGAGCAACCTCGACTGGTGTAC
AATGTTGCTAAAAAGCACTTTGATAGGTATGTTTCAATGATAAAATTTTATCTGAATCGACTGACTCAGTACAGAGATGCCTCCAAACCTTCAT
TGTTCCCACCTGCGTCTCATAAAACACTCCTCTTCCAAAACCGCTATAATGTTATCCATCAACGTCTCCTTCGCAATGAATCTTTTCAAACGCC
CGCTTTTCAAGGTGGCAAATCTTCCCTTCAACGCAGCACGTCCGCCATTACCACACAACAACAATCATACAAATTAACGCCGATAGCTAATCTT
CTCGGTCGCAATCGCAGCTCTCATATGCTTCTCGGTCTCCTCAGTATTTCACCCACTGGTACCCTCGCCATCAATGACCTGACGGGCAGTATCG
CTCTCGATCTTACACACGCAGCAGCCATTC

>Bcin11g01310(MLST5), partial sequence  [organism=Botrytis cinerea, strain C12_S_E7_02]
ACTGACATGGACCTCATGTGGAACCGGCGTAGAATGCGCAACGCTTGAAGTTCCGCTCGAATATGGCGATGCAACGTCAACGGCAAAAGCCAGT
GTTGCGCTTGCTCGTTATCCTGCCACTGTTGCCGCGAGCAAGAAGCTCGGGTCTCTCTTGATAAATCCCGGTGGACCCGGTGCCTCTGGTGTTG
GCTTTGTGCAGTCTGGAGCCGGTGCCGCCGTCTCGACACTGAGTGGTGGATTGTACGATATCATCGGATGGGATCCACGTGGAACCGGTGCTTC
GGCTCCTATTTTGGAATGTTTTGCAAATGCCAGTGCGGAGTATGATTTTAACAACGCGTTTCCATCTGCTCCGAATCTCTGGCTCGGACAATTT
GCGAATGCCAGCGCAAATTCTGCTGTTAGCTCTGCTATCACATCCTTTGACACTTCTGTCGCTGCTCTTGCAAAAGCTTGCGTGGCTCAGAAAT
CTCCCGCTCTTTACACCTCAACAGCAGCATATGTTGCTCGAGACATGGCAGCGATAGTCGATGCATTGGATGGGACCTCTGCAAAACTTAACTA
CTGGGGTTTCTCATATGGAACTATCTTCCTAGCTGAGTTTATCCAAACTTTCCCAGGCCGCGTGGGAAGAGTTCTTGCCGATGGTGTTTTCGAC
GCAAAGGCAAATGCACTCACATACGTTAGCCAACTTCCCAACGATCAACTCAGTGTTCGTGCTTCGTTGAACGATTTTGCAGCTTTCTGCACCA
CCGCCGGTAGTAAAGGTTGCTCTTTTGCCACCGCCCCTACTGGAACCTCAGGTACTGTTGCTACCAGACTGGACAACATAATGAAGGATATGTT
CCTCAATCCTATTGTTGCTTCGGGCTTAAGCATCA

>Bcin15g03910(MLST6), partial sequence [organism=Botrytis cinerea, strain C12_S_E7_02]
GCCAAAACACAAAATCATCCAACGATGAAGATGATACTCCACTTCCCTTGATTATCTGGCATGGACTCGGCGATAATTACAAAGCGGATGGTCT
TGCGCAAGTTGGAAAACTAGCTGAAGCCATTCATCCTGGGACTTTTGTCTACAATATTCATGTAGATGAGGATGCATCTGCAGATAGGACAGCT
ACCTTCTTTGGAAATCTCACTCGTGAGTACATCCCCTATTTTTCCTTTAAATATCATACTAACTCTCTTACCAAGTTCAAATCGAAAAGGTCTG
CGAAGACCTCGCCTCCCATCCTATTCTCTCTACCGCGCCCGCCGTCGACGCAATTGGATTCTCCCAAGGCGGCCAATTCTTGCGTGGTTACATA
TCCCGCTGCAATGCTCCACCCATCCGCTCTCTCCTGACCTTCGGTTCCCAACACAACGGCATTTCTGCCTTCCAAGCCTGTGGTCCTGCCGACC
TCCTCTGTCGCGGTGCTCAAACCCTTTTGCGATCCAACACCTGGTCAACCTTTGTCCAATCTCGTCTCGTACCCGCTCAATACTTCAGAGATCC
GGAAAACCTAGACTCTTACCTTGAATATTCCAATTTCCTTGCCGACATCAATAATGAGCGCGTTCTCAAGAACCAAACATATAAATCCAACATG
GAAAAATTGGAACGATTCGTAATGTATGTCTTTGAAGACGATACAACTGTCATCCCTAAGGAAAGTGGATGGTGGGCTGAAGTCAACGGCACGG
AAGTTACACCACTGAAAGAAAGAGCCATTTATAAAGAAGATTGGCTAGGTTTAAAGACATTGGATGAGGCCGGAAAATTAGTTTTCGAAACCAT
TCCAGGGGGACATATGACGTTAGGAGAGGAGATGCTAGAGAAGGCTTTCAAAGAGTATTTTGGTCCAGCAGGGAAGAAATTTGGG

>Bcin16g03460(MLST7), partial sequence [organism=Botrytis cinerea, strain C12_S_E7_02]
CATGAACCTCTTAATTTGAACTCTTCATTATTTGCAAGTGATGAACCCCTATTCTGCCCAGAGGATAGTTACAAGACGTATATCATTAGTCGAG
AACCACTCATGATATATATTGACGGATTTTTGAAAGCGAATGAAAGTAAACATTTGGTTGATGTTAGGTGTGTTATTTATTCTGATGAAATGAA
CAAGAGAGACTGATGAGATAGTGAACCGCTTTATGAACCGTCTACTGTTTCTCACGGGCAGGAAGTTACCATTGATACTTCAGTTCGAAATTCT
GAAGTGGCGGTTTTAGAGAGGGATGAGGTGGTCAGGTGTATTGAGCATAGAGCGAGGGCATTTCAGGGTGGAGGGGCGAGATGGGGATTGAGAA
GTTGAGGACGCAGAGGTATGGGGTTGGAGGACATTATGGGATGCATTTGTAAGTTTTGGGGGATTGACGAAGGCTTTTGTCTATTTTCTACCAG
TACGGATCTTGAAAGAAAGATAGCATGAACACGAGGGCTAATAACTAGGAAATAGCGACTGGAGCGGAGGTAAACGTGGCATAGACCGATTTAG
TACTTTCATGGTCTATGTCGACGTATCCTCTGATATCGAAGGTGGAGGAACGGAATTCCCACGTATCGTGGGACCAAAAGGAGGAAGGTGGGAG
GACTTCCTGGAAACTACGGAAGCATTGGATCCAAGAACTGGAGAAAATGTAACAGTAGAAGGGGTGACATTCAAACCAATCAAGGGAAATGCCG
TATTCTGGGAAAATACTGACAACAACGGGAGGGCTATGAT

>Bcin12g03020(MLST8), partial sequence [organism=Botrytis cinerea, strain C12_S_E7_02]
CGATTGGCTGCGAAGAAAACTGCGCAGCACCGAGTCACCAAAAGCACGAATAAACCATCGGCAAGGGAAATATTCTCTGAACCACAACATGACA
CGAGCGCGGAAGAGTATATCGGGCGAGAAGCTTCATCAAGAGCACCAAAGCGACAACGAGTCGATGATAATTATAACTCTTACGGTGGAAGAAA
TGAAAATACAGCAGCTTATGCTTCCGGGAAACTTCCATCTGGAAGTATAAATGTTGGTGGAGGTAGGAAGACCACTTTTCAAGAAGAACCACGA
ACGGCATTTGTCGCTGGCAAGTTGCCCCCTGGAAGTATCAACATTGGTGGAAAGAAGGCTACCCAAATCGAGGACGAGGGTAGAGCAGCTTATG
CTTCCGGAAAATTGCCTCCAGGAAGTATAAACTCTGGCGCAAAGAAGGCGATTTCATTCCAAGATGAAACGAGACCGGCTTATGTCTCCGGAAA
GCTTCCACATGGTAGTATCGACGGTATGCGAAACCGTGAAATGGCTGCCGTCCACCGCGAAATTGCTGAGGGTGGGAGGAAACCAGGCCAGGTT
GTCTCTTCTCTATTCACATTCAATCCTACTTCAAAGAAAACTTTTGATGAACCCGAAGAACAAGCGGAACCGGCAAAACCATCCAATGCGCCTT
TGACAGAGGAAATGGCGACTTTCACCAATCTAGGGTTATCGAGAAGGCTTGCAGCCCATCTATCGACTAAACTCGATATGAAAGCTCCGACCGC
CATTCAAAAAGCATCTGTACAGCAGTTGGTATCGGACGATAGCGATGCTTTCATACAAGCAGAGACTGGATCTGGAAAAACTTTGGCATATCTA
CTACCTATAGTCGAGCGAATATTAGCATTGAGTGAGAATGGCGTACAAA

>Bcin02g07770(MLST9), partial sequence [organism=Botrytis cinerea, strain C12_S_E7_02]
CAGCTTTCCCTTTCGGTCTTGGCATCTACAGTCATTGCCATCCCTACACCATCACAACTTGAGTCTCGGGCCGTTATCGATTCCGATGCCGTTG
TAGGATTTGCCGAAACTGTTCCCAGTGGGACCGTAGGAACAGTTTATGAGGCATATAAACCATTCCTTAAAGTCGTAAATGGATGCGTACCATT
```

CCCTGCCGTCGATGCATCGGGTAACACAGGGTATGTCCTTATATCTTTCTCTTCCACACGATTGCTATTGAGTCTCTAACATATTTTAGTGGTG
GTTTGTCACCAACTGGCAGTAGCAATGGTGGTTGCAGCAGCAGTACCGGTCAAGTATATGTTCGAGGAGGACAAAGCGGATCAAACTACGCCAT
CATGTACTCCTGGTAAGTTCTCTCTAAACTTCTCCTTATAGATCCAACCTAACAAAATCTTAGGTACATGCCAAAGGACGAGCCCTCAACCGGT
ATTGGTCACCGTCACGATTGGGAAGGTGTAATTGTCTGGCTCTCCAGCGCCACCGCCACAACTGCCGACAACATCTTAGCCGTTTGTCCTTCCG
CCCACGGAGGCTGGGATTGTTCCACGGATGGCTATTCCCTTTCTGGTACCAGCCCTCTTATCAAGTACGAAAGTATCTGGCCCGTCGATCACTC
AATGGGTCTTACTAGTACTGTTGGTGGAAAACAACCTATGATTGCTTGGGAGTCTTTACCAACTGCTGCTCAAACTGCTCTTGAGAACACCGAT
TTCGGTGCTGCGAATGTTCCATTCATTCCGGCTGTTTTCACAGACAATCT

>Bcin04g02090(MLST10), partial sequence [organism=Botrytis cinerea, strain C12_S_E7_02]
CGGAGGATGATATGGCAAAGTCTATGATTACCAAAGCATTTGTAGGCATGAGTAGTAAACTGTCGAATATGACTATGAATGATGTTTACAAGCC
CTACATCCATGTAAGAAATGTAGAATAGAGAGATCAGTAACTGGAACTAATATCGTTTGTAGGCTTTCAAGTTACTTACGCAGTTCAACCCAAT
CACTACAGCTATTGCCGAATCCCCACTATTTCAAATGGCTGTCTCAGCAAATACCATCGAAAAGTACACACTGCTAGGCCCTTTCTTCAGAATA
TCTCCTCTGCAACAGGAAGTTACCAGGGAATACTTCAGTGCGCCAAAGACGATAGATAGGCGACACATTGCCACATCTCAAGATGCCTTACGAT
TGACCTTACAAACCCATCAAAAAGATTTACTTGATATCATCAACCACTTTGTTCGAGCAAGTCCAATCGCCAAAAGCAAAACCTTGGATTGGTT
CGCCTACATTGTGAATCAAAATCACAAGCGTCGAGCACTTCAGGTAGACCCGAAAGAAGTGTCTTCTGATGGCTTTATGCACAATGTCACTGTC
GTTCTAGATGGTCTTTGTGAGCCATTCATGGATACCACATTCTCGAAAATTTCGAAGATTGATATTGATTATCTAAGACGTGCGCCTCGTGTAG
ATATCAAGGACGAGACCAAGTTGAACGCTGACGAGAAGGCTTCTGAGAAGTATTATGAGGACACTGTTCCTGGCACTTCTAATTTCATCTCTGA
GGTCTTCTTTCTCACATTGGCTGCTCATCATTATGGTAGTGAAGCTCTTAATGCCACGCATAAGAGTCTGGAGAAAGACATCAAATATATTCAA
AAGCAATTGACTGCCGTTGAAGCA

>Bcin01g07220(MLST1), partial sequence [organism=Botrytis cinerea, strain C12_S_E7_4]
TGATGGCATATCTTGTTTCTTTTCGCATTATGCTTTGGAACGTGCTGGACAAGCTTGGGTTAGTGCTCTGCGTGTAGAAGCACTAAAGAAGATT
CTCGCACAACCGAAGTCATGGTTTGAGGAATCCAGGAATTCACCTGGCCGGTTGAACGAAGTTTTGGATAGGAACTCTGAGGAAATGCGTAATC
TCGTTGGCCGCTTTGCTGGTATTGTATTCACAGCATTTTTTATGCTATTGATATCAATCATTTGGGCTTTTGTGAATACATGGAAACTGACATT
AGTCTCGATGGCAACTGGGCCAGTTATATACGCTGTCACCAAAACATCCAATCGCGTGAGTGGAAAATGGGAAAACAAGTGCAACTACGCCTCT
GAAATGACCACTGGCATATTTTCAGAGACTTTCTCCAACATCAAAGTGGTTCGGGCTTTTACTCTGGAAACTTACTTTGAGACAAAACACACCA
AAGCTACAGAAGAACTTTATAAAGTTGGACTAATACGAGCAAACTACTCGGGATTGCTGTGGGGATTGACAGATGCGATGTCATTCTTCATCAC
TGCAACTATCTTTTATTATGCCACGGTTCTCATTACCAAGAGAGAGATCAGTATCGCGACTGCACTACAGACTGTCAATCTTCTATTATTTGGT
ATTTCTAATAGTACGAATATGCTGGCCATGATACCACAAATCAACTCTTCTCGCGTTACAGCTACGCATATGCTTGCATTAGCCAATCTCGATT
CATCTTCCTCCCACGAAAATAAAGGAACCGAACGGCTTTCGACAATCTTTCCAATCAAATTCAACCGTCTTTCATTCACATACCCTACTCGTCC
TGAAAAACGAACGATATCATCCTTTTCTCTTTCCCTGATTCCTAACTCAACAACTGCACTTGTCGGACCCTCCGGCTCCGGAAAATCTACAATA
GTTGCTCTGCTCATTGGTCTCTATCCGCCAGATACTTCAACACCTCCACCGTTGACATTCAATCGCGTCTCCATAAGTAACTGTCACATTCCGT
CTCTCCGGGCTTCTCTCTCACTCGTTCCACAAATACCGATTCTATTTCCAGCTACCATTCTCCATAACATCATTTATGGTCTCCCAGAATCTTC
TCCTTGTGCTAGTCTTCCATCAGCTATTGCATCAGCAAAAGATGCTGGGATCCATGAATTTATCACATCGCTTCCACAAGGTTATGATACTATG

>Bcin05g07690(MLST2), partial sequence [organism=Botrytis cinerea, strain C12_S_E7_4]
CACCACTATCACCAAGTCCTTTTCTGCACTTTCCTCATCAAAAACGAACGAGAACATCATCAATAAATACTTTGCTGAAGTAATTGGGTTTTAT
TTTGGCCAGGATGCCTTCTCTCTACGACAGCAAGCCTTCGATGATATGTTGTGGGTAGTTCTTGGCTGGCTGGATACTGTCAAATTCATTGATT
TACATTCTGAATTGCACTATTCAAACGACTCTCAGCCAGAATGGTACGGACAACAATATAAACCTGCATTTGCACATCGAGCGCGACTATTTTG
GGAATTGGCTTCACAAGGATGGGATACTACTCTCTGTGGTGGTGGGATGATATGGTCACCATACCTTACTCCATACAAGAACGCAATTACCAAT
GAACTCTATATCGCAGCTTCGATATCGATGTACCTATATTTCCCCGGAGATGACAATCAATCCCCATTTATGCTTTCCAACCCTTCATATCCAC
CTCACGATCCGAAATATCTACAGGCAGCTGTTGATGCTTACAAATGGCTGAATGGTTCCAACATGACGGATTTACAAGGATTATATGTCGACGG
GTACCATATCTCGAATCTTTCTGGCGGTGAAAACACCCATTGCGATTCTAGAAATGAGATGGTATATACCTACAATCAAGGTGTTTTGCTTACT
GGACAACGTGGTTTGTATGACGCAACCGCCGCACGATCATACCTTGTAGATGGCCACAAACTCATCGCGAATGTTATTAATGCCACAGGCTATG
ACCTGAAACACAATGTTGTCATCTCACCGCCACCCAAAGATGGTTCCGCATTGGCAAAGTGGTTTGGCCTGGGTAGGAATGGAATACTGGAAGA
AGGATGCGATTCAAGTGCTTCGTGTTCTCAAAATGGACAAACTTTCAAAGGCATATTCTTTCATCACTTGATTGCGTTCTGTAGTGATTTGCCA
GGGGAGCCTATTGCAGGGACGAAGGAAAGCTTAGAACTCGACAGAGTGTGGCATTCTGACAAATGCTCACAGTATACAAAAT

>Bcin06g01710(MLST3), partial sequence [organism=Botrytis cinerea, strain C12_S_E7_4]
GTGAGTCTGACTTTTGTATTTGAGCGTTAAGATAGACACTGATATACCAAGGCAATCACCTAATTTCATGGCGTTCTTCCCTGCATCATCTACC
TACCCTGGAATGCTGGGAGAATTATACTCAGCAGCTTTCACAGCACCTGCTTTCAATTGGATCTGTTCCCCTGCTGTGACAGAATTGGAGACGG
TTGTAATGGATTGGCTGGCCAAGCTTCTCAATCTCCCAGACTGTTATTTGTCTTCGACTCATGGTGGTGGTGTCATCCAAGGATCAGCCTCGGA
AGCTATCGTTACCGTTATGGTTGCTGCCCGCGATAAATATCTTCGTGAAACTACTGAAGGTCTGTCGGGCATTGAACTCGAGGATGCGATTGCA
TATAAGAGGAGTAAGCTAGTTGCACTAGGAAGCGAAATGGCACACAGCTCCACGCAGAAAGCAGCGCAAATAGCTGGCGTTAGATTCCGATCGA
TTCCAGTACTCGCATCCAATGATTTCGCCATGACGGGTGATGATTTAGAGAAGGTATTGAAAGAATGCAAATCTCAAGGATTGGAACCCTTCTA
TCTAACTTCGACTTTGGGAACAACATCTACATGCGCAGTTGACGACTTCGCATCTATTGCAACAGTACTTTCAAAATATGCACCTCCAGATGTT
GCAGGCGAGATCTGGGTTCACGTCGATGCTGCTTATGCAGGTGCAGCTTTGGTTTGCCCTGAATACCATCATCTAACATCGTCTTTCCAGCATT

TCCATTCCTTCGATATGAACATGCACAAATGGCTTCTGACAAATTTCGACGCTTCTTGTCTATATGTCAAGAAACGCAAAGATCTGATCGATGC
ACTGTCCATAACACCAAGTTATCTTCGCAACGAGTTTTCAGAGAGTGGACTCGTAACCGACTATCGGGACTGGCAAATTCCTCTCGGAAGACGC
TTCCGAAGCTTAAAGATTTGGTTTGTCCTCAGAACCTACG

>Bcin09g03030(MLST4), partial sequence [organism=Botrytis cinerea, strain C12_S_E7_4]
ACCCTTCAACCCCACCGCAGCTCCTATACCGAAAGCGAGTATCCTACCAATTCTCCTTCCACCTGCGACTTTAAGACCATTGGCTTTCCGCACT
TTCACAAAAAAGCATAGTTTAACATTGACGTCGTCGGCATTACAAGTGTTGGCTACTTTTATTGGAAAGCATTGTGGGACAGGATGGAGGGAAG
AAGGACTGGCAGAGAGAGTCTTGGAGGAGGTCGCCAAGAGTTGGAAGAATAGGAGTGGCGGTGTCATTGTCGAGGGCGAGGGAACGGAATTGAA
GGAGATTCTGAAAGCTTTGGAAGGGAATATGAGTGGTGGAAGGATAGTCATAGGAAGAGAGCTAAGCCGGCAGAATAGTTTAGTACTGGGATCA
TCACAATATGGAGAGGTCAATCATACAAGACTTGGGCTACGGCCAGGGAATATACCCAGAGAGGATAGTCAGTCAAGTTTGGGAATGTCAACGT
TGGAGGTCAATGACGAGGAAGATGAGGATGGCCTGATGGATCCAAGAAGGTGGTTAAAAGTCATTGATGCATTTGAGCAACCTCGACTGGTGTA
CAATGTTGCTAAAAAGCACTTTGATAGGTATGTTTCAATGATAAAATTTCATCTGAATCGACTAACTCAGTACAGAGATACCTCCAAACCTTCA
TTGTTCCCACCTGCGTCTCATAAAACACTCCTCTTCCAAAACCGCTATAATGTTATCCATCAACGTCTCCTTCGCAATGAATCTTTTCAAACGC
CCGCTTTTCAAGGTGGCAAATCTTCCCTTCAACGCAGCACGTCCGCCATTACCACCCAACAACAATCATACAAATTAACGCCGATAGCTAATCT
TCTCGGTCGCAATCGCAGCTCTCATATGCTTCTCGGTCTCCTCAGTATTTCACCCACTGGTACCCTCGCCATCAATGACCTGACGGGCAGTATC
GCTCTCGATCTTACACACGCAGCAGCCATTC

>Bcin11g01310(MLST5), partial sequence [organism=Botrytis cinerea, strain C12_S_E7_4]
ACTGACATGGACCTCATGTGGAACCGGCGTAGAATGCGCAACGCTTGAAGTTCCGCTCGAATATGGCGATGCAACGTCAACGGCAAAAGCCAGT
GTTGCGCTTGCTCGTTATCCTGCCACTGTTGCCGCGAGCAAGAAGCTCGGGTCTCTCTTGATAAACCCCGGTGGACCCGGTGCCTCTGGTGTTG
GCTTTGTGCAGTCTGGAGCCGGTGCCGCCGTCTCGACACTGAGTGGTGGATTGTACGATATCATCGGATGGATCCACGTGGAACCGGTGCTTCG
GCTCCTATTTTGGAATGTTTTGCAAATGCCAGTGCGGAGTATGATTTTAACAACGCGTTTCCATCTGCTCCGAATCTCTGGCTCGGACAATTTG
CGAATGCCAGCGCAAATTCTGCTGTTAGCTCTGCTATCACATCCTTTGACACTTCTGTCGCTGCTCTTGCAAAAGCTTGCGTGGCTCAGAAATC
TCCCGCTCTTTACACCTCAACAGCAGCATATGTTGCTCGAGACATGGCAGCGATAGTCGATGCATTGGATGGGACCTCTGCAAAACTTAACTAC
TGGGGTTTCTCATATGGAACTATTTTCCTAGCTGAGTTTATCCAAACTTTCCCAGGCCGTGTGGGAAGAGTTCTTGCCGATGGTGTTTTCGACG
CAAAGGCAAATGCACTCACATACGTTAGCCAACTTCCCAACGATCAACTCAGTGTTCGTGCTTCGTTGACGATTTTGCAGCTTTCTGCACCACC
GCCGGTAGTAAAGGTTGCTCTTTTGCCACCGCCCCTACTGGAACCTCAGGTACTGTTGCTACCAGACTGGACAACATAATGAAGGATATGTTCC
TCAATCCTATTGTTGCTTCGGGCTTAAGCATCA

>Bcin15g03910(MLST6), partial sequence [organism=Botrytis cinerea, strain C12_S_E7_4]
GCCAAAACACAAAATCATCCAACGATGAAGATGATACTCCACTTCCCTTGATTATCTGGCATGGACTCGGCGATAATTACAAAGCGGATGGTCT
TGCGCAAGTTGGAAAACTAGCTGAAGCTATTCATCCTGGGACTTTTGTCTACAATATTCATGTAGATGAGGATGCATCTGCAGATAGGACAGCT
ACCTTCTTTGGAAATCTCACTCGTGAGTACATCCCCTATTTTTCCTTTAAATATCATACTAACTCTCTTACCAAGTTCAAATCGAAAAGGTCTG
CGAAGACCTCGCCTCCCATCCTATTCTCTCTACCGCGCCCGCCGTCGACGCAATTGGATTCTCCCAAGGCGGCCAATTCTTGCGTGGTTACATA
TCCCGCTGCAATGCTCCACCCATCCGCTCTCTCCTGACCTTCGGTTCCCAACACAACGGCATTTCTGCCTTCCAAGCCTGTGGTCCTGCCGATT
TCCTCTGTCGCGGTGCTCAAACCCTTTTGCGATCCAACACCTGGTCAACCTTTGTCCAATCTCGTCTCGTACCCGCTCAATACTTCAGAGATCC
GGAAAACCTAGACTCTTACCTTGAATATTCCAATTTCCTTGCCGACATCAATAATGAGCGCGTTCTCAAGAACCAAACATATAAATCCAACATG
GAAAAATTGGAACGATTCGTAATGTATGTCTTTGAAGACGATACAACTGTCATCCCTAAGGAAAGTGGATGGTGGGCTGAAGTCAACGGCACGG
AAGTTACACCACTGAAAGAAAGAGCCATTTATAAAGAAGATTGGCTAGGTTTAAAGACATTGGATGAGGCCGGAAAATTAGTTTTCGAAACCAT
TCCAGGGGGACATATGACGTTAGGAGAGGAGATGCTAGAGAAGGCTTTCAAAGAGTATTTTGGTCCAGCAGGGAAGAAATTTGGG

>Bcin16g03460(MLST7), partial sequence [organism=Botrytis cinerea, strain C12_S_E7_4]
ATGAACCTCTTGATTTGAACTCTTCATTAATTGCAAGTGATGAACCCCTATTCTGCCCAGAGGATAGTTACAAGACGTATATCATTAGTCGAGA
ACCACTCATGATATATATTGACGGATTTTTGAAAGCGAATGAAAGTAAACATTTGGTTGATGTTAGGTGTGTTATTTATTCTGATGAAATGAAC
AAGAGAGACTGATGAGATAGTGAGCCGCTTTATGAACCGTCAACTGTTTCTCACGGACAGGAAGTTACCATTGATCCTTCAGTTCGGAATTCTG
AAGTGGCGGTTTTAGAGAGGGATGAGGTGGTCAGGTGTATTGAGCATAGAGCGAGGGCATTTCAGGGGTGGAGGGGCGAGATGGGGATTGAGAA
GTTGAGGACGCAGAGGTATGGGGTTGGAGGACATTATGGGATGCATTTGTAAGTTTTGGGGGATTGACGAAGGCTTCTGTCTATTTTCTATCAG
CACGGATCTTGAAAGAAAGATAGCATGAACACGCGGGCTAATAACTAGGAAATAGCGATTGGAGCGGAGGTAAACGTGGCATAGACCGATTTAG
TACTTTCATGGTCTATGTCGACGTATCCTCTGATATCGAAGGTGGAGGAACGGAATTCCCACGTATCGTGGGACCAAAAGGAGGAAGGTGGGAG
GACTTCCTGGAAACTACGGAAGCATTGGATCCAAGAACTGGAGAAAATGTAACAGTAGAAGGGGTGACATTCAAACCAATCAAGGGAAATGCCG
TATTCTGGGAAAATACTGACAACAACGGGAGGGGCTATGAT

>Bcin12g03020(MLST8), partial sequence [organism=Botrytis cinerea, strain C12_S_E7_4]
TCGATTGGCTGCGAAGAAAACTGCGCAGCACCGAGTCACCAAAAGCACGAATAAACCATCGGCAAGGGAAATATTCTCTGAACCACAACATGAC
ACGAGCGCGGAAGAGTATATCGGGCGAGAAGCTTCATCAAGAGCACCAAAGCGACAACGAGTCGATGATAATTATAACTCTTACGGTGGAAGAA
ATGAAAATACAGCAGCTTATGCTTCCGGGAAACTTCCATCTGGAAGTATAAATGTTGGTGGAGGTAGGAAGACCACTTTTCAAGAAGAACCACG
AACGGCATTTGTCGCTGGCAAGTTGCCCCCTGGAAGTATCAACATTGGTGGAAAGAAGGCTACCCAAATCGAGGACGAGGGTAGAGCAGCTTAT

GCTTCCGGAAAATTGCCTCCAGGAAGTATAAACTCTGGCGCAAAGAAGGCGATTTCATTCCAAGATGAAACGAGACCGGCTTATGTCTCCGGAA
AGCTTCCACATGGTAGTATCGACGGTATGCGAAACCGTGAAATGGCTGCCGTCCACCGCGAAATTGCTGAGGGTGGAGGAAACCAGGCCAGGT
TGTCTCTTCTCTATTCACATTCAATCCTACTTCAAAGAAAACTTTTGATGAACCCGAAGAACAAGCGGAACCGGCAAAACCATCCAATGCGCCT
TTGACAGAGGAAATGGCGACTTTCACCAATCTAGGGTTATCGAGAAGGCTTGCAGCCCATCTATCGACTAAACTCGATATGAAAGCTCCGACCG
CCATTCAAAAAGCATCTGTACAGCAGTTGGTATCGGACGATAGCGATGCTTTCATACAAGCAGAGACTGGATCTGGAAAAACTTTGGCATATCT
ACTACCTATAGTCGAGCGAATATTAGCATTGAGTGAGAATGGCGTACAAA

>Bcin02g07770(MLST9), partial sequence [organism=Botrytis cinerea, strain C12_S_E7_4]
CAGCTTTCCCTTTCGGTCTTGGCATCTACAGTCATTGCCATCCCTACACCATCACAACTTGAGTCTCGGGCCGTTATCGATTCCGATGCCGTTG
TAGGATTTGCCGAAACTGTTCCCAGTGGGACCGTAGGAACAGTTTATGAGGCATATAAACCATTCCTTAAAGTCGTAAATGGATGCGTACCATT
CCCTGCCGTCGATGCATCGGGTAACACAGGGTATGTCCTTATATCTTTCTCTTCCACACGATTGCTATTGAGTCTCTAACATATTTTAGTGGTG
GTTTGTCACCAACTGGCAGTAGCAATGGTGGTTGCAGCAGCAGTACCGGTCAAGTATATGTTCGAGGAGGACAAAGCGGATCAAACTACGCCAT
CATGTACTCCTGGTAAGTTCTCTCTAAACTTCTCCTTATAGATCCAACCTAACAAAATCTTAGGTACATGCCAAAGGACGAGCCCTCAACCGGT
ATTGGTCACCGTCACGATTGGGAAGGTGTAATTGTCTGGCTCTCCAGCGCCACCGCCACAACTGCCGACAACATCTTAGCCGTTTGTCCTTCCG
CCCACGGAGGCTGGGATTGTTCCACGGATGGCTATTCCCTTTCTGGTACCAGCCCTCTTATCAAGTACGAAAGTATCTGGCCCGTCGATCACTC
AATGGGTCTTACTAGTACTGTTGGTGGAAAACAACCTATGATTGCTTGGGAGTCTTTACCAACTGCTGCTCAAACTGCTCTTGAGAACACCGAT
TTCGGTGCTGCGAATGTTCCATTCATTCCGGCTGTTTTCACAGACAATCT

>Bcin04g02090(MLST10), partial sequence [organism=Botrytis cinerea, strain C12_S_E7_4]
GCGGAGGATGATATGGCAAAGTCTATGATTACCAAAGCATTTGTAGGCATGAGTAGTAAACTGTCGAATATGACTATGAATGATGTTTACAAGC
CCTACATCCATGTAAGAAATGTAGAATAGAGAGATCAGTAACTGGAACTAATATCGTTTGTAGGCTTTCAAGTTACTTACGCAGTTCAACCCAA
TCACTACAGCTATTGCCGAATCCCCACTGTTTCAAATGGCCGTCTCAGCAAATACCATCGAAAAGTACACACTGCTAGGCCCTTTCTTCAGAAT
ATCTCCTCTGCAACAGGAAGTTACCAGGGAATACTTCAGTGCGCCAAAGACGATAGATAGGCGACACATTGCCACATCTCAAGATGCGTTACGA
TTGACCTTACAAACCCATCAAAAAGATTTACTTGATATCATCAACCACTTTGTTCGAGCAAGTCCAATCGCAAAAAGCAAAACCCTGGATTGGT
TCGCCTACATTGTGAATCAAAATCACAAGCGTCGAGCACTTCAGGTAGACCCGAAAGAAGTGTCTTCTGATGGCTTTATGCACAATGTCACTGT
CGTTCTAGATGGTCTTTGTGAGCCATTCATGGATACCACATTCTCGAAAATTTCGAAGATTGATATTGATTATCTAAGACGTGCGCCTCGTGTA
GATATCAAGGACGAGACCAAGTTGAACGCTGACGAGAAGGCTTCTGAGAAGTATTATGAGGACACTGTTCCTGGCACTTCTAATTTCATCTCTG
AGGTCTTCTTTCTCACATTGGCTGCTCATCATTATGGTAGTGAAGCTCTTAATGCCACGCATAAGAGTCTGGAGAAAGACATCAAATATATTCA
AAAGCAATTGACTGCCGTTGAAGCAG

>Bcin01g07220(MLST1), partial sequence [organism=Botrytis cinerea, strain CH14_ES_11_1]
ATGGCATATCTTGTTTCTTTTCGCATTATGCTTTGGAACGTGCTGGACAAGCTTGGGTTAGTGCTCTGCGTGTAGAAGCACTAAAGAAGATTCT
CGCACAACCGAAGCCATGGTTTGAGGAATCCAGGAATTCACCTAGCCGGTTGAACGAAGTTTTGGATAGGAACTCTGAGGAAATGCGTAATCTC
GTTGGCCGCTTTGCTGGTATTGTATTCACAGCATTTTTTATGCTATTGATATCAATCATTCGGGCTTTCGTGAATACATGGAAACTGACATTAG
TCTCAATGGCAACTGGGCCAGTTATATACGCTGTCACCAAAACGTTCAATCGCGTGAGTGGAAAATGGGAAAACAAGTGCAACTACGCATCTGA
AATGACCACTGGCATATTTTCAGAGACTTTCTCCAACATCAAAGTGGTTCGGGCTTTTACTCTGGAAACTTACTTTAAGACAAAACACACCAAA
GCTACAGAAGAACTCTATAAAGTTGGACTAATACGAGCAAACTACTCGGGATTGCTGTGGGGATTGACAGATGCAATGTCATTCTTCATCACTG
CAACTATCTTTTATTATGCCACGGTTCTCATTACCAAGAGAGAGATCAGTATCGCGGCTGCACTACAGACTGTCAATCTTCTATTATTTGGTAT
TTCTAATAGTACGAATATGCTGGCCATGATACCACAAATCAACTCTCCTCGCGTTACAGCTACGCATATGCTTGCATTAGCCAATCTCGATTCA
TCTTCCTCCCACGAAAATAAAGGAACCGAACGGCTTTCGACAATCTTTCCAATCAAATTCAACCGTCTTTCATTCACATACCCTACTCGTCCTG
AAAAACGAACGATATCATCCTTTTCTCTTTCCCTGATTCCTAACTCAACAACTGCACTTGTCGGACCCTCCGGCTCCGGAAAATCTACAATAGC
TGCTCTGCTCATTGGTCTCTATCCGCCAGATACTTCAACACCTCCACCGTTGACATTCAATCGCGTCTCCATAAGTAACTGTCACATTCCGTCT
CTCCGGGCTTCTCTCTCACTCGTTCCACAAATACCGATTCTATTTCCAGCTACCATTCTCCATAACATCATTTATGGTCTCCCAGAATCTTCTC
CTTGTGCTAGTCTTCCATCTGCTATTGCATCAGCAAAAGATGCTGGGATCCATGAATTTATCACATCGCTTCCACAAGGTTATGATACTAT

>Bcin05g07690(MLST2), partial sequence [organism=Botrytis cinerea, strain CH14_ES_11_1]
ACCACTATCACCAAGTCCTTTTCTGCACTTTCCTCATCAAAAACGAACGAGAACATCATCAATAAATACTTTGCTGAAGTAATTGGGTTTTATT
TTGGCCAGGATGCCTTCTCTCTACGACAGCAAGCCTTCGATGATATGTTGTGGGTAGTTCTTGGCTGGCTGGATACTGTCAAATTCATTGATTT
ACATTCTGAATTGCACTATTCAAACGACTCTCAGCCAGAATGGTACGGACAACAATATAAACCTGCATTTGCACATCGAGCGCGACTATTTTGG
GAATTGGCTTCACAAGGATGGGATACTACTCTCTGTGGTGGTGGGATGATATGGTCACCATACCTTACTCCATACAAGAACGCAATTACCAATG
AACTCTATATCGCAGCTTCGATATCGATGTACCTATATTTCCCCGGAGATGACAATCAATCCCCATTTATGCTTTCCAACCCTTCATATCCACC
TCACGATCCGAAATATCTACAGGCAGCTGTTGATGCTTACAAATGGCTGAATGGTTCCAACATGACGGATTTACAAGGATTATATGTCGACGGG
TACCATATCTCGAATCTTTCTGGCGGTGAAAACACCCATTGCGATTCTAGAAATGAGATGGTATATACCTACAATCAAGGTGTTTTGCTTACTG
GACAACGTGGTTTGTATGACGCAACCGCCGCACGATCATACCTTGTAGATGGCCACAAACTCATCGCGAATGTTATTAATGCCACAGGCTATGA
CCTGAAACACAATGTTGTCATCTCACCGCCACCCAAAGATGGTTCCGCATTGGCAAAGTGGTTTGGCCTGGGTAGGAATGGAATACTGGAAGAA
GGATGCGATTCAAGTGCTTCGTGTTCTCAAAATGGACAAACTTTCAAAGGCATATTCTTTCATCACTTGATTGCGTTCTGTAGTGATTTGCCAG
GGGAGCCTATTGCAGGGACGAAGGAAAGCTTAGAACTCGACAGAGTGTGGCATTCTGACAAATGCTCACAGTATACAAAAT

>Bcin06g01710(MLST3), partial sequence [organism=Botrytis cinerea, strain CH14_ES_11_1]
CATTGGTGAGTCTGACTTTTGTATTTGAGCGTTAAGATAGACACTGATATACCAAGGCAATCACCTAATTTCATGGCGTTCTTCCCTGCATCAT
CTACCTACCCTGGAATGCTGGGAGAATTATACTCAGCAGCTTTCACAGCACCTGCTTTCAATTGGATCTGTTCCCCTGCTGTGACAGAATTGGA
GACGGTTGTAATGGATTGGCTGGCCAAGCTTCTCAATCTCCCAGACTGTTATTTGTCTTCGACTCATGGTGGTGGTGTCATCCAAGGATCAGCC
TCGGAAGCTATCGTTACCGTTATGGTTGCTGCCCGCGATAAATATCTTCGTGAAACTACTGAAGGTCTGTCGGGCATTGAACTCGAGGATGCGA
TTGCATATAAGAGGAGTAAGCTAGTTGCACTAGGAAGCGAAATGGCACACAGCTCCACGCAGAAAGCAGCGCAAATAGCTGGCGTTAGATTCCG
ATCGATTCCAGTACTCGCATCCAATGATTTCGCCATGACGGGTGATGATTTAGAGAAGGTATTGAAAGAATGCAAATCTCAAGGATTGGAACCC
TTCTATCTAACTTCGACTTTGGGAACAACATCTACATGCGCAGTTGACGACTTCGCATCTATTGCAACAGTACTTTCAAAATATGCACCTCCAG
ATGTTGCAGGCGAGATCTGGGTTCACGTCGATGCTGCTTATGCAGGTGCAGCTTTGGTTTGCCCTGAATACCATCATCTAACATCGTCCTTCCA
GCATTTCCATTCCTTCGATATGAACATGCACAAATGGCTTCTGACAAATTTCGACGCTTCTTGTCTATATGTCAAGAAACGCAAAGATCTGATC
GATGCACTCTCCATAACACCAAGTTATCTTCGCAACGAGTTTTCAGAGAGTGGACTCGTAACCGACTATCGGGACTGGCAAATTCCTCTCGGAA
GACGCTTCCGAAGCTTAAAGATTTGGTTTGTCCTCAGAACCTAC

>Bcin09g03030(MLST4), partial sequence [organism=Botrytis cinerea, strain CH14_ES_11_1]
ACGACCCTTCAACCCCACCGCAGCTCCTATACCGAAAGCGAGTATCCTACCAATTCTCCTTCCACCTGCGACTTTAAGACCATTGGCTTTCCGC
ACTTTCACAAAAAAGCATAGTTTAACATTGACGTCGTCGGCATTACAAGTGTTGGCTACTTTTATTGGAAAGCATTGTGGGACAGGATGGAGGG
AAGAAGGACTGGCAGAGAGAGTCTTGGAGGAGGTCGCCAAGAGTTGGAAGAATAGGAGTGGCGGTGTCATTGTCGAGGGCGAGGGAACGGAATT
GAAGGAGATTCTGAAAGCTTTGGAAGGGAATATGAGTGGTGGAAGGATAGTCATAGGAAGAGAGCTAAGCCGGCAGAATAGTTTAGTACTGGGA
TCATCACAATATGGAGAGGTCAATCATACAAGACTTGGGCTACGGCCAGGGAATATACCCAGAGAGGATAGTCAGTCAAGTTTGGGAATGTCAA
CGTTGGAGGTCAATGACGAGGAAGATGAGGATGGCCTGATGGATCCAAGAAGGTGGTTAAAAGTCATTGATGCATTTGAGCAACCTCGACTGGT
GTACAATGTTGCTAAAAAGCACTTTGATAGGTATGTTTCAATGATAAAATTTTATCTGAATCGACTAACTCAGTACAGAGATACCTCCAAACCT
TCATTGTTCCCACCTGCGTCTCATAAAACACTCCTCTTCCAAAACCGCTATAATGTTATCCATCAACGTCTCCTTCGCAATGAATCTTTTCAAA
CGCCCGCTTTTCAAGGTGGCAAATCTTCCCTTCAACGCAGCACGTCCGCCATTACCACCCAACAACAATCATACAAATTAACGCCGATAGCTAA
TCTTCTCGGTCGCAATCGCAGCTCTCATATGCTTCTCGGTCTCCTCAGTATTTCACCCACTGGTACCCTCGCCATCAATGACCTGACGGGCAGT
ATCGCTCTCGATCTTACACACGCAGCAGCCATTCC

>Bcin11g01310(MLST5), partial sequence [organism=Botrytis cinerea, strain CH14_ES_11_1]
ACTGACATGGACCTCATGTGGAACCGGCGTAGAATGCGCAACGCTTGAAGTTCCGCTCGAATATGGCGATGCAACGTCAACGGCAAAAGCCAGT
GTTGCGCTTGCTCGTTATCCTGCCACTGTTGCCGCGAGCAAGAAGCTCGGGTCTCTCTTGATAAATCCCGGTGGACCCGGTGCCTCTGGTGTTG
GCTTTGTGCAGTCTGGAGCCGGTGCCGCCGTCTCGACACTGAGTGGTGGATTGTACGATATCATCGGATGGGATCCACGTGGAACCGGTGCTTC
GGCTCCTATTTTGGAATGTTTTGCAAATGCCAGTGCGGAGTATGATTTTAACAACGCGTTTCCATCTGCTCCGAATCTCTGGCTCGGACAATTT
GCGAATGCCAGCGCAAATTCTGCTGTTAGCTCTGCTATCACATCCTTTGACACTTCTGTCGCTGCTCTTGCAAAAGCTTGCGTGGCTCAGAAAT
CTCCCGCTCTTTACACCTCAACAGCAGCATATGTTGCTCGAGACATGGCAGCGATAGTCGATGCATTGGATGGGACCTCTGCAAAACTTAACTA
CTGGGGTTTCTCATATGGAACTATTTTCCTAGCTGAGTTTATCCAAACTTTCCCAGGCCGCGTGGGAAGAGTTCTTGCCGATGGTGTTTTCGAC
GCAAAGGCAAATGCACTCACATACGTTAGCCAACTTCCCAACGATCAACTCAGTGTTCGTGCTTCGTTGAACGATTTTGCAGCTTTCTGCACCA
CCGCCGGTAGTAAAGGTTGCTCTTTTGCCACCGCCCCTACTGGAACCACAGGTACTGTTGCTACCAGACTGGACAACATAATGAAGGATATGTT
CCTCAATCCTATTGTTGCTTCGGGCTTAAGCATCA

>Bcin15g03910(MLST6), partial sequence [organism=Botrytis cinerea, strain CH14_ES_11_1]
GCCAAAACACAAAATCATCCAACGATGAAGATGATACTCCACTTCCCTTGATTATCTGGCATGGACTCGGCGATAATTACAAAGCGGATGGTCT
TGCGCAAGTTGGAAAACTAGCTGAAGCTATTCATCCTGGGACTTTTGTCTACAATATTCATGTAGATGAGGATGCATCTGCAGATAGGACAGCT
ACCTTCTTTGGAAATCTCACTCGTGAGTACATCCCCTATTTTTCCTTTAAATATCATACTAACTCTCTTACCAAGTTCAAATCGAAAAGGTCTG
CGAAGACCTCGCCTCCCATCCTATTCTCTCTACCGCGCCCGCCGTCGACGCAATTGGATTCTCCCAAGGCGGCCAATTCTTGCGTGGTTACATA
TCCCGCTGCAATGCTCCACCCATCCGCTCTCTCCTGACCTTTGGTTCCCAACACAACGGCATTTCTGCCTTCCAAGCCTGTGGTCCTGCCGATT
TCCTCTGTCGCGGTGCTCAAACCCTTTTGCGATCCAACACCTGGTCAACCTTTGTCCAATCTCGTCTCGTACCCGCTCAATACTTCAGAGATCC
GGAAAACCTAGACTCTTACCTTGAATATTCCAATTTCCTTGCCGACATCAATAATGAGCGCGTTCTCAAGAACCAAACATATAAATCCAACATG
GAAAAATTGGAACGATTCGTAATGTATGTCTTTGAAGACGACACAACTGTCATTCCTAAGGAAAGTGGATGGTGGGCTGAAGTCAACGGCACGG
AAGTTACACCACTGAAAGAAAGAGCCATTTATAAAGAAGATTGGCTAGGTTTAAAGACATTGGATGAGGCCGGAAAATTAGTTTTCGAAACCAT
TCCAGGGGGACATATGACGTTAGGAGAGGAGATGCTAGAGAAGGCTTTCAAAGAGTATTTTGGTCCAGCAGGGAAGAAATTTGGG

>Bcin16g03460(MLST7), partial sequence [organism=Botrytis cinerea, strain CH14_ES_11_1]
ATGAACCTCTTGATTTGAACTCTTCATTAATTGCAAGTGATGAACCCCTATTCTGCCCAGAGGATAGTTACAAGACGTATATCATTAGTCGAGA
ACCACTCATGATATATATTGACGGATTTTTGAAAGCGAATGAAAGTAAACATTTGGTTGATGTTAGGTGTGTTATTTATTCTGATGAAATGAAC
AAGAGAGACTGATGAGATAGTGAGCCGCTTTATGAACCGTCAACTGTTTCTCACGGACAGGAAGTTACCATTGATCCTTCAGTTCGGAATTCTG
AAGTGGCGGTTTTAGAGAGGGATGAGGTGGTCAGGTGTATTGAGCATAGAGCGAGGGCATTTCAGGGGTGGAGGGGCGAGATGGGGATTGAGAA
GTTGAGGACGCAGAGGTATGGGGTTGGAGGACATTATGGGATGCATTTGTAAGTTTTGGGGGATTGACGAAGGCTTCTGTCTATTTTCTATCAG

```
CACGGATCTTGAAAGAAAGATAGCATGAACACGCGGGCTAATAACTAGGAAATAGCGATTGGAGCGGAGGTAAACGTGGCATAGACCGATTTAG
TACTTTCATGGTCTATGTCGACGTATCCTCTGATATCGAAGGTGGAGGAACGGAATTCCCACGTATCGTGGGACCAAAAGGAGGAAGGTGGGAG
GACTTCCTGGAAACTACGGAAGCATTGGATCCAAGAACTGGAGAAAATGTAACAGTAGAAGGGGTGACATTCAAACCAATCAAGGGAAATGCCG
TATTCTGGGAAAATACTGACAACAACGGGAGGGGCTATGAT

>Bcin12g03020(MLST8), partial sequence [organism=Botrytis cinerea, strain CH14_ES_11_1]
CGATTGGCTGCGAAGAAAACTGCGCAGCACCGAGTCACCAAAAGCACGAATAAACCATCGGCAAGGGAAATATTCTCTGAACCACAACATGACA
CGAGCGCGGAAGAGTATATCGGGCGAGAAGCTTCATCAAGAGCACCAAAGCGACAACGAGTCGATGATAATTATAACTCTTACGGTGGAAGAAA
TGAAAATACAGCAGCTTATGCTTCCGGGAAACTTCCATCTGGAAGTATAAATGTTGGTGGAGGTAGGAAGACCACTTTTCAAGAAGAACCACGA
ACGGCATTTGTCGCTGGCAAGTTGCCCCCTGGAAGTATCAACATTGGTGGAAAGAAGGCTACCCAAATCGAGGACGAGGGTAGAGCAGCTTATG
CTTCCGGAAAATTGCCTCCAGGAAGTATAAACTCTGGCGCAAAGAAGGCGATTTCATTCCAAGATGAAACGAGACCGGCTTATGTCTCCGGAAA
GCTTCCACATGGTAGTATCGACGGTATGCGAAACCGTGAAATGGCTGCCGTCCACCGCGAAATTGCTGAGGGTGGAGGAAACCAGGCCAGGTT
GTCTCTTCTCTATTCACATTCAATCCTACTTCAAAGAAAACTTTTGATGAACCCGAAGAACAAGCGGAACCGGCAAAACCATCCAATGCGCCTT
TGACAGAGGAAATGGCGACTTTCACCAATCTAGGGTTATCGAGAAGGCTTGCAGCCCATCTATCGACTAAACTCGATATGAAAGCTCCGACCGC
CATTCAAAAAGCATCTGTACAGCAGTTGGTATCGGACGATAGCGATGCTTTCATACAAGCAGAGACTGGATCTGGAAAAACTTTGGCATATCTA
CTACCTATAGTCGAGCGAATATTAGCATTGAGTGAGAATGGCGTACAAA

>Bcin02g07770(MLST9), partial sequence [organism=Botrytis cinerea, strain CH14_ES_11_1]
ATTACAGCTTTCCCTTTCGGTCTTGGCATCTACAGTCATTGCCATCCCTACACCATCACAACTTGAGTCTCGGGCCGTTATCGATTCCGATGCC
GTTGTAGGATTTGCCGAAACTGTTCCCAGTGGGACCGTAGGAACAGTTTATGAGGCATATAAACCATTCCTTAAAGTCGTAAATGGATGCGTAC
CATTCCCTGCCGTCGATGCATCGGGTAACACAGGGTATGTCCTTATATCTTTCTCTTCCACACGATTGCTATTGAGTCTCTAACATATTTTAGT
GGTGGTTTGTCACCAACTGGCAGTAGCAATGGTGGTTGCAGCAGCAGTACCGGTCAAGTATATGTTCGAGGAGGACAAAGCGGATCAAACTACG
CCATCATGTACTCCTGGTAAGTTCTCTCTAAACTTCTCCTTATAGATCCAACCTAACAAAATCTTAGGTACATGCCAAAGGACGAGCCCTCAAC
CGGTATTGGTCACCGTCACGATTGGGAAGGTGTAATTGTCTGGCTCTCCAGCGCCACCGCCACAACTGCCGACAACATCTTAGCCGTTTGTCCT
TCCGCCCACGGAGGCTGGGATTGTTCCACGGATGGCTATTCCCTTTCTGGTACCAGCCCTCTTATCAAGTACGAAAGTATCTGGCCCGTCGATC
ACTCAATGGGTCTTACTAGTACTGTTGGTGGAAAACAACCTATGATTGCTTGGGAGTCTTTACCAACTGCTGCTCAAACTGCTCTTGAGAACAC
CGATTTCGGTGCTGCGAATGTTCCATTCATTCCGGCTGTTTTCACAGACAATCTTG

>Bcin04g02090(MLST10), partial sequence [organism=Botrytis cinerea, strain CH14_ES_11_1]
GCGGAGGATGATATGGCAAAGTCTATGATTACCAAAGCATTTGTAGGCATGAGTAGTAAACTGTCGAATATGACTATGAATGATGTTTACAAGC
CCTACATCCATGTAAGAAATGTAGAATAGAGAGATCAGTAACTGGAACTAATATCGTTTGTAGGCTTTCAAGTTACTTACGCAGTTCAACCCAA
TCACTACAGCTATTGCCGAATCCCCACTGTTTCAAATGGCCGTCTCAGCAAATACCATCGAAAAGTACACACTGCTAGGCCCTTTCTTCAGAAT
ATCTCCTCTGCAACAGGAAGTTACCAGGGAATACTTCAGTGCGCCAAAGACGATAGATAGGCGACACATTGCCACATCTCAAGATGCGTTACGA
TTGACCTTACAAACCCATCAAAAAGATTTACTTGATATCATCAACCACTTTGTTCGAGCAAGTCCAATCGCAAAAAGCAAAACCCTGGATTGGT
TCGCCTACATTGTGAATCAAAATCACAAGCGTCGAGCACTTCAGGTAGACCCGAAAGAAGTGTCTTCTGATGGCTTTATGCACAATGTCACTGT
CGTTCTAGATGGTCTTTGTGAGCCATTCATGGATACCACATTCTCGAAAATTTCGAAGATTGATATTGATTATCTAAGACGTGCGCCTCGTGTA
GATATCAAGGACGAGACCAAGTTGAACGCTGACGAGAGGCTTCTGAGAAGTATTATGAGGACACTGTTCCTGGCACTTCTAATTTCATCTCTGA
GGTCTTCTTTCTCACATTGGCTGCTCATCATTATGGTAGTGAAACTCTTAATGCCACGCATAAGAGTCTGGAGAAAGACATCAAATATATTCAA
AAGCAATTGACTGCCGTTGAAGCA

>Bcin01g07220(MLST1), partial sequence [organism=Botrytis cinerea, strain CH14_ES_11_5]
GATGGCATATCTTGTTTCTTTTCGCATTATGCTTTGGAACGTGCTGGACAAGCTTGGGTTAGTGCTCTGCGTGTAGAAGCACTAAAGAAGATTC
TCGCACAACCGAAGCCATGGTTTGAGGAATCCAGGAATTCACCTAGCCGGTTGAACGAAGTTTTGGATAGGAACTCTGAGGAAATGCGTAATCT
CGTTGGCCGCTTTGCTGGTATTGTATTCACAGCATTTTTTATGCTATTGATATCAATCATTTGGGCTTTCGTGAATACATGGAAACTGACATTA
GTCTCAATGGCAACTGGGCCAGTTATATACGCTGTCACCAAAACGTTCAATCGCGTGAGTGGAAAATGGGAAAACAAGTGCAACTACGCATCTG
AAATGACCACTGGCATATTTTCAGAGACTTTCTCCAACATCAAAGTGGTTCGGGCTTTTACTCTGGAAACTTACTTTAAGACAAAACACACCAA
AGCTACAGAAGAACTCTATAAAGTTGGACTAATACGAGCAAACTACTCGGGATTGCTGTGGGGATTGACAGATGCAATGTCATTCTTCATCACT
GCAACTATCTTTTATTATGCCACGGTTCTCATTACCAAGAGAGAGAGATCAGTATCGCGGCTGCACTACAGACTGTCAATCTTCTATTATTTGGTA
TTTCTAATAGTACGAATATGCTGGCCATGATACCACAAATCAACTCTTCTCGCGTTACAGCTACGCATATGCTTGCATTAGCCAATCTCGATTC
ATCTTCCTCCCACGAAAATAAAGGAACCGAACGGCTTTCGACAATCTTTCCAATCAAATTCAACCGTCTTTCATTCACATACCCTACTCGTCCT
GAAAAACGAACGATATCATCCTTTTCTCTTTCCCTGATTCCTAACTCAACAACTGCACTTGTCGGACCCTCCGGCTCCGGAAAATCTACAATAG
CTGCTCTGCTCATTGGTCTCTATCCGCCAGATACTTCAACACCTCCACCGTTGACATTCAATCGCGTCTCCATAAGTAACTGTCACATTCCGTC
TCTCCGGGCTTCTCTCTCACTCGTTCCACAAATACCGATTCTATTTCCAGCTACCATTCTCCATAACATCATTTATGGTCTCCCAGAATCTTCT
CCTTGTGCTAGTCTTCCATCTGCTATTGCATCAGCAAAAGATGCTGGGATCCATGAATTTATCACATCGCTTCCACAAGGTTATGATACTAT

>Bcin05g07690(MLST2), partial sequence [organism=Botrytis cinerea, strain CH14_ES_11_5]
CACCACTATCACCAAGTCCTTTTCTGCACTTTCCTCATCAAAAACGAACGAGAACATCATCAATAAATACTTTGCTGAAGTAATTGGGTTTTAT
```

TTTGGCCAGGATGCCTTCTCTCTACGACAGCAAGCCTTCGATGATATGTTGTGGGTAGTTCTTGGCTGGCTGGATACTGTCAAATTCATTGATT
TACATTCTGAATTGCACTATTCAAACGACTCTCAGCCAGAATGGTACGGACAACAATATAAACCTGCATTTGCACATCGAGCGCGACTATTTTG
GGAATTGGCTTCACAAGGATGGGATACTACTCTCTGTGGTGGTGGGATGATATGGTCACCATACCTTACTCCATACAAGAACGCAATTACCAAT
GAACTCTATATCGCAGCTTCGATATCGATGTACCTATATTTCCCCGGAGATGACAATCAATCCCCATTTATGCTTTCCAACCCTTCATATCCAC
CTCACGATCCGAAATATCTACAGGCAGCTGTTGATGCTTACAAATGGCTGAATGGTTCCAACATGACGGATTTACAAGGATTATATGTCGACGG
GTACCATATCTCGAATCTTTCTGGCGGTGAAAACACCCATTGCGATTCTAGAAATGAGATGGTATATACCTACAATCAAGGTGTTTTGCTTACT
GGACAACGTGGTTTGTATGACGCAACCGCCGCACGATCATACCTTGTAGATGGCCACAAACTCATCGCGAATGTTATTAATGCCACAGGCTATG
ACCTGAAACACAATGTTGTCATCTCACCGCCACCCAAAGATGGTTCCGCATTGGCAAAGTGGTTTGGCCTGGGTAGGAATGGAATACTGGAAGA
AGGATGCGATTCAAGTGCTTCGTGTTCTCAAAATGGACAAACTTTCAAAGGCATATTCTTTCATCACTTGATTGCGTTCTGTAGTGATTTGCCA
GGGGAGCCTATTGCAGGGACGAAGGAAAGCTTAGAACTCGACAGAGTGTGGCATTCTGACAAATGCTCACAGTATACAAAAT

>Bcin06g01710(MLST3), partial sequence [organism=Botrytis cinerea, strain CH14_ES_11_5]
TGGTGAGTCTGACTTTTGTATTTGAGCGTTAAGATAGACACTGATATACCAAGGCAATCACCTAATTTCATGGCGTTCTTCCCTGCATCATCTA
CCTACCCTGGAATGCTGGGAGAATTATACTCAGCAGCTTTCACAGCACCTGCTTTCAATTGGATCTGTTCCCCTGCTGTGACAGAATTGGAGAC
GGTTGTAATGGATTGGCTGGCCAAGCTTCTCAATCTCCCAGACTGTTATTTGTCTTCGACTCATGGTGGTGGTGTCATCCAAGGATCAGCCTCG
GAAGCTATCGTTACCGTTATGGTTGCTGCCCGCGATAAATATCTTCGTGAAACTACTGAAGGTCTGTCGGGCATTGAACTCGAGGATGCGATTG
CATATAAGAGGAGTAAGCTAGTTGCACTAGGAAGCGAAATGGCACACAGCTCCACGCAGAAAGCAGCGCAAATAGCTGGCGTTAGATTCCGATC
GATTCCAGTACTCGCATCCAATGATTTCGCCATGACGGGTGATGATTTAGAGAAGGTATTGAAAGAATGCAAATCTCAAGGATTGGAACCCTTC
TATCTAACTTCGACTTTGGGAACAACATCTACATGCGCAGTTGACGACTTCGCATCTATTGCAACAGTACTTTCAAAATATGCACCTCCAGATG
TTGCAGGCGAGATCTGGGTTCACGTCGATGCTGCTTATGCAGGTGCAGCTTTGGTTTGCCCTGAATACCATCATCTAACATCGTCCTTCCAGCA
TTTCCATTCCTTCGATATGAACATGCACAAATGGCTTCTGACAAATTTCGACGCTTCTTGTCTATATGTCAAGAAACGCAAAGATCTGATCGAT
GCACTCTCCATAACACCAAGTTATCTTCGCAACGAGTTTTCAGAGAGTGGACTCGTAACCGACTATCGGGACTGGCAAATTCCTCTCGGAAGAC
GCTTCCGAAGCTTAAAGATTTGGTTTGTCCTCAGAACCTAC

>Bcin09g03030(MLST4), partial sequence [organism=Botrytis cinerea, strain CH14_ES_11_5]
ACCCTTCAACCCCACCGCAGCTCCTATACCGAAAGCGAGTATCCTACCAATTCTCCTTCCACCTGCGACTTTAAGACCATTGGCTTTCCGCACT
TTCACAAAAAAGCATAGTTTAACATTGACGTCGTCGGCATTACAAGTGTTGGCTACTTTTATTGGAAAGCATTGTGGGACAGGATGGAGGGAAG
AAGGACTGGCAGAGAGAGTCTTGGAGGAGGTCGCCAAGAGTTGGAAGAATAGGAGTGGCGGTGTCATTGTCGAGGGCGAGGGAACGGAATTGAA
GGAGATTCTGAAAGCTTTGGAAGGGAATATGAGTGGTGGAAGGATAGTCATAGGAAGAGAGCTAAGCCGGCAGAATAGTTTAGTACTGGGATCA
TCACAATATGGAGAGGTCAATCATACAAGACTTGGGCTACGGCCAGGGAATATACCCAGAGAGGATAGTCAGTCAAGTTTGGGAATGTCAACGT
TGGAGGTCAATGACGAGGAAGATGAGGATGGCCTGATGGATCCAAGAAGGTGGTTAAAAGTCATTGATGCATTTGAGCAACCTCGACTGGTGTA
CAATGTTGCTAAAAAGCACTTTGATAGGTATGTTTCAATGATAAAATTTTATCTGAATCGACTAACTCAGTACAGAGATACCTCCAAACCTTCA
TTGTTCCCACCTGCGTCTCATAAAACACTCCTCTTCCAAAACCGCTATAATGTTATCCATCAACGTCTCCTTCGCAATGAATCTTTTCAAACGC
CCGCTTTTCAAGGTGGCAAATCTTCCCTTCAACGCAGCACGTCCGCCATTACCACCCAACAACAATCATACAAATTAACGCCGATAGCTAATCT
TCTCGGTCGCAATCGCAGCTCTCATATGCTTCTCGGTCTCCTCAGTATTTCACCCACTGGTACCCTCGCCATCAATGACCTGACGGGCAGTATC
GCTCTCGATCTTACACACGCAGCAGCCATTCC

>Bcin11g01310(MLST5), partial sequence [organism=Botrytis cinerea, strain CH14_ES_11_5]
ACTGACATGGACCTCATGTGGAACCGGCGTAGAATGCGCAACGCTTGAAGTTCCGCTCGAATATGGCGATGCAACGTCAACGGCAAAAGCCAGT
GTTGCGCTTGCTCGTTATCCTGCCACTGTTGCCGCGAGCAAGAAGCTCGGGTCTCTCTTGATAAATCCCGGTGGACCCGGTGCCTCTGGTGTTG
GCTTTGTGCAGTCTGGAGCCGGTGCCGCCGTCTCGACACTGAGTGGTGGATTGTACGATATCATCGGATGGGATCCACGTGGAACCGGTGCTTC
GGCTCCTATTTTGGAATGTTTTGCAAATGCCAGTGCGGAGTATGATTTTAACAACGCGTTTCCATCTGCTCCGAATCTCTGGCTCGGACAATTT
GCGAATGCCAGCGCAAATTCTGCTGTTAGCTCTGCTATCACATCCTTTGACACTTCTGTCGCTGCTCTTGCAAAAGCTTGCGTGGCTCAGAAAT
CTCCCGCTCTTTACACCTCAACAGCAGCATATGTTGCTCGAGACATGGCAGCGATAGTCGATGCATTGGATGGGACCTCTGCAAAACTTAACTA
CTGGGGTTTCTCATATGGAACTATTTTCCTAGCTGAGTTTATCCAAACTTTCCCAGGCCGCGTGGGAAGAGTTCTTGCCGATGGTGTTTTCGAC
GCAAAGGCAAATGCACTCACATACGTTAGCCAACTTCCCAACGATCAACTCAGTGTTCGTGCTTCGTTGAACGATTTTGCAGCTTTCTGCACCA
CCGCCGGTAGTAAAGGTTGCTCTTTTGCCACCGCCCCTACTGGAACCACAGGTACTGTTGCTACCAGACTGGACAACATAATGAAGGATATGTT
CCTCAATCCTATTGTTGCTTCGGGCTTAAGCATCA

>Bcin15g03910(MLST6), partial sequence [organism=Botrytis cinerea, strain CH14_ES_11_5]
CGCCAAAACACAAAATCATCCAACGATGAAGATGATACTCCACTTCCCTTGATTATCTGGCATGGACTCGGCGATAATTACAAAGCGGATGGTC
TTGCGCAAGTTGGAAAACTAGCTGAAGCTATTCATCCTGGGACTTTTGTCTACAATATTCATGTAGATGAGGATGCATCTGCAGATAGGACAGC
TACCTTCTTTGGAAATCTCACTCGTGAGTACATCCCCTATTTTTCCTTTAAATATCATACTAACTCTCTTACCAAGTTCAAATCGAAAAGGTCT
GCGAAGACCTCGCCTCCCATCCTATTCTCTCTACCGCGCCCGCCGTCGACGCAATTGGATTCTCCCAAGGCGGCCAATTCTTGCGTGGTTACAT
ATCCCGCTGCAATGCTCCACCCATCCGCTCTCTCCTGACCTTTGGTTCCCAACACAACGGCATTTCTGCCTTCCAAGCCTGTGGTCCTGCCGAT
TTCCTCTGTCGCGGTGCTCAAACCCTTTTGCGATCCAACACCTGGTCAACCTTTGTCCAATCTCGTCTCGTACCCGCTCAATACTTCAGAGATC
CGGAAAACCTAGACTCTTACCTTGAATATTCCAATTTCCTTGCCGACATCAATAATGAGCGCGTTCTCAAGAACCAAACATATAAATCCAACAT

GGAAAAATTGGAACGATTCGTAATGTATGTCTTTGAAGACGACACAACTGTCATTCCTAAGGAAAGTGGATGGTGGGCTGAAGTCAACGGCACG
GAAGTTACACCACTGAAAGAAAGAGCCATTTATAAAGAAGATTGGCTAGGTTTAAAGACATTGGATGAGGCCGGAAAATTAGTTTTCGAAACCA
TTCCAGGGGGACATATGACGTTAGGAGAGGAGATGCTAGAGAAGGCTTTCAAAGAGTATTTTGGTCCAGCAGGGAAGAAATTTGGGG

>Bcin16g03460(MLST7), partial sequence [organism=Botrytis cinerea, strain CH14_ES_11_5]
ATGAACCTCTTGATTTGAACTCTTCATTAATTGCAAGTGATGAACCCCTATTCTGCCCAGAGGATAGTTACAAGACGTATATCATTAGTCGAGA
ACCACTCATGATATATATTGACGGATTTTTGAAAGCGAATGAAAGTAAACATTTGGTTGATGTTAGGTGTGTTATTTATTCTGATGAAATGAAC
AAGAGAGACTGATGAGATAGTGAGCCGCTTTATGAACCGTCAACTGTTTCTCACGGACAGGAAGTTACCATTGATCCTTCAGTTCGGAATTCTG
AAGTGGCGGTTTTAGAGAGGGATGAGGTGGTCAGGTGTATTGAGCATAGAGCGAGGGCATTTCAGGGGTGGAGGGGCGAGATGGGGATTGAGAA
GTTGAGGACGCAGAGGTATGGGGTTGGAGGACATTATGGGATGCATTTGTAAGTTTTGGGGGATTGACGAAGGCTTCTGTCTATTTTCTATCAG
CACGGATCTTGAAAGAAAGATAGCATGAACACGCGGGCTAATAACTAGGAAATAGCGATTGGAGCGGAGGTAAACGTGGCATAGACCGATTTAG
TACTTTCATGGTCTATGTCGACGTATCCTCTGATATCGAAGGTGGAGGAACGGAATTCCCACGTATCGTGGGACCAAAAGGAGGAAGGTGGGAG
GACTTCCTGGAAACTACGGAAGCATTGGATCCAAGAACTGGAGAAAATGTAACAGTAGAAGGGGTGACATTCAAACCAATCAAGGGAAATGCCG
TATTCTGGGAAAATACTGACAACAACGGGGGGGGCTATGAT

>Bcin12g03020(MLST8), partial sequence [organism=Botrytis cinerea, strain CH14_ES_11_5]
CGATTGGCTGCGAAGAAAACTGCGCAGCACCGAGTCACCAAAAGCACGAATAAACCATCGGCAAGGGAAATATTCTCTGAACCACAACATGACA
CGAGCGCGGAAGAGTATATCGGGCGAGAAGCTTCATCAAGAGCACCAAAGCGACAACGAGTCGATGATAATTATAACTCTTACGGTGGAAGAAA
TGAAAATACAGCAGCTTATGCTTCCGGGAAACTTCCATCTGGAAGTATAAATGTTGGTGGAGGTAGGAAGACCACTTTTCAAGAAGAACCACGA
ACGGCATTTGTCGCTGGCAAGTTGCCCCCTGGAAGTATCAACATTGGTGGAAAGAAGGCTACCCAAATCGAGGACGAGGGTAGAGCAGCTTATG
CTTCCGGAAAATTGCCTCCAGGAAGTATAAACTCTGGCGCAAAGAAGGCGATTTCATTCCAAGATGAAACGAGACCGGCTTATGTCTCCGGAAA
GCTTCCACATGGTAGTATCGACGGTATGCGAAACCGTGAAATGGCTGCCGTCCACCGCGAAATTGCTGAGGGTGGGAGGAAACCAGGCCAGGTT
GTCTCTTCTCTATTCACATTCAATCCTACTTCAAAGAAAACTTTTGATGAACCCGAAGAACAAGCGGAACCGGCAAAACCATCCAATGCGCCTT
TGACAGAGGAAATGGCGACTTTCACCAATCTAGGGTTATCGAGAAGGCTTGCAGCCCATCTATCGACTAAACTCGATATGAAAGCTCCGACCGC
CATTCAAAAAGCATCTGTACAGCAGTTGGTATCGGACGATAGCGATGCTTTCATACAAGCAGAGACTGGATCTGGAAAAACTTTGGCATATCTA
CTACCTATAGTCGAGCGAATATTAGCATTGAGTGAGAATGGCGTACAAA

>Bcin02g07770(MLST9), partial sequence [organism=Botrytis cinerea, strain CH14_ES_11_5]
CAGCTTTCCCTTTCGGTCTTGGCATCTACAGTCATTGCCATCCCTACACCATCACAACTTGAGTCTCGGGCCGTTATCGATTCCGATGCCGTTG
TAGGATTTGCCGAAACTGTTCCCAGTGGGACCGTAGGAACAGTTTATGAGGCATATAAACCATTCCTTAAAGTCGTAAATGGATGCGTACCATT
CCCTGCCGTCGATGCATCGGGTAACACAGGGTATGTCCTTATATCTTTCTCTTCCACACGATTGCTATTGAGTCTCTAACATATTTTAGTGGTG
GTTTGTCACCAACTGGCAGTAGCAATGGTGGTTGCAGCAGCAGTACCGGTCAAGTATATGTTCGAGGAGGACAAAGCGGATCAAACTACGCCAT
CATGTACTCCTGGTAAGTTCTCTCTAAACTTCTCCTTATAGATCCAACCTAACAAAATCTTAGGTACATGCCAAAGGACGAGCCCTCAACCGGT
ATTGGTCACCGTCACGATTGGGAAGGTGTAATTGTCTGGCTCTCCAGCGCCACCGCCACAACTGCCGACAACATCTTAGCCGTTTGTCCTTCCG
CCCACGGAGGCTGGGATTGTTCCACGGATGGCTATTCCCTTTCTGGTACCAGCCCTCTTATCAAGTACGAAAGTATCTGGCCCGTCGATCACTC
AATGGGTCTTACTAGTACTGTTGGTGGAAAACAACCTATGATTGCTTGGGAGTCTTTACCAACTGCTGCTCAAACTGCTCTTGAGAACACCGAT
TTCGGTGCTGCGAATGTTCCATTCATTCCGGCTGTTTTCACAGACAATCT

>Bcin04g02090(MLST10), partial sequence [organism=Botrytis cinerea, strain CH14_ES_11_5]
CGGAGGATGATATGGCAAAGTCTATGATTACCGAAGCATTTGTAGGCATGAGTAGTAAACTGTCGAATATGACTATGAATGATGTTTACAAGCC
CTACATCCATGTAAGAAATGTAGAATAGAGAGATCAGTAACTGGAACTAATATCGTTTGTAGGCTTTCAAGTTACTTACGCAGTTCAACCCAAT
CACTACAGCTATTGCCGAATCCCCACTGTTTTAAATGGCCGTCTCAGCAAATACCATCGAAAAGTACACACTGCTAGGCCCTTTCTTCAGAATA
TCTCCTCTGCAACAGGAAGTTACCAGGGAATACTTCAGTGCGCCAAAGACGATAGATAGGCGACACATTGCCACATCTCAAGATGCGTTACGAT
TGACCTTACAAACCCATCAAAAAGATTTACTTGATATCATCAACCACTTTGTTCGAGCAAGTCCAATCGCAAAAAGCAAAACCCTGGATTGGTT
CGCCTACATTGTGAATCAAAATCACAAGCGTCGAGCACTTCAGGTAGACCCGAAAGAAGTGTCTTCTGATGGCTTTATGCACAATGTCACTGTC
GTTCTAGATGGTCTTTGTGAGCCATTCATGGATACCACATTCTCGAAAATTTCGAAGATTGATATTGATTATCTAAGACGTGCGCCTCGTGTAG
ATATCAAGGACGAGACCAAGTTGAACGCTGACGAGAAGGCTTCTGAGAAGTATTATGAGGACACTGTTCCTGGCACTTCTAATTTCATCTCTGA
GGTCTTCTTTCTCACATTGGCTGCTCATCATTATGGTAGTGAAGCTCTTAATGCCACGCATAAGAGTCTGGAGAAAGACATCAAATATATTCAA
AAGCAATTGACTGCCGTTGAAGCAG

>Bcin01g07220(MLST1), partial sequence [organism=Botrytis cinerea, strain D06_1_30]
GATGGCATATCTTGTTTCTTTTCGCATTATGCTTTGGAACGTGCTGGACAAGCTTGGGTTAGTGCTCTGCGTGTAGAAGCACTAAAGAAGATTC
TCGCACAACCGAAGTCATGGTTTGAGGAATCCAGGAATTCACCTAGCCGGTTGAACGAAGTTTTGGATAGGAACTCTGAGGAAATGCGTAATCT
CGTTGGCCGCTTTGCTGGTATTGTATTCACAGCATTTTTTATGCTATTGATATCAATCATTTGGGCTTTCGTGAATACATGGAAACTGACATTA
GTCTCGATGGCAACTGGGCCAGTTATATACGCTGTCACCAAACGTTCAATCGCGTGAGTGGAAAATGGGAAAACAAGTGCAACTACGCCTCTGA
AATGACCACTGGCATATTTTCAGAGACGTTCTCCAACATCAAAGTGGTTCGGGCTTTTACTCTGGAAACTTACTTTGAGACAAAACACACCAAA
GCTACAGAAGAACTTTATAAAGTTGGACTAATACGAGCAAACTACTCGGGATTGCTGTGGGGATTGACAGATGCGATGTCATTCTTCATCACTG

CAACTATCTTTTATTATGCCACGGTTCTCATTACCAAGAGAGAGATCAGTATCGCGACTGCACTACAGACTGTCAATCTTCTATTATTTGGTAT
TTCTAATAGTACGAATATGCTGGCCATGATACCACAAATCAACTCTTCTCGCGTTACAGCTACGCATATGCTTGCATTAGCCAATCTCGATTCA
TCTTCCTCCCACGAAAATAAAGGAACCGAACGGCTTTCGACAATCTTTCCAATCAAATTCAACCGTCTTTCATTCACATACCCTACTCGTCCTG
AAAAACGAACGATATCATCCTTTTCTCTTTCCCTGATTCCTAACTCAACAACTGCACTTGTCGGACCCTCCGGCTCCGGAAAATCTACAATAGC
TGCTCTGCTCATTGGTCTCTATCCGCCAGATACTTCAACACCTCCACCGTTGACATTCAATCGCGTCTCCATAAGTAACTGTCACATTCCGTCT
CTCCGGGCTTCTCTCTCACTCGTTCCACAAATACCGATTCTATTTCCAGCTACCATTCTCCATAACATCATTTATGGTCTCCCAGAATCTTCTC
CTTGTGCTAGTCTTCCATCTGCTATTGCATCAGCAAAAGATGCTGGGATCCATGAATTTATCACATCGCTTCCACAAGGTTATGATACTAT

>Bcin05g07690(MLST2), partial sequence [organism=Botrytis cinerea, strain D06_1_30]
CACCACTATCACCAAGTCCTTTTCTGCACTTTCCTCATCAAAAACGAACGAGAACATCATCAATAAATACTTTGCTGAAGTAATTGGGTTTTAT
TTTGGCCAGGATGCCTTCTCTCTACGACAGCAAGCCTTCGATGATATGTTGTGGGTAGTTCTTGGCTGGCTGGATACTGTCAAATTCATTGATT
TACATTCTGAATTGCACTATTCAAACGACTCTCAGCCAGAATGGTACGGACAACAATATAAACCTGCATTTGCACATCGAGCGCGACTATTTTG
GGAATTGGCTTCACAAGGATGGGATACTACTCTCTGTGGTGGTGGGATGATATGGTCACCATACCTTACTCCATACAAGAACGCAATTACCAAT
GAACTCTATATCGCAGCTTCGATATCGATGTACCTATATTTCCCCGGAGATGACAATCAATCCCCATTTATGCTTTCCAACCCTTCATATCCAC
CTCACGATCCGAAATATCTACAGGCAGCTGTTGATGCTTACAAATGGCTGAATGGTTCCAACATGACGGATTTACAAGGATTATATGTCGACGG
GTACCATATCTCGAATCTTTCTGGCGGTGAAAACACCCATTGCGATTCTAGAAATGAGATGGTATATACCTACAATCAAGGTGTTTTGCTTACT
GGACAACGTGGTTTGTATGACGCAACCGCCGCACGATCATACCTTGTAGATGGCCACAAACTCATCGCGAATGTTATTAATGCCACAGGCTATG
ACCTGAAACACAATGTTGTCATCTCACCGCCACCCAAAGATGGTTCCGCATTGGCAAAGTGGTTTGGCCTGGGTAGGAATGGAATACTGGAAGA
AGGATGCGATTCAAGTGCTTCGTGTTCTCAAAATGGACAAACTTTCAAAGGCATATTCTTTCATCACTTGATTGCGTTCTGTAGTGATTTGCCA
GGGGAGCCTATTGCAGGGACGAAGGAAAGCTTAGAACTCGACAGAGTGTGGCATTCTGACAAATGCTCACAGTATACAAAAT

>Bcin06g01710(MLST3), partial sequence [organism=Botrytis cinerea, strain D06_1_30]
GTGAGTCTGACTTTTGTATTTGAGCGTTAAGATAGACACTGATATACCAAGGCAATCACCTAATTTCATGGCGTTCTTCCCTGCATCATCTACC
TACCCTGGAATGCTGGGAGAATTATACTCAGCAGCTTTCACAGCACCTGCTTTCAATTGGATCTGTTCCCCTGCTGTGACAGAATTGGAGACGG
TTGTAATGGATTGGCTGGCCAAGCTTCTCAATCTCCCAGACTGTTATTTGTCTTCGACTCATGGTGGTGGTGTCATCCAAGGATCAGCCTCGGA
AGCTATCGTTACCGTTATGGTTGCTGCCCGCGATAAATATCTTCGTGAAACTACTGAAGGTCTGTCGGGCATTGAGCTCGAGGATGCGATTGCA
TATAAGAGGAGTAAGCTAGTTGCACTAGGAAGCGAAATGGCACACAGCTCCACGCAGAAAGCAGCGCAAATAGCTGGCGTTAGATTCCGATCGA
TTCCAGTACTCGCATCCAATGATTTCGCCATGACGGGTGATGATTTAGAGAAGGTGTTGAAAGAATGCAAATCTCAAGGATTGGAACCCTTCTA
TCTAACTTCGACTTTGGGAACAACATCTACATGCGCAGTTGACGACTTCGCATCTATTGCAACAGTACTTTCAAAATATGCACCTCCAGATGTT
GCAGGCGAGATCTGGGTTCACGTCGATGCTGCTTATGCAGGTGCAGCTTTGGTTTGCCCTGAATACCATCATCTAACATCGTCCTTCCAGCATT
TCCATTCCTTCGATATGAACATGCACAAATGGCTTCTGACAAATTTCGACGCTTCTTGTCTATATGTCAAGAAACGCAAAGATCTGATCGATGC
ACTCTCCATAACACCAAGTTATCTTCGCAACGAGTTTTCAGAGAGTGGACTCGTAACCGACTATCGGGACTGGCAAATTCCTCTCGGAAGACGC
TTCCGAAGCTTAAAGATTTGGTTTGTCCTCAGAACCTAC

>Bcin09g03030(MLST4), partial sequence [organism=Botrytis cinerea, strain D06_1_30]
ACCCTTCAACCCCACCGCAGCTCCTATACCGAAAGCGAGTATCCTACCAATTCTCCTTCCACCTGCGACTTTAAGACCATTGGCTTTCCGCACT
TTCACAAAAAAGCATAGTTTAACATTGACGTCGTCGGCATTACAAGTGTTGGCTACTTTTATTGGAAAGCATTGTGGGACAGGATGGAGGGAAG
AAGGACTGGCAGAGAGAGAGTCTTGGAGGAGGTCGCCAAGAGTTGGAAGAATAGGAGTGGCGGTGTCATTGTCGAGGGCGAGGGAACGGAATTGAA
GGAGATTCTGAAAGCTTTGGAAGGGAATATGAGTGGTGGAAGGATAGTCATAGGAAGAGAGCTAAGCCGGCAGAATAGTTTAGTACTGGGATCA
TCACAATATGGAGAGGTCAATCATACAAGACTTGGGCTACGGCCAGGGAATATACCCAGAGAGGATAGTCAGTCAAGTTTGGGAATGTCAACGT
TGGAGGTCAATGACGAGGAAGATGAGGATGGCCTGATGGATCCAAGAAGGTGGTTAAAAGTCATTGATGCATTTGAGCAACCTCGACTGGTGTA
CAATGTTGCTAAAAAGCACTTTGATAGGTATGTTTCAATGATAAAATTTTATCTGAATCGACTAACTCAGTACAGAGATACCTCCAAACCTTCA
TTGTTCCCACCTGCGTCTCATAAAACACTCCTCTTCCAAAACCGCTATAATGTTATCCATCAACGTCTCCTTCGCAATGAATCTTTTCAAACGC
CCGCTTTTCAAGGTGGCAAATCTTCCCTTCAACGCAGCACGTCCGCCATTACCACCCAACAACAATCATACAAATTAACGCCGATAGCTAATCT
TCTCGGTCGCAATCGCAGCTCTCATATGCTTCTCGGTCTCCTCAGTATTTCACCCACTGGTACCCTCGCCATCAATGACCTGACGGGCAGTATC
GCTCTCGATCTTACACACGCAGCAGCCATTCCCGAAGATAGCGCCTGGTTT

>Bcin11g01310(MLST5), partial sequence [organism=Botrytis cinerea, strain D06_1_30]
ACTGACATGGACCTCATGTGGAACCGGCGTAGAATGCGCAACGCTTGAAGTTCCGCTCGAATATGGCGATGCAACGTCAACGGCAAAAGCCAGT
GTTGCGCTTGCTCGTTATCCTGCCACTGTTGCCGCGAGCAAGAAGCTCGGGTCTCTCTTGATAAACCCCGGTGGACCCGGTGCCTCTGGTGTTG
GCTTTGTGCAGTCTGGAGCCGGTGCCGCCGTCTCGACACTGAGTGGTGGATTGTACGATATCATCGGATGGGATCCACGTGGAACCGGTGCTTC
GGCTCCTATTTTGGAATGTTTTGCAAATGCCAGTGCGGAGTATGATTTTAACAACGCGTTTCCATCTGCTCCGAATCTCTGGCTCGGACAATTT
GCGAATGCCAGCGCAAATTCTGCTGTTAGCTCTGCTATCACATCCTTTGACACTTCTGTCGCTGCTCTTGCAAAAGCTTGCGTGGCTCAGAAAT
CTCCCGCTCTTTACACCTCAACAGCAGCATATGTTGCTCGAGACATGGCAGCGATAGTCGATGCATTGGATGGGACCTCTGCAAAACTTAACTA
CTGGGGTTTCTCATATGGAACTATTTTCCTAGCTGAGTTTATCCAAACTTTCCCAGGCCGTGTGGGAAGAGTTCTTGCCGATGGTGTTTTCGAC
GCAAAGGCAAATGCACTCACATACGTTAGCCAACTTCCCAACGATCAACTCAGTGTTCGTGCTTCGTTGAACGATTTTGCAGCTTTCTGCACCA
CCGCCGGTAGTAAAGGTTGCTCTTTTGCCACCGCCCCTACTGGAACCTCAGGTACTGTTGCTACCAGACTGGACAACATAATGAAGGATATGTT

CCTCAATCCTATTGTTGCTTCGGGCTTAAGCATCA

>Bcin15g03910(MLST6), partial sequence [organism=Botrytis cinerea, strain D06_1_30]
GCCAAAACACAAAATCATCCAACGATGAAGATGATACTCCACTTCCCTTGATTATCTGGCATGGACTCGGCGATAATTACAAAGCGGATGGTCT
TGCGCAAGTTGGAAAACTAGCTGAAGCTATTCATCCTGGGACTTTTGTCTACAATATTCATGTAGATGAGGATGCATCTGCAGATAGGACAGCT
ACCTTCTTTGGAAATCTCACTCGTGAGTACATCCCCTATTTTTCCTTTAAATATCATACTAACTCTCTTACCAAGTTCAAATCGAAAAGGTCTG
CGAAGACCTCGCCTCCCATCCTATTCTCTCTACCGCGCCCGCCGTCGACGCAATTGGATTCTCCCAAGGCGGCCAATTCTTGCGTGGCTACATA
TCCCGCTGCAATGCTCCACCCATCCGCTCTCTCCTGACCTTCGGTTCCCAACACAACGGCATTTCTGCCTTCCAAGCCTGTGGTCCTGCCGATT
TCCTCTGTCGCGGTGCTCAAACCCTTTTGCGATCCAACACCTGGTCAACCTTTGTCCAATCTCGTCTCGTACCCGCTCAATACTTCAGAGATCC
GGAAAACCTAGACTCTTACCTTGAATATTCCAATTTCCTTGCCGACATCAATAATGAGCGCGTTCTCAAGAACCAAACATATAAATCCAACATG
GAAAAATTGGAACGATTCGTAATGTATGTCTTTGAAGACGATACAACTGTCATCCCTAAGGAAAGTGGATGGTGGGCTGAAGTCAACGGCACGG
AAGTTACACCACTGAAAGAAAGAGCCATTTATAAAGAAGATTGGCTAGGTTTAAAGACATTGGATGAGGCCGGAAAATTAGTTTTCGAAACCAT
TCCAGGGGGACATATGACGTTAGGAGAGGAGATGCTAGAGAAGGCTTTCAAAGAGTATTTTGGTCCAGCAGGGAAGAAATTTGGG

>Bcin16g03460(MLST7), partial sequence [organism=Botrytis cinerea, strain D06_1_30]
ATGAACCTCTTGATTTGAACTCTTCATTAATTGCAAGTGATGAACCCCTATTCTGCCCAGAGGATAGTTACAAGACGTATATCATTAGTCGAGA
ACCACTCATGATATATATTGACGGATTTTTGAAAGCGAATGAAAGTAAACATTTGGTTGATGTTAGGTGTGTTATTTATTCTGATGAAATGAAC
AAGAGAGACTGATGAGATAGTGAGCCGCTTTATGAACCGTCAACTGTTTCTCACGGACAGGAAGTTACCATTGATCCTTCAGTTCGGAATTCTG
AAGTGGCGGTTTTAGAGAGGGATGAGGTGGTCAGGTGTATTGAGCATAGAGCGAGGGCATTTCAGGGGTGGAGGGGCGAGATGGGGATTGAGAA
GTTGAGGACGCAGAGGTATGGGGTTGGAGGACATTATGGGATGCATTTGTAAGTTTTGGGGGATTGACGAAGGCTTCTGTCTATTTTCTATCAG
CACGGATCTTGAAAGAAAGATAGCATGAACACGCGGGCTAATAACTAGGAAATAGCGATTGGAGCGGAGGTAAACGTGGCATAGACCGATTTAG
TACTTTCATGGTATATGTCGACGTATCCTCTGATATCGAAGGTGGAGGAACGGAATTCCCACGTATTGTGGGACCAAAAGGAGGAAGGTGGGAG
GACTTCCTGGAAACTACGGAAGCATTGGATCCAAGAACTGGAGAAAATGTAACAGTAGAAGGGGTGACATTCAAACCAATCAAGGGAAATGCCG
TATTCTGGGAAAATACTGACAACAACGGGAGGGGCTATGAT

>Bcin12g03020(MLST8), partial sequence [organism=Botrytis cinerea, strain D06_1_30]
CGATTGGCTGCGAAGAAAACTGCGCAGCACCGAGTCACCAAAAGCACGAATAAACCATCGGCAAGGGAAATATTCTCTGAACCACAACATGACA
CGAGCGCGGAAGAGTATATCGGGCGAGAAGCTTCATCAAGAGCACCAAAGCGACAACGAGTCGATGATAATTATAACTCTTACGGTGGAAGAAA
TGAAAATACAGCAGCTTATGCTTCCGGGAAACTTCCATCTGGAAGTATAAATGTTGGTGGAGGTAGGAAGACCACTTTTCAAGAAGAACCACGA
ACGGCATTTGTCGCTGGCAAGTTGCCCCCTGGAAGTATCAACATTGGTGGAAAGAAGGCTACCCAAATCGAGGACGAGGGTAGAGCAGCTTATG
CTTCCGGAAAATTGCCTCCAGGAAGTATAAACTCTGGCGCAAAGAAGGCGATTTCATTCCAAGATGAAACGAGACCGGCTTATGTCTCCGGAAA
GCTTCCACATGGTAGTATCGACGGTATGCGAAACCGTGAAATGGCTGCCGTCCACCGCGAAATTGCTGAGGGTGGAGGAAACCAGGCCAGGTT
GTCTCTTCTCTATTCACATTCAATCCTACTTCAAAGAAAACTTTTGATGAACCCGAAGAACAAGCGGAACCGGCAAAACCATCCAATGCGCCTT
TGACAGAGGAAATGGCGACTTTCACCAATCTAGGGTTATCGAGAAGGCTTGCAGCCCATCTATCGACTAAACTCGATATGAAAGCTCCGACCGC
CATTCAAAAAGCATCTGTACAGCAGTTGGTATCGGACGATAGCGATGCTTTCATACAAGCAGAGACTGGATCTGGAAAAACTTTGGCATATCTA
CTACCTATAGTCGAGCGAATATTAGCATTGAGTGAGAATGGCGTACAAATA

>Bcin02g07770(MLST9), partial sequence [organism=Botrytis cinerea, strain D06_1_30]
TACAGCTTTCCCTTTCGGTCTTGGCATCTACAGTCATTGCCATCCCTACACCATCACAACTTGAGTCTCGGGCCGTTATCGATTCCGATGCCGT
TGTAGGATTTGCCGAAACTGTTCCCAGTGGGACCGTAGGAACAGTTTATGAGGCATATAAACCATTCCTTAAAGTCGTAAATGGATGCGTACCA
TTCCCTGCCGTCGATGCATCGGGTAACACAGGGTATGTCCTTATATCTTTCTCTTCCACACGATTGCTATTGAGTCTCTAACATATTTTAGTGG
TGGTTTGTCACCAACTGGCAGTAGCAATGGTGGTTGCAGCAGCAGTACCGGTCAAGTATATGTTCGAGGAGGACAAAGCGGATCAAACTACGCC
ATCATGTACTCCTGGTAAGTTCTCTCTAAACTTCTCCTTATAGATCCAACCTAACAAAATCTTAGGTACATGCCAAAGGACGAGCCCTCAACCG
GTATTGGTCACCGTCACGATTGGGAAGGTGTAATTGTCTGGCTCTCCAGCGCCACCGCCACAACTGCCGACAACATCTTAGCCGTTTGTCCTTC
CGCCCACGGAGGCTGGGATTGTTCCACGGATGGCTATTCCCTTTCTGGTACCAGCCCTCTTATCAAGTACGAAAGTATCTGGCCCGTCGATCAC
TCAATGGGTCTTACTAGTACTGTTGGTGGAAAACAACCTATGATTGCTTGGGAGTCTTTACCAACTGCTGCTCAAACTGCTCTTGAGAACACCG
ATTTCGGTGCTGCGAATGTTCCATTCATTCCGGCTGTTTTCACAGACAATCT

>Bcin04g02090(MLST10), partial sequence [organism=Botrytis cinerea, strain D06_1_30]
CGGAGGATGATATGGCAAAGTCTATGATTACCAAAGCATTTGTAGGCATGAGTAGTAAACTGTCGAATATGACTATGAATGATGTTTACAAGCC
CTACATCCATGTAAGAAATGTAGAATAGAGAGATCAGTAACTGGAACTAATATCGTTTGTAGGCTTTCAAGTTACTTACGCAGTTCAACCCAAT
CACTACAGCTATTGCCGAATCCCCACTGTTTCAAATGGCCGTCTCAGCAAATACCATCGAAAAGTACACACTGCTAGGCCCCTTTCTTCAGAATA
TCTCCTCTGCAACAGGAAGTTACCAGGGAATACTTCAGTGCGCCAAAGACGATAGATAGGCGACACATTGCCACATCTCAAGATGCGTTACGAT
TGACCTTACAAACCCATCAAAAAGATTTACTTGATATCATCAACCACTTTGTTCGAGCAAGTCCAATCGCAAAAAGCAAAACCCTGGATTGGTT
CGCCTACATTGTGAATCAAAATCACAAGCGTCGAGCACTTCAGGTAGACCCGAAAGAAGTGTCTTCTGATGGCTTTATGCACAATGTCACTGTC
GTTCTAGATGGTCTTTGTGAGCCATTCATGGATACCACATTCTCGAAAATTTCGAAGATTGATATTGATTATCTAAGACGTGCGCCTCGTGTAG
ATATCAAGGACGAGACCAAGTTGAACGCTGACGAGAAGGCTTCTGAGAAGTATTATGAGGACACTGTTCCTGGCACTTCTAATTTCATCTCTGA

GGTCTTCTTTCTCACATTGGCTGCTCATCATTATGGTAGTGAAGCTCTTAATGCCACGCATAAGAGTCTGGAGAAAGACATCAAATATATTCAA
AAGCAATTGACTGCCGTTGAAGCA

>Bcin01g07220(MLST1), partial sequence [organism=Botrytis cinerea, strain D08_H_8_3]
ATGGCATATCTTGTTTCTTTTCGCATTATGCTTTGGAACGTGCTGGACAAGCTTGGGTTAGTGCTCTGCGTGTAGAAGCACTAAAGAAGATTCT
CGCACAACCGAAGTCATGGTTTGAGGAATCCAGGAATTCACCTGGCCGGCTGAACGAAGTTTTGGATAGGAACTCTGAGGAAATGCGTAATCTC
GTTGGCCGCTTTGCTGGTATTGTATTCACAGCATTTTTTATGCTATTGATATCAATCATTTGGGCTTTTGTGAATACATGGAAACTGACATTAG
TCTCGATGGCAACTGGGCCAGTTATATACGCTGTCACCAAAACATTCAATCGCGTGAGTGGAAAATGGGAAAACAAGTGCAACTACGCCTCTGA
AATGACCACTGGCATATTTTCAGAGACTTTCTCCAACATCAAAGTGGTTCGGGCTTTTACTCTGGAAACTTACTTTGAGACAAAACACACCAAA
GCTACAGAAGAACTTTATAAAGTTGGACTAATACGAGCAAACTACTCGGGATTGCTGTGGGGATTGACAGATGCGATGTCATTCTTCATCACTG
CAACTATCTTTTATTATGCCACGGTTCTCATTACCAAGAGAGAGATCAGTATCGCGACTGCACTACAGACTGTCAATCTTCTATTATTTGGTAT
TTCTAATAGTACGAATATGCTGGCCATGATACCACAAATCAACTCTTCTCGCGTTACAGCTACGCATATGCTTGCATTAGCCAATCTCGATTCA
TCTTCCTCCCACGAAAATAAAGGAACCGAACGGCTTTCGACAATCTTTCCAATCAAATTCAACCGTCTTTCATTCACATACCCTACTCGTCCTG
AAAAACGAACGATATCATCCTTTTCTCTTTCCCTGATTCCTAACTCAACAACTGCACTTGTCGGACCCTCCGGCTCCGGAAAATCTACAATAGC
TGCTCTGCTCATTGGTCTCTATCCGCCAGATACTTCAACACCTCCACCGTTGACATTCAATCGCGTCTCCATAAGTAACTGTCACATTCCGTCT
CTCCGGGCTTCTCTCTCACTCGTTCCACAAATACCGATTCTATTTCCAGCTACCATTCTCCATAACATCATTTATGGTCTCCCAGAATCTTCTC
CTTGTGCTGGTCTTCCATCTGCTATTGCATCAGCAAAAGATGCTGGGATCCATGAATTTATCACATCGCTTCCACAAGGTTATGATACTAT

>Bcin05g07690(MLST2), partial sequence [organism=Botrytis cinerea, strain D08_H_8_3]
ACCACTATCACCAAGTCCTTTTCTGCACTTTCCTCATCAAAAACGAACGAGAACATCATCAATAAATACTTTGCTGAAGTAATTGGGTTTTATT
TTGGCCAGGATGCCTTCTCTCTACGACAGCAAGCCTTCGATGATATGTTGTGGGTAGTTCTTGGCTGGCTGGATACTGTCAAATTCATTGATTT
ACATTCTGAATTGCACTATTCAAACGACTCTCAGCCAGAATGGTACGGACAACAATATAAACCTGCATTTGCACATCGAGCGCGACTATTTTGG
GAATTGGCTTCACAAGGATGGGATACTACTCTCTGTGGTGGTGGGATGATATGGTCACCATACCTTACTCCATACAAGAACGCAATTACCAATG
AACTCTATATCGCAGCTTCGATATCGATGTACCTATATTTCCCCGGAGATGACAATCAATCCCCATTTATGCTTTCCAACCCTTCATATCCACC
TCACGATCCGAAATATCTACAGGCAGCTGTTGATGCTTACAAATGGCTGAATGGTTCCAACATGACGGATTTACAAGGATTATATGTCGACGGG
TACCATATCTCGAATCTTTCTGGCGGTGAAAACACCCATTGCGATTCTAGAAATGAGATGGTATATACCTACAATCAAGGTGTTTTGCTTACTG
GACAACGTGGTTTGTATGACGCAACCGCCGCACGATCATACCTTGTAGATGGCCACAAACTCATCGCGAATGTTATTAATGCCACAGGCTATGA
CCTGAAACACAATGTTGTCATCTCACCGCCACCCAAAGATGGTTCCGCATTGGCAAAGTGGTTTGGCCTGGGTAGGAATGGAATACTGGAAGAA
GGATGCGATTCAAGTGCTTCGTGTTCTCAAAATGGACAAACTTTCAAAGGCATATTCTTTCATCACTTGATTGCGTTCTGTAGTGATTTGCCAG
GGGAGCCTATTGCAGGGACGAAGGAAAGCTTAGAACTCGACAGAGTGTGGCATTCTGACAAATGCTCACAGTATACAAAAT

>Bcin06g01710(MLST3), partial sequence [organism=Botrytis cinerea, strain D08_H_8_3]
GGTGAGTCTGACTTTTGTATTTGAGCGTTAAGATAGACACTGATATACCAAGGCAATCACCTAATTTCATGGCGTTCTTCCCTGCATCATCTAC
CTACCCTGGAATGCTGGGAGAATTATACTCAGCAGCTTTCACAGCACCTGCTTTCAATTGGATCTGTTCCCCTGCTGTGACAGAATTGGAGACG
GTTGTAATGGATTGGCTGGCCAAGCTTCTCAATCTCCCAGACTGTTATTTGTCTTCGACTCATGGTGGTGGTGTCATCCAAGGATCAGCCTCGG
AAGCTATCGTTACCGTTATGGTTGCTGCCCGCGATAAATATCTTCGTGAAACTACTGAAGGTCTGTCGGGCATTGAACTCGAGGATGCGATTGC
ATATAAGAGGAGTAAGCTAGTTGCACTAGGAAGCGAAATGGCACACAGCTCCACGCAGAAAGCAGCGCAAATAGCTGGCGTTAGATTCCGATCG
ATTCCAGTACTCGCATCCAATGATTTCGCCATGACGGGTGATGATTTAGAGAAGGTATTGAAAGAATGCAAATCTCAAGGATTGGAACCCTTCT
ATCTAACTTCGACTTTGGGAACAACATCTACATGCGCAGTTGACGACTTCGCATCTATTGCAACAGTACTTTCAAAATATGCACCTCCAGATGT
TGCAGGCGAGATCTGGGTTCACGTCGATGCTGCTTATGCAGGTGCAGCTTTGGTTTGCCCTGAATACCATCATCTAACATCGTCTTTCCAGCAT
TTCCATTCCTTCGATATGAACATGCACAAATGGCTTCTGACAAATTTCGACGCTTCTTGTCTATATGTCAAGAAACGCAAAGATCTGATCGATG
CACTGTCCATAACACCAAGTTATCTTCGCAACGAGTTTTCAGAGAGTGGACTCGTAACCGACTATCGGGACTGGCAAATTCCTCTCGGAAGACG
CTTCCGAAGCTTAAAGATTTGGTTTGTCCTCAGAACCTAC

>Bcin09g03030(MLST4), partial sequence [organism=Botrytis cinerea, strain D08_H_8_3]
ACCCTTCAACCCCACCGCAGCTCCTATACCGAAAGCGAGTATCCTACCAATTCTCCTTCCACCTGCGACTTTAAGACCATTGGCTTTCCGCACT
TTCACAAAAAAGCATAGTTTAACATTGACGTCGTCGGCATTACAAGTGTTGGCTACTTTTATTGGAAAGTATTGTGGGACAGGATGGAGGGAAG
AAGGACTGGCAGAGAGAGTCTTGGAGGAGGTCGCCAAGAGTTGGAAGAATAGGAGTGGCGGTGTCATTGTCGAGGGCGAGGGAACGGAATTGAA
GGAGATTCTGAAAGCTTTGGAAGGGAATATGAGAGGTGGAAGGATAGTCATAGGAAGAGAGCTAAGCCGGCAGAATAGTTTAGTACTGGGATCA
TCACAATATGGAGAGGTCAATCATACAAGACTTGGGCTACGGCCAGGGAATATACCCAGAGAGGATAGTCAGTCAAGTTTGGGAATGTCAACGT
TGGAGGTCAATGACGAAGAAGATGAGGATGGCCTGATGGATCCAAGAAGGTGGTTAAAAGTCATTGATGCATTTGAGCAACCTCGACTGGTGTA
CAATGTTGCTAAAAAGCACTTTGATAGGTATGTTTCAATGATAAAATTTTATCTGAATCGACTAACTCAGTACAGAGATGCCTCCAAACCTTCA
TTGTTCCCACCTGCGTCTCATAAAACACTCCTCTTCCAAAACCGCTATAATGTTATCCATCAACGTCTCCTTCGCAATGAATCTTTTCAAACGC
CCGCTTTTCAAGGTGGCAAATCTTCCCTTCAACGCAGCACGTCCGCCATTACCACACAACAACAATCATACAAATTAACGCCGATAGCTAATCT
TCTCGGTCGCAATCGCAGCTCTCATATGCTTCTCGGTCTCCTCAGTATTTCACCCACTGGTACCCTCGCCATCAATGACCTGACGGGCAGTATC
GCTCTCGATCTTACACACGCAGCAGCCATTC

```
>Bcin11g01310(MLST5), partial sequence [organism=Botrytis cinerea, strain D08_H_8_3]
TGGGCTGACATGGACCTCATGTGGAACCGGCGTAGAATGCGCAACGCTTGAAGTTCCGCTCGAATATGGCGATGCAACGTCAACGGCAAGAGCC
AGTGTTGCGCTTACTCGTTATCCTGCCACTGTTGCCGCGAGCAAGAAGCTCGGGTCTCTCTTGATAAACCCCGGTGGGCCCGGTGCCTCTGGTG
TTGGCTTTGTGCAGTCTGGAGCCGGTGCCGCCGTCTCGACACTGAGTGGTGGATTGTACGATATCATCGGATGGGATCCACGTGGAACCGGTGC
TTCGGCTCCTATTTTGGAATGTTTTGCAAATGCCAGTGCGGAGTATGATTTTAACAACGCGTTTCCATCTGCTCCGAATCTCTGGCTCGGACAA
TTTGCGAATGCCAGCGCAAATTCTGCTGTTAGCTCTGCTATCACATCCTTTGACACTTCTGTCGCTGCTCTTGCAAAAGCTTGCGTGGCTCAGA
AATCTCCCGCTCTTTACACCTCAACAGCAGCATATGTTGCTCGAGACGTGGCAGCGATAGTCGATGCATTGGATGGGACCTCTGCAAAACTTAA
CTACTGGGGTTTCTCATATGGAACTATCTTCCTAGCTGAGTTTATCCAAACTTTCCCAGGCCGCGTGGGAAGAGTTCTTGCCGATGGTGTTTTC
GACGCAAAGGCAAATGCACTCACATACGTTAGCCAACTTCCCAACGATCAACTCAGTGTTCGTGCTTCGTTGAACGATTTTGCAGCTTTCTGCA
CCACCGCCGGTAGTAAAGGTTGCTCTTTTGCCACCGCCCCTACTGGAACCACAGGTACTGTTGCTACCAGACTGGACAACATAATGAAGGATAT
GTTCCTCAATCCTATTGTTGCTTCGGGCTTAAGCATCAG

>Bcin15g03910(MLST6), partial sequence [organism=Botrytis cinerea, strain D08_H_8_3]
GGTGAGTCTGACTTTTGTATTTGAGCGTTAAGATAGACACTGATATACCAAGGCAATCACCTAATTTCATGGCGTTCTTCCCTGCATCATCTAC
CTACCCTGGAATGCTGGGAGAATTATACTCAGCAGCTTTCACAGCACCTGCTTTCAATTGGATCTGTTCCCCTGCTGTGACAGAATTGGAGACG
GTTGTAATGGATTGGCTGGCCAAGCTTCTCAATCTCCCAGACTGTTATTTGTCTTCGACTCATGGTGGTGGTGTCATCCAAGGATCAGCCTCGG
AAGCTATCGTTACCGTTATGGTTGCTGCCCGCGATAAATATCTTCGTGAAACTACTGAAGGTCTGTCGGGCATTGAACTCGAGGATGCGATTGC
ATATAAGAGGAGTAAGCTAGTTGCACTAGGAAGCGAAATGGCACACAGCTCCACGCAGAAAGCAGCGCAAATAGCTGGCGTTAGATTCCGATCG
ATTCCAGTACTCGCATCCAATGATTTCGCCATGACGGGTGATGATTTAGAGAAGGTATTGAAAGAATGCAAATCTCAAGGATTGGAACCCTTCT
ATCTAACTTCGACTTTGGGAACAACATCTACATGCGCAGTTGACGACTTCGCATCTATTGCAACAGTACTTTCAAAATATGCACCTCCAGATGT
TGCAGGCGAGATCTGGGTTCACGTCGATGCTGCTTATGCAGGTGCAGCTTTGGTTTGCCCTGAATACCATCATCTAACATCGTCTTTCCAGCAT
TTCCATTCCTTCGATATGAACATGCACAAATGGCTTCTGACAAATTTCGACGCTTCTTGTCTATATGTCAAGAAACGCAAAGATCTGATCGATG
CACTGTCCATAACACCAAGTTATCTTCGCAACGAGTTTTCAGAGAGTGGACTCGTAACCGACTATCGGGACTGGCAAATTCCTCTCGGAAGACG
CTTCCGAAGCTTAAAGATTTGGTTTGTCCTCAGAACCTAC

>Bcin16g03460(MLST7), partial sequence [organism=Botrytis cinerea, strain D08_H_8_3]
ATGAACCTCTTGATTTGAACTCTTCATTAATTGCAAGTGATGAACCCCTATTCTGCCCAGAGGATAGTTACAAGACGTATATCATTAGTCGAGA
ACCACTCATGATATATATTGACGGATTTTTGAAAGCGAATGAAAGTAAACATTTGGTTGATGTTAGGTGTGTTATTTATTCTGATGAAATGAAC
AAGAGAGACTGATGAGATAGTGAGCCGCTTTATGAACCGTCAACTGTTTCTCACGGACAGGAAGTTACCATTGATCCTTCAGTTCGGAATTCTG
AAGTGGCGGTTTTAGAGAGGGATGAGGTGGTCAGGTGTATTGAGCATAGAGCGAGGGCATTTCAGGGGTGGAGGGGCGAGATGGGGATTGAGAA
GTTGAGGACGCAGAGGTATGGCGTTGGAGGACATTATGGGATGCATTTGTAAGTTTTGGGGGATTGACGAAGGCTTCTGTCTATTTTCTACCAG
TACGGATCTTGAAAGAAAGATAGCATGAACACGAGGGCTAATAACTAGGAAATAGCGATTGGAGCGGAGGTAAACGTGGCATAGACCGATTTAG
TACTTTCATGGTCTATGTCGACGTATCCTCTGATATCGAAGGTGGAGGAACGGAATTCCCACGTATTGTGGACCAAAAGGAGGAAGGTGGGAG
GACTTCCTGGAAACTACGGAAGCATTGGATCCAAGAACTGGAGAAAATGTAACAGTAGAAGGGGTGACATTCAAACCAATCAAGGGAAATGCCG
TATTCTGGGAAAATACTGACAACAACGGGAGGGGCTATGAT

>Bcin12g03020(MLST8), partial sequence [organism=Botrytis cinerea, strain D08_H_8_3]
CGATTGGCTGCGAAGAAAACTGCGCAGCACCGAGTCACCAAAAGCACGAATAAACCATCGGCAAGGGAAATATTCTCTGAACCACAACATGACA
CGAGCGCGGAAGAGTATATCGGGCGAGAAGCTTCATCAAGAGCACCAAAGCGACAACGAGTCGATGATAATTATAACTCTTACGGTGGAAGAAA
TGAAAATACAGCAGCTTATGCTTCCGGGAAACTTCCATCTGGAAGTATAAATGTTGGTGGAGGTAGGAAGACCACTTTTCAAGAAGAACCACGA
ACGGCATTTGTCGCTGGCAAGTTGCCCCCTGGAAGTATCAACATTGGTGGAAAGAAGGCTACTCAAATCGAGGACGAGGGTAGAGCAGCTTATG
CTTCCGGAAAATTGCCTCCAGGAAGTATAAACTCTGGCGCAAAGAAGGCGATTTCATTCCAAGATGAAACGAGACCGGCTTATGTCTCCGGAAA
GCTTCCACATGGTAGTATCGACGGTATGCGAAACCGTGAAATGGCTGCCGTCCACCGCGAAATTGCTGAGGGTGGGAGGAAACCAGGCCAGGTT
GTCTCTTCTCTATTCACATTCAATCCTACTTCAAAGAAAACTTTTGATGAACCCGAAGAACAAGCGGAACCGGCAAAACCATCCAATGCGCCTT
TGACAGAGGAAATGGCGACTTTCACCAATCTAGGGTTATCGAGAAGGCTTGCAGCCCATCTATCGACTAAACTCGATATGAAAGCTCCGACCGC
CATTCAAAAAGCATCTGTACAGCAGTTGGTATCGGACGATAGCGATGCTTTCATACAAGCAGAGACTGGATCTGGAAAAACTTTGGCATATCTA
CTACCTATAGTCGAGCGAATATTAGCATTGAGTGAGAATGGCGTACAAA

>Bcin02g07770(MLST9), partial sequence [organism=Botrytis cinerea, strain D08_H_8_3]
CAGCTTTCCCTTTCGGTCTTGGCATCTACAGTCATTGCCATCCCTACACCATCACAACTTGAGTCTCGGGCCGTTATCGATTCCGATGCCGTTG
TAGGATTTGCCGAAACTGTTCCCAGTGGGACCGTAGGAACAGTTTATGAGGCATATAAACCATTCCTTAAAGTCGTAAATGGATGCGTACCATT
CCCTGCCGTCGATGCATCGGGTAACACAGGGTATGTCCTTATATCTTTCTCTTCCACACGATTGCTATTGAGTCTCTAACATATTTTAGTGGTG
GTTTGTCACCAACTGGCAGTAGCAATGGTGGTTGCAGCAGCAGTACCGGTCAAGTATATGTTCGAGGAGGACAAAGCGGATCAAACTACGCCAT
CATGTACTCCTGGTAAGTTCTCTCTAAACTTCTCCTTATAGATCCAACCTAACAAAATCTTAGGTACATGCCAAAGGACGAGCCCTCAACCGGT
ATTGGTCACCGTCACGATTGGGAAGGTGTAATTGTCTGGCTCTCCAGCGCCACCGCCACAACTGCCGACAACATCTTAGCCGTTTGTCCTTCCG
CCCACGGAGGCTGGGATTGTTCCACGGATGGCTATTCCCTTTCTGGTACCAGCCCTCTTATCAAGTACGAAAGTATCTGGCCCGTCGATCACTC
AATGGGTCTTACTAGTACTGTTGGTGGAAAACAACCTATGATTGCTTGGGAGTCTTTACCAACTGCTGCTCAAACTGCTCTTGAGAACACCGAT
```

TTCGGTGCTGCGAATGTTCCATTCATTCCGGCTGTTTTCACAGACAATCT

>Bcin04g02090(MLST10), partial sequence [organism=Botrytis cinerea, strain D08_H_8_3]
CGGAGGATGATATGGCAAAGTCTATGATTACCAAAGCATTTGTAGGCATGAGTAGTAAACTGTCGAATATGACTATGAATGATGTTTACAAGCC
CTACATCCATGTAAGAAATGTAGAATAGAGAGATCAGTAACTGGAACTAATATCGTTTGTAGGCTTTCAAGTTACTTACGCAGTTCAACCCAAT
CACTACAGCTATTGCCGAATCCCCACTGTTTCAAGTGGCTGTCTCAGCAAATACCATCGAAAAGTACACACTGCTAGGCCCTTTCTTCAGAATA
TCTCCTCTGCAACAGGAAGTTACCAGGGAATACTTCAGTGCGCCAAAGACGATAGATAGGCGACACATTGCCACATCTCAAGATGCGTTACGAT
TGACCTTACAAACCCATCAAAAAGATTTACTTGATATCATCAACCACTTTGTTCGAGCAAGTCCAATCGCAAAAAGCAAAACCCTGGATTGGTT
CGTCTACATTGTGAATCAAAATCACAAGCGTCGAGCACTTCAGGTAGACCCGAAAGAAGTGTCTTCTGATGGCTTTATGCACAATGTCACTGTC
GTTCTAGATGGTCTTTGTGAGCCATTCATGGATACCACATTCTCGAAAATTTCGAAGATTGATATTGATTATCTAAGACGTGCGCCTCGTGTAG
ATATCAAGGACGAGACCAAGTTGAACGCTGACGAGAAGGCTTCTGAGAAGTATTATGAGGACACTGTTCCTGGCACTTCTAATTTCATCTCTGA
GGTCTTCTTCCTCACATTGGCTGCTCATCATTATGGTAGTGAAGCTCTTAATGCCACGCATAAGAGTCTGGAGAAAGACATCAAATATATTCAA
AAGCAATTGACTGCCGTTGAAGCA

>Bcin01g07220(MLST1), partial sequence [organism=Botrytis cinerea, strain D09_K_2_3]
ATGGCATATCTTGTTTCTTTTCGCATTATGCTTTGGAACGTGCTGGACAAGCTTGGGTTAGTGCTCTGCGTGTAGAAGCACTAAAGAAGATTCT
CGCACAACCGAAGTCATGGTTTGAGGAATCCAGGAATTCACCTGGCCGGTTGAACGAAGTTTTGGATAGGAACTCTGAGGAAATGCGTAATCTT
GTTGGCCGCTTTGCTGGTATTGTATTCACAGCATTTTTCATGCTATTGATATCAATCATTTGGGCTTTCGTGAATACATGGAAACTGACATTAG
TCTCGATGGCAACTGGGCCAGTTATATACGCTGTCACCAAAACGTTCAATCGCGTGAGTGAAAAATGGGAAAACAAGTGCAACTACGCCTCTGA
AATGACCACTGGCATATTTTCAGAGACTTTCTCCAACATCAAAGTGGTTCGGGCTTTTACTCTGGAAACTTACTTTGAGACAAAACACACCAAA
GCTACAGAAGAACTTTATAAAGTTGGACTAATACGAGCAAACTACTCGGGATTGCTGTGGGGATTGACAGATGCGATGTCATTCTTCATCACTG
CAACTATCTTTTATTATGCCACGGTTCTCATTACCAAGAGAGAGATCAGTATCGCGACTGCACTACAGACTGTCAATCTTCTATTATTTGGTAT
TTCTAATAGTACGAATATGCTGGCCATGATACCACAAATCAACTCTTCTCGCGTTACAGCTACGCATATGCTTGCATTAGCCAATCTCGATTCA
TCTTCCTCCCACGAAAATAAAGGAACCGAACGGCTTTCGACAATCTTTCCAATCAAATTCAACCGTCTTTCATTCACATACCCTACTCGTCCCG
AAAAACGAACGATATCATCCTTTTCTCTTTCCCTGATTCCTAACTCAACAACTGCACTTGTCGGACCCTCCGGCTCCGGAAAATCTACAATAGC
TGCTCTGCTCATTGGTCTCTATCCGCCAGATACTTCAACACCTTCACCGTTGACATTCAATCGCGTCTCCATAAGTAACTGTCACATTCCGTCT
CTCCGGGCTTCTCTCTCACTCGTTCCACAAATACCGATTCTATTTCCAGCTACCATTCTCCATAACATCATTTATGGTCTCCCAGAATCTTCTC
CTTGTGCTAGTCTTCCATCTGCTATTGCATCAGCAAAAGATGCTGGGATCCATGAATTTATCACATCGCTTCCACAAGGTTATGATACTAT

>Bcin05g07690(MLST2), partial sequence [organism=Botrytis cinerea, strain D09_K_2_3]
TCACCACTATCACCAAGTCCTTTTCTGCACTTTCCTCATCAAAAACGAACGAGAACATCATCAATAAATACTTTGCTGAAGTAATTGGGTTTTA
TTTTGGCCAGGATGCCTTCTCTCTACGACAGCAAGCCTTCGATGATATGTTGTGGGTAGTTCTTGGCTGGCTGGATACTGTCAAATTTATTGAT
TTACATTCTGAATTGCACTATTCAAACGACTCTCAGCCAGAATGGTACGGACAACAATATAAACCTGCATTTGCACATCGAGCGCGACTATTTT
GGGAATTGGCTTCACAAGGATGGGATACTACTCTCTGTGGTGGTGGGATGATATGGTCACCATACCTTACTCCATACAAGAACGCAATTACCAA
TGAACTCTATATCGCAGCTTCGATATCGATGTACCTATATTTCCCCGGAGATGACAATCAATCCCCATTTATGCTTTCCAACCCTTCATATCCA
CCTCACGATCCGAAATATCTACAGGCAGCTGTTGATGCTTACAAATGGCTGAATGGTTCCAACATGACGGATTTACAAGGATTATATGTCGACG
GGTACCATATCTCGAATCTTTCTGGCGGTGAAAACACCCATTGCGATTCTAGAAATGAGATGGTATATACCTACAATCAAGGTGTTTTGCTTAC
CGGACAACGTGGTTTGTATGACGCAACCGCCGCACGATCATACCTTGTAGATGGCCACAAACTCATCGCGAATGTTATTAATGCCACAGGCTAT
GACCTGAAACACAATGTTGTCATCTCACCGCCACCCAAAGATGGTTCCGCATTGGCAAAGTGGTTTGGCCTGGGTAGGAATGGAATACTGGAAG
AAGGATGCGATTCAAGTGCTTCGTGTTCTCAAAATGGACAAACTTTCAAAGGCATATTCTTTCATCACTTGATTGCGTTCTGTAGTGATTTGCC
AGGGGAGCCTATTGCAGGGACGAAGGAAAGCTTAGAACTCGACAGAGTGTGGCATTCTGACAAATGCTCACAGTATACAAAAT

>Bcin06g01710(MLST3), partial sequence [organism=Botrytis cinerea, strain D09_K_2_3]
GTGAGTCTGACTTTTGTATTTGAGCGTTAAGATAGACACTGATATACCAAGGCAATCACCTAATTTCATGGCGTTCTTCCCTGCATCATCTACC
TACCCTGGAATGCTGGGAGAATTATACTCAGCAGCTTTCACAGCACCTGCTTTCAATTGGATCTGTTCCCCTGCTGTGACAGAATTGGAGACGG
TTGTAATGGATTGGCTGGCCAAGCTTCTCAATCTCCCAGACTGTTATTTGTCTTCGACTCATGGTGGTGGTGTCATCCAAGGATCAGCCTCGGA
AGCTATCGTTACCGTTATGGTTGCTGCCCGCGATAAATATCTTCGTGAAACTACTGAAGGTCTGTCGGGCATTGAACTCGAGGATGCGATTGCA
TATAAGAGGAGTAAGCTAGTTGCACTAGGAAGCGAAATGGCACACAGCTCCACGCAGAAAGCAGCGCAAATAGCTGGCGTTAGATTCCGATCGA
TTCCAGTACTCGCATCCAATGATTTCGCCATGACGGGTGATGATTTAGAGAAGGTATTGAAAGAATGCAAATCTCAAGGATTGGAACCCTTCTA
TCTAACTTCGACTTTGGGAACAACATCTACATGCGCAGTTGACGACTTCGCATCTATTGCAACAGTACTTTCAAAATATGCACCTCCAGATGTT
GCAGGCGAGATCTGGGTTCACGTCGATGCTGCTTATGCAGGTGCAGCTTTGGTTTGCCCTGAATACCATCATCTAACATCGTCTTTCCAGCATT
TCCATTCCTTCGATATGAACATGCACAAATGGCTTCTGACAAATTTCGACGCTTCTTGTCTATATGTCAAGAAACGCAAAGATCTGATCGATGC
ACTGTCCATAACACCAAGTTATCTTCGCAACGAGTTTTCAGAGAGTGGACTCGTAACCGACTATCGGGACTGGCAAATTCCTCTCGGAAGACGC
TTCCGAAGCTTAAAGATTTGGTTTGTCCTCAGAACCTAC

>Bcin09g03030(MLST4), partial sequence [organism=Botrytis cinerea, strain D09_K_2_3]
GACCCTTCAACCCCACCGCAGCTCCTATACCGAAAGCGAGTATCCTACCAATTCTCCTTCCACCTGCGACTTTAAGACCATTGGCTTTCCGCAC

TTTCACAAAAAAGCATAGTTTAACATTGACGTCGTCGGCATTACAAGTGTTGGCTACTTTTATTGGAAAGCATTGTGGGACAGGATGGAGGGAA
GAAGGACTGGCAGAGAGAGTCTTGGAGGAGGTCGCCAAGAGTTGGAAGAATAGGAGTGGCGGTGTCATTGTCGAGGGCGAGGGAACGGAATTGA
AGGAGATTCTGAAAGCTTTGGAAGGGAATATGAGTGGTGGAAGGATAGTCATAGGAAGAGAGCTAAGCCGACAGAATAGTTTAGTACTGGGATC
ATCACAATATGGAGAGGTCAATCATACAAGACTTGGGCTACGGCCAGGGAATATACCCAGAGAGGATAGTCAGTCAAGTTTGGGAATGTCAACG
TTGGAGGTCAATGACGAGGAAGATGAGGATGGCCTGATGGATCCAAGAAGGTGGTCAAAAGTCATTGATGCATTTGAGCAACCTCGACTGGTGT
ACAATGTTGCTAAAAAGCACTTTGATAGGTATGTTTCAATGATAAAATTTTATCTGAATCGACTAACACAGTACAGAGATACCTCCAAACCTTC
ATTGTTCCCACCTGCGTCTCATAAAACACTCCTCTTCCAAAACCGCTATAATGTTATCCATCAACGTCTCCTTCGCAATGAATCTTTTCAAACG
CCCGCTTTTCAAGGTGGCAAATCTTCCCTTCAACGCAGCACGTCCGCCATTACCACACAACAACAATCATACAAATTAACGCCGATAGCTAATC
TTCTCGGTCGCAATCGCAGCTCTCATATGCTTCTCGGTCTCCTCAGTATTTCACCCACTGGTACCCTCGCCATCAATGACCTGACGGGCAGTAT
CGCTCTCGATCTTACACACGCAGCAGCCATTC

>Bcin11g01310(MLST5), partial sequence [organism=Botrytis cinerea, strain D09_K_2_3]
ACTGACATGGACCTCATGTGGAACCGGCGTAGAATGCGCAACGCTTGAAGTTCCGCTCGAATATGGCGATGCAACGTCAACGGCAAAAGCCAGT
GTTGCGCTTGCTCGTTATCCTGCCACTGTTGCCGCGAGCAAGAAGCTCGGGTCTCTCTTGATAAACCCCGGTGGACCCGGTGCCTCTGGTGTTG
GCTTTGTGCAGTCTGGAGCCGGTGCCGCCGTCTCGACACTGAGTGGTGGATTGTACGATATCATCGGATGGGATCCACGTGGAACCGGTGCTTC
GGCTCCTATTTTGGAATGTTTTGCAAATGCCAGTGCGGAGTATGATTTTAACAACGCGTTTCCATCTGCTCCGAATCTCTGGCTCGGACAATTT
GCGAATGCCAGCGCAAATTCTGCTGTTAGCTCTGCTATCACATCCTTTGACACTTCTGTCGCTGCTCTTGCAAAAGCTTGCGTGGCTCAGAAAT
CTCCCGCTCTTTACACCTCAACAGCAGCATATGTTGCTCGAGACATGGCAGCGATAGTCGATGCATTGGATGGGACCTCTGCAAAACTTAACTA
CTGGGGTTTCTCATATGGAACTATCTTCCTAGCTGAGTTTATCCAAACTTTCCCAGGCCGCGTGGGAAGAGTTCTTGCCGATGGTGTTTTCGAC
GCAAAGGCAAATGCACTCACATACGTTAGCCAACTTCCCAACGATCAACTCAGTGTTCGTGCTTCGTTGAACGATTTTGCAGCTTTCTGCACCA
CCGCCGGTAGTAAAGGTTGCTCTTTTGCCACCGCCCCTACTGGAACCACAGGTACTGTTGCTACCAGACTGTACAACATAATGAAGGATATGTT
CCTCAATCCTATTGTTGCTTCGGGCTTAAGCATCAG

>Bcin15g03910(MLST6), partial sequence [organism=Botrytis cinerea, strain D09_K_2_3]
GCCAAAACACAAAATCATCCAACGATGAAGATGATACTCCACTTCCCTTGATTATTTGGCATGGACTCGGCGATAATTACAAAGCGGATGGTCT
TGCGCAAGTTGGAAAACTAGCTGAAGCTATTCATCCTGGGACTTTTGTCTACAATATTCATGTAGATGAGGATGCATCTGCAGATAGGACAGCT
ACCTTCTTTGGAAATCTCACTCGTGAGTACATCCCCTATTTTCCCTTTAAATACCATACTAACTCTCTTACCAAGTTCAAATCGAAAAGGTCTG
CGAAGACCTCGCCTCCCATCCTATTCTCTCTACCGCGCCCGCCGTCGACGCAATTGGATTCTCCCAAGGCGGCCAATTCTTGCGCGGTTACATA
TCCCGCTGCAATGCTCCACCCATCCGCTCTCTCCTGACCTTCGGTTCCCAACACAACGGCATTTCTGCCTTCCAAGCCTGTGGTCCTGCCGATT
TCCTCTGTCGCGGTGCTCAAACCCTTTTGCGATCCAACACCTGGTCAACCTTTGTCCAATCTCGTCTCGTACCCGCTCAATACTTCAGAGATCC
GGAAAACCTAGACTCTTACCTTGAATATTCCAATTTCCTTGCCGACATCAATAATGAGCGCGTTCTCAAGAACCAAACATATAAATCCAACATG
GAAAAATTGGAACGATTCGTAATGTATGTCTTTGAAGACGATACAACTGTCATCCCTAAGGAAAGTGGATGGTGGGCTGAAGTCAACGGCACGG
AAGTTACACCACTGAAAGAAAGAGCCATTTATAAAGAAGATTGGCTAGGTTTAAAGACATTGGATGAGGCCGGAAAATTAGTTTTCGAAACCAT
TCCAGGGGGACATATGACGTTAGGAGAGGAGATGCTAGAGAAGGCTTTCAAAGAGTATTTTGGTCCAGCAGGGAAGAAATTTGGG

>Bcin16g03460(MLST7), partial sequence [organism=Botrytis cinerea, strain D09_K_2_3]
CATGAACCTCTTGATTTGAACTCTTCATTAATTGCAAGTGATGAACCCCTATTCTGCCCAGAGGATAGTTACAAGACGTATATCATTAGTCGAG
AACCACTCATGATATATATTGACGGGTTTTTGAAAGCGAATGAAAGTAAACATTTGGTTGATGTTAGGTGTGTTATTTATTCTGATGAAATGAA
CAAGAGAGACTGATGAGATAGTGAGCCGCTTTATGAACCGTCAACTGTTTCTCACGGACAGGAAGTTACCATTGATCCTTCAGTTCGGAATTCT
GAAGTGGCGGTTTTAGAGAGGGATGAGGTGGTCAGGTGTATTGAGCATAGAGCGAGGGCATTTCAGGGGTGGAGGGGCGAGATGGGATTGAGAA
GTTGAGGACGCAGAGGTATGGGGTTGGAGGACATTATGGGATGCATTTGTAAGTTTTGGGGGATTGACGAAGGCTTCTGTCTATTTTCTATCAG
CACGGATCTTGAAAGAAAGATAGCATGAACACGCGGGCTAATAACTAGGAAATAGCGATTGGAGCGGAGGTAAACGTGGCATAGACCGATTTTA
GTACTTTCATGGTCTATGTCGACGTATCTCTGATATCGAAGGTGGAGGAACGGAATTCCCACGTATCGTGGGACCAAAAGGAGGAAGGTGGGAG
GACTTCCTGGAAACTACGGAAGCATTGGATCCAAGAACTGGAGAAAATGTAACAGTGAAGGGTGACATTCAAACCAATCAAGGGAAATGCCGTA
TTCTGGGAAAATACTGACAACAACGGGAGGGGCTATGAT

>Bcin12g03020(MLST8), partial sequence [organism=Botrytis cinerea, strain D09_K_2_3]
CGATTGGCTGCGAAGAAAACTGCGCAGCACCGAGTCACCAAAAGCACGAATAAACCATCGGCAAGGGAAATATTCTCTGAACCACAACATGACA
CGAGCGCGGAAGAGTATATCGGGCGAGAAGCTTCATCAAGAGCACCAAAGCGACAACGAGTCGATGATAATTATAACTCTTACGGTGGAAGAAA
TGAAAATACAGCAGCTTATGCTTCCGGGAAACTTCCATCTGGAAGTATAAATGTTGGTGGAGGTAGGAAGACCACTTTTCAAGAAGAACCTCGA
ACGGCATTTGTCGCTGGCAAGTTGCCCCCTGGAAGTATCAACATTGGTGGAAAGAAGGCTACCCAAATCGAGGACGAGGGTAGAGCAGCTTATG
CTTCCGGAAAATTGCCCCCAGGAAGTATAAACTCTGGCGCAAAGAAGGCGATTTCATTCCAAGATGAAACGAGACCGGCTTATGTCTCCGGAAA
GCTTCCACATGGTAGTATCGACGGTATGCGAAACCGTGAAATGGCTGCCGTCCACCGCGAAATTGCTGAGGGTGGGAGGAAACCAGGCCAGGTT
GTCTCTTCTCTATTCACATTCAATCCTACTTCAAAGAAAACTTTCGATGAACCAGAAGAACAAGCGGAACCGGCAAAACCATCCAATGCGCCTT
TGACAGAGGAAATGGCGACTTTCACCAATCTAGGGCTATCGAGAAGGCTTGCAGCCCATCTATCGACTAAACTCGATATGAAAGCCCCGACGGC
CATTCAAAAAGCATCTGTGCAGCAGTTGGTATCGGACGATAGCGATGCTTTCATACAAGCAGAGACTGGATCTGGAAAAACTTTGGCATATCTA
CTACCTATAGTCGAGCGAATATTAGCATTGAGTGAGAATGGCGTACAAATA

>Bcin02g07770(MLST9), partial sequence [organism=Botrytis cinerea, strain D09_K_2_3]
CAGCTTTCCCTTTCGGTCTTGGCATCTACAGTCATTGCCATCCCTACACCATCACAACTTGAGTCTCGGGCCGTTATCGATTCCGATGCCGTTG
TAGGATTTGCCGAAACTGTTCCCAGTGGGACCGTAGGAACAGTTTATGAGGCATATAAACCATTCCTTAAAGTCGTAAATGGATGCGTACCATT
CCCTGCCGTCGATGCATCGGGTAACACAGGGTATGTCCTTATATCTTTCTCTTCCACACGATTGCTATTGAGTCTCTAACATATTTTAGTGGTG
GTTTGTCACCAACTGGCAGTAGCAATGGTGGTTGCAGCAGCAGTACCGGTCAAGTATATGTTCGAGGAGGACAAAGCGGATCAAACTACGCCAT
CATGTACTCCTGGTAAGTTCTCTCTAAACTTCTCCTTATAGATCCAACCTAACAAAATCTTAGGTACATGCCAAAGGACGAGCCCTCAACCGGT
ATTGGTCACCGTCACGATTGGGAAGGTGTAATTGTCTGGCTCTCCAGCGCCACCGCCACAACTGCCGACAACATCTTAGCCGTTTGTCCTTCCG
CCCACGGAGGCTGGGATTGTTCCACGGATGGCTATTCCCTTTCTGGTACCAGCCCTCTTATCAAGTACGAAAGTATCTGGCCCGTCGATCACTC
AATGGGTCTTACTAGTACTGTTGGTGGAAAACAACCTATGATTGCTTGGGAGTCTTTACCAACTGCTGCTCAAACTGCTCTTGAGAACACCGAT
TTCGGTGCTGCGAATGTTCCATTCATTCCGGCTGTTTTCACAGACAATCT

>Bcin04g02090(MLST10), partial sequence [organism=Botrytis cinerea, strain D09_K_2_3]
CGGAGGATGATATGGCAAAGTCTATGATTACCAAAGCATTTGTAGGCATGAGTAGTAAACTGTCGAATATGACTATGAATGATGTTTACAAGCC
CTACATCCATGTAAGAAATGTAGAATAGAGAGATCAGTAACTGGAACTAATATCGTTTGTAGGCTTTCAAGTTACTTACGCAGTTCAACCCAAT
CACTACAGCTATTGCCGAATCCCCACTGTTTCAAATGGCCGTCTCAGCAAATACCATCGAAAAGTACACACTGCTAGGCCCTTTCTTCAGAATA
TCTCCTCTGCAACAGGAAGTTACCAGGGAATACTTCAGTGCGCCAAAGACGATAGATAGGCGACACATTGCCACATCTCAAGATGCGTTACGAT
TGACCTTACAAACCCATCAAAAAGATTTACTTGATATCATCAACCACTTTGTTCGAGCAAGTCCAATCGCAAAAAGCAAAACCCTGGATTGGTT
CGCCTACATTGTGAATCAAAATCACAAGCGTCGAGCACTTCAGGTAGACCCGAAAGAAGTGTCTTCTGATGGCTTTATGCACAATGTCACTGTC
GTTCTAGATGGTCTTTGTGAGCCATTCATGGATACCACATTCTCGAAAATTTCGAAGATTGATATTGATTATCTAAGACGTGCGCCTCGTGTAG
ATATCAAGGACGAGACCAAGTTGAACGCTGACGAGAAGGCTTCTGAGAAGTATTATGAGGACACTGTTCCTGGCACTTCTAATTTCATCTCTGA
GGTCTTCTTTCTCACATTGGCTGCTCATCATTATGGTAGTGAAGCTCTTAATGCCACGCATAAGAGTCTGGAGAAAGACATCAAATACATTCAA
AAGCAATTGACTGCCGTTGAAGCA

>Bcin01g07220(MLST1), partial sequence [organism=Botrytis cinerea, strain D09_K_4_1]
ATGGCATATCTTGTTTCTTTTCGCATTATGCTTTGGAACGTGCTGGACAAGCTTGGGTTAGTGCTCTGCGTGTAGAAGCACTAAAGAAGATTCT
CGCACAACCGAAGTCATGGTTTGAGGAATCCAGGAATTCACCTGCCGGTTGAACGAAGTTTTGGATAGGAACTCTGAGGAAATGCGTAATCTCG
TTGGCCGCTTTGCTGGTATTGTATTCACAGCATTTTTTATGCTATTGATATCAATCATTGGGCTTTCGTGAATACATGGAAACTGACATTAGTC
TCAATGGCGACTGGGCCAGTTATATACGCTGTCACCAAAACGTTCAATCGCGTGAGTGGAAAATGGGAAACAAGTGCAACTACGCATCTGAAAT
GACCACTGGCATATTTTCAGAGACTTTCTCCAACATCAAAGTGGTTCGGGCTTTTACCTGGAACTTACTTTGAGACAAAACACACCAAAGCTAC
AGAAGAACTCTATAAAATTGGACTAATACGAGCAAACTACTCGGGATTGCTGTGGGGATTGACAGATGCGATGTCATTCTTCATCACTGCAACT
ATCTTTTATTATGCCACGGTTCTCATTACCAAGAGAGAGATCAGTATCGCGACTGCACTGCAGACTGTCAATCTTCTATTATTTGGTATTTCTA
ATAGTACGAATATGCTGGCCACGATACCACAAATCAACTCTTCTCGCGTTACAGCTACGCATATGCTTGCATTAGCCAATCTCGATTCATCTTC
CTCCCACGAAAATAAAGGAGCCGAACGGCTTTCGACAATCTTTCCAATCAATTCAACAGTCTCTCATCACATATCCTACTCGTCCTGAAAAACG
AACGATATCATCCTTTCTCTTTCCCTGATCCTAACTCAACAACTGCACTTGTCGGACCCTCCGGCTCCGGAAAGTCTACAATAGCTGCTCTGCT
CGTTGGTCTCTATCCGCCAGATACTTCAACACCTCCACCGTTGACATTCATCGTGTCTCCATAAGTAACTGTCACATTCCGTCTCTCCGGGCTT
CTCTCTCACTCGTCCCACAATACCGATTCTATTTCCAGCTACCATTCTCCATAACATCATTTATGGTCTCCCAGAATCTTCTCCTTGTGCCAGT
CTTCCATCTGCTATTGCATCAGCAAAAGATGTTGGATCCATGAATTTTCACATCGCTTCCACAAGTTATGATACTAT

>Bcin05g07690(MLST2), partial sequence [organism=Botrytis cinerea, strain D09_K_4_1]
ACCACTATCACCAAGTCCTTTTCTGCACTTTCCTCATCAAAAACGAACGAGAACATCATCAATAATACTTTGCTGAAGTAATTGGGTTTTATTT
TGGCCAGGATGCCTTCTCTCTACGACAGCAAGCCTTCGATGATATGTTGTGGGTAGTTCTTGGCTGGCTGGATACTGTCAAATTCATTGATTTA
CATTCTGAATTGCACTATTCAAACGACTCTCAGCCAGAATGGTACGGACAACAATATAAACCTGCATTTGCACATCGAGCGCGACTATTTTGGG
AATTGGCTTCACAAGGATGGGATACTACTCTCTGTGGTGGTGGATGATATGGTCACCATACCTTACTCCATACAAGAACGCAATTACCAATGAA
ACTCTATATCGCAGCTTCGATATCGATGTACCTATATTTCCCCGGAGATGACAATCAATCCCCATTTATGATTTCCAACCCTTCATATCCACCT
CACGATCCGAAATATCTACAGGCAGCTGTTGATGCTTACAAATGGCTGAATGGTTCCAACATGACGGATTCACAAGGATTATATGTCGACGGGT
ACCATATCTCGAATCTTTCTGGCGGTTGAAAACACCCATTGCGATTCTAGAAATGAGATGGTATATACCTACAATCAAGGTGTTTTGCTTACTG
GACAACGTGGTTGTATGACGCAACCGCCGCACGATCATACCTTGTAGATGGCCACAAACTCATCGCGAATGTTATTAATGCCACAGGCTATGAC
CTGAAACACAATGTTGTCATCTCACCGCCACCCAAAGATGGTTCCGCATTGGCAAAGTGGTTTGGCCTGGGTAGGAATGGAATACTGGAAGAAG
GATGCGATCAAGTGCTTCGTGTTCTCAAAATGGACAAACTTTCAAAAGGCATATTCTTTCATCACTTGATTGCGTTCTGTAGTGATTTGCCAGG
GGAGCCTATTGCAGGGACGAAGGAAAGCTTAGAACTCGACAGAGTGTGGCATTCTGACAAATGCTCACAGTATACAAAT

>Bcin06g01710(MLST3), partial sequence [organism=Botrytis cinerea, strain D09_K_4_1]
GTGAGTCTGACTTTTGTGTTTGAGCGTTAAGATAGACACTGATGTACCAAGGCAATCACCTAATTTCATGGCGTTCTTCCCTGCATCATCTACC
TACCCTGGAATGCTGGGAGAATTATACTCAGCAGCTTTCACAGCACCTGCTTTCAATTGGATCTGTTCCCCTGCCGTGACAGAATTGGAGACGG
TTGTAATGGATTGGCTGGCCAAGCTTCTCAATCTCCCAGACTGTTATTTGTCTTCGACTCATGGTGGTGGTGTTATCCAAGGATCAGCCTCGGA
AGCTATCGTTACCGTTATGGTTGCTGCCCGCGATAAATATCTTCGTGAAACTACTGAAGGTCTGTCGGGCATTGAACTCGAGGATGCGATTGCA

TATAAGAGGAGTAAGCTAGTTGCACTAGGAAGCGAAATGGCACACAGCTCCACGCAGAAAGCAGCGCAGATAGCTGGCGTTAGATTCCGATCGA
TTCCAGTACTCGCATCCAATGATTTCGCCATGACGGGTGATGATTTAGAGAAGGTATTGGAAGAATGCAAATCTCAAGGATTGGAACCCTTCTA
TCTAACTTCGACTTTGGGAACAACATCTACATGCGCAGTTGACGACTTCGCATCTATTGCAACAGTACTTTCAAAATATGCACCTCCAGATGTT
GCAGGCGAGATCTGGGTTCACGTCGATGCTGCTTATGCAGGTGCAGCTTTGGTTTGCCCTGAATACCATCATCTAACATCGTCCTTCCAGCATT
TCCATTCCTTCGATATGAACATGCACAAATGGCTTCTGACAAATTTCGACGCTTCTTGTCTATATGTCAAGAAACGCAAAGATCTGATAGATGC
ACTCTCCATAACACCAAGTTATCTTCGCAACGAGTTTTCAGAGAGTGGACTCGTAACCGACTATCGGGATTGGCAAATTCCTCTCGGAAGACGC
TTCCGAAGCTTAAAGATTTGGTTTGTCCTCAGAACCTAC

>Bcin09g03030(MLST4), partial sequence [organism=Botrytis cinerea, strain D09_K_4_1]
CGACCCTTCAACCCCACCGCAGCTCCTATACCGAAAGCGAGTATCCTACCAATTCTCCTTCCACCTGCGACTTTAAGACCATTGGCTTTCCGCA
CTTTCACAAAAAAGCATAGTTTAACATTGACGTCGTCGGCATTACAAGTGTTGGCTACTTTTATTGGAAAGCATTGTGGGACAGGATGGAGGGA
AGAAGGACTGGCAGAGAGAGTCTTGGAGGAGGTCGCCAAGAGTTGGAAGAATAGGAGTGGCGGTGTCATTGTCGAGGGCGAGGGAACGGAATTG
AAGGAGATTCTGAAAGCTTTGGAAGGGAATATGAGTGGTGGAAGGATAGTCATAGGAAGAGAGCTAAGCCGGCAGAATAGTTTAGCACTGGGAT
CATCACAATATGGAGAGGTCAATCATACAAGACTTGGGCTACGGCCAGGGAATATACCCAGAGAGGATAGTCAGTCAAGTTTGGGAATGTCAAC
GTTGGAGGTCAATGACGAGGAGGATGAGGATGGCCTGATGGATCCAAGAAGGTGGTTAAAAGTCATTGATGCATTTGAGCAACCTCGACTGGTG
TACAATGTTGCTAAAAAGCACTTTGATAGGTATGTTTCAATGATAAAATTTTATCTGAATCTACTAACTCAGTACAGAGATACCTCCAAACCTT
CATTGTTCCCACCTGCGTCTCATAAAACACTCCTCTTCCAAAACCGCTATAATGTTATCCATCAACGTCTCCTTCGCAATGAATCTTTTCAAAC
GCCCGCTTTTCAAGGTGGCAAATCTTCCCTTCAACGCAGCACGTCCGCCATTACCACCCAACAACAATCATACAAATTAACGCCGATAGCTAAT
CTTCTCGGTCGCAATCGCAGCTCTCATATGCTTCTCGGTCTCCTCAGTATTTCACCCACTGGTACCCTCGCCATCAATGACCTGACGGGCAGTA
TCGCTCTCGATCTTACACACGCAGCAGCCATTC

>Bcin11g01310(MLST5), partial sequence [organism=Botrytis cinerea, strain D09_K_4_1
GCTGACATGGACCTCATGTGGAACCGGCGTAGAATGCGCAACGCTTGAAGTTCCGCTCGAATATGGCGATGCAACGTCAACGGCAAAAGCCAGT
GTTGCGCTTGCTCGTTATCCTGCCACTGTTGCCGCGAGCAAGAAGCTCGGGTCTCTCTTGATAAACCCCGGTGGACCCGGTGCCTCTGGTGTTG
GCTTTGTGCAGTCTGGAGCCGGTGCCGCCGTCTCGACACTGAGTGGTGGATTGTACGATATCATCGGATGGGATCCACGTGGAACCGGTGCTTC
GGCTCCTATTTTGGAATGTTTTGCAAATGCCAGTGCGGAGTATGATTTTAACAACGCGTTTCCATCTGCTCCGAATCTCTGGCTCGGACAATTT
GCGAATGCCAGCGCAAATTCTGCTGTTAGCTCTGCTATCACATCCTTTGACACTTCTGTCGCTGCTCTTGCAAAAGCTTGCGTGGCTCAGAAAT
CTCCCGCTCTTTACACCTCAACAGCAGCATATGTTGCTCGAGACATGGCAGCGATAGTCGATGCATTGGATGGGACCTCTGCAAAACTTAACTA
CTGGGGTTTCTCATATGGAACTATCTTCCTAGCTGAGTTTATCCAAACTTTCCCAGGCCGCGTGGGAAGAGTTCTTGCCGATGGTGTTTTCGAC
GCAAAGGCAAATGCACTCACATACGTTAGCCAACTTCCCAACGATCAACTCAGTGTTCGTGCTTCGTTGAACGATTTTGCAGCTTTCTGCACCA
CCGCCGGTAGTAAAGGTTGCTCTTTTGCCACCGCCCCTACTGGAACCTCAGGTACTGTTGCTACCAGACTGGACAACATAATGAAGGATATGTT
CCTCAATCCTATTGTTGCTTCGGGCTTAAGCATCA

>Bcin15g03910(MLST6), partial sequence [organism=Botrytis cinerea, strain D09_K_4_1]
GCCAAAACACAAAATCATCCAACGATGAAGATGATACTCCACTTCCCTTGATTATCTGGCATGGACTCGGCGATAATTACAAAGCGGATGGTCT
TGCGCAAGTTGGAAAACTAGCTGAAGCTATTCATCCTGGGACTTTTGTCTACAATATTCATGTAGATGAGGATGCATCTGCAGATAGGACAGCT
ACCTTCTTTGGAAATCTCACTCGTGAGTACATCCCCTATTTTTCCTTTAAATATCATACTAACTCTCTTACCAAGTTCAAATCGAAAAGGTCTG
CGAAGACCTCGCCTCCCATCCTATTCTCTCTACCGCGCCCGCCGTCGACGCAATTGGATTCTCCCAAGGCGGCCAATTCTTGCGTGGTTACATA
TCCCGCTGCAATGCTCCACCCATCCGCTCTCTCCTGACCTTTGGTTCCCAACACAACGGCATTTCTGCCTTCCAAGCCTGTGGTCCTGCCGATT
TCCTCTGTCGCGGTGCTCAAACCCTTTTGCGATCCAACACCTGGTCAACCTTTGTCCAATCTCGTCTCGTACCCGCTCAATACTTCAGAGATCC
GGAAAACCTAGACTCTTACCTTGAATATTCCAATTTCCTTGCCGACATCAATAATGAGCGCGTTCTCAAGAACCAAACATATAAATCCAACATG
GAAAAATTGGAACGATTCGTAATGTATGTCTTTGAAGACGACACAACTGTCATTCCTAAGGAAAGTGGATGGTGGGCTGAAGTCAACGGCACGG
AAGTTACACCACTGAAAGAAAGAGCCATTTATAAAGAAGATTGGCTAGGTTTAAAGACATTGGATGAGGCCGGAAAATTAGTTTTCGAAACCAT
TCCAGGGGGACATATGACGTTAGGAGAGGAGATGCTAGAGAAGGCTTTCAAAGAGTATTTTGGTCCAGCAGGGAAGAAATTTGGG

>Bcin16g03460(MLST7), partial sequence [organism=Botrytis cinerea, strain D09_K_4_1]
ATGAACCTCTTGATTTGAACTCTTCATTAATTGCAAGTGATGAACCCCTATTCTGCCCAGAGGATAGTTACAAGACGTATATCATTAGTCGAGA
ACCACTCATGATATATATTGACGGATTTTTGAAAGCGAATGAAAGTAAACATTTGGTTGATGTTAGGTGTGTTATTCATTCTGATGAAATGAAC
AAGAGAGACTGATGAGATAGTGAACCGCTTTATGAACCATCTACTGTTTCTCACGGACAGGAAGTTACCATTGATCCTTCAGTTCGGAATTCTG
AAGTGGCGGTTTTAGAGAGGGATGAGGTGGTCAGGTGTATTGAGCATAGAGCGAGGGCATTTCAGGGGTGGAGGGGCGAGATGGGGATTGAGAA
GTTGAGGACGCAGAGGTATGGGGTTGGAGGACATTATGGGATGCATTTGTAAGTTTTGGGGGATTGACGAAGGCTTTGTCTATTTTCTACCAGT
ACGGATCTTGAAAGAAAGATAGCATGAACACGAGGGCTAATAACTAGGAAATAGCGATTGGAGCGGAGGTAAACGTGGCATAGACCGATTTAGT
ACTTTCATGGTCTATGTCGACGTATCCTCTGATATCGAAGGTGGAGGAACGGAATTCCCACGTATTGTGGGACCAAAAGGAGGAAGGTGGGAGG
ACTTCCTGGAAACTACGGAAGCATTGGATCCAAGAACTGGAGAAAATGTAACAGTAGAAGGGGTGACATTCAAACCAATCAAGGGAAATGCCGT
ATTCTGGGAAAATACTGACAACAACGGGAGGGGCTATGAT

>Bcin12g03020(MLST8), partial sequence [organism=Botrytis cinerea, strain D09_K_4_1]

```
CGATTGGCTGCGAAGAAAACTGCGCAGCACCGAGTCACCAAAAGCACGAATAAACCATCGGCAAGGGAAATATTCTCTGAACCACAACATGACA
CGAGCGCGGAAGAGTATATCGGGCGAGAAGCTTCATCAAGAGCACCAAAGCGACAACGAGTCGATGATAATTATAACTCTTACGGTGGAAGAAA
TGAAAATACAGCAGCTTATGCTTCCGGGAAACTTCCATCTGGAAGTATAAATGTTGGTGGAGGTAGGAAGACCACTTTTCAAGAAGAACCACGA
ACGGCATTTGTCGCTGGCAAGTTGCCCCCTGGAAGTATCAACATTGGTGGAAAGAAGGCTACCCAAATCGAGGACGAGGGTAGAGCAGCTTATG
CTTCCGGAAAATTGCCTCCAGGAAGTATAAACTCTGGCGCAAAGAGGGCGATTTCATTCCAAGATGAAACGAGACCGGCTTATGTCTCCGGAAA
GCTTCCACATGGTAGTATCGACGGTATGCGAAACCGTGAAATGGCTGCCGTCCACCGCGAAATTGCTGAGGGTGGAGGAAACCAGGCCAGGTT
GTCTCTTCTCTATTCACATTCAATCCTACTTCAAAGAAAACTTTTGATGAACCCGAAGAACAAGCGGAACCGGCAAAACCATCCAATGCGCCTT
TGACAGAGGAAATGGCGACTTTCACCAATCTAGGGTTATCGAGAAGGCTTGCAGCCCATCTATCGACTAAACTCGATATGAAAGCCCGACGGCC
ATTCAAAAAGCATCTGTGCAGCAGTTGGTATCGGACGATAGCGATGCTTTCATACAAGCAGAGACTGGATCTGGAAAAACTTTGGCATATCTAC
TACCTATAGTCGAGCGAATATTAGCATTGAGTGAGAATGGCGTACAAA

>Bcin02g07770(MLST9), partial sequence [organism=Botrytis cinerea, strain D09_K_4_1]
CAGCTTTCCCTTTCGGTCTTGGCATCTACAGTCATTGCCATCCCTACACCCTCACAACTTGAGTCTCGGGCCGTTATCGATTCCGATGCCGTTG
TAGGATTTGCCGAAACTGTTCCCAGTGGGACCGTAGGAACAGTTTATGAGGCATATAAACCATTCCTTAAAGTCGTAAATGGATGCGTACCATT
CCCTGCCGTCGATGCATCGGGTAACACAGGGTATGTCCTTATACCTTTCTCTTCCACACGATTGCTATTGAGTCTCTAATATATTTTAGTGGTG
GTTTGTCACCAACTGGCAGCAGCAATGGTGAATGCAGCAGCAGTACCGGTCAAGTATATGTTCGAGGAGGACAAAGCGGATCAAACTACGCTAT
CATGTACTCGTGGTAAGTTCTCTTTAAACTTCTCCTTATAGATCCAACCTAACAAAATCTTAGGTACATGCCAAAGGACGAGCCCTCAACCGGT
ATTGGTCACCGTCACGATTGGGAAGGTGTAATTGTCTGGCTCTCCAGCGCCACCGCCACAACTGCCGACAACATCTTAGCCGTTTGTCCTTCCG
CCCACGGAGGCTGGGATTGTTCCACGGATGGCTATTCCCTTTCTGGTACCAGCCCTCTTATCAAGTACGAAAGTATCTGGCCCGTCGATCACTC
AATGGGTCTTACTAGTACTGTTGGTGGAAAACAACCTATGATTGCTTGGGAGTCTTTACCAACTGCTGCTCAAACTGCTCTTGAGAACACCGAT
TTCGGTGCTGCGAATGTTCCATTCATTCCGGCTGTTTTCACAGATAATCT

>Bcin04g02090(MLST10), partial sequence [organism=Botrytis cinerea, strain D09_K_4_1]
CGGAGGATGATATGGCAAAGTCTATGATTACCAAAGCATTTGTAGGCATGAGTAGTAAATTGTCGAATATGACTATGAATGATGTTTACAAGCC
CTACATCCATGTAAGAAATGTAGAATAAAGAGATCAGTAACTGGAACTAATATCGTTTGCAGGCTTTCAAGTTACTTACGCAGTTCAACCCAAT
CACTACAGCTATTGCCGAATCCCCACTATTTCAAATGGCTGTCTCAGCAAATACCATCGAAAAGTACACACTGCTAGGCCCTTTCTTCAGAATA
TCTCCTCTGCAACAGGAAGTTACCAGGGAATACTTCAGTGCGCCAAAGACGATAGATAGGCGACACATTGCCACATCTCAAGATGCGTTACGAT
TGACCTTACAAACCCATCAAAAAGATTTACTTGATATCATCAACCACTTTGTTCGAGCAAGTCCAATCGCAAAAAGCAAAACCCTGGATTGGTT
CGCCTACATTGTGAATCAAAATCACAAGCGTCGAGCACTTCAGGTAGACCCGAAAGAAGTGTCTTCTGATGGCTTTATGCACAATGTCACTGTC
GTTCTAGATGGTCTTTGTGAGCCATTCATGGATACCACATTCTCAAAAATTTCGAAGATTGATATTGATTATCTAAGACGTGCGCCTCGTGTAG
ATATCAAGGACGAGACCAAGTTGAACGCTGATGAGAAGGCTTCTGAGAAGTATTATGAGGACACTGTTCCTGGCACTTCTAATTTCATCTCTGA
GGTATTCTTTCTGACATTGGCTGCTCATCATTATGGTAGTGAAGCTCTTAATGCCACGCATAAGAGTCTGGAGAAAGACATCAAATATATTCAA
AAGCAATTGACTGCCGTTGAAGCA

>Bcin01g07220(MLST1), partial sequence  [organism=Botrytis cinerea, strain D09_K_4_2]
ATGGCATATCTTGTTTCTTTTCGCATTATGCTTTGGAACGTGCTGGGCAAGCTTGGGTTAGTGCTCTGCGTGTAGAAGCACTAAAGAAGATTCT
CGCACAACCGAAGTCATGGTTTGAGGAATCCAGGAATTCACCTAGCCGGTTGAACGAAGTTTTGGATAGGAACTCTGAGGAAATGCGTAATCTC
GTTGGCCGCTTTGCTGGTATTGTATTCACAGCATTTTTTATGCTATTGATATCAATCATTTGGGCTTTCGTGAATACATGGAAACTGACATTAG
TCTCAATGGCAACTGGGCCAGTTATATACGCTGTCACCAAAACGTTCAATCGCGTGAGTGGAAAATGGGAAAACAAGTGCAACTACGCATCTGA
AATGACCACTGGCATATTTTCAGAGACTTTCTCCAACATCAAAGTGGTTCGGGCTTTTACTCTGGAAACTTACTTTGAGACAAAACACACCAAA
GCTACAGAAGAACTCTATAAAGTTGGACTAATACGAGCAAACTACTCGGGATTGCTGTGGGGATTGACAGATGCGATGTCATTCTTCATCACTG
CAACTATCTTTTATTATGCCACGGTTCTCATTACCAAGAGAGAGATCAGTATTGCGACTGCACTACAGACTGTCAATCTTCTATTATTTGGTAT
TTCTAATAGTACGAATATGCTGGCTATGATACCACAAATCAACTCTTCTCGCGTTACAGCTACGCATATGCTTGCATTAGCCAATCTCGATTCA
TCTTCCTCCCACGAAAATAAAGGAACCGAACGGCTTTCGACAATCTTTCCAATCAAATTCAACCGTCTTTCATTCACATACCCTACTCGTCCTG
AAAAACGAACGATATCATCCTTTTCTCTTTCCCTGATTCCTAACTCAACAACTGCACTTGTCGGACCCTCCGGCTCCGGAAAATCTACGATAGC
TGCTCTGCTCATTGGTCTCTATCCGCCAGATACTTCAACACCTCCACCGTTGACATTCAATCGCGTCTCCATAAGTAACTGTCACATTCCGTCT
CTCCGGGCTTCTCTCTCACTCGTCCCACAAATACCGATTCTATTTCCAGCTACCATTCTCCATAACATCATTTATGGCCTCCCAGAATCTTCTC
CTTGTGCTAGTCTTCCATCTGCTATTGCATCAGCAAAAGATGCTGGGATCCATGAATTTATCACATCGCTTCCACAAGGTTATGATACTAT

>Bcin05g07690(MLST2), partial sequence  [organism=Botrytis cinerea, strain D09_K_4_2]
ACCACTATCACCAAGTCCTTCTCTGCACTTTCCTCATCAAAAACGAACGAGAACATCATCAATAAATACTTTGCTGAAGTAATTGGGTTTTATT
TTGGCCAGGATGCCTTCTCTCTACGACAGCAAGCCTTCGATGATATGTTGTGGGTAGTTCTTGGCTGGCTGGATACTGTCAAATTCATTGATTT
ACATTCTGAATTGCACTATTCAAACGACTCTCAGCCAGAATGGTACGGACAACAATATAAACCTGCATTTGCACATCGAGCGCGACTATTTTGG
GAATTGGCTTCACAAGGATGGGATACTACTCTCTGTGGTGGTGGGATGATATGGTCACCATACCTTACTCCATACAAGAATGCAATTACCAATG
AACTCTATATCGCAGCTTCGATATCGATGTACCTCTATTTCCCCGGAGATGACAATCAATCCCCATTTATGCTTTCCAACCCTTCATATCCACC
TCACGATCCGAAATATCTACAGGCAGCTGTTGATGCTTACAAATGGCTGAATGGTTCCAACATGACGGATTTACAAGGATTATATGTCGACGGG
TATCATATCTCGAATCTTTCTGGCGGTGAAAACACCCATTGCGATTCTAGAAATGAGATGGTATATACCTACAATCAAGGTGTTTTGCTTACTG
```

GACAACGTGGTTTGTATGACGCAACCGCCGCACGATCATACCTTGTGGATGGCCACAAACTCATCGCGAATGTTATTAATGCCACAGGCTATGA
CCTGAAACACAATGTTGCCATCTCACCGCCACCCAAAGATGGTTCCGCATTGGCAAAGTGGTTTGGCCTGGGTAGGAATGGAATACTGGAAGAA
GGATGCGATTCAAGTGCTTCGTGTTCTCAAAATGGACAAACTTTCAAAGGCATATTCTTTCACCACTTGATTGCGTTCTGTAGTGATTTGCCAG
GGGAGCCTATTGCAGGGACGAAGGAAGGCCTAGAACTCGACAGAGTGTGGCATTCTGACAAATGCTCACAGTATACAAAATGG

>Bcin06g01710(MLST3), partial sequence [organism=Botrytis cinerea, strain D09_K_4_2]
GGTGAGTCTGACTTTTGTATTTGAGCGTTAAGATGGACACTGATGTACCAAGGCAATCACCTAATTTCATGGCGTTCTTCCCTGCATCATCTAC
CTACCCTGGAATGCTGGGAGAATTATACTCAGCAGCTTTCACAGCTCCTGCTTTCAATTGGATCTGTTCCCCTGCTGTGACAGAATTGGAGACG
GTTGTAATGGATTGGTTGGCCAAGCTTCTCAATCTCCCAGACTGTTATTTGTCGTCGACTCATGGTGGTGGTGTTATCCAAGGATCAGCCTCGG
AAGCTATCGTTACCGTTATGGTTGCTGCCCGCGATAAGTATCTTCGTGAAACCACTGAAGGTCTGTCGGGAATTGAACTCGAGGATGCGATTGC
ATATAAGAGGAGTAAGCTAGTTGCACTAGGAAGCGAAATGGCACACAGCTCCACGCAGAAAGCAGCGCAGATAGCTGGCGTTAGATTCCGATCG
ATTCCAGTACTCGCATCCAATGATTTCGCCATGACGGGTGATGATTTAGAGAAGGTATTGAAAGAATGCAAATCTCAAGGATTGGAACCCTTCT
ATCTAACTTCGACGTTGGGAACAACATCTACATGCGCAGTTGACGACTTCGCATCTATTGCAACAGTACTTTCAAAATATGCACCTCCAGATGT
TGCAGGCGAGATCTGGGTTCACGTCGATGCTGCTTATGCAGGTGCAGCTTTGGTTTGCCCTGAATACCATCATCTAACATCGTCCTTCCAGCAT
TTCCATTCCTTTGATATGAACATGCACAAATGGCTTCTGACAAATTTCGACGCTTCTTGCCTATATGTCAAGAAACGCAAAGATCTGATCGATG
CACTCTCCATAACACCAAGTTATCTTCGCAACGAGTTTTCAGAGAGTGGACTCGTAACCGACTATCGGGACTGGCAAATTCCTCTCGGAAGACG
TTTCCGAAGCTTAAAGATTTGGTTTGTCCTCAGAACCTAC

>Bcin09g03030(MLST4), partial sequence [organism=Botrytis cinerea, strain D09_K_4_2]
ACCCTTCAACCCCACCGCAGCTCCTATACCGGAAGCGAGTATCCTACCAATTCTCCTTCCACCTGCGACTTTAAGACCATTGGCTTTTCGCACT
TTCACAAAAAAGCATAGTTTAACATTGACGTCGTCGGCATTACAAGTGTTGGCTACTTTTATTGGAAAGCATTGTGGGACAGGATGGAGGGAGG
AAGGACTGGCAGAGAGAGTCTTGGAGGAGGTCGCCAAGAGTTGGAAGAATAGGAGTGGCGGTGTCATTGTCGAGGGCGAGGGAACGGAATTGAA
GGAGATTCTGAAAGCTCTGGAAGGGAATATGAGTGGTGGAAGGATAGTCATAGGAAGAGAGCTAAGCCGGCAGAATAGTTTAGTACTGGGATCA
TCACAATATGGAGAGGTCAATCATACAAGACTTGGGCTACGGCCAGGGAATATACCCAGAGAGGATAGTCAGTCAAGTTTGGGAATGTCAACGT
TGGAGGTCAATGACGAGGAAGATGAGGATGGCCTGATGGATCCAAGAAGGTGGTTAAAAGTCGTTGATGCATTTGAGCAACCTCGACTGGTGTA
CAATGTTGCTAAAAAGCACTTTGATAGGTATGTTTCAATGATAAAATTTTATCTGAATCGACTAACTCAGTACAGAGATACCTCCAAACCTTCA
TTGTTCCCACCTGCGTCTCATAAAACACTCCTCTTCCAAAACCGCTATAATGTTATCCATCAACGTCTCCTTCGCAATGAATCTTTTCAAACGC
CCGCTTTTCAAGGTGGCAAATCTTCCCTTCAACGCAGCACGTCCGCCATTACCACCCAACAACAATCATACAAATTAACGCCGATAGCTAATCT
TCTCGGTCGCAATCGCAGCTCTCATATGCTTCTCGGTCTCCTCAGTATTTCACCCACTGGTACCCTCGCCATCAATGACCTGACGGGCAGTATC
GCTCTTGATCTTACACACGCAGCAGCCATTC

>Bcin11g01310(MLST5), partial sequence [organism=Botrytis cinerea, strain D09_K_4_2]
ACTGACATGGACCTCATGTGGAACCGGCGTAGAATGCGCAACGCTTGAAGTTCCGCTCGAATATGGCGATGCAACGTCAACGGCAAAAGCCAGT
GTTGCGCTTGCTCGTTATCCTGCCACTGTTGCCGCGAGCAAGAAGCTCGGGTCTCTCTTGATAAATCCCGGTGGACCCGGTGCCTCTGGTGTTG
GCTTTGTGCAGTCTGGAGCCGGTGCCGCCGTCTCGACACTGAGTGGTGGATTATACGATATCATCGGATGGGATCCACGTGGAACCGGTGCTTC
GGCTCCTATTTTGGAATGTTTTGCAAATGCCAGTGCGGAGTATGATTTTAACAACGCGTTTCCATCTGCTCCGAATCTCTGGCTCGGACAATTT
GCGAATGCCAGCGCAAATTCTGCTGTTAGCTCTGCTATCACATCCTTTGACACTTCTGTCGCTGCTCTTGCAAAAGCTTGCGTGGCTCAGAAAT
CTCCCGCTCTTTACACCTCAACAGCAGCATATGTTGCTCGAGACATGGCAGCGATAGTCGATGCATTGGATGGGACCTCTGCAAAACTTAACTA
CTGGGGTTTCTCATATGGAACTATCTTCCTAGCTGAGTTTATCCAAACTTTCCCAGGCCGCGTGGGAAGAGTTCTTGCCGATGGTGTTTTCGAC
GCAAAGGCAAATGCACTCACATACGTTAGCCAACTTCCCAACGATCAACTCAGTGTTCGTGCTTCGTTGAACGATTTTGCAGCTTTCTGCACCA
CCGCCGGTAGTAAAGGTTGCTCTTTTGCCACCGCCCCTACTGGAACCTCAGGTACTGTTGCTACCAGACTGGACAACATAATGAAGGATATGTT
CCTCAATCCTATTGTTGCTTCGGGCTTGAGCATCA

>Bcin15g03910(MLST6), partial sequence [organism=Botrytis cinerea, strain D09_K_4_2]
GCCAAAACACAAAATCATCCAACGATGAAGATGATACTCCACTTCCCTTGATTATCTGGCATGGACTCGGCGATAATTACAAAGCGGATGGTCT
TGCGCAAGTTGGAAAACTAGCTGAAGCTATTCATCCTGGGACTTTTGTCTATAATATTCATGTAGATGAGGATGCATCTGCAGATAGGACAGCT
ACCTTCTTTGGAAATCTCACTCGTGAGTACATCCCCTATTTTTCCTTTAAATACCATACTAACTCTCTTACCAAGTTCAAATCGAAAAGGTCTG
CGAAGACCTCGCCTCCCATCCTATTCTCTCTACCGCGCCCGCCGTCGACGCAATTGGATTCTCCCAAGGCGGCCAATTCTTGCGCGGTTACATA
TCCCGCTGCAATGCTCCACCCATCCGCTCTCTCCTGACCTTCGGTTCCCAACACAACGGCATTTCTGCCTTCCAAGCCTGTGGTCCTGCCGATT
TCCTCTGTCGCGGTGCTCAAACCCTTTTGCGATCCAACACCTGGTCAACCTTTGTCCAATCTCGTCTCGTACCTGCTCAATACTTCAGAGATCC
GGAAAACCTAGACTCTTACCTTGAATATTCCAATTTCCTTGCCGACATCAATAATGAGCGCGTTCTCAAGAACCAAACATATAAATCCAACATG
GAAAAATTGGAACGATTCGTAATGTATGTCTTTGAAGACGATACAACTGTCATCCCTAAGGAAAGTGGATGGTGGGCTGAAGTCAATGGCACGG
AAGTTACACCACTGAAAGAAAGAGCCATTTATAAAGAAGATTGGCTAGGGTTAAAGACATTGGATGAGGCCGGAAAATTAGTTTTCGAAACCAT
TCCAGGGGGACATATGACGTTAGGAGAGGAGATGCTAGAGAAGGCTTTCAAAGAGTACTTTGGTCCAGCAGGGAAAAAATTTGGG

>Bcin16g03460(MLST7), partial sequence [organism=Botrytis cinerea, strain D09_K_4_2]
ATGAACCTCTTAATTTGAACTCTTCATTAATTGCAAGTGATGAGCCCCTATTCTGCCCAGAGGATAGTTACAAGACGTATATCATTAGTCGAGA

ACCACTCATGATATACATTGACGGATTTTTGAAAGCGAATGAAAGTAAACACTTGGTTGATGTTAGGTGTGTTATTTATTCTGATGAAATGAAC
AGAGAGACTGATGAGATAGTGAACCGCTTTATGAACCGTCTACTGTTTCTCACGGACAGGAAGTTACCATTGATCCTTCGGTTCGAAATTCTGA
AGTGGCGGTTTTAGAGAGGGATGAGGTGGTCAGGTGTATTGAGCATAGAGCGAGGGCATTTCAGGGGTGGAGGGACGAGATGGGGATTGAGAAG
CTGAGGACGCAGAGGTATGGGGTTGGAGGACATTATGGGATGCATTTGTAAGTTTTGGGGATTGACGAAGGCCTCTGTCTATTTTCTATCAGTA
CGGATCTTGAAAGAAAGATAGCATGAACACGAGGGCTAATAACTAGGAAATAGCGATTGGAGCGGAGGTAAACGTGGCATAGACCGATTTAGTA
CTTTCATGGTCTATGTCGACGTATCCTCTGATATCGAGGTGGAGGAACGGAATTCCCACGTATTGTGGGACCAAAAGGAGGAAGGTGGGAGGAC
TTCCTGGAAACTACGGAAGCATTGGATCCAAGAACTGGAGAAAATGTAACAGTAGAAGGGGTGACATTCAAACCAATCAAGGGAAATGCCGTAT
TCTGGGAAAATGCTGACAACAACGGGAGGGGCTATGAT

>Bcin12g03020(MLST8), partial sequence [organism=Botrytis cinerea, strain D09_K_4_2]
CGATTGGCTGCGAAGAAAACTGCGCAGCACCGAGTCACCAAAAGCACGAATAAACCATCGGCAAGGGAAATATTCTCTGAACCACAACATGACA
CGAGCGCGGAAGAGTATATCGGGCGAGAAGCTTCATCAAGAGCACCAAAGCGACAACGAGTCGATGATAATTATAACTCTTACGGTGGAGGAAA
TGAGAATACAGCAGCTTATGCTTCCGGGAAACTTCCATCTGGAAGTATAAATGTTGGTGGAGGTAGGAAGACCACTTTTCAAGAAGAACCTCGA
ACGGCATTTGTCGCTGGCAAGTTGCCCCCTGGAAGTATCAACATTGGTGGAAAGAAGGCTACCCAAATCGAGGACGAGGGTAGAGCAGCTTATG
CTTCCGGAAAATTGCCCCCAGGAAGTATAAACTCTGGCGCAAAGAAGGCGATTTCATTCCAAGATGAAACGAGACCGGCTTATGTCTCTGGAAA
GCTTCCACATGGTAGTATCGACGGTATGCGAAACCGTGAAATGGCTGCCGTCCACCGCGAAATTGCTGAGGGTGGGAGGAAACCAGGCCAGGTT
GTCTCTTCTCTATTCACATTCAATCCTACTTCAAAGAAAACTTTCGATGAACCCGAAGAACAAGCGGAACCGGCAAAACCATCCAATGCACCTT
TGACAGAGGAAATGGCGACTTTCACCAATCTAGGGTTATCGAGAAGGCTTGCAGCCCATCTATCGACTAAACTCGATATGAAAGCCCCGACCGC
CATTCAAAAAGCATCTGTGCAGCAGTTGGTATCGGACGATAGCGATGCTTTCATACAAGCAGAGACTGGATCTGGAAAAACTTTGGCATATCTA
CTACCTATAGTCGAGCGAATATTAGCATTGAGTGAGAATGGCGTACAAAT

>Bcin02g07770(MLST9), partial sequence [organism=Botrytis cinerea, strain D09_K_4_2]
CAGCTTTCCCTTTCGGTCTTGGCATCTACAGTCATTGCCATCCCTACACCATCACAACTTGAGTCTCGGGCCGTTATCGATTCCGATGCCGTTG
TAGGATTTGCCGAAACTGTTCCCAGTGGGACCGTAGGAACAGTTTATGAGGCATATAAACCATTCCTTAAAGTCGTAAATGGATGCGTACCATT
CCCTGCCGTCGATGCATCGGGTAACACAGGGTATGTCCTTATACCATTCTCTTCCACACGATTGCTATTGAGTCTCTAACATATTTTAGTGGTG
GTTTGTCACCAACTGGCAGTAGCAATGGTGGTTGCAGCAGCAGTACCGGTCAAGTATATGTTCGAGGAGGACAAAGCGGATCAAACTACGCCAT
CATGTACTCCTGGTAAGTTCTCTCTAAACTTCTCCTTATAGATCCAACCTAACAAAATCTTAGGTACATGCCAAAGGACGAGCCCTCAACCGGT
ATTGGTCACCGTCACGATTGGGAAGGTGTAATTGTCTGGCTCTCAAGCGCCACCGCCACAACTGCCGACAACATCTTAGCCGTTTGTCCTTCCG
CCCACGGAGGCTGGGATTGTTCCACCGATGGATATTCCCTTTCTGGTACCAGCCCTCTTATCAAGTACGAAAGTATCTGGCCCGTCGATCATTC
AATGGGTCTTACCAGTACTGTTGGTGGAAAACAGCCTATGATTGCTTGGGAGTCTTTACCAACTGCTGCTCAAACTGCTCTTGAGAACACCGAT
TTCGGTGCTGCGAATGTTCCATTCATTCCGGCTGTTTTCACAGATAATCT

>Bcin04g02090(MLST10), partial sequence [organism=Botrytis cinerea, strain D09_K_4_2]
CGGAGGATGATATGGCAAAGTCTATGATTACCAAAGCATTTGTAGGCATGAGTAGTAAACTGTCGAATATGACTATGAATGATGTTTACAAGCC
CTACATCCATGTAAGAAATGTAGAATAGAGAGATCAGTAACTGGAACTAATATCGTTTGTAGGCTTTCAAGTTACTTACGCAGTTCAACCCAAT
CACTACAGCTATTGCCGAATCCCCACTATTTCAAATGGCTGTCTCAGCAAATACCATCGAAAAGTACACACTGCTAGGCCCTTTCTTCAGAATA
TCTCCTCTGCAACAGGAAGTTACCAGGGAATACTTCAGTGCGCCAAAGACGATAGATAGACGACACATTGCCACATCTCAAGATGCGTTACGAT
TAACCTTACAAACCCATCAAAAAGATTTACTTGATATCATCAACCACTTTGTTCGAGCAAGTCCAATCGCAAAAAGCAAAACCCTGGATTGGTT
CGCCTACATTGTGAATCAAAATCACAAACGTCGAGCACTTCAGGTAGACCCGAAAGAAGTGTCTTCTGATGGCTTTATGCACAATGTCACTGTC
GTTCTAGATGGTCTTTGTGAGCCATTCATGGATACCACATTCTCGAAAATTTCGAAGATTGATATTGATTATCTAAGACGTGCGCCTCGTGTAG
ATATCAAGGACGAGACCAAGTTGAACGCTGATGAGAAGGCTTCTGAGAAGTATTATGAGGACACTGTTCCTGGCACTTCTAATTTCATCTCTGA
GGTCTTCTTTCTGACATTGGCTGCTCATCATTATGGTAGTGAAGCTCTTAATGCCACGCATAAGAGTCTGGAGAAAGACATCAAATATATTCAA
AAGCAATTGACTGCCGTTGAAGCA

>Bcin01g07220(MLST1), partial sequence [organism=Botrytis cinerea, strain D10_B_F1_6]
ATGGCATATCTTGTTTCTTTTCGCATTATGCTTTGGAACGTGCTGGACAAGCTTGGGTTAGTGCTCTGCGTGTAGAAGCACTAAAGAAGATTCT
CGCACAACCGAAGTCATGGTTTGAGGAATCCAGGAATTCACCTGGCCGGTTGAACGAAGTTTTGGATAGGAACTCTGAGGAAATGCGTAATCTC
GTTGGCCGCTTTGCTGGTATTGTATTCACAGCATTTTTTATGCTATTGATATCAATCATTTGGGCTTTCGTGAATACATGGAAACTGACATTAG
TCTCAATGGCGACTGGGCCAGTTATATACGCTGTCACCAAAACGTTCAATCGCGTGAGTGGAAAATGGGAAAACAAGTGCAACTACGCATCTGA
AATGACCACTGGCATATTTTCAGAGACTTTCTCCAACATCAAAGTGGTTCGGGCTTTTACTCTGGAAACTTACTTTGAGACAAAACACACCAAA
GCTACAGAAGAACTCTATAAAATTGGACTAATACGAGCAAACTACTCGGGATTGCTGTGGGGATTGACAGATGCGATGTCATTCTTCATCACTG
CAACTATCTTTTATTATGCCACGGTTCTCATTACCAAGAGAGAGATCAGTATCGCGACTGCACTGCAGACTGTCAATCTTCTATTATTTGGTAT
TTCTAATAGTACGAATATGCTGGCCATGATACCACAAATCAACTCTTCTCGCGTTACAGCTACGCATATGCTTGCATTAGCCAATCTCGATTCA
TCTTCCTCCCGCGAAAATAAAGGAGCCGAACGGCTTTCGACAATCTTTCCAATCAAATTCAACAGTCTCTCATTCACATATCCTACTCGTCCTG
AAAAACGAACGACATCATCCTTTTCTCTTTCCCTGATTCCTAACTCAACAACTGCACTTGTCGGACCCTCCGGCTCCGGAAAATCTACAATAGC
TGCTCTGCTCGTTGGTCTCTATCCGCCAGATACTTCAACACCTCCACCGTTGACATTCAATCGTGTCTCCATAAGTAACTGTCACATTCCGTCT
CTCCGGGCTTCTCTCTCACTCGTCCCACAAATACCGATTCTACTTCCAGCTACCATTCTCCATAACATCATTTATGGTCTCCCAGAATCTTCTC

CTTGTGCTAGTCTTCCATCTGCTATTGCATCAGCAAAAGATGCTGGGATCCATGAATTTATCACATCGCTTCCACAAGGTTATGATACTAT

>Bcin05g07690(MLST2), partial sequence [organism=Botrytis cinerea, strain D10_B_F1_6]
ACCACTATCACCAAGTCCTTTTCTGCACTTTCCTCATCAAAAATGAACGAGAACATCATCAATAAATACTTTGCTGAAGTAATTGGGTTTTATT
TTGGCCAGGATGCCTTCTCTCTACGACAGCAAGCCTTCGATGATATGTTGTGGGTAGTTCTTGGCTGGCTGGATACTGTCAAATTCATTGATTT
ACATTCTGAATTGCACTATTCAAACGACTCTCAGCCAGAATGGTACGGACAACAATATAAACCTGCATTTGCACATCGAGCGCGACTATTTTGG
GAATTGGCTTCACAAGGATGGGATACTACTCTCTGTGGTGGTGGGATGATATGGTCACCATACCTTACTCCATACAAGAACGCAATTACCAATG
AACTCTATATCGCAGCTTCGATATCGATGTACCTATATTTCCCCGGAGATGACAATCAATCCCCATTTATGATTTCCAACCCTTCATATCCACC
TCACGATCCGAAATATCTACAGGCAGCTGTTGATGCTTACAAATGGCTGAATGGTTCCAACATGACGGATTCACAAGGATTATATGTCGACGGG
TACCATATCTCGAATCTTTCTGGCGGTGAAAACACCCATTGCGATTCTAGAAATGAGATGGTATATACCTACAATCAAGGTGTTTTGCTTACTG
GACAACGTGGTTTGTATGACGCAACCGCCGCACGATCATACCTTGTAGATGGCCACAAACTCATCGCGAATGTTATTAATGCCACAGGCTATGA
CCTGAAACACAATGTTGTCATCTCACCGCCACCCAAAGATGGTTCCGCATTGGCAAAGTGGTTTGGCCTGGGTAGGAATGGAATACTGGAAGAA
GGATGCGATTCAAGTGCTTCGTGTTCTCAAAATGGACAAACTTTCAAAGGCATATTCTTTCATCACTTGATTGCGTTCTGTAGTGATTTGCCAG
GGGAGCCTATTGCAGGGACGAAGGAAAGCTTAGAACTCGACAGAGTGTGGCATTCTGACAAATGCTCACAGTATACAAAAT

>Bcin06g01710(MLST3), partial sequence [organism=Botrytis cinerea, strain D10_B_F1_6]
GTGAGTCTGACTTTTGTATTTGAGCGTTAAGATAGACACTGATATACCAAGGCAATCACCTAATTTCATGGCGTTCTTCCCTGCATCATCTACC
TACCCTGGAATGCTGGGAGAATTATACTCAGCAGCTTTCACAGCTCCTGCTTTCAATTGGATCTGTTCCCCTGCCGTGACAGAATTGGAGACGG
TTGTAATGGATTGGCTGGCCAAGCTTCTCAATCTCCCAGACTGTTATTTGTCTTCGACTCATGGTGGTGGTGTTATCCAAGGATCAGCCTCGGA
AGCTATCGTTACCGTTATGGTTGCTGCCCGCGATAAATATCTTCGTGAAACTACTGAAGGTCTGTCGGGCATTGAACTCGAGGATGCGATTGCA
TATAAGAGGAGTAAGCTAGTTGCACTAGGAAGCGAAATGGCACACAGCTCCACGCAGAAAGCAGCGCAGATAGCTGGCGTTAGATTCCGATCGA
TTCCAGTACTCGCATCCAATGATTTCGCCATGACGGGTGATGATTTAGAGAAGGTATTGGAAGAATGCAAATCTCAAGGATTGGAACCCTTCTA
TCTAACTTCGACTTTGGGAACAACATCTACATGCGCAGTTGACGACTTCGCATCTATTGCAACAGTACTTTCAAAATATGCACCTCCAGATGTT
GCAGGCGAGATCTGGGTTCACGTCGATGCTGCTTATGCAGGTGCAGCTTTGGTTTGCCCTGAATACCATCATCTAACATCGTCCTTCCAGCATT
TCCATTCCTTCGATATGAACATGCACAAATGGCTTCTGACAAATTTCGACGCTTCTTGTCTATATGTCAAGAAACGCAAAGATCTGATCGATGC
ACTCTCCATAACACCAAGTTATCTTCGCAACGAGTTTTCAGAGAGTGGACTCGTAACCGACTATCGGGACTGGCAAATTCCTCTCGGAAGACGC
TTCCGAAGCTTAAAGATTTGGTTTGTCCTCAGAACCTAC

>Bcin09g03030(MLST4), partial sequence [organism=Botrytis cinerea, strain D10_B_F1_6]
ACCCTTCAACCCCACCGCAGCTCCTATACCGAAAGCGAGTATCCTACCAATTCTCCTTCCACCTGCGACTTTAAGACCATTGGCTTTCCGCACT
TTCACAAAAAAGCATAGTTTAACATTGACGTCGTCGGCATTACAAGTGTTGGCTACTTTTATTGGAAAGCATTGTGGGACAGGATGGAGGGAAG
AAGGACTGGCAGAGAGAGTCTTGGAGGAGGTCGCCAAGAGTTGGAAGAATAGGAGTGGCGGTGTCATTGTCGAGGGCGAGGGAACGGAATTGAA
GGAGATTCTGAAAGCTTTGGAAGGGAATATGAGTGGTGGAAGGATAGTCATAGGAAGAGAGCTAAGCCGGCAGAATAGTTTAGTACTGGGATCA
TCACAATATGGAGAGGTCAATCATACAAGACTTGGGCTACGGCCAGGGAATATACCCAGAGAGGATAGTCAGTCAAGTTTGGGAATGTCAACGT
TGGAGGTCAATGACGAGGAGGATGAGGATGGCCTGATGGATCCAAGAAGGTGGTTAAAAGTCATTGATGCATTTGAGCAACCTCGACTGGTGTA
CAATGTTGCTAAAAAGCACTTTGATAGGTATGTTTCAATGATAAAATTTTATCTGAATCTACTAACTCAGTACAGAGATACCTCCAAACCTTCA
TTGTTCCCACCTGCGTCTCATAAAACACTCCTCTTCCAAAACCGCTATAATGTTATCCATCAACGTCTCCTTCGCAATGAATCTTTTCAAACGC
CCGCTTTTCAAGGTGGCAAATCTTCCCTTCAACGCAGCACGTCCGCCATTACCACCCAACAACAATCATACAAATTAACGCCGATAGCTAATCT
TCTCGGTCGCAATCGCAGCTCTCATATGCTTCTCGGTCTCCTCAGTATTTCACCCACTGGTACCCTCGCCATCAATGACCTGACGGGCAGTATC
GCTCTCGATCTTACACACGCAGCAGCCATTC

>Bcin11g01310(MLST5), partial sequence [organism=Botrytis cinerea, strain D10_B_F1_6]
GCTGACATGGACCTCATGTGGAACCGGCGTAGAATGCGCAACGCTTGAAGTTCCGCTCGAATATGGCGATGCAACGTCAACGGCAAAAGCCAGT
GTTGCGCTTGCTCGTTATCCTGCCACTGTTGCCGCGAGCAAGAAGCTCGGGTCTCTCTTGATAAACCCCGGTGGACCCGGTGCCTCTGGTGTTG
GCTTTGTGCAGTCTGGAGCCGGTGCCGCCGTCTCGACACTGAGTGGTGGATTGTACGATATCATCGGATGGGATCCACGTGGAACCGGTGCTTC
GGCTCCTATTTTGGAATGTTTTGCAAATGCCAGTGCGGAGTATGATTTTAACAACGCGTTTCCATCTGCTCCGAATCTCTGGCTCGGACAATTT
GCGAATGCCAGCGCAAATTCTGCTGTTAGCTCTGCTATCACATCCTTTGACACTTCTGTCGCTGCTCTTGCAAAAGCTTGCGTGGCTCAGAAAT
CTCCCGCTCTTTACACCTCAACAGCAGCATATGTTGCTCGAGACATGGCAGCGATAGTCGATGCATTGGATGGGACCTCTGCAAAACTTAACTA
CTGGGGTTTCTCATATGGAACTATCTTCCTAGCTGAGTTTATCCAAACTTTCCCAGGCCGCGTGGGAAGAGTTCTTGCCGATGGTGTTTTCGAC
GCAAAGGCAAATGCACTCACATACGTTAGCCAACTTCCCAACGATCAACTCAGTGTTCGTGCTTCGTTGAACGATTTTGCAGCTTTCTGCACCA
CCGCCGGTAGTAAAGGTTGCTCTTTTGCCACCGCCCCTACTGGAACCTCAGGTACTGTTGCTACCAGACTGGACAACATAATGAAGGATATGTT
CCTCAATCCTATTGTTGCTTCGGGCTTAAGCATCA

>Bcin15g03910(MLST6), partial sequence [organism=Botrytis cinerea, strain D10_B_F1_6]
GCCAAAACACAAAATCATCCAACGATGAAGATGATACTCCACTTCCCTTGATTATCTGGCATGGACTCGGCGATAATTACAAAGCGGATGGTCT
TGCGCAAGTTGGAAAACTAGCTGAAGCTATTCATCCTGGGACTTTTGTCTACAATATTCATGTAGATGAGGATGCATCTGCAGATAGGACAGCT
ACCTTCTTTGGAAATCTCACTCGTGAGTACATCCCCTATTTTTCCTTTAAATATCATACTAACTCTCTTACCAAGTTCAAATCGAAAAGGTCTG

CGAAGACCTCGCCTCCCATCCTATTCTCTCTACCGCGCCCGCCGTCGACGCAATTGGATTCTCCCAAGGCGGCCAATTCTTGCGTGGTTACATA
TCCCGCTGCAATGCTCCACCCATCCGCTCTCTCCTGACCTTTGGTTCCCAACACAACGGCATTTCTGCCTTCCAAGCCTGTGGTCCTGCCGATT
TCCTCTGTCGCGGTGCTCAAACCCTTTTGCGATCCAACACCTGGTCAACCTTTGTCCAATCTCGTCTCGTACCCGCTCAATACTTCAGAGATCC
GGAAAACCTAGACTCTTACCTTGAATATTCCAATTTCCTTGCCGACATCAATAATGAGCGCGTTCTCAAGAACCAAACATATAAATCCAACATG
GAAAAATTGGAACGATTCGTAATGTATGTCTTTGAAGACGACACAACTGTCATTCCTAAGGAAAGTGGATGGTGGGCTGAAGTCAACGGCACGG
AAGTTACACCACTGAAAGAAAGAGCCATTTATAAAGAAGATTGGCTAGGTTTAAAGACATTGGATGAGGCCGGAAAATTAGTTTTCGAAACCAT
TCCAGGGGGACATATGACGTTAGGAGAGGAGATGCTAGAGAAGGCTTTCAAAGAGTATTTTGGTCCAGCAGGGAAGAAATTTGGG

>Bcin16g03460(MLST7), partial sequence [organism=Botrytis cinerea, strain D10_B_F1_6]
ATGAACCTCTTGATTTGAACTCTTCATTAATTGCAAGTGATGAACCCCTATTCTGCCCAGAGGATAGTTACAAGACGTATATCATTAGTCGAGA
ACCACTCATGATATATATTGACGGATTTTTGAAAGCGAATGAAAGTAAACATTTGGTTGATGTTAGGTGTGTTATTTATTCTGATGAAATGAAC
AAGAGAGACTGATGAGATAGTGAACCGCTTTATGAACCGTCTACTGTTTCTCACGGGCAGGAAGTTACCATTGATACTTCAGTTCGAAATTCTG
AAGTGGCGGTTTTAGAGAGGGATGAGGTGGTCAGGTGTATTGAGCATAGAGCGAGGGCATTTCAGGGGTGGAGGGGCGAGATGGGGATTGAGAA
GTTGAGGACGCAGAGGTATGGGGTTGGAGGACATTATGGGATGCATTTGTAAGTTTTGGGGGATTGACGAAGGCTTTTGTCTATTTTCTACCAG
TACGGATCTTGAAAGAAAGATAGCATGAACACGAGGGCTAATAACTAGGAAATAGCGATTGGAGCGGAGGTAAACGTGGCATAGACCGATTTAG
TACTTTCATGGTCTATGTCGACGTATCCTCTGATATCGAAGGTGGAGGAACGGAATTCCCACGTATCGTGGGACCAAAAGGAGGAAGGTGGGAG
GACTTCCTGGAAACTACGGAAGCATTGGATCCAAGAACTGGAGAAAATGTAACAGTAGAAGGGGTGACATTCAAACCAATCAAGGGAAATGCCG
TATTCTGGGAAAATACTGACAACAACGGGAGGGGCTATGATG

>Bcin12g03020(MLST8), partial sequence [organism=Botrytis cinerea, strain D10_B_F1_6]
TCGATTGGCTGCGAAGAAAACTGCGCAGCACCGAGTCACCAAAAGCACGAATAAACCATCGGCAAGGGAAATATTCTCTGAACCACAACATGAC
ACGAGCGCGGAAGAGTATATCGGGCGAGAAGCTTCATCAAGAGCACCAAAGCGACAACGAGTCGATGATAATTATAACTCTTACGGTGGAAGAA
ATGAAAATACAGCAGCTTATGCTTCCGGGAAACTTCCATCTGGAAGTATAAATGTTGGTGGAGGTAGGAAGACCACTTTTCAAGAAGAACCTCG
AACGGCATTTGTCGCTGGCAAGTTGCCCCCTGGAAGTATCAACATTGGTGGAAAGAAGGCTACCCAAATCGAGGACGAGGGTAGAGCAGCTTAT
GCTTCCGGAAAATTGCCCCCAGGAAGTATAAACTCTGGCGCAAAGAAGGCGATTTCATTCCAAGATGAAACGAGACCGGCTTATGTCTCCGGAA
AGCTTCCACATGGTAGTATCGACGGTATGCGAAACCGTGAAATGGCTGCCGTCCACCGCGAAATTGCTGAGGGTGGGAGGAAACCAGGCCAGGT
TGTCTCTTCTCTATTCACATTCAATCCTACTTCAAAGAAAACTTTCGATGAACCAGAAGAACAAGCGGAACCGGCAAAACCATCCAATGCGCCT
TTGACAGAGGAAATGGCGACTTTCACCAATCTAGGGCTATCGAGAAGGCTTGCAGCCCATCTATCGACTAAACTCGATATGAAAGCCCCGACGG
CCATTCAAAAAGCATCTGTGCAGCAGTTGGTATCGGACGATAGCGATGCTTTCATACAAGCAGAGACTGGATCTGGAAAAACTTTGGCATATCT
ACTACCTATAGTCGAGCGAATATTAGCATTGAGTGAGAATGGCGTACAAA

>Bcin02g07770(MLST9), partial sequence [organism=Botrytis cinerea, strain D10_B_F1_6]
CAGCTTTCCCTTTCGGTCTTGGCATCTACAGTCATTGCCATCCCTACACCATCACAACTTGAGTCTCGGGCCGTTATCGATTCCGATGCCGTTG
TAGGATTTGCCGAAACTGTTCCCAGTGGGACCGTAGGAACAGTTTATGAGGCATATAAACCATTCCTTAAAGTCGTAAATGGATGCGTACCATT
CCCTGCCGTCGATGCATCGGGTAACACAGGGTATGTCCTTATACCTTTCTCTTCCACACGATTGCTATTGAGTCTCTAACATATTTTAGTGGTG
GTTTGTCACCAACTGGCAGCAGCAATGGTGAATGCAGCAGCAGTACCGGTCAAGTATATGTTCGAGGAGGACAAAGCGGATCAAACTACGCTAT
CATGTACTCCTGGTAAGTTCTCTTTAAACTTCTCCTTATAGATCCAACCTAACAAAATCTTAGGTACATGCCAAAGGACGAGCCCTCAACCGGT
ATTGGTCACCGTCACGATTGGGAAGGTGTAATTGTCTGGCTCTCCAGCGCCACCGCCACAACTGCCGACAACATCTTAGCCGTTTGTCCTTCCG
CCCACGGAGGCTGGGATTGTTCCACGGATGGCTATTCCCTTTCTGGTACCAGCCCTCTTATCAAGTACGAAAGTATCTGGCCCGTCGATCACTC
AATGGGTCTTACTAGTACTGTTGGTGGAAAACAACCTATGATTGCTTGGGAGTCTTTACCAACTGCTGCTCAAACTGCTCTTGAGAACACCGAT
TTCGGTGCTGCGAATGTTCCATTCATTCCGGCTGTTTTCACAGATAATCTT

>Bcin04g02090(MLST10), partial sequence [organism=Botrytis cinerea, strain D10_B_F1_6]
CGGAGGATGATATGGCAAAGTCTATGATTACCAAAGCATTTGTAGGCATGAGTAGTAAATTGTCGAATATGACTATGAATGATGTTTACAAGCC
CTACATCCATGTAAGAAATGTAGAATAAAGAGATCAGTAACTGGAACTAATATCGTTTGCAGGCTTTCAAGTTACTTACGCAGTTCAACCCAAT
CACTACAGCTATTGCCGAATCCCCACTATTTCAAATGGCTGTCTCAGCAAATACCATCGAAAAGTACACACTGCTAGGCCCCTTTCTTCAGAATA
TCTCCTCTGCAACAGGAAGTTACCAGGGAATACTTCAGTGCGCCAAAGACGATAGATAGGCGACACATTGCCACATCTCAAGATGCGTTACGAT
TGACCTTACAAACCCATCAAAAAGATTTACTTGATATCATCAACCACTTTGTTCGAGCAAGTCCAATCGCAAAAAGCAAAACCCTGGATTGGTT
CGCCTACATTGTGAATCAAAATCACAAGCGTCGAGCACTTCAGGTAGACCCGAAAGAAGTGTCTTCTGATGGCTTTATGCACAATGTCACTGTC
GTTCTAGATGGTCTTTGTGAGCCATTCATGGATACCACATTCTCAAAAATTTCGAAGATTGATATTGATTATCTAAGACGTGCGCCTCGTGTAG
ATATCAAGGACGAGACCAAGTTGAACGCTGATGAGAAGGCTTCTGAGAAATATTATGAGGACACTGTTCCTGGCACTTCTAATTTCATCTCTGA
GGTATTCTTTCTGACATTGGCTGCTCATCATTATGGTAGTGAAGCTCTTAATGCCACGCATAAGAGTCTGGAGAAAGACATCAAATATATTCAA
AAGCAATTGACTGCCGTTGAAGCA

>Bcin01g07220(MLST1), partial sequence [organism=Botrytis cinerea, strain D10_B_F3_5]
ATGGCATATCTTGTTTCTTTTCGCATTATGCTTTGGAACGTGCTGGACAAGCTTGGGTTAGTGCTCTGCGTGTAGAAGCACTAAAGAAGATTCT
CGCACAACCGAAGTCATGGTTTGAGGAATCCAGGAATTCACCTGGCCGGTTGAACGAAGTTTTGGATAGGAACTCTGAGGAAATGCGTAATCTC

GTTGGCCGCTTTGCTGGTATTGTATTCACAGCATTTTTTATGCTATTGATATCAATCATTTGGGCTTTCGTGAATACATGGAAACTGACATTAG
TCTCAATGGCGACTGGGCCAGTTATATACGCTGTCACCAAAACGTTCAATCGCGTGAGTGGAAAATGGGAAAACAAGTGCAACTACGCATCTGA
AATGACCACTGGCATATTTTCAGAGACTTTCTCCAACATCAAAGTGGTTCGGGCTTTTACTCTGGAAACTTACTTTGAGACAAAACACACCAAA
GCTACAGAAGAACTCTATAAAATTGGACTAATACGAGCAAACTACTCGGGATTGCTGTGGGGATTGACAGATGCGATGTCATTCTTCATCACTG
CAACTATCTTTTATTATGCCACGGTTCTCATTACCAAGAGAGAGATCAGTATCGCGACTGCACTGCAGACTGTCAATCTTCTATTATTTGGTAT
TTCTAATAGTACGAATATGCTGGCCATGATACCACAAATCAACTCTTCTCGCGTTACAGCTACGCATATGCTTGCATTAGCCAATCTCGATTCA
TCTTCCTCCCACGAAAATAAAGGAGCCGAACGGCTTTCGACAATCTTTCCAATCAAATTCAACAGTCTCTCATTCACATATCCTACTCGTCCTG
AAAAACGAACGATATCATCCTTTTCTCTTTCCCTGATTCCTAACTCAACAACTGCACTTGTCGGACCCTCCGGCTCCGGAAAATCTACAATAGC
TGCTCTGCTCGTTGGTCTCTATCCGCCAGATACTTCAACACCTCCACCGTTGACATTCAATCGTGTCTCCATAAGTAACTGTCACATTCCGTCT
CTCCGGGCTTCTCTCTCACTCGTCCCACAAATACCGATTCTATTTCCAGCTACCATTCTCCATAACATCATTTATGGTCTCCCAGAATCTTCTC
CTTGTGCTAGTCTTCCATCTGCTATTGCATCAGCAAAAGATGCTGGGATCCATGAATTTATCACATCGCTTCCAAAAGGTTATGATACTATG

>Bcin05g07690(MLST2), partial sequence [organism=Botrytis cinerea, strain D10_B_F3_5]
CACCACTATCACCAAGTCCTTTTCTGCACTTTCCTCATCAAAAACGAACGAGAACATCATCAATAAATACTTTGCTGAAGTAATTGGGTTTTAT
TTTGGCCAGGATGCCTTCTCTCTACGACAGCAAGCCTTCGATGATATGTTGTGGGTAGTTCTTGGCTGGCTGGATACTGTCAAATTCATTGATT
TACATTCTGAATTGCACTATTCAAACGACTCTCAGCCAGAATGGTACGGACAACAATATAAACCTGCATTTGCACATCGAGCGCGACTATTTTG
GGAATTGGCTTCACAAGGATGGGATACTACTCTCTGTGGTGGTGGGATGATATGGTCACCATACCTTACTCCATACAAGAACGCAATTACCAAT
GAACTCTATATCGCAGCTTCGATATCGATGTACCTATATTTCCCCGGAGATGACAATCAATCCCCATTTATGATTTCCAACCCTTCATATCCAC
CTCACGATCCGAAATATCTACAGGCAGCTGTTGATGCTTACAAATGGCTGAATGGTTCCAACATGACGGATTCACAAGGATTATATGTCGACGG
GTACCATATCTCGAATCTTTCTGGCGGTGAAAACACCCATTGCGATTCTAGAAATGAGATGGTATATACCTACAATCAAGGTGTTTTGCTTACT
GGACAACGTGGTTTGTATGACGCAACCGCCGCACGATCATACCTTGTAGATGGCCACAAACTCATCGCGAATGTTATTAATGCCACAGGCTATG
ACCTGAAACACAATGTTGTCATCTCACCGCCACCCAAAGATGGTTCCGCATTGGCAAAGTGGTTTGGCCTGGGTAGGAATGGAATACTGGAAGA
AGGATGCGATTCAAGTGCTTCGTGTTCTCAAAATGGACAAACTTTCAAAGGCATATTCTTTCATCACTTGATTGCGTTCTGTAGTGATTTGCCA
GGGGAGCCTATTGCAGGGACGAAGGAAAGCTTAGAACTCGACAGAGTGTGGCATTCTGACAAATGCTCACAGTATACAAAATG

>Bcin06g01710(MLST3), partial sequence [organism=Botrytis cinerea, strain D10_B_F3_5]
GGTGAGTCTGACTTTTGTGTTTGAGCGTTAAGATAGACACTGATGTACCAAGGCAATCACCTAATTTCATGGCGTTCTTCCCTGCATCATCTAC
CTACCCTGGAATGCTGGGAGAATTATACTCAGCAGCTTTCACAGCACCTGCTTTCAATTGGATCTGTTCCCCTGCCGTGACAGAATTGGAGACG
GTTGTAATGGATTGGCTGGCCAAGCTTCTCAATCTCCCAGACTGTTATTTGTCTTCGACTCATGGTGGTGGTGTTATCCAAGGATCAGCCTCGG
AAGCTATCGTTACCGTTATGGTTGCTGCCCGCGATAAATATCTTCGTGAAACTACTGAAGGTCTGTCGGGCATTGAACTCGAGGATGCGATTGC
ATATAAGAGGAGTAAGCTAGTTGCACTAGGAAGCGAAATGGCACACAGCTCCACGCAGAAAGCAGCGCAGATAGCTGGCGTTAGATTCCGATCG
ATTCCAGTACTCGCATCCAATGATTTCGCCATGACGGGTGATGATTTAGAGAAGGTATTGGAAGAATGCAAATCTCAAGGATTGGAACCCTTCT
ATCTAACTTCGACTTTGGGAACAACATCTACATGCGCAGTTGACGACTTCGCATCTATTGCAACAGTACTTTCAAAATATGCACCTCCAGATGT
TGCAGGCGAGATCTGGGTTCACGTCGATGCTGCTTATGCAGGTGCAGCTTTGGTTTGCCCTGAATACCATCATCTAACATCGTCCTTCCAGCAT
TTCCATTCCTTCGATATGAACATGCACAAATGGCTTCTGACAAATTTCGACGCTTCTTGTCTATATGTCAAGAAACGCAAAGATCTGATAGATG
CACTCTCCATAACACCAAGTTATCTTCGCAACGAGTTTTCAGAGAGTGGACTCGTAACCGACTATCGGGATTGGCAAATTCCTCTCGGAAGACG
CTTCCGAAGCTTAAAGATTTGGTTTGTCCTCAGAACCTAC

>Bcin09g03030(MLST4), partial sequence [organism=Botrytis cinerea, strain D10_B_F3_5]
ACCCTTCAACCCCACCGCAGCTCCTATACCGAAAGCGAGTATCCTACCAATTCTCCTTCCACCTGCGACTTTAAGACCATTGGCTTTCCGCACT
TTCACAAAAAAGCATAGTTTAACATTGACGTCGTCGGCATTACAAGTGTTGGCTACTTTTATTGGAAAGCATTGTGGGACAGGATGGAGGGAAG
AAGGACTGGCAGAGAGAGTCTTGGAGGAGGTCGCCAAGAGTTGGAAGAATAGGAGTGGCGGTGTCATTGTCGAGGGCGAGGGAACGGAATTGAA
GGAGATTCTGAAAGCTTTGGAAGGGAATATGAGTGGTGGAAGGATAGTCATAGGAAGAGAGCTAAGCCGGCAGAATAGTTTAGTACTGGGATCA
TCACAATATGGAGAGGTCAATCATACAAGACTTGGGCTACGGCCAGGGAATATACCCAGAGAGGATAGTCAGTCAAGTTTGGGAATGTCAACGT
TGGAGGTCAATGACGAGGAGGATGAGGATGGCCTGATGGATCCAAGAAGGTGGTTAAAAGTCATTGATGCATTTGAGCAACCTCGACTGGTGTA
CAATGTTGCTAAAAAGCACTTTGATAGGTATGTTTCAATGATAAAATTTTATCTGAATCTACTAACTCAGTACAGAGATACCTCCAAACCTTCA
TTGTTCCCACCTGCGTCTCATAAAACACTCCTCTTCCAAAACCGCTATAATGTTATCCATCAACGTCTCCTTCGCAATGAATCTTTTCAAACGC
CCGCTTTTCAAGGTGGCAAATCTTCCCTTCAACGCAGCACGTCCGCCATTACCACCAACAACAATCATACAAATTAACGCCGATAGCTAATCTT
CTCGGTCGCAATCGCAGCTCTCATATGCTTCTCGGTCTCCTCAGTATTTCACCCACTGGTACCCTCGCCATCAATGACCTGACGGGCAGTATCG
CTCTCGATCTTACACACGCAGCAGCCATTC

>Bcin11g01310(MLST5), partial sequence [organism=Botrytis cinerea, strain D10_B_F3_5]
GGCTGACATGGACCTCATGTGGAACCGGCGTAGAATGCGCAACGCTTGAAGTTCCGCTCGAATATGGCGATGCAACGTCAACGGCAAAAGCCAG
TGTTGCGCTTGCTCGTTATCCTGCCACTGTTGCCGCGAGCAAGAAGCTCGGGTCTCTCTTGATAAACCCCGGTGGACCCGGTGCCTCTGGTGTT
GGCTTTGTGCAGTCTGGAGCCGGTGCCGCCGTCTCGACACTGAGTGGTGGATTGTACGATATCATCGGATGGGATCCACGTGGAACCGGTGCTT
CGGCTCCTATTTTGGAATGTTTTGCAAATGCCAGTGCGGAGTATGATTTTAACAACGCGTTTCCATCTGCTCCGAATCTCTGGCTCGGACAATT
TGCGAATGCCAGCGCAAATTCTGCTGTTAGCTCTGCTATCACATCCTTTGACACTTCTGTCGCTGCTCTTGCAAAAGCTTGCGTGGCTCAGAAA

TCTCCCGCTCTTTACACCTCAACAGCAGCATATGTTGCTCGAGACATGGCAGCGATAGTCGATGCATTGGATGGGACCTCTGCAAAACTTAACT
ACTGGGGTTTCTCATATGGAACTATCTTCCTAGCTGAGTTTATCCAAACTTTCCCAGGCCGCGTGGGAAGAGTTCTTGCCGATGGTGTTTTCGA
CGCAAAGGCAAATGCACTCACATACGTTAGCCAACTTCCCAACGATCAACTCAGTGTTCGTGCTTCGTTGAACGATTTTGCAGCTTTCTGCACC
ACCGCCGGTAGTAAAGGTTGCTCTTTTGCCACCGCCCCTACTGGAACCTCAGGTACTGTTGCTACCAGACTGGACAACATAATGAAGGATATGT
TCCTCAATCCTATTGTTGCTTCGGGCTTAAGCATCA

>Bcin15g03910(MLST6), partial sequence [organism=Botrytis cinerea, strain D10_B_F3_5]
GCCAAAACACAAAATCATCCAACGATGAAGATGATACTCCACTTCCCTTGATTATCTGGCATGGACTCGGCGATAATTACAAAGCGGATGGTCT
TGCGCAAGTTGGAAAACTAGCTGAAGCTATTCATCCTGGGACTTTTGTCTACAATATTCATGTAGATGAGGATGCATCTGCAGATAGGACAGCT
ACCTTCTTTGGAAATCTCACTCGTGAGTACATCCCCTATTTTTCCTTTAAATATCATACTAACTCTCTTACCAAGTTCAAATCGAAAAGGTCTG
CGAAGACCTCGCCTCCCATCCTATTCTCTCTACCGCGCCCGCCGTCGACGCAATTGGATTCTCCCAAGGCGGCCAATTCTTGCGTGGTTACATA
TCCCGCTGCAATGCTCCACCCATCCGCTCTCTCCTGACCTTTGGTTCCCAACACAACGGCATTTCTGCCTTCCAAGCCTGTGGTCCTGCCGATT
TCCTCTGTCGCGGTGCTCAAACCCTTTTGCGATCCAACACCTGGTCAACCTTTGTCCAATCTCGTCTCGTACCCGCTCAATACTTCAGAGATCC
GGAAAACCTAGACTCTTACCTTGAATATTCCAATTTCCTTGCCGACATCAATAATGAGCGCGTTCTCAAGAACCAAACATATAAATCCAACATG
GAAAAATTGGAACGATTCGTAATGTATGTCTTTGAAGACGACACAACTGTCATTCCTAAGGAAAGTGGATGGTGGGCTGAAGTCAACGGCACGG
AAGTTACACCACTGAAAGAAAGAGCCATTTATAAAGAAGATTGGCTAGGTTTAAAGACATTGGATGAGGCCGGAAAATTAGTTTTCGAAACCAT
TCCAGGGGGACATATGACGTTAGGAGAGGAGATGCTAGAGAAGGCTTTCAAAGAGTATTTTGGTCCAGCAGGGAAGAAATTTGGG

>Bcin16g03460(MLST7), partial sequence [organism=Botrytis cinerea, strain D10_B_F3_5]
ATGAACCTCTTAATTTGAACTCTTCATTATTTGCAAGTGATGAACCCCTATTCTGCCCAGAGGATAGTTACAAGACGTATATCATTAGTCGAGA
ACCACTCATGATATATATTGACGGATTTTTGAAAGCGAATGAAAGTAAACATTTGGTTGATGTTAGGTGTGTTATTTATTCTGATGAAATGAAC
AAGAGAGACTGATGAGATAGTGAACCGCTTTATGAACCGTCTACTGTTTCTCACGGGCAGGAAGTTACCATTGATACTTCAGTTCGAAATTCTG
AAGTGGCGGTTTTAGAGAGGGATGAGGTGGTCAGGTGTATTGAGCATAGAGCGAGGGCATTTCAGGGGTGGAGGGGCGAGATGGGGATTGAGAA
GTTGAGGACGCAGAGGTATGGGGTTGGAGGACATTATGGGATGCATTTGTAAGTTTTGGGGGATTGACGAAGGCTTTTGTCTATTTTCTACCAG
TACGGATCTTGAAAGAAAGATAGCATGAACACGAGGGCTAATAACTAGGAAATAGCGATTGGAGCGGAGGTAAACGTGGCATAGACCGATTTAG
TACTTTCATGGTCTATGTCGACGTATCCTCTGATATCGAAGGTGGAGGAACGGAATTCCCACGTATCGTGGGACCAAAAGGAGGAAGGTGGGAG
GACTTCCTGGAAACTACGGAAGCATTGGATCCAAGAACTGGAGAAAATGTAACAGTAGAAGGGGTGACATTCAAACCAATCAAGGGAAATGCCG
TATTCTGGGAAAATACTGACAACAACGGGAGGGGCTATGAT

>Bcin12g03020(MLST8), partial sequence [organism=Botrytis cinerea, strain D10_B_F3_5]
CGATTGGCTGCGAAGAAAACTGCGCAGCACCGAGTCACCAAAAGCACGAATAAACCATCGGCAAGGGAAATATTCTCTGAACCACAACATGACA
CGAGCGCGGAAGAGTATATCGGGCGAGAAGCTTCATCAAGAGCACCAAAGCGACAACGAGTCGATGATAATTATAACTCTTACGGTGGAAGAAA
TGAAAATACAGCAGCTTATGCTTCCGGGAAACTTCCATCTGGAAGTATAAATGTTGGTGGAGGTAGGAAGACCACTTTTCAAGAAGAACCTCGA
ACGGCATTTGTCGCTGGCAAGTTGCCCCCTGGAAGTATCAACATTGGTGGAAAGAAGGCTACCCAAATCGAGGACGAGGGTAGAGCAGCTTATG
CTTCCGGAAAATTGCCCCCAGGAAGTATAAACTCTGGCGCAAAGAAGGCGATTTCATTCCAAGATGAAACGAGACCGGCTTATGTCTCCGGAAA
GCTTCCACATGGTAGTATCGACGGTATGCGAAACCGTGAAATGGCTGCCGTCCACCGCGAAATTGCTGAGGGTGGGAGGAAACCAGGCCAGGTT
GTCTCTTCTCTATTCACATTCAATCCTACTTCAAAGAAAACTTTCGATGAACCAGAAGAACAAGCGGAACCGGCAAAACCATCCAATGCGCCTT
TGACAGAGGAAATGGCGACTTTCACCAATCTAGGGCTATCGAGAAGGCTTGCAGCCCATCTATCGACTAAACTCGATATGAAAGCCCCGACGGC
CATTCAAAAAGCATCTGTGCAGCAGTTGGTATCGGACGATAGCGATGCTTTCATACAAGCAGAGACTGGATCTGGAAAAACTTTGGCATATCTA
CTACCTATAGTCGAGCGAATATTAGCATTGAGTGAGAATGGCGTACAAA

>Bcin02g07770(MLST9), partial sequence [organism=Botrytis cinerea, strain D10_B_F3_5]
CAGCTTTCCCTTTCGGTCTTGGCATCTACAGTCATTGCCATCCCTACACCCTCACAACTTGAGTCTCGGGCCGTTATCGATTCCGATGCCGTTG
TAGGATTTGCCGAAACTGTTCCCAGTGGGACCGTAGGAACAGTTTATGAGGCATATAAACCATTCCTTAAAGTCGTAAATGGATGCGTACCATT
CCCTGCCGTCGATGCATCGGGTAACACAGGGTATGTCCTTATACCTTTCTCTTCCACACGATTGCTATTGAGTCTCTAATATATTTAGTGGTG
GTTTGTCACCAACTGGCAGCAGCAATGGTGAATGCAGCAGCAGTACCGGTCAAGTATATGTTCGAGGAGGACAAAGCGGATCAAACTACGCTAT
CATGTACTCGTGGTAAGTTCTCTTTAAACTTCTCCTTATAGATCCAACCTAACAAAATCTTAGGTACATGCCAAAGGACGAGCCCTCAACCGGT
ATTGGTCACCGTCACGATTGGGAAGGTGTAATTGTCTGGCTCTCCAGCGCCACCGCCACAACTGCCGACAACATCTTAGCCGTTTGTCCTTCCG
CCCACGGAGGCTGGGATTGTTCCACGGATGGCTATTCCCTTTCTGGTACCAGCCCTCTTATCAAGTACGAAAGTATCTGGCCCGTCGATCACTC
AATGGGTCTTACTAGTACTGTTGGTGGAAAACAACCTATGATTGCTTGGGAGTCTTTACCAACTGCTGCTCAAACTGCTCTTGAGAACACCGAT
TTCGGTGCTGCGAATGTTCCATTCATTCCGGCTGTTTTCACAGATAATCTT

>Bcin04g02090(MLST10), partial sequence [organism=Botrytis cinerea, strain D10_B_F3_5]
CGGAGGATGATATGGCAAAGTCTATGATTACCAAAGCATTTGTAGGCATGAGTAGTAAATTGTCGAATATGACTATGAATGATGTTTACAAGCC
CTACATCCATGTAAGAAATGTAGAATAAAGAGATCAGTAACTGGAACTAATATCGTTTGCAGGCTTTCAAGTTACTTACGCAGTTCAACCCAAT
CACTACAGCTATTGCCGAATCCCCACTATTTCAAATGGCTGTCTCAGCAAATACCATCGAAAAGTACACACTGCTAGGCCCTTTCTTCAGAATA
TCTCCTCTGCAACAGGAAGTTACCAGGGAATACTTCAGTGCGCCAAAGACGATAGATAGGCGACACATTGCCACATCTCAAGATGCGTTACGAT

TGACCTTACAAACCCATCAAAAAGATTTACTTGATATCATCAACCACTTTGTTCGAGCAAGTCCAATCGCAAAAAGCAAAACCCTGGATTGGTT
CGCCTACATTGTGAATCAAAATCACAAGCGTCGAGCACTTCAGGTAGACCCGAAAGAAGTGTCTTCTGATGGCTTTATGCACAATGTCACTGTC
GTTCTAGATGGTCTTTGTGAGCCATTCATGGATACCACATTCTCAAAAATTTCGAAGATTGATATTGATTATCTAAGACGTGCGCCTCGTGTAG
ATATCAAGGACGAGACCAAGTTGAACGCTGATGAGAAGGCTTCTGAGAAGTATTATGAGGACACTGTTCCTGGCACTTCTAATTTCATCTCTGA
GGTATTCTTTCTGACATTGGCTGCTCATCATTATGGTAGTGAAGCTCTTAATGCCACGCATAAGAGTCTGGAGAAAGACATCAAATATATTCAA
AAGCAATTGACTGCCGTTGAAGCA

>Bcin01g07220(MLST1), partial sequence [organism=Botrytis cinerea, strain D10_B_S6_1]
ATGGCATATCTTGTTTCTTTTCGCATTATGCTTTGGAACGTGCTGGGCAAGCTTGGGTTAGTGCTCTGCGTGTAGAAGCACTAAAGAAGATTCT
CGCACAACCGAAGTCATGGTTTGAGGAATCCAGGAATTCACCTAGCCGGTTGAACGAAGTTTTGGATAGGAACTCTGAGGAAATGCGTAATCTC
GTTGGCCGCTTTGCTGGTATTGTATTCACAGCATTTTTTATGCTATTGATATCAATCATTTGGGCTTTCGTGAATGCATGGAAACTGACATTAG
TCTCAATGGCAACTGGGCCAGTTATATACGCTGTCACCAAAACGTTCAATCGCGTGAGTGGAAAATGGGAAAACAAGTGCAACTACGCATCTGA
AATGACCACTGGCATATTTTCAGAGACTTTCTCCAACATCAAAGTGGTTCGGGCTTTTACTCTGGAAACTTACTTTGAGACAAAACACACCAAA
GCTACAGAAGAACTCTATAAAGTTGGACTAATACGAGCAAACTACTCGGGATTGCTGTGGGGATTGACAGATGCGATGTCATTCTTCATCACTG
CAACTATCTTTTATTATGCCACGGTTCTCATTACCAAGAGAGAGATCAGTATTGCGACTGCACTACAGACTGTCAATCTTCTATTATTTGGTAT
TTCTAATAGTACGAATATGCTGGCTATGATACCACAAATCAACTCTTCTCGCGTTACAGCTACGCATATGCTTGCATTAGCCAATCTCGATTCA
TCTTCCTCCCACGAAAATAAAGGAACCGAACGGCTTTCGACAATCTTTCCAATCAAATTCAACCGTCTTTCATTCACATACCCTACTCGTCCTG
AAAAACGAACGATATCATCCTTTTCTCTTTCCCTGATTCCTAACTCAACAACTACACTTGTCGGACCCTCCGGCTCCGGAAAATCTACGATAGC
TGCTCTGCTCATTGGTCTCTATCCGCCAGATACTTCAACACCTCCACCGTTGACATTCAATCGCGTCTCCATAAGTAACTGTCACATTCCGTCT
CTCCGGGCTTCTCTCTCACTCGTCCCACAAATACCGATTCTATTTCCAGCTACCATTCTCCATAACATCATTTATGGCCTCCCAGAATCTTCTC
CTTGTGCTAGTCTTCCATCTGCTATTGCATCAGCAAAAGATGCTGGGATCCATGAATTTATCACATCGCTTCCACAAGGTTATGATACTAT

>Bcin05g07690(MLST2), partial sequence [organism=Botrytis cinerea, strain D10_B_S6_1]
ACCACTATCACCAAGTCCTTTTCTGCACTTTCCTCATCAAAAACGAACGAGAACATCATCAATAAATACTTTGCTGAAGTAATTGGGTTTTATT
TTGGCCAGGATGCCTTCTCTCTACGACAGCAAGCCTTCGATGATATGTTGTGGGTAGTTCTTGGCTGGCTGGATACTGTCAAATTCATTGATTT
ACATTCTGAATTGCACTATTCAAACGACTCTCAGCCAGAATGGTACGGACAACAATATAAACCTGCATTTGCACATCGAGCGCGACTATTTTGG
GAATTGGCTTCACAAGGATGGGATACTACTCTCTGTGGTGGTGGGATGATATGGTCACCATACCTTACTCCATACAAGAATGCAATTACCAATG
AACTCTATATCGCAGCTTCGATATCGATGTACCTCTATTTCCCCGGAGATGACAATCAATCCCCATTTATGCTTTCCAACCCTTCATATCCACC
TCACGATCCGAAATATCTACAGGCAGCTGTTGATGCTTACAAATGGCTGAATGGTTCCAACATGACGGATTTACAAGGATTATATGTCGACGGG
TATCATATCTCGAATCTTTCTGGCGGTGAAAACACCCATTGCGATTCTAGAAATGAGATGGTATATACCTACAATCAAGGTGTTTTGCTTACTG
GACAACGTGGTTTGTATGACGCAACCGCCGCACGATCATACCTTGTGGATGGCCACAAACTCATCGCGAATGTTATTAATGCCACAGGCTATGA
CCTGAAACACAATGTTGCCATCTCACCGCCACCCAAAGATGGTTCCGCATTGGCAAAGTGGTTTGGCCTGGGTAGGAATGGAATACTGGAAGAA
GGATGCGATTCAAGTGCTTCGTGTTCTCAAAATGGACAAACTTTCAAAGGCATATTCTTTCACCACTTGATTGCGTTCTGTAGTGATTTGCCAG
GGGAGCCTATTGCAGGGACGAAGGAAGGCCTAGAACTCGACAGAGTGTGGCATTCTGACAAATGCTCACAGTATACAAAATG

>Bcin06g01710(MLST3), partial sequence [organism=Botrytis cinerea, strain D10_B_S6_1]
GTGAGTCTGACTTTTGTATTTGAGCGTTAAGATGGACACTGATGTACCAAGGCAATCACCTAATTTCATGGCGTTCTTCCCTGCATCATCTACC
TACCCTGGAATGCTGGGAGAATTATACTCAGCAGCTTTCACAGCTCCTGCTTTCAATTGGATCTGTTCCCCTGCTGTGACAGAATTGGAGACGG
TTGTAATGGATTGGTTGGCCAAGCTTCTCAATCTCCCAGACTGTTATTTGTCGTCGACTCATGGTGGTGGTGTTATCCAAGGATCAGCCTCGGA
AGCTATCGTTACCGTTATGGTTGCTGCCCGCGATAAGTATCTTCGTGAAACCACTGAAGGTCTGTCGGGAATTGAACTCGAGGATGCGATTGCA
TATAAGAGGAGTAAGCTAGTTGCACTAGGAAGCGAAATGGCACACAGCTCCACGCAGAAAGCAGCGCAGATAGCTGGCGTTAGATTCCGATCGA
TTCCAGTACTCGCATCCAATGATTTCGCCATGACGGGTGATGATTTAGAGAAGGTATTGAAAGAATGCAAATCTCAAGGATTGGAACCCTTCTA
TCTAACTTCGACGTTGGGAACAACATCTACATGCGCAGTTGACGACTTCGCATCTATTGCAACAGTACTTTCAAAATATGCACCTCCAGATGTT
GCAGGCGAGATCTGGGTTCACGTCGATGCTGCTTATGCAGGTGCAGCTTTGGTTTGCCCTGAATACCATCATCTAACATCGTCCTTCCAGCATT
TCCATTCCTTTGATATGAACATGCACAAATGGCTTCTGACAAATTTCGACGCTTCTTGCCTATATGTCAAGAAACGCAAAGATCTGATCGATGC
ACTCTCCATAACACCAAGTTATCTTCGCAACGAGTTTTCAGAGAGTAGACTCGTAACCGACTATCGGGACTGGCAAATTCCTCTCGGAAGACGT
TTCCGAAGCTTAAAGATTTGGTTTGTCCTCAGAACCTAC

>Bcin09g03030(MLST4), partial sequence [organism=Botrytis cinerea, strain D10_B_S6_1]
GACCCTTCAACCCCACCGCAGCTCCTATACCGAAAGCGAGTATCCTACCAATTCTCCTTCCACCTGCGACTTTAAGACCATTGGCTTTTCGCAC
TTTCACAAAAAAGCATAGTTTAACATTGACGTCGTCGGCATTACAAGTGTTGGCTACTTTTATTGGAAAGCATTGTGGGACAGGATGGAGGGAG
GAAGGACTGGCAGAGAGAGTCTTGGAGGAGGTCGCCAAGAGTTGGAAGAATAGGAGTGGCGGTGTCATTGTCGAGGGCGAGGGAACGGAATTGA
AGGAGATTCTGAAAGCTTTGGAAGGGAATATGAGTGGTGGAAGGATAGTCATAGGAAGAGAGCTAAGCCGGCAGAATAGTTTAGTACTGGGATC
ATCACAATATGGAGAGGTCAATCATACAAGACTTGGGCTACGGCCAGGGATATACCCAGAGAGGATAGTCAGTCAAGTTTGGGAATGTCAACGT
TGGAGGTCAATGACGAGGAAGATGAGGATGGCCTGATGGATCCAAGAAGGTGGTTAAAAGTCGTTGATGCATTTGAGCAACCTCGACTGGTGTA
CAATGTTGCTAAAAAGCACTTTGATAGGTATGTTTCAATGATAAAATTTTATCTGAATCGACTAACTCAGTACAGAGATACCTCCAAACCTTCA
TTGTTCCCACCTGCGTCTCATAAAACACTCCTCTTCCAAAACCGCTATAATGTTATCCATCAACGTCTCCTTCGCAATGAATCTTTTCAAACGC

CCGCTTTTCAAGGTGGCAAATCTTCCCTTCAACGCAGCACGTCCGCCATTACCACCCAACAACAATCATACAAATTAACGCCGATAGCTAATCT
TCTCGGTCGCAATCGCAGCTCTCATATGCTTCTCGGTCTCCTCAGTATTTCGCCCACTGGTACCCTCGCCATCAATGACCTGACGGGCAGTATC
GCTCTTGATCTTACACACGCAGCAGCCATTCCCG

>Bcin11g01310(MLST5), partial sequence [organism=Botrytis cinerea, strain D10_B_S6_1]
ACTGACATGGACCTCATGTGGAACCGGCGTAGAATGCGCAACGCTTGAAGTTCCGCTCGAATATGGCGATGCAACGTCAACGGCAAAAGCCAGT
GTTGCGCTTGCTCGTTATCCTGCCACTGTTGCCGCGAGCAAGAAGCTCGGGTCTCTCTTGATAAATCCCGGTGGACCCGGTGCCTCTGGTGTTG
GCTTTGTGCAGTCTGGAGCCGGTGCCGCCGTCTCGACACTGAGTGGTGGATTATACGATATCATCGGATGGGATCCACGTGGAACCGGTGCTTC
GGCTCCTATTTTGGAATGTTTTGCAAATGCCAGTGCGGAGTATGATTTTAACAACGCGTTTCCATCTGCTCCGAATCTCTGGCTCGGACAATTT
GCGAATGCCAGCGCAAATTCTGCTGTTAGCTCTGCTATCACATCCTTTGACACTTCTGTCGCTGCTCTTGCAAAAGCTTGCGTGGCTCAGAAAT
CTCCCGCTCTTTACACCTCAACAGCAGCATATGTTGCTCGAGACATGGCGGCGATAGTCGATGCATTGGATGGGACCTCTGCAAAACTTAACTA
CTGGGGTTTCTCATATGGAACTATCTTCCTAGCTGAGTTTATCCAAACTTTCCCAGGCCGCGTGGGAAGAGTTCTTGCCGATGGTGTTTTCGAC
GCAAAGGCAAATGCACTCACATACGTTAGCCAACTTCCCAACGATCAACTCAGTGTTCGTGCTTCGTTGAACGATTTTGCAGCTTTCTGCACCA
CCGCCGGTAGTAAAGGTTGCTCTTTGCCACCGCCCCTACTGGAACCTCAGGTACTGTTGCTACCAGACTGGACAACATAATGAAGGATATGTTC
CTCAATCCTATTGTTGCTTCGGGCTTGAGCATCA

>Bcin15g03910(MLST6), partial sequence [organism=Botrytis cinerea, strain D10_B_S6_1]
GCCAAAACACAAAATCATCCAACGATGAAGATGATACTCCACTTCCCTTGATTATCTGGCATGGACTCGGCGATAATTACAAAGCGGATGGTCT
TGCGCAAGTTGGAAAACTAGCTGAAGCTATTCATCCTGGGACTTTTGTCTATAATATTCATGTAGATGAGGATGCATCTGCAGATAGGACAGCT
ACCTTCTTTGGAAATCTCACTCGTGAGTACATCCCCTATTTTTCCTTTAAATACCATACTAACTCTCTTACCAAGTTCAAATCGAAAAGGTCTG
CGAAGACCTCGCCTCCCATCCTATTCTCTCTACCGCGCCCGCCGTCGACGCAATTGGATTCTCCCAAGGCGGCCAATTCTTGCGCGGTTACATA
TCCCGCTGCAATGCTCCACCCATCCGCTCTCTCCTGACCTTCGGTTCCCAACACAACGGCATTTCTGCCTTCCAAGCCTGTGGTCCTGCCGATT
TCCTCTGTCGCGGTGCTCAAACCCTTTTGCGATCCAACACCTGGTCAACCTTTGTCCAATCTCGTCTCGTACCTGCTCAATACTTCAGAGATCC
GGAAAACCTAGACTCTTACCTTGAATATTCCAATTTCCTTGCCGACATCAATAATGAGCGCGTTCTCAAGAACCAAACATATAAATCCAACATG
GAAAAATTGGAACGATTCGTAATGTATGTCTTTGAAGACGATACAACTGTCATCCCTAAGGAAAGTGGATGGTGGGCTGAAGTCAATGGCACGG
AAGTTACACCACTGAAAGAAAGAGCCATTTATAAAGAAGATTGGCTAGGGTTAAAGACATTGGATGAGGCCGGAAAATTAGTTTTCGAAACCAT
TCCAGGGGGACATATGACGTTAGGAGAGGAGATGCTAGAGAAGGCTTTCAAAGAGTACTTTGGTCCAGCAGGGAAAAAATTTGGG

>Bcin16g03460(MLST7), partial sequence [organism=Botrytis cinerea, strain D10_B_S6_1]
ATGAACCTCTTAATTTGAACTCTTCATTAATTGCAAGTGATGAACCCCTATTCTGCCCAGAGGATAGTTACAAGACGTATATCATTAGTCGAGA
ACCACTCATGATATACATTGACGGATTTTTGAAAGCGAATGAAAGTAAACACTTGGTTGATGTTAGGTGTGTTATTTATTCTGATGAAATGAAC
AAGAGAGACTGATGAGATAGTGAACCGCTTTATGAACCGTCTACTGTTTCTCACGGACAGGAAGTTACCATTGATCCTTCGGTTCGAAATTCTG
AAGTGGCGGTTTTAGAGAGGGATGAGGTGGTCAGGTGTATTGAGCATAGAGCGAGGGCATTTCAGGGGTGGAGGGGCGAGATGGGGATTGAGAA
GTTGAGGACGCAGAGGTATGGGGTTGGAGGACATTATGGGATGCATTTGTAAGTTTTGGGGGATTGACGAAGGCCTCTGTTTATTTTCTATCAG
TACGGATCTTGAAAGAAAGATAGCATGAACACGAGGGCTAATAACTAGGAAATAGCGATTGGAGCGGAGGTAAACGTGGCATAGACCGATTTAG
TACTTTCATGGTCTATGTCGACGTATCCTCTGATATCGAAGGTGGAGGAACGGAATTCCCACGTATTGTGGGACCAAAAGGAGGAAGGTGGGAG
GACTTCCTGGAAACTACGGAAGCATTGGATCCAAGAACTGGAGAAAATGTAACAGTAGAAGGGGTGACATTCAAACCAATCAAGGGAAATGCCG
TATTCTGGGAAAATACTGACAACAACGGGAGGGGCTATGAT

>Bcin12g03020(MLST8), partial sequence [organism=Botrytis cinerea, strain D10_B_S6_1]
TCGATTGGCTGCGAAGAAAACTGCGCAGCACCGAGTCACCAAAAGCACGAATAAACCATCGGCAAGGGAAATATTCTCTGAACCACAACATGAC
ACGAGCGCGGAAGAGTATATCGGGCGAGAAGCTTCATCAAGAGCACCAAAGCGACAACGAGTCGATGATAATTATAACTCTTACGGTGGAAGAA
ATGAGAATACAGCAGCTTATGCTTCCGGGAAACTTCCATCTGGAAGTATAAATGTTGGTGGAGGTAGGAAGACCACTTTTCAAGAAGAACCTCG
AACGGCATTTGTCGCTGGCAAGTTGCCCCCTGGAAGTATCAACATTGGTGGAAAGAAGGCTACCCAAATCGAGGACGAGGGTAGAGCAGCTTAT
GCTTCCGGAAAATTGCCCCCAGGAAGTATAAACTCTGGCGCAAAGAAGGCGATTTCATTCCAAGATGAAACGAGACCGGCTTATGTCTCTGGAA
AGCTTCCACATGGTAGTATCGACGGTATGCGAAACCGTGAAATGGCTGCCGTCCACCGCGAAATTGCTGAGGGTGGGAGGAAACCAGGCCAGGT
TGTCTCTTCTCTATTCACATTCAATCCTACTTCAAAGAAAACTTTCGATGAACCCGAAGAACAAGCGGAACCGGCAAAACCATCCAATGCACCT
TTGACAGAGGAAATGGCGACTTTCACCAATCTAGGGTTATCGAGAAGGCTTGCAGCCCATCTATCGACTAAACTCGATATGAAAGCCCCGACCG
CCATTCAAAAAGCATCTGTGCAGCAGTTGGTATCGGACGATAGCGATGCTTTCATACAAGCAGAGACTGGATCTGGAAAAACTTTGGCATATCT
ACTACCTATAGTCGAGCGAATATTAGCATTGAGTGAGAATGGCGTACAAA

>Bcin02g07770(MLST9), partial sequence [organism=Botrytis cinerea, strain D10_B_S6_1]
CAGCTTTCCCTTTCGGTCTTAGCATCTACAGTCATTGCCATCCCTACACCATCACAACTTGAGTCTCGGGCCGTTATCGATTCCGATGCCGTTG
TAGGATTTGCCGAAACTGTTCCCAGTGGGACCGTAGGAACAGTTTATGAGGCATATAAACCATTCCTTAAAGTCGTAAATGGATGCGTACCATT
CCCTGCCGTCGATGCATCGGGTAACACAGGGTATGTCCTTATACCATTCTCTTCCACACGATTGCTATTGAGTCTCTAACATATTTTAGTGGTG
GTTTGTCACCAACTGGCAGTAGCAATGGTGGTTGCAGCAGCAGTACCGGTCAAGTATATGTTCGAGGAGGACAAAGCGGATCAAACTACGCCAT
CATGTACTCCTGGTAAGTTCTCTCTAAACTTCTCCTTATAGATCCAACCTAACAAAATCTTAGGTACATGCCAAAGGACGAGCCCTCAACCGGT

ATTGGTCACCGTCACGATTGGGAAGGTGTAATTGTCTGGCTCTCAAGCGCCACCGCCACAACTGCCGACAACATCTTAGCCGTTTGTCCTTCCG
CCCACGGAGGCTGGGATTGTTCCACCGATGGATATTCCCTTTCTGGTACCAGCCCTCTTATCAAGTACGAAAGTATCTGGCCCGTCGATCATTC
AATGGGTCTTACTAGTACTGTTGGTGGAAAACAACCTATGATTGCTTGGGAGTCTTTACCAACTGCTGCTCAAACTGCTCTTGAGAACACCGAT
TTCGGTGCTGCGAATGTTCCATTCATTCCGGCTGTTTTCACAGATAATCTT

>Bcin04g02090(MLST10), partial sequence [organism=Botrytis cinerea, strain D10_B_S6_1]
CGGAGGATGATATGGCAAAGTCTATGATTACCAAAGCATTTGTAGGCATGAGTAGTAAACTGTCGAATATGACTATGAATGATGTTTACAAGCC
CTACATCCATGTAAGAAATGTAGAATAGAGAGATCAGTAACTGGAACTAATATCGTTTGTAGGCTTTCAAGTTACTTACGCAGTTCAACCCAAT
CACTACAGCTATTGCCGAATCCCCACTATTTCAAATGGCTGTCTCAGCAAATACCATCGAAAAGTACACACTGCTAGGCCCTTTCTTCAGAATA
TCTCCTCTGCAACAGGAAGTTACCAGGGAATACTTCAGTGCGCCAAAGACGATAGATAGACGACACATTGCCACATCTCAAGATGCGTTACGAT
TAACCTTACAAACCCATCAAAAAGATTTACTTGATATCATCAACCACTTTGTTCGAGCAAGTCCAATCGCAAAAAGCAAAACCCTGGATTGGTT
CGCCTACATTGTGAATCAAAATCACAAACGTCGAGCACTTCAGGTAGACCCGAAAGAAGTGTCTTCTGATGGCTTTATGCACAATGTCACTGTC
GTTCTAGATGGTCTTTGTGAGCCATTCATGGATACCACATTCTCGAAAATTTCGAAGATTGATATTGATTATCTAAGACGTGCGCCTCGTGTAG
ATATCAAGGACGAGACCAAGTTGAACGCTGATGAGAAGGCTTCTGAGAAGTATTATGAGGACACTGTTCCTGGCACTTCTAATTTCATCTCTGA
GGTCTTCTTTCTGACATTGGCTGCTCATCATTATGGTAGTGAAGCTCTTAATGCCACGCATAAGAGTCTGGAGAAAGACATCAAATATATTCAA
AAGCAATTGACTGCCGTTGAAGCA

>Bcin01g07220(MLST1), partial sequence [organism=Botrytis cinerea, strain D10_K_S11_06]
GATGGCACATCTTGTTTCTTTTCGCATTATGCTTTGGAACGTGCTGGGCAAGCTTGGGTTAGTGCTCTGCGTGTAGAAGCACTAAAGAAGATTC
TCGCACAACCGAAGTCATGGTTTGAGGAATCCAGGAATTCACCTAGCCGGTTGAACGAAGTTTTGGATAGGAACTCTGAGGAAATGCGTAATCT
CGTTGGCCGCTTTGCTGGTATTGTATTCACAGCATTTTTTATGCTATTGATATCAATCATTTGGGCTTTCGTGAATACATGGAAACTGACATTA
GTCTCAATGGCAACTGGGCCAGTTATATACGCTGTCACCAAAACGTTCAATCGCGTGAGTGGAAAATGGGAGAACAAGTGCAACTACGCATCTG
AAATGACCACTGGCATATTTTCAGAGACTTTCTCCAACATCAAAGTGGTTCGGGCTTTTACTCTGGAAACTTACTTTGAGACAAAACACACCAA
AGCTACAGAAGAACTCTATAAAGTTGGACTAATACGAGCAAACTACTCGGGATTGCTGTGGGGATTGACAGATGCGATGTCATTCTTCATCACT
GCAACTATCTTTTATTATGCCACGGTTCTCATTACCAAGAGAGAGATCAGTATTGCGACTGCACTACAGACTGTCAATCTTCTATTATTTGGTA
TTTCTAATAGTACGAATATGCTGGCATGATACCACAAATCAACTCTTCTCGCGTTACAGCTACGCATATGCTTGCATTAGCCAATCTCGATTCA
TCTTCCTCCCACGAAAATAAAGGAACCGAACGGCTTTCGACAATCTTTCCAATCAAATTCAACCGTCTTTCATTCACATACCCTACTCGTCCTG
AAAAACGAACGATATCATCCTTTTCTCTTTCCCTGATTCCTAACTCAACAACTGCACTTGTCGGACCCTCCGGCTCCGGAAAATCTACGATAGC
TGCTCTGCTCATTGGTCTCTATCCGCCAGATACTTCAACACCTCCACCGTTGACATTCAATCGCGTCTCCATAAGTAACTGTCACATTCCGTCT
CTCCGGGCTTCTCTCACTCGTCCCACAAATACCGATTCTATTTCCAGCTACCATTCTCCATAACATCATTTATGGTCTCCCAGAATCTTCTCCT
TGTGCTAGTCTTCCATCTGCTATTGCATCAGCAAAAGATGCTGGGATCCATGAATTTATCACATCGCTTCCACAAGGTTATGATACTAT

>Bcin05g07690(MLST2), partial sequence [organism=Botrytis cinerea, strain D10_K_S11_06]
CACCACTATCACCAAGTCCTTTTCTGCACTTTCCTCATCAAAAACGAACGAGAACATCATCAATAAATACTTTGCTGAAGTAATTGGGTTTTAT
TTTGGCCAGGATGCCTTCTCTCTACGACAGCAAGCCTTCGATGATATGTTGTGGGTAGTTCTTGGCTGGCTGGATACTGTCAAATTCATTGATT
TACATTCTGAATTGCACTATTCAAACGACTCTCAGCCAGAATGGTACGGACAACAATATAAACCTGCATTTGCACATCGAGCGCGACTATTTTG
GGAATTGGCTTCACAAGGATGGGATACTACTCTCTGTGGTGGTGGGATGATATGGTCACCATACCTTACTCCATACAAGAATGCAATTACCAAT
GAACTCTATATCGCAGCTTCGATATCGATGTACCTCTATTTCCCCGGAGATGACAATCAATCCCCATTTATGCTTTCCAACCCTTCATATCCAC
CTCACGATCCGAAATATCTACAGGCAGCTGTTGATGCTTACAAATGGCTGAATGGTTCCAACATGACGGATTTACAAGGATTATATGTCGACGG
GTATCATATCTCGAATCTTTCTGGCGGTGAAAACACCCATTGCGATTCTAGAAATGAGATGGTATATACCTACAATCAAGGTGTTTTGCTTACT
GGACAACGTGGTTTGTATGACGCAACCGCCGCACGATCATACCTTGTGGATGGCCACAAACTCATCGCGAATGTTATTAATGCCACAGGCTATG
ACCTGAAACACAATGTTGCCATCTCACCGCCACCCAAAGATGGTTCCGCATTGGCAAAGTGGTTTGGCCTGGGTAGGAATGGAATACTGGAAGA
AGGATGCGATTCAAGTGCTTCGTGTTCTCAAAATGGACAAACTTTCAAAGGCATATTCTTTCACCACTTGATTGCGTTCTGTAGTGATTTGCCA
GGGGAGCCTATTGCAGGGACGAAGGAAGGCCTAGAACTCGACAGAGTGTGGCATTCTGACAAATGCTCACAGTATACAAAATGGA

>Bcin06g01710(MLST3), partial sequence [organism=Botrytis cinerea, strain D10_K_S11_06]
GGTGAGTCTGACTTTTGTATTTGAGCGTTAAGATGGACACTGATGTACCAAGGCAATCACCTAATTTCATGGCGTTCTTCCCTGCATCATCTAC
CTACCCTGGAATGCTGGGAGAATTATACTCAGCAGCTTTCACAGCTCCTGCTTTCAATTGGATCTGTTCCCCTGCTGTGACAGAATTGGAGACG
GTTGTAATGGATTGGTTGGCCAAGCTTCTCAATCTCCCAGACTGTTATTTGTCGTCGACTCATGGTGGTGGTGTTATCCAAGGATCAGCCTCGG
AAGCTATCGTTACCGTTATGGTTGCTGCCCGCGATAAGTATCTTCGTGAAACCACTGAAGGTCTGTCGGGAATTGAACTCGAGGATGCGATTGC
ATATAAGAGGAGTAAGCTAGTTGCACTAGGAAGCGAAATGGCACACAGCTCCACGCAGAAAGCAGCGCAGATAGCTGGCGTTAGATTCCGATCG
ATTCCAGTACTCGCATCCAATGATTTCGCCATGACGGGTGATGATTTAGAGAAGGTATTGAAAGAATGCAAATCTCAAGGATTGGAACCCTTCT
ATCTAACTTCGACGTTGGGAACAACATCTACATGCGCAGTTGACGACTTCGCATCTATTGCAACAGTACTTTCAAAATATGCACCTCCAGATGT
TGCAGGCGAGATCTGGGTTCACGTCGATGCTGCTTATGCAGGTGCAGCTTTGGTTTGCCCTGAATACCATCATCTAACATCGTCCTTCCAGCAT
TTCCATTCCTTTGATATGAACATGCACAAATGGCTTCTGACAAATTTCGACGCTTCTTGCCTATATGTCAAGAAACGCAAAGATCTGATCGATG
CACTCTCCGTAACACCAAGTTATCTTCGCAACGAGTTTTCAGAGAGTAGACTCGTAACCGACTATCGGGACTGGCAAATTCCTCTCGGAAGACG
TTTCCGAAGCTTAAAGATTTGGTTTGTCCTCAGAACCTAC

>Bcin09g03030(MLST4), partial sequence [organism=Botrytis cinerea, strain D10_K_S11_06]
ACCCTTCAACCCCACCGCAGCTCCTATACCGAAAGCGAGTATCCTACCAATTCTCCTTCCACCTGCGACTTTAAGACCATTGGCTTTTCGCACC
TTCACAAAAAAGCATAGTTTAACATTGACGTCGTCGGCATTACAAGTGTTGGCTACTTTTATTGGAAAGCATTGTGGGACAGGATGGAGGGAGG
AAGGACTGGCAGAGAGAGTCTTGGAGGAGGTCGCCAAGAGTTGGAAGAATAGGAGTGGCGGTGTCATTGTCGAGGGCGAGGGAACGGAATTGAA
GGAGATTCTGAAAGCTTTGGAAGGGAATATGAGTGGTGGAAGGATAGTCATAGGAAGAGAGCTAAGCCGGCAGAATAGTTTAGTACTGGGATCA
TCACAATATGGAGAGGTCAATCATACAAGACTTGGGCTACGGCCAGGGAATATACCCAGAGAGGATAGTCAGTCAAGTTTGGGAATGTCAACGT
TGGAGGTCAATGACGAGGAAGATGAGGATGGCCTGATGGATCCAAGAAGGTGGTTAAAAGTCGTTGATGCATTTGAGCAACCTCGACTGGTGTA
CAATGTTGCTAAAAAGCACTTTGATAGGTATGTTTCAATGATAAAATTTTATCTGAATCGACTAACTCAGTACAGAGATACCTCCAAACCTTCA
TTGTTCCCACCTGCGTCTCATAAAACACTCCTCTTCCAAAACCGCTATAATGTTATCCATCAACGTCTCCTTCGCAATGAATCTTTTCAAACGC
CCGCTTTTCAAGGTGGCAAATCTTCCCTTCAACGCAGCACGTCCGCCATTACCACCCAACAACAATCATACAAATTAACGCCGATAGCTAATCT
TCTCGGTCGCAATCGCAGCTCTCATATGCTTCTCGGTCTCCTCAGTATTTCACCCACTGGTACCCTCGCCATCAATGACCTGACGGGCAGTATC
GCTCTTGATCTTACACACGCAGCAGCCATTC

>Bcin11g01310(MLST5), partial sequence [organism=Botrytis cinerea, strain D10_K_S11_06]
ACTGACATGGACCTCATGTGGAACCGGCGTAGAATGCGCAACGCTTGAAGTTCCGCTCGAATATGGCGATGCAACGTCAACGGCAAAAGCCAGT
GTTGCGCTTGCTCGTTATCCTGCCACTGTTGCCGCGAGCAAGAAGCTCGGGTCTCTCTTGATAAATCCCGGTGGACCCGGTGCCTCTGGTGTTG
GCTTTGTGCAGTCTGGAGCCGGTGCCGCCGTCTCGACACTGAGTGGTGGATTATACGATATCATCGGATGGGATCCACGTGGAACCGGTGCTTC
GGCTCCTATTTTGGAATGTTTTGCAAATGCCAGTGCGGAGTATGATTTTAACAACGCGTTTCCATCTGCTCCGAATCTCTGGCTCGGACAATTT
GCGAATGCCAGCGCAAATTCTGCTGTTAGCTCTGCTATCACATCCTTTGACACTTCTGTCGCTGCTCTTGCAAAAGCTTGCGTGGCTCAGAAAT
CTCCCGCTCTTTACACCTCAACAGCAGCATATGTTGCTCGAGACATGGCAGCGATAGTCGATGCATTGGATGGGACCTCTGCAAAACTTAACTA
CTGGGGTTTCTCATATGGAACTATCTTCCTAGCTGAGTTTATCCAAACTTTCCCAGGCCGCGTGGGAAGAGTTCTTGCCGATGGTGTTTTCGAC
GCAAAGGCAAATGCACTCACATACGTTAGCCAACTTCCCAACGATCAACTCAGTGTTCGTGCTTCGTTGAACGATTTTGCAGCTTTCTGCACCA
CCGCCGGTAGTAAAGGTTGCTCTTTTGCCACCGCCCCTACTGGAACCTCAGGTACTGTTGCTACCAGACTGGACAACATAATGAAGGATATGTT
CCTCAATCCTATTGTTGCTTCGGGCTTGAGCATCAG

>Bcin15g03910(MLST6), partial sequence [organism=Botrytis cinerea, strain D10_K_S11_06]
GCCAAAACACAAAATCATCCAACGATGAAGATGATACTCCACTTCCCTTGATTATCTGGCATGGACTCGGCGATAATTACAAAGCGGATGGTCT
TGCGCAAGTTGGAAAACTAGCTGAAGCTATTCATCCTGGGACTTTTGTCTATAATATTCATGTAGATGAGGATGCATCTGCAGATAGGACAGCT
ACCTTCTTTGGAAATCTCACTCGTGAGTACATCCCCTATTTTTCCTTTAAATACCATACTAACTCTCTTACCAAGTTCAAATCGAAAAGGTCTG
CGAAGACCTCGCCTCCCATCCTATTCTCTCTACCGCGCCCGCCGTCGACGCAATTGGATTCTCCCAAGGCGGCCAATTCTTGCGCGGTTACATA
TCCCGCTGCAATGCTCCACCCATCCGCTCTCTCCTGACCTTCGGTTCCCAACACAACGGCATTTCTGCCTTCCAAGCCTGTGGTCCTGCCGATT
TCCTCTGTCGCGGTGCTCAAACCCTTTTGCGATCCAACACCTGGTCAACCTTTGTCCAATCTCGTCTCGTACCTGCTCAATACTTCAGAGATCC
GGAAAACCTAGACTCTTACCTTGAATATTCCAATTTCCTTGCCGACATCAATAATGAGCGCGTTCTCAAGAACCAAACATATAAATCCAACATG
GAAAAATTGGAACGATTCGTAATGTATGTCTTTGAAGACGATACAACTGTCATCCCTAAGGAAAGTGGATGGTGGGCTGAAGTCAATGGCACGG
AAGTTACACCACTGAAAGAAAGAGCCATTTATAAAGAAGATTGGCTAGGGTTAAAGACATTGGATGAGGCCGGAAAATTAGTTTTCGAAACCAT
TCCAGGGGGACATATGACGTTAGGAGAGGAGATGCTAGAGAAGGCTTTCAAAGAGTACTTTGGTCCAGCAGGGAAAAAATTTGGG

>Bcin16g03460(MLST7), partial sequence [organism=Botrytis cinerea, strain D10_K_S11_06]
CATGAACCTCTTAATTTGAACTCTTCATTAATTGCAAGTGATGAACCCCTATTCTGCCCAGAGGATAGTTACAAGACGTATATCATTAGTCGAG
AACCACTCATGATATACATTGACGGATTTTTGAAAGCGAATGAAAGTAAACACTTGGTTGATGTTAGGTGTGTTATTTATTCTGATGAAATGAA
CAAGAGAGACTGATGAGATAGTGAACCGCTTTATGAACCGTCTACTGTTTCTCACGGACAGGAAGTTACCATTGATCCTTCGGTTCGAAATTCT
GAAGTGGCGGTTTTAGAGAGGGATGAGGTGGTCAGGTGTATTGAGCATAGAGCGAGGGCATTTCAGGGGTGGAGGGGCGAGATGGGGATTGAGA
AGTTGAGGACGCAGAGGTATGGGGTTGGAGGACATTATGGGATGCATTTGTAAGTTTTGGGGGATTGACGAAGGCCTCTGTTTATTTTCTATCA
GTACGGATCTTGAAAGAAAGATAGCATGAACACGAGGGCTAATAACTAGGAAATAGCGATTGGAGCGGAGGTAAACGTGGCATAGACCGATTTA
GTACTTTCATGGTCTATGTCGACGCATCCTCTGATATCGAAGGTGGAGGAACGGAATTCCCACGTATTGTGGGACCAAAAGGAGGAAGGTGGGA
GGACTTCCTGGAAACTACGGAAGCATTGGATCCAAGAACTGGAGAAAATGTAACAGTAGAAGGGGTGACATTCAAACCAATCAAGGGAAATGCC
GTATTCTGGGAAAATACTGACAACAACGGGAGGGGCTATGATG

>Bcin12g03020(MLST8), partial sequence [organism=Botrytis cinerea, strain D10_K_S11_06]
ATCGATTGGCTGCGAAGAAAACTGCGCAGCACCGAGTCACCAAAAGCACGAATAAACCATCGGCAAGGGAAATATTCTCTGAACCACAACATGA
CACGAGCGCGGAAGAGTATATCGGGCGAGAAGCTTCATCAAGAGCACCAAAGCGACAACGAGTCGATGATAATTATAACTCTTACGGTGGAAGA
AATGAGAATACAGCAGCTTATGCTTCCGGGAAACTTCCATCTGGAAGTATAAATGTTGGTGGAGGTAGGAAGACCACATTTCAAGAAGAACCTC
GAACGGCATTTGTCGCTGGCAAGTTGCCCCCTGGAAGTATCAACATTGGTGGAAAGAAGGCTACCCAAATCGAGGACGAGGGTAGAGCAGCTTA
TGCTTCCGGAAAATTGCCCCCAGGAAGTATAAACCCTGGCGCAAAGAAGGCGATTTCATTCCAAGATGAAACGAGACCGGCTTATGTCTCTGGA
AAGCTTCCACATGGTAGTATCGACGGTATGCGAAACCGTGAAATGGCTGCCGTCCACCGCGAAATTGCTGAGGGTGGGAGGAAACCAGGCCAGG
TTGTCTCTTCTCTATTCACATTCAATCCTACTTCAAAGAAAACTTTCGATGACCCGAAGAACAAGCGGAACCGGCAAAACCATCCAATGCACCT

TTGACAGAGGAAATGGCGACTTTCACCAATCTAGGGTTATCGAGAAGGCTTGCAGCCCATCTATCGACTAAACTCGATATGAAAGCCCCGACCG
CCATTCAAAAAGCATCTGTGCAGCAGTTGGTATCGGACGATAGCGATGCTTTCATACAAGCAGAGACTGGATCTGGAAAAACTTTGGCATATCT
ACTACCTATAGTCGAGCGAATATTAGCATTGAGTGAGAATGGCGTACAAA

>Bcin02g07770(MLST9), partial sequence [organism=Botrytis cinerea, strain D10_K_S11_06]
ACAGCTTTCCCTTTCGGTCTTGGCATCTACAGTCATTGCCATCCCTACACCATCACAACTTGAGTCTCGGGCCGTTATCGATTCCGATGCCGTT
GTAGGATTTGCCGAAACTGTTCCCAGTGGGACCGTAGGAACAGTTTACGAGGCATATAAACCATTCCTTAAAGTCGTAAATGGATGCGTACCAT
TCCCTGCCGTCGATGCATCGGGTAACACAGGGTATGTCCTTATACCATTCTCTTCCACACGATTGCTATTGAGTCTCTAACATATTTTAGTGGT
GGTTTGTCACCAACTGGCAGTAGCAATGGTGGTTGCAGCAGCAGTACCGGTCAAGTATATGTTCGAGGAGGACAAAGCGGATCAAACTACGCCA
TCATGTACTCCTGGTAAGTTCTCTCTAAACTTCTCCTTATAGATCCAACCTAACAAAATCTTAGGTACATGCCAAAGGACGAGCCCTCAACCGG
TATTGGTCACCGTCACGATTGGGAAGGTGTAATTGTCTGGCTCTCAAGCGCCACCGCCACAACTGCCGACAACATCTTAGCCGTTTGTCCTTCC
GCCCACGGAGGCTGGGATTGTTCCACCGATGGCTATTCCCTTTCTGGTACCAGCCCTCTTATCAAGTACGAAAGTATCTGGCCCGTCGATCATT
CAATGGGTCTTACTAGTACTGTTGGTGGAAAACAACCTATGATTGCTTGGGAGTCTTTACCAACTGCTGCTCAAACTGCTCTTGAGAACACCGA
TTTCGGTGCTGCGAATGTTCCATTCATTCCGGCTGTTTTCACAGATAATCT

>Bcin04g02090(MLST10), partial sequence [organism=Botrytis cinerea, strain D10_K_S11_06]
CGGAGGATGATATGGCAAAGTCTATGATTACCAAAGCATTTGTAGGCATGAGTAGTAAACTGTCGAATATGACTATGAATGATGTTTACAAGCC
CTACATCCATGTAAGAAATGTAGAATAGAGAGATCAGTAACTGGAACTAATATCGTTTGTAGGCTTTCAAGTTACTTACGCAGTTCAACCCAAT
CACTACAGCTATTGCCGAATCCCCACTATTTCAAATGGCTGTCTCAGCAAATACCATCGAAAAGTACACACTGCTAGGCCCTTTCTTCAGAATA
TCTCCTCTGCAACAGGAAGTTACCAGGGAATACTTCAGTGCGCCAAAGACGATAGATAGACGACACATTGCCACATCTCAAGATGCGTTACGAT
TAACCTTACAAACCCATCAAAAAGATTTACTTGATATCATCAACCACTTTGTTCGAGCAAGTCCAATCGCAAAAAGCAAAACCCTGGATTGGTT
CGCCTACATTGTGAATCAAAATCACAAACGTCGAGCACTTCAGGTAGACCCGAAAGAAGTGTCTTCTGATGGCTTTATGCACAATGTCACTGTC
GTTCTAGATGGTCTTTGTGAGCCATTCATGGATACCACATTCTCGAAAATTTCGAAGATTGATATTGATTATCTAAGACGTGCGCCTCGTGTAG
ATATCAAGGACGAGACCAAGTTGAACGCTGATGAGAAGGCTTCTGAGAAGTATTATGAGGACACTGTTCCTGGCACTTCTAATTTCATCTCTGA
GGTCTTCTTTCTGACATTGGCTGCTCATCATTATGGTAGTGAAGCTCTTAATGCCACGCATAAGAGTCTGGAGAAAGACATCAAATATATTCAA
AAGCAATTGACTGCCGTTGAAGCA

>Bcin01g07220(MLST1), partial sequence [organism=Botrytis cinerea, strain D10_KS12_11]
ATGGCATATCTTGTTTCTTTTCGCATTATGCTTTGGAACGTGCTGGACAGCTTGGGTTAGTGCTCTGCGTGTAGAAGCACTAAAGAAGATTCTC
GCACAACCGAAGTCATGGTTTGAGGAATCCAGGAATTCACCTGGCCGGTTGAACGAAGTTTTGGATAGGAACTCTGAGGAAATGCGTAATCTCG
TTGGCCGCTTTGCTGGTATTGTATTCACAGCATTTTTTATGCTATTGATATCAATCATTTGGGCTTTCGTGAATACATGGAAACTGACATTAGT
CTCAATGGCGACTGGGCCAGTTATATGCGCTGTCACCAAAACGTTCAATCGCGTGAGTGGAAAATGGGAAAACAAGTGCAACTACGCATCTGAA
ATGACCACTGGCATATTTTCAGAGACTTTCTCCAACATCAAAGTGGTTCGGGCTTTTACTCTGGAAACTTACTTTGAGACAAAACACACCAAAG
CTACAGAAGAACTCTATAAAATTGGACTAATACGAGCAAACTACTCGGGATTGCTGTGGGGATTGACAGATGCGATGTCATTCTTCATCACTGC
AACTATCTTTTATTATGCCACGGTTCTCATTACCAAGAGAGAGAGATCAGTATCGCGACTGCACTGCAGACTGTCAATCTTCTATTATTTGGTATT
TCTAATAGTACGAATATGCTGGCCATGATACCACAAATCAACTCTTCTCGCGTTACAGCTACGCATATGCTTGCATTAGCCAATCTCGATTCAT
CTTCCTCCCACGAAAATAAAGGAGCCGAACGGCTTTCGACAATCTTTCCAATCAAATTCAACAGTCTCTCATTCACATATCCTACTCGTCCTGA
AAAACGAACGATATCATCCTTTTCTCTTTCCCTGATTCCTAACTCAACAACTGCACTTGTCGGACCCTCCGGCTCCGGAAAATCTACAATAGCT
GCTCTGCTCGTTGGTCTCTATCCGCCAGATACTTCAACACCTCCACCGTTGACATTCAATCGTGTCTCCATAAGTAACTGTCACATTCCGTCTC
TCCGGGCTTCTCTCTCACTCGTCCCACAAATACCGATTCTATTTCCAGCTACCATTCTCCATAACATCATTTATGGTCTCCCAGAATCTTCTCC
TTGCGCTAGTCTTCCATCTGCTATTGCATCAGCAAAAGATGCTGGGATCCATGAATTTATCACATCGCTTCCAAAAGGTTATGATACTAT

>Bcin05g07690(MLST2), partial sequence [organism=Botrytis cinerea, strain D10_KS12_11]
CACCACTATCACCAAGTCCTTTTCTGCACTTTCCTCATCAAAAACGAACGAGAACATCATCAATAAATACTTTGCTGAAGTAATTGGGTTTTAT
TTTGGCCAGGATGCCTTCTCTCTACGACAGCAAGCCTTCGATGATATGTTGTGGGTAGTTCTTGGCTGGCTGGATACTGTCAAATTCATTGATT
TACATTCTGAATTGCACTATTCAAACGACTCTCAGCCAGAATGGTACGGACAACAATATAAACCTGCATTTGCACATCGAGCGCGACTATTTTG
GGAATTGGCTTCACAAGGATGGGATACTACTCTCTGTGGTGGTGGGATGATATGGTCACCATACCTTACTCCATACAAGAACGCAATTACCAAT
GAACTCTATATCGCAGCTTCGATATCGATGTACCTATATTTCCCCGGAGATGACAATCAATCCCCATTTATGATTTCCAACCCTTCATATCCAC
CTCACGATCCGAAATATCTACAGGCAGCTGTTGATGCTTACAAATGGCTGAATGGTTCCAACATGACGGATTCACAAGGATTATATGTCGACGG
GTACCATATCTCGAATCTTTCTGGCGGTGAAAACACCCATTGCGATTCTAGAAATGAGATGGTATATACCTACAATCAAGGTGTTTTGCTTACT
GGACAACGTGGTTTGTATGACGCAACCGCCGCACGATCATACCTTGTAGATGGCCACAAACTCATCGCGAATGTTATTAATGCCACAGGCTATG
ACCTGAAACACAATGTTGTCATCTCACCGCCACCCAAAGATGGTTCCGCATTGGCAAAGTGGTTTGGCCTGGGTAGGAATGGAATACTGGAAGA
AGGATGCGATTCAAGTGCTTCGTGTTCTCAAAATGGACAAACTTTCAAAGGCATATTCTTTCATCACTTGATTGCGTTCTGTAGTGATTTGCCA
GGGGAGCCTATTGCAGGGACGAAGGAAAGCTTAGAACTCGACAGAGTGTGGCATTCTGACAAATGCTCACAGTATACAAAATGGATCAGGCGAA
ATGCCGAA

>Bcin06g01710(MLST3), partial sequence [organism=Botrytis cinerea, strain D10_KS12_11]

GTGAGTCTGACTTTTGTGTTTGAGCGTTAAGATAGACACTGATGTACCAAGGCAATCACCTAATTTCATGGCGTTCTTCCCTGCATCATCTACC
TACCCTGGAATGCTGGGAGAATTATACTCAGCAGCTTTCACAGCACCTGCTTTCAATTGGATCTGTTCCCCTGCCGTGACAGAATTGGAGACGG
TTGTAATGGATTGGCTGGCCAAGCTTCTCAATCTCCCAGACTGTTATTTGTCTTCGACTCATGGTGGTGGTGTTATCAAGGATCAGCCTCGGAA
GCTATCGTTACCGTTATGGTTGCTGCCCGCGATAAATATCTTCGTGAAACTACTGAAGGTCTGTCGGGCATTGAACTCGAGGATGCGATTGCAT
ATAAGAGGAGTAAGCTAGTTGCACTAGGAAGCGAAATGGCACACAGCTCCACGCAGAAAGCAGCGCAGATAGCTGGCGTTAGATTCCGATCGAT
TCCAGTACTCGCATCCAATGATTTCGCCATGACGGGTGATGATTTAGAGAAGGTATTGGAAGAATGCAAATCTCAAGGATTGGAACCCTTCTAT
CTAACTTCGACTTTGGGAACAACATCTACATGCGCAGTTGACGATTTCGCATCTATTGCAACAGTACTTTCAAAATATGCACCTCCAGATGTTG
CAGGCGAGATCTGGGTTCACGTCGATGCTGCTTATGCAGGTGCAGCTTTGGTTTGCCCTGAATACCATCATCTAACATCGTCCTTCCAGCATTT
CCATTCCTTCGATATGAACATGCACAAATGGCTTCTGACAAATTTCGACGCTTCTTGTCTATATGTCAAGAAACGCAAAGATCTGATAGATGCA
CTCTCCATAACACCAAGTTATCTTCGCAACGAGTTTTCAGAGAGTGGACTCGTAACCGACTATCGGGATTGGCAAATTCCCCTCGGAAGACGCT
TCCGAAGCTTAAAGATTTGGTTTGTCCTCAGAACCTAC

>Bcin09g03030(MLST4), partial sequence [organism=Botrytis cinerea, strain D10_KS12_11]
ACCCTTCAACCCCACCGCAGCTCCTATACCGAAAGCGAGTATCCTACCAATTCTCCTTCCACCTGCGACTTTAAGACCATTGGCTTTCCGCACT
TTCACAAAAAAGCATAGTTTAACATGACGTCGTCGGCATTACAAGTGTTGGCTACTTTATTGGAAAGCATTGTGGGACAGGATGGAGGGAAGAA
GGACTGGCAGAGAGAGTCTTGGAGGAGGTCGCCAAGAGTTGGAAGAATAGGAGTGGCGGTGTCATTGTCGAGGGCGAGGGAACGGAATTGAAGG
AGATTCTGAAAGCTTTGGAAGAGAATATGAGTGGTGGAAGGATAGTCATAGGAAGAGAGCTAAGCCGGCAGAATAGTTTTAGTACTGGATCATC
ACAATATGGAGAGGTCAATCATACAAGACTTGGGCTACGGCCAGGGAATATACCCAGAGAGGATAGTCAGTCAAGTTTGGGAATGTCAACGTTG
GAGGTCAATGACGAGGAGGATGAGGATGGCCTGATGGATCCAAGAAGGTGGTTAAAAGTCATTGATGCATTTGAGCAACCTCGACTGGTGTACA
ATGTTGCTAAAAAGCACTTTGATAGGTATGTTTCAATGATAAAATTTTATCTGAATCTACTAACTCAGTACAGAGATACTCCAAACCTTCATTG
TTCCCACCTGCGTCTCATAAAACACTCCTCTTCCAAAACCGCTATAATGTTATCCATCAACGTCTCCTTCGCAATGAATCTTTTCAAACGCCCG
CTTTTCAAGGTGGCAAATCTTCCCTTCAACGCAGCACGTCCGCCATTACCACCCAACAACAATCATACAAATTAACGCCGATAGCTAATCTTCT
CGGTCGCAATCGCAGCTCTCATATGCTTCTCGGTCTCCTCAGTATTTCACCCACTGGTACCCTCGCCATCAATGACCTGACGGGCAGTATCGCT
CTCGATCTTACACACGCAGCAGCCATTC

>Bcin11g01310(MLST5), partial sequence [organism=Botrytis cinerea, strain D10_KS12_11]
GCTGACATGGACCTCATGTGGAACCGGCGTAGAATGCGCAACGCTTGAAGTTCCGCTCGAATATGGCGATGCAACGTCAACGGCAAAAGCCAGT
GTTGCGCTTGCTCGTTATCCTGCCACTGTTGCCGCGAGCAAGAAGCTCGGGTCTCTCTTGATAAACCCCGGTGGACCCGGTGCCTCTGGTGTTG
GCTTTGTGCAGTCTGGAGCCGGTGCCGCCGTCTCGACACTGAGTGGTGGATTGTACGATATCATCGGATGGGATCCACGTGGAACCGGTGCTTC
GGCTCCTATTTTGGAATGTTTTGCAAATGCCAGTGCGGAGTATGATTTTAACAACGCGTTTCCATCTGCTCCGAATCTCTGGCTCGGACAATTT
GCGAATGCCAGCGCAAATTCTGCTGTTAGCTCTGCTATCACATCCTTTGACACTTCTGTCGCTGCTCTTGCAAAAGCTTGCGTGGCTCAGAAAT
CTCCCGCTCTTTACACCTCAACAGCAGCATATGTTGCTCGAGACATGGCAGCGATAGTCGATGCATTGGATGGGACCTCTGCAAAACTTAACTA
CTGGGGTTTCTCATATGGAACTATCTTCCTAGCTGAGTTTATCCAAACTTTCCCAGGCCGCGTGGGAAGAGTTCTTGCCGATGGTGTTTTCGAC
GCAAAGGCAAATGCACTCACATACGTTAGCCAACTTCCCAACGATCAACTCAGTGTTCGTGCTTCGTTGAACGATTTTGCAGCTTTCTGCACCA
CCGCCGGTAGTAAAGGTTGCTCTTTTGCCACCGCCCCTACTGGAACCTCAGGTACTGTTGCTACCAGACTGGACAGCATAATGAAGGATATGTT
CCTCAATCCTATTGTTGCTTCGGGCTTAAGCATCA

>Bcin15g03910(MLST6), partial sequence [organism=Botrytis cinerea, strain D10_KS12_11]
CGCCAAAACACAAAATCATCCAACGATGAAGATGATACTCCACTTCCCTTGATTATCTGGCATGGACTCGGCGATAATTACAAAGCGGATGGTC
TTGCGCAAGTTGGAAAACTAGCTGAAGCTATTCATCCTGGGACTTTTGTCTACAATATTCGTGTAGATGAGGATGCATCTGCAGATAGGACAGC
TACCTTCTTTGGAAATCTCACTCGTGAGTACATCCCCTATTTTTCCTTAAATATCATACTAACTCTCTTACCAAGTTCAAATCGAAAAGGTCTG
CGAAGACCTCGCCTCCCATCCTATTCTCTCTACCGCGCCCGCCGTCGACGCAATTGGATTCTCCCAAGGCGGCCAATTCTTGCGTGGTTACATA
TCCCGCTGCAATGCTCCACCCATCCGCTCTCTCCTGACCTTTGGTTCCCAACACAACGGCATTTCTGCCTTCCAAGCCTGTGGTCCTGCCGATT
TCCTCTGTCGCGGTGCTCAAACCCTTTTGCGATCCAACACCTGGTCAACCTTTGTCCAATCTCGTCTCGTACCCGCTCAATACTTCAGAGATCC
GGAAAACCTAGACTCTTACCTTGAATATTCCAATTTCCTTGCCGACATCAATAATGAGCGCGTTCTCAAGAACCAAACATATAAATCCAACATG
GAAAAATTGGAACGATTCGTAATGTATGTCTTTGAAGACGACACAACTGTCATTCCTAAGGAAAGTGGATGGTGGGCTGAAGCAACGGCACGGA
AGTTACACCACTGAAAGAAAGAGCCATTTATAAAGAAGATTGGCTAGGTCTAAAGACATTGGATGAGGCCGGAAAATTAGTTTTCGAAACCATT
CCAGGGGGACATATGACGTTAGGAGAGGAGATGCTAGAGAAGGCTTTCAAAGAGTATTTTGGTCCAGCAGGGAAGAAATTTGGG

>Bcin16g03460(MLST7), partial sequence [organism=Botrytis cinerea, strain D10_KS12_11]
ATGAACCTCTTGATTTGAACTCTTCATTAATTGCAAGTGATGAACCCCTATTCTGCCCAGAGGATAGTTACAAGACGTATATCATTAGTCGAGA
ACCACTCATGATATATATTGACGGATTTTTGAAAGCGAATGAAAGTAAACATTTGGTTGATGTTAGGTGTGTTATTTATTCTGATGAAATGAAC
AAGAGAGACTGATGAGATAGTGAACCGCTTTATGAACCGTCTACTGTTTCTCACGGGCAGGAAGTTACCATTGATACTTCAGTTCGAAATTCTG
AAGTGGCGGTTTTAGAGAGGGATGAGGTGGTCAGGTGTATTGAGCATAGAGCGAGGGCATTTCAGGGGTGGAGGGGCGAGATGGGGATTGAGAA
GTTGAGGACGCAGAGGTATGGGGTTGGAGGACATTATGGGATGCATTTGTAAGTTTTGGGGGATTGACGAAGGCTTTTGTCTATTTTCTACCAG
TACGGATCTTGAAAGAAAGATAGCATGAACACGAGGGCTAATAACTAGGAAATAGCGATTGGAGCGGAGGTAAACGTGGCATAGACCGATTTAG
TACTTTCATGGTCTATGTCGACGTATCCTCTGATATCGAAGGTGGAGGAACGGAATTCCCACGTGTCGTGGGACCAAAAGGAGGAAGGTGGGAG

GACTTCCTGGAAACTACGGAAGCATTGGATCCAAGAACTGGAGAAAATGTGACAGTAGAAGGGGTGACATTCAAACCAATCAAGGGAAATGCCG
TATTCTGGGAAATACTGACAACAACGGGAGGGGCTATGATG

>Bcin12g03020(MLST8), partial sequence [organism=Botrytis cinerea, strain D10_KS12_11]
CGATTGGCTGCGAAGAAAACTGCGCAGCACCGAGTCACCAAAAGCACGAATAAACCATCGGCAAGGGAAATATTCTCTGAACCACAACATGACA
CGAGCGCGGAAGAGTATATCGGGCGAGAAGCTTCATCAAGAGCACCAAAGCGACAACGAGTCGATGATAATTATAACTCTTACGGTGGAAGAAA
TGAAAATACAGCAGCTTATGCTTCCGGGAAACTTCCATCTGGAAGTATAAATGTTGGTGGAGGTAGGAAGACCACTTTTCAAGAAGAACCTCGA
ACGGCATTTGTCGCTGGCAAGTTGCCCCCTGGAAGTATCAACATTGGTGGAAAGAAGGCTACCCAAATCGAGGACGAGGGTAGAGCAGCTTATG
CTTCCGGAAAATTGCCCCCAGGAAGTATAAACTCTGGCGCAAAGAAGGCGATTTCATTCCAAGATGAAACGAGACCGGCTTATGTCTCCGGAAA
GCTTCCACATGGTAGTATCGACGGTATGCGAAACCGTGAAATGGCTGCCGTCCACCGCGAAATTGCTGAGGGTGGGAGGAAACCAGGCCAGGTT
GTCTCTTCTCTATTCACATTCAATCCTACTTCAAAGAAAACTTTCGATGAACCAGAAGAACAAGCGGAACCGGCAAAACCATCCAATGCGCCTT
TGACAGAGGAAATGGCGACTTTCACCAATCTAGGGCTATCGAGAAGGCTTGCAGCCCATCTATCGACTAAACTCGATATGAAAGCCCCGACGGC
CATTCAAAAAGCATCTGTGCAGCAGTTGGTATCGGACGATAGCGATGCTTTCATACAAGCAGAGACTGGATCTGGAAAAACTTTGGCATATCTA
CTACCTATAGTCGAGCGAATATTAGCATTGAGTGAGAATGGCGTACAAA

>Bcin02g07770(MLST9), partial sequence [organism=Botrytis cinerea, strain D10_KS12_11]
CAGCTTTCCCTTTCGGTCTTGGCATCTACAGTCATTGCCATCCCTACACCATCACAACTTGAGTCTCGGGCCGTTATCGATTCCGATGCCGTTG
TAGGATTTGCCGAAACTGTTCCCAGTGGGACCGTAGGAACAGTTTATGAGGCATATAAACCATTCCTTAAAGTCGTAAATGGATGCGTACCATT
CCCTGCCGTCGATGCATCGGGTAACACAGGGTATGTCCTTATACCTTTCTCTTCCACACGATTGCTATTGAGTCTCTAACATATTTTAGTGGTG
GTTTGTCACCAACTGGCAGCAGCAATGGTGAATGCAGCAGCAGTACCGGTCAAGTATATGTTCGAGGAGGACAAAGCGGATCAAACTACGCTAT
CATGTACTCCTGGTAAGTTCTCTTTAAACTTCTCCTTATAGATCCAACCTAACAAAATCTTAGGTACATGCCAAAGGACGAGCCCTCAACCGGT
ATTGGTCACCGTCACGATTGGGAAGGTGTAATTGTCTGGCTCTCCAGCGCCACCGCCACAACTGCCGACAACATCTTAGCCGTTTGTCCTTCCG
CCCACGGAGGCTGGGATTGTTCCACGGATGGCTATTCCCTTTCTGGTACCAGCCCTCTTATCAAGTACGAAAGTATCTGGCCCGTCGATCACTC
AATGGGTCTTACTAGTACTGTTGGTGGAAAACAACCTATGATTGCTTGGGAGTCTTTACCAACTGCTGCTCAAACTGCTCTTGAGAACACCGAT
TTCGGTGCTGCGAATGTTCCATTCATTCCGGCTGTTTTCACAGATAATCT

>Bcin04g02090(MLST10), partial sequence [organism=Botrytis cinerea, strain D10_KS12_11]
CGGAGGATGATATGGCAAAGTCTATGATTACCAAAGCATTTGTAGGCATGAGTAGTAAATTGTCGAATATGACTATGAATGATGTTTACAAGCC
CTACATCCATGCAAGAAATGTAGAATAAAGAGATCAGTAACTGGAACTAATATCGTTTGCAGGCTTTCAAGTTACTTACGCAGTTCAACCCAAT
CACTACAGCTATTGCCGAATCCCCACTATTTCAAATGGCTGTCTCAGCAAATACCATCGAAAAGTACACACTGCTAGGCCCTTTCTTCAGAATA
TCTCCTCTGCAACAGGAAGTTACCAGGGAATACTTCAGTGCGCCAAAGACGATAGATAGGCGACACATTGCCACATCTCAAGATGCGTTACGAT
TGACCTTACAAACCCATCAAAAAGATTTACTTGATATCATCAACCACTTTGTTCGAGCAAGTCCAATCGCAAAAAGCAAAACCCTGGATTGGTT
CGCCTACATTGTGAATCAAAATCACAAGCGTCGAGCACTTCGGGTAGACCCGAAAGAAGTGTCTTCTGATGGCTTTATGCACAATGTCACTGTC
GTTCTAGATGGTCTTTGTGAGCCATTCATGGATACCACATTCTCAAAAATTTCGAAGATTGATATTGATTATCTAAGACGTGCGCCTCGTGTAG
ATATCAAGGACGAGACCAAGTTGAACGCTGATGAGAAGGCTTCTGAGAAGTATTATGAGGACACTGTTCCTGGCACTTCTAATTTCATCTCCGA
GGTATTCTTTCTGACATTGGCTGCTCATCATTATGGTAGTGAAGCTCTTAATGCCACGCATAAGAGTCTGGAGAAAGACATCAAATATATTCAA
AAGCAATTGACTGCCGTTGAAGCA

>Bcin01g07220(MLST1), partial sequence [organism=Botrytis cinerea, strain D11_H_R3_7]
ATGGCATATCTTGTTTCTTTTCGCATTATGCTTTGGAACGTGCTGGGCAAGCTTGGGTTAGTGCTCTGCGTGTAGAAGCACTAAAGAGATTCTC
GCACAACCGAAGTCATGGTTTGAGGAATCCAGGAATTCACCTAGCCGGTTGAACGAAGTTTTGGATAGGAACTCTGAGGAAATGCGTAATCTCG
TTGGCCGCTTTGCTGGTATTGTATTCACAGCATTTTTTATGCTATTGATATCAATCATTTGGGCTTTCGTGAATACATGGAAACTGACATTAGT
CTCAATGGCAACTGGGCCAGTTATATACGCTGTCACCAAAACGTTCAATCGCGTGAGTGGAAAATGGGAAAACAAGTGCAACTACGCATCTGAA
ATGACCACTGGCATATTTTCAGAGACTTTCTCCAACATCAAAGTGGTTCGGGCTTTTACTCTGGAAACTTACTTTGAGACAAAACACACCAAAG
CTACAGAAGAACTCTATAAAGTTGGACTAATACGAGCAAACTACTCGGGATTGCTGTGGGGATTGACAGATGCGATGTCATTCTTCATCACTGC
AACTATCTTTTATTATGCCACGGTTCTCATTACCAAGAGAGAGATCAGTATTGCGACTGCACTACAGACTGTCAATCTTCTATTATTTGGTATT
TCTAATAGTACGAATATGCTGGCTATGATACCACAAATCAACTCTTCTCGCGTTACAGCTACGCATATGCTTGCATTAGCCAATCTCGATTCAT
CTTCCTCCCACGAAAATAAAGGAACCGAACGGCTTTCGACAATCTTTCCAATCAAATTCAACCGTCTTTCATTCACATACCCTACTCGTCCTGA
AAAACGAACGATATCATCCTTTTTCTCTTTCCCTGATTCCTAACTCAACAACTGCACTTGTCGGACCCTCCGGCTCCGGAAAATCTACGATAGCT
GCTCTGCTCATTGGTCTCTATCCGCCAGATACTTCAACACCTCCACCGTTGACATTCAATCGCGTCTCCATAAGTAACTGTCACATTCCGTCTC
TCCGGGCTTCTCTCTCACTCGTCCCACAAATACCGGATTCTATTTCCAGCTACCATTCTCCATAACATCATTTATGGCCTCCCAGAATCTTCTC
CTTGTGCTAGTCTTCCATCTGCTATTGCATCAGCAAAAGATGCTGGGATCCATGAATTTATCACATCGCTTCCACAAGGTTATGATACTAT

>Bcin05g07690(MLST2), partial sequence [organism=Botrytis cinerea, strain D11_H_R3_7]
ACCACTATCACCAAGTCCTTTTCTGCACTTTCCTCATCAAAAACGAACGAGAACATCATCAATAAATACTTTGCTGAAGTAATTGGGTTTTATT
TTGGCCAGGATGCCTTCTCTCTACGACAGCAAGCCTTCGATGATATGTTGTGGGTAGTTCTTGGCTGGCTGGATACTGTCAAATTCATTGATTT
ACATTCTGAATTGCACTATTCAAACGACCCTCAGCCAGAATGGTACGGACAACAATATAAGCCTGCATTTGCACATCGAGCGCGACTATTTTGG

GAATTGGCTTCACAAGGATGGGATACTACTCTCTGTGGTGGTGGGATGATATGGTCACCATACCTTACTCCATACAAGAATGCAATTACCAATG
AACTCTATATCGCAGCTTCGATATCGATGTACCTCTATTTCCCCGGAGATGACAATCAATCCCCATTTATGCTTTCCAACCCTTCATATCCACC
TCACGATCCGAAATATCTACAGGCAGCTGTTGATGCTTACAAATGGCTGAATGGTTCCAACATGACGGATTTACAAGGATTATATGTCGACGGG
TATCATATCTCGAATCTTTCTGGCGGTGAAAACACCCATTGCGATTCTAGAAATGAGATGGTATATACCTACAATCAAGGTGTTTTGCTTACTG
GACAACGTGGTTTGTATGACGCAACCGCCGCACGATCATACCTTGTGGATGGCCACAAACTCATCGCGAATGTTATTAATGCCACAGGCTATGA
CCTGAAACACAATGTTGTCATCTCACCGCCACCCAAAGATGGTTCCGCATTGGCAAAGTGGTTTGGCCTGGGTAGGAATGGAATACTGGAAGAA
GGATGCGATTCAAGTGCTTCGTGTTCTCAAAATGGACAAACTTTCAAAGGCATATTCTTTCATCACTTGATTGCATTCTGTAGTGATTTGCCAG
GGGAGCCTATTGCAGGGACGAAAGAAGGCCTAGAACTCGACAGAGTGTGGCATTCTGACAAATGCTCACAGTATACAAAAT

>Bcin06g01710(MLST3), partial sequence [organism=Botrytis cinerea, strain D11_H_R3_7]
GTGAGTCTGACTTTTGTATTTGAGCGTTAAGATGGACACTGATGTACCAAGGCAATCACCTAATTTCATGGCGTTCTTCCCTGCATCATCTACC
TACCCTGGAATGCTGGGAGAATTATACTCAGCAGCTTTCACAGCTCCTGCTTTCAATTGGATCTGTTCCCCTGCTGTGACAGAATTGGAGACGG
TTGTAATGGATTGGTTGGCCAAGCTTCTCAATCTCCCAGACTGTTATTTGTCGTCGACTCATGGTGGTGGTGTTATCCAAGGATCAGCCTCGGA
AGCTATCGTTACCGTTATGGTTGCTGCCCGCGATAAGTATCTTCGTGAAACCACTGAAGGTCTGTCGGGAATTGAACTCGAGGATGCGATTGCA
TATAAGAGGAGTAAGCTAGTTGCACTAGGAAGCGAAATGGCACACAGCTCCACGCAGAAAGCAGCGCAGATAGCTGGCGTTAGATTCCGATCGA
TTCCAGTACTCGCATCCAATGATTTCGCCATGACGGGTGATGATTTAGAGAAGGTATTGAAAGAATGCAAATCTCAAGGATTGGAACCCTTCTA
TCTAACTTCGACGTTGGGAACAACATCTACATGCGCAGTTGACGACTTCGCATCTATTGCAACAGTACTTTCAAAATATGCACCTCCAGATGTT
GCAGGCGAGATCTGGGTTCACGTCGATGCTGCTTATGCAGGTGCAGCTTTGGTTTGCCCTGAATACCATCATCTAACATCGTCCTTCCAGCATT
TCCATTCCTTTGATATGAACATGCACAAATGGCTTCTGACAAATTTCGACGCTTCTTGCCTATATGTCAAGAAACGCAAAGATCTGATCGATGC
ACTCTCCATAACACCAAGTTATCTTCGCAACGAGTTTTCAGAGAGTGGACTCGTAACCGACTATCGGGACTGGCAAATTCCTCTCGGAAGACGT
TTCCGAAGCTTAAAGATTTGGTTTGTCCTCAGAACCTAC

>Bcin09g03030(MLST4), partial sequence [organism=Botrytis cinerea, strain D11_H_R3_7]
ACCCTTCAACCCCACCGCAGCTCCTATACCGAAAGCGAGTATCCTACCAATTCTCCTTCCACCTGCGACTTTAAGACCATTGGCTTTTCGCACT
TTCACAAAAAAGCATAGTTTAACATTGACGTCGTCGGCATTACAAGTGTTGGCTACTTTTATTGGAAAGCATTGTGGGACAGGATGGAGGGAGG
AAGGACTGGCAGAGAGAGTCTTGGAGGAGGTCGCCAAGTGTTGGAAGAATAGGAGTGGCGGTGTCATTGTCGAGGGCGAGGGAACGGAATTGAA
GGAGATTCTGAAAGCTTTGGAAGGGAATATGAGTGGTGGAAGGATAGTCATAGGAAGAGAGCTAAGCCGGCAGAATAGTTTAGTACTGGGATCA
TCACAATATGGAGAGGTCAATCATACAAGACTTGGGCTACGGCCAGGGAATATACCCAGAGAGGATAGTCAGTCAAGTTTGGGAATGTCAACGT
TGGAGGTCAATGACGAGGAAGATGAGGATGGCCTGATGGATCCAAGAAGGTGGTTAAAAGTCGTTGATGCATTTGAGCAACCTCGACTGGTGTA
CAATGTTGCTAAAAAGCACTTTGATAGGTATGTTTCAATGATAAAATTTTATCTGAATCGACTAACTCAGTACAGAGATACCTCCAAACCTTCA
TTGTTCCCACCTGCGTCTCATAAAACACTCCTCTTCCAAAACCGCTATAATGTTATCCATCAACGTCTCCTTCGCAATGAATCTTTTCAAACGC
CCGCTTTTCAAGGTGGCAAATCTTCCCTTCAGCGCAGCACGTCCGCCATTACCACCCAACAACAATCATACAAATTAACGCCGATAGCTAATCT
TCTCGGTCGCAATCGCAGCTCTCATATGCTTCTCGGTCTCCTCAGTATTTCACCCACTGGTACCCTCGCCATCAATGACCTGACGGGCAGTATC
GCTCTTGATCTTACACACGCAGCAGCCATTC

>Bcin11g01310(MLST5), partial sequence [organism=Botrytis cinerea, strain D11_H_R3_7]
ACTGACATGGACCTCATGTGGAACCGGCGTAGAATGCGCAACGCTTGAAGTTCCGCTCGAATATGGCGATGCAACGTCAACGGCAAAAGCCAGT
GTTGCGCTTGCTCGTTATCCTGCCACTGTTGCCGCGAGCAAGAAGCTCGGGTCTCTCTTGATAAATCCCGGTGGACCCGGTGCCTCTGGTGTTG
GCTTTGTGCAGTCTGGAGCCGGTGCCGCCGTCTCGACACTGAGTGGTGGTTATACGATATCATCGGATGGGATCCACGTGGAACCGGTGCTTCG
GCTCCTATTTTGGAATGTTTTGCAAATGCCAGTGCGGAGTATGATTTTAACAACGCGTTTCCATCTGCTCCGAATCTCTGGCTCGGACAATTTG
CGAATGCCAGCGCAAATTCTGCTGTTAGCTCTGCTATCACATCCTTTGACACTTCTGTCGCTGCTCTTGCAAAAGCTTGCGTGGCTCAGAAATC
TCCCGCTCTTTACACCTCAACAGCAGCATATGTTGCTCGAGACATGGCAGCGATAGTCGATGCATTGGATGGGACCTCTGCAAAACTTAACTAC
TGGGGTTTCTCATATGGAACTATCTTCCTAGCTGAGTTTATCCAAACTTTCCCAGGCCGCGTGGGAAGAGTTCTTGCCGATGGTGTTTTCGACG
CAAAGGCAAATGCACTCACATACGTTAGCCAACTTCCCAACGACCAACTCAGTGTTCGTGCTTCGTTGAACGATTTTGCAGCTTTCTGCACCAC
CGCCGGTAGTAAAGGTTGCTCTTTTGCCACCGCCCCCACTGGAACCTCAGGTACTGTTGCTACCAGACTGGACAACATAATGAAGGATATGTTC
CTCAATCCTATTGTTGCTTCGGGCTTGAGCATCA

>Bcin15g03910(MLST6), partial sequence [organism=Botrytis cinerea, strain D11_H_R3_7]
GCCAAAACACAAAATCATCCAACGATGAAGATGATACTCCACTTCCCTTGATTATCTGGCATGGACTCGGCGATAATTACAAAGCGGATGGTCT
TGCGCAAGTTGGAAAACTAGCTGAAGCTATTCATCCTGGGACTTTTGTCTATAATATTCATGTAGATGAGGATGCATCTGCAGATAGGACAGCT
ACCTTCTTTGGAAATCTCACTCGTGAGTACATCCCCTATTTTTCCTTTAAATACCATACTAACTCTCTTACCAAGTTCAAATCGAAAAGGTCTG
CGAAGACCTCGCCTCCCATCCTATTCTCTCTACCGCGCCCGCCGTCGACGCAATTGGATTCTCCCAAGGCGGCCAATTCTTGCGCGGTTACATA
TCCCGCTGCAATGCTCCACCCATCCGCTCTCTCCTGACCTTCGGTTCCCAACACAACGGCATTTCTGCCTTCCAAGCCTGTGGTCCTGCCGATT
TCCTCTGTCGCGGTGCTCAAACCCTTTTGCGATCCAACACCTGGTCAACCTTTGTCCAATCTCGTCTCGTACCTGCTCAATACTTCAGAGATCC
GGAAAACCTAGACTCTTACCTTGAATATTCCAATTTCCTTGCCGACATCAATAATGAGCGCGTTCTCAAGAACCAAACATATAAATCCAACATG
GAAAAATTGGAACGATTCGTAATGTATGTCTTTGAAGACGATACAACTGTCATCCCTAAGGAAAGTGGATGGTGGGCTGAAGTCAATGGCACGG
AAGTTACACCACTGAAAGAAAGAGCCATTTATAAAGAAGATTGGCTAGGGTTAAAGACATTGGATGAGGCCGGAAAATTAGTTTCGAAACCAT

TCCAGGGGGACATATGACGTTAGGAGAGGAGATGCTAGAGAAGGCTTTCAAAGAGTACTTTGGTCCAGCAGGGAAAAAATTTGGG

>Bcin16g03460(MLST7), partial sequence [organism=Botrytis cinerea, strain D11_H_R3_7]
ATGAACCTCTTAATTTGAACTCTTCATTAATTGCAAGTGATGAACCCCTATTCTGCCCAGAGGATAGTTACAAGACGTATATCATTAGCCGAGA
ACCACTCATGATATACATTGACGGATTTTTGAAAGCGAATGAAAGTAAACACTTGGTTGATGTTAGGTGTGTTATTTATTCTGATGAAATGAAC
AAGAGAGACTGATGAGATAGTGAACCGCTTTATGAACCGTCTACTGTTTCTCACGGACAGGAAGTTACCATTGATCCTTCGGTTCGAAATTCTG
AAGTGGCGGTTTTAGAGAGGGATGAGGTGGTCAGGTGTATTGAGCATAGAGCGAGGGCATTTCAGGGGTGGAGGGGCGAGATGGGGATTGAGAA
GTTGAGGACGCAGAGGTATGGGGTTGGAGGACATTATGGGATGCATTTGTAAGTTTTGGGGAATTGACGAAGGCCTCTGTTTATTTTCTATCAG
TACGGATCTTGAAAGAAAGATAGCATGAACACGAGGGCTAATAACTAGGAAATAGCGATTGGAGCGGAGGTAAACGTGGCATAGACCGATTTAG
TACTTTCATGGTCTATGTCGACGTATCCTCTGATATCGAAGGTGGAGGAACGGAATTCCCACGTATTGTGGGACCAAAAGGAGGAAGGTGGGAG
GACTTCCTGGAAACTACGGAAGCATTGGATCCAAGAACTGGAGAAAATGTAACAGTAGAGGGGGTGACATTCAAACCAATCAAGGGAAATGCCG
TATTCTGGGAAAATACTGACAACAACGGGAGGGGCTATGAT

>Bcin12g03020(MLST8), partial sequence [organism=Botrytis cinerea, strain D11_H_R3_7]
ATCGATTGGCTGCGAAGAAAACTGCGCAGCACCGAGTCACCAAAAGCACGAATAAACCATCGGCAAGGGAAATATTCTCTGAACCACAACATGA
CACGAGCGCGGAAGAGTATATCGGGCGAGAAGCTTCATCAAGAGCACCAAAGCGACAACGAGTCGATGATAATTATAACTCTTACGGTGGAAGA
AATGAGAATACAGCAGCTTATGCTTCCGGGAAACTTCCATCTGGAAGTATAAATGTTGGTGGAGGTAGGAAGACCACATTTCAAGAAGAACCTC
GAACGGCATTTGTCGCTGGCAAGTTGCCCCCTGGAAGTATCAACATTGGTGGAAAGAAGGCTACCCAAATCGAGGACGAGGGTAGAGCAGCTTA
TGCTTCCGGAAAATTGCCCCCAGGAAGTATAAACTCTGGCGCAAAGAAGGCGATTTCATTCCAAGATGAAACGAGACCGGCTTATGTCTCTGGA
AAGCTTCCACATGGTAGTATCGACGGTATGCGAAACCGTGAAATGGCTGCCGTCCACCGCGAAATTGCTGAGGGTGGGAGGAAACCAGGCCAGG
TTGTCTCTTCTCTATTCACATTCAATCCTACTTCAAAGAAAACTTTCGATGAACCCGAAGAACAAGCGGAACCGGCAAAACCATCCAATGCACC
TTTGACAGAGGAAATGGCGACTTTCACCAATCTAGGGTTATCGAGAAGGCTTGCAGCCCATCTATCGACTAAACTCGATATGAAAGCCCCGACC
GCCATTCAAAAAGCATCTGTGCAGCAGTTGGTATCGGACGATAGCGATGCTTTCATACAAGCAGAGACTGGATCTGGAAAAACTTTGGCATATC
TACTACCTATAGTCGAGCGAATATTAGCATTGAGTGAGAATGGCGTACAAAT

>Bcin02g07770(MLST9), partial sequence [organism=Botrytis cinerea, strain D11_H_R3_7]
CAGCTTTCCCTTTCGGTCTTAGCATCTACAGTCATTGCCATCCCTACACCATCACAACTTGAGTCTCGGGCCGTTATCGATTCCGATGCCGTTG
TAGGGTTTGCCGAAACTGTTCCCAGTGGGACCGTAGGAACAGTTTATGAGGCATATAAACCATTCCTTAAAGTCGTAAATGGATGCGTACCATT
CCCTGCCGTCGATGCATCGGGTAACACAGGGTATGTCCTTATACCATTCTCTTCCACACGATTGCTATTGAGTCTCTAACATATTTAGTGGTG
GTTTGTCACCAACTGGCAGTAGCAATGGTGGTTGCAGCAGCAGTACCGGTCAAGTATATGTTCGAGGAGGACAAAGCGGATCAAACTACGCCAT
CATGTACTCCTGGTAAGTTCTCTCTAAACTTCTCCTTATAGATCCAACCTAACAAAATCTTAGGTACATGCCAAAGGACGAGCCCTCAACCGGT
ATTGGTCACCGTCACGATTGGGAAGGTGTAATTGTCTGGCTCTCAAGCGCCACCGCCACAACTGCCGACAACATCTTAGCCGTTTGTCCTTCCG
CCCACGGAGGCTGGGATTGTTCCACCGATGGATATTCCCTTTCTGGTACCAGCCCTCTTATCAAGTACGAAAGTATCTGGCCCGTCGATCATTC
AACGGGTCTTACCAGTACTGTTGGTGGAAAACAGCCTATGATTGCTTGGGAGTCTTTACCAACTGCTGCTCAAACTGCTCTTGAGAACACCGAT
TTCGGTGCTGCGAATGTTCCATTCATTCCGGCTGTTTTCACAGATAATCT

>Bcin04g02090(MLST10), partial sequence [organism=Botrytis cinerea, strain D11_H_R3_7]
CGGAGGATGATATGGCAAAGTCTATGATTACCAAAGCATTTGTAGGCATGAGTAGTAAACTGTCGAATATGACTATGAATGATGTTTACAAGCC
CTACATCCATGTAAGAAATGTAGAATAGAGAGATCAGTAACTGGAACTAATATCGTTTGTAGGCTTTCAAGTTACTTACGCAGTTCAACCCAAT
CACTACAGCTATTGCCGAATCCCCACTATTTCAAATGGCTGTCTCAGCAAATACCATCGAAAAGTACACACTGCTAGGCCCTTTCTTCAGAATA
TCTCCTCTGCAACAGGAAGTTACCAGGGAATACTTCAGTGCGCCAAAGACGATAGATAGACGACACATTGCCACATCTCAAGATGCGTTACGAT
TAACCTTACAAACCCATCAAAAAGATTTACTTGATATCATCAACCACTTTGTTCGAGCAAGTCCAATCGCAAAAAGCAAAACCCTGGATTGGTT
CGCCTACATTGTGAATCAAAATCACAAACGTCGAGCACTTCAGGTAGACCCGAAAGAAGTGTCTTCTGATGGCTTTATGCACAATGTCACTGTC
GTTCTAGATGGTCTTTGTGAGCCATTCATGGATACCACATTCTCGAAAATTTCGAAGATTGATATTGATTATCTAAGACGTGCGCCTCGTGTAG
ATATCAAGGACGAGACCAAGTTGAACGCTGATGAGAAGGCTTCTGAGAAGTATTATGAGGACACTGTTCCTGGCACTTCTAATTTCATCTCTGA
GGTCTTCTTTCTGACATTGGCTGCTCATCATTATGGTAGTGAAGCTCTTAATGCCACGCATAAGAGTCTGGAGAAAGACATCAAATATATTCAA
AAGCAATTGACTGCCGTTGAAGCA

>Bcin01g07220(MLST1), partial sequence [organism=Botrytis cinerea, strain D11_KL_tax4]
ATGGCATATCTTGTTTCTTTTCGCATTATGCTTTGGAACGTGCTGGACAAGCTTGGGTTAGTGCTCTGCGTGTAGAAGCACTAAAGAAGATTCT
CGCACAACCGAAGTCATGGTTTGAGGAATCCAGGAATTCACCTGGCCGGTTGAACGAAGTTTTGGATAGGAACTCTGAGGAATGCGTAATCTCG
TTGGCCGCTTTGCTGGTATTGTATTCACAGCATTTTTTATGCTATGATATCAATCATTTGGGCTTTCGTGAATACATGGAAACTGACATTAGTC
TCGATGGCAACTGGGCCAGTTATATACGCTGTCACCAAAACGTTCAATCGCGTGAGTGGAAAATGGGAAAACAAGTGCAACTACGCCTCTGAAA
TGACCACTGGCATATTTTCAGAGACTTTCTCCAACATCAAAGTGGTTCGGGCTTTTACTCTGGAAACTTACTTTGAGACAAAACACACCAAAGC
TACAGAAGAACTTTGTAAAGTTGGACTAATACGAGCAAACTACTCGGGATTGCTGTGGGGATTGACAGATGCGATGTCATTCTTCATCACTGCA
ACTATCTTTTATTATGCCACGCTTCTCATTACCAAGAGAGAGATCAGTATCGCGACTGCACTACAGACTGTCAATCTTCTATTATTTGGTATTT
CTAATAGTACGAATATGCTGGCCATGATACCACAAATCAACTCTTCTCGCGTTACAGCTACGCATATGCTTGCATTAGCCAATCTCGATTCATC

TTCCTCCCACGAAAATAAAGGAACCGAACGGCTTTCGACAATCTTTCCAATCAAATTCAACCGTCTCTCATTCACATATCCTACTCGTCCTGAA
AAACGAACGATATCATCCTTTTCTCTTTCCCTGATTCCTAACTCAACAACTGCACTTGTCGGACCCTCCGGCTCCGGAAAATCTACAATAGCTG
CTCTGCTCATTGGTCTTTATCCGCCAGATATTTCAACACCTCCACCGTTGACATTCAATCGCGTCTCCATAAGTAACTGTCACATTCCGTCTCT
CCGGGCTTCTCTCTCACTCGTCCCACAAATACCGATTCTATTTCCAGCTACCATTCTCCATAACATCATTTATGGTCTCCCAGAATCTTCTCCT
TGTGCTAGTCTTCCATCTGCTATTGCATCAGCAGAAGATGCTGGGATCCATGAATTTATCACATCGCTTCCACAAGGTTATGATACTATG

>Bcin05g07690(MLST2), partial sequence [organism=Botrytis cinerea, strain D11_KL_tax4]
ACCACTATCACCAAGTCCTTTTCTGCACTTTCCTCATCAAAAACGAACGAGAACATCATCAATAAATACTTTGCTGAAGTAATTGGGTTTTATT
TTGGCCAGGATGCCTTCTCTCTACGACAGCAAGCCTTCGATGATATGTTGTGGGTAGTTCTTGGCTGGCTGGATACTGTCAAATTCATTGATTT
ACATTCTGAATTGCACTATTCAAACGACTCTCAGCCAGAATGGTACGGACAACAATATAAACCTGCATTTGCACATCGAGCGCGACTATTTTGG
GAATTGGCTTCACAAGGATGGGATACTACTCTCTGTGGTGGTGGGATGATATGGTCACCATACCTTACTCCATACAAGAACGCAATTACCAATG
AACTCTATATCGCAGCTTCGATATCGATGTACCTATATTTCCCCGGAGATGACAATCAATCCCCATTTATGCTTTCCAACCCTTCATATCCACC
TCACGATCCGAAATATCTACAGGCAGCTGTTGATGCTTACAAATGGCTGAATGGTTCCAACATGACGGATTTACAAGGATTATATGTCGACGGG
TACCATATCTCGAATCTTTCTGGCGGTGAAAACACCCATTGCGATTCTAGAAATGAGATGGTATATACCTACAATCAAGGTGTTTTGCTTACTG
GACAACGTGGTTTGTATGACGCAACCGCCGCACGATCATACCTTGTAGATGGCCACAAACTCATCGCGAATGTTATTAATGCCACAGGCTATGA
CCTGAAACACAATGTTGTCATCTCACCGCCACCCAAAGATGGTTCCGCATTGGCAAAGTGGTTTGGCCTGGGTAGGAATGGAATACTGGAAGAA
GGATGCGATTCAAGTGCTTCGTGTTCTCAAAATGGACAAACTTTCAAAGGCATATTCTTTCATCACTTGATTGCGTTCTGTAGTGATTTGCCAG
GGAGCCTATTGCAGGGACGAAGGAAAGCTTAGAACTCGACAGAGTGTGGCATTCTGACAAATGCTCACAGTATACAAAAT

>Bcin06g01710(MLST3), partial sequence [organism=Botrytis cinerea, strain D11_KL_tax4]
GTGAGTCTGACTTTTGTATTTGAGCGTTAAGATAGACACTGATATACCAAGGCAATCACCTAATTTCATGGCGTTCTTCCTGCATCATCTACCT
ACCCTGGAATGCTGGGAGAATTATACTCAGCAGCTTTCACAGCACCTGCTTTCAATTGGATCTGTTCCCCTGCTGTGACAGAATTGGAGACGGT
TGTAATGGATTGGCTGGCCAAGCTTCTCAATCTCCCAGACTGTTATTTGTCTTCGACTCATGGTGGTGGTGTCATCCAAGGATCAGCCTCGGAA
GCTATCGTTACCGTTATGGTTGCTGCCCGCGATAAATATCTTCGTGAAACTACTGAAGGTCTGTCGGGCATTGAACTCGAGGATGCGATTGCAT
ATAAGAGGAGTAAGCTAGTTGCACTAGGAAGCGAAATGGCACACAGCTCCACGCAGAAAGCAGCGCAAATAGCTGGCGTTAGATTCCGATCGAT
TCCAGTACTCGCATCCAATGATTTCGCCATGACGGGTGATGATTTAGAGAAGGTATTGAAAGAATGCAAATCTCAAGGATTGGAACCCTTCTAT
CCTAACTTCGACTTTGGGAACAACATCTACATGCGCAGTTGACGACTTCGCATCTATTGCAACAGTACTTTCAAAATATGCACCTCCAGATGTT
GCAGGCGAGATCTGGGTTCACGTCGATGCTGCTTATGCAGGTGCAGCTTTGGTTTGCCCTGAATACCATCATCTAACATCGTCCTTCCAGCATT
TCCATTCCTTCGATATGAACATGCACAAATGGCTTCTGACAAATTTCGACGCTTCTTGTCTATATGTCAAGAAACGCAAAGATCTGATCGATGC
ACTCTCCATAACACCAAGTTATCTTCGCAACGAGTTTTCAGAGAGTGGACTCGTAACCGACTATCGGGACTGGCAAATTCCTCTCGGAAGACGC
TTCCGAAGCTTAAAGATTTGGTTTGTCCTCAGAACCTAC

>Bcin09g03030(MLST4), partial sequence [organism=Botrytis cinerea, strain D11_KL_tax4]
ACCCTTCAACCCCACCGCAGCTCCTATACCGAAAGCGAGTATCCTACCAATTCTCCTTCCACCTGCGACTTTAAGACCATTGGCTTTCCGCACT
TTCACAAAAAAGCATAGTTTAACATTGACGTCGTCGGCATTACAAGTGTTGGCTACTTTTATTGGAAAGCATTGTGGGACAGGATGGAGGGAAG
AAGGACTGGCAGAGAGAGTCTTGGAGGAGGTCGCCAAGAGTTGGAAGAATAGGAGTGGCGGTGTCATTGTCGAGGGCGAGGGAACGGAATTGAA
GGAGATTCTGAAAGCTTTGGAAGGGAATATGAGTGGTGGAAGGATAGTCATAGGAAGAGAGCTAAGCCGGCAGAATAGTTTAGTACTGGGATCA
TCACAATATGGAGAGGTCAATCATACAAGACTTGGGCTACGGCCAGGGAATATACCCAGAGAGGATAGTCAGTCAAGTTTGGGAATGTCAACGT
TGGAGGTCAATGACGAGGAAGATGAGGATGGCCTGATGGATCCAAGAAGGTGGTTAAAAGTCATTGATGCATTTGAGCAACCTCGACTGGTGTA
CAATGTTGCTAAAAAGCACTTTGATAGGTATGTTTCAATGATAAAATTTCATCTGAATCGACTAACTCAGTACAGAGATACCTCCAAACCTTCA
TTGTTCCCACCTGCGTCTCATAAAACACTCCTCTTCCAAAACCGCTATAATGTTATCCGTCAACGTCTCCTTCGCAATGAATCTTTTCAAACGC
CCGCTTTTCAAGGTGGCAAATCTTCCCTTCAACGCAGCACGTCCGCCATTACCACCCAACAACAATCATACAAATTAACGCCGATAGCTAATCT
TCTCGGTCGCAATCGCAGCTCTCATATGCTTCTCGGTCTCCTCAGTATTTCACCCACTGGTACCCTCGCCATCAATGACCTGACGGGCAGTATC
GCTCTCGATCTTACACACGCAGCAGCCATTCCC

>Bcin11g01310(MLST5), partial sequence [organism=Botrytis cinerea, strain D11_KL_tax4]
ACTGACATGGACCTCATGTGGAACCGGCGTAGAATGCGCAACGCTTGAAGTTCCGCTCGAATATGGCGATGCAACGTCAACGGCAAAAGCCAGT
GTTGCGCTTGCTCGTTATCCTGCCACTGTTGCCGCGAGCAAGAAGCTCGGGTCTCTCTTGATAAACCCCGGTGGACCCGGTGCCTCTGGTGTTG
GCTTTGTGCAGTCTGGAGCCGGTGCCGCCGTCTCGACACTGAGTGGTGGATTGTACGATATCATCGGATGGGATCCACGTGGAACCGGTGCTTC
GGCTCCTATTTTGGAATGTTTTGCAAATGCCAGTGCGGAGTATGATTTTAACAACGCGTTTCCATCTGCTCCGAATCTCTGGCTCGGACAATTT
GCGAATGCCAGCGCAAATTCTGCTGTTAGCTCTGCTATCACATCCTTTGACACTTCTGTCGCTGCTCTTGCAAAAGCTTGCGTGGCTCAGAAAT
CTCCCGCTCTTTACACCTCAACAGCAGCATATGTTGCTCGAGACATGGCAGCGATAGTCGATGCATTGGATGGGACCTCTGCAAAACTTAACTA
CTGGGGTTTCTCATATGGAACTATTTTCCTAGCTGAGTTTATCCAAACTTTCCCAGGCCGTGTGGGAAGAGTTCTTGCCGATGGTGTTTTCGAC
GCAAAGGCAAATGCACTCACATACGTTAGCCAACTTCCCAACGATCAACTCAGTGTTCGTGCTTCGTTGAACGATTTTGCAGCTTTCTGCACCA
CCGCCGGTAGTAAAGGTTGCTCTTTTGCCACCGCCCCTACTGGAACCTCAGGTACTGTTGCTACCAGACTGGACAACATAATGAAGGATATGTT
CCTCAATCCTATTGTTGCTTCGGGCTTAAGCATCA

>Bcin15g03910(MLST6), partial sequence [organism=Botrytis cinerea, strain D11_KL_tax4]
GCCAAAACACAAAATCATCCAACGATGAAGATGATACTCCACTTCCCTTGATTATCTGGCATGGACTCGGCGATAATTACAAAGCGGATGGTCT
TGCGCAAGTTGGAAAACTAGCTGAAGCTATTCATCCTGGGACTTTTGTCTACAATATTCATGTAGATGAGGATGCATCTGCAGATAGGACAGCT
ACCTTCTTTGGAAATCTCACTCGTGAGTACATCCCCTATTTTTCCTTTAAATATCATACTAACTCTCTTACCAAGTTCAAATCGAAAAGGTCTG
CGAAGACCTCGCCTCCCATCCTATTCTCTCTACCGCGCCCGCCGTCGACGCAATTGGATTCTCCCAAGGCGGCCAATTCTTGCGTGGCTACATA
TCCCGCTGCAATGCTCCACCCATCCGCTCTCTCCTGACCTTCGGTTCCCAACACAACGGCATTTCTGCCTTCCAAGCCTGTGGTCCTGCCGATT
TCCTCTGTCGCGGTGCTCAAACCCTTTTGCGATCCAACACCTGGTCAACCTTTGTCCAATCTCGTCTCGTACCCGCTCAATACTTCAGAGATCC
GGAAAACCTAGACTCTTACCTTGAATATTCCAATTTCCTTGCCGACATCAATAATGAGCGCGTTCTCAAGAACCAAACATATAAATCCAACATG
GAAAAATTGGAACGATTCGTAATGTATGTCTTTGAAGACGATACAACTGTCATCCCTAAGGAAAGTGGATGGTGGGCTGAAGTCAACGGCACGG
AAGTTACACCACTGAAAGAAAGAGCCATTTATAAAGAAGATTGGCTAGGTTTAAAGACATTGGATGAGGCCGGAAAATTAGTTTTCGAAACCAT
TCCAGGGGGACATATGACGTTAGGAGAGGAGATGCTAGAGAAGGCTTTCAAAGAGTATTTTGGTCCAGCAGGGAAGAAATTTGGG

>Bcin16g03460(MLST7), partial sequence [organism=Botrytis cinerea, strain D11_KL_tax4]
ATGAACCTCTTGATTTGAACTCTTCATTAATTGCAAGTGATGAACCCCTATTCTGCCCAGAGGATAGTTACAAGACGTATATCATTAGTCGAGA
ACCACTCATGATATATATTGACGGATTTTTGAAAGCGAATGAAAGTAAACATTTGGTTGATGTTAGGTGTGTTATTTATTCTGATGAAAAGAAC
AAGAGAGACTGATGAGATAGTGAGCCGCTTTATGAACCGTCAACTGTTTCTCACGGACAGGAAGTTACCATTGATCCTTCAGTTCGGAATTCTG
AAGTGGCGGTTTTAGAGAGGGATGAGGTGGTCAGGTGTATTGAGCATAGAGCGAGGGCATTTCAGGGGTGGAGGGGCGAGATGGGGATTGAGAA
GTTGAGGACGCAGAGGTATGGGGTTGGAGGACATTATGGGATGCATTTGTAAGTTTTGGGGGATTGACGAAGGCTTTTGTCTATTCTTCTACCA
GTACGGATCTTGAAAGAAAGATAGCATGAACACGAGGGCTAATAACTAGGAAATAGCGATTGGAGCGGAGGTAAACGTGGCATAGACCGATTTA
GTACTTTCATGGTCTATGTCGACGTATCCTCTGATATCGAAGGTGGAGGAACGGAATTCCCACGTATCGTGGGACCAAAAGGAGGAAGGTGGGA
GGACTACCTGGAAACTACGGAAGCATTGGATCCAAGAACTGGAGAAAATGTAACAGTAGAAGGGGTGACATTCAAACCAATCAAGGGAAATGCC
GTATTCTGGGAAAATACTGACAACAACGGGAGGGGCTATGAT

>Bcin12g03020(MLST8), partial sequence [organism=Botrytis cinerea, strain D11_KL_tax4]
CGATTGGCTGCGAAGAAAACTGCGCAGCACCGAGTCACCAAAAGCACGAATAAACCATCGGCAAGGGAAATATTCTCTGAACCACAACATGACA
CGAGCGCGGAAGAGTATATCGGGCGAGAAGCTTCATCAAGAGCACCAAAGCGACAACGAGTCGATGATAATTATAACTCTTACGGTGGAAGAAA
TGAAAATACAGCAGCTTATGCTTCCGGGAAACTTCCATCTGGAAGTATAAATGTTGGTGGAGGTAGGAAGACCACTTTTCAAGAAGAACCACGA
ACGGCATTTGTCGCTGGCAAGTTGCCCCCTGGAAGTATCAACATTGGTGGAAAGAAGGCTACCCAAATCGAGGACGAGGGTAGAGCAGCTTATG
CTTCCGGAAATTGCCTCCAGGAAGTATAAACTCTGGCGCAAAGAGGCGATTTCATTCCAAGATGAAACGAGACCGGCTTATGTCTCCGGAAAGC
TTCCACATGGTAGTATCGACGGTATGCGAAACCGTGAAATGGCTGCCGTCCACCGCGAAATTGCTGAGGGTGGAGGAAACCAGGCCAGGTTGT
CTCTTCTCTATTCACATTCAATCCTACTTCAAAGAAAACTTTTGATGAACCCGAAGAACAAGCGGAACCGGCAAAACCATCCAATGCGCCTTTG
ACAGAGGAAATGGCGACTTTCACCAATCTAGGGTTATCGAGAAGGCTTGCAGCCCATCTATCGACTAAACTCGATATGAAAGCTCCGACCGCCA
TTCAAAAAGCATCTGTACAGCAGTTGGTATCGGACGATAGCGATGCTTTCATACAAGCAGAGACTGGATCTGGAAAAACTTTGGCATATCTACT
ACCTATAGTCGAGCGAATATTAGCATTGAGTGAGAATGGCGTACAAA

>Bcin02g07770(MLST9), partial sequence [organism=Botrytis cinerea, strain D11_KL_tax4]
CAGCTTTCCCTTTCGGTCTTGGCATCTACAGTCATTGCCATCCCTACACCATCACAACTTGAGTCTCGGGCCGTTATCGATTCCGATGCCGTTG
TAGGATTTGCCGAAACTGTTCCCAGTGGGACCGTAGGAACAGTTTATGAGGCATATAAACCATTCCTTAAAGTCGTAAATGGATGCGTACCATT
CCCTGCCGTCGATGCATCGGGTAACACAGGGTATGTCCTTATATCTTTCTCTTCCACACGATTGCTATTGAGTCTCTAACATATTTAGTGGTG
GTTTGTCACCAACTGGCAGTAGCAATGGTGGTTGCAGCAGCAGTACCGGTCAAGTATATGTTCGAGGAGGACAAAGCGGATCAAACTACGCCAT
CATGTACTCCTGGTAAGTTCTCTCTAAACTTCTCCTTATAGATCCAACCTAACAAAATCTTAGGTACATGCCAAAGGACGAGCCCTCAACCGGT
ATTGGTCACCGTCACGATTGGGAAGGTGTAATTGTCTGGCTCTCCAGCGCCACCGCCACAACTGCCGACAACATCTTAGCCGTTTGTCCTTCCG
CCCACGGAGGCTGGGATTGTTCCACGGATGGCTATTCCCTTTCTGGTACCAGCCCTCTTATCAAGTACGAAAGTATCTGGCCCGTCGATCACTC
AATGGGTCTTACTAGTACTGTTGGTGGAAAACAACCTATGATTGCTTGGGAGTCTTTACCAACTGCTGCTCAAACTGCTCTTGAGAACACCGAT
TTCGGTGCTGCGAATGTTCCATTCATTCCGGCTGTTTTCACAGACAATCT

>Bcin04g02090(MLST10), partial sequence [organism=Botrytis cinerea, strain D11_KL_tax4]
CGGAGGATGATATGGCAAGGTCTATGATTACCAAAGCATTTGTAGGCATGAGTAGTAAACTGTCGAATATGACTATGAATGATGTTTACAAGCC
CTACATCCATGTAAGAAATGTAGAATAGAGAGATCAGTAACTGGAACTAATATCGTTTGTAGGCTTTCAAGTTACTTACGCAGTTCAACCCAAT
CACTACAGCTATTGCCGAATCCCCACTATTTCAAATGGCTGTCTCAGCAAATACCATCGAAAAGTACACACTGCTAGGCCCTTTCTTCAGAATA
TCTCCTCTGCAACAGGAAGTTACCAGGGAATACTTCAGTGCGCCAAAGACGATAGATAGGCGACACATTGCCACATCTCAAGATGCCTTACGAT
TGACCTTACAAACCCATCAAAAAGATTTACTTGATATCATCAACCACTTTGTTCGAGCAAGTCCAATCGCCAAAAGCAAAACCTTGGATTGGTT
CGCCTACATTGTGAATCAAAATCACAAGCGTCGAGCACTTCAGGTAGACCCGAAAGAAGTGTCTTCTGATGGCTTTATGCACAATGTCACTGTC
GTTCTAGATGGTCTTTGTGAGCCATTCATGGATACCACATTCTCGAAAATTTCGAAGATTGATATTGATTATCTAAGACGTGCGCCTCGTGTAG
ATATCAAGGACGAGACCAAGTTGAACGCTGACGAGAAGGCTTCTGAGAAGTATTATGAGGACACTGTTCCTGGCACTTCTAATTTCATCTCTGA
GGTCTTCTTTCTCACATTGGCTGCCCATCATTATGGTAGTGAAGCTCTTAATGCCACGCATAAAGAGTCTGGAGAAAGACATCAAATATATTCA
AAAGCAATTGACTGCCGTTGAAGCA

>Bcin01g07220(MLST1), partial sequence [organism=Botrytis cinerea, strain D11_M_E01]
ATGGCATATCTTGTTTCTTTTCGCATTATGCTTTGGAACGTGCTGGACAAGCTTGGGTTAGTGCTCTGCGTGTAGAAGCACTAAAGAAGATTCT
CGCACAACCGAAGTCATGGTTTGAGGAATCCAGGAATTCACCTAGCCGGTTGAACGAAGTTTTGGATAGGAACTCTGAGGAAATGCGTAATCTC
GTTGGCCGCTTTGCTGGTATTGTATTCACAGCATTTTTTATGCTATTGATATCAATCATTTGGGCTTTCGTGAATACATGGAAACTGACATTAG
TCTCGATGGCAACTGGGCCAGTTATATACGCTGTCACCAAAACGTTCAATCGCGTGAGTGGAAAATGGGAAAACAAGTGCAACTACGCCTCTGA
AATGACCACTGGCATATTTTCAGAGACGTTCTCCAACATCAAAGTGGTTCGGGCTTTACTCTGGAAACTTACTTTGAGACAAAACACACCAAAG
CTACAGAAGAACTTTATAAAGTTGGACTAATACGAGCAAACTACTCGGGATTGCTGTGGGGATTGACAGATGCGATGTCATTCTTCATCACTGC
AACTATCTTTTATTATGCCACGGTTCTCATTACCAAGAGAGAGATCAGTATCGCGACTGCACTACAGACTGTCAATCTTCTATTATTTGGTATT
TCTAATAGTACGAATATGCTGGCCATGATACCACAAATCAACTCTTCTCGCGTTACAGCTACGCATATGCTTGCATTAGCCAATCTCGATTCAT
CTTCCTCCCACGAAAATAAAGGAACCGAACGGCTTTCGACAATCTTTCCAATCAAATTCAACCGTCTTTCATTCACATACCCTACTCGTCCTGA
AAAACGAACGATATCATCCTTTTCTCTTTCCCTGATTCCTAACTCAACAACTGCACTTGTCGGACCCTCCGGCTCCGGAAAATCTACAATAGCT
GCTCTGCTCATTGGTCTCTATCCGCCAGATACTTCAACACCTCCACCGTTGACATTCAATCGCGCCTCCATAAGTAACTGTCACATTCCGTCTC
TCCGGGCTTCTCTCTCACTCGTTCCACAAATACCGATTCTATTTCCAGCTACCATTCTCCATAACATCATTTATGGTCTCCCAGAATCTTCTCC
TTGTGCTAGTCTTCCATCTGCTATTGCGTCAGCAAAAGATGCTGGGATCCATGAATTTATCACATCGCTTCCACAAGGTTATGATACTATGG

>Bcin05g07690(MLST2), partial sequence [organism=Botrytis cinerea, strain D11_M_E01]
ACCACTATCACCAAGTCCTTTTCTGCACTTTCCTCATCAAAAACGAACGAGAACATCATCAATAAATACTTTGCTGAAGTAATTGGGTTTTATT
TTGGCCAGGATGCCTTCTCTCTACGACAGCAAGCCTTCGATGATATGTTGTGGGTAGTTCTTGGCTGGCTGGATACTGTCAAATTCATTGATTT
ACATTCTGAATTGCACTATTCAAACGACTCTCAGCCAGAATGGTACGGACAACAATATAAACCTGCATTTGCACATCGAGCGCGACTATTTTGG
GAATTGGCTTCACAAGGATGGGATACTACTCTCTGTGGTGGTGGGATGATATGGTCACCATACCTTACTCCATACAAGAACGCAATTACCAATG
AACTCTATATCGCAGCTTCGATATCGATGTACCTATATTTCCCCGGAGATGACAATCAATCCCCATTTATGCTTTCCAACCCTTCGTATCCACC
TCACGATCCGAAATATCTACAGGCAGCTGTTGATGCTTACAAATGGCTGAATGGTTCCAACATGACGGATTTACAAGGATTATATGTCGACGGG
TACCATATCTCGAATCTTTCTGGCGGTGAAAACACCCATTGCGATTCTAGAAATGAGATGGTATATACCTACAATCAAGGTGTTTTGCTTACTG
GACAACGTGGTTTGTATGACGCAACCGCCGCACGATCATACCTTGTAGATGGCCACAAACTCATCGCGAATGTTATTAATGCCACAGGCTATGA
CCTGAAACACAATGTTGTCATCTCACCGCCACCCAAAGATGGTTCCGCATTGGCAAAGTGGTTTGGCCTGGGTAGGAATGGAATACTGGAAGAA
GGATGCGATTCAAGTGCTTCGTGTTCTCAAAATGGACAAACTTTCAAAGGCATATTCTTTCATCACTTGATTGCGTTCTGTAGTGATTTGCCAG
GGGAGCCTATTGCAGGGACGAAGGAAAGCTTAGAACTCGACAGAGTGTGGCATTCTGACAAATGCTCACAGTATACAAAAT

>Bcin06g01710(MLST3), partial sequence [organism=Botrytis cinerea, strain D11_M_E01]
GTGAGTCTGACTTTTGTATTTGAGCGTTAAGATAGACACTGATATACCAAGGCAATCACCTAATTTCATGGCGTTCTTCCCTGCATCATCTACC
TACCCTGGAATGCTGGGAGAATTATACTCAGCAGCTTTCACAGCACCTGCTTTCAATTGGATCTGTTCCCCTGCTGTGACAGAATTGGAGACGG
TTGTAATGGATTGGCTGGCCAAGCTTCTCAATCTCCCAGACTGTTATTTGTCTTCGACTCATGGTGGTGGTGTCATCCAAGGATCAGCCTCGGA
AGCTATCGTTACCGTTATGGTTGCTGCCCGCGATAAATATCTTCGTGAAACTACTGAAGGTCTGTCGGGCATTGAGCTCGAGGATGCGATTGCA
TATAAGAGGAGTAAGCTAGTTGCACTAGGAAGCGAAATGGCACACAGCTCCACGCAGAAAGCAGCGCAAATAGCTGGCGTTAGATTCCGATCGA
TTCCAGTACTCGCATCCAATGATTTCGCCATGACGGGTGATGATTTAGAGAAGGTGTTGAAAGAATGCAAATCTCAAGGATTGGAACCCTTCTA
TCTAACTTCGACTTTGGGAACAACATCTACATGCGCAGTTGACGACTTCGCATCTATTGCAACAGTACTTTCAAAATATGCACCTCCAGATGTT
GCAGGCGAGATCTGGGTTCACGTCGATGCTGCTTATGCAGGTGCAGCTTTGGTTTGCCCTGAATACCATCATCTAACATCGTCCTTCCAGCATT
TCCATTCCTTCGATATGAACATGCACAAATGGCTTCTGACAAATTTCGACGCTTCTTGTCTATATGTCAAGAAACGCAAAGATCTGATCGATGC
ACTCTCCATAACACCAAGTTATCTTCGCAACGAGTTTTCAGAGAGTGGACTCGTAACCGACTATCGGGACTGGCAAATTCCTCTCGGAAGACGC
TTCCGAAGCTTAAAGATTTGGTTTGTCCTCAGAACCTAC

>Bcin09g03030(MLST4), partial sequence [organism=Botrytis cinerea, strain D11_M_E01]
ACCCTTCAACCCCACCGCAGCTCCTATACCGAAAGCGAGTATCCTACCAATTCTCCTTCCACCTGCGACTTTAAGACCATTGGCTTTCCGCACT
TTCACAAAAAAGCATAGTTTAACATTGACGTCGTCGGCATTACAAGTGTTGGCTACTTTTATTGGAAAGCATTGTGGGACAGGATGGAGGGAAG
AAGGACTGGCAGAGAGAGTCTTGGAGGAGGTCGCCAAGAGTTGGAAGAATAGGAGTGGCGGTGTCATTGTCGAGGGCGAGGGAACGGAATTGAA
GGAGATTCTGAAAGCTTTGGAAGGGAATATGAGTGGTGGAAGGATAGTCATAGGAAGAGAGCTAAGCCGGCAGAATAGTTTAGTACTGGGATCA
TCACAATATGGAGAGGTCAATCATACAAGACTTGGGCTACGGCCAGGGAATATACCCAGAGAGGATAGTCAGTCAAGTTTGGGAATGTCAACGT
TGGAGGTCAATGACGAGGAAGATGAGGATGGCCTGATGGATCCAAGAAGGTGGTTAAAAGTCATTGATGCATTTGAGCAACCTCGACTGGTGTA
CAATGTTGCTAAAAAGCACTTTGATAGGTATGTTTCAATGATAAAATTTTATCTGAATCGACTAACTCAGTACAGAGATACCTCCAAACCTTCA
TTGTTCCCACCTGCGTCTCATAAAACACTCCTCTTCCAAAACCGCTATAATGTTATCCATCAACGTCTCCTTCGCAATGAATCTTTTCAAACGC
CCGCTTTTCAAGGTGGCAAATCTTCCCTTCAACGCAGCACGTCCGCCATTACCACCCAACAACAATCATACAAATTAACGCCGATAGCTAATCT
TCTCGGTCGCAATCGCAGCTCTCATATGCTTCTCGGTCTCCTCAGTATTTCACCCACTGGTACCCTCGCCATCAATGACCTGACGGGCAGTATC
GCTCTCGATCTTACACACGCAGCAGCCATTC

>Bcin11g01310(MLST5), partial sequence [organism=Botrytis cinerea, strain D11_M_E01]
ACTGACATGGACCTCATGTGGAACCGGCGTAGAATGCGCAACGCTTGAAGTTCCGCTCGAATATGGCGATGCAACGTCAACGGCAAAAGCCAGT

GTTGCGCTTGCTCGTTATCCTGCCACTGTTGCCGCGAGCAAGAAGCTCGGGTCTCTCTTGATAAACCCCGGTGGACCCGGTGCCTCTGGTGTTG
GCTTTGTGCAGTCTGGAGCCGGTGCCGCCGTCTCGACACTGAGTGGTGGGTTGTACGATATCATCGGATGGGATCCACGTGGAACCGGTGCTTC
GGCTCCTATTTTGGAATGTTTTGCAAATGCCAGTGCGGAGTATGATTTTAACAACGCGTTTCCATCTGCTCCGAATCTCTGGCTCGGACAATTT
GCGAATGCCAGCGCAAATTCTGCTGTTAGCTCTGCTATCACATCCTTTGACACTTCTGTCGCTGCTCTTGCAAAAGCTTGCGTGGCTCAGAAAT
CTCCCGCTCTTTACACCTCAACAGCAGCATATGTTGCTCGAGACATGGCAGCGATAGTCGATGCATTGGATGGGACCTCTGCAAAACTTAACTA
CTGGGGTTTCTCATATGGAACTATTTTCCTAGCTGAGTTTATCCAAACTTTCCCAGGCCGCGTGGGAAGAGTTCTTGCCGATGGTGTTTTCGAC
GCAAAGGCAAATGCACTCACATACGTTAGCCAACTTCCCAACGATCAACTCAGTGTTCGTGCTTCGTTGAACGATTTTGCAGCTTTCTGCACCA
CCGCCGGTAGTAAAGGTTGCTCTTTTGCCACCGCCCCTACTGGAACCACAGGTACTGTTGCTACCAGACTGGACAACATAATGAAGGATATGTT
CCTCAATCCTATTGTTGCTTCGGGCTTAAGCATCAG

>Bcin15g03910(MLST6), partial sequence [organism=Botrytis cinerea, strain D11_M_E01]
GCCAAAACACAAAATCATCCAACGATGAAGATGATACTCCACTTCCCTTGATTATCTGGCATGGACTCGGCGATAATTACAAAGCGGATGGTCT
TGCGCAAGTTGGAAAACTAGCTGAAGCTATTCATCCTGGGACTTTTGTCTACAATATTCATGTAGATGAGGATGCATCTGCAGATAGGACAGCT
ACCTTCTTTGGAAATCTCACTCGTGAGTACATCCCCTATTTTTCCTTTAAATATCATACTAACTCTCTTACCAAGTTCAAATCGAAAAGGTCTG
CGAAGACCTCGCCTCCCATCCTATTCTCTCTACCGCGCCCGCCGTCGACGCAATTGGATTCTCCCAAGGCGGCCAATTCTTGCGTGGCTACATA
TCCCGCTGCAATGCTCCACCCATCCGCTCTCTCCTGACCTTCGGTTCCCAACACAACGGCATTTCTGCCTTCCAAGCCTGTGGTCCTGCCGATT
TCCTCTGTCGCGGTGCTCAAACCCTTTTGCGATCCAACACCTGGTCAACCTTTGTCCAATCTCGTCTCGTACCCGCTCAATACTTCAGAGATCC
GGAAAACCTAGACTCTTACCTTGAATATTCCAATTTCCTTGCCGACATCAATAATGAGCGCGTTCTCAAGAACCAAACATATAAATCCAACATG
GAAAAATTGGAACGATTCGTAATGTATGTCTTTGAAGACGATACAACTGTCATCCCTAAGGAAAGTGGATGGTGGGCTGAAGTCAACGGCACGG
AAGTTACACCACTGAAAGAAAGAGCCATTTATAAAGAAGATTGGCTAGGTTTAAAGACATTGGATGAGGCCGGAAAATTAGTTTCGAAACCAT
TCCAGGGGGACATATGACGTTAGGAGAGGAGATGCTAGAGAAGGCTTTCAAAGAGTATTTTGGTCCAGCAGGGAAGAAATTTGGG

>Bcin16g03460(MLST7), partial sequence [organism=Botrytis cinerea, strain D11_M_E01]
ATGAACCTCTTGATTTGAACTCTTCATTAATTGCAAGTGATGAACCCCTATTCTGCCCAGAGGATAGTTACAAGACGTATATCATTAGTCGAGA
ACCACTCATGATATATATTGACGGATTTTTGAAAGCGAATGAAAGTAAACATTTGGTTGATGTTAGGTGTGTTATTTATTCTGATGAAATGAAC
AAGAGAGACTGATGAGATAGTGAGCCGCTTTATGAACCGTCAACTGTTTCTCACGGACAGGAAGTTACCATTGATCCTTCAGTTCGGAATTCTG
AAGTGGCGGTTTTAGAGAGGGATGAGGTGGTCAGGTGTATTGAGCATAGAGCGAGGGCATTTCAGGGGTGGAGGGGCGAGATGGGGATTGAGAA
GTTGAGGACGCAGAGGTATGGGGTTGGAGGACATTATGGGATGCATTTGTAAGTTTTGGGGGATTGACGAAGGCTTTTGTCTATTTTCTACCAG
TACGGATCTTGAAAGAAAGATAGCATGAACACGAGGGCTAATAACTAGGAAATAGCGATTGGAGCGGAGGTAAACGTGGCATAGACCGATTTAG
TACTTTCATGGTCTATGTCGACGTATCCTCTGATATCGAAGGTGGAGGAACGGAATTCCCACGTATCGTGGGACCAAAGGAGGAAGGTGGGAG
GACTTCCTGGAAACTACGGAAGCATTGGATCCAAGAACTGGAGAAAATGTAACAGTAGAAGGGGTGACATTCAAACCAATCAAGGGAAATGCCG
TATTCTGGGAAAATACTGACAACAACGGGAGGGGCTATGAT

>Bcin12g03020(MLST8), partial sequence [organism=Botrytis cinerea, strain D11_M_E01]
CGATTGGCTGCGAAGAAAACTGCGCAGCACCGAGTCACCAAAAGCACGAATAAACCATCGGCAAGGGAAATATTCTCTGAACCACAACATGACA
CGAGCGCGGAAGAGTATATCGGGCGAGAAGCTTCATCAAGAGCACCAAAGCGACAACGAGTCGATGATAATTATAACTCTTACGGTGGAAGAAA
TGAAAATACAGCAGCTTATGCTTCCGGGAAACTTCCATCTGGAAGTATAAATGTTGGTGGAGGTAGGAAGACCACTTTTCAAGAAGAACCACGA
ACGGCATTTGTCGCTGGCAAGTTGCCCCCTGGAAGTATCAACATTGGTGGAAAGAAGGCTACCCAAATCGAGGACGAGGGTAGAGCAGCTTATG
CTTCCGGAAAATTGCCTCCAGGAAGTATAAACTCTGGCGCAAAGAAGGCGATTTCATTCCAAGATGAAACGAGACCGGCTTATGTCTCCGGAAA
GCTTCCACATGGTAGTATCGACGGTATGCGAAACCGTGAAATGGCTGCCGTCCACCGCGAAATTGCTGAGGGTGGGAGGAAACCAGGCCAGGTT
GTCTCTTCTCTATTCACATTCAATCCTACTTCAAAGAAAACTTTTGATGAACCCGAAGAACAAGCGGAACCGGCAAAACCATCCAATGCGCCTT
TGACAGAGGAAATGGCGACTTTCACCAATCTAGGGTTATCGAGAAGGCTTGCAGCCCATCTATCGACTAAACTCGATATGAAAGCTCCGACCGC
CATTCAAAAAGCATCTGTACAGCAGTTGGTATCGGACGATAGCGATGCTTTCATACAAGCAGAGACTGGATCTGGAAAAACTTTGGCATATCTA
CTACCTATAGTCGAGCGAATATTAGCATTGAGTGAGAATGGCGTACAAAT

>Bcin02g07770(MLST9), partial sequence [organism=Botrytis cinerea, strain D11_M_E01]
CAGCTTTCCCTTTCGGTCTTGGCATCTACAGTCATTGCCATCCCTACACCATCACAACTTGAGTCTCGGGCCGTTATCGATTCCGATGCCGTTG
TAGGATTTGCCGAAACTGTTCCCAGTGGGACCGTAGGAACAGTTTATGAGGCATATAAACCATTCCTTAAAGTCGTAAATGGATGCGTACCATT
CCCTGCCGTCGATGCATCGGGTAACACAGGGTATGTCCTTATATCTTTCTCTTCCACACGATTGCTATTGAGTCTCTAACATATTTTAGTGGTG
GTTTGTCACCAACTGGCAGTAGCAATGGTGGTTGCAGCAGCAGTACCGGTCAAGTATATGTTCGAGGAGGACAAAGCGGATCAAACTACGCCAT
CATGTACTCCTGGTAAGTTCTCTCTAAACTTCTCCTTATAGATCCAACCTAACAAAATCTTAGGTACATGCCAAAGGACGAGCCCTCAACCGGT
ATTGGTCACCGTCACGATTGGGAAGGTGTAATTGTCTGGCTCTCCAGCGCCACCGCCACAACTGCCGACAACATCTTAGCCGTTTGTCCTTCCG
CCCACGGAGGCTGGGATTGTTCCACGGATGGCTATTCCCTTTCTGGTACCAGCCCTCTTATCAAGTACGAAAGTATCTGGCCCGTCGATCACTC
AATGGGTCTTACTAGTACTGTTGGTGGAAAACAACCTATGATTGCTTGGGAGTCTTTACCAACTGCTGCTCAAACTGCTCTTGAGAACACCGAT
TTCGGTGCTGCGAATGTTCCATTCATTCCGGCTGTTTTCACAGACAATCT

>Bcin04g02090(MLST10), partial sequence [organism=Botrytis cinerea, strain D11_M_E01]

GCGGAGGATGATATGGCAAAGTCTATGATTACCAAAGCATTTGTAGGCATGAGTAGTAAACTGTCGAATATGACTATGAATGATGTTTACAAGC
CCTACATCCATGTAAGAAATGTAGAATAGAGAGATCAGTAACTGGAACTAATATCGTTTGTAGGCTTTCAAGTTACTTACGCAGTTCAACCCAA
TCACTACAGCTATTGCCGAATCCCCACTGTTTCAAATGGCCGTCTCAGCAAATACCATCGAAAAGTACACACTGCTAGGCCCTTTCTTCAGAAT
ATCTCCTCTGCAACAGGAAGTTACCAGGGAATACTTCAGTGCGCCAAAGACGATAGATAGGCGACACATTGCCACATCTCAAGATGCGTTACGA
TTGACCTTACAAACCCATCAAAAAGATTTACTTGATATCATCAACCACTTTGTTCGAGCAAGTCCAATCGCAAAAAGCAAAACCCTGGATTGGT
TCGCCTACATTGTGAATCAAAATCACAAGCGTCGAGCACTTCAGGTAGACCCGAAAGAAGTGTCTTCTGATGGCTTTATGCACAATGTCACTGT
CGTTCTAGATGGTCTTTGTGAGCCATTCATGGATACCACATTCTCGAAAATTTCGAAGATTGATATTGATTATCTAAGACGTGCGCCTCGTGTA
GATATCAAGGACGAGACCAAGTTGAACGCTGACGAGAAGGCTTCTGAGAAGTATTATGAGGACACTGTTCCTGGCACTTCTAATTTCATCTCTG
AGGTCTTCTTTCTCACATTGGCTGCTCATCATTATGGTAGTGAAGCTCTTAATGCCACGCATAAGAGTCTGGAGAAAGACATCAAATATATTCA
AAAGCAATTGACTGCCGTTGAAGCAG

>Bcin01g07220(MLST1), partial sequence [organism=Botrytis cinerea, strain D11_M_E07]
ATGGCATATCTTGTTTCTTTTCGCATTATGCTTTGGAACGTGCTGGACAAGCTTGGGTTAGTGCTCTGCGTGTAGAAGCACTAAAGAAGATTCT
CGCACAACCGAAGTCATGGTTTGAGGAATCCAGGAATTCACCTAGCCGGTTGAACGAAGTTTTGGATAGGAACTCTGAGGAAATGCGTAATCTC
GTTGGCCGCTTTGCTGGTATTGTATTCACAGCATTTTTTATGCTATTGATATCAATCATTTGGGCTTTCGTGAATACATGGAAACTGACATTAG
TCTCGATGGCAACTGGGCCAGTTATATACGCTGTCACCAAAACGTTCAATCGCGTGAGTGGAAAATGGGAAAACAAGTGCAACTACGCCTCTGA
AATGACCACTGGCATATTTTCAGAGACGTTCTCCAACATCAAAGTGGTTCGGGCTTTTACTCTGGAAACTTACTTTGAGACAAAACACACCAAA
GCTACAGAAGAACTTTATAAAGTTGGACTAATACGAGCAAACTACTCGGGATTGCTGTGGGGATTGACAGATGCGATGTCATTCTTCATCACTG
CAACTATCTTTTATTATGCCACGGTTCTCATTACCAAGAGAGAGATCAGTATCGCGACTGCACTACAGACTGTCAATCTTCTATTATTTGGTAT
TTCTAATAGTACGAATATGCTGGCCATGATACCACAAATCAACTCTTCTCGCGTTACAGCTACGCATATGCTTGCATTAGCCAATCTCGATTCA
TCTTCCTCCCACGAAAATAAAGGAACCGAACGGCTTTCGACAATCTTTCCAATCAAATTCAACCGTCTTTCATTCACATACCCTACTCGTCCTG
AAAAACGAACGATATCATCCTTTTCTCTTTCCCTGATTCCTAACTCAACAACTGCACTTGTCGGACCCTCCGGCTCCGGAAAATCTACAATAGC
TGCTCTGCTCATTGGTCTCTATCCGCCAGATACTTCAACACCTCCACCGTTGACATTCAATCGCGTCTCCATAAGTAACTGTCACATTCCGTCT
CTCCGGGCTTCTCTCTCACTCGTTCCACAAATACCGATTCTATTTCCAGCTACCATTCTCCATAACATCATTTATGGTCTCCCAGAATCTTCTC
CTTGTGCTAGTCTTCCATCTGCTATTGCATCAGCAAAAGATGCTGGGATCCATGAATTTATCACATCGCTTCCACAAGGTTATGATACTAT

>Bcin05g07690(MLST2), partial sequence [organism=Botrytis cinerea, strain D11_M_E07]
ACCACTATCACCAAGTCCTTTTCTGCACTTTCCTCATCAAAAACGAACGAGAACATCATCAATAAATACTTTGCTGAAGTAATTGGGTTTTATT
TTGGCCAGGATGCCTTCTCTCTACGACAGCAAGCCTTCGATGATATGTTGTGGGTAGTTCTTGGCTGGCTGGATACTGTCAAATTCATTGATTT
ACATTCTGAATTGCACTATTCAAACGACTCTCAGCCAGAATGGTACGGACAACAATATAAACCTGCATTTGCACATCGAGCGCGACTATTTTGG
GAATTGGCTTCACAAGGATGGGATACTACTCTCTGTGGTGGTGGGATGATATGGTCACCATACCTTACTCCATACAAGAACGCAATTACCAATG
AACTCTATATCGCAGCTTCGATATCGATGTACCTATATTTCCCCGGAGATGACAATCAATCCCCATTTATGCTTTCCAACCCTTCGTATCCACC
TCACGATCCGAAATATCTACAGGCAGCTGTTGATGCTTACAAATGGCTGAATGGTTCCAACATGACGGATTTGCAAGGATTATATGTCGACGGG
TACCATATCTCGAATCTTTCTGGCGGTGAAAACACCCATTGCGATTCTAGAAATGAGATGGTATATACCTACAATCAAGGTGTTTTGCTTACTG
GACAACGTGGTTTGTATGACGCAACCGCCGCACGATCATACCTTGTAGATGGCCACAAACTCATCGCGAATGTTATTAATGCCACAGGCTATGA
CCTGAAACACAATGTTGTCATCTCACCGCCACCCAAAGATGGTTCCGCATTGGCAAAGTGGTTTGGCCTGGGTAGGAATGGAATACTGGAAGAA
GGATGCGATTCAAGTGCTTCGTGTTCTCAAAATGGACAAACTTTCAAAGGCATATTCTTTCATCACTTGATTGCGTTCTGTAGTGATTTGCCAG
GGGAGCCTATTGCAGGGACGAAGGAAAGCTTAGAACTCGACAGAGTGTGGCATTCTGACAAATGCTCACAGTATACAAAAT

>Bcin06g01710(MLST3), partial sequence [organism=Botrytis cinerea, strain D11_M_E07]
GTGAGTCTGACTTTTGTATTTGAGCGTTAAGATAGACACTGATATACCAAGGCAATCACCTAATTTCATGGCGTTCTTCCCTGCATCATCTACC
TACCCTGGAATGCTGGGAGAATTATACTCAGCAGCTTTCACAGCACCTGCTTTCAATTGGATCTGTTCCCCTGCTGTGACAGAATTGGAGACGG
TTGTAATGGATTGGCTGGCCAAGCTTCTCAATCTCCCAGACTGTTATTTGTCTTCGACTCATGGTGGTGGTGTCATCCAAGGATCAGCCTCGGA
AGCTATCGTTACCGTTATGGTTGCTGCCCGCGATAAATATCTTCGTGAAACTACTGAAGGTCTGTCGGGCATTGAGCTCGAGGATGCGATTGCA
TATAAGAGGAGTAAGCTAGTTGCACTAGGAAGCGAAATGGCACACAGCTCCACGCAGAAAGCAGCGCAAATAGCTGGCGTTAGATTCCGATCGA
TTCCAGTACTCGCATCCGATGATTTCGCCATGACGGGTGATGATTTAGAGAAGGTATTGAAAGAATGCAAATCTCAAGGATTGGAACCCTTCTA
TCTAACTTCGACTTTGGGAACAACATCTACATGCGCAGTTGACGACTTCGCATCTATTGCAACAGTACTTTCAAAATATGCACCTCCAGATGTT
GCAGGCGAGATCTGGGTTCACGTCGATGCTGCTTATGCAGGTGCAGCTTTGGTTTGCCCTGAATACCATCATCTAACATCGTCCTTCCAGCATT
TCCATTCCTTCGATATGAACATGCACAAATGGCTTCTGACAAATTTCGACGCTTCTTGTCTATATGTCAAGAAACGCAAAGATCTGATCGATGC
ACTCTCCATAACACCAAGTTATCTTCGCAACGAGTTTTCAGAGAGTGGACTCGTAACCGACTATCGGGACTGGCAAATTCCTCTCGGAAGACGC
TTCCGAAGCTTAAAGATTTGGTTTGTCCTCAGAACCTAC

>Bcin09g03030(MLST4), partial sequence [organism=Botrytis cinerea, strain D11_M_E07]
ACCCTTCAACCCCACCGCAGCTCCTATACCGAAAGCGAGTATCCTACCAATTCTCCTTCCACCTGCGACTTTAAGACCATTGGCTTTCCGCACT
TTCACAAAAAAGCATAGTTTAACATTGACGTCGTCGGCATTACAAGTGTTGGCTACTTTTATTGGAAAGCATTGTGGGACAGGATGGAGGGAAG
AAGGACTGGCAGAGAGAGTCTTGGAGGAGGTCGCCAAGAGTTGGAAGAATAGGAGTGGCGGTGTCATTGTCGAGGGCGAGGGAACGGAATTGAA
GGAGATTCTGAAAGCTTTGGAAGGGAATATGAGTGGTGGAAGGATAGTCATAGGAAGAGAGCTAAGCCGGCAGAATAGTTTAGTACTGGGATCA

TCACAATATGGAGAGGTCAATCATACAAGACTTGGGCTACGGCCAGGGAATATACCCAGAGAGGATAGTCAGTCAAGTTTGGGAATGTCAACGT
TGGAGGTCAATGACGAGGAAGATGAGGATGGCCTGATGGATCCAAGAAGGTGGTTAAAAGTCATTGATGCATTTGAGCAACCTCGACTGGTGTA
CAATGTTGCTAAAAAGCACTTTGATAGGTATGTTTCAATGATAAAATTTTATCTGAATCGACTAACTCAGTACAGAGATACCTCCAAACCTTCA
TTGTTCCCACCTGCGTCTCATAAAACACTCCTCTTCCAAAACCGCTATAATGTTATCCATCAACGTCTCCTTCGCAATGAATCTTTTCAAACGC
CCGCTTTTCAAGGTGGCAAATCTTCCCTTCAACGCAGCACGTCCGCCATTACCACCCAACAACAATCATACAAATTAACGCCGATAGCTAATCT
TCTCGGTCGCAATCGCAGCTCTCATATGCTTCTCGGTCTCCTCAGTATTTCACCCACTGGTACCCTCGCCATCAATGACCTGACGGGCAGTATC
GCTCTCGATCTTACACACGCAGCAGCCATTC

>Bcin11g01310(MLST5), partial sequence [organism=Botrytis cinerea, strain D11_M_E07]
ACTGACATGGACCTCATGTGGAACCGGCGTAGAATGCGCAACGCTTGAAGTTCCGCTCGAATATGGCGATGCAACGTCAACGGCAAAAGCCAGT
GTTGCGCTTGCTCGTTATCCTGCCACTGTTGCCGCGAGCAAGAAGCTCGGGTCTCTCTTGATAAACCCCGGTGGACCCGGTGCCTCTGGTGTTG
GCTTTGTGCAGTCTGGAGCCGGTGCCGCCGTCTCGACACTGAGTGGTGGATTGTACGATATCATCGGATGGGATCCACGTGGAACCGGTGCTTC
GGCTCCTATTTTGGAATGTTTTGCAAATGCCAGTGCGGAGTATGATTTTAACAACGCGTTTCCATCTGCCCCGAATCTCTGGCTCGGACAATTT
GCGAATGCCAGCGCAAATTCTGCTGTTAGCTCTGCTATCACATCCTTTGACACTTCTGTCGCTGCTCTTGCAAAAGCTTGCGTGGCTCAGAAAT
CTCCCGCTCTTTACACCTCAACAGCAGCATATGTTGCTCGAGACATGGCAGCGATAGTCGATGCATTGGATGGGACCTCTGCAAAACTTAACTA
CTGGGGTTTCTCATATGGAACTATTTTCCTAGCTGAGTTTATCCAAACTTTCCCAGGCCGTGTGGGAAGAGTTCTTGCCGATGGTGTTTTCGAC
GCAAAGGCAAATGCACTCACATACGTTAGCCAACTTCCCAACGATCAACTCAGTGTTCGTGCTTCGTTGAACGATTTTGCAGCTTTCTGCACCA
CCGCCGGTAGTAAAGGTTGCTCTTTTGCCACCGCCCCTACTGGAACCTCAGGTACTGTTGCTACCAGACTGGACAACATAATGAAGGATATGTT
CCTCAATCCTATTGTTGCTTCGGGCTTAAGCATCA

>Bcin15g03910(MLST6), partial sequence [organism=Botrytis cinerea, strain D11_M_E07]
GCCAAAACACAAAATCATCCAACGATGAAGATGATACTCCACTTCCCTTGATTATCTGGCATGGACTCGGCGATAATTACAAAGCGGATGGTCT
TGCGCAAGTTGGAAAACTAGCTGAAGCTATTCATCCTGGGACTTTTGTCTACAATATTCATGTAGATGAGGATGCATCTGCAGATAGGACAGCT
ACCTTCTTTGGAAATCTCACTCGTGAGTACATCCCCTATTTTTCCTTTAAATATCATACTAACTCTCTTACCAAGTTCAAATCGAAAAGGTCTG
CGAAGACCTCGCCTCCCATCCTATTCTCTCTACCGCGCCCGCCGTCGACGCAATTGGATTCTCCCAAGGCGGCCAATTCTTGCGTGGTTACATA
TCCCGCTGCAATGCTCCACCCATCCGCTCTCTCCTGACCTTCGGTTCCCAACACAACGGCATTTCTGCCTTCCAAGCCTGTGGTCCTGCCGATT
TCCTCTGTCGCGGTGCTCAAACCCTTTTGCGATCCAACACCTGGTCAACCTTTGTCCAATCTCGTCTCGTACCCGCTCAATACTTCAGAGATCC
GGAAAACCTAGACTCTTACCTTGAATATTCCAATTTCCTTGCCGACATCAATAATGAGCGCGTTCTCAAGAACCAAACATATAAATCCAACATG
GAAAAATTGGAACGATTCGTAATGTATGTCTTTGAAGACGACACAACTGTCATCCCTAAGGAAAGTGGATGGTGGGCTGAAGTCAACGGCACGG
AAGTTACACCACTGAAAGAAAGAGCCATTTATAAAGAAGATTGGCTAGGTTTAAAGACATTGGATGAGGCCGGAAAATTAGTTTCGAAACCAT
TCCAGGGGGACATATGACGTTAGGAGAGGAGATGCTAGAGAAGGCTTTCAAAGAGTATTTTGGTCCAGCAGGGAAGAAATTTGGGG

>Bcin16g03460(MLST7), partial sequence [organism=Botrytis cinerea, strain D11_M_E07]
ATGAACCTCTTGATTTGAACTCTTCATTAATTGCAAGTGATGAACCCCTATTCTGCCCAGAGGATAGTTACAAGACGTATATCATTAGTCGAGA
ACCACTCATGATATATATTGACGGATTTTTGAAAGCGAATGAAAGTAAACATTTGGTTGATGTTAGGTGTGTTATTTATTCTGATGAAATGAAC
AAGAGAGACTGATGAGATAGTGAGCCGCTTTATGAACCGTCAACTGTTTCTCACGGACAGGAAGTTACCATTGATCCTTCAGTTCGGAATTCTG
AAGTGGCGGTTTTAGAGAGGGATGAGGTGGTCAGGTGTATTGAGCATAGAGCGAGGGCATTTCAGGGGTGGAGGGGCGAGATGGGGATTGAGAA
GTTGAGGACGCAGAGGTATGGGGTTGGAGGACATTATGGGATGCATTTGTAAGTTTTGGGGGATTGACGAAGGCTTCTGTCTATTTTCTATCAG
CACGGATCTTGAAAGAAAGATAGCATGAACACGCGGGCTAATAACTAGGAAATAGCGATTGGAGCGGAGGTAAACGTGGCATAGACCGATTTAG
TACTTTCATGGTCTATGTCGACGTATCCTCTGATATCGAAGGTGGAGGAACGGAATTCCCACGTATCGTGGGACCAAAAGGAGGAAGGTGGGAG
GACTTCCTGGAAACTACGGAAGCATTGGATCCAAGAACTGGAGAAAATGTAACAGTAGAAGGGGTGACATTCAAACCAATCAAGGGAAATGCCG
TATTCTGGGAAAATACTGACAACAACGGGAGGGGCTATGAT

>Bcin12g03020(MLST8), partial sequence [organism=Botrytis cinerea, strain D11_M_E07]
ATCGATTGGCTGCGAAGAAAACTGCGCAGCACCGAGTCACCAAAAGCACGAATAAACCATCGGCAAGGGAAATATTCTCTGAACCACAACATGA
CACGAGCGCGGAAGAGTATATCGGGCGAGAAGCTTCATCAAGAGCACCAAAGCGACAACGAGTCGATGATAATTATAACTCTTACGGTGGAAGA
AATGAAAATACAGCAGCTTATGCTTCCGGGAAACTTCCATCTGGAAGTATAAATGTTGGCGGAGGTAGGAAGACCACTTTTCAAGAAGAACCAC
GAACGGCATTTGTCGCTGGCAAGTTGCCCCCTGGAAGTATCAACATTGGTGGAAAGAAGGCTACCCAAATCGAGGACGAGGGTAGAGCAGCTTA
TGCTTCCGGAAAATTGCCTCCAGGAAGTATAAACTCTGGCGCAAAGAAGGCGATTTCATTCCAAGATGAAACGAGACCGGCTTATGTCTCCGGA
AAGCTTCCACATGGTAGTATCGACGGTATGCGAAACCGTGAAATGGCTGCCGTCCACCGCGAAATTGCTGAGGGTGGGAGGAAACCAGGCCAGG
TTGTCTCTTCTCTATTCACATTCAATCCTACTTCAAAGAAAACTTTTGATGAACCCGAAGAACAAGCGGAACCGGCAAAACCATCCAATGCGCC
TTTGACAGAGGAAATGGCGACTTTCACCAATCTAGGGTTATCGAGAAGGCTTGCAGCCCATCTATCGACTAAACTCGATATGAAAGCTCCGACC
GCCATTCAAAAAGCATCTGTACAGCAGTTGGTATCGGACGATAGCGATGCTTTCATACAAGCAGAGACTGGATCTGGAAAAACTTTGGCATATC
TACTACCTATAGTCGAGCGAATATTAGCATTGAGTGAGAATGGCGTACAAAT

>Bcin02g07770(MLST9), partial sequence [organism=Botrytis cinerea, strain D11_M_E07]
ACAGCTTTCCCTTTCGGTCTTGGCATCTACAGTCATTGCCATCCCTACACCATCACAACTTGAGTCTCGGGCCGTTATCGATTCCGATGCCGTT

GTAGGATTTGCCGAAACTGTTCCCAGTGGGACCGTAGGAACAGTTTATGAGGCATATAAACCATTCCTTAAAGTCGTAAATGGATGCGTACCAT
TCCCTGCCGTCGATGCATCGGGTAACACAGGGTATGTCCTTATATCTTTCTCTTCCACACGATTGCTATTGAGTCTCTAACATATTTTAGTGGT
GGTTTGTCACCAACTGGCAGTAGCAATGGTGGTTGCAGCAGCAGTACCGGTCAAGTATATGTTCGAGGAGGACAAAGCGGATCAAACTACGCCA
TCATGTACTCCTGGTAAGTTCTCTCTAAACTTCTCCTTATAGATCCAACCTAACAAAATCTTAGGTACATGCCAAAGGACGAGCCCTCAACCGG
TATTGGTCACCGTCACGATTGGGAAGGTGTAATTGTCTGGCTCTCCAGCGCCACCGCCACAACTGCCGACAACATCTTAGCCGTTTGTCCTTCC
GCCCACGGAGGCTGGGATTGTTCCACGGATGGCTATTCCCTTTCTGGTACCAGCCCTCTTATCAAGTACGAAAGTACCTGGCCCGTCGATCACT
CAATGGGTCTTACTAGTACTGTTGGTGGAAAACAACCTATGATTGCTTGGGAGTCTTTACCAACTGCTGCTCAAACTGCTCTTGAGAACACCGA
TTTCGGTGCTGCGAATGTTCCATTCATTCCGGCTGTTTTCACAGACAATCT

>Bcin04g02090(MLST10), partial sequence [organism=Botrytis cinerea, strain D11_M_E07]
TGCGGAGGATGATATGGCAAAGTCTATGATTACCAAAGCATTTGTAGGCATGAGTAGTAAACTGTCGAATATGACTATGAATGATGTTTACAAG
CCCTACATCCATGTAAGAAATGTAGAATAGAGAGATCAGTAACTGGAACTAATATCGTTTGTAGGCTTTCAAGTTACTTACGCAGTTCAACCCA
ATCACTACAGCTATTGCCGAATCCCCACTGTTTCAAATGGCCGTCTCAGCAAATACCATCGAAAAGTACACACTGCTAGGCCCTTTCTTCAGAA
TATCGCCTCTGCAACAGGAAAGTTACCAGGGAATACTTCAGTGCGCCAAAGACGATAGATAGGCGACACATTGCCACATCTCAAGATGCGTTAC
GATTGACCTTACAAACCCATCAAAAAGATTTACTTGATATCATCAACCACTTTGTTCGAGCAAGTCCAATCGCAAAAAGCAAAACCCTGGATTG
GTTCGCCTACATTGTGAATCAAAATCACAAGCGTCGAGCACTTCAGGTAGACCCGAAAGAAGTGTCTTCTGATGGCTTTATGCACAATGTCACT
GTCGTTCTAGATGGTCTTTGTGAGCCATTCATGGATACCACATTCTCGAAAATTTCGAAGATTGATATTGATTATCTAAGACGTGCGCCTCGTG
TAGATATCAAGGACGAGACCAAGTTGAACGCTGACGAGAAGGCTTCTGAGAAGTATTATGAGGACACTGTTCCTGGCACTTCTAATTTCATCTC
TGAGGTCTTCTTTCTCACATTGGCTGCTTCATCATTATGGTAGTGAAGCTCTTAATGCCACGCATAAGAGTCTGGAGAAAGACATCAAATATAT
CAAAAGCAATTGACTGCCGTTGAAGCA

>Bcin01g07220(MLST1), partial sequence [organism=Botrytis cinerea, strain D11_M_W04]
ATGGCATATCTTGTTTCTTTTCGCATTATGCTTTGGAACGTGCTGGACAAGCTTGGGTTAGTGCTCTGCGTGTAGAAGCACTAAAGAAGATTCT
CGCACAACCGAAGTCATGGTTTGAGGAATCCAGGAATTCACCTGGCCGGTTGAACGAAGTTTTGGATAGGAACTCTGAGGAAATGCGTAATCTC
GTTGGCCGCTTTGCTGGTATTGTATTCACAGCATTTTTTATGCTATTGATATCAATCATTTGGGCTTTTGTGAATACATGGAAACTGACATTAG
TCTCGATGGCAACTGGGCCAGTTATATACGCTGTCACCAAAACATTCAATCGCGTGAGTGGAAAATGGGAAAACAAGTGCAACTACGCCTCTGA
AATGACCACTGGCAAATTTTCAGAGACTTTCTCCAACATCAAAGTGGTTCGGGCTTTTACTCTGGAAACTTACTTTGAGACAAAACACACCAAA
GCTACAGAAGAACTTTATAAAAGTTGGACTAATACGAGCAAACTACTCGGGATTGCTGTGGGGATTGACAGATGCGATGTCATTCTTCATCACT
GCAACTATCTTTTATTATGCCACGGTTCTCATTACCAAGAGAGAGATCAGTATCGCGACTGCACTACAGACTGTCAATCTTCTATTATTTGGTA
TTTCTAATAGTACGAATATGCTGGCCATGATACCACAAATCAACTCTTCTCGCGTTACAGCTACGCATATGCTTGCATTAGCCAATCTCGATTC
ATCTTCCTCCCACGAAAATAAAGGAACCGAACGGCTTTCGACAATCCTTCCAATCAAATTCAACCGTCTTTCATTCACATACCCTACTCGTCCT
GAAAAACGAACGATATCATCCTTTTCTCTTTCCCTGATTCCTAACTCAACAACTGCACTTGTCGGACCCTCCGGCTCCGGAAAATCTACAATAG
CTGCTCTGCTCATTGGTCTCTATCCGCCAGATACTTCAACACCTCCACCGTTGACATTCAATCGCGTCTCCATAAGTAACTGTCACATTCCGTC
TCTCCGGGCTTCTCTCTCACTCGTTCCACAAATACCGATTCTATTTCCAGCTACCATTCTCCATAACATCATTTATGGTCTCCCAGAATCTTCT
CCTTGTGCTAGTCTTCCATCAGCTATTGCATCAGCAAAAGATGCTGGGATCCATGAATTTATCACATCGCTTCCACAAGGTTATGATACTATG

>Bcin05g07690(MLST2), partial sequence [organism=Botrytis cinerea, strain D11_M_W04]
ACCACTATCACCAAGTCCTTTTCTGCACTTTCCTCATCAAAAACGAACGAGAACATCATCAATAAATACTTTGCTGAAGTAATTGGGTTTTATT
TTGGCCAGGATGCCTTCTCTCTACGACAGCAAGCCTTCGATGATATGTTGTGGGTAGTTCTTGGCTGGCTGGATACTGTCAAATTCATTGATTT
ACATTCTGAATTGCACTATTCAAACGACTCTCAGCCAGAATGGTACGGACAACAATATAAACCTGCATTTGCACATCGAGCGCGACTATTTTGG
GAATTGGCTTCACAAGGATGGGATACTACTCTCTGTGGTGGTGGGATGATATGGTCACCATACCTTACTCCATACAAGAACGCAATTACCAATG
AACTCTATATCGCAGCTTCGATATCGATGTACCTATATTTCCCCGGAGATGACAATCAATCCCCATTTATGCTTTCCAACCCTTCATATCCACC
TCACGATCCGAAATATCTACAGGCAGCTGTTGATGCTTACAAATGGCTGAATGGTTCCAACATGACGGATTTACAAGGATTATATGTCGACGGG
TACCATATCTCGAATCTTTCTGGCGGTGAAAACACCCATTGCGATTCTAGAAATGAGATGGTATATACCTACAATCAAGGTGTTTTGCTTACTG
GACAACGTGGTTTGTATGACGCAACCGCCGCACGATCATACCTTGTAGATGGCCACAAACTCATCGCGAATGTTATTAATGCCACAGGCTATGA
CCTGAAACACAATGTTGTCATCTCACCGCCACCCAAAGATGGTTCCGCATTGGCAAAGTGGTTTGGCCTGGGTAGGAATGGAATACTGGAAGAA
GGATGCGATTCAAGTGCTTCGTGTTCTCAAAATGGACAAACTTTCAAAGGCATATTCTTTCATCACTTGATTGCGTTCTGTAGTGATTTGCCAG
GGGAGCCTATTGCAGGGACGAAGGAAAGCTTAGAACTCGACAGAGTGTGGCATTCTGACAAATGCTCACAGTATACAAAAT

>Bcin06g01710(MLST3), partial sequence [organism=Botrytis cinerea, strain D11_M_W04]
GTGAGTCTGACTTTTGTATTTGAGCGTTAAGATAGACTCTGATATACCAAGGCAATCACCTAATTTCATGGCGTTCTTCCCTGCATCATCTACC
TACCCTGGAATGCTGGGAGAATTATACTCAGCAGCTTTCACAGCTCCTGCTTTCAATTGGATCTGTTCCCCTGCTGTGACAGAATTGGAGACGG
TTGTAATGGATTGGTTGGCCAAGCTTCTCAATCTCCCAGACTGTTATTTGTCGTCGACTCATGGTGGTGGTGTTATCCAAGGATCAGCCTCGGA
AGCTATCGTTACCGTTATGGTTGCTGCCCGCGATAAGTATCTTCGTGAAACCACTGAAGGTCTGTCGGAATTGAACTCGAGGATGCGATTGCAT
ATAAGAGGAGTAAGCTAGTTGCACTAGGAAGCGAAATGGCACACAGCTCCACGCAGAAAGCAGCGCAGATAGCTGGCGTTAGATTCCGATCGAT
TCCAGTACTCGCATCCAATGATTTCGCCATGACGGGTGATGATTTAGAGAAGGTATTGAAAGAATGCAAATCTCAAGGATTGGAACCCTTCTAT
CTAACTTCGACGTTGGGAACAACATCTACATGCGCAGTTGACGACTTCGCATCTATTGCAACAGTACTTTCAAAATATGCACCTCCAGATGTTG

CAGGCGAGATCTGGGTTCACGTCGATGCTGCTTATGCAGGTGCAGCTTTGGTTTGCCCTGAATACCATCATCTAACATCGTCCTTCCAGCATTT
CCATTCCTTTGATATGAACATGCACAAATGGCTTCTGACAAATTTCGACGCTTCTTGCCTATATGTCAAGAAACGCAAAGATCTGATCGATGCA
CTCTCCATAACACCAAGTTATCTTCGCAACGAGTTTTCAGAGAGTGGACTCGTAACCGACTATCGGGACTGGCAAATTCCTCTCGGAAGACGCT
TCCGAAGCTTAAAGATTTGGTTTGTCCTCAGAACCTAC

>Bcin09g03030(MLST4), partial sequence [organism=Botrytis cinerea, strain D11_M_W04]
ACCCTTCAACCCCACCGCAGCTCCTATACCGAAAGCGAGTATCCTACCAATTCTCCTTCCACCTGCGACTTTAAGACCATTGGCTTTCCGCACT
TTCACAAAAAAGCATAGTTTAACATTGACGTCGTCGGCATTACAAGTGTTGGCTACTTTTATTGGAAAGCATTGTGGGACAGGATGGAGGGAAG
AAGGACTGGCAGAGAGAGTCTTGGAGGAGGTCGCCAAGAGTTGGAAGAATAGGAGTGGCGGTGTCATTGTCGAGGGCGAGGGAACGGAATTGAA
GGAGATTCTGAAAGCTTTGGAAGGGAATATGAGTGGTGGAAGGATAGTCATAGGAAGAGAGCTAAGCCGGCAGAATAGTTTAGTACTGGGATCA
TCACAATATGGAGAGGTCAATCATACAAGACTTGGGCTACGGCCAGGGAATATACCCAGAGAGGATAGTCAGTCAAGTTTGGGAATGTCAACGT
TGGAGGTCAATGACGAGGAAGATGAGGATGGCCTGATGGATCCAAGAAGGTGGTTAAAAGTCATTGATGCATTTGAGCAACCTCGACTGGTGTA
CAATGTTGCTAAAAAGCACTTTGATAGGTATGTTTCAATGATAAAATTTTATCTGAATCGACTAACTCAGTACAGAGATACCTCCAAACCTTCA
TTGTTCCCACCTGCGTCTCATAAAACACTCCTCTTCCAAAACCGCTATAATGTTATCCATCAACGTCTCCTTCGCAATGAATCTTTTCAAACGC
CCGCTTTTCAAGGTGGCAAATCTTCCCTTCAACGCAGCACGTCCGCCATTACCACCCAACAACAATCATACAAATTAACGCCGATAGCTAATCT
TCTCGGTCGCAATCGCAGCTCTCATATGCTTCTCGGTCTCCTCAGTATTTCACCCACTGGTACCCTCGCCATCAATGACCTGACGGGCAGTATC
GCTCTCGATCTTACACACGCAGCAGCCATTCC

>Bcin11g01310(MLST5), partial sequence [organism=Botrytis cinerea, strain D11_M_W04]
ACTGACATGGACCTCATGTGGAACCGGCGTAGAATGCGCAACGCTTGAAGTTCCGCTCGAATATGGCGATGCAACGTCAACGGCAAAAGCCAGT
GTTGCGCTTGCTCGTTATCCTGCCACTGTTGCCGCGAGCAAGAAGCTCGGGTCTCTCTTGATAAACCCCGGTGGACCCGGTGCCTCTGGTGTTG
GCTTTGTGCAGTCTGGAGCCGGTGCCGCCGTCTCGACACTGAGTGGTGGATTGTACGATATCATCGGATGGGATCCACGTGGAACCGGTGCTTC
GGCTCCTATTTTGGAATGTTTTGCAAATGCCAGTGCGGAGTATGATTTTAACAACGCGTTTCCATCTGCTCCGAATCTCTGGCTCGGACAATTT
GCGAATGCCAGCGCAAATTCTGCTGTTAGCTCTGCTATCACATCCTTTGACACTTCTGTCGCTGCTCTTGCAAAAGCTTGCGTGGCTCAGAAAT
CTCCCGCTCTTTACACCTCAACAGCAGCATATGTTGCTCGAGACATGGCAGCGATAGTCGATGCATTGGATGGGACCTCTGCAAAACTTAACTA
CTGGGGTTTCTCATATGGAACTATTTTCCTAGCTGAGTTTATCCAAACTTTCCCAGGCCGTGTGGGAAGAGTTCTTGCCGATGGTGTTTTCGAC
GCAAAGGCAAATGCACTCACATACGTTAGCCAACTTCCCAACGATCAACTCAGTGTTCGTGCTTCGTTGAACGATTTTGCAGCTTTCTGCACCA
CCGCCGGTAGTAAAGGTTGCTCTTTTGCCACCGCCCCTACTGGAACCTCAGGTACTGTTGCTACCAGACTGGACAACATAATGAAGGATATGTT
CCTCAATCCTATTGTTGCTTCGGGCTTAAGCATCA

>Bcin15g03910(MLST6), partial sequence [organism=Botrytis cinerea, strain D11_M_W04]
GCCAAAACACAAAATCATCCAACGATGAAGATGATACTCCACTTCCCTTGATTATCTGGCATGGACTCGGCGATAATTACAAAGCGGATGGTCT
TGCGCAAGTTGGAAAACTAGCTGAAGCTATTCATCCTGGGACTTTTGTCTACAATATTCATGTAGATGAGGATGCATCTGCAGATAGGACAGCT
ACCTTCTTTGGAAATCTCACTCGTGAGTACATCCCCTATTTTTCCTTTAAATATCATACTAACTCTCTTACCAAGTTCAAATCGAAAAGGTCTG
CGAAGACCTCGCCTCCCATCCTATTCTCTCTACCGCGCCCGCCGTCGACGCAATTGGATTCTCCCAAGGCGGCCAATTCTTGCGTGGTTACATA
TCCCGCTGCAATGCTCCACCCATCCGCTCTCTCCTGACCTTTGGTTCCCAACACAACGGCATTTCTGCCTTCCAAGCCTGTGGTCCTGCCGATT
TCCTCTGTCGCGGTGCTCAAACCCTTTTGCGATCCAACACCTGGTCAACCTTTGTCCAATCTCGTCTCGTACCCGCTCAATACTTCAGAGATCC
GGAAAACCTAGACTCTTACCTTGAATATTCCAATTTCCTTGCCGACATCAATAATGAGCGCGTTCTCAAGAACCAAACATATAAATCCAACATG
GAAAAATTGGAACGATTCGTAATGTATGTCTTTGAAGACGACACAACTGTCATTCCTAAGGAAAGTGGATGGTGGGCTGAAGTCAACGGCACGG
AAGTTACACCACTGAAAGAAAGAGCCATTTATAAAGAAGATTGGCTAGGTTTAAAGACATTGGATGAGGCCGGAAAATTAGTTTTCGAAACCAT
TCCAGGGGGACATATGACGTTAGGAGAGGAGATGCTAGAGAAGGCTTTCAAAGAGTATTTTGGTCCAGCAGGGAAGAAATTTGGGG

>Bcin16g03460(MLST7), partial sequence [organism=Botrytis cinerea, strain D11_M_W04]
ATGAACCTCTTGATTTGAACTCTTCATTAATTGCAAGTGATGAACCCCTATTCTGCCCAGAGGATAGTTACAAGACGTATATCATTAGTCGAGA
ACCACTCATGATATATATTGACGGATTTTTGAAAGCGAATGAAAGTAAACATTTGGTTGATGTTAGGTGTGTTATTTATTCTGATGAAATGAAC
AAGAGAGACTGATGAGATAGTGAGCCGCTTTATGAACCGTCAACTGTTTCTCACGGACAGGAAGTTACCATTGATCCTTCAGTTCGGAATTCTG
AAGTGGCGGTTTTAGAGAGGGATGAGGTGGTCAGGTGTATTGAGCATAGAGCGAGGGCATTTCAGGGGTGGAGGGGCGAGATGGGGATTGAGAA
GTTGAGGACGCAGAGGTATGGGGTTGGAGGACATTATGGGATGCATTTGTAAGTTTTGGGGGATTGACGAAGGCTTTTGTCTATTTTCTACCAG
TACGGATCTTGAAAGAAAGATAGCATGAACACGAGGGCTAATAACTAGGAAATAGCGATTGGAGCGGAGGTAAACGTGGCATAGACCGATTTAG
TACTTTCATGGTCTATGTCGACGTATCCTCTGATATCGAAGGTGGAGGAACGGAATTCCCACGTATCGTGGGACCAAAAGGAGGAAGGTGGGAG
GACTTCCTGGAAACTACGGAAGCATTGGATCCAAGAACTGGAGAAAATGTAACAGTAGAAGGGGTGACATTCAAACCAATCAAGGGAAATGCCG
TATTCTGGGAAAATACTGACAACAACGGGAGGGGCTATGATG

>Bcin12g03020(MLST8), partial sequence [organism=Botrytis cinerea, strain D11_M_W04]
CGATTGGCTGCGAAGAAAACTGCGCAGCACCGAGTCACCAAAAGCACGAATAAACCATCGGCAAGGGAAATATTCTCTGAACCACAACATGACA
CGAGCGCGGAAGAGTATATCGGGCGAGAAGCTTCATCAAGAGCACCAAAGCGACAACGAGTCGATGATAATTATAACTCTTACGGTGGAAGAAA
TGAAAATACAGCAGCTTATGCTTCCGGGAAACTTCCATCTGGAAGTATAAATGTTGGTGGAGGTAGGAAGACCACTTTTCAAGAAGAACCACGA

ACGGCATTTGTCGCTGGCAAGTTGCCCCCTGGAAGTATCAACATTGGTGGAAAGAAGGCTACCCAAATCGAGGACGAGGGTAGAGCAGCTTATG
CTTCCGGAAAATTGCCTCCAGGAAGTATAAACTCTGGCGCAAAGAAGGCGATTTCATTCCAAGATGAAACGAGACCGGCTTATGTCTCCGGAAA
GCTTCCACATGGTAGTATCGACGGTATGCGAAACCGTGAAATGGCTGCCGTCCACCGCGAAATTGCTGAGGGTGGAGGAAACCAGGCCAGGTT
GTCTCTTCTCTATTCACATTCAATCCTACTTCAAAGAAAACTTTTGATGAACCCGAAGAACAAGCGGAACCGGCAAAACCATCCAATGCGCCTT
TGACAGAGGAAATGGCGACTTTCACCAATCTAGGGTTATCGAGAAGGCTTGCAGCCCATCTATCGACTAAACTCGATATGAAAGCTCCGACCGC
CATTCAAAAAGCATCTGTACAGCAGTTGGTATCGGACGATAGCGATGCTTTCATACAAGCAGAGACTGGATCTGGAAAAACTTTGGCATATCTA
CTACCTATAGTCGAGCGAATATTAGCATTGAGTGAGAATGGCGTACAAA

>Bcin02g07770(MLST9), partial sequence [organism=Botrytis cinerea, strain D11_M_W04]
CAGCTTTCCCTTTCGGTCTTGGCATCTACAGTCATTGCCATCCCTACACCATCACAACTTGAGTCTCGGGCCGTTATCGATTCCGATGCCGTTG
TAGGATTTGCCGAAACTGTTCCCAGTGGGACCGTAGGAACAGTTTATGAGGCATATAAACCATTCCTTAAAGTCGTAAATGGATGCGTACCATT
CCCTGCCGTCGATGCATCGGGTAACACAGGGTATGTCCTTATATCTTTCTCTTCCACACGATTGCTATTGAGTCTCTAACATATTTTAGTGGTG
GTTTGTCACCAACTGGCAGTAGCAATGGTGGTTGCAGCAGCAGTACCGGTCAAGTATATGTTCGAGGAGGACAAAGCGGATCAAACTACGCCAT
CATGTACTCCTGGTAAGTTCTCTCTAAACTTCTCCTTATAGATCCAACCTAACAAAATCTTAGGTACATGCCAAAGGACGAGCCCTCAACCGGT
ATTGGTCACCGTCACGATTGGGAAGGTGTAATTGTCTGGCTCTCCAGCGCCACCGCCACAACTGCCGACAACATCTTAGCCGTTTGTCCTTCCG
CCCACGGAGGCTGGGATTGTTCCACGGATGGCTATTCCCTTTCTGGTACCAGCCCTCTTATCAAGTACGAAAGTATCTGGCCCGTCGATCACTC
AATGGGTCTTACTAGTACTGTTGGTGGAAAACAACCTATGATTGCTTGGGAGTCTTTACCAACTGCTGCTCAAACTGCTCTTGAGAACACCGAT
TTCGGTGCTGCGAATGTTCCATTCATTCCGGCTGTTTTCACAGACAATCT

>Bcin04g02090(MLST10), partial sequence [organism=Botrytis cinerea, strain D11_M_W04]
CGGAGGATGATATGGCAAAGTCTATGATTACCAAAGCATTTGTAGGCATGAGTAGTAAACTGTCGAATATGACTATGAATGATGTTTACAAGCC
CTACATCCATGTAAGAAATGTAGAATAGAGAGATCAGTAACTGGAACTAATATCGTTTGTAGGCTTTCAAGTTACTTACGCAGTTCAACCCAAT
CACTACAGCTATTGCCGAATCCCCACTGTTTCAAATGGCCGTCTCAGCAAATACCATCGAAAAGTACACACTGCTAGGCCCTTTCTTCAGAATA
TCTCCTCTGCAACAGGAAGTTACCAGGGAATACTTCAGTGCGCCAAAGACGATAGATAGGCGACACATTGCCACATCTCAAGATGCGTTACGAT
TGACCTTACAAACCCATCAAAAAGATTTACTTGATATCATCAACCACTTTGTTCGAGCAAGTCCAATCGCAAAAAGCAAAACCCTGGATTGGTT
CGCCTACATTGTGAATCAAAATCACAAGCGTCGAGCACTTCAGGTAGACCCGAAAGAAGTGTCTTCTGATGGCTTTATGCACAATGTCACTGTC
GTTCTAGATGGTCTTTGTGAGCCATTCATGGATACCACATTCTCGAAAATTTCGAAGATTGATATTGATTATCTAAGACGTGCGCCTCGTGTAG
ATATCAAGGACGAGACCAAGTTGAACGCTGACGAGAAGGCTTCTGAGAAGTATTATGAGGACACTGTTCCTGGCACTTCTAATTTCATCTCTGA
GGTCTTCTTTCTCACATTGGCTGCTCATCATTATGGTAGTGAAGCTCTTAATGCCACGCATAAGAGTCTGGAGAAAGACATCAAATATATTCAA
AAGCAATTGACTGCCGTTGAAGCAGA

>Bcin01g07220(MLST1), partial sequence [organism=Botrytis cinerea, strain D11_T_B09]
GATGGCATATCTTGTTTCTTTTCGCATTATGCTTTGGAACGTGCTGGACAAGCTTGGGTTAGTGCTCTGCGTGTAGAAGCACTAAAGAAGATTC
TCGCACAACCGAAGTCATGGTTTGAGGAATCCAGGAATTCACCTGGCCGGTTGAACGAAGTTTTGGATAGGAACTCTGAGGAAATGCGTAATCT
TGTTGGCCGCTTTGCTGGTATTGTATTCACAGCATTTTTCATGCTATTGATATCAATCATTTGGGCTTTCGTGAATACATGGAAACTGACATTA
GTCTCGATGGCAACTGGGCCAGTTATATACGCTGTCACCAAAACGTTCAATCGCGTGAGTGAAAAATGGGAAAACAAGTGCAACTACGCCTCTG
AAATGACCACTGGCATATTTTCAGAGACTTTCTCCAACATCAAAGTGGTTCGGGCTTTTACTCTGGAAACTTACTTTGAGACAAAACACACCAA
AGCTACAGAAGAACTTTATAAAGTTGGACTAATACGAGCAAACTACTCGGGATTGCTGTGGGGATTGACAGATGCGATGTCATTCTTCATCACT
GCAACTATCTTTTATTATGCCACGGTTCTCATTACCAAGAGAGAGATCAGTATCGCGACTGCACTACAGACTGTCAATCTTCTATTATTTGGTA
TTTCTAATAGTACGAATATGCTGGCCATGATACCACAAATCAACTCTTCTCGCGTTACAGCTACGCATATGCTTGCATTAGCCAATCTCGATTC
ATCTTCCTCCCACGAAAATAAAGGAACCGAACGGCTTTCGACAATCTTTCCAATCAAATTCAACCGTCTTTCATTCACATACCCTACTCGTCCC
GAAAAACGAACGATATCATCCTTTTCTCTTTCCCTGATTCCTAACTCAACAACTGCACTTGTCGGACCCTCCGGCTCCGGAAAATCTACAATAG
CTGCTCTGCTCATTGGTCTCTATCCGCCAGATACTTCAACACCTTCACCGTTGACATTCAATCGCGTCTCCATAAGTAACTGTCACATTCCGTC
TCTCCGGGCTTCTCTCTCACTCGTTCCACAAATACCGATTCTATTTCCAGCTACCATTCTCCATAACATCATTTATGGTCTCCCAGAATCTTCT
CCTTGTGCTAGTCTTCCATCTGCTATTGCATCAGCAAAAGATGCTGGGATCCATGAATTTATCACATCGCTTCCACAAGGTTATGATACTAT

>Bcin05g07690(MLST2), partial sequence [organism=Botrytis cinerea, strain D11_T_B09]
TCACCACTATCACCAAGTCCTTTTCTGCACTTTCCTCATCAAAAACGAACGAGAACATCATCAATAAATACTTTGCTGAAGTAATTGGGTTTTA
TTTTGGCCAGGATGCCTTCTCTCTACGACAGCAAGCCTTCGATGATATGTTGTGGGTAGTTCTTGGCTGGCTGGATACTGTCAAATTTATTGAT
TTACATTCTGAATTGCACTATTCAAACGATTCTCAGCCAGAATGGTACGGACAACAATATAAACCTGCATTTGCACATCGAGCGCGACTATTTT
GGGAATTGGCTTCACAAGGATGGGATACTACTCTCTGTGGTGGTGGGATGATATGGTCACCATACCTTACTCCATACAAGAACGCAATTACCAA
TGAACTCTATATCGCAGCTTCGATATCGATGTACCTATATTTCCCCGGAGATGACAATCAATCCCCATTTATGCTTTCCAACCCTTCATATCCA
CCTCACGATCCGAAATATCTACAGGCAGCTGTTGATGCTTACAAATGGCTGAATGGTTCCAACATGACGGATTTACATGGATTATATGTCGACG
GGTACCATATCTCGAATCTTTCTGGCGGTGAAAACACCCATTGCGATTCTAGAAATGAGATGGTATATACCTACAATCAAGGTGTTTTGCTTAC
CGGACAACGTGGTTTGTATGACGCAACCGCCGCACGATCATACCTTGTAGATGGCCACAAACTCATCGCGAATGTTATTAATGCCACAGGCTAT
GACCTGAAACACAATGTTGTCATCTCACCGCCACCCAAAGATGGTTCCGCATTGGCAAAGTGGTTTGGCCTGGGTAGGAATGGAATACTGGAAG
AAGGATGCGATTCAAGTGCTTCGTGTTCTCAAAATGGACAAACTTTCAAAGGCATATTCTTTCATCACTTGATTGCGTTCTGTAGTGATTTGCC

AGGGGAGCCTATTGCAGGGACGAAGGAAAGCTTAGAACTCGACAGAGTGTGGCATTCTGACAAATGCTCACAGTATACAAAAT

>Bcin06g01710(MLST3), partial sequence [organism=Botrytis cinerea, strain D11_T_B09]
GGTGAGTCTGACTTTTGTATTTGAGCGTTAAGATAGACACTGATATACCAAGGCAATCACCTAATTTCATGGCGTTCTTCCCTGCATCATCTAC
CTACCCTGGAATGCTGGGAGAATTATACTCAGCAGCTTTCACAGCACCTGCTTTCAATTGGATCTGTTCCCCTGCTGTGACAGAATTGGAGACG
GTTGTAATGGATTGGCTGGCCAAGCTTCTCAATCTCCCAGACTGTTATTTGTCTTCGACTCATGGTGGTGGTGTCATCCAAGGATCAGCCTCGG
AAGCTATCGTTACCGTTATGGTTGCTGCCCGCGATAAATATCTTCGTGAAACTACTGAAGGTCTGTCGGGCATTGAACTCGAGGATGCGATTGC
ATATAAGAGGAGTAAGCTAGTTGCACTAGGAAGCGAAATGGCACACAGCTCCACGCAGAAAGCAGCGCAAATAGCTGGCGTTAGATTCCGATCG
ATTCCAGTACTCGCATCCAATGATTTCGCCATGACGGGTGATGATTTAGAGAAGGTATTGAAAGAATGCAAATCTCAAGGATTGGAACCCTTCT
ATCTAACTTCGACTTTGGGAACAACATCTACATGCGCAGTTGACGACTTCGCATCTATTGCAACAGTACTTTCAAAATATGCACCTCCAGATGT
TGCAGGCGAGATCTGGGTTCACGTCGATGCTGCTTATGCAGGTGCAGCTTTGGTTTGCCCTGAATACCATCATCTAACATCGTCTTTCCAGCAT
TTCCATTCCTTCGATATGAACATGCACAAATGGCTTCTGACAAATTTCGACGCTTCTTGTCTATATGTCAAGAAACGCAAAGATCTGATCGATG
CACTGTCCATAACACCAAGTTATCTTCGCAACGAGTTTTCAGAGAGTGGACTCGTAACCGACTATCGGGACTGGCAAATTCCTCTCGGAAGACG
CTTCCGAAGCTTAAAGATTTGGTTTGTCCTCAGAACCTAC

>Bcin09g03030(MLST4), partial sequence [organism=Botrytis cinerea, strain D11_T_B09]
GACCCTTCAACCCCACCGCAGCTCCTATACCGAAAGCGAGTATCCTACCAATTCTCCTTCCACCTGCGACTTTAAGACCATTGGCTTTCCGCAC
TTTCACAAAAAAGCATAGTTTAACATTGACGTCGTCGGCATTACAAGTGTTGGCTACTTTTATTGGAAAGCATTGTGGGACAGGATGGAGGGAA
GAAGGACTGGCAGAGAGAGTCTTGGAGGAGGTCGCCAAGAGTTGGAAGAATAGGAGTGGCGGTGTCATTGTCGAGGGCGAGGGAACGGAATTGA
AGGAGATTCTGAAAGCTTTGGAAGGGAATATGAGTGGTGGAAGGATAGTCATAGGAAGAGAGCTAAGCCGACAGAATAGTTTAGTACTGGGATC
ATCACAATATGGAGAGGTCAATCATACAAGACTTGGGCTACGGCCAGGGAATATACCCAGAGAGGATAGTCAGTCAAGTTTGGGAATGTCAACG
TTGGAGGTCAATGACGAGGAAGATGAGGATGGCCTGATGGATCCAAGAAGGTGGTTAAAAGTCATTGATGCATTTGAGCAACCTCGACTGGTGT
ACAATGTTGCTAAAAAGCACTTTGATAGGTATGTTTCAATGATAAAATTTTATCTGAATCGACTAACTCAGTACAGAGATACCTCCAAACCTTC
ATTGTTCCCACCTGCGTCTCATAAAACACTCCTCTTCCAAAACCGCTATAATGTTATCCATCAACGTCTCCTTCGCAATGAATCTTTTCAAACG
CCCGCTTTTCAAGGTGGCAAATCTTCCCTTCAACGCAGCACGTCCGCCATTACCACACAACAACAATCATACAAATTAACGCCGATAGCTAATC
TTCTCGGTCGCAATCGCAGCTCTCATATGCTTCTCGGTCTCCTCAGTATTTCACCCACTGGTACCCTCGCCATCAATGACCTGACGGGCAGTAT
CGCTCTCGATCTTACACACGCAGCAGCCATTC

>Bcin11g01310(MLST5), partial sequence [organism=Botrytis cinerea, strain D11_T_B09]
ACTGACATGGACCTCATGTGGAACCGGCGTAGAATGCGCAACGCTTGAAGTTCCGCTCGAATATGGCGATGCAACGTCAACGGCAAAAGCCAGT
GTTGCGCTTGCTCGTTATCCTGCCACTGTTGCCGCGAGCAAGAAGCTCGGGTCTCTCTTGATAAACCCCGGTGGACCCGGTGCCTCTGGTGTTG
GCTTTGTGCAGTCTGGAGCCGGTGCCGCCGTCTCGACACTGAGTGGTGGATTGTACGATATCATCGGATGGGATCCACGTGGAACCGGTGCTTC
GGCTCCTATTTTGGAATGTTTTGCAAATGCCAGTGCGGAGTATGATTTTAACAACGCGTTTCCATCTGCTCCGAATCTCTGGCTCGGACAATTT
GCGAATGCCAGCGCAAATTCTGCTGTTAGCTCTGCTATCACATCCTTTGACACTTCTGTCGCTGCTCTTGCAAAAGCTTGCGTGGCTCAGAAAT
CTCCCGCTCTTTACACCTCAACAGCAGCATATGTTGCTCGAGACATGGCAGCGATAGTCGATGCATTGGATGGGACCTCTGCAAAACTTAACTA
CTGGGGTTTCTCATATGGAACTATCTTCCTAGCTGAGTTTATCCAAACTTTCCCAGGCCGCGTGGGAAGAGTTCTTGCCGATGGTGTTTTCGAC
GCAAAGGCAAATGCACTCACATACGTCAGCCAACTTCCCAACGATCAACTCAGTGTTCGTGCTTCGTTGAACGATTTTGCAGCTTTCTGCACCA
CCGCCGGTAGTAAAGGTTGCTCTTTTGCCACCGCCCCTACTGGAACCTCAGGTACTGTTGCTACCAGACTGGACAACATAATGAAGGATATGTT
CCTCAATCCTATTGTTGCTTCGGGCTTAAGCATCAG

>Bcin15g03910(MLST6), partial sequence [organism=Botrytis cinerea, strain D11_T_B09]
GCCAAAACACAAAATCATCCAACGATGAAGATGATACTCCACTTCCCTTGATTATTTGGCATGGACTCGGCGATAATTACAAAGCGGATGGTCT
TGCGCAAGTTGGAAAACTAGCTGAAGCTATTCATCCTGGGACTTTTGTCTACAATATTCATGTAGATGAGGATGCATCTGCAGATAGGACAGCT
ACCTTCTTTGGAAATCTCACTCGTGAGTACATCCCCTATTTTCCCTTTAAATACCATACTAACTCTCTTACCAAGTTCAAATCGAAAAGGTCTG
CGAAGACCTCGCCTCCCATCCTATTCTCTCTACCGCGCCCGCCGTCGACGCAATTGGATTCTCCCAAGGCGGCCAATTCTTGCGCGGTTACATA
TCCCGCTGCAATGCTCCACCCATCCGCTCTCTCCTGACCTTCGGTTCCCAACACAACGGCATTTCTGCCTTCCAAGCCTGTGGTCCTGCCGATT
TCCTCTGTCGCGGTGCTCAAACCCTTTTGCGATCCAACACCTGGTCAACCTTTGTCCAATCTCGTCTCGTACCCGCTCAATACTTCAGAGATCC
GGAAAACCTAGACTCTTACCTTGAATATTCCAATTTCCTTGCCGACATCAATAATGAGCGCGTTCTCAAGAACCAAACATATAAATCCAACATG
GAAAAATTGGAACGATTCGTAATGTATGTCTTTGAAGACGATACAACTGTCATCCCTAAGGAAAGTGGATGGTGGGCTGAAGTCAACGGCACGG
AAGTTACACCACTGAAAGAAAGAGCCATTTATAAAGAAGATTGGCTAGGTTTAAAGACATTGGATGAGGCCGGAAAATTAGTTTTCGAAACCAT
TCCAGGGGGACATATGACGTTAGGAGAGGAGATGCTAGAGAAGGCTTTCAAAGAGTATTTTGGTCCAGCAGGGAAGAAATTTGGGG

>Bcin16g03460(MLST7), partial sequence [organism=Botrytis cinerea, strain D11_T_B09]
ATGAACCTCTTGATTTGAACTCTTCATTAATTGCAAGTGATGAACCCCTATTCTGCCCAGAGGATAGTTACAAGACGTATATCATTAGTCGAGA
ACCACTCATGATATATATTGACGGGTTTTTGAAAGCGAATGAAAGTAAACATTTGGTTGATGTTAGGTGTGTTATTTATTCTGATGAAATGAAC
AAGAGAGACTGATGAGATAGTGAGCCGCTTTATGAACCGTCAACTGTTTCTCACGGACAGGAAGTTACCATTGATCCTTCAGTTCGGAATTCTG
AAGTGGCGGTTTTAGAGAGGGATGAGGTGGTCAGGTGTATTGAGCATAGAGCGAGGGCATTTCAGGGGTGGAGGGGCGAGATGGGGATTGAGAA

GTTGAGGACGCAGAGGTATGGGGTTGGAGGACATTATGGGATGCATTTGTAAGTTTTGGGGGATTGACGAAGGCTTCTGTCTATTTTCTATCAG
CACGGATCTTGAAAGAAAGATAGCATGAACACGCGGGCTAATAACTAGGAAATAGCGATTGGAGCGGAGGTAAACGTGGCATAGACCGATTTAG
TACTTTCATGGTCTATGTCGACGTATCCTCTGATATCGAAGGTGGAGGAACGGAATTCCCACGTATCGTGGACCAAAAGGAGGAAGGTGGGAG
GACTTCCTGGAAACTACGGAAGCATTGGATCCAAGAACTGGAGAAAATGTAACAGTAGAAGGGGTGACATTCAAACCAATCAAGGGAAATGCCG
TATTCTGGGAAAATACTGACAACAACGGGAGGGGCTATGAT

>Bcin12g03020(MLST8), partial sequence [organism=Botrytis cinerea, strain D11_T_B09]
CGATTGGCTGCGAAGAAAACTGCGCAGCACCGAGTCACCAAAAGCACGAATAAACCATCGGCAAGGGAAATATTCTCTGAACCACAACATGACA
CGAGCGCGGAAGAGTATATCGGGCGAGAAGCTTCATCAAGAGCACCAAAGCGACAACGAGTCGATGATAATTATAACTCTTACGGTGGAAGAAA
TGAAAATACAGCAGCTTATGCTTCCGGGAAACTTCCATCTGGAAGTATAAATGTTGGTGGAGGTAGGAAGACCACTTTTCAAGAAGAACCTCGA
ACGGCATTTGTCGCTGGCAAGTTGCCCCCTGGAAGTATCAACATTGGTGGAAAGAAGGCTACCCAAATCGAGGACGAGGGTAGAGCAGCTTATG
CTTCCGGAAAATTGCCCCCAGGAAGTATAAACTCTGGCGCAAAGAAGGCGATTTCATTCCAAGATGAAACGAGACCGGCTTATGTCTCCGGAAA
GCTTCCACATGGTAGTATCGACGGTATGCGAAACCGTGAAATGGCTGCCGTCCACCGCGAAATTGCTGAGGGTGGGAGGAAACCAGGCCAGGTT
GTCTCTTCTCCATTCACATTCAATCCTACTTCAAAGAAAACTTTCGATGAACCAGAAGAACAAGCGGAACCGGCAAAACCATCCAATGCGCCTT
TGACAGAGGAAATGGCGACTTTCACCAATCTAGGGCTATCGAGAAGGCTTGCAGCCCATCTATCGACTAAACTCGATATGAAAGCCCCGACGGC
CATTCAAAAAGCATCTGTGCAGCAGTTGGTATCGGACGATAGCGATGCTTTCATACAAGCAGAGACTGGATCTGGAAAAACTTTGGCATATCTA
CTACCTATAGTCGAGCGAATATTAGCATTGAGTGAGAATGGCGTACAAA

>Bcin02g07770(MLST9), partial sequence [organism=Botrytis cinerea, strain D11_T_B09]
CAGCTTTCCCTTTCGGTCTTGGCATCTACAGTCATTGCCATCCCTACACCATCACAACTTGAGTCTCGGGCCGTTATCGATTCCGATGCCGTTG
TAGGATTTGCCGAAACTGTTCCCAGTGGGACCGTAGGAACAGTTTATGAGGCATATAAACCATTCCTTAAAGTCGTAAATGGATGCGTACCATT
CCCTGCCGTCGATGCATCGGGTAACACAGGGTATGTCCTTATATCTTTCTCTTCCACACGATTGCTATTGAGTCTCTAACATATTTTAGTGGTG
GTTTGTCACCAACTGGCAGTAGCAATGGTGGTTGCAGCAGCAGTACCGGTCAAGTATATGTTCGAGGAGGACAAAGCGGATCAAACTACGCCAT
CATGTACTCCTGGTAAGTTCTCTCTAAACTTCTCCTTATAGATCCAACCTAACAAAATCTTAGGTACATGCCAAAGGACGAGCCCTCAACCGGT
ATTGGTCACCGTCACGATTGGGAAGGTGTAATTGTCTGGCTCTCCAGCGCCACCGCCACAACTGCCGACAACATCTTAGCCGTTTGTCCTTCCG
CCCACGGAGGCTGGGATTGTTCCACGGATGGCTATTCCCTTTCTGGTACCAGCCCTCTTATCAAGTACGAAAGTATCTGGCCCGTCGATCACTC
AATGGGTCTTACTAGTACTGTTGGTGGAAAACAACCTATGATTGCTTGGGAGTCTTTACCAACTGCTGCTCAAACTGCTCTTGAGAACACCGAT
TTCGGTGCTGCGAATGTTCCATTCATTCCGGCTGTTTTCACAGACAATCT

>Bcin04g02090(MLST10), partial sequence [organism=Botrytis cinerea, strain D11_T_B09]
CGGAAGATGATATGGCAAAGTCTATGATTACCAAAGCATTTGTAGGCATGAGTAGTAAACTGTCGAATATGACTATGAATGATGTTTACAAGCC
CTACATCCATGTAAGAAATGTAGAATAGAGAGATCAGTAACTGGAACTAATATCGTTTGTAGGCTTTCAAGTTACTTACGCAGTTCAACCCAAT
CACTACAGCTATTGCCGAATCCCCACTGTTTCAAATGGCCGTCTCAGCAAATACCATCGAAAAGTACACACTGCTAGGCCCTTTCTTCAGAATA
TCTCCTCTGCAACAGGAAGTTACCAGGGAATACTTCAGTGCGCCAAAGACTATAGATAGGCGACACATTGCCACATCTCAAGATGCGTTACGAT
TGACCTTACAAACCCATCAAAAAGATTTACTTGATATCATCAACCACTTTGTTCGAGCAAGTCCAATCGCAAAAAGCAAAACCCTGGATTGGTT
CGCCTACATTGTGAATCAAAATCACAAGCGTCGAGCACTTCAGGTAGACCCGAAAGAAGTGTCTTCTGATGGCTTTATGCACAATGTCACTGTC
GTTCTAGATGGTCTTTGTGAGCCATTCATGGATACCACATTCTCGAAAATTTCGAAGATTGATATTGATTATCTAAGACGTGCGCCTCGTGTAG
ATATCAAGGACGAGACCAAGTTGAACGCTGACGAGAAGGCTTCTGAGAAGTATTATGAGGACACTGTTCCTGGCACTTCTAATTTCATCTCTGA
GGTCTTCTTTCTCACATTGGCTGCTCATCATTATGGTAGTGAAGCTCTTAATGCCACGCATAAGAGTCTGGAGAAAGACATCAAATATATTCAA
AAGCAATTGACTGCCGTTGAAGCA

>Bcin01g07220(MLST1), partial sequence [organism=Botrytis cinerea, strain D11_T_B14]
ATGGCATATCTTGTTTCTTTTCGCATTATGCTTTGGAACGTGCTGGACAAGCTTGGGTTAGTGCTCTGCGTGTAGAAGCACTAAAGAAGATTCT
CGCACAACCGAAGTCATGGTTTGAGGAATCCAGGAATTCACCTAGCCGGTTGAACGAAGTTTTGGATAGGAACTCTGAGGAAATGCGTAATCTC
GTTGGCCGCTTTGCTGGTATTGTATTCACAGCATTTTTTATGCTATTGATATCAATCATTTGGGCTTTCGTGAATACATGGAAACTGACATTAG
TCTCGATGGCAACTGGGCCAGTTATATACGCTGTCACCAAAACGTTCAATCGCGTGAGTGGAAAATGGGAAAACAAGTGCAACTACGCCTCTGA
AATGACCACTGGCATATTTTCAGAGACTTTCTCCAACATCAAAGTGGTTCGGGCTTTTACTCTGGAAACTTACTTTGAGACAAACACACCAAAG
CTACAGAAGAACTTTATAAAGTTGGACTAATACGAGCAAACTACTCGGGATTGCTGTGGGGATTGACAGATGCGATGTCATTCTTCATCACTGC
AACTATCTTTTATTATGCCACGGTTCTCATTACCAAGAGAGAGATCAGTATCGCGACTGCACTACAGACTGTCAATCTTCTATTATTTGGTATT
TCTAATAGTACGAATATGCTGGCCATGATACCACAAATCAACTCTTCTCGCGTTACAGCTACGCATATGCTTGCATTAGCCAGTCTCGATTCAT
CTTCCTCCCACGAAAATAAAGGAACCGAACGGCTTTCGACAATCTTCCAATCAAATTCAACCGTCTTTCATTCACATACCCTACTCGTCCTGAA
AAACGAACGATATCATCCTTTTCTCTTTCCCTGATTCCTAACTCAACAACTGCACTTGTCGGACCCTCCGGCTCCGGAAAATCTACAATAGCTG
CTCTGCTCATTGGTCTCTATCCGCCAGATACTTCAACACCTCCACCGTTGACATTCAATCGCGTCTCCATAAGTAACTGTCACATTCCGTCTCT
CCGGGCTTCTCTCTCACTCGTTCCACAAATACCGATTCTATTTCCAGCTACCATTCTCCATAACATCATTTATGGTCTCCAGAATCTTCTCCTT
GTGCTAGTCTTCCATCTGCTATTGCATCAGCAAAAGATGCTGGGATCCATGAATTTATCACATCGCTTCCACAAGGTTATGATACTAT

>Bcin05g07690(MLST2), partial sequence [organism=Botrytis cinerea, strain D11_T_B14]

ACCACTATCACCAAGTCCTTTTCTGCACTTTCCTCATCAAAAACGAACGAGAACATCATCAATAAATACTTTGCTGAAGTAATTGGGTTTTATT
TTGGCCAGGATGCCTTCTCTCTACGACAGCAAGCCTTCGATGATATGTTGTGGGTAGTTCTTGGCTGGCTGGATACTGTCAAATTCATTGATTT
ACATTCTGAATTGCACTATTCAAATGACTCTCAGCCAGAATGGTACGGACAACAATATAAACCTGCATTTGCACATCGAGCGCGACTATTTTGG
GAATTGGCTTCACAAGGATGGGATACTACTCTCTGTGGTGGTGGGATGATATGGTCACCATATCTTACTCCATACAAGAACGCAATTACCAATG
AACTCTATATCGCAGCTTCGATATCGATGTACCTATATTTCCCCGGAGATGACAATCAATCCCCATTTATGCTTTCCAACCCTTCATATCCACC
TCACGATCCGAAATATCTACAGGCAGCTGTTGATGCTTACAAATGGCTGAATGGTTCCAACATGACGGATTTACAAGGATTATATGTCGACGGG
TACCATATCTCGAATCTTTCTGGCGGTGAAAACACCCATTGCGATTCTAGAAATGAGATGGTATATACCTACAATCAAGGTGTTTTGCTTACTG
GACAACGCGGTTTGTATGACGCAACCGCCGCACGATCATACCTTGTAGATGGCCACAAACTCATCGCGAATGTTATTAATGCCACAGGCTATGA
CCTGAAACACAATGTTGTCATCTCACCTCCACCCAAAGATGGTTCCGCATTGGCAAAGTGGTTTGGCCTGGGTAGGAATGGAATACTGGAAGAA
GGATGCGATTCAAGTGCTTCGTGTTCTCAAAATGGACAAACTTTCAAAGGCATATTCTTTCATCACTTGATTGCGTTCTGTAGTGATTTGCCAG
GGGAGCCTATTGCAGGGACGAAGGAAAGCTTAGAACTCGACAGAGTGTGGCATTCTGACAAATGCTCACAGTATACAAAAT

>Bcin06g01710(MLST3), partial sequence [organism=Botrytis cinerea, strain D11_T_B14]
GTGAGTCTGACTTTTGTATTTGAGCGTTAAGATAGACACTGATATACCAAGGCAATCACCTAATTTCATGGCGTTCTTCCCTGCATCATCTACC
TACCCTGGAATGCTGGGAGAATTATACTCAGCAGCTTTCACAGCACCTGCTTTCAATTGGATCTGTTCCCCTGCTGTGACAGAATTGGAGACGG
TTGTAATGGATTGGCTGGCCAAGCTTCTCAATCTCCCAGACTGTTATTTGTCTTCGACTCATGGTGGTGGTGTCATCCAAGGATCAGCCTCGGA
GGCTATCGTTACCGTTATGGTTGCTGCCCGCGATAAATATCTTCGTGAAACTACTGAAGGTCTGTCGGGCATTGAACTCGAGGATGCGATTGCA
TATAAGAGGAGTAAGCTAGTTGCACTAGGAAGCGAAATGGCACACAGCTCCACGCAGAAAGCAGCGCAAATAGCTGGCGTTAGATTCCGATCGA
TTCCAGTACTCGCATCCAATGATTTCGCCATGACGGGTGATGATTTAGAGAAGGTATTGAAAGAATGCAAATCTCAAGGATTGGAACCCTTCTA
TCTAACTTCGACTTTGGGAACAACATCTACATGCGCAGTTGACGACTTCGCATCTATTGCAACAGTACTTTCAAAATATGCACCTCCAGATGTT
GCAGGCGAGATCTGGGTTCACGTCGATGCTGCTTATGCAGGTGCAGCTTTGGTTTGCCCTGAATACCATCATCTAACATCGTCTTTCCAGCATT
TCCATTCCTTCGATATGAACATGCACAAATGGCTTCTGACAAATTTCGACGCTTCTTGTCTATATGTCAAGAAACGCAAAGATCTGATCGATGC
ACTGTCCATAACACCAAGTTATCTTCGCAACGAGTTTTCAGAGAGTGGACTCGTAACCGACTATCGGGACTGGCAAATTCCTCTCGGAAGACGC
TTCCGAAGCTTAAAGATTTGGTTTGTCCTCAGAACCTAC

>Bcin09g03030(MLST4), partial sequence [organism=Botrytis cinerea, strain D11_T_B14]
ACCCTTCAACCCCACCGCAGCTCCTATACCGAAAGCGAGTATCCTACCAATTCTCCTTCCACCTGCGACTTTAAGACCATTGGCTTTCCGCACT
TTCACAAAAAAGCATAGTTTAACATTGACGTCGTCGGCATTACAAGTGTTGGCTACTTTTATTGGAAAGCATTGTGGGACAGGATGGAGGGAAG
AAGGACTGGCAGAGAGAGTCTTGGAGGAGGTCGCCAAGAGTTGGAAGAATAGGAGTGGCGGTGTCATTGTCGAGGGCGAGGGAACGGAATTGAA
GGAGATTCTGAAAGCTTTGGAAGGGAATATGAGTGGTGGAAGGATAGTCATAGGAAGAGAGCTAAGCCGGCAGAATAGTTTAGTACTGGGATCA
TCACAATATGGAGAGGTCAATCATACAAGACTTGGGCTACGGCCAGGGAATATACCCAGAGAGGATAGTCAGTCAAGTTTGGGAATGTCAACGT
TGGAGGTCAATGACGAGGAAGATGAGGATGGCCTGATGGATCCAAGAAGGTGGTAAAAGTCATTGATGCATTTGAGCAACCTCGACTGGTGTAC
AATGTTGCTAAAAAGCACTTTGATAGGTATGTTTCAATGATAAAATTTTATCTGAATCGACTAACTCAGTACAGAGATACCTCCAAACCTTCAT
TGTTCCCACCTGCGTCTCATAAACACTCCTCTTCAAAACCGCTATAATGTTATCCATCAACGTCTCCTTCGCAATGAATCTTTTCAAACGCCCG
CTTTTCAAGGTGGCAAATCTTCCCTTCAACGCAGCACGTCCGCCATTACCACCCAACAACAATCATACAAATTAACGCCGATAGCTAATCTTCT
CGGTCGCAATCGCAGCTCTCATATGCTTCTCGGTCTCCTCAGTATTTCACCCACTGGTACCCTCGCCATCAATGACCTGACGGGCAGTATCGCT
CTCGATCTTACACACGCAGCAGCCATTC

>Bcin11g01310(MLST5), partial sequence [organism=Botrytis cinerea, strain D11_T_B14]
ACTGACATGGACCTCATGTGGAACCGGCGTAGAATGCGCAACGCTTGAAGTTCCGCTCGAATATGGCGATGCAACGTCAACGGCAAAAGCCAGT
GTTGCGCTTGCTCGTTATCCTGCCACTGTTGCCGCGAGCAAGAAGCTCGGGTCTCTCTTGATAAACCCCGGTGGACCCGGTGCCTCTGGTGTTG
GCTTTGTGCAGTCTGGAGCCGGTGCCGCCGTCTCGACACTGAGTGGTGGATTGTACGATATCATCGGATGGGATCCACGTGGAACCGGTGCTTC
GGCTCCTATTTTGGAATGTTTTGCAAATGCCAGTGCGGAGTATGATTTTAACAACGCGTTTCCATCTGCTCCGAATCTCTGGCTCGGACAATTT
GCGAATGCCAGCGCAAATTCTGCTGTTAGCTCTGCTATCACATCCTTTGACACTTCTGTCGCTGCTCTTGCAAAAGCTTGCGTGGCTCAGAAAT
CTCCCGCTCTTTACACCTCAACAGCAGCATATGTTGCTCGAGACATGGCAGCGATAGTCGATGCATTGGATGGGACCTCTGCAAAACTTAACTA
CTGGGGTTTCTCATATGGAACTATCTTCCTAGCTGAGTTTATCCAAACTTTCCCAGGCCGCGTGGGAAGAGTTCTTGCCGATGGTGTTTTCGAC
GCAAAGGCAAATGCACTCACATACGTTAGCCAACTTCCCAACGATCAACTCAGTGTTCGTGCTTCGTTGAACGATTTTGCAGCTTTCTGCACCA
CCGCCGGTAGTAAAGGTTGCTCTTTTGCCACCGCCCCTACTGGAACCACAGGTACTGTTGCTACCAGACTGGACAACATAATGAAGGATATGTT
CCTCAATCCTATTGTTGCTTCGGGCTTAAGCATCA

>Bcin15g03910(MLST6), partial sequence [organism=Botrytis cinerea, strain D11_T_B14]
GCCAAAACACAAAATCATCCAACGATGAAGATGATACTCCACTTCCCTTGATTATTTGGCATGGACTCGGCGATAATTACAAAGCGGATGGTCT
TGCGCAAGTTGGAAAACTAGCTGAAGCTATTCATCCTGGGACTTTTGTCTACAATATTCATGTAGATGAGGATGCATCTGCAGATAGGACAGCT
ACCTTCTTTGGAAATCTCACTCGTGAGTACATCCCCTATTTTCCCTTTAAATACCATACTAACTCTCTTACCAAGTTCAAATCGAAAAGGTCTG
CGAAGACCTCGCCTCCCATCCTATTCTCTCTACCGCGCCCGCCGTCGACGCAATTGGATTCTCCCAAGGCGGCCAATTCTTGCGCGGTTACATA
TCCCGCTGCAATGCTCCACCCATCCGCTCTCTCCTGACCTTCGGTTCCCAACACAACGGCATTTCTGCCTTCCAAGCCTGTGGTCCTGCCGATT
TCCTCTGTCGCGGTGCTCAAACCCTTTTGCGATCCAACACCTGGTCAACCTTTGTCCAATCTCGTCTCGTACCCGCTCAATACTTCAGAGATCC

GGAAAACCTAGACTCTTACCTTGAATATTCCAATTTCCTTGCCGACATCAATAATGAGCGCGTTCTCAAGAACCAAACATATAAATCCAACATG
GAAAAATTGGAACGATTCGTAATGTATGTCTTTGAAGACGATACAACTGTCATCCCTAAGGAAAGTGGATGGTGGGCTGAAGTCAACGGCACGG
AAGTTACACCACTGAAAGAAAGAGCCATTTATAAAGAAGATTGGCTAGGTTTAAAGACATTGGATGAGGCCGGAAAATTAGTTTTCGAAACCAT
TCCAGGGGGACATATGACGTTAGGAGAGGAGATGCTAGAGAAGGCTTTCAAAGAGTATTTTGGTCCAGCAGGGAAGAAATTTGGG

>Bcin16g03460(MLST7), partial sequence [organism=Botrytis cinerea, strain D11_T_B14]
ATGAACCTCTTAATTTGAACTCTTCATTATTTGCAAGTGATGAACCCCTATTCTGCCCAGAGGATAGTTACAAGACGTATATCATTAGTCGAGA
ACCACTCATGATATATATTGACGGATTTTTGAAAGCGAATGAAAGTAAACATTTGGTTGATGTTAGGTGTGTTATTTATTCTGATGAAATGAAC
AAGAGAGACTGATGAGATAGTGAGCCGCTTTATGAACCGTCAACTGTTTCTCACGGACAGGAAGTTACCATTGATCCTTCAGTTCGGAATTCTG
AAGTGGCGGTTTTAGAGAGGGATGAGGTGGTCAGGTGTATTGAGCATAGAGCGAGGGCATTTCAGGGGTGGAGGGGCGAGATGGGGATTGAGAA
GTTGAGGACGCAGAGGTATGGGGTTGGAGGACATTATGGGATGCATTTGTAAGTTTTGGGGGATTGACGAAGGCTTTTGTCTATTTTCTACCAG
TACGGATCTTGAAAGAAAGATAGCATGAACACGAGGGCTAATAACTAGGAAATAGCGATTGGAGCGGAGGTAAACGTGGCATAGACCGATTTAG
TACTTTCATGGTCTATGTCGACGTATCCTCTGATATCGAAGGTGGAGGAACGGAATTCCCACGTATCGTGGGACCAAAAGGAGGAAGGTGGGAG
GACTTCCTGGAAACTACGGAAGCATTGGATCCAAGAACTGGAGAAAATGTAACAGTAGAAGGGGTGACATTCAAACCAATCAAGGGAAATGCCG
TATTCTGGGAAAATACTGACAGCAACGGGAGGGGCTATGATG

>Bcin12g03020(MLST8), partial sequence [organism=Botrytis cinerea, strain D11_T_B14]
CGATTGGCTGCGAAGAAAACTGCGCAGCACCGAGTCACCAAAAGCACGAATAAACCATCGGCAAGGGAAATATTCTCTGAACCACAACATGACA
CGAGCGCGGAAGAGTATATCGGGCGAGAAGCTTCATCAAGAGCACCAAAGCGACAACGAGTCGATGATAATTATAACTCTTACGGTGGAAGAAA
TGAAAATACAGCAGCTTATACTTCCGGGAAACTTCCATCTGGAAGTATAAATGTTGGTGGAGGTAGGAAGACCACTTTTCAAGAAGAACCACGA
ACGGCATTTGTCGCTGGCAAGTTGCCCCCTGGAAGTATCAACATTGGTGGAAAGAAGGCTACCCAAATCGAGGACGAGGGTAGAGCAGCTTATG
CTTCCGGAAAATTGCCTCCAGGAAGTATAAACTCTGGCGCAAAGAAGGCGATTTCATTCCAAGATGAAACGAGACCGGCTTATGTCTCCGGAAA
GCTTCCACATGGTAGTATCGACGGTATGCGAAACCGTGAAATGGCTGCCGTCCACCGCGAAATTGCTGAGGGTGGGAGGAAACCAGGCCAGGTT
GTCTCTTCTCTATTCACATTCAATCCTACTTCAAAGAAAACTTTTGATGAACCCGAAGAACAAGCGGAACCGGCAAAACCATCCAATGCGCCTT
TGACAGAGGAAATGGCGACTTTCACCAATCTAGGGTTATCGAGAAGGCTTGCAGCCCATCTATCGACTAAACTCGATATGAAAGCTCCGACCGC
CATTCAAAAAGCATCTGTACAGCAGTTGGTATCGGACGATAGCGATGCTTTCATACAAGCAGAGACTGGATCTGGAAAAACTTTGGCATATCTA
CTACCTATAGTCGAGCGAATATTAGCATTGAGTGAGAATGGCGTACAAA

>Bcin02g07770(MLST9), partial sequence [organism=Botrytis cinerea, strain D11_T_B14]
CAGCTTTCCCTTTCGGTCTTGGCATCTACAGTCATTGCCATCCCTACACCATCACAACTTGAGTCTCGGGCCGTTATCGATTCCGATGCCGTTG
TAGGATTTGCCGAAACTGTTCCCAGTGGGACCGTAGGAACAGTTTATGAGGCATATAAACCATTCCTTAAAGTCGTAAATGGATGCGTACCATT
CCCTGCCGTCGATGCATCGGGTAACACAGGGTATGTCCTTATATCTTTCTCTTCCACACGATTGCTATTGAGTCTCTAACATATTTAGTGGTG
GTTTGTCACCAACTGGCAGTAGCAATGGTGGTTGCAGCAGCAGTACCGGTCAAGTATATGTTCGAGGAGGACAAAGCGGATCAAACTACGCCAT
CATGTACTCCTGGTAAGTTCTCTCTAAACTTCTCCTTATAGATCCAACCTAACAAAATCTTAGGTACATGCCAAAGGACGAGCCCTCAACCGGT
ATTGGTCACCGTCACGATTGGGAAGGTGTAATTGTCTGGCTCTCCAGCGCCACCGCCACAACTGCCGACAACATCTTAGCCGTTTGTCCTTCCG
CCCACGGAGGCTGGGATTGTTCCACGGATGGCTATTCCCTTTCTGGTACCAGCCCTCTTATCAAGTACGAAAGTATCTGGCCCGTCGATCACTC
AATGGGTCTTACTAGTACTGTTGGTGGAAAACAACCTATGATTGCTTGGGAGTCTTTACCAACTGCTGCTCAAACTGCTCTTGAGAACACCGAT
TTCGGTGCTGCGAATGTTCCATTCATTCCGGCTGTTTTCACAGACAATCT

>Bcin04g02090(MLST10), partial sequence [organism=Botrytis cinerea, strain D11_T_B14]
CGGAGGATGATATGGCAAAGTCTATGATTACCAAAGCATTTGTAGGCATGAGTAGTAAACTGTCGAATATGACTATGAATGATGTTTACAAGCC
CTACATCCATGTAAGAAATGTAGAATAGAGAGATCAGTAACTGGAACTAATATCGTTTGTAGGCTTTCAAGTTACTTACGCAGTTCAACCCAAT
CACTACAGCTATTGCCGAATCCCCACTGTTTCAAATGGCCGTCTCAGCAAATACCATCGAAAAGTACACACTGCTAGGCCCTTTCTTCAGAATA
TCTCCTCTGCAACAGGAAGTTACCAGGGAATACTTCAGTGCGCCAAAGACGATAGATAGGCGACACATTGCCACATCTCAAGATGCGTTACGAT
TGACCTTACAAACCCATCAAAAAGATTTACTTGATATCATCAACCACTTTGTTCGAGCAAGTCCAATCGCAAAAAGCAAAACCCTGGATTGGTT
CGCCTACATTGTGAATCAAAATCACAAGCGTCGAGCACTTCAGGTAGACCCGAAAGAAGTGTCTTCTGATGGCTTTATGCACAATGTCACTGTC
GTTCTAGATGGTCTTTGTGAGCCATTCATGGATACCACATTCTCGAAAATTTCGAAGATTGATATTGATTATCTAAGACGTGCGCCTCGTGTAG
ATATCAAGGACGAGACCAAGTTGAACGCTGACGAGAAGGCTTCTGAGAAGTATTATGAGGACACTGTTCCTGGCACTTCTAATTTCATCTCTGA
GGTCTTCTTTCTCACATTGGCTGCTCATCATTATGGTAGTGAAGCTCTTAATGCCACGCATAAGAGTCTGGAGAAAGACATCAAATATATTCAA
AAGCAATTGACTGCCGTTGAAGCAG

>Bcin01g07220(MLST1), partial sequence [organism=Botrytis cinerea, strain D11_T_B25]
ATGGCATATCTTGTTTCTTTTCGCATCATGCTTTGGAACGTGCTGGGCAAGCTTGGGTTAGTGCTCTGCGTGTAGAAGCACTAAAGAAGATTCT
CGCACAACCGAAGTCATGGTTTGAGGAATCCAGGAATTCACCTAGCCGGTTGAACGAAGTTTTGGATAGAACTTGAGGAAATGCGTAATCTCGT
TGGCCGCTTTGCTGGTATTGTATTCACAGCATTTTTATGCTATTGATATCAATCATTTGGCTTTCGTGAATACATGGAAACTGACATTAGTCTC
AATGGCAACTGGCCAGTTATATACGCTGTCACCAAACGTTCAATCGCGTGAGTGGAAATGGAAAACAAGTGCAACTACGCATCTGAAATGACCA
TGGCATATTTCAGAGACTTTCTCCAACATCAAAGTGTTCGGGCTTTTACTCTGGAAACTTACTTTGAGACAAAACACACCAAAGCTACAGAAGA

ACTCTATAAAGTTGGCTAATACGAGCAAACTACTCGGATTGCTGTGGGATTGACAGATGCGATGTCATCTTCATCACTGCACTATCTTATATTA
TGCCACGTTCTCATTACCAAGAGAGATCAGTATTGCGACTGCACTACAGACTGTCAATCTTCTATTATTTGTATTTCTAATAGTACGAATATGC
TGGCTATGATACCACAAATCAACTCTTCTCGCGTACAGCTACGCATATGCTTGCATTAGCCAATCTCGATTCATCTTCCTCCACGAAAATAAAG
GAACCGAACGGCTTCGACAATCTTTCCAATCAAATTCAACCGTCTTTCATCACATACCCTACTCGTCTGAAAACGAACGATATCATCCTTTTCT
CTTTCCTGATTCCTAACTCAACAACTGCACTTGTCGGACCTCGGCTCCGGAAATCTACGATAGCTGCTCTGCTCATTGTCTCTATCCGCAGATA
CTTCAACACCTCCACCGTTGACATTCAATCGCGTCTCCATAAGTAACTGTCACATTCCGTCTCTCCGGCTTCTCTCTCACTCGTCCACAAATAC
CGATTCTATTTCCAGCTACCATTCTCCATAACATCATTTATGGTCTCCAGAATCTTCTCCTTGTGCTAGTCTTCATCTGCTATTGCATCGCAAA
AGATGCTGGGATCATGAATTATCACATCGCTTCCACAAGGTTATGATACTAT

>Bcin05g07690(MLST2), partial sequence [organism=Botrytis cinerea, strain D11_T_B25]
CACCACTATCACCAAGTCCTTTTCTGCACTTTCCTCATCAAAAACGAACGAGAACATCATCAATAAATACTTTGCTGAAGTAATTGGGTTTTAT
TTTGGCCAGGATGCCTTCTCTCTACGACAGCAAGCCTTCGATGATATGTTGTGGGTAGTTCTTGGCTGGCTGGATACTGTCAAATTCATTGATT
TACATTCTGAATTGCACTATTCAAACGACTCTCAGCCAGAATGGTACGGACAACAATATAAGCCTGCATTTGCACATCGAGCGCGACTATTTTG
GGAATTGGCTTCACAAGGATGGGATACTACTCTCTGTGGTGGTGGGATGATATGGTCACCATACCTTACTCCATACAAGAATGCAATTACCAAT
GAACTCTATATCGCAGCTTCGATATCGATGTACCTCTATTTCCCCGGAGATGACAATCAATCCCCATTTATGCTTTCCAACCCTTCATATCCAC
CTCACGATCCGAAATATCTACAGGCAGCTGTTGATGCTTACAAATGGCTGAATGGTTCCAACATGACGGATTTACAAGGATTATATGTCGACGG
GTATCATATCTCGAATCTTTCTGGCGGTGAAAACACCCATTGCGATTCTAGAAATGAGATGGTATATACCTACAATCAAGGTGTTTTGCTTACT
GGACAACGTGGTTTGTATGACGCAACCGCCGCACGATCATACCTTGTGGATGGCCACAAACTCATCGCGAATGTTATTAATGCCACAGGCTATG
ACCTGAAACACAATGTTGTCATCTCACCGCCACCCAAAGATGGTTCCGCATTGGCAAAGTGGTTTGGCCTGGGTAGGAATGGAATACTGGAAGA
AGGATGCGATTCAAGTGCTTCGTGTTCTCAAAATGGACAAACTTTCAAAGGCATATTCTTTCATCACTTGATTGCATTCTGTAGTGATTTGCCA
GGGGAGCCTATTGCAGGGACGAAAGAAGGCCTAGAACTCGACAGAGTGTGGCATTCTGACAAATGCTCACAGTATACAAAAT

>Bcin06g01710(MLST3), partial sequence [organism=Botrytis cinerea, strain D11_T_B25]
GTGAGTCTGACTTTTGTATTTGAGCGTTAAGATGGACACTGATGTACCAAGGCAATCACCTAATTTCATGGCGTTCTTCCCTGCATCATCTACC
TACCCTGGAATGCTGGGAGAATTATACTCAGCAGCTTTCACAGCTCCTGCTTTCAATTGGATCTGTTCCCCTGCTGTGACAGAATTGGAGACGG
TTGTAATGGATTGGTTGGCCAAGCTTCTCAATCTCCCAGACTGTTATTTGTCGTCGACTCATGGTGGTGGTGTTATCCAAGGATCAGCCTCGGA
AGCTATCGTTACCGTTATGGTTGCTGCCCGCGATAAGTATCTTCGTGAAACCACTGAAGGTCTGTCGGGAATTGAACTCGAGGATGCGATTGCA
TATAAGAGGAGTAAGCTAGTTGCACTAGGAAGCGAAATGGCACACAGCTCCACGCAGAAAGCAGCGCAGATAGCTGGCGTTAGATTCCGATCGA
TTCCAGTACTCGCATCCAATGATTTCGCCATGACGGGTGATGATTTAGAGAAGGTATTGAAAGAATGCAAATCTCAAGGATTGGAACCCTTCTA
TCTAACTTCGACGTTGGGAACAACATCTACATGCGCAGTTGACGACTTCGCATCTATTGCAACAGTACTTTCAAAATATGCACCTCCAGATGTT
GCAGGCGAGATCTGGGTTCACGTCGATGCTGCTTATGCAGGTGCAGCTTTGGTTTGCCCTGAATACCATCATCTAACATCGTCCTTCCAGCATT
TCCATTCCTTTGATATGAACATGCACAAATGGCTTCTGACAAATTTCGACGCTTCTTGCCTATATGTCAAGAAACGCAAAGATCTGATCGATGC
ACTCTCCATAACACCAAGTTATCTTCGCAACGAGTTTTCAGAGAGTAGACTCGTAACCGACTATCGGGACTGGCAAATTCCTCTCGGAAGACGT
TTCCGAAGCTTAAAGATTTGGTTTGTCCTCAGAACCTAC

>Bcin09g03030(MLST4), partial sequence [organism=Botrytis cinerea, strain D11_T_B25]
GACCCTTCAACCCCACCGCAGCTCCTATACCGAAAGCGAGTATCCTACCAATTCTCCTTCCACCTGCGACTTTAAGACCATTGGCTTTTCGCAC
TTTCACAAAAAAGCATAGTTTAACATTGACGTCGTCGGCATTACAAGTGTTGGCTACTTTTATTGGAAAGCATTGTGGGACAGGATGGAGGGAG
GAAGGACTGGCAGAGAGAGTCTTGGAGGAGGTCGCCAAGAGTTGGAAGAATAGGAGTGGCGGTGTCATTGTCGAGGGCGAGGGAACGGAATTGA
AGGAGATTCTGAAAGCTTTGGAAGGGAATATGAGTGGTGGAAGGATAGTCATAGGAAGAGAGCTAAGCCGGCAGAATAGTTTAGTACTGGGATC
ATCACAATATGGAGAGGTCAATCATACAAGACTTGGGCTACGGCCAGGGAATATACCCAGAGAGGATAGTCAGTCAAGTTTGGGAATGTCAACG
TTGGAGGTCAATGACGAGGAAGATGAGGATGGCCTGATGGATCCAAGAAGGTGGTTAAAAGTCGTTGATGCATTTGAGCAACCTCGACTGGTGT
ACAATGTTGCTAAAAAGCACTTTGATAGGTATGTTTCAATGATAAAATTTTATCTGAATCGACTAACTCAGTACAGAGATACCTCCAAACCTTC
ATTGTTCCCACCTGCGTCTCATAAAACACTCCTCTTCCAAAACCGCTATAATGTTATCCATCAACGTCTCCTTCGCAATGAATCTTTTCAAACG
CCCGCTTTTCAAGGTGGCAAATCTTCCCTTCAACGCAGCACGTCCGCCATTACCACCCAACAACAATCATACAAATTAACGCCGATAGCTAATC
TTCTCGGTCGCAATCGCAGCTCTCATATGCTTCTCGGTCTCCTCAGTATTTCACCCACTGGTACCCTCGCCATCAATGACCTGACGGGCAGTAT
CGCTCTTGATCTTACACACGCAGCAGCCATTC

>Bcin11g01310(MLST5), partial sequence [organism=Botrytis cinerea, strain D11_T_B25]
ACTGACATGGACCTCATGTGGAACCGGCGTAGAATGCGCAACGCTTGAAGTTCCGCTCGAATATGGCGATGCAACGTCAACGGCAAAAGCCAGT
GTTGCGCTTGCTCGTTATCCTGCCACTGTTGCCGCGAGCAAGAAGCTCGGGTCTCTCTTGATAAATCCCGGTGGACCCGGTGCCTCTGGTGTTG
GCTTTGTGCAGTCTGGAGCCGGTGCCGCCGTCTCGACACTGAGTGGTGGATTATACGATATCATCGGATGGGATCCACGTGGAACCGGTGCTTC
GGCTCCTATTTTGGAATGTTTTGCAAATGCCAGTGCGGAGTATGATTTTAACAACGCGTTTCCATCTGCTCCGAATCTCTGGCTCGGACAATTT
GCGAATGCCAGCGCAAATTCTGCTGTTAGCTCTGCTATCACATCCTTTGACACTTCTGTCGCTGCTCTTGCAAAAGCTTGCGTGGCTCAGAAAT
CTCCCGCTCTTTACACCTCAACAGCAGCATATGTTGCTCGAGACATGGCAGCGATAGTCGATGCATTGGATGGGACCTCTGCAAAACTTAACTA
CTGGGGTTTCTCATATGGAACTATCTTCCTAGCTGAGTTTATCCAAACTTTCCCAGGCCGCGTGGGAAGAGTTCTTGCCGATGGTGTTTTCGAC
GCAAAGGCAAATGCACTCACATACGTTAGCCAACTTCCCAACGATCAACTCAGTGTTCGTGCTTCGTTGAACGATTTTGCAGCTTTCTGCACCA

CCGCCGGTAGTAAAGGTTGCTCTTTTGCCACCGCCCCTACTGGAACCTCAGGTACTGTTGCTACCAGACTGGACAACATAATGAAGGATATGTT
CCTCAATCCTATTGTTGCTTCGGGCTTGAGCATCA

>Bcin15g03910(MLST6), partial sequence [organism=Botrytis cinerea, strain D11_T_B25]
GCCAAAACACAAAATCATCCAACGATGAAGATGATACTCCACTTCCCTTGATTATCTGGCATGGACTCGGCGATAATTACAAAGCGGATGGTCT
TGCGCAAGTTGGAAAACTAGCTGAAGCTATTCATCCTGGGACTTTTGTCTATAATATTCATGTAGATGAGGATGCATCTGCAGATAGGACAGCT
ACCTTCTTTGGAAATCTCACTCGTGAGTACATCCCCTATTTTTCCTTTAAATACCATACTAACTCTCTTACCAAGTTCAAATCGAAAAGGTCTG
CGAAGACCTCGCCTCCCATCCTATTCTCTCTACCGCGCCCGCCGTCGACGCAATTGGATTCTCCCAAGGCGGCCAATTCTTGCGCGGTTACATA
TCCCGCTGCAATGCTCCACCCATCCGCTCTCTCCTGACCTTCGGTTCCCAACACAACGGCATTTCTGCCTTCCAAGCCTGTGGTCCTGCCGATT
TCCTCTGTCGCGGTGCTCAAACCCTTTTGCGATCCAACACCTGGTCAACCTTTGTCCAATCTCGTCTCGTACCTGCTCAATACTTCAGAGATCC
GGAAAACCTAGACTCTTACCTTGAATATTCCAATTTCCTTGCCGACATCAATAATGAGCGCGTTCTCAAGAACCAAACATATAAATCCAACATG
GAAAATTGGAACGATTCGTAATGTATGTCTTTGAAGACGATACAACTGTCGTCCCTAAGGAAAGTGGATGGTGGGCTGAAGTCAATGGCACGGA
AGTTACACCACTGAAAGAAAGAGCCATTTATAAAGAAGATTGGCTAGGGTTAAAGACATTGGATGAGGCCGGAAAATTAGTTTTCGAAACCATT
CCAGGGGGACATATGACGTTAGGAGAGGAGATGCTAGAGAAGGCTTTCAAAGAGTACTTTGGTCCAGCAGGGAAAAAATTTGGGG

>Bcin16g03460(MLST7), partial sequence [organism=Botrytis cinerea, strain D11_T_B25]
CATGAACCTCTTAATTTGAACTCTTCATTAATTGCAAGTGATGAACCCCTATTCTGCCCAGAGGATAGTTACAAGACGTATATCATTAGTCGAG
AACCACTCATGATATACATTGACGGATTTTTGAAAGCGAATGAAAGTAAACACTTGGTTGATGTTAGGTGTGTTATTTATTCTGATGAAATGAA
CAAGAGAGACTGATGAGATAGTGAACCGCTTTATGAACCGTCTACTGTTTCTCACGGACAGGAAGTTACCATTGATCCTTCGGTTCGAAATTCT
GAAGTGGCGATTTTAGAGAGGGATGAGGTGGTCAGGTGTATTGAGCATAGAGCGAGGGCATTTCAGGGGTGGAGGGGCGAGATGGGGATTGAGA
AGTTGAGGACGCAGAGGTATGGGGTTGGAGGACATTATGGGATGCATTTGTAAGTTTTGGGGGATTGACGAAGGCTTCTGTCTATTTTCTATCA
GTGCGGATCTTGAAAGAAAGATAGCATGAACACGAGGGCTAATAACTAGGAAATAGCGATTGGAGCGGAGGTAAACGTGGCATAGACCGATTTA
GTACTTTCATGGTCTATGTCGACGTATCCTCTGATATCGAAGGTGGAGGAACGGAATTCCCACGTATTGTGGGACCAAAAGGAGGAAGGTGGGA
GGACTTCCTGGAAACTACGGAAGCATTGGATCCAAGAACTGGAGAAAATGTAACAGTAGAAGGGGTGACATTCAAACCAATCAAGGGAAATGCC
GTATTCTGGGAAAATACTGACAACAACGGGAGGGGCTATGAT

>Bcin12g03020(MLST8), partial sequence [organism=Botrytis cinerea, strain D11_T_B25]
CGATTGGCTGCGAAGAAAACTGCGCAGCACCGAGTCACCAAAAGCACGAATAAACCATCGGCAAGGGAAATATTCTCTGAACCACAACATGACA
CGAGCGCGGAAGAGTATATCGGGTGAGAAGCTTCATCAAGAGCACCAAAGCGACAACGAGTCGATGATAATTATAACTCTTACGGTGGAAGAAA
TGAGAATACAGCAGCTTATGCTTCCGGGAAACTTCCATCTGGAAGTATAAATGTTGGTGGAGGTAGGAAGACCACTTTCAAGAAGAACCTCGAA
CGGCATTTGTCGCTGGCAAGTTGCCCCCTGGAAGTATCAACATTGGTGGAAAGAAGGCTACCCAAATCGAGGACGAGGGTAGAGCAGCTTATGC
TTCCGGAAAATTGCCCCAGGAAGTATAAACTCTGGCGCAAAGAAGGCGATTTCGTTCCAAGATGAAACGAGACCGGCTTATGTCTCTGGAAAGC
TTCCACATGGTAGTATCGACGGTATGCGAAACCGTGAAATGGCTGCCGTCCACCGCGAAATTGCTGAGGGTGGAGGAAAACCAGGCCAGGTTG
TCTCTTCTCTATTCACATTCAATCCTACTTCAAAGAAAACTTTCGATGAACCCGAAGAACAAGCGGAACCGGCAAAACCATCCAATGCACCTTT
GACAGAGGAAATGGCGACTTTCACCAATCTAGGGTTATCGAGAAGGCTTGCAGCCCATCTATCGACTAAACTCGATATGAAAGCCCCGACCGCC
ATTCAAAAAGCATCTGTGCAGCAGTTGGTATCGGACGATAGCGATGCTTTCATACAAGCAGAGACTGGATCTGGAAAAACTTTGGCATATCTAC
TACCTACAGTCGAGCGAATATTAGCATTGAGTGAGAATGGCGTACAAA

>Bcin02g07770(MLST9), partial sequence [organism=Botrytis cinerea, strain D11_T_B25]
CAGCTTTCCCTTTCGGTCTTGGCATCTACAGTCATTGCCATCCCTACACCATCACAACTTGAGTCTCGGGCCGTTATCGATTCCGATGCCGTTG
TAGGATTTGCCGAAACTGTTCCCAGTGGGACCGTAGGAACAGTTTACGAGGCATATAAACCATTCCTTAAAGTCGTAAATGGATGCGTACCATT
CCCTGCCGTCGATGCATCGGGTAACACAGGGTATGTCCTTATACCATTCTCTTCCACACGATTGCTATTGAGTCTCTAACATATTTTAGTGGTG
GTTTGTCACCAACTGGCAGTAGCAATGGTGGTTGCAGCAGCAGTACCGGTCAAGTATATGTTCGAGGAGGACAAAGCGGATCAAACTACGCCAT
CATGTACTCCTGGTAAGTTCTCTCTAAACTTCTCCTTATAGATCCAACCTAACAAAATCTTAGGTACATGCCAAAGGACGAGCCCTCAACCGGT
ATTGGTCACCGTCACGATTGGGAAGGTGTAATTGTCTGGCTCTCAAGCGCCACCGCCACAACTGCCGACAACATCTTAGCCGTTTGTCCTTCCG
CCCACGGAGGCTGGGATTGTTCCACCGATGGCTATTCCCTTTCTGGTACCAGCCCTCTTATCAAGTACGAAAGTATCTGGCCCGTCGATCATTC
AATGGGTCTTACTAGTACTGTTGGTGGAAAACAACCTATGATTGCTTGGGAGTCTTTACCAACTGCTGCTCAAACTGCTCTTGAGAACACCGAT
TTCGGTGCTGCGAATGTTCCATTCATTCCGGCTGTTTTCACAGATAATCT

>Bcin04g02090(MLST10), partial sequence [organism=Botrytis cinerea, strain D11_T_B25]
GCGGAGGATGATATGGCAAAGTCTATGATTACCAAAGCATTTGTAGGCATGAGTAGTAAACTGTCGAATATGACTATGAATGATGTTTACAAGC
CCTACATCCATGTAAGAAATGTAGAATAGAGAGATCAGTAACTGGAACTAATATCGTTTGTAGGCTTTCAAGTTACTTACGCAGTTCAACCCAA
TCACTACAGCTATTGCCGAATCCCCACTATTTCAAATGGCTGTCTCAGCAAATACCATCGAAAAGTACACACTGCTAGGCCCTTTCTTCAGAAT
ATCTCCTCTGCAACAGGAAGTTACCAGGGAATACTTCAGTGCGCCAAAGACGATAGATAGACGACACATTGCCACATCTCAAGATGCGTTACGA
TTAACCTTACAAACCCATCAAAAAGATTTACTTGATATCATCAACCACTTTGTTCGAGCAAGTCCAATCGCAAAAAGCAAAACCCTGGATTGGT
TCGCCTACATTGTGAATCAAAATCACAAACGTCGAGCACTTCAGGTAGACCCGAAAGAAGTGTCTTCTGATGGCTTTATGCACAATGTCACTGT
CGTTCTAGATGGTCTTTGTGAGCCATTCATGGATACCACATTCTCGAAAATTTCGAAGATTGATATTGATTATCTAAGACGTGCGCCTCGTGTA

GATATCAAGGACGAGACCAAGTTGAACGCTGATGAGAAGGCTTCTGAGAAGTATTATGAGGACACTGTTCCTGGCACTTCTAATTTCATCTCTG
AGGTCTTCTTTCTGACATTGGCTGCTCATCATTATGGTAGTGAAGCTCTTAATGCCACGCATAAGAGTCTGGAGAAAGACATCAAATATATTCA
AAAGCAATTGACTGCCGTTGAAGCAGAAC

>Bcin01g07220(MLST1), partial sequence [organism=Botrytis cinerea, strain D11_T_B45]
ATGGCATATCTTGTTTCTTTTCGCATTATGCTTTGGAACGTGCTGGACAAGCTTGGGTTAGTGCTCTGCGTGTAGAAGCACTAAAGAAGATTCT
CGCACAACCGAAGTCATGGTTTGAGGAATCCAGGAATTCACCTAGCCGGTTGAACGAAGTTTTGGATAGGAACTCTGAGGAAATGCGTAATCTC
GTTGGCCGCTTTGCTGGTATTGTATTCACAGCATTTTTTATGCTATTGATATCAATCATTTGGGCTTTCGTGAATACATGGAAACTGACATTAG
TCTCGATGGCAACTGGGCCAGTTATATACGCTGTCACCAAAACGTTCAATCGCGTGAGTGGAAAATGGGAAAACAAGTGCAACTACGCCTCTGA
AATGACCACTGGCATATTTTCAGAGACGTTCTCCAACATCAAAGTGGTTCGGGCTTTTACTCTGGAAACTTACTTTGAGACAAAACACACCAAA
GCTACAGAAGAACTTTATAAAGTTGGACTAATACGAGCAAACTACTCGGGATTGCTGTGGGGATTGACAGATGCGATGTCATTCTTCATCACTG
CAACTATCTTTTATTATGCCACGGTTCTCATTACCAAGAGAGAGATCAGTATCGCGACTGCACTACAGACTGTCAATCTTCTATTATTTGGTAT
TTCTAATAGTACGAATATGCTGGCCATGATACCACAAATCAACTCTTCTCGCGTTACAGCTACGCATATGCTTGCATTAGCCAATCTCGATTCA
TCTTCCTCCCACGAAAATAAAGGAACGAACGGCTTTCGACAATCTTTCCAATCAAATTCAACCGTCTTTCATTCACATACCCTACTCGTCCTGA
AAAACGAACGATATCATCCTTTTCTCTTTCCCTGATTCCTAACTCAACAACTGCACTTGTCGGACCCTCCGGCTCCGGAAAATCTACAATAGCT
GCTCTGCTCATTGGTCTCTATCCGCCAGGTACTTCAACACCTCCACCGTTGACATTCAATCGCGTCTCCATAAGTAACTGTCACATTCCGTCTC
TCCGGGCTTCTCTCTCACTCGTTCCACAAATACCGATTCTATTTCCAGCTACCATTCTCCATAACATCATTTATGGTCTCCCAGAATCTTCTCC
TTGTGCTAGTCTTCCATCTGCTATTGCATCAGCAAAAGATGCTGGGATCCATGAATTTATCACATCGCTTCCACAAGGTTATGATACTAT

>Bcin05g07690(MLST2), partial sequence [organism=Botrytis cinerea, strain D11_T_B45]
ACCACTATCACCAAGTCCTTTTCTGCACTTTCCTCATCAAAAACGAACGAGAACATCATCAATAAATACTTTGCTGAAGTAATTGGGTTTTATT
TTGGCCAGGATGCCTTCTCTCTACGACAGCAAGCCTTCGATGATATGTTGTGGGTAGTTCTTGGCTGGCTGGATACTGTCAAATTCATTGATTT
ACATTCTGAATTGCACTATTCAAACGACTCTCAGCCAGAATGGTACGGACAACAATATAAACCTGCATTTGCACATCGAGCGCGACTATTTTGG
GAATTGGCTTCACAAGGATGGGATACTACTCTCTGTGGTGGTGGGATGATATGGTCACCATACCTTACTCCATACAAGAACGCAATTACCAATG
AACTCTATATCGCAGCTTCGATATCGATGTACCTATATTTCCCCGGAGATGACAATCAATCCCCATTTATGCTTTCCAGCCCTTCGTATCCACC
TCACGATCCGAAATATCTACAGGCAGCTGTTGATGCTTACAAATGGCTGAATGGTTCCAACATGACGGATTTACAAGGATTATATGTCGACGGG
TACCATATCTCGAATCTTTCTGGCGGTGAAAACACCCATTGCGATTCTAGAAATGAGATGGTATATACCTACAATCAAGGTGTTTTGCTTACTG
GACAACGTGGTTTGTATGACGCAACCGCCGCACGATCATACCTTGTAGATGGCCACAAACTCATCGCGAATGTTATTAATGCCACAGGCTATGA
CCTGAAACACAATGTTGTCATCTCACCGCCACCCAAAGATGGTTCCGCATTGGCAAAGTGGTTTGGCCTGGGTAGGAATGGAATACTGGAAGAA
GGATGCGATTCAAGTGCTTCGTGTTCTCAAAATGGACAAACTTTCAAAGGCATATTCTTTCATCACTTGATTGCGTTCTGTAGTGATTTGCCAG
GGGAGCCTATTGCAGGGACGAAGGAAAGCTTAGAACTCGACAGAGTGTGGCATTCTGACAAATGCTCACAGTATACAAAAT

>Bcin06g01710(MLST3), partial sequence [organism=Botrytis cinerea, strain D11_T_B45]
GTGAGTCTGACTTTTGTATTTGAGCGTTAAGATAGACACTGATATACCAAGGCAATCACCTAATTTCATGGCGTTCTTCCCTGCATCATCTACC
TACCCTGGAATGCTGGGAGAATTATACTCAGCAGCTTTCACAGCACCTGCTTTCAATTGGATCTGTTCCCCTGCTGTGACAGAATTGGAGACGG
TTGTAATGGATTGGCTGGCCAAGCTTCTCAATCTCCCAGACTGTTATTTGTCTTCGACTCATGGTGGTGGTGTCATCCAAGGATCAGCCTCGGA
AGCTATCGTTACCGTTATGGTTGCTGCCCGCGATAAATATCTTCGTGAAACTACTGAAGGTCTGTCGGGCATTGAGCTCGAGGATGCGATTGCA
TATAAGAGGAGTAAGCTAGTTGCACTAGGAAGCGAAATGGCACACAGCTCCACGCAGAAAGCAGCGCAAATAGCTGGCGTTAGATTCCGATCGA
TTCCAGTACTCGCATCCGATGATTTCGCCATGACGGGTGATGATTTAGAGAAGGTATTGAAAGAATGCAAATCTCAAGGATTGGAACCCTTCTA
TCTAACTTCGACTTTGGGAACAACATCTACATGCGCAGTTGACGACTTCGCATCTATTGCAACAGTACTTTCAAAATATGCACCTCCAGATGTT
GCAGGCGAGATCTGGGTTCACGTCGATGCTGCTTATGCAGGTGCAGCTTTGGTTTGCCCTGAATACCATCATCTAACATCGTCCTTCCAGCATT
TCCATTCCTTCGATATGAACATGCACAAATGGCTTCTGACAAATTTCGACGCTTCTTGTCTATATGTCAAGAAACGCAAAGATCTGATCGATGC
ACTCTCCATAACACCAAGTTATCTTCGCAACGAGTTTTCAGAGAGTGGACTCGTAACCGACTATCGGGACTGGCAAATTCCTCTCGGAAGACGC
TTCCGAAGCTTAAAGATTTGGTTTGTCCTCAGAACCTAC

>Bcin09g03030(MLST4), partial sequence [organism=Botrytis cinerea, strain D11_T_B45]
ACCCTTCAACCCCACCGCAGCTCCTATACCGAAAGCGAGTATCCTACCAATTCTCCTTCCACCTGCGACTTTAAGACCATTGGCTTTCCGCACT
TTCACAAAAAAGCATAGTTTAACATTGACGTCGTCGGCATTACAAGTGTTGGCTACTTTTATTGGAAAGCATTGTGGGACAGGATGGAGGGAAG
AAGGACTGGCAGAGAGAGTCTTGGAGGAGGTCGCCAAGAGTTGGAAGAATAGGAGTGGCGGTGTCATTGTCGAGGGCGAGGGAACGGAATTGAA
GGAGATTCTGAAAGCTTTGGAGGGAATATGAGTGGTGGAAGGATAGTCATAGGAAGAGAGCTAAGCCGGCAGAATAGTTTAGTACTGGGATCAT
CACAATATGGAGAGGTCAATCATACAAGACTTGGGCTACGGCCAGGGAATATACCCAGAGAGGATAGTCAGTCAAGTTTGGGAATGTCAACGTT
GGAGGTCAATGACGAGGAAGATGAGGATGGCCTGATGGATCCAAGAAGGTGGTTAAAAGTCATTGATGCATTTGAGCAACCTCGACTGGTGTAC
AATGTTGCTAAAAAGCACTTTGATAGGTATGTTTCAATGATAAAATTTTATCTGAATCGACTAACTCAGTACAGAGATACCTCCAAACCTTCAT
TGTTCCCACCTGCGTCTCATAAAACACTCCTCTTCCAAAACCGCTATAATGTTATCCATCAACGTCTCCTTCGCAATGAATCTTTTCAAACGCC
CGCTTTTCAAGGTGGCAAATCTTCCCTTCAACGCAGCACGTCCGCCATTACCACCCAACAACAATCATACAAATTAACGCCGATAGCTAATCTT
CTCGGTCGCAATCGCAGCTCTCGTATGCTTCTCGGTCTCCTCAGTATTTCACCCACTGGTACCCTCGCCATCAATGACCTGACGGGCAGTATCA
CTCTCGATCTTACACACGCAGCAGCCATTCC

>Bcin11g01310(MLST5), partial sequence [organism=Botrytis cinerea, strain D11_T_B45]
GACTGACATGGACCTCATGTGGAACCGGCGTAGAATGCGCAACGCTTGAAGTTCCGCTCGAATATGGCGATGCAACGTCAACGGCAAAAGCCAG
TGTTGCGCTTGCTCGTTATCCTGCCACTGTTGCCGCGAGCAAGAAGCTCGGGTCTCTCTTGATAAACCCCGGTGGACCCGGTGCCTCTGGTGTT
GGCTTTGTGCAGTCTGGAGCCGGTGCCGCCGTCTCGACACTGAGTGGTGGATTGTACGATATCATCGGATGGGATCCACGTGGAACCGGTGCTT
CGGCTCCTATTTTGGAATGTTTTGCAAATGCCAGTGCGGAGTATGATTTTAACAACGCGTTTCCATCTGCTCCGAATCTCTGGCTCGGACAATT
TGCGAATGCCAGCGCAAATTCTGCTGTTAGCTCTGCTATCACATCCTTTGACACTTCTGTCGCTGCTCTTGCAAAAGCTTGCGTGGCTCAGAAA
TCTCCCGCTCTTTACACCTCAACAGCAGCATATGTTGCTCGAGACATGGCAGCGATAGTCGATGCATTGGATGGGACCTCTGCAAAACTTAACT
ACTGGGGTTTCTCATATGGAACTATTTTCCTAGCTGAGTTTATCCAAACTTTCCCAGGCCGTGTGGGAAGAGTTCTTGCCGATGGTGTTTTCGA
CGCAAAGGCAAATGCACTCACATACGTTAGCCAACTTCCCAACGATCAACTCAGTGTTCGTGCTTCGTTGAACGATTTTGCAGTTTCTGCACCA
CCGCCGGTAGTAAAGGTTGCTCTTTTGCCACCGCCCCTACTGGAACCTCAGGTACTGTTGCTACCAGACTGGACAACATAATGAAGGATATGTT
CCTCAATCCTATTGTTGCTTCGGGCTTAAGCATCA

>Bcin15g03910(MLST6), partial sequence [organism=Botrytis cinerea, strain D11_T_B45]
CGCCAAAACACAAAATCATCCAACGATGAAGATGATACTCCACTTCCCTTGATTATCTGGCATGGACTCGGCGATAATTACAAAGCGGATGGTC
TTGCGCAAGTTGGAAAACTAGCTGAAGCTATTCATCCTGGGACTTTTGTCTACAATATTCATGTAGATGAGGATGCATCTGCAGATAGGACAGC
TACCTTCTTTGGAAATCTCACTCGTGAGTACATCCCCTATTTTTCCTTTAAATATCATACTAACTCTCTTACCAAGTTCAAATCGAAAAGGTCT
GCGAAGACCTCGCCTCCCATCCTATTCTCTCTACCGCGCCCGCCGTCGACGCAATTGGATTCTCCCAAGGCGGCCAATTCTTGCGTGGCTACAT
ATCCCGCTGCAATGCTCCACCCATCCGCTCTCTCCTGACCTTCGGTTCCCAACACAATGGCATTTCTGCCTTCCAAGCCTGTGGTCCTGCCGAT
TTCCTCTGTCGCGGTGCTCAAACCCTTTTGCGATCCAACACCTGGTCAACCTTTGTCCAATCTCGTCTCGTACCCGCTCAATACTTCAGAGATC
CGGAAAACCTAGACTCTTACCTTGAATATTCCAATTTCCTTGCCGACATCAATAATGAGCGCGTTCTCAAGAACCAAACATATAAATCCAACAT
GGAAAAATTGGAACGATTCGTAATGTATGTCTTTGAAGACGATACAACTGTCATCCCTAAGGAAAGTGGATGGTGGGCTGAAGTCAACGGCACG
GAAGTTACACCACTGAAAGAAAGAGCCATTTATAAAGAAGATTGGCTAGGTTTAAAGACATTGGATGAGGCCGGAAAATTAGTTTTCGAAACCA
TTCCAGGGGGACATATGACGTTAGGAGAGGAGATGCTAGAGAAGGCTTTCAAAGAGTATTTTGGTCCAGCAGGGAAGAAATTTGGG

>Bcin16g03460(MLST7), partial sequence [organism=Botrytis cinerea, strain D11_T_B45]
ATGAACCTCTTGATTTGAACTCTTCATTAATTGCAAGTGATGAACCCCTATTCTGCCCAGAGGATAGTTACAAGACGTATATCATTAGTCGAGA
ACCACTCATGATATATATTGACGGGTTTTTGAGAGCGAATGAAAGTAAACATTTGGTTGATGTTAGGTGTGTTATTTATTCTGATGAAATGAAC
AAGAGAGACTGATGAGATAGTGAGCCGCTTTATGAACCGTCAACTGTTTCTCACGGACAGGAAGTTACCATTGATCCTTCAGTTCGGAATTCTG
AAGTGGCGGTTTTAGAGAGGGATGAGGTGGTCAGGTGTATTGAGCATAGAGCGAGGGCATTTCAGGGGTGGAGGGGCGAGATGGGGATTGAGAA
GTTGAGGACGCAGAGGTATGGGGTTGGAGGACATTATGGGATGCATTTGTAAGTTTTGGGGGATTGACGAAGGCTTTTGTCTATTTTCTACCAG
TACGGATCTTGAAAGAAAGATAGCATGAACACGAGGGCTAATAACTAGGAAATAGCGATTGGAGCGGAGGTAAACGTGGCATAGACCGATTTAG
TACTTTCATGGTCTATGTCGACGTATCCTCTGATATCGAAGGTGGAGGAACGGAATTCCCACGTATCGTGGGACCAAAAGGAGGAAGGTGGGAG
GACTTCCTGGAAACTACGGAAGCATTGGATCCAAGAACTGGAGAAAATGTAACAGTAGAAGGGGTGACATTCAAACCAATCAAGGGAAATGCCG
TATTCTGGGAAAATACTGACAACAACGGGAGGGGCTATGAT

>Bcin12g03020(MLST8), partial sequence [organism=Botrytis cinerea, strain D11_T_B45]
CGATTGGCTGCGAAGAAAACTGCGCAGCACCGAGTCACCAAAAGCACGAATAAACCATCGGCAAGGGAAATATTCTCTGAACCACAACATGACA
CGAGCGCGGAAGAGTATATCGGGCGAGAAGCTTCATCAAGAGCACCAAAGCGACAACGAGTCGATGATAATTATAACTCTTACGGTGGAAGAAA
TGAAAATACAGCAGCTTATGCTTCCGGGAAACTTCCATCTGGAAGTATAAATGTTGGTGGAGGTAGGAAGACCACTTTTCAAGAAGAACCACGA
ACGGCATTTGTCGCTGGCAAGTTGCCCCCTGGAAGTATCAACATTGGTGGAAAGAAGGCTACCCAAATCGAGGACGAGGGTAGAGCAGCTTATG
CTTCCGGAAAATTGCCTCCAGGAAGTATAAACTCTGGCGCAAAGAAGGCGATTTCATTCCAAGATGAAACGAGACCGGCTTATGTCTCCGGAAA
GCTTCCACATGGTAGTATCGACGGTATGCGAAACCGTGAAATGGCTGCCGTCCACCGCGAAATTGCTGAGGGTGGGAGGAAACCAGGCCAGGTT
GTCTCTTCTCTATTCACATTCAATCCTACTTCAAAGAAAACTTTTGATGAACCCGAAGAACAAGCGGAACCGGCAAAACCATCCAATGCGCCTT
TGACAGAGGAAATGGCGACTTTCACCAATCTAGGGTTATCGAGAAGGCTTGCAGCCCATCTATCGACTAAACTCGATATGAAAGCTCCGACCGC
CATTCAAAAAGCATCTGTACAGCAGTTGGTATCGGACGATAGCGATGCTTTCATACAAGCAGAGACTGGATCTGGAAAAACTTTGGCATATCTA
CTACCTATAGTCGAGCGAATATTAGCATTGAGTGAGAATGGCGTACAAA

>Bcin02g07770(MLST9), partial sequence [organism=Botrytis cinerea, strain D11_T_B45]
CAGCTTTCCCTTTCGGTCTTGGCATCTACAGTCATTGCCATCCCTACACCATCACAACTTGAGTCTCGGGCCGTTATCGATTCCGATGCCGTTG
TAGGATTTGCCGAAACTGTTCCCAGTGGGACCGTAGGAACAGTTTATGAGGCATATAAACCATTCCTTAAAGTCGTAAATGGATGCGTACCATT
CCCTGCCGTCGATGCATCGGGTAACACAGGGTATGTCCTTATATCTTTCTCTTCCACACGATTGCTATTGAGTCTCTAACATATTTAGTGGTG
GTTTGTCACCAACTGGCAGTAGCAATGGTGGTTGCAGCAGCAGTACCGGTCAAGTATATGTTCGAGGAGGACAAAGCGGATCAAACTACGCCAT
CATGTACTCCTGGTAAGTTCTCTCTAAACTTCTCCTTATAGATCCAACCTAACAAAATCTTAGGTACATGCCAAAGGACGAGCCCTCAACCGGT
ATTGGTCACCGTCACGATTGGGAAGGTGTAATTGTCTGGCTCTCCAGCGCCACCGCCACAACTGCCGACAACATCTTAGCCGTTTGTCCTTCCG
CCCACGGAGGCTGGGATTGTTCCACGGATGGCTATTCCCTTTCTGGTACCAGCCCTCTTATCAAGTACGAAAGTATCTGGCCCGTCGATCACTC
AATGGGTCTTACTAGTACTGTTGGTGGAAAACAACCTATGATTGCTTGGGAGTCTTTACCAACTGCTGCTCAAACTGCTCTTGAGAACACCGAT

TTCGGTGCTGCGAATGTTCCATTCATTCCGGCTGTTTTCACAGACAATCT

>Bcin04g02090(MLST10), partial sequence [organism=Botrytis cinerea, strain D11_T_B45]
CGGAGGATGATATGGCAAAGTCTATGATTACCAAAGCATTTGTAGGCATGAGTAGTAAACTGTCGAATATGACTATGAATGATGTTTACAAGCC
CTACATCCATGTAAGAAATGTAGAATAGAGAGATCAGTAACTGGAACTAATATCGTTTGTAGGCTTTCAAGTTACTTACGCAGTTCAACCCAAT
CACTACAGCTATTGCCGAATCCCCACTGTTTCAAATGGCCGTCTCAGCAAATACCATCGAAAAGTACACACTGCTAGGCCCTTTCTTCAGAATA
TCTCCTCTGCAACAGGAAGTTACCAGGGAATACTTCAGTGCGCCAAAGACGATAGATAGGCGACACATTGCCACATCTCAAGATGCGTTACGAT
TGACCTTACAAACCCATCAAAAAGATTTACTTGATATCATCAACCACTTTGTTCGAGCAAGTCCAATCGCAAAAAGCAAAACCCTGGATTGGTT
CGCCTACATTGTGAATCAAAATCACAAGCGTCGAGCACTTCAGGTAGACCCGAAAGAAGTGTCTTCTGATGGCTTTATGCACAATGTCACTGTC
GTTCTAGATGGTCTTTGTGAGCCATTCATGGATACCACATTCTCGAAAATTTCGAAGATTGATATTGATTATCTAAGACGTGCGCCTCGTGTAG
ATATCAAGGACGAGACCAAGTTGAACGCTGACGAGAAGGCTTCTGAGAAGTATTATGAGGACACTGTTCCTGGCACTTCTAATTTCATCTCTGA
GGTCTTCTTTCTCACATTGGCTGCTCATCATTATGGTAGTGAAGCTCTTAATGCCACGCATAAGAGTCTGGAGAAAGACATCAAATATATTCAA
AAGCAATTGACTGCCGTTGAAGCA

>Bcin01g07220(MLST1), partial sequence [organism=Botrytis cinerea, strain D11_T_E12]
ATGGCATATCTTGTTTCTTTTCGCATTATGCTTTGGAACGTGCTGGGCAAGCTTGGGTTAGTGCTCTGCGTGTAGAAGCACTAAAGAAGATTCT
CGCACAACCGAAGTCATGGTTTGAGGAATCCAGGAATTCACCTAGCCGGTTGAACGAAGTTTTGGATAGGAACTCTGAGGAAATGCGTAATCTC
GTTGGCCGCTTTGCTGGTATTGTATTCACAGCATTTTTTATGCTATTGATATCAATCATTTGGGCTTTCGTGAATACATGGAAACTGACATTAG
TCTCAATGGCAACTGGGCCAGTTATATACGCTGTCACCAAAACGTTCAATCGCGTGAGTGGAAAATGGGAAAACAAGTGCAACTACGCATCTGA
AATGACCACTGGCATATTTTCAGAGACTTTCTCCAACATCAAAGTGGTTCGGGCTTTTACTCTGGAAACTTACTTTGAGACAAAACACACCAAA
GCTACAGAAGAACTCTATAAAGTTGGACTAATACGAGCAAACTACTCGGGATTGCTGTGGGGATTGACAGATGCGATGTCATTCTTCATCACTG
CAACTATCTTTTATTATGCCACGGTTCTCATTACCAAGAGAGAGATCAGTATTGCGACTGCACTACAGACTGTCAATCTTCTATTATTTGGTAT
TTCTAATAGTACGAATATGCTGGCTATGATACCACAAATCAACTCTTCTCGCGTTACAGCTACGCATATGCTTGCATTAGCCAATCTCGATTCA
TCTTCCTCCCACGAAAATAAAGGAACCGAACGGCTTTCGACAATCTTTCCAATCAAATTCAACCGTCTTTCATCCACATACCCTACTCGTCCTG
AAAAACGAACGATATCATCCTTTTCTCTTTCCCTGATTCCTAACTCAACAACTGCACTTGTCGGACCCTCCGGCTCCGGAAAATCTACGATAGC
TGCTCTGCTCATTGGTCTCTATCCGCCAGATACTTCAACACCTCCACCGTTGACATTCAATCGCGTCTCCATAAGTAACTGTCACATTCCGTCT
CTCCGGGCTTCTCTCTCACTCGTCCCACAAATACCGATTCTATTTCCAGCTACCATTCTCCATAACATCATTTATGGTCTCCCAGAATCTTCTC
CTTGTGCTAGTCTTCCATCTGCTATTGCATCAGCAAAAGATGCTGGGATCCATGAATTTATCACATCGCTTCCACAAGGTTATGATACTAT

>Bcin05g07690(MLST2), partial sequence [organism=Botrytis cinerea, strain D11_T_E12]
CACCACTATCACCAAGTCCTTTTCTGCACTTTCCTCATCAAAAACGAACGAGAACATCATCAATAAATACTTTGCTGAAGTAATTGGGTTTTAT
TTTGGCCAGGATGCCTTCTCTCTACGACAGCAAGCCTTCGATGATATGTTGTGGGTAGTTCTTGGCTGGCTGGATACTGTCAAATTCATTGATT
TACATTCTGAATTGCACTATTCAAACGACTCTCAGCCAGAATGGTACGGACAACAATATAAACCTGCATTTGCACATCGAGCGCGACTATTTTG
GGAATTGGCTTCACAAAGATGGGATACTACTCTCTGTGGTGGTGGGATGATATGGTCACCATACCTTACTCCATACAAGAATGCAATTACCAAT
GAACTCTATATCGCAGCTTCGATATCGATGTACCTCTATTTCCCCGGAGATGACAATCAATCCCCATTTATGCTTTCCAACCCTTCATATCCAC
CTCACGATCCGAAATATCTACAGGCAGCTGTTGATGCTTACAAATGGCTGAATGGTTCCAACATGACGGATTTACAAGGATTATATGTCGACGG
GTATCATATCTCGAATCTTTCTGGCGGTGAAAACACCCATTGCGATTCTAGAAATGAGATGGTATATACCTACAATCAAGGTGTTTTGCTTACT
GGACAACGTGGTTTGTATGACGCAACCGCCGCACGATCATACCTTGTGGATGGCCACAAACTCATCGCGAATGTTATTAATGCCACAGGCTATG
ACCTGAAACACAATGTTGCCATCTCACCGCCACCCAAAGATGGTTCCGCATTGGCAAAGTGGTTTGGCCTGGGTAGGAATGGAATACTGGAAGA
AGGATGCGATTCAAGTGCTTCGTGTTCTCAAAATGGACGAACTTTCAAAGGCATATTCTTTCACCACTTGATTGCGTTCTGTAGTGATTTGCCA
GGGGAGCCTATTGCAGGGACGAAGGAAGGCCTAGAACTCGACAGAGTGTGGCATTCTGACAAATGCTCACAGTATACAAAATG

>Bcin06g01710(MLST3), partial sequence [organism=Botrytis cinerea, strain D11_T_E12]
GTGAGTCTGACTTTTGTATTTGAGCGTTAAGATGGACACTGATGTACCAAGGCAATCACCTAATTTCATGGCGTTCTTCCCTGCATCATCTACC
TACCCTGGAATGCTGGGAGAATTATACTCAGCAGCTTTCACAGCTCCTGCTTTCAATTGGATCTGTTCCCCTGCTGTGACAGAATTGGAGACGG
TTGTAATGGATTGGTTGGCCAAGCTTCTCAATCTCCCAGACTGTTATTTGTCGTCGACTCATGGTGGTGGTGTTATCCAAGGATCAGCCTCGGA
AGCTATCGTTACCGTTATGGTTGCTGCCCGCGATAAGTATCTTCGTGAAACCACTGAAGGTCTGTCGGGAATTGAACTCGAGGATGCGATTGCA
TATAAGAGGAGTAAGCTAGTTGCACTAGGAAGCGAAATGGCACACAGCTCCACGCAGAAAGCAGCGCAGATAGCTGGCGTTAGATTCCGATCGA
TTCCAGTACTCGCATCCAATGATTTCGCCATGACGGGTGATGATTTAGAGAAGGTATTGAAAGAATGCAAATCTCAAGGATTGGAACCCTTCTA
TCTAACTTCGACGTTGGGAACAACATCTACATGCGCAGTTGACGACTTCGCATCTATTGCAACAGTACTTTCAAAATATGCACCTCCAGATGTT
GCAGGCGAGATCTGGGTTCACGTCGATGCTGCTTATGCAGGTGCAGCTTTGGTTTGCCCTGAATACCATCATCTAACATCGTCCTTCCAGCATT
TCCATTCCTTTGATATGAACATGCACAAATGGCTTCTGACAAATTTCGACGCTTCTTGCCTATATGTCAAGAAACGCAAAGATCTGATCGATGC
ACTCTCCATAACACCAAGTTATCTTCGCAACGAGTTTTCAGAGAGTAGACTCGTAACCGACTATCGGGACTGGCAAATTCCTCTCGGAAGACGT
TTCCGAAGCTTAAAGATTTGGTTTGTCCTCAGAACCTAC

>Bcin09g03030(MLST4), partial sequence [organism=Botrytis cinerea, strain D11_T_E12]
ACCCTTCAACCCCACCGCAGCTCCTATACCGAAAGCGAGTATCCTACCAATTCTCCTTCCACCTGCGACTTTAAGACCATTGGCTTTTCGCACT

TTCACAAAAAAGCATAGTTTAACATTGACGTCGTCGGCATTACAAGTGTTGGCTACTTTTATTGGAAAGCATTGTGGGACAGGATGGAGGGAGG
AAGGACTGGCAGAGAGAGTCTTGGAGGAGGTCGCCAAGAGTTGGAAGAATAGGAGTGGCGGTGTCATTGTCGAGGGCGAGGGAACGGAATTGAG
GAGATTCTGAAAGCTTTGGAAGGGAATATGAGTGGTGGAAGGATAGTCATAGGAAGAGAGCTAAGCCGGCAGAATAGTTTAGTACTGGGATCAT
CACAATATGGAGAGGTCAATCATACAAGACTTGGGCTACGGCCAGGGAATATACCCAGAGAGGATAGTCAGTCAAGTTTGGGAATGTCAACGTT
GGAGGTCAATGACGAGGAAGATGAGGATGGCCTGATGGATCCAAGAAGGTGGTTAAAAGTCGTTGATGCATTTGAGCAACCTCGACTGGTGTAC
GATGTTGCTAAAAAGCACTTTGATAGGTATGTTTCAATGATAAAATTTTATCTGAATCGACTAACTCAGTACAGAGATACCTCCAAACCTTCAT
TGTTCCCACCTGCGTCTCATAAAACACTCCTCTTCCAAAACCGCTATAATGTTATCCATCAACGTCTCCTTCGCAATGAATCTTTTCAAACGCC
CGCTTTTCAAGGTGGCAAATCTTCCCTTCAACGCAGCACGTCCGCCATTACCACCCAACAACAATCATACAAATTAACGCCGATAGCTAATCTT
CTCGGTCGCAATCGCAGTTCTCATATGCTTCTCGGTCTCCTCAGTATTTCACCCACTGGTACCCTCGCCATCAATGACCTGACGGGCAGTATCG
CTCTTGATCTTACACACGCAGCAGCCATTC

>Bcin11g01310(MLST5), partial sequence [organism=Botrytis cinerea, strain D11_T_E12]
GACTGACATGGACCTCATGTGGAACCGGCGTAGAATGCGCAACGCTTGAAGTTCCGCTCGAATATGGCGATGCAACGTCAACGGCAAAAGCCAG
TGTTGCGCTTGCTCGTTATCCTGCCACTGTTGCCGCGAGCAAGAAGCTCGGGTCTCTCTTGATAAATCCCGGTGGACCCGGTGCCTCTGGTGTT
GGCTTTGTGCAGTCTGGAGCCGGTGCCGCCGTCTCGACACTGAGTGGTGGATTATACGATATCATCGGATGGGATCCACGTGGAACCGGTGCTT
CGGCTCCTATTTTGGAATGTTTTGCAAATGCCAGTGCGGAGTATGATTTTAACAACGCGTTTCCATCTGCTCCGAATCTCTGGCTCGGACAATT
TGCGAATGCCAGCGCAAATTCTGCTGTTAGCTCTGCTATCACATCCTTTGACACTTCTGTCGCTGCTCTTGCAAAAGCTTGCGTGGCTCAGAAA
TCTCCCGCTCTTTACACCTCAACAGCAGCATATGTTGCTCGAGACATGGCAGCGATAGTCGATGCATTGGATGGGACCTCTGCAAAACTTAACT
ACTGGGGTTTCTCATATGGAACTATCTTCCTAGCTGAGTTTATCCAAACTTTCCCAGGCCGCGTGGGAAGAGTTCTTGCCGATGGTGTTTTCGA
CGCAAAGGCAAATGCACTCACATACGTTAGCCAACTTCCCAACGATCAACTCAGTGTTCGTGCTTCGTTGAACGATTTTGCAGCTTTCTGCACC
ACCGCCGGTAGTAAAGGTTGCTCTTTTGCCACCGCCCCTACTGGAACCTCAGGTACTGTTGCTACCAGACTGGACAACATAATGAAGGATATGT
TCCTCAATCCTATTGTTGCTTCGGGCTTGAGCATCA

>Bcin15g03910(MLST6), partial sequence [organism=Botrytis cinerea, strain D11_T_E12]
CGCCAAAACACAAAATCATCCAACGATGAAGATGATACTCCACTTCCCTTGATTATCTGGCATGGACTCGGCGATAATTACAAAGCGGATGGTC
TTGCGCAAGTTGGAAAACTAGCTGAAGCTATTCATCCTGGGACTTTTGTCTATAATATTCATGTAGATGAGGATGCATCTGCAGATAGGACAGC
TACCTTCTTTGGAAATCTCACTCGTGAGTACATCCCCTATTTTTCCTTTAAATACCATACTAACTCTCTTACCAAGTTCAAATCGAAAAGGTCT
GCGAAGACCTCGCCTCCCATCCTATTCTCTCTACCGCGCCCGCCGTCGACGCAATTGGATTCTCCCAAGGCGGCCAATTCTTGCGCGGTTACAT
ATCCCGCTGCAATGCTCCACCCATCCGCTCTCTCCTGACCTTCGGTTCCCAACACAACGGCATTTCTGCCTTCCAAGCCTGTGGTCCTGCCGAT
TTCCTCTGTCGCGGTGCTCAAACCCTTTTGCGATCCAACACCTGGTCAACCTTTGTCCAATCTCGTCTCGTACCTGCTCAATACTTCAGAGATC
CGGAAAACCTAGACTCTTACCTTGAATATTCCAATTTCCTTGCCGACATCAATAATGAGCGCGTTCTCAAGAACCAAACATATAAATCCAACAT
GGAAAAATTGGAACGATTCGTAATGTATGTCTTTGAAGACGATACAACTGTCATCCCTAAGGAAAGTGGATGGTGGGCTGAAGTCAATGGCACG
GAAGTTACACCACTGAAAGAAAGAGCCATTTATAAAGAAGATTGGCTAGGGTTAAAGACATTGGATGAGGCCGGAAAATTAGTTTTCGAAACCA
TTCCAGGGGGACATATGACGTTAGGAGAGGAGATGCTAGAGAAGGCTTTCAAAGAGTACTTTGGTCCAGCAGGGAAAAAATTTGGG

>Bcin16g03460(MLST7), partial sequence [organism=Botrytis cinerea, strain D11_T_E12]
ATGAACCTCTTAATTTGAACTCTTCATTAATTGCAAGTGATGAACCCCTATTCTGCCCAGAGGATAGTTACAAGACGTATATCATTAGTCGAGA
ACCACTCATGATATACATTGACGGATTTTTGAAAGCGAATGAAAGTAAACACTTGGTTGATGTTAGGTGTGTTATTTATTCTGATGAAATGAAC
AAGAGAGACTGATGAGATAGTGAACCGCTTTATGAACCGTCTACTGTTTCTCACGGACAGGAAGTTACCATTGATCCTTCGGTTCGAAATTCTG
AAGTGGCGGTTTTAGAGAGGGATGAGGTGGTCAGGTGTATTGAGCATAGAGCGAGGGCATTTCAGGGGTGGAGGGACGAGATGGGGATTGAGAA
GCTGAGGACGCAGAGGTATGGGGTTGGAGGACATTATGGGATGCATTTGTAAGTTTTGGGGGATTGACGAAGGCCTCTGTCTATTTTCTATCAG
TACGGATCTTGAAAGAAAGATAGCATGAACACGAGGGCTAATAACTAGGAAATAGCGATTGGAGCGGAGGTAAACGTGGCATAGACCGATTTAG
TACTTTCATGGTCTATGTCGACGTATCCTCTGATATCGAAGGTGGAGGAACGGAATTCCCACGTATTGTGGGACCAAAAGGAGGAAGGTGGGAG
GACTTCCTGGAAACTACGGAAGCATTGGATCCAAGAACTGGAGAAAATGTAACAGTAGAAGGGGTGACATTCAAACCAATCAAGGGAAATGCCG
TATTCTGGGAAAATACTGACAACAACGGGAGGGGCTATGAT

>Bcin12g03020(MLST8), partial sequence [organism=Botrytis cinerea, strain D11_T_E12]
TCGATTGGCTGCGAAGAAAACTGCGCAGCACCGAGTCACCAAAAGCACGAATAAACCATCGGCAAGGGAAATATTCTCTGAACCACAACATGAC
ACGAGCGCGGAAGAGTATATCGGGCGAGAAGCTTCATCAAGAGCACCAAAGCGACAACGAGTCGATGATAATTATAACTCTTACGGTGGAAGAA
ATGAGAATACAGCAGCTTATGCTTCCGGGAAACTTCCATCTGGAAGTATAAATGTTGGTGGAGGTAGGAAGACCACATTTCAAGAAGAACCTCG
AACGGCATTTGTCGCTGGCAAGTTGCCCCCTGGAAGTATCAACATTGGTGGAAAGAAGGCTACCCAAATCGAGGACGAGGGTAGAGCAGCTTAT
GCTTCCGGAAAATTGCCCCCAGGAAGTATAAACTCTGGCGCAAAGAAGGCGATTTCATTCCAAGATGAAACGAGACCGGCTTATGTCTCTGGAA
AGCTTCCACATGGTAGTATCGACGGTATGCGAAACCGTGAAATGGCTGCCGTCCACCGCGAAATTGCTGAGGGTGGGAGGAAACCAGGCCAGGT
TGTCTCTTCTCTATTCACATTCAATCCTACTTCAAAGAAAACTTTCGATGAACCCGAAGAACAAGCGGAACCGGCAAAACCATCCAATGCACCT
TTGACAGAGGAAATGGCGACTTTCACCAATCTAGGGTTATCGAGAAGGCTTGCAGCCCATCTATCGACTAAACTCGATATGAAAGCCCCGACCG
CCATTCAAAAAGCATCTGTGCAGCAGTTGGTATCGGACGATAGCGATGCTTTCATACAAGCAGAGACTGGATCTGGAAAAACTTTGGCATATCT
ACTACCTATAGTCGAGCGAATATTAGCATTGAGTGAGAATGGCGTACAAA

```
>Bcin02g07770(MLST9), partial sequence [organism=Botrytis cinerea, strain D11_T_E12]
CAGCTTTCCCTTTCGGTCTTGGCATCTACAGTCATTGCCATCCCTACACCATCACAACTTGAGTCTCGGGCCGTTATCGATTCCGATGCCGTTG
TAGGATTTGCCGAAACTGTTCCCAGTGGGACCGTAGGAACAGTTTACGAGGCATATAAACCATTCCTTAAAGTCGTAAATGGATGCGTACCATT
CCCTGCCGTCGATGCATCGGGTAACACAGGGTATGTCCTTATACCATTCTCTTCCACACGATTGCTATTGAGTCTCTAACATATTTTAGTGGTG
GTTTGTCACCAACTGGCAGTAGCAATGGTGGTTGCAGCAGCAGTACCGGTCAAGTATATGTTCGAGGAGGACAAAGCGGATCAAACTACGCCAT
CATGTACTCCTGGTAAGTTCTCTCTAAACTTCTCCTTATAGATCCAACCTAACAAAATCTTAGGTACATGCCAAAGGACGAGCCCTCAACCGGT
ATTGGTCACCGTCACGATTGGGAAGGTGTAATTGTCTGGCTCTCAAGCGCCACCGCCACAACTGCCGACAACATCTTAGCCGTTTGTCCTTCCG
CCCACGGAGGCTGGGATTGTTCCACCGATGGATATTCCCTTTCTGGTACCAGCCCTCTTATCAAGTACGAAAGTATCTGGCCCGTCGATCATTC
AATGGGTCTTACTAGTACTGTTGGTGGAAAACAACCTATGATTGCTTGGGAGTCTTTACCAACTGCTGCTCAAACTGCTCTTGAGAACACCGAT
TTCGGTGCTGCGAATGTTCCATTCATTCCGGCTGTTTTCACAGATAATCT

>Bcin04g02090(MLST10), partial sequence [organism=Botrytis cinerea, strain D11_T_E12]
CGGAGGATGATATGGCAAAGTCTATGATTACCAAAGCATTTGTAGGCATGAGTAGTAAACTGTCGAATATGACTATGAATGATGTTTACAAGCC
CTACATCCATGTAAGAAATGTAGAATAGAGAGATCAGTAACTGGAACTAATATCGTTTGTAGGCTTTCAAGTTACTTACGCAGTTCAACCCAAT
CACTACAGCTATTGCCGAATCCCCACTATTTCAAATGGCTGTCTCAGCAAATACCATCGAAAAGTACACACTGCTAGGCCCTTTCTTCAGAATA
TCTCCTCTGCAACAGGAAGTTACCAGGGAATACTTCAGTGCGCCAAAGACGATAGATAGACGACACATTGCCACATCTCAAGATGCGTTACGAT
TAACCTTACAAACCCATCAAAAAGATTTACTTGATATCATCAACCACTTTGTTCGAGCAAGTCCAATCGCAAAAAGCAAAACCCTGGATTGGTT
CGCCTACATTGTGAATCAAAATCACAAACGTCGAGCACTTCAGGTAGACCCGAAAGAAGTGTCTTCTGATGGCTTTATGCACAATGTCACTGTC
GTTCTAGATGGTCTTTGTGAGCCATTCATGGATACCACATTCTCGAAAATTTCGAAGATTGATATTGATTATCTAAGACGTGCGCCTCGTGTAG
ATATCAAGGACGAGACCAAGTTGAACGCTGATGAGAAGGCTTCTGAGAAGTATTATGAGGACACTGTTCCTGGCACTTCTAATTTCATCTCTGA
GGTCTTCTTTCTGACATTGGCTGCTCATCATTATGGTAGTGAAGCTCTTAATGCCACGCATAAGAGTCTGGAGAAAGACATCAAATATATTCAA
AAGCAATTGACTGCCGTTGAAGCA

>Bcin01g07220(MLST1), partial sequence [organism=Botrytis cinerea, strain D11_T_E15]
ATGGCATATCCTGTTTCTTTTCGCATTATGCTTTGGAACGTGCTGGACAAGCTTGGGTTAGTGCTCTGCGTGTAGAAGCACTAAAGAAGATTCT
CGCACAACCGAAGTCATGGTTTGAGGAATCCAGAAATTCACCTGGCCGGTTGAACGAAGTTTTGGATAGGAACTCTGAGGAAATGCGTAATCTC
GTTGGCCGCTTTGCTGGTATTGCATTCACAGCGTTTTTTATGCTATTGATATCAATCATTTGGGCTTTCGTGAATACATGGAAACTGACATTAG
TCTCGATGGCAACTGGGCCAGTTATATACGCTGTCACCAAAACGTTCAATCGCGTGAGTGGAAAATGGAAAACAAGTGCAACTACGCCTCTGA
AATGACCACTGGCATATTTTCAGAGACTTTCTCCAACATCAAAGTGGTTCGGGCTTTTACTCTGGAAACTTACTTTGAGACAAAACACACCAAA
GCTACAGAAGAACTTTATAAAGTTGGACTAATACGAGCAAACTACTCGGGATTGCTGTGGGGATTGACAGATGCGATGTCATTCTTCATCACTG
CAACTATCTTTTATTATGCCACGGTTCTCATTACCAAGAGAGAGATCAGTATCGCGACTGCACTACAGACTGTCAATCTTCTATTATTTGGTAT
TTCTAATAGTACGAATATGCTGGCCATGATACCACAAATCAACTCTTCTCGCGTTACAGCTACGCATATGCTTGCATTAGCCAATCTCGATTCA
TCTTCCTCCCACGAAAATAAAGGAACCGAACGGCTTTCGACAATCTTTCCAATCAAATTCAACCGTCTCTCATTCACATATCCTACTCGTCCCG
AAAAACGAACGATATCATCCTTTTCTCTCTCCCTGATTCCTAACTCAACAACTGCACTTGTCGGACCCTCCGGCTCCGGAAAATCTACAATAGC
TGCTCTGCTCATTGGTCTCTATCCGCCAGATACTTCAACACCTCCACCGTTGACATCCAATCGCGTCTCCATAAGTAACTGTCACATCCCGTCT
CTCCGGGCTTCTCTCTCACTCGTTCCACAAATACCGATTCTATTTCCAGCTACCATTCTCCATAACATCATTTATGGTCTCCCAGAATCTTCTC
CTTGTGCTAGTCTTCCATCAGCTATTGCATCAGCAAAAGATGCTGGGATCCATGAATTTATCACATCGCTTCCACAAGGTTATGATACTAT

>Bcin05g07690(MLST2), partial sequence [organism=Botrytis cinerea, strain D11_T_E15]
ACCACTATCACCAAGTCCTTTTCTGCACTTTCCTCATCAAAAACGAACGAGAACATCATCAATAAATACTTTGCTGAAGTAATTGGGTTTTATT
TTGGCCAGGATGCCTTCTCTCTACGACAGCAAGCCTTCGATGATATGTTGTGGGTAGTTCTTGGCTGGCTGGATACTGTCAAATTCATTGATTT
ACATTCTGAATTGCACTATTCAAACGACTCTCAGCCAGAATGGTACGGACAACAATATAAGCCTGCATTTGCACATCGAGCGCGACTATTTTGG
GAATTGGCTTCACAAGGATGGGATACTACTCTCTGTGGTGGTGGGATGATATGGTCACCATACCTTACTCCATACAAGAATGCAATTACCAATG
AACTCTATATCGCAGCTTCGATATCGATGTACCTCTATTTCCCCGGAGATGACAATCAATCCCCATTTATGCTTTCCAACCCTTCATATCCACC
TCACGATCCGAAATATCTACAGGCAGCTGTTGATGCTTACAAATGGCTGAATGGTTCCAACATGACGGATTTACAAGGATTATATGTCGACGGG
TATCATATCTCGAATCTTTCTGGCGGTGAAAACACCCATTGCGATTCTAGAAATGAGATGGTATATACCTACAATCAAGGTGTTTTGCTTACTG
GACAACGTGGTTTGTATGACGCAACCGCCGCACGATCATACCTTGTGGATGGCCACAAACTCATCGCGAATGTTATTAATGCCACAGGCTATGA
CCTGAAACACAATGTTGTCATCTCACCGCCACCCAAAGATGGTTCCGCATTGGCAAAGTGGTTTGGCCTGGGTAGGAATGGAATACTGGAAGAA
GGATGCGATTCAAGTGCTTCGTGTTCTCAAAATGGACAAACTTTCAAAGGCATATTCTTTCATCACTTGATTGCATTCTGTAGTGATTTGCCAG
GGGAGCCTATTGCAGGGACGAAAGAAGGCCTAGAACTCGACAGAGTGTGGCATTCTGACAAATGCTCACAGTATACAAAAT

>Bcin06g01710(MLST3), partial sequence [organism=Botrytis cinerea, strain D11_T_E15]
GTGAGTCTGACTTTTGTATTTGAGCGTTAAGATGGACACTGATGTACCAAGGCAATCACCTAATTTCATGGCGTTCTTCCCTGCATCATCTACC
TACCCTGGAATGCTGGGAGAATTATACTCAGCAGCTTTCACAGCTCCTGCTTTCAATTGGATCTGTTCCCCTGCTGTGACAGAATTGGAGACGG
TTGTAATGGATTGGTTGGCCAAGCTTCTCAATCTCCCAGACTGTTATTTGTCGTCGACTCATGGTGGTGGTGTTATCCAAGGATCAGCCTCGGA
AGCTATCGTTACCGTTATGGTTGCTGCCCGCGATAAGTATCTTCGTGAAACCACTGAAGGTCTGTCGGGAATTGAACTCGAGGATGCGATTGCA
```

TATAAGAGGAGTAAGCTAGTTGCACTAGGAAGCGAAATGGCACACAGCTCCACGCAGAAAGCAGCGCAGATAGCTGGCGTTAGATTCCGATCGA
TTCCAGTACTCGCATCCAATGATTTCGCCATGACGGGTGATGATTTAGAGAAGGTATTGAAAGAATGCAAATCTCAAGGATTGGAACCCTTCTA
TCTAACTTCGACGTTGGGAACAACATCTACATGCGCAGTTGACGACTTCGCATCTATTGCAACAGTACTTTCAAAATATGCACCTCCAGATGTT
GCAGGCGAGATCTGGGTTCACGTCGATGCTGCTTATGCAGGTGCAGCTTTGGTTTGCCCTGAATACCATCATCTAACATCGTCCTTCCAGCATT
TCCATTCCTTTGATATGAACATGCACAAATGGCTTCTGACAAATTTCGACGCTTCTTGCCTATATGTCAAGAAACGCAAAGATCTGATCGATGC
ACTCTCCATAACACCAAGTTATCTTCGCAACGAGTTTTCAGAGAGTAGACTCGTAACCGACTATCGGGACTGGCAAATTCCTCTCGGAAGACGT
TTCCGAAGCTTAAAGATTTGGTTTGTCCTCAGAACCTACG

>Bcin09g03030(MLST4), partial sequence [organism=Botrytis cinerea, strain D11_T_E15]
ACCCTTCAACCCCACCGCAGCTCCTATACCGAAAGCGAGTATCCTACCAATTCTCCTTCCACCTGCGACTTTAAGACCATTGGCTTTTCGCACT
TTCACAAAAAAGCATAGTTTAACATTGACGTCGTCGGCATTACAAGTGTTGGCTACTTTTATTGGAAAGCATTGTGGGACAGGATGGAGGGAGG
AAGGACTGGCAGAGAGAGTCTTGGAGGAGGTCGCCAAGAGTTGGAAGAATAGGAGTGGCGGTGTCATTGTCGAGGGCGAGGGAACGGAATTGAA
GGAGATTCTGAAAGCTTTGGAAGGGAATATGAGTGGTGGAAGGATAGTCATAGGAAGAGAGCTAAGCCGGCAGAATAGTTTAGTACTGGGATCA
TCACAATATGGAGAGGTCAATCATACAAGACTTGGGCTACGGCCAGGGAATATACCCAGAGAGGATAGTCAGTCAAGTTTGGGAATGTCAACGT
TGGAGGTCAATGACGAGGAAGATGAGGATGGCCTGATGGATCCAAGAAGGTGGTTAAAAGTCGTTGATGCATTTGAGCAACCTCGACTGGTGTA
CAATGTTGCTAAAAAGCACTTTGATAGGTATGTTTCAATGATAAAATTTTATCTGAATCGACTAACTCAGTACAGAGATACCTCCAAACCTTCA
TTGTTCCCACCTGCGTCTCATAAAACACTCCTCTTCCAAAACCGCTATAATGTTATCCATCAACGTCTCCTTCGCAATGAATCTTTTCAAACGC
CCGCTTTTCAAGGTGGCAAATCTTCCCTTCAACGCAGCACGTCCGCCATTACCACCCAACAACAATCATACAAATTAACGCCGATAGCTAATCT
TCTCGGTCGCAATCGCAGCTCTCATATGCTTCTCGGTCTCCTCAGTATTTCACCCACTGGTACCCTCGCCATCAATGACCTGACGGGCAGTATC
GCTCTTGATCTTACACACGCAGCAGCCATTC

>Bcin11g01310(MLST5), partial sequence [organism=Botrytis cinerea, strain D11_T_E15]
ACTGACATGGACCTCATGTGGAACCGGCGTAGAATGCGCAACGCTTGAAGTTCCGCTCGAATATGGCGATGCAACGTCAACGGCAAAAGCCAGT
GTTGCGCTTGCTCGTTATCCTGCCACTGTTGCCGCGAGCAAGAAGCTCGGGTCTCTCTTGATAAATCCCGGTGGACCCGGTGCCTCTGGTGTTG
GCTTTGTGCAGTCTGGAGCCGGTGCCGCCGTCTCGACACTGAGTGGTGGATTATACGATATCATCGGATGGGATCCACGTGGAACCGGTGCTTC
GGCTCCTATTTTGGAATGTTTTGCAAATGCCAGTGCGGAGTATGATTTTAACAACGCGTTTCCATCTGCTCCGAATCTCTGGCTCGGACAATTT
GCGAATGCCAGCGCAAATTCTGCTGTTAGCTCTGCTATCACATCCTTTGACACTTCTGTCGCTGCTCTTGCAAAAGCTTGCGTGGCTCAGAAAT
CTCCCGCTCTTTACACCTCAACAGCAGCATATGTTGCTCGAGACATGGCAGCGATAGTCGATGCATTGGATGGGACCTCTGCAAAACTTAACTA
CTGGGGTTTCTCATATGGAACTATCTTCCTAGCTGAGTTTATCCAAACTTTCCCAGGCCGCGTGGGAAGAGTTCTTGCCGATGGTGTTTTCGAC
GCAAAGGCAAATGCACTCACATACGTTAGCCAACTTCCCAACGATCAACTCAGTGTTCGTGCTTCGTTGAACGATTTTGCAGCTTTCTGCACCA
CCGCCGGTAGTAAAGGTTGCTCTTTTGCCACCGCCCCTACTGGAACCTCAGGTACTGTTGCTACCAGACTGGACAACATAATGAAGGATATGTT
CCTCAATCCTATTGTTGCTTCGGGCTTGAGCATCA

>Bcin15g03910(MLST6), partial sequence [organism=Botrytis cinerea, strain D11_T_E15]
CGCCAAAACACAAAATCATCCAACGATGAAGATGATACTCCACTTCCCTTGATTATCTGGCATGGACTCGGCGATAATTACAAAGCGGATGGTC
TTGCGCAAGTTGGAAAACTAGCTGAAGCTATTCATCCTGGGACTTTTGTCTATAATATTCATGTAGATGAGGATGCATCTGCAGATAGGACAGC
TACCTTCTTTGGAAATCTCACTCGTGATACATCCCCTATTTTTCCTTTAAATACCATACTAACTCTCTTACCAAGTTCAAATCGAAAAGGTCTG
CGAAGACCTCGCCTCCCATCCTATTCCCTCTACCGCGCCCGCCGTCGACGCAATTGGATTCTCCCAAGGCGGCCAATTCTTGCGCGGTTACATA
TCCCGCTGCAATGCTCCACCCATCCGCTCTCTCCTGACCTTCGGTTCCCAACACAACGGCATTTCTGCCTTCCAATCCTGTGGTCCTGCCGATT
TCCTCTGTCGCGGTGCTCAAACCCTTTTGCGATCCAACACCTGGTCAACCTTTGTCCAATCTCGTCTCGTACCCGCTCAATACTTCAGAGATCC
GGAAAACCTAGACTCTTACCTTGAATATTCCAATTTCCTTGCCGACATCAATAATGAGCGCGTTCTCAAGAACCAAACATATAAATCCAACATG
GAAAAATTGGAACGATTCGTAATGTATGTCTTTGAAGACGATACAACTGTCATCCCTAAGGAAAGTGGATGGTGGGCTGAAGTCAACGGCACGG
AAGTTACACCACTGAAAGAAAGAGCCATTTATAAAGAAGATTGGCTAGGGTTAAAGACATTGGATGAGGCCGGAAAATTAGTTTTCGAAACCAT
TCCAGGGGGACATATGACGTTAGGAGAGGAGATGCTAGAGAAGGCTTTCAAAGAGTACTTTGGTCCAGCAGGGAAGAAATTTGGG

>Bcin16g03460(MLST7), partial sequence [organism=Botrytis cinerea, strain D11_T_E15]
ATGAACCTCTTAATTTGAACTCTTCATTAATTGCAAGTGATGAACCCCTATTCTGCCAGAGGATAGTTACAAGACGTATATCATTAGTCGAGAA
CCACTCATGATATACATTGACGGATTTTTGAAAGCGAATGAAGTAAACACTTGGTTGATGTTAGGTGTGTTATTTATTCTGATGAAATGAACAA
GAGAGACTGATGAGATAGTGAACCGCTTTATGAACCGTCTACTGTTTCTCACGGACAGGAAGTTACCATTGATCCTTCGGTTCGAAATTCTGAA
GTGGCGGTTTTAGAGAGGGATGAGGTGGTCAGGTGTATTGAGCATAGAGCGAGGGCATTTCAGGGGTGGAGGGCGAGATGGGGATTGAGAAGTT
GAGGACGCAGAGGTATGGGGTTGGAGGACATTATGGGATGCATTTGTAAGTTTTGGGGATTGACGAAGGCCTCTGTTTATTTTCTATCAGTACG
GATCTTGAAAGAAAGATAGCATGAACACGAGGGCTAATAACTAGGAAATAGCGATTGGAGCGGAGGTAAACGTGGCATAGACCGATTTAGTACT
TTCATGGTCTATGTCGACGTATCCTCTGATATCGAAGGTGGAGGAACGGAATTCCCACGTATTGTGGGACCAAAAGGAGGAAGGTGGGAGGACT
TCCTGGAAACTACGGAAGCATTGGATCCAAGAACTGGAGAAAATGTAACAGTAGAAGGGGTGACATTCAAACCAATCAAGGAAATGCCGTATTC
TGGGAAAATACTGACAACAACGGGAGGGGCTATGAT

>Bcin12g03020(MLST8), partial sequence [organism=Botrytis cinerea, strain D11_T_E15]

CGATTGGCTGCGAAGAAAACTGCGCAGCACCGAGTCACCAAAAGCACGAATAAACCATCGGCAAGGGAAATATTCTCTGAACCACAACATGACA
CGAGCGCGGAAGAGTATATCGGGCGAGAAGCTTCATCAAGAGCACCAAAGCGACAACGAGTCGATGATAATTATAACTCTTACGGTGGAAGAAA
TGAGAATACAGCAGCTTATGCTTCCGGGAAACTTCCATCTGGAAGTATAAATGTTGGTGGAGGTAGGAAGACCACTTTTCAAGAAGAACCTCGA
ACGGCATTTGTCGCTGGCAAGTTGCCCCCTGGAAGTATCAACATTGGTGGAAAGAAGGCTACCCAAATCGAGGACGAGGGTAGAGCAGCTTATG
CTTCCGGAAAATTGCCCCCAGGAAGTATAAACTCTGGCGCAAAGAAGGCGATTTCATTCCAAGATGAAACGAGACCGGCTTATGTCTCTGGAAA
GCTTCCACATGGTAGTATCGACGGTATGCGAAACCGTGAAATGGCTGCCGTCCACCGCGAAATTGCTGAGGGTGGAGGAAACCAGGCCAGGTT
GTCTCTTCTCTATTCACATTCAATCCTACTTCAAAGAAAACTTTCGATGAACCCGAAGAACAAGCGGAACCGGCAAAACCATCCAATGCACCTT
TGACAAAGGAAATGGCGACTTTCACCAATCTAGGGTTATCGAGAAGGCTTGCAGCCCATCTATCGACTAAACTCGATATGAAAGCCCCGACCGC
CATTCAAAAAGCATCTGTGCAGCAGTTGGTATCGGACGATAGCGATGCTTTCATACAAGCAAAGACTGGATCTGGAAAAACTTTGGCATATCTA
CTACCTATAGTCGAGCGAATATTAGCATTGAGTGAGAATGGCGTACAAA

>Bcin02g07770(MLST9), partial sequence [organism=Botrytis cinerea, strain D11_T_E15]
CAGCTTTCCCTTTCGGTCTTAGCATCTACAGTCATTGCCATCCCTACACCATCACAACTTGAGTCTCGGGCCGTTATCGATTCCGATGCCGTTG
TAGGATTTGCCGAAACTGTTCCCAGTGGGACCGTAGGAACAGTCTATGAGGCATATAAACCATTCCTTAAAGTCGTAAATGGATGCGTACCATT
CCCTGCCGTCGATGCATCGGGTAACACAGGGTATGTCCTTATACCATTCTCTTCCACACGATTGCTATTGAGTCTCTAACATATTTTAGTGGTG
GTTTGTCACCAACTGGCAGTAGCAATGGTGGTTGCAGCAGCAGTACCGGTCAAGTATATGTTCGAGGAGGACAAAGCGGATCAAACTACGCCAT
CATGTACTCCTGGTAAGTTCTCTCTAAACTTCTCCTTATAGATCCAACCTAACAAAATCTTAGGTACATGCCAAAGGACGAGCCCTCAACCGGT
ATTGGTCACCGTCACGATTGGGAAGGTGTAATTGTCTGGCTCTCAAGCGCCACCGCCACAACTGCCGACAACATCTTAGCCGTTTGTCCTTCCG
CCCACGGAGGCTGGGATTGTTCCACCGATGGATATTCCCTTTCTGGTACCAGCCCTCTTATCAAGTACGAAAGTATCTGGCCCGTCGATCATTC
AATGGGTCTTACTAGTACTGTTGGTGGAAAACAACCTATGATTGCTTGGGAGTCTTTACCAACTGCTGCTCAAACTGCTCTTGAGAACACCGAT
TTCGGTGCTGCGAATGTTCCATTCATTCCGGCTGTTTTCACAGATAATCT

>Bcin04g02090(MLST10), partial sequence [organism=Botrytis cinerea, strain D11_T_E15]
CGGAGGATGATATGGCAAAGTCTATGATTACCAAAGCATTTGTAGGCATGAGTAGTAAACTGTCGAATATGACTATGAATGATGTTTACAAGCC
CTACATCCATGTAAGAAATGTAGAATAGAGAGATCAGTAACTGGAACTAATATCGTTTGTAGGCTTTCAAGTTACTTACGCAGTTCAACCCAAT
CACTACAGCTATTGCCGAATCCCCACTATTTCAAATGGCTGTCTCAGCAAATACCATCGAAAAGTACACACTGCTAGGCCCTTTCTTCAGAATA
TCTCCTCTGCAACAGGAAGTTACCAGGGAATACTTCAGTGCGCCAAAGACGATAGATAGACGACACATTGCCACATCTCAAGATGCGTTACGAT
TAACCTTACAAACCCATCAAAAAGATTTACTTGATATCATCAACCACTTTGTTCGAGCAAGTCCAATCGCAAAAAGCAAAACCCTGGATTGGTT
CGCCTACATTGTGAATCAAAATCACAAACGTCGAGCACTTCAGGTAGACCCGAAAGAAGTGTCTTCTGATGGCTTTATGCACAATGTCACTGTC
GTTCTAGATGGTCTTTGTGAGCCATTCATGGATACCACATTCTCGAAAATTTCGAAGATTGATATTGATTATCTAAGACGTGCGCCTCGTGTAG
ATATCAAGGACGAGACCAAGTTGAACGCTGATGAGAAGGCTTCTGAGAAGTATTATGAGGACACTGTTCCTGGCACTTCTAATTTCATCTCTGA
GGTCTTCTTTCTGACATTGGCTGCTCATCATTATGGTAGTGAAGCTCTTAATGCCACGCATAAGAGTCTGGAGAAAGACATCAAATATATTCAA
AAGCAATTGACTGCCGTTGAAGCA

>Bcin01g07220(MLST1), partial sequence [organism=Botrytis cinerea, strain D11_T_E18]
ATGGCATATCTTGTTTCTTTTCGCATTATGCTTGGAACGTGCTGGACAAGCTTGGGTTAGTGCTCTGCGTGTAGAAGCACTAAAGAAGATTCTC
GCACAACCGAAGTCATGGTTTGAGGAATCCAGAAATTCACCTGGCCGGTTGAACGAAGTTTTGGATAGGAACTCTGAGGAAATGCGTAATCTCG
TTGGCCGCTTTGCTGGTATTGTATTCACAGCGTTTTTTATGCTATTGATATCAATCATTTGGGCTTTCGTGAATACATGGAAACTGACATTAGT
CTCGATGGCAACTGGGCCAGTTATATACGCTGTCACCAAAACGTTCAATCGCGTGAGTGGAAAATGGGAAAACAAGTGCAACTACGCCTCTGAA
ATGACCACTGGCATATTTTCAGAGACTTTCTCCAACATCAAAGTGGTTCGGGCTTTTACTCTGGAAACTTACTTTGAGACAAAACACACCAAAG
CTACAGAAGAACTTTATAAAGTTGGACTAATACGAGCAAACTACTCGGGATTGCTGTGGGGATTGACAGATGCGATGTCATTCTTCATCACTGC
AACTATCTTTTATTATGCCACGGTTCCATTACCAAGAGAGAGATCAGTATCGCGACTGCACTACAGACTGTCAATCTTCTATTATTTGGTATTT
CTAATAGTACGAATATGCTGGCCATGATACCACAAATCAACTCTTCTCGCGTTACAGCTACGCATATGCTTGCATTAGCCAATCTCGATTCATC
TTCCTCCCACGAAAATAAAGGAACCGAACGGCTTTCGACAATCTTTCCAATCAAATTCAACCGTCTCTCATTCACATATCCTACTCGTCCCGAA
AAACGAACGATATCATCCTTTTCTCTTTCCCTGATTCCTAACTCAACAACTGCACTTGTCGGACCCTCCGGCTCCGGAAAGTCTACAATAGCTG
CTCTGCTCATTGGTCTCTATCCGCCAGATACTTCAACACCTCCACCGTTGACATTCAATCGCGTCTCCATAAGTAACTGTCACATTCCGTCTCT
CCGGGCTTCTCTCTCACTCGTTCCACAAATACCGATTCTATTTCCAGCTACCATTCTCCATAACATCATTTATGGTCTCCCAGAATCTTCTCCT
TGTGCTAGTCTTCCATCAGCTACTGCATCAGCAAAAGATGCTGGGATCCATGAATTTATCACATCGCTTCCACAAGGTTATGATACTAT

>Bcin05g07690(MLST2), partial sequence [organism=Botrytis cinerea, strain D11_T_E18]
CACCACTATCACCAAGTCCTTCTCTGCACTTTCCTCATCAAAAACGAACGAGAACATCATCAATAAATACTTTGCTGAAGTAATTGGGTTTTAT
TTTGGCCAGGATGCCTTCTCTCTACGACAGCAAGCCTTCGATGATATGTTGTGGGTAGTTCTTGGCTGGCTGGATACTGTCAAATTCATTGATT
TACATTCTGAATTGCACTATTCAAACGACTCTCAGCCAGAATGGTACGGACAACAATATAAACCTGCATTTGCACATCGAGCGCGACTATTTTG
GGAATTGGCTTCACAAGGATGGGATACTACTCTCTGTGGTGGTGGGATGATATGGTCACCATACCTTACTCCATACAAGAATGCAATTACCAAT
GAACTCTATATCGCAGCTTCGATATCGATGTACCTCTATTTCCCCGGAGATGACAATCAATCCCCATTTATGCTTTCCAACCCTTCATATCCAC
CTCACGATCCGAAATATCTACAGGCAGCTGTTGATGCTTACAAATGGCTGAATGGTTCCAACATGACGGATTTACAAGGATTATATGTCGACGG
GTATCATATCTCGAATCTTTCTGGCGGTGAAAACACCCATTGCGATTCTAGAAATGAGATGGTATATACCTACAATCAAGGTGTTTTGCTTACT

GGACAACGTGGTTTGTATGACGCAACCGCCGCACGATCATACCTTGTGGATGGCCACAAACTCATCGCGAATGTTATTAATGCCACAGGCTATG
ACCTGAAACACAATGTTGCCATCTCACCGCCACCCAAAGATGGTTCCGCATTGGCAAAGTGGTTTGGCCTGGGTAGGAATGGAATACTGGAAGA
AGGATGCGATTCAAGTGCTTCGTGTTCTCAAAATGGACAAACTTTCAAAGGCATATTCTTTCACCACTTGATTGCGTTCTGTAGTGATTTGCCA
GGGGAGCCTATTGCAGGGACGAAGGAAGGCCTAGAACTCGACAGAGTGTGGCATTCTGACAAATGCTCACAGTATACAAAAT

>Bcin06g01710(MLST3), partial sequence [organism=Botrytis cinerea, strain D11_T_E18]
GTGAGTCTGACTTTTGTATTTGAGCGTTAAGATGGACACTGATGTACCAAGGCAATCACCTAATTTCATGGCGTTCTTCCCTGCATCATCTACC
TACCCTGGAATGCTGGGAGAATTATACTCAGCAGCTTTCACAGCTCCTGCTTTCAATTGGATCTGTTCCCCTGCTGTGACAGAATTGGAGACGG
TTGTAATGGATTGGTTGGCCAAGCTTCTCAATCTCCCAGACTGTTATTTGTCGTCGACTCATGGTGGTGGTGTTATCCAAGGATCAGCCTCGGA
AGCTATCGTTACCGTTATGGTTGCTGCCCGCGATAAGTATCTTCGTGAAACCACTGAAGGTCTGTCGGGAATTGAACTCGAGGATGCGATTGCA
TATAAGAGGAGTAAGCTAGTTGCACTAGGAAGCGAAATGGCACACAGCTCCACGCAGAAAGCAGCGCAGATAGCTGGCGTTAGATTCCGATCGA
TTCCAGTACTCGCATCCAATGATTTCGCCATGACGGGTGATGATTTAGAGAAGGTATTGAAAGAATGCAAATCTCAAGGATTGGAACCCTTCTA
TCTAACTTCGACGTTGGGAACAACATCTACATGCGCAGTTGACGACTTCGCATCTATTGCAACAGTACTTTCAAAATATGCACCTCCAGATGTT
GCAGGCGAGATCTGGGTTCACGTCGATGCTGCTTATGCAGGTGCAGCTTTGGTTTGCCCTGAATACCATCATCTAACATCGTCCTTCCAGCATT
TCCATTCCTTTGATATGAACATGCACAAATGGCTTCTGACAAATTTCGACGCTTCTTGCCTATATGTCAAGAAACGCAAAGATCTGATCGATGC
ACTCTCCATAACACCAAGTTATCTTCGCAACGAGTTTTCAGAGAGTGGACTCGTAACCGACTATCGGGACTGGCAAATTCCTCTCGGAAGACGT
TTCCGAAGCTTAAAGATTTGGTTTGTCCTCAGAACCTAC

>Bcin09g03030(MLST4), partial sequence [organism=Botrytis cinerea, strain D11_T_E18]
GACCCTTCAACCCCACCGCAGCTCCTATACCGAAAGCGAGTATCCTACCAATTCTCCTTCCACCTGCGACTTTAAGACCATTGGCTTTTCGCAC
TTTCACAAAAAAGCATAGTTTAACATTGACGTCGTCGGCATTACAAGTGTTGGCTACTTTTATTGGAAAGCATTGTGGGACAGGATGGAGGGAG
GAAGGACTGGCAGAGAGAGTCTTGGAGGAGGTCGCCAAGAGTTGGAAGAATAGGAGTGGCGGTGTCATTGTCGAGGGCGAGGGAACGGAATTGA
AGGAGATTCTGAAAGCTTTGGAAGGGAATATGAGTGGTGGAAGGATAGTCATAGGAAGAGAGCTAAGCCGGCAGAATAGTTTAGTACTGGGATC
ATCACAATATGGAGAGGTCAATCATACAAGACTTGGGCTACGGCCAGGGAATATACCCAGAGAGGATAGTCAGTCAAGTTTGGGAATGTCAACG
TTGGAGGTCAATGACGAGGAAGATGAGGATGGCCTGATGGATCCAAGAAGGTGGTTAAAAGTCGTTGATGCATTTGAGCAACCTCGACTGGTGT
ACAATGTTGCTAAAAAGCACTTTGATAGGTATGTTTCAATGATAAAATTTTATCTGAATCGACTAACTCAGTACAGAGATACCTCCAAACCTTC
ATTGTTCCCACCTGCGTCTCATAAAACACTCCTCTTCCAAAACCGCTATAATGTTATCCATCAACGTCTCCTTCGCAATGAATCTTTTCAAACG
CCCGCTTTTCAAGGTGGCAAATCTTCCCTTCAACGCAGCACGTCCGCCATTACCACCCAACAACAATCATACAAATTAACGCCGATAGCTAATC
TTCTCGGTCGCAATCGCAGCTCTCATATGCTTCTCGGTCTCCTCAGTATTTCACCCACTGGTACCCTCGCCATCAATGACCTGACGGGCAGTAT
CGCTCTTGATCTTACACACGCAGCAGCCATTC

>Bcin11g01310(MLST5), partial sequence [organism=Botrytis cinerea, strain D11_T_E18]
ACTGACATGGACCTCATGTGGAACCGGCGTAGAATGCGCAACGCTTGAAGTTCCGCTCGAATATGGCGATGCAACGTCAACGGCAAAAGCCAGT
GTTGCGCTTGCTCGTTATCCTGCCACTTTTGCCGCGAGCAAGAAGCTCGGGTCTCTCTTGATAAATCCCGGTGGACCCGGTGCCTCTGGTGTTG
GCTTTGTGCAGTCTGGAGCTGGTGCCGCCGTCTCGACACTGAGTGGTGGATTATACGATATCATCGGATGGGATCCACGTGGAACCGGTGCTTC
GGCTCCTATTTTGGAATGTTTTGCAAATGCCAGTGCGGAGTATGATTTTAACAACGCGTTTCCATCTGCTCCGAATCTCTGGCTCGGACAATTT
GCGAATGCCAGCGCAAATTCTGCTGTTAGCTCTGCTATCACATCCTTTGACACTTCTGTCGCTGCTCTTGCAAAAGCTTGCGTGGCTCAGAAAT
CTCCCGCTCTTTACACCTCAACAGCAGCATATGTTGCTCGAGACATGGCAGCGATAGTCGATGCATTGGATGGGACCTCTGCAAAACTTAACTA
CTGGGGTTTCTCATATGGAACTATCTTCCTAGCTGAGTTTATCCAAACTTTCCCAGGCCGCGTGGGAAGAGTTCTTGCCGATGGTGTTTTCGAC
GCAAAGGCAAATGCACTCACATACGTTAGCCAACTTCCCAACGATCAACTCAGTGTTCGTGCTTCGTTGAACGATTTTGCAGCTTTCTGCACCA
CCGCCGGTAGTAAAGGTTGCTCTTTTGCCACCGCCCCTACTGGAACCTCAGGTACTGTTGCTACCAGACTGGACAACATAATGAAGGATATGTT
CCTCAATCCTATTGTTGCTTCGGGCTTGAGCATCA

>Bcin15g03910(MLST6), partial sequence [organism=Botrytis cinerea, strain D11_T_E18]
GCCAAAACACAAAATCATCCAACGATGAAGATGATACTCCACTTCCCTTGATTATCTGGCATGGACTCGGCGATAATTACAAAGCGGATGGTCT
TGCGCAAGTTGGAAAACTAGCTGAAGCTATTCATCCTGGGACTTTTGTCTATAATATTCATGTAGATGAGGATGCATCTGCAGATAGGACAGCT
ACCTTCTTTGGAAATCTCACTCGTGAGTACATCCCCTATTTTTCCTTTAAATACCATACTAACTCTCTTACCAAGTTCAAATCGAAAAGGTCTG
CGAAGACCTCGCCTCCCATCCTATTCTCTCTACCGCGCCCGCCGTCGACGCAATTGGATTCTCCCAAGGCGGCCAATTCTTGCGCGGTTACATA
TCCCGCTGCAATGCTCCACCCATCCGCTCTCTCCTGACCTTCGGTTCCCAACACAACGGCATTTCTGCCTTCCAATCCTGTGGTCCTGCCGATT
TCCTCTGTCGCGGTGCTCAAACCCTTTTGCGATCCAACACCTGGTCAACCTTTGTCCAATCTCGTCTCGTACCCGCTCAATACTTCAGAGATCC
GGAAAACCTAGACTCTTACCTTGAATATTCCAATTTCCTTGCCGACATCAATAATGAGCGCGTTCTCAAGAACCAAACATATAAATCCAACATG
GAAAAATTGGAACGATTCGTAATGTATGTCTTTGAAGACGATACAACTGTCATCCCTAAGGAAAGTGGATGGTGGGCTGAAGTCAACGGCACGG
AAGTTACACCACTGAAAGAAAGAGCCATTTATAAAGAAGATTGGCTAGGGTTAAAGACATTGGATGAGGCCGGAAAATTAGTTTTCGAAACCAT
TCCAGGGGGACATATGACGTTAGGAGAGGAGATGCTAGAGAAGGCTTTCAAAGAGTACTTTGGTCCAGCAGGGAAGAAATTTGGGG

>Bcin16g03460(MLST7), partial sequence [organism=Botrytis cinerea, strain D11_T_E18]
ATGAACCTCTTAATTTGAACTCTTCATTAATTGCAAGTGATGAGCCCCTATTCTGCCCAGAGGATAGTTACAAGACGTATATCATTAGTCGAGA

ACCACTCATGATATACATTGACGGATTTTTGAAAGCGAATGAAAGTAAACACTTGGTTGATGTTAGGTGTGTTATTTATTCTGATGAAATGAAC
AAGAGAGACTGATGAGATAGTGAACCGCTTTATGAACCGTCTACTGTTTCTCACGGACAGGAAGTTACCATTGATCCTTCGGTTCGAAATTCTG
AAGTGGCGGTTTTAGAGAGGGATGAGGTGGTCAGGTGTATTGAGCATAGAGCGAGGGCATTTCAGGGGTGGAGGGACGAGATGGGGATTGAGAA
GCTGAGGACGCAGAGGTATGGGGTTGGAGGACATTATGGGATGCATTTGTAAGTTTTGGGGGATTGACGAAGGCCTCTGTCTATTTTCTATCAG
TACGGATCTTGAAAGAAAGATAGCATGAACACGAGGGCTAATAACTAGGAAATAGCGATTGGAGCGGAGGTAAACGTGGCATAGACCGATTTAG
TACTTTCATGGTCTATGTCGACGTATCCTCTGATATCGAAGGTGGAGGAACGGAATTCCCACGTATTGTGGGACCAAAAGGAGGAAGGTGGGAG
GACTTCCTGGAAACTACGGAAGCATTGGATCCAAGAACTGGAGAAAATGTAACAGTAGAAGGGGTGACATTCAAACCAATCAAGGGAAATGCCG
TATTCTGGGAAAATACTGACAACAACGGGAGGGGCTATGAT

>Bcin12g03020(MLST8), partial sequence [organism=Botrytis cinerea, strain D11_T_E18]
CGATTGGCTGCGAAGAAAACTGCGCAGCACCGAGTCACCAAAAGCACGAATAAACCATCGGCAAGGGAAATATTCTCTGAACCACAACATGACA
CGAGCGCGGAAGAGTATATCGGGCGAGAAGCTTCATCAAGAGCACCAAAGCGACAACGAGTCGATGATAATTATAACTCTTACGGTGGAAGAAA
TGAGAATACAGCAGCTTATGCTTCCGGGAAACTTCCATCTGGAAGTATAAATGTTGGTGGAGGTAGGAAGACCACTTTTCAAGAAGAACCTCGA
ACGGCATTTGTCGCTGGCAAGTTGCCCCCTGGAAGTATCAACATTGGTGGAAAGAAGGCTACCCAAATCGAGGACGAGGGTAGAGCAGCTTATG
CTTCCGGAAAATTGCCCCCAGGAAGTATAAACTCTGGCGCAAAGAAGGCGATTTCATTCCAAGATGAAACGAGACCGGCTTATGTCTCTGGAAA
GCTTCCACATGGTAGTATCGACGGTATGCGAAACCGTGAAATGGCTGCCGTCCACCGCGAAATTGCTGAGGGTGGGAGGAAACCAGGCCAGGTT
GTCTCTTCTCTATTCACATTCAATCCTACTTCAAAGAAAACTTTCGATGAACCCGAAGAACAAGCGGAACCGGCAAAACCATCCAATGCACCTT
TGACAGAGGAAATGGCGACTTTCACCAATCTAGGGTTATCGAGAAGGCTTGCAGCCCATCTATCGACTAAACTCGATATGAAAGCCCCGACCGC
CATTCAAAAAGCATCTGTGCAGCAGTTGGTATCGGACGATAGCGATGCTTTCATACAAGCAGAGACTGGATCTGGAAAAACTTTGGCATATCTA
CTACCTATAGTCGAGCGAATATTAGCATTGAGTGAGAATGGCGTACAAA

>Bcin02g07770(MLST9), partial sequence [organism=Botrytis cinerea, strain D11_T_E18]
CAGCTTTCCCTTTCGGTCTTGGCATCTACAGTCATTGCCATCCCTACACCATCACAACTTGAGTCTCGGGCCGTTATCGATTCCGATGCCGTTG
TAGGATTTGCCGAAACTGTTCCCAGTGGGACCGTAGGAACAGTTTACGAGGCATATAAACCATTCCTTAAAGTCGTAAATGGATGCGTACCATT
CCCTGCCGTCGATGCATCGGGTAACACAGGGTATGTCCTTATACCATTCTCTTCCACACGATTGCTATTGAGTCTCTAACATATTTTAGTGGTG
GTTTGTCACCAACTGGCAGTAGCAATGGTGGTTGCAGCAGCAGTACCGGTCAAGTATATGTTCGAGGAGGACAAAGCGGATCAAACTACGCCAT
CATGTACTCCTGGTAAGTTCTCTCTAAACTTCTCCTTATAGATCCAACCTAACAAAATCTTAGGTACATGCCAAAGGACGAGCCCTCAACCGGT
ATTGGTCACCGTCACGATTGGGAAGGTGTAATTGTCTGGCTCTCAAGCGCCACCGCCACAACTGCCGACAACATCTTAGCCGTTTGTCCTTCCG
CCCACGGAGGCTGGGATTGTTCCACCGATGGCTATTCCCTTTCTGGTACCAGCCCTCTTATCAAGTACGAAAGTATCTGGCCCGTCGATCATTC
AATGGGTCTTACTAGTACTGTTGGTGGAAAACAACCTATGATTGCTTGGGAGTCTTTACCAACTGCTGCTCAAACTGCTCTTGAGAACACCGAT
TTCGGTGCTGCGAATGTTCCATTCATTCCGGCTGTTTTCACAGATAATCT

>Bcin04g02090(MLST10), partial sequence [organism=Botrytis cinerea, strain D11_T_E18]
CGGAGGATGATATGGCAAAGTCTATGATTACCAAAGCATTTGTAGGCATGAGTAGTAAACTGTCGAATATGACTATGAATGATGTTTACAAGCC
CTACATCCATGTAAGAAATGTAGAATAGAGAGATCAGTAACTGGAACTAATATCGTTTGTAGGCTTTCAAGTTACTTACGCAGTTCAACCCAAT
CACTACAGCTATTGCCGAATCCCCACTATTTCAAATGGCTGTCTCAGCAAATACCATCGAAAAGTACACACTGCTAGGCCCTTTCTTCAGAATA
TCTCCTCTGCAACAGGAAGTTACCAGGGAATACTTCAGTGCGCCAAAGACGATAGATAGACGACACATTGCCACATCTCAAGATGCGTTACGAT
TAACCTTACAAACCCATCAAAAAGATTTACTTGATATCATCAACCACTTTGTTCGAGCAAGTCCAATCGCAAAAAGCAAAACCCTGGATTGGTT
CGCCTACATTGTGAATCAAAATCACAAACGTCGAGCACTTCAGGTAGACCCGAAAGAAGTGTCTTCTGATGGCTTTATGCACAATGTCACTGTC
GTTCTAGATGGTCTTTGTGAGCCATTCATGGATACCACATTCTCGAAAATTTCGAAGATTGATATTGATTATCTAAGACGTGCGCCTCGTGTAG
ATATCAAGGACGAGACCAAGTTGAACGCTGATGAGAAGGCTTCTGAGAAGTATTATGAGGACACTGTTCCTGGCACTTCTAATTTCATCTCTGA
GGTCTTCTTTCTGACATTGGCTGCTCATCATTATGGTAGTGAAGCTCTTAATGCCACGCATAAGAGTCTGGAGAAAGACATCAAATATATTCAA
AAGCAATTGACTGCCGTTGAAGCA

>Bcin01g07220(MLST1), partial sequence [organism=Botrytis cinerea, strain D11_T_WF43]
ATGGCATATCTTGTTTCTTTTCGCATTATGCTTTGGAACGTGCTGGACAAGCTTGGGTTAGTGCTCTGCGTGTAGAAGCACTAAAGAAGATTCT
CGCACAACCGAAGTCATGGTTTGAGGAATCCAGGAATTCACCTGGCCGGTTGAACGAAGTTTTGGATAGGAACTCTGAGGAAATGCGTAATCTC
GTTGGCCGCTTTGCTGGTATTGTATTCACAGCATTTTTTATGCTATTGATATCAATCATTTGGGCTTTCGTGAATACATGGAAACTGACATTAG
TCTCAATGGCGACTGGGCCAGTTATATACGCTGTCACCAAAACGTTCAATCGCGTGAGTGGAAAATGGGAAAACAAGTGCAACTACGCATCTGA
AATGACCACTGGCATATTTTCAGAGACTTTCTCCAACATCAAAGTGGTTCGGGCTTTTACTCTGGAAACTTACTTTGAGACAAAACACACCAAA
GCTACAGAAGAACTCTATAAAATTGGACTAATACGAGCAAACTACTCGGGATTGCTGTGGGGATTGACAGATGCGATGTCATTCTTCATCACTG
CAACTATCTTTTATTATGCCACGGTTCTCATTACCAAGAGAGAGATCAGTATCGCGACTGCACTGCAGACTGTCAATCTTCTATTATTTGGTAT
TTCTAATAGTACGAATATGCTGGCCATGATACCACAAATCAACTCTTCTCGCGTTACAGCTACGCATATGCTTGCATTAGCCAATCTCGATTCA
TCTTCCTCCCACGAAAATAAAGGAGCCGAACGGCTTTCGACAATCTTTCCAATCAAATTCAACAGTCTCTCATTCACATATCCTACTCGTCCTG
AAAAACGAACGATATCATCCTTTTCTCTTTCCCTGATTCCTAACTCAACAACTGCACTTGTCGGACCCTCCGGCTCCGGAAAATCTACAATAGC
TGCTCTGCTCGTTGGTCTCTATCCGCCAGATACTTCAACACCTCCACCGTTGACATTCAATCGTGTCTCCATAAGTAACTGTCACATTCCGTCT
CTCCGGGCTTCTCTCTCACTCGTCCCACAAATACCGATTCTATTTCCAGCTACCATTCTCCATAACATCATTTATGGTCTCCCAGAATCTTCTC

CTTGTGCTAGTCTTCCATCTGCTATTGCATCAGCAGAAGATGCTGGGATCCACGAATTTATCACATCGCTTCCACAAGGTTATGATACTAT

>Bcin05g07690(MLST2), partial sequence [organism=Botrytis cinerea, strain D11_T_WF43]
ACCACTATCACCAAGTCCTTTTCTGCACTTTCCTCATCAAAAACGAACGAGAACATCATCAATAAATACTTTGCTGAAGTAATTGGGTTTTATT
TTGGCCAGGATGCCTTCTCTCTACGACAGCAAGCCTTCGATGATATGTTGTGGGTAGTTCTTGGCTGGCTGGATACTGTCAAATTCATTGATTT
ACATTCTGAATTGCACTATTCAAACGACTCTCAGCCAGAATGGTACGGACAACAATATAAACCTGCATTTGCACATCGAGCGCGACTATTTTGG
GAATTGGCTTCACAAGGATGGGATACTACTCTCTGTGGTGGTGGGATGATATGGTCACCATACCTTACTCCATACAAGAACGCAATTACCAATG
AACTCTATATCGCAGCTTCGATATCGATGTACCTATATTTCCCCGGAGATGACAATCAATCCCCATTTATGATTTCCAACCCTTCATATCCACC
TCACGATCCGAAATATCTACAGGCAGCTGTTGATGCTTACAAATGGCTGAATGGTTCCAACATGACGGATTCACAAGGATTATATGTCGACGGG
TACCATATCTCGAATCTTTCTGGCGGTGAAAACACCCATTGCGATTCTAGAAATGAGATGGTATATACCTACAATCAAGGTGTTTTGCTTACTG
GACAACGTGGTTTGTATGACGCAACCGCCGCACGATCATACCTTGTAGATGGCCACAAACTCATCGCGAATGTTATTAATGCCACAGGCTATGA
CCTGAAACACAATGTTGTCATCTCACCGCCACCCAAAGATGGTTCCGCATTGGCAAAGTGGTTTGGCCTGGGTAGGAATGGAATACTGGAAGAA
GGATGCGATTCAAGTGCTTCGTGTTCTCAAAATGGACAAACTTTCAAAGGCATATTCTTTCATCACTTGATTGCGTTCTGTAGTGATTTGCCAG
GGGAGCCTATTGCAGGGACGAAGGAAAGCTTAGAACTCGACAGAGTGTGGCATTCTGACAAATGCTCACAGTATACAAAAT

>Bcin06g01710(MLST3), partial sequence [organism=Botrytis cinerea, strain D11_T_WF43]
GGTGAGTCTGACTTTTGTGTTTGAGCGTTAAGATAGACACTGATGTACCAAGGCAATCACCTAATTTCATGGCGTTCTTCCCTGCATCATCTAC
CTACCCTGGAATGCTGGGAGAATTATACTCAGCAGCTTTCACAGCACCTGCTTTCAATTGGATCTGTTCCCCTGCCGTGACAGAATTGGAGACG
GTTGTAATGGATTGGCTGGCCAAGCTTCTCAATCTCCCAGACTGTTATTTGTCTTCGACTCATGGTGGTGGTGTTATCCAAGGATCAGCCTCGG
AAGCTATCGTTACCGTTATGGTTGCTGCCCGCGATAAATATCTTCGTGAAACTACTGAAGGTCTGTCGGGCATTGAACTCGAGGATGCGATTGC
ATATAAGAGGAGTAAGCTAGTTGCACTAGGAAGCGAAATGGCACACAGCTCCACGCAGAAAGCAGCGCAGATAGCTGGCGTTAGATTCCGATCG
ATTCCAGTACTCGCATCCAATGATTTCGCCATGACGGGTGATGATTTAGAGAAGGTATTGGAAGAATGCAAATCTCAAGGATTGGAACCCTTCT
ATCTAACTTCGACTTTGGGAACAACATCTACATGCGCAGTTGACGACTTCGCATCTATTGCAACAGTACTTTCAAAATATGCACCTCCAGATGT
TGCAGGCGAGATCTGGGTTCACGTCGATGCTGCTTATGCAGGTGCAGCTTTGGTTTGCCCTGAATACCATCATCTAACATCGTCCTTCCAGCAT
TTCCATTCCTTCGATATGAACATGCACAAATGGCTTCTGACAAATTTCGACGCTTCTTGTCTATATGTCAAGAAACGCAAAGATCTGATAGATG
CACTCTCCATAACACCAAGTTATCTTCGCAACGAGTTTTCAGAGAGTGGACTCGTAACCGACTATCGGGATTGGCAAATTCCTCTCGGAAGACG
CTTCCGAAGCTTAAAGATTTGGTTTGTCCTCAGAACCTAC

>Bcin09g03030(MLST4), partial sequence [organism=Botrytis cinerea, strain D11_T_WF43]
ACCCTTCAACCCCACCGCAGCTCCTATACCGAAAGCGAGTATCCTACCAATTCTCCTTCCACCTGCGACTTTAAGACCATTGGCTTTCCGCACT
TTCACAAAAAAGCATAGTTTAACATTGACGTCGTCGGCATTACAAGTGTTGGCTACTTTTTATTGAAAGCATTGTGGGACAGGATGAGGGAAGA
AGGACTGGCAGAGAGAGTCTTGGAGGAGGTCGCCAAGAGTTGGAAGAATAGGAGTGGCGGTGTCATTGTCGAGGGCGAGGGAACGGAATTGAGG
AGATTCTGAAAGCTTTGAAGGGAATATGAGTGGTGGAAGGATAGTCATAGGAAGAGAGCTAAGCCGGCAGAATAGTTTAGCACTGGGATCATCA
CAATATGGAGAGGTCAATCATACAAGACTTGGGCTACGGCCAGGGAATATACCCAGAGAGGATAGTCAGTCAAGTTTGGGAATGTCAACGTTGG
AGGTCAATGACGAGGAGGATGAGGATGGCCTGATGGATCCAAGAAGGTGGTTAAAAAGTCATTGATGCATTTGAGCAACCTCGCACTGGTGTAC
AATGTTGCTAAAAAGCACTTTGATAGGTATGTTTCAATGATAAAATTTTATCTGAATCTACTAACTCAGTACAGAGATACCTCCAAACCTTCAT
TGTTCCCACCTGCGTCTCATAAAACACTCCTCTTCCAAAACCGCTATAATGTTATCCATCAACGTCTCCTTCGCAATGAATCTTTTCAAAAACG
CCCGCTTTTCAGGTGGCAAATCTTCCCTTCACGCAGCACGTCCGCCATTACCACCCAACAACAATCATACAAATTAACGCCGATAGCTAATCTT
CTCGGTCGCAATCGCAGCTCTCATATGCTTCTCGGTCTCCTCGGTATTTCACCCACTGGTACCCTCGCCATCAATGACCTGACGGGCAGTATCG
CTCTCGATCTACACACGCAGCAGCCATTC

>Bcin11g01310(MLST5), partial sequence [organism=Botrytis cinerea, strain D11_T_WF43]
GCTGACATGGACCTCATGTGGAACCGGCGTAGAATGCGCAACGCTTGAAGTTCCGCTCGAATATGGCGATGCAACGTCAACGGCAAAAGCCAGT
GTTGCGCTTGCTCGTTATCCTGCCACTGTTGCCGCGAGCAAGAAGCTCGGGTCTCTCTTGATAAACCCCGGTGGACCCGGTGCCTCTGGTGTTG
GCTTTGTGCAGTCTGGAGCCGGTGCCGCCGTCTCGACACTGAGTGGTGGATTGTACGATATCATCGGATGGGATCCACGTGGAACCGGTGCCTC
GGCTCCTATTTTGGAATGTTTTGCAAATGCCAGTGCGGAGTATGATTTTAACAACGCGTTTCCATCTGCTCCGAATCTCTGGCTCGGACAATTT
GCGAATGCCAGCGCAAATTCTGCTGTTAGCTCTGCTATCACATCCTTTGACACTTCTGTCGCTGCTCTTGCAAAAGCTTGCGTGGCTCAGAAAT
CTCCCGCTCTTTACACCTCAACAGCAGCATATGTTGCTCGAGACATGGCAGCGATAGTCGATGCATTGGATGGGACCTCTGCAAAACTTAACTA
CTGGGGTTTCTCATATGGAACTATCTTCCTAGCTGAGTTTATCCAAACTTTCCCAGGCCGCGTGGGAAGAGTTCTTGCCGATGGTGTTTTCGAC
GCAAAGGCAAATGCACTCACATACGTTAGCCAACTTCCCAACGATCAACTCAGTGTTCGTGCTTCGTTGAACGATTTTGCAGCTTTCTGCACCA
CCGCCGGTAGTAAAGGTTGCTCTTTTGCCACCGCCCCTACTGGAACCTCAGGTACTGTTGCTACCAGACTGGACAACATAATGAAGGATATGTT
CCTCAATCCTATTGTTGCTTCGGGCTTAAGCATCA

>Bcin15g03910(MLST6), partial sequence [organism=Botrytis cinerea, strain D11_T_WF43]
GCCAAAACACAAAATCATCCAACGATGAAGATGATACTCCACTTCCCTTGATTATCTGGCATGGACTCGGCGATAATTACAAAGCGGATGGTCT
TGCGCAAGTTGGAAAACTAGCTGAAGCTATTCATCCTGGGACTTTTGTCTACAATATTCATGTAGATGAGGATGCATCTGCAGATAGGACAGCT
ACCTTCTTTGGAAATCTCACTCGTGAGTACATCCCCTATTTTTCCTTTAAATATCATACTAACTCTCTTACCAAGTTCAAATCGAAAAGGTCTG

CGAAGACCTCGCCTCCCATCCTATTCTCTCTACCGCGCCCGCCGTCGACGCAATTGGATTCTCCCAAGGCGGCCAATTCTTGCGTGGTTACATA
TCCCGCTGCAATGCTCCACCCATCCGCTCTCTCCTGACCTTTGGTTCCCAACACAACGGCATTTCTGCCTTCCAAGCCTGTGGTCCTGCCGATT
TCCTCTGTCGCGGTGCTCAAACCCTTTTGCGATCCAACACCTGGTCAACCTTTGTCCAATCTCGTCTCGTACCCGCTCAATACTTCAGAGATCC
GGAAAACCTAGACTCTTACCTTGAATATTCCAATTTCCTTGCCGACATCAATAATGAGCGCGTTCTCAAGAACCAAACATATAAATCCAACATG
GAAAAATTGGAACGATTCGTAATGTATGTCTTTGAAGACGACACAACTGTCATTCCTAAGGAAAGTGGATGGTGGGCTGAAGTCAACGGCACGG
AAGTTACACCACTGAAAGAAAGAGCCATTTATAAAGAAGATTGGCTAGGTTTAAAGACATTGGATGAGGCCGGAAAATTAGTTTTCGAAACCAT
TCCAGGGGGACGTATGACGTTAGGAGAGGAGATGCTAGAGAAGGCTTTCAAAGAGTATTTTGGTCCAGCAGGGAAGAAATTTGGG

>Bcin16g03460(MLST7), partial sequence [organism=Botrytis cinerea, strain D11_T_WF43]
ATGAACCTCTTGATTTGAACTCTTCATTAATTGCAAGTGATGAACCCCTATTCTGCCCAGAGGATAGTTACAAGACGTATATCATTAGTCGAGA
ACCACTCATGATATATATTGACGGATTTTTGAAAGCGAATGAAAGTAAACATTTGGTTGATGTTAGGTGTGTTATTTATTCTGATGAAATGAAC
AAGAGAGACTGATGAGATAGTGAACCGCTTTATGAACCGTCTACTGTTTCTCACGGGCAGGAAGTTACCATTGATACTTCAGTTCGAAATTCTG
AAGTGGCGGTTTTAGAGAGGGATGAGGTGGTCAGGTGTATTGAGCATAGAGCGAGGGCATTTCAGGGTGGAGGGGCGAGATGGGGATTGAGAAG
TTGAGGACGCAGAGGTATGGGGTTGGAGGACATTATGGGATGCATTTGTAAGTTTTGGGGGATTGACGAAGGCTTCTGTCTATTTTCTACCAGT
ACGGATCTTGAAAGAAAGATAGCATGAACACGAGGGCTAATAACTAGGAAATAGCGATTGGAGCGGAGGTAAACGTGGCATAGACCGATTTAGT
ACTTTCATGGTCTATGTCGACGTATCCTCTGATATCGAAGGTGGAGGAACGGAATTCCCACGTATTGTGGGACCAAAAGGAGGAAGGTGGGAGG
ACTTCCTGGAAACTACGGAAGCATTGGATCCAAGAACTGGAGAAAATGTAACAGTAGAAGGGGTGACATTCAAACCAATCAAGGGAAATGCCGT
ATTCTGGGAAAATACTGACAACAACGGGAGGGGCTATGAT

>Bcin12g03020(MLST8), partial sequence [organism=Botrytis cinerea, strain D11_T_WF43]
CGATTGGCTGCGAAGAAAACTGCGCAGCACCGAGTCACCAAAAGCACGAATAAACCATCGGCAAGGGAAATATTCTCTGAACCACAACATGACA
CGAGCGCGGAAGAGTATATCGGGCGAGAAGCTTCATCAAGAGCACCAAAGCGACAACGAGTCGATGATAATTATAACTCTTACGGTGGAAGAAA
TGAAAATACAGCAGCTTATGCTTCCGGGAAACTTCCATCTGGAAGTATAAATGTTGGTGGAGGTAGGAAGACCACTTTTCAAGAAGAACCTCGA
ACGGCATTTGTCGCTGGCAAGTTGCCCCCTGGAAGTATCAACATTGGTGGAAAGAAGGCTACCCAAATCGAGGACGAGGGTAGAGCAGCTTATG
CTTCCGGAAAATTGCCCCCAGGAAGTATAAACTCTGGCGCAAAGAAGGCGATTTCATTCCAAGATGAAACGAGACCGGCTTATGTCTCCGGAAA
GCTTCCACATGGTAGTATCGACGGTATGCGAAACCGTGAAATGGCTGCCGTCCACCGCGAAATTGCTGAGGGTGGAGGAAACCAGGCCAGGTT
GTCTCTTCTCTATTCACATTCAATCCTACTTCAAAGAAAACTTTCGATGAGCCAGAAGAACAAGCGGAACCGGCAAAACCATCCAATGCGCCTT
TGACAGAGGAAATGGCGACTTTCACCAATCTAGGGCTATCGAGAAGGCTTGCAGCCCATCTATCGACTAAACTCGATATGAAAGCCCCGACGGC
CATTCAAAAAGCATCTGTGCAGCAGTTGGTATCGGACGATAGCGATGCTTTCATACAAGCAGAGACTGGATCTGGAAAAACTTTGGCATATCTA
CTACCTATAGTCGAGCGAATATTAGCATTGAGTGAGAATGGCGTACAAA

>Bcin02g07770(MLST9), partial sequence [organism=Botrytis cinerea, strain D11_T_WF43]
CAGCTTTCCCTTTCGGTCTTGGCATCTACAGTCATTGCCATCCCTACACCATCACAACTTGAGTCTCGGGCCGTTATCGATTCCGATGCCGTTG
TAGGATTTGCCGAAACTGTTCCCAGTGGGACCGTAGGAACAGTTTATGAGGCATATAAACCATTCCTTAAAGTCGTAAATGGATGCGTACCATT
CCCTGCCGTCGATGCATCGGGTAACACAGGGTATGTCCTTATACCTTTCTCTTCCACACGATTGCTATTGAGTCTCTAACATATTTTAGTGGTG
GTTTGTCACCAACTGGCAGCAGCAATGGTGAATGCAGCAGCAGTACCGGTCAAGTATATGTTCGAGGAGGACAAAGCGGATCAAACTACGCTAT
CATGTACTCCTGGTAAGTTCTCTTTAAACTTCTCCTTATAGATCCAACCTAACAAAATCTTAGGTACATGCCAAAGGACGAGCCCTCAACCGGT
ATTGGTCACCGTCACGATTGGGAAGGTGTAATTGTCTGGCTCTCCAGCGCCACCGCCACAACTGCCGACAACATCTTAGCCGTTTGTCCTTCCG
CCCACGGAGGCTGGGATTGTTCCACGGATGGCTATTCCCTTTCTGGTACCAGCCCTCTTATCAAGTACGAAAGTATCTGGCCCGTCGATCACTC
AATGGGTCTTACTAGTACTGTTGGTGGAAAACAACCTATGATTGCTTGGGAGTCTTTACCAACTGCTGCTCAAACTGCTCTTGAGAACACCGAT
TTCGGTGCTGCGAATGTTCCATTCATTCCGGCTGTTTTCACAGATAATCT

>Bcin04g02090(MLST10), partial sequence [organism=Botrytis cinerea, strain D11_T_WF43]
CGGAGGATGATATGGCAAAGTCTATGATTACCAAAGCATTTGTAGGCATGAGTAGTAAATTGTCGAATATGACTATGAATGATGTTTACAAGCC
CTACATCCATGTAAGAAATGTAGAATAAAGAGATCAGTAACTGGAACTAATATCGTTTGCAGGCTTTCAAGTTACTTACGCAGTTCAACCCAAT
CACTACAGCTATTGCCGAATCCCCACTATTTCAAATGGCTGTCTCAGCAAATACCATCGAAAAGTACACACTGCTAGGCCCTTTCTTCAGAATA
TCTCCTCGCAACAGGAAGTTACCAGGGAATACTTCAGTGCGCCAAAGACGATAGATAGGCGACACATTGCCACATCTCAAGATGCGTTACGATT
GACCTACAAACCCATCAAAAAGATTTACTTGATATCATCAACCACTTTGTTCGAGCAAGTCCAATCGCAAAAAGCAAAACCCTGGATTGGTTCG
CCTACATTGTGAATCAAAATCACAAGCGTCGAGCACTTCAGGTAGACCCGAAAGAAGTGTCTTCTGATGGCTTTATGCACAATGTCACTGTCGT
TCTAGATGGTCTTTGTGAGCCATTCATGGATACCACATTCCAAAAATTTCGAAGATTGATATTGATTATCTAAGACGTGCGCCTCGTGTAGATA
TCAAGGACGAGACCAAGTTGAACGCTGATGAGAAGGCTTCTGAGAAGTATTATGAGGACACTGTTCCTGGCACTTCTAATTTCATCTCTGAGGT
ATTCTTTCTGACATTGGCTGCTCATCATTATGGTAGTGAAGCTCTTAATGCCACGCATAAGAGTCTGGAGAAAGACATCAAATATATTCAAAAG
CAATTGACTGCCGTTGAAGCA

>Bcin01g07220(MLST1), partial sequence [organism=Botrytis cinerea, strain D11_T_WH08]
ATGGCATATCTTGTTTCTTTTCGCATTATGCTTTGGAACGTGCTGGACAAGCTTGGGTTAGTGCTCTGCGCGTAGAAGCACTAAAGAAGATTCT
CGCACAACCGAAGTCATGGTTTGAGGAATCCAGGAATTCACCTAGCCGGTTGAACGAAGTTTTGGATAGGAACTCTGAGGAAATGCGTAATCTC

GTTGGCCGCTTTGCTGGTATTGTATTCACAGCATTTTTTATGCTATTGATATCAATCATTTGGGCTTTCGTGAATACATGGAAACTGACATTAG
TCTCGATGGCAACTGGGCCAGTTATATACGCTGTCACCAAAACGTTCAATCGCGTGAGTGGAAAATGGGAAAACAAGTGCAACTACGCCTCTGA
AATGACCACTGGCATATTTTCAGAGACGTTCTCCAACATCAAAGTGGTTCGGGCTTTTACTCTGGAAACTTACTTTGAGACAAAACACACCAAA
GCTACAGAAGAACTTTATAAAGTTGGACTAATACGAGCAAACTACTCGGGATTGCTGTGGGGATTGACAGATGCGATGTCATTCTTCATCACTG
CAACTATCTTTTATTATGCCACGGTTCTCATTACCAAGAGAGAGATCAGTATCGCGACTGCACTACAGACTGTCAATCTTCTATTATTTGGTAT
TTCTAATAGTACGAATATGCTGGCCATGATACCACAAATCAACTCTTCTCGCGTTACAGCTACGCATATGCTTGCATTAGCCAATCTCGATTCA
TCTTCCTCCCACGAAAATAAAGGAACCGAACGGCTTTCGACAATCTTTCCAATCAAATTCAACCGTCTTTCATTCACATACCCTACTCGTCCTG
AAAAACGAACGATATCATCCTTTTCTCTTTCCCTGATTCCTAACTCAACAACTGCACTTGTCGGACCCTCCGGCTCCGGAAAATCTACAATAGC
TGCTCTGCTCATTGGTCTCTATCCGCCAGATACTTCAACACCTCCACCGTTGACATTCAATCGCGTCTCCATAAGTAACTGTCACATTCCGTCT
CTCCGGGCTTCTCTCTCACTCGTTCCACAAATACCGATTCTATTTCCAGCTACCATTCTCCATAACATCATTTATGGTCTCCCAGAATCTTCTC
CTTGTGCTAGTCTTCCATCTGCTATTGCATCAGCAAAAGATGCTGGGATCCATGAATTTATCACATCGCTTCCACAAGGTTATGATACTAT

>Bcin05g07690(MLST2), partial sequence [organism=Botrytis cinerea, strain D11_T_WH08]
ACCACTATCACCAAGTCCTTTTCTGCACTTTCCTCATCAAAAACGAACGAGAACATCATCAATAAATACTTTGCTGAAGTAATTGGGTTTTATT
TTGGCCAGGATGCCTTCTCTCTACGACAGCAAGCCTTCGATGATATGTTGTGGGTAGTTCTTGGCTGGCTGGATACTGTCAAATTCATTGATTT
ACATTCTGAATTGCACTATTCAAACGACTCTCAGCCAGAATGGTACGGACAACAATATAAACCTGCATTTGCACATCGAGCGCGACTATTTTGG
GAATTGGCTTCACAAGGATGGGATACTACTCTCTGTGGTGGTGGGATGATATGGTCACCATACCTTACTCCATACAAGAACGCAATTACCAATG
AACTCTATATCGCAGCTTCGATATCGATGTACCTATATTTCCCCGGAGATGACAATCAATCCCCATTTATGCTTTCCAACCCTTCGTATCCACC
TCACGATCCGAAATATCTACAGGCAGCTGTTGATGCTTACAAATGGCTGAATGGTTCCAACATGACGGATTTGCAAGGATTATATGTCGACGGG
TACCATATCTCGAATCTTTCTGGCGGTGAAAACACCCATTGCGATTCTAGAAATGAGATGGTATATACCTACAATCAAGGTGTTTTGCTTACTG
GACAACGTGGTTTGTATGACGCAACCGCCGCACGATCATACCTTGTAGATGGCCACAAACTCATCGCGAATGTTATTAATGCCACAGGCTATGA
CCTGAAACACAATGTTGTCATCTCACCGCCACCCAAAGATGGTTCCGCATTGGCAAAGTGGTTTGGCCTGGGTAGGAATGGAATACTGGAAGAA
GGATGCGATTCAAGTGCTTCGTGTTCTCAAAATGGACAAACTTTCAAAGGCATATTCTTTCATCACTTGATTGCGTTCTGTAGTGATTTGCCAG
GGGAGCCTATTGCAGGGACGAAGGAAAGCTTAGAACTCGACAGAGTGTGGCATTCTGACAAATGCTCACAGTATACAAAAT

>Bcin06g01710(MLST3), partial sequence [organism=Botrytis cinerea, strain D11_T_WH08]
GGTGAGTCTGACTTTTGTATTTGAGCGTTAAGATAGACACTGATATACCAAGGCAATCACCTAATTTCATGGCGTTCTTCCCTGCATCATCTAC
CTACCCTGGAATGCTGGGAGAATTATACTCAGCAGCTTTCACAGCACCTGCTTTCAATTGGATCTGTTCCCCTGCTGTGACAGAATTGGAGACG
GTTGTAATGGATTGGCTGGCCAAGCTTCTCAATCTCCCAGACTGTTATTTGTCTTCGACTCATGGTGGTGGTGTCATCCAAGGATCAGCCTCGG
AAGCTATCGTTACCGTTATGGTTGCTGCCCGCGATAAATATCTTCGTGAAACTACTGAAGGTCTGTCGGGCATTGAACTCGAGGATGCGATTGC
ATATAAGAGGAGTAAGCTAGTTGCACTAGGAAGCGAAATGGCACACAGCTCCACGCAGAAAGCAGCGCAAATAGCTGGCGTTAGATTCCGATCG
ATTCCAGTACTCGCATCCAATGATTTCGCCATGACGGGTGATGATTTAGAGAAGGTATTGAAAGAATGCAAATCTCAAGGATTGGAACCCTTCT
ATCTAACTTCGACTTTGGGAACAACATCTACATGCGCAGTTGACGACTTCGCATCTATTGCAACAGTACTTTCAAAATATGCACCTCCAGATGT
TGCAGGCGAGATCTGGGTTCACGTCGATGCTGCTTATGCAGGTGCAGCTTTGGTTTGCCCTGAATACCATCATCTAACATCGTCCTTCCAGCAT
TTCCATTCCTTCGATATGAACATGCACAAATGGCTTCTGACAAATTTCGACGCTTCTTGTCTATATGTCAAGAAACGCAAAGATCTGATCGATG
CACTCTCCATAACACCAAGTTATCTTCGCAACGAGTTTTCAGAGAGTGGACTCGTAACCGACTATCGGGACTGGCAAATTCCTCTCGGAAGACG
CTTCCGAAGCTTAAAGATTTGGTTTGTCCTCAGAACCTACG

>Bcin09g03030(MLST4), partial sequence [organism=Botrytis cinerea, strain D11_T_WH08]
ACCCTTCAACCCCACCGCAGCTCCTATACCGAAAGCGAGTATCCTACCAATTCTCCTTCCACCTGCGACTTTAAGACCATTGGCTTTCCGCACT
TTCACAAAAAAGCATAGTTTAACATTGACGTCGTCGGCATTACAAGTGTTGGCTACTTTTATTGGAAAGCATTGTGGGACAGGATGGAGGGAAG
AAGGACTGGCAGAGAGAGTCTTGGAGGAGGTCGCCAAGAGTTGGAAGAATAGGAGTGGCGGTGTCATTGTCGAGGGCGAGGGAACGGAATTGAA
GGAGATTCTGAAAGCTTTGGAAGGGAATATGAGTGGTGGAAGGATAGTCATAGGAAGAGAGCTAAGCCGGCAGAATAGTTTAGTACTGGGATCA
TCACAATATGGAGAGGTCAATCATACAAGACTTGGGCTACGGCCAGGGAATATACCCAGAGAGGATAGTCAGTCAAGTTTGGGAATGTCAACGT
TGGAGGTCAATGACGAGGAAGATGAGGATGGCCTGATGGATCCAAGAAGGTGGTTAAAAGTCATTGATGCATTTGAGCAACCTCGACTGGTGTA
CAATGTTGCTAAAAAGCACTTTGATAGGTATGTTTCAATGATAAAATTTTATCTGAATCGACTAACTCAGTACAGAGATACCTCCAAACCTTCA
TTGTTCCCACCTGCGTCTCATAAAACACTCCTCTTCCAAAACCGCTATAATGTTATCCATCAACGTCTCCTTCGCAATGAATCTTTTCAAACGC
CCGCTTTTCAAGGTGGCAAATCTTCCCTTCAACGCAGCACGTCCGCCATTACCACCCAACAACAATCATACAAATTAACGCCGATAGCTAATCT
TCTCGGTCGCAATCGCAGCTCTCATATGCTTCTCGGTCTCCTCAGTATTTCACCCACTGGTACCCTCGCCATCAATGACCTGACGGGCAGTATC
GCTCTCGATCTTACACACGCAGCAGCCATTC

>Bcin11g01310(MLST5), partial sequence [organism=Botrytis cinerea, strain D11_T_WH08]
ACTGACATGGACCTCATGTGGAACCGGCGTAGAATGCGCAACGCTTGAAGTTCCGCTCGAATATGGCGATGCAACGTCAACGGCAAAAGCCAGT
GTTGCGCTTGCTCGTTATCCTGCCACTGTTGCCGCGAGCAAGAAGCTCGGGTCTCTCTTGATAAACCCCGGTGGACCCGGTGCCTCTGGTGTTG
GCTTTGTGCAGTCTGGAGCCGGTGCCGCCGTCTCGACACTGAGTGGTGGATTGTACGATATCATCGGATGGGATCCACGTGGAACCGGTGCTTC
GGCTCCTATTTTGGAATGTTTTGCAAATGCCAGTGCGGAGTATGATTTTAACAACGCGTTTCCATCTGCTCCGAATCTCTGGCTCGGACAATTT
GCGAATGCCAGCGCAAATTCTGCTGTTAGCTCTGCTATCACATCCTTTGACACTTCTGTCGCTGCTCTTGCAAAAGCTTGCGTGGCTCAGAAAT

CTCCCGCTCTTTACACCTCAACAGCAGCATATGTTGCTCGAGACATGGCAGCGATAGTCGATGCATTGGATGGGACCTCTGCAAAACTTAACTA
CTGGGGTTTCTCATATGGAACTATTTTCCTAGCTGAGTTTATCCAAACTTTCCCAGGCCGTGTGGGAAGAGTTCTTGCCGATGGTGTTTTCGAC
GCAAAGGCAAATGCACTCACATACGTTAGCCAACTTCCCAACGATCAACTCAGTGTTCGTGCTTCGTTGAACGATTTTGCAGCTTTCTGCACCA
CCGCCGGTAGTAAAGGTTGCTCTTTTGCCACCGCCCCTACTGGAACCTCAGGTACTGTTGCTACCAGACTGGACAACATAATGAAGGATATGTT
CCTCAATCCTATTGTTGCTTCGGGCTTAAGCATCA

>Bcin15g03910(MLST6), partial sequence [organism=Botrytis cinerea, strain D11_T_WH08]
TCGCCAAAACACAAAATCATCCAACGATGAAGATGATACTCCACTTCCCTTGATTATCTGGCATGGACTCGGCGATAATTACAAAGCGGATGGT
CTTGCGCAAGTTGGAAAACTAGCTGAAGCTATTCATCCTGGGACTTTTGTCTACAATATTCATGTAGATGAGGATGCATCTGCAGATAGGACAG
CTACCTTCTTTGGAAATCTCACTCGTGAGTACATCCCCTATTTTTCCTTTAAATATCATACTAACTCTCTTACCAAGTTCAAATCGAAAAGGTC
TGCGAAGACCTCGCCTCCCATCCTATTCTCTCTACCGCGCCCGCCGTCGACGCAATTGGATTCTCCCAAGGCGGCCAATTCTTGCGTGGTTACA
TATCCCGCTGCAATGCTCCACCCATCCGCTCTCTCCTGACCTTCGGTTCCCAACACAACGGCATTTCTGCCTTCCAAGCCTGTGGTCCTGCCGA
TTTCCTCTGTCGCGGTGCTCAAACCCTTTTGCGATCCAACACCTGGTCAACCTTTGTCCAATCTCGTCTCGTACCCGCTCAATACTTCAGAGAT
CCGGAAAACCTAGACTCTTACCTTGAATATTCCAATTTCCTTGCCGACATCAATAATGAGCGCGTTCTCAAGAACCAAACATATAAATCCAACA
TGGAAAAATTGGAACGATTCGTAATGTATGTCTTTGAAGACGATACAACTGTCATCCCTAAGGAAAGTGGATGGTGGGCTGAAGTCAACGGCAC
GGAAGTTACACCACTGAAAGAAAGAGCCATTTATAAAGAAGATTGGCTAGGTTTAAAGACATTGGATGAGGCCGGAAAATTAGTTTTCGAAACC
ATTCCAGGGGGACATATGACGTTAGGAGAGGAGATGCTAGAGAAGGCTTTCAAAGAGTATTTTGGTCCAGCAGGGAAGAAATTTGGG

>Bcin16g03460(MLST7), partial sequence [organism=Botrytis cinerea, strain D11_T_WH08]
ATGAACCTCTTGATTTGAACTCTTCATTAATTGCAAGTGATGAACCCCTATTCTGCCCAGAGGATAGTTACAAGACGTATATCATTAGTCGAGA
ACCACTCATGATATATATTGACGGGTTTTTGAGAGCGAATGAAAGTAAACATTTGGTTGATGTTAGGTGTGTTATTTATTCTGATGAAATGAAC
AAGAGAGACTGATGAGATAGTGAGCCGCTTTATGAACCGTCAACTGTTTCTCACGGACAGGAAGTTACCATTGATCCTTCAGTTCGGAATTCTG
AAGTGGCGGTTTTAGAGAGGGATGAGGTGGTCAGGTGTATTGAGCATAGAGCGAGGGCATTTCAGGGGTGGAGGGGCGAGATGGGGATTGAGAA
GTTGAGGACGCAGAGGTATGGGGTTGGAGGACATTATGGGATGCATTTGTAAGTTTTGGGGGATTGACGAAGGCTTTTGTCTATTTTCTACCAG
TACGGATCTTGAAAGAAAGATAGCATGAACACGAGGGCTAATAACTAGGAAATAGCGATTGGAGCGGAGGTAAACGTGGCATAGACCGATTTAG
TACTTTCATGGTCTATGTCGACGTATCCTCTGATATCGAAGGTGGAGGAACGGAATTCCCACGTATCGTGGGACCAAAAGGAGGAAGGTGGGAG
GACTTCCTGGAAACTACGGAAGCATTGGATCCAAGAACTGGAGAAAATGTAACAGTAGAAGGGGTGACATTCAAACCAATCAAGGGAAATGCCG
TATTCTGGGAAAATACTGACAACAACGGGAGGGGCTATGAT

>Bcin12g03020(MLST8), partial sequence [organism=Botrytis cinerea, strain D11_T_WH08]
CGATTGGCTGCGAAGAAAACTGCGCAGCACCGAGTCACCAAAAGCACGAATAAACCATCGGCAAGGGAAATATTCTCTGAACCACAACATGACA
CGAGCGCGGAAGAGTATATCGGGCGAGAAGCTTCATCAAGAGCACCAAAGCGACAACGAGTCGATGATAATTATAACTCTTACGGTGGAAGAAA
TGAAAATACAGCAGCTTATGCTTCCGGGAAACTTCCATCTGGAAGTATAAATGTTGGCGGAGGTAGGAAGACCACTTTTCAAGAAGAACCACGA
ACGGCATTTGTCGCTGGCAAGTTGCCCCTGGAAGTATCAACATTGGTGGAAAGAAGGCTACCCAAATCGAGGACGAGGGTAGAGCAGCTTATGC
TTCCGGAAAATTGCCTCCAGGAAGTATAAACTCTGGCGCAAAGAAGGCGATTTCATTCCAAGATGAAACGAGACCGGCTTATGTCTCCGGAAAG
CTTCCACATGGTAGTATCGACGGTATGCGAAACCGTGAAATGGCTGCCGTCCACCGCGAAATTGCTGAGGGTGGGAGGAAACCAGGCCAGGTTG
TCTCTTCTCTATTCACATTCAATCCTACTTCAAAGAAAACTTTTGATGAACCCGAAGAACAAGCGGAACCGGCAAAACCATCCAATGCGCCTTT
GACAGAGGAAATGGCGACTTTCACCAATCTAGGGTTATCGAGAAGGCTTGCAGCCCATCTATCGACTAAACTCGATATGAAAGCTCCGACCGCC
ATTCAAAAAGCATCTGTACAGCAGTTGGTATCGGACGATAGCGATGCTTTCATACAAGCAGAGACTGGATCTGGAAAAACTTTGGCATATCTAC
TACCTATAGTCGAGCGAATATTAGCATTGAGTGAGAATGGCGTACAAA

>Bcin02g07770(MLST9), partial sequence [organism=Botrytis cinerea, strain D11_T_WH08]
CAGCTTTCCCTTTCGGTCTTGGCATCTACAGTCATTGCCATCCCTACACCATCACAACTTGAGTCTCGGGCCGTTATCGATTCCGATGCCGTTG
TAGGATTTGCCGAAACTGTTCCCAGTGGGACCGTAGGAACAGTTTATGAGGCATATAAACCATTCCTTAAAGTCGTAAATGGATGCGTACCATT
CCCTGCCGTCGATGCATCGGGTAACACAGGGTATGTCCTTATATCTTTCTCTTCCACACGATTGCTATTGAGTCTCTAACATATTTTAGTGGTG
GTTTGTCACCAACTGGCAGTAGCAATGGTGGTTGCAGCAGCAGTACCGGTCAAGTATATGTTCGAGGAGGACAAAGCGGATCAAACTACGCCAT
CATGTACTCCTGGTAAGTTCTCTCTAAACTTCTCCTTATAGATCCAACCTAACAAAATCTTAGGTACATGCCAAAGGACGAGCCCTCAACCGGT
ATTGGTCACCGTCACGATTGGGAAGGTGTAATTGTCTGGCTCTCCAGCGCCACCGCCACAACTGCCGACAACATCTTAGCCGTTTGTCCTTCCG
CCCACGGAGGCTGGGATTGTTCCACGGATGGCTATTCCCTTTCTGGTACCAGCCCTCTTATCAAGTACGAAAGTATCTGGCCCGTCGATCACTC
AATGGGTCTTACTAGTACTGTTGGTGGAAAACAACCTATGATTGCTTGGGAGTCTTTACCAACTGCTGCTCAAACTGCTCTTGAGAACACCGAT
TTCGGTGCTGCGAATGTTCCATTCATTCCGGCTGTTTTCACAGACAATCTT
>Bcin04g02090(MLST10), partial sequence [organism=Botrytis cinerea, strain D11_T_WH08]
CGGAGGATGATATGGCAAAGTCTATGATTACCAAAGCATTTGTAGGCATGAGTAGTAAACTGTCGAATATGACTATGAATGATGTTTACAAGCC
CTACATCCATGTAAGAAATGTAGAATAGAGAGATCAGTAACTGGAACTAATATCGTTTGTAGGCTTTCAAGTTACTTACGCAGTTCAACCCAAT
CACTACAGCTATTGCCGAATCCCCACTGTTTCAAATGGCCGTCTCAGCAAATACCATCGAAAAGTACACACTGCTAGGCCCTTTCTTCAGAATA
TCGCCTCTGCAACAGGAAGTTACCAGGGAATACTTCAGTGCGCCAAAGACGATAGATAGGCGACACATTGCCACATCTCAAGATGCGTTACGAT
TGACCTTACAAACCCATCAAAAAGATTTACTTGATATCATCAACCACTTTGTTCGAGCAAGTCCAATCGCAAAAAGCAAAACCCTGGATTGGTT

CGCCTACATTGTGAATCAAAATCACAAGCGTCGAGCACTTCAGGTAGACCCGAAAGAAGTGTCTTCTGATGGCTTTATGCACAATGTCACTGTC
GTTCTAGATGGTCTTTGTGAGCCATTCATGGATACCACATTCTCGAAAATTTCGAAGATTGATATTGATTATCTAAGACGTGCGCCTCGTGTAG
ATATCAAGGACGAGACCAAGTTGAACGCTGACGAGAAGGCTTCTGAGAAGTATTATGAGGACACTGTTCCTGGCACTTCTAATTTCATCTCTGA
GGTCTTCTTTCTCACATTGGCTGCTCATCATTATGGTAGTGAAGCTCTTAATGCCACGCATAAGAGTCTGGAGAAAGACATCAAATATATTCAA
AAGCAATTGACTGCCGTTGAAGCA

>Bcin01g07220(MLST1), partial sequence [organism=Botrytis cinerea, strain D12_BH20_4]
ATGGCATATCTTGTTTCTTTTCGCATTATGCTTTGGAACGTGCTGGACAAGCTTGGGTTAGTGCTCTGCGTGTAGAAGCACTAAAGAAGATTCT
CGCACAACCGAAGTCATGGTTTGAGGAATCCAGGAATTCACCTGGCCGGTTGAACGAAGTTTTGGATAGGAACTCTGAGGAAATGCGTAATCTC
GTTGGCCGCTTTGCTGGTATTGTATTCACAGCATTTTTTATGCTATTGATATCAATCATTTGGGCTTTTGTGAATACATGGAAACTGACATTAG
TCTCGATGGCAACTGGGCCAGTTATATACGCTGTCACCAAAACATTCAATCGCGTGAGTGGAAAATGGAAAACAAGTGCAACTACGCCTCTGAA
ATGACCACTGGCATATTTCAGAGACTTTCTCCAACATCAAAGTGGTTCGGGCTTTTACTCTGGAAACTTACTTTGAGACAAAACACACCAAAGC
TACAGAGGAACTTTATAAAGTTGGACTAATACGAGCAAACTACTCGGGATTGCTGTGGGGATTGACAGATGCGATGTCATTCTTCATCACTGCA
ACTATCTTTTATTATGCCACGGTTCTCATTACCAAGAGAGAGATCAGTATCGCGACTGCACTACAGACTGTCAATCTTCTATTATTTGGTATTT
CTAATAGTACGAATATGCTGGCCATGATACCACAAATCAACTCTTCTCGCGTTACAGCTACGCATATGCTTGCATTAGCCAATCTCGATTCATC
TTCCTCCCACGAAAATAAAGGAACCGAACGGCTTTCGACAATCTTTCCAATCAAATTCAACCGTCTTTCATTCACATACCCTACTCGTCCTGAA
AAACGAACGATATCATCCTTTTCTCTTTCCCTGATTCCTAACTCAACAACTGCACTTGTCGGACCCTCCGGCTCCGGAAAATCTACGATAGTTG
CTCTGCTCATTGGTCTCTATCCGCCAGATACTTCAACACCTCCACCGTTGACATTCAATCGCGTCTCCATAAGTAACTGTCACATTCCGTCTCT
CCGGGCTTCTCTCTCACTCGTTCCACAAATACCGATTCTATTCCAGCTACCATTCTCCATAACATCATTTATGGTCTCCCAGAATCTTCTCCTT
GTGCTAGTCTTCCATCAGCTATTGCATCAGCAAAAGATGCTGGGATCCATGAATTTATCACATCGCTTCCACAAGGTTATGATACTAT

>Bcin05g07690(MLST2), partial sequence [organism=Botrytis cinerea, strain D12_BH20_4]
ACCACTATCACCAAGTCCTTTTCTGCACTTTCCTCATCAAAAACGAACGAGAACATCATCAATAAATACTTTGCTGAAGTAATTGGGTTTTATT
TTGGCCAGGATGCCTTCTCTCTACGACAGCAAGCCTTCGATGATATGTTGTGGGTAGTTCTTGGCTGGCTGGATACTGTCAAATTCATTGATTT
ACATTCTGAATTGCACTATTCAAACGACTCTCAGCCAGAATGGTACGGACAACAATATAAACCTGCATTTGCACATCGAGCGCGACTATTTTGG
GAATTGGCTTCACAAGGATGGGATACTACTCTCTGTGGTGGTGGGATGATATGGTCACCATACCTTACTCCATACAAGAACGCAATTACCAATG
AACTCTATATCGCAGCTTCGATATCGATGTACCTATATTTCCCCGGAGATGACAATCAATCCCCATTTATGCTTTCCAACCCTTCATATCCACC
TCACGATCCGAAATATCTACAGGCAGCTGTTGATGCTTACAAATGGCTGAATGGTTCCAACATGACGGATTTACAAGGATTATATGTCGACGGG
TACCATATCTCGAATCTTTCTGGCGGTGAAAACACCCATTGCGATTCTAGAAATGAGATGGTATATACCTACAATCAAGGTGTTTTGCTTACTG
GACAACGTGGTTTGTATGACGCAACCGCCGCACGATCATACCTTGTAGATGGCCACAAACTCATCGCGAATGTTATTAATGCCACAGGCTATGA
CCTGAAACACAATGTTGTCATCTCACCGCCACCCAAAGATGGTTCCGCATTGGCAAAGTGGTTTGGCCTGGGTAGGAATGGAATACTGGAAGAA
GGATGCGATTCAAGTGCTTCGTGTTCTCAAAATGGACAAACTTTCAAAGGCATATTCTTTCATCACTTGATTGCGTTCTGTAGTGATTTGCCAG
GGGAGCCTATTGCAGGGACGAAGGAAAGCTTAGAACTCGACAGAGTGTGGCATTCTGACAAATGCTCACAGTATACAAAATG

>Bcin06g01710(MLST3), partial sequence [organism=Botrytis cinerea, strain D12_BH20_4]
GGTGAGTCTGACTTTTGTATTTGAGCGTTAAGATAGACACTGATATACCAAGGCAATCACCTAATTTCATGGCGTTCTTCCCTGCATCATCTAC
CTACCCTGGAATGCTGGGAGAATTATACTCAGCAGCTTTCACAGCACCTGCTTTCAATTGGATCTGTTCCCCTGCTGTGACAGAATTGGAGACG
GTTGTAATGGATTGGCTGGCCAAGCTTCTCAATCTCCCAGACTGTTATTTGTCTTCGACTCATGGTGGTGGTGTCATCCAAGGATCAGCCTCGG
AAGCTATCGTTACCGTTATGGTTGCTGCCCGCGATAAATATCTTCGTGAAACTACTGAAGGTCTGTCGGGCATTGAACTCGAGGATGCGATTGC
ATATAAGAGGAGTAAGCTAGTTGCACTAGGAAGCGAAATGGCACACAGCTCCACGCAGAAAGCAGCGCAAATAGCTGGCGTTAGATTCCGATCG
ATTCCAGTACTCGCATCCAATGATTTCGCCATGACGGGTGATGATTTAGAGAAGGTATTGAAAGAATGCAAATCTCAAGGATTGGAACCCTTCT
ATCTAACTTCGACTTTGGGAACAACATCTACATGCGCAGTTGACGACTTCGCATCTATTGCAACAGTACTTTCAAAATATGCACCTCCAGATGT
TGCAGGCGAGATCTGGGTTCACGTCGATGCTGCTTATGCAGGTGCAGCTTTGGTTTGCCCTGAATACCATCATCTAACATCGTCTTTCCAGCAT
TTCCATTCCTTCGATATGAACATGCACAAATGGCTTCTGACAAATTTCGACGCTTCTTGTCTATATGTCAAGAAACGCAAAGATCTGATCGATG
CACTGTCCATAACACCAAGTTATCTTCGCAACGAGTTTTCAGAGAGTGGACTCGTAACCGACTATCGGGACTGGCAAATTCCTCTCGGAAGACG
CTTCCGAAGCTTAAAGATTTGGTTTGTCCTCAGAACCTAC

>Bcin09g03030(MLST4), partial sequence [organism=Botrytis cinerea, strain D12_BH20_4]
ACCCTTCAACCCCACCGCAGCTCCTATACCGAAAGCGAGTATCCTACCAATTCTCCTTCCACCTGCGACTTTAAGACCATTGGCTTTCCGCACT
TTCACAAAAAAGCATAGTTTAACATTGACGTCGTCGGCATTACAAGTGTTGGCTACTTTTATTGGAAAGCATTGTGGGACAGGATGGAGGGAAG
AAGGACTGGCAGAGAGAGTCTTGGAGGAGGTCGCCAAGAGTTGGAAGAATAGGAGTGGCGGTGTCATTGTCGAGGGCGAGGGAACGGAATTGAA
GGAGATTCTGAAAGCTTTGGAAGGGAATATGAGTGGTGGAAGGATAGTCATAGGAAGAGAGCTAAGCCGGCAGAATAGTTTAGTACTGGGATCA
TCACAATATGGAGAGGTCAATCATACAAGACTTGGGCTACGGCCAGGGAATATACCCAGAGAGGATAGTCAGTCAAGTTTGGGAATGTCAACGT
TGGAGGTCAATGACGAGGAAGATGAGGATGGCCTGATGGATCCAAGAAGGTGGTTAAAAGTCATTGATGCATTTGAGCAACCTCGACTGGTGTA
CAATGTTGCTAAAAAGCACTTTGATAGGTATGTTTCAATGATAAAATTTCATCTGAATCGACTAACTCAGTACAGAGATACCTCCAAACCTTCA
TTGTTCCCACCTGCGTCTCATAAAACACTCCTCTTCCAAAACCGCTATAATGTTATCCATCAACGTCTCCTTCGCAATGAATCTTTTCAAACGC
CCGCTTTTCAAGGTGGCAAATCTTCCCTTCAACGCAGCACGTCCGCCATTACCACCCAACAACAATCATACAAATTAACGCCGATAGCTAATCT

```
TCTCGGTCGCAATCGCAGCTCTCATATGCTTCTCGGTCTCCTCAGTATTTCACCCACTGGTACCCTCGCCATCAATGACCTGACGGGCAGTATC
GCTCTCGATCTTACACACGCAGCAGCCATTC

>Bcin11g01310(MLST5), partial sequence [organism=Botrytis cinerea, strain D12_BH20_4]
ACTGACATGGACCTCATGTGGAACCGGCGTAGAATGCGCAACGCTTGAAGTTCCGCTCGAATATGGCGATGCAACGTCAACGGCAAAAGCCAGT
GTTGCGCTTGCTCGTTATCCTGCCACTGTTGCCGCGAGCAAGAAGCTCGGGTCTCTCTTGATAAACCCCGGTGGACCCGGTGCCTCTGGTGTTG
GCTTTGTGCAGTCTGGAGCCGGTGCCGCCGTCTCGACACTGAGTGGTGGATTGTACGATATCATCGGATGGGATCCACGTGGAACCGGTGCTTC
GGCTCCTATTTTGGAATGTTTTGCAAATGCCAGTGCGGAGTATGATTTTAACAACGCGTTTCCATCTGCTCCGAATCTCTGGCTCGGACAATTT
GCGAATGCCAGCGCAAATTCTGCTGTTAGCTCTGCTATCACATCCTTTGACACTTCTGTCGCTGCTCTTGCAAAAGCTTGCGTGGCTCAGAAAT
CTCCCGCTCTTTACACCTCAACAGCAGCATATGTTGCTCGAGACATGGCAGCGATAGTCGATGCATTGGATGGGACCTCTGCAAAACTTAACTA
CTGGGGTTTCTCATATGGAACTATTTTCCTAGCTGAGTTTATCCAAACTTTCCCAGGCCGTGTGGGAAGAGTTCTTGCCGATGGTGTTTTCGAC
GCAAAGGCAAATGCACTCACATACGTTAGCCAACTTCCCAACGATCAACTCAGTGTTCGTGCTTCGTTGAACGATTTTGCAGCTTTCTGCACCA
CCGCCGGTAGTAAAGGTTGCTCTTTTGCCACCGCCCCTACTGGAACCTCAGGTACTGTTGCTACCAGACTGGACAACATAATGAAGGATATGTT
CCTCAATCCTATTGTTGCTTCGGGCTTAAGCATCA

>Bcin15g03910(MLST6), partial sequence [organism=Botrytis cinerea, strain D12_BH20_4]
TCGCCAAAACACAAAATCATCCAACGATGAAGATGATACTCCACTTCCCTTGATTATCTGGCATGGACTCGGCGATAATTACAAAGCGGATGGT
CTTGCGCAAGTTGGAAAACTAGCTGAAGCTATTCATCCTGGGACTTTTGTCTACAATATTCATGTAGATGAGGATGCATCTGCAGATAGGACAG
CTACCTTCTTTGGAAATCTCACTCGTGAGTACATCCCCTATTTTTCCTTTAAATATCATACTAACTCTCTTACCAAGTTCAAATCGAAAAGGTC
TGCGAAGACCTCGCCTCCCATCCTATTCTCTCTACCGCGCCCGCCGTCGACGCAATTGGATTCTCCCAAGGCGGCCAATTCTTGCGTGGCTACA
TATCCCGCTGCAATGCTCCACCCATCCGCTCTCTCCTGACCTTCGGTTCCCAACACAACGGCATTTCTGCCTTCCAAGCCTGTGGTCCTGCCGA
TTTCCTCTGTCGCGGTGCTCAAACCCTTTTGCGATCCAACACCTGGTCAACCTTTGTCCAATCTCGTCTCGTACCCGCTCAATACTTCAGAGAT
CCGGAAAACCTAGACTCTTACCTTGAATATTCCAATTTCCTTGCCGACATCAATAATGAGCGCGTTCTCAAGAACCAAACATATAAATCCAACA
TGGAAAAATTGGAACGATTCGTAATGTATGTCTTTGAAGACGATACAACTGTCATCCCTAAGGAAAGTGGATGGTGGGCTGAAGTCAACGGCAC
GGAAGTTACACCACTGAAAGAAAGAGCCATTTATAAAGAAGATTGGCTAGGTTTAAAGACATTGGATGAGGCCGGAAAATTAGTTTTCGAAACC
ATTCCAGGGGGACATATGACGTTAGGAGAGGAGATGCTAGAGAAGGCTTTCAAAGAGTATTTTGGTCCAGCAGGGAAGAAATTTGGG

>Bcin16g03460(MLST7), partial sequence [organism=Botrytis cinerea, strain D12_BH20_4]
ATGAACCTCTTGATTTGAACTCTTCATTAATTGCAAGTGATGAACCCCTATTCTGCCCAGAGGATAGTTACAAGACGTATATCATTAGTCGAGA
ACCACTCATGATATATATTGACGGATTTTTGAAAGCGAATGAAAGTAAACATTTGGTTGATGTTAGGTGTGTTATTTATTCTGATGAAATGAAC
AAGAGAGACTGATGAGATAGTGAGCCGCTTTATGAACCGTCAACTGTTTCTCACGGACAGGAAGTTACCATTGATCCTTCAGTTCGGAATTCTG
AAGTGGCGGTTTTAGAGAGGGATGAGGTGGTCAGGTGTATTGAGCATAGAGCGAGGGCATTTCAGGGGTGGAGGGGCGAGATGGGGATTGAGAA
GTTGAGGACGCAGAGGTATGGGGTTGGAGGACATTATGGGATGCATTTGTAAGTTTTGGGGGATTGACGAAGGCTTCTGTCTATTTTCTATCAG
CACGGATCTTGAAAGAAAGATAGCATGAACACGCGGGCTAATAACTAGGAAATAGCGATTGGAGCGGAGGTAAACGTGGCATAGACCGATTTAG
TACTTTCATGGTATATGTCGACGTATCCTCTGATATCGAAGGTGGAGGAACGGAATTCCCACGTATTGTGGGACCAAAAGGAGGAAGGTGGGAG
GACTTCCTGGAAACTACGGAAGCATTGGATCCAAGAACTGGAGAAAATGTAACAGTAGAAGGGGTGACATTCAAACCAATCAAGGGAAATGCCG
TATTCTGGGAAAATACTGACAACAACGGGAGGGGCTATGATG

>Bcin12g03020(MLST8), partial sequence [organism=Botrytis cinerea, strain D12_BH20_4]
CGATTGGCTGCGAAGAAAACTGCGCAGCACCGAGTCACCAAAAGCACGAATAAACCATCGGCAAGGGAAATATTCTCTGAACCACAACATGACA
CGAGCGCGGAAGAGTATATCGGGCGAGAAGCTTCATCAAGAGCACCAAAGCGACAACGAGTCGATGATAATTATAACTCTTACGGTGGAAGAAA
TGAAAATACAGCAGCTTATGCTTCCGGGAAACTTCCATCTGGAAGTATAAATGTTGGTGGAGGTAGGAAGACCACTTTTCAAGAAGAACCACGA
ACGGCATTTGTCGCTGGCAAGTTGCCCCCTGGAAGTATCAACATTGGTGGAAAGAAGGCTACCCAAATCGAGGACGAGGGTAGAGCAGCTTATG
CTTCCGGAAAATTGCCTCCAGGAAGTATAAACTCTGGCGCAAAGAAGGCGATTTCATTCCAAGATGAAACGAGACCGGCTTATGTCTCCGGAAA
GCTTCCACATGGTAGTATCGACGGTATGCGAAACCGTGAAATGGCTGCCGTCCACCGCGAAATTGCTGAGGGTGGGAGGAAACCAGGCCAGGTT
GTCTCTTCTCTATTCACATTCAATCCTACTTCAAAGAAAACTTTTGATGAACCCGAAGAACAAGCGGAACCGGCAAAACCATCCAATGCGCCTT
TGACAGAGGAAATGGCGACTTTCACCAATCTAGGGTTATCGAGAAGGCTTGCAGCCCATCTATCGACTAAACTCGATATGAAAGCTCCGACCGC
CATTCAAAAAGCATCTGTACAGCAGTTGGTATCGGACGATAGCGATGCTTTCATACAAGCAGAGACTGGATCTGGAAAAACTTTGGCATATCTA
CTACCTATAGTCGAGCGAATATTAGCATTGAGTGAGAATGGCGTACAAA

>Bcin04g02090(MLST10), partial sequence  [organism=Botrytis cinerea, strain D12_BH20_4]
CGGAGGATGATATGGCAAAGTCTATGATTACCAAAGCATTTGTAGGCATGAGTAGTAAACTGTCGAATATGACTATGAATGATGTTTACAAGCC
CTACATCCATGTAAGAAATGTAGAATAGAGAGATCAGTAACTGGAACTAATATCGTTTGTAGGCTTTCAAGTTACTTACGCAGTTCAACCCAAT
CACTACAGCTATTGCCGAATCCCCACTGTTTCAAATGGCCGTCTCAGCAAATACCATCGAAAAGTACACACTGCTAGGCCCTTTCTTCAGAATA
TCTCCTCTGCAACAGGAAGTTACCAGGGAATACTTCAGTGCGCCAAAGACGATAGATAGGCGACACATTGCCACATCTCAAGATGCGTTACGAT
TGACCTTACAAACCCATCAAAAAGATTTACTTGATATCATCAACCACTTTGTTCGAGCAAGTCCAATCGCAAAAAGCAAAACCCTGGATTGGTT
CGCCTACATTGTGAATCAAAATCACAAGCGTCGAGCACTTCAGGTAGACCCGAAAGAAGTGTCTTCTGATGGCTTTATGCACAATGTCACTGTC
```

GTTCTAGATGGTCTTTGTGAGCCATTCATGGATACCACATTCTCGAAAATTTCGAAGATTGATATTGATTATCTAAGACGTGCGCCTCGTGTAG
ATATCAAGGACGAGACCAAGTTGAACGCTGACGAGAAGGCTTCTGAGAAGTATTATGAGGACACTGTTCCTGGCACTTCTAATTTCATCTCTGA
GGTCTTCTTTCTCACATTGGCTGCTCATCATTATGGTAGTGAAGCTCTTAATGCCACGCATAAGAGTCTGGAGAAAGACATCAAATATATTCAA
AAGCAATTGACTGCCGTTGAAGCA

>Bcin01g07220(MLST1), partial sequence [organism=Botrytis cinerea, strain D12_E_cal13]
GATGGCATATCTTGTTTCTTTTCGCATTATGCTTTGGAACGTGCTGGACAAGCTTGGGTTAGTGCTCTGCGTGTAGAAGCACTAAAGAAGATTC
TCGCACAACCGAAGTCATGGTTTGAGGTATCCAGAAACTCACCTGGCCGGTTGAACGAAGTTTTGGATAGGAACTCTGAGGAAATGCGTAATCT
CGTTGGCCGCTTTGCTGGTATTGTATTCACAGCGTTTTTTATGCTATTGATATCAATCATTTGGGCTTTCGTGAATACATGGAAACTGACATTA
GTCTCGATGGCAACTGGGCCAGTTATATACGCTGTCACCAAAACGTTCAATCGCGTGAGTGGAAAATGGGAAAACAAGTGCAACTACGCCTCTG
AAACGACCACTGGCATATTTTCAGAGACTTTCTCCAACATCAAAGTGGTCGGGCTTTTACTCTGGAAACTTACTTTGAGACAAAACACACCAAA
GCTACAGAAGAACTTTATAAAGTTGGACTAATACGAGCAAACTACTCGGATTGCTGTGGGGATTGACAGATGCGATGTCATTCTTCATCACTGC
AACTATCTTTTATTATGCCACGGTTCTCATTACCAAGAGAGAGATCAGTATCGCGACTGCACTACAGACTGTCAATCTTCTATTATTTGGTATT
TCTAATAGTACGAATATGCTGGCCATGATACCACAAATCAACTCTTCTCGCGTTACAGCTACGCATATGCTTGCATTAGCCAATCTCGATTCAT
CTTCCTCCCACGAAAATAAAGGAACCGAACGGCTTTCGACAATCTTTCCAATCAAATTCAACCGTCTCTCATTCACATATCCTACTCGTCCCGA
AAAACGAACGATATCATCCTTTTCTCTTTCCCTGATTCCTAACTCAACAACTGCACTTGTCGGACCCTCCGGCTCCGGAAAATCTACAATAGCT
GCTCTGCTCATTGGTCTCTATCCGCCAGATACTTCAACACCTCCACCGTTGACATTCAATCGCGTCTCCATAAGTAACTGTCACATTCCGTCTC
TCCGGGCTTCTCTCTCACTCGTTCCACAAATACCGATTCTATTTCCAGCTACCATTCTCCATAACATCATTTATGGTCTCCCAGAATCTTCTCC
TTGTGCTAGTCTTCCATCAGCTATTGCATCAGCAAAAGATGCTGGGATCCATGAATTTATCACATCGCTTCCACAAGGTTATGATACTATG

>Bcin05g07690(MLST2), partial sequence [organism=Botrytis cinerea, strain D12_E_cal13]
ACCACTATCACCAAGTCCTTTTCTGCACTTTCCTCATCAAAAACGAACGAGAACATCATCAATAAATACTTTGCTGAAGTAATTGGGTTTTATT
TTGGCCAGGATGCCTTCTCTCTACGACAGCAAGCCTTCGATGATATGTTGTGGGTAGTTCTTGGCTGGCTGGATACTGTCAAATTCATTGATTT
ACATTCTGAATTGCACTATTCAAACGACTCTCAGCCAGAATGGTACGGACAACAATATAACCTGCATTTGCACATCGAGCGCGACTATTTTGGG
AATTGGCTTCACAAGGATGGGATACTACTCTCTGTGGTGGTGGGATGATATGGTCACCATACCTTACTCCATACAAGAACGCAATTACCAATGA
ACTCTATATCGCAGCTTCGATATCGATGTACCTATATTTCCCGGAGATGACAATCAATCCCCATTTATGCTTTCCAACCCTTCATATCCACCTC
ACGATCCGAAATATCTACAGGCAGCTGTTGATGCTTACAAATGGCTGAATGGTTCCCACATGACGGATTTACAAGGATTATATGTCGACGGTAC
CATATCTCGAATCTTTCTGGCGGTGAAAACACCCATTGCGATTCTAGAAATGAGATGGTATATACCTACAATCAAGGTGTTTTGCTTACTGGAC
AACGTGGTTTGTATGACGCAACCGCCGCACGATCATACCTTGTAGATGGCCACAAACTCATCGCGAATGTTATTAATGCCACAGGCTATGACCT
GAAACACAATGTTGTCATCTCACCGCCACCCAAAGATGGTTCCGCATTGGCAAAGTGGTTTGGCCTGGGTAGGAATGGAATACTGGAAGAAGGA
TGCGATTCAAGTGCTTCGTGTTCTCAAAATGGACAAACTTTCAAAGGCATATTCTTTCATCACTTGATTGCGTTCTGTAGTGATTTGCCAGGGG
AGCCTATTGCAGGGACGAAGGAAAGCTTAGAACTCGACAGAGTGTGGCATTCTGACAAATGCTCACAGTATACAAAAT

>Bcin06g01710(MLST3), partial sequence [organism=Botrytis cinerea, strain D12_E_cal13]
GTGAGTCTGACTTTTTTATTTGAGCATTAAGATAGACACTGATATACCAAGGCAATCACCTAATTTCATGGCGTTCTTCCCTGCATCATCTACT
TACCCTGGAATGCTGGGAGAATTATACTCGGCAGCTCTCACAGCACCTGCTTTCAATTGGATCTGTTCCCCTGCTGTGACAGAATTGGAGACGG
TTGTAATGGATTGGCTGGCCAAGCTTCTCAATCTCCCAGACTGTTATTTGTCTTCGACTCATGGTGGTGGTGTTATCCAAGGATCAGCCTCGGA
AGCTGTCGTTACCGTTATGGTTGCCGCCCGCGATAAATATCTTCGTGAAACTACTGAAGGTCTGTCGGGCATTGAACTCGAGGATGCGATTGCA
TATAAGAGGAGTAAGCTAGTTGCACTAGGAAGCGAAATGGCACACAGTTCCACGCAGAAAGCAGCGCAGATAGCTGGCGTTAGATTCCGATCGA
TTCCAGTACTCGCATCCAATGATTTTGCCATGACGGGTGATGATTTAGAGAAGGTATTGAAAGAATGCAAATCTCAAGGATTGGAACCCTTCTA
TCTAACTTCGACTTTGGGAACAACATCTACATGCGCAGTTGACGACTTCGCATCTATTACAACAGTACTTTCAAAATATGCACCTCCAGATGTT
GCAGGCGAGATCTGGGTTCACGTCGATGCTGCTTATGCAGGTGCAGCTTTGGTTTGCCCTGAATACCATCATCTAACATCGTCCTTCCAGCATT
TCCATTCCTTCGATATGAACATGCACAAATGGCTTCTGACAAATTTCGACGCTTCTTGTCTATATGTCAAGAAACGCAAAGATCTGATAGATGC
ACTCTCCATAACACCAAGTTATCTTCGCAACGAGTTTTCAGAGAGTGGACTCGTAACCGACTATCGGGATTGGCAAATTCCTCTCGGAAGACGC
TTCCGAAGCTTAAAGATTTGGTTTGTCCTCAGAACCTAC

>Bcin09g03030(MLST4), partial sequence [organism=Botrytis cinerea, strain D12_E_cal13]
ACCCTTCAACCCCACCGCAGCTCCTATACCGAAAGCGAGTATCCTACCAATTCTCCTTCCACCTGCGACTTTAAGACCATTGGCTTTCCGCACT
TTCACAAAAAAGCATAGTTTAACATTGACGTCGTCGGCATTACAAGTGTTGGCTACTTTTATTGGAAGCATTGTGGGACAGGATGGAGGGAAGA
AGGACTGGCAGAGAGAGTCTTGGAGGAGGTCGCCAAGAGTTGGAAGAATAGGAGTGGCGGTGTCATTGTCGAGGGCGAGGGAACGGAATTGAAG
GAGATTCTGAAAGCTTTGGAAGGGAATATGAGTGGTGGAAGGATAGTCATAGGAAGAGAGCTAAGCCGGCAAAATAGTTTAGTACTGGGATCAT
CACAATATGGAGAGGTCAATCATACAAGACTTGGGCTACGGCCAGGGAATATACCCAGAGAGGATAGTCAGTCAAGTTTGGGAATGTCAACGTT
GGAGGTCAATGACGAGGAAGATGAGGATGGCCTGATGGATCCAAGAAGGTGGTTAAAAGTCATTGATGCATTTGAGCAACCTCGACTAGTGTAC
AATGTTGCTAAAAAGCACTTTGATAGGTATGTTTCAATGATAAAATTTTATCTGAATCGACTAACTCAGTACAGAGATACCTCCAAACCTTCAT
TGTTCCCACCTGCGTCTCATAAAACACTCCTCTTCCAAAACCGCTATAATGTTATCCATCAACGTCTCCTTCGCAATGAATCTTTTCAAACGCC
CGCTTTTCAAGGTGGCAAATCTTCCCTTCAACGCAGCACGTCCGCCATTACCACCCAACAACAATCATACAAATTAACGCCGATAGCTAATCTT
CTCGGTCGCAATCGCAGCTCTCATATGCTTCTCGGTCTCCTCAGTATTTCACCCACTGGTACCCTCGCCATCAATGACCTGACGGGCAGTATCG

CTCTCGATCTTACACACGCAGCAGCCATTC

>Bcin11g01310(MLST5), partial sequence [organism=Botrytis cinerea, strain D12_E_cal13]
GGACTGACATGGACCTCATGTGGAACCGGCGTAGAATGCGCAACGCTTGAAGTTCCGCTCGAATATGGCGATGCAACGTCAACGGCAAAAGCCA
GTGTTGCGCTTGCTCGTTATCCTGCCACTGTTGCCGCGAGCAAGAAGCTCGGGTCTCTCTTGATAAACCCCGGTGGGCCCGGTGCCTCTGGTGT
TGGCTTTGTGCAGTCTGGAGCCGGTGCCGCCGTCTCGACACTGAGTGATGGATTGTACGATATCATCGGATGGGATCCACGTGGAACCGGTGCT
TCGGCTCCTATTTTGGAATGTTTTGCAAATGCCAGTGCGGAGTATGATTTTAACAACGCGTTTCCATCTGCTCCGAATCTCTGGCTCGGACAAT
TTGCGAATGCCAGCGCAAATTCTGCTGTTAGCTCTGCTATCACATCCTTTGACACTTCTGTCGCTGCTCTTGCAAAAGCTTGCGTGGCTCAGAA
ATCTCCCGCTCTTTACACCTCAACAGCAGCATATGTTGCTCGAGACATGGCAGCGATAGTCGATGCATTGGATGGGACCTCTGCAAAACTTAAC
TACTGGGGCTTCTCATATGGAACTATTTTCCTAGCTGAGTTTATCCAAACTTTCCCAGGCCGCGTGGGAAGAGTTCTTGCCGATGGTGTTTTCG
ACGCAAAGGCAAATGCACTCACATACGTTAGCCAACTTCCCAACGATCAACTCAGTGTTCGTGCTTCGTTGAACGATTTTGCAGCTTTCTGCAC
CACCGCCGGTAGTAAAGGTTGCTCTTTTGCCACCGCCCCTACTGGAACCACAGGTACTGTTGCTACCAGACTGGACAACATAATGAAGGATATG
TTCCTCAATCCTATTGTTGCTTCGGGCTTGAGCATCA

>Bcin15g03910(MLST6), partial sequence [organism=Botrytis cinerea, strain D12_E_cal13]
GCCAAAACACAAAATCATCCAACGATGAAGATGATACTCCACTTCCCTTGATTATCTGGCATGGACTCGGCGATAATTACAAAGCGGATGGTCT
TGCGCAAGTTGGAAAACTAGCTGAAGCTATTCATCCTGGGACTTTTGTCTACAATATTCATGTAGATGAGGATGCATCTGCAGATAGGACAGCT
ACCTTCTTTGGAAATCTCACTCGTGAGTACATCCCCTATTTTTCCTTTAAATATCATACTAACTCTCTTACCAAGTTCAAATCGAAAGGGTCTG
CGAAGACCTCGCCTCCCATCCTATTCTCTCTACCGCGCCCGCCGTCGACGCAATTGGATTCTCCCAAGGCGGCCAATTCTTGCGTGGTTACATA
TCCCGCTGCAATGCTCCACCCATCCGCTCTCTCCTGACCTTCGGTTCCCAACACAACGGCATTTCTGCCTTCCAAGCCTGTGGTCCTGCCGATT
TCCTCTGTCGCGGTGCTCAAACCCTTTTGCGATCCAACACCTGGTCAACCTTTGTCCAATCTCGTCTCGTACCCGCTCAATACTTCAGAGATCC
GGAAAACCTAGACTCTTACCTTGAATATTCCAATTTCCTTGCCGACATCAATAATGAGCGCGTTCTCAAGAACCAAACATATAAATCCAACATG
GAAAAATTGGAACGATTCGTAATGTATGTCTTTGAAGACGACACAACTGTCATCCCTAAGGAAAGTGGATGGTGGGCTGAAGTCAACGGCACGG
AAGTTACACCACTGAAAGAAAGAGCCATTTATAAAGAAGATTGGCTAGGTTTAAAGACATTGGATGAGGCCGGAAAATTAGTTTTCGAAACCAT
TCCAGGGGGACATATGACGTTAGGAGAGGAGATGCTAGAGAAGGCTTTCAAAGAGTATTTTGGTCCAGCAGGGAAGAAATTTGGG

>Bcin16g03460(MLST7), partial sequence [organism=Botrytis cinerea, strain D12_E_cal13]
ATGAACCTCTTAATTTGAACTCTTCATTATTTGCAAGTGATGAACCCCTATTCTGCCCAGAGGATAGTTACAAGACGTATATCATTAGTCGAGA
ACCACTCATGATATACATTGACGGATTTTTGAAAGCGAATGAAAGTAAACACTTGGTTGATGTTAGGTGTGTTATTTATTCTGATGAAATGAAC
AAGAGAGACTGATGAGATAGTGAACCGCTTTATGAACCGTCTACTGTTTCTCACGGGCAGGAAGTTACCATTGATACTTCAGTTCGAAATTCTG
AAGTGGCGGTTTTAGAGAGGGATGAGGTGGTCAGGTGTATTGAGCATAGAGCGAGGGCATTTCAGGGGTGGAGGGGCGAGATGGGGATTGAGAA
GTTGAGGACGCAGAGGTATGGGGTTGGAGGACATTATGGGATGCATTTGTAAGTTTTGGGGGATTGACGAAGGCTTTTGTCTATTTTCTACCAG
TACGGATCTTGAAAGAAAGATAGCATGAACACGAGGGCTAATAACTAGGAAATAGCGATTGGAGCGGAGGTAAACGTGGCATAGACCGATTTAG
TACTTTCATGGTCTATGTCGACGTATCCTCTGATATCGAAGGTGGAGGAACGGAATTCCCACGTATTGTGGGACCAAAAGGAGGAAGGTGGGAG
GACTTCCTGGAAACTACGGAAGCATTGGATCCAAGGACTGGAGAAAATGTAACAGTAGAAGGGGTGACATTCAAACCAATCAAGGGAAATGCCG
TATTCTGGGAAAATACTGACAACAACGGGAGGGGCTATGAT

>Bcin12g03020(MLST8), partial sequence [organism=Botrytis cinerea, strain D12_E_cal13]
CGATTGGCTGCGAAGAAAACTGCGCAGCACCGAGTCACCAAAAGCACGAATAAACCATCGGCAAGGGAAATATTCTCTGAACCACAACATGACA
CGAGCGCGGAAGAGTATATCGGGCGAGAAGCTTCATCAAGAGCACCAAAGCGACAACGAGTCGATGATAATTATAACTCTTACGGTGGAAGAAA
TGAGAATACAGCAGCTTATGCTTCCGGGAAACTTCCATCTGGAAGTATAAATGTTGGTGGAGGTAGGAAGACCACTTTTCAAGAAGAACCTCGA
ACGGCATTTGTCGCTGGCAAGTTGCCCCCTGGAAGTATCAACATTGGTGGAAAGAAGGCTACCCAAATCGAGGACGAGGGTAGAGCAGCTTATG
CTTCCGGAAAATTGCCCCCAGGAAGTATAAACTCTGGCGCAAAGAAGGCGATTTCATTCCAAGATGAAACGAGACCGGCTTATGTCTCTGGAAA
GCTTCCACATGGTAGTATCGACGGTATGCGAAACCGTGAAATGGCTGCCGTCCACCGCGAAATTGCTGAGGGTGGAGGAAACCAGGCCAGGTT
GTCTCTTCTCTATTCACATTCAATCCTACTTCAAAGAAAACTTTCGATGAACCCGAAGAACAAGCGGAACCGGCAAAACCATCCAATGCACCTT
TGACAGAGGAAATGGCGACTTTCACCAATCTAGGGTTATCGAGAAGGCTTGCAGCCCATCTATCGACTAAACTCGATATGAAAGCCCCGACCGC
CATTCAAAAAGCATCTGTGCAGCAGTTGGTATCGGACGATAGCGATGCTTTCATACAAGCAGAGACTGGATCTGGAAAAACTTTGGCATATCTA
CTACCTATAGTCGAGCGAATATTAGCATTGAGTGAGAATGGCGTACAAA

>Bcin02g07770(MLST9), partial sequence [organism=Botrytis cinerea, strain D12_E_cal13]
CAGCTTTCCCTTTCGGTCTTGGCATCTACAGTCATTGCCATCCCTACACCATCACAACTTGAGTCTCGGGCCGTTATCGATTCCGATGCCGTTG
TAGGATTTGCCGAAACTGTTCCCAGTGGGACCGTAGGAACAGTTTATGAGGCATATAAACCATTCCTTAAAGTCGTAAATGGATGCGTACCATT
CCCTGCCGTCGATGCATCGGGTAACACAGGGTATGTCCTTATGCCTTTCTCTTCCACACGATTGCTATTGAGTCTCTAACATATTTAGTGGTG
GTTTGTCACCAACTGGCAGCAGCAATGGTGAATGCAGCAGCAGTACCGGTCAAGTATATGTTCGAGGAGGACAAAGCGGGTCAAACTACGCTAT
CATGTACTCCTGGTAAGTTCTCTCTAAACTTCTCCTTTATAGATCCAACCTAACAAAGTCTTAGGTACATGCCAAAGGACGAGCCCTCAACCGG
TATTGGTCACCGTCACGATTGGGAAGGTGTAATTGTCTGGCTCTCCAGCGCCACCGCCACAACTGCCGACAACATCTTAGCCGTTTGTCCTTCC
GCCCACGGAGGCTGGGATTGTTCCACGGATGGCTATTCCCTTTCTGGTACCAGCCCTCTTATCAAGTACGAAAGTATCTGGCCCGTCGATCACT

CAATGGGTCTTACTAGTACTGTTGGTGGAAAACAACCTATGATCGCTTGGGAGTCTTTACCTACTGCTGCTCAAACTGCTCTTGAGAACACCGA
TTTCGGTGCTGCGAATGTTCCATTCATTCCGGCTGTTTTCACAGATAATCT

>Bcin01g07220(MLST1), partial sequence [organism=Botrytis cinerea, strain D12_H_BioH01]
ATGGCATATCTTGTTTCTTTTCGCATTATGCTTTGGAACGTGCTGGACAAGCTTGGGTTAGTGCTCTGCGTGTAGAAGCACTAAAGAAGATTCT
CGCACAACCGAAGTCATGGTTTGAGGAATCCAGGAATTCACCTAGCCGGTTGAACGAAGTTTTGGATAGGAACTCTGAGGAAATGCGTAATCTC
GTTGGCCGCTTTGCTGGTATTGTATTCACAGCATTTTTTATGCTATTGATATCAATCATTTGGGCTTTTGTGAATACATGGAAACTGACATTAG
TCTCGATGGCAACTGGGCCAGTTATATACGCTGTCACCAAAACATTCAATCGCGTGAGTGGAAAATGGGAAAACAAGTGCAACTACGCCTCTGA
AATGACCACTGGCATATTTTCAGAGACTTTCTCCAACATCAAAGTGGTTCGGGCTTTTACTCTGGAAACTTACTTTGAGACAAAACACACCAAA
GCTACAGAAGAACTTTATAAAGTTGGACTAATACGAGCAAACTACTCGGGATTGCTGTGGGGATTGACAGATGCGATGTCATTCTTCATCACTG
CAACTATCTTTTATTATGCCACGGTTCTCATTACCAAGAGAGAGATCAGTATCGCGACTGCACTACAGACTGTCAATCTTCTATTATTTGGTAT
TTCTAATAGTACGAATATGCTGGCCATGATACCACAAATCAACTCTTCTCGCGTTACAGCTACGCATATGCTTGCATTAGCCAATCTCGATTCA
TCTTCCTCCCACGAAAATAAAGGAACCGAACGGCTTTCGACAATCTTTCCAATCAAATTCAACCGTCTTTCATTCACATACCCTACTCGTCCTG
AAAAACGAACGATATCATCCTTTTCTCTTTCCCTGATTCCTAACTCAACCACTGCACTTGTCGGACCCTCCGGCTCCGGAAAATCTACAATAGC
TGCTCTGCTCATTGGTCTCTATCCGCCAGATACTTCAACACCTCCACCGTTGACATTCAATCGCGTCTCCATAAGTAACTGTCACATTCCGTCT
CTCCGGGCTTCTCTCTCACTCGTTCCACAAATACCGATTCTATTTCCAGCTACCATTCTCCATAACATCATTTATGGTCTCCCAGAATCTTCTC
CTTGTGCTAGTCTTCCATCAGCTATTGCATCAGCAAAAGATGCTGGGATCCATGAATTTATCACATCGCTTCCACAAGGTTATGATACTAT

>Bcin05g07690(MLST2), partial sequence [organism=Botrytis cinerea, strain D12_H_BioH01]
ACCACTATCACCAAGTCCTTTTCTGCACTTTCCTCATCAAAAACGAACGAGAACATCATCAATAAATACTTTGCTGAAGTAATTGGGTTTTATT
TTGGCCAGGATGCCTTCTCTCTACGACAGCAAGCCTTCGATGATATGTTGTGGGTAGTTCTTGGCTGGCTGGATACTGTCAAATTCATTGATTT
ACATTCTGAATTGCACTATTCAAATGACTCTCAGCCAGAATGGTACGGACAACAATATAAACCTGCATTTGCACATCGAGCGCGACTATTTTGG
GAATTGGCTTCACAAGGATGGGATACTACTCTCTGTGGTGGTGGGATGATATGGTCACCATATCTTACTCCATACAAGAACGCAATTACCAATG
AACTCTATATCGCAGCTTCGATATCGATGTACCTATATTTCCCCGGAGATGACAATCAATCCCCATTTATGCTTTCCAACCCTTCATATCCACC
TCACGATCCGAAATATCTACAGGCAGCTGTTGATGCTTACAAATGGCTGAATGGTTCCAACATGACGGATTTACAAGGATTATATGTCGACGGG
TACCATATCTCGAATCTTTCTGGCGGTGAAAACACCCATTGCGATTCTAGAAATGAGATGGTATATACCTACAATCAAGGTGTTTTGCTTACTG
GACAACGCGGTTTGTATGACGCAACCGCCGCACGATCATACCTTGTAGATGGCCACAAACTCATCGCGAATGTTATTAATGCCACAGGCTATGA
CCTGAAACACAATGTTGTCATCTCACCTCCACCCAAAGATGGTTCCGCATTGGCAAAGTGGTTTGGCCTGGGTAGGAATGGAATACTGGAAGAA
GGATGCGATTCAAGTGCTTCGTGTTCTCAAAATGGACAAACTTTCAAAGGCATATTCTTTCATCACTTGATTGCGTTCTGTAGTGATTTGCCAG
GGGAGCCTATTGCAGGGACGAAGGAAAGCTTAGAACTCGACAGAGTGTGGCATTCTGACAAATGCTCACAGTATACAAAAT

>Bcin06g01710(MLST3), partial sequence [organism=Botrytis cinerea, strain D12_H_BioH01]
GTGAGTCTGACTTTTGTATTTGAGCGTTAAGATAGACACTGATATACCAAGGCAATCACCTAATTTCATGGCGTTCTTCCCTGCATCATCTACC
TACCCTGGAATGCTGGGAGAATTATACTCAGCAGCTTTCACAGCACCTGCTTTCAATTGGATCTGTTCCCCTGCTGTGACAGAATTGGAGACGG
TTGTAATGGATTGGCTGGCCAAGCTTCTCAATCTCCCAGACTGTTATTTGTCTTCGACTCATGGTGGTGGTGTCATCCAAGGATCAGCCTCGGA
AGCTATCGTTACCGTTATGGTTGCTGCCCGCGATAAATATCTTCGTGAAACTACTGAAGGTCTGTCGGGCATTGAACTCGAGGATGCGATTGCA
TATAAGAGGAGTAAGCTAGTTGCACTAGGAAGCGAAATGGCACACAGCTCCACGCAGAAAGCAGCGCAAATAGCTGGCGTTAGATTCCGATCGA
TTCCAGTACTCGCATCCAATGATTTCGCCATGACGGGTGATGATTTAGAGAAGGTATTGAAAGAATGCAAATCTCAAGGATTGGAACCCTTCTA
TCTAACTTCGACTTTGGGAACAACATCTACATGCGCAGTTGACGACTTCGCATCTATTGCAACAGTACTTTCAAAATATGCACCTCCAGATGTT
GCAGGCGAGATCTGGGTTCACGTCGATGCTGCTTATGCAGGTGCAGCTTTGGTTTGCCCTGAATACCATCATCTAACATCGTCTTTCCAGCATT
TCCATTCCTTCGATATGAACATGCACAAATGGCTTCTGACAAATTTCGACGCTTCTTGTCTATATGTCAAGAAACGCAAAGATCTGATCGATGC
ACTGTCCATAACACCAAGTTATCTTCGCAACGAGTTTTCAGAGAGTGGACTCGTAACCGACTATCGGGACTGGCAAATTCCTCTCGGAAGACGC
TTCCGAAGCTTAAAGATTTGGTTTGTCCTCAGAACCTAC

>Bcin09g03030(MLST4), partial sequence [organism=Botrytis cinerea, strain D12_H_BioH01]
ACCCTTCAACCCCACCGCAGCTCCTATACCGAAAGCGAGTATCCTACCAATTCTCCTTCCACCTGCGACTTTAAGACCATTGGCTTTCCGCACT
TTCACAAAAAAGCATAGTTTAACATTGACGTCGTCGGCATTACAAGTGTTGGCTACTTTTATTGGAAAGCATTGTGGGACAGGATGGAGGGAAG
AAGGACTGGCAGAGAGAGTCTTGGAGGAGGTCGCCAAGAGTTGGAAGAATAGGAGTGGCGGTGTCATTGTCGAGGGCGAGGGAACGGAATTGAA
GGAGATTCTGAAAGCTTTGGAAGGGAATATGAGTGGTGGAAGGATAGTCATAGGAAGAGAGCTAAGTCGGCAAAATAGTTTAGTACTGGGATCA
TCACAATATGGAGAGGTCAATCATACAAGACTTGGGCTACGGCCAGGGAATATACCCAGAGAGGATAGTCAGTCAAGTTTGGGAATGTCAACGT
TGGAGGTCAATGACGAGGAAGATGAGGATGGCCTGATGGATCCAAGAAGGTGGTTAAAAGTCATTGATGCATTTGAGCAACCTCGACTAGTGTA
CAATGTTGCTAAAAAGCACTTTGATAGGTATGTTTCAATGATAAAATTTTATCTGAATCGACTAACTCAGTACAGAGATACCTCCAAACCTTCA
TTGTTCCCACCTGCGTCTCATAAAACACTCCTCTTCCAAAACCGCTATAATGTTATCCATCAACGTCTCCTTCGCAATGAATCTTTTCAAACGC
CCGCTTTTCAAGGTGGCAAATCTTCCCTTCAACGCAGCACGTCCGCCATTACCACCCAACAACAATCATACAAATTAACGCCGATAGCTAATCT
TCTCGGTCGCAATCGCAGCTCTCATATGCTTCTCGGTCTCCTCAGTATTTCACCCACTGGTACCCTCGCCATCAGTGACCTGACGGGCAGTATC
GCTCTCGATCTTACACACGCAGCAGCCATT

>Bcin11g01310(MLST5), partial sequence [organism=Botrytis cinerea, strain D12_H_BioH01]
GACTGACATGGACCTCATGTGGAACCGGCGTAGAATGCGCAACGCTTGAAGTTCCGCTCGAATATGGCGATGCAACGTCAACGGCAAAAGCCAG
TGTTGCGCTTGCTCGTTATCCTGCCACTGTTGCTGCGAGCAAGAAGCTCGGGTCTCTCTTGATAAACCCCGGTGGACCCGGTGCCTCTGGTGTT
GGCTTTGTGCAGTCTGGAGCCGGTGCCGCCGTCTCGACACTGAGTGGTGGATTGTACGATATCATCGGATGGGATCCACGTGGAACCGGTGCTT
CGGCTCCTATTTTGGAATGTTTTGCAAATGCCAGTGCGGAGTATGATTTTAACAACGCGTTTCCATCTGCTCCGAATCTCTGGCTCGGACAATT
TGCGAATGCCAGCGCAAATTCTGCTGTTAGCTCTGCTATCACATCCTTTGACACTTCTGTCGCTGCTCTTGCAAAAGCTTGCGTGGCTCAGAAA
TCTCCCGCTCTTTACACCTCAACAGCAGCATATGTTGCTCGAGACATGGCAGCGATAGTCGATGCATTGGATGGGACCTCTGCAAAACTTAACT
ACTGGGGTTTCTCATATGGAACTATTTTCCTAGCTGAGTTTATCCAAACTTTCCCAGGCCGTGTGGGAAGAGTTCTTGCCGATGGTGTTTTCGA
CGCAAAGGCAAATGCACTCACATACGTTAGCCAACTTCCCAACGATCAACTCAGTGTTCGTGCTTCGTTGAACGATTTTGCAGCTTTCTGCACC
ACCGCCGGTAGTAAAGGTTGCTCTTTTGCCACCGCCCCTACTGGAACCACAGGTACTGTTGCTACCAGACTGGACAACATAATGAAGGATATGT
TCCTCAATCCTATTGTTGCTTCGGGCTTAAGCATCA

>Bcin15g03910(MLST6), partial sequence [organism=Botrytis cinerea, strain D12_H_BioH01]
GCCAAAACACAAAATCATCCAACGATGAAGATGATACTCCACTTCCCTTGATTATTTGGCATGGACTCGGCGATAATTACAAAGCGGATGGTCT
TGCGCAAGTTGGAAAACTAGCTGAAGCTATTCATCCTGGGACTTTTGTCTACAATATTCATGTAGATGAGGATGCATCTGCAGATAGGACAGCT
ACCTTCTTTGGAAATCTCACTCGTGAGTACATCCCCTATTTTCCCTTTAAATACCATACTAACTCTCTTACCAAGTTCAAATCGAAAAGGTCTG
CGAAGACCTCGCCTCCCATCCTATTCTCTCTACCGCGCCCGCCGTCGACGCAATTGGATTCTCCCAAGGCGGCCAATTCTTGCGCGGTTACATA
TCCCGCTGCAATGCTCCACCCATCCGCTCTCTCCTGACCTTCGGTTCCCAACACAACGGCATTTCTGCCTTCCAAGCCTGTGGTCCTGCCGATT
TCCTCTGTCGCGGTGCTCAAACCCTTTTGCGATCCAACACCTGGTCAACCTTTGTCCAATCTCGTCTCGTACCCGCTCAATACTTCAGAGATCC
GGAAAACCTAGACTCTTACCTTGAATATTCCAATTTCCTTGCCGACATCAATAATGAGCGCGTTCTCAAGAACCAAACATATAAATCCAACATG
GAAAAATTGGAACGATTCGTAATGTATGTCTTTGAAGACGATACAACTGTCATCCCTAAGGAAAGTGGATGGTGGGCTGAAGTCAACGGCACGG
AAGTTACACCACTGAAAGAAAGAGCCATTTATAAAGAAGATTGGCTAGGTTTAAAGACATTGGATGAGGCCGGAAAATTAGTTTTCGAAACCAT
TCCAGGGGGACATATGACGTTAGGAGAGGAGATGCTAGAGAAGGCTTTCAAAGAGTATTTTGGTCCAGCAGGGAAGAAATTTGGG

>Bcin16g03460(MLST7), partial sequence [organism=Botrytis cinerea, strain D12_H_BioH01]
ATGAACCTCTTGATTTGAACTCTTCATTAATTGCAAGTGATGAACCCCTATTCTGCCCAGAGGATAGTTACAAGACGTATATCATTAGTCGAGA
ACCACTCATGATATATATTGACGGATTTTTGAAAGCGAATGAAAGTAAACATTTGGTTGATGTTAGGTGTGTTATTTATTCTGATGAAATGAAC
AAGAGAGACTGATGAGATAGTGAGCCGCTTTATGAACCGTCAACTGTTTCTCACGGACAGGAAGTTACCATTGATCTTTCAGTTCGGAATTCTG
AAGTGGCGGTTTTAGAGAGGGATGAGGTGGTCAGGTGTATTGAGCATAGAGCGAGGGCATTTCAGGGGTGGAGGGGCGAGATGGGGATTGAGAA
GTTGAGGACGCAGAGGTATGGCGTTGGAGGACATTATGGGATGCATTTGTAAGTTTTGGGGGATTGACGAAGGCTTCTGTCTATTTTCTACCAG
TACGGATCTTGAAAGAAAGATAGCATGAACACGAGGGCTAATAACTAGGAAATAGCGATTGGAGCGGAGGTAAACGTGGCATAGACCGATTTAG
TACTTTCATGGTCTATGTCGACGTATCCTCTGATATCGAAGGTGGAGGAACGGAATTCCCACGTATTGTGGGACCAAAAGGAGGAAGGTGGGAG
GACTTCCTGGAAACTACGGAAGCATTGGATCTAAGAACTGGAGAAAATGTAACAGTAGAAGGGGTGACATTCAAACCAATCAAGGGAAATGCCG
TATTCTGGGAAAATACTGACAACAACGGGAGGGGCTATGAT

>Bcin12g03020(MLST8), partial sequence [organism=Botrytis cinerea, strain D12_H_BioH01]
CGATTGGCTGCGAAGAAAACTGCGCAGCACCGAGTCACCAAAAGCACGAATAAACCATCGGCAAGGGAAATATTCTCTGAACCACAACATGACA
CGAGCGCGGAAGAGTATATCGGGCGAGAAGCTTCATCAAGAGCACCAAAGCGACAACGAGTCGATGATAATTATAACTCTTACGGTGGAAGAAA
TGAAAATACAGCAGCTTATGCTTCCGGGAAACTTCCATCTGGAAGTATAAATGTTGGTGGAGGTAGGAAGACCACTTTTCAAGAAGAACCACGA
ACGGCATTTGTCGCTGGCAAGTTGCCCCCTGGAAGTATCAACATTGGTGGAAAGAAGGCTACCCAAATCGAGGACGAGGGTAGAGCAGCTTATG
CTTCCGGAAAATTGCCTCCAGGAAGTATAAACTCTGGCGCAAAGAAGGCGATTTCATTCCAAGATGAAACGAGACCGGCTTATGTCTCCGGAAA
GCTTCCACATGGTAGTATCGACGGTATGCGAAACCGTGAAATGGCTGCCGTCCACCGCGAAATTGCTGAGGGTGGAGGAAACCAGGCCAGGTT
GTCTCTTCTCTATTCACATTCAATCCTACTTCAAAGAAAACTTTTGATGAACCCGAAGAACAAGCGGAACCGGCAAAACCATCCAATGCGCCTT
TGACAGAGGAAATGGCGACTTTCACCAATCTAGGGTTATCGAGAAGGCTTGCAGCCCATCTATCGACTAAACTCGATATGAAAGCTCCGACCGC
CATTCAAAAAGCATCTGTACAGCAGTTGGTATCGGACGATAGCGATGCTTTCATACAAGCAGAGACTGGATCTGGAAAAACTTTGGCATATCTA
CTACCTATAGTCGAGCGAATATTAGCATTGAGTGAGAATGGCGTACAAA

>Bcin02g07770(MLST9), partial sequence [organism=Botrytis cinerea, strain D12_H_BioH01]
CAGCTTTCCCTTTCGGTCTTGGCATCTACAGTCATTGCCATCCCTACACCATCACAACTTGAGTCTCGGGCCGTTATCGATTCCGATGCCGTTG
TAGGATTTGCCGAAACTGTTCCCAGTGGGACCGTAGGAACAGTTTATGAGGCATATAAACCATTCCTTAAAGTCGTAAATGGATGCGTACCATT
CCCTGCCGTCGATGCATCGGGTAACACAGGGTATGTCCTTATATCTTTCTCTTCCACACGATTGCTATTGAGTCTCTAACATATTTAGTGGTG
GTTTGTCACCAACTGGCAGTAGCAATGGTGGTTGCAGCAGCAGTACCGGTCAAGTATATGTTCGAGGAGGACAAAGCGGATCAAACTACGCCAT
CATGTACTCCTGGTAAGTTCTCTCTAAACTTCTCCTTATAGATCCAACCTAACAAAATCTTAGGTACATGCCAAAGGACGAGCCCTCAACCGGT
ATTGGTCACCGTCACGATTGGGAAGGTGTAATTGTCTGGCTCTCCAGCGCCACCGCCACAACTGCCGACAACATCTTAGCCGTTTGTCCTTCCG
CCCACGGAGGCTGGGATTGTTCCACGGATGGCTATTCCCTTTCTGGTACCAGCCCTCTTATCAAGTACGAAAGTATCTGGCCCGTCGATCACTC
AATGGGTCTTACTAGTACTGTTGGTGGAAAACAACCTATGATTGCTTGGGAGTCTTTACCAACTGCTGCTCAAACTGCTCTTGAGAACACCGAT
TTCGGTGCTGCGAATGTTCCATTCATTCCGGCTGTTTTCACAGACAATCT

>Bcin04g02090(MLST10), partial sequence [organism=Botrytis cinerea, strain D12_H_BioH01]
CGGAGGATGATATGGCAAAGTCTATGATTACCAAAGCATTTGTAGGCATGAGTAGTAAACTGTCGAATATGACTATGAATGATGTTTACAAGCC
CTACATCCATGTAAGAAATGTAGAATAGAGAGATCAGTAACTGGAACTAATATCGTTTGTAGGCTTTCAAGTTACTTACGCAGTTCAACCCAAT
CACTACAGCTATTGCCGAATCCCCACTGTTTCAAATGGCCGTCTCAGCAAATACCATCGAAAAGTACACACTGCTAGGCCCTTTCTTCAGAATA
TCTCCTCTGCAACAGGAAGTTACCAGGGAATACTTCAGTGCGCCAAAGACGATAGATAGGCGACACATTGCCACATCTCAAGATGCGTTACGAT
TGACCTTACAAACCCATCAAAAAGATTTACTTGATATCATCAACCACTTTGTTCGAGCAAGTCCAATCGCAAAAAGCAAAACCCTGGATTGGTT
CGCCTACATTGTGAATCAAAATCACAAGCGTCGAGCACTTCAGGTAGACCCGAAAGAAGTGTCTTCTGATGGCTTTATGCGCAATGTCACTGTC
GTTCTAGATGGTCTTTGTGAGCCATTCATGGATACCACATTCTCGAAAATTTCGAAGATTGATATTGATTATCTAAGACGTGCGCCTCGTGTAG
ATATCAAGGACGAGACCAAGTTGAACGCTGACGAGAAGGCTTCTGAGAAGTATTATGAGGACACTGTTCCTGGCACTTCTAATTTCATCTCTGA
GGTCTTCTTTCTCACATTGGCTGCTCATCATTATGGTAGTGAAGCTCTTAATGCCACGCATAAGAGTCTGGAGAAAGACATCAAATATATTCAA
AAGCAATTGACTGCCGTTGAAGCA

>Bcin01g07220(MLST1), partial sequence [organism=Botrytis cinerea, strain D13_MR_S9]
ATGGCATATCTTGTTTCTTTTCGCATTATGCTTTGGAACGTGCTGGACAAGCTTGGGTTAGTGCTCTGCGTGTAGAAGCACTAAAGAAGATTCT
CGCACAACCGAAGTCATGGTTTGAGGAATCCAGGAATTCACCTGGCCGGTTGAACGAAGTTTTGGATAGGAACTCTGAGGAAATGCGTAATCTC
GTTGGCCGCTTTGCTGGTATTGTATTCACAGCATTTTTTATGCTATTGATATCAATCATTTGGGCTTTCGTGAATACATGGAAACTGACATTAG
TCTCAATGGCGACTGGGCCAGTTATATACGCTGTCACCAAAACGTTCAATCGCGTGAGTGGAAAATGGGAAAACAAGTGCAACTACGCATCTGA
AATGACCACTGGCATATTTTCAGAGACTTTCTCCAACATCAAAGTGGTTCGGGCTTTTACTCTGGAAACTTACTTTGAGACAAAACACACCAAA
GCTACAGAAGAACTCTATAAAATTGGACTAATACGAGCAAACTACTCGGGATTGCTGTGGGGATTGACAGATGCGATGTCATTCTTCATCACTG
CAACTATCTTTTATTATGCCACGGTTCTCATTACCAAGAGAGAGATCAGTATCGCGACTGCACTGCAGACTGTCAATCTTCTATTATTTGGTAT
TCCTAATAGTACGAATATGCTGGCCATGATACCACAAATCAACTCTTCTCGCGTTACAGCTACGCATATGCTTGCATTAGCCAATCTCGATTCA
TCTTCCTCCCACGAAAATAAAGGAGCCGAACGGCTTTCGACAATCTTTCCAATCAAATTCAACAGTCTCTCATTCACATATCCTACTCGTCCTG
AAAAACGAACGATATCATCCTTTTCTCTTTCCCTGATTCCTAACTCAACAACTGCACTTGTCGGACCCTCCGGCTCCGGAAAATCTACAATAGC
TGCTCTGCTCGTTGGTCTCTATCCGCCAGATACTTCAACACCTCCACCGTTGACATTCAATCGTGTCTCCATAAGTAACTGTCACATTCCGTCT
CTCCGGGCTTCTCTCTCACTCGTCCCACAAATACCGATTCTATTTCCAGCTACCATTCTCCATAACATCATTTATGGTCTCCCAGAATCTTCTC
CTTGTGCTAGTCTTCCATCTGCTATTGCATCAGCAAAAGATGCTGGGATCCATGAATTTATCACATCGCTTCCACAAGGTTATGATACTAT

>Bcin05g07690(MLST2), partial sequence [organism=Botrytis cinerea, strain D13_MR_S9]
ACCACTATCACCAAGTCCTTTTCTGCACTTTCCTCATCAAAAACGAACGAGAACATCATCAATAAATACTTTGCTGAAGTAATTGGGTTTTATT
TTGGCCAGGATGCCTTCTCTCTACGACAGCAAGCCTTCGATGATATGTTGTGGGTAGTTCTTGGCTGGCTGGATACTGTCAAATTCATTGATTT
ACATTCTGAATTGCACTATTCAAACGACTCTCAGCCAGAATGGTACGGACAACAATATAAACCTGCATTTGCACATCGAGCGCGACTATTTTGG
GAATTGGCTTCACAAGGATGGGATACTACTCTCTGTGGTGGTGGGATGATATGGTCACCATACCTTACTCCATACAAGAACGCAATTACCAATG
AACTCTATATCGCAGCTTCGATATCGATGTACCTATATTTCCCCGGAGATGACAATCAATCCCCATTTATGATTTCCAACCCTTCATATCCACC
TCACGATCCGAAATATCTACAGGCAGCTGTTGATGCTTACAAATGGCTGAATGGTTCCAACATGACGGATTCACAAGGATTATATGTCGACGGG
TACCATATCTCGAATCTTTCTGGCGGTGAAAACACCCATTGCGATTCTAGAAATGAGATGGTATATACCTACAATCAAGGTGTTTTGCTTACTG
GACAACGTGGTTTGTATGACGCAACCGCCGCACGATCATACCTTGTAGATGGCCACAAACTCATCGCGAATGTTATTAATGCCACAGGCTATGA
CCTGAAACACAATGTTGTCATCTCACCGCCACCCAAAGATGGTTCCGCATTGGCAAAGTGGTTTGGCCTGGGTAGGAATGGAATACTGGAAGAA
GGATGCGATTCAAGTGCTTCGTGTTCTCAAAATGGACAAACTTTCAAAGGCATATTCTCTCATCACTTGATTGCGTTCTGTAGTGATTTGCCAG
GGGAGCCTATTGCAGGGACGAAGGAAAGCTTAGAACTCGACAGAGTGTGGCATTCTGACAAATGCTCACAGTATACAAAAT

>Bcin06g01710(MLST3), partial sequence [organism=Botrytis cinerea, strain D13_MR_S9]
GTGAGTCTGACTTTTGTGTTTGAGCGTTAAGCTAGACACTGATGTACCAAGGCAATCACCTAATTTCATGGCGTTCTTCCCTGCATCATCTACC
TACCCTGGAATGCTGGGAGAATTATACTCAGCAGCTTTCACAGCACCTGCTTTCAATTGGATCTGTTCCCCTGCCGTGACAGAATTGGAGACGG
TTGTAATGGATTGGCTGGCCAAGCTTCTCAATCTCCCAGACTGTTATTTGTCTTCGACTCATGGTGGTGGTGTTATCCAAGGATCAGCCTCGGA
AGCTATCGTTACCGTTATGGTTGCTGCCCGCGATAAATATCTTCGTGAAACTACTGAAGGTCTGTCGGGCATTGAACTCGAGGATGCGATTGCA
TATAAGAGGAGTAAGCTAGTTGCACTAGGAAGCGAAATGGCACACAGCTCCACGCAGAAAGCAGCGCAGATAGCTGGCGTTAGATTCCGATCGA
TTCCAGTACTCGCATCCAATGATTTCGCCATGACGGGTGATGATTTAGAGAAGGTATTGGAAGAATGCAAATCTCAAGGATTGGAACCCTTCTA
TCTAACTTCGACTTTGGGAACAACATCTACATGCGCAGTTGACGACTTCGCATCTATTGCAACAGTACTTTCAAAATATGCACCTCCAGATGTT
GCAGGCGAGATCTGGGTTCACGTCGATGCTGCTTATGCAGGTGCAGCTTTGGTTTGCCCTGAATACCATCATCTAACATCGTCCTTCCAGCATT
TCCATTCCTTCGATATGAACATGCACAAATGGCTTCTGACAAATTTCGACGCTTCTTGTCTATATGTCAAGAAACGCAAAGATCTGATAGATGC
ACTCTCCATAACACCAAGTTATCTTCGCAACGAGTTTTCAGAGAGTGGACTCGTAACCGACTATCGGATTGGCAAATTCCTCTCGGAAGACGCT
TCCGAAGCTTAAAGATTTGGTTTGTCCTCAGAACCTAC

>Bcin09g03030(MLST4), partial sequence [organism=Botrytis cinerea, strain D13_MR_S9]
ACCCTTCAACCCCACCGCAGCTCCTATACCGAAAGCGAGTATCCTACCAATTCTCCTTCCACCTGCGACTTTAAGACCATTGGCTTTCCGCACT
TTCACAAAAAAGCATAGTTTAACATTGACGTCGTCGGCATTACAAGTGTTGGCTACTTTTATTGGAAAGCATTGTGGGACAGGATGGAGGGAAG

AAGGACTGGCAGAGAGAGTCTTGGAGGAGGTCGCCAAGAGTGGAAGAATAGGAGTGGCGGTGTCATTGTCGAGGGCGAGGGAACGGAATTGAAG
GAGATTCTGAAAGCTTTGGAAGGGAATATGAGTGGTGGAAGGATAGTCATAGGAAGAGAGCTAAGCCGGCAGAATAGTTTAGTACTGGGATCAT
CACAATATGGAGAGGTCAATCATACAAGACTTGGGCTACGGCCAGGGAATATACCCAGAGAGGATAGTCAGTCAAGTTTGGGAATGTCAACGTT
GGAGGTCAATGACGAGGAGGATGAGGATGGCCTGATGGATCCAAGAAGGTGGTTAAAAGTCATTGATGCATTTGAGCAACCTCGACTGGTGTAC
AATGTTGCTAAAAAAGCACTTTGATAGGTATGTTTCAATGATAAAATTTTATCTGAATCTACTAACTCAGTACAGAGATACCTCCAAACCTTCA
TTGTTCCCACCTGCGTCTCATAAAACACTCCTCTTCCAAAACCGCTATAATGTTATCCATCAACGTCTCCTTCGCAATGAGTCTTTTCAAACGC
CCGCTTTTCAAGGTGGCAAATCTTCCCTTCAACGCAGCACGTCCGCCATTACCACCCAACAACAATCATACAAATTAACGCCGATAGCTAATCT
TCTCGGTCGCAATCGCAGCTCTCATATGCTTCTCGGTCTCCTCAGTATTTCACCCACTGGTACCCTCGCCATCAATGACCTGACGGGCAGTATC
GCTCTCGATCTTACACACGCAGCAGCCATTC

>Bcin11g01310(MLST5), partial sequence [organism=Botrytis cinerea, strain D13_MR_S9]
GCTGACATGGACCTCATGTGGAACCGGCGTAGAATGCGCAACGCTTGAAGTTCCGCTCGAATATGGCGATGCAACGTCAACGGCAAAAGCCAGT
GTTGCGCTTGCTCGTTATCCTGCCACTGTTGCCGCGAGCAAGAAGCTCGGGTCTCTCTTGATAAACCCCGGTGGACCCGGTGCCTCTGGTGTTG
GCTTTGTGCAGTCTGGAGCCGGTGCCGCCGTCTCGACACTGAGTGGTGGATTGTACGATATCATCGGATGGGATCCACGTGGAACCGGTGCTTC
GGCTCCTATTTTGGAATGTTTTGCAAATGCCAGTGCGGAGTATGATTTTAACAACGCGTTTCCATCTGCTCCGAATCTCTGGCTCGGACAATTT
GCGAATGCCAGCGCAAATTCTGCTGTTAGCTCTGCTATCACATCCTTTGACACTTCTGTCGCTGCTCTTGCAAAAGCTTGCGTGGCTCAGAAAT
CTCCCGCTCTTTACACCTCAACAGCAGCATATGTTGCTCGAGACATGGCAGCGATAGTCGATGCATTGGATGGGACCTCTGCAAAACTTAACTA
CTGGGGTTTCTCATATGGAACTATCTTCCTAGCTGAGTTTATCCAAACTTTCCCAGGCCGCGTGGGAAGAGTTCTTGCCGATGGTGTTTTCGAC
GCAAAGGCAAATGCACTCACATACGTTAGCCAACTTCCCAACGATCAACTCAGTGTTCGTGCTTCGTTGAACGATTTTGCAGCTTTCTGCACCA
CCGCCGGTAGTAAAGGTTGCTCTTTTGCCACCGCCCCTACTGGAACCTCAGGTACTGTTGCTACCAGACTGGACAACATAATGAAGGATATGTT
CCTCAATCCTATTGTTGCTTCGGGCTTAAGCATCA

>Bcin15g03910(MLST6), partial sequence [organism=Botrytis cinerea, strain D13_MR_S9]
GCCAAAACACAAAATCATCCAACGATGAAGATGACACTCCACTTCCCTTGATTATCTGGCATGGACTCGGCGATAATTACAAAGCGGATGGTCT
TGCGCAAGTTGGAAAACTAGCTGAAGCTATTCATCCTGGGACTTTTGTCTACAATATTCATGTAGATGAGGATGCATCTGCAGATAGGACAGCT
ACCTTCTTTGGAAATCTCACTCGTGAGTACATCCCCTATTTTTCCTTTAAATATCATACTAACTCTCTTACCAAGTTCAAATCGAAAAGGTCTG
CGAAGACCTCGCCTCCCATCCTATTCTCTCTACCGCGCCCGCCGTCGACGCAATTGGATTCTCCCAAGGCGGCCAATTCTTGCGTGGTTACATA
TCCCGCTGCAATGCTCCACCCATCCGCTCTCTCCTGACCTTTGGTTCCCAACACAACGGCATTTCTGCCTTCCAAGCCTGTGGTCCTGCCGATT
TCCTCTGTCGCGGTGCTCAAACCCTTTTGCGATCCAACACCTGGTCAACCTTTGTCCAATCTCGTCTCGTACCCGCTCAATACTTCAGAGATCC
GGAAAACCTAGACTCTTACCTTGAATATTCCAATTTCCTTGCCGACGTCAATAATGAGCGCGTTCTCAAGAACCAAACATATAAATCCAACATG
GAAAAATTGGAACGATTCGTAATGTATGTCTTTGAAGACGACACAACTGTCATTCCTAAGGAAAGTGGATGGTGGGCTGAAGTCAACGGCACGG
AAGTTACACCACTGAAAGAAAGAGCCATTTATAAAGAAGATTGGCTAGGTTTAAAGACATTGGATGAGGCCGGAAAATTAGTTTTCGAAACCAT
TCCAGGGGGACATATGACGTTAGGAGAGGAGATGCTAGAGAAGGCTTTCAAAGAGTATTTTGGTCCAGCAGGGAAGAAATTTGGG

>Bcin16g03460(MLST7), partial sequence [organism=Botrytis cinerea, strain D13_MR_S9]
ATGAACCTCTTGATTTGAACTCTTCATTAATTGCAAGTGATGAACCCCTATTCTGCCCAGAGGATAGTTACAAGACGTATATCATTAGTCGAGA
ACCACTCATGATATATATTGACGGATTTTTGAAAGCGAATGAAAGTAAACATTTGGTTGATGTTAGGTGTGTTATTCATTCTGATGAAATGAAC
AAGAGAGACTGATGAGATAGTGAACCGCTTTATGAACCATCTACTGTTTCTCACGGACAGGAAGTTACCATTGATCCTTCAGTTCGGAATTCTG
AAGTGGCGGTTTTAGAGAGGGATGAGGTGGTCAGGTGTATTGAGCATAGAGCGAGGGCATTTCAGGGGTGGAGGGGCGAGATGGGGATTGAGAA
GTTGAGGACGCAGAGGTATGGGGTTGGAGGACATTATGGGATGCATTTGTAAGTTTTGGGGGATTGACGAAGGCTTTTGTCTATTTTCTACCAG
TACGGATCTTGAAAGAAAGATAGCATGAACACGAGGGCTAATAACTAGGAAATAGCGATTGGAGCGGAGGTAAACGTGGCATAGACCGATTTAG
TACTTTCATGGTCTATGTCGACGTATCCTCTGATATCGAAGGTGGAGGAACGGAATTCCCACGTATTGTGGGACCAAAAGGAGGAAGGTGGGAG
GACTTCCTGGAAACTACGGAAGCATTGGATCCAAGAACTGGAGAAAATGTAACAGCAGAAGGGGTGACATTCAAACCAATCAAGGGAAATGCCG
TATTCTGGGAAAATACTGACAACAACGGGAGGGGCTATGAT

>Bcin12g03020(MLST8), partial sequence [organism=Botrytis cinerea, strain D13_MR_S9]
CGATTGGCTGCGAAGAAAACTGCGCAGCACCGAGTCACCAAAAGCACGAATAAACCATCGGCAAGGGAAATATTCTCTGAACCACAACATGACA
CGAGCGCGGAAGAGTATATCGGGCGAGAAGCTTCATCAAGAGCACCAAAGCGACAACGAGTCGATGATAATTATAACTCTTACGGTGGAAGAAA
TGAAAATACAGCAGCTTATGCTTCCGGGAAACTTCCATCTGGAAGTATAAATGTTGGTGGAGGTAGGAAGACCACTTTTCAAGAAGAACCTCGA
ACGGCATTTGTCGCTGGCAAGTTGCCCCCTGGAAGTATCAACATTGGTGGAAAGAAGGCTACCCAAATCGAGGACGAGGGTAGAGCAGCTTATG
CTTCCGGAAAATTGCCCCCAGGAAGTATAAACTCTGGCGCAAAGAAGGCGATTTCATTCCAAGATGAAACGAGACCGGCTTATGTCTCCGGAAA
GCTTCCACATGGTAGTATCGACGGTATGCGAAACCGTGAAATGGCTGCCGTCCACCGCGAAATTGCTGAGGGTGGGAGGAAACCAGGCCAGGTT
GTCTCTTCTCTATTCACATTCAATCCTACTTCAAAGAAAACTTTCGATGAACCAGAAGAACAAGCGGAACCGGCAAAACCATCCAATGCGCCTT
TGACAGAGGAAATGGCGACTTTCACCAATCTAGGGCTATCGAGAAGGCTTGCAGCCCATCTATCGACTAAACTCGATATGAAAGCCCCGACGGC
CATTCAAAAAGCATCTGTGCAGCAGTTGGTATCGGACGATAGCGATGCTTTCATACAAGCAGAGACTGGATCTGGAAAAACTTTGGCATATCTA
CTACCTATAGTCGAGCGAATATTAGCATTGAGTGAGAATGGCGTACAAA

>Bcin02g07770(MLST9), partial sequence [organism=Botrytis cinerea, strain D13_MR_S9]
CAGCTTTCCCTTTCGGTCTTGGCATCTACAGTCATTGCCATCCCTACACCATCACAACTTGAGTCTCGGGCCGTTATCGATTCCGATGCCGTTG
TAGGATTTGCCGAAACTGTTCCCAGTGGGACCGTAGGAACAGTTTATGAGGCATATAAACCATTCCTTAAAGTCGTAAATGGATGCGTACCATT
CCCTGCCGTCGATGCATCGGGTAACACAGGGTATGTCCTTATACCTTTCTCTTCCACACGATTGCTATTGAGTCTCTAACATATTTTAGTGGTG
GTTTGTCACCAACTGGCAGCAGCAATGGTGAATGCAGCAGCAGTACCGGTCAAGTATATGTTCGAGGAGGACAAAGCGGATCAAACTACGCTAT
CATGTACTCCTGGTAAGTTCTCTTTAAACTTCTCCTTATAGATCCAACCTAACAAAATCTTAGGTACATGCCAAAGGACGAGCCCTCAACCGGT
ATTGGTCACCGTCACGATTGGGAAGGTGTAATTGTCTGGCTCTCCAGCGCCACCGCCACAACTGCCGACAACATCTTAGCCGTTTGTCCTTCCG
CCCACGGAGGCTGGGATTGTTCCACGGATGGCTATTCCCTTTCTGGTACCAGCCCTCTTATCAAGTACGAAAGTATCTGGCCCGTCGATCACTC
AATGGGTCTTACTAGTACTGTTGGTGGAAAACAACCTATGATTGCTTGGGAGTCTTTACCAACTGCTGCTCAAACTGCTCTTGAGAACACCGAT
TTCGGTGCTGCGAATGTTCCATTCATTCCGGCTGTTTTCACAGATAATCT

>Bcin04g02090(MLST10), partial sequence [organism=Botrytis cinerea, strain D13_MR_S9]
CGGAGGATGATATGGCAAAGTCTATGATTACCAAGGCATTTGTAGGCATGAGTAGTAAATTGTCGAATATGACTATGAATGATGTTTACAAGCC
CTACATCCATGTAAGAAATGTAGAATAAAGAGATCGGTAACTGGAACTAATATCGTTTGCAGGCTTTCAAGTTACTTACGCAGTTCAACCCAAT
CACTACAGCTATTGCCGAATCCCCACTATTTCAAATGGCTGTCACAGCAAATACCATCGAAAAGTACACACTGCTAGGCCCTTTCTTCAGGAAT
ATCTCCTCTGCAACAGGAAGTTACCAGGGAATACTTCAGTGCGCCAAAGACGATAGATAGGCGACACATTGCCACATCTCAAGATGCGTTACGA
TTGACCTTACAAACCCATCAAAAGATTTACTTGATATCATCAACCACTTTGTTCGAGCAAGTCCAATCGCAAAAGCAAAACCCTGGATTGGTTC
GCCTACATTGTGAATCAAAATCACAAGCGTCGAGCACTTCAGGTAGACCCGAAGAAGTGTCTTCTGATGGCTTATGCACAATGTCACTGTCGTT
CTAGATGGTCTTTGTGAGCCATTCATGGATACCACATTCTCAAAAATTTCGAAGATTGATATTGATTATCTAAGACGCGCGCCTCGTGTAGATA
TCAAGGACGAGACCAAGTTGAACGCTGATGAGAAGGCTTCTGAGAAGTATTATGAGGACACTGTTCCTGGCACTTCTAATTTCATCTCTGAGGT
ATTCTTTCTGACATTGGCTGCTCATCATTATGGTAGTGAAGCTCTTAATGCCACGCATAAGAGTCTGGAGAAGACATCAAATATATTCAAAAGC
AATTGACTGCCGTTGAAGCA

>Bcin01g07220(MLST1), partial sequence [organism=Botrytis cinerea, strain D13_B_KF1_25]
ATGGCATATCTTGTTTCTTTTCGCATTATGCTTTGGAACGTGCTGGACAAGCTTGGGTTAGTGCTCTGCGTGTAGAAGCACTAAAGAAGATTCT
CGCACAACCGAAGTCATGGTTTGAGGAATCCAGGAATTCACCTGGCCGGTTGAACGAAGTTTTGGATAGGAACTCTGAGGAAATGCGTAATCTC
GTTGGCCGCTTTGCTGGTATTGTATTCACAGCATTTTTATGCTATTGATATCAATCATTTGGGCTTTTGTGAATACATGGAAACTGACATTAGT
CTCGATGGCAACTGGGCCAGTTATATACGCTGTCACCAAAACATTCAATCGCGTGAGTGGAAAATGGGAAAACAAGTGCAACTACGCCTCTGAA
ATGACCACTGGCATATTTTCAGAGACTTTCTCCAACATCAAAGTGGTTCGGGCTTTTACTCTGGAAACTTACTTTGAGACAAAACACACCAAAG
CTACAGAAGAACTTTATAAAGTTGGACTAATACGAGCAAACTACTCGGGATTGCTGTGGGGATTGACAGATGCGATGTCATTCTTCATCACTGC
AACTATCTTTTATTATGCCACGGTTCTCATTACCAAGAGAGAGATCAGTATCGCGACTGCACTACAGACTGTCAATCTTCTATTATTTGGTATT
TCTAATAGTACGAATATGCTGGCCATGATACCACAAATCAACTCTTCTCGCGTTACAGCTACGCATATGCTTGCATTAGCCAATCTCGATTCAT
CTTCCTCCCACGAAAATAAAGGAACCGAACGGCTTTCGACAATCTTTCCAATCAAATTCAACCGTCTTTCATTCACATACCCTACTCGTCCTGA
AAAACGAACGATATCATCCTTTTCTCTTTCCCTGATTCCTAACTCAACAACTGCACTTGTCGGACCCTCCGGCTCCGGAAAATCTACAATAGTT
GCTCTGCTCATTGGTCTCTATCCGCCAGATACTTCAACACCTCCACCGTTGACATTCAATCGCGTCTCCATAAGTAACTGTCACATTCCGTCTC
TCCGGGCTTCTCTCTCACTCGTTCCACAAATACCGATTCTATTTCCAGCTACCATTCTCCATAACATCATTTATGGTCTCCCAGAATCTTCTCC
TTGTGCTAGTCTTCCATCAGCTATTGCATCAGCAAAAGATGCTGGGATCCATGAATTTATCACATCGCTTCCACAAGGTTATGATACTAT

>Bcin05g07690(MLST2), partial sequence [organism=Botrytis cinerea, strain D13_B_KF1_25]
CACCACTATCACCAAGTCCTTTCTGCACTTTCCTCATCAAAAACGAACGAGAACATCATCAATAAATACTTTGCTGAAGTAATTGGGTTTTATT
TTGGCCAGGATGCCTTCTCTCTACGACAGCAAGCCTTCGATGATATGTTGTGGGTAGTTCTTGGCTGGCTGGATACTGTCAAATTCATTGATTT
ACATTCTGAATTGCACTATTCAAACGACTCTCAGCCAGAATGGTACGGACAACAATATAAACCTGCATTTGCACATCGAGCGCGACTATTTTGG
GAATTGGCTTCACAAGGATGGGATACTACTCTCTGTGGTGGTGGGATGATATGGTCACCATACCTTACTCCATACAAGAACGCAATTACCAATG
AACTCTATATCGCAGCTTCGATATCGATGTACCTATATTTCCCCGGAGATGACAATCGATCCCCATTTATGCTTTCCAACCCTTCGTATCCACC
TCACGATCCGAAATATCTACAGGCAGCTGTTGATGCTTACAAATGGCTGAATGGTTCCAACATGACGGATTTACAAGGATTATATGTCGACGGG
TACCATATCTCGAATCTTTCTGGCGGTGAAAACACCCATTGCGATTCTAGAAATGAGATGGTATATACCTACAATCAAGGTGTTTTGCTTACTG
GACAACGTGGTTTGTATGACGCAACCGCCGCACGATCATACCTTGTAGATGGCCACAAACTCATCGCGAATGTTATTAATGCCACAGGCTATGA
CCTGAAACACAATGTTGTCATCTCACCGCCACCCAAAGATGGTTCCGCATTGGCAAAGTGGTTTGGCCTGGGTAGGAATGGAATACTGGAAGAA
GGATGCGATTCAAGTGCTTCGTGTTCTCAAAATGGACAAACTTTCAAAGGCATATTCTTTCATCACTTGATTGCGTTCTGTAGTGATTTGCCAG
GGGAGCCTATTGCAGGGACGAAGGAAAGCTTAGAACTCGACAGAGTGTGGCATTCTGACAAATGCTCACAGTATACAAAATG

>Bcin06g01710(MLST3), partial sequence [organism=Botrytis cinerea, strain D13_B_KF1_25]
GTGAGTCTGACTTTTGTATTTGAGCGTTAAGATAGACACTGATATACCAAGGCAATCACCTAATTTCATGGCGTTCTTCCCTGCATCATCTACC
TACCCTGGAATGCTGGGAGAATTATACTCAGCAGCTTTCACAGCACCTGCTTTCAATTGGATCTGTTCCCCTGCTGTGACAGAATTGGAGACGG
TTGTAATGGATTGGCTGGCCAAGCTTCTCAATCTCCCAGACTGTTATTTGTCTTCGACTCATGGTGGTGGTGTCATCCAAGGATCAGCCTCGGA
AGCTATCGTTACCGTTATGGTTGCTGCCCGCGATAAATATCTTCGTGAAACTACTGAAGGTCTGTCGGGCATTGAACTCGAGGATGCGATTGCA
TATAAGAGGAGTAAGCTAGTTGCACTAGGAAGCGAAATGGCACACAGCTCCACGCAGAAAGCAGCGCAAATAGCTGGCGTTAGATTCCGATCGA

TTCCAGTACTCGCATCCAATGATTTCGCCATGACGGGTGATGATTTAGAGAAGGTATTGAAAGAATGCAAATCTCAAGGATTGGAACCCTTCTA
TCTAACTTCGACTTTGGGAACAACACCTACATGCGCAGTTGACGACTTCGCATCTATTGCAACAGTACTTTCAAAATATGCACCTCCGGATGTT
GCAGGCGAGACCTGGGTTCACGTCGATGCTGCTTATGCAGGTGCAGCTTTGGTTTGCCCTGAATACCATCATCTAACATCGTCTTTCCAGCATT
TCCATTCCTTCGATGTGAACATGCACAAATGGCTTCTGACAAATTTCGACGCTTCTTGTCTATATGTCAAGAAACGCAAAGATCTGATCGATGC
ACTGTCCATAACACCAAGTTATCTTCGCAACGAGTTTTCAGAGAGTGGACTCGTAACCGACTATCGGGACTGGCAAATTCCTCTCGGAAGACGC
TTCCGAAGCTTAAAGATTTGGTTTGTCCTCAGAACCTACG

>Bcin09g03030(MLST4), partial sequence [organism=Botrytis cinerea, strain D13_B_KF1_25]
ACCCTTCAACCCCACCGCAGCTCCTATACCGAGAGCGAGTATCCTACCAATTCTCCTTCCACCTGCGACTTTAAGACCATTGGCTTTCCGCACT
TTCACAAAAAAGCATAGTTTAACATTGACGTCGTCGGCATTACAAGTGTTGGCTACTTTTATTGGAAAGCATTGTGGGACAGGATGGAGGGAAG
AAGGACTGGCAGAGAGAGTCTTGGAGGAGGTCGCCAAGAGTTGGAAGAATAGGAGTGGCGGTGTCATTGTCGAGGGCGAGGGAACGGAATTGAA
GGAGATTCTGAAAGCTTTGGAAGGGAATATGAGTGGTGGAAGGATAGTCATAGGAAGAGAGCTAAGCCGGCAGAATAGTTTAGTACTGGGATCA
TCACAATATGGAGAGGTCAATCATACAAGACTTGGGCTACGGCCAGGGAATATACCCAGAGAGGATAGTCAGTCAAGTTTGGGAATGTCAACGT
TGGAGGTCAATGACGAGGAAGATGAGGATGGCCTGATGGATCCAAGAAGGTGGTTAAAAGTCATTGATGCATTTGAGCAACCTCGACTGGTGTA
CAATGTTGCTAAAAAGCACTTTGATAGGTATGTTTCAATGATAAAATTTCATCTGAATCGACCAACTCAGTACAGAGATACCTCCAAACCTTCA
TTGTTCCCACCTGCGTCTCATAAAACACTCCTCTTCCAAAACCGCTATAATGTTATCCATCAACGTCTCCTTCGCAATGAATCTTTTCAAACGC
CCGCTTTTCAAGGTGGCAAATCTTCCCTTCAACGCAGCACGTCCGCCATTACCACCCAACAACAATCATACAAATTAACGCCGATAGCTAATCT
TCTCGGTCGCAATCGCAGCTCTCATATGCTTCTCGGTCTCCTCAGTATTTCACCCACTGGTACCCTCGCCATCAATGACCTGACGGGCAGTATC
GCTCTCGATCTTACACACGCAGCAGCCATTC

>Bcin11g01310(MLST5), partial sequence [organism=Botrytis cinerea, strain D13_B_KF1_25]
GCTGACATGGACCTCATGTGGAACCGGCGTAGAATGCGCAACGCTTGAAGTTCCGCTCGAATATGGCGATGCAACGTCAACGGCAAAAGCCAGT
GTTGCGCTTGCTCGTTATCCTGCCACTGTTGCCGCGAGCAAGAAGCTCGGGTCTCTCCTGATAAACCCCGGTGGACCCGGTGCCTCTGGTGTTG
GCTTTGTGCAGTCTGGAGCCGGTGCCGCCGTCTCGACACTGAGTGGTGGATTGTACGATATCATCGGATGGATCCACGTGGAACCGGTGCTTCG
GCTCCTATTTTGGAATGTTTTGCAAATGCCAGTGCGGAGTATGATTTTAACAACGCGTTTCCATCTGCTCCGAATCTCTGGCTCGGACAATTTG
CGAATGCCAGCGCAAATTCTGCTGTTAGCTCTGCTATCACATCCTTTGACACTTCTGTCGCTGCTCTTGCAAAAGCTTGCGTGGCTCAGAAATC
TCCCGCTCTTTACACCTCAACAGCAGCATATGTTGCTCGAGACATGGCAGCGATAGTCGATGCATTGGATGGGACCTCTGCAAAACTTAACTAC
TGGGGTTTCTCATATGGAACTATCTTCCTAGCTGAGTTTATCCAAACTTTCCCAGGCCGCGTGGGAAGAGTTCTTGCCGATGGTGTTTTCGACG
CAAAGGCAAATGCACTCACATACGTTAGCCAACTTCCCAACGATCAACTCAGTGTTCGTGCTTCGTTGAACGATTTTGCAGCTTTCTGCACCAC
CGCCGGTAGTAAAGGTTGCTCTTTTGCCACCGCCCCTACTGGAACCTCAGGTACTGTTGCTACCAGACTGGACAACATAATGAAGGATATGTTC
CTCAATCCTATTGTTGCTTCGGGCTTAAGCATCA

>Bcin15g03910(MLST6), partial sequence [organism=Botrytis cinerea, strain D13_B_KF1_25]
GCCAAAACACAAAATCATCCAACGATGAAGATGATACTCCACTTCCCTTGATTATCTGGCATGGACTCGGCGATAATTACAAAGCGGATGGTCT
TGCGCAAGTTGGAAAACTAGCTGAAGCTATTCATCCTGGGACTTTTGTCTACAATATTCATGTAGATGAGGATGCATCTGCAGATAGGACAGCT
ACCTTCTTTGGAAATCTCACTCGTGAGTACATCCCCTATTTTTCCTTTAAATATCATACTAACTCTCTTACCAAGTTCAAATCGAAAAGGTCTG
CGAAGACCTCGCCTCCCATCCTATTCTCTCTACCGCGCCCGCCGTCGACGCAATTGGATTCTCCCAAGGCGGCCAATTCTTGCGTGGTTACATA
TCCCGCTGCAATGCTCCACCCATCCGCTCTCTCCTGACCTTTGGTTCCCAACACAACGGCATTTCTGCCTTCCAAGCCTGTGGTCCTGCCGATT
TCCTCTGTCGCGGTGCTCAAACCCTTTTGCGATCCAACACCTGGTCAACCTTTGTCCAATCTCGTCTCGTACCCGCTCAATACTTCAGAGATCC
GGAAAACCTAGACTCTTACCTTGAATATTCCAATTTCCTTGCCGACATCAATAATGAGCGCGTTCTCAAGAACCAAACATATAAATCCAACATG
GAAAAATTGGAACGATTCGTAATGTATGTCTTTGAAGACGACACAACTGTCATTCCTAAGGAAAGTGGATGGTGGGCTGAAGTCAACGGCACGG
AAGTTACACCACTGAAAGAAAGAGCCATTTATAAAGAAGATTGGCTAGGTTTAAAGACATTGGATGAGGCCGGAAAATTAGTTTTCGAAACCAT
TCCAGGGGGACATATGACGTTAGGAGAGGAGATGCTAGAGAAGGCTTTCAAAGAGTATTTTGGTCCAGCAGGGAAGAAATTTGGG

>Bcin16g03460(MLST7), partial sequence [organism=Botrytis cinerea, strain D13_B_KF1_25]
ATGAACCTCTTGATTTGAACTCTTCATTAATTGCAAGTGATGAACCCCTATTCTGCCCAGAGGATAGTTACAAGACGTATATCATTAGTCGAGA
ACCACTCATGATATATATTGACGGATTTTTGAAAGCGAATGAAAGTAAACATTTGGTTGATGTTAGGTGTGTTATTTATTCTGATGAAATGAAC
AAGAGAGACTGATGAGATAGTGAGCCGCTTTATGAACCGTCAACTGTTTCTCACGGACAGGAAGTTACCATTGATCCTTCAGTTCGGAATTCTG
AAGTGGCGGTTTTAGAGAGGGATGAGGTGGTCAGGTGTATTGAGCATAGAGCGAGGGCATTTCAGGGGTGGAGGGGCGAGATGGGGATTGAGAA
GTTGAGGACGCAGAGGTATGGGGTTGGAGGACATTATGGGATGCATTTGTAAGTTTTGGGGGATTGACGAAGGCTTCTGTCTATTTTCTATCAG
CACGGATCTTGAAAGAAAGATAGCATGAACACGCGGGCTAATAACTAGGAAATAGCGATTGGAGCGGAGGTAAACGTGGCATAGACCGATTTAG
TACTTTCATGGTCTATGTCGACGTATCCTCTGATATCGAAGGTGGAGGAACGGAATTCCCACGTATCGTGGGACCAAAAGGAGGAAGGTGGGAG
GACTTCCTGGAAACTACGGAAGCATTGGATCCAAGAACTGGAGAAAATGTAACAGTAGAAGGGGTGACATTCAAACCAATCAAGGGAAATGCCG
TATTCTGGGAAAATACTGACAACAACGGGAGGGGCTATGAT

>Bcin12g03020(MLST8), partial sequence [organism=Botrytis cinerea, strain D13_B_KF1_25]
CGATTGGCTGCGAAGAAAACTGCGCAGCACCGAGTCACCAAAAGCACGAATATACCATCGGCAAGGGAAATATTCTCTGAACCACAACATGACA

CGAGCGCGGAAGAGTATATCGGGCGAGAAGCTTCATCAAGAGCACCAAAGCGACAACGAGTCGATGATAATTATAACTCTTACGGTGGAAGAAA
TGAAAATACAGCAGCTTATGCTTCCGGGAAACTTCCATCTGGAAGTATAAATGTTGGTGGAGGTAGGAAGACCACTTTTCAAGAAGAACCTCGA
ACGGCATTTGTCGCTGGCAAGTTGCCCCCTGGAAGTATCAACATTGGTGGAAAGAAGGCTACCCAAATCGAGGACGAGGGTAGAGCAGCTTATG
CTTCCGGAAAATTGCCCCCAGGAAGTATAAACTCTGGCGCAAAGAAGGCGATTTCATTCCAAGATGAAACGAGACCGGCTTATGTCTCCGGAAA
GCTTCCACATGGTAGTATCGACGGTATGCGAAACCGTGAAATGGCTGCCGTCCACCGCGAAATTGCTGAGGGTGGAGGAAACCAGGCCAGGTT
GTCTCTTCTCTATTCACATTCAATCCTACTTCAAAGAAAACTTTCGATGAACCAGAAGAACAAGCGGAACCGGCAAAACCATCCAATGCGCCTT
TGACAGAGGAAATGGCGACTTTCACCAATCTAGGGCTATCGAGAAGGCTTGCAGCCCATCTATCGACTAAACTCGATATGAAAGCCCCGACGGC
CATTCAAAAAGCATCTGTGCAGCAGTTGGTATCGGACGATAGCGATGCTTTCATACAAGCAGAGACTGGATCTGGAAAAACTTTGGCATATCTA
CTACCTATAGTCGAGCGAATATTAGCATTGGGTGAGAATGGCGTACAAA

>Bcin02g07770(MLST9), partial sequence [organism=Botrytis cinerea, strain D13_B_KF1_25]
CAGCTTTCCCTTTCGGTCTTGGCATCTACAGTCATTGCCATCCCTACACCCTCACAACTTGAGTCTCGGGCCGTTATCGATTCCGATGCCGTTG
TAGGATTTGCCGAAACTGTTCCCAGTGGGACCGTAGGAACAGTTTATGAGGCATATAAACCATTCCTTAAAGTCGTAAATGGATGCGTACCATT
CCCTGCCGTCGATGCATCGGGTAACACAGGGTATGTCCTTATACCTTTCTCTTCCACACGATTGCTATTGAGTCTCTAATATATTTTAGTGGTG
GTTTGTCACCAACTGGCAGCAGCAATGGTGAATGCAGCAGCAGTACCGGTCAAGTATATGTTCGAGGAGGACAAAGCGGATCAAACTACGCTAT
CATGTACTCGTGGTAAGTTCTCTTTAAACTTCTCCTTATAGATCCAACCTAACAAAATCTTAGGTACATGCCAAAGGACGAGCCCTCAACCGGT
ATTGGTCACCGTCACGATTGGGAAGGTGTAATTGTCTGGCTCTCCAGCGCCACCGCCACAACTGCCGACAACATCTTAGCCGTTTGTCCTTCCG
CCCACGGAGGCTGGGATTGTTCCACGGATGGCTATTCCCTTTCTGGTACCAGCCCTCTTATCAAGTACGAAAGTATCTGGCCCGTCGATCACTC
AATGGGTCTTACTAGTACTGTTGGTGGAAAACAACCTATGATTGCTTGGGAGTCTTTACCAACTGCTGCTCAAACTGCTCTTGAGAACACCGAT
TTCGGTGCTGCGAATGTTCCATTCATTCCGGCTGTTTTCACAGATAATCT

>Bcin01g07220(MLST1), partial sequence [organism=Botrytis cinerea, strain D13_MR_S11]
ATGGCATATCTTGTTTCTTTTCGCATTATGCTTTGGAACGTGCTGGACAAGCTTGGGTTAGTGCTCTGCGTGTAGAAGCACTAAAGAAGATTCT
CGCACAACCGAAGCCATGGTTTGAGGAATCCAGGAATTCACCTAGCCGGTTGAACGAAGTTTTGGATAGGAACTCTGAGGAAATGCGTAATCTC
GTTGGCCGCTTTGCTGGTATTGTATTCACAGCATTTTTTATGCTATTGATATCAATCATTTGGGCTTTCGTGAATACATGGAAACTGACATTA
GTCTCAATGGCAACTGGGCCAGTTATATACGCTGTCACCAAAACGTTCAATCGCGTGAGTGGAAAATGGGAAAACAAGTGCAACTACGCATCTG
AAATGACCACTGGCATATTTTCAGAGACTTTCTCCAACATCAAAGTGGTTCGGGCTTTTACTCTGGAAACTTACTTTAAGACAAAACACACCAA
AGCTACAGAAGAACTCTATAAAGTTGGACTAATACGAGCAAACTACTCGGGATTGCTGTGGGGATTGACAGATGCAATGTCATTCTTCATCACT
GCAACTATCTTTTATTATGCCACGGTTCTCATTACCAAGAGAGAGATCAGTGTCGCGGCTGCACTACAGACTGTCAATCTTCTATTATTTGGTA
TTTCTAATAGTACGAATATGCTGGCCATGATACCACAAATCAACTCTTCTCGCGTTACAGCTACGCATATGCTTGCATTAGCCAATCTCGATTC
ATCTTCCTCCCACGAAAATAAAGGAACCGAACGGCTTTCGACAATCTTTCCAATCAAATTCAACCGTCTTTCATTCACATACCCTACTCGTCCT
GAAAAACGAACGATATCATCCTTTTCTCTTTCCCTGATTCCTAACTCAACAACTGCACTTGTCGGACCCTCCGGCTCCGGAAAATCTACAATAG
CTGCTCTGCTCATTGGTCTCTATCCGCCAGATACTTCAACACCTCCACCGTTGACATTCAATCGCGTCTCCATAAGTAACTGTCACATTCCGTC
TCTCCGGGCTTCTCTCTCACTCGTTCCACAAATACCGATTCTATTTCCAGCTACCATTCTCCATAACATCATTTATGGTCTCCCAGAATCTTCT
CCTTGTGCTAGTCTTCCATCTGCTATTGCGTCAGCAAAAAATGCTGGGATCCATGAATTTATCACATCGCTTCCACAAGGTTATGATACTAT

>Bcin05g07690(MLST2), partial sequence [organism=Botrytis cinerea, strain D13_MR_S11]
ACCACTATCACCAAGTCCTTTTCTGCACTTTCCTCATCAAAAACGAACGAGAACATCATCAATAAATACTTTGCTGAAGTAATTGGGTTTTATT
TTGGCCAGGATGCCTTCTCTCTACGACAGCAAGCCTTCGATGATATGTTGTGGGTAGTTCTTGGCTGGCTGGATACTGTCAAATTCATTGATTT
ACATTCTGAATTGCACTATTCAAACGACTCTCAGCCAGAATGGTACGGACAACAATATAAACCTGCATTTGCACATCGAGCGCGACTATTTTGG
GAATTGGCTTCACAAGGATGGGATACTACTCTCTGTGGTGGTGGGATGATATGGTCACCATACCTTACTCCATACAAGAACGCAATTACCAATG
AACTCTATATCGCAGCTTCGATATCGATGTACCTATATTTCCCCGGAGATGACAATCAATCCCCATTTATGCTTTCCAACCCTTCATATCCACC
TCACGATCCGAAATATCTACAGGCAGCTGTTGATGCTTACAAATGGCTGAATGGTTCCAACATGACGGATTTACAAGGATTATATGTCGACGGG
TACCATATCTCGAATCTTTCTGGCGGTGAAAACACCCATTGCGATTCTAGAAATGAGATGGTATATACCTACAATCAAGGTGTTTTGCTTACTG
GACAACGTGGTTTGTATGACGCAACCGCCGCACGATCATACCTTGTAGATGGCCACAAACTCATCGCGAATGTTATTAATGCCACAGGCTATGA
CCTGAAACACAATGTTGTCATCTCACCGCCACCCAAAGATGGTTCCGCATTGGCAAAGTGGTTTGGCCTGGGTAGGAATGGAATACTGGAAGAA
GGATGCGATTCAAGTGCTTCGTGTTCTCAAAATGGACAAACTTTCAAAGGCATATTCTTTCATCACTTGATTGCGTTCTGTAGTGATTTGCCAG
GGGAGCCTATTGCAGGGACGAAGGAAAGCTTAGAACTCGACAGAGTGTGGCATTCTGACAAATGCTCACAGTATACAAAAT

>Bcin06g01710(MLST3), partial sequence [organism=Botrytis cinerea, strain D13_MR_S11]
GTGAGTCTGACTTCTGTGTTTGAGCGTTAAGATAGACACTGATGTACCAAGGCAATCACCTAATTTCATGGCGTTCTTCCCTGCATCATCTACC
TACCCTGGAATGCTGGGAGAATTATACTCAGCAGCTTTCACAGCACCTGCTTTCAATTGGACCTGTTCCCCTGCCGTGACAGAATTGGAGACGG
TTGTAATGGATTGGCTGGCCAAGCTTCTCAATCTCCCAGACTGTTATTTGTCTTCGACTCATGGTGGTGGTGTTATCCAAGGATCAGCCTCGGA
AGCTATCGTTACCGTTATGGTTGCTGCCCGCGATAAATATCTTCGTGAAACTACTGAAGGTCTGTCGGGCATTGAACTCGAGGATGCGATTGCA
TATAAGAGGAGTAAGCTAGTTGCACTAGGAAGCGAAATGGCACACAGCTCCACGCAGAAAGCAGCGCAGATAGCTGGCGTTAGATTCCGATCGA
TTCCAGTACTCGCATCCAATGATTTCGCCATGACGGGTGATGATTTAGAGAAGGTATTGGAAGAATGCAAATCTCAAGGATTGGAACCCTTCTA
TCTAACTTCGACTTTGGGAACAACATCTACATGCGCAGTTGACGACTTCGCATCTATTGCAACAGTACTTTCAAAATATGCACCTCCAGATGTT

GCAGGCGAGATCTGGGTTCACGTCGATGCTGCTTATGCAGGTGCAGCTTTGGTTTGCCCTGAATACCATCATCTAACATCGTCCTTCCAGCATT
TCCATTCCTTCGATATGAACATGCACAAATGGCTTCTGACAAATTTCGACGCTTCTTGTCTATATGTCAAGAGACGCAAAGATCTGATAGATGC
ACTCTCCATAACACCAAGTTATCTTCGCAACGAGTTTTCAGAGAGTGGACTCGTAACCGACTATCGGGATTGGCAAATTCCTCTCGGAAGACGC
TTCCGAAGCTTAAAGATTTGGTTTGTCCTCAGAACCTACG

>Bcin11g01310(MLST5), partial sequence [organism=Botrytis cinerea, strain D13_MR_S11]
ACTGACATGGACCTCATGTGGAACCGGCGTAGAATGCGCAACGCTTGAAGTTCCGCTCGAATATGGCGATGCAACGTCAACGGCAAAAGCCAGT
GTTGCGCTTGCTCGTTATCCTGCCACTGTTGCCGCGAGCAAGAAGCTCGGGTCTCTCTTGATAAACCCCGGTGGACCCGGTGCCTCTGGTGTTG
GCTTTGTGCAGTCTGGAGCCGGTGCCGCCGTCTCGACACTGAGTGGTGGATTGTACGATATCATCGGATGGGATCCACGTGGAACCGGTGCTTC
GGCTCCTATTTTGGAATGTTTTGCAAATGCCAGTGCGGAGTATGATTTTAACAACGCGTTTCCATCTGCTCCGAATCTCTGGCTCGGACAATTT
GCGAATGCCAGCGCAAATTCTGCTGTTAGCTCTGCTATCACATCCTTTGACACTTCTGTCGCTGCTCTTGCAAAAGCTTGCGTGGCTCAGAAAT
CTCCCGCTCTTTACACCTCAACAGCAGCATATGTTGCTCGAGACATGGCAGCGATAGTCGATGCATTGGATGGGACCTCTGCAAAACTTAACTA
CTGGGGTTTCTCATATGGAACTATTTTCCTAGCTGAGTTTATCCAAACTTTCCCAGGCCGTGTGGGAAGAGTTCTTGCCGATGGTGTTTTCGAC
GCAAAGGCAAATGCACTCACATACGTTAGCCAACTTCCCAACGATCAACTCAGTGTTCGTGCTTCGTTGAACGATTTTGCAGCTTTCTGCACCA
CCGCCGGTAGTAAAGGTTGCTCTTTTGCCACCGCCCCTACTGGAACCTCAGGTACTGTTGCTACCAGACTGGACAACATAATGAAGGATATGTT
CCTCAATCCTATTGTTGCTTCGGGCTTAAGCATCA

>Bcin15g03910(MLST6), partial sequence [organism=Botrytis cinerea, strain D13_MR_S11]
GCCAAAACACAAAATCATCCAACGATGAAGATGATACTCCACTTCCCTTGATTATCTGGCATGGACTCGGCGATAATTACAAAGCGGATGGTCT
TGCGCAAGTTGGAAAACTAGCTGAAGCTATTCATCCTGGGACTTTTGTCTACAATATTCATGTAGATGAGGATGCATCTGCAGATAGGACAGCT
ACCTTCTTTGGAAATCTCACTCGTGAGTACATCCCCTATTTTTCCTTTAAATATCATACTAACTCTCTTACCAAGTTCAAATCGAAAAGGTCTG
CGAAGACCTCGCCTCCCATCCTATTCTCTCTACCGCGCCCGCCGTCGACGCAATTGGATTCTCCCAAGGCGGCCAATTCTTGCGTGGCTACATA
TCCCGCTGCAATGCTCCACCCATCCGCTCTCTCCTGACCTTCGGTTCCCAACACAACGGCATTTCTGCCTTCCAAGCCTGTGGTCCTGCCGACT
TCCTCTGTCGCGGTGCTCAAACCCTTTTGCGATCCAACACCTGGTCAACCTTTGTCCAATCTCGTCTCGTACCCGCTCAATACTTCAGAGATCC
GGAAAACCTAGACTCTTACCTTGAATATTCCAATTTCCTTGCCGACATCAATAATGAGCGCGTTCTCAAGAACCAAACATATAAATCCAACATG
GAAAAATTGGAACGATTCGTAATGTATGTCTTTGAAGACGATACAACTGTCATCCCTAAGGAAAGTGGATGGTGGGCTGAAGTCAACGGCACGG
AAGTTACACCACTGAAAGAAAGAGCCATTTATAAAGAAGATTGGCTAGGTTTAAAGACATTGGATGAGGCCGGAAAATTAGTTTTCGAAACCAT
TCCAGGGGGACATATGACGTTAGGAGAGGAGATGCTAGAGAAGGCTTTCAAAGAGTATTTTGGTCCAGCAGGGAAGAAATTTGGGG

>Bcin16g03460(MLST7), partial sequence [organism=Botrytis cinerea, strain D13_MR_S11]
ATGAACCTCTTGATTTGAACTCTTCATTAATTGCAAGTGATGAACCCCTATTCTGCCCAGAGGATAGTTACAAGACGTATATCATTAGTCGAGA
ACCACTCATGATATATATTGACGGATTTTTGAAAGCGAATGAAAGTAAACATTTGGTTGATGTTAGGTGTGTTATTCATTCTGATGAAATGAAC
AAGAGAGACTGATGAGATAGTGAACCGCTTTACGAACCATCTACTGTTTCTCACGGACAGGAAGTTACCATTGATCCTTCAGTTCGGAATTCTG
AAGTGGCGGTTTTAGAGAGGGATGAGGTGGTCAGGTGTATTGAGCATAGAGCGAGGGCATTTCAGGGGTGGAGGGGCGAGATGGGGATTGAGAA
GTTGAGGACGCAGAGGTATGGGGTTGGAGGACATTATGGGATGCATTTGTAAGTTTTGGGGGATTGACGAAGGCTTTTGTCTATTTTCTACCAG
TACGGATCTTGAAAGAAAGATAGCATGAACACGAGGGCTAATAACTAGGAAATGGCGATTGGAGCGGAGGTAAACGTGGCATAGACCGATTTAG
TACTTTCATGGTCTATGTCGACGTATCCTCTGATATCGAAGGTGGAGGAACGGAATTCCCACGTATCGTGGGACCAAAAGGAGGAAGGTGGGAG
GACTTCCTGGAAACTACGGAAGCATTGGATCCAAGAACTGGAGAAAATGTAACAGTAGAAGGGGTGACATTCAAACCAATCAAGGGAAATGCCG
TATTCTGGGAAAATACTGACAACAACGGGAGGGGCTATGAT

>Bcin12g03020(MLST8), partial sequence [organism=Botrytis cinerea, strain D13_MR_S11]
CGATTGGCTGCGAAGAAAACTGCGCAGCACCGAGTCACCAAAAGCACGAATAAACCATCGGCAAGGGAAATATTCTCTGAACCACAACATGACA
CGAGCGCGGAAGAGTATATCGGGCGAGAAGCTTCATCAAGAGCACCAAAGCGACAACGAGTCGATGATAATTATAACTCTTACGGTGGAAGAAA
TGAAAATACAGCAGCTTATGCTTCCGGGAAACTTCCATCTGGAAGTATAAATGTTGGTGGAGGTAGGAAGACCACTTTTCAAGAAGAACCACGA
ACGGCATTTGTCGCTGGCAAGTTGCCCCCTGGAAGTATCAACATTGGTGGAAAGAAGGCTACCCAAATCGAGGACGAGGGTAGAGCAGCTTATG
CTTCCGGAAAATTGCCTCCAGGAAGTATAAACTCTGGCGCAAAGAAGGCGACTTCATCCCAAGATGAAACGAGACCGGCTTATGTCTCCGGAAA
GCTTCCACATGGTAGTATCGACGGTATGCGAAACCGTGAAATGGCTGCCGTCCACCGCGAAATTGCTGAGGGTGGAGGAAACCAGGCCAGGTT
GTCTCTTCTCTATTCACATTCAATCCTACTTCAAAGAAAACTTTTGATGAACCCGAAGAACAAGCGGAACCGGCAAAACCATCCAATGCGCCTT
TGACAGAGGAAATGGCGACTTTCACCAATCTAGGGTTATCGAGAAGGCTTGCAGCCCATCTATCGACTAAACTCGATATGAAAGCTCCGACCGC
CATTCAAAAAGCATCTGCACAGCAGTTGGTATCGGACGATAGCGATGCTTTCATACAAGCAGAGACTGGATCTGGAAAAACTTTGGCATATCTA
CTACCTATAGTCGAGCGAATATTAGCATTGAGTGAGAATGGCGTACAAA

>Bcin02g07770(MLST9), partial sequence [organism=Botrytis cinerea, strain D13_MR_S11]
CAGCTTTCCCTTTCGGTCTTGGCATCTACAGTCATTGCCATCCCTACACCATCACAACTTGAGTCTCGGGCCGTTATCGATTCCGATGCCGTTG
TAGGATTTGCCGAAACTGTTCCCAGTGGGACCGTAGGAACAGTTTATGAGGCATATAAACCATTCCTTAAAGTCGTAAATGGATGCGTACCATT
CCCTGCCGTCGATGCATCGGGTAACACAGGGTATGTCCTTATATCTTTCTCTTCCACACGATTGCTATTGAGTCTCTAACATATTTAGTGGTG
GTTTGTCACCAACTGGCAGTAGCAATGGTGGTTGCAGCAGCAGTACCGGTCAAGTATATGTTCGAGGGGACAAAGCGGATCAAACTACGCCATC

ATGTACTCCTGGTAAGTTCTCTCTAAACTTCTCCTTATAGATCCAACCTAACAAAATCTTAGGTACATGCCAAAGGACGAGCCCTCAACCGGTA
TTGGTCACCGTCACGATTGGGAAGGTGTAATTGTCTGGCTCTCCAGCGCCACCGCCACAACTGCCGACAACATCTTAGCCGTTTGTCCTTCCGC
CCACGGAGGCTGGGATTGTTCCACGGATGGCTATTCCCTTTCTGGTACCAGCCCTCTTATCAAGTACGAAAGTATCTGGCCCGTCGATCACTCA
ATGGGTCTTACTAGTACTGTTGGTGGAAAACAACCTATGATTGCTTGGGAGTCTTTACCAACTGCTGCTCAAACTGCTCTTGAGAACACCGATT
TCGGTGCTGCGAATGTTCCATTCATTCCGGCTGTTTTCACAGACAATCT

>Bcin01g07220(MLST1), partial sequence [organism=Botrytis cinerea, strain D13_E_IL7]
ATGGCATATCCTGTTTCTTTTCGCATTATGCTTTGGAACGTGCTGGACAAGCTTGGTTAGTGCTCTGCGTGTAGAAGCACTAAAGAAGATTCTC
GCACACCGAAGTCATGGTTGAGGAATCCAGGAATTCACCTGGCCGGTTGAACGAAGTTTGGATAGGAACTCTGAGGAAATGCGTAATCTCGTTG
GCCGCTTTGCTGGTATTGTATTCACAGCATTTTTATGCTATTGATATCATCATTTGGGCTTTCATGAATACATGGAAACTGACATTAGTCTCGA
TGGCAACTGGCCAGTTATATACGCTGTCACCAAAACGTTCAATCGCGTGAGTGGAAAATGGGAAAACAAGTGCAACTACGCCTCTGAAATGACC
ACTGGCATATTTTCAGAGACTTTCTCCAACATCAAAGTGGTTCGGCTTTTACTCTGGAAACTTACTTTGAGACAAAACACACCAAAGCTACAGA
AGAACTTTATAAAGTTGGACTAATACGAGCAAACTACTCGGGATTGCTGTGGGGATTGACAGATGCGATGTCATTCTTCATCACTGCAACTATC
TTTATTATGCCACGGTTCTCATTACCAAGAGAGAGATCAGTATCGCGACTGCACTACAGACTGTCAATCTTCTATTATTGTATTTCTAATAGTA
CGAATATGCTGGCCATGATACCACAAATCAACTCTTCTCGCGTTACAGCTACGCATATGCTTGCATTAGCCAATCTCGATTCATCTTCCTCCAC
GAAAATAAAGGAACGAACGGCTTTCGACATCTTTCCAATCAAATCAACCGTCTCTCATTCACATACCCTACTCGTCCTGAAAAACGAACGATAT
CATCCTTTTCTCTTTCCCTGATTCCTAACTCAACAACTGCACCTGTCGGACCCTCCGGCTCCGGAAAATCTACAATAGCTGCTCTGCTCATTGG
TCTCTATCCGCCAGATACTTCAACACCTCCACCGTTGACATTCAATCGCGTCTCCATAAGTAACTGTCACATTCCGTCTCTCCGGGCTTCTCTC
TCACTCGTTCCACAAATACCGATTCTATTTCCAGCTACCATTCTCCATAACATCATTTATGGTCTCCCAGAATCTTCTCCTTGTGCTAGTCTTC
CATCAGCTATTGCATCAGCAAAAGATGCTGGGATCCATGAATTTATCACATCGCTTCCACAAGGCTATGATACTAT

>Bcin05g07690(MLST2), partial sequence [organism=Botrytis cinerea, strain D13_E_IL7]
CACCACTATCACCAAGTCCTTTTCTGCACTTTCCTCATCAAAAACGAACGAGAACATCATCAATAAATACTTTGCTGAAGTAATTGGGTTTTAT
TTTGGCCAGGATGCCTTCTCTCTACGACAGCAAGCCTTCGATGATACGTTGTGGGTAGTTCTTGGCTGGCTGGATACTGTCAAATTCATTGATT
TACATTCTGAATTGCACTATTCAAACGACTCTCAGCCAGAATGGTACGGACAACAATATAAACCTGCATTTGCACATCGAGCGCGACTATTTTG
GGAATTGGCTTCACAAGGATGGGATACTACTCTCTGTGGTGGTGGGATGATATGGTCACCATACCTTACTCCATACAAGAACGCAATTACCAAT
GAACTCTATATCGCAGCTTCGATATCGATGTACCCATATTTCCCCGGAGATGACAATCAATCCCCATTTATGATTTCCAACCCTTCATATCCAC
CTCACGATCCGAAATATCTACAGGCAGCTGTTGATGCTTACAAATGGCTGAATGGTTCCAACATGACGGATTCACAAGGATTATATGTCGACGG
GTACCATATCTCGAATCTTTCTGGCGGTGAAAACACCCATTGCGATTCTAGAAATGAGATGGTATATACCTACAATCAAGGTGTTTTGCTTACT
GGACAACGTGGTTTGTATGACGCAACCGCCGCACGATCATACCTTGTAGATGGCCACAAACTCATCGCGAATGTTATTAATGCCACAGGCTATG
ACCTGAAACACAATGTTGTCATCTCACCGCCACCCAAAGATGGTTCCGCATTGGCAAAGTGGTTTGGCCTGGGTAGGAATGGAATACTGGAAGA
AGGATGCGATTCAAGTGCTTCGTGTTCTCAAATGGACAAACTTTCAAAGGCATATTCTTTCATCACTTGATTGCGTTCTGTAGTGATTTGCCAG
GGGAGCCTATTGCAGGGACGAAGGAAAGCTTAGAACTCGACAGAGTGTGGCATTCTGACAAATGCTCACAGTATACAAAAT

>Bcin06g01710(MLST3), partial sequence [organism=Botrytis cinerea, strain D13_E_IL7]
GTGAGTCTGACTTTTTTATTTGAGCATTAAGATAGACACTGATATACCAAGGCAATCACCTAATTTCATGGCGTTCTTCCCTGCATCATCTACC
TACCCTGGAATGCTGGGAGAATTATACTCAGCAGCTCTCACAGCACCTGCTTTCAATTGGATCTGTTCCCCTGCTGTGACAGAATTGGAGACGG
TTGTAATGGATTGGCTGGCCAAGCTTCTCAATCTCCCAGACTGTTATTTGTCTTCGACTCATGGTGGTGGTGTTATCCAAGGATCAGCCTCGGA
AGCTGTCGTTACCGTTATGGTTGCTGCCCGCGATAAATATCTTCGTGAAACTACTGAAGGTCTGTCGGGTATTGAACTCGAGGATGCGATTGCA
TATAAAAGGAGTAAGCTAGTTGCACTAGGAAGCGAAATGGCACACAGTTCCACGCAGAAAGCAGCGCAGATAGCTGGCGTTAGATTCCGATCGA
TTCCAGTACTCGCATCCAATGATTTCGCCATGACGGGTGATGATTTAGAGAAGGTATTGAAAGAATGCAAATCTCAGGGATTGGAACCCTTCTA
TCTAACTTCGACTTTGGGAACAACATCTACATGCGCAGTTGACGACTTCGCATCTATTACAACAGTACTTTCAAAATATGCACCTCCAGATGTT
GCAGGCGAGATCTGGGTTCACGTCGATGCTGCTTATGCAGGTGCAGCTTTGGTTTGCCCTGAATACCATCATCTAACATCGTCCTTCCAGCATT
TCCATTCCTTCGATATGAACATGCACAAATGGCTTCTGACAAATTTCGACGCTTCTTGTCTATATGTCAAGAAACGCAAAGATCTGATCGATGC
ACTATCCATAACACCAAGTTATCTTCGCAACGAGTTTTCAGAGAGTGGACTCGTAACCGACTATCGTGACTGGCAAATTCCTCTCGGAAGACGC
TTCCGAAGCTTAAAGATTTGGTTTGTCCTCAGAACCTAC

>Bcin11g01310(MLST5), partial sequence [organism=Botrytis cinerea, strain D13_E_IL7]
GGACTGACATGGACCTCATGTGGAACCGGCGTAGAATGCGCAACGCTTGAAGTTCCGCTCGAATATGGCGATGCAACGTCAACGGCAAAAGCCA
GTGTTGCGCTTGCTCGTTATCCTGCCACTGTTGCCGCGAGCAAGAAGCTCGGGTCTCTCTTGATAAACCCCGGTGGACCCGGTGCCTCTGGTGT
TGGCTTTGTGCAGTCTGGAGCCGGTGCCGCCGTCTCGACACTGAGTGGTGGATTGTACGATATCATCGGATGGGATCCACGTGGAACCGGTGCT
TCGGCTCCTATTTTGGAATGTTTTGCAAATGCCAGTGCGGAGTATGATTTTAACAACGCGTTTCCATCTGCTCCGAATCTCTGGCTCGGACAAT
TTGCGAATGCCAGCGCAAATTCTGCTGTTAGCTCTGCTATCACATCCTTTGACACTTCTGTCGCTGCTCTTGCAAAAGCTTGCGTGGCTCAGAA
ATCTCCCGCTCTTTACACCTCAACAGCAGCATATGTTGCTCGAGACATGGCAGCGATAGTCGATGCATTGGATGGGACCTCTGCAAAACTTAAC
TACTGGGGTTTCTCATATGGAACTATTTTCCTAGCTGAGTTTATCCAAACTTTCCCAGGCCGCGTGGGAAGAGTTCTTGCCGATGGTGTTTTCG
ACGCAAAGGCAAATGCACTCACATACGTTAGCCAACTTCCCAACGATCAACTCAGTGTTCGTGCTTCGTTGAACGATTTTGCAGCTTTCTGCAC
CACCGCCGGTAGTAAAGGTTGCTCTTTTGCCACCGCCCCTACTGGAACCTCAGGTACTGTTGCTACCAGACTGGACAACATAATGAAAGATATG

TTCCTCAATCCTATTGTTGCTTCGGGCTTAAGCATCA

>Bcin15g03910(MLST6), partial sequence [organism=Botrytis cinerea, strain D13_E_IL7]
GCCAAAACACGAAATCATCCAACGATGAAGATGATACTCCACTTCCCTTGATTATCTGGCATGGACTCGGCGATAATTACAAAGCGGATGGTCT
TGCGCAAGTTGGAAAACTAGCTGAAGCTATTCATCCTGGACTTTTGTCTACAATATTCATGTAGATGAGGATGCATCTGCAGATAGGACAGCTA
CCTTCTTTGGAAATCTCACTCGTGAGTACATTCCCTATTTTTCCTTTAAATATCATACTAACTCTCTTACCAAGTTCAAATCGAAAAGGTCTGC
GAAGACCTCGCCTCCCATCCTATTCTCTCTACCGCGCCCGCCGTCGACGCAATTGGATTCTCCCAAGGCGGCCAATTCTTGCGTGGTTACATAT
CCCGCTGCAATGCTCCACCCATCCGCTCTCTCCTGACCTTCGGTTCCCAACACAACGGCATTTCTGCCTTCCAAGCCTGTGGTCCTGCCGATTT
CCTCTGTCGCGGTGCTCAAACCCTTTTGCGATCCAACACCTGGTCAACCTTTGTCCAATCTCGTCTCGTACCCGCTCAATACTTCAGAGATCCG
GAAAACCTAGACTCTTACCTTGAATATTCCAATTTCCTTGCCGACATCAATAATGAGCGCGTTCTCAAGAACCAAACATATAAATCCAACATGG
AAAAATTGGAACGATTCGTAATGTATGTCTTTGAAGACGACACAACTGTCATCCCTAAGGAAAGTGGATGGTGGGCTGAAGTCAACGGCACGGA
AGTTACACCACTGAAAGAAAGAGCCATTTATAAAGAAGATTGGCTAGGTTTAAAGACATTGGATGAGGCCGGAAAATTAGTTTTCGAAACCATT
CCAGGGGACATATGACGTTAGGAGAGGAGATGCTAGAGAAGGCTTTCAAAGAGTATTTTGGTCCAGCAGGGAAGAAATTTGGG

>Bcin16g03460(MLST7), partial sequence [organism=Botrytis cinerea, strain D13_E_IL7]
ATGAACCTCTTAATTTGAACTCTTCATTATTTGCAAGTGATGAACCCCTATTCTGCCCAGAGGATAGTTACAAGACGTATATCATTAGTCGAGA
ACCACTCATGATATATATGACGGATTTTTGAAAGCGAATGAAAGTAGACACTTGGTTGATGTTAGGTGTGTTATTTATTCTGATGAAATGAACA
AGAGAGACTGATGAGATAGTGAACCGCTTTATGAACCGTCTACTGTTTCTCACGGGCAGGAAGTTACCATTGATACTTCAGTTCGAAATTCTGA
AGTGGCGGTTTTAGAGAGGGATGAGGTGGTCAGGTGTATTGAGCATAGAGCGAGGGCATTTCAGGGGTGGAGGGCGAGATGGGGATTGAGAAGT
TGAGGACGCAGAGGTATGGGGTTGGAGGACATTATGGGATGCATTTGTAAGTTTTGGGGATTGACGAAGGCTTTTGTCTATTTTCTACCAGTAC
GGATCTTGAAAGAAAGATAGCATGAACACGAGGGCTAATAACTAGGAAATAGCGATTGGAGTGGAGGTAAACGTGGCATAGACCGATTTAGTAC
TTTCATGGTCTATGTCGACGTATCCTCTGATATCGAAGGTGGAGGAACGGAATTCCCACGTATCGTGGGACCAAAAGGAGGAAGGTGGGAGGAC
TTCCTGGAAACTACGGAAGCATTGGATCCAAGAACTGGAGAAAATGTAACAGTAGAAGGGGTGACATTCAAACCAATCAAGGGAAATGCCGTAT
TCTGGGAAAATACTGACAACAACGGGAGGGGCTATGAT

>Bcin12g03020(MLST8), partial sequence [organism=Botrytis cinerea, strain D13_E_IL7]
TCGATTGCTGCGAAGAAAACTGCGCAGCACCGAGTCACCAAAAGCACGAATAAACCATCGGCAAGGGAAATATTCTCTGAACCACAACATGACA
CGAGCGCGGAAGAGTATATCGGGCGAGAAGCTTCATCAAGAGCACCAAAGCGACAACGAGTCGATGATAATTATAACTCTTACGGTGGAAGAAA
TGAAAATACAGCAGCTTATGCTTCCGGGAAACTTCCATCTGGAAGTATAAATGTTGGTGGAGGCAGGAAGACCACTTTTCAAGAAGAACCACGA
ACGGCATTTGTCGCTGGCAAGTTGCCCCCTGGAAGTATCAACATTGGTGGAAAGAAGGCTACCCAAATCGAGGACGAGGGTAGAGCAGCTTATG
CTTCCGGAAAATTGCCTCCAGGAAGTATAAACTCTGGCGCAAAGAAGGCGATTTCATTCCAAGATGAAACGAGACCGGCTTATGTCTCCGGAAA
GCTTCCACATGGTAGTATCGACGGTATGCGAAACCGTGAAATGGCTGCCGTCCACCGCGAAATTGCTGAGGGTGGAGGAAACCAGGCCAGGTT
GTCTCTTCTCTATTCACATTCAATCCTACTTCAAAGAAAACTTTTGATGAACCCGAAGAACAAGCGGAACCGCAAAACCATCCAATGCGCCTTT
GACAGAGGAAATGGCGACTTTCACCAATCTAGGGTTATCGAGAAGGCTTGCAGCCCATCTATCGACTAAACTCGATATGAAAGCTCCGACCGCC
ATTCAAAAAGCATCTGTACAGCAGTTGGTATCGGACGATAGCGATGCTTTCATACAAGCAGAGACTGGATCTGGAAAAACTTTGGCATATCTAC
TACCTATAGTCGAGCGAATATTAGCATTGAGTGAGAATGGCGTACAAA

>Bcin02g07770(MLST9), partial sequence [organism=Botrytis cinerea, strain D13_E_IL7]
CAGCTTTCCCTTTCGGTCTTGGCATCTACAGTCATTGCCATCCCTACACCATCACAACTTGAGTCTCGGGCCGTTATCGATTCCGATGCCGTTG
TAGGATTTGCCGAAACTGTTCCCAGTGGGACCGTAGGAACAGTTTATGAGGCATATAAACCATTCCTTAAAGTCGTAAATGGATGCGTACCATT
CCCTGCCGTCGATGCATCGGGTAACACAGGGTATGTCCTTATACCTTTCTCTTCCACACGATTGCTATTGAGTCTCTAACATATTTTAGTGGTG
GTTTGTCACCAACTGGCAGCAGCAATGGTGAATGCAGCAGCAGTACCGGTCAAGTATATGTTCGAGGAGGACAAAGCGGATCAAACTACGCTAT
CATGTACTCCTGGTAAGTTCTCTCTAAACTTCTCCTTATAGATCCAACCTAACAAAATCTTAGGTACATGCCAAAGGACGAGCCCTCAACCGGT
ATTGGTCACCGTCACGATTGGGAAGGTGTAATTGTCTGGCTCTCCAGCGCCACCGCCACAACTGCCGACAACATCTTAGCCGTTTGTCCTTCCG
CCCACGGAGGCTGGGATTGTTCCACGGATGGCTATTCCCTTTCTGGTACCAGCCCTCTTATCAAGTACGAAAGTATCTGGCCCGTCGATCACTC
AATGGGTCTTACTAGTACTGTTGGTGGAAAACAACCTATGATTGCTTGGGAGTCTTTACCAACTGCTGCTCAAACTGCTCTTGAGAACACCGAT
TTCGGTGCTGCGAATGTTCCATTCATTCCGGCTGTTTTCACAGACAATCT

>Bcin04g02090(MLST10), partial sequence [organism=Botrytis cinerea, strain D13_E_IL7]
CGGAGGATGATATGGCAAAGTCTATGATTACCAAAGCATTTGTAGGCATGAGTAGTAAACTGTCGAATATGACTATGAATGATGTTTACAAGCC
CTACATCCATGTAAGAAATGTAGAATAGAGAGATCAGTAACTGGAACTAATATCGTTTGTAGGCTTTCAAGTTACTTACGCAGTTCAACCCAAT
CACTACAGCTATTGCCGAATCCCCACTATTTCAAATGGCTGTCTCAGCAAATACCATCGAAAAGTACACACTGCTAGGCCCCTTTCTTCAGAATA
TCTCCTCTGCAACAGGAAGTTACCAGGGAATACTTCAGTGCGCCAAAGACGATAGATAGGCGACACATTGCCACATCTCAAGATGCCTTACGAT
TGACCTTACAAACCCATCAAAAAGATTTACTTGATATCATCAACCACTTTGTTCGAGCAAGTCCAATCGCAAAAAGCAAAACCCTGGATTGGTT
CGCCTACATTGTGAATCAAAATCACAAGCGTCGAGCACTTCAGGTAGACCCGAAAGAAGTGTCTTCTGATGGCTTTATGCACAATGTCACTGTC
GTTCTAGATGGTCTTTGTGAGCCATTCATGGATACCACATTCTCGAAAATTTCGAAGATTGATATTGATTATCTAAGACGTGCGCCTCGTGTAG
ATATCAAGGACGAGACCAAGTTGAACGCTGACGAGAAGGCTTCTGAGAAGTATTATGAGGACACTGTTCCTGGCACTTCTAATTTCATCTCTGA

GGTCTTCTTTCTCACATTGGCTGCTCATCATTATGGTAGTGAAGCTCTTAATGCCACGCATAAGAGTCTGGAGAAAGACATCAAATATATTCAA
AAGCAATTGACTGCCGTTGAAGCA

>Bcin01g07220(MLST1), partial sequence [organism=Botrytis cinerea, strain D13_MR_S2]
ATGGCATATCTTGTTTCTTTTCGCATTATGCTTTGGAACGTGCTGGACAAGCTTGGGTTAGTGCTCTGCGTGTAGAAGCACTAAAGAAGATTCT
CGCACAACCGAAGTCATGGTTTGAGGAATCCAGGAATTCACCTAGCCGGTTGAACGAAGTTTTGGATAGGAACTCTGAGGAAATGCGTAATCTC
GTTGGCCGCTTTGCTGGTATTGTATTCACAGCATTTTTTATGCTATTGATATCAATCATTTGGGCTTTTGTGAATACATGGAAACTGACATTAG
TCTCGATGGCAACTGGGCCAGTTATATACGCTGTCACCAAAACATTCAATCGCGTGAGTGGAAAATGGGAAAACAAGTGCAACTACGCCTCTGA
AATGACCACTGGCATATTTTCAGAGACTTTCTCCAACATCAAAGTGGTTCGGGCTTTTACTCTGGAAACTTACTTTGAGACAAAACACACCAAA
GCTACAGAAGAACTTTATAAAGTTGGACTAATACGAGCAAACTACTCGGGATTGCTGTGGGGACTGACAGATGCGATGTCATTCTTCATCACTG
CAACTATCTTTTATTATGCCACGGTTCTCATTACCAAGAGAGAGATCAGTATCGCGACTGCACTACAGACTGTCAATCTTCTATTATTTGGTAT
TTCTAATAGTACGAATATGCTGGCCATGATACCACAAATCAACTCTTCTCGCGTTACAGCTACGCATATGCTTGCATTAGCCAATCTCGATTCA
TCTTCCTCCCACGAAAATAAAGGAACCGAACGGCTTTCGACAATCTTTCCAATCAAATTCAACCGTCTTTCATTCACATACCCTACTCGTCCTG
AAAAACGAACGATATCATCCTTTTCTCTTTCCCTGATTCCTAACTCAACAACTGCACTTGTCGGACCCTCCGGCTCCGGAAAATCTACAATAGC
TGCTCTGCTCATTGGTCTCTATCCGCCAGATACTTCAACACCTCCACCGTTGACATTCAATCGCGTCTCCATAAGTAACTGTCACATTCCGTCT
CTCCGGGCTTCTCTCTCACTCGTTCCACAAATACCGATTCTATTTCCAGCTACCATTCTCCATAACATCATTTATGGTCTCCCAGAATCTTCTC
CTTGTGCTAGTCTTCCATCAGCTATTGCATCAGCAAAAGATGCTGGATCCATGAATTTATCACATCGCTTCCACAAGGTTATGATACTAT

>Bcin05g07690(MLST2), partial sequence [organism=Botrytis cinerea, strain D13_MR_S2]
ACCACTATCACCAAGTCCTTTTCTGCACTTTCCTCATCAAAAACGAACGAGAACATCATCAATAAATACTTTGCTGAAGTAATTGGGTTTTATT
TTGGCCAGGATGCCTTCTCTCTACGACAGCAAGCCTTCGATGATATGTTGTGGGTAGTTCTTGGCTGGCTGGATACTGTCAAATTCATTGATTT
ACATTCTGAATTGCACTATTCAAACGACTCTCAGCCAGAATGGTACGGACAACAATATAAACCTGCATTTGCACATCGAGCGCGACTATTTTGG
GAATTGGCTTCACAAGGATGGGATACTACTCTCTGTGGTGGTGGGATGATATGGTCACCATACCTTACTCCATACAAGAACGCAATTACCAATG
AACTCTATATCGCAGCTTCGATATCGATGTACCTATATTTCCCCGGAGATGACAATCAATCCCCATTTATGATTTCCAACCCTTCATATCCACC
TCACGATCCGAAATATCTACAGGCAGCTGTTGATGCTTACAAATGGCTGAATGGTTCCAACATGACGGATTCACAAGGATTATATGTCGACGGG
TACCATATCTCGAATCTTTCTGGCGGTGAAAACACCCATTGCGATTCTAGAAATGAGATGGTATATACCTACAATCAAGGTGTTTTGCTTACTG
GACAACGTGGTTTGTATGACGCAACCGCCGCACGATCATACCTTGTAGATGGCCACAAACTCATCGCGAATGTTATTAATGCCACAGGCTATGA
CCTGAAACACAATGTTGTCATCTCACCGCCACCCAAAGATGGTTCCGCATTGGCAAAGTGGTTTGGCCTGGGTAGGAATGGAATACTGGAAGAA
GGATGCGATTCAAGTGCTTCGTGTTCTCAAAATGGACAAACTTTCAAAGGCATATTCTTTCATCACTTGATTGCGTTCTGTAGTGATTTGCCAG
GGGAGCCTATTGCAGGGACGAAGGAAAGCTTAGAACTCGACAGAGTGTGGCATTCTGACAAATGCTCACAGTATACAAAAT

>Bcin06g01710(MLST3), partial sequence [organism=Botrytis cinerea, strain D13_MR_S2]
GTGAGTCTGACTTTTGTATTTGAGCGTTAAGATAGACACTGATATACCAAGGCAATCACCTAATTTCATGGCGTTCTTCCCTGCATCATCTACC
TACCCTGGAATGCTGGGAGAATTATACTCAGCAGCTTTCACAGCACCTGCTTTCAATTGGATCTGTTCCCCTGCTGTGACAGAATTGGAGACGG
TTGTAATGGATTGGCTGGCCAAGCTTCTCAATCTCCCAGACTGTTATTTGTCTTCGACTCATGGTGGTGGTGTCATCCAAGGATCAGCCTCGGA
AGCTATCGTTACCGTTATGGTTGCTGCCCGCGATAAATATCTTCGTGAAACTACTGAAGGTCTGTCGGGCATTGAGCTCGAGGATGCGATTGCA
TATAAGAGGAGTAAGCTAGTTGCACTAGGAAGCGAAATGGCACACAGCTCCACGCAGAAAGCAGCGCAAATAGCTGGCGTTAGATTCCGATCGA
TTCCAGTACTCGCATCCAATGATTTCGCCATGACGGGTGATGATTTAGAGAAGGTGTTGAAAGAATGCAAATCTCAAGGATTGGAACCCTTCTA
TCTAACTTCGACTTTGGGAACAACATCTACATGCGCAGTTGACGACTTCGCATCTATTGCAACAGTACTTTCAAAATATGCACCTCCAGATGTT
GCAGGCGAGATCTGGGTTCACGTCGATGCTGCTTATGCAGGTGCAGCTTTGGTTTGCCCTGAATACCATCATCTAACATCGTCCTTCCAGCATT
TCCATTCCTTCGATATGAACATGCACAAATGGCTTCTGACAAATTTCGACGCTTCTTGTCTATATGTCAAGAAACGCAAAGATCTGATCGATGC
ACTCTCCATAACACCAAGTTATCTTCGCAACGAGTTTTCAGAGAGTGGACTCGTAACCGACTATCGGGACTGGCAAATTCCTCTCGGAAGACGC
TTCCGAAGCTTAAAGATTTGGTTTGTCCTCAGAACCTACGG

>Bcin09g03030(MLST4), partial sequence [organism=Botrytis cinerea, strain D13_MR_S2]
ACCCTTCAACCCCACCGCAGCTCCTATACCGAAAGCGAGTATCCTACCAATTCTCCTTCCACCTGCGACTTTAAGACCATTGGCTTTCCGCACT
TTCACAAAAAAGCATAGTTTAACATTGACGTCGTCGGCATTACAAGTGTTGGCTACTTTTATTGGAAAGCATTGTGGGACAGGATGGAGGGAAG
AAGGACTGGCAGAGAGAGTCTTGGAGGAGGTCGCCAAGAGTTGGAAGAATAGGAGTGGCGGTGTCATTGTCGAGGGCGAGGGAACGGAATTGAA
GGAGATTCTGAAAGCTTTGGAAGGGAATATGAGTGGTGGAAGGATAGTCATAGGAAGAGAGCTAAGCCGGCAGAATAGTTTAGTACTGGGATCA
TCACAATATGGAGAGGTCAATCATACAAGACTTGGGCTACGGCCAGGGAATATACCCAGAGAGGATAGTCAGTCAAGTTTGGGAATGTCAACGT
TGGAGGTCAATGACGAGGAAGATGAGGATGGCCTGATGGATCCAAGAAGGTGGTTAAAAGTCATTGATGCATTTGAGCAACCTCGACTGGTGTA
CAATGTTGCTAAAAAGCACTTTGATAGGTATGTTTCAATGATAAAATTTCATCTGAATCGACTAACTCAGTACAGAGATACCTCCAAACCTTCA
TTGTTCCCACCTGCGTCTCATAAAACACTCCTCTTCCAAAACCGCTATAATGTTATCCATCAACGTCTCCTTCGCAATGAATCTTTTCAAACGC
CCGCTTTTCAAGGTGGCAAATCTTCCCTTCAACGCAGCACGTCCGCCATTACCACCCAACAACAATCATACAAATTAACGCCGATAGCTAATCT
TCTCGGTCGCAATCGCAGCTCTCATATGCTTCTCGGTCTCCTCAGTATTTCACCCACTGGTACCCTCGCCATCAATGACCTGACGGGCAGTATC
GCTCTCGATCTTACACACGCAGCAGCCATTCC

```
>Bcin11g01310(MLST5), partial sequence [organism=Botrytis cinerea, strain D13_MR_S2]
ACTGACATGGACCTCATGTGGAACCGGCGTAGAATGCGCAACGCTTGAAGTTCCGCTCGAATATGGCGATGCAACGTCAACGGCAAAAGCCAGT
GTTGCGCTTGCTCGTTATCCTGCCACTGTTGCCGCGAGCAAGAAGCTCGGGTCTCTCTTGATAAATCCGGTGGACCCGGTGCCTCTGGTGTTGG
CTTTGTGCAGTCTGGAGCCGGTGCCGCCGTCTCGACACTGAGTGGTGGATTGTACGATATCATCGGATGGGATCCACGTGGAACCGGTGCTTCG
GCTCCTATTTTGGAATGTTTTGCAAATGCCAGTGCGGAGTATGATTTTAACAACGCGTTTCCATCTGCTCCGAATCTCTGGCTCGGATAATTTG
CGAATGCCAGCGCAAATTCTGCTGTTAGCTCTGCTATCACATCCTTTGACACTTCTGTCGCTGCTCTTGCAAAAGCTTGCGTGGCTCAGAAATC
TCCCGCTCTTTACACCTCAACAGCAGCATATGTTGCTCGAGACATGGCAGCGATAGTCGATGCATTGGATGGGACCTCTGCAAAACTTAACTAC
TGGGGTTTCTCATATGGAACTATTTTCCTAGCTGAGTTTATCCAAACTTTCCCAGGCCGCGTGGGAAGAGTTCTTGCCGATGGTGTTTTCGACG
CAAAGGCAAATGCACTCACATACGTTAGCCAACTTCCCAACGATCAACTCAGTGTTCGTGCTTCGTTGAACGATTTTGCAGCTTTCTGCACCAC
CGCCGGTAGTAAAGGTTGCTCTTTTGCCACCGCCCCTACTGGAACCACAGGTACTGTTGCTACCAGACTGGACAACATAATGAAGGATATGTTC
CTCAATCCTATTGTTGCTTCGGGCTTAAGCATCA

>Bcin15g03910(MLST6), partial sequence [organism=Botrytis cinerea, strain D13_MR_S2]
TCGCCAAAACACAAAATCATCCAACGATGAAGATGATACTCCACTTCCCTTGATTATTTGGCATGGACTCGGCGATAATTACAAAGCGGATGGT
CTTGCGCAAGTTGGAAAACTAGCTGAAGCTATTCATCCTGGGACTTTTGTCTACAATATTCATGTAGATGAGGATGCATCTGCAGATAGGACAG
CTACCTTCTTTGGAAATCTCACTCGTGAGTACATCCCCTATTTTCCCTTTAAATACCATACTAACTCTCTTACCAAGTTCAAATCGAAAAGGTC
TGCGAAGACCTCGCCTCCCATCCTATTCTCTCTACCGCGCCCGCCGTCGACGCAATTGGATTCTCCCAAGGCGGCCAATTCTTGCGCGGTTACA
TATCCCGCTGCAATGCTCCACCCATCCGCTCTCTCCTGACCTTCGGTTCCCAACACAACGGCATTTCTGCCTTCCAAGCCTGTGGTTCCTGCCG
ATTTCCTCTGTCGCGGTGCTCAAACCCTTTTGCGATCCAACACCTGGTCAACCTTTGTCCAATCTCGTCTCGTACCCGCTCAATACTTCAGAGA
TCCGGAAAACCTAGACTCTTACCTTGAATATTCCAATTTCCTTGCCGACATCAATAATGAGCGCGTTCTCAAGAACCAAACATATAAATCCAAC
ATGGAAAAATTGGAACGATTCGTAATGTATGTCTTTGAAGACGATACAACTGTCATCCCTAAGGAAAGTGGATGGTGGGCTGAAGTCAACGGCA
CGGAAGTTACACCACTGAAAGAAAGAGCCATTTATAAAAGAAGATTGGCTAGGTTTAAAGACATTGGATGAGGCCGGAAAATTAGTTTTCGAAA
CCATTCCAGGGGGACATATGACGTTAGGAGAGGAGATGCTAGAGAAGGCTTTCAAAGAGTATTTTGGTCCAGCAGGGAAGAAATTTGGG

>Bcin16g03460(MLST7), partial sequence [organism=Botrytis cinerea, strain D13_MR_S2]
ATGAACCTCTTGATTTGAACTCTTCATTAATTGCAAGTGATGAACCCCTATTCTGCCCAGAGGATAGTTACAAGACGTATATCATTAGTCGAGA
ACCACTCATGATATATATTGACGGATTTTTGAAAGCGAATGAAAGTAAACATTTGGTTGATGTTAGGTGTGTTATTTATTCTGATGAAATGAAC
AAGAGAGACTGATGAGATAGTGAGCCGCTTTATGAACCGTCAACTGTTTCTCACGGACAGGAAGTTACCATTGATCCTTCAGTTCGGAATTCTG
AAGTGGCGGTCTTAGAGAGGGATGAGGTGGTCAGGTGTATTGAGCATAGAGCGAGGGCATTTCAGGGGTGGAGGGGCGAGATGGGGATTGAGAA
GTTGAGGACGCAGAGGTATGGGGTTGGAGGACATTATGGGATGCATTTGTAAGTTTTGGGGGATTGACGAAGGCTTCTGTCTATTTTCTATCAG
CACGGATCTTGAAAGAAAGATAGCATGAACACGCGGGCTAATAACTAGGAAATAGCGATTGGAGCGGAGGTAAACGTGGCATAGACCGATTTAG
TACTTTCATGGTCTATGTCGACGTATCCTCTGATATCGAAGGTGGAGGAACGGAATTCCCACGTATTGTGGGACCAAAAGGAGGAAGGTGGGAG
GACTTCCTGGAAACTACGGAAGCATTGGATCCAAGAACTGGAGAAAATGTAACAGTAGAAGGGGTGACATTCAAACCAATCAAGGGAAATGCCG
TATTCTGGGAAAATACTGACAACAACGGGAGGGGCTATGAT

>Bcin12g03020(MLST8), partial sequence [organism=Botrytis cinerea, strain D13_MR_S2]
CGATTGGCTGCGAAGAAAACTGCGCAGCACCGAGTCACCAAAAGCACGAATAAACCATCGGCAAGGGAAATATTCTCTGAACCACAACATGACA
CGAGCGCGGAAGAGTATATCGGGCGAGAAGCTTCATCAAGAGCACCAAAGCGACAACGAGTCGATGATAATTATAACTCTTACGGTGGAAGAAA
TGAAAATACAGCAGCTTATGCTTCCGGGAAACTTCCATCTGGAAGTATAAATGTTGGTGGAGGTAGGAAGACCACTTTTCAAGAAGAACCTCGA
ACGGCATTTGTCGCTGGCAAGTTGCCCCCTGGAAGTATCAACATTGGTGGAAAGAAGGCTACCCAAATCGAGGACGAGGGTAGAGCAGCTTATG
CTTCCGGAAAATTGCCCCCAGGAAGTATAAACTCTGGCGCAAAGAAGGCGATTTCATTCCAAGATGAAACGAGACCGGCTTATGTCTCCGGAAA
GCTTCCACATGGTAGTATCGACGGTATGCGAAACCGTGAAATGGCTGCCGTCCACCGCGAAATTGCTGAGGGTGGGAGGAAACCAGGCCAGGTT
GTCTCTTCTCTATTCACATTCAATCCTACTTCAAAGAAAACTTTCGATGAACCAGAAGAACAAGCGGAACCGGCAAAACCATCCAATGCGCCTT
TGACAGAGGAAATGGCGACTTTCACCAATCTAGGGCTATCGAGAAGGCTTGCAGCCCATCTATCGACTAAACTCGATATGAAAGCCCCGACGGC
CATTCAAAAAGCATCTGTGCAGCAGTTGGTATCGGACGATAGCGATGCTTTCATACAAGCAGAGACTGGATCTGGAAAAACTTTGGCATATCTA
CTACCTATAGTCGAGCGAATATTAGCATTGAGTGAGAATGGCGTACAAA

>Bcin02g07770(MLST9), partial sequence [organism=Botrytis cinerea, strain D13_MR_S2]
CAGCTTTCCCTTTCGGTCTTGGCATCTACAGTCATTGCCATCCCTACACCATCACAACTTGAGTCTCGGGCCGTTATCGATTCCGATGCCGTTG
TAGGATTGCTGAAACTGTTCCCAGTGGGACCGTAGGAACAGTTTATGAGGCATATAAACCATTCCTTAAAGTCGTAAATGGATGCGTACCATTC
CCTGCCGTCGATGCATCGGGTAACACAGGGTATGTCCTTATATCTTTCTCTTCCACACGATTGCTATTGAGTCTCTAACATATTTTAGTGGTGG
TTTGTCACCAACTGGCAGTAGCAATGGTGGTTGCAGCAGCAGTACCGGTCAAGTATATGTTCGAGGAGGACAAAGCGGATCAAACTACGCCATC
ATGTACTCCTGGTAAGTTCTCTCTAAACTTCTCCTTATAGATCCAACCTAACAAAATCTTAGGTACATGCCAAAGGACGAGCCCTCAACCGGTA
TTGGTCACCGTCACGATTGGGAAGGTGTAATTGTCTGGCTCTCCAGCGCCACCGCCACAACTGCCGACAACATCTTAGCCGTTTGTCCTTCCGC
CCACGGAGGCTGGGATTGTTCCACGGATGGCTATTCCCTTTCTGGTACCAGCCCTCTTATCAAGTACGAAAGTATCTGGCCCGTCGATCACTCA
ATGGGTCTTACTAGTACTGTTGGTGGAAAACAACCTATGATTGCTTGGGAGTCTTTACCAACTGCTGCTCAAACTGCTCTTGAGAACACCGATT
TCGGTGCTGCGAATGTTCCATTCATTCCGGCTGTTTTCACAGACAATCTT
```

>Bcin04g02090(MLST10), partial sequence [organism=Botrytis cinerea, strain D13_MR_S2]
CGGAGGATGATATGGCAAAGTCTATGATTACCAAAGCATTTGTAGGCATGAGTAGTAAACTGTCGAATATGACTATGAATGATGTTTACAAGTC
CTACATCCATGTAAGAAATGTAGAATAGAGAGATCAGTAACTGGAACTAATATCGTTTGTAGGCTTTCAAGTTACTTACGCAGTTCAACCCAAT
CACTACAGCTATTGCCGAATCCCCACTGTTTCAAATGGCTGTCTCAGCAAATACCATCGAAAAGTACACACTGCTAGGCCCTTTCTTCAGAATA
TCTCCTCTGCAACAGGAAGTTACCAGGGAATACTTCAGTGCGCCAAAGACGATAGATAGGCGACACATTGCCACATCTCAAGATGCGTTACGAT
TGACCTTACAAACCCATCAAAAAGATTTACTTGATATCATCAACCACTTTGTTCGAGCAAGTCCAATCGCAAAAAGCAAAACCCTGGATTGGTT
CGCCTACATTGTGAATCAAAATCACAAGCGTCGAGCACTTCAGGTAGACCCGAAAGAAGTGTCTTCTGATGGCTTTATGCACAATGTCACTGTC
GTTCTAGATGGTCTTTGTGAGCCATTCATGGATACCACATTCTCGAAAATTTCGAAGATTGATATTGATTATCTAAGACGTGCGCCTCGTGTAG
ATATCAAGGACGAGACCAAGTTGAACGCTGACGAGAAGGCTTCTGAGAAGTATTATGAGGACACTGTTCCTGGCACTTCTAATTTCATCTCTGA
GGTCTTCTTTCTCACATTGGCTGCTCATCATTATGGTAGTGAAGCTCTTAATGCCACGCATAAGAGTCTGGAGAAAGACATCAAATATATTCAA
AAGCAATTGACTGCCGTTGAAGCA

>Bcin01g07220(MLST1), partial sequence [organism=Botrytis cinerea, strain D13_MR_S1]
ATGGCATATCTTGTTTCTTTTCGCATTATGCTTTGGAACGTGCTGGACAAGCTTGGGTTAGTGCTCTGCGTGTAGAAGCACTAAAGAAGATTCT
CGCACAACCGAAGTCATGGTTTGAGGAATCCAGGAATTCACCTGGCCGGTTGAACGAAGTTTTGGATAGGAACTCTGAGGAAATGCGTAATCTC
GTTGGCCGCTTTGCTGGTATTGTATTCACAGCATTTTTTATGCTATTGATATCAATCATTTGGGCTTTCGTGAATACATGGAAACTGACATTAG
TCTCAATGGCGACTGGGCCAGTTATATACGCTGTCACCAAAACGTTCAATCGCGTGAGTGGAAAATGGGAAAACAAGTGCAACTACGCATCTGA
AATGACCACTGGCATATTTTCAGAGACTTTCTCCAACATCAAAGTGGTTCGGGCTTTTACTCTGGAAACTTACTTTGAGACAAAACACACCAAA
GCTACAGAAGAACTCTATAAAATTGGACTAATACGAGCAAACTACTCGGGATTGCTGTGGGGATTGACAGATGCGATGTCATTCTTCATCACTG
CAACTATCTTTTATTATGCCACGGTTCTCATTACCAAGAGAGAGATCAGTATCGCGACTGCACTGCAGACTGTCAATCTTCTATTATTTGGTAT
TTCTAATAGTACGAATATGCTGGCCATGATACCACAAATCAACTCTTCTCGCGTTACAGCTACGCATATGCTTGCATTAGCCAATCTCGATTCA
TCTTCCTCCCACGAAAATAAAGGAGCCGAACGGCTTTCGACAATCTTTCCAATCAAATTCAACAGTCTCTCATTCACATATCCTACTCGTCCTG
AAAAACGAACGATATCATCCTTTTCTCTTTCCCTGATTCCTAACTCAACAACTGCACTTGTCGGACCCTCCGGCTCCGGAAAATCTACAATAGC
TGCTCTGCTCGTTGGTCTCTATCCGCCAGATACTTCAACACCTCCACCGTTGACATTCAATCGTGTCTCCATAAGTAACTGTCACATTCCGTCT
CTCCGGGCTTCTCTCTCACTCGTCCCACAAATACCGATTCTATTTCCAGCTACCATTCTCCATAACATCATTTATGGTCTCCCAGAATCTTCTC
CTTGTGCTAGTCTTCCATCTGCTATTGCATCAGCAAAAGATGCTGGGATCCATGAATTTATCACATCGCTTCCAAAAGGTTATGATACTATGG

>Bcin05g07690(MLST2), partial sequence [organism=Botrytis cinerea, strain D13_MR_S1]
ACCACTATCACCAAGTCCTTTTCTGCACTTTCCTCATCAAAAACGAACGAGAACATCATCAATAAATACTTTGCTGAAGTAATTGGGTTTTATT
TTGGCCAGGATGCCTTCTCTCTACGACAGCAAGCCTTCGATGATATGTTGTGGGTAGTTCTTGGCTGGCTGGATACTGTCAAATTCATTGATTT
ACATTCTGAATTGCACTATTCAAACGACTCTCAGCCAGAATGGTACGGACAACAATATAAACCTGCATTTGCACATCGAGCGCGACTATTTTGG
GAATTGGCTTCACAAGGATGGGATACTACTCTCTGTGGTGGTGGGATGATATGGTCACCATACCTTACTCCATACAAGAACGCAATTACCAATG
AACTCTATATCGCAGCTTCGATATCGATGTACCTATATTTCCCCGGAGATGACAATCAATCCCCATTTATGATTTCCAACCCTTCATATCCACC
TCACGATCCGAAATATCTACAGGCAGCTGTTGATGCTTACAAATGGCTGAATGGTTCCAACATGACGGATTCACAAGGATTATATGTCGACGGG
TACCATATCTCGAATCTTTCTGGCGGTGAAAACACCCATTGCGATTCTAGAAATGAGATGGTATATACCTACAATCAAGGTGTTTTGCTTACTG
GACAACGTGGTTTGTATGACGCAACCGCCGCACGATCATACCTTGTAGATGGCCACAAACTCATCGCGAATGTTATTAATGCCACAGGCTATGA
CCTGAAACACAATGTTGTCATCTCACCGCCACCCAAAGATGGTTCCGCATTGGCAAAGTGGTTTGGCCTGGGTAGGAATGGAATACTGGAAGAA
GGATGCGATTCAAGTGCTTCGTGTTCTCAAAATGGACAAACTTTCAAAGGCATATTCTTTCATCACTTGATTGCGTTCTGTAGTGATTTGCCAG
GGGAGCCTATTGCAGGGACGAAGGAAAGCTTAGAACTCGACAGAGTGTGGCATTCTGACAAATGCTCACAGTATACAAAAT

>Bcin06g01710(MLST3), partial sequence [organism=Botrytis cinerea, strain D13_MR_S1]
GGTGAGTCTGACTTTTGTGTTTGAGCGTTAAGATAGACACTGATGTACCAAGGCAATCACCTAATTTCATGGCGTTCTTCCCTGCATCATCTAC
CTACCCTGGAATGCTGGGAGAATTATACTCAGCAGCTTTCACAGCACCTGCTTTCAATTGGATCTGTTCCCCTGCCGTGACAGAATTGGAGACG
GTTGTAATGGATTGGCTGGCCAAGCTTCTCAATCTCCCAGACTGTTATTTGTCTTCGACTCATGGTGGTGGTGTTATCCAAGGATCAGCCTCGG
AAGCTATCGTTACCGTTATGGTTGCTGCCCGCGATAAATATCTTCGTGAAACTACTGAAGGTCTGTCGGGCATTGAACTCGAGGATGCGATTGC
ATATAAGAGGAGTAAGCTAGTTGCACTAGGAAGCGAAATGGCACACAGCTCCACGCAGAAAGCAGCGCAGATAGCTGGCGTTAGATTCCGATCG
ATTCCAGTACTCGCATCCAATGATTTCGCCATGACGGGTGATGATTTAGAGAAGGTATTGGAAGAATGCAAATCTCAAGGATTGGAACCCTTCT
ATCTAACTTCGACTTTGGGAACAACATCTACATGCGCAGTTGACGACTTCGCATCTATTGCAACAGTACTTTCAAAATATGCACCTCCAGATGT
TGCAGGCGAGATCTGGGTTCACGTCGATGCTGCTTATGCAGGTGCAGCTTTGGTTTGCCCTGAATACCATCATCTAACATCGTCCTTCCAGCAT
TTCCATTCCTTCGATATGAACATGCACAAATGGCTTCTGACAAATTTCGACGCTTCTTGTCTATATGTCAAGAAACGCAAAGATCTGATAGATG
CACTCTCCATAACACCAAGTTATCTTCGCAACGAGTTTTCAGAGAGTGGACTCGTAACCGACTATCGGGATTGGCAAATTCCTCTCGGAAGACG
CTTCCGAAGCTTAAAGATTTGGTTTGTCCTCAGAACCTACG

>Bcin09g03030(MLST4), partial sequence [organism=Botrytis cinerea, strain D13_MR_S1]
GACCCTTCAACCCCACCGCAGCTCCTATACCGAAAGCGAGTATCCTACCAATTCTCCTTCCACCTGCGACTTTAAGACCATTGGCTTTCCGCAC
TTTCACAAAAAAGCATAGTTTAACATTGACGTCGTCGGCATTACAAGTGTTGGCTACTTTTATTGGAAAGCATTGTGGGACAGGATGGAGGGAA

GAAGGACTGGCAGAGAGAGTCTTGGAGGAGGTCGCCAAGAGTTGGAAGAATAGGAGTGGCGGTGTCATTGTCGAGGGCGAGGGAACGGAATTGA
AGGAGATTCTGAAAGCTTTGGAAGGGAATATGAGTGGTGGAAGGATAGTCATAGGAAGAGAGCTAAGCCGGCAGAATAGTTTAGTACTGGGATC
ATCACAATATGGAGAGGTCAATCATACAAGACTTGGGCTACGGCCAGGGAATATACCCAGAGAGGATAGTCAGTCAAGTTTGGGAATGTCAACG
TTGGAGGTCAATGACGAGGAAGATGAGGATGGCCTGATGGATCCAAGAAGGTGGTTAAAAGTCATTGATGCATTTGAGCAACCTCGACTGGTGT
ACAATGTTGCTAAAAAGCACTTTGATAGGTATGTTTCAATGATAAAATTTCATCTGAATCGACTAACTCAGTACAGAGATACCTCCAAACCTTC
ATTGTTCCCACCTGCGTCTCATAAAACACTCCTCTTCCAAAACCGCTATAATGTTATCCATCAACGTCTCCTTCGCAATGAATCTTTTCAAACG
CCCGCTTTTCAAGGTGGCAAATCTTCCCTTCAACGCAGCACGTCCGCCATTACCACCCAACAACAATCATACAAATTAACGCCGATAGCTAATC
TTCTCGGTCGCAATCGCAGCTCTCATATGCTTCTCGGTCTCCTCAGTATTTCACCCACTGGTACCCTCGCCATCAATGACCTGACGGGCAGTAT
CGCTCTCGATCTTACACACGCAGCAGCCATTC

>Bcin11g01310(MLST5), partial sequence [organism=Botrytis cinerea, strain D13_MR_S1]
GACTGACATGGACCTCATGTGGAACCGGCGTAGAATGCGCAACGCTTGAAGTTCCGCTCGAATATGGCGATGCAACGTCAACGGCAAAAGCCAG
TGTTGCGCTTGCTCGTTATCCTGCCACTGTTGCCGCGAGCAAGAAGCTCGGGTCTCTCTTGATAAACCCCGGTGGACCCGGTGCCTCTGGTGTT
GGCTTTGTGCAGTCTGGAGCCGGTGCCGCCGTCTCGACACTGAGTGGTGGATTGTACGATATCATCGGATGGGATCCACGTGGAACCGGTGCTT
CGGCTCCTATTTTGGAATGTTTTGCAAATGCCAGTGCGGAGTATGATTTTAACAACGCGTTTCCATCTGCTCCGAATCTCTGGCTCGGACAATT
TGCGAATGCCAGCGCAAATTCTGCTGTTAGCTCTGCTATCACATCCTTTGACACTTCTGTCGCTGCTCTTGCAAAAGCTTGCGTGGCTCAGAAA
TCTCCCGCTCTTTACACCTCAACAGCAGCATATGTTGCTCGAGACATGGCAGCGATAGTCGATGCATTGGATGGGACCTCTGCAAAACTTAACT
ACTGGGGTTTCTCATATGGAACTATTTTCCTAGCTGAGTTTATCCAAACTTTCCCAGGCCGTGTGGGAAGAGTTCTTGCCGATGGTGTTTTCGA
CGCAAAGGCAAATGCACTCACATACGTTAGCCAACTTCCCAACGATCAACTCAGTGTTCGTGCTTCGTTGAACGATTTTGCAGCTTTCTGCACC
ACCGCCGGTAGTAAAGGTTGCTCTTTTGCCACCGCCCCTACTGGAACCTCAGGTACTGTTGCTACCAGACTGGACAACATAATGAAGGATATGT
TCCTCAATCCTATTGTTGCTTCGGGCTTAAGCATCA

>Bcin15g03910(MLST6), partial sequence [organism=Botrytis cinerea, strain D13_MR_S1]
GCCAAAACACAAAATCACCCAACGATGAAGATGATACTCCACTTCCCTTGATTATCTGGCATGGACTCGGCGATAATTACAAAGCGGATGGTCT
TGCGCAAGTTGGAAAACTAGCTGAAGCTATTCATCCTGGGACTTTTGTCTACAATATTCATGTAGATGAGGATGCATCTGCAGATAGGACAGCT
ACCTTCTTTGGAAATCTCACTCGTGAGTACATCCCTATTTTTCCTTTAAATATCATACTAACTCTCTTACCAAGTTCAAATCGAAAAGGTCTGC
GAAGACCTCGCCTCCCATCCTATTCTCTCTACCGCGCCCGCCGTCGACGCAATTGGATTCTCCCAAGGCGGCCAATTCTTGCGTGGTTACATAT
CCCGCTGCAATGCTCCACCCATCCGCTCTCTCCTGACCTTTGGTTCCCAACACAACGGCATTTCTGCCTTCCAAGCCTGTGGTCCTGCCGATTT
CCTCTGTCGCGGTGCTCAAACCCTTTTGCGATCCAACACCTGGTCAACCTTTGTCCAATCTCGTCTCGTACCCGCTCAATACTTCAGAGATCCG
GAAAACCTAGACTCTTACCTTGAATATTCCAATTTCCTTGCCGACATCAATAATGAGCGCGTTCTCAAGAACCAAACATATAAATCCAACATGG
AAAAATTGGAACGATTCGTAATGTATGTCTTTGAAGACGACACAACTGTCATTCCTAAGGAAAGTGGATGGTGGGCTGAAGTCAACGGCACGGA
AGTTACACCACTGAAAGAAAGAGCCATTTATAAAGAAGATTGGCTAGGTTTAAAGACATTGGATGAGGCCGGAAAATTAGTTTTCGAAACCATT
CCAGGGGGACATATGACGTTAGGAGAGGAGATGCTAGAGAAGGCTTTCAAAGAGTATTTTGGTCCAGCAGGGAAGAAATTTGGGG

>Bcin16g03460(MLST7), partial sequence [organism=Botrytis cinerea, strain D13_MR_S1]
TCATGAACCTCTTGATTTGAACTCTTCATTAATTGCAAGTGATGAACCCCTATTCTGCCCAGAGGATAGTTACAAGACGTATATCATTAGTCGA
GAACCACTCATGATATATATTGACGGATTTTTGAAAGCGAATGAAAGTAAACATTTGGTTGATGTTAGGTGTGTTATTTATTCTGATGAAATGA
ACAAGAGAGACTGATGAGATAGTGAGCCGCTTTATGAACCGTCAACTGTTTCTCACGGACAGGAAGTTACCATTGATCCTTCAGTTCGGAATTC
TGAAGTGGCGGTTTTAGAGAGGGATGAGGTGGTCAGGTGTATTGAGCATAGAGCGAGGGCATTTCAGGGGTGGAGGGGCGAGATGGGGATTGAG
AAGTTGAGGACGCAGAGGTATGGGGTTGGAGGACATTATGGGATGCATTTGTAAGTTTTGGGGGATTGACGAAGGCTTTTGTCTATTTTCTACC
AGTACGGATCTTGAAAGAAAGATAGCATGAACACGAGGGCTAATAACTAGGAAATAGCGATTGGAGCGGAGGTAAACGTGGCATAGACCGATTT
AGTACTTTCATGGTCTATGTCGACGTATCCTCTGATATCGAAGGTGGAGGAACGGAATTCCCACGTATCGTGGGACCAAAAGGAGGAAGGTGGG
AGGACTTCCTGGAAACTACGGAAGCATTGGATCCAAGAACTGGAGAAAATGTAACAGTAGAAGGGGTGACATTCAAACCAATCAAGGGAAATGC
CGTATTCTGGGAAAATACTGACAACAACGGGAGGGGCTATGAT

>Bcin12g03020(MLST8), partial sequence [organism=Botrytis cinerea, strain D13_MR_S1]
CGATTGGCTGCGAAGAAAACTGCGCAGCACCGAGTCACCAAAAGCACGAATAAACCATCGGCAAGGGAAATATTCTCTGAACCACAACATGACA
CGAGCGCGGAAGAGTATATCGGGCGAGAAGCTTCATCAAGAGCACCAAAGCGACAACGAGTCGATGATAATTATAACTCTTACGGTGGAAGAAA
TGAAAATACAGCAGCTTATGCTTCCGGGAAACTTCCATCTGGAAGTATAAATGTTGGTGGAGGTAGGAAGACCACTTTTCAAGAAGAACCACGA
ACGGCATTTGTCGCTGGCAAGTTGCCCCCTGGAAGTATCAACATTGGTGGAAAGAAGGCTACCCAAATCGAGGACGAGGGTAGAGCAGCTTATG
CTTCCGGAAAATTGCCTCCAGGAAGTATAAACTCTGGCGCAAAGAAGGCGATTTCATTCCAAGATGAAACGAGACCGGCTTATGTCTCCGGAAA
GCTTCCACATGGTAGTATCGACGGTATGCGAAACCGTGAAATGGCTGCCGTCCACCGCGAAATTGCTGAGGGTGGGAGGAAACCAGGCCAGGTT
GTCTCTTCTCTATTCACATTCAATCCTACTTCAAAGAAAACTTTTGATGAACCCGAAGAACAAGCGGAACCGGCAAAACCATCCAATGCGCCTT
TGACAGAGGAAATGGCGACTTTCACCAATCTAGGGTTATCGAGAAGGCTTGCAGCCCATCTATCGACTAAACTCGATATGAAAGCCCCGACGGC
CATTCAAAAGCATCTGTGCAGCAGTTGGTATCGGACGATAGCGATGCTTTCATACAAGCAGAGACTGGATCTGGAAAAACTTTGGCATATCTAC
TACCTATAGTCGAGCGAATATTAGCATTGAGTGAGAATGGCGTACAAA

>Bcin02g07770(MLST9), partial sequence [organism=Botrytis cinerea, strain D13_MR_S1]
ACAGCTTTCCCTTTCGGTCTTGGCATCTACAGTCATTGCCATCCCTACACCATCACAACTTGAGTCTCGGGCCGTTATCGATTCCGATGCCGTT
GTAGGATTTGCCGAAACTGTTCCCAGTGGGACCGTAGGAACAGTTTATGAGGCATATAAACCATTCCTTAAAGTCGTAAATGGATGCGTACCAT
TCCCTGCCGTCGATGCATCGGGTAACACAGGGTATGTCCTTATACCTTTCTCTTCCACACGATTGCTATTGAGTCTCTAACATATTTTAGTGGT
GGTTTGTCACCAACTGGCAGCAGCAATGGTGAATGCAGCAGCAGTACCGGTCAAGTATATGTTCGAGGAGGACAAAGCGGATCAAACTACGCTA
TCATGTACTCCTGGTAAGTTCTCTTTAAACTTCTCCTTATAGATCCAACCTAACAAAATCTTAGGTACATGCCAAAGGACGAGCCCTCAACCGG
TATTGGTCACCGTCACGATTGGGAAGGTGTAATTGTCTGGCTCTCCAGCGCCACCGCCACAACTGCCGACAACATCTTAGCCGTTTGTCCTTCC
GCCCACGGAGGCTGGGATTGTTCCACGGATGGCTATTCCCTTTCTGGTACCAGCCCTCTTATCAAGTACGAAAGTATCTGGCCCGTCGATCACT
CAATGGGTCTTACTAGTACTGTTGGTGGAAAACAACCTATGATTGCTTGGGAGTCTTTACCAACTGCTGCTCAAACTGCTCTTGAGAACACCGA
TTTCGGTGCTGCGAATGTTCCATTCATTCCGGCTGTTTTCACAGATAATCTT

>Bcin04g02090(MLST10), partial sequence [organism=Botrytis cinerea, strain D13_MR_S1]
CGGAGGATGATATGGCAAAGTCTATGATTACCAAAGCATTTGTAGGCATGAGTAGTAAATTGTCGAATATGACTATGAATGATGTTTACAAGCC
CTACATCCATGTAAGAAATGTAGAATAAAGAGATCAGTAACTGGAACTAATATCGTTTGCAGGCTTTCAAGTTACTTACGCAGTTCAACCCAAT
CACTACAGCTATTGCCGAATCCCCACTATTTCAAATGGCTGTCTCAGCAAATACCATCGAAAAGTACACACTGCTAGGCCCTTTCTTCAGAGTA
TCTCCTCTGCAACAGGAAGTTACCAGGGAATACTTCAGTGCGCCAAAGACGATAGATAGGCGACACATTGCCACATCTCAAGATGCGTTACGAT
TGACCTTACAAACCCATCAAAAAGATTTACTTGATATCATCAACCACTTTGTTCGAGCAAGTCCAATCGCAAAAAGCAAAACCCTGGATTGGTT
CGCCTACATTGTGAATCAAAATCACAAGCGTCGAGCACTTCAGGTAGACCCGAAAGAAGTGTCTTCTGATGGCTTTATGCACAATGTCACTGTC
GTTCTAGATGGTCTTTGTGAGCCATTCATGGATACCACATTCTCAAAAATTTCGAAGATTGATATTGATTATCTAAGACGTGCGCCCCGTGTAG
ATATCAAGGACGAGACCAAGTTGAACGCTGATGAGAAGGCTTCTGAGAAGTATTATGAGGACACTGTTCCTGGCACTTCTAATTTCATCTCTGA
GGTATTCTTTCTGACATTGGCTGCTCATCATTATGGTAGTGAAGCTCTTAATGCCACGCATAAGAGTCTGGAGAAAGACATCAAATATATTCAA
AAGCAATTGACTGCCGTTGAAGCA

>Bcin01g07220(MLST1), partial sequence [organism=Botrytis cinerea, strain D13_MR_S29]
ATGGCATATCTTGTTTCTTTTCGCATTATGCTTTGGAACGTGCTGGACAAGCTTGGGTTAGTGCTCTGCGTGTAGAAGCACTAAAGAAGATTCT
CGCACAACCGAAGTCATGGTTTGAGGAATCCAGGAATTCACCTGGCCGGTTGAACGAAGTTTTGGATAGGAACTCTGAGGAAATGCGTAATCTC
GTTGGCCGCTTTGCTGGTATTGTATTCACAGCATTTTTTATGCTATTGATATCAATCATTTGGGCTTTTGTGAATACATGGAAACTGACATTAG
TCTCGATGGCAACTGGGCCAGTTATATACGCTGTCACCAAAACATTCAATCGCGTGAGTGGAAAATGGGAAAACAAGTGCAACTACGCCTCTGA
AATGACCACTGGCATATTTTCAGAGACTTTCTCCAACATCAAAGTGGTTCGGGCTTTTACTCTGGAAACTTACTTTGAGACAAAACACACCAAA
GCTACAGAAGAACTTTATAAAGTTGGACTAATACGAGCAAACTACTCGGGATTGCTGTGGGGATTGACAGATGCGATGTCATTCTTCATCACTG
CAACTATCTTTTATTATGCCACGGTTCTCATTACCAAGAGAGAGATCAGTATCGCGACTGCACTACAGACTGTCAATCTTCTATTATTTGGTAT
TTCTAATAGTACGAATATGCTGGCCATGATACCACAAATCAACTCTTCTCGCGTTACAGCTACGCATATGCTTGCATTAGCCAATCTCGATTCA
TCTTCCTCCCACGAAAATAAAGGAACCGAACGGCTTTCGACAATCTTTCCAATCAAATTCAACCGTCTTTCATTCACATACCCTACTCGTCCTG
AAAAACGAACGATATCATCCTTTTCTCTTTCCCTGATTCCTAACTCAACAACTGCACTTGTCGGACCCTCCGGCTCCGGAAAATCTACAATAGT
TGCTCTGCTCATTGGTCTCTATCCGCCAGATACTTCAACACCTCCACCGTTGACATTCAATCGCGTCTCCATAAGTAACTGTCACATTCCGTCT
CTCCGGGCTTCTCTCTCACTCGTTCCACAAATACCGATTCTATTTCCAGCTACCATTCTCCATAACATCATTTATGGTCTCCCAGAATCTTCTC
CTTGTGCTAGTCTTCCATCAGCTATTGCATCAGCAAAAGATGCTGGGATCCATGAATTTATCACATCGCTTCCACAAGGTTATGATACTAT

>Bcin05g07690(MLST2), partial sequence [organism=Botrytis cinerea, strain D13_MR_S29]
ACCACTATCACCAAGTCCTTTTCTGCACTTTCCTCATCAAAAACGAACGAGAACATCATCAATAAATACTTTGCTGAAGTAATTGGGTTTTATT
TTGGCCAGGATGCCTTCTCTCTACGACAGCAAGCCTTCGATGATATGTTGTGGGTAGTTCTTGGCTGGCTGGATACTGTCAAATTCATTGATTT
ACATTCTGAATTGCACTATTCAAACGACTCTCAGCCAGAATGGTACGGACAACAATATAAACCTGCATTTGCACATCGAGCGCGACTATTTTGG
GAATTGGCTTCACAAGGATGGGATACTACTCTCTGTGGTGGTGGGATGATATGGTCACCATACCTTACTCCATACAAGAACGCAATTACCAATG
AACTCTATATCGCAGCTTCGATATCGATGTACCTATATTTCCCCGGAGATGACAATCAATCCCCATTTATGATTTCCAACCCTTCATATCCACC
TCACGATCCGAAATATCTACAGGCAGCTGTTGATGCTTACAAATGGCTGAATGGTTCCAACATGACGGATTCACAAGGATTATATGTCGACGGG
TACCATATCTCGAATCTTTCTGGCGGTGAAAACACCCATTGCGATTCTAGAAATGAGATGGTATATACCTACAATCAAGGTGTTTTGCTTACTG
GACAACGTGGTTTGTATGACGCAACCGCCGCACGATCATACCTTGTAGATGGCCACAAACTCATCGCGAATGTTATTAATGCCACAGGCTATGA
CCTGAAACACAATGTTGTCATCTCACCGCCACCCAAAGATGGTTCCGCATTGGCAAAGTGGTTTGGCCTGGGTAGGAATGGAATACTGGAAGAA
GGATGCGATTCAAGTGCTTCGTGTTCTCAAAATGGACAAACTTTCAAAGGCATATTCTTTCATCACTTGATTGCGTTCTGTAGTGATTTGCCAG
GGGAGCCTATTGCAGGGACGAAGGAAAGCTTAGAACTCGACAGAGTGTGGCATTCTGACAAATGCTCACAGTATACAAAAT

>Bcin06g01710(MLST3), partial sequence [organism=Botrytis cinerea, strain D13_MR_S29]
GTGAGTCTGACTTTTGTATTTGAGCGTTAAGATAGACACTGATATACCAAGGCAATCACCTAATTTCATGGCGTTCTTCCCTGCATCATCTACC
TACCCTGGAATGCTGGGAGAATTATACTCAGCAGCTTTCACAGCACCTGCTTTCAATTGGATCTGTTCCCCTGCTGTGACAGAATTGGAGACGG
TTGTAATGGATTGGCTGGCCAAGCTTCTCAATCTCCCAGACTGTTATTTGTCTTCGACTCATGGTGGTGGTGTCATCCAAGGATCAGCCTCGGA
AGCTATCGTTACCGTTATGGTTGCTGCCCGCGATAAATATCTTCGTGAAACTACTGAAGGTCTGTCGGGCATTGAACTCGAGGATGCGATTGCA
TATAAGAGGAGTAAGCTAGTTGCACTAGGAAGCGAAATGGCACACAGCTCCACGCAGAAAGCAGCGCAAATAGCTGGCGTTAGATTCCGATCGA

TTCCAGTACTCGCATCCAATGATTTCGCCATGACGGGTGATGATTTAGAGAAGGTATTGAAAGAATGCAAATCTCAAGGATTGGAACCCTTCTA
TCTAACTTCGACTTTGGGAACAACATCTACATGCGCAGTTGACGACTTCGCATCTATTGCAACAGTACTTTCAAAATATGCACCTCCAGATGTT
GCAGGCGAGATCTGGGTTCACGTCGATGCTGCTTATGCAGGTGCAGCTTTGGTTTGCCCTGAATACCATCATCTAACATCGTCTTTCCAGCATT
TCCATTCCTTCGATATGAACATGCACAAATGGCTTCTGACAAATTTCGACGCTTCTTGTCTATATGTCAAGAAACGCAAAGATCTGATCGATGC
ACTGTCCATAACACCAAGTTATCTTCGCAACGAGTTTTCAGAGAGTGGACTCGTAACCGACTATCGGGACTGGCAAATTCCTCTCGGAAGACGC
TTCCGAAGCTTAAAGATTTGGTTTGTCCTCAGAACCTAC

>Bcin09g03030(MLST4), partial sequence [organism=Botrytis cinerea, strain D13_MR_S29]
ACCCTTCAACCCCACCGCAGCTCCTATACCGAAAGCGAGTATCCTACCAATTCTCCTTCCACCTGCGACTTTAAGACCATTGGCTTTCCGCACT
TTCACAAAAAAGCATAGTTTAACATTGACATCGTCGGCATTACAAGTGTTGGCTACTTTTATTGGAAAGCATTGTGGGACAGGATGGAGGGAAG
AAGGACTGGCAGAGAGAGTCTTGGAGGAGGTCGCCAAGAGTTGGAAGAATAGGAGTGGCGGTGTCATTGTCGAGGGCGAGGGAACGGAATTGAA
GGAGATTCTGAAAGCTTTGGAAGGGAATATGAGTGGTGGAAGGATAGTCATAGGAAGAGAGCTAAGCCGGCAGAATAGTTTAGTACTGGGATCA
TCACAATATGGAGAGGTCAATCATACAAGACTTGGGCTACGGCCAGGGAATATACCCAGAGAGGATAGTCAGTCAAGTTTGGGAATGTCAACGT
TGGGGTCAATGACGAGGAAGATGAGGATGGCCTGATGGATCCAAGAAGGTGGTTAAAAGTCATTGATGCATTTGAGCAACCTCGACTGGTGTAC
AATGTTGCTAAAAAGCACTTTGATAGGTATGTTTCAATGATAAAATTTCATCTGAATCGGCTAACTCAGTACAGAGATACCTCCAAACCTTCAT
TGTTCCCACCTGCGTCTCATAAAACACTCCTCTTCCAAAACCGCTATAATGTTATCCATCAACGTCTCCTTCGCAATGAATCTTTTCAAACGCC
CGCTTTTCAAGGTGGCAAATCTTCCCTTCAACGCAGCACGTCCGCCATTACCACCCAACAACAATCATACAAATTAACGCCGATAGCTAATCTT
CTCGGTCGCAATCGCAGCTCTCATATGCTTCTCGGTCTCCTCAGTATTTCACCCACTGGTACCCTCGCCATCAATGACCTGACGGGCAGTATCG
CTCTCGATCTTACACACGCAGCAGCCATTC

>Bcin11g01310(MLST5), partial sequence [organism=Botrytis cinerea, strain D13_MR_S29]
GGCTGACATGGACCTCATGTGGAACCGGCGTAGAATGCGCAACGCTTGAAGTTCCGCTCGAATATGGCGATGCAACGTCAACGGCAAAAGCCAG
TGTTGCGCTTGCTCGTTATCCTGCCACTGTTGCCGCGAGCAAGAAGCTCGGGTCTCTCTTGATAAACCCCGGTGGACCCGGTGCCTCTGGTGTT
GGCTTTGTGCAGTCTGGAGCCGGTGCCGCCGTCTCGACACTGAGTGGTGGATTGTACGATATCATCGGATGGGATCCACGTGGAACCGGTGCTT
CGGCTCCTATTTTGGAATGTTTTGCAAATGCCAGTGCGGAGTATGATTTTAACAACGCGTTTCCATCTGCTCCGAATCTCTGGCTCGGACAATT
TGCGAATGCCAGCGCAAATTCTGCTGTTAGCTCTGCTATCACATCCTTTGACACTTCTGTCGCTGCTCTTGCAAAAGCTTGCGTGGCTCAGAAA
TCTCCCGCTCTTTACACCTCAACAGCAGCATATGTTGCTCGAGACATGGCAGCGATAGTCGATGCATTGGATGGGACCTCTGCAAAACTTAACT
ACTGGGGTTTCTCATATGGAACTATCTTCCTAGCTGAGTTTATCCAAACTTTCCCAGGCCGCGTGGGAAGAGTTCTTGCCGATGGTGTTTTCGA
CGCAAAGGCAAATGCACTCACATACGTTAGCCAACTTCCCAACGATCAACTCAGTGTTCGTGCTTCGTTGAACGATTTTGCAGCTTTCTGCACC
ACCGCCGGTAGTAAAGGTTGCTCTTTTGCCACCGCCCCTACTGGAACCTCAGGTACTGTTGCTACCAGACTGGACAACATAATGAAGGATATGT
TCCTCAATCCTATTGTTGCTTCGGGCTTAAGCATCAG

>Bcin15g03910(MLST6), partial sequence [organism=Botrytis cinerea, strain D13_MR_S29]
CGCCAAAACACAAAATCATCCAACGATGAAGATGATACTCCACTTCCCTTGATTATCTGGCATGGACTCGGCGATAATTACAAAGCGGATGGTC
TTGCGCAAGTTGGAAAACTAGCTGAAGCTATTCATCCTGGGACTTTTGTCTACAATATTCATGTAGATGAGGATGCATCTGCAGATAGGACAGC
TACCTTCTTTGGAAATCTCACTCGTGAGTACATCCCCTATTTTTCCTTTAAATATCATACTAACTCTCTTACCAAGTTCAAATCGAAAAGGTCT
GCGAAGACCTCGCCTCCCATCCTATTCTCTCTACCGCGCCCGCCGTCGACGCAATTGGATTCTCCCAAGGCGGCCAATTCTTGCGTGGTTACAT
ATCCCGCTGCAATACTCCACCCATCCGCTCTCTCCTGACCTTCGGTTCCCAACACAACGGCATTTCTGCCTTCCAAGCCTGTGGTCCTGCCGAT
TTCCTCTGTCGCGGTGCTCAAACCCTTTTGCGATCCAACACCTGGTCAACCTTTGTCCAATCTCGTCTCGTACCCGCTCAATACTTCAGAGATC
CGGAAAACCTAGACTCTTACCTTGAATATTCCAATTTCCTTGCCGACATCAATAATGAGCGCGTTCTCAAGAACCAAACATATAAATCCAACAT
GGAAAAATTGGAACGATTCGTAATGTATGTCTTTGAAGACGACACAACTGTCATTCCTAAGGAAAGTGGATGGTGGGCTGAAGTCAACGGCACG
GAAGTTACACCACTGAAAGAAAGAGCCATTTATAAAGAAGATTGGCTAGGTTTAAAGACATTGGATGAGGCCGGAAAATTAGTTTTCGAAACCA
TTCCAGGGGGACATATGACGTTAGGAGAGGAGATGCTAGAGAAGGCTTTCAAAGAGTATTTTGGTCCAGCAGGGAAGAAATTTGGG

>Bcin16g03460(MLST7), partial sequence [organism=Botrytis cinerea, strain D13_MR_S29]
CATGAACCTCTTAATTTGAACTCTTCATTATTTGCAAGTGATGAACCCCTATTCTGCCCAGAGGATAGTTACAAGACGTATATCATTAGTCGAG
AACCACTCATGATATATATTGACGGATTTTTGAAAGCGAATGAAAGTAAACATTTGGTTGATGTTAGGTGTGTTATTTATTCTGATGAAATGAA
CAAGAGAGACTGATGAGATAGTGAACCGCTTTATGAACCGTCTACTGTTTCTCACGGGCAGGAAGTTACCATTGATACTTCAGTTCGAAATTCT
GAAGTGGCGGTTTTAGAGAGGGATGAGGTGGTCAGGTGTATTGAGCATAGAGCGAGGGCATTTCAGGGGTGGAGGGGCGAGATGGGGATTGAGA
AGTTGAGGACGCAGAGGTATGGGGTTGGAGGACATTATGGGATGCATTTGTAAGTTTTGGGGGATTGACGAAGGCTTTTGTCTATTTTCTACCA
GTACGGATCTTGAAAGAAAGATAGCATGAACACGAGGGCTAATAACTAGGAAATAGCGATTGGAGCGGAGGTAAACGTGGCATAGACCGATTTA
GTACTTTCATGGTCTATGTCGACGTATCCTCTGATATCGAAGGTGGAGGAACGGAATTCCCACGTATCGTGGGACCAAAAGGAGGAAGGTGGGA
GGACTTCCTGGAAACTACGGAAGCATTGGATCCAAGAACTGGAGAAAATGTAACAGTAGAAGGGGTGACATTCAAACCAATCAAGGGAAATGCC
GTATTCTGGGAAAATACTGACAACAACGGGAGGGGCTATGAT

>Bcin12g03020(MLST8), partial sequence [organism=Botrytis cinerea, strain D13_MR_S29]
CGATTGGCTGCGAAGAAAACTGCGCAGCACCGAGTCACCAAAAGCACGAATAAACCATCGGCAAGGGAAATATTCTCTGAACCACAACATGACA

CGAGCGCGGAAGAGTATATCGGGCGAGAAGCTTCATCAAGAGCACCAAAGCGACAACGAGTCGATGATAATTATAACTCTTACGGTGGAAGAAA
TGAAAATACAGCAGCTTATGCTTCCGGGAAACTTCCATCTGGAAGTATAAATGTTGGTGGAGTAGGAAGACCACTTTTCAAGAAGAACCACGAA
CGGCATTTGTCGCTGGCAAGTTGCCCCCTGGAAGTATCAACATTGGTGGAAAGAAGGCTACCCAAATCGAGGACGAGGGTAGAGCAGCTTATGC
TTCCGGAAAATTGCCTCCAGGAAGTATAAACTCTGGCGCAAAGAAGGCGATTTCATTCCAAGATGAAACGAGACCGGCTTATGTCTCCGGAAAG
CTTCCACATGGTAGTATCGACGGTATGCGAAACCGTGAAATGGCTGCCGTCCACCGCGAAATTGCTGAGGGTGGGAGGAAACCAGGCCAGGTTG
TCTCTTCTCTATTCACATTCAATCCTACTTCAAAGAAAACTTTTGATGAACCCGAAGAACAAGCGGAACCGGCAAAACCATCCAATGCGCCTTT
GACAGAGGAAATGGCGACTTTCACCAATCTAGGGTTATCGAGAAGGCTTGCAGCCCATCTATCGACTAAACTCGATATGAAAGCCCCGACGGCC
ATTCAAAAAGCATCTGTGCAGCAGTTGGTATCGGACGATAGCGATGCTTTCATACAAGCAGAGACTGGATCTGGAAAAACTTTGGCATATCTAC
TACCTATAGTCGAGCGAATATTAGCATTGAGTGAGAATGGCGTACAAA

>Bcin02g07770(MLST9), partial sequence [organism=Botrytis cinerea, strain D13_MR_S29]
CAGCTTTCCCTTTCGGTCTTGGCATCTACAGTCATTGCCATCCCTACACCATCACAACTTGAGTCTCGGGCCGTTATCGATTCCGATGCCGTTG
TAGGATTTGCCGAAACTGTTCCCAGTGGGACCGTAGGAACAGTTTATGAGGCATATAAACCATTCCTTAAAGTCGTAAATGGATGCGTACCATT
CCCTGCCGTCGATGCATCGGGTAACACAGGGTATGTCCTTATATCTTTCTCTTCCACACGATTGCTATTGAGTCTCTAACATATTTTAGTGGTG
GTTTGTCACCAACTGGCAGTAGCAATGGTGGTTGCAGCAGCAGTACCGGTCAAGTATATGTTCGAGGAGGACAAAGCGGATCAAACTACGCCAT
CATGTACTCCTGGTAAGTTCTCTCTAAACTTCTCCTTATAGATCCAACCTAACAAAATCTTAGGTACATGCCAAAGGACGAGCCCTCAACCGGT
ATTGGTCACCGTCACGATTGGGAAGGTGTAATTGTCTGGCTCTCCAGCGCCACCGCCACAACTGCCGACAACATCTTAGCCGTTTGTCCTTCCG
CCCACGGAGGCTGGGATTGTTCCACGGATGGCTATTCCCTTTCTGGTACCAGCCCTCTTATCAAGTACGAAAGTATCTGGCCCGTCGATCACTC
AATGGGTCTTACTAGTACTGTTGGTGGAAAACAACCTATGATTGCTTGGGAGTCTTTACCAACTGCTGCTCAAACTGCTCTTGAGAACACCGAT
TTCGGTGCTGCGAATGTTCCATTCATTCCGGCTGTTTTCACAGACAATCT

>Bcin04g02090(MLST10), partial sequence [organism=Botrytis cinerea, strain D13_MR_S29]
CGGAGGATGATATGGCAAAGTCTATGATTACCAAAGCATTTGTAGGCATGAGTAGTAAATTGTCGAATATGACTATGAATGATGTTTACAAGCC
CTACATCCATGTAAGAAATGTAGAATAAAGAGATCAGTAACTGGAACTAATATCGTTTGCAGGCTTTCAAGTTACTTACGCAGTTCAACCCAAT
CACTACAGCTATTGCCGAATCCCCACTATTTCAAATGGCTGTCTCAGCAAATACCATCGAAAAGTACACACTGCTAGGCCCCTTTCTTCAGAATA
TCTCCTCTGCAACAGGAAGTTACCAGGGAATACTTCAGTGCGCCAAAGACGATAGATAGGCGACACATTGCCACATCTCAAGATGCGTTACGAT
TGACCTTACAAACCCATCAAAAAGATTTACTTGATATCATCAACCACTTTGTTCGAGCAAGTCCAATCGCAAAAAGCAAAACCCTGGATTGGTT
CGCCTACATTGTGAATCAAAATCACAAGCGTCGAGCACTTCAGGTAGACCCGAAAGAAGTGTCTTCTGATGGCTTTATGCACAATGTCACTGTC
GTTCTAGATGGTCTTTGTGAGCCATTCATGGATACCACATTCTCAAAAATTTCGAAGATTGATATTGATTATCTAAGACGTGCGCCTCGTGTAG
ATATCAAGGACGAGACCAAGTTGAACGCTGATGAGAAGGCTTCTGAGAAGTATTATGAGGACACTGTTCCTGGCACTTCTAATTTCATCTCTGA
GGTATTCTTTCTGACATTGGCTGCTCATCATTATGGTAGTGAAGCTCTTAATGCCACGCATAAGAGTCTGGAGAAAGACATCAAATATATTCAA
AAGCAATTGACTGCCGTTGAAGCA

>Bcin01g07220(MLST1), partial sequence [organism=Botrytis cinerea, strain D13_MR_S4]
ATGGCATATCTTGTTTCTTTTCGCATTATGCTTTGGAACGTGCTGGACAAGCTTGGGTTAGTGCTCTGCGTGTAGAAGCACTAAAGAAGATTCT
CGCACAACCGAAGTCATGGTTTGAGGAATCCAGGAATTCACCTGGCCGGTTGAACGAAGTTTTGGATAGGAACTCTGAGGAAATGCGTAATCTC
GTTGGCCGCTTTGCTGGTATTGTATTCACAGCATTTTTTATGCTATTGATATCAATCATTTGGGCTTTTGTGAATACATGGAAACTGACATTAG
TCTCGATGGCAACTGGGCCAGTTATATACGCTGTCACCAAAACATTCGATCGCGTGAGTGGAAAATGGAAAACAAGTGCAACTACGCCTCTGA
AATGACCACTGGCATATTTTCAGAGACTTTCTCCAACATCAAAGTGGTTCGGGCTTTTACTCTGGAAACTTACTTTGAGACAAAACACACCAAA
GCTACAGAAGAACTTTATAAAGTTGGACTAATACGAGCAAACTACTCGGGATTGCTGTGGGGATTGACAGATGCGATGTCATTCTTCATCACTG
CAACTATCTTTTATTATGCCACGGTTCTCATTACCAAGAGAGAGATCAGTATCGCGACTGCACTACAGACTGTCAATCTTCTATTATTTGGTAT
TTCTAATAGTACGAATATGCTGGCCATGATACCACAAATCAACTCTTCTCGCGTTACAGCTACGCATATGCTTGCATTAGCCAATCTCGATTCA
TCTTCCTCCCACGAAAATAAAGGAACCGAACGGCTTTCGACAATCTTTCCAATCAAATTCAACCGTCTTTCATTCACATACCCTACTCGTCCTG
AAAAACGAACGATATCATCCTTTTCTCTTTCCCTGATTCCTAACTCAACAACTGCACTTGTCGGACCCTCCGGCTCCGGAAAATCTACAATAGT
TGCTCTGCTCATTGGTCTCTATCCGCCAGATACTTCAACACCTCCACCGCTGACATTCAATCGCGTCTCCATAAGTAACTGTCACATTCCGTCT
CTCCGGGCTTCTCTCTCACTCGTTCCACAAATACCGATTCTATTTCCAGCTACCATTCTCCATAACATCATTTATGGTCTCCCAGAATCTTCTC
CTTGTGCTAGTCTTCCATCAGCTATTGCATCAGCAAAAGATGCTGGGATCCATGAATTTATCACATCGCTTCCACAAGGTTATGATACTAT

>Bcin05g07690(MLST2), partial sequence [organism=Botrytis cinerea, strain D13_MR_S4]
ACCACTATCACCAAGTCCTTTTCTGCACTTTCCTCATCAAAAACGAACGAGAACATCATCAATAAATACTTGCTGAAGTAATTGGGTTTTATTT
TGGCCAGGATGGCTTCTCTCTACGACAGCAAGCCTTCGATGATATGTTGTGGGTAGTTCTTGGCTGGCTGGATACTGTCAAATTCATTGATTTA
CATTCTGAATTGCACTATTCAAACGACTCTCAGCCAGAATGGTACGGACAACAATATAAACCTGCATTTGCACATCGAGCGCGACTATTTTGGG
AATTGGCTTCACAAGGATGGGATACTACTCTCTGTGGTGGTGGGATGATATGGTCACCATACCTTACTCCATACAAGAACGCAATTACCAATGA
ACTCTATATCGCAGCTTCGATATCGATGTACCTATATTTCCCCGGAGATGACAATCAATCCCCATTTATGATTTCCAACCCTTCATATCCACCT
CACGATCCGAAATATCTACAGGCAGCTGTTGATGCTTACAAATGGCTGAATGGTTCCAACATGACGGATTCACAGGATTATATGTCGACGGGTA
CCATATCTCGAATCTTTCTGGCGGTGAAAACACCCATTGCGATTCTAGAAATGAGATGGTATATACCTACAATCAAGGTGTTTTGCTTACTGGA
CAACGTGGTTTGTATGACGCAACCGCCGCACGATCATACCTTGTAGATGGCCACAAACTCATCGCGAATGTTATTAATGCCACGGGCTATGACC

TGAAACACAATGTTGTCATCTCACCGCCACCCAAAGATGGTTCCGCATTGGCAAAGTGGTTTGGCCTGGGTAGGAATGGAATACTGGAAGAAGG
ATGCGATTCAAGTGCTTCGTGTTCTCAAAATGGACAAACTTTCAAAGGCATATTCTTTCATCACTTGATTGCGTTCTGTAGTGATTTGCCAGGG
GAGCCTATTGCAGGGACGAGGAAAGCTTAGAACTCGACAGAGTGTGGCATTCTGACAAATGCTCACAGTATACAAAAT

>Bcin06g01710(MLST3), partial sequence [organism=Botrytis cinerea, strain D13_MR_S4]
GTGAGTCTGACTTTTGTGTTTGAGCGTTAAGATAGACACTGATGTACCAAGGCAATCACCTAATTTCATGGCGTTCTTCCCTGCATCATCTACC
TACCCTGGAATGCTGGGAGAATTATACTCAGCAGCTTTCACAGCACCTGCTTTCAATTGGATCTGTTCCCCTGCCGTGACAGAATTGGAGACGG
TTGTAATGGATTGGCTGGCCAAGCTTCTCAATCTCCCAGACTGTTATTTGTCTTCGACTCATGGTGGTGGTGTTATCCAAGGATCAGCCTCGGA
AGCTATCGTTACCGTTATGGTTGCTGCCCGCGATAAATATCTTCGTGAAACTACTGAAGGTCTGTCGGGCATTGAACTCGAGGATGCGATTGCA
TATAAGAGGAGTAAGCTAGTTGCACTAGGAAGCGAAATGGCACACAGCTCCACGCAGAAAGCAGCGCAGATAGCTGGCGTTAGATTCCGATCGA
TTCCAGTACTCGCATCCAATGATTTCGCCATGACGGGTGATGATTTAGAGAAGGTATTGGAAGAATGCAAATCTCAAGGATTGGAACCCTTCTA
TCTAACTTCGACTTTGGGAACAACATCTACATGCGCAGTTGACGACTTCGCATCTATTGCAACAGTACTTTCAAAATATGCACCTCCAGATGTT
GCAGGCGAGATCTGGGTTCACGTCGATGCTGCTTATGCAGGTGCAGCTTTGGTTTGCCCTGAATACCATCATCTAACATCGTCCTTCCAGCATT
TCCATTCCTTCGATATGAACATGCACAAATGGCTTCTGACAAATTTCGACGCTTCTTGTCTATATGTCAAGAAACGCAAAGATCTGATAGATGC
ACTCTCCATAACACCAAGTTATCTTCGCAACGAGTTTTCAGAGAGTGGACTCGTAACCGACTATCGGGATTGGCAAATTCCTCTCGGAAGACGC
TTCCGAAGCTTAAAGATTTGGTTTGTCCTCAGAACCTAC

>Bcin09g03030(MLST4), partial sequence [organism=Botrytis cinerea, strain D13_MR_S4]
ACCCTTCAACCCCACCGCAGCTCCTATACCGAAAGCGAGTATCCTACCAATTCTCCTTCCACCTGCGACTTTAAGACCATTGGCTTTCCGCACT
TTCACAAAAAAGCATAGTTTAACATTGACGTCGTCGGCATTACAAGTGTTGGCTACTTTTATTGGAAAGCATTGTGGGACAGGATGGAGGGAAG
AAGGACTGGCAGAGAGAGTCTTGGAGGAGGTCGCCAAGAGTTGGAAGAATAGGAGTGGCGGTGTCATTGTCGAGGGCGAGGGAACGGAATTGAA
GGAGATTCTGAAAGCTTTGGAAGGGAATATGAGTGGTGGAAGGATAGTCATAGGAAGAGAGCTAAGCCGGCAGAATAGTTTAGTACTGGGATCA
TCACAATATGGAGAGGTCAATCATACAAGACTTGGGCTACGGCCAGGGAATATACCCAGAGAGGATAGTCAGTCAAGTTTGGGAATGTCAACGT
TGGAGGTCAATGACGAGGAAGATGAGGATGGCCTGATGGATCCAAGAAGGTGGTTAAAAGTCATTGATGCATTTGAGCAACCTCGACTGGTGTA
CAATGTTGCTAAAAAGCACTTTGATAGGTATGTTTCAATGATAAAATTTCATCTGAATCGACTAACTCAGTACAGAGATACCTCCAAACCTTCA
TTGTTCCCACCTGCGTCTCATAAAACACTCCTCTTCCAAAACCGCTATAATGTTATCCATCAACGTCTCCTTCGCAATGAATCTTTTCAAACGC
CCGCTTTTCAAGGTGGCAAATCTTCCCTTCAACGCAGCACGTCCGCCATTACCACCCAACAACAATCATACAAATTAACGCCGATAGCTAATCT
TCTCGGTCGCAATCGCAGCTCTCATATGCTTCTCGGTCTCCTCAGTATTTCACCCACTGGTACCCTCGCCATCAATGACCTGACGGGCAGTATC
GCTCTCGATCTTACACACGCAGCAGCCATTC

>Bcin11g01310(MLST5), partial sequence [organism=Botrytis cinerea, strain D13_MR_S4]
ACTGACATGGACCTCATGTGGAACCGGCGTAGAATGCGCAACGCTTGAAGTTCCGCTCGAATATGGCGATGCAACGTCAACGGCAAAAGCCAGT
GTTGCGCTTGCTCGTTATCCTGCCACTGTTGCCGCGAGCAAGAAGCTCGGGTCTCTCTTGATAAATCCCGGTGGACCCGGTGCCTCTGGTGTTG
GCTTTGTGCAGTCTGGAGCCGGTGCCGCCGTCTCGACACTGAGTGGTGGATTGTACGATATCATCGGATGGGATCCACGTGGAACCGGTGCTTC
GGCTCCTATTTTGGAATGTTTTGCAAATGCCAGTGCGGAGTATGATTTTAACAACGCGTTTCCATCTGCTCCGAATCTCTGGCTCGGACAATTT
GCGAATGCCAGCGCAAATTCTGCTGTTAGCTCTGCTATCACATCCTTTGACACTTCTGTCGCTGCTCTTGCAAAAGCTTGCGTGGCTCAGAAAT
CTCCCGCTCTTTACACCTCAACAGCAGCATATGTTGCTCGAGACATGGCAGCGATAGTCGATGCATTGGATGGGACCTCTGCAAAACTTAACTA
CTGGGGTTTCTCATATGGAACTATCTTCCTAGCTGAGTTTATCCAAACTTTCCCAGGCCGCGTGGGAAGAGTTCTTGCCGATGGTGTTTTCGAC
GCAAAGGCAAATGCACTCACATACGTTAGCCAACTTCCCAACGATCAACTCAGTGTTCGTGCTTCGTTGAACGATTTTGCAGCTTTCTGCACCA
CCGCCGGTAGTAAAGGTTGCTCTTTTGCCACCGCCCCTACTGGAACCACAGGTACTGTTGCTACCAGACTGGACAACATAATGAAGGATATGTT
CCTCAATCCTATTGTTGCTTCGGGCTTAAGCATCA

>Bcin15g03910(MLST6), partial sequence [organism=Botrytis cinerea, strain D13_MR_S4]
GCCAAAACACAAAATCATCCAACGATGAAGATGATACTCCACTTCCCTTGATTATCTGGCATGGACTCGGCGATAATTACAAAGCGGATGGTCT
TGCGCAAGTTGGAAAACTAGCTGAAGCTATTCATCCTGGGACTTTTGTCTACAATATTCATGTAGATGAGGATGCATCTGCAGATAGGACAGCT
ACCTTCTTTGGAAATCTCACTCGTGAGTACATCCCCTATTTTTCCTTTAAATATCATACTAACTCTCTTACCAAGTTCAAATCGAAAAGGTCTG
CGAAGACCTCGCCTCCCATCCTATTCTCTCTACCGCGCCCGCCGTCGACGCAATTGGATTCTCCCAAGGCGGCCAATTCTTGCGTGGTTACATA
TCCCGCTGCAATGCTCCACCCATCCGCTCTCTCCTGACCTTCGGTTCCCAACACAACGGCATTTCTGCCTTCCAAGCCTGTGGTCCTGCCGATT
TCCTCTGTCGCGGTGCTCAAACCCTTTTGCGATCCAACACCTGGTCAACCTTTGTCCAATCTCGTCTCGTACCCGCTCAATACTTCAGAGATCC
GGAAAACCTAGACTCTTACCTTGAATATTCCAATTTCCTTGCCGACATCAATAATGAGCGCGTTCTCAAGAACCAAACATATAAATCCAACATG
GAAAAATTGGAACGATTCGTAATGTATGTCTTTGAAGACGATACAACTGTCATCCCTAAGGAAAGTGGATGGTGGGCTGAAGTCAACGGCACGG
AAGTTACACCACTGAAAGAAAGAGCCATTTATAAAGAAGATTGGCTAGGTTTAAAGACATTGGATGAGGCCGGAAAATTAGTTTTCGAAACCAT
TCCAGGGGGACATATGACGTTAGGAGAGGAGATGCTAGAGAAGGCTTTCAAAGAGTATTTTGGTCCAGCAGGGAAGAAATTTGGG

>Bcin16g03460(MLST7), partial sequence [organism=Botrytis cinerea, strain D13_MR_S4]
ATGAACTCTTGATTTGAACTCTTCATTAATTGCAAGTGATGAACCCTATTCTGCCCAGAGGATAGTTACAAGACGTATATCATTAGTCGAGAAC
CACTCATGATATATATTGACGGATTTTTGAAAGCGAATGAAAGTAAACATTTGGTTGATGTTAGGTGTGTTATTTATTCTGATGAAATGAACAA

GAGAGACTGATGAGATAGTGAGCCGCTTTATGAACCGTCAACTGTTTCTCACGGACAGGAAGTTACCATTGATCCTTCAGTTCGGAATTCTGAA
GTGGCGGTTTTAGAGAGGGATGAGGTGGTCAGGTGTATTGAGCATAGAGCGAGGGCATTTCAGGGGTGGAGGGGCGAGATGGGATTGAGAAGTT
GAGGACGCAGAGGTATGGGTTGGAGGACATTATGGGATGCATTTGTAAGTTTTGGGGGATTGACGAAGGCTTTTGTCTATTTCTACCAGTACGA
ATCTTGAAAGAAAGATAGCATGAACACGAGGGCTAATAACTAGGAAATAGCGATTGGAGCGGAGGTAAACGTGGCATAGACCGATTTAGTACTT
TCATGGTCTATGTCGACGTATCCTCTGATATCGAAGGTGGAGGAACGGAATTCCCACGTATCGTGGGACCAAAAGGAGGAAGGTGGAGGACTTC
CTGGAAACTACGGAAGCATTGGATCCAAGAACTGGAGAAAATGTAACAGTAGAAGGGGTGACATTCAAACCAATCAAGGGAAATGCCGTATTCT
GGGAAAATACTGACAACAACGGGAGGGCTATGAT

>Bcin12g03020(MLST8), partial sequence [organism=Botrytis cinerea, strain D13_MR_S4]
CGATTGGCTGCGAAGAAAACTGCGCAGCACCGAGTCACCAAAAGCACGAATAAACCATCGGCAAGGGAAATATTCTCTGAACCACAACATGACA
CGAGCGCGGAAGAGTATATCGGGCGAGAAGCTTCATCAAGAGCACCAAAGCGACAACGAGTCGATGATAATTATAACTCTTACGGTGGAAGAAA
TGAAAATACAGCAGCTTATGCTTCCGGGAAACTTCCATCTGGAAGTATAAATGTTGGTGGAGGTAGGAAGACCACTTTTCAAGAAGAACCTCGA
ACGGCATTTGTCGCTGGCAAGTTGCCCCCTGGAAGTATCAACATTGGTGGAAAGAAGGCTACCCAAATCGAGGACGAGGGTAGAGCAGCTTATG
CTTCCGGAAAATTGCCCCCAGGAAGTATAAACTCTGGCGCAAAGAAGGCGATTTCATTCCAAGATGAAACGAGACCGGCTTATGTCTCCGGAAA
GCTTCCACATGGTAGTATCGACGGTATGCGAAACCGTGAAATGGCTGCCGTCCACCGCGAAATTGCTGAGGGTGGGTGGAAACCAGGCCAGGTT
GTCTCTTCTCTATTCACATTCAATCCTACTTCAAAGAAAACTTTCGATGAACCAGAAGAACAAGCGGAACCGGCAAAACCATCCAATGCGCCTT
TGACAGAGGAAATGGCGACTTTCACCAATCTAGGGCTATCGAGAAGGCTTGCAGCCCATCTATCGACTAAACTCGATATGAAAGCCCCGACGGC
CATTCAAAAAGCATCTGTGCAGCAGTTGGTATCGGACGATAGCGATGCTTTCATACAAGCAGAGACTGGATCTGGAAAAACTTTGGCATATCTA
CTACCTATAGTCGAGCGAATATTAGCATTGAGTGAGAATGGCGTACAAA

>Bcin02g07770(MLST9), partial sequence [organism=Botrytis cinerea, strain D13_MR_S4]
ACAGCTTTCCCTTTCGGTCTTGGCATCTACAGTCATTGCCATCCCTACACCATCACAACTTGAGTCTCGGGCCGTTATCGATTCCGATGCCGTT
GTAGGATTTGCCGAAACTGTTCCCAGTGGGACCGTAGGAACAGTTTATGAGGCATATAAACCATTCCTTAAAGTCGTAAATGGATGCGTACCAT
TCCCTGCCGTCGATGCATCGGGTAACACAGGGTATGTCCTTATATCTTTCTCTTCCACACGATTGCTATTGAGTCTCTAACATATTTTAGTGGT
GGTTTGTCACCAACTGGCAGTAGCAATGGTGGTTGCAGCAGCAGTACCGGTCAAGTATATGTTCGAGGAGGACAAAGCGGATCAAACTACGCCA
TCATGTACTCCTGGTAAGTTCTCTCTAAACTTCTCCTTATAGATCCAACCTAACAAAATCTTAGGTACATGCCAAAGGACGAGCCCTCAACCGG
TATTGGTCACCGTCACGATTGGGAAGGTGTAATTGTCTGGCTCTCCAGCGCCACCGCCACAACTGCCGACAACATCTTAGCCGTTTGTCCTTCC
GCCCACGGAGGCTGGGATTGTTCCACGGATGGCTATTCCCTTTCTGGTACCAGCCCTCTTATCAAGTACGAAAGTATCTGGCCCGTCGATCACT
CAATGGGTCTTACTAGTACTGTTGGTGGAAAACAACCTATGATTGCTTGGGAGTCTTTACCAACTGCTGCTCAAACTGCTCTTGAGAACACCGA
TTTCGGTGCTGCGAATGTTCCATTCATTCCGGCTGTTTTCACAGACAATCT

>Bcin04g02090(MLST10), partial sequence [organism=Botrytis cinerea, strain D13_MR_S4]
CGGAGGATGATATGGCAAAGTCTATGATTACCAAAGCATTTGTAGGCATGAGTAGTAAATTGTCGAATATGACTATGAATGATGTTTACAAGCC
CTACATCCATGTAAGAAATGTAGAATAAAGAGATCAGTAACTGGAACTAATATCGTTTGCAGGCTTTCAAGTTACTTACGCAGTTCAACCCAAT
CACTACAGCTATTGCCGAATCCCCACTATTTCAAATGGCTGTCTCAGCAAATACCATCGAAAAGTACACACTGCTAGGCCCTTTCTTCAGAATA
TCTCCTCTGCAACAGGAAGTTACCAGGGAATACTTCAGTGCGCCAAAGACGATAGATAGGCGACACATTGCCACATCTCAAGATGCGTTACGAT
TGACCTTACAAACCCATCAAAAAGATTTACTTGATATCATCAACCACTTTGTTCGAGCAAGTCCAATCGCAAAAGCAAAACCCTGGATTGGTTC
GCCTACATTGTGAATCAAAATCACAAGCGTCGAGCACTTCAGGTAGACCCGAAAGAAGTGTCTTCTGATGGCTTTATGCACAATGTCACTGTCG
TTCTAGATGGTCTTTGTGAGCCATTCATGGATACCACATTCTCAAAATTTCGAAGATTGATATTGATTATCTAAGACGTGCGCCTCGTGTAGAT
ATCAAGGACGAGACCAAGTTGAACGCTGATGAGAAGGCTTCTGAGAAGTATTATGAGGACACTGTTCCTGGCACTTCTAATTTCATCTCTGAGG
TATTCTTTCTGACATTGGCTGCTCATCATTATGGTAGTGAAGCTCTTAATGCCACGCATAAGAGTCTGGAGAAAGACATCAAATATATTCAAAA
GCAATTGACTGCCGTTGAAGCA

>Bcin01g07220(MLST1), partial sequence [organism=Botrytis cinerea, strain D13_MR_S14]
ATGGCATATCTTGTTTCTTTTCGCATTATGCTTTGGAACGTGCTGGACAAGCTTGGGTTAGTGCTCTGCGTGTAGAAGCACTAAAGAAGATTCT
CGCACAACCGAAGTCATGGTTTGAGGAATCCAGGAATTCACCTGGCCGGTTGAACGAAGTTTTGGATAGGAACTCTGAGGAAATGCGTAATCTC
GTTGGCCGCTTTGCTGGTATTGTATTCACAGCATTTTTTATGCTATTGATATCAATCATTTGGGCTTTTGTGAATACATGGAAACTGACATTAG
TCTCGATGGCAACTGGGCCAGTTATATACGCTGTCACCAAAACATTCAATCGCGTGAGTGGAAAATGGGAAAACAAGTGCAACTACGCCTCTGA
AATGACCACTGGCATATTTTCAGAGACTTTCTCCAACATCAAAGTGGTTCGGGCTTTTACTCTGGAAACTTACTTTGAGACAAAACACACCAAA
GCTACAGGAGAACTTTATAAAGTTGGACTAATACGAGCAAACTACTCGGGATTGCTGTGGGGATTGACAGATGCGATGTCATTCTTCATCACTG
CAACTATCTTTTATTATGCCACGGTTCTCATTACCAAGAGAGAGATCAGTATCGCGACTGCACTACAGACTGTCAATCTTCTATTATTTGGTAT
TTCTAATAGTACGAATATGCTGGCCATGATACCACAAATCAACTCTTCTCGCGTTACAGCTACGCATATGCTTGCATTAGCCAATCTCGATTCA
TCTTCCTCCCACGAAAATAAAGGAACCGAACGGCTTTCGACAATCTTTCCAATCAAATTCAACCGTCTTTCATTCACATACCCTACTCGTCCTG
AAAAACGAACGATATCATCCTTTTCTCTTTCCCTGATTCCTAACTCAACAACTGCACTTGTCGGACCCTCCGGCTCCGGAAAATCTACAATAGT
TGCTCTGCTCATTGGTCTCTATCCGCCAGATACTTCAACACCTCCACCGTTGACATTCAATCGCGTCTCCATAAGTAACTGTCACATTCCGTCT
CTCCGGGCTTCTCTCTCACTCGTTCCACAAATACCGATTCTATTTCCAGCTACCATTCTCCATAACATCATTTATGGTCTCCCAGAATCTTCTC
CTTGTGCTAGTCTTCCATCAGCTATTGCATCAGCAAAAGATGCTGGGATCCATGAATTTATCACATCGCTTCCACAAGGTTATGATACTAT

>Bcin05g07690(MLST2), partial sequence [organism=Botrytis cinerea, strain D13_MR_S14]
ACCACTATCACCAAGTCCTTTTCTGCGCTTTCCTCACCAAAAACGAACGAGAACATCATCAATAAATACTTTGCTGAAGTAATTGGGTTTTATT
TTGGCCAGGATGCCTTCTCTCTACGACAGCAAGCCTTCGATGATATGTTGTGGGTAGTTCTTGGCTGGCTGGATACTGTCAAATTCATTGATTT
ACATTCTGAATTGCACTATTCAAACGACTCTCAGCCAGAATGGTACGGACAACAATATAAACCTGCATTTGCACATCGAGCGCGACTATTTTGG
GAATTGGCTTCACAAGGATGGGATACTACTCTCTGTGGTGGTGGGATGATATGGTCACCATACCTTACTCCATACAAGAACGCAATTACCAATG
AACTCTATATCGCAGCTTCGATATCGATGTACCTATATTTCCCCGGAGATGACAATCAATCCCCATTTATGATTTCCAACCCTTCATATCCACC
TCACGATCCGAAATATCTACAGGCAGCTGTTGATGCTTACAAATGGCTGAATGGTTCCAACATGACGGATTCACAAGGATTATATGTCGACGGG
TACCATATCTCGAATCTTTCTGGCGGTGAAAACACCCATTGCGATTCTAGAAATGAGATGGTATATACCTACAATCAAGGTGTTTTGCTTACTG
GACAACGTGGTTTGTATGACGCAACCGCCGCACGATCATACCTTGTAGATGGCCACAAACTCATCGCGAATGTTATTAATGCCACAGGCTATGA
CCTGAAACACAATGTTGTCATCTCACCGCCACCCAAAGATGGTTCCGCATTGGCAAAGTGGTTTGGCCTGGGTAGGAATGGAATACTGGAAGAA
GGATGCGATTCAAGTGCTTCGTGTTCTCAAAATGGACAAACTTTCAAAGGCATATTCTTTCATCACTTGATTGCGTTCTGTAGTGATTTGCCAG
GGGGCCTATTGCAGGGACGAAGGAAAGCTTAGAACTCGACAGAGTGTGGCATTCTGACAAATGCTCACAGTATACAAAAT

>Bcin06g01710(MLST3), partial sequence [organism=Botrytis cinerea, strain D13_MR_S14]
TCTGACACATTGGTGAGTCTGACTTTTGTGTTTGAGCGTTAAGATAGACACTGATGTACCAAGGCAATCACCTAATTTCATGGCGTTCTTCCCT
GCATCATCTACCTACCCTGGAATGCTGGGAGAATTATACTCAGCAGCTTTCACAGCACCTGCTTTCAATTGGTCTGTTCCCCTGCCGTGACAGA
ATTGGAGACGGTTGTAATGGATTGGCTGGCCAAGCTTCTCAATCTCCCAGACTGTTATTTGTCTTCGACTCATGGTGGTGGTGTTATCCAAGGA
TCAGCCTCGGAAGCTATCGTTACCGTTATGGTTGCTGCCCGCGATAAATATCTTCGTGAAACTACTGAAGGTCTGTCGGGCATTGAACTCGAGG
ATGCGATTGCATATAAGAGGAGTAAGCTAGTTGCACTAGGAAGCGAAATGGCACACAGCTCCACGCAGAAAGCAGCGCAGATAGCTGGCGTTAG
ATTCCGATCGATTCCAGTACTCGCATCCAATGATTTCGCCATGACGGGTGATGATTTAGAGAAGGTATTGGAAGAATGCAAATCTCAAGGATTG
GAACCCTTCTATCTAACTTCGACTTTGGGAACAACATCTACATGCGCAGTTGACGACTTCGCATCTATTGCAACAGTACTTTCAAAATATGCAC
CTCCAGATGTTGCAGGCGAGATCTGGGTTCACGTCGATGCTGCTTATGCAGGTGCAGCTTTGGTTTGCCCTGAATACCATCATCTAACATCGTC
CTTCCAGCATTTCCATTCCTTCGATATGAACATGCACAAATGGCTTCTGACAAATTTCGACGCTTCTTGTCTATATGTCAAGAAACGCAAAGAT
CTGATAGATGCACTCTCCATAACACCAAGTTATCTTCGCAACGAGTTTTCAGAGAGTGGACTCGTAACCGACTATCGGGATTGGCAAATTCCTC
TCGGAAGACGCTTCCGAAGCTTAAAGATTTGGTCTGTCCTCAGAACCTAC

>Bcin09g03030(MLST4), partial sequence [organism=Botrytis cinerea, strain D13_MR_S14]
GACCCTTCAACCCCACCGCAGCTCCTATACCGAAAGCGAGTATCCTAACCAATTCTCCTTCCACCTGCGACTTTAAGACCATTGGCTTTCCGCA
CTTTCACAAAAAGCATAGTTTAACATTGACGTCGTCGGCATTACAAGTGTTGGCTACTTTTATTGGAAAGCATTGTGGGACAGGATGGAGGGAA
GAAGGACTGGCAGAGAGAGTCTTGGAGGAGGTCGCCAAGAGTTGGAAGAATAGGAGTGGCGGTGTCATTGTCGAGGGCGAGGGAACGGAATTGA
AGGAGATTCTGAAAGCTTTGGAAGGGAATATGAGTGGTGGAAGGATAGTCATAGGAAGAGAGCTAAGCCGGCAGAATAGTTTAGTACTGGGATC
ATCACAATATGGAGAGGTCAATCATACAAGACTTGGGCTACGGCCAGGGAATATACCCAGAGAGGATAGTCAGTCAAGTTTGGGAATGTCAACG
TTGGAGGTCAATGACGAGGAAGATGAGGATGGCCTGATGGATCCAAGGAGGTGGTTAAAAGTCATTGATGCATTTGAGCAACCTCGACTGGTGT
ACAATGTTGCTAAAAAGCACTTTGATAGGTATGTTTCAATGATAAAATTTCATCTGAATCGACTAACTCAGTACAGAGATACCTCCAAACCTTC
ATTGTTCCCACCTGCGTCTCATAAAACACTCCTCTTCCAAAACCGCTATAATGTTATCCATCAACGTCTCCTTCGCAATGAATCTTTTCAAACG
CCCGCTTTTCAAGGTGGCAAATCTTCCCTTCAACGCAGCACGTCCGCCATTACCACCAACAACAATCATACAAATTAACGCCGATAGCTAATCT
TCTCGGTCGCAATCGCAGCTCTCATATGCTTCTCGGTCTCCTCAGTATTTCACCCACTGGTACCCTCGCCATCAATGACCTGACGGGCAGTATC
GCTCTCGATCTTACACACGCAGCAGCCATT

>Bcin11g01310(MLST5), partial sequence [organism=Botrytis cinerea, strain D13_MR_S14]
ACTGACATGGACCTCATGTGGAACCGGCGTAGAATGCGCAACGCTTGAAGTTCCGCTCGAATATGGCGATGCAACGTCAACGGCAAAAGCCAGT
GTTGCGCTTGCTCGTTATCCTGCCACTGTTGCCGCGAGCAAGAAGCTCGGGTCTCTCTTGATAAATCCCGGTGGACCCGGTGCCTCTGGTGTTG
GCTTTGTGCAGTCTGGAGCCGGTGCCGCCGTCTCGACACTGAGTGGTGGATTGTACGATATCATCGGATGGGATCCACGTGGAACCGGTGCTTC
GGCTCCTATTTTGGAATGTTTTGCAAATGCCAGTGCGGAGTATGATTTTAACAACGCGTTTCCATCTGCTCCGAATCTCTGGCTCGGACAATTT
GCGAATGCCAGCGCAAATTCTGCTGTTAGCTCTGCTATCACATCCTTTGACACTTCTGTCGCTGCTCTTGCAAAAGCTTGCGTGGCTCAGAAAT
CTCCCGCTCTTTACACCTCAACAGCAGCATATGTTGCTCGAGACATGGCAGCGATAGTCGATGCATTGGATGGGACCTCTGCAAAACTTAACTA
CTGGGGTTTCTCATATGGAACTATCTTCCTAGCTGAGTTTATCCAAACTTTCCCAGGCCGCGTGGGAAGAGTTCTTGCCGATGGTGTTTTCGAC
GCAAAGGCAAATGCACTCACATACGTTAGCCAACTTCCCAACGATCAACTCAGTGTTCGTGCTTCGTTGAACGATTTTGCAGCTTTCTGCACCA
CCGCCGGTAGTAAAGGTTGCTCTTTTGCCACCGCCCCTACTGGAACCACAGGTACTGTTGCTACCAGACTGGACAACATAATGAAGGATATGTT
CCTCAATCCTATTGTTGCTTCGGGCTTAAGCATCA

>Bcin15g03910(MLST6), partial sequence [organism=Botrytis cinerea, strain D13_MR_S14]
GCCAAAACACACAAAATCATCCAACGATGAAGATGATACTCCACTTCCCTTGATTATCTGGCATGGACTCGGCGATAATTACAAAGCGGATGGTCT
TGCGCAAGTTGGAAAACTAGCTGAAGCTATTCATCCTGGGACTTTTGTCTACAATATTCATGTAGATGAGGATGCATCTGCAGATAGGACAGCT
ACCTTCTTTGGAAATCTCACTCGTGAGTACATCCCCTATTTTTCCTTTAAATATCATACTAACTCTCTTACCAAGTTCAAATCGAAAAGGTCTG
CGAAGACCTCGCCTCCCATCCTATTCTCTCTACCGCGCCCGCCGTCGACGCAATTGGATTCTCCCAAGGCGGCCAATTCTTGCGTGGTTACATA

TCCCGCTGCAATGCTCCACCCATCCGCTCTCTCTGACCTTCGGTTCCCAACACAACGGCATTTCTGCCTTCCAAGCCTGTGGTCCTGCCGATTT
CCTCTGTCGCGGTGCTCAAACCCTTTTGCGATCCAACACCTGGTCAACCTTTGTCCAATCTCGTCTCGTACCCGCTCAATACTTCAGAGATCCG
GAAAACCTAGACTCTTACCTTGAATATTCCAATTTCCTTGCCGACATCAATAATGAGCGCGTTCTCAAGAACCAAACATATAAATCCAACATGG
AAAAATTGGAACGATTCGTAATGTATGTCTTTGAAGACGATACAACTGTCATCCCTAAGGAAAGTGGATGGTGGGCTGAAGTCAACGGCACGGA
AGTTACACCACTGAAAGAAAGAGCCATTTATAAAGAAGATTGGCTAGGTTTAAAGACATTGGATGAGGCCGGAAAATTAGTTTTCGAAACCATT
CCAGGGGGACATATGACGTTAGGAGAGGAGATGCTAGAGAAGGCTTTCAAAGAGTATTTTGGTCCAGCAGGGAAGAAATTTGGG

>Bcin16g03460(MLST7), partial sequence [organism=Botrytis cinerea, strain D13_MR_S14]
ATGAACCTCTTGATTTGAACTCTTCATTAATTGCAAGTGATGAACCCCTATTCTGCCCAGAGGATAGTTACAAGACGTATATCATTAGTCGAGG
ACCACTCATGATATATATTGACGGATTTTTGAAAGCGAATGAAAGTAAACATTTGGTTGATGTTAGGTGTGTTATTTATTCTGATGAAATGAAC
AAGAGAGACTGATGAGATAGTGAGCCGCTTTATGAACCGTCAACTGTTTCTCACGGACAGGAAGTTACCATTGATCCTTCAGTTCGGAATTCTG
AAGTGGCGGTTTTAGAGAGGGATGAGGTGGTCAGGTGTATTGAGCATAGAGCGAGGGCATTTCAGGGGTGGAGGGGCGAGATGGGGATTGAGAA
GTTGAGGACGCAGAGGTATGGGGTTGGAGGACATTATGGGATGCATTTGTAAGTTTTGGGGGATTGACGAAGGCTTTTGTCTATTTTCTACCAG
TACGGATCTTGAAAGAAAGATAGCATGAACACGAGGGCTAATAACTAGGAAATAGCGATTGGAGCGGAGGTAAACGTGGCATAGACCGATTTAG
TACTTTCATGGTCTATGTCGACGTATCCTCTGAATTATCGAAGAGGTGGAGGAACGGAATTCCCACGTATCGTGGGACCAAAAGGAGGAAGGTG
GGAGGACTTCCTGGAAACTACGGAAGCATTGGATCCAAGAACTGGAGAAAATGTAACAGTAGAAGGGGTGACATTCAAACCAATCAAGGGAAAT
GCCGTATTCTGGGAAAATACTGACAACAACGGGAGGGGCTATGAT

>Bcin12g03020(MLST8), partial sequence [organism=Botrytis cinerea, strain D13_MR_S14]
CGATTGGCTGCGAAGAAAACTGCGCAGCACCGAGTCACCAAAAGCACGAATAAACCATCGGCAAGGGAAATATTCTCTGAACCACAACATGACA
CGAGCGCGGAAGAGTATATCGGGCGAGAAGCTTCATCAAGAGCACCAAAGCGACAACGAGTCGATGATAATTATAACTCTTACGGTGGAAGAAA
TGAAAATACAGCAGCTTATGCTTCCGGGAAACTTCCATCTGGAAGTATAAATGTTGGTGGAGGTAGGAAGACCACTTTTCAAGAAGAACCTCGA
ACGGCATTTGTCGCTGGCAAGTTGCCCCCTGGAAGTATCAACATTGGTGGAAAGAAGGCTACCCAAATCGAGGACGAGGGTAGAGCAGCTTATG
CTTCCGGAAAATTGCCCCCAGGAAGTATAAACTCTGGCGCAAAGAAGGCGATTTCATTCCAAGATGAAGCGAGACCGGCTTATGTCTCCGGAAA
GCTTCCACATGGTAGTATCGACGGTATGCGAAACCGTGAAATGGCTGCCGTCCACCGCGAAATTGCTGAGGGTGGGAGGAAACCAGGCCAGGTT
GTCTCTTCTCTATTCACATTCAATCCTACTTCAAAGAAAACTTTCGATGAACCAGAAGAACAAGCGGAACCGGCAAAACCATCCAATGCGCCTT
TGACAGAGGAAATGGCGACTTTCACCAATCTAGGGCTATCGAGAAGGCTTGCAGCCCATCTATCGACTAAACTCGATATGAAAGCCCCGACGGC
CATTCAAAAAGCATCTGTGCAGCAGTTGGTATCGGACGATAGCGATGCTTTCATACAAGCAGAGACTGGATCTGGAAAAACTTTGGCATATCTA
CTACCTATAGTCGAGCGAATATTAGCATTGAGTGAGAATGGCGTACAAA

>Bcin02g07770(MLST9), partial sequence [organism=Botrytis cinerea, strain D13_MR_S14]
CAGCTTTCCCTTTCGGTCTTGGCATCTACAGTCATTGCCATCCCTACACCATCACAACTTGAGTCTCGGGCCGTTATCGATTCCGATGCCGTTG
TAGGATTTGCCGAAACTGTTCCCAGTGGGACCGTAGGAACAGTTTATGAGGCATATAAACCATTCCTTAAAGTCGTAAATGGATGCGTACCATT
CCCTGCCGTCGATGCATCGGGTAACACAGGGTATGTCCTTATATCTTTCTCTTCCACACGATTGCTATTGAGTCTCTAACATATTTTAGTGGTG
GTTTGTCACCAACTGGCAGTAGCAATGGTGGTTGCAGCAGCAGTACCGGTCAAGTATATGTTCGAGGAGGACAAAGCGGATCAAACTACGCCAT
CATGTACTCCTGGTAAGTTCTCTCTAAACTTCTCCTTATAGATCCAACCTAACAAAATCTTAGGTACATGCCAAAGGACGAGCCCTCAACCGGT
ATTGGTCACCGTCACGATTGGGAAGGTGTAATTGTCTGGCTCTCCAGCGCCACCGCCACAACTGCCGACAACATCTTAGCCGTTTGTCCTTCCG
CCCACGGAGGCTGGGATTGTTCCACGGATGGCTATTCCCTTTCTGGTACCAGCCCTCTTATCAAGTACGAAAGTATCTGGCCCGTCGATCACTC
AATGGGTCTTACTAGTACTGTTGGTGGAAAACAACCTATGATTGCTTGGGAGTCTTTACCAACTGCTGCTCAAACTGCTCTTGAGAACACCGAT
TTCGGTGCTGCGAATGTTCCATTCATTCCGGCTGTTTTCACAGACAATCT

>Bcin04g02090(MLST10), partial sequence [organism=Botrytis cinerea, strain D13_MR_S14]
CGGAGGATGATATGGCAAAGTCTATGATTACCAAAGCATTTGTAGGCATGAGTAGTAAATTGTCGAATATGACTATGAATGATGTTTACAAGCC
CTACATCCATGTAAGAAATGTAGAATAAAGAGATCAGTAACTGGAACTAATATCGTTTGCAGGCTTTCAAGTTACTTACGCAGTTCAACCCAAT
CACTACAGCTATTGCCGAATCCCCACTATTTCAAATGGCTGTCTCAGCAGATACCATCGAAAAGTACACACTGCTAGGCCCTTTCTTCAGAATA
TCTCCTCTGCAACAGGAAGTTACCAGGGAATACTTCAGTGGCGCCAAAGACGATAGATAGGCGACACATTGCCACATCTCAAGATGCGTTACGA
TTGACCTTACAAACCCATCAAAAAGATTTACTTGATATCATCAACCACTTTGTTCGAGCAAGTCCAATCGCAAAAAGCAAAACCCTGGATTGGT
TCGCCTACATTGTGAATCAAAATCACAAGCGTCGAGCACTTCAGGTAGACCCGAAAGAAGTGTCTTCTGATGGCTTTATGCACAATGTCACTGT
CGTTCTAGATGGTCTTTGTGAGCCATTCATGGATACCACATTCTCAAAAAATTTCGAAGATTGATATTGATTATCTAAGACGTGCGCCTCGTGTA
GATATCAAGGACGAGACCAAGTTGAACGCTGAATGAGAAGGCTTCTGAGAAGTATTATGAGGACACTGTTCCTGGCACTTCTAATTTTCATCTC
TGAGGTATTCTTTCTGACATTGGCTGCTCATCATTATGGTAGTGAAGCTCTTAATGCCACGCATAAGAGTCTGGAGAAAGACATCAAATATATT
CAAAAGCAATTGACTGCCGTTGAAGCA

>Bcin01g07220(MLST1), partial sequence [organism=Botrytis cinerea, strain D14_Heid15]
ATGGCATATCTTGTTTCTTTTCGCATTATGCTTTGGAACGTGCTGGACAAGCTTGGGTTAGTGCTCTGCGTGTAGAAGCACTAAAGAAGATTCT
CGCACAACCGAAGTCATGGTTTGAGGAATCCAGGAATTCACCTGGCCGGTTGAACGAAGTTTTGGATAGGAACTCTGAGGAAATGCGTAATCTC
GTTGGCCGCTTTGCTGGTATTGTATTCACAGCATTTTTTATGCTATTGATATCGATCATTTGGGCTTTCGTGAATACATGGAAACTGACATTAG

TCTCAATGGCAACTGGGCCAGTTATATATGCTGTCACCAAAACGCTCAATCGCGTGAGTGGAAAATGGGAAAACAAGTGCAACTACGCCTCTGA
AATGACCACTGGCATATTTTCAGAGACTTTCTCCAACATCAAAGTGGTTCGGGCTTTTACTCTGGAAACTTACTTTGAGACAAAACACACCAAA
GCTACAGAAGAACTTTATAAAGTTGGATTAATACGAGCAAACTACTCGGGATTGCTGTGGGGATTGACAGATGCGATGTCATTCTTCATCACTG
CAACTATCTTTTATTATGCCACGGTTCTCATTACCAAGAGAGAGATCAGTATCGCGACTGCACTACAGACTGTCAATCTTCTATTATTTGGTAT
TTCTAATAGTACGAATATGCTGGCCATGATACCACAAATCAACTCTTCTCGCGTTACAGCTACGCATATGCTTGCATTAGCCAATCTCGATTCA
TCTTCCTCCCACGAAAATAAAGGAACCGAACGGCTTTCGACAATCTTTCCAATCAAATTCAACCGTCTCTCATTCACATATCCTACTCGTCCTG
AAAAACGAACGATATCATCCTTTTCTCTTTCCCTGATTCCTAACTCAACAACTGCACTTGTCGGACCCTCCGGCTCCGGAAAATCTACAATAGC
TGCTCTGCTCATTGGTCTCTATCCTCCAGATACTTCAACACCTCCACCGTTGACATTCAATCGCGTCTCCATAAGTAACTGTCACATTCCGTCT
CTCCGGGCTTCTCTCTCACTCGTCCACAAATACCGATTCTATTTCCAGCTACCATTCTCCATAACATCATTTATGGTCTCCCAGAATCTTCTCC
TTGTGCTAGTCTTCCATCTGCTATTGCATCAGCAAAAGATGCTGGGATCCATGAATTTATCACATCGCTTCCACAAGGTTATGATACTAT

>Bcin05g07690(MLST2), partial sequence [organism=Botrytis cinerea, strain D14_Heid15]
ACCACTATCACCAAGTCCTTTTCTGCACTTTCCTCATCAAAAACGAACGAGAACATCATCAATAAATACTTTGCTGAAGTAATTGGGTTTATTT
TGGCCAGGATGCCTTCTCTCTACGACAGCAAGCCTTCGATGATATGTTGTGGGTAGTTCTTGGCTGGCTGGATACTGTCAAATTCATTGATTTA
CATTCTGAATTACACTATTCAAACGACTCTCAGCCAGAATGGTACGGACAACAATATAAACCTGCATTTGCACATCGAGCGCGACTATTTTGGG
AATTGGCTTCACAAGGATGGGATACTACTCTCTGTGGTGGTGGGATGATATGGTCACCATACCTCACTCCATACAAGAACGCAATTACCAATGA
ACTCTATATCGCAGCTTCGATATCAATGTACCTCTATTTCCCCGGAGATGACAATCAATCCCCATTTATGCTTTCCAACCCTTCATATCCACCT
CACGATCCGAAATATCTACAGGCAGCTGTTGATGCTTACAAATGGCTGAATGGTTCCAACATGACGGATTTACAAGGATTATACGTCGACGGGT
ACCATATCTCGAATCTTTCTGGCGGTGAAAACACCCATTGCGATTCTAGAAATGAGATGGTATATACCTACAATCAAGGTGTTTTGCTTACTGG
ACAACGTGGTTTGTATGACGCAGCCGCCGCACGATCATACCTTGTAGATGGCCACAAACTCATCGCGAATGTTATTAATGCCACAGCTATGACC
TGAAACACAATGTTGTCATCTCACCGCCACCCAAAGATGGTTCCGCATTGGCAAAGTGGTTTGGCCTGGGTAGGAATGGAATACTGGAAGAAGG
ATGCGATTCAAGTGCTTCGTGTTCTCAAAATGGACAAACTTTCAAAGGCATATTCTTTCACCACTTGATTGCGTTCTGTAGTAATTTGCCAGGG
GAGCCTATTGCAGGGACGAAGGAAGGCCTAGAACTCGACAGAGTGTGGCATTCTGACAAATGCTCACAGTATACAAAAT

>Bcin06g01710(MLST3), partial sequence [organism=Botrytis cinerea, strain D14_Heid15]
GTGAGTCTGACTTTTGTATTTGAGCGTTAAGATAGACTCTGATATACCAAGGCAATCACCTAATTTCATGGCGTTCTTCCCTGCATCATCTACC
TACCCTGGAATGCTGGGAGAATTATACTCAGCAGCTTTCACAGCTCCTGCTTTCAATTGGATCTGTTCCCCTGCTGTGACAGAGTTGGAGACGG
TTGTAATGGATTGGTTGGCCAAGCTTCTCAATCTCCCAGACTGTTATTTGTCGTCGACTCATGGTGGTGGTGTTATCCAAGGATCAGCCTCGGA
AGCTATCGTTACCGTTATGGTTGCTGCCCGCGATAAGTATCTTCGTGAAACCACTGAAGGTCTGTCGGGAATTGAACTCGAGGATGCGATTGCA
TATAAGAGGAGTAAGCTAGTTGCACTAGGAAGCGAAATGGCACACAGCTCCACGCAGAAAGCAGCGCAGATAGCTGGCGTTAGATTCCGATCGA
TTCCAGTACTCGCATCCAATGATTTCGCCATGACGGGTGATGATTTAGAGAAGGTATTGAAAGAATGCAAATCTCAAGGATTGGAACCCTTCTA
TCTAACTTCGACGTTGGGAACAACATCTACATGCGCAGTTGACGACTTCGCATCTATTGCAACAGTACTTTCAAAATATGCACCTCCAGATGTT
GCAGGCGAGATCTGGGTTCACGTCGATGCTGCTTATGCAGGTGCAGCTTTGGTTTGCCCTGAATACCATCATCTAACATCGTCCTTCCAGCATT
TCCATTCCTTTGATATGAACATGCACAAATGGCTTCTGACAAATTTCGACGCTTCTTGCCTATATGTCAAGAAACGCAAAGATCTGATCGATGC
ACTCTCCATAACACCAAGTTATCTTCGCAACGAGTTTTCAGAGAGTGGACTCGTAACCGACTATCGGGACTGGCAAATTCCTCTCGGAAGACGC
TTCCGAAGCTTAAAGATTTGGTTTGTCCCAGAACCTAC

>Bcin09g03030(MLST4), partial sequence [organism=Botrytis cinerea, strain D14_Heid15]
CGACCCTTCAACCCCACCGCAGCTCCTATACCGAAAGCGAGTATCCTACCAATTCTCCTTCCACCTGCGACTTTAAGACCATTGGCTTTTCGCA
CTTTCACAAAAAAGCATAGTTTAACATTGACGTCGTCGGCATTACAAGTGTTGGCTACTTTTATTGGAAAGCATTGTGGGACAGGATGGAGGGA
GGAAGGACTGGCAGAGAGAGTCTTGGAGGAGGTCGCCAAGAGTTGGAAGAATAGGAGTGGCGGTGTCATTGTCGAGGGCGAGGGAACGGAATTG
AAGGAGATTCTGAAAGCTTTGGAAGGGAATATGAGTGGTGGAAGGATAGTCATAGGAAGAGAGCTAAGCCGGCAGAATAGTTTAGTACTGGGAT
CATCACAATATGGAGAGGTCAATCATACAAGACTTGGGCTACGGCCAGGGAATATACCCAGAGAGGATAGTCAGTCAAGTTTGGGAATGTCAAC
GTTGGAGGTCAATGACGAGGAAGATGAGGATGGCCTGATGGATCCAAGAAGGTGGTTAAAAGTCGTTGATGCATTTGAGCAACCTCGACTGGTG
TACAATGTTGCTAAAAAGCACTTTGATAGGTATGTTTCAATGATAAAATTTTATCTGAATCGGCTAACTCAGTACAGAGATACCTCCAAACCTT
CATTGTTCCCACCTGCGTCTCATAAAACACTCCTCTTCCAAAACCGCTATAATGTTATCCATCAACGTCTCCTTCGCAATGAATCTTTTCAAAC
GCCCGCTTTTCAAGGTGGCAAATCTTCCCTTCAACGCAGCACGTCCGCCATTACCACCCAACAACAATCATACAAATTAACGCCGATAGCTAAT
CTTCTCGGTCGCAATCGCAGCTCTCATATGCTTCTCGGTCTCCTCAGTATTTCACCCACTGGTACCCTCGCCATCAATGACCTGACGGGCAGTA
TCGCTCTTGATCTTACACACGCAGCAGCCATTC

>Bcin11g01310(MLST5), partial sequence [organism=Botrytis cinerea, strain D14_Heid15]
ACTGACATGGACCTCATGTGGAACCGGCGTAGAATGCGCAACGCTTGAAGTTCCGCTCGAATATGGCGATGCAACGTCAACAGCAAAAGCCAGT
GTTGCGCTTGCTCGTTATCCTGCCACTGTTGCCGCGAGCAAGAAGCTCGGGTCTCTCTTGATAAATCCCGGTGGACCCGGTGCCTCTGGTGTTG
GCTTTGTGCAGTCTGGAGCTGGTGCCGCCGTCTCGACACTGAGTGGTGGATTATACGATATCATCGGATGGGATCCACGTGGAACCGGTGCTTC
GGCTCCTATTTTGGAATGTTTTGCAAATGCCAGTGCGGAGTATGATTTTAACAACGCGTTTCCATCTGCTCCGAATCTCTGGCTCGGACAATTT
GCGAATGCCAGCGCAAATTCTGCTGTTAGCTCTGCTATCACATCCTTTGACACTTCTGTCGCTGCTCTTGCAAAAGCTTGCGTGGCTCAGAAAT
CTCCCGCTCTTTACACCTCAACAGCAGCATATGTTGCTCGAGACATGGCAGCGATAGTCGATGCATTGGATGGGACCTCTGCAAAACTTAACTA

CTGGGGTTTCTCATATGGAACTATCTTCCTAGCTGAGTTTATCCAAACTTTCCCAGGCCGCGTGGGAAGAGTTCTTGCCGATGGTGTTTTCGAC
GCAAAGGCAAATGCACTCACATACGTTAGCCAACTTCCCAACGATCAACTCAGTGTTCGTGCTTCGTTGAACGATTTTGCAGCTTTCTGCGCCA
CCGCCGGTAGTAAAGGTTGCTCTTTTGCCACCGCCCCTACTGGAACCTCAGGTACTGTTGCTACCAGACTGGACAACATAATGAAGGATATGTT
CCTCAATCCTATTGTTGCTTCGGGCTTAAGCATCAG

>Bcin15g03910(MLST6), partial sequence [organism=Botrytis cinerea, strain D14_Heid15]
CGCCAAAACACAAAATCATCCAACGATGAAGATGATACTCCACTTCCCTTGATTATCTGGCATGGACTCGGCGATAATTACAAAGCGGATGGTC
TTGCGCAAGTTGGAAAACTAGCTGAAGCTATTCATCCTGGGACTTTTGTCTATAATATTCATGTAGATGAGGATGCATCTGCAGATAGGACAGC
TACCTTCTTTGGAAATCTCACTCGTGAGTACATCCCCTATTTTTCCTTTAAATACCATACTAACTCTCTTACCAAGTTCAAATCGAAAAGGTCT
GCGAAGACCTCGCCTCCCATCCTATTCTCTCTACCGCGCCCGCCGTCGACGCAATTGGATTCTCCCAAGGCGGCCAATTCTTGCGCGGTTACAT
ATCCCGCTGCAATGCTCCACCCATCCGCTCTCTCCTGACCTTCGGTTCCCAACACAACGGCATTTCTGCCTTCCAAGCCTGTGGTCCTGCCGAT
TTCCTCTGTCGCGGTGCTCAAACCCTTTTGCGATCCAACACCTGGTCAACCTTTGTCCAATCTCGTCTCGTACCTGCTCAATACTTCAGAGATC
CGGAAAACCTAGACTCTTACCTTGAATATTCCAATTTCCTTGCCGACATCAATAATGAGCGCGTTCTCAAGAACCAAACATATAAATCCAACAT
GGAAAAATTGGAACGATTCGTAATGTATGTCTTTGAAGACGATACAACTGTCATCCCTAAGGAAAGTGGATGGTGGGCTGAAGTCAATGGCACG
GAAGTTACACCACTGAAAGAAAGAGCCATTTATAAAGAAGATTGGCTAGGGTTAAAGACATTGGATGAGGCCGGAAAATTAGTTTTCGAAACCA
TTCCAGGGGGACATATGACGTTAGGAGAGGAGATGCTAGAGAAGGCTTTCAAAGAGTACTTTGGTCCAGCAGGGAAGAAATTTGGG

>Bcin16g03460(MLST7), partial sequence [organism=Botrytis cinerea, strain D14_Heid15]
TCATGAACCTCTTAATTTGAACTCTTCATTATTTGCAAGTGATGAACCCCTATTCTGCCCAGAGGATAGTTACAAGACGTATATCATTAGTCGA
GAACCACTCATGATATACATTGACGGATTTTTGAAAGCGAATGAAAGTAAACACTTGGTTGATGTTAGGTGTGTTATTTATTCTGATGAAATGA
ACAAGAGAGACTGATGAGATAGTAAACCGCTTTATGAACCGTCTACTGTTTCTCACGGACAGGAAGTTACCATTGATCCTTCGGTTCGAAATTC
TGAAGTGGCGGTTTTAGAGAGGGATGAGGTGGTCAGGTGTATTGAGCATAGAGCGAGGGCATTTCGGGGTGGAGGGGCGAGATGGGGATTGAGA
AGTTGAGGACGCAGAGGTATGGGGTTGGAGGACATTATGGGATGCATTTGTAAGTTTTGGGGGATTGACGAAGGCCTCTGTTTATTTTCTATCA
GTACGGATCTTGAAAGAAAGATAGCATGAACACGAGGGCTAATAACTAGGAAATAGCGATTGGAGCGGAGGTAAACGTGGCATAGACCGATTTA
GTACTTTCATGGTCTATGTCGACGTATCCTCTGATATCGAAGGTGGAGGAACGGAATTCCCACGTATTGTGGGACCAAAAGGAGGAAGGTGGGA
GGACTTCCTGGAAACTACGGAAGCATTGGATCCAAGAACTGGAGAAAATGTAACAGTAGAAGGGGTGACATTCAAACCAATCAAGGGGAATGCC
GTATTCTGGGAAAATACTGACAACAACGGGAGGGGCTATGATG

>Bcin12g03020(MLST8), partial sequence [organism=Botrytis cinerea, strain D14_Heid15]
CGATTGGCTGCGAAGAAAACTGCGCAGCACCGAGTCACCAAAAGCACGAATAAACCATCGGCAAGGGAAATATTCTCTGAACCACAACATGACA
CGAGCGCGGAAGAGTATATCGGGCGAGAAGCTTCATCAAGAGCACCAAAGCGACAACGAGTCGATGATAATTATAACTCTTACGGTGGAAGAAA
TGAGAATACAGCAGCTTATGCTCCGGGAAACTTCCATCTGGAAGTATAAATGTTGGTGGAGGTAGGAAGACCACTTTTCAAGAAGAACCTCGAA
CGGCATTTGTCGCTGGCAAGTTGCCCCCTGGAAGTATCAACATTGGTGGAAAGAAGGCTACCCAAATCGAGGACGAGGGTAGAGCAGCTTATGC
TTCCGGAAAATTGCCCCCAGGAAGTATAAACTCTGGCGCAAAGAAGGCGATTTCATTCCAAGATGAAACGAGACCGGCTTATGTCTCTGGAAAG
CTTCCACATGGTAGTATCGACGGTATGCGAAACCGTGGAATGGCTGCCGTCCACCGCGAAATTGCTGAGGGTGGAGGAAACCAGGCCAGGTTG
TCTCTTCTCTATTCACATTCAATCCTACTTCAAAGAAAACTTTCGATGAACCCGAAGAACAAGCGGAACCGGCAAAACCATCCAATGCACCTTT
GACAGAGGAAATGGCGACTTTCACCAATCTAGGGTTATCGAGAAGGCTTGCAGCCCATCTATCGACTAAACTCGATATGAAAGCCCCGACCGCC
ATTCAAAAAGCATCTGTGCAGCAGTTGGTATCGGACGATAGCGATGCTTTTATACAAGCAGAGACTGGATCTGGAAAAACTTTGGCATATCTAC
TACCTATAGTCGAGCGAATATTAGCATTGAGTGAGAATGGCGTACAAAT

>Bcin02g07770(MLST9), partial sequence [organism=Botrytis cinerea, strain D14_Heid15]
TTACAGCTTTCCCTTTCGGTCTTGGCATCTACAGTCATTGCCATCCCTACACCATCACAACTTGAGTCTCGGGCCGTTATCGATTCCGATGCCG
TTGTAGGATTTGCCGAAACTGTTCCCAGTGGGACCGTAGGAACAGTTTATGAGGCATATAAACCATTCCTTAAAGTCGTAAATGGATGCGTACC
ATTCCCTGCCGTCGATGCATCGGGTAACACAGGGTATGTCCTTATACCATTCTCTTCCACACGATTGCTATTGAGTCTCTAACATATTTTAGTG
GTGGTTTGTCACCAACTGGCAGTAGCAATGGTGGTTGCAGCAGCAGTACCGGTCAATTATATGTTCGAGGAGGACAAAGCGGATCAAACTACGC
CATCATGTACTCCTGGTAAGTTCTCTCTAAACTTCTCCTTATAGATCCAACCTAACAAAATCTTAGGTACATGCCAAAGGACGAGCCCTCAACC
GGTATTGGTCACCGTCACGATTGGGAAGGTGTAATTGTCTGGCTCTCAAGCGCCACCGCCACAACTGCCGACAACATCTTAGCCGTTTGTCCTT
CCGCCCACGGAGGCTGGGATTGTTCCACCGATGGATATTCCCTTTCTGGTACCAGCCCTCTTATCAAGTACGAAAGTATCTGGCCCGTCGATCA
TTCAATGGGTCTTACTAGTACTGTTGGTGGAAAACAACCTATGATTGCTTGGGAGTCTTTACCAACTGCTGCTCAAACTGCTCTTGAGAACACC
GATTTCGGTGCTGCGAATGTTCCATTCATTCCGGCTGTTTTCACAGATAATCT

>Bcin04g02090(MLST10), partial sequence [organism=Botrytis cinerea, strain D14_Heid15]
CGGAGGATGATATGGCAAAGTCTATGATTACCAAAGCATTTGTAGGCATGAGTAGTAAACTGTCGAATATGACTATGAATGATGTTTACAAGCC
CTACATCCATGTAAGAAATGTAGAATAGAGAGATCAGTAACTGGAACTAATATCGTTTGCAGGCTTTCAAGTTACTTACGCAGTTCAACCCAAT
CACTACAGCTATTGCCGAATCCCCACTATTTCAAATGGCTGTCTCAGCAAATACCATCGAAAAGTACACACTGCTAGGCCCTTTCTTCAGAATA
TCTCCTCTGCAACAGGAAGTTACCAGGGAATACTTCAGTGCGCCAAAGACGATAGATAGGCGACACATTGCCACATCTCAAGATGCCTTACGAT
TGACCTTACAAACCCATCAAAAAGATTTACTTGATATCATCAACCACTTTGTTCGAGCAAGTCCAATCGCCAAAAGCAAAACCCTGGATTGGTT

CGCCTACATTGTGAATCAAAATCACAAGCGTCGAGCACTTCAGGTAGACCCGAAAGAAGTGTCTTCTGATGGTTTTATGCACAATGTCACTGTC
GTTCTAGATGGTCTTTGTGAGCCATTCATGGATACCACATTCTCAAAAATTTCGAAGATTGATATTGATTATCTAAGACGTGCGCCTCGTGTAG
ATATCAAGGACGAGACCAAGTTGAACGCTGATGAGAAGGCTTCTGAGAAGTATTATGAGGACACTGTTCCTGGCACTTCTAATTTCATCTCTGA
GGTCTTCTTTCTGACATTGGCTGCTCATCATTATGGTAGTGAAGCTCTTAATGCCACGCATAAGAGTCTGGAGAAAGACATCAAATATATTCAA
AAGCAATTGACTGCCGTTGAAGCA

>Bcin01g07220(MLST1), partial sequence [organism=Botrytis cinerea, strain D14_Ki11]
ATGGCATATCTTGTTTCTTTTCGCATTATGCTTTGGAACGTGCTGGACAAGCTTGGGTTAGTGCTCTGCGTGTAGAAGCACTAAAGAAGATTCT
CGCACAACCGAAGTCATGGTTTGAGGAATCCAGGAATTCACCTGGCCGGTTGAACGAAGTTTTGGATAGGAACTCTGAGGAAATGCGTAATCTC
GTTGGCCGCTTTGCTGGTATTGTATTCACAGCATTTTTTATGCTATTGATATCAATCATTTGGGCTTTCGTGAATACATGGAAACTGACATTAG
TCTCGATGGCAACTGGGCCAGTTATATACGCTGTCACCAAAACGTTCAATCGCGTGAGTGGAAAATGGGAAAACAAGTGCAACTACGCCTCTGA
AATGACCACTGGCATATTTTCAGAGACTTTCTCCAACATCAAAGTGGTTCGGGCTTTTACTCTGGAAACTTACTTTGAGACAAAACACACCAAA
GCTACAGAAGAACTTTATAAAGTTGGACTAATACGAGCAAACTACTCGGGATTGCTGTGGGGATTGACAGATGCGATGTCATTCTTCATCACTG
CAACTATCTTTTATTATGCCACGCTTCTCATTACCAAGAGAGAGATCAGTATCGCGACTGCACTACAGACTGTCAATCTTCTATTATTTGGTAT
TTCTAATAGTACGAATATGCTGGCCATGATACCACAAATCAACTCTTCTCGCGTTACAGCTACGCATATGCTTGTATTAGCCAATCTCGATTCA
TCTTCCTCCACGAAAATAAAGGAACCGAACGGCTTTCGACAATCTTTCCAATCAAATTCAACCGTCTCTCATTCACATATCCTACTCGTCCTGA
AAAACGAACGATATCATCCTTTTCTCTTTCCCTGATTCCTAACTCAACAACTGCACTTGTCGGACCCTCCGGCTCCGGAAAATCTACAATAGCT
GCTCTGCTCATTGGTCTTTATCCGCCAGATATTTCAACACCTCCACCGTTGACATTCAATCGCGTCTCCATAAGTAACTGTCACATTCCGTCTC
TCCGGGCTTCTCTCTCACTCGTCCACAAATACCGATTCTATTTCCAGCTACCATTCTCCATAACATCATTTATGGTCTCCCAGAATCTTCTCCT
TGTGCTAGTCTTCCATCTGCTATTGCATCAGCAAAAGATGCTGGGATCCATGAATTTATCACATCGCTTCCACAAGGTTATGATACTATG

>Bcin05g07690(MLST2), partial sequence [organism=Botrytis cinerea, strain D14_Ki11]
ACCACTATCACCAAGTCCTTTTCTGCACTTTCCTCATCAAAAACGAACGAGAACATCATCAATAAATACTTTGCTGAAGTAATTGGGTTTTATT
TTGGCCAGGATGCCTTCTCTCTACGACAGCAAGCCTTCGATGATATGTTGTGGGTAGTTCTTGGCTGGCTGGATACTGTCAAATTCATTGATTT
ACATTCTGAATTGCACTATTCAAACGACTCTCAGCCAGAATGGTACGGACAACAATATAAACCTGCATTTGCACATCGAGCGCGACTATTTTGG
GAATTGGCTTCACAAGGATGGGATACTACTCTCTGTGGTGGTGGGATGATATGGTCACCATACCTTACTCCATACAAGAACGCAATTACCAATG
AACTCTATATCGCAGCTTCGATATCGATGTACCTATATTTCCCCGGAGATGACAATCAATCCCCATTTATGCTTTCCAACCCTTCATATCCACC
TCACGATCCGAAATATCTACAGGCAGCTGTTGATGCTTACAAATGGCTGAATGGTTCCAACATGACGGATTTACAAGGATTATATGTCGACGGG
TACCATATCTCGAATCTTTCTGGCGGTGAAAACACCCATTGCGATTCTAGAAATGAGATGGTATATACCTACAATCAAGGTGTTTTGCTTACTG
GACAACGTGGTTTGTATGACGCAACCGCCGCACGATCATACCTTGTAGATGGCCACAAACTCATCGCGAATGTTATTAATGCCACAGGCTATGA
CCTGAAACACAATGTTGTCATCTCACCGCCACCCAAAGATGGTTCCGCATTGGCAAAGTGGTTTGGCCTGGGTAGGAATGGAATACTGGAAGAA
GGATGCGATTCAAGTGCTTCGTGTTCTCAAAATGGACAAACTTTCAAAGGCATATTCTTTCATCACTTGATTGCGTTCTGTAGTGATTTGCCAG
GGGAGCCTATTGCAGGGACGAAGGAAAGCTTAGAACTCGACAGAGTGTGGCATTCTGACAAATGCTCACAGTATACAAAAT

>Bcin06g01710(MLST3), partial sequence [organism=Botrytis cinerea, strain D14_Ki11]
GGTGAGTCTGACTTTTGTATTTGAGCGTTAAGATAGACACTGATATACCAAGGCAATCACCTAATTTCATGGCGTTCTTCCCTGCATCATCTAC
CTACCCTGGAATGCTGGGAGAATTATACTCAGCAGCTTTCACAGCACCTGCTTTCAATTGGATCTGTTCCCCTGCTGTGACAGAATTGGAGACG
GTTGTAATGGATTGGCTGGCCAAGCTTCTCAATCTCCCAGACTGTTATTTGTCTTCGACTCATGGTGGTGGTGTCATCCAAGGATCAGCCTCGG
AAGCTATCGTTACCGTTATGGTTGCTGCCCGCGATAAATATCTTCGTGAAACTACTGAAGGTCTGTCGGGCATTGAACTCGAGGATGCGATTGC
ATATAAGAGGAGTAAGCTAGTTGCACTAGGAAGCGAAATGGCACACAGCTCCACGCAGAAAGCAGCGCAAATAGCTGGCGTTAGATTCCGATCG
ATTCCAGTACTCGCATCCAATGATTTCGCCATGACGGGTGATGATTTAGAGAAGGTATTGAAAGAATGCAAATCTCAAGGATTGGAACCCTTCT
ATCTAACTTCGACTTTGGGAACAACATCTACATGCGCAGTTGACGACTTCGCATCTATTGCAACAGTACTTTCAAAATATGCACCTCCAGATGT
TGCAGGCGAGATCTGGGTTCACGTCGATGCTGCTTATGCAGGTGCAGCTTTGGTTTGCCCTGAATACCATCATCTAACATCGTCTTTCCAGCAT
TTCCATTCCTTCGATATGAACATGCACAAATGGCTTCTGACAAATTTCGACGCTTCTTGTCTATATGTCAAGAAACGCAAAGATCTGATCGATG
CACTGTCCATAACACCAAGTTATCTTCGCAACGAGTTTTCAGAGAGTGGACTCGTAACCGACTATCGGGACTGGCAAATTCCTCTCGGAAGACG
CTTCCGAAGCTTAAAGATTTGGTTTGTCCTCAGAACCTAC

>Bcin09g03030(MLST4), partial sequence [organism=Botrytis cinerea, strain D14_Ki11]
ACCCTTCAACCCCACCGCAGCTCCTATACCGAAAGCGAGTATCCTACCAATTCTCCTTCCACCTGCGACTTTAAGACCATTGGCTTTCCGCACT
TTCACAAAAAAGCATAGTTTAACATTGACGTCGTCGGCATTACAAGTGTTGGCTACTTTTATTGGAAAGCATTGTGGGACAGGATGGAGGGAAG
AAGGACTGGCAGAGAGAGAGTCTTGGAGGAGGTCGCCAAGAGTTGGAAGAATAGGAGTGGCGGTGTCATTGTCGAGGGCGAGGGAACGGAATTGAA
GGAGATTCTGAAAGCTTTGGAAGGGAATATGAGTGGTGGAAGGATAGTCATAGGAAGAGAGCTAAGCCGACAGAATAGTTTAGTACTGGGATCA
TCACAATATGGAGAGGTCAATCATACAAGACTTGGGCTACGGCCAGGGAATATACCCAGAGAGGATAGTCAGTCAAGTTTGGGAATGTCAACGT
TGGAGGTCAATGACGAGGAAGATGAGGATGGCCTGATGGATCCAAGAAGGTGGTTAAAAGTCATTGATGCATTTGAGCAACCTCGACTGGTGTA
CAATGTTGCTAAAAAGCACTTTGATAGGTATGTTTCAATGATAAAATTTTATCTGAATCGACTAACTCAGTACAGAGATACCTCCAAACCTTCA
TTGTTCCCACCTGCGTCTCATAAAACACTCCTCTTCCAAAACCGCTATAATGTTATCCATCAACGTCTCCTTCGCAATGAATCTTTTCAAACGC
CCGCTTTTCAAGGTGGCAAATCTTCCCTTCAACGCAGCACGTCCGCCATTACCACACAACAACAATCATACAAATTAACGCCGATAGCTAATCT

TCTCGGTCGCAATCGCAGCTCTCATATGCTTCTCGGTCTCCTCAGTATTTCACCCACTGGTACCCTCGCCATCAATGACCTGACGGGCAGTATC
GCTCTCGATCTTACACACGCAGCAGCCATTC

>Bcin11g01310(MLST5), partial sequence [organism=Botrytis cinerea, strain D14_Ki11]
GACTGACATGGACCTCATGTGGAACCGGCGTTAGAATGCGCAACGCTTGAAGTTCCGCTCGAATATGGCGATGCAACGTCAACGGCAAAAGCCA
GTGTTGCGCTTGCTCGTTATCCTGCCACTGTTGCCGCGAGCAAGAAGCTCGGGTCTCTCTTGATAAACCCCGGTGGACCCGGTGCCTCTGGTGT
TGGCTTTGTGCAGTCTGGAGCCGGTGCCGCCGTCTCGACACTGAGTGGTGGATTGTACGATATCATCGGATGGGATCCACGTGGAACCGGTGCT
TCGGCTCCTATTTTGGAATGTTTTGCAAATGCCAGTGCGGAGTATGATTTTAACAACGCGTTTCCATCTGCTCCGAATCTCTGGCTCGGACAAT
TTGCGAATGCCAGCGCAAATTCTGCTGTTAGCTCTGCTATCACATCCTTTGACACTTCTGTCGCTGCTCTTGCAAAAGCTTGCGTGGCTCAGAA
ATCTCCCGCTCTTTACACCTCAACAGCAGCATATGTTGCTCGAGACATGGCAGCGATAGTCGATGCATTGGATGGGACCTCTGCAAAACTTAAC
TACTGGGGTTTCTCATATGGAACTATCTTCCTAGCTGAGTTTATCCAAACTTTCCAGGCCGCGTGGGAAGAGTTCTTGCCGATGGTGTTTTCGA
CGCAAAGGCAAATGCACTCACATACGTTAGCCAACTTCCCAACGATCAACTCAGTGTTCGTGCTTCGTTGAACGATTTTGCAGCTTTCTGCACC
ACCGCCGGTAGTAAAGGTTGCTCTTTTGCCACCGCCCCTACTGGAACCACAGGTACTGTTGCTACCAGACTGGACAACATAATGAAGGATATGT
TCCTCAATCCTATTGTTGCTTCGGGCTTAAGCATCA

>Bcin15g03910(MLST6), partial sequence [organism=Botrytis cinerea, strain D14_Ki11]
GCCAAAACACAAAATCATCCAACGATGAAGATGATACTCCACTTCCCTTGATTATTGGCATGGACTCGGCGATAATTACAAAGCGGATGGTCTT
GCGCAAGTTGGAAAACTAGCTGAAGCTATTCATCCTGGGACTTTTGTCTACAATATTCATGTAGATGAGGATGCATCTGCAGATAGGACAGCTA
CCTTCTTTGGAAATCTCACTCGTGAGTACATCCCCTATTTTCCCTTTAAATACCATACTAACTCTCTTACCAAGTTCAAATCGAAAAGGTCTGC
GAAGACCTCGCCTCCCATCCTATTCTCTCTACCGCGCCCGCCGTCGACGCAATTGGATTCTCCCAAGGCGGCCAATTCTTGCGCGGTTACATAT
CCCGCTGCAATGCTCCACCCATCCGCTCTCTCCTGACCTTCGGTTCCCAACACAACGGCATTTCTGCCTTCCAAGCCTGTGGTCCTGCCGATTT
CCTCTGTCGCGGTGCTCAAACCCTTTTGCGATCCAACACCTGGTCAACCTTTGTCCAATCTCGTCTCGTACCCGCTCAATACTTCAGAGATCCG
GAAAACCTAGACTCTTACCTTGAATATTCCAATTTCCTTGCCGACATCAATAATGAGCGCGTTCTCAAGAACCAAACATATAAATCCAACATGG
AAAAATTGGAACGATTCGTAATGTATGTCTTTGAAGACGATACAACTGTCATCCCTAAGGAAAGTGGATGGTGGGCTGAAGTCAACGGCACGGA
AGTTACACCACTGAAAGAAAGAGCCATTTATAAAGAAGATTGGCTAGGTTTAAAGACATTGGATGAGGCCGGAAAATTAGTTTTCGAAACCATT
CCAGGGGGACATATGACGTTAGGAGAGGAGATGCTAGAGAAGGCTTTCAAAGAGTATTTTGGTCCAGCAGGGAAGAAATTTGGG

>Bcin16g03460(MLST7), partial sequence [organism=Botrytis cinerea, strain D14_Ki11]
ATGAACCTCTTGATTTGAACTCTTCATTAATTGCAAGTGATGAACCCCTATTCTGCCCAGAGGATAGTTACAAGACGTATATCATTAGTCGAGA
ACCACTCATGATATATATTGACGGGTTTTTGAAAGCGAATGAAAGTAAACATTTGGTTGATGTTAGGTGTGTTATTTATTCTGATGAAATGAAC
AAGAGAGACTGATGAGATAGTGAGCCGCTTTATGAACCGTCAACTGTTTCTCACGGACAGGAAGTTACCATTGATCCTTCAGTTCGGAATTCTG
AAGTGGCGGTTTTAGAGAGGGATGAGGTGGTCAGGTGTATTGAGCATAGAGCGAGGGCATTTCAGGGGTGGAGGGGCGAGATGGGGATTGAGAA
GTTGAGGACGCAGAGGTATGGGGTTGGAGGACATTATGGGATGCATTTGTAAGTTTTGGGGGATTGACGAAGGCTTCTGTCTATTTTCTATCAG
CACGGATCTTGAAAGAAAGATAGCATGAACACGCGGGCTAATAACTAGGAAATAGCGATTGGAGCGGAGGTAAACGTGGCATAGACCGATTTAG
TACTTTCATGGTCTATGTCGACGTATCCTCTGATATCGAAGGTGGAGGAACGGAATTCCCACGTATCGTGGGACCAAAAGGAGGAAGGTGGGAG
GACTTCCTGGAAACTACGGAAGCATTGGATCCAAGAACTGGAGAAAATGTAACAGTAGAAGGGGTGACATTCAAACCAATCAAGGGAAATGCCG
TATTCTGGGAAAATACTGACAACAACGGGAGGGGCTATGAT

>Bcin12g03020(MLST8), partial sequence [organism=Botrytis cinerea, strain D14_Ki11]
CGATTGGCTGCGAAGAAAACTGCGCAGCACCGAGTCACCAAAAGCACGAATAAACCATCGGCAAGGGAAATATTCTCTGAACCACAACATGACA
CGAGCGCGGAAGAGTATATCGGGCGAGAAGCTTCATCAAGAGCACCAAAGCGACAACGAGTCGATGATAATTATAACTCTTACGGTGGAAGAAA
TGAAAATACAGCAGCTTATGCTTCCGGGAAACTTCCATCTGGAAGTATAAATGTTGGTGGAGGTAGGAAGACCACTTTTCAAGAAGAACCTCGA
ACGGCATTTGTCGCTGGCAAGTTGCCCCCTGGAAGTATCAACATTGGTGGAAAGAAGGCTACCCAAATCGAGGACGAGGGTAGAGCAGCTTATG
CTTCCGGAAAATTGCCCCAGGAAGTATAAACTCTGGCGCAAAGAAGGCGATTTCATTCCAAGATGAAACGAGACCGGCTTATGTCTCCGGAAAG
CTTCCACATGGTAGTATCGACGGTATGCGAAACCGTGAAATGGCTGCCGTCCACCGCGAAATTGCTGAGGGTGGAGGAAACCAGGCCAGGTTG
TCTCTTCTCTATTCACATTCAATCCTACTTCAAAGAAAACTTTCGATGAACCAGAAGAACAAGCGGAACCGGCAAAACCATCCAATGCGCCTTT
GACAGAGGAAATGGCGACTTTCACCAATCTAGGGCTATCGAGAAGGCTTGCAGCCCATCTATCGACTAAACTCGATATGAAAGCCCCGACGGCC
ATTCAAAAAGCATCTGTGCAGCAGTTGGTATCGGACGATAGCGATGCTTTCATACAAGCAGAGACTGGATCTGGAAAAACTTTGGCATATCTAC
TACCTATAGTCGAGCGAATATTAGCATTGAGTGAGAATGGCGTACAAAT

>Bcin02g07770(MLST9), partial sequence [organism=Botrytis cinerea, strain D14_Ki11]
CAGCTTTCCCTTTCGGTCTTGGCATCTACAGTCATTGCCATCCCTACACCATCACAACTTGAGTCTCGGGCCGTTATCGATTCCGATGCCGTTG
TAGGATTTGCCGAAACTGTTCCCAGTGGGACCGTAGGAACAGTTTATGAGGCATATAAACCATTCCTTAAAGTCGTAAATGGATGCGTACCATT
CCCTGCCGTCGATGCATCGGGTAACACAGGGTATGTCCTTATATCTTTCTCTTCCACACGATTGCTATTGAGTCTCTAACATATTTAGTGGTG
GTTTGTCACCAACTGGCAGTAGCAATGGTGGTTGCAGCAGCAGTACCGGTCAAGTATATGTTCGAGGAGGACAAAGCGGATCAAACTACGCCAT
CATGTACTCCTGGTAAGTTCTCTCTAAACTTCTCCTTATAGATCCAACCTAACAAAATCTTAGGTACATGCCAAAGGACGAGCCCTCAACCGGT
ATTGGTCACCGTCACGATTGGGAAGGTGTAATTGTCTGGCTCTCCAGCGCCACCGCCACAACTGCCGACAACATCTTAGCCGTTTGTCCTTCCG

CCCACGGAGGCTGGGATTGTTCCACGGATGGCTATTCCCTTTCTGGTACCAGCCCTCTTATCAAGTACGAAAGTATCTGGCCCGTCGATCACTC
AATGGGTCTTACTAGTACTGTTGGTGGAAAACAACCTATGATTGCTTGGGAGTCTTTACCAACTGCTGCTCAAACTGCTCTTGAGAACACCGAT
TTCGGTGCTGCGAATGTTCCATTCATTCCGGCTGTTTTCACAGACAATCT

>Bcin04g02090(MLST10), partial sequence [organism=Botrytis cinerea, strain D14_Ki11]
CGGAGGATGATATGGCAAAGTCTATGATTACCAAAGCATTTGTAGGCATGAGTAGTAAACTGTCGAATATGACTATGAATGATGTTTACAAGCC
CTACATCCATGTAAGAAATGTAGAATAGAGAGATCAGTAACTGGAACTAATATCGTTTGTAGGCTTTCAAGTTACTTACGCAGTTCAACCCAAT
CACTACAGCTATTGCCGAATCCCCACTGTTTCAAATGGCCGTCTCAGCAAATACCATCGAAAAGTACACACTGCTAGGCCCTTTCTTCAGAATA
TCTCCTCTGCAACAGGAAGTTACCAGGGAATACTTCAGTGCGCCAAAGACGATAGATAGGCGACACATTGCCACATCTCAAGATGCGTTACGAT
TGACCTTACAAACCCATCAAAAAGATTTACTTGATATCATCAACCACTTTGTTCGAGCAAGTCCAATCGCAAAAAGCAAAACCCTGGATTGGTT
CGCCTACATTGTGAATCAAAATCACAAGCGTCGAGCACTTCAGGTAGACCCGAAAGAAGTGTCTTCTGATGGCTTTATGCACAATGTCACTGTC
GTTCTAGATGGTCTTTGTGAGCCATTCATGGATACCACATTCTCGAAAATTTCGAAGATTGATATTGATTATCTAAGACGTGCGCCTCGTGTAG
ATATCAAGGACGAGACCAAGTTGAACGCTGACGAGAAGGCTTCTGAGAAGTATTATGAGGACACTGTTCCTGGCACTTCTAATTTCATCTCTGA
GGTCTTCTTTCTCACATTGGCTGCTCATCATTATGGTAGTGAAGCTCTTAATGCCACGCATAAGAGTCTGGAGAAAGACATCAAATATATTCAA
AAGCAATTGACTGCCGTTGAAGCA

>Bcin01g07220(MLST1), partial sequence [organism=Botrytis cinerea, strain D14_Ki12]
GATGGCATATCTTGTTTCTTTTCGCATTATGCTTTGGAACGTGCTGGGCAAGCTTGGGTTAGTGCTCTGCGTGTAGAAGCACTAAAGAAGATTC
TCGCACAACCGAAGTCATGGTTTGAGGAATCCAGGAATTCACCTAGCCGGTTGAACGAAGTTTTGGATAGGAACTCTGAGGAAATGCGTAATCT
CGTTGGCCGCTTTGCTGGTATTGTATTCACAGCATTTTTTATGCTATTGATATCAATCATTTGGGCTTTCGTGAATACATGGAAACTGACATTA
GTCTCAATGGCAACTGGGCCAGTTATATACGCTGTCACCAAAACGTTCAATCGCGTGAGTGGAAAATGGGAAAACAAGTGCAACTACGCATCTG
AAATGACCACTGGCATATTTTCAGAGACTTTCTCCAACATCAAGTGGTTCGGGCTTTTACTCTGGAAACTTACTTTGAGACAAAACACACCAAA
GCTACAGAAGAACTCTATAAAGTTGGACTAATACGAGCAAACTACTCGGGATTGCTGTGGGGATTGACAGATGCGATGTCATTCTTCATCACTG
CAACTATCTTTTATTATGCCACGGTTCTCATTACCAAGAGAGAGATCAGTATTGCGACTGCACTACAGACTGTCAATCTTCTATTATTTGGTAT
TTCTAATAGTACGAATATGCTGGCTATGATACCACAAATCAACTCTTCTCGCGTTACAGCTACGCATATGCTTGCATTAGCCAATCTCGATTCA
TCTTCCTCCCACGAAAATAAAGGAACCGAACGGCTTTCGACAATCTTTCCAACCAAATTCAACCGTCTTTCATTCACATACCCTACTCGTCCTG
AAAAACGAACGATATCATCCTTTTCTCTTTCCCTGATTCCTAACTCAACAACTGCACTTGTCGGACCCTCCGGCTCCGGAAAATCTACGATAGC
TGCTCTGCTCATTGGTCTCTATCCGCCAGATACTTCAACACCTCCACCGTTGACATTCAATCGCGTCTCCATAAGTAACTGTCACATTCCGTCT
CTCCGGGCTTCTCTCTCACTCGTCCCACAAATACCGATTCTATTTCCAGCTACCATTCTCCATAACATCATTTATGGTCTCCCAGAATCTTCTC
CTTGTGCTAGTCTTCCATCTGCTATTGCATCAGCAAAAGATGCTGGGATCCATGAATTTATCACATCGCTTCCACAAGGTTATGATACTATG

>Bcin05g07690(MLST2), partial sequence [organism=Botrytis cinerea, strain D14_Ki12]
TCACCACTATCACCAAGTCCTTTTCTGCACTTTCCTCATCAAAAACGAACGAGAACATCATCAATAAATACTTTGCTGAAGTAATTGGGTTTTA
TTTTGGCCAGGATGCCTTCTCTCTACGACAGCAAGCCTTCGATGATATGTTGTGGGTAGTTCTTGGCTGGCTGGATACTGTCAAATTCATTGAT
TTACATTCTGAATTGCACTATTCAAACGACTCTCAGCCAGAATGGTACGGACAACAATATAAACCTGCATTTGCACATCGAGCGCGACTATTTT
GGGAATTGGCTTCACAAGGATGGGATACTACTCTCTGTGGTGGTGGGATGATATGGTCACCATACCTTACTCCATACAAGAATGCAATTACCAA
TGAACTCTATATCGCAGCTTCGATATCGATGTACCTCTATTTCCCCGGAGATGACAATCAATCCCCATTTATGCTTTCCAACCCTTCATATCCA
CCTCACGATCCGAAATATCTACAGGCAGCTGTTGATGCTTACAAATGGCTGAATGGTTCCAACATGACGGATTTACAAGGATTATATGTCGACG
GGTATCATATCTCGAATCTTTCTGGCGGTGAAAACACCCATTGCGATTCTAGAAATGAGATGGTATATACCTACAATCAAGGTGTTTTGCTTAC
TGGACAACGTGGTTTGTATGACGCAACCGCCGCACGATCATACCTTGTGGATGGCCACAAACTCATCGCGAATGTTATTAATGCCACAGGCTAT
GACCTGAAACACAATGTTGCCATCTCACCGCCACCCAAAGATGGTTCCGCATTGGCAAAGTGGTTTGGCCTGGGTAGGAATGGAATACTGGAAG
AAGGATGCGATTCAAGTGCTTCGTGTTCTCAAAATGGACAAACTTTCAAAGGCATATTCTTTCACCACTTGATTGCGTTCTGTAGTGATTTGCC
AGGGGAGCCTATTGCAGGGACGAAGGAAGGCCTAGAACTCGACAGAGTGTGGCATTCTGACAAATGCTCACAGTATACAAAATG

>Bcin06g01710(MLST3), partial sequence [organism=Botrytis cinerea, strain D14_Ki12]
GTGAGTCTGACTTTTGTATTTGAGCGTTAAGATGGACACTGATGTACCAAGGCAATCACCTAATTTCATGGCGTTCTTCCCTGCATCATCTACC
TACCCTGGAATGCTGGGAGAATTATACTCAGCAGCTTTCACAGCTCCTGCTTTCAATTGGATCTGTTCCCCTGCTGTGACAGAATTGGAGACGG
TTGTAATGGATTGGTTGGCCAAGCTTCTCAATCTCCCAGACTGTTATTTGTCGTCGACTCATGGTGGTGGTGTTATCCAAGGATCAGCCTCGGA
AGCTATCGTTACCGTTATGGTTGCTGCCCGCGATAAGTATCTTCGTGAAACCACTGAAGGTCTGTCGGGAATTGAACTCGAGGATGCGATTGCA
TATAAGAGGAGTAAGCTAGTTGCACTAGGAAGCGAAATGGCACACAGCTCCACGCAGAAAGCAGCGCAGATAGCTGGCGTTAGATTCCGATCGA
TTCCAGTACTCGCATCCAATGATTTCGCCATGACGGGTGATGATTTAGAGAAGGTATTGAAAGAATGCAAATCTCAAGGATTGGAACCCTTCTA
TCTAACTTCGACGTTGGGAACAACATCTACATGCGCAGTTGACGACTTCGCATCTATTGCAACAGTACTTTCAAAATATGCACCTCCAGATGTT
GCAGGCGAGATCTGGGTTCACGTCGATGCTGCTTATGCAGGTGCAGCTTTGGTTTGCCCTGAATACCATCATCTAACATCGTCCTTCCAGCATT
TCCATTCCTTTGATATGAACATGCACAAATGGCTTCTGACAAATTTCGACGCTTCTTGCCTATATGTCAAGAAACGCAAAGATCTGATCGATGC
ACTCTCCATAACACCAAGTTATCTTCGCAACGAGTTTTCAGAGAGTAGACTCGTAACCGACTATCGGGACTGGCAAATTCCTCTCGGAAGACGT
TTCCGAAGCTTAAAGATTTGGTTTGTCCTCAGAACCTACGG

>Bcin09g03030(MLST4), partial sequence [organism=Botrytis cinerea, strain D14_Ki12]
GACCCTTCAACCCCACCGCAGCTCCTATACCGAAAGCGAGTATCCTACCAATTCTCCTTCCACCTGCGACTTTAAGACCATTGGCTTTTCGCAC
TTTCACAAAAAAGCATAGTTTAACATTGACGTCGTCGGCATTACAAGTGTTGGCTACTTTTATTGGAAAGCATTGTGGGACAGGATGGAGGGAG
GAAGGACTGGCAGAGAGAGTCTTGGAGGAGGTCGCCAAGAGTTGGAAGAATAGGAGTGGCGGTGTCATTGTCGAGGGCGAGGGAACGGAATTGA
AGGAGATTCTGAAAGCTTTGGAAGGGAATATGAGTGGTGGAAGGATAGTCATAGGAAGAGAGCTAAGCCGGCAGAATAGTTTAGTACTGGGATC
ATCACAATATGGAGAGGTCAATCATACAAGACTTGGGCTACGGCCAGGGAATATACCCAGAGAGGATAGTCAGTCAAGTTTGGGAATGTCAACG
TTGGAGGTCAATGACGAGGAAGATGAGGATGGCCTGATGGATCCAAGAAGGTGGTTAAAAGTCGTTGATGCATTTGAGCAACCTCGACTGGTGT
ACAATGTTGCTAAAAAGCACTTTGATAGGTATGTTTCAATGATAAAATTTTATCTGAATCGACTAACTCAGTACAGAGATACCTCCAAACCTTC
ATTGTTCCCACCTGCGTCTCATAAAACACTCCTCTTCCAAAACCGCTATAATGTTATCCATCAACGTCTCCTTCGCAATGAATCTTTTCAAACG
CCCGCTTTTCAAGGTGGCAAATCTTCCCTTCAACGCAGCACGTCCGCCATTACCACCCAACAACAATCATACAAATTAACGCCGATAGCTAATC
TTCTCGGTCGCAATCGCAGCTCTCATATGCTTCTCGGTCTCCTCAGTATTTCACCCACTGGTACCCTCGCCATCAATGACCTGACGGGCAGTAT
CGCTCTTGATCTTACACACGCAGCAGCCATTC

>Bcin11g01310(MLST5), partial sequence [organism=Botrytis cinerea, strain D14_Ki12]
ACTGACATGGACCTCATGTGGAACCGGCGTAGAATGCGCAACGCTTGAAGTTCCGCTCGAATATGGCGATGCAACGTCAACGGCAAAAGCCAGT
GTTGCGCTTGCTCGTTATCCTGCCACTGTTGCCGCGAGCAAGAAGCTCGGGTCTCTCTTGATAAATCCCGGTGGACCCGGTGCCTCTGGTGTTG
GCTTTGTGCAGTCTGGAGCCGGTGCCGCCGTCTCGACACTGAGTGGTGGATTATACGATATCATCGGATGGGATCCACGTGGAACCGGTGCTTC
GGCTCCTATTTTGGAATGTTTTGCAAATGCCAGTGCGGAGTATGATTTTAACAACGCGTTTCCATCTGCTCCGAATCTCTGGCTCGGACAATTT
GCGAATGCCAGCGCAAATTCTGCTGTTAGCTCTGCTATCACATCCTTTGACACTTCTGTCGCTGCTCTTGCAAAAGCTTGCGTGGCTCAGAAAT
CTCCCGCTCTTTACACCTCAACAGCAGCATATGTTGCTCGAGACATGGCAGCGATAGTCGATGCATTGGATGGGACCTCTGCAAAACTTAACTA
CTGGGGTTTCTCATATGGAACTATCTTCCTAGCTGAGTTTATCCAAACTTTCCCAGGCCGCGTGGGAAGAGTTCTTGCCGATGGTGTTTTCGAC
GCAAAGGCAAATGCACTCACATACGTTAGCCAACTTCCCAACGATCAACTCAGTGTTCGTGCTTCGTTGAACGATTTTGCAGCTTTCTGCACCA
CCGCCGGTAGTAAAGGTTGCTCTTTTGCCACCGCCCCTACTGGAACCTCAGGTACTGTTGCTACCAGACTGGACAACATAATGAAGGATATGTT
CCTCAATCCTATTGTTGCTTCGGGCTTGAGCATCA

>Bcin15g03910(MLST6), partial sequence [organism=Botrytis cinerea, strain D14_Ki12]
GCCAAAACACAAAATCATCCAACGATGAAGATGATACTCCACTTCCCTTGATTATCTGGCATGGACTCGGCGATAATTACAAAGCGGATGGTCT
TGCGCAAGTTGGAAAACTAGCTGAAGCTATTCATCCTGGGACTTTTGTCTATAATATTCATGTAGATGAGGATGCATCTGCAGATAGGACAGCT
ACCTTCTTTGGAAATCTCACTCGTGAGTACATCCCCTATTTTTCCTTTAAATACCATACTAACTCTCTTACCAAGTTCAAATCGAAAAGGTCTG
CGAAGACCTCGCCTCCCATCCTATTCTCTCTACCGCGCCCGCCGTCGACGCAATTGGATTCTCCCAAGGCGGCCAATTCTTGCGCGGTTACATA
TCCCGCTGCAATGCTCCACCCATCCGCTCTCTCCTGACCTTCGGTTCCCAACACAACGGCATTTCTGCCTTCCAAGCCTGTGGTCCTGCCGATT
TCCTCTGTCGCGGTGCTCAAACCCTTTTGCGATCCAACACCTGGTCAACCTTTGTCCAATCTCGTCTCGTACCTGCTCAATACTTCAGAGATCC
GGAAAACCTAGACTCTTACCTTGAATATTCCAATTTCCTTGCCGACATCAATAATGAGCGCGTTCTCAAGAACCAAACATATAAATCCAACATG
GAAAAATTGGAACGATTCGTAATGTATGTCTTTGAAGACGATACAACTGTCATCCCTAAGGAAAGTGGATGGTGGGCTGAAGTCAATGGCACGG
AAGTTACACCACTGAAAGAAAGAGCCATTTATAAAGAAGATTGGCTAGGGTTAAAGACATTGGATGAGGCCGGAAAATTAGTTTTCGAAACCAT
TCCAGGGGGACATATGACGTTAGGAGAGGAGATGCTAGAGAAGGCTTTCAAAGAGTACTTTGGTCCAGCAGGGAAAAAATTTGGG

>Bcin16g03460(MLST7), partial sequence [organism=Botrytis cinerea, strain D14_Ki12]
ATGAACCTCTTAATTTGAACTCTTCATTAATTGCAAATGATGAACCCCTATTCTGCCCAGAGGATAGTTACAAGACGTATATCATTAGTCGAGA
ACCACTCATGATATACATTGACGGATTTTTGAAAGCGAATGAAAGTAAACACTTGGTTGATGTTAGGTGTGTTATTTATTCTGATGAAATGAAC
AAGAGAGACTGATGAGATAGTGAACCGCTTTATGAACCGTCTACTGTTTCTCACGGACAGGAAGTTACCATTGATCCTTCGGTTCGAAATTCTG
AAGTGGCGATTTTAGAGAGGGATGAGGTGGTCAGGTGTATTGAGCATAGAGCGAGGGCATTTCAGGGGTGGAGGGGCGAGATGGGGATTGAGAA
GTTGAGGACGCAGAGGTATGGGGTTGGAGGACATTATGGGATGCATTTGTAAGTTTTGGGGGATTGACGAAGGCTTCTGTCTATTTTCTATCAG
TGCGGATCTTGAAAGAAAGATAGCATGAACACGAGGGCTAATAACTAGGAAATAGCGATTGGAGCGGAGGTAAACGTGGCATAGACCGATTTAG
TACTTTCATGGTCTATGTCGACGTATCCTCTGATATCGAAGGTGGAGGAACGGAATTCCCACGTATTGTGGGACCAAAAGGAGGAAGGTGGGAG
GACTTCCTGGAAACTACGGAAGCATTGGATCCAAGAACTGGAGAAAATGTAACAGTAGAAGGGGTGACATTCAAACCAATCAAGGGAAATGCCG
TATTCTGGGAAAATACTGACAACAACGGGAGGGGCTATGAT

>Bcin12g03020(MLST8), partial sequence [organism=Botrytis cinerea, strain D14_Ki12]
CGATTGGCTGCGAAGAAAACTGCGCAGCACCGAGTCACCAAAAGCACGAATAAACCATCGGCAAGGGAAATATTCTCTGAACCACAACATGACA
CGAGCGCGGAAGAGTATATCGGGCGAGAAGCTTCATCAAGAGCACCAAAGCGACAACGAGTCGATGATAATTATAACTCTTACGGTGGAAGAAA
TGAGAATACAGCAGCTTATGCTTCCGGGAAACTTCCATCTGGAAGTATAAATGTTGGTGGAGGTAGGAAGACCACATTTCAAGAAGAACCTCGA
ACGGCATTTGTCGCTGGCAAGTTGCCCCCTGGAAGTATCAACATTGGTGGAAAGAAGGCTACCCAAATCGAGGACGAGGGTAGAGCAGCTTATG
CTTCCGGAAAATTGCCCCCAGGAAGTATAAACTCTGGCGCAAAGAAGGCGATTTCATTCCAAGATGAAACGAGACCGGCTTATGTCTCTGGAAA
GCTTCCACATGGTAGTATCGACGGTATGCGAAACCGTGAAATGGCTGCCGTCCACCGCGAAATTGCTGAGGGTGGGAGGAAACCAGGCCAGGTT
GTCTCTTCTCTATTCACATTCAATCCTACTTCAAAGAAAACTTTCGATGAACCCGAAGAACAAGCGGAACCGGCAAAACCATCCAATGCACCTT
TGACAGAGGAAATGGCGACTTTCACCAATCTAGGGTTATCGAGAAGGCTTGCAGCCCATCTATCGACTAAACTCGATATGAAAGCCCCGACCGC

CATTCAAAAAGCATCTGTGCAGCAGTTGGTATCGGACGATAGCGATGCTTTCATACAAGCAGAGACTGGATCTGGAAAAACTTTGGCATATCTA
CTACCTATAGTCGAGCGAATATTAGCATTGAGTGAGAATGGCGTACAAA

>Bcin02g07770(MLST9), partial sequence [organism=Botrytis cinerea, strain D14_Ki12]
CAGCTTTCCCTTTCGGTCTTGGCATCTACAGTCATTGCCATCCCTACACCATCACAACTTGAGTCTCGGGCCGTTATCGATTCCGATGCCGTTG
TAGGATTTGCCGAAACTGTTCCCAGTGGGACCGTAGGAACAGTTTACGAGGCATATAAACCATTCCTTAAAGTCGTAAATGGATGCGTACCATT
CCCTGCCGTCGATGCATCGGGTAACACAGGGTATGTCCTTATACCATTCTCTTCCACACGATTGCTATTGAGTCTCTAACATATTTTAGTGGTG
GTTTGTCACCAACTGGCAGTAGCAATGGTGGTTGCAGCAGCAGTACCGGTCAAGTATATGTTCGAGGAGGACAAAGCGGATCAAACTACGCCAT
CATGTACTCCTGGTAAGTTCTCTCTAAACTTCTCCTTATAGATCCAACCTAACAAAATCTTAGGTACATGCCAAAGGACGAGCCCTCAACCGGT
ATTGGTCACCGTCACGATTGGGAAGGTGTAATTGTCTGGCTCTCAAGCGCCACCGCCACAACTGCCGACAACATCTTAGCCGTTTGTCCTTCCG
CCCACGGAGGCTGGGATTGTTCCACCGATGGCTATTCCCTTTCTGGTACCAGCCCTCTTATCAAGTACGAAAGTATCTGGCCCGTCGATCATTC
AATGGGTCTTACTAGTACTGTTGGTGGAAAACAACCTATGATTGCTTGGGAGTCTTTACCAACTGCTGCTCAAACTGCTCTTGAGAACACCGAT
TTCGGTGCTGCGAATGTTCCATTCATTCCGGCTGTTTTCACAGATAATCT

>Bcin04g02090(MLST10), partial sequence [organism=Botrytis cinerea, strain D14_Ki12]
GCGGAGGATGATATGGCAAAGTCTATGATTACCAAAGCATTTGTAGGCATGAGTAGTAAACTGTCGAATATGACTATGAATGATGTTTACAAGC
CCTACATCCATGTAAGAAATGTAGAATAGAGAGATCAGTAACTGGAACTAATATCGTTTGCAGGCTTTCAAGTTACTTACGCAGTTCAACCCAA
TCACTACAGCTATTGCCGAATCCCCACTATTTCAAATGGCTGTCTCAGCAAATACCATCGAAAAGTACACACTGCTAGGCCCTTTCTTCAGAAT
ATCTCCTCTGCAACAGGAAGTTACCAGGGAATACTTCAGTGCGCCAAAGACGATAGATAGGCGACACATTGCCACATCTCAAGATGCCTTACGA
TTGACCTTACAAACCCATCAAGAAGATTTACTTGATATCATCAACCACTTTGTTCGAGCAAGTCCAATCGCCAAAAGCAAAACCCTGGATTGGT
TCGCCTACATTGTGAATCAAAATCACAAGCGTCGAGCACTTCAGGTAGACCCGAAAGAAGTGTCTTCTGATGGTTTTATGCACAATGTCACTGT
CGTTCTAGATGGTCTTTGTGAGCCATTCATGGATACCACATTCTCAAAAATTTCGAAGATTGATATTGATTATCTAAGACGTGCACCTCGTGTA
GATATCAAGGACGAGACCAAGTTGAACGCTGATGAGAAGGCTTCTGAGAAGTATTATGAGGACACTGTTCCTGGCACTTCTAATTTCATCTCTG
AGGTCTTCTTTCTGACATTGGCTGCTCATCATTATGGTAGTGAAGCTCTTAATGCCACGCATAAGAGTCTGGAGAAAGACATCAAATATATTCA
AAAGCAATTGACTGCCGTTGAAGCA

>Bcin01g07220(MLST1), partial sequence [organism=Botrytis cinerea, strain G09_S04]
ATGGCATATCTTGTTTCTTTTCGCATTTATGCTTTGGAACGTGCTGGACAAGCTTGGGTTAGTGCTCTGCGTGTAGAAGCACTAAAGAAGATTC
TCGCACAACCGAAGTCATGGTTTGAGGAATCCAGGAATTCACCTGGCCGGTTGAACGAAGTTTTGGATAGGAACTCTGAGGAAATGCGTAATCT
CGTTGGCCGCTTTGCTGGTATTGTATTCACAGCATTTTTATGCTATTGATATCAATCATTTGGGCTTTCGTGAATACATGGAAACTGACATTAG
TCTCGATGGTAACTGGGCCAGTTATATACGCTGTCACCAAAACGTTCAATCGCGTGAGTGGAAAATGGGAAAACAAGTGCAACTACGCCTCTGA
AATGACCACTGGCATATTTTCAGAGACTTTCTCCAACATCAAAGTGGTTCGGGCTTTTACTCTGGAAACTTACTTTGAGACAAAACACACCAAA
GCTACAGAAGAACTTTATAAAGTTGGACTAATACGAGCAAACTACTCGGGATTGCTGTGGGGATTGACAGATGCGATGTCATTCTTCATCACTG
CAACTATCTTTTATTATGCCACGGTTCTCATTACCAAGAGAGAGATCAGTATCGCGACTGCACTACAGACTGTCAATCTTCTATTATTTGGTAT
TTCTAATAGTACGAATATGCTGGCCATGATACCACAAATCAACTCTTCTCGCGTTACAGCTACGCATATGCTTGCATTAGCCAATCTCGATTCA
TCTTCCTCCCACGAAAATAAAGGAACCGAACGGCTTTCGACAATCTTTCCAATCAAAATTCAACCGTCTCTCATTCACATATCCTACTCGTCCT
GAAAAACGAACGATATCATCCTTTTCTCTTTCCCTGATTCCTAACTCAACAACTGCACTTGTCGGACCCTCCGCTCCGGAAAATCTACAATAGC
TGCTCTGCTCATTGGTCTCTATCCGCCAGATACTTCAACACCTCCACCGTTGACATTCAATCGCGTCTCCATAAGTAACTGTCACATTCCGTCT
CTCCGGGCTTTCTCTCTCACTCGTTCCACAAATACCGATTCTATTTCCAGCTACCATTCTCCATAACATCATTTATGGTCTCCCAGAATCTTCT
CCTTGTGCTAGTCTTCCATCAGCTATTGCATCAGCAAAAGATGCTGGGATCCATGAATTTATCACATCGCTTCCACAAGGTTATGATACTAT

>Bcin05g07690(MLST2), partial sequence [organism=Botrytis cinerea, strain G09_S04]
ACCACTATCACCAAGTCCTTTTCTGCACTTTCCTCATCAAAAACGAACGAGAACATCATCAATAAATACTTTGCTGAAGTAATTGGGTTTTATT
TTGGCCAGGATGCCTTCTCTCTACGACAGCAAGCCTTCGATGATATGTTGTGGGTAGTTCTTGGCTGGCTGGATACTGTCAAATTCATTGATTT
ACATTCTGAATTGCACTATTCAAACGACTCTCAGCCAGAATGGTACGGACAACAATATAAACCTGCATTTGCACATCGAGCGCGACTATTTTGG
GAATTGGCTTCACAAGGATGGGATACTACTCTCTGTGGTGGTGGGATGATATGGTCACCATACCTTACTCCATACAAGAACGCAATTACCAATG
AACTCTATATCGCAGCTTCGATATCGATGTACCTATATTTCCCCGGAGATGACAATCAATCCCCATTTATGCTTTCCAACCCTTCATATCCACC
TCACGATCCGAAATATCTACAGGCAGCTGTTGATGCTTACAAATGGCTGAATGGTTCCAACATGACGGATTTACAAGGATTATATGTCGACGGG
TACCATATCTCGAATCTTTCTGGCGGTGAAAACACCCATTGCGATTCTAGAAATGAGATGGTATATACCTACAATCAAGGTGTTTTGCTTACTG
GACAACGTGGTTTGTATGACGCAACCGCCGCACGATCATACCTTGTAGATGGCCACAAACTCATCGCGAATGTTATTAATGCCACAGGCTATGA
CCTGAAACACAATGTTGTCATCTCACCGCCACCCAAAGATGGTTCCGCATTGGCAAAGTGGTTTGGCCTGGGTAGGAATGGAATACTGGAAGAA
GGATGCGATTCAAGTGCTTCGTGTTCTCAAAATGGACAAACTTTCAAAGGCATATTCTTTCATCACTTGATTGCGTTCTGTAGTGATTTGCCAG
GGGAGCCTATTGCAGGGACGAAGGAAAGCTTAGAACTCGACAGAGTGTGGCATTCTGACAAATGCTCACAGTATACAAAAT

>Bcin06g01710(MLST3), partial sequence [organism=Botrytis cinerea, strain G09_S04]
GTGAGTCTGACTTTTGTATTTGAGCGTTAAGATAGACACTGATATACCAAGGCAATCACCTAATTTCATGGCGTTCTTCCCTGCATCATCTACC
TACCCTGGAATGCTGGGAGAATTATACTCAGCAGCTTTCACAGCTCCTGCTTTCAATTGGATCTGTTCCCCTGCTGTGACAGAATTGGAGACGG

TTGTAATGGATTGGCTGGCCAAGCTTCTCAATCTCCCAGACTGTTATTTGTCTTCGACTCATGGTGGTGGTGTTATCCAAGGATCAGCCTCGGA
AGCTATCGTTACCGTTATGGTTGCTGCCCGCGATAAATATCTTCGTGAAACCACTGAAGGTCTGTCGGGCATTGAACTCGAGGATGCGATTGCA
TATAAGAGGAGTAAGCTAGTTGCACTAGGAAGCGAAATGGCACACAGCTCCACGCAAAAAGCAGCGCAGATAGCTGGCGTTAGATTCCGATCGA
TTCCAGTACTCGCATCCAATGATTTCGCCATGACGGGTGATGATTTAGAGAAGGTATTGAAAGAATGCAAATCTCAAGGATTGGAACCCTTCTA
TCTAACTTCGACTTTGGGAACAACATCTACATGCGCAGTTGACGACTTCGCATCTATTGCAACAGTACTTTCAAAATATGCACCTCCAGATGTT
GCAGGCGAGATCTGGGTTCACGTCGATGCTGCTTATGCAGGTGCAGCTTTGGTTTGCCCTGAATACCATCATCTAACATCGTCCTTCCAGCATT
TCCATTCCTTCGATATGAACATGCACAAATGGCTTCTGACAAATTTCGACGCTTCTTGTCTATATGTCAAGAAACGCAAAGATCTGATCGATGC
ACTCTCCATAACACCAAGTTATCTTCGCAACGAGTTTTCAGAGAGTGGACTCGTAACCGACTATCGGGACTGGCAAATTCCTCTCGGAAGACGC
TTCCGAAGCTTAAAGATTTGGTTTGTCCTCAGAACCTAC

>Bcin09g03030(MLST4), partial sequence [organism=Botrytis cinerea, strain G09_S04]
GACCTTCAACCCACCGCAGCTCCTATACGAAAGCGAGTATCCTACCAATTCTCCTTCCACCTGCGACTTTAAGACCATTGGCTTTCCGCACTTT
CACAAAGAAGCATAGTTTAACATTGACGTCGTCGGCATTACAAGTGTTGGCTACTTTTATTGGAAAGCATTGTGGACAGGATGGAGGGAAGAAG
GACTGGCAGAGAGAGTCTTGGAGGAGGTCGCCAAGAGTTGGAAGAATAGGAGTGGCGGTGTCATTGTCGAGGGCGAGGGAACGGAATTGAAGGA
GATTCTGAAAGCTTTGGAAGGGAATATGAGTGGTGGAAGGATAGTCATAGGAAGAGAGCTAAGCCGGCAGAATAGTTTAGTACTGGGATCATCA
CAATATGGAGAGGTCAATCATACAAGACTTGGGCTACGGCCAGGGAATATACCCAGAGAGGATAGTCAGTCAAGTTTGGGAATGTCAACGTTGG
AGGTCAATGACGAGGAAGATGAGGATGGCCTGATGGATCCAAGAAGGTGGTTAAAAGTCATTGATGCATTTGAGCAACCTCGACTGGTGTACAA
TGTTGCTAAAAAGCACTTTGATAGGTATGTTTCAATGATAGGATTTTATCTGAATCGACTAACTCAGTACAGAGATACCTCCAAACCTTCATTG
TTCCCACCTGCGTCTCATAAAACACTCCTCTTCCAAAACCGCTATAATGTTATCCATCAACGTCTCCTTCGCAATGAATCTTTTCAAACGCCCG
CTTTTCAAGGTGGCAAATCTTCCCTTCAACGCAGCACGTCCGCCATTACCACCCAACAACAATCATACAAATTAACGCCGATAGCTAATCTTCT
CGGTCGCAATCGCAGCTCTCATATGCTTCTCGGTCTCCTCAGTATTTCACCCACTGGTACCCTCGCCATCAATGACCTGACGGGCAGTATCGCT
CTCGATCTTACACACGCAGCAGCCATTCC

>Bcin11g01310(MLST5), partial sequence [organism=Botrytis cinerea, strain G09_S04]
ACTGACATGGACCTCATGTGGAACCGGCGTAGAATGCGCAACGCTTGAAGTTCCGCTCGAATATGGCGATGCAACGTCAACGGCAAAAGCCAGT
GTTGCGCTTGCTCGTTATCCTGCCACTGTTGCCGCGAGCAAGAAGCTCGGGTCTCTCTTGATAAACCCCGGTGGACCCGGTGCCTCTGGTGTTG
GCTTTGTGCAGTCTGGAGCCGGTGCCGCCGTCTCGACACTGAGTGGTGGATTGTACGATATCATCGGATGGGATCCACGTGGAACCGGTGCTTC
GGCTCCTATTTTGGAATGTTTTGCAAATGCCAGTGCGGAGTATGATTTTAACAACGCGTTTCCATCTGCTCCGAATCTCTGGCTCGGACAATTT
GCGAATGCCAGCGCAAATTCTGCTGTTAGCTCTGCTATCACATCCTTTGACACTTCTGTCGCTGCTCTTGCAAAAGCTTGCGTGGCTCAGAAAT
CTCCCGCTCTTTACACCCCAACAGCAGCATATGTTGCTCGAGACATGGCAGCGATAGTCGATGCATCGGATGGACCTCTGCAAAACTTAACTA
CTGGGGTTTCTCATATGGAACTATTTTCCTAGCTGAGTTTATCCAAACTTTCCCAGGCCGCGTGGGAAGAGTTCTTGCCGATGGTGTTTTCGAC
GCAAAGGCAAATGCACTCACATACGTTAGCCAACTTCCCAACGATCAACTCAGTGTTCGTGCTTCGTTGAACGATTTTGCAGCTTTCTGCACCA
CCGCCGGTAGTAAAGGTTGCTCTTTTGCCACCGCCCCTACTGGAACCTCAGGTACTGTTGCTACCAGACTGGACAACATAATGAAGGATATGTT
CCTCAATCCTATTGTTGCTTCGGGCTTAAGCATCA

>Bcin15g03910(MLST6), partial sequence [organism=Botrytis cinerea, strain G09_S04]
CGCCAAAACACAAAATCATCCAACGATGAAGATGATACTCCACTTCCCTTGATTATCTGGCATGGACTCGGCGATAATTACAAAGCGGATGGTC
TTGCGCAAGTTGGAAAACTAGCTGAAGCTATTCATCCTGGGACTTTTGTCTACAATATTCATGTAGATGAGGATGCATCTGCAGATAGGACAGC
TACCTTCTTTGGAAATCTCACTCGTGAGTACATCCCCTATTTTTCCTTTAAATATCATACTAACTCTCTTACCAAGTTCAAATCGAAAAGGTCT
GCGAAGACCTCGCCTCCCATCCTATTCTCTCTACCGCGCCCGCCGTCGACGCAATTGGATTCTCCCAAGGCGGCCAATTCTTGCGTGGTTACAT
ATCCCGCTGCAATGCTCCACCCATCCGCTCTCTCCTGACCTTCGGTTCCCAACACAACGGCATTTCTGCCTTCCAAGCCTGTGGTCCTGCCGAT
TTCCTCTGTCGCGGTGCTCAAACCCTTTTGCGATCCAACACCTGGTCAACCTTTGTCCAATCTCGTCTCGTACCCGCTCAATACTTCAGAGATC
CGGAAAACCTAGACTCTTACCTTGAATATTCCAATTTCCTTGCCGACATCAATAATGAGCGCGTTCTCAAGAACCAAACATATAAATCCAACAT
GGAAAAATTGGAACGATTCGTAATGTATGTCTTTGAAGACGATACAACTGTCATCCCTAAGGAAAGTGGATGGTGGGCTGAAGTCAACGGCACG
GAAGTTACACCACTGAAAGAAAGAGCCATTTATAAAGAAGATTGGTTAGGTTTAAAGACATTGGATGAGGCCGGAAAATTAGTTTTCGAAACCA
TTCCAGGGGGACATATGACGTTAGGAGAGGAGATGCTAGAGAAGGCTTTCAAAGAGTATTTTGGTCCAGCAGGGAAGAAATTTGGGGA

>Bcin16g03460(MLST7), partial sequence [organism=Botrytis cinerea, strain G09_S04]
ATGAACCTCTTAATTTGAACTCTTCATTATTTGCAAGTGATGAACCCCTATTCTGCCCAGAGGATAGTTACAAGACGTATATCATTAGTCGAGA
ACCACTCATGATATATATTGACGGATTTTTGAAAGCGAATGAAAGTAAACATTTGGTTGATGTTAGGTGTGTTATTTATTCTGATGAAATGAAC
AAGAGAGACTGATGAGATAGTGAACCGCTTTATGAACCGTCTACTGTTTCTCACGGGCAGGAAGTTACCATTGATACTTCAGTTCGAAATTCTG
AAGTGGCGGTTTTAGAGAGGGATGAGGTGGTCAGGTGTATTGAGCATAGAGCGAGGGCATTTCAGGGGTGGAGGGGCGAGATGGGGATTGAGAA
GTTGAGGACGCAGAGGTATGGGGTTGGAGGACATTATGGGATGCATTTGTAAGTTTTGGGGGATTGACGAAGGCTTCTGTCTATTTTCTACCAG
TACGGATCTTGAAAGAAAGATGGCATGAACACGAGGGCTAATAACTAGGAAATAGCGATTGGAGCGGAGGTAAACGTGGCATAGACCGATTTAG
TACTTTCATGGTCTATGTCGACGTATCCTCTGATATCGAAGGTGGAGGAACGGAATTCCCACGTATTGTGGGACCAAAAGGAGGAAGGTGGGAG
GACTTCCTGGAAACTACGGAAGCATTGGATCCAAGAACTGGAGAAAATGTAACAGTAGAAGGGGTGACATTCAAACCAATCAAGGGAAATGCCG
TATTCTGGGAAAATACTGACAACAACGGGAGGGGCTATGATGA

>Bcin12g03020(MLST8), partial sequence [organism=Botrytis cinerea, strain G09_S04]
CGATTGGCTGCGAAGAAAACTGCGCAGCACCGAGTCACCAAAAGCACGAATAAACCATCGGCAAGGGAAATATTCTCTGAACCACAACATGACA
CGAGCGCGGAAGAGTATATCGGGCGAGAAGCTTCATCAAGAGCACCAAAGCGACAACGAGTCGATGATAATTATAACTCTTACGGTGGAAGAAA
TGAAAATACAGCAGCTTATGCTTCCGGGAAACTTCCATCTGGAAGTATAAATGTTGGTGGAGGTAGGAAGACCACTTTTCAAGAAGAACCACGA
ACGGCATTTGTCGCTGGCAAGTTGCCCCCTGGAAGTATCAACATTGGTGGAAAGAAGGCTACCCAAATCGAGGACGAGGGTAGAGCAGCTTATG
CTTCCGGAAAATTGCCTCCAGGAAGTATAAACTCTGGCGCAAAGAAGGCGATTTCATTCCAAGATGAAACGAGACCGGCTTATGTCTCCGGAAA
GCTTCCACATGGTAGTATCGACGGTATGCGAAACCGTGAAATGGCTGCCGTCCACCGCGAAATTGCTGAGGGTGGAGGAAACCAGGCCAGGTT
GTCTCTTCTCTATTCACATTCAATCCTACTTCAAAGAAAACTTTTGATGAACCCGAAGAACAAGCGGAACCGGCAAAACCATCCAATGCGCCTT
TGACAGAGGAAATGGCGACTTTCACCAATCTAGGGTTATCGAGAAGGCTTGCAGCCCATCTATCGACTAAACTCGATATGAAAGCTCCGACCGC
CATTCAAAAAGCATCTGTACAGCAGTTGGTATCGGACGATAGCGATGCTTTCATACAAGCAGAGACTGGATCTGGAAAAACTTTGGCATATCTA
CTACCTATAGTCGAGCGAATATTAGCATTGAGTGAGAATGGCGTACAAA

>Bcin02g07770(MLST9), partial sequence [organism=Botrytis cinerea, strain G09_S04]
AAAATCATTACAGCTTTCCCTTTCGGTCTTGGCATCTACAGTCATTGCCATCCCTACACCATCACAACTTGAGTCTCGGGCCGTTATCGATTCC
GATGCCGTTGTAGGATTTGCCGAAACTGTTCCCAGTGGGACCGTAGGAACAGTTTATGAGGCATATAAACCATTCCTTAAAGTCGTAAATGGAT
GCGTACCATTCCCTGCCGTCGATGCATCGGGTAACACAGGGTATGTCCTTATACCTTTCTCTTCCACACGATTGCTATTGAGTCTCTAACATAT
TTTAGTGGTGGTTTGTCACCAACTGGCAGTAGCAATGGTGGTTGCAGCAGCAGTACCGGTCAAGTATATGTTCGAGGAGGACAAAGCGGATCAA
ACTACGCCATCATGTACTCCTGGTAAGTTCTCTCTAAACTTCTCCTTATAGATCCAACCTAACAAAATCTTAGGTACATGCCAAAGGACGAGCC
TTCAACCGGTATTGGTCACCGTCACGATTGGGAAGGTGTAATTGTCTGGCTCTCCAGCGCCACCGCCACAACTGCCGACAACATCTTAGCCGTT
TGTCCTTCCGCCCACGGAGGCTGGGATTGTTCCACGGATGGCTATTCCCTTTCTGGTACCAGCCCTCTTATCAAGTACGAAAGTATCTGGCCCG
TCGATCACTCAATGGGTCTTACTAGTACTGTTGGTGGAAAACAACCTATGATTGCTTGGGAGTCTTTACCAACTGCTGCTCAAACTGCTCTTGA
GAACACCGATTTCGGTGCTGCGAATGTTCCATTCATTCCGGCTGTTTTCACAGACAATCTT

>Bcin04g02090(MLST10), partial sequence [organism=Botrytis cinerea, strain G09_S04]
CGGAGGATGATATGGCAAAGTCTATGATTACCAAAGCATTTGTAGGCATGAGTAGTAAACTGTCGAATATGACTATGAATGATGTTTACAAGCC
CTACATCCATGTAAGAAATGTAGAATAGAGAGATCAGTAACTGGAACTAATATCGTTTGTAGGCTTTCAAGTTACTTACGCAGTTCAACCCAAT
CACTACAGCTATTGCCGAATCCCCACTATTTCAAATGGCTGTCTCAGCAAATACCATCGAAAAGTACACACTGCTAGGCCCTTTCTTCAGAATA
TCTCCTCTGCAACAGGAAGTTACCAGGGAATACTTCAGTGCGCCAAAGACGATAGATAGGCGACACATTGCCACATCTCAAGATGCCTTACGAT
TGACCTTACAAACCCATCAAAAAGATTTACTTGATATCATCAACCACTTTGTTCGAGCAAGTCCAATCGCAAAAAGCAAAACCCTGGATTGGTT
CGCCTACATTGTGAATCAAAATCACAAGCGTCGAGCACTTCAGGTAGACCCGAAAGAAGTGTCTTCTGATGGCTTTATGCACAATGTCACTGTC
GTTCTAGATGGTCTTTGTGAGCCATTCATGGATACCACATTCTCGAAAATTTCGAAGATTGATATTGATTATCTAAGACGTGCGCCTCGTGTAG
ATATCAAGGACGAGACCAAGTTGAACGCTGACGAGAAGGCTTCTGAGAAGTATTATGAGGACACTGTTCCTGGCACTTCTAATTTCATCTCTGA
GGTCTTCTTTCTCACATTGGCTGCTCATCATTATGGTAGTGAAGCTCTTAATGCCACGCATAAGAGTCTGGAGAAAGACATCAAATATATTCAA
AAGCAATTGACTGCCGTTGAAGCA

>Bcin01g07220(MLST1), partial sequence [organism=Botrytis cinerea, strain G11_MG1_E22]
ATGGCATATCTTGTTTCTTTCGCATTATGCTTTGGAACGTGCTGGACAAGCTTGGGTTAGTGCTCTGCGTGTAGAAGCACTAAAGAAGATTCTC
GCACAACCGAAGTCATGGTTTGAGGAATCCAGGAATTCACCTGGCCGGTTGAACGAAGTTTTGGATAGGAACTCTGAGGAAATGCGTAATCTCG
TTGGCCGCTTTGCTGGTATTGTATTCACAGCATTTTTTATGCTATTGATATCAATCATTTGGGCTTTCGTGAATACATGGAAACTGACATTAGT
CTCGATGGCAACTGGGCCAGTTATATACGCTGTCACCAAAACGTTCAATCGCGTGAGTGGAAAATGGGAAAACAAGTGCAACTACGCCTCTGAA
ATGACCACTGGCATATTTTCAGAGACTTTCTCCAACATCAAAGTGGTTCGGGTTTTACTCTGGAAACTTACTTTGAGACAAAACACACCAAAGC
TACAGAAGAACTTTATAAAGTTGGACTAATACGAGCAAACTACTCGGGATTGCTGTGGGATTGACAGATGCGATGTCATTCTTTATCACTGCAA
CTATCTTTTATTATGCCACGGTTCTCATTACCAAGAGAGAGAGATCAGTATCGCGACTGCACTACAGACTGTCAATCTTCTATTATTTGGTATTTC
TAATAGTACGAATATGCTGGCCATGATACCACAAATCAACTCTTCTCGCGTTACAGCTACGCATATGCTTGCATTAGCCAATCTCGATTCATCT
TCCTCCCACGAAAATAAAGGAACCGAACGGCTTTCGACAATCTTTCCAATCAAATTCAACCGTCTTTCATTCACATACCCTACTCGTCCTGAAA
AACGAACGATATCATCCTTTTCTCTTTCCCTGATTCCTAACTCAACAACTGCACTTGTCGGACCCTCCGCTCCGGAAAATCTACAATAGCTGCT
CTGCTCATTGGTCTCTATCCGCCAGATACTTCAACACCTCCACCGTTGACATTCAATCGCGTCTCCATAAGTAACTGTCACATTCCGTCTCTCC
GGGCTTCTCTCTCACTCGTTCCACAAATACCGATTCTATTTCCAGCTACCATTCTCCATAACATCATTTATGGTCTCCCAGAATCTTCTCCTTG
TGCTAGTCTTCCATCTGCTATTGCATCAGCAAAAGATGCTGGGATCCATGAATTTATCACATCGCTTCCGCAAGGTTATGATACTATG

>Bcin05g07690(MLST2), partial sequence [organism=Botrytis cinerea, strain G11_MG1_E22]
ACCACTATCACCAAGTCCTTTTCTGCACTTTCCTCATCAAAAACGAACGAGAACATCATCAATAAATACTTTGCTGAAGTAATTGGGTTTTATT
TTGGCCAGGATGCCTTCTCTCTACGACAGCAAGCCTTCGATGATATGTTGTGGGTAGTTCTTGGCTGGCTGGATACTGTCAAATTCATTGATTT
ACATTCTGAATTGCACTATTCAAACGACTCTCAGCCAGAATGGTACGGACAACAATATAAACCTGCATTTGCACATCGAGCGCGACTATTTTGG
GAATTGGCTTCACAAGGATGGGATACTACTCTCTGTGGTGGTGGGATGATATGGTCACCATACCTTACTCCATACAAGAACGCAATTACCAATG
AACTCTATATCGCAGCTTCGATATCGATGTACCTATATTTCCCCGGAGATGACAATCAATCCCCATTTATGCTTTCCAACCCTTCATATCCACC

TCACAATCCGAAATATCTACAGGCAGCTGTTGATGCTTACAAATGGCTGAATGGTTCCAACATGACGGATTTACAAGGATTATATGTCGACGGG
TACCATATCTCGAATCTTTCTGGCGGTGAAAACACCCATTGCGATTCTAGAAATGAGATGGTATATACCTACAATCAAGGTGTTTTGCTTACTG
GACAACGTGGTTTGTATGACGCAACCGCCGCACGATCATACCTTGTAGATGGCCACAAACTCATCGCGAATGTTATTAATGCCACAGGCTATGA
CCTGAAACACAATGTTGTCATCTCACCGCCACCCAAAGATGGTTCCGCATTGGCAAAGTGGTTTGGCCTGGGTAGGAATGGAATACTGGAAGAA
GGATGCGATTCAAGTGCTTCGTGTTCTCAAAATGGACAAACTTTCAAAGGCATATTCTTTCATCACTTGATTGCGTTCTGTAGTGATTTGCCAG
GGGAGCCTATTGCAGGGACGAAGGAAAGCTTAGAACTCGACAGAGTGTGGCATTCTGACAAATGCTCACAGTATACAAAATG

>Bcin06g01710(MLST3), partial sequence [organism=Botrytis cinerea, strain G11_MG1_E22]
GTGAGTCTGACTTTTGTATTTGAGCGTTAAGATAGACACTGATATACCAAGGCAATCACCTAATTTCATGGCGTTCTTCCCTGCATCATCTACC
TACCCTGGAATGCTGGGAGAATTATACTCAGCAGCTTTCACAGCTCCTGCTTTCAATTGGATCTGTTCCCCTGCCGTGACAGAATTGGAGACGG
TTGTAATGGATTGGCTGGCCAAGCTTCTCAATCTCCCAGACTGTTATTTGTCTTCGACTCATGGTGGTGGTGTTATCCAAGGATCAGCCTCGGA
AGCTATCGTTACCGTTATGGTTGCTGCCCGCGATAAATATCTTCGTGAAACTACTGAAGGTCTGTCGGGCATTGAACTCGAGGATGCGATTGCA
TATAAGAGGAGTAAGCTAGTTGCACTAGGAAGCGAAATGGCACACAGCTCCACGCAGAAAGCAGCGCAGATAGCTGGCGTTAGATTCCGATCGA
TTCCAGTACTCGCATCCAATGATTTCGCCATGACGGGTGATGATTTAGAGAAGGTATTGGAAGAATGCAAATCTCAAGGATTGGAACCCTTCTA
TCTAACTTCGACTTTGGGAACAACATCTACATGCGCAGTTGACGACTTCGCATCTATTGCAACAGTACTTTCAAAATATGCACCTCCAGATGTT
GCAGGCGAGATCTGGGTTCACGTCGATGCTGCTTATGCAGGTGCAGCTTTGGTTTGCCCTGAATACCATCATCTAACATCGTCCTTCCAGCATT
TCCATTCCTTCGATATGAACATGCACAAATGGCTTCTGACAAATTTCGACGCTTCTTGTCTATATGTCAAGAAACGCAAAGATCTGATCGATGC
ACTCTCCATAACACCAAGTTATCTTCGCAACGAGTTTTCAGAGAGTGGACTCGTAACCGACTATCGGGACTGGCAAATTCCTCTCGGAAGACGC
TTCCGAAGCTTAAAGATTTGGTTTGTCCTCAGAACCTAC

>Bcin09g03030(MLST4), partial sequence [organism=Botrytis cinerea, strain G11_MG1_E22]
GACCCTTCAACCCCACCGCAGCTCCTATACCGAAAGCGAGTATCCTACCAATTCTCCTTCCACCTGCGACTTTAAGACCATTGGCTTTCCGCAC
TTTCACAAAAAAGCATAGTTTAACATTGACGTCGTCGGCATTACAAGTGTTGGCTACTTTTATTGGAAAGCATTGTGGGACAGGATGGAGGGAA
GAAGGACTGGCAGAGAGAGTCTTGGAGGAGGTCGCCAAGAGTTGGAAGAATAGGAGTGGCGGTGTCATTGTCGAGGGCGAGGGAACGGAATTGA
AGGAGATTCTGAAAGCTTTGGAAGGGAATATGAGTGGTGGAAGGATAGTCATAGGAAGAGAGCTAAGCCGGCAGAATAGTTTAGTACTGGGATC
ATCACAATATGGAGAGGTCAATCATACAAGACTTGGGCTACGGCCAGGGAATATACCCAGAGAGGATAGTCAGTCAAGTTTGGGAATGTCAACG
TTGGAGGTCAATGACGAGGAAGATGAGGATGGCCTGATGGATCCAAGAAGGTGGTTAAAAGTCATTGATGCATTTGAGCAACCTCGACTGGTGT
ACAATGTTGCTAAAAAGCACTTTGATAGGTATGTTTCAATGATAAAATTTTATCTGAATCGACTAACTCAGTACAGAGATACCTCCAAACCTTC
ATTGTTCCCACCTGCGTCTCATAAAACACTCCTCTTCCAAAACCGCTATAATGTTATCCATCAACGTCTCCTTCGCAATGAATCTTTTCAAACG
CCCGCTTTTCAAGGTGGCAAATCTTCCCTTCAACGCAGCACGTCCGCCATTACCACCCAACAACAATCATACAAATTAACGCCGATAGCTAATC
TTCTCGGTCGCAATCGCAGCTCTCATATGCTTCTCGGTCTCCTCAGTATTCACCCACTGGTACCCTCGCCATCAATGACCTGACGGGCAGTATC
GCTCTCGATCTTACACACGCAGCAGCCATTC

>Bcin11g01310(MLST5), partial sequence [organism=Botrytis cinerea, strain G11_MG1_E22]
GCTGACATGGACCTCATGTGGAACCGGCGTAGAATGCGCAACGCTTGAAGTTCCGCTCGAATATGGCGATGCAACGTCAACGGCAAAAGCCAGT
GTTGCGCTTGCTCGTTATCCTGCCACTGTTGCCGCGAGCAAGAAGCTCGGGTCTCTCTTGATAAATCCCGGTGGACCCGGTGCCTCTGGTGTTG
GCTTTGTGCAGTCTGGAGCCGGTGCCGCCGTCTCGACACTGAGTGGTGGATTGTACGATATCATCGGATGGGATCCACGTGGAACCGGTGCTTC
GGCTCCTATTTTGGAATGTTTTGCAAATGCCAGTGCGGAGTATGATTTTAACAACGCGTTTCCATCTGCTCCGAATCTCTGGCTCGGACAATTT
GCGAATGCCAGCGCAAATTCTGCTGTTAGCTCTGCTATCACATCCTTTGACACTTCTGTCGCTGCTCTTGCAAAAGCTTGCGTGGCTCAGAAAT
CTCCCGCTCTTTACACCTCAACAGCAGCATATGTTGCTCGAGACATGGCAGCGATAGTCGATGCATTGGATGGGACCTCTGCAAAACTTAACTA
CTGGGGCTTCTCATATGGAACTATCTTCCTAGCTGAGTTTATCCAAACTTTCCCAGGCCGCGTGGGAAGAGTTCTTGCCGATGGTGTTTTCGAC
GCAAAGGCAAATGCACTCACATACGTTAGCCAACTTCCCAACGATCAACTCAGTGTTCGTGCTTCGTTGAACGATTTTGCAGCTTTCTGCACCA
CCGCCGGTAGTAAAGGTTGCTCTTTTGCCACCGCCCCTACTGGAACCTCAGGTACTGTTGCTACCAGACTGGACAACATAATGAAGGATATGTT
CCTCAATCCTATTGTTGCTTCGGGCTTAAGCATCA

>Bcin15g03910(MLST6), partial sequence [organism=Botrytis cinerea, strain G11_MG1_E22]
GCCAAAACACAAAATCATCCAACGATGAAGATGATACTCCACTTCCCTTGATTATCTGGCATGGACTCGGCGATAATTACAAAGCGGATGGTCT
TGCGCAAGTTGGAAAACTAGCTGAAGCTATTCATCCTGGGACTTTTGTCTACAATATTCATGTAGATGAGGATGCATCTGCAGATAGGACAGCT
ACCTTCTTTGGAAATCTCACTCGTGAGTACATCCCCTATTTTTCCTTTAAATATCATACTAACTCTCTTACCAAGTTCAAATCGAAAAGGTCTG
CGAAGACCTCGCCTCCCATCCTATTCTCTCTACCGCGCCCGCCGTCGACGCAATTGGATTCTCCCAAGGCGGCCAATTCTTGCGTGGTTACATA
TCCCGCTGCAATGCTCCACCCATCCGCTCTCTCCTGACCTTCGGTTCCCAACACAACGGCATTTCTGCCTTCCAAGCCTGTGGTCCTGCCGATT
TCCTCTGTCGCGGTGCTCAAACCCTTTTGCGATCCAACACCTGGTCAACCTTTGTCCAATCTCGTCTCGTACCCGCTCAATACTTCAGAGATCC
GGAAAACCTAGACTCTTACCTTGGATATTCCAATTTCCTTGCCGACATCAATAATGAGCGCGTTCTCAAGAACCAAACATATAAATCCAACATG
GAAAAATTGGAACGATTCGTAATGTATGTCTTTGAAGACGATACAACTGTCATCCCTAAGGAAAGTGGATGGTGGGCTGAAGTCAACGGCACGG
AAGTTACACCACTGAAAGAAAGAGCCATTTATAAAGAAGATTGGCTAGGTTTAAAGACATTGGATGAGGCCGGAAAATTAGTTTTCGAAACCAT
TCCAGGGGGACATATGACGTTAGGAGAGGAGATGCTAGAGAAGGCTTTCAAAGAGTATTTTGGTCCAGCAGGGAAGAAATTTGGG

```
>Bcin16g03460(MLST7), partial sequence [organism=Botrytis cinerea, strain G11_MG1_E22]
ATGAACCTCTTGATTTGAACTCTTCATTAATTGCAAGTGATGAACCCCTATTCTGCCCAGAGGATAGTTACAAGACGTATATCATTAGTCGAGA
ACCACTCATGATATATATTGACGGATTTTTGAAAGCGAATGAAAGTAAACATTTGGTTGATGTTAGGTGTGTTATTCATTCTGATGAAATGAAC
AAGAGAGACTGATGAGATAGTGAACCGCTTTATGAACCATCTACTGTTTCTCACGGACAGGAAGTTACCATTGATCCTTCAGTTCGGAATTCTG
AAGTGGCGGTTTTAGAGAGGGATGAGGTGGTCAGGTGTATTGAGCATAGAGCGAGGGCATTTCAGGGGTGGAGGGGCGAGATGGGGATTGAGAA
GTTGAGGACGCAGAGGTATGGGGTTGGAGGACATTATGGGATGCATTTGTAAGTTTTGGGGGATTGACGAAGGCTTTTGTCTATTTTCTACCAG
TACGGATCTTGAAAGAAAGATAGCATGAACACGAGGGCTAATAACTAGGAAATAGCGATTGGAGCGGAGGTAAACGTGGCATAGACCGATTTAG
TACTTTCATGGTCTATGTCGACGTATCCTCTGATATCGAAGGTGGAGGAACGGAATTCCCACGTATCGTGGGACCAAAAGGAGGAAGGTGGGAG
GACTTCCTGGAAACTACGGAAGCATTGGATCCAAGAACTGGAGAAAATGTAACAGTAGAAGGGGTGACATTCAAACCAATCAAGGGAAATGCCG
TATTCTGGGAAAATACTGACAACAACGGGAGGGGCTATGAT

>Bcin12g03020(MLST8), partial sequence [organism=Botrytis cinerea, strain G11_MG1_E22]
CGATTGGCTGCGAAGAAAACTGCGCAGCACCGAGTCACCAAAAGCACGAATAAACCATCGGCAAGGGAAATATTCTCTGAACCACAACATGACA
CGAGCGCGGAAGAGTATATCGGGCGAGAAGCTTCATCAAGAGCACCAAAGCGACAACGAGTCGATGATAATTATAACTCTTACGGTGGAAGAAA
TGAAAATACAGCAGCTTATGCTTCCGGGAAACTTCCATCTGGAAGTATAAATGTTGGTGGAGGTAGGAAGACCACTTTTCAAGAAGAACCACGA
ACGGCATTTGTCGCTGGCAAGTTGCCCCCTGGAAGTATCAACATTGGTGGAAAGAAGGCTACCCAAATCGAGGACGAGGGTAGAGCAGCTTATG
CTTCCGGAAAATTGCCTCCAGGAAGTATAAACTCTGGCGCAAAGAAGGCGATTTCATTCCAAGATGGAACGAGACCGGCTTATGTCTCCGGAAA
GCTTCCGCATGGTAGTATCGACGGTATGCGAAACCGTGAAATGGCTGCCGTCCACCGCGAAATTGCTGAGGGTGGAGGAAACCAGGCCAGGTT
GTCTCTTCTCTATTCACATTTAATCCTACTTCAAAGAAAACTTTTGATGAACCCGAAGAACAAGCGGAACCGGCAAAACCATCCAATGCGCCTT
TGACAGAGGAAATGGCGACTTTCACCAATCTAGGGTTATCGAGAAGGCTTGCAGCCCATCTATCGACTAAACTCGGTATGAAAGCTCCGACCGC
CATTCAAAAAGCATCTGTACAGCAGTTGGTATCGGACGATAGCGATGCTTTCATACAAGCAGAGACTGGATCTGGAAAAACTTTGGCATATCTA
CTACCTATAGTCGAGCGAATATTAGCATTGAGTGAGAATGGCGTACAAA

>Bcin02g07770(MLST9), partial sequence [organism=Botrytis cinerea, strain G11_MG1_E22]
CAGCTTTCCCTTTCGGTCTTGGCATCTACAGTCATTGCCATCCCTACACCATCACAACTTGAGTCTCGGGCCGTTATCGATTCCGATGCCGTTG
TAGGATTTGCCGAAACTGTTCCCAGTGGGACCGTAGGAACAGTTTATGAGGCATATAAACCATTCCTTAAAGTCGTAAATGGATGCGTACCATT
CCCTGCCGTCGATGCATCGGGTAACACAGGGTATGTCCTTATACCTTTCTCTTCCACACGATTGCTATTGAGTCTCTGACATATTTTAGTGGTG
GTTTGTCACCAACTGGCAGTAGCAATGGTGGTTGCAGCAGCAGTACCGGTCAAGTATATGTTCGAGGAGGACAAAGCGGATCAAACTACGCCAT
CATGTACTCCTGGTAAGTTCTCTCTAAACTTCTCCTTATAGATCCAACCTAACAAAATCTTAGGTACATGCCAAAGGACGAGCCTTCAACCGGT
ATTGGTCACCGTCACGATTGGGAAGGTGTAATTGTCTGGCTCTCCAGCGCCACCGCCACAACTGCCGACAACATCTTAGCCGTTTGTCCTTCTG
CCCACGGAGGCTGGGATTGTTCCACCGATGGCTATTCCCTTTCTGGTACCAGCCCTCTTATCAAGTACGAAAGTATCTGGCCCGTCGATCATTC
AATGGGTCTTACTAGTACTGTTGGTGGAACACAACCTATGATCGCTTGGGAGTCTTTACCTACTGCTGCTCAAACTGCTCTTGAGAACACCGAT
TTCGGTGCTGCGAATGTTCCATTCATTCCGGCTGTTTTCACAGATAATCT

>Bcin04g02090(MLST10), partial sequence [organism=Botrytis cinerea, strain G11_MG1_E22]
CGGAGGATGATATGGCAAAGTCTATGATTACCAAAGCATTTGTAGGCATGAGTAGTAAACTGTCGAATATGACTATGAATGATGTTTACAAGCC
CTACATCCATGTAAGAAATGTAGAATAGAGAGATCAGTAACTGGAACTAATATCGTTTGTAGGCTTTCAAGTTACTTACGCAGTTCAACCCAAT
CACTACAGCTATTGCCGAATCCCCACTGTTTCAAATGGCCGTCTCAGCAAATACCATCGAAAAGTACACACTGCTAGGCCCCTTTCTTCAGAATA
TCTCCTCTGCAACAGGAAGTTACCAGGGAATACTTCAGTGCGCCAAAGACGATAGATAGGCGACACATTGCCACATCTCAAGATGCGTTACGAT
TGACTCTACAAACCCATCAAAAAGATTTACTTGATATCATCAACCACTTTGTTCGAGCAAGTCCAATCGCAAAAAGCAAAACCCTGGATTGGTT
CGCCTACATTGTGAATCAAAATCACAAGCGTCGAGCACTTCAGGTAGACCCGAAAGAAGTGTCTTCTGATGGCTTTATGCACAATGTCACTGTC
GTTCTAGATGGTCTTTGTGAGCCATTCATGGATACCACATTCTCGAAAATTTCGAAGATTGATATTGATTATCTAAGACGTGCGCCTCGTGTAG
ATATCAAGGACGAGACCAAGTTGAACGCTGACGAGAAGGCTTCTGAGAAGTATTATGAGGACACTGTTCCTGGCACTTCTAATTTCATCTCTGA
GGTCTTCTTTCTCACATTGGCTGCTCATCATTATGGTAGTGAAGCTCTTAATGCCACGCATAAGAGTCTGGAGAAAGACATCAAATATATTCAA
AAGCAATTGACTGCCGTTGAAGCA

>Bcin01g07220(MLST1), partial sequence [organism=Botrytis cinerea, strain G13_EBio04]
ATGGCATATCTTGTTTCTTTTCGCATTATGCTTTGGAACGTGCTGGACAAGCTTGGTTAGTGCTCTGCGTGTAGAAGCACTAAAGAAGATTCTC
GTACAACCGAAGTCATGGTTTGAGGAATCCAGGAATTCACCTAGCCGGTTGAACGAAGTTTTGGATAGGAACTCTGAGGAAATGCGTAATCTCG
TTGGCCGCTTTGCTGGTATTGTATTCACAGCATTTTTTATGCTATTGATATCAATCATTTGGGCTTTCGTGAATACATGGAAACTGACATTAGT
CTCAATGGCAACTGGGCCAGTTATATACGCTGTCACCAAAACGTTCAATCGCGTGAGTGGAAAATGGGAAAACAAGTGCAACTACGCATCTGAA
ATGACCACTGGCATATTTTCAGAGACTTTCTCCAACATCAAAGTGGTTCGGGCTTTTACTCTGGAAACTTACTTTAAGACAAAACACACCAAAG
CTACAGAAGAACTCTATAAAGTTGGACTAATACGAGCAAACTACTCGGATTGCTGTGGGATTGACAGATGCAATGTCATTCTTCATCACTGCAA
CTATCTTTTATTATGCCACGGTTCTCATTACCAAGAGAGAGAGATCAGTATCGCGGCTGCACTACAGACTGTCAATCTTCTATTATTTGGTATTTC
TAATAGTACGAATATGCTGGCCATGATACCACAAATCAACTCTTCTCGCGTTACAACTACGCATATGCTTGCATTAGCCAATCTCGATTCATCT
TCCTCCACGAAAATAAAGGAACCGAACGGCTTTCGACAATCTTTCCAATCAAATTCAACCGTCTTTCATTCACATACCCTACTCGTCCTGAAAA
ACGAACGATATCATCCTTTTCTCTTTCCCTGATTCCTAACTCAACAACTGCACTTGTCGGACCCTCCGGCTCCGGAAAATCTACAATAGCTGCT
```

CTGCTCATTGGTCTCTATCCGCCAGATACTTCAACACCTCCACCGTTGACATTCAATCGCGCCTCCATAAGTAACTGTCACATTCCGTCTCTCC
GGGCTTCTCTCTCACTCGTTCCACAAATACCGATTCTATTTCCAGCTACCATTCTCCATAACATCATTTATGGTCTCCCAGAATCTTCTCCTTG
TGCTAGTCTTCCATCTGCTATTGCATCAGCAAAAGATGCTGGATCCATGAACTTATCACATCGCTTCCACAAGGTTATGATACTAT

>Bcin05g07690(MLST2), partial sequence [organism=Botrytis cinerea, strain G13_EBio04]
ACCACTATCACCAAGTCCTTTTCTGCACTTTCCTCATCAAAAACGAACGAGAACATCATCAATAAATACTTTGCTGAAGTAATTGGGTTTTATT
TTGGCCAGGATGCCTTCTCTCTACGACAGCAAGCCTTCGATGATATGTTGTGGGTAGTTCTTGGCTGGCTGGATACTGTCAAATTCATTGATTT
ACATTCTGAATTGCACTATTCAAACGACTCTCAGCCAGAATGGTACGGACAACAATATAAACCTGCATTTGCACATCGAGCGCGACTATTTTGG
GAATTGGCTTCACAAGGATGGGATACTACTCTCTGTGGTGGTGGGATGATATGGTCACCATACCTTACTCCATACAAGAACGCAATTACCAATG
AACTCTATATCGCAGCTTCGATATCGATGTACCTATATTTCCCCGGAGATGACAATCAATCCCCATTTATGCTTTCCAACCCTTCATATCCACC
TCACGATCCGAAATATCTACAGGCAGCTGTTGATGCTTACAAATGGCTGAATGGTTCCAACATGACGGATTTACAAGGATTATATGTCGACGGG
TACCATATCTCGAATCTTTCTGGCGGTGAAAACACCCATTGCGATTCTAGAAATGAGATGGTATATACCTACAATCAAGGTGTTTTGCTTACTG
GACAACGTGGTTTGTATGACGCAACCGCCGCACGATCATACCTTGTAGATGGCCACAAACTCATCGCGAATGTTATTAATGCCACAGGCTATGA
CCTGAAACACAATGTTGTCATCTCACCGCCACCCAAAGATGGTTCCGCATTGGCAAAGTGGTTTGGCCTGGGTAGGAATGGAATACTGGAAGAA
GGATGCGATTCAAGTGCTTCGTGTTCTCAAAATGGACAAACTTTCAAAGGCATATTCTTTCATCACTTGATTGCGTTCTGTTGTGATTTGCCAG
GGGAGCCTATTGCAGGGACGAAGGAAAGCTTAGAACTCGACAGAGTGTGGCATTCTGACAAATGCTCACAGTATACAAAAT

>Bcin06g01710(MLST3), partial sequence [organism=Botrytis cinerea, strain G13_EBio04]
TTGGTGAGTCTGACTTTTGTATTTGAGCGTTAAGATAGACACTGATATACCAAGGCAATCACCTAATTTCATGGCGTTCTTCCCTGCATCATCT
ACCTACCCTGGAATGCTGGGAGAATTATACTCAGCAGCTTTCACAGCACCTGCTTTCAATTGGATCTGTTCCCCTGCTGTGACAGAATTGGAGA
CGGTTGTAATGGATTGGCTGGCCAAGCTTCTCAATCTCCCAGACTGTTATTTGTCTTCGACTCATGGTGGTGGTGTCATCCAAGGATCAGCCTC
GGAAGCTATCGTTACCGTTATGGTTGCTGCCCGCGATAAATATCTTCGTGAAACTACTGAAGGTCTGTCGGGCATTGAACTCGAGGATGCGATT
GCATATAAGAGGAGTAAGCTAGTTGCACTAGGAAGCGAAATGGCACACAGCTCCACGCAGAAAGCAGCGCAAATAGCTGGCGTTAGATTCCGAT
CGATTCCAGTACTCGCATCCAATGATTTCGCCATGACGGGTGATGATTTAGAGAAGGTATTGAAAGAATGCAAATCTCAAGGATTGGAACCCTT
CTATCTAACTTCGACTTTGGGAACAACATCTACATGCGCAGTTGACGACTTCGCATCTATTGCAACAGTACTTTCAAAATATGCACCTCCAGAT
GTTGCAGGCGAGATCTGGGTTCACGTCGATGCTGCTTATGCAGGTGCAGCTTTGGTTTGCCCTGAATACCATCATCTAACATCGTCTTTCCAGC
ATTTCCATTCCTTCGATATGAACATGCACAAATGGCTTCTGACAAATTTCGACGCTTCTTGTCTATATGTCAAGAAACGCAAAGATCTGATCGA
TGCACTGTCCATAACACCAAGTTATCTTCGCAACGAGTTTTCAGAGAGTGGACTCGTAACCGACTATCGGGACTGGCAAATTCCTCTCGGAAGA
CGCTTCCGAAGCTTAAAGATTTGGTTTGTCCTCAGAACCTAC

>Bcin09g03030(MLST4), partial sequence [organism=Botrytis cinerea, strain G13_EBio04]
ACCCTTCAACCCCACCGCAGCTCCTATACCGAAAGCGAGTATCCTACCAATTCTCCTTCCACCTGCGACTTTAAGACCATTGGCTTTCCGCACT
TTCACAAAAAAGCATAGTTTAACATTGACGTCGTCGGCATTACAAGCGTTGGCTACTTTTATTGGAAAGCATTGTGGGACAGGATGGAGGGAAG
AAGGACTGGCAGAGAGAGTCTTGGAGGAGGTCGCCAAGAGTTGGAAGAATAGGAGTGGCGGTGTCATTGTCGAGGCGAGGGAACGGAATTGAAG
GAGATTCTGAAAGCTTTGGAAGGGAATATGAGTGGTGGAAGGATAGTCATAGGAAGAGAGCTAAGCCGGCAGAATAGTTTAGTACTGGGATCAT
CACAATATGGAGAGGTCAATCATACAAGACTTGGGCTACGGCCAGGGAATATACCCAGAGAGGATAGTCAGTCAAGTTTGGGAATGTCAACGTT
GGAGGTCAATGACGAGGAAGATGAGGATGGCCTGATGGATCCAAGAAGGTGGTTAAAAGTCATTGATGCATTTGAGCAACCTCGACTGGTGTAC
AATGTTGCTAAAAAGCACTTTGATAGGTATGTTTCAATGATAAAATTTTATCTGAATCGACTAACTCAGTACAGAGATACCTCCAAACCTTCAT
TGTTCCCACCTGCGTCTCATAAAACACTCCTCTTCCAAAACCGCTATAATGTTATCCATCAGCGTCTCCTTCGCAATGAATCTTTTCAAACGCC
CGCTTTTCAAGGTGGCAAATCTTCCCTTCAACGCAGCACGTCCGCCATTACCACCCAACAACAATCATACAAATTAACGCCGATAGCTAATCTT
CTCGGTCGCAATCGCAGCTCTCATATGCTTCTCGGTCTCCTCAGTATTTCACCCACTGGTACCCTCGCCATCAATGACCTGACGGGCAGTATCG
CTCTCGATCTTACACACGCAGCAGCCATTC

>Bcin11g01310(MLST5), partial sequence [organism=Botrytis cinerea, strain G13_EBio04]
ACTGACATGGACCTCATGTGGAACCGGCGTAGAATGCGCAACGCTTGAAGTTCCGCTCGAATATGGCGATGCAACGTCAACGGCAAAAGCCAGT
GTTGCGCTTGCTCGTTATCCTGCCACTGTTGCCGCGAGCAAGAAGCTCGGGTCTCTCTTGATAAACCCCGGTGGACCCGGTGCCTCTGGTGTTG
GCTTTGTGCAGTCTGGAGCCGGTGCCGCCGTCTCGACACTGAGTGGTGGATTGTACGATATCATCGGATGGGATCCACGTGGAACCGGTGCTTC
GGCTCCTATTTTGGAATGTTTTGCAAATGCCAGTGCGGAGTATGATTTTAACAACGCGTTTCCATCTGCTCCGAATCTCTGGCTCGGACAATTT
GCGAATGCCAGCGCAAATTCTGCTGTTAGCTCTGCTATCACATCCTTTGACACTTCTGTCGCTGCTCTTGCAAAAGCTTGCGTGGCTCAGAAAT
CTCCCGCTCTTTACACCTCAACAGCAGCATATGTTGCTCGAGACATGGCAGCGATAGTCGATGCATTGGATGGGACCTCTGCAAAACTTAACTA
CTGGGGTTTCTCATATGGAACTATTTTCCTAGCTGAGTTTATCCAAACTTTCCCAGGCCGCGTGGGAAGAGTTCTTGCCGATGGTGTTTTCGAC
GCAAAGGCAAATGCACTCACATACGTTAGCCAACTTCCCAACGATCAACTCAGTGTTCGTGCTTCGTTGAACGATTTTGCAGCTTTCTGCACCA
CCGCCGGTAGTAAAGGTTGCTCTTTTGCCACCGCCCCTACTGGAACCACAGGTACTGTTGCTACCAGACTGGACAACATAATGAAGGATATGTT
CCTCAATCCTATTGTTGCTTCGGGCTTAAGCATCA

>Bcin15g03910(MLST6), partial sequence [organism=Botrytis cinerea, strain G13_EBio04]
GCCAAAACACAAAATCATCCAACGATGAAGATGATACTCCACTTCCCTTGATTATCTGGCATGGACTCGGCGATAATTACAAAGCGGATGGTCT

TGCGCAAGTTGGAAAACTAGCTGAAGCTATTCATCCTGGGACTTTTGTCTACAATATTCATGTAGATGAGGATGCATCTGCAGATAGGACAGCT
ACCTTCTTTGGAAATCTCACTCGTGAGTACATCCCCTATTTTTCCTTTAAATATCATACTAACTCTCTTACCAAGTTCAAATCGAAAAGGTCTG
CGAAGACCTCGCCTCCCATCCTATTCTCTCTACCGCGCCCGCCGTCGACGCAATTGGATTCTCCCAAGGCGGCCAATTCTTGCGTGGTTACATA
TCCCGCTGCAATGCTCCACCCATCCGCTCTCTCCTGACCTTCGGTTCCCAACACAACGGCATTTCTGCCTTCCAAGCCTGTGGTCCTGCCGATT
TCCTCTGTCGCGGTGCTCAAACCCTTTTGCGATCCAACACCTGGTCAACCTTTGTCCAATCTCGTCTCGTACCCGCTCAATACTTCAGAGATCC
GGAAAACCTAGACTCTTACCTTGAATATTCCAATTTCCTTGCCGACATCAATAATGAGCGCGTTCTCAAGAACCAAACATATAAATCCAACATG
GAAAAATTGGAACGATTCGTAATGTATGTCTTTGAAGACGATACAACTGTCATCCCTAAGGAAAGTGGATGGTGGGCTGAAGTCAACGGCACGG
AAGTTACACCACTGAAAGAAAGAGCCATTTATAAAGAAGATTGGCTAGGTTTAAAGACATTGGATGAGGCCGGAAAATTAGTTTTCGAAACCAT
TCCAGGGGACATATGACGTTAGGAGAGGAGATGCTGGAGAAGGCTTTCAAAGAGTATTTTGGTCCAGCAGGGAAGAAATTTGGG

>Bcin16g03460(MLST7), partial sequence [organism=Botrytis cinerea, strain G13_EBio04]
ATGAACCTCTTGATTTGAACTCTTCATTAATTGCAAGTGATGAACCCCTATTCTGCCCAGAGGATAGTTACAAGACGTATATCATTAGTCGAGA
ACCACTCATGATATATATTGACGGATTTTTGAAAGCGAATGAAAGTAAACATTTGGTTGATGTTAGGTGTGTTATTTATTCTGATGAAATGAAC
AAGAGAGACTGATGAGATAGTGAGCCGCTTTATGAACCGTCAACTGTTTCTCACGGACAGGAAGTTACCATTGATCCTTCAGTTCGGAATTCTG
AAGTGGCGGTTTTAGAGAGGGATGAGGTGGTCAGGTGTATTGAGCATAGAGCGAGGGCATTTCAGGGGTGGAGGGGCGAGATGGGGATTGAGAA
GTTGAGGACGCAGAGGTATGGGGTTGGAGGACATTATGGGATGCATTTGTAAGTTTTGGGGGATTGACGAAGGCTTCTGTCTATTTTCTATCAG
CACGGATCTTGAAAGAAAGATAGCATGAACACGCGGGCTAACAACTAGGAAATAGCGATTGGAGCGGAGGTAAACGTGGCATAGACCGATTTAG
TACTTTCATGGTCTATGTCGACGTATCCTCTGATATCGAAGGTGGAGGAACGGAATTCCCACGTATCGTGGGACCAAAAGGAGGAAGGTGGGAG
GACTTCCTGGAAACTACGGAAGCATTGGATCCAAGAACTGGAGAAAATGTAACAGTAGAAGGGGTGACATTCAAACCAATCAAGGGAAATGCCG
TATTCTGGGAAAATACTGACAACAACGGGAGGGGCTATGAT

>Bcin12g03020(MLST8), partial sequence [organism=Botrytis cinerea, strain G13_EBio04]
CGATTGGCTGCGAAGAAAACTGCGCAGCACCGAGTCACCAAAAGCACGAATAAACCATCGGCAAGGGAAATATTCTCTGAACCACAACATGACA
CGAGCGCGGAAGAGTATATCGGGCGAGAAGCTTCATCAAGAGCACCAAAGCGACAACGAGTCGATGATAATTATAACTCTTACGGTGGAAGAAA
TGAAAATACAGCAGCTTATGCTTCCGGGAAACTTCCATCTGGAAGTATAAATGTTGGTGGAGGTAGGAAGACCACTTTTCAAGAAGAACCACGA
ACGGCATTTGTCGCTGGCAAGTTGCCCCCTGGAAGTATCAACATTGGTGGAAAGAAGGCTACCCAAATCGAGGACGAGGGTAGAGCAGCTTATG
CTTCCGGAAAATTGCCTCCAGGAAGTATAAACTCTGGCGCAAAGAAGGCGATTTCATTCCAAGATGAAACGAGACCGGCTTATGTCTCCGGAAA
GCTTCCACATGGTAGTATCGACGGTATGCGAAACCGTGAAATGGCTGCCGTCCACCGCGAAATTGCTGAGGGTGGAGGAAACCAGGCCAGGTT
GTCTCTTCTCTATTCACATTCAATCCTACTTCAAAGAAAACTTTTGATGAACCCGAAGAACAAGCGGAACCGGCAAAACCATCCAATGCGCCTT
TGACAGAGGAAATGGCGACTTTCACCAATCTAGGGTTATCGAGAAGGCTTGCAGCCCATCTATCGACTAAACTCGATATGAAAGCTCCGACCGC
CATTCAAAAAGCATCTGTACAGCAGTTGGTATCGGACGATAGCGATGCTTTCATACAAGCAGAGACTGGATCTGGAAAAACTTTGGCATATCTA
CTACCTATAGTCGAGCGAATATTAGCATTGAGTGAGAATGGCGTACAAA

>Bcin02g07770(MLST9), partial sequence [organism=Botrytis cinerea, strain G13_EBio04]
ACAGCTTTCCCTTTCGGTCTTGGCATCTACAGTCATTGCCATCCCTACACCATCACAACTTGAGTCTCGGGCCGTTATCGATTCCGATGCCGTT
GTAGGATTTGCCGAAACTGTTCCCAGTGGGACCGTAGGAACAGTTTATGAGGCATATAAACCATTCCTTAAAGTCGTAAATGGATGCGTACCAT
TCCCTGCCGTCGATGCATCGGGTAACACAGGGTATGTCCTTATACCTTTCTCTTCCACACGATTGCTATTGAGTCTCTAACATATTTTAGTGGT
GGTTTGTCACCAACTGGCAGTAGCAATGGTGGTTGCAGCAGCAGTACCGGTCAAGTATATGTTCGAGGAGGACAAAGCGGATCAAACTACGCCA
TCATGTACTCCTGGTAAGTTCTCTCTAAACTTCTCCTTATAGATCCAACCTAACAAAATCTTAGGTACATGCCAAAGGACGAGCCTTCAACCGG
TATTGGTCACCGTCACGATTGGGAAGGTGTAATTGTCTGGCTCTCCAGCGCCACCGCCACAACTGCCGACAACATCTTAGCCGTTTGTCCTTCT
GCCCACGGAGGCTGGGATTGTTCCACCGATGGCTATTCCCTTTCTGGTACCAGCCCTCTTATCAAGTACGAAAGTATCTGGCCCGTCGATCATT
CAATGGGTCTTACTAGTACTGTTGGTGGAACACAACCTATGATCGCTTGGGAGTCTTTACCTACTGCTGCTCAAACTGCTCTTGAGAACACCGA
TTTCGGTGCTGCGAATGTTCCATTCATTCCGGCTGTTTTCACAGACAATCT

>Bcin04g02090(MLST10), partial sequence [organism=Botrytis cinerea, strain G13_EBio04]
CGGAGGATGATATGGCAAAGTCTATGATTACCAAAGCATTTGTAGGCATGAGTAGTAAACTGTCGAATATGACTATGAATGATGTTTACAAGCC
CTACATCCATGTAAGAAATGTAGAATAGAGAGATCAGTAACTGGAACTAATATCGTTTGTAGGCTTTCAAGTTACTTACGCAGTTCAACCCAAT
CACTACAGCTATTGCCGAATCCCCACTATTTCAAATGGCTGTCTCAGCAAATACCATCGAAAAGTACACACTGCTAGGCCCTTTCTTCAGAATA
TCTCCTCTGCAACAGGAAGTTACCAGGGAATACTTCAGTGCGCCAAAGACGATAGATAGGCGACACATTGCCACATCTCAAGATGCCTTACGAT
TGACCTTACAAACCCATCAAAAAGATTTACTTGATATCATCAACCACTTTGTTCGAGCAAGTCCAATCGCCAAAAGCAAAACCTTGGATTGGTT
CGCCTACATTGTGAATCAAAATCACAAGCGTCGAGCACTTCAGGTAGACCCGAAAGAAGTGTCTTCTGATGGCTTTATGCACAATGTCACTGTC
GTTCTAGATGGTCTTTGTGAGCCATTCATGGATACCACATTCTCGAAAATTTCGAAGATTGATATTGATTATCTAAGACGTGCGCCTCGTGTAG
ATATCAAGGACGAGACCAAGTTGAACGCTGACGAGAAGGCTTCTGAGAAGTATTATGAGGACACTGTTCCTGGCACTTCTAATTTCATCTCTGA
GGTCTTCTTTCTCACATTGGCTGCTCATCATTATGGTAGTGAAGCTCTTAATGCCACGCATAAGAGTCTGGAGAAAGACATCAAATATATTCAA
AAGCAATTGACTGCCGTTGAAGCA

>Bcin01g07220(MLST1), partial sequence [organism=Botrytis cinerea, strain N11_K_W02]

ATGGCATATCTTGTTTCTTTTCGCATTATGCTTTGGAACGTGCTGGACAAGCTTGGGTTAGTGCTCTGCGTGTAGAAGCACTAAAGAAGATTCT
CGCACAACCGAAGTCATGGTTTGAGGAATCCAGGAATTCACCTGGCCGGTTGAACGAAGTTTTGGATAGGAACTCTGAGGAAATGCGTAATCTC
GTTGGCCGCTTTGCTGGTATTGTATTCACAGCATTTTTTATGCTATTGATATCAATCATTTGGGCTTTCGTGAATACATGGAAACTGACATTAG
TCTCGATGGCAACTGGGCCAGTTATATACGCTGTCACCAAAACGTTCAATCGCGTGAGTGGAAAATGGGAAAACAAGTGCAACTACGCCTCTGA
AATGACCACTGGCATATTTTCAGAGACTTTCTCCAACATCAAAGTGGTTCGGGCTTTTACTCTGGAAACTTACTTTGAGACAAAACACACCAAA
GCTACAGAAGAACTTTATAAAGTTGGACTAATACGAGCAAACTACTCGGATTGCTGTGGGGATTGACAGATGCGATGTCATTCTTCATCACTGC
AACTATCTTTTATTATGCCACGCTTCTCATTACCAAGAGAGAGATCAGTATCGCGACTGCACTACAGACTGCCAATCTTCTATTATTTGGTATT
TCTAATAGTACGAATATGCTGGCCATGATACCACAAATCAACTCTTCTCGCGTTACAGCTACGCATATGCTTGTATTAGCCAATCTCGATTCAT
CTTCCTCCCACGAAAATAAAGGAACCGAACGGCTTTCGACAATCTTTCCAATCAAATTCAACCGTCTCTCATTCACATATCCTACTCGTCCTGA
AAAACGAACGATATCATCCTTTTCTCTTTCCCTGATTCCTAACTCAACAACTGCACTTGTCGGACCCTCCGGCTCCGGAAAATCTACAATAGCT
GCTCTGCTCATTGGTCTTTATCCGCCAGATATTTCAACACCTCCACCGTTGACATTCAATCGCGTCTCCATAAGTAACTGTCACATTCCGTCTC
TCCGGGCTTCTCTCTCACTCGTCCACAAATACCGATTCTATTTCCAGCTACCATTCTCCATAACATCATTTATGGTCTCCCAGAATCTTCTCCT
TGTGCTAGTCTTCCATCTGCTATTGCATCAGCAAAAGATGCTGGGATCCATGAATTTATCACATCGCTTCCACAAGGTTATGATACTATG

>Bcin05g07690(MLST2), partial sequence [organism=Botrytis cinerea, strain N11_K_W02]
ACCACTATCACCAAGTCCTTTTCTGCACTTTCCTCATCAAAAACGAACGAGAACATCATCAATAAATACTTTGCTGAAGTAATTGGGTTTTATT
TTGGCCAGGATGCCTTCTCTCTACGACAGCAAGCCTTCGATGATATGTTGTGGGTAGTTCTTGGCTGGCTGGATACTGTCAAATTCATTGATTT
ACATTCTGAATTGCACTATTCAAACGACTCTCAGCCAGAATGGTACGGACAACAATATAAACCTGCATTTGCACATCGAGCGCGACTATTTTGG
GAATTGGCTTCACAAGGATGGGATACTACTCTCTGTGGTGGTGGGATGATATGGTCACCATACCTTACTCCATACAAGAACGCAATTACCAATG
AACTCTATATCGCAGCTTCGATATCGATGTACCTATATTTCCCCGGAGATGACAATCAATCCCCATTTATGCTTTCCAACCCTTCATATCCACC
TCACGATCCGAAATATCTACAGGCAGCTGTTGATGCTTACAAATGGCTGAATGGTTCCAACATGACGGATTTACAAGGATTATATGTCGACGGG
TACCATATCTCGAATCTTTCTGGCGGTGAAAACACCCATTGCGATTCTAGAAATGAGATGGTATATACCTACAATCAAGGTGTTTTGCTTACTG
GACAACGTGGTTTGTATGACGCAACCGCCGCACGATCATACCTTGTAGATGGCCACAAACTCATCGCGAATGTTATTAATGCCACAGGCTATGA
CCTGAAACACAATGTTGTCATCTCACCGCCACCCAAAGATGGTTCCGCATTGGCAAAGTGGTTTGGCCTGGGTAGGAATGGAATACTGGAAGAA
GGATGCGATTCAAGTGCTTCGTGTTCTCAAAATGGACAAACTTTCAAAGGCATATTCTTTCATCACTTGATTGCGTTCTGTAGTGATTTGCCAG
GGGAGCCTATTGCAGGGACGAAGGAAAGCTTAGAACTCGACAGAGTGTGGCATTCTGACAAATGCTCACAGTATACAAAAT

>Bcin06g01710(MLST3), partial sequence [organism=Botrytis cinerea, strain N11_K_W02]
GTGAGTCTGACTTTTGTATTTGAGCGTTAAGATAGACGCTGATATACCAAGGCAATCACCTAATTTCATGGCGTTCTTCCCTGCATCATCTACC
TACCCTGGAATGCTGGGAGAATTATACTCAGCAGCTTTCACAGCACCTGCTTTCAATTGGATCTGTTCCCTGCTGTGACAGAATTGGAGACGGT
TGTAATGGATTGGCTGGCCAAGCTTCTCAATCTCCCAGACTGTTATTTGTCTTCGACTCATGGTGGTGGTGTCATCCGAGGATCAGCCTCGGAA
GCTATCGTTACCGTTATGGTTGCTGCCCGCGATAAATATCTTCGTGAAACTACTGAAGGTCTGTCGGGCATTGAACTCGAGGATGCGATTGCAT
ATAAGAGGAGTAAGCTAGTTGCACTAGGAAGCGAAATGGCACACAGCTCCACGCAGAAAGCAGCGCAAATAGCTGGCGTTAGATTCCGATCGAT
TCCAGTACTCGCATCCAATGATTTCGCCATGACGGGTGATGATTTAGAGGAGGTATTGAAAGAATGCAAATCTCAAGGATTGGAACCCTTCTAT
CTAACTTCGACTTTGGGAACAACATCTACATGCGCAGTTGACGACTTCGCATCTATTGCAACAGTACTTTCAAAATATGCACCTCCAGATGTTG
CAGGCGAGATCTGGGTTCACGTCGATGCTGCTTATGCAGGTGCAGCTTTGGTTTGCCCTGAATACCATCATCTAACATCGTCTTTCCAGCATTT
CCATTCCTTCGATATGAACATGCACAAATGGCTTCTGACAAATTTCGACGCTTCTTGTCTATATGTCAAGAAACGCAAAGATCTGATCGATGCA
CTGTCCATAACACCAAGTTATCTTCGCAACGAGTTTTCAGAGAGTGGACTCGTAACCGACTATCGGGACTGGCAAATTCCTCTCGGAAGACGCT
TCCGAAGCTTAAAGATTTGGTCTGTCCTCAGAACCTAC

>Bcin09g03030(MLST4), partial sequence [organism=Botrytis cinerea, strain N11_K_W02]
ACCCTTCAACCCCACCGCAGCTCCTATACCGAAAGCGAGTATCCTACCAATTCTCCTTCCACCTGCGACTTTAAGACCATTGGCTTTCCGCACT
TTCACAAAAAAGCATAGTTTAACATTGACGTCGTCGGCATTACAAGTGTTGGCTACTTTTATTGGAAAGCATTGTGGGACAGGATGGAGGGAAG
AAGGACTGGCAGAGAGAGTCTTGGAGGAGGTCGCCAAGAGTTGGAAGAATAGGAGTGGCGGTGTCATTGTCGAGGGCGAGGGAACGGAATTGAA
GGAGATTCTGAAAGCTTTGGAAGGGAATATGAGTGGTGGAAGGATAGTCATAGGAAGAGAGCTAAGCCGGCAGAATAGTTTAGTACTGGGATCA
TCACAATATGGAGAGGTCAATCATACAAGACTTGGGCTACGGCCAGGGAATATACCCAGAGAGGATAGTCAGTCAAGTTTGGGAATGTCAACGT
TGGAGGTCAATGACGAGGAAGATGAGGATGGCCTGATGGATCCAAGAAGGTGGTTAAAAGTCATTGATGCATTTGAGCAACCTCGACTGGTGTA
CAATGTTGCTAAAAAGCACTTTGATAGGTATGTTTCAATGATAAAATTTTATCTGAATCGACTAACTCAGTACAGAGATACCTCCAAACCTTCA
TTGTTCCCACCTGCGTCTCATAAAACACTCCTCTTCCAAAACCGCTATAATGTTATCCATCAACGTCTCCTTCGCAATGAATCTTTTCAAACGC
CCGCTTTTCAAGGTGGCAAATCTTCCCTTCAACGCAGCACGTCCGCCATTACCACCCAACAACAATCATACAAATTAACGCCGATAGCTAATCT
TCTCGGTCGCAATCGCAGCTCTCATATGCTTCTCGGTCTCCTCAGTATTTCACCCACTGGTACCCTCGCCATCAATGACCTGACGGGCAGTATC
GCTCTCGATCTTACACACGCAGCAGCCATTC

>Bcin11g01310(MLST5), partial sequence [organism=Botrytis cinerea, strain N11_K_W02]
ACTGACATGGACCTCATGTGGAACCGGCGTAGAATGCGCAACGCTTGAAGTTCCGCTCGAATATGGCGATGCAACGTCAACGGCAAAAGCCAGT
GTTGCGCTTGCTCGTTATCCTGCCACTGTTGCCGCGAGCAAGAAGCTCGGGTCTCTCTTGATAAACCCCGGTGGACCCGGTGCCTCTGGTGTTG
GCTTTGTGCAGTCTGGAGCCGGTGCCGCCGTCTCGACACTGAGTGGTGGATTGTACGATATCATCGGATGGGATCCACGTGGAACCGGTGCTTC

GGCTCCTATTTTGGAATGTTTTGCAAATGCCAGTGCGGAGTATGATTTTAACAACGCGTTTCCATCTGCTCCGAATCTCTGGCTCGGACAATTT
GCGAATGCCAGCGCAAATTCTGCTGTTAGCTCTGCTATCACATCCTTTGACACTTCTGTCGCTGCTCTTGCAAAAGCTTGCGTGGCTCAGAAAT
CTCCCGCTCTTTACACCTCAACAGCAGCATATGTTGCTCGAGACATGGCAGCGATAGTCGATGCATTGGATGGGACCTCTGCAAAACTTAACTA
CTGGGGTTTCTCATATGGAACTATCTTCCTAGCTGAGTTTATCCAAACTTTCCCAGGCCGCGTGGGAAGAGTTCTTGCCGATGGTGTTTTCGAC
GCAAAGGCAAATGCACTCACATACGTTAGCCAACTTCCCAACGATCAACTCAGTGTTCGTGCTTCGTTGAACGATTTTGCAGCTTTCTGCACCA
CCGCCGGTAGTAAAGGTTGCTCTTTTGCCACCGCCCCTACTGGAACCACAGGTACTGTTGCTACCAGACTGGACAACATAATGAAGGATATGTT
CCTCAATCCTATTGTTGCTTCGGGCTTAAGCATCAG

>Bcin15g03910(MLST6), partial sequence [organism=Botrytis cinerea, strain N11_K_W02]
GCCAAAACACAAAATCATCCAACGATGAAGATGATACTCCACTTCCCTTGATTATTTGGCATGGACTCGGCGATAATTACAAAGCGGATGGTCT
TGCGCAAGTTGGAAAACTAGCTGAAGCTATTCATCCTGGGACTTTTGTCTACAATATTCATGTAGATGAGGATGCATCTGCAGATAGGACAGCT
ACCTTCTTTGGAAATCTCACTCGTGAGTACATCCCCTATTTTCCCTTTAAATACCATACTAACTCTCTTACCAAGTTCAAATCGAAAAGGTCTG
CGAAGACCTCGCCTCCCATCCTATTCTCTCTACCGCGCCCGCCGTCGACGCAATTGGATTCTCCCAAGGCGGCCAATTCTTGCGCGGTTACATA
TCCCGCTGCAATGCTCCACCCATCCGCTCTCTCCTGACCTTCGGTTCCCAACACAACGGCATTTCTGCCTTCCAAGCCTGTGGTCCTGCCGATT
TCCTCTGTCGCGGTGCTCAAACCCTTTTGCGATCCAACACCTGGTCAACCTTTGTCCAATCTCGTCTCGTACCCGCTCAATACTTCAGAGATCC
GGAAAACCTAGACTCTTACCTTGAATATTCCAATTTCCTTGCCGACATCAATAATGAGCGCGTTCTCAAGAACCAAACATATAAATCCAACATG
GAAAAATTGGAACGATTCGTAATGTATGTCTTTGAAGACGATACAACTGTCATCCCTAAGGAAAGTGGATGGTGGGCTGAAGACAACGGCACGG
AAGTTACACCACTGAAAGAAAGAGCCATTTATAAAGAAGATTGGCTAGGTTTAAAGACATTGGATGAGGCCGGAAAATTAGTTTTCGAAACCAT
TCCAGGGGGACATATGACGTTAGGAGAGGAGATGCTAGAGAAGGCTTTCAAAGAGTATTTTGGTCCAGCAGGGAAGAAATTTGGG

>Bcin16g03460(MLST7), partial sequence [organism=Botrytis cinerea, strain N11_K_W02]
ATGAACCTCTTGATTTGAACTCTTCATTAATTGCAAGTGATGAACCCCTATTCTGCCCAGAGGATAGTTACAAGACGTATATCATTAGTCGAGA
ACCACTCATGATATATATTGACGGTTTTTTGAAAGCGAATGAAAGTAAACATTTGGTTGATGTTAGGTGTGTTATTTATTCTGATGAAATGAAC
AAGAGAGACTGATGAGATAGTGAGCCGCTTTATGAACCGTCAACTGTTTCTCACGGACAGGAAGTTACCATTGATCCTTCAGTTCGGAATTCTG
AAGTGGCGGTTTTAGAGAGGGATGAGGTGGTCAGGTGTATTGAGCATAGAGCGAGGGCATTTCAGGGGTGGAGGGGCGAGATGGGGATTGAGAA
GTTGAGGACGCAGAGGTATGGGGTTGGAGGACATTATGGGATGCATTTGTAAGTTTTGGGGGATTGACGAAGGCTTCTGTCTATTTTCTATCAG
CACGGATCTTGAAAGAAAGATAGCATGAACACGCGGGCTAATAACTAGGAAATAGCGATTGGAGCGGAGGTAAACGTGGCATAGACCGATTTAG
TACTTTCATGGTCTATGTCGACGTATCCTCTGATATCGAAGGTGGAGGAACGGAATTCCCACGTATTGTGGGACCAAAAGGAGGAAGGTGGGAG
GACTTCCTGGAAACTACGGAAGCATTGGATCCAAGAACTGGAGAAAATGTAACAGTAGAAGGGGTGACATTCAAACCAATCAAGGGAAATGCCG
TATTCTGGGAAAATACTGACAACAACGGGAGGGGCTATGAT

>Bcin12g03020(MLST8), partial sequence [organism=Botrytis cinerea, strain N11_K_W02]
CGATTGGCTGCGAAGAAAACTGCGCAGCACCGAGTCACCAAAAGCACGAATAAACCATCGGCAAGGGAAATATTCTCTGAACCACAACATGACA
CGAGCGCGGAAGAGTATATCGGGCGAGAAGCTTCATCAAGAGCACCAAAGCGACAACGAGTCGATGATAATTATAACTCTTACGGTGGAAGAAA
TGAAAATACAGCAGCTTATGCTTCCGGGAAACTTCCATCTGGAAGTATAAATGTTGGTGGAGGTAGGAAGACCACTTTTCAAGAAGAACCTCGA
ACGGCATTTGTCGCTGGCAAGTTGCCCCCTGGAAGTATCAACATTGGTGGAAAGAAGGCTACCCAAATCGAGGACGAGGGTAGAGCAGCTTATG
CTTCCGGAAAATTGCCCCCAGGAAGTATAAACTCTGGCGCAAAGAAGGCGATTTCATTCCAAGATGAAACGAGACCGGCTTATGTCTCCGGAAA
GCTTCCACATGGTAGTATCGACGGTATGCGAAACCGTGAAATGGCTGCCGTCCACCGCGAAATTGCTGAGGGTGGGAGGAAACCAGGCCAGGTT
GTCTCTTCTCTATTCACATTCAATCCTACTTCAAAGAAAACTTTCGATGAACCAGAAGAACAAGCGGAACCGGCAAAACCATCCAATGCGCCTT
TGACAGAGGAAATGGCGACTTTCACCAATCTAGGGCTATCGAGAAGGCTTGCAGCCCATCTATCGACTAAACTCGATATGAAAGCCCCGACGGC
CATTCAAAAAGCATCTGTGCAGCAGTTGGTATCGGACGATAGCGATGCTTTCATACAAGCAGAGACTGGATCTGGAAAAACTTTGGCATATCTA
CTACCTATAGTCGAGCGAATATTAGCATTGAGTGAGAATGGCGTACAAA

>Bcin02g07770(MLST9), partial sequence [organism=Botrytis cinerea, strain N11_K_W02]
ACAGCTTTCCCTTTCGGTCTTGGCATCTACAGTCATTGCCATCCCTACACCATCACAACTTGAGTCTCGGGCCGTTATCGATTCCGATGCCGTT
GTAGGATTTGCCGAAACTGTTCCCAGTGGGACCGTAGGAACAGTTTATGAGGCATATAAACCATTCCTTAAAGTCGTAAATGGATGCGTACCAT
TCCCTGCCGTCGATGCATCGGGTAACACAGGGTATGTCCTTATATCTTTCTCTTCCACACGATTGCTATTGAGTCTCTAACATATTTTAGTGGT
GGTTTGTCACCAACTGGCAGTAGCAATGGTGGTTGCAGCAGCAGTACCGGTCAAGTATATGTTCGAGGAGGACAAAGCGGATCAAACTACGCCA
TCATGTACTCCTGGTAAGTTCTCTCTAAACTTCTCCTTATAGATCCAACCTAACAAAATCTTAGGTACATGCCAAAGGACGAGCCCTCAACCGG
TATTGGTCACCGTCACGATTGGGAAGGTGTAATTGTCTGGCTCTCCAGCGCCACCGCCACAACTGCCGACAACATCTTAGCCGTTTGTCCTTCC
GCCCACGGAGGCTGGGATTGTTCCACGGATGGCTATTCCCTTTCTGGTACCGGCCCTCTTATCAAGTACGAAAGTATCTGGCCCGTCGATCACT
CAATGGGTCTTACTAGTACTGTTGGTGGAAAACAACCTATGATTGCTTGGGAGTCTTTACCAACTGCTGCTCAAACTGCTCTTGAGAACACCGA
TTTCGGTGCTGCGAATGTTCCATTCATTCCGGCTGTTTTCACAGACAATCT

>Bcin04g02090(MLST10), partial sequence [organism=Botrytis cinerea, strain N11_K_W02]
CGGAGGATGATATGGCAAAGTCTATGATTACCAAAGCATTTGTAGGCATGAGTAGTAAATTGTCAAATATGACTATGAATGATGTTTACAAGCC
CTACATTCATGTAGGAAATGTAGAATAGAGAGATCAGTAACTGGAACTAATATCGTTTGTAGGCTTTCAAGTTACTTACGCAGTTCAACCCAAT

CACTACAGCTATTGCCGAATCCCCACTGTTTCAAATGGCCGTCTCAGCAAATACCATCGAAAAGTACACACTGCTAGGCCCTTTCTTCAGAATA
TCTCCTCTGCAACAGGAAGTTACCAGGGAATACTTCAGTGCGCCAAAGACGATAGATAGGCGACACATTGCCACATCTCAAGATGCGTTACGAT
TGACCTTACAAACCCATCAAAAAGATTTACTTGATATCATCAACCACTTTGTTCGAGCAAGTCCAATCGCAGAAAGCAAAACCCTGGATTGGTT
CGCCTACATTGTGAATCAAAATCACAAGCGTCGAGCACTTCAGGTAGACCCGAAAGAAGTGTCTTCTGATGGCTTTATGCACAATGTCACTGTC
GTTCTAGATGGTCTTTGTGAGCCATTCATGGATACCACATTCTCGAAAATTTCGAAGATTGACATTGATTATCTAAGACGTGCGCCTCGTGTAG
ATATCAAGGACGAGACCAAGTTGAACGCTGACGAGAAGGCTTCTGAGAAGTATTATGAGGACACTGTTCCTGGCACTTCTAATTTCATCTCTGA
GGTCTTCTTTCTCACATTGGCTGCTCATCATTATGGTAGTGAAGCTCTTAATGCCACGCATAAGAGTCTGGAGAAAGACATCAAATATATTCAA
AAGCAATTGACTGCCGTTGAAGCA

>Bcin01g07220(MLST1), partial sequence [organism=Botrytis cinerea, strain N11_K_W03]
ATGGCATATCTTGTTTCTTTTCGCATTATGCTTTGGAACGTGCTGGACAAGCTTGGGTTAGTGCTCTGCGTGTAGAAGCACTAAAGAAGATTCT
CGCACAACCGAAGTCATGGTTTGAGGAATCCAGGAATTCACCTGGCCGGTTGAACGAAGTTTTGGATAGGAACTCTGAGGAAATGCGTAATCTC
GTTGGCCGCTTTGCTGGTATTGTATTCACAGCATTTTTTATGCTATTGATATCGATCATTTGGGCTTTCGTGAATACATGGAAACTGACATTAG
TCTCAATGGCAACTGGGCCAGTTATATATGCTGTCACCAAAACGTTCAATCGCGTGAGTGGAAAATGGGAAAACAAGTGCAACTACGCCTCTGA
AATGACCACTGGCATATTTTCAGAGACTTTCTCCAACATCAAAGTGGTTCGGGCTTTTACTCTGGAAACTTACTTTGAGACAAAACACACCAAA
GCTACAGAAGAACTTTATAAAGTTGGATTAATACGAGCAAACTACTCGGGATTGCTGTGGGGATTGACAGATGCGATGTCATTCTTCATCACTG
CAACTATCTTTTATTATGCCACGGTTCTCATTACCAAGAGAGAGATCAGTATCGCGACTGCACTACAGACTGTCAATCTTCTATTATTTGGTAT
TTCTAATAGTACGAATATGCTGGCCATGATACCACAAATCAACTCTTCTCGCGTTACAGCTACGCATATGCTTGCATTAGCCAATCTCGATTCA
TCTTCCTCCCACGAAAATAAAGGAACCGAACGGCTTTCGACAATCTTTCCAATCAAATTCAACCGTCTCTCATTCACATATCCTACTCGTCCTG
AAAAACGAACGATATCATCCTTTTCTCTTTCCCTGATTCCTAACTCAACAACTGCACTTGTCGGACCCTCCGGCTCCGGAAAATCTACAATAGC
TGCTCTGCTCATTGGTCTCTATCCTCCAGATACTTCAACACCTCCACCGTTGACATTCAATCGCGTCTCCATAAGTAACTGTCACATTCCGTCT
CTCCGGGCTTCTCTCTCACTCGTCCCACAAATACCGATTCTATTTCCAGCTACCATTCTCCATAACATCATTTATGGTCTCCCAGAATCTTCTC
CTTGTGCTAGTCTTCCATCTGCTATTGCATCAGCAAAAGATGCTGGGATCCATGAATTTATCACATCGCTTCCACAAGGTTATGATACTAT

>Bcin05g07690(MLST2), partial sequence [organism=Botrytis cinerea, strain N11_K_W03]
ACCACTATCACCAAGTCCTTTTCTGCACTTTCCTCATCAAAAACGAACGAGAACATCATCAATAAATACTTTGCTGAAGTAATTGGGTTTTATT
TTGGCCAGGATGCCTTCTCTCTACGACAGCAAGCCTTCGATGATATGTTGTGGGTAGTTCTTGGCTGGCTGGATACTGTCAAATTCATTGATTT
ACATTCCGAATTACACTATTCAAACGACTCTCAGCCAGAATGGTACGGACAACAATATAAACCTGCATTTGCACATCGAGCGCGACTATTTTGG
GAATTGGCTTCACAAGGATGGGATACTACTCTCTGTGGTGGTGGGATGATATGGTCACCATACCTTACTCCATACAAGAACGCAATTACCAATG
AACTCTATATCGCAGCTTCGATATCAATGTACCTCTATTTCCCCGGAGATGACAATCAATCCCCATTTATGCTTTCCAACCCTTCATATCCACC
TCACGATCCGAAATATCTACAGGCAGCTGTTGATGCTTACAAATGGCTGAATGGTTCCAACATGACGGATTTACAAGGATTATATGTCGACGGG
TACCATATCTCGAATCTTTCTGGCGGTGAAAACACCCATTGCGATTCTAGAAATGAGATGGTATATACCTACAATCAAGGTGTTTTGCTTACTG
GACAACGTGGTTTGTATGACGCAACCGCCGCACGATCATACCTTGTAGATGGCCACAAACTCATCGCGAATGTTATTAATGCCACAGGCTATGA
CCTGAAACACAATGTTGTCATCTCACCGCCACCCAAAGATGGTTCCGCATTGGCAAAGTGGTTTGGCCTGGGTAGGAATGGAATACTGGAAGAA
GGATGCGATTCAAGTGCTTCGTGTTCTCAAAATGGACAAACTTTCAAAGGCATATTCTTTCACCACTTGATTGCGTTCTGTAGTGATTTGCCAG
GGGAGCCTATTGCAGGGACGAAGGAAGGCCTAGAACTTGACAGAGTGTGGCATTCTGACAAATGCTCACAGTATACAAAAT

>Bcin06g01710(MLST3), partial sequence [organism=Botrytis cinerea, strain N11_K_W03]
GTGAGTCTGACTTTTGTATTTGAGCGTTAAGATAGACTCTGATATACCAAGGCAATCACCTAATTTCATGGCGTTCTTCCCTGCATCATCTACC
TACCCTGGAATGCTGGGAGAATTATACTCAGCAGCTTTCACAGCTCCTGCTTTCAATTGGATCTGTTCCCCTGCTGTGACAGAATTGGAGACGG
TTGTAATGGATTGGTTGGCCAAGCTTCTCAATCTCCCAGACTGTTATTTGTCGTCGACTCATGGTGGTGGTGTTATCCAAGGATCAGCCTCGGA
AGCTATCGTTACCGTTATGGTTGCTGCCCGCGATAAGTATCTTCGTGAAACCACTGAAGGTCTGTCGGGAATTGAACTCGAGGATGCGATTGCA
TATAAGAGGAGTAAGCTAGTTGCACTAGGAAGCGAAATGGCACACAGCTCCACGCAGAAAGCAGCGCAGATAGCTGGCGTTAGATTCCGATCGA
TTCCAGTACTCGCATCCAATGATTTCGCCATGACGGGTGATGATTTAGAGAAGGTATTGAAAGAATGCAAATCTCAAGGATTGGAACCCTTCTA
TCTAACTTCGACGTTGGGAACAACATCTACATGCGCAGTTGACGACTTCGCATCTATTGCAACAGTACTTTCAAAATATGCACCTCCAGATGTT
GCAGGCGAGATCTGGGTTCACGTCGATGCTGCTTATGCAGGTGCAGCTTTGGTTTGCCCTGAATACCATCATCTAACATCGTCCTTCCAGCATT
TCCATTCCTTTGATATGAACATGCACAAATGGCTTCTGACAAATTTCGACGCTTCTTGCCTATATGTCAAGAAACGCAAAGATCTGATCGATGC
ACTCTCCATAACACCAAGTTATCTTCGCAACGAGTTTTCAGAGAGTGGACTCGTAACCGACTATCGGGACTGGCAAATTCCTCTCGGAAGACGC
TTCCGAAGCTTAAAGATTTGGTTTGTCCTCAGAACCTAC

>Bcin09g03030(MLST4), partial sequence [organism=Botrytis cinerea, strain N11_K_W03]
ACCCTTCAACCCCACCGCAGCTCCTATACCGAAAGCGAGTATCCTACCAATTCTCCTTCCACCTGCGACTTTAAGACCATTGGCTTTTCGCACT
TTCACAAAAAAGCATAGTTTAACATTGACGTCGTCGGCATTACAAGTGTTGGCTACTTTTATTGGGAAGCATTGTGGGACAGGATGGAGGGAGG
AAGGACTGGCAGAGAGAGTCTTGGAGGAGGTCGCCAAGAGTTGGAAGAATAGGAGTGGCGGTGTCATTGTCGAGGGCGAGGGAACGGAATTGAA
GGAGATTCTGAAAGCTTTGGAAGGGAATATGAGTGGTGGAAGGATAGTCATAGGAAGAGAGCTAAGCCGGCAGAATAGTTTAGTACTGGGATCA
TCACAATATGGAGAGGTCAATCATACAAGACTTGGGCTACGGCCAGGGAATATACCCAGAGAGGATAGTCAGTCAAGTTTGGGAATATCAACGT
TGGAGGTCAATGACGAGGAAGATGAGGATGGCCTGATGGATCCAAGAAGGTGGTTAAAAGTCGTTGATGCTTTGAGCAACCTCGACTGGTGTAC

AATGTTGCTAAAAAGCACTTTGATAGGTATGTTTCAATGATAAAATTTTATCTGAATCGACTAACTCAGTACAGAGATACCTCCAAACCTTCAT
TGTTCCCACCTGCGTCTCATAAAACACTCCTCTTCCAAAACCGCTATAATGTTATCCATCAACGTCTCCTTCGCAATGAATCTTTTCAAACGCC
CGCTTTTCAAGGTGGCAAATCTTCCCTTCAACGCAGCACGTCCGCCATTACCACCCAACAACAATCATACAAATTAACGCCGATAGCTAATCTT
CTCGGTCGCAATCGCAGCTCTCATATGCTTCTCGGTCTCCTCAGTATTTCACCCACTGGTACCCTCGCCATCAATGACCTGACGGGCAGTATCG
CTCTTGATCTTACACACGCAGCAGCCATTC

>Bcin11g01310(MLST5), partial sequence [organism=Botrytis cinerea, strain N11_K_W03]
GACTGACATGGACCTCATGTGGAACCGGCGTAGAATGCACAACGCTTGAAGTTCCGCTCGAATATGGCGATGCAACGTCAACGGCAAAAGCCAG
TGTTGCGCTTGCTCGTTATCCTGCCACTGTTGCCGCGAGCAAGAAGCTCGGGTCTCTCTTGATAAATCCCGGTGGACCCGGTGCCTCTGGTGTT
GGCTTTGTGCAGTCTGGAGCTGGTGCCGCCGTCTCGACACTGAGTGGTGGATTATACGATATCATCGGATGGGATCCACGTGGAACCGGTGCTT
CGGCTCCTATTTTGGAATGTTTTGCAAATGCCAGTGCGGAGTATGATTTTAACAACGCGTTTCCATCTGCTCCGAATCTCTGGCTCGGACAATT
TGCGAATGCCAGCGCAAATTCTGCTGTTAGCTCTGCTATCACATCCTTTGACACTTCTGTCGCTGCTCTTGCAAAAGCTTGCGTGGCTCAGAAA
TCTCCCGCTCTTTACACCTCAACAGCAGCATATGTTGCTCGAGACATGGCAGCGATAGTCGATGCATTGGATGGGACCTCTGCAAAACTTAACT
ACTGGGGTTTCTCATATGGAACTATCTTCCTAGCTGAGTTTATCCAAACTTTCCCAGGCCGCGTGGGAAGAGTTCTTGCCGATGGTGTTTTCGA
CGCAAAGGCAAATGCACTCACATACGTTAGCCAACTTCCCAACGATCAACTCAGTGTTCGTGCTTCGTTGAACGATTTTGCAGCTTTCTGCACC
ACCGCCGGTAGTAAAGGTTGCTCTTTTGCCACCGCCCCTACTGGAACCTCAGGTACTGTTGCTACCAGACTGGACAACATAATGAAGGATATGT
TCCTCAATCCTATTGTTGCTTCGGGCTTGAGCATCA

>Bcin15g03910(MLST6), partial sequence [organism=Botrytis cinerea, strain N11_K_W03]
GCCAAAACACAAAATCTTCCAACGATGAAGATGATACTCCACTTCCCTTGATTATCTGGCATGGACTCGGCGATAATTACAAAGCGGATGGTCT
TGCGCAAGTTGGAAAACTAGCTGAAGCTATTCATCCTGGGACTTTTGTCTATAATATTCATGTAGATGAGGATGCATCTGCAGATAGGACAGCT
ACCTTCTTTGGAAATCTCACTCGTGAGTACATCCCCTATTTTTCCTTTAAATACCATACTAACTCTCTTACCAAGTTCAAATCGAAAAGGTCTG
CGAAGACCTCGCCTCCCATCCTATTCTCTCTACCGCGCCCGCCGTCGACGCAATTGGATTCTCCCAAGGCGGCCAATTCTTGCGCGGTTACATA
TCCCGCTGCAATGCTCCACCCATCCGCTCTCTCCTGACCTTCGGTTCCCAACACAACGGCATTTCTGCCTTCCAAGCCTGTGGTCCTGCCGATT
TCCTCTGTCGCGGTGCTCAAACCCTTTTGCGATCCAACACCTGGTCAACCTTTGTCCAATCTCGTCTCGTACCCGCTCAATACTTCAGAGATCC
GGAAAACCTAGACTCTTACCTTGAATATTCCAATTTCCTTGCCGACATCAATAATGAGCGCGTTCTCAAGAACCAAACATATAAATCCAACATG
GAAAAATTGGAACGATTCGTAATGTATGTCTTTGAAGACGATACAACTGTCATCCCTAAGGAAAGTGGATGGTGGGCTGAAGTCAACGGCACGG
AAGTTACACCACTGAAAGAAAGAGCCATTTATAAAGAAGATTGGCTAGGTTTAAAGACATTGGATGAGGCCGGAAAATTAGTTTTCGAAACCAT
CCCAGGGGGACATATGACGTTAGGAGAGGAGATGCTAGAGAAGGCTTTCAAAGAGTACTTTGGTCCAGCAGGGAAGAAATTTGGG

>Bcin16g03460(MLST7), partial sequence [organism=Botrytis cinerea, strain N11_K_W03]
ATGAACCTCTTAATTTGAACTCTTCATTAATTGCAAGTGATGAGCCCCTATTCTGCCCAGAGGATAGTTACAAGACGTATATCATTAGTCGAGA
ACCACTCATGATATACATTGACGGATTTTTGAAAGCGAATGAAAGTAAACACTTGGTTGATGTTAGGTGTGTTATTTATTCTGATGAAATGAAC
AAGAGAGACTGATGAGATAGTGAACCGCTTTATGAACCGTCTACTGTTTCTCACGGACAGGAAGTTTACCATTGATCCTTCGGTTCGAAATTCT
GAAGTGGCGGTTTTAGAGAGGGATGAGGTGGTCAGGTGTATTGAGCATAGAGCGAGGGGCATTTCAGGGGTGGAGGGACGAGATGGGGATTGAG
AAGCTGAGGACGCAGAGGTATGGGGTTGGAGGACATTATGGGATGCATTTGTAAGTTTTGGGGGATTGACGAAGGCTTCTGTCTATTTTCTATC
AGTGCGGATCTTGAAAGAAAGATAGCATGAATACGAGGGCTAATAACTAGGAAATAGCGATTGGAGCGGAGGTAAACGTGGCATAGACCGATTT
AGTACTTTCATGGTCTATGTCGACGTATCCTCTGATATCGAAGGTGGAGGAACGGAATTCCCACGTATTGTGGGACCAAAAGGAGGAAGGTGGG
AGGACTTCCTGGAAACTACGGAAGCATTGGATCCAAGAACTGGAGAAAATGTAACAGTAGAAGGGGTGACATTCAAACCAATCAAGGGAAATGC
CGTATTCTGGGAAAATACTGACAACAACGGGAGGGGCTATGATG

>Bcin12g03020(MLST8), partial sequence [organism=Botrytis cinerea, strain N11_K_W03]
CGATTGGCTGCGAAGAAAACTGCGCAGCACCGAGTCACCAAAAGCACGAATAAACCATCGGCAAGGGAAATATTCTCTGAACCACAACATGACA
CGAGCGCGGAAGAGTATATCGGGCGAGAAGCTTCATCAAGAGCACCAAAGCGACAACGAGTCGATGATAATTATAACTCTTACGGTGGAAGAAA
TGAGAATACAGCAGCTTATGCTTCCGGGAAACTTCCATCTGGAAGTATAAATGTTGGTGGAGGTAGGAAGACCACTTTTCAAGAAGAACCTCGA
ACGGCATTTGTCGCTGGCAAGTTGCCCCCTGGAAGTATCAACATTGGTGGAAAGAAGGCTACCCAAATCGAGGACGAGGGTAGAGCAGCTTATG
CTTCCGGAAAATTGCCCCCAGGAAGTATAAACTCTGGCGCAAAGAAGGCGATTTCATTCCAAGATGAAACGAGACCGGCTTATGTCTCTGGAAA
GCTTCCACATGGTAGTATGGACGGTATGCGAAACCGTGAAATGGCTGCCGTCCACCGCGAAATTGCTGAGGGTGGGAGGAAACCAGGCCAGGTT
GTCTCTTCTCTATTCACATTCAATCCTACTTCAAAGAAAACTTTCGATGAACCCGAAGAACAAGCGGAACCGGCAAAACCATCCAATGCACCTT
TGACAGAGGAAATGGCGACTTTCACCAATCTAGGGTTATCGAGAAGGCTTGCAGCCCATCTATCGACTAAACTCGATATGAAAGCCCCGACCGC
CATTCAAAAAGCATCTGTGCAGCAGTTGGTATCGGACGATAGCGATGCTTTCATACAAGCAGAGACTGGATCTGGAAAAACTTTGGCATATCTA
CTACCTATAGTCGAGCGAATATTAGCATTGAGTGAGAATGGCGTACAAA

>Bcin02g07770(MLST9), partial sequence [organism=Botrytis cinerea, strain N11_K_W03]
CAGCTTTCCCTTTCGGTCTTGGCATCTACAGTCATTGCCATCCCTACACCATCACAACTTGAGTCTCGGGCCGTTATCGATTCCGATGCCGTTG
TAGGATTTGCCGAAACTGTTCCCAGTGGGACCGTAGGAACAGTTTATGAGGCATATAAACCATTCCTTAAAGTCGTAAATGGATGCGTACCATT
CCCTGCCGTCGATGCATCGGGTAACACAGGGTATGTCCTTATACCATTCTCTTCCACACGATTGCTATTGAGTCTCTAACATATTTTAGTGGTG

GTTTGTCACCAACTGGCAGTAGCAATGGTGGTTGCAGCAGCAGTACCGGTCAAGTATATGTTCGAGGAGGACAAAGCGGATCAAACTACGCCAT
CATGTACTCCTGGTAAGTTCTCTCTAAACTTCTCCTTATAGATCCAACCTAACAAAATCTTAGGTACATGCCAAAGGACGAGCCCTCAACCGGT
ATTGGTCACCGTCACGATTGGGAAGGTGTAATTGTCTGGCTCTCAAGCGCCACCGCCACAACTGCCGACAACATCTTAGCCGTTTGTCCTTCCG
CCCACGGAGGCTGGGATTGTTCCACCGATGGATATTCCCTTTCTGGTACCAGCCCTCTTATCAAGTACGAAAGTATCTGGCCCGTCGATCATTC
AATGGGTCTTACTAGTACTGTTGGTGGAAAACAACCTATGATTGCTTGGGAGTCTTTACCAACTGCTGCTCAAACTGCTCTTGAGAACACCGAT
TTCGGTGCTGCGAATGTTCCATTCATTCCGGCTGTTTTCACAGATAATCT

>Bcin04g02090(MLST10), partial sequence [organism=Botrytis cinerea, strain N11_K_W03]
CGGAGGATGATATGGCAAAGTCTATGATTACCAAAGCATTTGTAGGCATGAGTAGTAAACTGTCGAATATGACTATGAATGATGTTTACAAGCC
CTACATCCATGTAAGAAATGTAGAATAGAGAGATCAGTAACTGGAACTAATATCGTTTGCAGGCTTCAAGTTACTTACGCAGTTCAACCCAATC
ACTACAGCTATTGCCGAATCCCCACTATTTCAAATGGCTGTCTCAGCAAATACTATCGAAAAGTACACACTGCTAGGCCCTTTCTTCAGAATAT
CTCCTCTGCAACAGGAAGTTACCAGGGAATACTTCAGTGCGTCAAAGACGATAGATAGGCGACACATTGCCACATCTCAAGATGCCTTACGATT
GACTTACAACCCATCAAAAAGATTTACTTGATATCATCAACCACTTTGTTCGAGCAAGTCCAATCGCCAAAAGCAAAACCCTGGATTGGTTCGC
CTACATTGTGAATCAAAATCACAAGCGTCGAGCACTTCAGGTAGACCCGAAAGAAGTGTCTTCTGATGGTTTTATGCACAATGTCACTGTCGTT
CTAGATGGTCTTTGTGAGCCATTCATGGATACCACATTCTCAAAAATTTCGAAGATTGATATTGATTATCTAAGACGTGCGCCTCGTGTAGATA
TCAGGACGAGACCAAGTTGAACGCTGATGAGAAGGCTTCTGAGAAGTATTATGAGGACACTGTTCCTGGCACTTCTAATTTCATCTCTGAGGTC
TTCTTTCTGACATTGGCTGCTCATCATTATGGTAGTGAAGCTCTTAATGCCACGCATAAGAGTCTGGAGAAAGACATCAAATATATTCAAAAGC
AATTGACTGCCGTTGAAGCAG

>Bcin01g07220(MLST1), partial sequence [organism=Botrytis cinerea, strain N11_K_W06]
ATGGCATATCTTGTTTCTTTTCGCATTATGCTTTGGAACGTGCTGGACAAGCTTGGGTTAGTGCTCTGCGTGTAGAAGCACTAAAGAAGATTCT
CCAACAACCGAAGTCATGGTTTGAGGAATCCAGGAATTCACCTGGCCGGTTGAACGAAGTTTTGGATAGGAACTCTGAGGAAATGCGTAATCTC
GTTGGCCGCTTTGCTGGTATTGTATTCACAGCATTTTTTATGCTATTGATATCAATCATTTGGGCTTTCGTGAATACATGGAAACTGACATTAG
CCTCAATGGCAACTGGCCAGTTATATACGCTGTCACCAAAACGTTCAATCGCGTGAGTGGAAAAATGGGAAAACAAGTGCAACTACGCATCTGA
AATGACCACTGGCATATTTTCAGAGACTTTCTCCAACATCAAAGTGGTTCGGGCTTTTACTCTGGAAACTTACTTTGAGACAAAACACACCAAA
GCTACAGAAGAACTTTATAAAGTTGGACTAATACGAGCAAACTACTCGGGATTGCTGTGGGGATTGACAGATGCGATGTCATTTTTCATCACTG
CAACTATCTTTTATTATGCCACGGTTCTCATTACCAAGAGAGAGATCAGTATCGCGACTGCACTACAGACTGTCAATCTTCTATTATCTGGTAT
TTCTAATAGTACGAATATGCTGGCCATGATACCACAAATCAACTCTTCTCGCGTTACAGCTACGCATATGCTTGCATTAGCCAATCTCGATTCA
TCTTCCTCCCACGAAAATAAAGGAACCGAACGGCTTTCGACAATCTTTCCAATCAAATTCAACGTCTTTCATTCACATACCCTACTCGTCCTGA
AAAACGAACGATATCATCCTTTTCTCTTTCCCTGATTCATAACTCAACAACTGCACTTGTCGGACCCTCCGGCTCCGGAAAATCTACGATAGCT
GCTCTGCTCATTGGTCTCTATCCGCCAGATACTTCAACACCTCCACCGTTGACATTCAATCGCGTCTCCATAAGTAACTGTCACATTCCGTCTC
TCCGGGCTCTCTCTCACTCGTCCCACAAATACCGATTCTATTTCCAGCTACCATTCTCCATAACATCATTTATGGTCTCCAGAATCTTCTCCTT
GTGCTAGTCTTCCATCTGCTATTGCATCAGCAAAAGATGCTGGGATCCATGAATTTATCACATCGCTTCCACAAGGTTATGATACTAT

>Bcin05g07690(MLST2), partial sequence [organism=Botrytis cinerea, strain N11_K_W06]
ACCACTATCACGAAGTCCTTTTCTGCACTTTCCTCATCAAAAACGAACGAGAACATCATCAATAAATACTTTGCTGAAGTAATTGGGTTTTATT
TTGGCCAGGATGCCTTCTCTCTACGACAGCAAGCCTTCGATGATATGTTGTGGGTAGTTCTTGGCTGGCTGGATACTGTCAAATTCATTGATTT
ACATTCTGAATTGCACTATTCAAACGACTCTCAGCTAGAATGGTACGGACAACAATATAAACCTGCATTTGCACATCGAGCGCGACTATTTTGG
GAATTGGCTTCACAAGGATGGGATACTACTCTCTGTGGTGGTGGGATGATATGGTCACCATACCTTACTCCATACAAGAATGCAATTACCAATG
AACTCTATATCGCAGCTTCGATATCGATGTACCTCTATTTCCCCGGAGATGACAATCAATCCCCATTTATGCTTTCCAACCCTTCATATCCACC
TCACGATCCGAAATATCTACAGGCAGCTGTTGATGCTTACAAATGGCTGAATGGTTCCAACATGACGGATTTACAAGGATTATATGTCGACGGG
TATCATATCTCGAATCTTTCTGGCGGTGAAAACACCCATTGCGATTCTAGAAATGAGATGGTATATACCTACAATCAAGGTGTTTTGCTTACTG
GACAACGTGGTTTGTATGACGCAACCGCCGCACGATCATACCTTGTGGATGGCCACAAACTCATCGCGAATGTTATTAATGCCACAGGCTATGA
CCTGAAACACAATGTTGTCATCTCACCGCCACCCAAAGATGGTTCCGCATTGGCAAAGTGGTTTGGCCTGGGTAGGAATGGAATACTGGAAGAA
GGATGCGATTCAAGTGCTTCGTGTTCTCAAAATGGACAAACTTTCAAAGGCATATTCTTTCACCACTTGATTGCGTTCTGTAGTGATTTGCCAG
GGGAGCCTATTGCAGGGACGAAGGAAGGCCTAGAACTCGACAGAGTGTGGCATTCTGACAAATGCTCACAGTATACAAAAT

>Bcin06g01710(MLST3), partial sequence [organism=Botrytis cinerea, strain N11_K_W06]
GGTGAGTCTGACTTTTGTATTTGAGCGTTAAGATGGACACTGATGTACCAAGGCAATCACCTAATTTCATGGCGTTCTTCCCTGCATCATCTAC
CTACCCTGGAATGCTGGGAGAATTATACTCAGCAGCTTTCACAGCTCCTGCTTTCAATTGGATCTGTTCCCCTGCTGTGACAGAATTGGAGACG
GTTGTAATGGATTGGTTGGCCAAGCTTCTCAATCTCCCAGACTGTTATTTGTCGTCGACTCATGGTGGTGGTGTTATCCAAGGATCAGCCTCGG
AAGCTATCGTTACCGTTATGGTTGCTGCCCGCGATAAGTATCTTCGTGAAACCACTGAAGGTCTGTCGGGAATTGAACTCGAGGATGCGATTGC
ATATAAGAGGAGTAAGCTAGTTGCACTAGGAAGCGAAATGGCACACAGCTCCACGCAGAAAGCAGCGCAGATAGCTGGCGTTAGATTCCGATCG
ATTCCAGTACTCGCATCCAATGATTTCGCCATGACGGGTGATGATTTAGAGAAGGTATTGAAAGAATGCAAATCTCAAGGATTGGAACCCTTCT
ATCTAACTTCGACGTTGGGAACAACATCTACATGCGCAGTTGACGACTTCGCATCTATTGCAACAGTACTTTCAAAATATGCACCTCCAGATGT
TGCAGGCGAGATCTGGGTTCACGTCGATGCTGCTTATGCAGGTGCAGCTTTGGTTTGCCCTGAATACCATCATCTAACATCGTCCTTCCAGCAT
TTCCATTCCTTTGATATGAACATGCACAAATGGCTTCTGACAAATTTCGACGCTTCTTGCCTATATGTCAAGAAACGCAAAGATCTGATCGATG

CACTCTCCATAACACCAAGTTATCTTCGCAACGAGTTTTCAGAGAGTGGACTCGTAACCGACTATCGGGACTGGCAAATTCCTCTCGGAAGACG
TTTCCGAAGCTTAAAGATTTGGTTTGTCCTCAGAACCTAC

>Bcin09g03030(MLST4), partial sequence [organism=Botrytis cinerea, strain N11_K_W06]
ACCCTTCAACCCCACCGCAGCTCCTATACCGAAAGCGAGTATCCTACCAATTCTCCTTCCACCTGCGACTTTAAGACCATTGGCTTTTCGCACT
TTCACAAAAAAGCATAGTTTAACATTGACGTCGTCGGCATTACAAGTGTTGGCTACTTTTATTGGAAAGCATTGTGGGACAGGATGGAGGGAGG
AAGGACTGGCAGAGAGAGTCTTGGAGGAGGTCGCCAAGAGTTGGAAGAATAGGAGTGGCGGTGTCATTGTCGAGGGCGAGGGAACGGAATTGAA
GGAGATTCTGAAAGCTTTGGAAGGGAATATGAGTGGTGGAAGGATAGTCATAGGAAGAGAGCTAAGCCGGCAGAATAGTTTAGTACTGGGATCA
TCACAATATGGAGAGGTCAATCATACAAGACTTGGGCTACGGCCAGGGAATATACCCAGAGAGGATAGTCAGTCAAGTTTGGGAATGTCAACGT
TGGAGGTCAATGACGAGGAAGATGAGGATGGCCTGATGGATCCAAGAAGGTGGTTAAAAGTCGTTGATGCATTTGAGCAACCTCGACTGGTGTA
CAATGTTGCTAAAAAGCACTTTGATAGGTATGTTTCAATGATAAAATTTTATCTGAATCGACTAACTCAGTACAGAGATACCTCCAAACCTTCA
TTGTTCCCACCTGCGTCTCATAAAACACTCCTCTTCCAAAACCGCTATAATGTTATCCATCAACGTCTCCTTCGCAATGAATCTTTTCAAACGC
CCGCTTTTCAAGGTGGCAAATCTTCCCTTCAACGCAGCACGTCCGCCATTACCACCCAACAACAATCATACAAATTAACGCCGATAGCTAATCT
TCTCGGTCGCAATCGCAGCTCTCATATGCTTCTCGGTCTCCTCAGTATTTCACCCACTGGTACCCTCGCCATCAATGACCTGACGGGCAGTATC
GCTCTTGATCTTACACACGCAGCAGCCATTC

>Bcin11g01310(MLST5), partial sequence [organism=Botrytis cinerea, strain N11_K_W06]
ACTGACATGGACCTCATGTGGAACCGGCGTAGAATGCGCAACGCTTGAAGTTCCGCTCGAATATGGCGATGCAACGTCAACGGCAAAAGCCAGT
GTTGCGCTTGCTCGTTATCCTGCCACTGTTGCCGCGAGCAAGAAGCTCGGGTCTCTCTTGATAAATCCCGGTGGACCCGGTGCCTCTGGTGTTG
GCTTTGTGCAGTCTGGAGCCGGTGCCGCCGTCTCGACACTGAGTGGTGGATTATACGATATCATCGGATGGGATCCACGTGGAACCGGTGCTTC
GGCTCCTATTTTGGAATGTTTTGCAAATGCCAGTGCGGAGTATGATTTTAACAACGCGTTTCCATCTGCTCCGAATCTCTGGCTCGGACAATTT
GCGAATGCCAGCGCAAATTCTGCTGTTAGCTCTGCTATCACATCCTTTGACACTTCTGTCGCTGCTCTTGCAAAAGCTTGCGTGGCTCAGAAAT
CTCCCGCTCTTTACACCTCAACAGCAGCATATGTTGTTCGAGACATGGCAGCGATAGTCGATGCATTGGATGGGACCTCTGCAAAACTTAACTA
CTGGGGTTTCTCATATGGAACTATCTTCCTAGCTGAGTTTATCCAAACTTTCCCAGGCCGCGTGGGAAGAGTTCTTGCCGATGGTGTTTTCGAC
GCAAAGGCAAATGCACTCACATACGTTAGCCAACTTCCCAACGATCAACTCAGTGTTCGTGCTTCGTTGAACGATTTTGCAGCTTTCTGCACCA
CCGCCGGTAGTAAAGGTTGCTCTTTTGCCACCGCCCCTACTGGAACCTCAGGTACTGTTGCTACCAGACTGGACAACATAATGAAGGATATGTT
CCTCAATCCTATTGTTGCTTCGGGCTTAAGCATCA

>Bcin15g03910(MLST6), partial sequence [organism=Botrytis cinerea, strain N11_K_W06]
CGCCAAAACACAAAATCATCCAACGATGAAGATGATACTCCACTTCCCTTGATTATCTGGCATGGACTCGGCGATAATTACAAAGCGGATGGTC
TTGCGCAAGTTGGAAAACTAGCTGAAGCTATTCATCCTGGGACTTTTGTCTATAATATTCATGTAGATGAGGATGCATCTGCAGATAGGACAGC
TACCTTCTTTGGAAATCTCACTCGTGAGTACATCCCCTATTTTTCCTTTAAATACCATACTAACTCTCTTACCAAGTTCAAATCGAAAAGGTCT
GCGAAGACCTCGCCTCCCATCCTATTCTCTCTACCGCGCCCGCCGTCGACGCAATTGGATTCTCCCAAGGCGGCCAATTCTTGCGCGGTTACAT
ATCCCGCTGCAATGCTCCACCCATCCGCTCTCTCCTGACCTTCGGTTCCCAACACAACGGCATTTCTGCCTTCCAAGCCTGTGGTCCTGCCGAT
TTCCTCTGTCGCGGTGCTCAAACCCTTTTGCGATCCAACACCTGGTCAACCTTTGTCCAATCTCGTCTCGTACCTGCTCAATACTTCAGAGATC
CGGAAAACCTAGACTCTTACCTTGAATATTCCAATTTCCTTGCCGACATCAATAATGAGCGCGTTCTCAAGAACCAAACATATAAATCCAACAT
GGAAAAATTGGAACGATTCGTAATGTATGTCTTTGAAGACGATACAACTGTCATCCCTAAGGAAAGTGGATGGTGGGCTGAAGTCAATGGCACG
GAAGTTACACCACTGAAAGAAAGAGCCATTTATAAAGAAGATTGGCTAGGGTTAAAGACATTGGATGAGGCCGGAAAATTAGTTTTCGAAACCA
TTCCAGGGGGACATATGACGTTAGGAGAGGAGATGCTAGAGAAGGCTTTCAAAGAGTACTTTGGTCCAGCAGGGAAGAAATTTGGG

>Bcin16g03460(MLST7), partial sequence [organism=Botrytis cinerea, strain N11_K_W06]
ATGAACCTCTTAATTTGAACTCTTCATTAATTGCAAGTGATGAGCCCCTATTCTGCCCAGAGGATAGTTACAAGACGTATATCATTAGTCGAGA
ACCACTCATGATATACATTGACGGATTTTTGAAAGCGAATGAAAGTAAACACTTGGTTGATGTTAGGTGTGTTATTTATTCTGATGAAATGAAC
AAGAGAGACTGATGAGATAGTGAACCGCTTTATGAACCGTCAACTGTTTCTCACGGACAGGAAGTTACCATTGATCCTTCAGTTCGGAATTCTG
AAGTGGCGGTTTTAGAGAGGGATGAGGTGGTCAGGTGTATTGAGCATAGAGCGAGGGCATTTCAGGGGTGGAGGGGCGAGATGGGGATTGAGAA
GTTGAGGACGCAGAGGTATGGGGTTGGAGGACATTATGGGATGCATTTGTAAGTTTTGGGGGATTGACGAAGGCCTCTGTTTATTTTCTATCAG
TACGGATCTTGAAAGAAAGATAGCATGAACACGAGGGCTAATAACTAGGAAATAGCGATTGGAGCGGAGGTAAACGTGGCATAGACCGATTTAG
TACTTTCATGGTCTATGTCGACGTATCCTCTGATATCGAAGGTGGAGGAACGGAATTCCCACGTATTGTGGGACCAAAAGGAGGAAGGTGGGAG
GACTTCCTGGAAACTACGGAAGCATTGGATCCAAGAACTGGAGAAAATGTAACAGTAGAAGGGGTGACATTCAAACCAATCAAGGGAAATGCCG
TATTCTGGGAAAATACTGACAACAACGGGAGGGGCTATGAT

>Bcin12g03020(MLST8), partial sequence [organism=Botrytis cinerea, strain N11_K_W06]
CGATTGGCTGCGAAGAAAACTGCGCAGCACCGAGTCACCAAAAGCACGAATAAACCATCGGCAAGGGAAATATTCTCTGAACCACAACATGACA
CGAGCGCGGAAGAGTATATCGGGCAAGAAGCTTCATCAAGAGCACCAAAGCGACAACGAGTCGATGATAATTATAACTCTTACGGTGGAAGAAA
TGAGAATACAGCAGCTTATGCTTCCGGGAAACTTCCATCTGGAAGTATAAATGTTGGTGGAGGTAGGAAGACCACTTTTCAAGAAGAACCTCGA
ACGGCATTTGTCGCTGGCAAGTTGCCCCCTGGAAGTATCAACATTGGTGGAAAGAAGGCTACCCAAATCGAGGACGAGGGTAGAGCAGCTTATG
CTTCCGGAAAATTGCCCCCAGGAAGTATAAACTCTGGCGCAAAGAAGGCGATTTCATTCCAAGATGAAACGAGACCGGCTTATGTCTCTGGAAA

GCTTCCACATGGTAGTATCGACGGTATGCGAAACCGTGAAATGGCTGCCGTCCACCGCGAAATTGCTGAGGGTGGGAGGAAACCAGGCCAGGTT
GTCTCTTCTCTATTCACATTCAATCCTACTTCAAAGAAAACTTTCGATGAACCCGAAGAACAAGCGGAACCGGCAAAACCATCCAATGCACCTT
TGACAGAGGAAATGGCGACTTTCACCAATCTAGGGTTATCGAGAAGGCTTGCAGCCCATCTATCGACTAAACTCGATATGAAAGCCCCGACCGC
CATTCAAAAAGCATCTGTGCAGCAGTTGGTATCGGACGATAGCGATGCTTTCATACAAGCAGAGACTGGATCTGGAAAAACTTTGGCATATCTA
CTACCTATAGTCGAGCGAATATTAGCATTGAGTGAGAATGGCGTACAAA

>Bcin02g07770(MLST9), partial sequence [organism=Botrytis cinerea, strain N11_K_W06]
CAGCTTTCCCTTTCGGTCTTGGCATCTACAGTCATTGCCATCCCTACACCATCACAACTTGAGTCTCGGGCCGTTATCGATTCCGATGCCGTTG
TAGGATTTGCCGAAACTGTTCCCAGTGGGACCGTAGGAACAGTTTACGAGGCATATAAACCATTCCTTAAAGTCGTAAATGGATGCGTACCATT
CCCTGCCGTCGATGCATCGGGTAACACAGGGTATGTCCTTATACCATTCTCTTCCACACGATTGCTATTGAGACTCTAACATATTTTAGTGGTG
GTTTGTCACCAACTGGCAGTAGCAATGGTGGTTGCAGCAGCAGTACCGGTCAAGTATATGTTCGAGGAGGACAAAGCGGATCAAACTACGCCAT
CATGTACTCCTGGTAAGTTCTCTCTAAACTTCTCCTTATAGATCCAACCTAACAAAATCTTAGGTACATGCCAAAGGACGAGCCCTCAACCGGT
ATTGGTCACCGTCACGATTGGGAAGGTGTAATTGTCTGGCTCTCAAGCGCCACCGCCACAACTGCCGACAACATCTTAGCCGTTTGTCCTTCCG
CCCACGGAGGCTGGGATTGTTCCACCGATGGCTATTCCCTTTCTGGTACCAGCCCTCTTATCAAGTACGAAAGTATCTGGCCCGTCGATCATTC
AATGGGTCTTACTAGTACTGTTGGTGGAAAACAACCTATGATTGCTTGGGAGTCTTTACCAACTGCTGCTCAAACTGCTCTTGAGAACACCGAT
TTCGGTGCTGCGAATGTTCCATTCATTCCGGCTGTTTTCACAGATAATCT

>Bcin04g02090(MLST10), partial sequence [organism=Botrytis cinerea, strain N11_K_W06]
CGGAGGATGATATGGCAAAGTCTATGATTACCAAAGCATTTGTAGGCATGAGTAGTAAACTGTCGAATATGACTATGAATGATGTTTACAAGCC
CTACATCCATGTAAGAAATGTAGAATAGAGAGATCAGTAACTGGAACTAATATCGTTTGCAGGCTTTCAAGTTACTTACGCAGTTCAACCCAAT
CACTACAGCTATTGCCGAATCCCCACTATTTCAAATGGCTGTCTCAGCAAATACCATCGAAAAGTACACACTGCTAGGCCCTTTCTTCAGAATA
TCTCCTCTGCAACAGGAAGTTACCAGGGAATACTTCAGTGCGCCAAAAACGATAGATAGGCGACACATTGCCACATCTCAAGATGCCTTACGAT
TGACCTTACAAACCCATCAAAAAGATTTACTTGATATCATCAACCACTTTGTTCGAGCAAGTCCAATCGCCAAAAGCAAAACCCTGGATTGGTT
CGCCTACATTGTGAATCAAAATCACAAGCGTCGAGCACTTCAGGTAGACCCGAAAGAAGTGTCTTCTGATGGTTTTATGCACAATGTCACTGTC
GTTCTAGATGGTCTTTGTGAGCCATTCATGGATACCACATTCTCAAAAATTTCGAAGATTGATATTGATTATCTAAGACGTGCGCCTCGTGTAG
ATATCAAGGACGAGACCAAGTTGAACGCTGATGAGAAGGCTTCTGAGAAGTATTATGAGGACACTGTTCCTGGCACTTCTAATTTCATCTCTGA
GGTCTTCTTTCTGACATTGGCTGCTCATCATTATGGTAGTGAAGCTCTTAATGCCACGCATAAGAGTCTGGAGAAAGACATCAAATATATTCAA
AAGCAATTGACTGCCGTTGAAGCA

>Bcin01g07220(MLST1), partial sequence [organism=Botrytis cinerea, strain N11_K_W08b]
ATGGCATATCTTGTTTCTTTTCGCATTATGCTTTGGAACGTGCTGGACAAGCTTGGGTTAGTGCTCTGCGTGTAGAAGCACTAAAGAAGATTCT
CGCACAACCGAAGCCATGGTTTGAGGAATCCAGGAATTCACCTAGCCGGTTGAACGAAGTTTTGGATAGGAACTCTGAGGAAATGCGTAATCTC
GTTGGCCGCTTTGCTGGTATTGTATTCACAGCATTTTTTATGCTATTGATATCAATCATTTGGGCTTTCGTGAATACATGGAAACTGACATTAG
TCTCAATGGCAACTGGGCCAGTTATATACGCTGTCACCAAAACGTTCAATCGCGTGAGTGGAAAATGGGAAAACAAGTGCAACTACGCATCTGA
AATGACCACTGGCATATTTTCAGAGACTTTCTCCAACATCAAAGTGGTTCGGGCTTTTACTCTGGAAACTTACTTTAAGACAAAACACACCAAA
GCTACAGAAGAACTCTATAAAGTTGGACTAATACGAGCAAACTACTCGGGATTGCTGTGGGGATTGACAGATGCAATGTCATTCTTCATCACTG
CAACTATCTTTTATTATGCCACGGTTCTCATTACCAAGAGAGAGATCAGTATCGCGGCTGCACTACAGACTGTCAATCTTCTATTATTTGGTAT
TTCTAATAGTACGAATATGCTGGCCATGATACCACAAATCAACTCTTCTCGCGTTACAGCTACGCATATGCTTGCATTAGCCAATCTCGATTCA
TCTTCCTCCCACGAAAATAAAGGAACCGAACGGCTTTCGACAATCTTTCCAATCAAATTCAACCGTCTTTCATTCACATACCCTACTCGTCCTG
AAAAACGAACGATATCATCCTTTTCTCTTTCCCTGATTCCTAACTCAACAACTGCACTTGTCGGACCCTCCGGCTCCGGAAAATCTACAATAGC
TGCTCTGCTCATTGGTCTCTATCCGCCAGATACTTCAACACCTCCACCGTTGACATTCAATCGCGTCTCCATAAGTAACTGTCACATTCCGTCT
CTCCGGGCTTCTCTCTCACTCGTTCCACAAATACCGATTCTATTTCCAGCTACCATTCTCCATAACATCATTTATGGTCTCCCAGAATCTTCTC
CTTGTGCTAGTCTTCCATCTGCTATTGCATCAGCAAAAGATGCTGGGATCCATGAATTTATCACATCGCTTCCACAAGGTTATGATACTATG

>Bcin05g07690(MLST2), partial sequence [organism=Botrytis cinerea, strain N11_K_W08b]
ACCACTATCACCAAGTCCTTTTCTGCACTTTCCTCATCAAAAACGAACGAGAACATCATCAATAAATACTTTGCTGAAGTAATTGGGTTTTATT
TTGGCCAGGATGCCTTCTCTCTACGACAGCAAGCCTTCGATGATATGTTGTGGGTAGTTCTTGGCTGGCTGGATACTGTCAAATTCATTGATTT
ACATTCTGAATTGCACTATTCAAACGACTCTCAGCCAGAATGGTACGGACAACAATATAAACCTGCATTTGCACATCGAGCGCGACTATTTTGG
GAATTGGCTTCACAAGGATGGGATACTACTCTCTGTGGTGGTGGGATGATATGGTCACCATACCTTACTCCATACAAGAACGCAATTACCAATG
AACTCTATATCGCAGCTTCGATATCGATGTACCTATATTTCCCCGGAGATGACAATCAATCCCCATTTATGCTTTCCAACCCTTCATATCCACC
TCACGATCCGAAATATCTACAGGCAGCTGTTGATGCTTACAAATGGCTGAATGGTTCCAACATGACGGATTTACAAGGATTATATGTCGACGGG
TACCATATCTCGAATCTTTCTGGCGGTGAAAACACCCATTGCGATTCTAGAAATGAGATGGTATATACCTACAATCAAGGTGTTTTGCTTACTG
GACAACGTGGTTTGTATGACGCAACCGCCGCACGATCATACCTTGTAGATGGCCACAAACTCATCGCGAATGTTATTAATGCCACAGGCTATGA
CCTGAAACACAATGTTGTCATCTCACCGCCACCCAAAGATGGTTCCGCATTGGCAAAGTGGTTTGGCCTGGGTAGGAATGGAATACTGGAAGAA
GGATGCGATTCAAGTGCTTCGTGTTCTCAAAATGGACAAACTTTCAAAGGCATATTCTTTCATCACTTGATTGCGTTCTGTAGTGATTTGCCAG
GGGAGCCTATTGCAGGGACGAAGGAAAGCTTAGAACTCGACAGAGTGTGGCATTCTGACAAATGCTCACAGTATACAAAAT

>Bcin06g01710(MLST3), partial sequence [organism=Botrytis cinerea, strain N11_K_W08b]
GTGAGTCTGACTTTTGTATTTGAGCGTTAAGATAGACACTGATATACCAAGGCAATCACCTAATTTCATGGCGTTCTTCCCTGCATCATCTACC
TACCCTGGAATGCTGGGGAATTATACTCAGCAGCTTTCACAGCACCTGCTTTCAATTGGATCTGTTCCCCTGCTGTGACAGAATTGGAGACGGT
TGTAATGGATTGGCTGGCCAAGCTTCTCAATCTCCCAGACTGTTATTTGTCTCCGACTCATGGTGGTGGTGTCATCCAAGGATCAGCCTCGAAG
CTATCGTTACCGTTATGGTTGCTGCCCGCGATAAATATCTTCGTGAAACTACTGAAGGTCTGTCGGGCATTGAGCTCGAGGATGCGATTGCATA
TAAGAGGAGTAAGCTAGTTGCACTAGGAAGCGAAATGGCACACAGCTCCACGCAGAAAGCAGCGCAAATAGCTGGCGTTAGATTCGATCGATTC
CAGTACTCGCATCCGATGATTTCGCCATAACGGGTGATGATTTAGAGAAGGTATTGAAAGAATGCAAATCTCAAGGATTGGAACCCTTCTATCT
AACTTCGACTTTGGGAACAACATCTACATGCGCAGTTGACGACTTCGCATCTATTGCAACAGTACTTTCAAAATATGCACCTCCAGATGTTGCA
GGCGAGATCTGGGTTCACGTCGATGCTGCTTATGCAGGTGCAGCTTTGGTTTGCCCTGAATACCGTCATCTAACATCGTCCTTCCAGCATTTCC
ATTCCTTCGATATGGACATGCACAAATGGCTTCTGACAAATTTCGACGCTTCTTGTCTATATGTCAAGAAACGCAAAGATCTGATCGATGCACT
CTCCATAACACCAAGTTATCTTCGCCAACGAGTTTTCAGAGAGTGGACTCGTAACCGACTATCGGGACTGGCAAATTCCTCTCGGAAGACGCTT
CCGAAGCTTAAAGATTTGGTTTGTCCTCAGAACCTA

>Bcin09g03030(MLST4), partial sequence [organism=Botrytis cinerea, strain N11_K_W08b]
ACCCTTCAACCCCACCGCAGCTCCTATACCGAAAGCGAGTATCCTACCAATTCTCCTTCCACCTGCGACTTTAAGACCATTGGCTTTCCGCACT
TTCACAAAAAAGCATAGTTTAACATTGACGTCGTCGGCATTACAAGTGTTGGCTACTTTTATTGGAAAGCATTGTGGGACAGGATGGAGGGAAG
AAGGACTGGCAGAGAGAGTCTTGGAGGAGGTCGCCAAGAGTTGGAAGAATAGGAGTGGCGGTGTCATTGTCGAGGGCGAGGGAACGGAATTGAA
GGAGATTCTGAAAGCTTTGGAAGGGAATATGAGTGGTGGAAGGATAGTCATAGGAAGAGAGCTAAGCCGGCAGAATAGTTTAGTACTGGGATCA
TCACAATATGGAGAGGTCAATCATACAAGACTTGGGCTACGGCCAGGGAATATACCCAGAGAGGATAGTCAGTCAAGTTTGGGAATGTCAACGT
TGGAGGTCAATGACGAGGAAGATGAGGATGGCCTGATGGATCCAAGAAGGTGGTTAAAAGTCATTGATGCATTTGAGCAACCTCGACTGGTGTA
CAATGTTGCTAAAAAGCACTTTGATAGGTATGTTTCAATGATAAAATTTTATCTGAATCGACTAACTCAGTACAGAGATACCTCCAAACCTTCA
TTGTTCCCACCTGCGTCTCATAAAACACTCCTCTTCCAAAACCGCTATAATGTTATCCATCAACGTCTCCTTCGCAATGAATCTTTTCAAACGC
CCGCTTTTCAAGGTGGCAAATCTTCCCTTCAACGCAGCACGTCCGCCATTACCACCCAACAACAATCATACAAATTAACGCCGATAGCTAATCT
TCTCGGTCGCAATCGCAGCTCTCATATGCTTCTCGGTCTCCTCAGTATTTCACCCACTGGTACCCTCGCCATCAATGACCTGACGGGCAGTATC
GCTCTCGATCTTACACACGCAGCAGCCATTCC

>Bcin11g01310(MLST5), partial sequence [organism=Botrytis cinerea, strain N11_K_W08b]
ACTGACATGGACCTCATATGGAACCGGCGTAGAATGCGCAACGCTTGAAGTTCCGCTCGAATATGGCGATGCAACGTCGACGGCAAAAGCCAGT
GTTGCGCTTGCTCGTTATCCTGCCACTGTTGCCGCGAGCAAGAAGCTCGGGTCTCTCTTGATAAACCCCGGTGGACCCGGTGCCTCTGGTGTTG
GCTTTGTGCAGTCTGGAGCCGGTGCCGCCGTCTCGACACTGAGTGGTGGATTGTACGATATCATCGGATGGGATCCACGTGGAACCGGTGCTTC
GGCTCCTATTTTGGAATGTTTTGCAAATGCCAGTGCGGAGTATGATTTTAACAACGCGCTTCCATCTGCTCCGAATCTCTGGCTCGGACAATTT
GCGAATGCCAGCGCAAATTCTGCTGTTAGCTCTGCTATCACATCCTTTGACACTTCTGTCGCTGCTCTTGCAAAAGCTTGCGTGGCTCAGAAAT
CTCCCGCTCTTTACACCTCAACAGCAGCATATGTTGCTCGAGACATGGCAGCGATAGTCGATGCATTGGATGGGACCTCTGCAAAACTTAACTA
CTGGGGTTTCTCATATGGAACTATTTTCCTAGCTGAGTTTATCCAAACTTTCCCAGGCCGTGTGGGAAGAGTTCTTGCCGATGGTGTTTTCGAC
GCAAAGGCAAATGCACTCACATACGTTAGCCAACTTCCCAACGATCAACTCAGTGTTCGTGCTTCGTTGAACGATTTTGCAGCTTTCTGCACCA
CCGCCGGTAGTAAAGGTTGCTCTTTTGCCACCGCCCCTACTGGAACCTCAGGTACTGTTGCTACCAGACTGGACAACATAATGAAGGATATGTT
CCTCAATCCTATTGTTGCTTCGGGCTTAAGCATCAG

>Bcin15g03910(MLST6), partial sequence [organism=Botrytis cinerea, strain N11_K_W08b]
GCCAAAACACAAAATCATCCAACGATGAAGATGATACTCCACTTCCCTTGATTATTTGGCATGGACTCGGCGATAATTACAAAGCGGATGGTCT
TGCGCAAGTTGGAAAACTAGCTGAAGCTATTCATCCTGGGACTTTTGTCTACAATATTCATGTAGATGAGGATGCATCTGCAGATAGGACAGCT
ACCTTCTTTGGAAATCTCACTCGTGAGTACATCCCCTATTTTCCCTTTAAATACCATACTAACTCTCCTACCAAGTTCAAATCGAAAAGGTCTG
CGAAGACCTCGCCTCCCATCCTATTCTCTCTACCGCGCCCGCCGTCGACGCAATTGGATTCTCCCAAGGCGGCCAATTCTTGCGCGGTTACATA
TCCCGCTGCAATGCTCCACCCATCCGCTCTCTCCTGACCTTCGGTTCCCAACACAACGGCATTTCTGCCTTCCAAGCCTGTGGTCCTGCCGATT
TCCTCTGTCGCGGTGCTCAAACCCTTTTGCGATCCAACACCTGGTCAACCTTTGTCCAATCTCGTCTCGTACCCGCTCAATACTTCAGAGATCC
GGAAAACCTAGACTCTTACCTTGAATATTCCAATTTCCTTGCCGACATCAATAATGAGCGCGTTCTCAAGAACCAAACATATAAATCCAACATG
GAAAAATTGGAACGATTCGTAATGTATGTCTTTGAAGACGATACAACTGTCATCCCTAAGGAAAGTGGATGGTGGGCTGAAGTCAACGGCACGG
AAGTTACACCACTGAAAGAAAGAGCCATTTATAAAGAAGATTGGCTAGGTTTAAAGACATTGGATGAGGCCGGAAAATTAGTTTTCGAAACCAT
TCCAGGGGGACATATGACGTTAGGAGAGGAGATGCTAGAGAAGGCTTTCAAAGAGTATTTTGGTCCAGCAGGGAAGAAATTTGGG

>Bcin16g03460(MLST7), partial sequence [organism=Botrytis cinerea, strain N11_K_W08b]
CATGAACCTCTTGATTTGAACTCTTCATTAATTGCAAGTGATGAACCCCTATTCTGCCCAGAGGATAGTTACAAGACGTATATCATTAGTCGAG
AACCACTCATGATATATATTGACGGATTTTTGAAAGCGAATGAAAGTAAACATTTGGTTGATGTTAGGTGTGTTATTTATTCTGATGAAATGAA
CAAGAGAGACTGATGAGATAGTGAGCCGCTTTATGAACCGTCAACTGTTTCTCACGGACAGGAAGTTACCATTGATCCTTCAGTTCGGAATTCT
GAAGTGGCGGTTTTAGAGAGGGATGAGGTGGTCAGGTGTATTGAGCATAGAGCGAGGGCATTTCAGGGGTGGAGGGGCGAGATGGGGATTGAGA
AGTTGAGGACGCAGAGGTATGGGGTTGGAGGACATTATGGGATGCATTTGTAAGTTTTGGGGGATTGACGAAGGCTTCTGTCTATTTTCTATCA
GCACGGATCTTGAAAGAAAGATAGCATGAACACGCGGGCTAATAACTAGGAAATAGCGATTGGAGCGGAGGTAAACGTGGCATAGACCGATTTA

GTACTTTCATGGTCTATGTCGACGTATCCTCTGATATCGAAGGTGGAGGAACGGAATTCCCACGTATCGTGGGACCAAAAGGAGGAAGGTGGGA
GGACTTCCTGGAAACTACGGAAGCATTGGATCCAAGAACTGGAGAAAATGTAACAGTAGAAGGGGTGACATTCAAACCAATCAAGGGAAATGCC
GTATTCTGGGAAAATACTGACAACAACGGGAGGGGCTATGAT

>Bcin12g03020(MLST8), partial sequence [organism=Botrytis cinerea, strain N11_K_W08b]
CGATTGGCTGCGAAGAAAACTGCGCAGCACCGAGTCACCAAAAGCACGAATAAACCATCGGCAAGGGAAATATTCTCTGAACCACAACATGACA
CGAGCGCGGAAGAGTATATCGGGCGAGAAGCTTCATCAAGAGCACCAAAGCGACAACGAGTCGATGATAATTATAACTCTTACGGTGGAAGAAA
TGAAAATACAGCAGCTTATGCTTCCGGGAAACTTCCATCTGGAAGTATAAATGTTGGTGGAGGTAGGAAGACCACTTTTCAAGAAGAACCACGA
ACGGCATTTGTCGCTGGCAAGTTGCCCCCTGGAAGTATCAACATTGGTGGAAAGAAGGCTACCCAAATCGAGGACGAGGGTAGAGCAGCTTATG
CTTCCGGAAAATTGCCTCCAGGAAGTATAAACTCTGGCGCAAAGAAGGCGATTTCATTCCAAGATGAAACGAGACCGGCTTATGTCTCCGGAAA
GCTTCCACATGGTAGTATCGACGGTATGCGAAACCGTGAAATGGCTGCCGTCCACCGCGAAATTGCTGAGGGTGGAGGAAACCAGGCCAGGTT
GTCTCTTCTCTATTCACATTCAATCCTACTTCAAAGAAAACTTTTGATGAACCCGAAGAACAAGCGGAACCGGCAAAACCATCCAATGCGCCTT
TGACAGAGGAAATGGCGACTTTCACCAATCTAGGGTTATCGAGAAGGCTTGCAGCCCATCTATCGACTAAACTCGATATGAAAGCTCCGACCGC
CATTCAAAAAGCATCTGTACAGCAGTTGGTATCGGACGATAGCGATGCTTTCATACAAGCAGAGACTGGATCTGGAAAAACTTTGGCATATCTA
CTACCTATAGTCGAGCGAATATTAGCATTGAGTGAGAATGGCGTACAAA

>Bcin02g07770(MLST9), partial sequence [organism=Botrytis cinerea, strain N11_K_W08b]
CAGCTTTCCCTTTCGGTCTTGGCATCTACAGTCATTGCCATCCCTACACCATCACAACTTGAGTCTCGGGCCGTTATCGATTCCGATGCCGTTG
TAGGATTTGCCGAAACTGTTCCCAGTGGGACCGTAGGAACAGTTTATGAGGCATATAAACCATTCCTTAAAGTCGTAAATGGATGCGTACCATT
CCCTGCCGTCGATGCATCGGGTAACACAGGGTATGTCCTTATATCTTTCTCTTCCACACGATTGCTATTGAGTCTCTAACATATTTTAGTGGTG
GTTTGTCACCAACTGGCAGTAGCAATGGTGGTTGCAGCAGCAGTACCGGTCAAGTATATGTTCGAGGAGGACAAAGCGGATCAAACTACGCCAT
CATGTACTCCTGGTAAGTTCTCTCTAAACTTCTCCTTATAGATCCAACCTAACAAAATCTTAGGTACATGCCAAAGGACGAGCCCTCAACCGGT
ATTGGTCACCGTCACGATTGGGAAGGTGTAATTGTCTGGCTCTCCAGCGCCACCGCCACAACTGCCGACAACATCTTAGCCGTTTGTCCTTCCG
CCCACGGAGGCTGGGATTGTTCCACGGATGGCTATTCCCTTTCTGGTACCAGCCCTCTTATCAAGTACGAAAGTATCTGGCCCGTCGATCACTC
AATGGGTCTTACTAGTACTGTTGGTGGAAAACAACCTATGATTGCTTGGGAGTCTTTACCAACTGCTGCTCAAACTGCTCTTGAGAACACCGAT
TTCGGTGCTGCGAATGTTCCATTCATTCCGGCTGTTTTCACAGACAATCT

>Bcin04g02090(MLST10), partial sequence [organism=Botrytis cinerea, strain N11_K_W08b]
CGGAGGATGATATGGCAAAGTCTATGATTACCAAAGCATTTGTAGGCATGAGTAGTAAACTGTCGAATATGACTATGAATGATGTTTACAAGCC
CTACATCCATGTAAGAAATGTAGAATAGAGAGATCAGTAACTGGAACTAATATCGTTTGTAGGCTTTCAAGTTACTTACGCAGTTCAACCCAAT
CACTACAGCTATTGCCGAATCCCCACTATTTCAAATGGCTGTCTCAGCAAATACCATCGAAAAGTACACACTGCTAGGCCCTTTCTTCAGAATA
TCTCCTCTGCAACAGGAAGTTACCAGGGAATACTTCAGTGCGCCAAAGACGATAGATAGGCGACACATTGCCACATCTCAAGATGCCTTACGAT
TGACCTTACAAACCCATCAAAAAGATTTACTTGATATCATCAACCACTTTGTTCGAGCAAGTCCAATCGCCAAAAGCAAAACCTTGGATTGGTT
CGCCTACATTGTGAATCAAAATCACAAGCGTCGAGCACTTCAGGTAGACCCGAAAGAAGTGTCTTCTGATGGCTTTATGCACAATGTCACTGTC
GTTCTAGATGGTCTTTGTGAGCCATTCATGGATACCACATTCTCGAAAATTTCGAAGATTGATATTGATTATCTAAGACGTGCGCCTCGTGTAG
ATATCAAGGACGAGACCAAGTTGAACGCTGACGAGAAGGCTTCTGAGAAGTATTATGAGGACACTGTTCCTGGCACTTCTAATTTCATCTCTGA
GGTCTTCTTTCTCACATTGGCTGCTCATCATTATGGTAGTGAAGCTCTTAATGCCACGCATAAGAGTCTGGAGAAAGACATCAAATATATTCAA
AAGCAATTGACTGCCGTTGAAGCAG

>Bcin01g07220(MLST1), partial sequence [organism=Botrytis cinerea, strain N11_K_W11]
ATGGCATATCTTGTTTCTTTTCGCATTATGCTTTGGAACGTGCTGGACAAGCTTGGGTTAGTGCTCTGCGTGTAGAAGCACTAAAGAAGATTCT
CGCACAACCGAAGTCATGGTTTGAGGAATCCAGGAATTCACCTAGCCGGTTGAACGAAGTTTTGGATAGGAACTCTGAGGAAATGCGTAATCTC
GTTGGCCGCTTTGCTGGTATTGTATTCACAGCATTTTTTATGCTATTGATATCAATCATTTGGGCTTTTGTGAATACATGGAAACTGACATTAG
TCTCGATGGCAACTGGGCCAGTTATATACGCTGTCACCAAAACATTCAATCGCGTGAGTGAAAAATGGGAAAACAAGTGCAACTACGCCTCTGA
AATGACCACTGGCATATTTTCAGAGACTTTCTCCAACATCAAAGTGGTTCGGGCTTTTACTCTGGAAACTTACTTTGAGACAAAACACACCAAA
GCTACAGAAGAACTTTATAAAGTTGGACTAATACGAGCAAACTACTCGGGATTGCTGTGGGGATTGACAGATGCGATGTCATTCTTCATCACTG
CAACTATCTTTTATTATGCCACGGTTCTCATTACCAAGAGAGAGATCAGTATCGCGACTGCACTACAGACTGTCAATCTTCTATTATTTGGTAT
TTCTAATAGTACGAATATGCTGGCCATGATACCACAAATCAACTCTTCTCGCGTTACAGCTACGCATATGCTTGCATTAGCCAATCTCGATTCA
TCTTCCTCCCACGAAAATAAAGGAACCGAACGGCTTTCGACAATCTTTCCAATCAAATTCAACCGTCTTTCATTCACATACCCTACTCGTCCTG
AAAAACGAACGATATCATCCTTTTCTCTTTCCCTGATTCCTAACTCAACAACTGCACTTGTCGGACCCTCCGGCTCCGGAAAATCTACAATAGC
TGCTCTGCTCATTGGTCTCTATCCGCCAGATACTTCAACACCTCCACCGTTGACATTCAATCGCGTCTCCATAAGTAACTGTCACATTCCGTCT
CTCCGGGCTTCTCTCTCACTCGTTCCACAAATACCGATTCTATTTCCAGCTACCATTCTCCATAACATCATTTATGGTCTCCCAGAATCTTCTC
CTTGTGCTAGTCTTCCATCAGCTATTGCATCAGCAAAAGATGCTGGGATCCATGAATTTATCACATCGCTTCCACAAGGTTATGATACTAT

>Bcin05g07690(MLST2), partial sequence [organism=Botrytis cinerea, strain N11_K_W11]
ACCACTATCACCAAGTCCTTTTCTGCACTTTCCTCATCAAAAACGAACGAGAACATCATCAATAAATACTTTGCTGAAGTAATTGGGTTTTATT
TTGGCCAGGATGCCTTCTCTCTACGACAGCAAGCCTTCGATGATATGTTGTGGGTAGTTCTTGGCTGGCTGGATACTGTCAAATTCATTGATTT

ACATTCTGAATTGCACTATTCAAACGACTCTCAGCCAGAATGGTACGGACAACAATATAAACCTGCATTTGCACATCGAGCGCGACTATTTTGG
GAATTGGCTTCACAAGGATGGGATACTACTCTCTGTGGTGGTGGGATGATATGGTCACCATACCTTACTCCATACAAGAACGCAATTACCAATG
AACTCTATATCGCAGCTTCGATATCGATGTACCTATATTTCCCCGGAGATGACAATCAATCCCCATTTATGCTTTCCAACCCTTCATATCCACC
TCACGATCCGAAATATCTACAGGCAGCTGTTGATGCTTACAAATGGCTGAATGGTTCCAACATGACGGATTTACAAGGATTATATGTCGACGGG
TACCATATCTCGAATCTTTCTGGCGGTGAAAACACCCATTGCGATTCTAGAAATGAGATGGTATATACCTACAATCAAGGTGTTTTGCTTACTG
GACAACGTGGTTTGTATGACGCAACCGCCGCACGATCATACCTTGTAGATGGCCACAAACTCATCGCGAATGTTATTAATGCCACAGGCTATGA
CCTGAAACACAATGTTGTCATCTCACCGCCACCCAAAGATGGTTCCGCATTGGCAAAGTGGTTTGGCCTGGGTAGGAATGGAATACTGGAAGAA
GGATGCGATTCAAGTGCTTCGTGTTCTCAAAATGGACAAACTTTCAAAGGCATATTCTTTCATCACTTGATTGCGTTCTGTAGTGATTTGCCAG
GGGAGCCTATTGCAGGGACGAAGGAAAGCTTAGAACTCGACAGAGTGTGGCATTCTGACAAATGCTCACAGTATACAAAATGG

>Bcin06g01710(MLST3), partial sequence [organism=Botrytis cinerea, strain N11_K_W11]
GTGAGTCTGACTTTTGTATTTGAGCGTTAAGATAGACACTGATGTACCAAGGCAATCACCTAATTTCATGGCGTTCTTCCCTGCATCATCTACC
TACCCTGGAATGCTGGGAGAATTATACTCAGCAGCTTTCACAGCACCTGCTTTCAATTGGATCTGTTCCCCTGCTGTGACAGAATTGGAGACGG
TTGTAATGGATTGGCTGGCCAAGCTTCTCAATCTCCCAGACTGTTATTTGTCTTCGACTCATGGTGGTGGTGTCATCCAAGGATCAGCCTCGGA
AGCTATCGTTACCGTTATGGTTGCTGCCCGCGATAAATATCTTCGTGAAACTACTGAAGGTCTGTCGGGCATTGAGCTCGAGGATGCGATTGCA
TATAAGAGGAGTAAGCTAGTTGCACTAGGAAGCGAAATGGCACACAGCTCCACGCAGAAAGCAGCGCAAATAGCTGGCGTTAGATTCCGATCGA
TTCCAGTACTCGCATCCAATGATTTCGCCATGACGGGTGATGATTTAGAGAAGGTATTGAAAGAATGCAAATCTCAAGGATTGGAACCCTTCTA
TCTAACTTCGACTTTGGGAACAACATCTACATGCGCAGTTGACGACTTCGCATCTATTGCAACAGTACTTTCAAAATATGCACCTCCAGATGTT
GCAGGCGAGATCTGGGTTCACGTCGATGCTGCTTATGCAGGTGCAGCTTTGGTTTGCCCTGAATACCATCATCTAACATCGTCCTTCCAGCATT
TCCATTCCTTCGATATGAACATGCACAAATGGCTTCTGACAAATTTCGACGCTTCTTGTCTATATGTCAAGAAACGCAAAGATCTGATCGATGC
ACTCTCCATAACACCAAGTTATCTTCGCAACGAGTTTTCAGAGAGTGGACTCGTAACCGACTATCGGGACTGGCAAATTCCTCTCGGAAGACGC
TTCCGAAGCTTAAAGATTTGGTTTGTCCTCAGAACCTAC

>Bcin09g03030(MLST4), partial sequence [organism=Botrytis cinerea, strain N11_K_W11]
GACCCTTCAACCCCACCGCAGCTCCTATACCGAAAGCGAGTATCCTACCAATTCTCCTTCCACCTGCGACTTTAAGACCATTGGCTTTCCGCAC
TTTCACAAAAAAGCATAGTTTAACATTGACGTCGTCGGCATTACAAGTGTTGGCTACTTTTATTGGAAAGCATTGTGGGACAGGATGGAGGGAA
GAAGGACTGGCAGAGAGAGTCTTGGAGGAGGTCGCCAAGAGTTGGAAGAATAGGAGTGGCGGTGTCATTGTCGAGGGCGAGGGAACGGAATTGA
AGGAGATTCTGAAAGCTTTGGAAGGGAATATGAGTGGTGGAAGGATAGTCATAGGAAGAGAGCTAAGCCGGCAGAATAGTTTAGTACTGGGATC
ATCACAATATGGAGAGGTCAATCATACAAGACTTGGGCTACGGCCAGGGAATATACCCAGAGAGGATAGTCAGTCAAGTTTGGGAATGTCAACG
TTGGAGGTCAATGACGAGGAAGATGAGGATGGCCTGATGGATCCAAGAAGGTGGTTAAAAGTCATTGATGCATTTGAGCAACCTCGACTGGTGT
ACAATGTTGCTAAAAAGCACTTTGATAGGTATGTTTCAATGATAAAATTTTATCTGAATCGACTAACTCAGTACAGAGATACCTCCAAACCTTC
ATTGTTCCCACCTGCGTCTCATAAAACACTCCTCTTCCAAAACCGCTATAATGTTATCCATCAACGTCTCCTTCGCAATGAATCTTTTCAAACG
CCCGCTTTTCAAGGTGGCAAATCTTCCCTTCAACGCAGCACGTCCGCCATTACCACCCAACAACAATCATACAAATTAACGCCGATAGCTAATC
TTCTCGGTCGCAATCGCAGCTCTCATATGCTTCTCGGTCTCCTCAGTATTTCACCCACTGGTACCCTCGCCATCAATGACCTGACGGGCAGTAT
CGCTCTCGATCTTACACACGCAGCAGCCATTC

>Bcin11g01310(MLST5), partial sequence [organism=Botrytis cinerea, strain N11_K_W11]
ACTGACATGGACCTCATGTGGAACCGGCGTAGAATGCGCAACGCTTGAAGTTCCGCTCGAATATGGCGATGCAACGTCAACGGCAAAAGCCAGT
GTTGCGCTTGCTCGTTATCCTGCCACTGTTGCCGCGAGCAAGAAGCTCGGGTCTCTCTTGATAAACCCCGGTGGACCCGGTGCCTCTGGTGTTG
GCTTTGTGCAGTCTGGAGCCGGTGCCGCCGTCTCGACACTGAGTGGTGGATTGTACGATATCATCGGATGGGATCCACGTGGAACCGGTGCTTC
GGCTCCTATTTTGGAATGTTTTGCAAATGCCAGTGCGGAGTATGATTTTAACAACGCGTTTCCATCTGCTCCGAATCTCTGGCTCGGACAATTT
GCGAATGCCAGCGCAAATTCTGCTGTTAGCTCTGCTATCACATCCTTTGACACTTCTGTCGCTGCTCTTGCAAAAGCTTGCGTGGCTCAGAAAT
CTCCCGCTCTTTACACCTCAACAGCAGCATATGTTGCTCGAGACATGGCAGCGATAGTCGATGCATTGGATGGGACCTCTGCAAAACTTAACTA
CTGGGGTTTCTCATATGGAACTATCTTCCTAGCTGAGTTTATCCAAACTTTCCCAGGCCGCGTGGGAAGAGTTCTTGCCGATGGTGTTTTCGAC
GCAAAGGCAAATGCACTCACATACGTTAGCCAACTTCCCAACGATCAACTCAGTGTTCGTGCTTCGTTGAACGATTTTGCAGCTTTCTGCACCA
CCGCCGGTAGTAAAGGTTGCTCTTTTGCCACCGCCCCTACTGGAACCACAGGTACTGTTGCTACCAGACTGGACAACATAATGAAGGATATGTT
CCTCAATCCTATTGTTGCTTCGGGCTTAAGCATCA

>Bcin15g03910(MLST6), partial sequence [organism=Botrytis cinerea, strain N11_K_W11]
GCCAAAACACAAAATCATCCAACGATGAAGATGATACTCCACTTCCCTTGATTATCTGGCATGGACTCGGCGATAATTACAAAGCGGATGGTCT
TGCGCAAGTTGGAAAACTAGCTGAAGCTATTCATCCTGGGACTTTTGTCTACAATATTCATGTAGATGAGGATGCATCTGCAGATAGGACAGCT
ACCTTCTTTGGAAATCTCACTCGTGAGTACATCCCCTATTTTTCCTTTAAATATCATACTAACTCTCTTACCAAGTTCAAATCGAAAAGGTCTG
CGAAGACCTCGCCTCCCATCCTATTCTCTCTACCGCGCCCGCCGTCGACGCAATTGGATTCTCCCAAGGCGGCCAATTCTTGCGTGGTTACATA
TCCCGCTGCAATGCTCCACCCATCCGCTCTCTCCTGACCTTCGGTTCCCAACACAACGGCATTTCTGCCTTCCAAGCCTGTGGTCCTGCCGATT
TCCTCTGTCGCGGTGCTCAAACCCTTTTGCGATCCAACACCTGGTCAACCCTTTGTCCAATCTCGTCTCGTACCCGCTCAATACTTCAGAGATCC
GGAAAACCTAGACTCTTACCTTGAATATTCCAATTTCCTTGCCGACATCAATAATGAGCGCGTTCTCAAGAACCAAACATATAAATCCAACATG
GAAAAATTGGAACGATTCGTAATGTATGTCTTTGAAGACGATACAACTGTCATCCCTAAGGAAAGTGGATGGTGGGCTGAAGTCAACGGCACGG

AAGTTACACCACTGAAAGAAAGAGCCATTTATAAAGAAGATTGGCTAGGTTTAAAGACATTGGATGAGGCCGGAAAATTAGTTTTCGAAACCAT
TCCAGGGGGACATATGACGTTAGGAGAGGAGATGCTAGAGAAGGCTTTCAAAGAGTATTTTGGTCCAGCAGGGAAGAAATTTGGG

>Bcin16g03460(MLST7), partial sequence [organism=Botrytis cinerea, strain N11_K_W11]
CATGAACCTCTTAATTTGAACTCTTCATTATTTGCAAGTGATGAACCCCTATTCTGCCCAGAGGATAGTTACAAGACGTATATCATTAGTCGAG
AACCACTCATGATATATATTGACGGATTTTTGAAAGCGAATGAAAGTAAACATTTGGTTGATGTTAGGTGTGTTATTTATTCTGATGAAATGAA
CAAGAGAGACTGATGAGATAGTGAGCCGCTTTATGAACCGTCAACTGTTTCTCACGGACAGGAAGTTACCATTGATCCTTCAGTTCGGAATTCT
GAAGTGGCGGTTTTAGAGAGGGATGAGGTGGTCAGGTGTATTGAGCATAGAGCGAGGGCATTTCAGGGGTGGAGGGGCGAGATGGGGATTGAGA
AGTTGAGGACGCAGAGGTATGGGGTTGGAGGACATTATGGGATGCATTTGTAAGTTTTGGGGGATTGACGAAGGCTTTTGTCTATTTTCTACCA
GTACGGATCTTGAAAGAAAGATAGCATGAACACGAGGGCTAATAACTAGGAAATAGCGATTGGAGCGGAGGTAAACGTGGCATAGACCGATTTA
GTACTTTCATGGTCTATGTCGACGTATCCTCTGATATCGAAGGTGGAGGAACGGAATTCCCACGTATCGTGGGACCAAAAGGAGGAAGGTGGGA
GGACTTCCTGGAAACTACGGAAGCATTGGATCCAAGAACTGGAGAAAATGTAACAGTAGAAGGGGTGACATTCAAACCAATCAAGGGAAATGCC
GTATTCTGGGAAAATACTGACAACAACGGGAGGGGCTATGAT

>Bcin12g03020(MLST8), partial sequence [organism=Botrytis cinerea, strain N11_K_W11]
CGATTGGCTGCGAAGAAAACTGCGCAGCACCGAGTCACCAAAAGCACGAATAAACCATCGGCAAGGGAAATATTCTCTGAACCACAACATGACA
CGAGCGCGGAAGAGTATATCGGGCGAGAAGCTTCATCAAGAGCACCAAAGCGACAACGAGTCGATGATAATTATAACTCTTACGGTGGAAGAAA
TGAAAATACAGCAGCTTATGCTTCCGGGAAACTTCCATCTGGAAGTATAAATGTTGGTGGAGGTAGGAAGACCACTTTTCAAGAAGAACCACGA
ACGGCATTTGTCGCTGGCAAGTTGCCCCCTGGAAGTATCAACATTGGTGGAAAGAAGGCTACTCAAATCGAGGACGAGGGTAGAGCAGCTTATG
CTTCCGGAAAATTGCCTCCAGGAAGTATAAACTCTGGCGCAAAGAAGGCGATTTCATTCCAAGATGAAACGAGACCGGCTTATGTCTCCGGAAA
GCTTCCACATGGTAGTATCGACGGTATGCGAAACCGTGAAATGGCTGCCGTCCACCGCGAAATTGCTGAGGGTGGAGGAAACCAGGCCAGGTT
GTCTCTTCTCTATTCACATTCAATCCTACTTCAAAGAAAACTTTTGATGAACCCGAAGAACAAGCGGAACCGGCAAAACCATCCAATGCGCCTT
TGACAGAGGAAATGGCGACTTTCACCAATCTAGGGTTATCGAGAAGGCTTGCAGCCCATCTATCGACTAAACTCGATATGAAAGCTCCGACCGC
CATTCAAAAAGCATCTGTACAGCAGTTGGTATCGGACGATAGCGATGCTTTCATACAAGCAGAGACTGGATCTGGAAAAACTTTGGCATATCTA
CTACCTATAGTCGAGCGAATATTAGCATTGAGTGAGAATGGCGTACAAA

>Bcin02g07770(MLST9), partial sequence [organism=Botrytis cinerea, strain N11_K_W11]
CAGCTTTCCCTTTCGGTCTTGGCATCTACAGTCATTGCCATCCCTACACCATCACAACTTGAGTCTCGGGCCGTTATCGATTCCGATGCCGTTG
TAGGATTTGCCGAAACTGTTCCCAGTGGGACCGTAGGAACAGTTTATGAGGCATATAAACCATTCCTTAAAGTCGTAAATGGATGCGTACCATT
CCCTGCCGTCGATGCATCGGGTAACACAGGGTATGTCCTTATATCTTTCTCTTCCACACGATTGCTATTGAGTCTCTAACATATTTTAGTGGTG
GTTTGTCACCAACTGGCAGTAGCAATGGTGGTTGCAGCAGCAGTACCGGTCAAGTATATGTTCGAGGAGGACAAAGCGGATCAAACTACGCCAT
CATGTACTCCTGGTAAGTTCTCTCTAAACTTCTCCTTATAGATCCAACCTAACAAAATCTTAGGTACATGCCAAAGGACGAGCCCTCAACCGGT
ATTGGTCACCGTCACGATTGGGAAGGTGTAATTGTCTGGCTCTCCAGCGCCACCGCCACAACTGCCGACAACATCTTAGCCGTTTGTCCTTCCG
CCCACGGAGGCTGGGATTGTTCCACGGATGGCTATTCCCTTTCTGGTACCAGCCCTCTTATCAAGTACGAAAGTATCTGGCCCGTCGATCACTC
AATGGGTCTTACTAGTACTGTTGGTGGAAAACAACCTATGATTGCTTGGGAGTCTTTACCAACTGCTGCTCAAACTGCTCTTGAGAACACCGAT
TTCGGTGCTGCGAATGTTCCATTCATTCCGGCTGTTTTCACAGACAATCT

>Bcin04g02090(MLST10), partial sequence [organism=Botrytis cinerea, strain N11_K_W11]
CGGAGGATGATATGGCAAAGTCTATGATTACCAAAGCATTTGTAGGCATGAGTAGTAAACTGTCGAATATGACTATGAATGATGTTTACAAGCC
CTACATCCATGTAAGAAATGTAGAATAGAGAGATCAGTAACTGGAACTAATATCGTTTGTAGGCTTTCAAGTTACTTACGCAGTTCAACCCAAT
CACTACAGCTATTGCCGAATCCCCACTGTTTCAAATGGCCGTCTCAGCAAATACCATCGAAAAGTACACACTGCTAGGCCCTTTCTTCAGAATA
TCTCCTCTGCAACAGGAAGTTACCAGGGAATACTTCAGTGCGCCAAAGACGATAGATAGGCGACACATTGCCACATCTCAAGATGCGTTACGAT
TGACCTTACAAACCCATCAAAAAGATTTACTTGATATCATCAACCACTTTGTTCGAGCAAGTCCAATCGCAAAAAGCAAAACCCTGGATTGGTT
CGCCTACATTGTGAATCAAAATCACAAGCGTCGAGCACTTCAGGTAGACCCGAAAGAAGTGTCTTCTGATGGCTTTATGCACAATGTCACTGTC
GTTCTAGATGGTCTTTGTGAGCCATTCATGGATACCACATTCTCGAAAATTTCGAAGATTGATATTGATTATCTAAGACGTGCGCCTCGTGTAG
ATATCAAGGACGAGACCAAGTTGAACGCTGACGAGAAGGCTTCTGAGAAGTATTATGAGGACACTGTTCCTGGCACTTCTAATTTCATCTCTGA
GGTCTTCTTTCTCACATTGGCTGCTCATCATTATGGTAGTGAAGCTCTTAATGCCACGCATAAGAGTCTGGAGAAAGACATCAAATATATTCAA
AAGCAATTGACTGCCGTTGAAGCA

>Bcin01g07220(MLST1), partial sequence [organism=Botrytis cinerea, strain N11_K_W14]
ATGGCATATCTTGTTTCTTTTCGCATTATGCTTTGGAACGTGCTGGACAAGCTTGGGTTAGTGCTCTGCGTGTAGGAGCACTAAAGAAGATTCT
CGCACAACCGAAGTCATGGTTTGAGGAATCCAGGAATTCACCTAGCCGGTTGAACGAAGTTTTGGATAGGAACTCTGAGGAAATGCGTAATCTC
GTTGGCCGCTTTGCTGGTATTGTATTCACAGCATTTTTTATGCTATTGATATCAATCATTTGGGCTTTCGTGAATACATGGAAACTGACATTAG
TCTCGATGGCAACTGGGCCAGTTATATACGCTGTCACCAAAACGTTCAATCGCGTGAGTGGAAAATGGGAAAACAAGTGCAACTACGCCTCTGA
AATGACCACTGGCATATTTTCAGAGACGTTCTCCAACATCAAAGTGGTTCGGGCTTTACTCCGGAAACTTACTTTGAGACAAAACACACCAAAG
CTACAGAAGAACTTTATAAAGTTGGACTAATACGAGCAAACTACTCGGATTGCTGTGGGGATTGACAGATGCGATGTCATTCTTCATCACCGCA
ACTATCTTTTATTATGCCACGGTTCTCATTACCAAGAGAGAGATCAGTATCGCGACTGCACTACAGACTGTCAATCTTCTATTATTTGGTATTT

CTAATAGTACGAATATGCTGGCCATGATACCACAAATCAACTCTTCTCGCGTTACAGCTACGCATATGCTTGCATTAGCCAATCTCGATTCATC
TTCCTCCCACGAAAATAAAGGAACCGAACGGCTTTCGACAATCTTTCCAATCAAATTCAACCGTCTTTCATTCACATACCCTACTCGTCCTGAA
AAACGAACGATATCATCCTTTTCTCTTTCCCTGATTCCTAACTCAACAACTGCACTTGTCGGACCCTCCGGCTCCGGAAAATCTACAATAGCTG
CTCTGCTCATTGGTCTCTATCCGCCAGATACTTCAACACCTCCACCGTTGACATTCAATCGCGTCTCCATAAGTAACTGTCACATTCCGTCTCT
CCGGGCTTCTCTCTCACTCGTTCCACAAATACCGATTCTATTTCCAGCTACCATCCTCCATAACATCATTTATGGTCTCCCAGAATCTTCTCCT
TGTGCTAGTCTTCCATCTGCTATTGCATCAGCAAAAGATGCTGGGATCCATGAATTTATCACATCGCTTCCACAAGGTTATGATACTAT

>Bcin05g07690(MLST2), partial sequence [organism=Botrytis cinerea, strain N11_K_W14]
ACCACTATCACCAAGTCCTTTTCTGCACTTTCCTCATCAAAAACGAACGAGAACATCATCAATAAATACTTTGCTGAAGTAATTGGGTTTTATT
TTGGCCAGGATGCCTTCTCTCTACGACAGCAAGCCTTCGATGATATGTTGTGGGTAGTTCTTGGCTGGCTGGATACTGTCAAATTCATTGATTT
ACATTCTGAATTGCACTATTCAAACGACTCTCAGCCAGAATGGTACGGACAACAATATAAACCTGCATTTGCACATCGAGCGCGACTATTTTGG
GAATTGGCTTCACAAGGATGGGATACTACTCTCTGTGGTGGTGGGATGATATGGTCACCATACCTTACTCCATACAAGAACGCAATTACCAATG
AACTCTATATCGCAGCTTCGATATCGATGTACCTATACTTCCCCGGAGATGACAATCAATCCCCATTTATGCTTTCCAACCCTTCGTATCCACC
TCACGATCCGAAATATCTACAGGCAGCTGTTGATGCTTACAAATGGCTGAATGGTTCCAACATGACGGATTTACAAGGATTATATGTCGACGGG
TACCATATCTCGAATCTTTCTGGCGGTGAAAACACCCATTGCGATTCTAGAAATGAGATGGTATATACCGACAATCAAGGTGTTTTGCTTACTG
GACAACGTGGTTTGTATGACGCAACCGCCGCACGATCATACCTTGTAGATGGCCACAAACTCATCGCGAATGTTATTAATGCCACAGGCTATGA
CCTGAAACACAATGTTGTCATCTCACCGCCACCCAAAGATGGTTCCGCATTGGCAAAGTGGTTTGGCCTGGGTAGGAATGGAATACTGGAAGAG
GATGCGATTCAAGTGCTTCGTGTTCTCAAAATGGACAAACTTTCAAAGGCATATTCTTTCATCACTTGATTGCGTTCTGTAGTGATTTGCCAGG
GGAGCCTATTGCAGGGACGAAGGAAAGCTTAGAACTCGACAGAGTGTGGCATTCTGACAAATGCTCACAGTATACAAAAT

>Bcin06g01710(MLST3), partial sequence [organism=Botrytis cinerea, strain N11_K_W14]
GTGAGTCTGACTTTTGTATTTGAGCGTTAAGATAGACACTGATATACCAAGGCAATCACCTAATTTCATGGCGTTCTTCCCTGCATCATCTACC
TACCCTGGAATGCTGGGAGAATTATACTCAGCAGCTTTCACAGCACCTGCTTTCAATTGGATCTGTTCCCCTGCTGTGACAGAATTGGAGACGG
TTGTAATGGATTGGCTGGCCAAGCTTCTCAATCTCCCAGACTGTTATTTGTCTTCGACTCATGGTGGTGGTGTCATCCAAGGATCAGCCTCGGA
AGCTATCGTTACCGTTATGGTTGCTGCCCGCGATAAATATCTTCGTGAAACTACTGAAGGTCTGTCGGGCATTGAACTCGAGGATGCGATTGCA
TATAAGAGGAGTAAGCTAGTTGCACTAGGGAGCGAAATGGCACACAGCTCCACGCAGAAAGCAGCGCAAATAGCTGGCGTTAGATTCCGATCGA
TTCCAGTACTCGCATCCAATGATTTCGCCATGACGGGTGATGATTTAGAGAAGGTATTGAAAGAATGCAAATCTCAAGGATTGGAACCCTTCTA
TCTAACTTCGACTTTGGGAACAACATCTACATGCGCAGTTGACGACTTCGCATCTATTGCAACAGTACTTTCAAAATATGCACCTCCAGATGTT
GCAGGCGAGATCTGGGTTCACGTCGATGCTGCTTATGCAGGTGCAGCTTTGGTTTGCCCTGAATACCATCATCTAACATCGTCCTTCCAGCATT
TCCATTCCTTCGATATGAACATGCACAAATGGCTTCTGACAAATTTCGACGCTTCTTGTCTATATGTCAAGAAACGCAAAGATCTGATCGATGC
ACTCTCCATAACACCAAGTTATCTTCGCAACGAGTTTTCAGAGAGTGGACTCGTAACCGACTATCGGGACTGGCAAATTCCTCTCGGAAGACGC
TTCCGAAGCCTAAAGATTTGGTTTGTCCTCAGAACCTACG

>Bcin09g03030(MLST4), partial sequence [organism=Botrytis cinerea, strain N11_K_W14]
ACCCTTCAACCCCACCGCAGCTCCTATACCGAAAGCGAGTATCCTACCAATTCTCCTTCCACCTGCGACTTTAAGACCATTGGCTTTCCGCACT
TTCACAAAAAAGCATAGTTTAACATTGACGTCGTCGGCATTACAAGTGTTGGCTACTTTTATTGGAAAGCATTGTGGGACAGGATGGAGGGAAG
AAGGACTGGCAGAGAGAGTCTTGGAGGAGGTCGCCAAGAGTTGGAAGAATAGGAGTGGCGGTGTCATTGTCGAGGGCGAGGGAACGGAATTGAA
GGAGATTCTGAAAGCTTTGGAAGGGAATATGAGTGGTGGAAGGATAGTCATAGGAAGAGAGCTAAGTCGGCAAAATAGTTTAGTACTGGGATCA
TCACAATATGGAGAGGTCAATCATACAAGACTTGGGCTACGGCCAGGGAATATACCCAGAGAGGATAGTCAGTCAAGTTTGGGAATGTCAACGT
TGGAGGTCAATGACGAGGAAGATGAGGATGGCCTGATGGATCCAAGAAGGTGGTTAAAAGTCATTGATGCATTTGAGCAACCTCGACTAGTGTA
CAATGTTGCTAAAAAGCACTTTGATAGGTATGTTTCAATGATAAAATTTTATCTGAATCGACTAACTCAGTACAGAGATACCTCCAAACCTTCA
TTGTTCCCACCTGCGTCTCATAAAACACTCCTCTTCCAAAACCGCTATAATGTTATCCATCAACGTCTCCTTCGCAATGAATCTTTTCAAACGC
CCGCTTTTCAAGGTGGCAAATCTTCCCTTCAACGCAGCACGTCCGCCATTACCACCCAACAACAATCATACAAATTAACGCCGATAGCTAATCT
TCTCGGTCGCAATCGCAGCTCTCATATGCTTCTCGGTCTCCTCAGTATTTCACCCACTGGTACCCTCGCCATCAATGACCTGACGGGCAGTATC
GCTCTCGATCTTACACACGCAGCAGCCATTC

>Bcin11g01310(MLST5), partial sequence [organism=Botrytis cinerea, strain N11_K_W14]
ACTGACATGGACCTCATGTGGAACCGGCGTAGAATGCGCAACGCTTGAAGTTCCGCTCGAATATGGCGATGCAACGTCAACGGCAAAAGCCAGT
GTTGCGCTTGCTCGTTATCCTGCCACTGTTGCCGCGAGCAAGAAGCTCGGGTCTCTCTTGATAAATCCCGGTGGACCCGGTGCCTCTGGTGTTG
GCTTTGTGCAGTCTGGAGCCGGTGCCGCCGTCTCGACACTGAGTGGTGGATTGTACGATATCATCGGATGGGATCCACGTGGAACCGGTGCTTC
GGCTCCTATTTTGGAATGTTTTGCAAATGCCAGTGCGGAGTATGATTTTAACAACGCGTTTCCATCTGCTCCGAATCTCTGGCTCGGACAATTT
GCGAATGCCAGCGCAAATTCTGCTGTTAGCTCTGCTATCACATCCTTTGACACTTCTGTCGCTGCTCTTGCAAAAGCTTGCGTGGCTCAGAAAT
CTCCCGCTCTTTACACCTCAACAGCAGCATATGTTGCTCGAGACATGGCAGCGATAGTCGATGCATTGGATGGGACCTCTGCAAAACTTAACTA
CTGGGGTTTCTCATATGGAACTATTTTCCTAGCTGAGTTTATCCAAACTTTCCCAGGCCGCGTGGGAAGAGTTCTTGCCGATGGTGTTTTCGAC
GCAAAGGCAAATGCACTCACATACGTTAGCCAACTTCCCAACGATCAACTCAGTGTTCGTGCTTCGTTGAACGATTTTGCAGCTTTCTGCACCA
CCGCCGGTAGTAAAGGTTGCTCTTTTGCCACCGCCCCTACTGGAACCACAGGTACTGTTGCTACCAGACTGGACAACATAATGAAGGATATGTT
CCTCAATCCTATTGTTGCTTCGGGCTTAAGCATCA

>Bcin15g03910(MLST6), partial sequence [organism=Botrytis cinerea, strain N11_K_W14]
GCCAAAACACAAAATCATCCAACGATGAAGATGATACTCCACTTCCCTTGATTATCTGGCATGGACTCGGCGATAATTACAAAGCGGATGGTCT
TGCGCAAGTTGGAAAACTAGCTGAAGCTATTCATCCTGGGACTTTTGTCTACAATATTCATGTAGATGAGGATGCATCTGCAGATAGGACAGCT
ACCTTCTTTGGAAATCTCACTCGTGAGTACATCCCCTATTTTTCCTTTAAATATCATACTAACTCTCTTACCAAGTTCAAATCGAAAAGGTCTG
CGAAGACCTCGCCTCCCATCCTATTCTCTCTACCGCGCCCGCCGTCGACGCAATTGGATTCTCCCAAGGCGGCCAATTCTTGCGTGGCTACATA
TCCCGCTGCAATGCTCCACCCATCCGCTCTCTCCTGACCTTCGGTTCCCAACACAACGGCATTTCTGCCTTCCAAGCCTGTGGTCCTGCCGATT
TCCTCTGTCGCGGTGCTCAAACCCTTTTGCGATCCAACACCTGGTCAACCTTTGTCCAATCTCGTCTCGTACCCGCTCAATACTTCAGAGATCC
GGAAAACCTAGACTCTTACCTTGAATATTCCAATTTCCTTGCCGACATCAATAATGAGCGCGTTCTCAAGAACCAAACATATAAATCCAACATG
GAAAAATTGGAACGATTCGTAATGTATGTCTTTGAAGACGATACAACTGTCATCCCTAAGGAAAGTGGATGGTGGGCTGAAGTCAACGGCACGG
AAGTTACACCACTGAAAGAAAGAGCCATTTATAAAGAAGATTGGCTAGGTTTAAAGACATTGGATGAGGCCGGAAAATTAGTTTTCGAAACCAT
TCCAGGGGGACATATGACGTTAGGAGAGGAGATGCTAGAGAAGGCTTTCAAAGAGTATTTTGGTCCAGCAGGGAAGAAATTTGGG

>Bcin16g03460(MLST7), partial sequence [organism=Botrytis cinerea, strain N11_K_W14]
ATGAACCTCTTGATTTGAACTCTTCATTAATTGCAAGTGATGAACCCCTATTCTGCCCAGAGGATAGTTACAAGACGTATATCATTAGTCGAGA
ACCACTCATGATATATATTGACGGATTTTTGAAAGCGAATGAAAGTAAACATTTGGTTGATGTTAGGTGTGTTATTTATTCTGATGAAATGAAC
AAGAGAGACTGATGAGATAGTGAGCCGCTTTATGAACCGTCAACTGTTTCTCACGGACAGGAAGTTACCATTGATCCTTCAGTTCGGAATTCTG
AAGTGGCGGTTTTAGAGAGGGATGAGGTGGTCAGGTGTATTGAGCATAGAGCGAGGGCATTTCAGGGGTGGAGGGGCGAGATGGGGATTGAGAA
GTTGAGGACGCAGAGGTATGGGGTTGGAGGACATTATGGGATGCATTTGTAAGTTTTGGGGGATTGACGAAGGCTTCTGTCTATTTTCTATCAG
CACGGATCTTGAAAGAAAGATAGCATGAACACGCGGGCTAATAACTAGGAAATAGCGATTGGAGCGGAGGTAAACGTGGCATAGACCGATTTAG
TACTTTCATGGTCTATGTCGACGTATCCTCTGATATCGAAGGTGGAGGAACGGAATTCCCACGTATCGTGGACCAAAAGGAGGAAGGTGGGAG
GACTTCCTGGAAACTACGGAAGCATTGGATCCAAGAACTGGAGAAAATGTAACAGTAGAAGGGGTGACATTCAAACCAATCAAGGGAAATGCCG
TATTCTGGGAAAATACTGACAACAACGGGAGGGGCTATGAT

>Bcin12g03020(MLST8), partial sequence [organism=Botrytis cinerea, strain N11_K_W14]
TCGATTGGCTGCGAAGAAAACTGCGCAGCACCGAGTCACCAAAAGCACGAATAAACCATCGGCAAGGGAAATATTCTCTGAACCACAACATGAC
ACGAGCGCGGAAGAGTATATCGGGCGAGAAGCTTCATCAAGAGCACCAAAGCGACAACGAGTCGATGATAATTATAACTCTTACGGTGGAAGAA
ATGAAAATACAGCAGCTTATGCTTCCGGGAAACTTCCATCTGGAAGTATAAATGTTGGTGGAGGTAGGAAGACCACTTTTCAAGAAGAACCACG
AACGGCATTTGTCGCTGGCAAGTTGCCCCCTGGAAGTATCAACATTGGTGGAAAGAAGGCTACTCAAATCGAGGACGAGGGTAGAGCAGCTTAT
GCTTCCGGAAAATTGCCTCCAGGAAGTATAAACTCTGGCGCAAAGAAGGCGATTTCATTCCAAGATGAAACGAGACCGGCTTATGTCTCCGGAA
AGCTTCCACATGGTAGTATCGACGGTATGCGAAACCGTGAAATGGCTGCCGTCCACCGCGAAATTGCTGAGGGTGGGAGGAAACCAGGCCAGGT
TGTCTCTTCTCTATTCACATTCAATCCTACTTCAAAGAAAACTTTTGATGAACCCGAAGAACAAGCGGAACCGGCAAAACCATCCAATGCGCCT
TTGACAGAGGAAATGGCGACTTTCACCAATCTAGGGTTATCGAGAAGGCTTGCAGCCCATCTATCGACTAAACTCGATATGAAAGCTCCGACCG
CCATTCAAAAAGCATCTGTACAGCAGTTGGTATCGGACGATAGCGATGCTTTCATACAAGCAGAGACTGGATCTGGAAAAACTTTGGCATATCT
ACTACCTATAGTCGAGCGAATATTAGCATTGAGTGAGAATGGCGTACAAAT

>Bcin02g07770(MLST9), partial sequence [organism=Botrytis cinerea, strain N11_K_W14]
CAGCTTTCCCTTTCGGTCTTGGCATCTACAGTCATTGCCATCCCTACACCATCACAACTTGAGTCTCGGGCCGTTATCGATTCCGATGCCGTTG
TAGGATTTGCCGAAACTGTTCCCAGTGGGACCGTAGGAACAGTTTATGAGGCATATAAACCATTCCTTAAAGTCGTAAATGGATGCGTACCATT
CCCTGCCGTCGATGCATCGGGTAACACAGGGTATGTCCTTATATCTTTCTCTTCCACACGATTGCTATTGAGTCTCTAACATATTTTAGTGGTG
GTTTGTCACCAACTGGCAGTAGCAATGGTGGTTGCAGCAGCAGTACCGGTCAAGTATATGTTCGAGGAGGACAAAGCGGATCAAACTACGCCAT
CATGTACTCCTGGTAAGTTCTCTCTAAACTTCTCCTTATAGATCCAACCTAACAAAATCTTAGGTACATGCCAAAGGACGAGCCCTCAACCGGT
ATTGGTCACCGTCACGATTGGGAAGGTGTAATTGTCTGGCTCTCCAGCGCCACCGCCACAACTGCCGACAACATCTTAGCCGTTTGTCCTTCCG
CCCACGGAGGCTGGGATTGTTCCACGGATGGCTATTCCCTTTCTGGTACCAGCCCTCTTATCAAGTACGAAAGTATCTGGCCCGTCGATCACTC
AATGGGTCTTACTAGTACTGTTGGTGGAAAACAACCTATGATTGCTTGGGAGTCTTTACCAACTGCTGCTCAAACTGCTCTTGAGAACACCGAT
TTCGGTGCTGCGAATGTTCCATTCATTCCGGCTGTTTTCACAGACAATCT

>Bcin04g02090(MLST10), partial sequence [organism=Botrytis cinerea, strain N11_K_W14]
CGGAGGATGATATGGCAAAGTCTATGATTACCAAAGCATTTGTAGGCATGAGTAGTAAACTGTCGAATATGACTATGAATGATGTTTACAAGCC
CTACATCCATGTAAGAAATGTAGAATAGAGAGATCAGTAACTGGAACTAATATCGTTTGTAGGCTTTCAAGTTACTTACGCAGTTCAACCCAAT
CACTACAGCTATTGCCGAATCCCCACTGTTTCAAATGGCCGTCTCAGCAAATACCATCGAAAAGTACACACTGCTAGGCCCTTTCTTCAGAATA
TCTCCTCTGCAACAGGAAGTTACCAGGGAATACTTCAGTGCGCCAAAGACGATAGATAGGCGACACATTGCCACATCTCAAGATGCGTTACGAT
TGACCTTACAAACCCATCAAAAAGATTTACTTGATATCATCAACCACTTTGTTCGAGCAAGTCCAATCGCAAAAAGCAAAACCCTGGATTGGTT
CGCCTACATTGTGAATCAAAATCACAAGCGTCGAGCACTTCAGGTAGACCCGAAAGAAGTGTCTTCTGATGGCTTTATGCACAATGTCACTGTC
GTTCTAGATGGTCTTTGTGAGCCATTCATGGATACCACATTCTCGAAAATTTCGAAGATTGATATTGATTATCTAAGACGTGCGCCTCGTGTAG
ATATCAAGGACGAGACCAAGTTGAACGCTGACGAGAAGGCTTCTGAGAAGTATTATGAGGACACTGTTCCTGGCACTTCTAATTTCATCTCTGA
GGTCTTCTTTCTCACATTGGCTGCTCATCATTATGGTAGTGAAGCTCTTAATGCCACGCATAAGAGTCTGGAGAAAGACATCAAATATATTCAA

AAGCAATTGACTGCCGTTGAAGCA

>Bcin01g07220(MLST1), partial sequence [organism=Botrytis cinerea, strain N11_S_E08]
ATGGCATATCTTGTTTCTTTTCGCATTATGCTTTGGAACGTGCTGGACAAGCTTGGGTTAGTGCTCTGCGTGTAGAAGCACTAAAGAAGATTCT
CGCACAACCGAAGTCATGGTTTGAGGAATCCAGGAATTCACCTGGCCGGTTGAACGAAGTTTTGGATAGGAACTCTGAGGAAATGCGTAATCTC
GTTGGCCGCTTTGCTGGTATTGTATTCACAGCATTTTTTATGCTATTGATATCAATCATTTGGGCTTTCGTGAATACATGGAAACTGACATTAG
TCTCGATGGCAACTGGGCCAGTTATATACGCTGTCACCAAAACGTTCAATCGCGTGAGTGGAAAATGGGAAAACAAGTGCAACTACGCCTCTGA
AATGACCACTGGCATATTTTCAGAGACTTTCTCCAACATCAAAGTGGTTCGGGCTTTTACTCTGGAAACTTACTTTGAGACAAAACACACCAAA
GCTACAGAAGAACTTTATAAAGTTGGACTAATACGAGCAAACTACTCGGGATTGCTGTGGGGATTGACAGATGCGATGTCATTCTTCATCACTG
CAACTATCTTTTATTATGCCACGCTTCTCATTACCAAGAGAGAGATCAGTATCGCGACTGCACTACAGACTGTCAATCTTCTATTATTTGGTAT
TTCTAATAGTACGAATATGCTGGCCATGATACCACAAATCAACTCTTCTCGCGTTACAGCTACGCATATGCTTGTATTAGCCAATCTCGATTCA
TCTTCCTCCCACGAAAATAAAGGAACCGAACGGCTTTCGACAATCTTTCCAATCAAATTCAACCGTCTCTCATTCACATATCCTACTCGTCCTG
AAAAACGAACGATATCATCCTTTTCTCTTTCCCTGATTCCTAACTCAACAACTGCACTTGTCGGACCCTCCGGCTCCGGAAAATCTACAATAGC
TGCTCTGCTCATTGGTCTTTATCCGCCAGATATTTCAACACCTCCACCGTTGACATTCAATCGCGTCTCCATAAGTAACTGTCACATTCCGTCT
CTCCGGGCTTCTCTCTCACTCGTCCCACAAATACCGATTCTATTTCCAGCTACCATTCTCCATAACATCATTTATGGTCTCCCAGAATCTTCTC
CTTGTGCTAGTCTTCCATCTGCTATTGCATCAGCAAAAGATGCTGGGATCCATGAATTTATCACATCGCTTCCACAAGGTTATGATACTATG

>Bcin05g07690(MLST2), partial sequence [organism=Botrytis cinerea, strain N11_S_E08]
ACCACTATCACCAAGTCCTTTTCTGCACTTTCCTCATCAAAAACGAACGAGAACATCATCAATAAATACTTTGCTGAAGTAATTGGGTTTTATT
TTGGCCAGGATGCCTTCTCTCTACGACAGCAAGCCTTCGATGATATGTTGTGGGTAGTTCTTGGCTGGCTGGATACTGTCAAATTCATTGATTT
ACATTCTGAATTGCACTATTCAAACGACTCTCAGCCAGAATGGTACGGACAACAATATAAACCTGCATTTGCACATCGAGCGCGACTATTTTGG
GAATTGGCTTCACAAGGATGGGATACTACTCTCTGTGGTGGTGGGATGATATGGTCACCATACCTTACTCCATACAAGAACGCAATTACCAATG
AACTCTATATCGCAGCTTCGATATCGATGTACCTATATTTCCCCGGAGATGACAATCAATCCCCATTTATGCTTTCCAACCCTTCATATCCACC
TCACGATCCGAAATATCTACAGGCAGCTGTTGATGCTTACAAATGGCTGAATGGTTCCAACATGACGGATTTACAAGGATTATATGTCGACGGG
TACCATATCTCGAATCTTTCTGGCGGTGAAAACACCCATTGCGATTCTAGAAATGAGATGGTATATACCTACAATCAAGGTGTTTTGCTTACTG
GACAACGTGGTTTGTATGACGCAACCGCCGCACGATCATACCTTGTAGATGGCCACAAACTCATCGCGAATGTTATTAATGCCACAGGCTATGA
CCTGAAACACAATGTTGTCATCTCACCGCCACCCAAAGATGGTTCCGCATTGGCAAAGTGGTTTGGCCTGGGTAGGAATGGAATACTGGAAGAA
GGATGCGATTCAAGTGCTTCGTGTTCTCAAAATGGACAAACTTTCAAAGGCATATTCTTTCATCACTTGATTGCGTTCTGTAGTGATTTGCCAG
GGGAGCCTATTGCAGGGACGAAGGAAAGCTTAGAACTCGACAGAGTGTGGCATTCTGACAAATGCTCACAGTATACAAAATG

>Bcin06g01710(MLST3), partial sequence [organism=Botrytis cinerea, strain N11_S_E08]
GTGAGTCTGACTTTTGTATTTGAGCGTTAAGATAGACACTGATATACCAAGGCAATCACCTAATTTCATGGCGTTCTTCCCTGCATCATCTACC
TACCCTGGAATGCTGGGAGAATTATACTCAGCAGCTTTCACAGCACCTGCTTTCAATTGGATCTGTTCCCCTGCTGTGACAGAATTGGAGACGG
TTGTAATGGATTGGCTGGCCAAGCTTCTCAATCTCCCAGACTGTTATTTGTCTTCGACTCATGGTGGTGGTGTCATCCAAGGATCAGCCTCGGA
AGCTATCGTTACCGTTATGGTTGCTGCCCGCGATAAATATCTTCGTGAAACTACTGAAGGTCTGTCGGGCATTGAACTCGAGGATGCGATTGCA
TATAAGAGGAGTAAGCTAGTTGCACTAGGAAGCGAAATGGCACACAGCTCCACGCAGAAAGCAGCGCAAATAGCTGGCGTTAGATTCCGATCGA
TTCCAGTACTCGCATCCAATGATTTCGCCATGACGGGTGATGATTTAGAGAAGGTATTGAAAGAATGCAAATCTCAAGGATTGGAACCCTTCTA
TCTAACTTCGACTTTGGGAACAACATCTACATGCGCAGTTGACGACTTCGCATCTATTGCAACAGTACTTTCAAAATATGCACCTCCAGATGTT
GCAGGCGAGATCTGGGTTCACGTCGATGCTGCTTATGCAGGTGCAGCTTTGGTTTGCCCTGAATACCATCATCTAACATCGTCTTTCCAGCATT
TCCATTCCTTCGATATGAACATGCACAAATGGCTTCTGACAAATTTCGACGCTTCTTGTCTATATGTCAAGAAACGCAAAGATCTGATCGATGC
ACTGTCCATAACACCAAGTTATCTTCGCAACGAGTTTTCAGAGAGTGGACTCGTAACCGACTATCGGGACTGGCAAATTCCTCTCGGAAGACGC
TTCCGAAGCTTAAAGATTTGGTTTGTCCTCAGAACCTACG

>Bcin09g03030(MLST4), partial sequence [organism=Botrytis cinerea, strain N11_S_E08]
CGACCCTTCAACCCCACCGCAGCTCCTATACCGAAAGCGAGTATCCTACCAATTCTCCTTCCACCTGCGACTTTAAGACCATTGGCTTTCCGCA
CTTTCACAAAAAAGCATAGTTTAACATTGACGTCGTCGGCATTACAAGTGTTGGCTACTTTTATTGGAAAGCATTGTGGGACAGGATGGAGGGA
AGAAGGACTGGCAGAGAGAGTCTTGGAGGAGGTCGCCAAGAGTTGGAAGAATAGGAGTGGCGGTGTCATTGTCGAGGGCGAGGGAACGGAATTG
AAGGAGATTCTGAAAGCTTTGGAAGGGAATATGAGTGGTGGAAGGATAGTCATAGGAAGAGAGCTAAGCCGACAGAATAGTTTAGTACTGGGAT
CATCACAATATGGAGAGGTCAATCATACAAGACTTGGGCTACGGCCAGGGAATATACCCAGAGAGGATAGTCAGTCAAGTTTGGGAATGTCAAC
GTTGGAGGTCAATGACGAGGAAGATGAGGATGGCCTGATGGATCCAAGAAGGTGGTTAAAAGTCATTGATGCATTTGAGCAACCTCGACTGGTG
TACAATGTTGCTAAAAAGCACTTTGATAGGTATGTTTCAATGATAAAATTTTATCTGAATCGACTAACTCAGTACAGAGATACCTCCAAACCTT
CATTGTTCCCACCTGCGTCTCATAAAACACTCCTCTTCCAAAACCGCTATAATGTTATCCATCAACGTCTCCTTCGCAATGAATCTTTTCAAAC
GCCCGCTTTTCAAGGTGGCAAATCTTCCCTTCAACGCAGCACGTCCGCCATTACCACACAACAACAATCATACAAATTAACGCCGATAGCTAAT
CTTCTCGGTCGCAATCGCAGCTCTCATATGCTTCTCGGTCTCCTCAGTATTTCACCCACTGGTACCCTCGCCATCAATGACCTGACGGGCAGTA
TCGCTCTCGATCTTACACACGCAGCAGCCATTCC

>Bcin11g01310(MLST5), partial sequence [organism=Botrytis cinerea, strain N11_S_E08]

ACTGACATGGACCTCATGTGGAACCGGCGTAGAATGCGCAACGCTTGAAGTTCCGCTCGAATATGGCGATGCAACGTCAACGGCAAAAGCCAGT
GTTGCGCTTGCTCGTTATCCTGCCACTGTTGCCGCGAGCAAGAAGCTCGGGTCTCTCTTGATAAACCCCGGTGGACCCGGTGCCTCTGGTGTTG
GCTTTGTGCAGTCTGGAGCCGGTGCCGCCGTCTCGACACTGAGTGGTGGATTGTACGATATCATCGGATGGGATCCACGTGGAACCGGTGCTTC
GGCTCCTATTTTGGAATGTTTTGCAAATGCCAGTGCGGAGTATGATTTTAACAACGCGTTTCCATCTGCTCCGAATCTCTGGCTCGGACAATTT
GCGAATGCCAGCGCAAATTCTGCTGTTAGCTCTGCTATCACATCCTTTGACACTTCTGTCGCTGCTCTTGCAAAAGCTTGCGTGGCTCAGAAAT
CTCCCGCTCTTTACACCTCAACAGCAGCATATGTTGCTCGAGACATGGCAGCGATAGTCGATGCATTGGATGGGACCTCTGCAAAACTTAACTA
CTGGGGTTTCTCATATGGAACTATCTTCCTAGCTGAGTTTATCCAAACTTTCCCAGGCCGCGTGGGAAGAGTTCTTGCCGATGGTGTTTTCGAC
GCAAAGGCAAATGCACTCACATACGTTAGCCAACTTCCCAACGATCAACTCAGTGTTCGTGCTTCGTTGAACGATTTTGCAGCTTTCTGCACCA
CCGCCGGTAGTAAAGGTTGCTCTTTTGCCACCGCCCCTACTGGAACCACAGGTACTGTTGCTACCAGACTGGACAACATAATGAAGGATATGTT
CCTCAATCCTATTGTTGCTTCGGGCTTAAGCATCA

>Bcin15g03910(MLST6), partial sequence [organism=Botrytis cinerea, strain N11_S_E08]
GCCAAAACACAAAATCATCCAACGATGAAGATGATACTCCACTTCCCTTGATTATTTGGCATGGACTCGGCGATAATTACAAAGCGGATGGTCT
TGCGCAAGTTGGAAAACTAGCTGAAGCTATTCATCCTGGGACTTTTGTCTACAATATTCATGTAGATGAGGATGCATCTGCAGATAGGACAGCT
ACCTTCTTTGGAAATCTCACTCGTGAGTACATCCCCTATTTTCCCTTTAAATACCATACTAACTCTCTTACCAAGTTCAAATCGAAAAGGTCTG
CGAAGACCTCGCCTCCCATCCTATTCTCTCTACCGCGCCCGCCGTCGACGCAATTGGATTCTCCCAAGGCGGCCAATTCTTGCGCGGTTACATA
TCCCGCTGCAATGCTCCACCCATCCGCTCTCTCCTGACCTTCGGTTCCCAACACAACGGCATTTCTGCCTTCCAAGCCTGTGGTCCTGCCGATT
TCCTCTGTCGCGGTGCTCAAACCCTTTTGCGATCCAACACCTGGTCAACCTTTGTCCAATCTCGTCTCGTACCCGCTCAATACTTCAGAGATCC
GGAAAACCTAGACTCTTACCTTGAATATTCCAATTTCCTTGCCGACATCAATAATGAGCGCGTTCTCAAGAACCAAACATATAAATCCAACATG
GAAAAATTGGAACGATTCGTAATGTATGTCTTTGAAGACGATACAACTGTCATCCCTAAGGAAAGTGGATGGTGGGCTGAAGTCAACGGCACGG
AAGTTACACCACTGAAAGAAAGAGCCATTTATAAAGAAGATTGGCTAGGTTTAAAGACATTGGATGAGGCCGGAAAATTAGTTTTCGAAACCAT
TCCAGGGGGACATATGACGTTAGGAGAGGAGATGCTAGAGAAGGCTTTCAAAGAGTATTTTGGTCCAGCAGGGAAGAAATTTGGGG

>Bcin16g03460(MLST7), partial sequence [organism=Botrytis cinerea, strain N11_S_E08]
ATGAACCTCTTGATTTGAACTCTTCATTAATTGCAAGTGATGAACCCCTATTCTGCCCAGAGGATAGTTACAAGACGTATATCATTAGTCGAGA
ACCACTCATGATATATATTGACGGGTTTTTGAAAGCGAATGAAAGTAAACATTTGGTTGATGTTAGGTGTGTTATTTATTCTGATGAAATGAAC
AAGAGAGACTGATGAGATAGTGAGCCGCTTTATGAACCGTCAACTGTTTCTCACGGACAGGAAGTTACCATTGATCCTTCAGTTCGGAATTCTG
AAGTGGCGGTTTTAGAGAGGGATGAGGTGGTCAGGTGTATTGAGCATAGAGCGAGGGCATTTCAGGGGTGGAGGGGCGAGATGGGGATTGAGAA
GTTGAGGACGCAGAGGTATGGGGTTGGAGGACATTATGGGATGCATTTGTAAGTTTTGGGGGATTGACGAAGGCTTCTGTCTATTTTCTATCAG
CACGGATCTTGAAAGAAAGATAGCATGAACACGCGGGCTAATAACTAGGAAATAGCGATTGGAGCGGAGGTAAACGTGGCATAGACCGATTTAG
TACTTTCATGGTCTATGTCGACGTATCCTCTGATATCGAAGGTGGAGGAACGGAATTCCCACGTATCGTGGGACCAAAAGGAGGAAGGTGGGAG
GACTTCCTGGAAACTACGGAAGCATTGGATCCAAGAACTGGAGAAAATGTAACAGTAGAAGGGGTGACATTCAAACCAATCAAGGGAAATGCCG
TATTCTGGGAAAATACTGACAACAACGGGAGGGGCTATGAT

>Bcin12g03020(MLST8), partial sequence [organism=Botrytis cinerea, strain N11_S_E08]
CGATTGGCTGCGAAGAAAACTGCGCAGCACCGAGTCACCAAAAGCACGAATAAACCATCGGCAAGGGAAATATTCTCTGAACCACAACATGACA
CGAGCGCGGAAGAGTATATCGGGCGAGAAGCTTCATCAAGAGCACCAAAGCGACAACGAGTCGATGATAATTATAACTCTTACGGTGGAAGAAA
TGAAAATACAGCAGCTTATGCTTCCGGGAAACTTCCATCTGGAAGTATAAATGTTGGTGGAGGTAGGAAGACCACTTTTCAAGAAGAACCTCGA
ACGGCATTTGTCGCTGGCAAGTTGCCCCCTGGAAGTATCAACATTGGTGGAAAGAAGGCTACCCAAATCGAGGACGAGGGTAGAGCAGCTTATG
CTTCCGGAAAATTGCCCCCAGGAAGTATAAACTCTGGCGCAAAGAAGGCGATTTCATTCCAAGATGAAACGAGACCGGCTTATGTCTCCGGAAA
GCTTCCACATGGTAGTATCGACGGTATGCGAAACCGTGAAATGGCTGCCGTCCACCGCGAAATTGCTGAGGGTGGAGGAAACCAGGCCAGGTT
GTCTCTTCTCTATTCACATTCAATCCTACTTCAAAGAAAACTTTCGATGAACCAGAAGAACAAGCGGAACCGGCAAAACCATCCAATGCGCCTT
TGACAGAGGAAATGGCGACTTTCACCAATCTAGGGCTATCGAGAAGGCTTGCAGCCCATCTATCGACTAAACTCGATATGAAAGCCCCGACGGC
CATTCAAAAAGCATCTGTGCAGCAGTTGGTATCGGACGATAGCGATGCTTTCATACAAGCAGAGACTGGATCTGGAAAAACTTTGGCATATCTA
CTACCTATAGTCGAGCGAATATTAGCATTGAGTGAGAATGGCGTACAAATA

>Bcin02g07770(MLST9), partial sequence [organism=Botrytis cinerea, strain N11_S_E08]
CAGCTTTCCCTTTCGGTCTTGGCATCTACAGTCATTGCCATCCCTACACCATCACAACTTGAGTCTCGGGCCGTTATCGATTCCGATGCCGTTG
TAGGATTTGCCGAAACTGTTCCCAGTGGGACCGTAGGAACAGTTTATGAGGCATATAAACCATTCCTTAAAGTCGTAAATGGATGCGTACCATT
CCCTGCCGTCGATGCATCGGGTAACACAGGGTATGTCCTTATATCTTTCTCTTCCACACGATTGCTATTGAGTCTCTAACATATTTTAGTGGTG
GTTTGTCACCAACTGGCAGTAGCAATGGTGGTTGCAGCAGCAGTACCGGTCAAGTATATGTTCGAGGAGGACAAAGCGGATCAAACTACGCCAT
CATGTACTCCTGGTAAGTTCTCTCTAAACTTCTCCTTATAGATCCAACCTAACAAAATCTTAGGTACATGCCAAAGGACGAGCCCTCAACCGGT
ATTGGTCACCGTCACGATTGGGAAGGTGTAATTGTCTGGCTCTCCAGCGCCACCGCCACAACTGCCGACAACATCTTAGCCGTTTGTCCTTCCG
CCCACGGAGGCTGGGATTGTTCCACGGATGGCTATTCCCTTTCTGGTACCAGCCCTCTTATCAAGTACGAAAGTATCTGGCCCGTCGATCACTC
AATGGGTCTTACTAGTACTGTTGGTGGAAAACAACCTATGATTGCTTGGGAGTCTTTACCAACTGCTGCTCAAACTGCTCTTGAGAACACCGAT
TTCGGTGCTGCGAATGTTCCATTCATTCCGGCTGTTTTCACAGACAATCT

>Bcin04g02090(MLST10), partial sequence [organism=Botrytis cinerea, strain N11_S_E08]
CGGAGGATGATATGGCAAAGTCTATGATTACCAAAGCATTTGTAGGCATGAGTAGTAAACTGTCGAATATGACTATGAATGATGTTTACAAGCC
CTACATCCATGTAAGAAATGTAGAATAGAGAGATCAGTAACTGGAACTAATATCGTTTGTAGGCTTTCAAGTTACTTACGCAGTTCAACCCAAT
CACTACAGCTATTGCCGAATCCCCACTGTTTCAAATGGCCGTCTCAGCAAATACCATCGAAAAGTACACACTGCTAGGCCCTTTCTTCAGAATA
TCTCCTCTGCAACAGGAAGTTACCAGGGAATACTTCAGTGCGCCAAAGACGATAGATAGGCGACACATTGCCACATCTCAAGATGCGTTACGAT
TGACCTTACAAACCCATCAAAAAGATTTACTTGATATCATCAACCACTTTGTTCGAGCAAGTCCAATCGCAAAAAGCAAAACCCTGGATTGGTT
CGCCTACATTGTGAATCAAAATCACAAGCGTCGAGCACTTCAGGTAGACCCGAAAGAAGTGTCTTCTGATGGCTTTATGCACAATGTCACTGTC
GTTCTAGATGGTCTTTGTGAGCCATTCATGGATACCACATTCTCGAAAATTTCGAAGATTGATATTGATTATCTAAGACGTGCGCCTCGTGTAG
ATATCAAGGACGAGACCAAGTTGAACGCTGACGAGAAGGCTTCTGAGAAGTATTATGAGGACACTGTTCCTGGCACTTCTAATTTCATCTCTGA
GGTCTTCTTTCTCACATTGGCTGCTCATCATTATGGTAGTGAAGCTCTTAATGCCACGCATAAGAGTCTGGAGAAAGACATCAAATATATTCAA
AAGCAATTGACTGCCGTTGAAGCA

Bcin01g07220(MLST1), partial sequence [organism=Botrytis cinerea, strain N11_S_E09]
ATGGCATATCTTGTTTCTTTTCGCATTATGCTTTGGAACGTGCTGGGCAAGCTTGGGTTAGTGCTCTGCGTGTAGAAGCACTAAAGAAGATTCT
CGCACAACCGAAGTCATGGTTTGAGGAATCCAGGAATTCACCTAGCCGGTTGAACGAAGTTTTGGATAGGAACTCTGAGGAAATGCGTAATCTC
GTTGGCCGCTTTGCTGGTATTGTATTCACAGCATTTTTTATGCTATTGATATCAATCATTTGGGCTTTCGTGAATACATGGAAACTGACATTAG
TCTCAATGGCAACTGGGCCAGTTATATACGCTGTCACCAAAACGTTCAATCGCGTGAGTGGAAAATGGGAAAACAAGTGCAACTACGCATCTGA
AATGACCACTGGCATATTTTCAGAGACTTTCTCCAACATCAAAGTGGTTCGGGCTTTTACTCTGGAAACTTACTTTGAGACAAAACACACCAAA
GCTACAGAAGAACTCTATAAAGTTGGACTAATACGAGCAAACTACTCGGGATTGCTGTGGGGATTGACAGATGCGATGTCATTCTTCATCACTG
CAACTATCTTTTATTATGCCACGGTTCTCATTACCAAGAGAGAGATCAGTATTGCGACTGCACTACAGACTGTCAATCTTCTATTATTTGGTAT
TTCTAATAGTACGAATATGCTGGCTATGATACCACAAATCAACTCTTCTCGCATTACAGCTACGCATATGCTTGCATTAGCCAATCTCGATTCA
TCTTCCTCCCACGAAAATAAAGGAACTGAACGGCTTTCGACAATCTTTCCAATCAAATTCAACCGTCTTTCATTCACATACCCTACTCGTCCTG
AAAAACGAACGATATCATCCTTTTCTCTTTCCCTGATTCCTAACTCAACAACTGCACTTGTCGGACCCTCCGGCTCCGGAAAATCTACAATAGC
TGCTCTGCTCATTGGTCTCTATCCTCCAGATACTTCAACACCTCCACCGTTGACATTCAATCGCGTCTCCATAAGTAACTGTCACATTCCGTCT
CTCCGGGCTTCTCTCTCACTCGTCCCACAAATACCGATTCTATTTCCAGCTACCATTCTCCATAACATCATTTATGGTCTCCCAGAATCTTCTC
CTTGTGCTAGTCTTCCATCTGCTATTGCATCAGCAAAAGATGCTGGGATCCATGAATTTATCACATCGCTTCCACAAGGTTATGGTACTAT

>Bcin05g07690(MLST2), partial sequence [organism=Botrytis cinerea, strain N11_S_E09]
ACCACTATCACCAAGTCCTTTTCTGCACTTTCCTCATCAAAAACGAACGAGAACATCATCAATAAATACTTTGCTGAAGTAATTGGGTTTTATT
TTGGCCAGGATGCCTTCTCTCTACGACAGCAAGCCTTCGATGATATGTTGTGGGTAGTTCTTGGCTGGCTGGATACTGTCAAATTCATTGATTT
ACATTCTGAATTACACTATTCAAACGACTCTCAGCCAGAATGGTACGGACAACAATATAAACCTGCATTTGCACATCGAGCGCGACTATTTTGG
GAATTGGCTTCACAAGGATGGGATACTACTCTCTGTGGTGGTGGGATGATATGGTCACCATACCTTACTCCATACAAGAACGCAATTACCAATG
AACTCTATATCGCAGCTTCGATATCAATGTACCTCTATTTCCCCGGAGATGACAATCAATCCCCATTTATGCTTTCCAACCCTTCATATCCACC
TCACGATCCGAAATATCTACAGGCAGCTGTTGATGCTTACAAATGGCTGAATGGTTCCAACATGACGGATTTACAAGGATTATATGTCGACGGG
TACCATATCTCGAATCTTTCTGGCGGTGAAAACACCCATTGCGATTCTAGAAATGAGATGGTATATACCTACAATCAAGGTGTTTTGCTTACTG
GACAACGTGGTTTGTATGACGCAACCGCCGCACGATCATACCTTGTAGATGGCCACAAACTCATCGCGAATGTTATTAATGCCACAGGCTATGA
CCTGAAACACAATGTTGTCATCTCACCGCCACCCAAAGATGGTTCCGCATTGGCAAAGTGGTTTGGCCTGGGTAGGAATGGAATACTGGAAGAA
GGATGCGATTCAAGTGCTTCGTGTTCTCAAAATGGACAAACTTTCAAAGGCATATTCTTTCACCACTTGATTGCGTTCTGTAGTGATTTGCCAG
GGGAGCCTATTGCAGGGACGAAGGAAGGCCTAGAACTCGACAGAGTGTGGCATTCTGACAAATGCTCACAGTATACAAAAT

>Bcin06g01710(MLST3), partial sequence [organism=Botrytis cinerea, strain N11_S_E09]
TGGTGAGTCTGACTTTTGTATTTGAGCGTTAAGATAGACTCTGATATACCAAGGCAATCACCTAATTTCATGGCGTTCTTCCCTGCATCATCTA
CCTACCCTGGAATGCTGGGAGAATTATACTCAGCAGCTTTCACAGCTCCTGCTTTCAATTGGATCTGTTCCCCTGCTGTGACAGAATTGGAGAC
GGTTGTAATGGATTGGTTGGCCAAGCTTCTCAATCTCCCAGACTGTTATTTGTCGTCGACTCATGGTGGTGGTGTTATCCAAGGATCAGCCTCG
GAAGCTATCGTTACCGTTATGGTTGCTGCCCGCGATAAGTATCTTCGTGAAACCACTGAAGGTCTGTCGGGAATTGAACTCGAGGATGCGATTG
CATATAAGAGGAGTAAGCTAGTTGCACTAGGAAGCGAAATGGCACACAGCTCCACGCAGAAAGCAGCGCAGATAGCTGGCGTTAGATTCCGATC
GATTCCAGTACTCGCATCCAATGATTTCGCCATGACGGGTGATGATTTAGAGAAGGTATTGAAAGAATGCAAATCTCAAGGATTGGAACCCTTC
TATCTAACTTCGACGTTGGGAACAACATCTACATGCGCAGTTGACGACTTCGCATCTATTGCAACAGTACTTTCAAAATATGCACCTCCAGATG
TTGCAGGCGAGATCTGGGTTCACGTCGATGCTGCTTATGCAGGTGCAGCTTTGGTTTGCCCTGAATACCATCATCTAACATCGTCCTTCCAGCA
TTTCCATTCCTTTGATATGAACATGCACAAATGGCTTCTGACAAATTTCGACGCTTCTTGCCTATATGTCAAGAAACGCAAAGATCTGATCGAT
GCACTCTCCATAACACCAAGTTATCTTCGCAACGAGTTTTCAGAGAGTGGACTCGTAACCGACTATCGGGACTGGCAAATTCCTCTCGGAAGAC
GCTTCCGAAGCTTAAAGATTTGGTTTGTCCTCAGAACCTAC

>Bcin09g03030(MLST4), partial sequence [organism=Botrytis cinerea, strain N11_S_E09]
ACCCTTCAACCCCACCGCAGCTCCTATACCGAAAGCGAGTATCCTACCAATTCTCCTTCCACCTGCGACTTTAAGACCATTGGCTTTTCGCACT
TTCACAAAAAAGCATAGTTTAACATTGACGTCGTCGGCATTACAAGTGTTGGCTACTTTTATTGGAAAGCATTGTGGGACAGGATGGAGGGAGG
AAGGACTGGCAGAGAGAGTCTTGGAGGAGGTCGCCAAGAGTTGGAAGAATAGGAGTGGCGGTGTCATTGTCGAGGGCGAGGGAACGGAATTGAA

GGAGATTCTGAAAGCTTTGGAAGGGAATATGAGTGGTGGAAGGATAGTCATAGGAAGAGAGCTAAGCCGGCAGAATAGTTTAGTACTGGGATCA
TCACAATATGGAGAGGTCAATCATACAAGACTTGGGCTACGGCCAGGGAATATACCCAGAGAGGATAGTCAGTCAAGTTTGGGAATGTCAACGT
TGGAGGTCAATGACGAGGAAGATGAGGATGGCCTGATGGATCCAAGAAGGTGGTTAAAAGTCGTTGATGCATTTGAGCAACCTCGACTGGTGTA
CAATGTTGCTAAAAAGCACTTTGATAGGTATGTTTCAATGATAAAATTTTATCTGAATCGACTAACTCAGTACAGAGATACCTCCAAACCTTCA
TTGTTCCCACCTGCGTCTCATAAAACACTCCTCTTCCAAAACCGCTATAATGTTATCCATCAACGTCTCCTTCGCAATGAATCTTTTCAAACGC
CCGCTTTTCAAGGTGGCAAATCTTCCCTTCAACGCAGCACGTCCGCCATTACCACCCAACAACAATCATACAAATTAACGCCGATAGCTAATCT
TCTCGGTCGCAATCACAGCTCTCATATGCTTCTCGGTCTCCTCAGTATTTCACCCACTGGTACCCTCGCCATCAATGACCTGACGGGCAGTATC
GCTCTTGATCTTACACACGCAGCAGCCATTC

>Bcin11g01310(MLST5), partial sequence [organism=Botrytis cinerea, strain N11_S_E09]
ACTGACATGGACCTCATGTGGAACCGGCGTAGAATGCGCAACGCTTGAAGTTCCGCTCGAATATGGCGATGCAACGTCAACGGCAAAAGCCAGT
GTTGCGCTTGCTCGTTATCCTGCCACTGTTGCCGCGAGCAAGAAGCTCGGGTCTCTCTTGATAAATCCCGGTGGACCCGGTGCCTCTGGTGTTG
GCTTTGTGCAGTCTGGAGCCGGTGCCGCCGTCTCGACACTGAGTGGTGGATTATACGATATCATCGGATGGGATCCACGTGGAACCGGTGCTTC
GGCTCCTATTTTGGAATGTTTTGCAAATGCCAGTGCGGAGTATGATTTTAACAACGCGTTTCCATCTGCTCCGAATCTCTGGCTCGGACAATTT
GCGAATGCCAGCGCAAATTCTGCTGTTAGCTCTGCTATCACATCCTTTGACACTTCTGTCGCTGCTCTTGCAAAAGCTTGCGTGGCTCAGAAAT
CTCCCGCTCTTTACACCTCAACAGCAGCATATGTTGCTCGAGACATGGCAGCGATAGTCGATGCATTGGATGGGACCTCTGCAAAACTTAACTA
CTGGGGTTTCTCATATGGAACTATCTTCCTAGCTGAGTTTATCCAAACTTTCCCAGGCCGCGTGGGAAGAGTTCTTGCCGATGGTGTTTTCGAC
GCAAAGGCAAATGCACTCACATACGTTAGCCAACTTCCCAACGGTCAACTCAGTGTTCGTGCTTCGTTGAACGATTTTGCAGCTTTCTGCACCA
CCGCCGGTAGTAAAGGTTGCTCTTTTGCCACCGCCCCTACTGGAACCTCAGGTACTGTTGCTACCAGACTGGACAACATAATGAAGGATATGTT
CCTCAATCCTATTGTTGCTTCGGGCTTGAGCATCA

>Bcin15g03910(MLST6), partial sequence [organism=Botrytis cinerea, strain N11_S_E09]
GCCAAAACACAAAATCATCCAACGATGAAGATGATACTCCACTTCCCTTGATTATCTGGCATGGACTCGGCGATAATTACAAAGCGGATGGTCT
TGCGCAAGTTGGAAAACTAGCTGAAGCTATTCATCCTGGGACTTTTGTCTATAATATTCATGTAGATGAGGATGCATCTGCAGATAGGACAGCT
ACCTTCTTTGGAAATCTCACTCGTGAGTACATCCCCTATTTTTCCTTTAAATACCATACTAACTCTCTTACCAAGTTCAAATCGAAAAGGTCTG
CGAAGACCTCGCCTCCCATCCTATTCTCTCTACCGCGCCCGCCGTCGACGCAATTGGATTCTCCCAAGGCGGCCAATTCTTGCGCGGTTACATA
TCCCGCTGCAATGCTCCACCCATCCGCTCTCTCCTGACCTTCGGTTCCCAACACAACGGCATTTCTGCCTTCCAAGCCTGTGGTCCTGCCGATT
TCCTCTGTCGCGGTGCTCAAACCCTTTTGCGATCCAACACCTGGTCAACCTTTGTCCAATCTCGTCTCGTACCTGCTCAATACTTCAGAGATCC
GGAAAACCTAGACTCTTACCTTGAATATTCCAATTTCCTTGCCGACATCAATAATGAGCGCGTTCTCAAGAACCAAACATATAAATCCAACATG
GAAAAATTGGAACGATTCGTAATGTATGCTTTGAAGACGATACAACTGTCATCCCTAAGGAAAGTGGATGGTGGGCTGAAGTCAATGGCACGGA
AGTTACACCACTGAAAGAAAGAGCCATTTATAAAGAAGATTGGCTAGGGTTAAAGACATTGGATGAGGCCGGAAAATTAGTTTTCGAAACCATT
CCAGGGGGACATATGACGTTAGGAGAGGAGATGCTAGAGAAGGCTTTCAAAGAGTACTTTGGTCCAGCAGGGAAGAAATTTGGG

>Bcin16g03460(MLST7), partial sequence [organism=Botrytis cinerea, strain N11_S_E09]
CATGAACCTCTTAATTTGAACTCTTCATTAATTGCAAGTGATGAGCCCCTATTCTGCCCAGAGGATAGCTACAAGACGTATATCATTAGTCGAG
AACCACTCATGATATACATTGACGGATTTTTGAAAGCGAATGAAAGTAAACACTTGGTTGATGTTAGGTGTGTTATTTATTCTGATGAAATGAA
CAAGAGAGACTGATGAGATAGTGAACCGCTTTATGAACCGTCTACTGTTTCTCACGGACAGGAAGTTACCATTGATCCTTCGGTTCGAAATTCT
GAAGTGGCGGTTTTAGAGAGGGATGAGGTGGTCAGGTGTATTGAGCATAGAGCGAGGGCATTTCAGGGGTGGAGGGACGAGATGGGGATTGAGA
AGCTGAGGACGCAGAGGTATGGGGTTGGAGGACATTATGGGATGCATTTGTAAGTTTTGGGGGATTGACGAAGGCTTCTGTCTATTTTCTATCA
GTGCGGATCTTGAAAGAAAGATAGCATGAATACGAGGGCTAATAACTAGGAAATAGCGATTGGAGCGGAGGTAAACGTGGCATAGACCGATTTA
GTACTTTCATGGTCTATGTCGACGTATCCTCTGATATCGAAGGTGGAGGAACGGAATTCCCACGTATTGTGGGACCAAAAGGAGGAAGGTGGGA
GGACTTCCTGGAAACTACGGAAGCATTGGATCCAAGAACTGGAGAAAATGTAACAGTAGAAGGGGTGACATTCAAACCAATCAAGGGAAATGCC
GTATTCTGGGAAAATACTGACAACAACGGGAGGGGCTATGATG

>Bcin12g03020(MLST8), partial sequence [organism=Botrytis cinerea, strain N11_S_E09]
TCGATTGGCTGCGAAGAAAACTGCGCAGCACCGAGTCACCAAAAGCACGAATAAACCATCGGCAAGGGAAATATTCTCTGAACCACAACATGAC
ACGAGCGCGGAAGAGTATATCGGGCGAGAAGCTTCATCAAGAGCACCAAAGCGACAACGAGTCGATGATAATTATAACTCTTACGGTGGAAGAA
ATGAGAATACAGCAGCTTATGCTTCCGGGAAACTTCCATCTGGAAGTATAAATGTTGGTGGAGGTAGGAAGACCACTTTTCAAGAAGAACCTCG
AACGGCATTTGTCGCTGGCAAGTTGCCCCCTGGAAGTATCAACATTGGTGGAAAGAAGGCTACCCAAATCGAGGACGAGGGTAGAGCAGCTTAT
GCTTCCGGAAAATTGCCCCCAGGAAGTATAAACTCTGGCGCAAAGAAGGCGATTTCACTCCAAGATGAAACGAGACCGGCTTATGTCTCTGGAA
AGCTTCCACATGGTAGTATGGACGGTATGCGAAACCGTGAAATGGCTGCCGTCCACCGCGAAATTGCTGAGGGTGGGAGGAAACCAGGCCAGGT
TGTCTCTTCTCTATTCACATTCAATCCTACTTCAAAGAAAACTTTCGATGAACCCGAAGAACAAGCGGAACCGGCAAAACCATCCAATGCACCT
TTGACAGAGGAAATGGCGACTTTCACCAATCTAGGGTTATCGAGAAGGCTTGCAGCCCATCTATCGACTAAACTCGATATGAAAGCCCCGACCG
CCATTCAAAAAGCATCTGTGCAGCAGTTGGTATCGGACGATAGCGATGCTTTCATACAAGCAGAGACTGGATCTGGAAAAACTTTGGCATATCT
ACTACCTATAGTCGAGCGAATATTAGCATTGAGTGAGAATGGCGTACAAAT

>Bcin02g07770(MLST9), partial sequence [organism=Botrytis cinerea, strain N11_S_E09]

ACAGCTTTCCCTTTCGGTCTTGGCATCTACAGTCATTGCCATCCCTACACCATCACAACTTGAGTCTCGGGCCGTTATCGATTCCGATGCCGTT
GTAGGATTTGCCGAAACTGTTCCCAGTGGGACCGTAGGAACAGTTTATGAGGCATATAAACCATTCCTTAAAGTCGTAAATGGATGCGTACCAT
TCCCTGCCGTCGATGCATCGGGTAACACAGGGTATGTCCTTATACCATTCTCTTCCACACGATTGCTATTGAGTCTCTAACATATTTTAGTGGT
GGTTTGTCACCAACTGGCAGTAGCAATGGTGGTTGCAGCAGCAGTACCGGTCAAGTATATGTTCGAGGAGGACAAAGCGGATCAAACTACGCCA
TCATGTACTCCTGGTAAGTTCTCTCTAAACTTCTCCTTATAGATCCAACCTAACAAAATCTTAGGTACATGCCAAAGGACGAGCCCTCAACCGG
TATTGGTCACCGTCACGATTGGGAAGGTGTAATTGTCTGGCTCTCAAGCGCCACCGCCACAACTGCCGACAACATCTTAGCCGTTTGTCCTTCC
GCCCACGGAGGCTGGGATTGTTCCACCGATGGCTATTCCCTTTCTGGTACCAGCCCTCTTATCAAGTACGAAAGTATCTGGCCCGTCGATCATT
CAATGGGTCTTACTAGTACTGTTGGTGGAAAACAACCTATGATTGCTTGGGAGTCTTTACCAACTGCTGCTCAAACTGCTCTTGAGAACACCGA
TTTCGGTGCTGCGAATGTTCCATTCATTCCGGCTGTTTTCACAGATAATCT

>Bcin04g02090(MLST10), partial sequence [organism=Botrytis cinerea, strain N11_S_E09]
CGGAGGATGATATGGCAAAGTCTATGATTACCAAAGCATTTGTAGGCATGAGTAGTAAACTGTCGAATATGACTATGAATGATGTTTACAAGCC
CTACATCCATGTAAGAAATGTAGAATAGAGAGATCAGTAACTGGAACTAATATCGTTTGCAGGCTTTCAAGTTACTTACGCAGTTCAACCCAAT
CACTACAGCTATTGCCGAATCCCCACTATTTCAAATGGCTGTCTCAGCAAATACCATCGAAAAGTACACACTGCTAGGCCCTTTCTTCAGAATA
TCTCCTCTGCAACAGGAAGTTACCAGGGAATACTTCAGTGCGCCAAAGACGATAGATAGGCGACACATTGCCACATCTCAAGATGCCTTACGAT
TGACCTTACAAACCCATCAAAAAGATTTACTTGATATCATCAACCACTTTGTTCGAGCAAGTCCAATCGCCAAAAGCAAAACCCTGGATTGGTT
CGCCTACATTGTGAATCAAAATCACAAGCGTCGAGCACTTCAAGTAGACCCGAAAGAAGTGTCTTCTGATGGTTTTATGCACAATGTCACTGTC
GTTCTAGATGGTCTTTGTGAGCCATTCATGGATACCACATTCTCAAAAATTTCGAAGATTGATATTGATTATCTAAGACGTGCGCCTCGTGTAG
ATATCAAGGACGAGACCAAGTTGAACGCTGATGAGAAGGCTTCTGAGAAGTATTATGAGGACACTGTTCCTGGCACTTCTAATTTCATCTCTGA
GGTCTTCTTTCTGACATTGGCTGCTCATCATTATGGTAGTGAAGCTCTTAATGCCACGCATAAGGGTCTGGAGAAAGACATCAAATATATTCAA
AAGCAATTGACTGCCGTTGAAGCA

>Bcin01g07220(MLST1), partial sequence [organism=Botrytis cinerea, strain Pepper]
ATGGCATATCTTGTTTCTTTTCGCATTATGCCTTGGAACGTGCTGGACAAGCTTGGGTTAGTGCTCTGCGTGTAGAAGCACTAAAGAAGATTCT
CGCGCAACCGAAGTCATGGTTTGAGGAATCCAGGAATTCACCTGGCCGGTTGAACGAAGTTTTGGATAGGAACTCTGAGGAAATGCGTAATCTC
GTTGGCCGCTTTGCTGGTATTGTATTCACAGCATTTTTTATGCTATTGATATCGATCATTTGGGCTTTCGTGAATACATGGAAACTGACATTAG
TCTCAATGGCAACTGGGCCAGTTATATACGCTGTCACTAAAACGTTCAGTCGCGTGAGTGGAAAATGGGAAAACAAGTGCAACTACGCATCTGA
AATGACCACTGGCATATTTTCAGAGACTTTCTCCAACATCAAAGTGGTTCGGGCTTTTACTCTGGAAACTTACTTTGAGACAAAACACACCAAA
GCTACAGAAGAACTCTATAAAGTTGGACTAATACGAGCAAACTACTCAGGATTGCTGTGGGGATTGACAGATGCGATGTCATTCTACATCACTG
CAACTATCTTTTATTATGCCACGGTTCTCATTACCAAGAGAGAGATCAGTATCGCGACTGCACTGCAGACTGTCAATCTTCTATTATTTGGTAT
TTCTAATAGTACGAATATGCTGGCCATGATACTACAAATCAACTCTTCTCGCGTTACAGCTACGCATATGCTTGCATTAGCCAATCTCGATTCA
TCTTCCTCCCACGAAAATAAAGGAGCCGAACGGCTTTCGACAATCTTTCCAATCAAATTCAACAGTCTCTCATTCACATATCCTGCTCGTCCTG
AAAAACGAACGATATCATCCTTTTCTCTTTCCCTGATTCCTAACTCAACAACTGCACTTGTCGGACCCTCCGGCTCCGGAAAATCTACAATAGC
TGCTCTGCTCGTTGGTCTCTATCCGCCAGATACTTCAACACCTCCACCGTTGACATTCAATCGTGTCTCCATAAGTAACTGTCACATTCCGTCT
CTCCGGGCTTCTCTCTCACTCGTCCCACAAATACCGATTCTATTTCCAGCTACCATTCTCCATAACATCATTTATGGTCTCCCAGAATCCTCTC
CTTGTGCTAGTCTTCCATCTGCTATTGCATCAGCAAAAGATGCTGGGATCCATGAATTTATCACATCGCTTCCAAAAGGTTATGATACTAT

>Bcin05g07690(MLST2), partial sequence [organism=Botrytis cinerea, strain Pepper]
ACCACTATCACCAAGTCCTTTTCTGCACTTTCCTCATCAAAAACGAACGAGAACATCATCAATAAATACTTTGCTGAAGTAATTGGGTTTTATT
TTGGCCAGGATGCCTTCTCTCTACGACAGCAAGCCTTCGATGATATGTTGTGGGTAGTTCTTGGCTGGCTGGATACTGTCAAATTCATTGATTT
ACATTCTGAATTGCACTATTCAAACGACTCTCAGCCAGAATGGTACGGACAACAATATAAACCTGCATTTGCACATCGAGCGCGACTATTTTGG
GAATTGGCTTCACAAGGATGGGATACTACTCTCTGTGGTGGTGGGATGATATGGTCACCATACCTTACTCCATACAAGACGCAATTACCAATGA
ACTCTATATCGCAGCTTCGATATCGATGTACCTATATTTCCCCGGAGATGACAATCAATCCCCATTTATGCTTTCCAACCCTTCATATCCACCT
CACGATCCGAAATATCTACAGGCAGCTGTTGATGCTTACAAATGGCTGAATGGTTCCAACATGACGGATTTACAAGGATTATATGTCGACGGGT
ACCATATCTCGAATCTTTCTGGCGGTGAAAACACCCATTGCGATTCTAGAAATGAGATGGTATATACCTACAATCAAGGTGTTTTGCTTACTGG
ACAACGTGGTTTGTATGACGCAACCGCCGCACGATCATACCTTGTAGATGGCCACAAACTCATCGCGAATGTTATTAATGCCACAGGCTATGAC
CTGAAACACAATGTTGTCATCTCACCGCCACCCAAAGATGGTTCCGCATTGGCAAAGTGGTTTGGCCTGGGTAGGAATGGAATACTGGAAGAAG
GATGCGATTCAAGTGCTTCGTGTTCTCAAAATGGACAAACTTTCAAAGGCATATTCTTTCATCACTTGATTGCGTTCTGTAGTGATTTGCCAGG
GGAGCCTATTGCAGGGACGAAGGAAAGCTTAGAACTCGACAGAGTGTGGCATTCTGACAAATGCTCACAGTATACAAAAT

>Bcin06g01710(MLST3), partial sequence [organism=Botrytis cinerea, strain Pepper]
GTGAGCCTGACTTTTGTATTTGAGCGTTAAGATAGACACTGATATACCAAGGCAATCACCTAATTTCATGGCGTTCTTCCCTGCATCATCTACC
TACCCTGGAATGCTGGGAGAATTATACTCAGCAGCTTTCACAGCACCTGCTTTCAATTGGATCTGTTCCCCTGCTGTGACAGAATTGGAGACGG
TTGTAATGGATTGGCTGGCCAAGCTTCTCAATCTCCCAGACTGTTATTTGTCTTCGACTCATGGTGGTGGTGTCATCCAAGGATCAGCCTCGGA
AGCTATCGTTACCGTTATGGTTGCTGCCCGCGATAAATATCTTCGTGAAACTACTGAAGGTCTGTCGGGCATTGAACTCGAGGATGCGATTGCA
TATAAGAGGAGTAAGCTAGTTGCACTAGGGAGCGAAATGGCACACAGCTCCACGCAGAAAGCAGCGCAAATAGCTGGCGTTAGACTCCGATCGA
TTCCAGTACTCGCATCCAATGATTTCGCCATGACGGGTGATGATTTAGAGAAGGTATTGAAAGAATGCAAATCTCAAGGATTGGAACCCTTCTA

TCTAACTTCGACTTTGGGAACAACATCTACATGCGCAGTTGACGACTTCGCATCTATTGCAACAGTACTTTCAAAATATGCACCTCCAGATGTT
GCAGGCGAGATCTGGGTTCACGTCGATGCTGCTTATGCAGGTGCAGCTTTGGTTTGCCCTGAATACCATCATCTAACATCGTCCTTCCAGCATT
TCCATTCCTTCGACATGAACATGCACAAATGGCTTCTGACAAATTTCGACGCTTCTTGTCTATATGTCAAGAAACGCAAAGATCTGATCGATGC
ACTCTCCATAACACCAAGTTATCTTCGCAACGAGTTTTCAGAGAGTGGACTCGTAACCGACTATCGGGACTGGCAAATTCCTCTCGGAAGACGC
TTCCGAAGCTTAAAGATTTGGTTTGTCCTCAGAACCTAC

>Bcin09g03030(MLST4), partial sequence [organism=Botrytis cinerea, strain Pepper]
ACCCTTCAACCCCACCGCAGCTCCTATACCGAAAGCGAGTATCCTACCAATTCTCCTTCCACCGCGACTTTAAGACCATTGGCTTTCCGCACTT
TCACAAAAAAGCATAGTTTAACATTGACGTCGTCGGCATTACAAGTGTTGGCTACTTTTATTGGAAAGCATTGTGGGACAGGATGGAGGGAAGA
AGGACTGGCAGAGAGAGTCTTGGAGGAGGTCGCCAAGAGTTGGAAGAATAGGAGTGGCGGTGTCATTGTCGAGGGCGAGGGAACGGAATTGAAG
GAGATTCTGAAAGCTTTGGAAGGGAATATGAGTGGTGGAAGGATAGTCATAGGAAGAGAGCTAAGCCGACAGAATAGTTTAGTACTGGGATCAT
CACAATATGGAGAGGTCAATCATACAAGACTTGGGCTACGGCCAGGGAATATACCCAGAGAGGATAGTCAGTCAAGTTTGGGAATGTCAACGTT
GGAGGTCAATGACGAGGAAGATGAGGATGGCCTGATGGATCCAAGAAGGTGGTTAAAAGTCATTGATGCATTTGAGCAACCTCGACTGGTGTAC
AATGTTGCTAAAAAGCACTTTGATAGGTATGTTTCAATGATAAAATTTTATCTGAATCGACTAACTCAGTACAGAGATACCTCCAAACCTTCAT
TGTTCCCACCTGCGTCTCATAAAACACTCCTCTTCCAAAACCGCTATAATGTTATCCATCAACGTCTCCTTCGCAATGAATCTTTTCAAACGCC
CGCTTTTCAAGGTGGCAAATCTTCCCTTCAACGCAGCACGTCCGCCATTACCACACAACAACAATCATACAAATTAACGCCGATAGCTAATCTT
CTCGGTCGCAACCGCAGCTCTCATATGCTTCTCGGTCTCCTCAGTATTTCACCCACTGGTACCCTCGCCATCAATGACCTGACGGGCAGTATCG
CTCTCGATCTTACACACGCAGCAGCCATTC

>Bcin11g01310(MLST5), partial sequence [organism=Botrytis cinerea, strain Pepper]
ACTGACATGGACCTCATGTGGAACCGGCGTAGAATGCGCAACGCTTGAAGTTCCGCTCGAATATGGCGATGCAACGTCAACGGCAAAAGCCAGT
GTTGCGCTTGCTCGTTATCCTGCCACTGTTGCCGCGAGCAAGAAGCTCGGGTCTCTCTTGATAAACCCCGGTGGACCCGGTGCCTCTGGTGTTG
GCTTTGTGCAGTCTGGAGCCGGTGCCGCCGTCTCGACACTGAGTGGTGGATTGTACGATATCATCGGATGGGATCCACGTGGAACCGGTGCTTC
GGCTCCTATTTTGGAATGTTTTGCAAATGCCAGTGCGGAGTATGATTTTAACAACGCGTTTCCATCTGCTCCGAATCTCTGGCTCGGACAATTG
CGAATGCCAGCGCAAATTCTGCCTGTTAGCTCTGCTATCACATCCTTTGACACTTCTGTCGCTGCTCTTGCAAAAGCTTGCGTGGCTCAGAAAT
CTCCCGCTCTTTACACCTCAACAGCAGCATATGTTGCTCGAGACATGGCAGCGATAGTCGATGCATTGGATGGGACCTCTGCAAAACTTAACTA
CTGGGGTTTCTCATATGGAACTATTTTCCTAGCTGAGTTTATCCAAACTTTCCCAGGCCGTGTGGGAAGAGTTTCTTGCCGATGGTGTTTTCGA
CGCAAAGGCAAATGCACTCACATACGTTAGCCAACTTCCCAACGATCAACTCAGTGTTCGTGCTTCGTTGAACGATTTTGCAGCTTTCTGCACC
ACCGCCGGTAGTAAAGGTTGCTCTTTTGCCACCGCCCCTACTGGAACCTCAGGTACTGTTGCTACCAGACTGGACAACATAATGAAGGATATGT
TCCTCAATCCTATTGTTGCTTCGGGCTTAAGCATCA

>Bcin15g03910(MLST6), partial sequence [organism=Botrytis cinerea, strain Pepper]
GCCAAAACACAAAATCATCCAACGATGAAGATGATACTCCACTTCCCTTGATTATCTGGCATGGGCTCGGCGATAATTACAAAGCGGATGGTCT
TGCGCAAGTTGGAAAACTAGCTGAAGCTATTCATCCTGGGACTTTTGTCTACAATATTCATGTAGATGAGGATGCATCTGCAGATAGGACAGCT
ACCTTCTTTGGAAATCTCACTCGTGAGTACATCCCCTATTTTTCCTTTAAATATCATACTAACTCTCTTACCAAGTTCAAATCGAAAAGGTCTG
CGAAGACCTCGCCTCCCATCCTATTCTCTCTACCGCGCCCGCCGTCGACGCAATTGGATTCTCCCAAGGCGGCCAATTCTTGCGTGGCTACATA
TCCCGCTGCAATGCTCCACCCATCCGCTCTCTCCTGACCTTCGGTTCCCAACACAACGGCATTTCTGCCTTCCAAGCCTGTGGTCCTGCCGATT
TCCTCTGTCGCGGTGCTCAAACCCTTTTGCGATCCAACACCTGGTCAACCTTTGTCCAATCTCGTCTCGTACCCGCTCAATACTTCAGAGATCC
GGAAAACCTAGACTCTTACCTTGAATATTCCAATTTCCTTGCCGACATCAATAATGAGCGCGTTCTCAAGAACCAAACATATAAATCCAACATG
GAAAAATTGGAACGATTCGTAATGTATGTCTTTGAAGACGATACAACTGTCATCCCTAAGGAAAGTGGATGGTGGGCTGAAGTCAACGGCACGG
AAGTTACACCACTGAAAGAAAGAGCCATTTATAAAGAAGATTGGCTAGGTTTAAAGACATTGGATGAGGCCGGAAAATTAGTTTTCGAAACCAT
TCCAGGGGGACATATGACGTTAGGAGAGGAGATGCTAGAGAAGGCTTTCAAAGAGTATTTTGGTCCAGCAGGGAAGAAATTTGGG

>Bcin16g03460(MLST7), partial sequence [organism=Botrytis cinerea, strain Pepper]
ATGAACCTCTTGATTTGAACTCTTCATTAATTGCAAGTGATGAACCCCTATTCTGCCCAGAGGATAGTTACAAGACGTATATCATTAGTCGAGA
ACCACTCATGATATATATTGACGGATTTTTGAAAGCGAATGAAAGTAAACATTTGGTTGATGTTAGGTGTGTTATTTATTCTGATGAAATGAAC
AAGAGAGACTGATGAGATAGTGAGCCGCTTTATGAACCGTCAACTGTTTCTCACGGACAGGAAGTTACCATTGATCCTTCAGTTCGGAATTCTG
AAGTGGCGGTTTTAGAGAGGGATGAGGTGGTCAGGTGTATTGAGCATAGAGCGAGGGCATTTCAGGGGTGGAGGGGCGAGATGGGGATTGAGAA
GTTGAGGACGCAGAGGTATGGGGTTGGAGGACATTATGGGATGCATTTGTAAGTTTTGGGGGATTGACGAAGGCTTTTGTCTATTTTCTACCAG
TACGGATCTTGAAAGAAAGATAGCATGAACACGAGGGCTAATAACTAGGAAATAGCGATTGGAGCGGAGGTAAACGTGGCATAGACCGATTTAG
TACTTTCATGGTCTATGTCGACGTATCCTCTGATATCGAAGGTGGAGGAACGGAATTCCCACGTATCGTGGGACCAAAAGGAGGAAGGTGGGAG
GACTTCCTGGAAACTACGGAAGCATTGGATCCAAGAACTGGAGAAAATGTAACAGTAGAAGGGGTGACATTCAAACCAATCAAGGGAAATGCCG
TATTCTGGGAAAATACTGACAACAACGGGAGGGGCTATGAT

>Bcin12g03020(MLST8), partial sequence [organism=Botrytis cinerea, strain Pepper]
CGATTGGCTGCGAAGAAAACTGCGCAGCACCGAGTCACCAAAAGCACGAATAAACCATCGGCAAGGGAAATATTCTCTGAACCACAACATGACA
CGAGCGCGGAAGAGTATATCGGGCGAGAAGCTTCATCAAGAGCACCAAAGCGACAACGAGTCGATGATAATTATAACTCTTACGGTGGAAGAAA

TGAAAATACAGCAGCTTATGCTTCCGGGAAACTTCCATCTGGAAGTATAAATGTTGGTGGAGGTAGGAAGACCACTTTTCAAGAAGAACCACGA
ACGGCATTTGTCGCTGGCAAGTTGCCCCCTGGAAGTATCAACATTGGTGGAAAGAAGGCTACTCAAATCGAGGACGAGGGTAGAGCAGCTTATG
CTTCCGGAAAATTGCCTCCAGGAAGTATAAACTCTGGCGCAAAGAAGGCGATTTCATTCCAAGATGAAACGAGACCGGCTTATGTCTCCGGAAA
GCTTCCACATGGTAGTATCGACGGTATGCGAAACCGTGAAATGGCTGCCGTCCACCGCGAAATTGCTGAGGGTGGGAGGAAACCAGGCCAGGTT
GTCTCTTCTCTATTCACATTCAATCCTACTTCAAAGAAAACTTTTGATGAACCCGAAGAACAAGCGGAACCGGCAAAACCATCCAATGCGCCTT
TGACAGAGGAAATGGCGACTTTCACCAATCTAGGGTTATCGAGAAGGCTTGCAGCCCATCTATCGACTAAACTCGATATGAAAGCTCCGACCGC
CATTCAAAAAGCATCTGTACAGCAGTTGGTATCGGACGATAGCGATGCTTTCATACAAGCAGAGACTGGATCTGGAAAAACTTTGGCATATCTA
CTACCTATAGTCGAGCGAATATTAGCATTGAGTGAGAATGGCGTACAAA

>Bcin02g07770(MLST9), partial sequence [organism=Botrytis cinerea, strain Pepper]
CAGCTTTCCCTTTCGGTCTTGGCATCTACAGTCATTGCCATCCCTACACCATCACAACTTGAGTCTCGGGCCGTTATCGATTCCGATGCCGTTG
TAGGATTTGCCGAAACTGTTCCCAGTGGGACCGTAGGAACAGTTTATGAGGCATATAAACCATTCCTTAAAGTCGTAAATGGATGCGTACCATT
CCCTGCCGTCGATGCATCGGGTAACACAGGGTATGTCCTTATATCTTTCTCTTCCACACGATTGCTATTGAGTCTCTAACATATTTTAGTGGTG
GTTTGTCACCAACTGGCAGTAGCAATGGTGGTTGCAGCAGCAGTACCGGTCAAGTATATGTTCGAGGAGGACAAAGCGGATCAAACTACGCCAT
CATGTACTCCTGGTAAGTTCTCTCTAAACTTCTCCTTATAGATCCAACCTAACAAAATCTTAGGTACATGCCAAAGGACGAGCCCTCAACCGGT
ATTGGTCACCGTCACGATTGGGAAGGTGTAATTGTCTGGCTCTCCAGCGCCACCGCCACAACTGCCGACAACATCTTAGCCGTTTGTCCTTCCG
CCCACGGAGGCTGGGATTGTTCCACGGATGGCTATTCCCTTTCTGGTACCAGCCCTCTTATCAAGTACGAAAGTATCTGGCCCGTCGATCACTC
AATGGGTCTTACTAGTACTGTTGGTGGAAAACAACCTATGATTGCTTGGGAGTCTTTACCAACTGCTGCTCAAACTGCTCTTGAGAACACCGAT
TTCGGTGCTGCGAATGTTCCATTCATTCCGGCTGTTTTCACAGACAATCT

>Bcin04g02090(MLST10), partial sequence [organism=Botrytis cinerea, strain Pepper]
CGGAGGATGATATGGCAAAGTCTATGATTACCAAAGCATTTGTAGGCATGAGTAGTAAACTGTCGAATATGACTATGAATGATGTTTACAAGCC
CTACATCCATGTAAGAAATGTAGAATAGAGAGATCAGTAACTGGAACTAATATCGTTTGTAGGCTTTCAAGTTACTTACGCAGTTCAACCCAAT
CACTACAGCTATTGCCGAATCCCCACTGTTTCAAATGGCCGTCTCAGCAAATACCATCGAAAAGTACACACTGCTAGGCCCTTTCTTCAGAATA
TCTCCTCTGCAACAGGAAGTTACCAGGGAATACTTCAGTGCGCCAAAGACGATAGATAGGCGACACATTGCCACATCTCAAGATGCGTTACGAT
TGACCTTACAAACCCATCAAAAAGATTTACTTGATATCATCAACCACTTTGTTCGAGCAAGTCCAATCGCAAAAAGCAAAACCCTGGATTGGTT
CGCCTACATTGTGAATCAAAATCACAAGCGTCGAGCACTTCAGGTAGACCCGAAAGAAGTGTCTTCTGATGGCTTTATGCACAATGTCACTGTC
GTTCTAGATGGTCTTTGTGAGCCATTCATGGATACCACATTCTCGAAAATTTCGAAGATTGATATTGATTATCTAAGACGTGCGCCTCGTGTAG
ATATCAAGGACGAGACCAAGTTGAACGCCGACGAGAAGGCTTCTGAGAAGTATTATGAGACACTGTTCCTGGCACTTCTAATTTCATCTCTGAG
GTCTTCTTTCTCACATTGGCTGCTCATCATTATGGTAGTGAAGCTCTTAATGCCACGCATAAGAGTCTGGAGAAAGACATCAAATATATTCAAA
AGCAATTGACTGCCGTTGAAGCA

>Bcin01g07220(MLST1), partial sequence [organism=Botrytis cinerea, strain U11_SC_BR02]
ATGGCATATCTTGTTTCTTTTCGCATTATGCTTTGGAACGTGCTGGACAAGCTTGGGTTAGTGCTCTGCGTGTAGAAGCACTAAAGAAGATTCT
CGCACAACCGAAGTCATGGTTTGAGGAATCCAGGAATTCACCTGGCCGGTTGAACGAAGTTTTGGATAGGAACTCTGAGGAAATGCGTAATCTC
GTTGGCCGCTTTGCTGGTATTGTATTCACAGCATTTTTTATGCTATTGATATCAATCATTTGGGCTTTTGTGAATACATGGAAACTGACATTAG
TCTCGATGGCAACTGGGCCAGTTATATACGCTGTCACCAAAACATTCAATCGCGTGAGTGGAAAATGGGAAAACAAGTGCAACTACGCCTCTGA
AATGACCACTGGCATATTTTCAGAGACTTTCTCCAACATCAAAGTGGTTCGGGCTTTTACTCTGGAAACTTACTTTGAGACAAAACACACCAAA
GCTACAGAAGAACTTTATAAAGTTGGACTAATACGAGCAAACTACTCGGGATTGCTGTGGGGATTGACAGATGCGATGTCATTCTTCATCACTG
CAACTGTCTTTTATTATGCCACGGTTCTCATTACCAAGAGAGAGATCAGTATCGCGACTGCACTACAGACTGTCAATCTTCTATTATTTGGTAT
TTCTAATAGTACGAATATGCTGGCCATGATACCACAAATCAACTCTTCTCGCGTTACAGCTACGCATATGCTTGCATTAGCCAATCTCGATTCA
TCTTCCTCCCACGAAAATAAAGGAACCGAACGGCTTTCGACAATCTTTCCAATCAAATTCAACCGTCTTTCATTCACATACCCTACTCGTCCTG
AAAAACGAACGATATCATCCTTTTCTCTTTCCCTGATTCCTAACTCAACAACTGCACTTGTCGACCCTCCGGCTCCGGAAAATCTACAATAGTT
GCTCTGCTCATTGGTCTCTATCCGCCAGATACTTCAACACCTCCACCGTTGACATTCAATCGCGTCTCCATAAGTAACTGTCGCATTCCGTCTC
TCCGGGCTTCTCTCTCACTCGTTCCACAAATACCGATTCTATCTCCAGCTACCATTCTCCATAACATCATTTATGGTCTCCCAGAATCTTCTCC
TTGTGCTAGTCTTCCATCAGCTATTGCATCAGCAAAAGATGCTGGGATCCATGAATTTATCACATCGCTTCCACAAGGTTATGATACTAT

>Bcin05g07690(MLST2), partial sequence [organism=Botrytis cinerea, strain U11_SC_BR02]
ACCACTATCACCAAGTCCTTTTCTGCACTTTCCTCATCAAAAACGAACGAGAACATCATCAATAAATACTTTGCTGAAGTAATTGGGTTTTATT
TTGGCCAGGATGCCTTCTCTCTACGACAGCAAGCCTTCGATGATATGTTGTGGGTAGTTCTTGGCTGGCTGGATACTGTCAAATTCATTGATTT
ACATTCTGAATTGCACTATTCAAACGACTCTCAGCCAGAATGGTACGGACAACAATATAAACCTGCATTTGCACATCGAGCGCGACTATTTTGG
GAATTGGCTTCACAAGGATGGGATACTACTCTCTGTGGTGGTGGGATGATATGGTCACCATACCTTACTCCATACAAGAACGCAATTACCAATG
AACTCTATATCGCAGCTTCGATATCGATGTACCTATATTTCCCCGGAGATGACAATCAATCCCCATTTATGCTTTCCAACCCTTCATATCCACC
TCACGATCCGAAATATCTACAGGCAGCTGTTGATGCTTACAAATGGCTGAATGGTTCCAACATGACGGATTTACAAGGATTATATGTCGACGGG
TACCATATCTCGAATCTTTCTGGCGGTGAAAACACCCATTGCGATTCTAGAAATGAGATGGTATATACCTACAATCAAGGTGTTTTGCTTACTG
GACAACGTGGTTTGTATGACGCAACCGCCGCACGATCATACCTTGTAGATGGCCACAAACTCATCGCGAATGTTATTAATGCCACAGGCTATGA
CCTGAAACACAATGTTGTCATCTCACCGCCACCCAAAGATGGTTCCGCATTGGCAAAGTGGTTTGGCCTGGGTAGGAATGGAATACTGGAAGAA

```
GGATGCGATTCAAGTGCTTCGTGTTCTCAAAATGGACAAACTTTCAAAGGCATATTCTTTCATCACTTGATTGCGTTCTGTAGTGATTTGCCAG
GGGAGCCTATTGCAGGGACGAAGGAAAGCTTAGAACTCGACAGAGTGTGGCATTCTGACAAATGCTCACAGTATACAAAATG

>Bcin06g01710(MLST3), partial sequence [organism=Botrytis cinerea, strain U11_SC_BR02]
GTGAGTCTGACTTTTGTATTTGAGCGTTAAGATAGACACTGATATACCAAGGCAATCACCTAATTTCATGGCGTTCTTCCCTGCATCATCTACC
TACCCTGGAATGCTGGGAGAATTATACTCAGCAGCTTTCACAGCACCTGCTTTCAATTGGATCTGTTCCCCTGCTGTGACAGAATTGGAGACGG
TTGTAATGGATTGGCTGGCCAAGCTTCTCAATCTCCCAGACTGTTATTTGTCTTCGACTCATGGTGGTGGTGTCATCCAAGGATCAGCCTCGGA
AGCTATCGTTACCGTTATGGTTGCTGCCCGCGATAAATATCTTCGTGAAACTACTGAAGGTCTGTCGGGCATTGAGCTCGAGGATGCGATTGCA
TATAAGAGGAGTAAGCTAGTTGCACTAGGAAGCGAAATGGCACACAGCTCCACGCAGAAAGCAGCGCAAATAGCTGGCGTTAGATTCCGATCGA
TTCCAGTACTCGCATCCAATGATTTCGCCATGACGGGTGATGATTTAGAGAAGGTGTTGAAAGAATGCAAATCTCAAGGATTGGAACCCTTCTA
TCTAACTTCGACTTTGGGAACAACATCTACATGCGCAGTTGACGACTTCGCATCTATTGCAACAGTACTTTCAAAATATGCACCTCCAGATGTT
GCAGGCGAGATCTGGGTTCACGTCGATGCTGCTTATGCAGGTGCAGCTTTGGTTTGCCCTGAATACCATCATCTAACATCGTCCTTCCAGCATT
TCCATTCCTTCGATATGAACATGCACAAATGGCTTCTGACAAATTTCGACGCTTCTTGTCTATATGTCAAGAAACGCAAAGATCTGATCGATGC
ACTCTCCATAACACCAAGTTATCTTCGCAACGAGTTTTCAGAGAGTGGACTCGTAACCGACTATCGGGACTGGCAAATTCCTCTCGGAAGACGC
TTCCGAAGCTTAAAGATTTGGTTTGTCCTCAGAACCTAC

>Bcin09g03030(MLST4), partial sequence [organism=Botrytis cinerea, strain U11_SC_BR02]
GACCCTTCAACCCCACCGCAGCTCCTATACCGAAAGCGAGTATCCTACCAATTCTCCTTCCACCTGCGACTTTAAGACCATTGGCTTTCCGCAC
TTTCACAAAAAAGCATAGTTTAACATTGACGTCGTCGGCATTACAAGTGTTGGCTACTTTTATTGGAAAGCATTGTGGGACAGGATGGAGGGAA
GAAGGACTGGCAGAGAGAGTCTTGGAGGAGGTCGCCAAGAGTTGGAAGAATAGGAGTGGCGGTGTCATTGTCGAGGGCGAGGGAACGGAATTGA
AGGAGATTCTGAAAGCTTTGGAAGGGAATATGAGTGGTGGAAGGATAGTCATAGGAAGAGAGCTAAGCCGGCAGAATAGTTTAGTACTGGGATC
ATCACAATATGGAGAGGTCAATCATACAAGACTTGGGCTACGGCCAGGGAATATACCCAGAGAGGATAGTCAGTCAAGTTTGGGAATGTCAACG
TTGGAGGTCAATGACGAGGAAGATGAGGATGGCCTGATGGATCCAAGAAGGTGGTTAAAAGTCATTGATGCATTTGAGCAACCTCGACTGGTGT
ACAATGTTGCTAAAAAGCACTTTGATAGGTATGTTTCAATGATAAAATTTCATCTGAATCGACTAACTCAGTACAGAGATACCTCCAAACCTTC
ATTGTTCCCACCTGCGTCTCATAAAACACTCCTCTTCCAAAACCGCTATAATGTTATCCATCAACGTCTCCTTCGCAATGAATCTTTTCAAACG
CCCGCTTTTCAAGGTGGCAAATCTTCCCTTCAACGCAGCACGTCCGCCATTACCACCCAACAACAATCATACAAATTAACGCCGATAGCTAATC
TTCTCGGTCGCAATCGCAGCTCTCATATGCTTCTCGGTCTCCTCAGTATTTCACCCACTGGTACCCTCGCCATCAATGACCTGACGGGCAGTAT
CGCTCTCGATCTTACACACGCAGCAGCCATTC

>Bcin11g01310(MLST5), partial sequence [organism=Botrytis cinerea, strain U11_SC_BR02]
ACTGACATGGACCTCATGTGGAACCGGCGTAGAATGCGCAACGCTTGAAGTTCCGCTCGAATATGGCGATGCAACGTCAACGGCAAAAGCCAGT
GTTGCGCTTGCTCGTTATCCTGCCACTGTTGCCGCGAGCAAGAAGCTCGGGTCTCTCTTGATAAATCCCGGTGGACCCGGTGCCTCTGGTGTTG
GCTTTGTGCAGTCTGGAGCCGGTGCCGCCGTCTCGACACTGAGTGGTGGATTGTACGATATCATCGGATGGGATCCACGTGGAACCGGTGCTTC
GGCTCCTATTTTGGAATGTTTTGCAAATGCCAGTGCGGAGTATGATTTTAACAACGCGTTTCCATCTGCTCCGAATCTCTGGCTCGGACAATTT
GCGAATGCCAGCGCAAATTCTGCTGTTAGCTCTGCTATCACATCCTTTGACACTTCTGTCGCTGCTCTTGCAAAAGCTTGCGTGGCTCAGAAAT
CTCCCGCTCTTTACACCTCAACAGCAGCATATGTTGCTCGAGACATGGCAGCGATAGTCGATGCATTGGATGGGACCTCTGCAAAACTTAACTA
CTGGGGTTTCTCATATGGAACTATCTTCCTAGCTGAGTTTATCCAAACTTTCCCAGGCCGCGTGGGAAGAGTTCTTGCCGATGGTGTTTTCGAC
GCAAAGGCAAATGCACTCACATACGTTAGCCAACTTCCCAACGATCAACTCAGTGTTCGTGCTTCGTTGAACGATTTTGCAGCTTTCTGCACCA
CCGCCGGTAGTAAAGGTTGCTCTTTTGCCACCGCCCCTACTGGAACCACAGGTACTGTTGCTACCAGACTGGACAACATAATGAAGGATATGTT
CCTCAATCCTATTGTTGCTTCGGGCTTAAGCATCA

>Bcin15g03910(MLST6), partial sequence [organism=Botrytis cinerea, strain U11_SC_BR02]
CGCCAAAACACAAAATCATCCAACGATGAAGATGATACTCCACTTCCCTTGATTATCTGGCATGGACTCGGCGATAATTACAAAGCGGATGGTC
TTGCGCAAGTTGGAAAACTAGCTGAAGCTATTCATCCTGGGACTTTTGTCTACAATATTCATGTAGATGAGGATGCATCTGCAGATAGGACAGC
TACCTTCTTTGGAAATCTCACTCGTGAGTACATCCCCTATTTTTCCTTTAAATATCATACTAACTCTCTTACCAAGTTCAAATCGAAAAGGTCT
GCGAAGACCTCGCCTCCCATCCTATTCTCTCTACCGCGCCCGCCGTCGACGCAATTGGATTCTCCCAAGGCGGCCAATTCTTGCGTGGTTACAT
ATCCCGCTGCAATGCTCCACCCATCCGCTCTCTCCTGACCTTCGGTTCCCAACACAACGGCATTTCTGCCTTCCAAGCCTGTGGTCCTGCCGAT
TTCCTCTGTCGCGGTGCTCAAACCCTTTTGCGATCCAACACCTGGTCAACCTTTGTCCAATCTCGTCTCGTACCCGCTCAATACTTCAGAGATC
CGGAAAACCTAGACTCTTACCTTGAATATTCCAATTTCCTTGCCGACATCAATAATGAGCGCGTTCTCAAGAACCAAACATATAAATCCAACAT
GGAAAAATTGGAACGATTCGTAATGTATGTCTTTGAAGACGATACAACTGTCATCCCTAAGGAAAGTGGATGGTGGGCTGAAGTCAACGGCACG
GAAGTTACACCACTGAAAGAAAGAGCCATTTATAAAGAAGATTGGCTAGGTTTAAAGACATTGGATGAGGCCGGAAAATTAGTTTTCGAAACCA
TTCCAGGGGGACATATGACGTTAGGAGAGGAGATGCTAGAGAAGGCTTTCAAAGAGTATTTTGGTCCAGCAGGGAAGAAATTTGGG

>Bcin16g03460(MLST7), partial sequence [organism=Botrytis cinerea, strain U11_SC_BR02]
CATGAACCTCTTGATTTGAACTCTTCATTAATTGCAAGTGATGAACCCCTATTCTGCCCAGAGGATAGTTACAAGACGTATATCATTAGTCGAG
AACCACTCATGATATATATTGACGGATTTTTGAAAGCGAATGAAAGTAAACATTTGGTTGATGTTAGGTGTGTTATTTATTCTGATGAAATGAA
CAAGAGAGACTGATGAGATAGTGAGCCGCTTTATGAACCGTCAACTGTTTCTCACGGACAGGAAGTTACCATTGATCCTTCAGTTCGGAATTCT
```

GAAGTGGCGGTTTTAGAGAGGGATGAGGTGGTCAGGTGTATTGAGCATAGAGCGAGGGCATTTCAGGGGTGGAGGGGCGAGATGGGGATTGAGA
AGTTGAGGACGCAGAGGTATGGGGTTGGAGGACATTATGGGATGCATTTGTAAGTTTTGGGGGATTGACGAAGGCTTTTGTCTATTTTCTACCA
GTACGGATCTTGAAAGAAAGATAGCATGAACACGAGGGCTAATAACTAGGAAATAGCGATTGGAGCGGAGGTAAACGTGGCATAGACCGATTTA
GTACTTTCATGGTCTATGTCGACGTATCCTCTGATATCGAAGGTGGAGGAACGGAATTCCCACGTATCGTGGGACCAAAAGGAGGAAGGTGGGA
GGACTTCCTGGAAACTACGGAAGCATTGGATCCAAGAACTGGAGAAAATGTAACAGTAGAAGGGGTGACATTCAAACCAATCAAGGGAAATGCC
GTATTCTGGGAAAATACTGACAACAACGGGAGGGGCTATGAT

>Bcin12g03020(MLST8), partial sequence [organism=Botrytis cinerea, strain U11_SC_BR02]
CGATTGGCTGCGAAGAAAACTGCGCAGCACCGAGTCACCAAAAGCACGAATAAACCATCGGCAAGGGAAATATTCTCTGAACCACAACATGACA
CGAGCGCGGAAGAGTATATCGGGCGAGAAGCTTCATCAAGAGCACCAAGCGACAACGAGTCGATGATAATTATAACTCTTACGGTGGAAGAAAT
GAAAATACAGCAGCTTATGCTTCCGGGAAACTTCCATCTGGAAGTATAAATGTTGGTGGAGGTAGGAAGACCACTTTTCAAGAAGAACCACGAA
CGGCATTTGTCGCTGGCAAGTTGCCCCCTGGAAGTATCAACATTGGTGGAAAGAAGGCTACCCAAATCGAGGACGAGGGTAGAGCAGCTTATGC
TTCCGGAAAATTGCCTCCAGGAAGTATAAACTCTGGCGCAAAGAAGGCGATTTCATTCCAAGATGAAACGAGACCGGCTTATGTCTCCGGAAAG
CTTCCACATGGTAGTATCGACGGTATGCGAAACCGTGAAATGGCTGCCGTCCACCGCGAAATTGCTGAGGGTGGGAGGAAACCAGGCCAGGTTG
TCTCTTCTCTATTCACATTCAATCCTACTTCAAAGAAAACTTTTGATGAACCCGAAGAACAAGCGGAACCGGCAAAACCATCCAATGCGCCTTT
GACAGAGGAAATGGCGACTTTCACCAATCTAGGGTTATCGAGAAGGCTTGCAGCCCATCTATCGACTAAACTCGATATGAAAGCCCCGACGGCC
ATTCAAAAAGCATCTGTGCAGCAGTTGGTATCGGACGATAGCGATGCTTTCATACAAGCAGAGACTGGATCTGGAAAAACTTTGGCATATCTAC
TACCTATAGTCGAGCGAATATTAGCATTGAGTGAGAATGGCGTACAAAT

>Bcin02g07770(MLST9), partial sequence [organism=Botrytis cinerea, strain U11_SC_BR02]
CAGCTTTCCCTTTCGGTCTTGGCATCTACAGTCATTGCCATCCCTACACCATCACAACTTGAGTCTCGGGCCGTTATCGATTCCGATGCCGTTG
TAGGATTTGCCGAAACTGTTCCCAGTGGGACCGTAGGAACAGTTTATGAGGCATATAAACCATTCCTTAAAGTCGTAAATGGATGCGTACCATT
CCCTGCCGTCGATGCATCGGGTAACACAGGGTATGTCCTTATATCTTTCTCTTCCACACGATTGCTATTGAGTCTCTAACATATTTTAGTGGTG
GTTTGTCACCAACTGGCAGTAGCAATGGTGGTTGCAGCAGCAGTACCGGTCAAGTATATGTTCGAGGAGGACAAAGCGGATCAAACTACGCCAT
CATGTACTCCTGGTAAGTTCTCTCTAAACTTCTCCTTATAGATCCAACCTAACAAAATCTTAGGTACATGCCAAAGGACGAGCCCTCAACCGGT
ATTGGTCACCGTCACGATTGGGAAGGTGTAATTGTCTGGCTCTCCAGCGCCACCGCCACAACTGCCGACAACATCTTAGCCGTTTGTCCTTCCG
CCCACGGAGGCTGGGATTGTTCCACGGATGGCTATTCCCTTTCTGGTACCAGCCCTCTTATCAAGTACGAAAGTATCTGGCCCGTCGATCACTC
AATGGGTCTTACTAGTACTGTTGGTGGAAAACAACCTATGATTGCTTGGGAGTCTTTACCAACTGCTGCTCAAACTGCTCTTGAGAACACCGAT
TTCGGTGCTGCGAATGTTCCATTCATTCCGGCTGTTTTCACAGACAATCTT

>Bcin04g02090(MLST10), partial sequence [organism=Botrytis cinerea, strain U11_SC_BR02]
CGGAGGATGATATGGCAAAGTCTATGATTACCAAAGCATTTGTAGGCATGAGTAGTAAACTGTCGAATATGACTATGAATGGTGTTTACAAGCC
CTACATCCATGTAAGAAATGTAGAATAGAGAGATCAGTAACTGGAACTAATATCGTTTGTAGGCTTTCAAGTTACTTACGCAGTTCAACCCAAT
CACTACAGCTATTGCCGAATCCCCACTATTTCAAATGGCTGTCTCAGCAAATACCATCGAAAAGTACACACTGCTAGGCCCTTTCTTCAGAATA
TCTCCTCTGCAACAGGAAGTTACCAGGGAATACTTCAGTGCGCCAAAGACGATAGATAGGCGACACATTGCCACATCTCAAGATGCCTTACGAT
TGACCTTACAAACCCATCAAAAAGATTTACTTGATATCATCAACCGCTTTGTTCGAGCAAGTCCAATCGCCAAAAGCAAAACCTTGGATTGGTT
CGCCTACATTGTGAATCAAAATCACAAGCGTCGAGCACTTCAGGTAGACCCGAAAGAAGTGTCTTCTGATGGCTTTATGCACAATGTCACTGTC
GTTCTAGATGGTCTTTGTGAGCCATTCATGGATACCACATTCTCGAAAATTTCGAAGATTGATATTGATTATCTAAGACGTGCGCCTCGTGTAG
ATATCAAGGACGAGACCAAGTTGAACGCTGACGAGAAGGCTTCTGAGAAGTATTATGAGGACACTGTTCCTGGCACTTCTAATTTCATCTCTGA
GGTCTTCTTTCTCACATTGGCTGCTCATCATTATGGTAGTGAAGCTCTTAATGCCACGCATAAGAGTCTGGAGAAAGACATCAAATATATTCAA
AAGCAATTGACTGCCGTTGAAGCA

>Bcin01g07220(MLST1), partial sequence [organism=Botrytis fabae, strain 2230]
ATGGCATATCTTGTTTCTTTTCGCATTAAGCCTTGGAACGTGCTGGACAAGCTTGGGTTAGTGCTCTGCGTGTAGAAGCACTAAAGAAGATTCT
CGCACAACCGAAGTCATGGTTTGAGGATCCAGGAATTCACCTGGCCGGTTGAACGAAGTTTTGGATAGGAACTCTGAGGAAATGCGTAATCTCG
TTGGCCGCTTTGCTGGTATTGTATTCACAGCATTTTTTATGCTATTGATATCGATCATTTGGGCTTTCGTGAATACATGGAAACTGACATTAGT
CTCAATGGCAACTGGGCCAGTTATATACGCTGTCACTAAAACGTTCAATCGCGTGAGTGGAAAATGGGAAAACAAGTGCAACTACGCATCTGAA
ATGACCACTGGCATATTTTCAGAGACTTTCTCCAACATCAAAGTGGTTCGGGCTTTTACTCTGGAAACTTACTTTGAGACAAAACACACCAAAG
CTACAGAAGAACTTTATAAAGTTGGACTAATACGAGCAAACTACTCAGGATTGCTGTGGGGATTGACAGATGCGATGTCATTCTTCATCACTGC
AACTATCTTTTATTATGCCACGGTTCTCATTACCAAGAGAGAGATCAGTATCGCGACTGCACTACAGACTGTCAATCTTCTATTATTTGGTATT
TCTAATAGTACGAATATGCTGGCCATGATACCACAAATCAACTCTTCTCGCGTTACAGCTACGCATATGCTTGCATTAGCCAATCTCGATTCAT
CTTCCTCCCACGAAAATAAAGGAACCGAACGGCTTTCGACAATCTTTCCAATCAAATTCAACCGTCTCTCATTCACATATCCTACACGTCCTGA
AAAACGAACGATATCATCCTTTTCTCTTTCCCTGATTCCTAACTCAACAACTGCACTTGTCGGACCCTCCGGCTCCGGAAAATCTACAATAGCT
GCTCTGCTCATTGGTCTCTATCCGCCAGATACTTCAACACCTCCACCGTTGACATTCAATCGCGTCTCCATAAGTAACTGTCACATTCCGTCTC
TCCGGGCTTCTATCTCACTCGTCCCACAAATACCGATTCTATTTCCAGCTACCATTCTCCATAACATCATTTATGGTCTCCCAGAATCTTCTCC
TTGTGCTAGTCTTCCATCTGCTATTGCATCAGCAAAAGATGCTGGGATCCATGAATTTATCACATCGCTTCCACAAGGTTATGATACTAT

>Bcin05g07690(MLST2), partial sequence [organism=Botrytis fabae, strain 2230]
ACCACTATCACCAAGTCCTTTTCTGCACTTTCCTCATCCAAAAAGAACGAGAACATCATCAACAAATACTTTGCTGAAGTAATTGGGTTTTATT
TTGGCCAGGATGCCTTCTCTCTACGACAGCAAGCCTTCGATGATATGTTGTGGGTAGTTCTTGGTTGGCTGGATACTGTCAAGTTCATTGATTT
ACATTCTGAATTGCACTATTCAAACGACTCTCAGCCAGAATGGTACGGACAACAATATAAACCTGCATTTGCACATCGAGCGCGACTATTTTGG
GAATTGGCTTCACAAGGATGGGATACTACTCTCTGTGGTGGTGGGATGATATGGTCACCATACCTTACTCCACACAAGAACGCAATTACCAATG
AACTCTATATCGCAGCTTCGATATCGATGTACCTCTATTTCCCCGGAGATGGCAATCAATCCCCATTTATACTTTCCAACTCTTCATATCCACC
TCACGATCCGAAATATCTACAGGCAGCTGTTGACGCTTACAAATGGCTGAATGGTTCCAACATGACGGATTTACAAGGATTATATGTCGACGGA
TATCATATCTCGAATCTTTCTGGCGGTGAAAACACCCATTGCGATTCTAGAAATGAGATGGTATATACCTACAATCAGGGTGTTTTGCTTACTG
GACAACGTGGTCTGTATGACGCAACCGCCGCACGATCATACCTTGTGGATGGCCACAAACTCATTGCGAATGTTATTAATGCCACAGGCTATGA
CCTGAAACACAATGTTGTCATCTCACCGCCACCCAAAGATGGTTCCGCATTGGCAAAGTGGTTTGGCCTGGGTAGGAATGGAATACTGGAAGAA
GGACGCGATTCAAGTGCTTCGTGTTCTCAAAATGGACAAACTTTCAAAGGCATATTCTCTCATCACTTGATTGCATTCTGTAGTGATTTGCCAG
GGGAGCCTATTGCAGGGACGAAAGAAGGCCTAGAACTCGACAGAGTGTGGCATTCTGACAAATGCTCACAGTATACAAAATG

>Bcin06g01710(MLST3), partial sequence [organism=Botrytis fabae, strain 2230]
GTGAGTCTGACTTTTGTATTTGAGCGTTAAGATAGACACTGATATACCAAGGCAATCACCTAATTTCATGGCGTTCTTCCCTGCATCATCTACC
TACCCTGGAATGCTGGGAGAATTATACTCAGCAGCTTTCACAGCACCTGCTTTCAATTGGATCTGTTCCCCTGCTGTGACAGAATTGGAGACGG
TTGTAATGGATTGGCTGGCCAAGCTTCTCAATCTCCCAGACTGTTATTTGTCTTCGACTCATGGTGGTGGTGTTATCCAAGGATCAGCCTCGGA
AGCTATCGTTACCGTTATGGTTGCTGCCCGCGATAAATATCTTCGTGAAACCACTGAAGGTCTGTCGGGCATTGAACTCGAGGATGCGATTGCA
TATAAGAGGAGTAAGCTAGTTGCACTAGGAAGCGAAATGGCACACAGCTCCACGCAGAAAGCAGCGCAGATAGCTGGCGTTAGATTCCGATCGA
TTCCAGTACTCGCATCCAATGATTTTGCCATGACGGGTGATGATTTAGAGAAGGTATTGAAAGAATGCAAATCTCAAGGATTGGAACCATTCTA
TCTAACTTCGACTTTGGGAACAACATCTACATGCGCAGTTGACGACTTCGCATCTATTGCAACAGTACTTTCAAAATATGCACCTCCAGATGTT
GCAGGCGAGATCTGGGTTCACGTCGATGCTGCTTATGCAGGTGCAGCTTTGGTTTGCCCTGAATACCATCATCTAACATCGTCCTTCCAGCATT
TTCATTCCTTCGATATGAACATGCACAAATGGCTTCTGACAAATTTCGACGCTTCTTGTCTATATGTCAAGAAACGCAAAGATCTGATCGATGC
ACTCTCCATAACACCAAGCTATCTTCGCAACGAGTTTTCAGAGAGTGGACTCGTAACCGACTATCGGGACTGGCAAATTCCTCTCGGAAGACGC
TTCCGAAGCTTAAAGATTTGGTTTGTCCTCAGAACCTACG

>Bcin09g03030(MLST4), partial sequence [organism=Botrytis fabae, strain 2230]
ACCCTTCAACCCCACCGCAGCTCCTATACCGAAAGCGAGCATCCTGCCAATTCTCCTTCCACCTGCGACTTTAAGACCATTGGCTTTTCGCACT
TTCACAAAAAAGCATAGCTTAACATTGACGTCGTCGGCATTACAAGTGTTGGCTACTTTTATTGGGAAGCATTGTGGGACAGGATGGAGGGAAG
AAGGACTGGCAGAGAGAGTCTTGGAGGAGGTCGCCAAGAGTTGGAAGAATAGGAGTGGCGGTATTATTGTCGAGGGCGAGGGAACGGAATTGAA
GGAGATTCTGAAAGCTTTGGAAGGGAATATGAGTGGTGGAAGGATAGTCGTAGGAAGAGAGCTAAGCCGGCAGAATAGTTTAGTACTGGGATCA
TCACAATATGGAGAGGTCAATCATACAAGACTTGGGCTACGGCCAGGGAATATACCCAGAGAGGATAGTCAGTCAAGTTTGGGAATGTCAACGT
TGGAGGTCAATGACGAGGAAGATGAGGATGGCCTGATGGATCCAAGAAGGTGGATAAAAGTCATTGATGCATTTGAGCAACCTCGACTGGTGTA
TAATGTTGCTAAAAAGCACTTTGATAGGTATGTTTCAATGATAAAATTTTGTCTGAATCGACTAACTCAGTACAGAGATACCTCCAAACCTTCA
TTGTTCCCACCTGCGTCTCATAAAACACTCCTCTTCCAAAACCGCTATAATGTTATCCATCAACGTCTCCTTCGCAATGAATCTTTTCAAACGC
CCGCTTTTCAAGGTGGCAAAGCTTCCCTTCAACGCAGCACGTCCGCCATTACCACCCAACAACAATCATACAAATTAACGCCGATAGCTAATCT
TCTCGGTCGCAATCGCAGCTCTCATATGCTTCTCGGTCTCCTCAGTATTTCGCCCACTGGTACCCTCGCCATCAATGACCTGACGGGCAGCATC
GCTCTCGATCTTACACACGCAGCAGCCATTC

>Bcin11g01310(MLST5), partial sequence [organism=Botrytis fabae, strain 2230]
ACTGACATGGACCTCATGTGGAACCGGCGTAGAATGCGCAACGCTTGAAGTTCCGCTCGAATATGGCGATACAACGTCAACGGCAAAAGCCAGT
GTTGCGCTTGCTCGTTATCCTGCCACTGTTGCCGCGAGCAAGAAGCTCGGGTCTCTATTGATAAATCCCGGTGGACCCGGTGCCACTGGTGTTG
GCTTTTTGCAGTCTGGAGCCGGTGCTGCCGCCTCGACACTGAGTGGTGGATTATACGATATCATCGGATGGGATCCACGTGGAACCGGTGCTTC
GGCTCCTATTTTGGAATGTTTTGCAAATGCCAGTGCGGAGTATGATTTTAACAACGCGTTTCCATCTGCTCCGAATCTCTGGCTCGGACAATTT
CAAATGCCAGCGCAAATTCTGCTGTTAGCTCTGCTATCACATCCTTTGACACTTCTGTCGCTGCTCTTGCAAAAGCTTGCGTGGCTCAGAAATC
TCCCGCTCTTTACACCTCAACAGCGGCATATGTTGCTCGAGACATGGCAGCGATAGTCGATGCATTGGATGGGACCTCTGCAAAACTTAACTAC
TGGGGTTTCTCATATGGAACTATCTTCCTAGCTGAGTTTATCCAAACTTTCCCAGGCCGCGTGGGAAGAGTTCTTGCCGATGGTGTTTTCGACG
CAAAGGCAAATGCACTCACATACGTTAGCCAACTTCCCAACGATCAACTCAGTGTTCGTGCTTCGTTGAACGATTTTGCAGCTTTCTGCACCAC
CGCCGGTAGTAAAGGTTGCTCTTTTGCCACCGCCCCTACTGGAACCTCAGGTATTGTTGCTACCAGACTGGACAACATAATGAAGGATATGTTC
CTCAATCCTATTGTTGCTTCGGGCTTGAGCATCA

>Bcin15g03910(MLST6), partial sequence [organism=Botrytis fabae, strain 2230]
GTCAAAACACAAAATCATCCAACGATGAAGATGATACTCCACTTCCCTTGATTATCTGGCATGGACTCGGCGATAATTACAAAGCGGATGGTCT
TGCGCAAGTTGGAAAACTAGCTGAAGCTATTCATCCTGGGACTTTTGTCTATAATATTCATGTAGACGAGGATGCATCTGCAGATAGGACAGCT
ACCTTCTTTGGAAATCTCACTCGTGAGTACATCCCCTATTTTTCCTTTAAATACCATACTAACTCTCTTACCAAGTTCAAATCGAAAGGTCTGC
GAAGACCTCGCCTCCCATCCTATTCTCTCTACCGCGCCCGCCGTCGACGCAATTGGATTCTCCCAAGGCGGCCAATTCTTGCGCGGTTACATAT
CCCGCTGCAATGCTCCACCCATCCGCTCTCTCCTGACCTTCGGTTCCCAACACAACGGCATTTCTGCCTTCCAAGCCTGTGGTCCTGCCGATTT

CCTCTGTCGCGGTGCTCAAACCCTTTTGCGATCCAACACCTGGTCAACCTTTGTCCAATCTCGTCTCGTACCCGCTCAATACTTCAGAGATCCG
GAAAACCTAGACTCTTACCTTGAATATTCCAATTTCCTTGCCGACATCAATAATGAGCGCGTTCTCAAGAACCAAACATATAAATCCAACATGG
AAAAATTGGAACGATTCGTAATGTATGTCTTTGAAGACGATACAACTGTCATCCCTAAGGAAAGTGGATGGTGGGCTGAAGTCAACGGCACGGA
AGTTACACCACTGAAAGAAAGAGCCATTTATAAAGAAGATTGGCTAGGTTTAAAGACATTGGATGAGGCCGGAAAATTAGTTTTCGAAACCATT
CCAGGGGGACATATGACGTTAGGAGAGGAGATGCTAGAGAAGACTTTCAAGGAGTATTTTGGTCCAGCAGGGAAGAATTTGGGG

>Bcin16g03460(MLST7), partial sequence [organism=Botrytis fabae, strain 2230]
ATGAACCTCTTAATTTGAACTCTTCATTAATTGCAAGTGATGAACCCTTATTTTGCCCAGAGGATAGTTACAAGACGTATATCATTAGTCGAGA
ACCACTCATGATATATATTGACGGATTTTTGAAAGCGAATGAAAGTAAACATTTGGTTGATGTTAGGTGTGTTAATCATTCTGATGAAATGAAC
AAGAGAGACTGATGAGATAGTGAACCGCTTTATGAACCGTCTACTGTTTCTCACGGGCAGGAAGTTACCATTGATACTTCAGTTCGAAATTCTG
AAGTGGCGGTTTTAGAGAGGGATGAGGTGGTCAGGTGTATTGAGCATAGAGCGAGGGCATTTCAGGGGTGGAGGGGCGAGATGGGGATTGAGAA
GTTGAGGACGCAGAGGTATGGGGTTGGAGGACATTATGGGATGCATTTGTAAGTTTTGGGGGATTGACGAAGGCTTCTGTCTATTTTCTATCAG
TACGGACCTTGAAAGAAAGATAGCATGAACACGCGGGCTAACAACTAGGAAATAGCGATTGGAGCGGAGGTAAACGTGACATAGACCGATTTAG
TACTTTCATGGTCTATGTCGACGTATCCTCTGATATCGAAGGTGGAGGAACGGAATTCCCACGTATTGTGGGACCAAAAGGAGGAAGGTGGGAG
GACTTCCTGGAAACTACGGAAGCATTGGATCCAAGAACTGGACAAAATGTAACAGTAGAAGGGGTGACATTCAAACCAATCAAGGGAAATGCCG
TATTCTGGGAAAATACTGACAGCAACGGGAGGGGCTACGAT

>Bcin12g03020(MLST8), partial sequence [organism=Botrytis fabae, strain 2230]
CGATTGGCTGCGAAGAAAACTGCGCAGCACCGAGTCACCAAAAGCACGAATAAACCATCGGCAAGAGAAATATTCTCTGAACCACAACATGACA
CGAGCGCGGAAGAGTATATCGGGCGAGAAGCTTCATCAAGAGCACCAAAGTGACAACGAGTCGATGATAATTATAACTCTTACGGTGGAAGAAA
TGAAAATACAGCAGCTTATGCTTCCGGGAAACTTCCATCTGGAAGTATAAATGTTGGTGGAGGTAGGAAGACCACTTTTCAAGAAGAACCACGA
ACGGCATTTGTCGCTGGTAAGTTGCCCCCTGGAAGTATCAACATTGGTGGAAAGAAGGCTACCCAAATCGAGGACGAGGGTAGAGCAGCTTATG
CTTCCGGAAAATTGCCTCCAGGAAGTATAAACTCTGGCGCAAAGAAGGCGATTTCATTCCAAGATGAAACGAGACCGGCTTATGTCTCCGGAAA
GCTTCCACATGGTAGTATCGACAGTATGCGAAACCGTGAAATGGCTGCCGTCCACCGCGAAATTGCTGAGGGTGGGAGGAAACCAGGCCAGGTT
GTCTCTTCTCTATTCACATTCAATCCTACTTCAAAGAAAACCTTCGATGAACCCGAAGAACAAGCGGAACCGGCAAAACCATCCAATGCGCCTT
TGACAGAGGAAATGGCGACTTTCACCAATCTAGGGTTATCGAGAAGGCTTGCAGCCCATCTATCGACTAAACTCGATATGAAAGCCCCGACAGC
CATTCAAAAAGCATCTGTACAGCAGTTGGTATCGGACGATAGCGATGCTTTCATACAAGCAGAGACTGGATCTGGAAAAACTTTGGCATATCTA
CTACCTATAGTCGAGCGAATATTAGCATTGAGTGAGAATGGCGTACAGA

>Bcin02g07770(MLST9), partial sequence [organism=Botrytis fabae, strain 2230]
CAGCTTTCCCTTTCGGTCTTGGCATCTACAGTCATTGCCATCCCTACACCATCACAACTTGAGTCTCGGGCCGTTATCGATTCCGATGCCGTTG
TAGGATTTGCCGAAACTGTTCCCAGTGGGACCGTAGGAACAGTTTATGAGGCATATAAACCATTCCTTAAAGTCGTAAATGGATGCGTACCATT
CCCTGCCGTCGATGCATCGGGTAACACAGGGTATGTCCTTATACTTTTCTCTTCCACACGATTGCTATTGAGTCTCTAACATATTTTAGTGGTG
GTTTGTCACCAACTGGCAGTAGCAATGGTGGTTGCAGCAGCAGTACCGGTCAAGTATACGTTCGAGGAGGACAAAGCGGATCAAACTACGCTAT
CATGTACTCCTGGTAAGTTCTCTCTAAACTTCTCCTTATAGATTCAACCTAACAAAATCTTAGGTACATGCCAAAGGACGAGCCCTCAACCGGT
ATTGGTCACCGTCACGATTGGGAAGGTGTAATTGTCTGGCTCTCAAGCGCCACCGCCACAACTGCCGACAACATCTTAGCCGTTTGTCCTTCCG
CCCACGGAGGCTGGGATTGTTCCACCGATGGCTATTCCCTTTCTGGTACCAGCCCTCTTATCAAGTACGAAAGTATCTGGCCCGTCGATCATTC
AATGGGTCTTACTAGTACTGTTGGTGGAAAACAACCTTTGATTGCTTGGGAGTCTTTACCAACCGCTGCTCAAACTGCTCTTGAGAACACCGAT
TTCGGTGCTGCGAATGTTCCATTCATTCCGGCTGTTTTCACAGATAATCT

>Bcin04g02090(MLST10), partial sequence [organism=Botrytis fabae, strain 2230]
CGGAGGATGATATGGCAAAGTCTATGATTACCAAAGCATTTGTAGGCATGAGTAGTAAACTGTCGAATATGACTATGAATGATGTTTACAAGCC
CTACATCCATGTAAGAAATGTAGAATAGAGAGATCAGTAACTGGAACTAATATCGTTTGCAGGCTTTCAAGTTACTTACGCAGTTCAACCCAAT
CACTACAGCTATTGCCGAATCCCCACTATTTCAAATGGCTGTCTCAGCAAATACCATCGAAAAGTACACACTGCTAGGCCCTTTCTTCAGAATA
TCTCCTCTGCAACAGGAAGTTACCAGGGAATACTTCAGTGCGCCGAAGACGATAGATAGGCGACACATTGCCACATCTCAAGATGCGTTACGAT
TGACCTTACAAACCCATCAAAAAGATTTACTTGATATCATCAACCACTTTGTTCGAGCAAGTCCAATCGCAAAAAGCAAAACCCTGGATTGGTT
CGCCTACATTGTGAATCAAAATCACAAACGTCGAGCACTTCAGGTAGACCCGAAAGAAGTGTCTTCTGATGGCTTTATGCACAATGTCACTGTC
GTTCTAGATGGTCTTTGTGAGCCATTCATGGATACCACATTCTCGAAAATTTCGAAGATTGATATTGATTATCTAAGACGTGCGCCTCGTGTAG
ATATCAAGGACGAGACCAAGTTGAACGCTGACGAGAAGGCTTCTGAGAAGTATTATGAGGACACTGTTCCTGGCACTTCTAATTTCATCTCTGA
GGTCTTCTTTCTCACATTGGCTGCTCATCATTATGGTAGTGAAGCTCTTAATGCCACGCATAAGAGTCTGGAGAAAGACATCAAATATATTCAA
AAGCAATTGACTGCCGTTGAAGCA

>Bcin01g07220(MLST1), partial sequence [organism=Botrytis fabae, strain 2235]
ATGGCATATCTTGTTTCTTTTCGCATTATGCCTTGGAACGTGCTGGACAAGCTTGGGTTAGTGCTCTGCGTGTAGAAGCACTAAAGAAGATTCT
CGCACAACCGAAGTCATGGTTTGAGGAATCCGGGAATTCACCTGGCCGGTTGAACGAAGTTTTGGATAGGAACTCTGAGGAAATGCGTAATCTC
GTTGGCCGCTTTGCTGGTATTGTATTCACAGCATTTTTATGCTATTGATATCGATCATTTGGGCTTTCGTGAATACATGGAAACTGACATTAGT
CTCAATGGCAACTGGGCCAGTTATATACGCTGTCACTAAAACGTTCAATCGCGTGAGTGGAAAATGGGAAAACAAGTGCAACTACGCATCTGAA

ATGACCACTGGCATATTTTCAGAGACTTTCTCCAACATCAAAGTGGTTCGGGCTTTTACTCTGGAAACTTACTTTGAGACAAAACACACCAAAG
CTACAGAAGAACTTTATAAAGTTGGACTAATACGAGCAAACTACTCAGGATTGCTGTGGGGATTGACAGATGCGATGTCATTCTTCATCACTGC
AACTATCTTTTATTATGCCACGGTTCTCATTACCAAGAGAGAGATCAGTATCGCGACTGCACTACAGACTGTCAATCTTCTATTATTTGGTATT
TCTAATAGTACGAATATGCTGGCCATGATACCACAAATCAACTCTTCTCGCGTTACAGCTACGCATATGCTTGCATTAGCCAATCTCGATTCAT
CTTCCTCCCACGAAAATAAAGGAACCGAACGGCTTTCGACAATCTTTCCAATCAAATTCAACCGCCTCTCATTCACATATCCTACACGTCCTGA
AAAACGAACGATATCATCCTTTTCTCTTTCCCTGATTCCTAACTCAACAACTGCACTTGTCGGACCCTCCGGCTCCGGAAAATCTACAATAGCT
GCTCTGCTCATTGGTCTCTATCCGCCAGATACTTCAACACCTCCACCGTTGACATTCAATCGCGTCTCCATAAGTAACTGTCACATTCCGTCTC
TCCGGGCTTCTATCTCACTCGTCCCACAAATACCGATTCTATTTCCAGCTACCATTCTCCATAACATCATTTATGGTCTCCCAGAATCTTCTCC
TTGTGCTAGTCTTCCATCTGCTATTGCATCAGCAAAAGATGCTGGGATCCATGAATTTATCACATCGCTTCCACAAGGTTATGATACTAT

>Bcin05g07690(MLST2), partial sequence [organism=Botrytis fabae, strain 2235]
CACCACTATCACCAAGTCCTTTTCTGCACTTTCCTCATCCAAAAAGAACGAGAACATCATCAACAAATACTTTGCTGAAGTAATTGGGTTTTAT
TTTGGCCAGGATGCCTTCTCTCTACGACAGCAAGCCTTCGATGATATGTTGTGGGTAGTTCTTGGTTGGCTGGATACTGTCAAGTTCATTGATT
TACATTCTGAATTGCACTATTCAAACGACTCTCAGCCAGAATGGTACGGACAACAATATAAACCTGCATTTGCACATCGAGCGCGACTATTTTG
GGAATTGGCTTCACAAGGATGGGATACTACTCTCTGTGGTGGTGGGATGATATGGTCACCATACCTTACTCCATACAAGAACGCAATTACCAAT
GAACTCTATATCGCAGCTTCGATATCGATGTACCTCTATTTCCCCGGAGATGGCAATCAATCCCCATTTATACTTTCCAACTCTTCATATCCAC
CTCACGATCCGAAATATCTACAGGCAGCTGTTGACGCTTACAAATGGCTGAATGGTTCCAACATGACGGATTTACAAGGATTATATGTCGACGG
ATATCATATCTCGAATCTTTCTGGCGGTGAAAACACCCATTGCGATTCTAGAAATGAGATGGTATATACCTACAATCAGGGTGTTTTGCTTACT
GGACAACGTGGTCTGTATGACGCAACCGCCGCACGATCATACCTTGTGGATGGCCACAAACTCATTGCGAATGTTATTAATGCCACAGGCTATG
ACCTGAAACACAATGTTGTCATCTCACCGCCACCCAAAGATGGTTCCGCATTGGCAAAGTGGTTTGGCCTGGGTAGGAATGGAATACTGGAAGA
AGGATGCGATTCAAGTGCTTCGTGTTCTCAAAATGGACAAACTTTCAAAGGCATATTCTTTCATCACTTGATTGCATTCTGTAGTGATTTGCCA
GGGGAGCCTATTGCAGGGACGAAAGAAGGCCTAGAACTCGACAGAGTGTGGCATTCTGACAAATGCTCACAGTATACAAAAT

>Bcin06g01710(MLST3), partial sequence [organism=Botrytis fabae, strain 2235]
GGTGAGTCTGACCTTTTGTATTTGAGCGTTAAGATAGACACTGATATACCAAGGCAATCACCTAATTTTCATGGCGTTCTTTCCCTGCATCATC
TTACCTACCCTGGAATGCTGGAGAATTATACTCAGCAGCTTTCACAGCACCTGCTTTCAATTGGATCTGTTCCCCTGCTGTGACAGAATTGGAG
ACGGTTGTAATGGATTGGCTGGGCCAAGCTTCTCAATCTCCCAGACTGTTATTTGTCTTCGACTCATGGTGGTGGTGTTATCCAAGGATCAGCC
TCGGAAGCTATCGTTACCGTTATGGTTGCTGCCCCGCGATAAATATCTTCGTGAAACCACTGAAGGTCTGTCGGGCATTGAACTCGAGGATGCG
ATTGCATATAAGAGGAGTAAGCTAGTTGCACTAGGAAGCGAAATGGCACACAGCTCCACGCAGAAAGCAGCGCAGATAGCTGGCGTTAGATTCC
GATCGATTCCAGTACTCGCATCCAATGATTTTGCCATGACGGGTGATGATTTAGGAGAAGGTATTGAAAGAATGCAAATCTCAAGGATTGGAAC
CATTCTATCTAACTTCGACTTTGGAACAACATCTACATGCGCAGTTCGACGACTTCGCATCTATTGCAACAGTACTTTCAAAATATGCACCTCC
AGATGGTTGCAGGCGAGATCTGGGTTCACGTCGATGCTGCTTATGCAGGTGCAGCTTTGGTTTGCCCTGAATACCATCAATCTAACATCGTCCT
TCCAGCATTTTCATTCCTTCGATATGAACATGCACAAATGGCTTCTGACAAAATTTCGACGCTTCTTGTCTATATGTCAAGAAACGCAAAGATC
TGATCGATGCACTCTCCATAACACCAAGCTATCTTCGCAACGAGTTTTCAGAGAGTGGACTCGTAACCGGACTATCGGGACTGGCAAATTCCTC
TCGGAAGACGCTTCCGAAGCTTAAAGATTTGGTTTGTCCTCAGAACCTAC

>Bcin09g03030(MLST4), partial sequence [organism=Botrytis fabae, strain 2235]
ACCCTTCAACCCCACCGCAGCTCCTATACCGAAAGCGAGCATCCTGCCAATTCTCCTTCCACCTGCGACTTTAAGACCATTGGCTTTTCGCACT
TTCACAAAAAAGCATAGCTTAACATTGACGTCGTCGGCATTACAAGTGTTGGCTACTTTTATTGGAAAGCATTGTGGGACAGGATGGAGGGAAG
AAGGACTGGCAGAGAGAGTCTTGGAGGAGGTCGCCAAGAGTTGGAAGAATAGGAGTGGCGGTATTATTGTCGAGGGCGAGGGAACGGAATTGAA
GGAGATTCTGAAAGCTTTGGAAGGGAATATGAGTGGTGGAAGGATAGTCGTAGGAAGAGAGCTAAGCCGGCAGAATAGTTTAGTACTGGGATCA
TCACAATATGGAGAGGTCAATCATACAAGACTTGGGCTACGGCCAGGGAATATACCAGAGAGGATAGTCAGTCAAGTTTGGGAATGTCAACGTT
GGAGGTCAATGACGAGGAAGATGAGGATGGCCTGATGGATCCAAGAAGGTGGATAAAAGTCATTGATGCATTTGAGCAACCTCGACTGGTGTAT
AATGTTGCTAAAAAGCACTTTGATAGGTATGTTTCAATGATAAAATTTTGTCTGAATCGACTAACTCAGTACAGAGATACCTCCAAACCTTCAT
TGTTCCCACCTGCGTCTCATAAAACACTCCTCTTCCAAAACCGCTATAATGTTATCCATCAACGTCTCCTTCGCAATGAATCTTTTCAAACGCC
CGCTTTTCAAGGTGGCAAAGCTTCCCTTCAACGCAGCACGTCCGCCATTACCACCCAACAACAATCATACAAATTAACGCCGATAGCTAATCTT
CTCGGTCGCAATCGCAGCTCTCATATGCTTCTCGGTCTCCTCAGTATTTTCGCCCACTGGTACCCTCGCCATCAATGACCTGACGGGCAGCATC
GCTCTCGATCTTACACACGCAGCAGCCATTC

>Bcin11g01310(MLST5), partial sequence [organism=Botrytis fabae, strain 2235]
GACTGACATGGACCTCATGTGGAACCGGCGTAGAATGCGCAACGCTTGAAGTTCCGCTCGAATATGGCGATACAACGTCAACGGCAAAAGCCAG
TGTTGCGCTTGCTCGTTATCCTGCCACTGTTGCCGCGAGCAAGAAGCTCGGGTCTCTATTGATAAATCCCGGTGGACCCGGTGCCACTGGTGTT
GGCTTTTTGCAGTCTGGAGCCGGTGCTGCCGTCTCGACACTGAGTGGTGGATTATACGATATCATCGGATGGGATCCACGTGGAACCGGTGCTT
CGGCTCCTATTTTGGAATGTTTTGCAAATGCCAGTGCGGAGTATGATTTTAACAACGCGTTTCCATCTGCTCCGAATCTCTGGCTCGGACAATT
TTCAAATGCCAGCGCAAATTCTGCTGTTAGCTCTGCTATCACATCCTTTGACACTTCTGTCGCTGCTCTTGCAAAAGCTTGCGTGGCTCAGAAA
TCTCCCGCTCTTTACACCTCAACAGCAGCATATGTTGCTCGAGACATGGCAGCGATAGTCGATGCATTGGATGGGACCTCTGCAAAACTTAACT
ACTGGGGTTTCTCATATGGAACTATCTTCCTAGCTGAGTTTATCCAAACTTTCCCAGGCCGCGTGGGAAGAGTTCTTGCCGATGGTGTTTTCGA

CGCAAAGGCAAATGCACTCACATACGTTAGCCAACTTCCCAACGATCAACTCAGTGTTCGTGCTTCGTTGAACGATTTTGCAGCTTTCTGCACC
ACCGCCGGTAGTAAAGGTTGCTCTTTTGCCACCGCCCCTACTGGAACCTCAGGTATTGTTGCTACCAGACTGGACAACATAATGAAGGATATGT
TCCTCAATCCTATTGTTGCTTCGGGCTTGAGCATCAG

>Bcin15g03910(MLST6), partial sequence [organism=Botrytis fabae, strain 2235]
GTCAAAACACAAAATCATCCAACGATGAAGATGATACTCCACTTCCCTTGATTATCTGGCATGGACTCGGCGATAATTACAAAGCGGATGGTCT
TGCGCAAGTTGGAAAACTAGCTGAAGCTATTCATCCTGGGACTTTTGTCTATAATATTCATGTAGACGAGGATGCATCTGCAGATAGGACAGCT
ACCTTCTTTGGAAATCTCACTCGTGAGTACATCCCCTATTTTTCCTTTAAATACCATACTAACTCTCTTACCAAGTTCAAATCGAAAAGGTCTG
CGAAGACCTCGCCTCCCATCCTATTCTCTCTACCGCGCCCGCCGTCGACGCAATTGGATTCTCCCAAGGCGGCCAATTCTTGCGCGGTTACATA
TCCCGCTGCAATGCTCCACCCATCCGCTCTCTCCTGACCTTCGGTTCCCAACACAACGGCATTTCTGCCTTCCAAGCCTGTGGTCCTGCCGATT
TCCTCTGTCGCGGTGCTCAAACCCTTTTGCGATCCAACACCTGGTCAACCTTTGTCCAATCTCGTCTCGTACCCGCTCAATACTTCAGAGATCC
GGAAAACCTAGACTCTTACCTTGAATATTCCAATTTCCTTGCCGACATCAATAATGAGCGCGTTCTCAAGAACCAAACATATAAATCCAACATG
GAAAAATTGGAACGATTCGTAATGTATGTCTTTGAAGACGATACAACTGTCATCCCTAAGGAAAGTGGATGGTGGGCTGAAGTCAACGGCACGG
AAGTTACACCACTGAAAGAAAGAGCCATTTATAAAGAAGATTGGCTAGGTTTAAAGACATTGGATGAGGCCGGAAAATTAGTTTTCGAAACCAT
TCCAGGGGGACATATGACGTTAGGAGAGGAGATGCTAGAGAAGACTTTCAAGGAGTATTTTGGTCCAGCAGGGAAGAAATTTGGG

>Bcin16g03460(MLST7), partial sequence [organism=Botrytis fabae, strain 2235]
CATGAACCTCTTAATTTGAACTCTTCATTAATTGCAAGTGATGAACCCTTATTTTGCCCAGAGGATAGTTACAAGACGTATATCATTAGTCGAG
AACCACTCATGATATATATTGACGGATTTTTGAAAGCGAATGAAAGTAAACATTTGGTTGATGTTAGGTGTGTTAATCATTCTGATGAAATGAA
CAAGAGAGACTGATGAGATAGTGAACCGCTTTATGAACCGTCTACTGTTTCTCACGGGCAGGAAGTTACCATTGATACTTCAGTTCGAAATTCT
GAAGTGGCGGTTTTAGAGAGGGATGAGGTGGTCAGGTGTATTGAGCATAGAGCGAGGGCATTTCAGGGGTGGAGGGGCGAGATGGGGATTGAGA
AGTTGAGGACGCAGAGGTATGGGGTTGGAGGACATTATGGGATGCATTTGTAAGTTTTGGGGGATTGACGAAGGCTTCTGTCTATTTTCTATCA
GTACGGACCTTGAAAGAAAGATAGCATGAACACGCGGGCTAACAACTAGGAAATAGCGATTGGAGCGGAGGTAAACGTGGCATAGACCGATTTA
GTACTTTCATGGTCTATGTCGACGTATCCTCTGATATCGAAGGTGGAGGAACGGAATTCCCACGTATTGTGGGACCAAAAGGAGGAAGGTGGGA
GGACTTCCTGGAAACTACGGAAGCATTGGATCCAAGAACTGGACAAAATGTAACAGTAGAAGGGGTGACATTCAAACCAATCAAGGGAAATGCC
GTATTCTGGGAAAATACTGACAGCAACGGGAGGGGCTACGAT

>Bcin12g03020(MLST8), partial sequence [organism=Botrytis fabae, strain 2235]
CGATTGGCTGCGAAGAAAACTGCGCAGCACCGAGTCACCAAAAGCACGAATAAACCATCGGCAAGAGAAATATTCTCTGAACCACAACATGACA
CGAGCGCGGAAGAGTATATCGGGCGAGAAGTTTCATCAAGAGCACCAAAGCGACAACGAGTCGATGATAATTATAACTCTTACGGTGGAAGAAA
TGAAAATACAGCAGCTTATGCTTCCGGGAAACTTCCATCTGGAAGTATAAATGTTGGTGGAGGTAGGAAGACCACTTTTCAAGAAGAACCACGA
ACGGCATTTGTCGCTGGTAAGTTGCCCCCTGGAAGTATCAACATTGGTGGAAAGAAGGCTACCCAAATCGAGGACGAGGGTAGAGCAGCTTATG
CTTCCGGAAAATTGCCTCCAGGAAGTATAAACTCTGGCGCAAAGAAGGCGATTTCATTCCAAGATGAAACGAGACCGGCTTATGTCTCCGGAAA
GCTTCCACATGGTAGTATCGACAGTATGCGAAACCGTGAAATGGCTGCCGTCCACCGCGAAATTGCTGAGGGTGGGGGAAACCAGGCCAGGTTG
TCTCTTCTCTATTCACATTCAATCCTACTTCAAAGAAAACCTTCGATGAACCCGAAGAACAAGCGGAACCGGCAAAACCATCCAATGCGCCTTT
GACAGAGGAAATGGCGACTTTCACCAATCTAGGGTTATCGAGAAGGCTTGCAGCCCATCTATCGACTAAACTCGATATGAAAGCCCCGACAGCC
ATTCAAAAAGCATCTGTACAGCAGTTGGTATCGGACGATAGCGATGCTTTCATACAAGCAGAGACTGGATCTGGAAAAACTTTGGCATACCTAC
TACCTATAGTCGAGCGAATATTAGCATTGAGTGAGAATGGCGTACAAA

>Bcin02g07770(MLST9), partial sequence [organism=Botrytis fabae, strain 2235]
CAGCTTTCCCTTTCGGTCTTGGCATCTACAGTCATTGCCATCCCTACACCATCACAACTTGAGTCTCGGGCCGTTATCGATTCCGATGCCGTTG
TAGGATTTGCCGAAACTGTTCCCAGTGGGACCGTAGGAACAGTTTATGAGGCATATAAACCATTCCTTAAAGTCGTAAATGGATGCGTACCATT
CCCTGCCGTCGATGCATCGGGTAACACAGGGTATGTCCTTATACTTTTCTCTTCCACACGATTGCTATTGAGTCTCTAACATATTTTAGTGGTG
GTTTGTCACCAACTGGCAGTAGCAATGGTGGTTGCAGCAGCAGTACCGGTCAAGTATACGTTCGAGGAGGACAAAGCGGATCAAACTACGCTAT
CATGTACTCCTGGCAAGTTCTCTCTAAACTTCTCCTTATAGATTCAACCTAACAAAATCTTAGGTACATGCCAAAGGACGAGCCCTCAACCGGT
ATTGGTCACCGTCACGATTGGGAAGGTGTAATTGTCTGGCTCTCAAGCGCCACCGCCACAACTGCCGACAACATCTTAGCCGTTTGTCCTTCCG
CCCACGGAGGCTGGGATTGTTCCACCGATGGCTATTCCCTTTCTGGTACCAGCCCTCTTATCAAGTACGAAAGTATCTGGCCCGTCGATCATTC
AATGGGTCTTACTAGTACTGTTGGTGGAAAACAACCTTTGATTGCTTGGGAGTCTTTACCAACCGCTGCTCAAACTGCTCTTGAGAACACCGAT
TTCGGTGCTGCGAATGTTCCATTCATTCCGGCTGTTTTCACAGATAATCT

>Bcin04g02090(MLST10), partial sequence [organism=Botrytis fabae, strain 2235]
CGGAGGATGATATGGCAAAGTCTATGATTACCAAAGCATTTGTAGGCATGAGTAGTAAACTGTCGAATATGACTATGAATGATGTTTACAAGCC
CTACATCCATGTAAGAAATGTAGAATAGAGAGATCAGTAACTGGAACTAATATCGTTTGCAGGCTTTCAAGTTACTTACGCAGTTCAACCCAAT
CACTACAGCTATTGCCGAATCCCCACTATTTCAAATGGCTGTCTCAGCAAATACCATCGAAAAGTACACACTGCTAGGCCCTTTCTTCAGAATA
TCTCCTCTGCAACAGGAAGTTACCAGGGAATACTTCAGTGCGCCAAAGACGATAGATAGGCGACACATTGCCACATCTCAAGATGCGTTACGAT
TGACCTTACAAACCCATCAAAAGATTTACTTGATATCATCAACCACTTTGTTCGAGCAAGTCCAATCGCAAAAAGCAAAACCCTGGATTGGTTC
GCCTACATTGTGAATCAAAATCACAAACGTCGAGCACTTCAGGTAGACCCGAAAGAAGTGTCTTCTGATGGCTTTATGCACAATGTCACTGTCG

TTCTAGATGGTCTTTGTGAGCCATTCATGGATACCACATTCTCGAAAATTTCGAAGATTGATATTGATTATCTAAGACGTGCGCCTCGTGTAGA
TATCAAGGACGAGACCAAGTTGAACGCTGACGAGAAGGCTTCTGAGAAGTATTATGAGGACACTGTTCCTGGCACTTCTAATTTCATCTCTGAG
GTCTTCTTTCTCACATTGGCTGCTCATCATTATGGTAGTGAAGCTCTTAATGCCACGCATAAGAGTCTGGAGAAAGACATCAAATATATTCAAA
AGCAATTGACTGCCGTTGAAGCA

>Bcin01g07220(MLST1), partial sequence [organism=Botrytis fabae, strain 2240]
ATGGCATATCTTGTTTCTTTTCGCATTATGCCTTGGAACGTGCTGGACAAGCTTGGGTTAGTGCTCTGCGTGTAGAAGCACTAAAGAAGATTCT
CGCACAACCGAAGTCATGGTTTGAGGAATCCAGGAATTCACCTGGCCGGTTGAACGAAGTTTTGGATAGGAACTCTGAGGAAATGCGTAATCTC
GTTGGCCGCTTTGCTGGTATTGTATTCACAGCATTTTTTATGCTATTGATATCGATCATTTGGGCTTTCGTGAATACATGGAAACTGACATTAG
TCTCAATGGCAACTGGGCCAGTTATATACGCTGTCACTAAAACGTTCAATCGCGTGAGTGGAAAATGGGAAAACAAGTGCAACTACGCATCTGA
AATGACCACTGGCATATTTTCAGAGACTTTCTCCAACATCAAAGTGGTTCGGGCTTTTACTCTGGAAACTTACTTTGAGACAAAACACACCAAA
GCTACAGAAGAACTTTATAAAGTTGGACTAATACGAGCAAACTACTCAGGATTGCTGTGGGGATTGACAGATGCGATGTCATTCTTCATCACTG
CAACTACCTTTTATTATGCCACGGTTCTCATTACCAAGAGAGAGATCAGTATCGCGACTGCACTACAGACTGTCAATCTTCTATTATTTGGTAT
TTCTAATAGTACGAATATGCTGGCCATGATACCACAAATCAACTCTTCTCGCGTTACAGCTACGCATATGCTTGCATTAGCCAATCTCGATTCA
TCTTCCTCCCACGAAAATAAAGGAACCGAACGGCTTTCGACAATCTTTCCAATCAAATTCAACCGTCTCTCATTCACATATCCTACACGTCCTG
AAAAACGAACGATATCATCCTTTTCTCTTTCCCTGATTCCTAACTCAACAACTGCACTTGTCGGACCCTCCGGCTCCGGAAAATCTACAATAGC
TGCTCTGCTCATTGGTCTCTATCCGCCAGATACTTCAACGCCTCCACCGTTGACATTCAATCGCGTCTCCATAAGTAACTGTCACATTCCGTCT
CTCCGGGCTTCTATCTCACTCGTCCCACAAATACCGATTCTATTTCCAGCTACCATTCTCCATAACATCATTTATGGTCTCCCAGAATCTTCTC
CTTGTGCTAGTCTTCCATCTGCTATTGCATCAGCAAAAGATGCTGGGATCCATGAATTTATCACATCGCTTCCACAAGGTTATGATACTAT

>Bcin05g07690(MLST2), partial sequence [organism=Botrytis fabae, strain 2240]
ACCACTATCACCAAGTCCTTTTCTGCACTTTCCTCATCCAAAAAGAACGAGAACATCATCAACAAATACTTTGCTGAAGTAATTGGGTTTTATT
TTGGCCAGGATGCCTTCTCTCTACGACAGCAAGCCTTCGATGATATGTTGTGGGTAGTTCTTGGTTGGCTGGATACTGTCAAGTTCATTGATTT
ACATTCTGAATTGCACTATTCAAACGACTCTCAGCCAGAATGGTACGGACAACAATATAAACCTGCATTTGCACATCGAGCGCGACTATTTTGG
GAATTGGCTTCACAAGGATGGGATACTACTCTCTGTGGTGGTGGGATGATATGGTCACCATACCTTACTCCATACAAGAACGCAATTACCAATG
AACTCTATATCGCAGCTTCGATATCGATGTACCTCTATTTCCCCGGAGATGGCAATCAATCCCCATTTATACTTTCCAACTCTTCATATCCACC
TCACGATCCGAAATATCTACAGGCAGCTGTTGACGCTTACAAATGGCTGAATGGTTCCAACATGACGGATTTACAAGGATTATATGTCGACGGA
TATCATATCTCGAATCTTTCTGGCGGTGAAAACACCCATTGCGATTCTAGAAATGAGATGGTATATACCTACAATCAGGGTGTTTTGCTTACTG
GACAACGTGGTCTGTATGACGCAACCGCCGCACGATCATACCTTGTGGATGGCCACAAACTCATTGCGAATGTTATTAATGCCACAGGCTATGA
CCTGAAACACAATGTTGTCATCTCACCGCCACCCAAAGATGGTTCCGCATTGGCAAAGTGGTTTGGCCTGGGTAGGAATGGAATACTGGAAGAA
GGATGCGATTCAAGTGCTTCGTGTTCTCAAAATGGACAAACTTTCAAAGGCATATTCTTTCATCACTTGATTGCATCCTGTAGTGATTTGCCAG
GGGAGCCTATTGCAGGGACGAAAGAAGGCCTAGAACTCGACAGAGTGTGGCATTCTGACAAATGCTCACAGTATACAAAAT

>Bcin06g01710(MLST3), partial sequence [organism=Botrytis fabae, strain 2240]
GGTGAGTCTGACTTTTGTATTTGAGCGTTAAGATAGACACTGATATACCAAGGCAATCACCTAATTTCATGGCGTTCTTCCCTGCATCATCTAC
CTACCCTGGAATGCTGGGAGAATTATACTCAGCAGCTTTCACAGCACCTGCTTTCAATTGGATCTGTTCCCCTGCTGTGACAGAATTGGAGACG
GTTGTAATGGATTGGCTGGCCAAGCTTCTCAATCTCCCAGACTGTTATTTGTCTTCGACTCATGGTGGTGGTGTTATCCAAGGATCAGCCTCGG
AAGCTATCGTTACCGTTATGGTTGCTGCCCGCGATAAATATCTTCGTGAAACCACTGAAGGTCTGTCGGGCATTGAACTCGAGGATGCGATTGC
ATATAAGAGGAGTAAGCTAGTTGCACTAGGAAGCGAAATGGCACACAGCTCCACGCAGAAAGCAGCGCAGATAGCTGGCGTTAGATTCCGATCG
ATTCCAGTACTCGCATCCAATGATTTTGCCATGACGGGTGATGATTTAGAGAAGGTATTGAAAGAATGCAAATCTCAAGGATTGGAACCATTCT
ATCTAACTTCGACTTTGGGAACAACATCTACATGCGCAGTTGACGACTTCGCATCTATTGCAACAGTACTTTCAAAATATGCACCTCCAGATGT
TGCAGGCGAGATCTGGGTTCACGTCGATGCTGCTTATGCAGGTGCAGCTTTGGTTTGCCCTGAATACCATCATCTAACATCGTCCTTCCAGCAT
TTTCATTCCTTCGATATGAACATGCACAAATGGCTTCTGACAAATTTCGACGCTTCTTGTCTATATGTCAAGAAACGCAAAGATCTGATCGATG
CACTCTCCATAACACCAAGCTATCTTCGCAACGAGTTTTCAGAGAGTGGACTCGTAACCGACTATCGGGACTGGCAAATTCCTCTCGGAAGACG
CTTCCGAAGCTTAAAGATTTGGTTTGTCCTCAGAACCTAC

>Bcin09g03030(MLST4), partial sequence [organism=Botrytis fabae, strain 2240]
ACCCTTCAACCCCACCGCAGCTCCTATACCGAAAGCGAGCATCCTGCCAATTCTCCTTCCACCTGCGACTTTAAGACCATTGGCTTTTCGCACT
TTCACAAAAAAGCATAGCTTAACATTGACGTCGTCGGCATTACAAGTGTTGGCTACTTTTATTGGAAAGCATTGTGGGACAGGATGGAGGGAAG
AAGGACTGGCAGAGAGAGTCTTGGAGGAGGTCGCCAAGAGTTGGAAGAATAGGAGTGGCGGTATTATTGTCGAGGGCGAGGGAACGGAATTGAA
GGAGATTCTGAAAGCTTTGGAAGGGAATATGAGTGGTGGAAGGATAGTCGTAGGAAGAGAGCTAAGCCGGCAGAATAGTTTAGTACTGGGATCA
TCACAATATGGAGAGGTCAATCATACAAGACTTGGGCTACGGCCAGGGAATATACCCAGAGAGGATAGTCAGTCAAGTTTGGGAATGTCAACGT
TGGAGGTCAATGACGAGGAAGATGAGGATGGCCTGATGGATCCAAGAAGGTGGATAAAAGTCATTGATGCATTTGAGCAACCTCGACTGGTGTA
TAATGTTGCTAAAAAGCACTTTGATAGGTATGTTTCAATGATAAAATTTTGTCTGAATCGACTAACTCAGTACAGAGATACCTCCAAACCTTCA
TTGTTCCCACCTGCGTCTCATAAAACACTCCTCTTCCAAAACCGCTATAATGTTATCCATCAACGTCTCCTTCGCAATGAATCTTTTCAAACGC
CCGCTTTTCAAGGTGGCAAAGCTTCCCTTCAACGCAGCACGTCCGCCATTACCACCCAACAACAATCATACAAATTAACGCCGATAGCTAATCT
TCTCGGTCGCAATCGCAGCTCTCATATGCTTCTCGGTCTCCTCAGTATTTCGCCCACTGGTACCCTCGCCATCAATGACCTGACGGGCAGCATC

GCTCTCGATCTTACACACGCAGCAGCCATTC

>Bcin11g01310(MLST5), partial sequence [organism=Botrytis fabae, strain 2240]
GACTGACATGGACCTCATGTGGAACCGGCGTAGAATGCGCAACGCTTGAAGTTCCGCTCGAATATGGCGATACAACGTCAACGGCAAAAGCCAG
TGTTGCGCTTGCTCGTTATCCTGCCACTGTTGCCGCGAGCAAGAAGCTCGGGTCTCTATTGATAAATCCCGGTGGACCCGGTGCCACTGGTGTT
GGCTTTTTGCAGTCTGGAGCCGGTGCTGCCGTCTCGACACTGAGTGGTGGATTATACGATATCATCGGATGGGATCCACGTGGAACCGGTGCTT
CGGCTCCTATTTTGGAATGTTTTGCAAAATGCCAGTGCGGAGTATGATTTTAACAACGCGTTTCCATCTGCTCCGAATCTCTGGCTCGGACAAT
TTTCAAATGCCAGCGCAAATTCTGCTGTTAGCTCTGCTATCACATCCTTTGACACTTCTGTCGCTGCTCTTGCAAAAGCTTGCGTGGCTCAGAA
ATCTCCCGCTCTTTACACCTCAACAGCAGCATATGTTGCTCGAGACATGGCAGCGATAGTCGATGCATTGGATGGGACCTCTGCAAAACTTAAC
TACTGGGGTTTCTCATATGGAACTATCTTCCTAGCTGAGTTTATCCAAACTTTCCAGGCCGCGTGGGAAGAGTTCTTGCCGATGGTGTTTTCGA
CGCAAAGGCAAATGCACTCACATACGTTAGCCAACTTCCCAACGATCAACTCAGTGTTCGTGCTCGTTGAACGATTTTGCAGCTTTCTGCACCA
CCGCCGGTAATAAAGGTTGCTCTTTTGCCACCGCCCCTACTGGAACCTCAGGTATTGTTGCTACCAGACTGGACAACATAATGAAGGATATGTT
CCTCAATCCTATTGTTGCTTCGGGCTTGAGCATCA

>Bcin15g03910(MLST6), partial sequence [organism=Botrytis fabae, strain 2240]
CGTCAAAACACAAAATCATCCAACGATGAAGATGATACTCCACTTCCCTTGATTATCTGGCATGGACTCGGCGATAATTACAAAGCGGATGGTC
TTGCGCAAGTTGGAAAACTAGCTGAAGCTATTCATCCTGGGACTTTTGTCTATAATATTCATGTAGACGAGGATGCATCTGCAGATAGGACAGC
TACCTTCTTTGGAAATCTCACTCGTGAGTACATCCCCTATTTTTCCTTTAAATACCATACTAACTCTCTTACCAAGTTCAAATCGAAAAGGTCT
GCGAAGACCTCGCCTCCCATCCTATTCTCTCTACCGCGCCCGCCGTCGACGCAATTGGATTCTCCCAAGGCGGCCAATTCTTGCGCGGTTACAT
ATCCCGCTGCAATGCTCCACCCATCCGCTCTCTCCTGACCTTCGGTTCCCAACACAACGGCATTTCTGCCTTCCAAGCCTGTGGTCCTGCCGAT
TTCCTCTGTCGCGGTGCTCAAACCCTTTTGCGATCCAACACCTGGTCAACCTTTGTCCAATCTCGTCTCGTACCCGCTCAATACTTCAGAGATC
CGGAAAACCTAGACTCTTACCTTGAATATTCCAATTTCCTTGCCGACATCAATAATGAGCGCGTTCTCAAGAACCAAACATATAAATCCAACAT
GGAAAAATTGGAACGATTCGTAATGTATGTCTTTGAAGACGATACAACTGTCATCCCTAAGGAAAGTGGATGGTGGGCTGAAGTCAACGGCACG
GAAGTTACACCACTGAAAGAAAGAGCCATTTATAAAGAAGATTGGCTAGGTTTAAAGACATTGGATGAGGCCGGAAAATTAGTTTTCGAAACCA
TTCCAGGGGGACATATGACGTTAGGAGAGGAGATGCTAGAGAAGACTTTCAAGGAGTATTTTGGTCCAGCAGGGAAGAAATTTGGG

>Bcin16g03460(MLST7), partial sequence [organism=Botrytis fabae, strain 2240]
ATGAACCTCTTAATTTGAACTCTTCATTAATTGCAAGTGATGAACCCTTATTTTGCCCAGAGGATAGTTACAAGACGTATATCATTAGTCGAGA
ACCACTCATGATATATATTGACGGATTTTTGAAAGCGAATGAAAGTAAACATTTGGTTGATGTTAGGTGTGTTAATCATTCTGATGAAATGAAC
AAGAGAGACTGATGAGATAGTGAACCGCTTTATGAACCGTCTACTGTTTCTCACGGGCAGGAAGTTACCATTGATACTTCAGTTCGAAATTCTG
AAGTGGCGGTTTTAGAGAGGGATGAGGTGGTCAGGTGTATTGAGCATAGAGCGAGGGCATTTCAGGGGTGGAGGGGCGAGATGGGGATTGAGAA
GTTGAGGACGCAGAGGTATGGGGTTGGAGGACATTATGGGATGCATTTGTAAGTTTTGGGGGATTGACGAAGGCTTCTGTCTATTTTCTATCAG
TACGGACCTTGAAAGAAAGATAGCATGAACACGCGGGCTAACAACTAGGAAATAGCGATTGGAGCGGAGGTAAACGTGGCATAGACCGATTTAG
TACTTTCATGGTCTATGTCGACGTATCCTCTGATATCGAAGGTGGAGGAACGGAATTCCCACGTATTGTGGGACCAAAAGGAGGAAGGTGGGAG
GACTTCCTGGAACTACGGAAGCATTGGATCCAAGAACTGACAAAATGTAACAGTAGAAGGGGTGACATTCAAACCAATCAAGGGAAATGCCGTA
TTCTGGGAAAATACTGACAGCAACGGGAGGGGCTACGAT

>Bcin12g03020(MLST8), partial sequence [organism=Botrytis fabae, strain 2240]
CGATTGGCTGCGAAGAAAACTGCGCAGCACCGAGTCACCAAAAGCACGAATAAACCATCGGCAAGAGAAATATTCTCTGAACCACAACATGACA
CGAGCGCGGAAGAGTATATCGGGCGAGAAGCTTCATCAAGAGCACCAAAGCGACAACGAGTCGATGATAATTATAACTCTTACGGTGGAAGAAA
TGAAAATACAGCAGCTTATGCTTCCGGGAAACTTCCATCTGGAAGTATAAATGTTGGTGGAGGTAGGAAGACCACTTTTCAAGAAGAACCACGA
ACGGCATTTGTCGCTGGTAAGTTGCCCCCTGGAAGTATCAACATTGGTGGAAAGAAGGCTACCCAAATCGAGGACGAGGGTAGAGCAGCTTATG
CTTCCGGAAAATTGCCTCCAGGAAGTATAAACTCTGGCGCAAAGAAGGCGATTTCATTCCAAGATGAAACGAGACCGGCTTATGTCTCCGGAAA
GCTTCCACATGGTAGTATCGACAGTATGCGAAACCGTGAAATGGCTGCCGTCCACCGCGAAATTGCTGAGGGTGGGAGGAAACCAGGCCAGGTT
GTCTCTTCTCTATTCACATTCAATCCTACTTCAAAGAAAACCTTCGATGAACCCGAAGAACAAGCGGAACCGGCAAAACCATCCAATGCGCCTT
TGACAGAGGAAATGGCGACTTTCACCAATCTAGGGTTATCGAGAAGGCTTGCAGCCCATCTATCGACTAAACTCGATATGAAAGCCCCGACAGC
CATTCAAAAAGCATCTGTACAGCAGTTGGTATCGGACGATAGCGATGCTTTCATACAAGCAGAGACTGGATCTGGAAAAACTTTGGCATATCTA
CTACCTATAGTCGAGCGAATATTAGCATTGAGTGAGAATGGCGTACAAA

>Bcin02g07770(MLST9), partial sequence [organism=Botrytis fabae, strain 2240]
CAGCTTTCCTTTCGGTCTTGGCATCTACAGTCATTGCCATCCCTACACCATCACAACTTGAGTCTCGGGCCGTTATCGATTCCGATGCCGTTGT
AGGATTTGCCGAAACTGTTCCCAGTGGGACCGTAGGAACAGTTTATGAGGCATATAAACCATTCCTTAAAGTCGTAAATGGATGCGTACCATTC
CCTGCCGTCGATGCATCGGGTAACACAGGGTATGTCCTTATACTTTTCTCCTCCACACGATTGCTATTGAGTCTCTAACATATTTTAGTGGTGG
TTTGTCACCAACTGGCAGTAGCAATGGTGGTTGCAGCAGCAGTACCGGTCAAGTATACGTTCGAGGAGGACAAAGCGGATCAAACTACGCTATC
ATGTACTCCTGGTAAGTTCTCTCTAAACTTCTCCTTATAGATTCAACCTAACAAAATCTTAGGTACATGCCAAAGGACGAGCCCTCAACCGGTA
TTGGTCACCGTCACGATTGGGAAGGTGTAATTGTCTGGCTCTCAAGCGCCACCGCCACAACTGCCGACAACATCTTAGCCGTTTGTCCTTCCGC
CCACGGAGGCTGGGATTGTTCCACCGATGGCTATTCCCTTTCTGGTACCAGCCCTCTTATCAAGTACGAAAGTATCTGGCCCGTCGATCATTCA

ATGGGTCTTACTAGTACTGTTGGTGGAAAACAACCTTTGATTGCTTGGGAGTCTTTACCAACCGCTGCTCAAACTGCTCTTGAGAACACCGATT
TCGGTGCTGCGAATGTTCCATTCATTCCGGCTGTTTTCACAGATAATCT

>Bcin04g02090(MLST10), partial sequence [organism=Botrytis fabae, strain 2240]
CGGAGGATGATATGGCAAAGTCTATGATTACCAAAGCATTTGTAGGCATGAGTAGTAAACTGTCGAATATGACTATGAATGATGTTTACAAGCC
CTACATCCATGTAAGAAATGTAGAATAGAGAGATCAGTAACTGGAACTAATATCGTTTGCAGGCTTTCAAGTTACTTACGCAGTTCAACCCAAT
CACTACAGCTATTGCCGAATCCCCACTATTTCAAATGGCTGTCTCAGCAAATACCATCGAAAAGTACACACTGCTAGGCCCTTTCTTCAGAATA
TCTCCTCTGCAACAGGAAGTTACCAGGGAATACTTCAGTGCGCCAAAGACGATAGATAGGCGACACATTGCCACATCTCAAGATGCGTTACGAT
TGACCTTACAAACCCATCAAAAAGATTTACTTGATATCATCAACCACTTTGTTCGAGCAAGTCCAATCGCAAAAAGCAAAACCCTGGATTGGTT
CGCCTACATTGTGAATCAAAATCACAAACGTCGAGCACTTCAGGTAGACCCGAAAGAAGTGTCTTCTGATGGCTTTATGCACAATGTCACTGTC
GTTCTAGATGGTCTTTGTGAGCCATTCATGGATACCACATTCTCGAAAATTTCGAAGATTGATATTGATTATCTAAGACGTGCGCCTCGTGTAG
ATATCAAGGACGAGACCAAGTTGAACGCTGACGAGAAGGCTTCTGAGAAGTATTATGAGGACACTGTTCCTGGCACTTCTAATTTCATCTCTGA
GGTCTTCTTTCTCACATTGGCTGCTCATCATTATGGTAGTGAAGCTCTTAATGCCACGCATAAGAGTCTGGAGAAAGACATCAAATATATTCAA
AAGCAATTGACTGCCGTTGAAGCA

>Bcin01g07220(MLST1), partial sequence [organism=Botrytis fabae, strain D12_B_B02]
ATGGCATATCTTGTTTCTTTTCGCATTATGCCTTGGAACGTGCTGGACAAGCTTGGGTTAGTGCTCTGCGTGTAGAAGCACTAAAGAAGATTCT
CGCACAACCGAAGTCATGGTTTGAGGAATCCAGGAATTCACCTGGCCGGTTGAACGAAGTTTTGGATAGGAACTCTGAGGAAATGCGTAATCTC
GTTGGCCGCTTTGCTGGTATTGTATTCACAGCATTTTTTATGCTATTGATATCGATCATTTGGGCTTTCGTGAATACATGGAAACTGACATTAG
TCTCAATGGCAACTGGGCCAGTTATATACGCTGTCACTAAAACGTTCAATCGCGTGAGTGGAAAATGGGAAAACAAGTGCAACTACGCATCTGA
AATGACCACTGGCATATTTTCAGAGACTTTCTCCAACATCAAAGTGGTTCGGGCTTTTACTCTGGAAACTTACTTTGAGACAAAACACACCAAA
GCTACAGAAGAACTTTATAAAGTTGGACTAATACGAGCAAACTACTCAGGATTGCTGTGGGGATTGACAGATGCGATGTCATTCTTCATCACTG
CAACTATCTTTTATTATGCCACGGTTCTCATTACCAAGAGAGAGATCAGTATCGCGACTGCACTACAGACTGTCAATCTTCTATTATTTGGTAT
TTCTAATAGTACGAATATGCTGGCCATGATACCACAAATCAACTCTTCTCGCGTTACAGCTACGCATATGCTTGCATTAGCCAATCTCGATTCA
TCTTCCTCCCACGAAAATAAAGGAACCGAACGGCTTTCGACAATCTTTCCAATCAAATTCAACCGTCTCTCATTCACATATCCTACACGTCCTG
AAAAACGAACGATATCATCCTTTTCTCTTTCCCTGATTCCTAACTCAACAACTGCACTTGTCGGACCCTCCGGCTCCGGAAAATCTACAATAGC
TGCTCTGCTCATTGGTCTCTATCCGCCAGATACTTCAACACCTCCACCGTTGACATTCAATCGCGTCTCCATAAGTAACTGTCACATTCCGTCT
CTCCGGGCTTCTATCTCACTCGTCCCACAAATACCGATTCTATTTCCAGCTACCATTCTCCATAACATCATTTATGGTCTCCCAGAATCTTCTC
CTTGTGCTAGTCTTCCATCTGCTATTGCATCAGCAAAAGATGCTGGATCCATGAATTTATCACATCGCTTCCACAAGGTTATGATACTAT

>Bcin05g07690(MLST2), partial sequence [organism=Botrytis fabae, strain D12_B_B02]
CACCACTATCACCAAGTCCTTTTCTGCACTTTCCTCATCCAAAAAGAACGAGAACATCATCAACAATACTTTGCTGAAGTAATTGGGTTTTATT
TTGGCCAGGATGCCTTCTCTCTACGACAGCAAGCCTTCGATGATATGTTGTGGGTAGTTCTTGGTTGGCTGGATACTGTCAAATTCATTGATTT
ACATTCTGAATTGCACTATTCAAACGACTCTCAGCCAGAATGGTACGGACAACAATATAAACCTGCATTTGCACATCGAGCGCGACTATTTTGG
GAATTGGCTTCACAAGGATGGGATACTACTCTCTGTGGTGGTGGGATGATATGGTCACCATACCTTACTCCATACAAGAACGCAATTACCAATG
AACTCTATATCGCAGCTTCGATATCGATGTACCTCTATTTCCCCGGAGATGGCAATCAATCCCCATTTATACTTTCCAACTCTTCATATCCACC
TCACGATCCGAAATATCTACAGGCAGCTGTTGACGCTTACAAATGGCTGAATGGTTCCAACATGACGGATTTACAAGGATTATATGTCGACGGA
TATCATATCTCGAATCTTTCTGGCGGTGAAAACACCCATTGCGATTCTAGAAATGAGATGGTATATACCTACAATCAGGGTGTTTTGCTTACTG
GACAACGTGGTCTGTATGACGCAACCGCCGCACGATCATACCTTGTGGATGGCCACAAACTCATCGCGAATGTTATTAATGCCACAGGCTATGA
CCTGAAACACAATGTTGTCATCTCACCGCCACCCAAAGATGGTTCCGCATTGGCAAAGTGGTTTGGCCTGGGTAGGAATGGAATACTGGAAGAA
GGATGCGATTCAAGTGCTTCGTGTTCTCAAAATGGACAAACTTTCAAAGGCATATTCTTTCATCACTTGATTGCATTCTGTAGTGATTTGCCAG
GGGAGCCTATTGCAGGGACGAAAGAAGGCCTAGAACTCGACAGAGTGTGGCATTCTGACAAATGCTCACAGTATACAAAATGG

>Bcin06g01710(MLST3), partial sequence [organism=Botrytis fabae, strain D12_B_B02]
GTGAGTCTGACTTTTGTATTTGAGCGTTAAGATAGACACTGATATACCAAGGCAATCACCTAATTTCATGGCGTTCCTCCCTGCATCATCTACC
TACCCTGGAATGCTGGGAGAATTATACTCAGCAGCTTTCACAGCACCTGCTTTCAATTGGATCTGTTCCCCTGCTGTGACAGAATTGGAGACGG
TTGTAATGGATTGGCTGGCCAAGCTCCTCAATCTCCCAGACTGTTATTTGTCTTCGACTCATGGTGGTGGTGTTATCCAAGGATCAGCCTCGGA
AGCTATCGTTACCGTTATGGTTGCTGCCCGCGATAAATATCTTCGTGAAACCACTGAAGGTCTGTCGGGCATTGAACTCGAGGATGCGATTGCA
TATAAGAGGAGTAAGCTAGTTGCACTAGGAAGCGAAATGGCACACAGCTCCACGCAGAAAGCAGCGCAGATAGCTGGCGTTAGATTCCGATCGA
TTCCAGTACCCGCATCCAATGATTTTGCCATGACGGGTGATGATTTAGAGAAGGTATTGAAAGAATGCAAATCTCAAGGATTGGAACCATTCTA
TCTAACTTCGACTTTGGGAACAACATCTACATGCGCAGTTGACGACTTCGCATCTATTGCAACAGTACTTTCAAAATATGCACCTCCAGATGTT
GCAGGCGAGATCTAGGTTCACGTCGATGCTGCTTATGCAGGTGCAGCTTTGGTTTGCCCTGAATACCATCATCTAACATCGTCCTTCCAGCATT
TTCATTCCTTCGATATGAACATGCACAAATGGCTTCTGACAAATTTCGACGCTTCTTGTCTATATGTCAAGAAACGCAAAGATCTGATCGATGC
ACTCTCCATAACACCAAGCTATCTTCGCAACGAGTTTTCAGAGAGTGGACTCGTAACCGACTATCGGGACTGGCAAATTCCTCTCGGAAGACGC
TTCCGAAGCTTAAAGATTTGGTTTGTCCTCAGAACCTACG

>Bcin09g03030(MLST4), partial sequence [organism=Botrytis fabae, strain D12_B_B02]

CGACCTTCAACCCCACCGCAGCTCCTATACCGAAAGCGAGCATCCTGCCAATTCTCCTTCCACCTGCGACTTTAAGACCATTGGCTTTTCGCAC
TTTCACAAAAAGCATAGCTTAACATTGACGTCGTCGGCATTACAAGTGTTGGCTACTTTTATTGGAAAGCATTGTGGACAGGATGGAGGGAAAA
GGACTGGCAGAGAGAGTCTTGGAGGAGGTCGCCAAGAGTTGGAAGAATAGGAGTGGCGGTGTTATTGTCGAGGGCGAGGGAACGAATTGAAGGA
GATTCTGAAAGCTTGGAAGGGAATATGAGTGGTGTGAAGGATAGTCGTAGGAAGAGAGCTAAGCCGGCAGAATAGTTTAGTACTGGGATCATCA
CAATATGGAGAGGTCAATCATACAAGACTTGGCTACGGCCAGGGAATATACCCAGAGAGGATAGTCAGTCAAGTTTGGGAATGTCAACGTTGGA
GGTCAAGGACGAGGAAGATGAGGATGGCCTGATGGATCAAGAAGGTGGATAAAAGTCATTGATGCATTTGAGCAACCTCGACTTGGTGTATAAT
GTTGCTAAAAAGCACTTTGATAGGTATGTTTTCAATGATAAAATTTTGTCTGAATTCGACTAACTCAGTACAGAGATACCTCCAAACCTTCATT
GTTCCCACCTGCGTCTCATAAAACACTCCTCTTCCAAAACCGCTATAATGTTATCCATCAACGTCTCCTTCGCAAATGAATCTTTTCAAACGCC
CGCTTTTCAAAAGGTGGCAAAGCTTCCCTTCAACGCAGCACGTCCGCCATTACCACCCAACAACAATCATACAAATTAACGCCGATAGCTAATC
TTCTCAGGTCGCAATCGCAGCTCTCATATGCTTCTCGGTCTCCTCAGTATTTCGCCCACTGGTACCCCCGCCATCAATGACCTGACGGGCAGCA
TCGCTCTCGATCTTACACACGCAGCAGCCATTC

>Bcin11g01310(MLST5), partial sequence [organism=Botrytis fabae, strain D12_B_B02]
ACTGACATGGACCTCATGTGGAACCGGCGTAGAATGCGCAACGCTTGAAGTTCCGCTCGAATATGGCGATACAACGTCAACGGCAAAAGCCAGT
GTTGCGCTTGCTCGTTATCCTGCCACTGTTGCCGCGAGCAAGAAGCTCGGGTCTCTATTGATAAATCCCGGTGGACCCGGTGCCACTGGTGTTG
GCTTTTTGCAGTCTGGAGCCGGTGCTGCCGTCTCGACACTGAGTGGTGGATTATACGATATCATCGGATGGGATCCACGTGGAACCGGTGCTTC
GGCTCCTATTTTGGAATGTTTTGCAAATGCCAGTGCGGAGTATGATTTTAACAACGCGTTTCCATCTGCTCCGAATCTCTGGCTCGGACAATTT
TCAAATGCCAGCGCAAATTCTGCTGTTAGCTCTGCTATCACATCCTTTGACACTTCTGTCGCTGCTCTTGCAAAAGCTTGCGTGGCTCAGAAAT
CTCCCGCTCTTTACACCTCAACAGCAGCATATGTTGCTCGAGACATGGCAGCGATAGTCGATGCATTGGATGGGACCTCTGCAAAACTTAACTA
CTGGGGTTTCTCATATGGAACTATCTTCCTAGCTGAGTTTATCCAAACTTTCCCAGGCCGCGTGGGAAGAGTTCTTGCCGATGGTGTTTTCGAC
GCAAAGGCAAATGCACTCACATACGTTAGCCAACTTCCCAACGATCAACTCAGTGTTCGTGCTTCGTTGAACGATTTTGCAGCTTTCTGCACCA
CCGCCGGTAGTAAAGGTTGCTCTTTTGCCACCGCCCCTACTGGAACCTCAGGTATTGTTGCTACCAGACTGGACAACATAATGAAGGATATGTT
CCTCAATCCTATTGTTGCTTCGGGCTTGAGCATCA

>Bcin15g03910(MLST6), partial sequence [organism=Botrytis fabae, strain D12_B_B02]
GTCAAAACACAAAATCATCCAACGATGAAGATGATACTCCACTTCCCTTGATTATCTGGCATGGACTCGGCGATAATTACAAAGCGGATGGTCT
TGCGCAAGTTGGAAAACTAGCTGAAGCTATTCATCCTGGGACTTTTGTCTATAATATTCATGTAGACGAGGATGCATCTGCAGATAGGACAGCT
ACCTTCTTTGGAAATCTCACTCGTGAGTACATCCCCTATTTTTCCTTTAAATACCATACTAACTCTCTTACCAAGTTCAAATCGAAAAGGTCTG
CGAAGACCTCGCCTCCCATCCTATTCTCTCTACCGCGCCCGCCGTCGACGCAATTGGATTCTCCCAAGGCGGCCAATTCTTGCGCGGTTACATA
TCCCGCTGCAATGCTCCACCCATCCGCTCTCTCCTGACCTTCGGTTCCCAACACAACGGCATTTCTGCCTTCCAAGCCTGTGGTCCTGCCGATT
TCCTCTGTCGCGGTGCTCAAACCCTTTTGCGATCCAACACCTGGTCAACCTTTGTCCAATCTCGTCTCGTACCCGCTCAATACTTCAGAGATCC
GGAAAACCTAGACTCTTGCCTTGAATATTCCAATTTCCTTGCCGACATCAATAATGAGCGCGTTCTCAAGAACCAAACATATAAATCCAACATG
GAAAAATTGGAACGATTCGTAATGTATGTCTTTGGAGACGATACAACTGTCATCCCTAAGGAAAGTGGATGGTGGGCTGAAGTCAACGGCACGG
AAGTTACACCACTGAAAGAAAGAGCCATTTATAAAGAAGATTGGCTAGGTTTAAAGACATTGGATGAGGCCGGAAAATTAGTTTTCGAAACCAT
TCCAGGGGGACATATGACGTTAGGAGAGGAGATGCTAGAGAAGACTTTCAAGGAGTATTTTGGTCCAGCAGGGAAGAAATTTGGG

>Bcin16g03460(MLST7), partial sequence [organism=Botrytis fabae, strain D12_B_B02]
ATGAACCTCTTAATTTGAACTCTTCATTAATTGCAAGTGATGAACCCTTATTTTGCCCAGAGGATAGTTACAAGACGTATATCATTAGTCGAGA
ACCACTCATGATATATATTGACGGATTTTTGAAAGCGAATGAAAGTAAACATTTGGTTGATGTTAGGTGTGTTAATCATTCTGATGAAATGAAC
AAGAGAGACTGATGAGATAGTGAACCGCTTTATGAACCGTCTACTGTTTCTCACGGGCAGGAAGTTACCATTGATACTTCAGTTCGAAATTCTG
AAGTGGCGGTTTAGAGAGGGATGAGGTGGTCAGGTGTATTGAGCATAGAGCGAGGGCATTTCAGGGGTGGAGGGGCGAGATGGGGATTGAGAAG
TTGAGGACGCAGAGGTATGGGGTTGGAGGACATTATGGGATGCATTTGTAAGTTTTGGGGGATTGACGAAGGCTTCTGTCTATTTTCTATCAGT
ACGGACCTTGAAAGAAAGATAGCATGAACACGCGGGCTAACAACTAGGAAATAGCGATTGGAGCGGAGGTAAACGTGGCATAGACCGATTTAGT
ACTTTCATGGTCTATGTCGACGTATCCTCTGATATCGAAGGTGGAGGAACGGAATTCCCACGTATTGTGGGACCAAAAGGAGGAAGGTGGGAGG
ACTTCCTGGAAACTACGGAAGCATTGGATCCAAGAACTGGACAAAATGTAACAGTAGAAGGGGTGACATTCAAACCAATCAAGGGAAATGCCGT
ATTCTGGGAAAATACTGACAGCAACGGGAGGGGCTACGATG

>Bcin12g03020(MLST8), partial sequence [organism=Botrytis fabae, strain D12_B_B02]
CGATTGGCTGCGAAGAAAACTGCGCAGCACCGAGTCACCAAAAGCACGAATAAACCATCGGCAAGAGAAATATTCTCTGAACCACAACATGACA
CGAGCGCGGAAGAGTATATCGGGCGAGAAGCTTCATCAAGAGCACCAAAGCGACAACGAGTCGATGATAATTATAACTCTTACGGTGGAAGAAA
TGAAAATACAGCAGCTTATGCTTCCGGGAAACTTCCATCTGGAAGTATAAATGTTGGTGGAGGTAGGAAGACCACTTTTCAAGAAGAACCACGA
ACGGCATTTGTCGCTGGTAAGTTGCCCCCTGGAAGTATCAACATTGGTGGAAAGAAGGCTACCCAAATCGAGGACGAGGGTAGAGCAGCTTATG
CTTCCGGAAAATTGCCTCCAGGAAGTATAAACTCTGGCGCAAAGAAGGCGATTTCATTCCAAGATGAAACGAGACCGGCTTATGTCTCCGGAAA
GCTTCCACATGGTAGTATCGACAGTATGCGAAACCGTGAAATGGCTGCCGTCCACCGCGAAATTGCTGAGGGTGGAGGAAACCAGGCCAGGTT
GTCTCTTCTCTATTCACATTCAATCCTACTTCAAAGAAAACCTTCGATGAACCCGAAGAACAAGCGGAACCGGCAAAACCATCCAATGCGCCTT
TGACAGAGGAAATGGCGACTTTCACCAATCTAGGGTTATCGAGAAGGCTTGCAGCCCATCTATCGACTAAACTCGATATGAAAGCCCCGACAGC
CATTCAAAAAGCATCTGTACAGCAGTTGGTATCGGACGATAGCGATGCTTTCATACAAGCAGAGACTGGATCTGGAAAAACTTTGGCATATCTA

CTACCTATAGTCGAGCGAATATTAGCATTGAGTGAGAATGGCGTACAAA

>Bcin02g07770(MLST9), partial sequence [organism=Botrytis fabae, strain D12_B_B02]
CAGCTTTCCCTTTCGGTCTTGGCATCTACAGTCATTGCCATCCCTACACCATCACAACTTGAGTCTCGGGCCGTTATCGATTCCGATGCCGTTG
TAGGATTTGCCGAAACTGTTCCCAGTGGGACCGTAGGAACAGTTTATGAGGCATATAAACCATTCCTTAAAGTCGTAAATGGATGCGTACCATT
CCCTGCCGTCGATGCATCGGGTAACACAGGGTATGTCCTTATACTTTTCTCTTCCACACGATTGCTATTGAGTCTCTAACATATTTTAGTGGTG
GTTTGTCACCAACTGGCAGTAGCAATGGTGGTTGCAGCAGCAGTACCGGTCAAGTATACGTTCGAGGAGGACAAAGCGGATCAAACTACGCTAT
CATGTACTCCTGGTAAGTTCTCTCTAAACTTCTCCTTATAGATTCAACCTAACAAAATCTTAGGTACATGCCAAAGGACGAGCCCTCAACCGGT
ATTGGTCACCGTCACGATTGGGAAGGTGTAATTGTCTGGCTCTCAAGCGCCACCGCCACAACTGCCGACAACATCTTAGCCGTTTGTCCTTCCG
CCCACGGAGGCTGGGATTGTTCCACCGATGGCTATTCCCTTTCTGGTACCAGCCCTCTTATCAAGTACGAAAGTATCTGGCCCGTCGATCATTC
AATGGGTCTTACTAGTACTGTTGGTGGAAAACAACCTTTGATTGCTTGGGAGTCTTTACCAACCGCTGCTCAAACTGCTCTTGAGAACACCGAT
TTCGGTGCTGCGAATGTTCCATTCATTCCGGCTGTTTTCACAGATAATCTT

>Bcin04g02090(MLST10), partial sequence [organism=Botrytis fabae, strain D12_B_B02]
CGGAGGATGATATGGCAAAGTCTATGATTACCAAAGCATTTGTAGGCATGAGTAGTAAACTGTCGAATATGACTATGAATGATGTTTACAAGCC
CTACATCCATGTAAGAAATGTAGAATAGAGAGATCAGTAACTGGAACTAATATCGTTTGCAGGCTTTCAAGTTACTTACGCAGTTCAACCCAAT
CACTACAGCTATTGCCGAATCCCCACTATTTCAAATGGCTGTCTCAGCAAATACCATCGAAAAGTACACACTGCTAGGCCCTTTCTTCAGAATA
TCTCCTCTGCAACAGGAAGTTACCAGGAATACTTCAGTGCGCCAAAGACGATAGATAGGCGACACATTGCCACATCTCAAGATGCGTTACGATT
GACCTTACAAACCCATCAAAAAGATTTACTTGATATCATCAACCACTTTGTTCGAGCAAGTCCAATCGCAAAAAGCAAAACCCTGGATTGGTTC
GCCTACATTGTGAATCAAAATCACAAACGTCGAGCACTTCAGGTAGACCCGAAAGAAGTGTCTTCTGATGGCTTTATGCACAATGTCACTGTCG
TTCTAGATGGTCTTTGTGAGCCATTCATGGATACCACATTCTCGAAAATTTCGAAGATTGATATTGATTATCTAAGACGTGCGCCTCGTGTAGA
TATCAAGGACGAGACCAAGTTGAACGCTGACGAGAAGGCTTCTGAGAAGTATTATGAGGACACTGTTCCTGGCACTTCTAATTTCATCTCTGAG
GTCTTCTTTCTCACGTTGGCTGCTCACCATTATGGTAGTGAAGCTCTTAATGCCACGCATAAGAGTCTGGAGAAAGACATCAAATATATTCAAA
AGCAATTGACTGCCGTTGAAGCA

>Bcin01g07220(MLST1), partial sequence [organism=Botrytis fabae, strain D12_B_B28]
GATGGCATATCTTGTTTCTTTTCGCATTATGCCTTGGAACGTGCTGGACAAGCTTGGGTTAGTGCTCTGCGTGTAGAAGCACTAAAGAAGATTC
TCGCACAACCGAAGTCATGGTTTGAGGAATCCAGGAATTCACCTGGCCGGTTGAACGAAGTTTTGGATAGGAACTCTGAGGAAATGCGTAATCT
CGTTGGCCGCTTTGCTGGTATTGTATTCACAGCATTTTTTATGCTATTGATATCGATCATTTGGGCTTTCGTGAATACATGGAAACTGACATTA
GTCTCAATGGCAACTGGGCCAGTTATATACGCTGTCACTAAAACGTTCAATCGCGTGAGTGGAAAATGGGAAAACAAGTGCAACTACGCATCTG
AAATGACCACTGGCATATTTTCAGAGACTTTCTCCAACATCAAAGTGGTTCGGGCTTTTACTCTGGAAACTTACTTTGAGACAAAACACACCAA
AGCTACAGAAGAACTTTATAAAGTTGGACTAATACGAGCAAACTACTCAGGATTGCTGTGGGGATTGACAGATGCGATGTCATTCTTCATCACT
GCAACTATCTTTTATTATGCCACGGTTCTCATTACCAAGAGAGAGACCAGTATCGCGACTGCACTACAGACTGTCAATCTTCTATTATTTGGTA
TTTCTAATAGTACGAATATGCTGGCCATGATACCACAAATCAACTCTTCTCGCGTTACAGCTACGCATATGCTTGCATTAGCCAATCTCGATTC
ATCTTCCTCCCACGAAAATAAAGGAACCGAACGGCTTTCGACAATCTTTCCAATCAAATTCAACCGTCTCTCATTCACATATCCTACACGTCCT
GAAAAACGAACGATATCATCCTTTTCTCTTTCCCTGATTCCTAACTCAACAACTGCACTTGTCGGACCCTCCGGCTCCGGAAAATCTACAATAG
CTGCTCTGCTCATTGGTCTCTATCCGCCAGATACTTCAGCACCTCCACCGTTGACATTCAATCGCGTCTCCATAAGTAACTGTCACATTCCGTC
TCTCCGGGCTTCTATCTCACTCGTCCCACAAATACCGATTCTATTTCCAGCTACCATTCTCCATAACATCATTTATGGTCTCCCAGAATCTTCT
CCTTGTGCTAGTCTTCCATCTGCTATTGCATCAGCAAAAGATGCTGGGATCCATGAATTTATCACATCGCTTCCACAAGGTTATGATACTATG

>Bcin05g07690(MLST2), partial sequence [organism=Botrytis fabae, strain D12_B_B28]
ACCACTATCACCAAGTCCTTTTCTGCACTTTCCTCATCCAAAAAGAACGAGAACATCATCAACAAATACTTTGCTGAAGTAATTGGGTTTTATT
TTGGCCAGGATGCCTTCTCTCTACGACAGCAAGCCTTCGATGATATGTTGTGGGTAGTTCTTGGTTGGCTGGATACTGTCAAGTTCATTGATTT
ACATTCTGAATTGCACTATTCAAACGACTCTCAGCCAGAATGGTACGGACAACAATATAAACCTGCATTTGCACATCGAGCGCGACTATTTTGG
GAATTGGCTTCACAAGGATGGGATACTACTCTCTGTGGTGGTGGGATGATATGGTCACCATACCTTACTCCATACAAGAACGCAATTACCAATG
AACTCTATATCGCAGCTTCGATATCGATGTACCTCTATTTCCCCGGAGATGGCAATCAATCCCCATTTATACTTTCCAACTCTTCATATCCACC
TCACGATCCGAAATATCTACAGGCAGCTGTTGACGCTTACAAATGGCTGAATGGTTCCAACATGACGGATTTACAAGGATTATATGTCGACGGA
TATCATATCTCGAATCTTTCTGGCGGTGAAAACACCCATTGCGATTCTAGAAATGAGATGGTATATACCTACAATCAGGGTGTTTTGCTTACTG
GACAACGTGGTCTGTATGACGCAACCGCCGCACGATCATACCTTGTGGATGGCCACAAACTCATTGCGAATGTTATTAATGCCACAGGCTATGA
CCTGAAACACAATGTTGTCATCTCACCGCCACCCAAAGATGGTTCCGCATTGGCAAAGTGGTTTGGCCTGGGTAGGAATGGAATACTGGAAGAA
GGATGCGATTCAAGTGCTTCGTGTTCTCAAAATGGACAAACTTTCAAAGGCATATTCTTTCATCACTTGATTGCATTCTGTAGTGATTTGCCAG
GGGAGCCTATTGCAGGGACGAAAGAAGGCCTAGAACTCGACAGAGTGTGGCATTCTGACAAATGCTCACAGTATACAAAAT

>Bcin06g01710(MLST3), partial sequence [organism=Botrytis fabae, strain D12_B_B28]
GTGAGTCTGACTTTTGTATTTGAGCGTTAAGATAGACACTGATATACCAAGGCAATCACCTAATTTCATGGCGTTCTTCCCTGCATCATCTACC
TACCCTGGAATGCTGGGAGAATTATACTCAGCAGCTTTCACAGCACCTGCTTTCAATTGGATCTGTTCCCCTGCTGTGACAGAATTGGAGACGG
TTGTAATGGATTGGCTGGCCAAGCTTCTCAATCTCCCAGACTGTTATTTGTCTTCGACTCATGGTGGTGGTGTTATCCAAGGATCAGCCTCGGA

AGCTATCGTTACCGTTATGGTTGCTGCCCGCGATAAATATCTTCGTGAAACCACTGAAGGTCTGTCGGGCATTGAACTCGAGGATGCGATTGCA
TATAAGAGGAGTAAGCTAGTTGCACTAGGAAGCGAAATGGCACACAGCTCCACGCAGAAAGCAGCGCAGATAGCTGGCGTTAGATTCCGATCGA
TTCCAGTACTCGCATCCAATGATTTTGCCATGACGGGTGATGATTTAGAGAAGGTATTGAAAGAATGCAAATCTCAAGGATTGGAACCATTCTA
TCTAACTTCGACTTTGGGAACAACATCTACATGCGCAGTTGACGACTTCGCATCTATTGCAACAGTACTTTCAAAATATGCACCTCCAGATGTT
GCAGGCGAGATCTGGGTTCACGTCGATGCTGCTTATGCAGGTGCAGCTTTGGTTTGCCCTGAATACCATCATCTAACATCGTCCTTCCAGCATT
TTCATTCCTTCGATATGAACATGCACAAATGGCTTCTGACAAATTTCGACGCTTCTTGTCTATATGTCAAGAAACGCAAAGATCTGATCGATGC
ACTCTCCATAACACCAAGCTATCTTCGCAACGAGTTTTCAGAGAGTGGACTCGTAACCGACTATCGGGACTGGCAAATTCCTCTCGGAAGACGC
TTCCGAAGCTTAAAGATTTGGTTTGTCCTCAGAACCTAC

>Bcin09g03030(MLST4), partial sequence [organism=Botrytis fabae, strain D12_B_B28]
ACCCTTCAACCCCACCGCAGCTCCTATACCGAAAGCGAGCATCCTGCCAATTCTCCTTCCACCTGCGACTTTAAGACCATTGGCTTTTCGCACT
TTCACAAAAAAGCATAGCTTAACATTGACGTCGTCGGCATTACAAGTGTTGGCTACTTTTATTGGAAAGCATTGTGGGACAGGATGGAGGGAAG
AAGGACTGGCAGAGAGAGTCTTGGAGGAGGTCGCCAAGAGTTGGAAGAATAGGAGTGGCGGTATTATTGTCGAGGGCGAGGGAACGGAATTGAA
GGAGATTCTGAAAGCTTTGGAAGGGAATATGAGTGGTGGAAGGATAGTCGTAGGAAGAGAGCTAAGCCGGCAGAATAGTTTAGTACTGGGATCA
TCACAATATGGAGAGGTCAATCATACAAGACTTGGGCTACGGCCAGGGAATATACCCAGAGAGGATAGTCAGTCAAGTTTGGGAATGTCAACGT
TGGAGGTCAATGACGAGGAAGATGAGGATGGCCTGATGGATCCAAGAAGGTGGATAAAAGTCATTGATGCATTTGAGCAACCTCGACTGGTGTA
TAATGTTGCTAAAAAGCACTTTGATAGGTATGTTTCAATGATAAAATTTTGTCTGAATCGACTAACTCAGTACAGAGATACCTCCAAACCTTCA
TTGTTCCCACCTGCGTCTCATAAAACACTCCTCTTCCAAAACCGCTATAATGCTATCCATCAACGTCTCCTTCGCAATGAATCTTTTCAAACGC
CCGCTTTTCAAGGTGGCAAAGCTTCCCTTCAACGCAGCACGTCCGCCATTACCACCCAACAACAATCATACAAATTAACGCCGATAGCTAATCT
TCTCGGTCGCAATCGCAGCTCTCATATGCTTCTCGGTCTCCTCAGTATTTCGCCCACTGGTACCCTCGCCATCAATGACCTGACGGGCAGCATC
GCTCTCGATCTTACACACGCAGCAGCCATTCC

>Bcin11g01310(MLST5), partial sequence [organism=Botrytis fabae, strain D12_B_B28]
GACTGACATGGACCTCATGTGGAACCGGCGTAGAATGCGCAACGCTTGAAGTTCCGCTCGAATATGGCGATACAACGTCAACGGCAAAAGCCAG
TGTTGCGCTTGCTCGTTATCCTGCCACTGTTGCCGCGAGCAAGAAGCTCGGGTCTCTATTGATAAATCCCGGTGGACCCGGTGCCACTGGTGTT
GGCTTTTTGCAGTCTGGAGCCGGTGCTGCCGTCTCGACACTGAGTGGTGGATTATACGATATCATCGGATGGGATCCACGTGGAACCGGTGCTT
CGGCTCCTATTTTGGAATGTTTTGCAAATGCCAGTGCGGAGTATGATTTTAACAACGCGTTTCCATCTGCTCCGAATCTCTGGCTCGGACAATT
TTCAAATGCCAGCGCAAATTCTGCTGTTAGCTCTGCTATCACATCCTTTGACACTTCTGTCGCTGCTCTTGCAAAAGCTTGCGTGGCTCAGAAA
TCTCCCGCTCTTTACACCTCAACAGCAGCATATGTTGCTCGAGACATGGCAGCGATAGTCGATGCATTGGATGGGACCTCTGCAAAACTTAACT
ACTGGGGTTTCTCATATGGAACTATCTTCCTAGCTGAGTTTATCCAAACTTTCCCAGGCCGCGTGGGAAGAGTTCTTGCCGATGGTGTTTTCGA
CGCAAAGGCAAATGCACTCACATACGTTAGCCAACTTCCAACGATCAACTCAGTGTTCGTGCTTCGTTGAACGATTTTGCAGCTTTCTGCACCA
CCGCCGGTAGTAAAGGTTGCTCTTTTGCCACCGCCCCTACTGGAACCTCAGGTATTGTTGCTACCAGACTGGACAACATAATGAAGGATATGTT
CCTCAATCCTATTGTTGCTTCGGGCTTGAGCATCA

>Bcin15g03910(MLST6), partial sequence [organism=Botrytis fabae, strain D12_B_B28]
GTCAAAACACAAAATCATCCAACGATGAAGATGATACTCCACTTCCCTTGATTATCTGGCATGGACTCGGCGATAATTACAAAGCGGATGGTCT
TGCGCAAGTTGGAAAACTAGCTGAAGCTATTCATCCTGGGACTTTTGTCTATAATATTCATGTAGACGAGGATGCATCTGCAGATAGGACAGCT
ACCTTCTTTGGAAATCTCACTCGTGAGTACATCCCCTATTTTTCCTTTAAATACCATACTAACTCTCTTACCAAGTTCAAATCGAAAAGGTCTG
CGAAGACCTCGCCTCCCATCCTATTCTCTCTACCGCGCCCGCCGTCGACGCAATTGGATTCTCCCAAGGCGGCCAATTCTTGCGCGGTTACATA
TCCCGCTGCAATGCTCCACCCATCCGCTCTCTCCTGACCTTCGGTTCCCAACACAACGGCATTTCTGCCTTCCAAGCCTGTGGTCCTGCCGATT
TCCTCTGTCGCGGTGCTCAAACCCTTTTGCGATCCAACACCTGGTCAACCTTTGTCCAATCTCGTCTCGTACCCGCTCAATACTTCAGAGATCC
GGAAAACCTAGACTCTTACCTTGAATATTCCAATTTCCTTGCCGACATCAATAATGAGCGCGTTCTCAAGAACCAAACATATAAATCCAACATG
GAAAAATTGGAACGATTCGTAATGTATGTCTTTGAAGACGATACAACTGTCATCCCTAAGGAAAGTGGATGGTGGGCTGAAGTCAACGGCACGG
AAGTTACACCACTGAAAGAAAGAGCCATTTATAAAGAAGATTGGCTAGGTTTAAAGACATTGGATGAGGCCGGAAAATTAGTTTTCGAAACCAT
TCCAGGGGGACATATGACGTTAGGAGAGGAGATGCTAGAGAAGACTTTCAAGGAGTATTTTGGTCCAGCAGGGAAGAAATTTGGGG

>Bcin16g03460(MLST7), partial sequence [organism=Botrytis fabae, strain D12_B_B28]
ATGAACCTCTTAATTTGAACTCTTCATTAATTGCAAGTGATGAACCCTTATTTTGCCCAGAGGATAGTTACAAGGCGTATATCATTAGTCGAGA
ACCACTCATGATATACATTGACGGATTTTTGAAAGCGAATGAAAGTAAACATTTGGTTGATGTTAGGTGTGTTAATCATTCTGATGAAATGAAC
AAGAGAGACTGATGAGATAGTGAACCGCTTTATGAACCGTCTACTGTTTCTCACGGGCAGGAAGTTACCATTGATACTTCAGTTCGAAATTCTG
AAGTGGCGGTTTTAGAGAGGGATGAGGTGGTCAGGTGTATTGAGCATAGAGCGAGGGCATTTCAGGGGTGGAGGGGCGAGATGGGGATTGAGAA
GTTGAGGACGCAGAGGTATGGGGTTGGAGGACATTATGGGATGCATTTGTAAGTTTTGGGGGATTGACGAAGGCCTCTGTCTATTTTCTATCAG
TACGGACCTTGAAAGGAAGATAGCATGAACACGCGGGCTAACAACTAGGAAATAGCGATTGGAGCGGAGGTAAACGTGGCATAGACCGATTTAG
TACTTTCATGGTCTATGTCGACGTATCCTCTGATATCGAAGGTGGAGGAACGGAATTCCCACGTATTGTGGGACCAAAAGGAGGAAGGTGGGAG
GACTTCCTGGAAACTACGGAAGCATTGGATCCAGGAACTGGACAAAATGTAACAGTAGAAGGGGTGACATTCAAACCAATCAAGGGAAATGCCG
TATTCTGGGAAAATACTGACAGCAACGGGAGGGGCTACGAT

```
>Bcin12g03020(MLST8), partial sequence [organism=Botrytis fabae, strain D12_B_B28]
TCGATTGGCTGCGAAGAAAACTGCGCAGCACCGAGTCACCAAAAGCACGAATAAACCATCGGCAAGAGAAATATTCTCTGAACCACAACATGAC
ACGAGCGCGGAAGAGTATATCGGGCGAGAAGCTTCATCAAGAGCACCAAAGCGACAACGAGTCGATGATAATTATAACTCTTACGGTGGAAGAA
ATGAAAATACAGCAGCTTATGCTTCCGGGAAACTTCCATCTGGAAGTATAAATGTTGGTGGAGGTAGGAAGACCACTTTTCAAGAAGAACCACG
AACGGCATTTGTCGCTGGTAAGTTGCCCCCTGGAAGTATCAACATTGGTGGAAAGAAGGCTACCCAAATCGAGGACGAGGGTAGAGCAGCTTAT
GCTTCCGGAAAATTGCCTCCAGGAAGTATAAACTCTGGCGCAAAGAAGGCGATTTCATTCCAAGATGAAACGAGACCGGCTTATGTCTCCGGAA
AGCTTCCACATGGTAGTATCGACAGTATGCGAAACCGTGAAATGGCTGCCGTCCACCGCGAAATTGCTGAGGGTGGGAGGAAACCAGGCCAGGT
TGTCTCTTCTCTATTCACATTCAATCCTACTTCAAAGAAAACCTTCGATGAACCCGAAGAACAAGCGGAACCGGCAAAACCATCCAATGCGCCT
TTGACAGAGGAAATGGCGACTTTCACCAATCTAGGGTTATCGAGAAGGCTTGCAGCCCATCTATCGACTAAACTCGATATGAAAGCCCCGACAG
CCATTCAAAAAGCATCTGTACAGCAGTTGGTATCGGACGATAGCGATGCTTTCATACAAGCAGAGACTGGATCTGGAAAAACTTTGGCATATCT
ACTACCTATAGTCGAGCGAATATTAGCATTGAGTGAGAATGGCGTACAAA

>Bcin02g07770(MLST9), partial sequence [organism=Botrytis fabae, strain D12_B_B28]
CAGCTTTCCCTTTCGGTCTTGGCATCTACAGTCATTGCCATCCCTACACCATCACAACTTGAGTCTCGGGCCGTTATCGATTCCGATGCCGTTG
TAGGATTTGCCGAAACTGTTCCCAGTGGGACCGTAGGAACAGTTTATGAGGCATATAAACCATTCCTTAAAGTCGTAAATGGATGCGTACCATT
CCCTGCCGTCGATGCATCGGGTAACACAGGGTATGTCCTTATACTTTTCTCTTCCACACGATTGCTATTGAGTCTCTAACATATTTTAGTGGTG
GTTTGTCACCAACTGGCAGTAGCAATGGTGGTTGCAGCAGCAGTACCGGTCAAGTATACGTTCGAGGAGGACAAAGCGGATCAAACTACGCTAT
CATGTACTCCTGGTAAGTTCTCTCTAAACTTCTCCTTATAGATTCAACCTAACAAAATCTTAGGTACATGCCAAAGGACGAGCCCTCAACCGGT
ATTGGTCACCGTCACGATTGGGAAGGTGTAATTGTCTGGCTCTCAAGCGCCACCGCCACAACTGCCGACAACATCTTAGCCGTTTGTCCTTCCG
CCCACGGAGGCTGGGATTGTTCCACCGATGGCTATTCCCTTTCTGGTACCAGCCCTCTTATCAAGTACGAAAGTATCTGGCCCGTCGATCATTC
AATGGGTCTTACTAGTACTGTTGGTGGAAAACAACCTTTGATTGCTTGGGAGTCTTTACCAACCGCTGCTCAAACTGCTCTTGAGAACACCGAT
TTCGGTGCTGCGAATGTTCCATTCATTCCGGCTGTTTTCACAGATAATCT

>Bcin04g02090(MLST10), partial sequence [organism=Botrytis fabae, strain D12_B_B28]
CGGAGGATGATATGGCAAAGTCTATGATTACCAAAGCATTTGTAGGCATGAGTAGTAAACTGTCGAATATGACTATGAATGATGTTTACAAGCC
CTACATCCATGTAAGAAATGTAGAATAGAGAGATCAGTAACTGGAACTAATATCGTTTGCAGGCTTTCAAGTTACTTACGCAGTTCAACCCAAT
CACTACAGCTATTGCCGAATCCCCACTATTTCAAATGGCTGTCTCAGCAAATACCATCGAAAAGTACACACTGCTAGGCCCTTTCTTCAGAATA
TCTCCTCTGCAACAGGAAGTTACCAGGGAATACTTCAGTGCGCCAAAGACGATAGATAGGCGACACATTGCCACATCTCAAGATGCGTTACGAT
TGACCTTACAAACCCATCAAAAAGATTTACTTGATATCATCAACCACTTTGTTCGAGCAAGTCCAATCGCAAAAAGCAAAACCCTGGATTGGTT
CGCCTACATTGTGAATCAAAATCACAAACGTCGAGCACTTCAGGTAGACCCGAAAGAAGTGTCTTCTGATGGCTTTATGCACAATGTCACTGTC
GTTCTAGATGGTCTTTGTGAGCCATTCATGGATACCACATTCTCGAAAATTTCGAAGATTGATATTGATTATCTAAGACGTGCGCCTCGTGTAG
ATATCAAGGACGAGACCAAGTTGAACGCTGACGAGAAGGCTTCTGAGAAGTATTATGAGGACACTGTTCCTGGCACTTCTAATTTCATCTCTGA
GGTCTTCTTTCTCACATTGGCTGCTCATCATTATGGTAGTGAAGCTCTTAATGCCACGCATAAGAGTCTGGAGAAAGACATCAAATATATTCAA
AAGCAATTGACTGCCGTTGAAGCA

>Bcin01g07220(MLST1), partial sequence [organism=Botrytis fabae, strain G12_B03B]
GTGGCATATCTTGTTTCTTCTCGCATTATGCCTTGGAACGTGCTGGACAAGCTTGGGTTAGTGCTCTGCGTGTAGAAGCACTAAAGAAGATTCT
CGCACAACCGAAGTCATGGTTTGAGGAATCCAGGAATTCACCTGGCCGGTTGAACGAAGTTTTGGATAGGAACTCTGAGGAAATGCGTAATCTC
GTTGGCCGCTTTGCTGGTATTGTATTCACAGCATTTTTTATGCTATTGATATCGATCATTTGGGCTTTCGTGAATACATGGAAACTGACATTAG
TCTCAATGGCAACTGGGCCAGTTATATACGCTGTCACTAAAACGTTCAATCGCGTGAGTGGAAAATGGGAAAACAAGTGCAACTACGCATCTGA
AATGACCACTGGCATATTTTCAGAGACTTTCTCCAACATCAAAGTGGTTCGGGCTTTTACTCTGGAAACTTACTTTGAGACAAAACACACCAAA
GCTACAGAAGAACTTTATAAAGTTGGACTAATACGAGCAAACTACTCAGGATTGCTGTGGGGATTGACAGATGCGATGTCATTCTTCATCACTG
CAACTATCTTTTATTATGCCACGGTTCTCATTACCAAGAGAGAGATCAGTATCGCGACTGCACTACAGACTGTCAATCTTCTATTATTTGGTAT
TTCTAATAGTACGAATATGCTGGCCATGATACCACAAATCAACTCTTCTCGCGTTACAGCTACGCATATGCTTGCATTAGCCAATCTCGATTCA
TCTTCCTCCCACGAAAATAAAGGAACCGAACGGCTTTCGACAATCTTTCCAATCAAATTCAACCGTCTCTCATTCACATATCCTACACGTCCTG
AAAAACGAACGATATCATCCTTTTCTCTTTCCCTGATTCCTAACTCAACAACTGCACTTGTCGGACCCTCCGGCTCCGGAAAATCTACAATAGC
TGCTCTGCTCATTGGTCTCTATCCGCCAGATACTTCAACACCTCCACCGTTGACATTCAATCGCGTCTCCATAAGTAACTGTCACATTCCGTCT
CTCCGGGCTTCTATCTCACTCGTCCCACAAATACCGATTCTATTTCCAGCTACCATTCTCCATAACATCATTTATGGTCTCCCAGAATCTTCTC
CTTGTGCTAGTCTTCCATCTGCTATTGCATCAGCAAAAGATGCTGGGATCCATGAATTTATCACATCGCTTCCACAAGGTTATGATACTAT

>Bcin05g07690(MLST2), partial sequence [organism=Botrytis fabae, strain G12_B03B]
ACCACTATCACCAAGTCCTTTTCTGCACTTTCCTCATCCAAAAAGAACGAGAACATCATCAACAAATACTTTGCTGAAGTAATTGGGTTTTATT
TTGGCCAGGATGCCTTCTCTCTACGACAGCAAGCCTTCGATGATATGTTGTGGGTAGTTCTTGGTTGGCTGGATACTGTCAAGTTCATTGATTT
ACATTCTGAATTGCACTATTCAAACGACTCTCAGCCAGAATGGTACGGACAACAATATAAACCTGCATTTGCACATCGAGCGCGACTATTTTGG
GAATTGGCTTCACAAGGATGGGATACTACTCTCTGTGGTGGTGGGATGATATGGTCACCATACCTTACTCCATACAAGAACGCAATTACCAATG
AACTCTATATCGCAGCTTCGATATCGATGTACCTCTATTTCCCCGGAGATGGCAATCAATCCCCATTTATACTTTCCAACTCTTCATATCCACC
TCACGATCCGAAATATCTACAGGCAGCTGTTGACGCTTACAAATGGCTGAATGGTTCCAACATGACGGATTTACAAGGATTATATGTCGACGGA
```

TATCATATCTCGAATCTTTCTGGCGGTGAAAACACCCATTGCGATTCTAGAAATGAGATGGTATATACCTACAATCAGGGTGTTTTGCTTACTG
GACAACGTGGTCTGTATGACGCAACCGCCGCACGATCATACCTTGTGGATGGCCACAAACTCATCGCGAATGTTATTAATGCCACAGGCTATGA
CCTGAAACACAATGTTGTCATCTCACCGCCACCCAAAGATGGTTCCGCATTGGCAAAGTGGTTTGGCCTGGGTAGGAATGGAATACTGGAAGAA
GGATGCGATTCAAGTGCTTCGTGTTCTCAAAATGGACAAACTTTCAAAGGCATATTCTTTCATCACTTGATTGCATTCTGTAGTGATTTGCCAG
GGGAGCCTATTGCAGGGACGAAAGAAGGCCTAGAACTCGACAGAGTGTGGCATTCTGACAAATGCTCACAGTATACAAAAT

>Bcin06g01710(MLST3), partial sequence [organism=Botrytis fabae, strain G12_B03B]
GGTGAGTCTGACTTTTGTATTTGAGCGTTAAGATAGACACTGATATACCAAGGCAATCACCTAATTTCATGGCGTTCTTCCCTGCATCATCTAC
CTACCCTGGAATGCTGGGAGAATTATACTCAGCAGCTTTCACAGCACCTGCTTTCAATTGGATCTGTTCCCCTGCTGTGACAGAATTGGAGACG
GTTGTAATGGATTGGCTGGCCAAGCTTCTCAATCTCCCAGACTGTTATCTGTCTTCGACTCATGGTGGTGGTGTTATCCAAGGATCAGCCTCGG
AAGCTATCGTTACCGTTATGGTTGCTGCCCGCGATAAATATCTTCGTGAAACCACTGAAGGTCTGTCGGGCATTGAACTCGAGGATGCGATTGC
ATATAAGAGGAGTAAGCTAGTTGCACTAGGAAGCGAAATGGCACACAGCTCCACGCAGAAAGCAGCGCAGACAGCTGGCGTTAGATTCCGATCG
ATTCCAGTACCCGCATCCAATGATTTTGCCATGACGGGTGATGATTTAGAGAAGGTATTGAAAGAATGCAAATCTCAAGGATTGGAACCATTCT
ATCTAACTTCGACTTTGGGAACAACATCTACATGCGCAGTTGACGACTTCGCATCTATTGCAACAGTACTTTCAAAATATGCACCTCCAGATGT
TGCAGGCGAGATCTGGGTTCACGTCGATGCTGCTTATGCAGGTGCAGCTTTGGTTTGCCCTGAATACCATCATCTAACATCGTCCTTCCAGCAT
TTTCATTCCTTCGATATGAACATGCACAAATGGCTTCTGACAAATTTCGACGCTTCTTGTCTATATGTCAAGAAACGCAAAGATCTGGTCGATG
CACTCTCCATAACACCAAGCTATCTTCGCAACGAGTTTTCAGAGAGTGGACTCGTAACCGACTATCGGGACTGGCAAATTCCTCTCGGAAGACG
CTTCCGAAGCTTAAAGATTTGGTTTGTCCTCAGAACCTAC

>Bcin09g03030(MLST4), partial sequence [organism=Botrytis fabae, strain G12_B03B]
ACCCTTCAACCCCACCGCAGCTCCTATACCGAAAGCGAGCATCCTGCCAATTCTCCTTCCACCTGCGACTTTAAGACCATTGGCTTTTCGCACT
TTCACAAAAAAGCATAGCTTAACATTGACGTCGTCGGCATTACAAGTGTTGGCTACTTTTATTGGAAAGCATTGTGGGACAGGATGGAGGGAAG
AAGGACTGGCAGAGAGAGTCTTGGAGGAGGTCGCCAAGAGTTGGAAGAATAGGAGTGGCGGTGTTATTGTCGAGGGCGAGGGAACGGAATTGAA
GGAGATTCTGAAAGCTTTGGAAGGGAATATGAGTGGTGGAAGGATAGTCGTAGGAAGAGAGCTAAGCCGGCAGAATAGTTTAGTACTGGGATCA
TCACAATATGGAGAGGTCAATCATACAAGACTTGGGCTACGGCCAGGGAATATACCCAGAGAGGATAGTCAGTCAAGTTTGGGAATGTCAACGT
TGGAGGTCAATGACGAGGAAGATGAGGATGGCCTGATGGATCCAAGAAGGTGGATAAAAGTCATTGATGCATTTGAGCAACCTCGACTGGTGTA
TAATGTTGCTAAAAAGCACTTTGATAGGTATGTTTCAATGATAAAATTTTGTCTGAATCGACTAACTCAGTACAGAGATACCTCCAAACCTTCA
TTGTTCCCACCTGCGTCTCATAAAACACTCCTCTTCCAAAACCGCTATAATGTTATCCATCAACGTCTCCTTCGCAATGAATCTTTTCAAACGC
CCGCTTTTCAAGGTGGCAAAGCTTCCCTTCAACGCAGCACGTCCGCCATTACCACCCAACAACAATCATACAAATTAACGCCGATAGCTAATCT
TCTCGGTCGCAATCGCAGCTCTCATATGCTTCTCGGTCTCCTCAGTATTTCGCCCACTGGTACCCTCGCCATCAATGACCTGACGGGCAGCATC
GCTCTCGATCTTACACACGCAGCAGCCATTC

>Bcin11g01310(MLST5), partial sequence [organism=Botrytis fabae, strain G12_B03B]
ACTGACATGGACCTCATGTGGAACCGGCGTAGAATGCGCAACGCTTGAAGTTCCGCTCGAATATGGCGATACAACGTCAACGGCAAAAGCCAGT
GTTGCGCTTGCTCGTTATCCTGCCACTGTTGCCGCGGCAAGAAGCTCGGGTCTCTATTGATAAATCCCGGTGGACCCGGTGCCACTGGTGTTGG
CTTTTTGCAGTCTGGAGCCGGTGCTGCCGTCTCGACACTGAGTGGTGGATTATACGATATCATCGGACGGGATCCACGTGGAACCGGTGCTTCG
GCTCCTATTTTGGAATGTTTTGCAAATGCCAGTGCGGAGTATGATTTTAACAACGCGTTTCCATCTGCTCCGATCTCTGGCTCGGACAATTTCA
AATGCCAGCGCAAATTCTGCTGTTAGCTCTGCTATCACATCCTTTGACACTTCTGTCGCTGCTCTTGCAAAAGCTTGCGTGGCTCAGAAATCTC
CCGCTCTTTACACCTCAACAGCAGCATATGTTGCTCGAGACATGGCAGCGATAGTCGATGCATTGGATGGGACCTCTGCAAAACTTAACTACTG
GGGTTTCTCATATGGAACTATCTTCCTAGCTGAGTTTATCCAAACTTTCCCAGGCCGCGTGGGAAGAGTTCTTGCCGATGGTGTTTTCGACGCA
AAGGCAAATGCACTCACATACGTTAGCCAACTTCCCAACGATCAACTCAGTGTTCGTGCTTCGTTGAACGATTTTGCAGCTTTCTGCACCACCG
CCGGTAGTAAAGGTTGCTCTTTTGCCACCGCCCCTACTGGAACCTCAGGTATTGTTGCTACCAGACTGGACAACATAATGAAGGATATGTTCCT
CAATCCTATTGTTGCTTCGGGCTTGAGCATCA

>Bcin15g03910(MLST6), partial sequence [organism=Botrytis fabae, strain G12_B03B]
GTCAAAACACAAAATCATCCAACGATGAAGATGATACTCCACTTCCCTTGATTATCTGGCATGGACTCGGCGATAATTACAAAGCGGATGGTCT
TGCGCAAGTTGGAAAACTAGCTGAAGCTATTCATCCTGGGACTTTTGTCTATAATATTCATGTAGACGAGGATGCATCTGCAGATAGGACAGCT
ACCTTCTTTGGAAATCTCACTCGTGAGTACATCCCCTATTTTTCCTTTAAATACCATACTAACTCTCTTACCAAGTTCAAATCGAAAAGGTCTG
CGAAGACCTCGCCTCCCATCCTATTCTCTCTACCGCGCCCGCCGTCGACGCAATTGGATTCTCCCAAGGCGGCCAATTCTTGCGCGGTTACATA
TCCCGCTGCAATGCTCCACCCATCCGCTCTCTCCTGACCTTCGGTTCCCAACACAACGGCATTTCTGCCTTCCAAGCCTGTGGTCCTGCCGATT
TCCTCTGTCGCGGTGCTCAAACCCTTTTGCGATCCAACACCTGGTCAACCTTTGTCCAATCTCGTCTCGTACCCGCTCAATACTTCAGAGATCC
GGAAAACCTAGACTCTTACCTTGAATATTCCAATTTCCTTGCCGACATCAATAATGAGCGCGTTCTCAAGAACCAAACATATAAATCCAACATG
GAAAAATTGGAACGATTCGTAATGTATGTCTTTGAAGACGATACAACTGTCATCCCTAAGGAAAGTGGATGGTGGGCTGAAGTCAACGGCACGG
AAGTTACACCACTGAAAGAAAGAGCCATTTATAAAGAAGATTGGCTAGGTTTAAAGACATTGGATGAGGCCGGAAAATTAGTTTTCGAAACCAT
TCCAGGGGGACATATGACGTTAGGAGAGGAGATGCTAGAGAAGACTTTCAGGGAGTATTTTGGTCCAGCAGGGAAGAAATTTGGG

>Bcin16g03460(MLST7), partial sequence [organism=Botrytis fabae, strain G12_B03B]

```
ATGAACCTCTTAATTTGAACTCTTCATTAATTGCAAGTGATGAACCCTTATTTTGCCCAGAGGATAGTTACAAGACGTATATCATTAGTCGAGA
ACCACTCATGATATATATTGACGGATTTTTGAAAGCGAATGAAAGTAACATTTGGTTGATGTTAGGTGTGTTAATCATTCTGATGAAATGAACA
AGAGAGACTGATGAGATAGTGAACCGCTTTATGAACCGTCTACTGTTTCTCACGGGCAGGAAGTTACCATTGATACTTCAGTTCGAAATTCTGA
AGTGGCGGTTTTAGAGAGGGATGAGGTGGTCAGGTGTATTGAGCATAGAGCGAGGGCATTTCAGGGGTGGAGGGGCGAGATGGGGATTGAGAAG
TTGAGGACGCAGAGGTATGGGTTGGAGGACATTATGGGATGCATTTGTAAGTTTTGGGGGATTGACGAAGGCTTCTGTCTATTTTCTATCAGTA
CGGACCTTGAAAGAAGGATAGCATGAACACGCGGGCTAACAACTAGGAAATAGCGATTGGAGCGGAGGTAAACGTGGCATAGACCGATTTAGTA
CTTTCATGGTCTATGTCGACGTATCCTCTGATATCGAAGGTGGAGGAACGGAATTCCCACGTATTGTGGGACCAAAAGGAGGAAGGTGGGAGGA
CTTCCTGGAAACTACGGAAGCATTGGATCCAAGAACTGGACAAAATGTAACAGTAGAAGGGGTGACATTCAAACCAATCAAGGGAAATGCCGTA
TTCTGGGAAAATACTGACAGCAACGGAAGGGGCTACGAT

>Bcin12g03020(MLST8), partial sequence [organism=Botrytis fabae, strain G12_B03B]
CGATTGGCTGCGAAGAAAACTGCGCAGCACCGAGTCACCAAAAGCACGAATAAACCATCGGCAAGAGAAATATTCTCTGAACCACAACATGACA
CGAGCGCGGAAGAGTATATCGGGCGAGAAGCTTCATCAAGAGCACCAAAGCGACAACGAGTCGATGATAATTATAACTCTTACGGTGGAAGAAA
TGAAAATACAGCAGCTTATGCTTCCGGGAAACTTCCATCTGGAAGTATAAATGTTGGTGGAGGTAGGAAGACCACTTTTCAAGAAGAACCACGA
ACGGCATTTGTCGCTGGTAAGTTGCCCCCTGGAAGTATCAACATTGGTGGAAAGAAGGCTACCCAAATCGAGGACGAGGGTAGAGCAGCTTATG
CTTCCGGAAAATTGCCTCCAGGAAGTATAAACTCTGGCGCAAAGAAGGCGATTTCATTCCAAGATGAAACGAGACCGGCTTATGTCTCCGGAAA
GCTTCCACATGGTAGTATCGACAGTATGCGAAACCGTGAAATGGCTGCCGTCCACCGCGAAATTGCTGAGGGTGGGAGGAAACCAGGCCAGGTT
GTCTCTTCTCTATTCACATTCAATCCTACTTCAAAGAAAACCTTCGATGAACCCGAAGAACAAGCGGAACCGGCAAAACCATCCAATGCGCCTT
TGACAGAGGAAATGGCGACTTTCACCAATCTAGGGTTATCGAGAAGGCTTGCAGCCCATCTATCGACTAAACTCGATATGAAAGCCCCGACAGC
CATTCAAAAAGCATCTGTACAGCAGTTGGTATCGGACGATAGCGATGCTTTCATACAAGCAGAGACTGGATCTGGAAAAACTTTGGCATATCTA
CTACCTATAGTCGAGCGAATATTAGCATTGAGTGAGAATGGCGTACAAA

>Bcin02g07770(MLST9), partial sequence  [organism=Botrytis fabae, strain G12_B03B]
CAGCTTTCCCTTTCGGTCTTGGCATCTACAGTCATTGCCATCCCTACACCATCACAACTTGAGTCTCGGGCCGTTATCGATTCCGATGCCGTTG
TAGGATTTGCCGAAACTGTTCCCAGTGGGACCGTAGGAACAGTTTATGAGGCATATAAACCATTCCTTAAAGTCGTAAATGGATGCGTACCATT
CCCTGCCGTCGATGCATCGGGTAACACAGGGTATGTCCTTATACTTTTCTCTTCCACACGATTGCTATTGAGTCTCTAACATATTTTAGTGGTG
GTTTGTCACCAACTGGCAGTAGCAATGGTGGTTGCAGCAGCAGTACCGGTCAAGTATACGTTCGAGGAGGACAAAGCGGATCAAACTACGCTAT
CATGTACTCCTGGTAAGTTCTCTCTAAACTTCTCCTTATAGATTCAACCTAACAAAATCTTAGGTACATGCCAAAGGACGAGCCCTCAACCGGT
ATTGGTCACCGTCACGATTGGGAAGGTGTAATTGTCTGGCTCTCAAGCGCCACCGCCACAACTGCCGACAACATCTTAGCCGTTTGTCCTTCCG
CCCACGGAGGCTGGGATTGTTCCACCGATGGCTATTCCCTTTCTGGTACCAGCCCTCTTATCAAGTACGAAAGTATCTGGCCCGTCGATCATTC
AATGGGTCTTACTAGTACTGTTGGTGGAAAACAACCTTTGATTGCTTGGGAGTCTTTACCAACCGCTGCTCAAACTGCTCTTGAGAACACCGAT
TTCGGTGCTGCGAATGTTCCATTCATTCCGGCTGTTTTCACAGATAATCTT

>Bcin04g02090(MLST10), partial sequence [organism=Botrytis fabae, strain G12_B03B]
GCGGAGGATGATATGGCAAAGTCTATGATTACCAAAGCATTTGTAGGCATGAGTAGTAAACTGTCGAATATGACTATGAATGATGTTTACAAGC
CCTACATCCATGTAAGAAATGTAGAATAGAGAGATCAGTAACTGGAACTAATATCGTTTGCAGGCTTTCAAGTTACTTACGCAGTTCAACCCAA
TCACTACAGCTATTGCCGAATCCCCACTATTTCAAATGGCTGTCTCAGCAAATACCATCGAAAAGTACACACTGCTAGGCCCTTTCTTCAGAAT
ATCTCCTCTGCAACAGGAAGTTACCAGGGAATACTTCAGTGCGCCAAAGACGATAGATAGGCGACACATTGCCACATCTCAAGATGCGTTACGA
TTGACCTTACAAACCCATCAAAAAGATTTACTTGATATCATCAACCACTTTGTTCGAGCAAGTCCAATCGCAAAAAGCAAAACCCTGGATTGGT
TCGCCTACATTGTGAATCAAAATCACAAACGTCGAGCACTTCAGGTAGACCCGAAAGAAGTGTCTTCTGATGGCTTTATGCACAATGTCACTGT
CGTTCTAGATGGTCTTTGTGAGCCATTCATGGATACCACATTCTCGAAAATTTCGAAGATTGATATTGATTATCTAAGACGTGCGCCTCGTGTA
GATATCAAGGACGAGACCAAGTTGAACGCTGACGAGAAGGCTTCTGAGAAGTATTATGAGGACACTGTTCCTGGCACTTCTAATTTCATCTCTG
AGGTCTTCTTTCTCACATTGGCTGCTCATCATTATGGTAGTGAAGCTCTTAATGCCACGCATAAGAGTCTGGAGAAAGACATCAAATATATTCA
AAAGCAATTGACTGCCGTTGAAGCA

>Bcin05g07690(MLST2), partial sequence  [organism=Botrytis pseudocinerea, strain D08_H_8_15]
ACCACTATCACCAAGTCCTTTTCTGCACTTTCCTCATCAAAAACGAACGAGAACATCATCAATAAATACTTTGCTGAAGTAATTGGGTTTTATT
TTGGCCAGGATGCCTTCTCTCTACGACAGCAAGCCTTCGATGATATGTTGTGGGTAGTTCTTGGTTGGTTGGATACTGTCAAATTCATTGATTT
ACATTCTGAATTGCACTATTCAAATGACTCTCAGCCAGAATGGTACGGACAACAATATAAACCTGCATTTGCACATCGAGCGCGACTATTTTGG
GAATTGGCGTCACAAGGATGGGATACTACTCTCTGTGGTGGTGGGATGATATGGTCACCATACCTTACTCCATACAAGAACGCAATTACCAATG
AACTCTATATCGCAGCTTCGATATCGATGTACCTCTATTTCCCTGGAGATGACAATCAATCCCCATTTATGCTTTCCAACCCTTCATATCCACC
TCGCGATCCGAAATATCTACAGGCAGCTGTTGATGCTTACAAATGGCTGAATGGTTCCAATATGACGGATTTACAAGGATTATATGTCGACGGA
TATCATATCTCGAATCTTTCTGGCGGTGAAAACACCCATTGCGATTCTAGAAATGAGATGGTATATACCTACAATCAAGGTGTTTTGCTTACTG
GACAACGTGGTTTGTATGACGCAACCGCCGCACGATCATACCTTGTGGATGGCCACAAACTCATCGCGAATGTTATTAACGCCACAGGCTATGA
TCTGAAACACGATGTTGTTATCTCACCGCCACCCAAAGATGGTTCCGCATTGGCAAAATGGTTTGGCCTGGGTAGGAATGGAATACTGGAAGAA
GGTGCGATTCAAGTGCTTCGTGTTCTCAAAATGGACAAACTTTCAAAGGCATATTCTTTCATCACTTGATTGCATTCTGTAGTGATTTGCCAGG
GAGCCTATTGCAGGGACGAAAGAAAGCCTGGAACTCGACAGAGAGTGGCATTCTGACAAATGCTCGCAGTATACAAAAT
```

>Bcin06g01710(MLST3), partial sequence [organism=Botrytis pseudocinerea, strain D08_H_8_15]
GTGAGTCCGGCTTTTGTATCTGAGCGTTAAGATAGACACTGATATACCAAGGCAATCACCTAATTTCATGGCTTTCTTCCCTGCATCATCTACC
TACCCTGGAATGCTTGGAGAATTATACTCCGCAGCTTTCACAGCTCCTGCTTTCAATTGGATCTGTTCCCCTGCTGTGACAGAATTGGAGACGG
TTGTAATGGATTGGCTGGCCAAGCTTCTCAATCTCCCAGACTGTTATTTGTCTTCGACTCATGGTGGTGGTGTTATCCAAGGATCAGCCTCGGA
AGCTATCGTTACCGTTATGGTTGCTGCCCGCGATAAATATCTTCGTGAAACTACTGAAGGTCTGTCAGGCATTGAACTCGAGGATGCGATTGCA
TATAAGAGGAGTAAGCTAGTTGCACTAGGAAGCGAAATGGCACACAGCTCCACGCAGAAAGCAGCGCAGATAGCTGGCGTTAGATTCCGATCGA
TTCCAGTACTCGCATCCAATGATTTCGCCATGACGGGTGATGATCTAGAGAAGGTATTGAAAGAATGCAAATCTCAAGGATTGGAACCCTTCTA
TCTAACTTCGACTTTGGGAACAACATCTACATGTGCAGTTGATGACTTCGCATCTATCGCAACAGTACTTTCGAAATATGCACCTCCAGATGTT
GCAGGCGAGATATGGGTTCACGTCGATGCTGCTTATGCAGGCGCAGCTTTGGTTTGCCCTGAATACCATCATCTAACATCGTCCTTCCAGCATT
TTCATTCCTTCGATATGAACATGCACAAATGGCTTTTGACAAATTTCGACGCTTCTTGTCTATATGTCAAGAAACGCAAAGATCTGATCGATGC
ACTCTCTATAACACCAAGTTATCTTCGCAACGAGTTTTCAGAGAGTGGACTCGTGACCGACTATCGGGACTGGCAAATTCCTCTCGGAAGACGC
TTCCGAAGCTTAAAGATTTGGTTTGTCCTCAGAACCTAC

>Bcin09g03030(MLST4), partial sequence [organism=Botrytis pseudocinerea, strain D08_H_8_15]
ACCCTTCAATCCCACCGCAGCTCCTATACCGAAAGCGAGCATCCTACCAATTCTCCTTCCACCTGCGACTTTAAGACCATTGGCTTTTCGCACT
TTCACAAAAAAGCATAGTTTAACATTGACGTCGTCCGCATTACAAGTGTTGGCTACTTTTATTGGAAAGCATTGTGGGACAGGATGGAGGGAAG
AAGGACTGGCAGAGAGAGTTTTAGAGGAGGTCGCCAAGAGTTGGAAGAATAGGAGTGGTGGTGTTATTGTCGAGGGCGAGGGAACGGAATTGAA
GGAGATTCTGAAAGCTTTGGAAGGGAATATGAGTGGTGGAAGGATAGTCGTAGGAAGAGAGCTAAGCCGGCAGAACAGTTTAGTACTGGATCAT
CACAATATGGAGAGGTCAATCATACAAGACTTGGGCTACGGCCAGGGAATATACCCAGAGAGGATAGTCAGTCAAGTTTGGGAATGTCAACGTT
GGAGGTCAATGACGAGGAAGATGAGGATGGCCTGATGGATCCAAGGAGGTGGTTGAAAGTCATTGATGCATTCGAGCAACCCCGACTGGTGTAT
AATGTTGCTAAGAAGCACTTTGATAGGTATGTTTCAATGATAAAATTTTATCTGAATCAACTAACTCAGTACAGAGATACCTCCAAACCTTCAT
TGTTCCCACCTGCGTCTCATAAAACACTCCTCTTCCAAAACCGCTATAATGATATCCATCAACGTCTCCTTCGCAATGAATCTTTTCAAACGCC
CGCTTTTCAAGGTGGCAAAGCTTCCCTTCAACGCAGCACGTCCGCCATTACCACTCAACAACAATCATACAAATTAACGCCGATAGCTAATCTT
CTCGGTCGCAATCGCAGCTCTCATATGCTTCTCGGCCTCCTCAGCATTTCACCCACTGGTACCCTCGCCATCAATGACCTGACGGGCAGCATCG
CTCTCGATCTTACACACGCAGCAGCCATTC

>Bcin11g01310(MLST5), partial sequence [organism=Botrytis pseudocinerea, strain D08_H_8_15]
ACTGACATGGACTTCATGTGGAACCGGCGTACAATGCGCAACGCTTGAAGTTCCGCTCGAATATGGCGATGCAACGTCAACAGCGAAAGCCAGT
ATTGCGCTTGCTCGTTATCCTGCCACTGTTGCCGCGAGCAAGAAGCTCGGGTCTCTCTTGATAAATCCCGGTGGACCCGGTGCCACTGGTGTTG
GCTTTGTGCAGTCTGGAGCCGGTGCCGCCATCTCGACACTGAGTGGTGGATTATACGATATCATCGGATGGGATCCACGTGGAACCGGTGCTTC
CGCTCCTATTTTGGAATGTTTTCCAAATGCCAGTGCGGAGTATGATTTTAACAACGCGTTTCCATCTGCTCCCAATCTCTGGCTCGGACAATTT
TCAAATGCCAGCGCAAATTCTGCCGTTAGCTCTGCTATCACATCTTTTGACACTTCTGTCGCTGCTCTTGCAAAAGCTTGTGTAGCTCAGAAGT
CCCCCGCTCTTTACACCTCAACAGCAGCATATGTTGCTCGAGACATGGCAGCGATAGTCGATGCCTTGGATGGAACCTCTGCAAAACTCAACTA
CTGGGGTTTCTCATATGGAACTATTTTCCTTGCCGAGTTTATCCAAACTTTCCCAGGCCGCGTGGGAAGAGTTCTTGCCGATGGGGTTTTCGAC
GCAAAGGCAAATGCACTCACATACGTTAGCCAACTTCCCAACGATCAACTCAGTGTTCGTGCTTCGTTGAACGATTTTGCAGCTTTCTGTACCA
CCGCCGGTAGTAAAGGTTGCTCTTTTGCCACCGCCCCTACTGGAACCACCGGTACCGTTGCTACCAGACTGGACAACATAATGAAGGATATGTT
CCTCAATCCCATTGTTGCTTCGGGCTTAAGCATCA

>Bcin15g03910(MLST6), partial sequence [organism=Botrytis pseudocinerea, strain D08_H_8_15]
GCCAAACACAAAATCATCCAACGATGAAGATGATACTCCACTGCCCTTAATTATCTGGCACGGACTCGGCGATAATTACAAAGCGGATGGTCTT
GCGCAAGTTGGAAAGCTAGCTGAAGCTATTCATCCTGGGACTTTTGTCTATAATATTCATGTAGATGAGGATGCATCTGCAGATAGGACAGCTA
CCTTCTTTGGAAATCTCACTCGTGGGTACATCCCCTAGTCTTCTTTTAAATGCCATGCTGACTCTCTTACCAAGTTCAAATCGAAAAGGTCTGC
GAAGACCTCGCCTCCCATCCTATTCTCTCTACCGCGCCTGCCGTCGACGCAATCGGATTCTCCCAAGGCGGCCAATTCTTGCGCGGTTACATAT
CCCGCTGCAATTCTCCACCCATCCGCTCTCTCCTGACCTTCGGTTCCCAACACAACGGCATTTCTGCCTTCCAAGCCTGTGGTCCTGCCGATTT
CCTCTGTCGTGGTGCTCAAACTCTTTTGCGATCCAACACCTGGTCAACCTTTGTCCAATCTCGTCTCGTACCCGCTCAATACTTCAGAGATCCG
GAAAACCTAGACTCTTACCTTGAATATTCCAATTTCCTTGCCGACATCAATAATGAGCGCGTTCTCAAGAACCAAACATATAAATCCAACATGG
AAAAATTGGAACGATTCGTAATGTATGTCTTTGAAGATGATACAACTGTCATCCCTAAGGAAAGTGGACGGTGGGCTGAAGTCAACGGCACGGA
AGTTACACCACTGAAGAAAGAGCCATTTATAAGGAAGATTGGCTAGGTTTAAAGACATTGGATGAGGCCGGAAAATTAGTTTTCGAAACCATTC
CAGGGGACATATGACGTTAGGAGAGGAGATGCTAGAGAAGGCTTTCAAAGAGTACTTTGGCCCAGCAGGGAAGAAATTTGGG

>Bcin16g03460(MLST7), partial sequence [organism=Botrytis pseudocinerea, strain D08_H_8_15]
ATGAACCTCTTAATTTGAACTCTTCATTAATCGCAAGTGATGAACCCCTATTTTGCCCAGAGGATAGTTACAAGACGTATATCATTAGTCGAGA
ACCACTCATGATATATATTGACGGATTTTTGAAAGCGAATGAAAGTAAACATCTGGTTGATGTTAGGTATGTTACTTATTCTGATGAAATGAAC
AAGAGAAACTGATGAGATAGTGAACCGCTTTATGAACCGTCTACTGTTTCTCACGGACAGGAAGTTACCATTGATACTTCAGTTCGAAATTCGG
AAGTGGCGGTTTTAGAGAGGGATGAGGTGGTCAGGTGTATTGAGCATAGAGCGAGGGCATTTCAGGGGTGGAGGGATGAGATGGGGATTGAGAA
GTTGAGGACGCAGAGGTATGGGGTTGGAGGACATTATGGGATGCATTTGTAAGTTTTGGGGGATTGACGAAGGCTTTTGTCTATTTCCTATCAG

TACGGATCTTAAAAGAAAGATAGCATGAACACGAGGGCTAACAACTAGGAAATAGCGATTGGAGCGGAGGTAAACGTGGCATAGACCGATTTAG
TACTTTCATGGTCTATGTCGACGTATCTTCTGATATCGAAGGTGGAGGAACGGAATTCCCACGTATTGTGGGACCACAAGGAGGAAGGTGGGAG
GAATTCCTGGAAACTACGGAAGCATTGGATCCAAGAACTGGAAAAAAATGTAACAGTAGAAGGGGTGACATTCAAACCAATCAAGGGAAATGCCG
TATTCTGGGAAAATACTGACAACAACGGGAGGGGATATGAT

>Bcin12g03020(MLST8), partial sequence [organism=Botrytis pseudocinerea, strain D08_H_8_15]
CGATTGGCTGCGAAGAAAACTGCGCAGCACCGAGTCACCAAAAGCACGAATAAACCGTCGGCAAGAGAAATATTCTCTGAACCACAACATGACA
CGAGCGCGGAAGAGTATATTGGGCGAGAAGCTTCATCAAGAGCACCAAAGCGACAACGAGTCGATGATAATTATAACTCTTACGGTGGAAGAAA
TGAAAATACAGCAGCTTATGCTTCCGGAAAACTTCCATCTGGAAGTATAAATGTTGGTGGAGGTAGGAAGACCACTTTTCAAGAAGAACCTCGA
ACGGCATTTGTCGCTGGCAAGTTGCCCCCTGGAAGTATCAACATCGGTGGAAAGAAGGCTACCCAAATCGAGGACGAAGGAAGAGCAGCTTATG
CTTCCGGGAAATTGCCCCCAGGAAGTATCAGCTCTGGCGCAAAGAAGGCCATTTCATTCCAAGATGAAACGAGACCGGCCTATGTCTCTGGAAA
GCTCCCACATGGCAGTATCGATGGTATGCGAAACCGCGAAATGGCTGCCGTCCACCGCGAAATTGCTGAGGGTGGGAGGAAACCAGGCCAGGTT
GTCTCTTCTCTATTCACATTCAATCCTACATCAAAGAAAACTTTCGATGAATCCGAAGAACAAGCGGAACCTGCAAAACCATCCAATGCGCCTT
TGACAGAGGAAATGGAGACATTCACCAATCTAGGATTATCGAGAAGGCTTGCAGCCCATCTATCGACTAAACTCGATATGAAAGCCCCGACCGC
CATTCAAAAAGCATCTGTGCAGCAGTTGGTATCGGACGATAGCGATGCTTTCATACAAGCAGAGACTGGATCTGGAAAAACTTTGGCATATCTA
CTACCTATAGTCGAGCGAATATTAGCATTGAGTGAGAATGGCGTACAAA

>Bcin02g07770(MLST9), partial sequence [organism=Botrytis pseudocinerea, strain D08_H_8_15]
CAGCTTTCCCTTTCGGTCTTGGCATCTACAGTCATTGCCATGCCTACACCATCACAACTTGAGTCTCGGGCCGTTATCGATTCCGATGCCGTTG
TAGGATTTGCCGAAGCTGTTCCCAGTGGGACCGTAGGAACAGTTTATGAGGCATATAAACCATTCCTTAAAGTCGTAAATGGATGCGTACCATT
CCCTGCCGTCGATGCATCGGGTAACACAGGGTATGTCCTTATACCTTTCTCTTCCTCACGATTGCTATTGAGTCTCTAACATATTTTAGTGGTG
GTTTGTCACCAACTGGCAGTAGCAATGGTGGTTGCAGCAGCAGTACCGGTCAAGTATATGTTCGAGGAGGACAAAGCGGATCAAACTACGCTAT
CATGTACTCCTGGTAAGTTCTCTCTAAACTTCTCCTTATAGATCCAATATCTAACAGAATCTTAGGTACATGCCAAAGGACGAGCCCTCAACCG
GTATTGGTCACCGTCACGATTGGGAAGGTGTAATCGTCTGGCTCTCCAGCGCCACCGCCACAACTGCCGACAACATCTTAGCTGTTTGTCCTTC
GGCCCACGGAGGCTGGGATTGTTCCACCGATGGTTATTCCCTTTCTGGTACCAGCCCTCTTATCAAGTACGAAAGTATCTGGCCCGTCGACCAT
TCAATGGGTCTTACTAGTACTGTTGGTGGACAACAACCTATGATTGCTTGGGAGTCTTTACCTACTGCTGCTCAAACTGCTCTTGAGAACACCG
ATTTCGGTGCTGCGAATGTTCCATTTATTCCAGCTGTTTTCACAAACAATCT

>Bcin04g02090(MLST10), partial sequence [organism=Botrytis pseudocinerea, strain D08_H_8_15]
CGGAGGATGATATGGCAAAGTCTATGATTACCAAAGCATTTGTAGGCATGAGTAGTAAACTCTCAAATATGACTATGAATGATGTTTACAAGCC
CTACATCCATGTAAGAAATGTAGAATAGAGAGATCAATAACTGGAACTAATATCGTTTGTAGGCTTTCAAGTTACTTACGCAGTTCAACCCAAT
CACTACAGCTATTGCCGAATCCCCACTATTTCAAATGGCTGTCTCAGCAAATACCATCGAAAAGTACACACTGCTAGGCCCTTTCTTCAGAATA
TCTCCTCTGCAACAGGAAGTTACCAGGGAATACTTCAGTGCACCAAAGACGATAGATAGGCGACACATTGCCACATCTCAAGATGCGTTACGAC
TGACCTTACAAACCCATCAAAAAGATTTACTCGATATCATCAACCACTTTGTTCGAGCAAGTCCAATCGCCAAAAGCAAAACCTTGGATTGGTT
CGCCTACATTGTGAATCAAAATCACAAGCGTCGAGCACTTCAGGTAGACCCGAAAGAAGTATCTTCTGATGGCTTTATGCACAATGTCACTGTC
GTTCTAGATGGTCTTTGTGAGCCATTCATGGATACCACATTCTCGAAAATTTCGAAGATTGATATTGATTATCTAAGACGTGCGCCTCGTGTAG
ATATCAAGGACGAGACCAAGTTGAACGCCGATGAGAAGGCTTCTGAGAAGTACTATGAGGACACGGTTCCTGGCACTTCCAATTTCATCTCTGA
GGTGTTCTTTCTGACATTGGCTGCTCATCATTATGGTAGTGAAGCTCTTAATGCCACGCACAAGAGTCTGGAGAAAGACATCAAATATATTCAA
AAGCAATTGACTGCCGTTGAAGCA
>Bcin01g07220(MLST1), partial sequence [organism=Botrytis pseudocinerea, strain D08_H_8_17]
ATGGCATATCTTGTTTCTTTTCGCATTATGCTTTGGAACGTGCTGGACAAGCTTGGGTTAGTGCCCTGCGTGTAGAAGCACTAAAGAAGATTCT
TGCACAACCGAAGTCGTGGTTTGAGGAATCCAGGAATTCACCTGGCCGATTGAACGAAGTTTTGGATAGGAACTCTGAGGAAATGCGTAATCTC
GTTGGCCGCTTTGTTGGTATTGTACTCACAGCATTTTTTATGTTATTGATATCAATCATTTGGGCTTTCGTGAATACGTGGAAGCTGACATTAG
TCTCAATGGCAACTGGGCCAGTTATATACGCTGTCACCAAAACGTTCAATCGCGTGAGTGGAAAATGGGAAAACAAGTGCAACTACGCATCTGA
AATGACCACTAGCATATTTTCAGAGACGTTCTCCAACATCAAAGTGGTTCGGGCTTTTACTCTGGAAACTTACTTTGCGACAAAACACACCAAA
GCTACAGAAGAACTTTATAAAGTTGGACTAATACGAGCAAACTACTCGGGATTGCTGTGGGGATTGACAGACGCGATGTCATTCTTCATCACTG
CAACTATCTTTTATTATGCCACGGTTCTCATTACCAAGAGAGAGATCAGTATCGCGACTGCACTACAGACTGTCAATCTTCTATTATTTGGTAT
TTCTAATAGTACGAATATGCTGGCCATGATACCACAAATCAACTCTTCTCGCGTTACAGCTACGCATATGCTTGCATTAGCCAATCTCGATTCA
TCTTCCTCCCACGAAAATCAAGGAACCGAACGACTTTCGACAATCTTTCCAATCAAATTCAACCGTCTCTCATTCACATATCCTAATCGTCCTG
AAAAACGAACGATATCATCCTTTTCCCTTTCCCTGATTCCTAACACAACAACTGCACTTGTCGGACCCTCCGGCTCCGGAAAATCTACAATAGC
TGCTCTGCTCATTGGCCTCTATCCACCAGATACTTCAACACCTCCACCGTTGACATTCAATCGCGTCCCCATAAGTAACTGTCACATCCCGTCT
CTCCGGGCTTCTGTCTCACTCGTCCCACAAATACCGATTCTATTTCCAGCTACCATTCTCCATAACATCATTTATGGGCTCCCAGAATCTTCTC
CTTGTGCTAATCTTCCATCTGCTATTGTATCAGCCAAAGATGCTGGGATCCATGAGTTTATCACATCGCTTCCACAAGGTTATAATGCTAT
>Bcin05g07690(MLST2), partial sequence [organism=Botrytis pseudocinerea, strain D08_H_8_17]
ACCACTATCACCAAGTCCTTTTCTGCACTTTCCTCATCAAAAACGAACGAGAACATCATCAATAAATACTTTGCTGAAGTAATTGGGTTTTATT
TTGGCCAGGATGCCTTCTCTCTACGACAGCAAGCCTTCGATGATATGTTGTGGGTAGTTCTTGGTTGGTTGGATACTGTCAAATTCATTGATTT
ACATTCTGAATTGCACTATTCAAATGACTCTCAGCCAGAATGGTACGGACAACAATATAAACCTGCATTTGCACATCGAGCGCGACTATTTTGG

GAATTGGCGTCACAAGGATGGGATACTACTCTCTGTGGTGGTGGGATGATATGGTCACCATACCTTACTCCATACAAGAACGCAATTACCAATG
AACTCTATATCGCAGCTTCGATATCGATGTACCTCTATTTCCCTGGAGATGACAATCAATCCCCATTTATGCTTTCCAACCCTTCATATCCACC
TCGCGATCCGAAATATCTACAGGCAGCTGTTGATGCTTACAAATGGCTGAATGGTTCCAATATGACGGATTTACAAGGATTATATGTCGACGGA
TATCATATCTCGAATCTTTCTGGCGGTGAAAACACCCATTGCGATTCTAGAAATGAGATGGTATATACCTACAATCAAGGTGTTTTGCTTACTG
GACAACGTGGTTTGTATGACGCAACCGCCGCACGATCATACCTTGTGGATGGCCACAAACTCATCGCGAATGTTATTAACGCCACAGGCTATGA
TCTGAAACACGATGTTGTTATCTCACCGCCACCCAAAGATGGTTCCGCATTGGCAAAATGGTTTGGCCTGGGTAGGAATGGAATACTGGAAGAA
GGATGCGATTCAAGTGCTTCGTGTTCTCAAAATGGACAAACTTTCAAAGGCATATTCTTTCATCACTTGATTGCATTCTGTAGTGATTTGCCAG
GGGAGCCTATTGCAGGGACGAAAGAAAGCCTGGAACTCGACAGAGAGTGGCATTCTGACAAATGCTCGCAGTATACAAAAT

>Bcin06g01710(MLST3), partial sequence [organism=Botrytis pseudocinerea, strain D08_H_8_17]
GTGAGTCCGGCTTTTGTATCTGAGCGTTAAGATAGACACTGATATACCAAGGCAATCACCTAATTTCATGGCTTTCTTCCCTGCATCATCTACC
TACCCTGGAATGCTTGGAGAATTATACTCCGCAGCTTTCACAGCTCCTGCTTTCAATTGGATCTGTTCCCCTGCTGTGACAGAATTGGAGACGG
TTGTAATGGATTGGCTGGCCAAGCTTCTCAATCTCCCAGACTGTTATTTGTCTTCGACTCATGGTGGTGGTGTTATCCAAGGATCAGCCTCGGA
AGCTATCGTTACCGTTATGGTTGCTGCCCGCGATAAATATCTTCGTGAAACTACTGAAGGTCTGTCAGGCATTGAACTCGAGGATGCGATTGCA
TATAAGAGGAGTAAGCTAGTTGCACTAGGAAGCGAAATGGCACACAGCTCCACGCAGAAAGCAGCGCAGATAGCTGGCGTTAGATTCCGATCGA
TTCCAGTACTCGCATCCAATGATTTCGCCATGACGGGTGATGATCTAGAGAAGGTATTGAAAGAATGCAAATCTCAAGGATTGGAACCCTTCTA
TCTAACTTCGACTTTGGGAACAACATCTACATGTGCAGTTGATGACTTCGCATCTATCGCAACAGTACTTTCGAAATATGCACCTCCAGATGTT
GCAGGCGGGATATGGGTTCACGTCGATGCTGCTTATGCAGGCGCAGCTTTGGTTTGCCCTGAATACCATCATCTAACATCGTCCTTCCAGCATT
TTCATTCCTTCGATATGAACATGCACAAATGGCTTTTGACAAATTTCGACGCTTCTTGTCTATATGTCAAGAAACGCAAAGATCTGATCGATGC
ACTCTCTATAACACCAAGTTATCTTCGCAACGAGTTTTCAGAGAGTGGACTCGTGACCGACTATCGGGACTGGCAAATTCCTCTCGGAAGACGC
TTCCGAAGCTTAAAGATTTGGTTTGTCCTCAGAACCTACGGA

>Bcin09g03030(MLST4), partial sequence [organism=Botrytis pseudocinerea, strain D08_H_8_17]
ACCCTTCAATCCCACCGCAGCTCCTATACCGAAAGCGAGCATCCTACCAATTCTCCTTCCACCTGCGACTTTAAGACCATTGGCTTTTCGCACT
TTCACAAAAAAGCATAGTTTAACATTGACGTCGTCCGCATTACAAGTGTTGGCTACTTTTATTGGAAAGCATTGTGGGACAGGATGGAGGGAAG
AAGGACTGGCAGAGAGAGAGTTTTAGAGGAGGTCGCCAAGAGTTGGAAGAATAGGAGTGGTGGTGTTATTGTCGAGGGCGAGGGAACGGAATTGAA
GGAGATTCTGAAAGCTTTGGAAGGGAATATGAGTGGTGGAAGGATAGTCGTAGGAAGAGAGCTAAGCCGGCAGAACAGTTTAGTACTGGGATCA
TCACAATATGGAGAGGTCAATCATACAAGACTTGGGCTACGGCCAGGGAATATACCCAGAGAGGATAGTCAGTCAAGTTTGGGAATGTCAACGT
TGGAGGTCAATGACGAGGAAGATGAGGATGGCCTGATGGATCCAAGGAGGTGGTTGAAAGTCATTGATGCATTTGAGCAACCCCGACTGGTGTA
TAATGTTGCTAAGAAGCACTTTGATAGGTATGTTTCAATGATAAAATTTTATCTGAATCAACTAACTCAGTACAGAGATACCTCCAAACCTTCA
TTGTTCCCACCTGCGTCTCATAAAACACTCCTCTTCCAAAACCGCTATAATGTTATCCATCAACGTCTCCTTCGCAATGAATCTTTTCAAACGC
CCGCTTTTCAAGGTGGCAAAGCTTCCCTTCAACGCAGCACGTCCGCCATTACCACTCAACAACAATCATACAAATTAACGCCGATAGCTAATCT
TCTCGGTCGCAATCGCAGCTCTCATATGCTTCTCGGCCTCCTCAGCATTTCACCCACTGGTACCCTCGCCATCAATGACCTGACGGGCAGCATC
GCTCTCGATCTTACACACGCAGCAGCCATTC

>Bcin11g01310(MLST5), partial sequence [organism=Botrytis pseudocinerea, strain D08_H_8_17]
ACTGACATGGACTTCATGTGGAACCGGCGTACAATGCGCAACGCTTGAAGTTCCGCTCGAATATGGCGATGCAACGTCAACAGCGAAAGCCAGT
ATTGCGCTTGCTCGTTATCCTGCCACTGTTGCCGCGAGCAAGAAGCTCGGGTCTCTCTTGATAAATCCCGGTGGACCCGGTGCCACTGGTGTTG
GCTTTGTGCAGTCTGGAGCCGGTGCCGCCATCTCGACACTGAGTGGTGGATTATACGATATCATCGGATGGGATCCACGTGGAACCGGTGCTTC
CGCTCCTATTTTGGAATGTTTTCCAAATGCCAGTGCGGAGTATGATTTTAACAACGCGTTTCCATCTGCTCCCAATCTCTGGCTCGGACAATTT
TCAAATGCCAGCGCAAATTCTGCCGTTAGCTCTGCTATCACATCTTTTGACACTTCTGTCGCTGCTCTTGCAAAAGCTTGTGTAGCTCAGAAGT
CCCCCGCTCTTTACACCTCAACAGCAGCATATGTTGCTCGAGACATGGCAGCGATAGTCGATGCCTTGGATGGACCTCTGCAAAACTCAACTAC
TGGGGTTTCTCATATGGAACTATTTTCCTTGCCGAGTTTATCCAAACTTTCCCAGGCCGCGTGGGAAGAGTTCTTGCCGATGGGGTTTTCGACG
CAAAGGCAAATGCACTCACATACGTTAGCCAACTTCCCAACGATCAACTCAGTGTTCGTGCTTCGTTGAACGATTTTGCAGCTTTCTGTACCAC
CGCCGGTAGTAAAGGTTGCTCTTTTGCCACCGCCCCTACTGGAACCACCGGTACCGTTGCTACCAGACTGGACAACATAATGAAGGATATGTTC
CTCAATCCCATTGTTGCTTCGGGCTTAAGCATCA

>Bcin15g03910(MLST6), partial sequence [organism=Botrytis pseudocinerea, strain D08_H_8_17]
GCCAAAACACAAAATCATCCAACGATGAAGATGATACTCCACTGCCCTTAATTATCTGGCACGGACTCGGCGATAATTACAAAGCGGATGGTCT
TGCGCAAGTTGGAAAGCTAGCTGAAGCTATTCATCCTGGGACTTTTGTCTATAATATTCATGTAGATGAGGATGCATCTGCAGATAGGACAGCT
ACCTTCTTTGGAAATCTCACTCGTGGGTACATCCCCTAGTCTTCTTTTAAATGCCATGCTGACTCTCTACCAAGTTCAAATCGAAAAGGTCTGC
GAAGACCTCGCCTCCCATCCTATTCTCTCTACCGCGCCTGCCGTCGACGCAATCGGATTCTCCCAAGGCGGCCAATTCTTGCGCGGTTACATAT
CCCGCTGCAATTCTCCACCCATCCGCTCTCTCCTGACCTTCGGTTCCCACACAACGGCATTTCTGCCTTCCAAGCCTGTGGTCCTGCCGATTTC
CTCTGTCGTGGTGCTCAAACTCTTTTGCGATCCAACACCTGGTCAACCTTTGTCCAATCTCGTCTCGTACCCGCTCAATACTTCAGAGATCCGG
AAAACCTAGACTCTTACCTTGAATATTCCAATTTCCTTGCCGACATCAATAATGAGCGCGTTCTCAAGAACCAAACATATAAATCCAACATGGA
AAAATTGGAACGATTCGTAATGTATGTCTTTGAAGATGATACAACTGTCATCCCTAAGAAAGTGGATGGTGGGCTGAAGTCAACGGCACGGAAG
TTACACCACTGAAAGAAAGAGCCATTTATAAGGAAGATTGGCTAGGTTTAAAGACATTGGATGAGGCCGGAAAATTAGTTTCGAAACCATTCCA

GGGGGACATATGACGTTAGGAGAGGAGATGCTAGAGAAGGCTTTCAAAGAGTACTTTGGCCCAGCAGGGAAGAAATTTGGG

>Bcin16g03460(MLST7), partial sequence [organism=Botrytis pseudocinerea, strain D08_H_8_17]
CATGAACCTCTTAATTTGAACTCTTCATTAATCGCAAGTGATGAACCCCTATTTGCCCAGAGGATAGTTACAAGACGTATATCATTAGTCGAGA
ACCACTCATGATATATATTGACGGATTTTTGAAAAGCGAATGAAAGTAAACATCTGGTTGATGTTAGGTATGTTACTTATTCTGATGAAATGAA
CAAGAGAAACTGATGAGATAGTGAACCGCTTTATGAACCGTCTACTGTTTCCTCACGGACAGGAAGTTACCATTGATACTTCAGTTCGAAATTC
GGAAGTGGCGGTTTTAGAGAGGGATGAAGGTGGTCAGGTGTATTGAGCATAGAGCGAGGGCATTTCAGGGGTGGAGGGATGAGATGGGGATTGA
GAAGTTGAGGACGCAGAGGTATGGGGTTGGAGGACATTATTGGGATGCATTTGTTAAGTTTTGGGGGATGACGAAGGCTTTTGTCTATTTCCTA
TCAGTACGGATCTTAAAAGAAAGATAGCATGAACACGAGGGCTAACAACTAGGAAATAGCGAATTGGAGCGGAGGTAAACGTGGCATAGACCGA
TTTAGTACTTTCATGGTCTATGTCGACGTATCTTTCTGATATCGAAGGTGGAGGAACGGAATTCCCACGTATTGTGGGGCCACAAGGAGGAAGG
TGGGAGGAATTCCTGGAAACTACGGAAGCATTGGATTCCAAGAACTGGAAAAAAATGTAACAGTAGAAGGGGTGACATTCAAACCAATCAAGGGA
AATGCCGTATTCTGGGAAAATACTGACAACAACGGGAGGGATATGAT

>Bcin12g03020(MLST8), partial sequence [organism=Botrytis pseudocinerea, strain D08_H_8_17]
CGATTGGCTGCGAAGAAAACTGCGCAGCACCGAGTCACCAAAAGCACGAATAAACCGTCGGCAAGAGAAATATTCTCTGAACCACAACATGACA
CGAGCGCGGAAGAGTATATTGGGCGAGAAGCTTCATCAAGAGCACCAAAGCGACAACGAGTCGATGATAATTATAACTCTTACGGTGGAAGAAA
TGAAAATACAGCAGCTTATGCTTCCGGAAAACTTCCATCTGGAAGTATAAATGTTGGTGGAGGTAGGAAGACCACTTTTCAAGAAGAACCTCGA
ACGGCATTTGTCGCTGGCAAGTTGCCCCCTGGAAGTATCAACATCGGTGGAAAGAAGGCTACCCAAATCGAGGACGAAGGAAGAGCAGCTTATG
CTTCCGGGAAATTGCCCCCAGGAAGTATCAGCTCTGGCGCAAAGAAGGCCATTTCATTCCAAGATGAAACGAGACCGGCCTATGTCTCTGGAAA
GCTCCCACATGGCAGTATCGATGGTATGCGAAACCGCGAAATGGCTGCCGTCCACCGCGAAATTGCTGAGGGTGGGAGGAAACCAGGCCAGGTT
GTCTCTTCTCTATTCACATTCAATCCTACATCAAAGAAAACTTTCGATGAATCCGAAGAACAAGCGGAACCTGCAAAACCATCCAATGCGCCTC
TGACAGAGGAAATGGAGACATTCACCAATCTAGGATTATCGAGAAGGCTTGCAGCCCATCTATCGACTAAACTCGATATGAAAGCCCGACCGCC
ATTCAAAAAGCATCTGTGCAGCAGTTGGTATCGGACGATAGCGATGCTTTCATACAAGCAGAGACTGGATCTGGAAAAACTTTGGCATATCTAC
TACCTATAGTCGAGCGAATATTAGCATTGAGTGAGAATGGCGTACAAA

>Bcin02g07770(MLST9), partial sequence [organism=Botrytis pseudocinerea, strain D08_H_8_17]
CAGCTTTCCCTTTCGGTCTTGGCATCTACAGTCATTGCCATGCCTACACCATCACAACTTGAGTCTCGGGCCGTTATCGATTCCGATGCCGTTG
TAGGATTTGCCGAAGCTGTTCCCAGTGGACCGTAGGAACGGTTTATGAGGCATATAAACCATTCCTTAAAGTCGTAAATGGATGCGTACCATTC
CTGCCGTCGATGCATCGGGTAACACAGGGTATGTCCTTATACCTTTCTCTTCCTCACGATTGCTATTGAGTCTCTAACATATTTTAGTGGTGGT
TTGTCACCAACTGGCAGTAGCAATGGTGGTTGCAGCAGCAGTACCGGTCAAGTATATGTTCGAGGAGGACAAAGCGGATCAAACTACGCTATCA
TGTACTCCTGGTAAGTTCTCTCTAAACTTCTCCTTATAGATCCAATATCTAACAGAATCTTAGGTACATGCCAAAGGACGAGCCCTCAACCGGT
ATTGGTCACCGTCACGATTGGGAAGGTGTAATCGTCTGGCTCTCCAGCGCCACCGCCACAACTGCCGACAACATCTTAGCTGTTTGTCCTTCGG
CCCACGGAGGCTGGATTGTTCCACCGATGGTTATTCCCTTTCTGGTACCAGCCCTCTTATCAAGTGCGAAAGTATCTGGCCGTCGACCATTCAA
TGGTCTTACTAGTACTGTTGGTGGACAACAACCTATGATTGCTTGGGAGTCTTTACCTACTGCTGCTCAAACTGCTCTTGAGAACACCGATTTC
GGTGCTGCGAATGTTCCATTTATTCCAGCTGTTTTCACAAACAATCTT

>Bcin04g02090(MLST10), partial sequence [organism=Botrytis pseudocinerea, strain D08_H_8_17]
CGGAGGATGATATGGCAAAGTCTATGATTACCAAAGCATTTGTAGGCATGAGTAGTAAACTCTCAAATATGACTATGAATGATGTTTACAGGCC
CTACATCCATGTAAGAAATGTAGAATAGAGAGATCAATAACTGGAACTAATATCGTTTGTAGGCTTTCAGGTTACTTACGCAGTTCAACCCAAT
CACTACAGCTATTGCCGAATCCCCACTATTTCAAATGGCTGTCTCAGCAAATACCATCGAAAAATACACACTGCTAGGCCCTTTCTTCAGAATA
TCTCCTCTGCAACAGGAAGTCACCAGGGAATACTTCAGTGCACCAAAGACGATAGATAGGCGACACATTGCCACATCTCAAGATGCGTTACGAC
TGACCTTACAAACCCATCAAAAAGATTTACTCGATATCATCAACCACTTTGTTCGAGCAAGTCCAATCGCCAAAAGCAAAACCCTGGATTGGTT
CGCCTACATTGTGAATCAAAATCACAAGCGTCGAGCACTTCAGGTAGACCCGAAAGAAGTATCTTCTGATGGCTTTATGCACAATGTCACTGTC
GTTCTAGATGGTCTTTGTGAGCCATTCATGGATACCACATTCTCGAAAATTTCGAAGATTGATATTGATTATCTAAGACGTGCGCCTCGTGTAG
ATATCAAGGACGAGACCAAGTTGAACGCCGATGAGAAGGCTTCTGAGGAGTACTATGAGGACACGGTTCCTGGCACTTCCAATTTCATCTCTGA
GGTGTTCTTTCTGACATTGGCTGCTCATCATTATGGTAGTGAAGCTCTTAATGCCACGCACAAGAGTCTGGAGAAAGACATCAAGTATATTCAA
AAGCAATTGACTGCCGTTGAAGCA

>Bcin01g07220(MLST1), partial sequence [organism=Botrytis pseudocinerea, strain D11_M_E27]
ATGGCATATCTTGTTTCTTTTCGCATTATGCTTTGGAACGTGCTGGACAAGCTTGGGTTAGTGCTCTGCGTGTAGAAGCACTAAAGAAGATTCT
TGCACAACCGAAGTCATGGTTTGAGGAATCCAGGAATTCACCTGGCCGATTGAACGAAGTTTTGGATAGGAACTCTGAGGAAATGCGTAATCTC
GTTGGCCGCTTTGTTGGTATTGTATTCACAGCATTTTTTATGTTATTGATATCAATCATTTGGGCCTTCGTGAATACGTGGAAGCTGACATTAG
TCTCAATGGCAACTGGGGCCAGTTATATACGCTGTCACCAAAACGTTCAATCGCGTGAGTGGAAAATGGGAAAACAAGTGCAACTACGCATCTG
AAATGACCACTAGCATATTTTCAGAGACGTTCTCCAACATTCAAAGTGGTTCGGGCTTTTACTCTGGAAACTTACTTTGCGACAAAACACACCA
AAGCTACAGAAGAACTTTATAAAGTTGGACTAATACGAGCAAACTACTCGGGATTGCTGTGGGGATTGACAGATGCGATGTCATTCTTCATCAC
TGCAACTATCTTTTATTATGCCACGGTTCTCATTACCAAGAGAGAGATCAGTATCGCGACTGCACTACAGACTGTCAATCTTCTATTATTTGGT
ATTTCTAATAGTACGAATATGCTGGCCATGATACCACAAATCAACTCTTCTCGCGTTACAGCTACGCATATGCTTGCATTAGCCAATCTCGATT

CATCTTCCTCCCACGAAAATCAAGGAACCGAACGACTTTCGACAATCTTTCCAATCAAATTCAACCGTCTCTCATTCACATATCCTAATCGTCC
TGAAAAACGAACGATATCATCCTTTTCTCTTTCCCTGATTCCTAACACAACAACTGCACTTGTCGGACCTCCGGCTCCGGAAAATCTACAATAG
CTGCTCTGCTCATTGGCCTCTATCCACCAGATACTTCAAACACCTCCACCGTTGACATTCAATCGCGTCTCCATAAGTAACTGTCACATCCCGT
CTCTCCGGGCTTCTGTCTCACTCGTCCCACAAATACCGATTCTATTTCCAGCTACCATTCTCCATAACATCATTTATGGGCTCCCAGAATCTTC
TCCTTGTGCTAATCTTCCATCTGCTATTGTATCAGCCAAAGATGCTGGGATCCATGAGTTTATCACATCGCTTCCACAAGGTTATAATGCTATG

>Bcin05g07690(MLST2), partial sequence [organism=Botrytis pseudocinerea, strain D11_M_E27]
ACCACTATCACCAAGTCCTTTTCTGCACTTTCCTCATCAAAAACGAACGAGAACATCATCAATAAATACTTTGCTGAAGTAATTGGGTTTTATT
TTGGCCAGGATGCCTTCTCTCTACGACAGCAAGCCTTCGATGATATGTTGTGGGTAGTTCTTGGTTGGTTGGATACTGTCAAATTCATTGATTT
ACATTCTGAATTGCACTATTCAAATGACTCTCAGCCAGAATGGTACGGACAACAATATAAACCTGCATTTGCACATCGAGCGCGACTATTTTGG
GAATTGGCGTCACAAGGATGGGATACTACTCTCTGTGGTGGTGGGATGATATGGTCACCATACCTTACTCCATACAAGAACGCAATTACCAATG
AACTCTATATCGCAGCTTCGATATCGATGTACCTCTATTTCCCTGGAGATGACAATCAATCCCCATTTATGCTTTCCAACCCTTCATATCCACC
TCGCGATCCGAAATATCTACAGGCAGCTGTTGATGCTTACAAATGGCTGAATGGTTCCAATATGACGGATTTACAAGGATTATATGTCGACGGA
TATCATATCTCGAATCTTTCTGGCGGTGAAAACACCCATTGCGATTCTAGAAATGAGATGGTATATACCTACAATCAAGGTGTTTTGCTTACTG
GACAACGTGGTTTGTATGACGCAACCGCCGCACGATCATACCTTGTGGATGGCCACAAACTCATCGCGAATGTTATTAACGCCACAGGCTATGA
TCTGAAACACGATGTTGTTATCTCACCGCCACCCAAAGATGGTTCCGCATTGGCAAAATGGTTTGGCCTGGGTAGGAATGGAATACTGGAAGAA
GGATGCGATTCAAGTGCTTCGTGTTCTCAAAATGGACAAACTTTCAAAGGCATATTCTTTCATCACTTGATTGCATTCTGTAGTGATTTGCCAG
GGGAGCCTATTGCAGGGACGAAAGAAAGCCTGGAACTCGACAGAGAGTGGCATTCTGACAAATGCTCGCAGTATACAAAAT

>Bcin06g01710(MLST3), partial sequence [organism=Botrytis pseudocinerea, strain D11_M_E27]
GTGAGTCCGGCTTTTGTATCTGAGCGTTAAGATAGACACTGATATACCAAGGCAATCACCTAATTTCATGGCTTTCTTCCCTGCATCATCTACC
TACCCTGGAATGCTTGGAGAATTATACTCCGCAGCTTTCACAGCTCCTGCTTTCAATTGGATCTGTTCCCCTGCTGTGACAGAATTGGAGACGG
TTGTAATGGATTGGCTGGCCAAGCTTCTCAATCTCCCAGACTGTTATTTGTCTTCGACTCATGGTGGTGGTGTTATCCAAGGATCAGCCTCGGA
AGCTATCGTTACCGTTATGGTTGCTGCCCGCGATAAATATCTTCGTGAAACTACTGAAGGTCTGTCAGGCATTGAACTCGAGGGTGCGATTGCA
TATAAGAGGAGTAAGCTAGTTGCACTAGGAAGCGAAATGGCACACAGCTCCACGCAGAAAGCAGCGCAGATAGCTGGCGTTAGATTCCGATCGA
TTCCAGTACTCGCATCCAATGATTTCGCCATGACGGGTGATGATCTAGAGAAGGTATTGAAAGAATGCAAATCTCAAGGATTGGAACCCTTCTA
TCTAACTTCGACTTTGGGAACAACATCTACATGTGCAGTTGATGACTTCGCATCTATCGCAACAGTACTTTCGAAATATGCACCTCCAGATGTT
GCAGGCGAGATATGGGTTCACGTCGATGCTGCTTATGCAGGCGCAGCTTTGGTTTGCCCTGAATACCATCATCTAACATCGTCCTTCCAGCATT
TTCATTCCTTCGATATGAACATGCACAAATGGCTTTTGACAAATTTCGACGCTTCTTGTCTATATGTCAAGAAACGCAAAGATCTGATCGATGC
ACTCTCTATAACACCAAGTTATCTTCGCAACGAGTTTTCAGAGAGTGGACTCGTGACCGACTATCGGGACTGGCAAATTCCTCTCGGAAGACGC
TTCCGAAGCTTAAAGATTTGGTTTGTCCTCAGAACCTACG

>Bcin09g03030(MLST4), partial sequence [organism=Botrytis pseudocinerea, strain D11_M_E27]
ACCCTTCAATCCCACCGCAGCTCCTATACCGAAAGCGAGCATCCTACCAATTCTCCTTCCACCTGCGACTTTAAGACCATTGGCTTTTCGCACT
TTCACAAAAAAGCATAGTTTAACATTGACGTCGTCCGCATTACAAGTGTTGGCTACTTTTATTGGAAAGCATTGTGGGACAGGATGGAGGGAAG
AAGGACTGGCAGAGAGAGTTTTAGAGGAGGTCGCCAAGAGTTGGAAGAATAGGAGTGGTGGTGTTATTGTCGAGGGCGAGGGAACGGAATTGAA
GGAGATTCTGAAAGCTTTGGAAGGGAATATGAGTGGTGGAAGGATAGTCGTAGGAAGAGAGCTAAGCCGGCAGAACAGTTTAGTACTGGGATCA
TCACAATATGGAGAGGTCAATCATACAAGACTTGGGCTACGGCCAGGGAATATACCCAGAGAGGATAGTCAGTCAAGTTTGGGAATGTCAACGT
TGGAGGTCAATGACGAGGAAGATGAGGATGGCCTGATGGATCCAAGGAGGTGGTTGAAAGTCATTGATGCATTTGAGCAACCCCGACTGGTGTA
TAATGTTGCTAAGAAGCACTTTGATAGGTATGTTTCAATGATAAAATTTTATCTGAATCAACTAACTCAGTACAGAGATACCTCCAAACCTTCA
TTGTTCCCACCTGCGTCTCATAAAACACTCCTCTTCCAAAACCGCTATAATGTTATCCATCAACGTCTCCTTCGCAATGAATCTTTTCAAACGC
CCGCTTTTCAAGGTGGCAAAGCTTCCCTTCAACGCAGCACGTCCGCCATTACCACTCAACAACAATCATACAAATTAACGCCGATAGCTAATCT
TCTCGGTCGCAATCGCAGCTCTCATATGCTTCTCGGCCTCCTCAGCATTTCACCCACTGGTACCCTCGCCATCAATGACCTGACGGGCAGCATC
GCTCTCGATCTTACACACGCAGCAGCCATTC

>Bcin11g01310(MLST5), partial sequence [organism=Botrytis pseudocinerea, strain D11_M_E27]
ACTGACATGGACTTCATGTGGAACCGGCGTACAATGCGCAACGCTTGAAGTTCCGCTCGAATATGGCGATGCAACGTCAACAGCGAAAGCCAGT
ATTGCGCTTGCTCGTTATCCTGCCACTGTTGCCGCGAGCAAGAAGCTCGGGTCTCTCTTGATAAATCCCGGTGGACCCGGTGCCACTGGTGTTG
GCTTTGTGCAGTCTGGAGCCGGTGCCGCCATCTCGACACTGAGTGGTGGATTATACGATATCATCGGATGGGATCCACGTGGAACCGGTGCTTC
CGCTCCTATTTTGGAATGTTTTCCAAATGCCAGTGCGGAGTATGATTTTAACAACGCGTTTCCATCTGCTCCCAATCTCTGGCTCGGACAATTT
TCAAATGCCAGCGCAAATTCTGCCGTTAGCTCTGCTATCACATCTTTTGACACTTCTGTCGCTGCTCTTGCAAAAGCTTGTGTAGCTCAGAAGT
CCCCCGCTCTTTACACCTCAACAGCAGCATATGTTGCTCGAGACATGGCAGCGATAGTCGATGCCTTGGATGGAACCTCTGCAAAACTCAACTA
CTGGGGTTTCTCATATGGAACTATTTTCCTTGCCGAGTTTATCCAAACTTTCCCAGGCCGCGTGGGAAGAGTTCTTGCCGATGGGGTTTTCGAC
GCAAAGGCAAATGCACTCACATACGTTAGCCAACTTCCCAACGATCAACTCAGTGTTCGTGCTTCGTTGAACGATTTTGCAGCTTTCTGTACCA
CCGCCGGTAGTAAAGGTTGCTCTTTTGCCACCGCCCCTACTGGAACCACCGGTACCGTTGCTACCAGACTGGACAACATAATGAAGGATATGTT
CCTCAATCCCATTGTTGCTTCGGGCTTAAGCATCA

```
>Bcin15g03910(MLST6), partial sequence  [organism=Botrytis pseudocinerea, strain D11_M_E27]
GCCAAAACACAAAATCATCCAACGATGAAGATGATACTCCACTGCCCTTAATTATCTGGCACGGACTCGGCGATAATTACAAAGCGGATGGTCT
TGCGCAAGTTGGAAAGCTAGCTGAAGCTATTCATCCTGGGACTTTTGTCTATAATATTCATGTAGATGAGGATGCATCTGCAGATAGGACAGCT
ACCTTCTTTGGAAATCTCACTCGTGGGTACATCCCCTAGTCTTCTTTTAAATGCCATGCTGACTCTCTTACCAAGTTCAAATCGAAAAGGTCTG
CGAAGACCTCGCCTCCCATCCTATTCTCTCTACCGCGCCTGCCGTCGACGCAATCGGATTCTCCCAAGGCGGCCAATTCTTGCGCGGTTACATA
TCCCGCTGCAATTCTCCACCCATCCGCTCTCTCCTGACCTTCGGTTCCCAACACAACGGCATTTCTGCCTTCCAAGCCTGTGGTCCTGCCGATT
TCCTCTGTCGTGGTGCTCAAACTCTTTTGCGATCCAACACCTGGTCAACCTTTGTCCAATCTCGTCTCGTACCCGCTCAATACTTCAGAGATCC
GGAAAACCTAGACTCTTACCTTGAATATTCCAATTTCCTTGCCGACATCAATAATGAGCGCGTTCTCAAGAACCAAACATATAAATCCAACATG
GAAAAATTGGAACGATTCGTAATGTATGTCTTTGAAGATGATACAACTGTCATCCCTAAGGAAAGTGGATGGTGGGCTGAAGTCAACGGCACGG
AAGTTACACCACTGAAAGAAAGAGCCATTTATAAGGAAGATTGGCTAGGTTTAAAGACATTGGATGAGGCCGGAAAATTAGTTTTCGAAACCAT
TCCAGGGGGACATATGACGTTAGGAGAGGAGATGCTAGAGAAGGCTTTCAAAGAGTACTTTGGCCCAGCAGGGAAGAAATTTGGG

>Bcin16g03460(MLST7), partial sequence [organism=Botrytis pseudocinerea, strain D11_M_E27]
ATGAACCTCTTAATTTGAACTCTTCATTAATCGCAAGTGATGAACCCCTATTTTGCCCAGAGGATAGTTACAAGACGTATATCATTAGTCGAGA
ACCACTCATGATATATATTGACGGATTTTTGAAAGCGAATGAAAGTAAACATCTGGTTGATGTTAGGTATGTTACTTATTCTGATGAAATGAAC
AAGAGAAACTGATGAGATAGTGAACCGCTTTATGAACCGTCTACTGTTTCTCACGGACAGGAAGTTACCATTGATACTTCAGTTCGAAATTCGG
AAGTGGCGGTTTTAGAGAGGGATGAGGTGGTCAGGTGTATTGAGCATAGAGCGAGGGCATTTCAGGGGTGGAGGGATGAGATGGGGATTGAGAA
GTTGAGGACGCAGAGGTATGGGGTTGGAGGACATTATGGGATGCATTTGTAAGTTTTGGGGGATTGACGAAGGCTTTTGTCTATTTCCTATCAG
TACGGATCTTAAAAGAAAGATAGCATGAACACGAGGGCTAACAACTAGGAAATAGCGATTGGAGCGGAGGTAAACGTGGCATAGACCGATTTAG
TACTTTCATGGTCTATGTCGACGTATCTTCTGATATCGAAGGTGGAGGAACGGAATTCCCACGTATTGTGGGACCACAAGGAGGAAGGTGGGAG
GAATTCCTGGAAACTACGGAAGCATTGGATCCAAGAACTGGAAAAAATGTAACAGTAGAAGGGGTGACATTCAAACCAATCAAGGGAAATGCCG
TATTCTGGGAAAATACTGACAACAACGGGAGGGGATATGAT

>Bcin12g03020(MLST8), partial sequence [organism=Botrytis pseudocinerea, strain D11_M_E27]
CGATTGGCTGCGAAGAAAACTGCGCAGCACCGAGTCACCAAAAGCACGAATAAACCGTCGGCAAGAGAAATATTCTCTGAACCACAACATGACA
CGAGCGCGGAAGAGTATATTGGGCGAGAAGCTTCATCAAGAGCACCAAAGCGACAACGAGTCGATGATAATTATAACTCTTACGGTGGAAGAAA
TGAAAATACAGCAGCTTATGCTTCCGGAAAACTTCCATCTGGAAGTATAAATGTTGGTGGAGGTAGGAAGACCACTTTTCAAGAAGAACCTCGA
ACGGCATTTGTCGCTGGCAAGTTGCCCCCTGGAAGTATCAACATCGGTGGAAAGAAGGCTACCCAAATCGAGGACGAAGGAAGAGCAGCTTATG
CTTCCGGGAAATTGCCCCCAGGAAGTATCAGCTCTGGCGCAAAGAAGGCCATTTCATTCCAAGATGAAACGAGACCGGCCTATGTCTCTGGAAA
GCTCCCACATGGCAGTATCGATGGTATGCGAAACCGCGAAATGGCTGCCGTCCACCGCGAAATTGCTGAGGGTGGGAGGAAACCAGGCCAGGTT
GTCTCTTCTCTATTCACATTCAATCCTACATCAAAGAAAACTTTCGATGAATCCGAAGAACAAGCGGAACCTGCAAAACCATCCAATGCGCCTT
TGACAGAGGAAATGGAGACATTCACCAATCTAGGATTATCGAGAAGGCTTGCAGCCCATCTATCGACTAAACTCGATATGAAAGCCCCGACCGC
CATTCAAAAAGCATCTGTGCAGCAGTTGGTATCGGACGATAGCGATGCTTTCATACAAGCAGAGACTGGATCTGGAAAAACTTTGGCATATCTA
CTACCTATAGTCGAGCGAATATTAGCATTGAGTGAGAATGGCGTACAAA

>Bcin02g07770(MLST9), partial sequence [organism=Botrytis pseudocinerea, strain D11_M_E27]
CAGCTTTCCCTTTCGGTCTTGGCATCTACAGTCATTGCCATGCCTACACCATCACAACTTGAGTCTCGGGCCGTTATCGATTCCGATGCCGTTG
TAGGATTTGCCGAAGCTGTTCCCAGTGGGACCGTAGGAACAGTTTATGAGGCATATAAACCATTCCTTAAAGTCGTAAATGGATGCGTACCATT
CCCTGCCGTCGATGCATCGGGTAACACAGGGTATGTCCTTATACCTTTCTCTTCCTCACGATTGCTATTGAGTCTCTAACATATTTAGTGGTG
GTTTGTCACCAACTGGCAGTAGCAATGGTGGTTGCAGCAGCAGTACCGGTCAAGTATATGTTCGAGGAGGACAAAGCGGATCAAACTACGCTAT
CATGTACTCCTGGTAAGTTCTCTCTAAACTTCTCCTTATAGATCCAATATCTAACAGAATCTTAGGTACATGCCAAAGGACGAGCCCTCAACCG
GTATTGGTCACCGTCACGATTGGGAAGGTGTAATCGTCTGGCTCTCCAGCGCCACCGCCACAACTGCCGACAACATCTTAGCTGTTTGTCCTTC
GGCCCACGGAGGCTGGGATTGTTCCACCGATGGTTATTCCCTTTCTGGTACCAGCCCTCTTATCAAGTACGAAAGTATCTGGCCCGTCGACCAT
TCAATGGGTCTTACTAGTACTGTTGGTGGACAACAACCTATGATTGCTTGGGAGTCTTTACCTACTGCTGCTCAAACTGCTCTTGAGAACACCG
ATTTCGGTGCTGCGAATGTTCCATTTATTCCAGCTGTTTTCACAAACAATCT

>Bcin04g02090(MLST10), partial sequence [organism=Botrytis pseudocinerea, strain D11_M_E27]
CGGAGGATGATATGGCAAAGTCTATGATTACCAAAGCATTTGTAGGCATGAGTAGTAAACTCTCAAATATGACTATGAATGATGTTTACAAGCC
CTACATCCATGTAAGAAATGTAGAATAGAGAGATCAATAACTGGAACTAATATCGTTTGTAGGCTTTCAAGTTACTTACGCAGTTCAACCCAAT
CACTACAGCTATTGCCGAATCCCCACTATTTCAAATGGCTGTCTCAGCAAATACCATCGAAAAGTACACACTGCTAGGCCCTTTCTTCAGAATA
TCTCCTCTGCAACAGGAAGTTACCAGGGAATACTTCAGTGCACCAAAGACGATAGATAGGCGACACATTGCCACATCTCAAGATGCGTTACGAC
TGACCTTACAAACCCATCAAAAAGATTTACTCGATATCATCAACCACTTTGTTCGAGCAAGTCCAATCGCCAAAAGCAAAACCTTGGATTGGTT
CGCCTACATTGTGAATCAAAATCACAAGCGTCGAGCACTTCAGGTAGACCCGAAAGAAGTATCTTCTGATGGCTTTATGCACAATGTCACTGTC
GTTCTAGATGGTCTTTGTGAGCCATTCATGGATACCACATTCTCGAAAATTTCGAAGATTGATATTGATTATCTAAGACGTGCGCCTCGTGTAG
ATATCAAGGACGAGACCAAGTTGAACGCCGATGAGAAGGCTTCTGAGAAGTACTATGAGGACACGGTTCCTGGCACTTCCAATTTCATCTCTGA
GGTGTTCTTTCTGACATTGGCTGCTCATCATTATGGTAGTGAAGCTCTTAATGCCACGCACAAGAGTCTGGAGAAAGACATCAAATATATTCAA
AAGCAATTGACTGCCGTTGAAGCA
```

>Bcin01g07220(MLST1), partial sequence [organism=Botrytis pseudocinerea, strain D11_T_B18]
GATGGCATATCTTGTTTCTTTTCGCATTATGCTTTGGAACGTGCTGGACAAGCTTGGGTTAGTGCTCTGCGTGTAGAAGCACTAAAGAAGATTC
TCGCACAACCGAAGTCATGGTTTGAGGAATCCAGAAATTCACCTGGCCGGTTGAACGAAGTTTTGGATAGGAACTCTGAGGAAATGCGTAATCT
CGTTGGCCGCTTTGCTGGTATTGTATTCACAGCGTTTTTTATGCTATTGATATCAATCATTTGGGCTTTCGTGAATACATGGAAACTGACATTA
GTCTCGATGGCAACTGGGCCAGTTATATACGCTGTCACCAAAACGTTCAATCGCGTGAGTGGAAAATGGGAAACAAGTGCAACTACGCCTCTGA
AATGACCACTGGCATATTTTCAGAGACTTTCTCCAACATCAAAGTGGTTCGGGCTTTTACTCTGGAAACTTACTTTGGGACAAAACACACCAAA
GCTACAGAAGAACTTTATAAAGTTGGACTAATACGAGCAAACTACTCGGGATTGCTGTGGGGATTGACAGATGCGATGTCATCCTTCATCACTG
CAACTATCTTTTATTATGCCACGGTTCTCATTACCAAGAGAGAGATCAGTATCGCGACTGCACTACAGACTGTCAATCTTCTATTATTTGGTAT
TTCTAATAGTACGAATATGCTGGCCATGATACCACAATCAACTCTTCTCGCGTTACAGCTACGCATATGCTTGCATTAGCCAATCTCGATTCAT
CTTCCTCCCACGAAAATAAAGGAACCGAACGGCTTTCGACAATCTTTCCAATCAAATTCAACCGTCTCTCATTCACATATCCTACTCGTCCCGA
AAAACGAACGATATCATCCTTTTCTCTTTCCCTGATTCCTAACTCAACAACTGCACTTGTCGGACCCTCCGGCTCCGGAAAATCTACAATAGCT
GCTCTGCTCATTGGTCTCTATCCGCCAGATACTTCAACACCTCCACCGTTGACATTCAATCGCGTCTCCATAAGTAACTGTCACATTCCGCCTC
TCCGGGCTTCTCTCTCACTCGTTCCACAAATACCGATTCTATTTCCAGCTACCATACTCCATAACATCATTTATGGTCTCCCAGAATCTTCTCC
TTGTGCTAGTCTTCCATCAGCTATTGCATCAGCAAAAGATGCTGGGATCCATGAATTTATCACATCGCTTCCACAAGGTTATGATACTAT

>Bcin05g07690(MLST2), partial sequence [organism=Botrytis pseudocinerea, strain D11_T_B18]
ACCACTATCACCAAGTCCTTCTCTGCACTTTCCTCATCAAAAACGAACGAGAACATCATCAATAAATACTTTGCTGAAGTAATTGGGTTTTATT
TTGGCCAGGATGCCTTCTCTCTACGACAGCAAGCCTTCGATGATATGTTGTGGGTAGTTCTTGGCTGGCTGGATACTGTCAAATTCATTGATTT
ACATTCTGAATTGCACTATTCAAACGACTCTCAGCCAGAATGGTACGGACAACAATATAAACCTGCATTTGCACATCGAGCGCGACTATTTTGG
GAATTGGCTTCACAAGGATGGGATACTACTCTCTGTGGTGGTGGGATGATATGGTCACCATACCTTACTCCATACAAGAATGCAATTACCAATG
AACTCTATATCGCAGCTTCGATATCGATGTACCTCTATTTCCCCGGAGATGACAATCAATCCCCATTTATGCTTTCCAACCCTTCATATCCACC
TCACGATCCGAAATATCTACAGGCAGCTGTTGATGCTTACAAATGGCTGAATGGTTCCAACATGACGGATTTACAAGGATTATATGTCGACGGG
TATCATATCTCGAATCTTTCTGGCGGTGAAAACACCCATTGCGATTCTAGAAATGAGATGGTATATACCTACAATCAAGGTGTTTTGCTTACTG
GACAACGTGGTTTGTATGACGCAACCGCCGCACGATCATACCTTGTGGATGGCCACAAACTCATCGCGAATGTTATTAATGCCACAGGCTATGA
CCTGAAACACAATGTTGCCATCTCACCGCCACCCAAAGATGGTTCCGCATTGGCAAAGTGGTTTGGCCTGGGTAGGAATGGAATACTGGAAGAA
GGATGCGATTCAAGTGCTTCGTGTTCTCAAAATGGACAAACTTTCAAAGGCATATTCTTTCACCACTTGATTGCGTTCTGTAGTGATTTGCCAG
GGGAGCCTATTGCAGGGACGAAGGAAGGCCTAGAACTCGACAGAGTGTGGCATTCTGACAAATGCTCACAGTATACAAAAT

>Bcin06g01710(MLST3), partial sequence [organism=Botrytis pseudocinerea, strain D11_T_B18]
GGTGAGTCTGACTTTTGTATTTGAGCGTTAAGATGGACACTGATGTACCAAGGCAATCACCTAATTTCATGGCGTTCTTCCCTGCATCATCTAC
CTACCCTGGAATGCTGGGAGAATTATACTCAGCAGCTTTCACAGCTCCTGCTTTCAATTGGATCTGTTCCCCTGCTGTGACAGAATTGGAGACG
GTTGTAATGGATTGGTTGGCCAAGCTTCTCAATCTCCCAGACTGTTATTTGTCGTCGACTCATGGTGGTGGTGTTATCCAAGGATCAGCCTCGG
AAGCTATCGTTACCGTTATGGTTGCTGCCCGCGATAAGTATCTTCGTGAAACCACTGAAGGTCTGTCGGGAATTGAACTCGAGGATGCGATTGC
ATATAAGAGGAGTAAGCTAGTTGCACTAGGAAGCGAAATGGCACACAGCTCCACGCAGAAAGCAGCGCAGATAGCTGGCGTTAGATTCCGATCG
ATTCCAGTACTCGCATCCAATGATTTCGCCATGACGGGTGATGATTTAGAGAAGGTATTGAAAGAATGCAAATCTCAAGGATTGGAACCCTTCT
ATCTAACTTCGACGTTGGGAACAACATCTACATGCGCAGTTGACGACTTCGCATCTATTGCAACAGTACTTTCAAAATATGCACCTCCAGATGT
TGCAGGCGAGATCTGGGTTCACGTCGATGCTGCTTATGCAGGTGCAGCTTTGGTTTGCCCTGAATACCATCATCTAACATCGTCCTTCCAGCAT
TTCCATTCCTTTGATATGAACATGCACAAATGGCTTCTGACAAATTTCGACGCTTCTTGCCTATATGTCAAGAAACGCAAAGATCTGATCGATG
CACTCTCCATAACACCAAGTTATCTTCGCAACGAGTTTTCAGAGAGTGGACTCGTAACCGACTATCGGGACTGGCAAATTCCTCTCGGAAGACG
TTTCCGAAGCTTAAAGATTTGGTTTGTCCTCAGAACCTAC

>Bcin09g03030(MLST4), partial sequence [organism=Botrytis pseudocinerea, strain D11_T_B18]
ACCCTTCAACCCCACCGCAGCTCCTATACCGAAAGCGAGTATCCTACCAATTCTCCTTCCACCTGCGACTTTAAGACCATTGGCTTTTCGCACT
TTCACAAAAAGCATAGTTTAACATTGACGTCGTCGGCATTACAAGTGTTGGCTACTTTTATTGGAAAGCATTGTGGGACAGGATGGAGGGAGGA
AGGACTGGCAGAGAGAGTCTTGGAGGAGGTCGCCAAGAGTTGGAAGAATAGGAGTGGCGGTGTCATTGTCGAGGGCGAGGGAACGGAATTGAAG
GAGATTCTGAAAGCTTTGGATGGGAATATGAGTGGTGGAAGGATAGTCATAGGAAGAGAGCTAAGCCGGCAGAATAGTTTAGTACTGGGATCAT
CACAATATGGAGAGGTCAATCATACAAGACTTGGGCTACGGCCAGGGAATATACCCAGAGAGGATAGTCAGTCAAGTTTGGGAATGTCAACGTT
GGAGGTCAATGACGAGGAAGATGAGGATGGCCTGATGGATCCAAGAAGGTGGTTAAAAGTCGTTGATGCACTTGAGCAACCTCGACTGGTGTAC
AATGTTGCTAAAAAGCACTTTGATAGGTATGTTTCAATGATAAAATTTTATCTGAATCGACTAACTCAGTACAGAGATACCTCCAAACCTTCAT
TGTTCCCACCTGCGTCTCATAAAACACTCCTCTTCCAAAACCGCTATAATGTTATCCATCAACGTCTCCTTCGCAATGAATCTTTTCAAACGCC
CGCTTTCCAAGGTGGCAAATCTTCCCTTCAACGCAGCACGTCCGCCATTACCACCCAACAACAATCATACAAATTAACGCCGATATCTAATCTT
CTCGGTCGCAATCGCAGCTCTCATATGCTTCTCGGTCTCCTCAGTATTTCACCCACTGGTACCCTCGCCATCAATGACCTGACGGGCAGTATCG
CTCTTGATCTTACACACGCAGCAGCCATTC

>Bcin11g01310(MLST5), partial sequence [organism=Botrytis pseudocinerea, strain D11_T_B18]
ACTGACATGGACCTCATGTGGAACCGGCGTAGAATGCGCAACGCTTGAAGTTCCGCTCGAATATGGCGATGCAACGTCAACGGCAAAAGCCAGT

GTTGCGCTTGCTCGTTATCCTGCCACTTTTGCCGCGAGCAAGAAGCTCGGGTCTCTCTTGATAAATCCCGGTGGACCCGGTGCCTCTGGTGTTG
GCTTTGTGCAGTCTGGAGCTGGTGCCGCCGTCTCGACACTGAGTGGTGGATTATACGATATCATCGGATGGGATCCACGTGGAACCGGTGCTTC
GGCTCCTATTTTGGAATGTTTTGCAAATGCCAGTGCGGAGTATGATTTTAACAACGCGTTCCATCTGCTCCGAATCTCTGGCTCGGACAATTTG
CGAATGCCAGCGCAAATTCTGCTGTTAGCTCTGCTATCACATCCTTTGACACTTCTGTCGCTGCTCTTGCAAAAGCTTGCGTGGCTCAGAAATC
TCCCGCTCTTTACACCTCAACAGCAGCATATGTTGCTCGAGACATGGCAGCGATAGTCGATGCATTGGATGGGACCTCTGCAAAACTTAACTAC
TGGGGTTTCTCATATGGAACTATCTTCCTAGCTGAGTTTATCCAAACTTTCCCAGGCCGCGTGGGAAGAGTTCTTGCCGATGGTGTTTTCGACG
CAAAGGCAAATGCACTCACATACGTTAGCCAACTTCCCAACGATCAACTCAGTGTTCGTGCTTCGTTGAACGATTTTGCAGCTTTCTGCACCAC
CGCCGGTAGTAAAGGTTGCTCTTTTGCCACCGCCCCTACTGGAACCTCAGGTACTGTTGCTACCAGACTGGACAACATAATGAAGGATATGTTC
CTCAATCCTATTGTTGCTTCGGGCTTGAGCATCA

>Bcin15g03910(MLST6), partial sequence [organism=Botrytis pseudocinerea, strain D11_T_B18]
GCCAAAACACAAAATCATCCAACGATGAAGATGATACTCCACTTCCCTTGATTATCTGGCATGGACTCGGCGATAATTACAAAGCGGATGGTCT
TGCGCAAGTTGGAAAACTAGCTGAAGCTATTCATCCTGGGACTTTTGTCTATAATATTCATGTAGATGAGGATGCATCTGCAGATAGGACAGCT
ACCTTCTTTGGAAATCTCACTCGTGAGTACATCCCCTATTTTTCCTTTAAATACCATACTAACTCTCTTACCAAGTTCAAATCGAAAAGGTCTG
CGAAGACCTCGCCTCCCATCCTATTCTCTCTACCGCGCCCGCCGTCGACGCAATTGGATTCTCCCAAGGCGGCCAATTCTTGCGCGGTTACATA
CCCCGCTGCAATGCTCCACCCATCCGCTCTCTCCTGACCTTCGGTTCCCAACACAACGGCATTTCTGCCTTCCAATCCTGTGGTCCTGCCGATT
TCCTCTGTCGCGGTGCTCAAACCCTTTTGCGATCCAACACCTGGTCAACCTTTGTCCAATCTCGTCTCGTACCCGCTCAATACTTCAGAGATCC
GGAAAACCTAGACTCTTACCTTGAATATTCCAATTTCCTTGCCGACATCAATAATGAGCGCGTTCTCAAGAACCAAACATATAAATCCAACATG
GAAAAATTGGAACGATTCGTAATGTATGTCTTTGAAGACGATACAACTGTCATCCCTAAGGAAAGTGGATGGTGGGCTGAAGTCAACGGCACGG
AAGTTACACCACTGAAAGAAAGAGCCATTTATAAAGAAGATTGGCTAGGGTTAAAGACGTTGGATGAGGCCGGAAAATTAGTTTTCGAAACCAT
TCCAGGGGGACATATGACGTTAGGAGAGGAGATACTAGAGAAGGCTTTCGAAGAGTACTTTGGTCCAGCAGGGAAGAAATTTGGG

>Bcin16g03460(MLST7), partial sequence [organism=Botrytis pseudocinerea, strain D11_T_B18]
ATGAACCTCTTAATTTGAACTCTTCATTAATTGCAAGTGATGAGCCCCTATTCTGCCCAGAGGATAGTTACAAGACGTATATCATTAGTCGAGA
ACCACTCATGATATACATTGACGGATTTTTGAAAGCGAATGAAAGTAAACACTTGGTTGATGTTAGGTGTGTTATTTATTCTGATGAAATGAAC
AAGAGAGACTGATGAGATAGTGAACCGCTTTATGAACCGTCTACTGTTTCTCACGGACAGGAAGTTACCATTGATCCTTCGGTTCGAAATTCTG
AAGTGGCGGTTTTAGAGAGGGATGAGGTGGTCAGGTGTATTGAGCATAGAGCGAGGGCATTTCAGGGGTGGAGGGACGAGATGGGGATTGAGAA
GCTGAGGACGCAGAGGTATGGGGTTGGAGGACATTATGGGATGCATTTGTAAGTTTTGGGGGATTGACGAAGGCCTCTGTCTATTTTCTATCAG
TACGGATCTTGAAAGAAAGATAGCATGAACACGAGGGCTAATAACTAGGAAATAGCGATTGGAGCGGAGGTAAACGTGGCATAGACCGATTTAG
TACTTTCATGGTCTATGTCGACGTATCCTCTGATATCGAAGGTGGAGGAACGGAATTCCCACGTATTGTGGGACCAAAAGGAGGAAGGTGGGAG
GACTTCCTGGAAACTACGGAAGCATTGGATCCAAGAACTGGAGAAAATGTAACAGTAGAAGGGGTGACATTCAAACCAATCAAGGGAAATGCCG
TATTCTGGGAAAATACTGACAACAACGGGAGGGGCTATGAT

>Bcin12g03020(MLST8), partial sequence [organism=Botrytis pseudocinerea, strain D11_T_B18]
CGATTGGCTGCGAAGAAAACTGCGCAGCACCGAGTCACCAAAAGCACGATAAACCATCGGCAAGGGAAATATTCTCTGAACCACAACATGACAC
GAGCGCGGAAGAGTATATCGGGCGAGAAGCTTCATCAAGAGCACCAAAGCGACAACGAGTCGATGATAATTATAACTCTTACGGTGGAAGAAAT
GAGAATACAGCAGCTTATGCTTCCGGGAAACTTCCATCTGGAAGTATAAATGTTGGTGGAGGTAGGAAGACCACTTTTCAAGAAGAACCTCGAA
CGGCATTTGTCGCTGGCAAGTTGCCCCTGGAAGTATCAACATTGGTGGAAAGAAGGCTACCCAAATCGAGGACGAGGGTAGAGCAGCTTATGCT
TCCGGAAAATTGCCCCCAGGAAGTATAAACTCTGGCGCAAAGAAGGCGATTTCATTCCAAGATGAAACGAGACCGGCTTATGTCTCTGGAAAGC
TTCCACATGGTAGTATCGACGGTATGCGAAACCGTGAAATGGCTGCCGTCCACCGCGAAATTGCTGAGGGTGGGAGGAAACCAGGCCAGGTTGT
CTCTTCTCTATTCACATTCAATCCTACTTCAAAGAAAACTTTCGATGAACCGAAGAACAAGCGGAACCGGCAAAACCATCCAATGCACCTTTGA
CAGAGGAAATGGCGACTTTCACCAATCTAGGGTTATCGAGAAGGCTTGCAGCCCATCTATCGACTAAACTCGATATGAAAGCCCCGACCGCCAT
TCAAAAAGCATCTGTGCAGCAGTTGGTATCGGACGATAGCGATGCTTCCATACAAGCAGAGACTGGATCTGGAAAAACTTTGGCATATCTACTA
CCTATAGTCGAGCGAATATTAGCATTGAGTGAGAATGGCGTACAAA

>Bcin02g07770(MLST9), partial sequence [organism=Botrytis pseudocinerea, strain D11_T_B18]
CAGCTTTCCCTTTCGGTCTTGGCATCTACAGTCATTGCCATCCCTACACCATCACAACTTGAGTCTCGGGCCGTTATCGATTCCGATGCCGTTG
TAGGATTTGCCGAAACTGTTCCCAGTGGGACCGTAGGAACAGTTTACGAGGCATATAAACCATTCCTTAAAGTCGTAAATGGATGCGTACCATT
CCCTGCCGTCGATGCATCGGGTAACACAGGGTATGTCCTTATACCATTCTCTTCCACACGATTGCTATTGAGTCTCTAACATATTTTAGTGGTG
GTTTGTCACCAACTGGCAGTAGCAATGGTGGTTGCAGCAGCAGTACCGGTCAAGTATATGTTCGAGGAGGACAAAGCGGATCAAACTACGCCAT
CATGTACTCCTGGTAAGTTCTCTCTAAACTTCTCCTTATAGATCCAACCTAACAAAATCTTAGGTACATGCCAAAGGACGAGCCCTCAACCGGT
ATTGGTCACCGTCACGATTGGGAAGGTGTAATTGTCTGGCTCTCAAGCGCCACCGCCACAACTGCCGACAACATCTTAGCCGTTTGTCCTTCCG
CCCACGGAGGCTGGGATTGTTCCACCGATGGCTATTCCCTTTCTGGTACCAGCCCTCTTATCAAGTACGAAAGTATCTGGCCCGTCGATCATTC
AATGGGTCTTACTAGTACTGTTGGTGGAAAACAACCTGTGATTGCTTGGGAGTCTTTACCAACTGCTGCTCAAACTGCTCTTGAGAACACCGAT
TTCGGTGCTGCGAATGTTCCATTCATTCCGGCTGTTTTCACAGATAATCT

>Bcin04g02090(MLST10), partial sequence [organism=Botrytis pseudocinerea, strain D11_T_B18]

CGGAGGATGATATGGCAAAGTCTATGATTACCAAAGCATTTGTAGGCATGAGTAGTAAACTGTCGAATATGACTATGAATGATGTTTACAAGCC
CTACATCCATGTAAGAAATGTAGAATAGAGAGATCAGTAACTGGAACTAATATCGTTTGTAGGCTTTCAAGTTACTTACGCAGTTCAACCCAAT
CACTACAGCTATTGCCGAATCCCCACTATTTCAAATGGCTGTCTCAGCAAATACCATCGAAAAGTACACACTGCTAGGCCCTTTCTTCAGAATA
TCTCCTCTGCAACAGGAAGTTACCAGGGAATACTTCAGTGCGCCAAAGACGATAGATAGACGACACATTGCCACATCTCAAGATGCGTTACGAT
TAACCTTACAAACCCATCAAAAAGATTTACTTGATATCATCAACCACTTTGTTCGAGCAAGTCCAATCGCAAAAAGCAAAACCCTGGATTGGTT
CGCCTACATTGTGAATCAAAATCACAAACGTCGAGCACTTCAGGTAGACCCGAAAGAAGTGTCTTCTGATGGCTTTATGCACAATGTCACTGTC
GTTCTAGATGGTCTTTGTGAGCCATTCATGGATACCACATTCTCGAAAATTTCGAAGATTGATATTGATTATCTAAGACGTGCGCCTCGTGTAG
ATATCAAGGACGAGACCAAGTTGAACGCTGATGAGAAGGCTTCTGAGAAGTATTATGAGGACACTGTTCCTGGCACTTCTAATTTCATCTCTGA
GGTCTTCTTTCTGACATTGGCTGCTCATCATTATGGTAGTGAAGCTCTTAATGCCACGCATAAGAGTCTGGAGAAAGACATCAAATATATTCAA
AAGCAATTGACTGCCGTTGAAGCA

>Bcin01g07220(MLST1), partial sequence [organism=Botrytis pseudocinerea, strain D11_T_E01]
GATGGCATATCTTGTTTCTTTTCGCATTATGCTTTGGAACGTGCTGGACAAGCTTGGGTTAGTGCTCTGCGTGTAGAAGCACTAAAGAAGATTC
TTGCACAACCGAAGTCATGGTTTGAGGAATCCAGGAATTCACCTGGCCGATTGAACGAAGTTTTGGATAGGAACTCTGAGGAAATGCGTAATCT
CGTTGGCCGCTTTGTTGGTATTGTATTCACAGCATTTTTTATGTTATTGATATCAATCATTTGGGCTTTCGTGAATACGTGGAAGCTGACATTA
GTCTCAATGGCAACTGGGCCAGTTATATACGCTGTCACCAAAACGTTCAATCGCGTGAGTGGAAAATGGGAAAACAAGTGCAACTACGCATCTG
AAATGACCACTAGCATATTTTCAGAGACGTTCTCCGACATCAAAGTGGTTCGGGCTTTTACTCTGGAAACTTACTTTGCGACAAAACACACCAA
AGCTACAGAAGAACTTTATAAGGTTGGACTAATACGAGCAAACTACTCGGGATTGCTGTGGGGATTGACAGATGCGATGTCATTCTTCATCACT
GCAACTATCTTTTATTATGCCACGGTTCTCATTACCAAGAGAGAGATCAGTATCGCGACTGCACTACAGACTGTCAATCTTCTATTATTTGGTA
TTTCTAATAGTACGAATATGCTGGCCATGATACCACAAATCAACTCTTCTCGCGTTACAGCTACGCATATGCTTGCATTAGCCAATCTCGATTC
ATCTTCCTCCCACGAAAATCAAGGAACCGAACGACTTTCGACAATCTTTCCAATCAAATTCAACCGTCTCTCATTCACATATCCTAATCGTCCT
GAAAAACGAACGATATCATCCTTTTCTCTTTCCCTGATTCCTAACACAACAACTGCACTTGTCGGACCCTCCGGCTCCGGAAAATCTACAATAG
CTGCTCTGCTCATTGGCCTCTATCCACCAGATACTTCAACACCTCCACCGTTGACATTCAATCGCGTCTCCATAAGTAACTGTCACATCCCGTC
TCTCCGGCTTCTGTCTCACTCGTCCCACAAATACCGATTCTATTTCCAGCTACCATTCTCCTTAACATCATTTATGGGCTCCCAGAATCTTCTC
CTTGTGCTAATCTTCCATCTGCTATTGTACCAGCCAAAGATGCTGGATCCATGAGTTTATCACATCGCTTCACAAGGTTATAATGCAAT

>Bcin05g07690(MLST2), partial sequence [organism=Botrytis pseudocinerea, strain D11_T_E01]
ACCACTATCACCAAGTCCTTTTCTGCACTTTCCTCATCAAAAACGAACGAGAACATCATCAATAAATACTTTGCTGAAGTAATTGGGTTTTATT
TTGGCCAGGATGCCTTCTCTCTACGACAGCAAGCCTTCGATGATATGTTGTGGGTAGTTCTTGGTTGGTTGGATACTGTCAAATTCATTGATTT
ACATTCTGAATTGCACTATTCAAATGACTCTCAGCCAGAATGGTACGGACAACAATATAAACCTGCATTTGCACATCGAGCGCGACTATTTTGG
GAATTGGCGTCACAAGGATGGGATACTACTCTCTGTGGTGGTGGGATGATATGGTCACCATACCTTACTCCATACAAGAACGCAATTACCAATG
AACTCTATATCGCAGCTTCGATATCGATGTACCTCTATTTCCCTGGAGATGACAATCAATCCCCATTTATGCTTTCCAACCCTTCATATCCACC
TCGCGATCCGAAATATCTACAGGCAGCTGTTGATGCTTACAAATGGCTGAATGGTTCCAATATGACGGATTTACAAGGATTATATGTCGACGGA
TATCATATCTCGAATCTTTCTGGCGGTGAAAACACCCATTGCGATTCTAGAAATGAGATGGTATATACCTACAATCAAGGTGTTTTGCTTACTG
GACAACGTGGTTTGTATGACGCAACCGCCGCACGATCATACCTTGTGGATGGCCACAAACTCATCGCGAATGTTATTAACGCCACAGGCTATGA
TCTGAAACACGATGTTGTTATCTCACCGCCACCCAAAGATGGTTCCGCATTGGCAAAATGGTTTGGCCTGGGTAGGAATGGAATACTGGAAGAA
GGATGCGATTCAAGTGCTTCGTGTTCTCAAAATGGACAAACTTTCAAAGGCATATTCTTTCATCACTTGATTGCATTCTGTAGTGATTTGCCAG
GGGAGCCTATTGCAGGGACGAAAGAAAGCCTGGAACTCGACAGAGAGTGGCATTCTGACAAATGCTCGCAGTATACAAAAT

>Bcin06g01710(MLST3), partial sequence [organism=Botrytis pseudocinerea, strain D11_T_E01]
GTGAGTCCGGCTTTTGTATCTGAGCGTTAAGATAGACACTGATATACCAAGGCAATCACCTAATTTCATGGCTTTCTTCCCTGCATCATCTACC
TACCCTGGAATGCTTGGAGAATTATACTCCGCAGCTTTCACAGCTCCTGCTTTCAATTGGATCTGTTCCCCTGCTGTGACAGAATTGGAGACGG
TTGTAATGGATTGGCTGGCCAAGCTTCTCAATCTCCCAGACTGTTATTTGTCTTCGACTCATGGTGGTGGTGTTATCCAAGGATCAGCCTCGGA
AGCTATCGTTACCGTTATGGTTGCTGCCCGCGATAAATATCTTCGTGAAACTACTGAAGGTCTGTCAGGCATTGAACTCGAGGGTGCGATTGCA
TATAAGAGGAGTAAGCTAGTTGCACTAGGAAGCGAAATGGCACACAGCTCCACGCAGAAAGCAGCGCAGATAGCTGGCGTTAGATTCCGATCGA
TTCCAGTACTCGCATCCAATGATTTCGCCATGACGGGTGATGATCTAGAGAAGGTATTGAAAGAATGCAAATCTCAAGGATTGGAACCCTTCTA
TCTAACTTCGACTTTGGGAACAACATCTACATGTGCAGTTGATGACTTCGCATCTATCGCAACAGTACTTTCGAAATATGCACCTCCAGATGTT
GCAGGCGAGATATGGGTTCACGTCGATGCTGCTTATGCAGGCGCAGCTTTGGTTTGCCCTGAATACCATCATCTAACATCGTCCTTCCAGCATT
TTCATTCCTTCGATATGAACATGCACAAATGGCTTTTGACAAATTTCGACGCTTCTTGTCTATATGTCAAGAAACGCAAAGATCTGATCGATGC
ACTCTCTATAACACCAAGTTATCTTCGCAACGAGTTTTCAGAGAGTGGACTCGTGACCGACTATCGGGACTGGCAAATTCCTCTCGGAAGACGC
TTCCGAAGCTTAAAGATTTGGTTTGTCCTCAGAACCTAC

>Bcin09g03030(MLST4), partial sequence [organism=Botrytis pseudocinerea, strain D11_T_E01]
ACCCTTCAATCCACCGCAGCTCCTATACCGAAAGCGAGCATCCTACCAATTCTCCTTCCACCTGCGACTTTAAGACCATCGGCTTTTCGCACTT
TCACAAAAAAGCATAGTTTAACATTGACGTCGTCCGCATTACAAGTGTTGGCTACTTTTATTGGAAAGCATTGTGGGACAGGATGGAGGGAAGA
AGGACTGCAGAAAGAGTTTTAAAGGAGGTCGCCAAGAGTTGGAAGAATAGGAGTGGTGGTGTTATTGTCGAGGGCGAGGGAACGGAATTGAAGG
AGATTCTGAAAGCTTTGGAAGGGAATATGAGTGGTGGAAGGATAGTCGTAGGAAGAGAGCTAAGCCGGCAGAACAGTTTAGTACTGGGATCATC

ACAATATGGAGAGGTCAATCATACAAGACTTGGGCTACGGCCAGGGAATATACCCAGAGAGGATAGTCAGTCAAGTTTGGGAATGTCAACGTTG
GAGGTCAATGACGAGGAAGATGAGGATGGCCTGATGGATCCAAGGAGGTGGTTGAAAGTCATTGATGCATTTGAGCAACCCCGACTGGTGTATA
ATGTTGCTAAGAAGCACTTTGATAGGTTATGTTTCAATGATAAAATTTTATCTGAATCAAACTAACTCAGTACAGAGATACCTCCAAACCTTCA
TTGTTCCACCTGCGTCTCATAAAACACTCCTCTTCCAAAACCGCTATAATGTTATCCATCAACGTCTCCTTCGCAATGAATCTTTTCAAACGCC
CGCTTTTCAAGGTGGCAAAGCTTCCCTTCAACGCAGCACGTCCGCCATTACCACTCAACAACAATCATACAAATTAACGCCGATAGCTAATCTT
CTCGGTCGCAATCGCAGCTCTCATATGCTTCTCGGCCTCCTCAGCATTTCACCCACTGGTACCCTCGCCATCAATGACCTGACGGGCAGCATCG
CTCTCGATCTTACACACGCAGCAGCCATTC

>Bcin11g01310(MLST5), partial sequence [organism=Botrytis pseudocinerea, strain D11_T_E01]
ACTGACATGGACTTCATGTGGAACCGGCGTACAATGCGCAACGCTTGAAGTTCCGCTCGAATATGGCGATGCAACGTCAACAGCGAAAGCCAGT
ATTGCGCTTGCTCGTTATCCTGCCACTGTTGCCGCGAGCAAGAAGCTCGGGTCTCTCTTGTAAATCCCGGTGGACCCGGTGCCACTGGTGTTGG
CTTTGTGCAGTCTGGAGCCGGTGCCGCCATCTCGACACTGAGTGGTGGATTATACGATATCATCGGATGGGATCCACGTGGAACCGGTGCTTCC
GCTCCTATTTTGGAATGTTTTCCAAATGCCAGTGCGGAGTATGATTTTAACAACGCGTCTCCATCTGCTCCCAATCTCTGGCTCGGACAATTTT
CAAATGCCAGCGCAAATTCTGCCGTTAGCTCTGCTATCACATCTTTTGACACTTCTGTCGCTGCTCTTGCAAAAGCTTGTGTAGCTCAGAAGTC
CCCCGCTCTTTACACCTCAACAGCAGCATATGTTGCTCGAGACATGGCAGCGATAGTCGATGCCTTGGGTGGAACCTCTGCAAAACTCAACTAC
TGGGGTTTCTCATATGGAACTATTTTCCTTGCCGAGTTTATCCAAACTTTCCCAGGCCGCGTGGGAAGAGTTCTTGCCGATGGGGTTTTCGACG
CAAAGGCAAATGCACTCACATACGTTAGCCAACTTCCCAACGATCAACTCAGTGTTCGTGCTTCGTTGAACGATTTTGCAGCTTTCTGTACCAC
CGCCGGTAGTAAAGGTTGCTCTTTTGCCACCGCCCCTACTGGAACCACCGGTACCGTTGCTACCAGACTGGACAACATAATGAAGGATATGTTC
CTCAATCCCATTGTTGCTTCGGGCTTAAGCATCA

>Bcin15g03910(MLST6), partial sequence [organism=Botrytis pseudocinerea, strain D11_T_E01]
GCCAAAACACAAAATCATCCAACGATGAAGATGATACTCCACTGCCCTTAATTATCTGGCACGGACTCGGCGATAATTACAAAGCGGATGGTCT
TGCGCAAGTTGGAAAGCTAGCTGAAGCTATTCATCCTGGGACTTTTGTCTATAATATTCATGTAGATGAGGATGCATCTGCAGATAGGACAGCT
ACCTTCTTTGGAAATCTCACTCGTGGGTACATCCCCTAGTCTTCTTTTAAATGCCATGCTGACTCTCTTACCAAGTTCAAATCGAAAAGGTCTG
CGAAGACCTCGCCTCCCATCCTATTCTCTCTACCGCGCCTGCCGTCGACGCAATCGGATTCTCCCAAGGCGGCCAATTCTTGCGCGGTTACATA
TCCCGCTGCAATTCTCCACCCATCCGCTCTCTCCTGACCTTCGGTTCCCAACACAACGGCATTTCTGCCTTCCAAGCCTGTGGTCCTGCCGATT
TCCTCTGTCGTGGTGCTCAAACTCTTTTGCGATCCAACACCTGGTCAACCTTTGTCCAATCTCGTCTCGTACCCGCTCAATACTTCAGAGATCC
GGAAAACCTAGACTCTTACCTTGAATATTCCAATTTCCTTGCCGACATCAATAATGAGCGCGTTCTCAAGAACCAAACATATAAATCCAACATG
GAAAAATTGGAACGATTCGTAATGTATGTCTTTGAAGATGATACAACTGTCATCCCTAAGGAAAGTGGATGGTGGGCTGAAGTCAACGGCACGG
AAGTTACACCACTGAAAGAAAGAGCCATTTATAAGGAAGATTGGCTAGGTTTAAAGACATTGGATGAGGCCGGAAAATTAGTTTTCGAAACCAT
TCCAGGGGGACATATGACGTTAGGAGAGGAGATGCTAGAGAAGGCTTTCAAAGAGTACTTTGGCCCAGCAGGGAAGAAATTTGGGG

>Bcin16g03460(MLST7), partial sequence [organism=Botrytis pseudocinerea, strain D11_T_E01]
CATGAACCTCTTAATTTGAACTCTTCATTAATCGCAAGTGATGAACCCTATTTTGCCCAGAGGATAGTTACAAGACGTATATCATTAGTCGAGA
ACCACTCATGATATATATTGACGGATTTTTGAAAGCGAATGAAAGTAAACATCTGGTTGATGTTAGGTATGTTACTTATTCTGATGAAATGAAC
AAGAGAAACTGATGAGATAGTGAACCGCTTTATGAACCGTCTACTGTTTCTCACGGACAGGAAGTTACCATTGATACTTCAGTTCGAAATTCGG
AAGTGGCGGTTTTAGAGAGGGATGAGGTGGTCAGGTGTATTGAGCATAGAGCGAGGGCATTTCAGGGGTGGAGGGATGAGATGGGGATTGAGAA
GTTGAGGACGCAGAGGTATGGGGTTGGAGGACATTATGGGATGCATTTGTAAGTTTTGGGGGATTGACGAAGGCTTTTGTCTATTTCCTATCAG
TACGGATCTTAAAAGAAAGATAGCATGAACACGAGGGCTAACAACTAGGAAATAGCGATTGGAGCGGAGGTAAACGTGGCATAGACCGATTTAG
TACTTTCATGGTCTATGTCGACGTATCTTCTGATATCGAAGGTGGAGGAACGGAATTCCCACGTATTGTGGGACCACAAGGAGGAAGGTGGGAG
GAATTCCTGGAAACTACGGAAGCATTGGATCCAAGAACTGGAAAAAAATGTACAGTAGAAGGGGTGACATTCAAACCAATCAAGGGAAATGCCGT
ATTCTGGGAAAATACTGACAACAACGGAGGGGATATGAT

>Bcin12g03020(MLST8), partial sequence [organism=Botrytis pseudocinerea, strain D11_T_E01]
TCGATTGGCTGCGAAGAAAACTGCGCAGCACCGAGTCACCAAAAGCACGAATAAACCGTCGGCAAGAGAAATATTCTCTGAACCACAACATGAC
ACGAGCGCGGAAGAGTATATTGGGCGAGAAGCTTCATCAAGAGCACCAAAGCGACAACGAGTCGATGATAATTATAACTCTTACGGTGGAAGAA
ATGAAAATACAGCAGCTTATGCTTCCGGAAAACTTCCATCTGGAAGTATAAATGTTGGTGGAGGTAGGAAGACCACTTTTCAAGAAGAACCTCG
AACGGCATTTGTCGCTGGCAAGTTGCCCCCTGGAAGTATCAACATCGGTGGAAAGAAGGCTACCCAAATCGAGGACGAAGGAAGAGCAGCTTAT
GCTTCCGGGAAATTGCCCCCAGGAAGTATCAGCTCTGGCGCAAAGAAGGCCATTTCATTCCAAGATGAAACGAGACCGGCCTATGTCTCTGGAA
AGCTCCCACATGGCAGTATCGATGGTATGCGAAACCGCGAAATGGCTGCCGTCCACCGCGAAATTGCTGAGGGTGGGAGGAAACCAGGCCAGGT
TGTCTCTTCTCTATTCACATTCAATCCTACATCAAAGAAAACTTTCGATGAATCCGAAGAACAAGCGGAACCTGCAAAACCATCCAATGCGCCT
TTGACAGAGGAAATGGAGACATTCACCAATCTAGGATTATCGAGAAGGCTTGCAGCCCATCTATCGACTAAACTCGATATGAAAGCCCCGACCG
CCATTCAAAAAGCATCTGTGCAGCAGTTGGTATCGGACGATAGCGATGCTTTCATACAAGCAGAGACTGGATCTGGAAAAACTTTGGCATATCT
ACTACCTATAGTCGAGCGAATATTAGCATTGAGTGAGAATGGCGTACAAA

>Bcin02g07770(MLST9), partial sequence [organism=Botrytis pseudocinerea, strain D11_T_E01]
CAGCTTTCCCTTTCGGTCTTGGCATCTACAGTCATTGCCATGCCTACACCATCACAACTTGAGTCTCGGGCCGTTATCGATTCCGATGCCGTTG

TAGGATTTGCCGAAGCTGTTCCCAGTGGGACCGTAGGAACAGTTTATGAGGCATATAAACCATTCCTTAAAGTCGTAAATGGATGCGTACCATT
CCCTGCCGTCGATGCATCGGGTAACACAGGGTATGTCCTTATACCTTTCTCTTCCTCACGATTGCTATTGAGTCTCTAACATATTTAGTGGTG
GTTTGTCACCAACTGGCAGTAGCAATGGTGGTTGCAGCAGCAGTACCGGTCAAGTATATGTTCGAGGAGGACAAAGCGGATCAAACTACGCTAT
CATGTACTCCTGGTAAGTTCTCTCTAAACTTCTCCTTATAGATCCAATATCTAACAGAATCTTAGGTACATGCCAAAGGACGAGCCCTCAACCG
GTATTGGTCACCGTCACGATTGGGAAGGTGTAATCGTCTGGCTCTCCAGCGCCACCGCCACAACTGCCGACAACATCTTAGCTGTTTGTCCTTC
GGCCCACGGAGGCTGGGATTGTTCCACCGATGGTTATTCCCTTTCTGGTACCAGCCCTCTTATCAAGTACGAAAGTATCTGGCCCGTCGACCAT
TCAATGGGTCTTACTAGTACTGTTGGTGGACAACAACCTATGATTGCTTGGGAGTCTTTACCTACTGCTGCTCAAACTGCTCTTGAGAACACCG
ATTTCGGTGCTGCGAATGTTCCATTTATTCCAGCTGTTTTCACAAACAATCT

>Bcin04g02090(MLST10), partial sequence [organism=Botrytis pseudocinerea, strain D11_T_E01]
GCGGAGGATGATATGGCAAAGTCTATGATTACCAAAGCATTTGTAGGCATGAGTAGTAAACTCTCAAATATGACTATGAATGATGTTTACAAGC
CCTACATCCATGTAAGAAATGTAGAATAGAGAGATCAATAACTGGAACTAATATCGTTTGTAGGCTTTCAAGTTACTTACGCAGTTCAACCCAA
TCACTACAGCTATTGCCGAATCCCCACTATTTCAAATGGCTGTCTCAGCAAATACCATCGAAAAGTACACGCTGCTAGGCCCTTTCTTCAGAAT
ATCTCCTCTGCAACAGGAAGTTACCAGGGAATACTTCAGTGCACCAAAGACGATAGATAGGCGACACATTGCCACATCTCAAGATGCGTTACGA
CTGACCTTACAAACCCATCAAAAAGATTTACTCGATATCATCAACCACTTTGTTCGAGCAAGTCCAATCGCCAAAAGCAAAACCTTGGATTGGT
TCGCCTACATTGTGAATCAAAATCACAAGCGTCGAGCACTTCAGGTAGACCCGAAAGAAGTATCTTCTGATGGCTTTATGCACAATGTCACTGT
CGTTCTAGATGGTCTTTGTGAGCCATTCATGGATACCACACTCCCGAAAATTTCGAAGATTGATATTGATTATCTAAGACGTGCGCCTCGTGTA
GATATCAAGGACGAGACCAAGTTGAACGCCGATGAGAAGGCTTCTGAGAAGTACTATGAGGACACGGTTCCTGGCACTTCCAATTTCATCTCTG
AGGTGTTCTTTCTGACATTGGCTGCTCATCATTATGGTAGTGAAGCTCTTAATGCCACGCACAAGAGTCTGGAGGAAGACATCAAATATATTCA
AAAGCAATTGACTGCCGT
TGAAGCA

>Bcin01g07220(MLST1), partial sequence [organism=Botrytis pseudocinerea, strain D12_E_cal10]
ATGGCATATCTTGTTTCTTTTCGCATTATGCTTTGGAACGTGCTGGACAAGCTTGGGTTAGTGCTCTGCGTGTAGAAGCACTAAAGAAGATTCT
TGCACAACCGAAGTCATGGTTTGAGGAATCCAGGAATTCACCTGGCCGATTGAACGAAGTTTTGGATAGGAACTCTGAGGAAATGCGTAATCTC
GTTGGCCGCTTTGTTGGTATTGTATTCACAGCATTTTTTATGTTATTGATATCAATCATTTGGGCTTTCGTGAATACGTGGAAGCTGACATTAG
TCTCAATGGCAACTGGGCCAGTTATATACGCTGTCACCAAAACGTTCAATCGCGTGAGTGGAAAATGGGAAAACAAGTGCAACTACGCATCTGA
AATGACCACTAGCATATTTTCAGAGACGTTCTCCAACATCAAAGTGGTTCGGGCTTTTACTCTGGAAACTTACTTTGCGACAAAACACACCAAA
GCTACAGAAGAACTTTATAAAGTTGGACTAATACGAGCAAACTACTCGGGATTGCTGTGGGGATTGACAGATGCGATGTCATTCTTCATCACTG
CAACTATCTTTTATTATGCCACGGTTCTCATTACCAAGAGAGAGATCAGTATCACGACTGCACTACAGACTGTCAATCTTCTATTATTTGGTAT
TTCTAATAGTACGAATATGCTGGCCATGATACCACAAATCAACTCTCTCGCGTTACAGCTACGCATATGCTTGCATTAGCCAATCTCGATTCAT
CTTCCTCCCACGAAAATCAAGGAACCGAACGACTTTCGACAATCTTTCCAATCAAATTCAACCGTCTCTCATTCACATATCCTAATCGTCCTGA
AAAACGAACGATATCATCCTTTTCTCTTTCCCTGATTCCTAACACAACAACTGCACTTGTCGGACCCTCCGGCTCCGGAAAATCTACAATAGCT
GCTCTGCTCATTGGCCTCTATCCACCAGATACTTCAACACCTCCACCGTTGACATTCAATCGCGTCTCCATAAGTAACTGTCACATCCCGTCTC
TCCGGGCTTCTGTCTCACTCGTCCCACAAATACCGATTCTATTTCCAGCTACCATTCTCCATAACATCATTTATGGGCTCCCAGAATCTTCTCC
TTGTGCTAATCTTCCATCTGCTATTGTATCAGCCAAAGATGCTGGGATCCATGAGTTTATCACATCGCTTCCACAAGGTTATAATGCTAT

>Bcin05g07690(MLST2), partial sequence [organism=Botrytis pseudocinerea, strain D12_E_cal10]
TCACCACTATCACCAAGTCCTTTTCTGCACTTTCCTCATCAAAAACGAACGAGAACATCATCAATAAATACTTTGCTGAAGTAATTGGGTTTTA
TTTTGGCCAGGATGCCTTCTCTCTACGACAGCAAGCCTTCGATGATATGTTGTGGGTAGTTCTTGGTTGGTTGGATACTGTCAAATTCATTGAT
TTACATTCTGAATTGCACTATTCAAATGACTCTCAGCCAGAATGGTACGGACAACAATATAAACCTGCATTTGCACATCGAGTGCGACTATTTT
GGGAATTGGCGTCACAAGGATGGGATACTACTCTCTGTGGTGGTGGGATGATATGGTCACCATACCTTACTCCATACAAGAACGCAATTACCAA
TGAACTCTATATCGCAGCTTCGATATCGATGTACCTCTATTTCCCTGGAGATGACAATCAATCCCCATTTATGCTTTCCAACCCTTCATATCCA
CCTCGCGATCCGAAATATCTACAGGCAGCTGTTGATGCTTACAAATGGCTGAATGGTTCCAATATGACGGATTTACAAGGATTATATGTCGACG
GATATCATATCTCGAATCTTTCTGGCGGTGAAAACACCCATTGCGATTCTAGAAATGAGATGGTATATACCCACAATCAAGGTGTTTTGCTTAC
TGGACAACGTGGTTTGTATGACGCAACCGCCGCACGATCATACCTTGTGGATGGCCACAAACTCATCGCGAATGTTATTAACGCCACAGGCTAT
GATCTGAAACACGATGTTGTTATCTCACCGCCACCCAAAGATGGTTCCGCATTGGCAAAATGGTTTGGCCTGGGTAGGAATGGAATACTGGAAG
AAGGATGCGATTCAAGTGCTTCGTGTTCTCAAAATGGACAAACTTTCAAAGGCATATTCTTTCATCACTTGATTGCATTCTGTAGTGATTTGCC
AGGGGAGCCTATTGCAGGGACGAAAGAAAGCCTGGAACTCGACAGAGAGTGGCATTCTGACAAATGCTCGCAGTATACAAAATG

>Bcin06g01710(MLST3), partial sequence [organism=Botrytis pseudocinerea, strain D12_E_cal10]
GTGAGTCCGGCTTTTGTATCTGAGCGTTAAGATAGACACTGATATACCAAGGCAATCACCTAATTTCATGGCTTTCTTCCCTGCATCATCTACC
TACCCTGGAATGCTTGGAGAATTATACTCCGCAGCTTTCACAGCTCCTGCTTTCAATTGGATCTGTTCCCCTGCTGTGACAGAATTGGAGACGG
TTGTAATGGATTGGCTGGCCAAGCTTCTCAATCTCCCAGACTGTTATTTGTCTTCGACTCATGGTGGTGGTGTTATCCAAGGATCAGCCTCGGA
AGCTATCGTTACCGTTATGGTTGCTGCCCGCGATAAATATCTTCGTGAAACTACTGAAGGTCTGTCAGGCATTGAACTCGAGGGTGCGATTGCA
TATAAGAGGAGTAAGCTAGTTGCACTAGGAAGCGAAATGGCACACAGCTCCACGCAGAAAGCAGCGCAGATAGCTGGCGTTAGATTCCGATCGA
TTCCAGTACTCGCATCCAATGATTTCGCCATGACGGGTGATGATCTAGAGAAGGTATTGAAAGAATGCAAATCTCAAGGATTGGAACCCTTCTA

TCTAACTTCGACTTTGGGAACAACATCTACATGTGCAGTTGATGACTTCGCATCTATCGCAACAGTACTTTCGAAATATGCACCTCCAGATGTT
GCAGGCGAGATATGGGTTCACGTCGATGCTGCTTATGCAGGCGCAGCTTTGGTTTGCCCTGAATACCATCATCTAACATCGTCCTTCCAGCATT
TTCATTCCTTCGATATGAACATGCACAAATGGCTTTTGACAAATTTCGACGCTTCTTGTCTATATGTCAAGAAACGCAAAGATCTGATCGATGC
ACTCTCTATAACACCAAGTTATCTTCGCAACGAGTTTTCAGAGAGTGGACTCGTGACCGACTATCGGGACTGGCAAATTCCTCTCGGAAGACGC
TTCCGAAGCTTAAAGATTTGGTTTGTCCTCAGAACCTAC

>Bcin09g03030(MLST4), partial sequence [organism=Botrytis pseudocinerea, strain D12_E_cal10]
ACCCTTCAATCCCACCGCAGCTCCTATACCGAAAGCGAGCATCCTACCAATTCTCCTTCCACCTGCGACTTTAAGACCATTGGCTTTTCGCACT
TTCACAAAAAGCATAGTTTAACATTGACGTCGTCCGCATTACAAGTGTTGGCTACTTTTATTGGAAAGCATTGTGGGACAGGATGGAGGGAAGA
AGGACTGGCAGAGAGAGTTTTAGAGGAGGTCGCCAAGAGTTGGAAGAATAGGAGTGGTGGTGTTATTGTCGAGGGCGAGGGAACGGAATTGAAG
GAGATTCTGAAAGCTTTGGAAGGGAATATGAGTGGTGGAAGGATAGTCGTAGGAAGAGAGCTAAGCCGGCAGAACAGTTTAGTACTGGGATCAT
CACAATATGGAGAGGTCAATCATACAAGACTTGGGCTACGGCCAGGGAATATACCCAGAGAGGATAGTCAGTCAAGTTTGGGAATGTCAACGTT
GGAGGTCAATGACGAGGAAGATGAGGATGGCCTGATGGATCCAAGGAGGTGGTTGAAAGTCATTGATGCATTTGAGCAACCCCGACTGGTGTAT
AATGTTGCTAAGAAGCACTTTGATAGGTATGTTTCAATGATAAAATTTTATCTGAATCAACTAACTCAGTACAGAGATACCTCCAAACCTTCAT
TGTTCCCACCTGCGTCTCATAAAACACTCCTCTTCCAAAACCGCTATAATGTTATCCATCAACGTCTCCTTCGCAATGAATCTTTTCAAACGCC
CGCTTTTCAAGGTGGCAAAGCTTCCTTCAACGCAGCACGTCCGCCATTACCACTCAACAACAATCATACAAATTAACGCCGATAGCTAATCTTC
TCGGTCGCAATCGCAGCTCTCATATGCTTCTCGGCCTCCTCAGCATTTCACCCACTGGTACCCTCGCCATCAATGACCTGACGGGCAGCATCGC
TCTCGATCTTACACACGCAGCAGCCATTCC

>Bcin11g01310(MLST5), partial sequence [organism=Botrytis pseudocinerea, strain D12_E_cal10]
ACTGACATGGACTTCATGTGGAACCGGCGTACAATGCGCAACGCTTGAAGTTCCGCTCGAATATGGCGATGCAACGTCAACAGCGAAAGCCAGT
ATTGCGCTTGCTCGTTATCCTGCCACTGTTGCCGCGAGCAAGAAGCTCGGGTCTCTCTTGATAAATCCCGGTGGACCCGGTGCCACTGGTGTTG
GCTTTGTGCAGTCTGGAGCCGGTGCCGCCATCTCGACACTGAGTGGTGGATTATACGATATCATCGGATGGGATCCACGTGGAACCGGTGCTTC
CGCTCCTATTTTGGAATGTTTTCCAAATGCCAGTGCGGAGTATGATTTTAACAACGCGTTTCCATCTGCTCCCAATCTCTGGCTCGGACAATTT
TCAAATGCCAGCGCAAATTCTGCCGTTAGCTCTGCTATCACATCTTTTGACACTTCTGTCGCTGCTCTTGCAAAAGCTTGTGTAGCTCAGAAGT
CCCCCGCTCTTTACACCTCAACAGCAGCATATGTTGCTCGAGACATGGCAGCGATAGTCGATGCCTTGGATGGAACCTCTGCAAAACTCAACTA
CTGGGGTTTCTCATATGGAACTATTTTCCTTGCCGAGTTTATCCAAACTTTCCCAGGCCGCGTGGGAAGAGTTCTTGCCGATGGGGTTTTCGAC
GCAAAGGCAAATGCACTCACATACGTTAGCCAACTTCCCAACGATCAACTCAGTGTTCGTGCTTCGTTGAACGATTTTGCAGCTTTCTGTACCA
CCGCCGGTAGTAAAGGTTGCTCTTTTGCCACCGCCCCTACTGGAACCACCGGTACCGTTGCTACCAGACTGGACAACATAATGAAGGATATGTT
CCTCAATCCCATTGTTGCTTCGGGCTTAAGCATCA

>Bcin15g03910(MLST6), partial sequence [organism=Botrytis pseudocinerea, strain D12_E_cal10]
GCCAAAACACAAAATCATCCAACGATGAAGATGATACTCCACTGCCCTTAATTATCTGGCACGGACTCGGCGATAATTACAAAGCGGATGGTCT
TGCGCAAGTTGGAAAGCTAGCTGAAGCTATTCATCCTGGGACTTTTGTCTATAATATTCATGTAGATGAGGATGCATCTGCAGATAGGACAGCT
ACCTTCTTTGGAAATCTCACTCGTGGGTACATCCCCTAGTCTTCTTTTAAATGCCATGCTGACTCTCTTACCAAGTTCAAATCGAAAAGGTCTG
CGAAGACCTCGCCTCCCATCCTATTCTCTCTACCGCGCCTGCCGTCGACGCGATCGGATTCTCCCAAGGCGGCCAATTCTTGCGCGGTCACATA
TCCCGCTGCAATTCTCCACCCATCCGCTCTCTCCTGACCTTCGGTTCCCAACACAACGGCATTTCTGCCTTCCAAGCCTGTGGTCCTGCCGATT
TCCTCTGTCGTGGTGCTCAAACTCTTTTGCGATCCAACACCTGGTCAACCTTTGTCCAATCTCGTCTCGTACCCGCTCAATACTTCAGAGATCC
GGAAAACCTAGACTCTTACCTTGAATATTCCAATTTCCTTGCCGACATCAATAATGAGCGCGTTCTCAAGAACCAAACATATAAATCCAACATG
GAAAATTGGAACGATTCGTAATGTATGTCTTTGAAGATGATACAACTGTCATCCCTAAGGAAAGTGGATGGTGGGCTGAAGTCAACGGCACGGA
AGTTACACCACTGAAAGAAAGAGCCATTTATAAGGAAGATTGGCTAGGTTTAAAGACATTGGATGAGGCCGGAAAATTAGTTTTCGAAACCATT
CCAGGGGACATATGACGTTAGGAGAGGAGATGCTAGAGAAGGCTTCAAAGAGTACTTTGGCCCAGCAGGGAAGAAATTTGGG

>Bcin16g03460(MLST7), partial sequence [organism=Botrytis pseudocinerea, strain D12_E_cal10]
ATGAACCTCTTAATTTGAACTCTTCATTAATCGCAAGTGATGAACCCCTATTTTGCCCAGAGGATAGTTACAAGACGTATATCATTAGTCGAGA
ACCACTCATGATATATATTGACGGATTTTTGAAAGCGAATGAAAGTAAACATCTGGTTGATGTTAGGTATGTTACTTATTCTGATGAAATGAAC
AAGAGAAACTGATGAGATAGTGAACCGCTTTATGAACCGTCTACTGTTTCTCACGGACAGGAAGTTACCATTGATACTTCAGTTCGAAATTCGG
AAGTGGCGGTTTTAGAGAGGGATGAGGTGGTCAGGTGTATTGAGCATAGAGCGAGGGCATTTCAGGGGTGGAGGGATGAGATGGGGATTGAGAG
GTTGAGGACGCAGAGGTATGGGGTTGGAGGACATTATGGGATGCATTTGTAAGTTTTGGGGGATTGACGAAGGCTTTTGTCTATTTCCTATCAG
TACGGATCTTAAAAGAAAGATAGCATGAACACGAGGGCTAACAACTAGGAAATAGCGATTGGAGCGGAGGTAAGCGTGGCATAGACCGATTTAG
TACTTTCATGGTCTATGTCGACGTATCTTCTGATATCGAAGGTGGAGGAACGGAATTCCCACGTATTGTGGGACCACAAGGAGGAAGGTGGGAG
GAATTCCTGGAAACTACGGAAGCATTGGATCCAAGAACTGGAAAAAAATGTAACAGTAGAAGGGGTGACATTCAAACCAATCAAGGGAAATGCCG
TATTCTGGGAAAATACTGACAACAACGGGAGGGGATATGAT

>Bcin12g03020(MLST8), partial sequence [organism=Botrytis pseudocinerea, strain D12_E_cal10]
CGATTGGCTGCGAAGAAAACTGCGCAGCACCGAGTCACCAAAAGCACGAATAAACCGTCGGCAGAGAAATATTCTCTGAACCACAACATGACAC
GAGCGCGGAAGAGTATATTGGGCGAGAAGCTTCATCAAGAGCACCAAAGCGACAACGAGTCGATGATAATTATAACTCTTACGGTGGAAGAAAT

GAAAATACAGCAGCTTATGCTTCCGGAAAACTTCCATCTGGAAGTATAAATGTTGGTGGAGGTAGGAAGACCACTTTTCAAGAAGAACCTCGAA
CGGCATTTGTCGCTGGCAAGTTGCCCCCTGGAAGTATCAACATCGGTGGAAAGAAGGCTACCCAAATCGAGGACGAAGGAAGAGCAGCTTATGC
TTCCGGGAAATTGCCCCCAGGAAGTATCAGCTCTGGCGCAAAGAAGGCCATTTCATTCCAAGATGAAACGAGACCGGCCTATGTCTCTGGAAAG
CTCCCACATGGCAGTATCGATGGTATGCGAAACCGCGAAATGGCTGCCGTCCACCGCGAAATTGCTGAGGGTGGGAGGAAACCAGGCCAGGTTG
TCTCTTCTCTATTCACATTCAATCCTACATCAAAGAAAACTTTCGATGAATCCGAAGAACAAGCGGAACCTGCAAAACCATCCAATGCGCCTTT
GACAGAGGAAATGGAGACATTCACCAATCTAGGATTATCGAGAAGGCTTGCAGCCCATCTATCGACTAAACTCGATATGAAAGCCCCGACCGCC
ATTCAAAAAGCATCTGTGCAGCAGTTGGTATCGGACGATAGCGATGCTTTCATACAAGCAGAGACTGGATCTGGAAAAACTTTGGCATATCTAC
TACCTATAGTCGAGCGAATATTAGCATTAAGTGAGAATGGCGTACAAA

>Bcin02g07770(MLST9), partial sequence [organism=Botrytis pseudocinerea, strain D12_E_cal10]
CAGCTTTCCCTTTCGGTCTTGGCATCTACAGTCATTGCCATGCCTACACCATCACAACTTGAGTCTCGGGCCGTTATCGATTCCGATGCCGTTG
TAGGATTTGCCGAAGCTGTTCCCAGTGGGACCGTAGGAACAGTTTATGAGGCATATAAACCATTCCTTAAAGTCGTAAATGGATGCGTACCATT
CCCTGCCGTCGATGCATCGGGTAACACAGGGTATGTCCTTATACCTTTCTCTTCCTCACGATTGCTATTGAGTCTCTAACATATTTTAGTGGTG
GTTTGTCACCAACTGGCAGTAGCAATGGTGGTTGCAGCAGCAGTACCGGCCAAGTATATGTTCGAGGAGGACAAAGCGGATCAAACTACGCTAT
CATGTACTCCTGGTAAGTTCTCTCTAAACTTCTCCTTATAGATCCAATATCTAACAGAATCTTAGGTACATGCCAAAGGACGAGCCCTCAACCG
GTATTGGTCACCGTCACGATTGGGAAGGTGTAATCGTCTGGCTCTCCAGCGCCACCGCCACAACTGCCGACAACATCTTAGCTGTTTGTCCTTC
GGCCCACGGAGGCTGGGATTGTTCCACCGATGGTTATTCCCTTTCTGGTACCAGCCCTCTTATCAAGTACGAAAGTATCTGGCCCGTCGACCAT
TCAATGGGTCTTACTAGTACTGTTGGTGGACAACAACCTATGATTGCTTGGGAGTCTTTACCTACTGCTGCTCAAACTGCTCTTGAGAACACCG
ATTTCGGTGCTGCGAATGTTCCATTTATTCCAGCTGTTTTCACAAACAATCT

>Bcin04g02090(MLST10), partial sequence [organism=Botrytis pseudocinerea, strain
D12_E_cal10]
CGGAGGATGATATGGCAAAGTCTATGATTACCAAAGCATTTGTAGGCATGAGTAGTAAACTCTCAAATATGACTATGAATGATGTTTACAAGCC
CTACATCCATGTAAGAAATGTAGAATAGAGAGATCAATAACTGGAACTAATATCGTTTGTAGGCTTTCAAGTTACTTACGCAGTTCAACCCAAT
CACTACAGCTATTGCCGAATCCCCACTATTTCAAATGGCTGTCTCAGCAAATACCATCGAAAAGTACACACTACTAGGCCCTTTCTTCAGAATA
TCTCCTCTGCAACAGGAAGTTACCAGGGAATACTTCAGTGCACCAAAGACGATAGATAGGCGACACATTGCCACATCTCAAGATGCGTTACGAC
TGACCTCACAAACCCATCAAAAAGATTTACTCGATATCATCAACCACTTTGTTCGAGCAAGTCCAATCGCCAAAAGCAAAACCTTGGATTGGTT
CGCCTACATTGTGAATCAAAATCACAAGCGTCGAGCACTTCAGGTAGACCCGAAAGAAGTATCTTCTGATGGCTTTATGCACAATGTCACTGTC
GTTCTAGATGGTCTTTGTGAGCCATTCATGGATACCACATTCTCGAAAATTTCGAAGATTGATATTGATTATCTAAGACGTGCGCCTCGTGTAG
ATATCAAGGACGAGACCAAGTTGAACGCCGATGAGAAGGCTTCTGAGAAGTACTATGAGGACACGGTTCCTGGCACTTCCAATTTCATCTCTGA
GGTGTTCTTTCTGACATTGGCTGCTCATCATTATGGTAGTGAAGCTCTTAATGCCACGCACAAGAGTCTGGAGAAAGACATCAAATATATTCAA
AAGCAATTGACTGCCGTTGAAGCA
>Bcin01g07220(MLST1), partial sequence [organism=Botrytis pseudocinerea, strain D12_KL_cal2]
ATGGCATATCTTGTTTCTTTTCGCATTATGCTTTGGAACGTGCTGGACAAGCTGGTTAGTGCTCTGCGTGTAGAAGCACTAAAGAAGATTCTTG
CACAACCGAAGTCATGGTTTGAGGAATCCAGGAATTCACCTGGCCGATTGAACGAAGTTTTGGATAGGAACTCTGAGGAAATGCGTAATCTCGT
TGGCCGCTTTGTTGGTATTGTATTCACAGCATTTTTTATGTTATTGATATCAATCATTTGGGCTTTCGTGAATACGTGGAAGCTGACATTAGTC
TCAATGGCAACTGGCCAGTTATATACGCTGTCACCAAAACGTTCAATCGCGTGAGTGGAAAATGGGAAAACAAGTGCAACTACGCATCTGAAAT
GACCACTAGCATATTTTCAGAGACGTTCTCCAACATCAAAGTGGTTCGGGCTTTTACTCTGGAAACTTACTTTGCGACAAAACACACCAAAGCA
CAGAAGAACTTTATAAAGTTGGACTAATACGAGCAAACTACTCGGGATTGCTGTGGGATTGACAGATGCGATGTCATTCTCATCACTGCAACTA
TCTTTTATTATGCCACGGTTCTCATTACCAAGAGAGAGATCAGTATCGCGACTGCACTACAGACTGTCAATCTTCTATTATTTGGTATTTCTAA
TAGTACGAATATGCTGGCCATGATACCACAAATCAACTCTTCTCGCGTTACAGCTACGCATATGCTTGCATTAGCCAATCTCGATTCATCTTCC
TCCCACGAAAATCAAGGAACCGAACGACTTTCGACAATCTTTCCAATCAAATTCAACCGTCTCTCATTCACATATCCTAATCGTCCTGAAAAAC
GAACGATATCATCCTTTTCTCTTTCCCTGATTCCTAACACAACAACTGCACTTGTCGGACCCTCCGGCTCCGGAAAATCTACAATAGCTGCTCT
GCTCATTGGCCTCTATCCACCAGATACTTCAACACCTCCACCGTTGACATTCAATCGCGTCTCCATAAGTAACTGTCACATCCGTCTCTCCGGC
TTCTGTCTCACTCGTCCACAAATACCGATTCTATTTCCAGCTACCATTCTCCATAACATCATTTATGGGCTCCCAGAATCTTCCCTTGTGCTAA
TCTTCCATCTGCTATTGTATCAGCCAAAGATGCTGGATCCATGAGTTTATCACATCGCTTCCACAAGGTTATAATGCTAT

>Bcin05g07690(MLST2), partial sequence [organism=Botrytis pseudocinerea, strain D12_KL_cal2]
TCACCACTATCACCAAGTCCTTTTCTGCACTTTCCTCATCAAAAACGAACGAGAACATCATCAATAAATACTTTGCTGAAGTAATTGGGTTTTA
TTTTGGCCAGGATGCCTTCTCTCTACGACAGCAAGCCTTCGATGATATGTTGTGGGTAGTTCTTGGTTGGTTGGATACTGTCAAATTCATTGAT
TTACATTCTGAATTGCACTATTCAAATGACTCTCAGCCAGAATGGTACGGACAACAATATAAACCTGCATTTGCACATCGAGCGCGACTATTTT
GGGAATTGGCGTCACAAGGATGGGATACTACTCTCTGTGGTGGTGGGATGATATGGTCACCATACCTTACTCCATACAAGAACGCAATTACCAA
TGAACTCTATATCGCAGCTTCGATATCGATGTACCTCTATTTCCCTGGAGATGACAATCAATCCCCATTTATGCTTTCCAACCCTTCATATCCA
CCTCGCGATCCGAAATATCTACAGGCAGCTGTTGATGCTTACAAATGGCTGAATGGTTCCAATATGACGGATTTACAAGGATTATATGTCGACG
GATATCATATCTCGAATCTTTCTGGCGGTGAAAACACCCATTGCGATTCTAGAAATGAGATGGTATATACCTACAATCAAGGTGTTTTGCTTAC
TGGACAACGTGGTTTGTATGACGCAACCGCCGCACGATCATACCTTGTGGATGGCCACAAACTCATCGCGAATGTTATTAACGCCACAGGCTAT
GATCTGAAACACGATGTTGTTATCTCACCGCCACCCAAAGATGGTTCCGCATTGGCAAAATGGTTTGGCCTGGGTAGGAATGGAATACTGGAAG

AAGGATGCGATTCAAGTGCTTCGTGTTCTCAAAATGGACAAACTTTCAAAGGCATATTCTTTCATCACTTGATTGCATTCTGTAGTGATTTGCC
AGGGGAGCCTATTGCAGGGACGAAAGAAAGCCTGGAACTCGACAGAGAGTGGCATTCTGACAAATGCTCGCAGTATACAAAAT

>Bcin06g01710(MLST3), partial sequence [organism=Botrytis pseudocinerea, strain D12_KL_cal2]
GGTGAGTCCGGCTTTTGTATCTGAGCGTTAAGATAGACACTGATATACCAAGGCAATCACCTAATTTCATGGCTTTCTTCCCTGCATCATCTAC
CTACCCTGGAATGCTTGGAGAATTATACTCCGCAGCTTTCACAGCTCCTGCTTTCAATTGGATCTGTTCCCCTGCTGTGACAGAATTGGAGACG
GTTGTAATGGATTGGCTGGCCAAGCTTCTCAATCTCCCAGACTGTTATTTGTCTTCGACTCATGGTGGTGGTGTTATCCAAGGATCAGCCTCGG
AAGCTATCGTTACCGTTATGGTTGCTGCCCGCGATAAATATCTTCGTGAAACTACTGAAGGTCTGTCAGGCATTGAACTCGAGGATGCGATTGC
ATATAAGAGGAGTAAGCTAGTTGCACTAGGAAGCGAAATGGCACACAGCTCCACGCAGAAAGCAGCGCAGATAGCTGGCGTTAGATTCCGATCG
ATTCCAGTACTCGCATCCAATGATTTCGCCATGACGGGTGATGATCTAGAGAAGGTATTGAAAGAATGCAAATCTCAAGGATTGGAACCCTTCT
ATCTAACTTCGACTTTGGGAACAACATCTACATGTGCAGTTGATGACTTCGCATCTATCGCAACAGTACTTTCGAAATATGCACCTCCAGATGT
TGCAGGCGAGATATGGGTTCACGTCGATGCTGCTTATGCAGGCGCAGCTTTGGTTTGCCCTGAATACCATCATCTAACATCGTCCTTCCAGCAT
TTTCATTCCTTCGATATGAACATGCACAAATGGCTTTTGACAAATTTCGACGCTTCTTGTCTATATGTCAAGAAACGCAAAGATCTGATCGATG
CACTCTCTATAACACCAAGTTATCTTCGCAACGAGTTTTCAGAGAGTGGACTCGTGACCGACTATCGGGACTGGCAAATTCCTCTCGGAAGACG
CTTCCGAAGCTTAAAGATTTGGTTTGTCCTCAGAACCTAC

>Bcin09g03030(MLST4), partial sequence [organism=Botrytis pseudocinerea, strain D12_KL_cal2]
ACCCTTCAATCCCACCGCAGCTCCTATACCGAAAGCGAGCATCCTACCAATTCTCCTTCCACCTGCGACTTTAAGACCATTGGCTTTTCGCACT
TTCACAAAAAAGCATAGTTTAACATTGACGTCGTCCGCATTACAAGTGTTGGCTACTTTTATTGGAAAGCATTGTGGGACAGGATGGAGGGAAG
AAGGACTGGCAGAGAGAGTTTTAGAGGAGGTCGCCAAGAGTTGGAAGAATAGGAGTGGTGGTGTTATTGTCGAGGGCGAGGGAACGGAATTGAA
GGAGATTCTGAAAGCTTTGGAAGGGAATATGAGTGGTGGAAGGATAGTCGTAGGAAGAGAGCTAAGCCGGCAGAACAGTTTAGTACTGGGATCA
TCACAATATGGAGAGGTCAATCATACAAGACTTGGGCTACGGCCAGGGAATATACCCAGAGAGGATAGTCAGTCAAGTTTGGGAATGTCAACGT
TGGAGGTCAATGACGAGGAAGATGAGGATGGCCTGATGGATCCAAGGAGGTGGTTGAAAGTCATTGATGCATTTGAGCAACCCCGACTGGTGTA
TAATGTTGCTAAGAAGCACTTTGATAGGTATGTTTCAATGATAAAATTTTATCTGAATCAACTAACTCAGTACAGAGATACCTCCAAACCTTCA
TTGTTCCCACCTGCGTCTCATAAAACACTCCTCTTCCAAAACCGCTATAATGTTATCCATCAACATCTCCTTCGCAATGAATCTTTTCAAACGC
CCGCTTTTCAAGGTGGCAAAGCTTCCCTTCAACGCAGCACGTCCGCCATTACCACTCAACAACAATCATACAAATTAACGCCGATAGCTAATCT
TCTCGGTCGCAATCGCAGCTCTCATATGCTTCTCGGCCTCCTCAGCATTTCACCCACTGGTACCCTCGCCATCAATGACCTGACGGGCAGCATC
GCTCTCGATCTTACACACGCAGCAGCCATTC

>Bcin11g01310(MLST5), partial sequence [organism=Botrytis pseudocinerea, strain D12_KL_cal2]
ACTGACATGGACTTCATGTGGAACCGGCGTACAATGCGCAACGCTTGAAGTTCCGCTCGAATATGGCGATGCAACGTCAACAGCGAAAGCCAGT
ATTGCGCTTGCTCGTTATCCTGCCACTGTTGCCGCGAGCAAGAAGCTCGGGTCTCTCTTGATAAATCCCGGTGGACCCGGTGCCACTGGTGTTG
GCTTTGTGCAGTCTGGAGCCGGTGCCGCCATCTCGACACTGAGTGGTGGATTATACGATATCATCGGATGGGATCCACGTGGAACCGGTGCTTC
CGCTCCTATTTTGGAATGTTTTCCAAATGCCAGTGCGGAGTATGATTTTAACAACGCGTTTCCATCTGCTCCCAATCTCTGGCTCGGACAATTT
TCAAATGCCAGCGCAAATTCTGCCGTTAGCTCTGCTATCACATCTTTTGACACTTCTGTCGCTGCTCTTGCAAAAGCTTGTGTAGCTCAGAAGT
CCCCCGCTCTTTACACCTCAACAGCAGCATATGTTGCTCGAGACATGGCAGCGATAGTCGATGCCTTGGATGGAACCTCTGCAAAACTCAACTA
CTGGGGTTTCTCATATGGAACTATTTTCCTTGCCGAGTTTATCCAAACTTTCCCAGGCCGCGTGGGAAGAGTTCTTGCCGATGGGGTTTTCGAC
GCAAAGGCAAATGCACTCACATACGTTAGCCAACTTCCCAACGATCAACTCAGTGTTCGTGCTTCGTTGAACGATTTTGCAGCTTTCTGTACCA
CCGCCGGTAGTAAAGGTTGCTCTTTTGCCACCGCCCCTACTGGAACCACCGGTACCGTTGCTACCAGACTGGACAACATAATGAAGGATATGTT
CCTCAATCCCATTGTTGCTTCGGGCTTAAGCATCA

>Bcin15g03910(MLST6), partial sequence [organism=Botrytis pseudocinerea, strain D12_KL_cal2]
GCCAAAACACAAAATCATCCAACGATGAAGATGATACTCCACTGCCCTTAATTATCTGGCACGGACTCGGCGATAATTACAAAGCGGATGGTCT
TGCGCAGTTGGAAAGCTAGCTGAAGCTATTCATCCTGGACTTTTGTCTATAATATTCATGTAGATGAGGATGCATCTGCAGATAGGACAGCTAC
CTTCTTTGGAAATCTCACTCGTGGGTACATCCCCTAGTCTTCTTTTAAATGCCATGCTGACTCTCTTACCAAGTTCAAATCGAAAAGGTCTGCG
AAGACCTCGCCTCCCATCCTATTCTCTCTACCGCGCCTGCCGTCGACGCAATCGGATTCTCCCAAGGCGGCCAATTCTTGCGCGGTTACATATC
CCGCTGCAATTCTCCACCCATCCGCTCTCTCCTGACCTTCGGTTCCCAACACAACGGCATTTCTGCCTTCCAAGCCTGTGGTCCTGCCGATTTC
CTCTGTCGTGGTGCTCAAACTCTTTTGCGATCCAACACCTGGTCAACCTTTGTCCGATCTCGTCTCGTACCCGCTCAATACTTCAGAGATCCGG
AAAACCTAGACTCTTACCTTGAATATTCCAATTTCCTTGCCGACATCAATAATGAGCGCGTTCTCAAGAACCAAACATATAAATCCAACATGGA
AAAATTGGAACGATTCGTAATGTATGTCTTTGAAGATGATACAACTGTCATCCCTAAGGAAAGTGGATGGTGGGCTGAAGTCAACGGCACGGAA
GTTACACCACTGAAAGAAAGAGCCATTTATAAGGAAGATTGGCTAGGTTTAAAGACATTGGATGAGGCCGGAAAATTAGTTTTCGAAACCATTC
CAGGGGGACATATGACGTTAGGAGAGGAGATGCTAGAGAAGGCTTTCAAAGAGTACTTTGGCCCAGCAGGGAAGAAATTTGGG

>Bcin16g03460(MLST7), partial sequence [organism=Botrytis pseudocinerea, strain D12_KL_cal2]
ATGAACCTCTTAATTTGAACTCTTCATTAATCGCAAGTGATGAACCCCTATTTTGCCCAGAGGATAGTTACAAGACGTATATCATTAGTCGAGA
ACCACTCATGATATATATTGACGGATTTTTGAAAGCGAATGAAAGTAAACATCTGGTTGATGTTAGGTATGTTACTTATTCTGATGAAATGAAC
AAGAGAAACTGATGAGATAGTGAACCGCTTTATGAACCGTCTACTGTTTCTCACGGACAGGAAGTTACCATTGATACTTCAGTTCGAAATTCGG

AAGTGGCGGTTTTAGAGAGGGATGAGGCGGTCAGGTGTATTGAGCATAGAGCGAGGGCATTTCAGGGGTGGAGGGATGAGATGGGGATTGAGAA
GTTGAGGACGCAGAGGTATGGGGTTGGAGGACATTATGGGATGCATTTGTAAGTTTTGGGGGATTGACGAAGGCTTTTGTCTATTTCCTATCAG
TACGGATCTTAAAAGAAAGATAGCATGAACACGAGGGCTAACAACTAGGAAATAGCGATTGGAGCGGAGGTAAACGTGGCATAGACCGATTTAG
TACTTTCATGGTCTATGTCGACGTATCTTCTGATATCGAAGGTGGAGGAACGGAATTCCCACGTATTGTGGGACCACAAGGAGGAAGGTGGGAG
GAATTCCTGGAAACTACGGAAGCATTGGATCCAAGAACTGGAAAAAATGTAACAGTAGAAGGGGTGACATTCAAACCAATCAAGGGAAATGCCG
TATTCTGGGAAAATACTGACAACAACGGGAGGGGATATGAT

>Bcin12g03020(MLST8), partial sequence [organism=Botrytis pseudocinerea, strain D12_KL_cal2]
CGATTGGCTGCGAAGAAAACTGCGCAGCACCGAGTCACCAAAAGCACGAATAAACCGTCGGCAAGAGAAATATTCTCTGAACCACAACATGACA
CGAGCGCGGAAGAGTATATTGGGCGAGAAGCTTCATCAAGAGCACCAAAGCGACAACGAGTCGATGATAATTATAACTCTTACGGTGGAAGAAA
TGAAAATACAGCAGCTTATGCTTCCGGAAAACTTCCATCTGGAAGTATAAATGTTGGTGGAGGTAGGAAGACCACTTTTCAAGAAGAACCTCGA
ACGGCATTTGTCGCTGGCAAGTTGCCCCCTGGAAGTATCAACATCGGTGGAAAGAAGGCTACCCAAATCGAGGACGAAGGAAGAGCAGCTTATG
CTTCCGGGAAATTGCCCCCAGGAAGTATCAGCTCTGGCGCAAAGAAGGCCATTTCATTCCAAGATGAAACGAGACCGGCCTATGTCTCTGGAAA
GCTCCCACATGGCAGTATCGATGGTATGCGAAACCGCGAAATGGCTGCCGTCCACCGCGAAATTGCTGAGGGTGGGAGGAAACCAGGCCAGGTT
GTCTCTTCTCTATTCACATTCAATCCTACATCAAAGAAAACTTTCGATGAATCCGAAGAACAAGCGGAACCTGCAAAACCATCCAATGCGCCTT
TGACAGAGGAAATGGAGACATTCACCAATCTAGGATTATCGAGAAGGCTTGCAGCCCATCTATCGACTAAACTCGATATGAAAGCCCCGACCGC
CATTCAAAAAGCATCTGTGCAGCAGTTGGTATCGGACGATAGCGATGCTTTCATACAAGCAGAGACTGGATCTGGAAAAACTTTGGCATATCTA
CTACCTATAGTCGAGCGAATATTAGCATTGAGTGAGAATGGCGTACAAA

>Bcin02g07770(MLST9), partial sequence [organism=Botrytis pseudocinerea, strain D12_KL_cal2]
CAGCTTTCCCTTTCGGTCTTGGCATCTACAGTCATTGCCATGCCTACACCATCACAACTTGAGTCTCGGGCCGTTATCGATTCCGATGCCGTTG
TAGGATTTGCCGAAGCTGTTCCCAGTGGGACCGTAGGAACAGTTTATGAGGCATATAAACCATTCCTTAAAGTCGTAAATGGATGCGTACCATT
CCCTGCCGTCGATGCATCGGGTAACACAGGGTATGTCCTTATACCTTTCTCTTCCTCACGATTGCTATTGAGTCTCTAACATATTTTAGTGGTG
GTTTGTCACCAACTGGCAGTAGCAATGGTGGTTGCAGCAGCAGTACCGGTCAAGTATATGTTCGAGGAGGACAAAGCGGATCAAACTACGCTAT
CATGTACTCCTGGTAAGTTCTCTCTAAACTTCTCCTTATAGATCCAATATCTAACAGAATCTTAGGTACATGCCAAAGGACGAGCCCTCAACCG
GTATTGGTCACCGTCACGATTGGGAAGGTGTAATCGTCTGGCTCTCCAGCGCCACCGCCACAACTGCCGACAACATCTTAGCTGTTTGTCCTTC
GGCCCACGGAGGCTGGGATTGTTCCACCGATGGTTATTCCCTTTCTGGTACCAGCCCTCTTATCAAGTACGAAAGTATCTGGCCCGTCGACCAT
TCAATGGGTCTTACTAGTACTGTTGGTGGACAACAACCTATGATTGCTTGGGAGTCTTTACCTACTGCTGCTCAAACTGCTCTTGAGAACACCG
ATTTCGGTGCTGCGAATGTTCCATTTATTCCAGCTGTTTTCACAAACAATCT

>Bcin04g02090(MLST10), partial sequence [organism=Botrytis pseudocinerea, strain D12_KL_cal2]
CGGAGGATGATATGGCAAAGTCTATGATTACCAAAGCATTTGTAGGCATGAGTAGTAAACTCTCAAATATGACTATGAATGATGTTTACAAGCC
CTACATCCATGTAAGAAATGTAGAATAGAGAGATCAATAACTGGAACTAATATCGTTTGTAGGCTTTCAAGTTACTTACGCAGTTCAACCCAAT
CACTACAGCTATTGCCGAATCCCCACTATTTCAAATGGCTGTCTCAGCAAATACCATCGAAAAGTACACACTGCTAGGCCCTTTCTTCAGAATA
TCTCCTCTGCAACAGGAAGTTACCAGGGAATACTTCAGTGCACCAAAGACGATAGATAGGCGACACATTGCCACATCTCAAGATGCGTTACGAC
TGACCTTACAAACCCATCAAAAAGATTTACTCGATATCATCAACCACTTTGTTCGAGCAAGTCCAATCGCCAAAAGCAAAACCTTGGATTGGTT
CGCCTACATTGTGAATCAAAATCACAAGCGTCGAGCACTTCAGGTAGACCCGAAAGAAGTATCTTCTGATGGCTTTATGCACAATGTCACTGTC
GTTCTAGATGGTCTTTGTGAGCCATTCATGGATACCACATTCTCGAAAATTTCGAAGATTGATATTGATTATCTAAGACGTGCGCCTCGTGTAG
ATATCAAGGACGAGACCAAGTTGAACGCCGATGAGAAGGCTTCTGAGAAGTACTATGAGGACACGGTTCCTGGCACTTCCAATTTCATCTCTGA
GGTGTTCTTTCTGACATTGGCTGCTCATCATTATGGTAGTGAAGCTCTTAATGCCACGCACAAGAGTCTGGAGAAAGACATCAAATATATTCAA
AAGCAATTGACTGCCGTTGAAGCA

>Bcin01g07220(MLST1), partial sequence [organism=Botrytis pseudocinerea, strain D13_E_IF04]
ATGGCATATCTTGTTTCTTTTCGCATTATGCTTTGGAACGTGCTGGACAAGCTTGGGTTAGTGCTCTGCGTGTAGAAGCACTAAAGAAGATTCT
TGCACAACCGAAGTCATGGTTTGAGGAATCCAGGAATTCACCTGGCCGATTGAACGAAGTTTTGGATAGGAACTCTGAGGAAATGCGTAATCTC
GTTGGCCGCTTTGTTGGTATTGTATTCACAGCATTTTTTATGTTATTGATATCAATCATTTGGGCTTTCGTGAATACGTGGAAGCTGACATTAG
TCTCAATGGCAACTGGGCCAGTTATATACGCTGTCACCAAAACGTTCAATCGCGTGAGTGGAAAATGGGAAAACAAGTGCAACTACGCATCTGA
AATGACCACTAGCATATTTTCAGAGACGTTCTCCAACATCAAAGTGGTTCGGGCTTTTACTCTGGAAACTTACTTTGCGACAAAACACACCAAA
GCTACAGAAGAACTTTATAAAGTTGGACTAATACGAGCAAACTACTCGGGATTGCTGTGGGGATTGACAGATGCGATGTCATTCTTCATCACTG
CAACTATCTTTTATTATGCCACGGTTCTCATTACCAAGAGAGAGATCAGTATCGCGACTGCACTACAGACTGTCAATCTTCTATTATTTGGTAT
TTCTAATAGTACGAATATGCTGGCCATGATACCACAAATCAACTCTTCTCGCGTTACAGCTACGCATATGCTTGCATTAGCCAATCTCGATTCA
TCTTCCTCCCACGAAAATCAAGGAACCGAACGACTTTCGACAATCTTTCCAATCAAATTCAACCGTCTCTCATTCACATATCCTAATCGTCCTG
AAAAACGAACGATATCATCCTTTTCTCTTTCCCTGATTCCTAACACAACAACTGCACTTGTCGGACCCTCCGGCTCCGGAAAATCTACAATAGC
TGCTCTGCTCATTGGCCTCTATCCACCAGATACTTCAACACCTCCACCGTTGACATTCAATCGCGTCTCCATAAGTAACTGTCACATCCCGTCT
CTCCGGGCTTCTGTCTCACTCGTCCCACAAATACCGATTCTATTTCCAGCTACCATTCTCCATAACATCATTTATGGGCTCCCAGAATCTTCTC
CTTGTGCTAATCTTCCATCTGCTATTGTATCAGCCAAAGATGCTGGGATCCATGAGTTTATCACATCGCTTCCACAAGGTTATAATGCTAT

```
>Bcin05g07690(MLST2), partial sequence [organism=Botrytis pseudocinerea, strain D13_E_IF04]
ACCACTATCACCAAGTCCTTTTCTGCACTTTCCTCATCAAAAACGAACGAGAACATCATCAATAAATACTTTGCTGAAGTAATTGGGTTTTATT
TTGGCCAGGATGCCTTCTCTCTACGACAGCAAGCCTTCGATGATATGTTGTGGGTAGTTCTTGGTTGGTTGGATACTGTCAAATTCATTGATTT
ACATTCTGAATTGCACTATTCAAATGACTCTCAGCCAGAATGGTACGGACAACAATATAAACCTGCATTTGCACATCGAGCGCGACTATTTTGG
GAATTGGCGTCACAAGGATGGGATACTACTCTCTGTGGTGGTGGGATGATATGGTCACCATACCTTACTCCATACAAGAACGCAATTACCAATG
AACTCTATATCGCAGCTTCGATATCGATGTACCTCTATTTCCCTGGAGATGACAATCAATCCCCATTTATGCTTTCCAACCCTTCATATCCACC
TCGCGATCCGAAATATCTACAGGCAGCTGTTGATGCTTACAAATGGCTGAATGGTTCCAATATGACGGATTTACAAGGATTATATGTCGACGGA
TATCATATCTCGAATCTTTCTGGCGGTGAAAACACCCATTGCGATTCTAGAAATGAGATGGTATATACCTACAATCAAGGTGTTTTGCTTACTG
GACAACGTGGTTTGTATGACGCAACCGCCGCACGATCATACCTTGTGGATGGCCACAAACTCATCGCGAATGTTATTAACGCCACAGGCTATGA
TCTGAAACACGATGTTGTTATCTCACCGCCACCCAAAGATGGTTCCGCATTGGCAAAATGGTTTGGCCTGGGTAGGAATGGAATACTGGAAGAA
GGATGCGATTCAAGTGCTTCGTGTTCTCAAAATGGACAAACTTTCAAAGGCATATTCTTTCATCACTTGATTGCATTCTGTAGTGATTTGCCAG
GGGAGCCTATTGCAGGGACGAAAGAAAGCCTGGAACTCGACAGAGAGTGGCATTCTGACAAATGCTCGCAGTATACAAAAT

>Bcin06g01710(MLST3), partial sequence [organism=Botrytis pseudocinerea, strain D13_E_IF04]
GTGAGTCCGGCTTTTGTATCTGAGCGTTAAGATAGACACTGATATACCAAGGCAATCACCTAATTTCATGGCTTTCTTCCCTGCATCATCTACC
TACCCTGGAATGCTTGGAGAATTATACTCCGCAGCTTTCACAGCTCCTGCTTTCAATTGGATCTGTTCCCCTGCTGTGACAGAATTGGAGACGG
TTGTAATGGATTGGCTGGCCAAGCTTCTCAATCTCCCAGACTGTTATTTGTCTTCGACTCATGGTGGTGGTGTTATCCAAGGATCAGCCTCGGA
AGCTATCGTTACCGTTATGGTTGCTGCCCGCGATAAATATCTTCGTGAAACTACTGAAGGTCTGTCAGGCATTGAACTCGAGGGTGCGATTGCA
TATAAGAGGAGTAAGCTAGTTGCACTAGGAAGCGAAATGGCACACAGCTCCACGCAGAAAGCAGCGCAGATAGCTGGCGTTAGATTCCGATCGA
TTCCAGTACTCGCATCCAATGATTTCGCCATGACGGGTGATGATCTAGAGAAGGTATTGAAAGAATGCAAATCTCAAGGATTGGAACCCTTCTA
TCTAACTTCGACTTTGGGAACAACATCTACATGTGCAGTTGATGACTTCGCATCTATCGCAACAGTACTTTCGAAATATGCACCTCCAGATGTT
GCAGGCGAGATATGGGTTCACGTCGATGCTGCTTATGCAGGCGCAGCTTTGGTTTGCCCTGAATACCATCATCTAACATCGTCCTTCCAGCATT
TTCATTCCTTCGATATGAACATGCACAAATGGCTTTTGACAAATTTCGACGCTTCTTGTCTATATGTCAAGAAACGCAAAGATCTGATCGATGC
ACTCTCTATAACACCAAGTTATCTTCGCAACGAGTTTTCAGAGAGTGGACTCGTGACCGACTATCGGGACTGGCAAATTCCTCTCGGAAGACGC
TTCCGAAGCTTAAAGATTTGGTTTGTCCTCAGAACCTAC

>Bcin09g03030(MLST4), partial sequence [organism=Botrytis pseudocinerea, strain D13_E_IF04]
ACCCTTCAATCCCACCGCAGCTCCTATACCGAAAGCGAGCATCCTACCAATTCTCCTTCCACCTGCGACTTTAAGACCATTGGCTTTTCGCACT
TTCACAAAAAAGCATAGTTTAACATTGACGTCGTCCGCATTACAAGTGTTGGCTACTTTTATTGGAAAGCATTGTGGGACAGGATGGAGGGAAG
AAGGACTGGCAGAGAGAGTTTTAGAGGAGGTCGCCAAGAGTTGGAAGAATAGGAGTGGTGGTGTTATTGTCGAGGGCGAGGGAACGGAATTGAA
GGAGATTCTGAAAGCTTTGGAAGGGAATATGAGTGGTGGAAGGATAGTCGTAGGAAGAGAGCTAAGCCGGCAGAACAGTTTAGTACTGGGATCA
TCACAATATGGAGAGGTCAATCATACAAGACTTGGGCTACGGCCAGGGAATATACCCAGAGAGGATAGTCAGTCAAGTTTGGGAATGTCAACGT
TGGAGGTCAATGACGAGGAAGATGAGGATGGCCTGATGGATCCAAGGAGGTGGTTGAAAGTCATTGATGCATTTGAGCAACCCCGACTGGTGTA
TAATGTTGCTAAGAAGCACTTTGATAGGTATGTTTCAATGATAAAATTTTATCTGAATCAACTAACTCAGTACAGAGATACCTCCAAACCTTCA
TTGTTCCCACCTGCGTCTCATAAAACACTCCTCTTCCAAAACCGCTATAATGTTATCCATCAACGTCTCCTTCGCAATGAATCTTTTCAAACGC
CCGCTTTTCAAGGTGGCAAAGCTTCCCTTCAACGCAGCACGTCCGCCATTACCACTCAACAACAATCATACAAATTAACGCCGATAGCTAATCT
TCTCGGTCGCAATCGCAGCTCTCATATGCTTCTCGGCCTCCTCAGCATTTCACCCACTGGTACCCTCGCCATCAATGACCTGACGGGCAGCATC
GCTCTCGATCTTACACACGCAGCAGCCATCC

>Bcin11g01310(MLST5), partial sequence [organism=Botrytis pseudocinerea, strain D13_E_IF04]
ACTGACATGGACTTCATGTGGAAACCGGCGTACAATGCGCAACGCTTGAAGTTCCGCTCGAATATGGCGATGCAACGTCAACAGCGAAAGCCAG
TATTGCGCTTGCTCGTTATCCTGCCACTGTTTGCCGCGAGCAAGAAGCTCGGGTCTCTCTTGATAAATCCCGGTGGACCCGGTGCCACTGGTGT
TGGCTTTGTGCAGTCTGGAGCCGGTGCCGCCATCTCGACACTGAGTGGTGGATTATACGATATCATCGGATGGGATCCACGTGGAACCGGTGCT
TCCGCTCCTATTTTGGAATGTTTTCCAAATGCCAGTGCGGAGTATGATTTTAACAACGCGTTTCCATCTGCTCCCAATCTCTGGCTCGGACAAT
TTTCAAATGCCAGCGCAAATTCTGCCGTTAGCTCTGCTATCACATCTTTTGACACTTCTGTCGCTGCTCTTGCAAAAGCTTGTGTAGCTCAGAA
GTCCCCGCTCTTTACACCTCAACAGCAGCATATGTTGCTCGAGACATGGCAGCGATAGTCGATGCCTTGGATGGAACCTCTGCAAAACTCAACT
ACTGGGGTTTCTCATATGGAACTATTTTCCTTGCCGAGTTTATCCAAACTTTCCCAGGCCGCGTGGGAAGAGTTCTTGCCGATGGGTTTTCGAC
GCAAAGGCAAATGCACTCACATACGTTAGCCAACTTCCCAACGATCAACTCAGTGTTCGTGCTTCGTTGAACGATTTTGCAGCTTTCTGTACCA
CCGCCGGTAGTAAAGGTTGCTCTTTTGCCACCGCCCCTACTGGAACCACCGGTACCGTTGCTACCAGACTGGACAACATAATGAAGGATATGTT
CCTCAATCCATTGTTGCTTCGGGCTTAAGCATCA

>Bcin15g03910(MLST6), partial sequence [organism=Botrytis pseudocinerea, strain D13_E_IF04]
GCCAAAGCACAAAATCATCCAACGATGAAGATGATACTCCACTGCCCTTAATTATCTGGCACGGACTCGGCGATAATTACAAAGCGGATGGTCT
TGCGCAAGTTGGAAAGCTAGCTGAAGCTATTCATCCTGGGACTTTTGTCTATAATATTCATGTAGATGAGGATGCATCTGCAGATAGGACAGCT
ACCTTCTTTGGAAATCTCACTCGTGGGTACATCCCCTAGTCTTCTTTTAAATGCCATGCTGACTCTCTTACCAAGTTCAAATCGAAAAGGTCTG
CGAAGACCTCGCCTCCCATCCTATTCTCTCTACCGCGCCTGCCGTCGACGCAATCGGATTCTCCCAAGGCGGCCAATTCTTGCGCGGTTACATA
TCCCGCTGCAATTCTCCACCCATCCGCTCTCTCCTGACCTTCGGTTCCCAACACAACGGCATTTCTGCCTTCCAAGCCTGTGGTCCTGCCGATT
```

TCCTCTGTCGTGGTGCTCAAACTCTTTTGCGATCCAACACCTGGTCAACCTTTGTCCAATCTCGTCTCGTACCCGCTCAATACTTCAGAGATCC
GGAAAACCTAGACTCTTACCTTGAATATTCCAATTTCCTTGCCGACATCAATAATGAGCGCGTTCTCAAGAACCAAACATATAAATCCAACATG
GAAAAATTGGAACGATTCGTAATGTATGTCTTTGAAGATGATACAACTGTCATCCCTAAGGAAAGTGGATGGTGGGCTGAAGTCAACGGCACGG
AAGTTACACCACTGAAAGAAAGAGCCATTTATAAGGAAGATTGGCTAGGTTTAAAGACATTGGATGAGGCCGGAAAATTAGTTTTCGAAACCAT
TCCAGAGGGACATATGACGTTAGGAGAGGAGATGCTAGAGAAGGCTTTCAAAGAGTACTTTGGCCCAGCAGGGAAGAAATTTGGG

>Bcin16g03460(MLST7), partial sequence [organism=Botrytis pseudocinerea, strain D13_E_IF04]
ATGAACCTCTTAATTTGAACTCTTCATTAATCGCAAGTGATGAACCCCTATTTTGCCCAGAGGATAGTTACAAGACGTATATCATTAGTCGAGA
ACCACTCATGATATATATTGACGGATTTTTGAAAGCGAATGAAAGTAAACATCTGGTTGATGTTAGGTATGTTACTTATTCTGATGAAATGAAC
AAGAGAAACTGATGAGATAGTGAACCGCTTTATGAACCGTCTACTGTTTCTCACGGACAGGAAGTTACCATTGATACTTCAGTTCGAAATTCGG
AAGTGGCGGTTTTAGAGAGGGATGAGGTGGTCAGGTGTATTGAGCATAGAGCGAGGGCATTTCAGGGGTGGAGGGATGAGATGGGGATTGAGAA
GTTGAGGACGCAGAGGTATGGGGTTGGAGGACATTATGGGATGCATTTGTAAGTTTTGGGGGATTGACGAAGGCTTTTGTCTATTTCCTATCAG
TACGGATCTTAAAAGAAAGATAGCATGAACACGAGGGCTAACAACTAGGAAATAGCGATTGGAGCGGAGGTAAACGTGGCATAGACCGATTTAG
TACTTTCATGGTCTATGTCGACGTATCTTCTGATATCGAAGGTGGAGGAACGGAATTCCCACGTATTGTGGGACCACAAGGAGGAAGGTGGGAG
GAATTCCTGGAAACTACGGAAGCATTGGATCCAAGAACTGGAAAAAATGTAACAGTAGAAGGGGTGACATTCAAACCAATCAAGGGAAATGCCG
TATTCTGGGAAAATACTGACAACAACGGGAGGGGATATGAT

>Bcin12g03020(MLST8), partial sequence [organism=Botrytis pseudocinerea, strain D13_E_IF04]
CGATTGGCTGCGAAGAAAACTGCGCAGCACCGAGTCACCAAAAGCACGAATAAACCGTCGGCAAGAGAAATATTCTCTGAACCACAACATGACA
CGAGCGCGGAAGAGTATATTGGGCGAGAAGCTTCATCAAGAGCACCAAAGCGACAACGAGTCGATGATAATTATAACTCTTACGGTGGAAGAAA
TGAAAATACAGCAGCTTATGCTTCCGGAAAACTTCCATCTGGAAGTATAAATGTTGGTGGAGGTAGGAAGACCACTTTTCAAGAAGAACCTCGA
ACGGCATTTGTCGCTGGCAAGTTGCCCCCTGGAAGTATCAACATCGGTGGAAAGAAGGCTACCCAAATCGAGGACGAAGGAAGAGCAGCTTATG
CTTCCGGGAAATTGCCCCCAGGAAGTATCAGCTCTGGCGCAAAGAAGGCCATTTCATTCCAAGATGAAACGAGACCGGCCTATGTCTCTGGAAA
GCTCCCACATGGCAGTATCGATGGTATGCGAAACCGCGAAATGGCTGCCGTCCACCGCGAAATTGCTGAGGGTGGGAGGAAACCAGGCCAGGTT
GTCTCTTCTCTATTCACATTCAATCCTACATCAAAGAAAACTTTCGATGAATCCGAAGAACAAGCGGAACCTGCAAAACCATCCAATGCGCCTT
TGACAGAGGAAATGGAGACATTCACCAATCTAGGATTATCGAGAAGGCTTGCAGCCCATCTATCGACTAAACTCGATATGAAAGCCCCGACCGC
CATTCAAAAAGCATCTGTGCAGCAGTTGGTATCGGACGATAGCGATGCTTTCATACAAGCAGAGACTGGATCTGGAAAAACTTTGGCATATCTA
CTACCTATAGTCGAGCGAATATTAGCATTGAGTGAGAATGGCGTACAAA

>Bcin02g07770(MLST9), partial sequence [organism=Botrytis pseudocinerea, strain D13_E_IF04]
ACAGCTTCCCTTTTCGGTCTTGGCATCTACAGTCATTGCCATGCCTACACCATCACAAACTTGAGTCTCGGGCCGTTATCGATTCCGATGCCGT
TGTAGGATTTGCCGAAGCTGTTCCAGTGGGACCGTAGGAACAGTTTATGAGGCATATAAACCATTCCTTAAAGTCGTAAATGGATGCGTACCAT
TCCCTGCCGTCGATGCATCGGGTAACACAGGGTATGTCCTTATACCTTTCTCTTCCTCACGATTGCTATTGAGTCTCTAACATATTTTAGTGGT
GGTTTGTCACCAACTGGCAGTAGCAATGGTGGTTGCAGCAGCAGTACCGGTCAAGTATATGTTCGAGGAGGACAAAGCGGATCAAACTACGCTA
TCATGTACTCCTGGTAAGTTTCTCTCTAAACTTCTCCTTATAGATCCAATATCTAACAGAATCTTAGGTACATGCCAAAGGACGAGCCCTCAAC
CGGTATTGGTCACCGTCACGATTGGAAGGTGTAATCGTCTGGCTCTCCAGCGCCACCGCCACAACTGCCGACAACATCTTAGCTGTTTGTCCTT
CGGCCCACGGAGGCTGGGATTGTTCCACCGATGGTTATTCCTTTCTGGTACCAGCCCTCTTATCAAGTACGAAAGTATCTGGCCCGTCGACCAT
TCAATGGGTCTTACTAGTACTGTTGGTGGACAACAACCTATGATTGCTTGGGAGTCTTTACCTACTGCTGCTCAAACTGCTCTTGAGAACACCG
ATTTCGGTGCTGCGAATGTTCCATTTATTCCAGCTGCTTTCACAAACAATCT

>Bcin04g02090(MLST10), partial sequence [organism=Botrytis pseudocinerea, strain D13_E_IF04]
CGGAGGATGATATGGCAAAGTCTATGATTACCAAAGCATTTTGTAGGCATGAGTAGTAAACTCTCAAATATGACTATGAATGATGTTTACAAGC
CCTACATCCATGTAAGAAATGTAGAATAGAGAGATCAATAACTGGAACTAATATCGTTTGTAGGCTTTCAAGTTACTTACGCAGTTTCAACCCA
ATCACTACAGCTATTGCCGAATCCCCACTATTTCAAATGGCTGTCTCAGCAAATACCATCGAAAAGTACACACTGCTAGGCCCTTTCTTCAGAA
TATCTCCTCTGCAACAGGAAGTTACCAGGGAATACTTCAGTGCACCAAAGACGATAGATAGGCGACACATTGCCACATCTCAAGATGCGTTACG
ACTGACCTTACAAACCCATCAAAAAGATTTACTCGATATCATCAACCACTTTGTTCGAGCAAGTCCAATCGCCAAAAGCAAAACCTTGGATTGG
TTCGCCTACATTGTGAATCAAAATCACAAGCGTCGAGCACTTCAGGTAGACCCGAAAGAAGTATCTTCTGATGGCTTTATGCACAATGTCACTG
TCGTTCTAGATGGTCTTTGTGAGCCATTCATGGATACCGCATTCTCGAAAATTTCGAAGATTGATATTGATTATCTAAGACGTGCGCCTCGTGT
AGATATCAAGGACGAGACCAAGTTGAACGCCGATGAGAAGGCTTCTGAGAAGTACTATGAGGACACGGTTCCTGGCACTTCCAATTTCATCTCT
GAGGTGTTCTTTCTGACATTGGCTGCTCATCATTATGGTAGTGAGGCTCTTAATGCCACGCACAAGAGTCTGGAGAAAGACATCAAATATATTC
AAAAGCAATTGACTGCCGTTGAAGCA

>Bcin01g07220(MLST1), partial sequence [organism=Botrytis pseudocinerea, strain N11_K_W15]
ATGGCATATCTTGTTTCTTTTCGCATTATGCTTTGGAACGTGCTGGACAAGCTTGGGTTAGTGCTCTGCGTGTAGAAGCACTAAAGAAGATTCT
TGCACAACCGAAGTCATGGTTTGAGGAATCCAGGAATTCACCTGGCCGATTGAACGAAGTTTTGGATAGGAACTCTGAGGAAATGCGTAATCTC
GTTGGCCGCTTTGTTGGTATTGTATTCACAGCATTTTTTATGTTATTGATATCAATCATTTGGGCTTTCGTGAATACGTGGAAGCTGACATTAG
TCTCAATGGCAACTGGGCCAGTTATATACGCTGTCACCAAAACGTTCAATCGCGTGAGTGGAAAATGGGAAAACAAGTGCAACTACGCATCTGA

```
AATGACCACTAGCATATTTTCAGAGACGTTCTCCAACATCAAAGTGGTTCGGGCTTTTACTCTGGAAACTTACTTTGCGACAAAACACACCAAA
GCTACAGAAGAACTTTATAAAGTTGGACTAATACGAGCAAACTACTCGGGATTGCTGTGGGATTGACAGATGCGATGTCATTCTTCATCACTGC
AACTATCTTTTATTATGCCACGGTTCTCATTACCAAGAGAGAGATCAGTATCGCGACTGCACTACAGACTGTCAATCTTCTATTATTTGGTATT
TCTAATAGTACGAATATGCTGGCCATGATACCACAAATCAACTCTTCTCGCGTTACAGCTACGCATATGCTTGCATTAGCCAATCTCGATTCAT
CTTCCTCCCACGAAAATCAAGGAACCGAACGACTTTCGACAATCTTTCCAATCAAATTCAACCGTCTCTCATTCACATATCCTAATCGTCCTGA
AAAACGAACGATATCATCCTTTTCTCTTTCCCTGATTCCTAACACAACAACTGCACTTGTCGGACCTCCGGCTCCGGAAAATCTACAATAGCTG
CTCTGCTCATTGGCCTCTATCCACCAGATACTTCAACACCTCCACCGTTGACATTCAATCGCGTCTCCATAAGTAACTGTCACATCCCGTCTCT
CCGGGCTTCTGTCTCACTCGTCCACAAATACCGATTCTATTTCCAGCTACCATTCTCCATAACATCATTTATGGCTCCAGAATCTTCTCCTTGT
GCTAATCTTCCATCTGCTATTGTATCAGCCAAAGATGCTGGATCCATGAGTTTATCACATCGCTTCCACAAGGTTATAATGCTAT

>Bcin05g07690(MLST2), partial sequence [organism=Botrytis pseudocinerea, strain N11_K_W15]
ACCACTATCACCAAGTCCTTTTCTGCACTTTCCTCATCAAAAACGAACGAGAACATCATCAATAAATACTTTGCTGAAGTAATTGGGTTTTATT
TTGGCCAGGATGCCTTCTCTCTACGACAGCAAGCCTTCGATGATATGTTGTGGGTAGTTCTTGGTTGGTTGGATACTGTCAAATTCATTGATTT
ACATTCTGAATTGCACTATTCAAATGACTCTCAGCCAGAATGGTACGGACAACAATATAAACCTGCATTTGCACATCGAGCGCGACTATTTTGG
GAATTGGCGTCACAAGGATGGGATACTACTCTCTGTGGTGGTGGGATGATATGGTCACCATACCTTACTCCATACAAGAACGCAATTACCAATG
AACTCTATATCGCAGCTTCGATATCGATGTACCTCTATTTCCCTGGAGATGACAATCAATCCCCATTTATGCTTTCCAACCCTTCATATCCACC
TCGCGATCCGAAATATCTACAGGCAGCTGTTGATGCTTACAAATGGCTGAATGGTTCCAATATGACGGATTTACAAGGATTATATGTCGACGGA
TATCATATCTCGAATCTTTCTGGCGGTGAAAACACCCATTGCGATTCTAGAAATGAGATGGTATATACCTACAATCAAGGTGTTTTGCTTACTG
GACAACGTGGTTTGTATGACGCAACCGCCGCACGATCATACCTTGTGGATGGCCACAAACTCATCGCGAATGTTATTAACGCCACAGGCTATGA
TCTGAAACACGATGTTGTTATCTCACCGCCACCCAAAGATGGTTCCGCATTGGCAAAATGGTTTGGCCTGGGTAGGAATGGAATACTGGAAGAA
GGATGCGATTCAAGTGCTTCGTGTTCTCAAAATGGACAAACTTTCAAAGGCATATTCTTTCATCACTTGATTGCATTCTGTAGTGATTTGCCAG
GGGAGCCTATTGCAGGGACGAAAGAAAGCCTGGAACTCGACAGAGAGTGGCATTCTGACAAATGCTCGCAGTATACAAAAT

>Bcin06g01710(MLST3), partial sequence [organism=Botrytis pseudocinerea, strain N11_K_W15]
GGTGAGTCCGGCTTTTGTATCTGAGCGTTAAGATAGACACTGATATACCAAGGCAATCACCTAATTTCATGGCTTTCTTCCCTGCATCATCTAC
CTACCCTGGAATGCTTGGAGAATTATACTCCGCAGCTTTCACAGCTCCTGCTTTCAATTGGATCTGTTCCCCTGCTGTGACAGAATTGGAGACG
GTTGTAATGGATTGGCTGGCCAAGCTTCTCAATCTCCCAGACTGTTATTTGTCTTCGACTCATGGTGGTGGTGTTATCCAAGGATCAGCCTCGG
AAGCTATCGTTACCGTTATGGTTGCTGCCCGCGATAAATATCTTCGTGAAACTACTGAAGGTCTGTCAGGCATTGAACTCGAGGATGCGATTGC
ATATAAGAGGAGTAAGCTAGTTGCACTAGGAAGCGAAATGGCACACAGCTCCACGCAGAAAGCAGCGCAGATAGCTGGCGTTAGATTCCGATCG
ATTCCAGTACTCGCATCCAATGATTTCGCCATGACGGGTGATGATCTAGAGAAGGTATTGAAAGAATGCAAATCTCAAGGATTGGAACCCTTCT
ATCTAACTTCGACTTTGGGAACAACATCTACATGTGCAGTTGATGACTTCGCATCTATCGCAACAGTACTTTCGAAATATGCACCTCCAGATGT
TGCAGGCGAGATATGGGTTCACGTCGATGCTGCTTATGCAGGCGCAGCTTTGGTTTGCCCTGAATACCATCATCTAACATCGTCCTTCCAGCAT
TTTCATTCCTTCGATATGAACATGCACAAATGGCTTTTGACAAATTTCGACGCTTCTTGTCTATATGTCAAGAAACGCAAAGATCTGATCGATG
CACTCTCTATAACACCAAGTTATCTTCGCAACGAGTTTTCAGAGAGTGGACTCGTGACCGACTATCGGGACTGGCAAATTCCTCTCGGAAGACG
CTTCCGAAGCTTAAAGATTTGGTTTGTCCTCAGAACCTACG

>Bcin09g03030(MLST4), partial sequence  [organism=Botrytis pseudocinerea, strain N11_K_W15]
ACCCTTCAATCCCACCGCAGCTCCTATACCGAAAGCGAGCATCCTACCAATTCTCCTTCCACCTGCGACTTTAAGACCATTGGCTTTTCGCACT
TTCACAAAAAAGCATAGTTTAACATTGACGTCGTCCGCATTACAAGTGTTGGCTACTTTTATTGGAAAGCATTGTGGGACAGGATGGAGGGAAG
AAGGACTGGCAGAGAGAGTTTTAGAGGAGGTCGCCAAGAGTTGGAAGAATAGGAGTGGTGGTGTTATTGTCGAGGGCGAGGGAACGGAATTGAA
GGAGATTCTGAAAGCTTTGGAAGGGAATATGAGTGGTGGAAGGATAGTCGTAGGAAGAGAGCTAAGCCGGCAGAACAGTTTAGTACTGGGATCA
TCACAATATGGAGAGGTCAATCATACAAGACTTGGGCTACGGCCAGGGAATATACCCAGAGAGGATAGTCAGTCAAGTTTGGGAATGTCAACGT
TGGAGGTCAATGACGAGGAAGATGAGGATGGCCTGATGGATCCAAGGAGGTGGTTGAAAGTCATTGATGCATTTGAGCAACCCCGACTGGTGTA
TAATGTTGCTAAGAAGCACTTTGATAGGTATGTTTCAATGATAAAATTTTATCTGAATCAACTAACTCAGTACAGAGATACCTCCAAACCTTCA
TTGTTCCCACCTGCGTCTCATAAAACACTCCTCTTCCAAAACCGCTATAATGTTATCCATCAACGTCTCCTTCGCAATGAATCTTTTCAAACGC
CCGCTTTTCAAGGTGGCAAAGCTTCCCTTCAACGCAGCACGTCCGCCATTACCACTCAACAACAATCATACAAATTAACGCCGATAGCTAATCT
TCTCGGTCGCAATCGCAGCTCTCATATGCTTCTCGGCCTCCTCAGCATTTCACCCACTGGTACCCTCGCCATCAATGACCTGACGGGCAGCATC
GCTCTCGATCTTACACACGCAGCAGCCATTC

>Bcin11g01310(MLST5), partial sequence [organism=Botrytis pseudocinerea, strain N11_K_W15]
ACTGACATGGACTTCATGTGGAACCGGCGTACAATGCGCAACGCTTGAAGTTCCGCTCGAATATGGCGATGCAACGTCAACAGCGAAAGCCAGT
ATTGCGCTTGCTCGTTATCCTGCCACTGTTGCCGCGAGCAAGAAGCTCGGGTCTCTCTTGATAAATCCCGGTGGACCCGGTGCCACTGGTGTTG
GCTTTGTGCAGTCTGGAGCCGGTGCCGCCATCTCGACACTGAGTGGTGGATTATACGATATCATCGGATGGGATCCACGTGGAACCGGTGCTTC
CGCTCCTATTTTGGAATGTTTTCCAAATGCCAGTGCGGAGTATGATTTTAACAACGCGTTTCCATCTGCTCCCAATCTCTGGCTCGGACAATTT
TCAAATGCCAGCGCAAATTCTGCCGTTAGCTCTGCTATCACATCTTTTGACACTTCTGTCGCTGCTCTTGCAAAAGCTTGTGTAGCTCAGAAGT
CCCCCGCTCTTTACACCTCAACAGCAGCATATGTTGCTCGAGACATGGCAGCGATAGTCGATGCCTTGGATGGAACCTCTGCAAAACTCAACTA
CTGGGGTTTCTCATATGGAACTATTTTCCTTGCCGAGTTTATCCAAACTTTCCCAGGCCGCGTGGGAAGAGTTCTTGCCGATGGGGTTTTCGAC
```

GCAAAGGCAAATGCACTCACATACGTTAGCCAACTTCCCAACGATCAACTCAGTGTTCGTGCTTCGTTGAACGATTTTGCAGCTTTCTGTACCA
CCGCCGGTAGTAAAGGTTGCTCTTTTGCCACCGCCCCTACTGGAACCACCGGTACCGTTGCTACCAGACTGGACAACATAATGAAGGATATGTT
CCTCAATCCCATTGTTGCTTCGGGCTTAAGCATCA

>Bcin15g03910(MLST6), partial sequence [organism=Botrytis pseudocinerea, strain N11_K_W15]
GCCAAAACACAAAAATCATCCAACGATGAAGATGATACTCCACTGCCCTTAATTATCTGGCACGGACTCGGCGATAATTACAAAGCGGATGGTC
TTGCGCAAGTTGGAAAGCTAGCTGAAGCTATTCATCCTGGGACTTTTGTCTATAACATTCATGTAGATGAGGATGCATCTGCAGATAGGACAGC
TACCTTCTTTGGAAATCTCACTCGTGGGTACATCCCCTAGTCTTCTTTTAAATGCCATGCTGACTCTCTTACCAAGTTCAAATCGAAAAGGTCT
GCGAAGACCTCGCCTCCCATCCTATTCTCTCTACCGCGCCTGCCGTCGACGCAATCGGATTCTCCCAAGGCGGCCAATTCTTGCGCGGTTACAT
ATCCCGCTGCAATTCTCCACCCATCCGCTCTCTCCTGACCTTCGGTTCCCAACACAACGGCATTTCTGCCTTCCAAGCCTGTGGTCCTGCCGAT
TTCCTCTGTCGTGGTGCTCAAACTCTTTTGCGATCCAACACCTGGTCAACCTTTGTCCAATCTCGTCTCGTACCCGCTCAATACTTCAGAGATC
CGGAAAACCTAGACTCTTACCTTGAATATTCCAATTTCCTTGCCGACATCAATAATGAGCGCGTTCTCAAGAACCAAACATATAAATCCAACAT
GGAAAAATTGGAACGATTCGTAATGTATGTCTTTGAAGATGATACAACTGTCATCCCTAAGGAAAGTGGATGGTGGGCTGAAGTCAACGGCACG
GAAGTTACACCACTGAAAGAAAGAGCCATTTATAAGGAAGATTGGCTAGGTTTAAAGACATTGGATGAGGCCGGAAAATTAGTTTTCGAAACCA
TTCCAGGGGGACATATGACGTTAGGAGAGGAGATGCTAGAGAAGGCTTTCAAAGAGTACTTTGGCCCAGCAGGGAAGAAATTTGGG

>Bcin16g03460(MLST7), partial sequence [organism=Botrytis pseudocinerea, strain N11_K_W15]
ATGAACCTCTTAATTTGAACTCTTCATTAATCGCAAGTGATGAACCCCTATTTTGCCCAGAGGATAGTTACAAGACGTATATCATTAGTCGAGA
ACCACTCATGATATATATTGACGGATTTTTGAAAGCGAATGAAAGTAAACATCTGGTTGATGTTAGGTATGTTACTTATTCTGATGAAATGAAC
AAGAGAAACTGATGAGATAGTGAACCGCTTTATGAACCGTCTACTGTTTCTCACGGACAGGAAGTTACCATTGATACTTCAGTTCGAAATTCGG
AAGTGGCGGTTTTAGAGAGGGATGAGGTGGTCAGGTGTATTGAGCATAGAGCGAGGGCATTTCAGGGGTGGAGGGATGAGATGGGGATTGAGAA
GTTGAGGACGCAGAGGTATGGGGTTGGAGGACATTATGGGATGCATTTGTAAGTTTTGGGGGATTGACGAAGGCTTTTGTCTATTTCCTATCAG
TACGGATCTTAAAAGAAAGATAGCATGAACACGAGGGCTAACAACTAGGAAATAGCGATTGGAGCGGAGGTAAACGTGGCATAGACCGATTTAG
TACTTTCATGGTCTATGTCGACGTATCTTCTGATATCGAAGGTGGAGGAACGGAATTCCCACGTATTGTGGGACCACAAGGAGGAAGGTGGGAG
GAATTCCTGGAAACTACGGAAGCATTGGATCCAAGAACTGGAAAAAATGTAACAGTAGAAGGGGTGACATTCAAACCAATCAAGGGAAATGCCG
TATTCTGGGAAAATACTGACAACAACGGGAGGGGATATGAT

>Bcin12g03020(MLST8), partial sequence [organism=Botrytis pseudocinerea, strain N11_K_W15]
CGATTGGCTGCGAAGAAAACTGCGCAGCACCGAGTCACCAAAAGCACGAATAAACCGTCGGCAAGAGAAATATTCTCTGAACCACAACATGACA
CGAGCGCGGAAGAGTATATTGGGCGAGAAGCTTCATCAAGAGCACCAAAGCGACAACGAGTCGATGATAATTATAACTCTTACGGTGGAAGAAA
TGAAAATACAGCAGCTTATGCTTCCGGAAAACTTCCATCTGGAAGTATAAATGTTGGTGGAGGTAGGAAGACCACTTTTCAAGAAGAACCTCGA
ACGGCATTTGTCGCTGGCAAGTTGCCCCCTGGAAGTATCAACATCGGTGGAAAGAAGGCTACCCAAATCGAGGACGAAGGAAGAGCAGCTTATG
CTTCCGGGAAATTGCCCCCAGGAAGTATCAGCTCTGGCGCAAAGAAGGCCATTTCATTCCAAGATGAAACGAGACCGGCCTATGTCTCTGGAAA
GCTCCCACATGGCAGTATCGATGGTATGCGAAACCGCGAAATGGCTGCCGTCCACCGCGAAATTGCTGAGGGTGGGAGGAAACCAGGCCAGGTT
GTCTCTTCTCTATTCACATTCAATCCTACATCAAAGAAAACTTTCGATGAATCCGAAGAACAAGCGGAACCTGCAAAACCATCCAATGCGCCTT
TGACAGAGGAAATGGAGACATTCACCAATCTAGGATTATCGAGAAGGCTTGCAGCCCATCTATCGACTAAACTCGATATGAAAGCCCCGACCGC
CATTCAAAAAGCATCTGTGCAGCAGTTGGTATCGGACGATAGCGATGCTTTCATACAAGCAGAGACTGGATCTGGAAAAACTTTGGCATATCTA
CTACCTATAGTCGAGCGAATATTAGCATTGAGTGAGAATGGCGTACAAA

>Bcin02g07770(MLST9), partial sequence [organism=Botrytis pseudocinerea, strain N11_K_W15]
CAGCTTTCCCTTTCGGTCTTGGCATCTACAGTCATTGCCATGCCTACACCATCACAACTTGAGTCTCGGGCCGTTATCGATTCCGATGCCGTTG
TAGGATTTGCCGAAGCTGTTCCCAGTGGGACCGTAGGAACAGTTTATGAGGCATATAAACCATTCCTTAAAGTCGTAAATGGATGCGTATCATT
CCCTGCCGTCGATGCATCGGGTAACACAGGGTATGTCCTTATACCTTTCTCTTCCTCACGATTGCTATTGAGTCTCTAACATATTTAGTGGTG
GTTTGTCACCAACTGGCAGTAGCAATGGTGGTTGCAGCAGCAGTACCGGTCAAGTATATGTTCGAGGAGGACAAAGCGGATCAAACTACGCTAT
CATGTACTCCTGGTAAGTTCTCTCTAAACTTCTCCTTATAGATCCAATATCTAACAGAATCTTAGGTACATGCCAAAGGACGAGCCCTCAACCG
GTATTGGTCACCGTCACGATTGGGAAGGTGTAATCGTCTGGCTCTCCAGCGCCACCGCCACAACTGCCGACAACATCTTAGCTGTTTGTCCTTC
GGCCCACGGAGGCTGGGATTGTTCCACCGATGGTTATTCCCTTTCTGGTACCAGCCCTCTTATCAAGTACGAAAGTATCTGGCCCGTCGACCAT
TCAATGGGTCTTACTAGTACTGTTGGTGGACAACAACCTATGATTGCTTGGGAGTCTTTACCTACTGCTGCTCAAACTGCTCTTGAGAACACCG
ATATCGGTGCTGCGAATGTTCCATTTATTCCAGCTGTTTTCACAAACAATCT

>Bcin04g02090(MLST10), partial sequence [organism=Botrytis pseudocinerea, strain N11_K_W15]
GCGGAGGATGATATGGCAAGGTCTATGATTACCAAAGCATTTGTAGGCATGAGTAGTAAACTCTCAAATATGACTATGAATGATGTTTACAAGC
CCTACATCCATGTAAGAAATGTAGAATAGAGAGATCAATAACTGGAACTAATATCGTTTGTAGGCTTTCAAGTTACTTACGCAGTTCAACCCAA
TCACTACAGCTATTGCCGAATCCCCACTATTTCAAATGGCTGTCTCAGCAAATACCATCGAAAAGTACACACTGCTAGGCCCTTTCTTCAGAAT
ATCTCCTCTGCAACAGGAAGTTACCAGGGAATACTTCAGTGCACCAAAGACGATAGATAGGCGACACATTGCCACATCTCAAGATGCGTTACGA
CTGACCTTACAAACCCATCAAAAAGATTTACTCGATATCATCAACCACTTTGTTCGAGCAAGTCCAATCGCCAAAAGCAAAACCTTGGATTGGT
TCGCCTACATTGTGAATCAAAATCACAAGCGTCGAGCACTTCAGGTAGACCCGAAAGAAGTATCTTCTGATGGCTTTATGCACAATGTCACTGT

CGTTCTAGATGGTCTTTGTGAGCCATTCATGGATACCACATTCTCGAAAATTTCGAAGATTGATATTGATTATCTAAGACGTGCGCCTCGTGTA
GATATCAAGGACGAGACCAAGTTGAACGCCGATGAGAAGGCTTCTGAGAAGTACCATGAGGACACGGTTCCTGGCACTTCCAATTTCATCTCTG
AGGTGTTCTTTCTGACATTGGCTGCTCATCATTATGGTAGTGAAGCTCTTAATGCCACGCACAAGAGTCTGGAGAAAGACATCAAATATATTCA
AAAGCAATTGACTGCCGTTGAAGCAGA

>Bcin01g07220(MLST1), partial sequence [organism=Botrytis pseudocinerea, strain N11_S_E06]
ATGGCATATCTTGTTTCTTTTCGCATTATGCTTTGGAACGTGCTGGACAAGCTTGGGTTAGTGCTCTGCGTGTAGAAGCACTAAAGAAGATTCT
TGCACAACCGAAGTCATGGTTTGAGGAATCCAGGAATTCACCTGGCCGATTGAACGAAGTTTTGGATAGGAACTCTGAGGAAATGCGTAATCTC
GTTGGCCGCTTTGTTGGTATTGTATTCACAGCATTTTTTATGTTATTGATATCAATCATTTGGGCTTTCGTGAATACGTGGAAGCTGACATTAG
TCTCAATGGCAACTGGGCCAGTTATATACGCTGTCACCAAAACGTTCAATCGCGTGAGTGGAAAATGGGAAAACAAGTGCAACTACGCATCTGA
AATGACCACTAGCATATTTTCAGAGACGTTCTCCAACATCAAAGTGGTTCGGGCTTTTACTCTGGAAACTTACTTTGCGACAAAACACACCAAA
GCTACAGAAGAACTTTATAAAGTTGGACTAATACGAGCAAACTACTCGGGATTGCTGTGGGGATTGACAGATGCGATGTCATTCTTCATCACTG
CAACTATCTTTTATTATGCCACGGTTCTCATTACCAAGAGAGAGATCAGTATCGCGACTGCACTACAGACTGTCAATCTTCTATTATTTGGTAT
TTCTAATAGTACGAATATGCTGGCCATGATACCACAAATCAACTCTTCTCGCGTTACAGCTACGCATATGCTTGCATTAGCCAATCTCGATTCA
TCTTCCTCCCACGAAAATCAAGGAACCGAACGACTTTCGACAATCTTTCCAATCAAATTCAACCGTCTCTCATTCACATATCCTAATCGTCCTG
AAAAACGAACGATATCATCCTTTTCTCTTTCCCTGATTCCTAACACAACAACTGCACTTGTCGGACCCTCCGGCTCCGGAAAATCTACAATAGC
TGCTCTGCTCATTGGCCTCTATCCACCAGATACTTCAACACCTCCACCGTTGACATTCAATCGCGTCTCCATAAGTAACTGTCACATCCCGTCT
CTCCGGGCTTCTGTCTCACTCGTCCCACAAATACCGATTCTATTTCCAGCTACCATTCTCCATAACATCATTTATGGGCTCCCAGAATCTTCTC
CTTGTGCTAATCTTCCATCTGCTATTGTATCAGCCAAAGATGCTGGGATCCATGAGTTTATCACATCGCTTCCACAAGGTTATAATGCTAT

>Bcin05g07690(MLST2), partial sequence [organism=Botrytis pseudocinerea, strain N11_S_E06]
ACCACTATCACCAAGTCCTTTTCTGCACTTTCCTCATCAAAAACGAACGAGAACATCATCAATAAATACTTTGCTGAAGTAATTGGGTTTTATT
TTGGCCAGGATGCCTTCTCTCTACGACAGCAAGCCTTCGATGATATGTTGTGGGTAGTTCTTGGTTGGTTGGATACTGTCAAATTCATTGATTT
ACATTCTGAATTGCACTATTCAAATGACTCTCAGCCAGAATGGTACGGACAACAATATAAACCTGCATTTGCACATCGAGCGCGACTATTTTGG
GAATTGGCGTCACAAGGATGGGATACTACTCTCTGTGGTGGTGGGATGATATGGTCACCATACCTTACTCCATACAAGAACGCAATTACCAATG
AACTCTATATCGCAGCTTCGATATCGATGTACCTCTATTTCCCTGGAGATGACAATCAATCCCCATTTATGCTTTCCAACCCTTCATATCCACC
TCGCGATCCGAAATATCTACAGGCAGCTGTTGATGCTTACAAATGGCTGAATGGTTCCAATATGACGGATTTACAAGGATTATATGTCGACGGA
TATCATATCTCGAATCTTTCTGGCGGTGAAAACACCCATTGCGATTCTAGAAATGAGATGGTATATACCTACAATCAAGGTGTTTTGCTTACTG
GACAACGTGGTTTGTATGACGCAACCGCCGCACGATCATACCTTGTGGATGGCCACAAACTCATCGCGAATGTTATTAACGCCACAGGCTATGA
TCTGAAACACGATGTTGTTATCTCACCGCCACCCAAAGATGGTTCCGCATTGGCAAAATGGTTTGGCCTGGGTAGGAATGGAATACTGGAAGAA
GGATGCGATTCAAGTGCTTCGTGTTCTCAAAATGGACAAACTTTCAAAGGCATATTCTTTCATCACTTGATTGCATTCTGTAGTGATTTGCCAG
GGGAGCCTATTGCAGGGACGAAAGAAAGCCTGGAACTCGACAGAGAGTGGCATTCTGACAAATGCTCGCAGTATACAAAAT

>Bcin06g01710(MLST3), partial sequence [organism=Botrytis pseudocinerea, strain N11_S_E06]
TGGTGAGTCCGGCTTTTGTATCTGAGCGTTAAGATAGACACTGATATACCAAGGCAATCACCTAATTTCATGGCTTTCTTCCCTGCATCATCTA
CCTACCCTGGAATGCTTGGAGAATTATACTCCGCAGCTTTCACAGCTCCTGCTTTCCAATTGGATCTGTTCCCCTGCTGTGACAGAATTGGAGA
CGGTTGTAATGGATTGGCTGGCCAAGCTTCTCAATCTCCCAGACTGTTATTTGTCTTCGACTCATGGTGGTGGTGTTATCCAAGGATCAGCCTC
GGAAGCTATCGTTACCGTTATGGTGCTGCCCGCGATAAATATCTTCGTGAAACTACTGAAGGTCTGTCAGGCATTGAACTCGAGGATGCGATTG
CATATAAGAGGAGTAAGCTAGTTGCACTGGAAGCGAAATGGCACACAGCTCCACGCAGAAAGCAGCGCAGATAGCTGGCGTTAGATTCCCGATC
GATTCCAGTACTCGCATCCAATGATTTCGCCATGACGGGTGATGATCTAGAGAAGGTATTGAAAGAATGCAAATCTCAAGGATTGGAACCCTTC
TATCTAACTTCGACTTTGGGAACAACATCTACATGTGCAGTTGATGACTTCGCATCTATCGCAACAGTACTTTCGAAATATGCACCTCCAGATG
TTGCAGGCGAGATATGGGTTCACGTCGATGCTGCTTATGCAGGCGCAGCTTTGGTTTGCCCTGAATACCATCATCTAACATCGTCCTTCCAGCA
TTTTCATTTCCTTCGATATGACATGCACAAATGGCTTTCGGACAAATTTCGACGCTTCTTGTCTATATGTCAAGAAACGCAAAGATCTGATCGA
TGCACTCTCTATAACACCAAGTTATCTTCGCAACGAGTTTTCAGAGAGTGGACTCGTGACCGACTATCGGACTGGCAAATTCCTCTCGGAAGAC
GCTTCCGAAGCTTAAAGATTTGGTTTGTCCTCAGACCTACG

>Bcin09g03030(MLST4), partial sequence [organism=Botrytis pseudocinerea, strain N11_S_E06]
ACCCTTCAATCCCACCGCAGCTCCTATACCGAAAGCGAGCATCCTACCAATTCTCCTTCCACCTGCGACTTTAAGACCATTGGCTTTTCGCACT
TTCACAAAAAAGCATAGTTTAACATTGACGTCGTCCGCATTACAAGTGTTGGCTACTTTTATTGGAAAGCATTGTGGGACAGGATGGAGGGAAG
AAGGACTGGCAGAGAGAGTTTTAGAGGAGGTCGCCAAGAGTTGGAAGAATAGGAGTGGTGGTGTTATTGTCGAGGGCGAGGGAACGGAATTGAA
GGAGATTCTGAAAGCTTTGGAAGGGAATATGAGTGGTGGAAGGATAGTCGTAGGAAGAGAGCTAAGCCGGCAGAACAGTTTAGTACTGGGATCA
TCACAATATGGAGAGGTCAATCATACAAGACTTGGGCTACGGCCAGGGAATATACCCAGAGAGGATAGTCAGTCAAGTTTGGGAATGTCAACGT
TGGAGGTCAATGACGAGGAAGATGAGGATGGCCTGATGGATCCAAGGAGGTGGTTGAAAGTCATTGATGCATTTGAGCAACCCCGACTGGTGTA
TAATGTTGCTAAGAAGCACTTTGATAGGTATGTTTCAATGATAAAATTTTATCTGAATCAACTAACTCAGTACAGAGATACCTCCAAACCTTCA
TTGTTCCCACCTGCGTCTCATAAAACACTCCTCTTCCAAAACCGCTATAATGTTATCCATCAACGTCTCCTTCGCAATGAATCTTTTCAAACGC
CCGCTTTTCAAGGTGGCAAAGCTTCCCTTCAACGCAGCACGTCCGCCATTACCACTCAACAACAATCATACAAATTAACGCCGATAGCTAATCT
TCTCGGTCGCAATCGCAGCTCTCATATGCTTCTCGGCCCCTCAGCATTTCACCCACTGGTACCCTCGCCATCAATGACCTGACGGGCAGCATCG

```
CTCTCGATCTTACACACGCAGCAGCCATTC

>Bcin11g01310(MLST5), partial sequence [organism=Botrytis pseudocinerea, strain N11_S_E06]
GACTGACATGGACTTCATGTGGAACCGGCGTACAATGCGCAACGCTTGAAGTTCCGCTCGAATATGGCGATGCAACGTCAACAGCGAAAGCCAG
TATTGCGCTTGCTCGTTATCCTGCCACTGTTGCCGCGAGCAAGAAGCTCGGGTCTCTCTTGATAAATCCCGGTGGACCCGGTGCCACTGGTGTT
GGCTTTGTGCAGTCTGGAGCCGGTGCCGCCATCTCGACACTGAGTGGTGGATTGTACGATATCATCGGATGGGATCCACGTGGAACCGGTGCTT
CCGCTCCTATTTTGGAATGTTTTCCAAATGCCAGTGCGGAGTATGATTTTAACAACGCGTTTCCATCTGCTCCCAATCTCTGGCTCGGACAATT
TTCAAATGCCAGCGCAAATTCTGCCGTTAGCTCTGCTATCACATCTTTTGACACTTCTGTCGCTGCTCTTGCAAAAGCTTGTGTAGCTCAGAAG
TCCCCCGCTCTTTACACCTCAACAGCAGCATATGTTGCTCGAGACATGGCAGCGATAGTCGATGCCTTGGATGGAACCTCTGCAAAACTCAACT
ACTGGGGTTTCTCATATGGAACTATTTTCCTTGCCGAGTTTATCCAAACTTTCCCAGGCCGCGTGGGAAGAGTTCTTGCCGATGGGGTTTTCGA
CGCAAAGGCAAATGCACTCACATACGTTAGCCAACTTCCCAACGATCAACTCAGTGTTCGTGCTTCGTTGAACGATTTTGCAGCTTTCTGTACC
ACCGCCGGTAGTAAAGGTTGCTCTTTTGCCACCGCCCCTACTGGAACCACCGGTACCGTTGCTACCAGACTGGACAACATAATGAAGGATATGT
TCCTCAATCCCATTGTTGCTTCGGGCTTAAGCATCAG

>Bcin15g03910(MLST6), partial sequence [organism=Botrytis pseudocinerea, strain N11_S_E06]
GCCAAAACACAAAATCATCCAACGATGAAGATGATACTCCACTGCCCTTAATTATCTGGCACGGACTCGGCGATAATTACAAAGCGGATGGTCT
TGCGCAAGTTGGAAAGCTAGCTGAAGCTATTCATCCTGGGACTTTTGTCTATAATATTCATGTAGATGAGGATGCATCTGCAGATAGGACAGCT
ACCTTCTTTGGAAATCTCACTCGTGGGTACATCCCCTAGTCTTCTTTTAAATGCCATGCTGACTCTCTTACCAAGTTCAAATCGAAAAGGTCTG
CGAAGACCTCGCCTCCCATCCTATTCTCTCTACCGCGCCTGCCGTCGACGCAATCGGATTCTCCCAAGGCGGCCAATTCTTGCGCGGTTACATA
TCCCGCTGCAATTCTCCACCCATCCGCTCTCTCCTGACCTTCGGTTCCCAACACAACGGCATTTCTGCCTTCCAAGCCTGTGGTCCTGCCGATT
CCTCTGTCGTGGTGCTCAAACTCTTTTGCGATCCAACACCTGGTCAACCTTTGTCCAATCTCGTCTCGTACCCGCTCAATACTTCAGAGATCCG
GAAAACCTAGACTCTTACCTTGAATATTCCAATTTCCTTGCCGACATCAATAATGAGCGCGTTCTCAAGAACCAAACATATAAATCCAACATGG
AAAAATTGGAACGATTCGTAATGTATGTCTTTGAAGATGATACAACTGTCATCCCTAAGGAAAGTGGATGGTGGGCTGAAGTCAACGGCACGGA
AGTTACACCACTGAAAGAAAGAGCCATTTATAAGGAAGATTGGCTAGGTTTAAAGACATTGGATGAGGCCGGAAAATTAGTTTTCGAAACCATT
CCAGGGGGACATATGACGTTAGGAGAGGAGATGCTAGAGAAGGCTTTCAGAGAGTACTTTGGCCCAGCAGGGAAGAAATTTGGG

>Bcin16g03460(MLST7), partial sequence [organism=Botrytis pseudocinerea, strain N11_S_E06]
ATGAACCTCTTAATTTGAACTCTTCATTAATCGCAAGTGATGAACCCCTATTTTGCCCAGAGGATAGTTACAAGACGTATATCATTAGTCGAGA
ACCACTCATGATATATATTGACGGATTTTTGAAAGCGAATGAAAGTAAACATCTGGTTGATGTTAGGTATGTTACTTATTCTGATGAAATGAAC
AAGAGAAACTGATGAGATAGTGAACCGCTTTATGAACCGTCTACTGTTTCTCACGGACAGGAAGTTACCATTGATACTTCAGTTCGAAATCGGA
AGTGGCGGTTTTAGAGAGGGATGAGGTGGTCAGGTGTATTGAGCATAGAGCGAGGGCATTTCAGGGGTGGAGGGATGAGATGGGGATTGAGAAG
TTGAGGACGCAGAGGTATGGGGTTGGAGGACATTATGGGATGCATTTGTAAGTTTTTGGGGATTGACGAAGGCTTTTGTCTATTTCCTATCAGT
ACGGATCTAAAGAAAGATAGCATGAACACGAGGGCTAACAACTAGGAAATAGCGATTGGAGCGGAGGTAAACGTGGCATAGACCGATTTAGTAC
TTTCATGGTCTATGTCGACGTATCTTCTGATATCGAAGGTGGAGGAACGGAATTCCCACGTATTGTGGGACCACAAGGAGGAAGGTGGGAGGAA
TTCCTGGAAACTACGGAAGCATTGGATCCAAGAACTGGAAAAAATGTAACAGTAGAAGGGGTGACATTCAAACCAATCAAGGGAAATGCCGTAT
TCTGGGAAAATACTGACAACAACGGGAGGGGATATGAT

>Bcin12g03020(MLST8), partial sequence  [organism=Botrytis pseudocinerea, strain N11_S_E06]
CGATTGGCTGCGAAGAAAACTGCGCAGCACCGAGTCACCAAAAGCACGAATAAACCGTCGGCAAGAGAAATATTCTCTGAACCACAACATGACA
CGAGCGCGGAAGAGTATATTGGGCGAGAAGCTTCATCAAGAGCACCAAAGCGACAACGAGTCGATGATAATTATAACTCTTACGGTGGAAGAAA
TGAAAATACAGCAGCTTATGCTTCCGGAAAACTTCCATCTGGAAGTATAAATGTTGGTGGAGGTAGGAAGACCACTTTTCAAGAAGAACCTCGA
ACGGCATTTGTCGCTGGCAAGTTGCCCCCTGGAAGTATCAACATCGGTGGAAAGAAGGCTACCCAAATCGAGGACGAAGGAAGAGCAGCTTATG
CTTCCGGGAAATTGCCCCCAGGAAGTATCAGCTCTGGCGCAAAGAAGGCCATTTCATTCCAAGATGAAACGAGACCGGCCTATGTCTCTGGAAA
GCTCCCACATGGCAGTATCGATGGTATGCGAAACCGCGAAATGGCTGCCGTCCACCGCGAAATTGCTGAGGGTGGGAGGAAACCAGGCCAGGTT
GTCTCTTCTCTATTCACATTCAATCCTACATCAAAGAAAACTTTCGATGAATCCGAAGAACAAGCGGAACCTGCAAAACCATCCAATGCGCCTT
TGACAGAGGAAATGGAGACATTCACCAATCTAGGATTATCGAGAAGGCTTGCAGCCCATCTATCGACTAAACTCGATATGAAAGCCCCGACCGC
CATTCAAAAAGCATCTGTGCAGCAGTTGGTATCGGACGATAGCGATGCTTTCATACAAGCAGAGACTGGATCTGGAAAAACTTTGGCATATCTA
CTACCTATAGTCGAGCGAATATTAGCATTGAGTGAGAATGGCGTACAAATA

>Bcin02g07770(MLST9), partial sequence [organism=Botrytis pseudocinerea, strain N11_S_E06]
CAGCTTTCCCTTTCGGTCTTGGCATCTACAGTCATTGCCATGCCTACACCATCACAACTTGAGTCTCGGGCCGTTATCGATTCCGATGCCGTTG
TAGGATTTGCCGAAGCTGTTCCCAGTGGGACCGTAGGAACAGTTTATGAGGCATATAAACCATTCCTTAAAGTCGTAAACGGAAGCGTACCATT
CCCTGCCGTCGATGCATCGGGTAACACAGGGTATGTCCTTATACCTTTCTCTTCCTCACGATTGCTATTGAGTCTCTAACATATTTAGTGGTG
GTTTGTCACCAACTGGCAGTAGCAATGGTGGTTGCAGCAGCAGTACCGGTCAAGTATATGTTCGAGGAGGACAAAGCGGATCAAACTACGCTAT
CATGTACTCCTGGTAAGTTCTCTCTAAACTTCTCCTTATAGATCCAATATCTAACAGAATCTTAGGTACATGCCAAAGGACGAGCCTCAACCGG
TATTGGTCACCGTCACGATTGGGAAGGTGTAATCGTCTGGCTCTCCAGCGCCACCGCCACAACTGCCGACAACATCTTAGCTGTTTGTCCTTCG
GCCCACGGAGGCTGGGATTGTTCCACCGATGGTTATTCCCTTTCTGGTACCAGCCCTCTTATCAAGTACGAAAGTATCTGGCCCGTCGACCATT
```

CAATGGGTCTTACTAGTACTGTTGGTGGACAACAACCTATGATTGCTTGGGAGTCTTTACCTACTGCTGCTCAAACTGCTCTTGAGAACACCGA
TTTCGGTGCTGCGAATGTTCCATTTATTCCAGCTGTTTTCACAAACAATCT

>Bcin04g02090(MLST10), partial sequence [organism=Botrytis pseudocinerea, strain N11_S_E06]
CGGAGGATGATATGGCAAAGTCTATGATTACAAAGCATTTGTAGGCATGAGTAGTAAACTCTCAAATATGACTATGAATGATGTTTACAAGCCC
TACATCCATGTAGAAATGTAGAATAGAGAGATCAATAACTGGAACTAATATCGTTTGTAGGCTTTCAAGTTACTTACGCAGTTCAACCCAATCA
CTACAGCTATTGCCGAATCCCACTATTTCAAATGGCTGTCTCAGCAAATACCATCGAAAAGTACACACTACTAGGCCCTTCTTCAGAATATCTC
CTCCGCAACAGGAAGTTACCAGGGAATACTTCAGTGCACCAAAGACGATAGATAGGCGACACATTGCCACATCTCAAGATGCGTTACGACTGAC
CTTACAAACCCATCAAAAAGATTTACTTCGATATCATCAACCACTTTGTTCGAGCAAGTCCAATCGCCAAAAGCAAAACCTTGGATTGGTTCGC
CTACATTGTGAATCAAAATCACAAGCGTCGAGCACTTCAGGTAGACCCGAAAGAAGTATCTTCTGATGGCTTTATGCACAATGTCACTGTCGTT
CTAGATGGTCTTTGTGAGCCATTCATGGATACCACATTCTCGAAAATTTCGAAGATTGATATTGATTATCTAAGACGTGCGCCTCGTGTAGATA
TCAAGGACGAGACCAAGTTGAACGCCGATGAGAAGGCTTCTGAGAAGTACTATGAGGACACGGTTCCTGGCACTTCCAATTTCATCTCTGAGGT
GTTCTTTCTGACATTGGCTGCTCATCACTATGGTAGTGAAGCTCTTAATGCCACGCACAAGAGTCTGGAGAAAGACATCAAATATATTCAAAAG
CAATTGACTGCCGTTGAAGCAG

>Bcin01g07220(MLST1), partial sequence [organism=Botrytis pseudocinerea, strain VD256]
GATGGCATATCTTGTTTCTTTTCGCATTATGCTTTGGAACGTGCTGGACAAGCTTGGGTTAGTGCTCTGCGTGTAGAAGCACTAAAGAAGATTC
TTGCACAACCGAAGTCATGGTTTGAGGAATCCAGGAATTCACCTGGCCGATTGAACGAAGTTTTGGATAGGAACTCTGAGGAAATGCGTAATCT
CGTTGGCCGCTTTGTTGGTATTGTATTCACAGCATTTTTTATGTTATTGATATCAATCATTTGGGCTTTCGTGAATACGTGGAAGCTGACATTA
GTCTCAATGGCAACTGGGCCAGTTATATACGCTGTCACCAAAACGTTCAATCGCGTGAGTGGAAAATGGGAAAACAAGTGCAACTACGCATCTG
AAATGACCACTAGCATATTTTCAGAGACGTTCTCCAACATCAAAGTGGTTCGGGCTTTTACTCTGGAAACTTACTTTGCGACAAAACACACCAA
AGCTACAGAAGAACTTTATAAAGTTGGACTAATACGAGCAAACTACTCGGGATTGCTGTGGGGATTGACAGATGCGATGTCATTCTTCATCACT
GCAACTATCTTTTATTATGCCACGGTTCTCATTACCAAGAGAGAGATCAGTATCGCGACTGCACTACAGACTGTCAATCTTCTATTATTTGGTA
TTTCTAATAGTACGAATATGCTGGCCATGATACCACAAATCAACTCTTCTCGCGTTACAGCTACGCATATGCTTGCATTAGCCAATCTCGATTC
ATCTTCCTCCCACGAAAATCAAGGAACCGAACGACTTTCGACAATCTTTCCAATCAAATTCAACCGTCTCTCATTCACATATCCTAATCGTCCT
GAAAAACGAACGATATCATCCTTTTCTCTTTCCCTGATTCCTAACACAACAACTGCACTTGTCGGACCCTCCGGCTCCGGAAAATCTACAATAG
CTGCTCTGCTCATTGGCCTCTATCCACCAGGTACTTCAACACCTCCACCGTTGACATTCAATCGCGTCTCCATAAGTAACTGTCACATCCCGTC
TCTCCGGGCTTCTGTCTCACTCGTCCCACAAATACCGATTCTATTTCCAGCTACCATTCTCCATAACATCATTTATGGGCTCCCAGAATCTTCT
CCTTGTGCTAATCTTCCATCTGCTATTGTATCAGCCAAAGATGCTGGGATCCATGAGTTTATCACATCGCTTCCACAAGGTTATAATGCTAT

>Bcin05g07690(MLST2), partial sequence [organism=Botrytis pseudocinerea, strain VD256]
ACCACTATCACCAAGTCCTTTTCTGCACTTTCCTCATCAAAAACGAACGAGAACATCATCAATAAATACTTTGCTGAAGTAATTGGGTTTTATT
TTGGCCAGGATGCCTTCTCTCTACGACAGCAAGCCTTCGATGATATGTTGTGGGTAGTTCTTGGTTGGTTGGATACTGTCAAATTCATTGATTT
ACATTCTGAATTGCACTATTCAAATGACTCTCAGCCAGAATGGTACGGACAACAATATAAACCTGCATTTGCACATCGAGCGCGACTATTTTGG
GAATTGGCGTCACAAGGATGGGATACTACTCTCTGTGGTGGTGGGATGATATGGTCACCATACCTTACTCCATACAAGAACGCAATTACCAATG
AACTCTATATCGCAGCTTCGATATCGATGTACCTCTATTTCCCTGGAGATGACAATCAATCCCCATTTATGCTTTCCAACCCTTCATATCCACC
TCGCGATCCGAAATATCTACAGGCAGCTGTTGATGCTTACAAATGGCTGAATGGTTCCAATATGACGGATTTACAAGGATTATATGTCGACGGA
TATCATATCTCGAATCTTTCTGGCGGTGAAAACACCCATTGCGATTCTAGAAATGAGATGGTATATACCTACAATCAAGGTGTTTTGCTTACTG
GACAACGTGGTTTGTATGACGCAACCGCCGCACGATCATACCTTGTGGATGGCCACAAACTCATCGCGAATGTTATTAACGCCACAGGCTATGA
TCTGAAACACGATGTTGTTATCTCACCGCCACCCAAAGATGGTTCCGCATTGGCAAAATGGTTTGGCCTGGGTAGGAATGGAATACTGGAAGAA
GGATGCGATTCAAGTGCTTCGTGTTCTCAAAATGGACAAACTTTCAAAGGCATATTCTTTCATCACTTGATTGCATTCTGTAGTGATTTGCCAG
GGGAGCCTATTGCAGGGACGAAAGAAAGCCTGGAACTCGACAGAGAGTGGCATTCTGACAAATGCTCGCAGTATACAAAAT

>Bcin06g01710(MLST3), partial sequence [organism=Botrytis pseudocinerea, strain VD256]
GTGAGTCCGGCTTTTGTATCTGAGCGTTAAGATAGACACTGATATACCAAGGCAATCACCTAATTTCATGGCTTTCTTCCCTGCATCATCTACC
TACCCTGGAATGCTTGGAGAATTATACTCCGCAGCTTTCACAGCTCCTGCTTTCAATTGGATCTGTTCCCCTGCTGTGACAGAATTGGAGACGG
TTGTAATGGATTGGCTGGCCAAGCTTCTCAATCTCCCAGACTGTTATTTGTCTTCGACTCATGGTGGTGGTGTTATCCAAGGATCAGCCTCGGA
AGCTATCGTTACCGTTATGGTTGCTGCCCGCGATAAATGTCTTCGTGAAACTACTGAAGGTCTGTCAGGCATTGAACTCGAGGGTGCGATTGCA
TATAAGAGGAGTAAGCTAGTTGCACTAGGAAGCGAAATGGCACACAGCTCCACGCAGAAAGCAGCGCAGATAGCTGGCGTTAGATTCCGATCGA
TTCCAGTACTCGCATCCAATGATTTCGCCATGACGGGTGATGATCTAGAGAAGGTATTGAAAGAATGCAAATCTCAAGGATTGGAACCCTTCTA
TCTAACTTCGACTTTGGGAACAACATCTACATGTGCAGTTGATGACTTCGCATCTATCGCAACAGTACTTTCGAAATATGCACCTCCAGATGCT
GCAGGCGAGATATGGGTTCACGTCGATGCTGCTTATGCAGGCGCAGCTTTGGTTTGCCCTGAATACCACCATCTAACATCGTCCTTCCAGCATT
TTCATTCCTTCGATATGAACATGCACAAATGGCTTTTGACAAATTTCGACGCTTCTTGTCTATATGTCAAGAAACGCAAAGATCTGATCGATGC
ACTCTCTATAACACCAAGTTATCTTCGCAACGAGTTTTCAGAGAGTGGACTCGTGACCGACTATCGGGACTGGCAAATTCCTCTCGGAAGACGC
TTCCGAAGCTTAAAGATTTGGTTTGTCCTCAGAACCTAC

>Bcin09g03030(MLST4), partial sequence [organism=Botrytis pseudocinerea, strain VD256]

ACGACCCTTCAATCCCACCGCAGCTCCTATACCGAAAGCGAGCATCCTACCAATTCTCCTTCCACCTGCGACTTTAAGACCATTGGCTTTTCGC
ACTTTCACAAAAAAGCATAGTTTAACATTGACGTCGTCCGCATTACAAGTGTTGGCTACTTTTATTGGAAAGCATTGTGGGACAGGATGGAGGG
AAGAAGGACTGGCAGAGAGAGTTTTAGAGGAGGTCGCCAAGAGTTGGAAGAATAGGAGTGGTGGTGTTATTGTCGAGGGCGAGGGAACGGAATT
GAAGGAGATTCTGAAAGCTTTGGAAGGGAATATGAGTGGTGGAAGGATAGTCGTAGGAAGAGAGCTAAGCCGGCAGAACAGTTTAGTACTGGGA
TCATCACAATATGGAGAGGTCAATCATACAAGACTTGGGCTACGGCCAGGGAATATACCCAGAGAGGATAGTCAGTCAAGTTTGGGAATGTCAA
CGTTGGAGGTCAATGACGAGGAAGATGAGGATGGCCTGATGGATCCAAGGAGGTGGTTGAAAGTCATTGATGCATTTGAGCAACCCCGACTGGT
GTATAATGTTGCTAAGAAGCACTTTGATAGGTATGTTTCAATGATAAAATTTTATCTGAATCAACTAACTCAGTACAGAGATACCTCCAAACCT
TCATTGTTCCCACCTGCGTCTCATAAAACACTCCTCTTCCAAAACCGCTATAATGTTATCCATCAACGTCTCCTTCGCAATGAATCTTTTCAAA
CGCCCGCTTTTCAAGGTGGCAAAGCTTCCCTTCAACGCAGCACGTCCGCCATTACCACTCAACAACAATCATACAAATTAACGCCGATAGCTAA
TCTTCTCGGTCGCAATCGCAGCTCTCATATGCTTCTCGGCCTCCTCAGCATTTCACCCACTGGTACCCTCGCCATCAATGACCTGACGGGCAGC
ATCGCTCTCGATCTTACACACGCAGCAGCCATTC

>Bcin11g01310(MLST5), partial sequence [organism=Botrytis pseudocinerea, strain VD256]
ACTGACATGGACTTCATGTGGAACCGGCGTACAATGCGCAACGCTTGAAGTTCCGCTCGAATATGGCGATGCAACGTCAACAGCGAAAGCCAGT
ATTGCGCTTGCTCGTTATCCTGCCACTGTTGCCGCGAGCAAGAAGCTCGGGTCTCTCTTGATAAATCCCGGTGGACCCGGTGCCACTGGTGTTG
GCTTTGTGCAGTCTGGAGCCGGTGCCGCCATCTCGACACTGAGTGGTGGATTATACGATATCATCGGATGGGATCCACGTGGAACCGGTGCTTC
CGCTCCTATTTTGGAATGTTTTCCAAATGCCAGTGCGGAGTATGATTTTAACAACGCGTTTCCATCTGCTCCCAATCTCTGGCTCGGACAATTT
TCAAATGCCAGCGCAAATTCTGCCGTTAGCTCTGCTATCACATCTTTTGACACTTCTGTCGCTGCTCTTGCAAAAGCTTGTGTAGCTCAGAAGT
CCCCCGCTCTTTACACCTCAACAGCAGCATATGTTGCTCGAGACATGGCAGCGATAGTCGATGCCTTGGATGGAACCTCTGCAAAACTCAACTA
CTGGGGTTTCTCATATGGAACTATTTTCCTTGCCGAGTTTATCCAAACTTTCCCAGGCCGCGTGGGAAGAGTTCTTGCCGATGGGGTTTTCGAC
GCAAAGGCAAATGCACTCACATACGTTAGCCAACTTCCCAACGATCAACTCAGTGTTCGTGCTTCGTTGAACGATTTTGCAGCTTTCTGTACCA
CCGCCGGTAGTAAAGGTTGCTCTTTTGCCACCGCCCCTACTGGAACCACCGGTACCGTTGCTACCAGACTGGACAACATAATGAAGGATATGTT
CCTCAATCCCATTGTTGCTTCGGGCTTAAGCATCA

>Bcin15g03910(MLST6), partial sequence [organism=Botrytis pseudocinerea, strain VD256]
GCCAAAACACAAAATCATCCAACGATGAAGATGATACTCCACTGCCCTTAATTATCTGGCACGGACTCGGCGATAATTACAAAGCGGATGGTCT
TGCGCAAGTTGGAAAGCTAGCTGAAGCTATTCATCCTGGGACTTTTGTCTATAATATTCATGTAGATGAGGATGCATCTGCAGATAGGACAGCT
ACCTTCTTTGGAAATCTCACTCGTGGGTACATCCCCTAGTCTTCTTTTAAATGCCATGCTGACTCTCTTACCAAGTTCAAATCGAAAAGGTCTG
CGAAGACCTCGCCTCCCATCCTATTCTCTCTACCGCGCCTGCCGTCGACGCAATCGGATTCTCCCAAGGCGGCCAATTCTTGCGCGGTTACATA
TCCCGCTGCAATTCTCCACCCATCCGCTCTCTCCTGACCTTCGGTTCCCAACACAACGGCATTTCTGCCTTCCAAGCCTGTGGTCCTGCCGATT
TCCTCTGTCGTGGTGCTCAAACTCTTTTGCGATCCAACACCTGGTCAACCTTTGTCCAATCTCGTCTCGTACCCGCTCAATACTTCAGAGATCC
GGAAAACCTAGACTCTTACCTTGAATATTCCAATTTCCTTGCCGACATCAATAATGAGCGCGTTCTCAAGAACCAAACATATAAATCCAACATG
GAAAAATTGGAACGATTCGTAATGTATGTCTTTGAAGATGATACAACTGTCATCCCTAAGGAAAGTGGATGGTGGGCTGAAGTCAACGGCACGG
AAGTTACACCACTGAAAGAAAGAGCCATTTATAAGGAAGATTGGCTAGGTTTAAAGACATTGGATGAGGCCGGAAAATTAGTTTTCGAAACCAT
TCCAGGGGGACATATGACGTTAGGAGAGGAGATGCTAGAGAAGGCTTTCAAAGAGTACTTTGGCCCAGCAGGGAAGAAATTTGGGG

>Bcin16g03460(MLST7), partial sequence [organism=Botrytis pseudocinerea, strain VD256]
ATGAACCTCTTAATTTGAACTCTTCATTAATCGCAAGTGATGAACCCCTATTTTGCCCAGAGGATAGTTACAAGACGTATATCATTAGTCGAGA
ACCACTCATGATATATATTGACGGATTTTTGAAAGCGAATGAAAGTAAACATCTGGTTGATGTTAGGTATGTTACTTATTCTGATGAAATGAAC
AAGAGAAACTGATGAGATAGTGAACCGCTTTATGAACCGTCTACTGTTTCTCACGGACAGGAAGTTACCATTGATACTTCAGTTCGAAATTCGG
AAGTGGCGGTTTTAGAGAGGGATGAGGTAGTCAGGTGTATTGAGCATAGAGCGAGGGCATTTCAGGGGTGGAGGGATGAGATGGGGATTGAGAA
GTTGAGGACGCAGAGGTATGGGGTTGGAGGACATTATGGGATGCATTTGTAAGTTTTGGGGGATTGACGAAGGCTTTTGTCTATTTCCTATCAG
TACGGATCTTAAAAGAAAGATAGCATGAACACGAGGGCTAACAACTAGGAAATAGCGATTGGAGCGGAGGTAAACGTGGCATAGACCGATTTAG
TACTTTCATGGTCTATGTCGACGTATCTTCTGATATCGAAGGTGGAGGAACGGAATTCCCACGTATTGTGGACCACAAGGAGGAAGGTGGGAG
GAATTCCTGGAAACTACGGAAGCATTGGATCCAAGAACTGGAAAAAATGTAACAGTAGAAGGGGTGACATTCAAACCAATCAAGGGAAATGCCG
TATTCTGGGAAAATACTGACAACAACGGGAGGGGATATGAT

>Bcin12g03020(MLST8), partial sequence [organism=Botrytis pseudocinerea, strain VD256]
TCGATTGGCTGCGAAGAAAACTGCGCAGCACCGAGTCACCAAAAGCACGAATAAACCGTCGGCAAGAGAAATATTCTCTGAACCACAACATGAC
ACGAGCGCGGAAGAGTATATTGGGCGAGAAGCTTCATCAAGAGCACCAAAGCGACAACGAGTCGATGATAATTATAACTCTTACGGTGGAAGAA
ATGAAAATACAGCAGCTTATGCTTCCGGAAAACTTCCATCTGGAAGTATAAATGTTGGTGGAGGTAGGAAGACCACTTTTCAAGAAGAACCTCG
AACGGCATTTGTCGCTGGCAAGTTGCCCCCTGGAAGTATCAACATCGGTGGAAAGAAGGCTACCCAAATCGAGGACGAAGGAAGAGCAGCTTAT
GCTTCCGGGAAATTGCCCCCAGGAAGTATCAGCTCTGGCGCAAAGAAGGCCATTTCATTCCAAGATGAAACGAGACCGGCCTATGTCTCTGGAA
AGCTCCCACATGGCAGTATCGATGGTATGCGAAACCGCGAAATGGCTGCCGTCCACCGCGAAATTGCTGAGGGTGGGAGGAAACCAGGCCAGGT
TGTCTCTTCTCTATTCACATTCAATCCTACATCAAAGAAAACTTTCGATGAATCCGAAGAACAAGCGGAACCTGCAAAACCATCCAATGCGCCT
TTGACAGAGGAAATGGAGACATTCACCAATCTAGGATTATCGAGAAGGCTTGCAGCCCATCTATCGACTAAACTCGATATGAAAGCCCCGACCG
CCATTCAAAAAGCATCTGTGCAGCAGTTGGTATCGGACGATAGCGATGCTTTCATACAAGCAGAGACTGGATCTGGAAAAACTTTGGCATATCT

ACTACCTATAGTCGAGCGAATATTAGCATTGAGTGAGAATGGCGTACAAATA

>Bcin02g07770(MLST9), partial sequence [organism=Botrytis pseudocinerea, strain VD256]
CAGCTTTCCCTTTCGGTCTTGGCATCTACAGTCATTGCCATGCCTACACCATCACAACTTGAGTCTCGGGCCGTTATCGATTCCGATGCCGTTG
TAGGATTTGCCGAAGCTGTTCCCAGTGGGACCGTAGGAACAGTTTATGAGGCATATAAACCATTCCTTAAAGTCGTAAATGGATGCGTACCATT
CCCTGCCGTCGATGCATCGGGTAACACAGGGTATGTCCTTATACCTTTCTCTTCCTCACGATTGCTATTGAGTCTCTAACATATTTTAGTGGTG
GTTTGTCACCAACTGGCAGTAGCAATGGTGGTTGCAGCAGCAGTACCGGTCAAGTATATGTTCGAGGAGGACAAAGCGGATCAAACTACGCTAT
CATGTACTCCTGGTAAGTTCTCTCTAAACTTCTCCTTATAGATCCAATATCTAACAGAATCTTAGGTACATGCCAAAGGACGAGCCCTCAACCG
GTATTGGTCACCGTCACGATTGGGAAGGTGTAATCGTCTGGCTCTCCAGCGCCACCGCCACAACTGCCGACAACATCTTAGCTGTTTGTCCTTC
GGCCCACGGAGGCTGGGATTGTTCCACCGATGGTTATTCCCTTTCTGGTACCAGCCCTCTTATCAAGTACGAAAGTATCTGGCCCGTCGACCAT
TCAATGGGTCTTACTAGTACTGTTGGTGGACAACAACCTATGATTGCTTGGGAGTCTTTACCTACTGCTGCTCAAACTGCTCTTGAGAACACCG
ATTTCGGTGCTGCGAATGTTCCATTTATTCCAGCTGTTTTCACAAACAATCT

>Bcin04g02090(MLST10), partial sequence [organism=Botrytis pseudocinerea, strain VD256]
CGGAGGATGATATGGCAAAGTCTATGATTACCAAAGCATTTGTAGGCATGAGTAGTAAACTCTCAAATATGACTATGAATGATGTTTACAAGCC
CTACATCCATGTAAGAAATGTAGAATAGAGAGATCAATAACTGGAACTAATATCGTTTGTAGGCTTTCAAGTTACTTACGCAGTTCAACCCAAT
CACTACAGCTATTGCCGAATCCCCACTATTTCAAATGGCTGTCTCAGCAAATACCATCGAAAAGTACACACTGCTAGGCCCTTTCTTCAGAATA
TCTCCTCTGCAACAGGAAGTTACCAGGGAATACTTCAGTGCACCAAAGACGATAGATAGGCGACACATTGCCACATCTCAAGATGCGTTACGAC
TGACCTTACAAACCCATCAAAAAGATTTACTCGATATCATCAACCACTTTGTTCGAGCAAGTCCAATCGCCAAAAGCAAAACCTTGGATTGGTT
CGCCTACATTGTGAATCAAAATCACAAGCGTCGAGCACTTCAGGTAGACCCGAAAGAAGTATCTTCTGATGGCTTTATGCACAATGTCACTGTC
GTTCTAGATGGTCTTTGTGAGCCATTCATGGATACCACATTCTCGAAAATTTCGAAGATTGATATTGATTATCTAAGACGTGCGCCTCGTGTAG
ATATCAAGGACGAGACCAAGTTGAACGCCGATGAGAAGGCTTCTGAGAAGTACTATGAGGACACGGTTCCTGGCACTTCCAATTTCATCTCTGA
GGTGTTCTTTCTGACATTGGCTGCTCATCATTATGGTAGTGAAGCTCTTAATGCCACGCACAAGAGTCTGGAGAAAGACATCAAATATATTCAA
AAGCAATTGACTGCCGTTGAAGCA

>Bcin01g07220(MLST1), partial sequence   [organism=Botrytis cinerea, strain C12_S_E1_5]
ACGAAGTTTTGGATAGGAACTCTGAGGAAATGCGTAATCTCGTTGGCCGCTTTGCTGGTATTGTATTCACAGCATTTTTTATGCTATTGATATC
AATCATTTGGGCTTTCGTGAATACATGGAAACTGACATTAGTCTCAATGGCGACTGGGCCAGTTATATACGCTGTCACCAAAACGTTCAATCGC
GTGAGTGGAAAATGGGAAAACAAGTGCAACTACGCATCTGAAATGACCACTGGCATATTTTCAGAGACTTTCTCCAACATCAAAGTGGTTCGGG
CTTTTACTCTGGAAACTTACTTTGAGACAAAACACACCAAAGCTACAGAAGAACTCTATAAAATTGGACTAATACGAGCAAACTACTCGGGGTT
GCTGTGGGGATTGACAGATGCGATGTCATTCTTCATCACTGCAACTATCTTTTATTATGCCACGGTTCTCATTACCAAGAGAGAGATCAGTATC
GCGACTGCACTGCAGACTGTCAATCTTCTATTATTTGGTATTTCTAATAGTACGAATATGCTGGCCATGATACCACAAATCAACTCTTCTCGCG
TTACAGCTACGCATATGCTTGCATTAGCCAATCTCGATTCATCTTCCTCCCACGAAAATAAAGGAGCCGAACGGCTTTCGACAATCTTTCCAAT
CAAATTCAACCGTCTCTCATTCACATATCCTACTCGTCCTGAAAAACGAACGATATCATCCTTTTCTCTTTCCCTGATTCCTAACTCAACAACT
GCACTTGTCGGACCCTCCGGCTCCGGAAAATCTACATAGCTGCTCTGCTCGTTGGTCTCTATCCGCCAGATACTTCACACC

>Bcin05g07690(MLST2), partial sequence [organism=Botrytis cinerea, strain C12_S_E1_5]
AGGATGCCTTCTCTCTACGACAGCAAGCCTTCGATGATATGTTGTGGGTAGTTCTTGGCTGGCTGGATACTGTCAAATTCATTGATTTACATTC
TGAATTGCACTATTCAAACGACTCTCAGCCAGAATGGTACGGACAACAATATAAACCTGCATTTGCACATCGAGCGCGACTATTTTGGGAATTG
GCTTCACAAGGATGGGATACTACTCTCTGTGGTGGTGGGATGATATGGTCACCATACCTTACTCCATACAAGAACGCAATTACCAATGAACTCT
ATATCGCAGCTTCGATATCGATGTACCTGTATTTCCCCGGAGATGACAATCAATCCCCATTTATGCTTTCCAACCCTTCATATCCACCTCACGA
TCCGAAATATCTACAGGCAGCTGTTGATGCTTACAAATGGCTGAATGGTTCCAACATGACGGATTTACAAGGATTATATGTCGACGGGTACCAT
ATCTCGAATCTTTCTGGCGGTGAAAACACCCATTGCGATTCTAGAAATGAGATGGTATATACCTACAATCAAGGTGTTTTGCTTACTGGACAAC
GTGGTTTGTATGACGCAACCGCCGCACGATCATACCTTGTAGATGGCCACAAACTCATCGCGAATGTTATTAATGCCACAGGCTATGACCTGAA
ACACAATGTTGTCATCTCACCGCCACCCAAAGATGGTTCCGCATTGGCAAAGTGGTTTGGCCTGGGTAGGAATGGAATACTGGAAGAAGGATGC
GATTCAAGTGCTTCGTGTTCTCAAAATGGACAAACTTTCAAAGGCATATTCTTTCATCACTTGATTGCGTTCTGTAGTGATTTGCCAGGGGAGC
CTATTGCAGGGACGAAGGAA

>Bcin06g01710(MLST3), partial sequence [organism=Botrytis cinerea, strain C12_S_E1_5]
CAAGGCAATCACCTAATTTCATGGCGTTCTTCCCTGCATCATCTACCTACCCTGGAATGCTGGGAGAATTATACTCAGCAGCTTTCACAGCTCC
TGCTTTCAATTGGATCTGTTCCCCTGCTGTGACAGAATTGGAGACGGTTGTAATGGATTGGCTGGCCAAGCTTCTCAATCTCCCAGACTGTTAT
TTGTCTTCGACTCATGGTGGTGGTGTTATCCAAGGATCAGCCTCGGAAGCTATCGTTACCGTTATGGTTGCTGCCCGCGATAAATATCTTCGTG
AAACCACTGAAGGTCTGTCGGGCATTGAACTCGAGGATGCGATTGCATATAAGAGGAGTAAGCTAGTTGCACTAGGAAGCGAAATGGCACACAG
CTCCACGCAGAAAGCAGCGCAGATAGCTGGCGTTAGATTCCGATCGATTCCAGTACTCGCATCCAATGATTTCGCCATGACGGGTGATGATTTA
GAGAAGGTATTGAAAGAATGCAAATCTCAAGGATTGGAACCCTTCTATCTAACTTCGACTTTGGGAACAACATCTACATGCGCAGTTGACGACT
TCGCATCTATTGCAACAGTACTTTCAAAATATGCACCTCCAGATGTTGCAGGCGAGATCTGGGTTCACGTCGATGCTGCTTATGCAGGTGCAGC
TTTGGTTTGCCCTGAATACCATCATCTAACATCGTCCTTCCAGCATTTCCATTCCTTCGATATGAACATGCACAAATGGCTTCTGACAAATTTC

GACGCTTCTTGTCTATATGTCAAGAAACGCAAAGATCTGATCGATGCACTCTCCATAACACCAAGTTATCTTCGCAACGAGTTTTCAGAGAGCG
GACTCGTAACCGACTATCGGGACTGGCAAATTCCTCTCGGAAGACGCTTCCGAAGCT

>Bcin09g03030(MLST4), partial sequence [organism=Botrytis cinerea, strain C12_S_E1_5]
GCAGAGAGAGTCTTGGAGGAGGTCGCCAAGAGTTGGAAGAATAGGAGTGGCGGTGTCATTGTCGAGGGCGAGGGAACGGAATTGAAGGAGATTC
TGAAAGCTTTGGAAGGGAATATGAGTGGTGGAAGGATAGTCATAGGAAGAGAGCTAAGCCGGCAGAATAGTTTAGTACTGGGATCATCACAATA
TGGAGAGGTCAATCATACAAGACTTGGGCTACGGCCAGGGAATATACCCAGAGAGGATAGTCAGTCAAGTTTGGGAATGTCAACGTTGGAGGTC
AATGACGAGGAAGATGAGGATGGCCTGATGGATCCAAGAAGGTGGTTAAAAGTCATTGATGCATTTGAGCAACCTCGACTGGTGTACAATGTTG
CTAAAAAGCACTTTGATAGGTATGTTTCAATGATAAAATTTTATCTGAATCGACTAACTCAGTACAGAGATACCTCCAAACCTTCATTGTTCCC
ACCTGCGTCTCATAAAACACTCCTCTTCCAAAACCGCTATAATGTTATCCATCAACGTCTCCTTCGCAATGAATCTTTTCAAACGCCCGCTTTT
CAAGGTGGCAAATCTTCCCTTCAACGCAGCACGTCCGCCATTACCACCCAACAACAATCATACAAATTAACGCCGATAGCTAATCTTCTCGGTC
GCAATCGCAGCTCTCATATGCTTCTCGGTCTCCTCAGTATTTCACCCACTGGTACCCTCGCCATCAATGACCTG

>Bcin11g01310(MLST5), partial sequenceorganism=Botrytis cinerea, strain C12_S_E1_5]
TCATGTGGAACCGGCGTAGAATGCGCAACGCTTGAAGTTCCGCTCGAATATGGCGATGCAACGTCAACGGCAAAAGCCAGTGTTGCGCTTGCTC
GTTATCCTGCCACTGTTGCCGCGAGCAAGAAGCTCGGGTCTCTCTTGATAAATCCCGGTGGACCCGGTGCCTCTGGTGTTGGCTTTGTGCAGTC
TGGAGCCGGTGCCGCCGTCTCGACACTGAGTGGTGGATTGTACGATATCATCGGATGGGATCCACGTGGAACCGGTGCTTCGGCTCCTATTTTG
GAATGTTTTGCAAATGCCAGTGCGGAGTATGATTTTAACAACGCGTTTCCATCTGCTCCGAATCTCTGGCTCGGACAATTTGCGAATGCCAGCG
CAAATTCTGCTGTTAGCTCTGCTATCACATCCTTTGACACTTCTGTCGCTGCTCTTGCAAAAGCTTGCGTGGCTCAGAAATCTCCCGCTCTTTA
CACCTCAACAGCAGCATATGTTGCTCGAGACATGGCAGCGATAGTCGATGCATTGGATGGGACCTCTGCAAAACTTAACTACTGGGGTTTCTCA
TATGGAACTATCTTCCTAGCTGAGTTTATCCAAACTTTCCCAGGCCGCGTGGGAAGAGTTCTTGCCGATGGTGTTTTCGACGCAAAGGCAAATG
CACTCACATACGTTAGCCAACTTCCCAACGATCAACTCAGTGTTCGTGCTTCGTTGAACGATTTTGCAGCTTTCTGCACCACCGCCGGTAGTAA
AGGTTGCTCTTTTGCCACCGCCCCTACTGGAACCACAGGTACTGTTGCTACCAGACTGGACAACATAATGAAGGATATG

>Bcin15g03910(MLST6), partial sequence [organism=Botrytis cinerea, strain C12_S_E1_5]
ATGATACTCCACTTCCCTTGATTATCTGGCATGGACTCGGCGATAATTACAAAGCGGATGGTCTTGCGCAAGTTGGAAAACTAGCTGAAGCTAT
TCATCCTGGGACTTTTGTCTACAATATTCATGTAGATGAGGATGCATCTGCAGATAGGACAGCTACCTTCTTTGGAAATCTCACTCGTGAGTAC
ATCCCCTATTTTTCCTTTAAATATCATACTAACTCTCTTACCAAGTTCAAATCGAAAAGGTCTGCGAAGACCTCGCCTCCCATCCTATTCTCTC
TACCGCGCCCGCCGTCGACGCAATTGGATTCTCCCAAGGCGGCCAATTCTTGCGTGGTTACATATCCCGCTGCAATGCTCCACCCATCCGCTCT
CTCCTGACCTTTGGTTCCCAACACAACGGCATTTCTGCCTTCCAAGCCTGTGGTCCTGCCGATTTCCTCTGTCGCGGTGCTCAAACCCTTTTGC
GATCCAACACCTGGTCAACCTTTGTCCAATCTCGTCTCGTACCCGCTCAATACTTCAGAGATCCGGAAAACCTAGACTCTTACCTTGAATATTC
CAATTTCCTTGCCGACATCAATAATGAGCGCGTTCTCAAGAACCAAACATATAAATCCAACATGGAAAAATTGGAACGATTCGTAATGTATGTC
TTTGAAGACGATACAACTGTCATCCCTAAGGAAAGTGGATGGTGGGCTGAAGTCAACGGCACGGAAGTTACACCACTGAAAGAAAGAGCCATTT
ATAAAGAAGATTGGCTAGGTTTAAAGACATTGGGATGAGGCCGGAAAATTAGTTTTCGAAACCATTCCAGGGGGACATATGACGTTAGGAGAGG
AGATGCTAGAGAAGGCTTTCAAAGA

>Bcin16g03460(MLST7), partial sequence [organism=Botrytis cinerea, strain C12_S_E1_5]
TCTTCATGAACCTCTTAATTTGAACTCTTCATTATTTGCAAGTGATGAACCCCTATTTCTGCCCAGAGGATAGTTACAAGACGTATATCATTAG
TCGAGAACCACTCATGATATATATTGACGGATTTTTGAAAGCGAATGAAAGTAAACATTTGGTTGATGTTAGGTGTGTTATTTATTCTGATGAA
ATGAACAAGAGAGACTGATGAGATAGTGAGCCGCTTTATGAACCGTCAACTGTTTCTCACGGACAGGAAGTTACCATTGATCCTTCAGTTCGGA
ATTCTGAAGTGGCGGTTTTAGAGAGGGATGAGGTGGTCAGGTGTATTGAGCATAGAGCGAGGGCATTTCAGGGGTGGAGGGGCGAGATGGGGAT
TGAGAAGTTGAGGACGCAGAGGTATGGGGTTGGAGGACATTATGGGATGCATTTGTAAGTTTTGGGGGATTGACGAAGGCTTCTGTCTATTTTC
TATCAGCACGGATCTTGAAAGAAAGATAGCATGAACACGCGGGCTAATAACTAGGAAATAGCGATTGGAGCGGAGGTAAACGTGGCATAGACCG
ATTTAGTACTTTCATGGTCTATGTCGACGTATCCTCTGATATCGAAGGTGGAGGAACGGAATTCCCACGTATTGTGGGACCAAAAGGAGGAAGG
TGGGAGGACTTCCTGGAAACTACGGAAGCATTGGATCCAAGAACTGGAGAAAATGTAACAGTAGAAGGGGTGACATTCAAACCAATCAAGGGAA
ATGCCGTATTCT

>Bcin12g03020(MLST8), partial sequence [organism=Botrytis cinerea, strain C12_S_E1_5]
GTCACCAAAAGCACGAATAAACCATCGGCAAGGGAAATATTCTCTGAACCACAACATGACATGAGCGCGGAAGAGTATATCGGGCGAGAAGCTT
CATCAAGAGCACCAAAGCGACAACGAGTCGATGATAATTATAACTCTTACGGTGGAAGAAATGAAAATACAGCAGCTTATGCTTCCGGGAAACT
TCCATCTGGAAGTATAAATGTTGGTGGAGGTAGGAAGACCACTTTTCAAGAAGAACCTCGAACGGCATTTGTCGCTGGCAAGTTGCCCCCTGGA
AGTATCAACATTGGTGGAAAGAAGGCTACCCAAATCGAGGACGAGGGTAGAGCAGCTTATGCTTCCGGAAAATTGCCCCCAGGAAGTATAAACT
CTGGCGCAAAGAAGGCGATTTCATTCCAAGATGAAACGAGACCGGCTTATGTCTCCGGAAAGCTTCCACATGGTAGTATCGACGGTATGCGAAA
CCGTGAAATGGCTGCCGTCCACCGCGAAATTGCTGAGGGTGGAGGAAACCAGGTCAGGTTGTCTCTTCTCTATTCACATTCAATCCTACTTCA
AAGAAAACTTTCGATGAACCAGAAGAACAAGCGGAACCGGCAAAACCATCCAATGCGCCTTTGACAGAGGAAATGGCGACTTTCACCAATCTAG
GGCTATCGAGAAGGCTTGCAGCCCATCTATCGACTAAACTCGATATGAAAGCCCCGACGGCCATTCAAAAAGCATCTGTGCAGCAGTTGGTATC
GGACGATAGCGATGCTTTCATACAAGCAGAGACTGGATCTGGAAAAACTTTGGCATATCTACTACCTATAGTCGAGCGAATATTAGCATTGAGT

GAGAATGGCGT

>Bcin02g07770(MLST9), partial sequence [organism=Botrytis cinerea, strain C12_S_E1_5]
TGTAGGATTTGCCGAAACTGTTCCCAGTGGGACCGTAGGAACAGTTTATGAGGCATATAAACCATTCCTTAAAGTCGTAAATGGATGCGTACCA
TTCCCTGCCGTCGATGCATCGGGTAACACAGGGTATGTCCTTATACCTTTCTCTTCCACACGATTGCTATTGAGTCTCTAACATATTTTAGTGG
TGGTTTGTCACCAACTGGCAGCAGCAATGGTGAATGCAGCAGCAGTACCGGTCAAGTATATGTTCGAGGAGGACAAAGCGGATCAAACTACGCT
ATCATGTACTCCTGGTAAGTTCTCTCTAAACTTCTCCTTATAGATCCAACCTAACAAAATCTTAGGTACATGCCAAAGGACGAGCCCTCAACCG
GTATTGGTCACCGTCACGATTGGGAAGGTGTAATTGTCTGGCTCTCCAGCGCCACCGCCACAACTGCCGACAACATCTTAGCCGTTTGTCCTTC
CGCCCACGGAGGCTGGGATTGTTCCACGGATGGCTATTCCCTTTCTGGTACCAGCCCTCTTATCAAGTACGAAAGTATCTGGCCCGTCGATCAC
TCAATGGGTCTTACTAGTACTGTTGGTGGAAAACAACCTATGATTGCTTGGGAGTCTTTACCAACTGCTGCTCAAACTGCTCTTGAGAACACCG
ATTTCGGTGCTGCGAATGTTCCATTCATTCCGGCTGTTTTCACAGATAATCTTGC

>Bcin04g02090(MLST10), partial sequence [organism=Botrytis cinerea, strain C12_S_E1_5]
AACTGTCGAATATGACTATGAATGATGTTTACAAGCCCTACATCCATGTAAGAGATGTAGAATAGAGAGATCAGTAACTGGAACTAATATCGTT
TGTAGGCTTTCAAGTTACTTACGCAGTTCAACCCAATCACTACAGCTATTGCCGAATCCCCACTGTTTCAAATGGCTGTCTCAGCAAATACCAT
CGAAAAGTACACACTGCTAGGCCCTTTCTTCAGAATATCTCCTCTGCAACAGGAAGTTACCAGGGAATACTTCAGTGCGCCAAAGACGATAGAT
AGGCGACACATTGCCACATCTCAAGATGCGTTACGATTGACCTTACAAACCCATCAAAAAGATTTACTTGATATCATCAACCACTTTGTTCGAG
CAAGTCCAATCGCAAAAAGCAAAACCCTGGATTGGTTCGCCTACATTGTGAATCAAAATCACAAGCGTCGAGCACTTCAGGTAGACCCGAAAGA
AGTGTCTTCTGATGGCTTTATGCACAATGTCACTGTCGTTCTAGATGGTCTTTGTGAGCCATTCATGGATACCACATTCTCGAAAATTTCGAAG
ATTGATATTGATTATCTAAGACGTGCGCCTCGTGTAGATATCAAGGACGAGACCAAGTTGAACGCTGACGAGAAGGCTTCTGAGAAGTATTATG
AGGACACTGTTCCTGGCACTTCTAATTTCATCTCTGAGGTCTTCTTTCTCACATTGG

>Bcin01g07220(MLST1), partial sequence [organism=Botrytis cinerea, strain D08_H_8_4]
ATGACAATATTGAAAAGCAAAGGCTCGAAGAGTCACAAAAAATACAGTCATTCTACTTCTACCTCACGCATCCGATTGCCAGATGATGATCAGA
ATTCGTCCATCGAATTGCGAAATATCAATGACTACCACGCCGATGAAGAAATCAAATCATCTTTCAAATCCCTATTCACATTCACAACCAAAAA
GCACATATCTACCATAGTTCCATGTATAGTATTTGCAATCATTAGCGGCGTTCTAAAACCTATATCGGCAATATTTTACGGAAATATCTTTGGT
ACTCTTACAAATTTTGGGTCCGGTGTTCTCACCGCACAAGAGACCTTACAACATGTCTCGAAATGGTGCATAGCTATAACTGTTCTCGGGGGCG
CAGTATGGTTGTTCGAAGGCTTGTTTCTGTGTTCGTGGATGGTATTTGGAGAGCTCCAGGCTAGAAGCGTGCGCGAGAAAATGTTTGCCGGCAT
GTTGGAGAAGGATTTGGAATGGTTTGATCTGCGTAGAGATGGCATTGGATCATTATTAATTCGAATTGAAACGTGTGTGGAATCCATTTATGGT
CGAAATGAAAACATACTGATTGAACAATAGACAAATTCGAGAGCTCCAATTATCCACATCCCAGCCTCTTGGGTTTCTCCTGTTTGAGACTGCT
AGCGCATGTGCAGCTCTTGGGACTGCCTTCTTTTACTCTTGGAATCTGACTCTTGTCATTATAGCGACATTTCCAATTGCCGGTGGAATACTTT
ATCTAATTTCTAGAAACATGGGCCCAGCCATCGAAGCCCAGAAAAGAGAGCTATCGCAAGCTTCGAAATTTACAAATACAGCCATTACAGCCAT
CGATACTGTCAAAGCATTCAACGGACAGGACCAGGAAGTTTGGCAATACTTCTTAGCAATCAAAAAATCAACAGTTCATTACATGATCCAAGCC
AGGTCAAATGCATTTCAATTTGGGATTACCAAATTTGTCATGGTGGCCATCTTTGTTCAAGGCTTTGGGTATGGTCTCACGTTAGTCGATCATG
GACTAGATGCCGGAAAGGTTCTTACAACTTTCTATGCTTGTTTGACTGGGATGATGGCTATAGAAGTCGTTTTACCACAATGGCTTGTCTTAGC
AAAAGGAATGTCCGCTGGAGCGACTCTCAAGTCTATCATGAACCAAGTGAATAGAGGAGGAGTTGCAAAACACCCAGAAGGGTTAATGGTACCT
AAAACCTGCAACGGTGATATAGAAGTCAATAATGTAAGTAGAGATAGCCACGATAAACCATCAAGACTAATCAAGATCAGGTTACATTTGGATA
TCCTTCCAATCGACAGCACAATGCTCTTATAAAAACAACATTCTTCTTTCCTGCTGGCGAGACTGCTTTTGTAGTTGGTAAAAGTGGTTCTGGA
AAGAGTACATTGGGAAACTTGTTGCTMAAATTTTACGAGCCACAGGAGGGAAGTATCTTGATAGATGGCCAACGTATACAGGATCTCGATACCG
ATTGGCTGAGACGCAACATCACTCTCGTACTGCAGCAGAGTGTGGTATTCAACGAGACGGTTAGGAAAAACATAGAATTTGGCAAGGAAGGAAT
AAAAAATGAAGCTGATATCATAGACGCCTGTTCTACAGCCAATCTAGAACAAGTAATTGCCGATCTTCCTGATGGACTTGATACTATGATTGGC
TCTAAGGAGAGACAACTGAGTGGTGGACAAAAACAACGTGTTGCATTGGCACGAGCTAGATTACGAGATGCACCAATCTTGATTCTTGACGAAG
GAACTAGTGCTCTTGATCTGACCAACCGACTCAAAATCATGGATAATATTCGAGAATGGAGGAAAGGAAAAACGACAATTATTGTGACTCATGA
TATTTCCCAGATATTGGATGATGACTACGTTTACGTTATGGATCAGAGTAAGGTTGTCCAGGAAGGATATCGTAGGAAGCTTTCTGCGAAAGCA
AACGGCACTTTCGCTACATTTTCGCAACTCCCGGGATTGCCCGTTGAACAGTCGACAGACTTGTCCAACATCAGAAGAAACTCGGACCCTGGAA
CACCATTAACTGGAAGCTTTGAAGACTTTGTTCAAGAATTGAAACCTCGTTTCTCTACCAAGTCTACAATCTATGGTCCAAATGCAGCAAATCG
TAGGTCCTTGGATATATCAAACAGACGATTATCTCTTGGATATGCTTCAATAGCCTATGCCAATGACCTACAATCAGATAACATTTGGACATCT
GGAATTGACAGTTCGCGTTCAAGGTTTGATGATTTGCAAAGACCTACCTCACAGTTTCAGAAGTTCCAAGCGTCGGCTAGTAGGCAGTCTATAC
ATCCTATCAGGCCACCAAGGTATCAAGACGCAGGATTCCAATCGATCCCAGAACATCCCTCAGACAAGCCAAGCTTTGATAGAAAACCTCTGAT
TCAAAGCCCTCTCAGCTTGGTATCAATCGAAAAGAATCGAGACTCTAAAGTCGCGCCTATGGATTCTTTGTTGGAACACTCAGATGACTCTGCT
GATAATGCGGGCGATTCTGAAGAGGAGAACGAAACGGGTCATCTCAAACCCGATACTTTAACTAAGATCTTAAAGACTGTTTGGCCCATGCTGA
ACAAGAAGGAACGCACAATCCTTTCGGTTGCATTTGGCGCAGCATTTATTGTGGCAGTATCAACCCCAGCTTTTGCTATAATATATACCAAACT
TCTTGACACTTTTTACCAAAAGGAGAACCGCAACTCGAATGCTTTAAAATGGTCACTTGTCTTACTCGGAATCGCCATCATTGATGGCATATCT
TGTTTCTTTTCGCATTATGCTTTGGAACGTGCTGGACAAGCTTGGGTTAGTGCTCTGCGTGTAGAAGCACTAAAGAAGATTCTCGCACAACCGA
AGTCATGGTTTGAGGAATCCAGGAATTCACCTGGCCGGTTGAACGAAGTTTTGGATAGGAACTCTGAGGAAATGCGTAATCTCGTTGGCCGCTT
TGCTGGTATTGTATTCACAGCATTTTTTATGCTATTGATATCAATCATTTGGGCTTTCGTGAATACATGGAAACTGACATTAGTCTCAATGGCG
ACTGGGCCAGTTATATACGCTGTCACCAAAACGTTCAATCGCGTGAGTGGAAAATGGGAAAACAAGTGCAACTACGCATCTGAAATGACCACTG

GCATATTTTCAGAGACTTTCTCCAACATCAAAGTGGTTCGGGCTTTTACTCTGGAAACTTACTTTGAGACAAAACACACCAAAGCTACAGAAGA
ACTCTATAAAATTGGACTAATACGAGCAAACTACTCGGGATTGCTGTGGGGATTGACAGATGCGATGTCATTCTTCATCACTGCAACTATCTTT
TATTATGCCACGGTTCTCATTACCAAGAGAGAGATCAGTATCGCGACTGCACTGCAGACTGTCAATCTTCTATTATTTGGTATTTCTAATAGTA
CGAATATGCTGGCCATGATACCACAAATCAACTCTTCTCGCGTTACAGCTACGCATATGCTTGCATTAGCCAATCTCGATTCATCTTCTTCCCA
CGAAAATAAAGGAGCCGAACGGCTTTCGACAATCTTTCCAATCAAATTCAACAGTCTCTCATTCACATATCCTACTCGTCCTGAAAAACGAACG
ATATCATCCTTTTCTCTTTCCCTGATTCCTAACTCAACAACTGCACTTGTCGGACCCTCCGGCTCCGGAAAATCTACAATAGCTGCTCTGCTCG
TTGGTCTCTATCCGCCAGATACTTCAACACCTCCACCGTTGACATTCAATCGTGTCTCCATAAGTAACTGTCACATTCCGTCTCTCCGGGCTTC
TCTCTCACTCGTCCCACAAATACCGATTCTATTTCCAGCTACCATTCTCCATAACATCATTTATGGTCTCCCAGAATCTTCTCCTTGTGCTAGT
CTTCCATCTGCTATTGCATCAGCAAAAGATGCTGGGATCCATGAATTTATCACATCGCTTCCACAAGGTTATGATACTATGGTAGGAGATGGAG
GACAAGAGTTATCGGGAGGACAGGCCCAGAGAATTGTGATATCGAGAGCACTGGTCAGAAAACCAACTTTATTGATATTAGATGAAGCGACGAG
TGGATTAGATGGTGGTAGTGCAGAGGTGATAAGAGAGACTGTGCAGAAGTTGAAGGAGAGAGAAGGAATGGCCACGCTGGTTGTTAGTCATACG
GCTGAAATGATGAAGATAGCGGGACGAGTCGTGGTGATGGAAGAGGGAGAGATAGTTGAGAGTGGTGGTTTCGATGAGTTGAAGAATAAAGTTG
AGGGGAAATTCAGGACTTTAGTTAGAGATGATTCGGGGGATAAAGTGGTGCTGAAGGAAGAGAATGATGCTGGAGAGGCTAGTAGTAACGCTAG
TAGCGATGCTCATAAAGGTGGGTTGGGTATTAGAATTCCTGGTTACGATGAGGTAGAATTCCATACTGGAAGAGACACGCCAATTCGAGATGCA
AGAAGGAGAGATACTTGGCTGCGGCAAAACAATCATTGA

>Bcin05g07690(MLST2), partial sequence [organism=Botrytis cinerea, strain D08_H_8_4]
ATGATTTCGTTCTCAACACAGATCAATCCACGACCGCGTATATCTTTGCTACTGCTGTGCTTATTCTGCGCACTATCTTTCTCAATTGGCGTAG
ATGGGAGGCCCAACGATGTTGTGCGACAGAATGATTTGATTCAACGACCACTTACGAATAATGAAGATATCACAATCAATGGGAGCCCTCAGGA
GCTCATCAAGCCTGGAGACTCAAAGGTTCTACCGGCAATGTTAAGCGCGCTGGACGTGCTACAAGAAGACTACTTTGCGACATGGCAAGGCATC
TATCCAACAGGAATCGATTGGACATCTGCAGTGATTGGTACAATACTTTCTTTGCATGTTGAAACCACTTTAGAGCAAGAGCTAACACCACTCT
CTAAACAGGCACCTATGTTGCTGGTGCACTCACCACTATCACCAAGTCCTTTTCTGCACTTTCCTCATCAAAAACGAACGAGAACATCATCAAT
AAATACTTTGCTGAAGTAATTGGGTTTTATTTTGGCCAGGATGCCTTCTCTCTACGACAGCAAGCCTTCGATGATATGTTGTGGGTAGTTCTTG
GCTGGCTGGATACTGTCAAATTCATTGATTTACATTCTGAATTGCACTATTCAAACGACTCTCAGCCAGAATGGTACGGACAACAATATAAACC
TGCATTTGCACATCGAGCGCGACTATTTTGGGAATTGGCTTCACAAGGATGGGATACTACTCTCTGTGGTGGTGGGATGATATGGTCACCATAC
CTTACTCCATACAAGAACGCAATTACCAATGAACTCTATATCGCAGCTTCGATATCGATGTACCTATATTTCCCCGGAGATGACAATCAATCCC
CATTTATGATTTCCAACCCTTCATATCCACCTCACGATCCGAAATATCTACAGGCAGCTGTTGATGCTTACAAATGGCTGAATGGTTCCAACAT
GACGGATTCACAAGGATTATATGTCGACGGGTACCATATCTCGAATCTTTCTGGCGGTGAAAACACCCATTGCGATTCTAGAAATGAGATGGTA
TATACCTACAATCAAGGTGTTTTGCTTACTGGACAACGTGGTTTGTATGACGCAACCGCCGCACGATCATACCTTGTAGATGGCCACAAACTCA
TCGCGAATGTTATTAATGCCACAGGCTATGACCTGAAACACAATGTTGTCATCTCACCGCCACCCAAAGATGGTTCCGCATTGGCAAAGTGGTT
TGGCCTGGGTAGGAATGGAATACTGGAAGAAGGATGCGATTCAAGTGCTTCGTGTTCTCAAAATGGACAAACTTTCAAAGGCATATTCTTTCAT
CACTTGATTGCGTTCTGTAGTGATTTGCCAGGGGAGCCTATTGCAGGGACGAAGGAAAGCTTAGAACTCGACAGAGTGTGGCATTCTGACAAAT
GCTCACAGTATACAAAATGGATCAGGCGAAATGCCGAAGCTGCGTTAAGTACCAAGAATGAAGAAGGGAAATTTGGTATGTGGTGGGGTGTACC
GGCTACACAGAGTTCTTTAGCGGACTATACACAGGACCAGACATCGAGAGGCTCTGTAGATTATCGAAATACTGGAGTTCCAAAGAACACCGAA
TGGAGAGGAGAAGAATACCCCAGAGAAGTGAAAGCGAGCAAGACTAAAGAAGGTGCAGATGTATATAGTGGGGTAGAAGATCCCAATGATCGAG
GTAGAGGAAGAACGGTAGAAACTCAGGGTGGAGGATTGTCTGTCTTGAGAGCATTGTGGGAGGTAGAGCTGAGATAA

>Bcin06g01710(MLST3), partial sequence [organism=Botrytis cinerea, strain D08_H_8_4]
ATGGATTCGAAGCAGTTTAGAGAGGCGGCGACGTCGGCGATTGACGAAAGTAAGTTCCTGCTTCCCAAGATGAAGTACTGTATACTGACCAACA
TAGTCATCCAATATTATGATAATATTCATGAACGAAGAGTAATTTCAAATGTCGAGCCTGGTTACCTGAAGAAGATTCTTCCAGATGGTCCACC
GGAAGAGGGTGAATCATGGGCAGAGATCCAGAAAGATATCGAATCTAAAATTATGCCTGGTCTGACACATTGGTGAGTCTGACTTTTGTATTTG
AGCGTTAAGATAGACACTGATATACCAAGGCAATCACCTAATTTCATGGCGTTCTTCCCTGCATCATCTACCTACCCTGGAATGCTGGGAGAAT
TATACTCAGCAGCTTTCACAGCTCCTGCTTTCAATTGGATCTGTTCCCCTGCCGTGACAGAATTGGAGACGGTTGTAATGGATTGGCTGGSCAA
GCTTCTCAATCTCCCAGACTGTTATTTGTCTTCGACTCATGGTGGTGGTGTTATCCAAGGATCAGCCTCGGAAGCTATCGTTACCGTTATGGTT
GCTGCCCGCGATAAATATCTTCGTGAAACTACTGAAGGTCTGTCGGGCATTGAACTCGAGGATGCGATTGCATATAAGAGGAGTAAGCTAGTTG
CACTAGGAAGCGAAATGGCACACAGCTCCACGCAGAAAGCAGCGCAGATAGCTGGCGTTAGATTCCGATCGATTCCAGTACTCGCATCCAATGA
TTTCGCCATGACGGGTGATGATTTAGAGAAGGTATTGGAAGAATGCAAATCTCAAGGATTGGAACCCTTCTATCTAACTTCGACTTTGGGAACA
ACATCTACATGCGCAGTTGACGACTTCGCATCTATTGCAACAGTACTTTCAAAATATGCACCTCCAGATGTTGCAGGCGAGATCTGGGTTCACG
TCGATGCTGCTTATGCAGGTGCAGCTTTGGTTTGCCCTGAATACCATCATCTAACATCGTCCTTCCAGCATTTCCATTCCTTCGATATGAACAT
GCACAAATGGCTTCTGACAAATTTCGACGCTTCTTGTCTATATGTCAAGAAACGCAAAGATCTGATCGATGCACTCTCCATAACACCAAGTTAT
CTTCGCAACGAGTTTTCAGAGAGTGGACTCGTAACCGACTATCGGGACTGGCAAATTCCTCTCGGAAGACGCTTCCGAAGCTTAAAGATTTGGT
TTGTCCTCAGAACCTACGGAGTCAAGGGCCTACAAGAGCACATCCGAAAGCACGTGAAGCTCGGAGAATTACTTGTCAGCCTGCTCAAGACACG
AGAAGATCTATTCAATATAGTTACAGGGCCCAATTTCGCCCTCACTGTTCTGAACATTGTTCCCAAATCCACAAATGCGGATGCACAGAATAGT
CTCACGAAAGAAGTCTACGAGCTGATTAACAAAAGAAGCGAAATCTATCTCACAGCTGGCGTGGTGGCGGGTGCATATGTAATCCGAGTCGTGA
GTGCGAATCCCAAGGCGGAAGAAAGTTATATCCGCAAAGCTTTTGATATCCTAGTTGATACGACTGAAGAGGTCCGGGATGGGAAAGCGAGCAA
GCGGGGAAATCTCAAAGGGGTTGTTATGAATGGGAAGGCGGAGGGTGTGGGTGAAGTCGCTGTTGTTAATGGGAATGGATTGCCAAATCAGAGC
TAG

>Bcin09g03030(MLST4), partial sequence [organism=Botrytis cinerea, strain D08_H_8_4]
ATGGCTCSCCTCAATCCCACAACGAAATCAGAAGCCCCTCCGCTCTTCAGGCCTCCTGCTACCCCCGCTACAAATTCGAATCCTATCCCCTCAT
CATCGCCTGCATTTGCAACTCCGGTACATCCTATACGACCCTTCAACCCCACCGCAGCTCCTATACCGAAAGCGAGTATCCTACCAATTCTCCT
TCCACCTGCGACTTTAAGACCATTGGCTTTCCGCACTTTCACAAAAAAGCATAGTTTAACATTGACGTCGTCGGCATTACAAGTGTTGGCTACT
TTTATTGGAAAGCATTGTGGGACAGGATGGAGGGAAGAAGGACTGGCAGAGAGAGTCTTGGAGGAGGTCGCCAAGAGTTGGAAGAATAGGAGTG
GCGGTGTCATTGTCGAGGGCGAGGGAACGGAATTGAAGGAGATTCTGAAAGCTTTGGAAGGGAATATGAGTGGTGGAAGGATAGTCATAGGAAG
AGAGCTAAGCCGGCAGAATAGTTTAGTACTGGGATCATCACAATATGGAGAGGTCAATCATACAAGACTTGGGCTACGGCCAGGGAATATACCC
AGAGAGGATAGTCAGTCAAGTTTGGGAATGTCAACGTTGGAGGTCAATGACGAGGAGGATGAGGATGGCCTGATGGATCCAAGAAGGTGGTTAA
AAGTCATTGATGCATTTGAGCAACCTCGACTGGTGTACAATGTTGCTAAAAAGCACTTTGATAGGTATGTTTCAATGATAAAATTTTATCTGAA
TCTACTAACTCAGTACAGAGATACCTCCAAACCTTCATTGTTCCCACCTGCGTCTCATAAAACACTCCTCTTCCAAAACCGCTATAATGTTATC
CATCAACGTCTCCTTCGCAATGAATCTTTTCAAACGCCCGCTTTTCAAGGTGGCAAATCTTCCCTTCAACGCAGCACGTCCGCCATTACCACCC
AACAACAATCATACAAATTAACGCCGATAGCTAATCTTCTCGGTCGCAATCGCAGCTCTCATATGCTTCTCGGTCTCCTCAGTATTTCACCCAC
TGGTACCCTCGCCATCAATGACCTGACGGGCAGTATCGCTCTCGATCTTACACACGCAGCAGCCATTCCCGAAGATAGCGCCTGGTTTGCCCCT
GGGATGATGGTACTCGTAGACGGCACGTACGAGGAAGATGAAACTGGGACGTCATCTCGTCTTGGTGGAAATGGGGGCGTAGGGGGTACTATTT
CTGGAAAATTTGTCGGCTTCTTCATCGGCCACCCTCCCCCCGAACGCCGTCATGTGACTTTAGGCACAGCCGGTGAGGGAGATACCACAGCCGG
CGGTGGTTTCGGCTGGGTAGACTTCCTAGGCGTGGGTAGTTCTCGCGCCCTAGGCAACAAAATGCAACGACTGGAACAAAAGCTTCTCCGACCC
CCTCCCCCTGACACAGATACAGATGATCCCCCGCCTCCATCCAGAGGCCGTGTAGTCATTCTTGGTGATGTACATCTCGACATTCCTCAAACTC
TTCAAGCCCTCAAAAAGATTCTCTCTCTATACTCATCTGAACCAGAAGGCTGCACACCTATGACCTTTATCCTCCTCGGCTCTTTCGTCTCTCA
TGCCGTACTGGCTCGGGGCGGATCGGGAGGCTCTATAGAATATAAAGAGTATTTTGATTCCCTTGCAGCCGTTCTCTCTGAATACCCTACCATC
TTAAGTACAGCAACATTCATATTTATCCCTGGCCCCAACGATGCATGGGTTTCAGCCTTCTCATCCGGGTCTACTGTTCCTCTGCCCAGAAAAC
CCGTACCGGAAATGTTCACATCCCGCATAAAACGTGCCTTTGCAAACGCAAACACTGAAATGGAGAAAGAAAACGGTAATAAAGGAGATGGAGA
GGCCATTTGGACGAGTAATCCTGCAAGAGTCAGTTTATTTGGCATGAGCTGTGAACTGGTAGTATTCCGCGACGACGTTAGCGGACGATTGCGT
CGTACAGCTGTTACACTCAAGTCATCTCAAACGTCGAACCCTGAAAACGAAGATGAAGATATAGATATGTCACCACCTCCATCTTCAATTCCCT
CTTCCACCCCTCCTCCAGAAATCGATCCCGATATCCACACCGCGCGTCGTCTTACCAGGACACTCCTCGATCAGGGACACCTTTCACCCTTTCC
TCTAAACATCGCACCCCAGCATTGGGATTTCTCAAATGCGTTGAGCATTTATCCATTGCCAACAGCAATTGTTATGTGCGATGTAGATAGCCCG
GCATTTTGCTTGACGTACGAGGGGTGTCATGTAATGAATCCAGCGAGTGTAGTAGCAAAGGGGAGAAGAGGCGTAGCGAGATGGATAGAGTATG
ATGTTTGGGGACGGGCTGGGAAGGTCAGAGAGGTGGGATTTTAG

>Bcin11g01310(MLST5), partial sequence [organism=Botrytis cinerea, strain D08_H_8_4]
ATGCCTTCCATTAGCAGCATTCTAGTCGCCACTGTCAATTTGTCGGTCCTTGTGAATGGGCTGACATGGACCTCATGTGGAACCGGCGTAGAAT
GCGCAACGCTTGAAGTTCCGCTCGAATATGGCGATGCAACGTCAACGGCAAAAGCCAGTGTTGCGCTTGCTCGTTATCCTGCCACTGTTGCCGC
GAGCAAGAAGCTCGGGTCTCTCTTGATAAACCCCGGTGGACCCGGTGCCTCTGGTGTTGGCTTTGTGCAGTCTGGAGCCGGTGCCGCCGTCTCG
ACACTGAGTGGTGGATTGTACGATATCATCGGATGGGATCCACGTGGAACCGGTGCTTCGGCTCCTATTTTGGAATGTTTTGCAAATGCCAGTG
CGGAGTATGATTTTAACAACGCGTTTCCATCTGCTCCGAATCTCTGGCTCGGACAATTTGCGAATGCCAGCGCAAATTCTGCTGTTAGCTCTGC
TATCACATCCTTTGACACTTCTGTCGCTGCTCTTGCAAAAGCTTGCGTGGCTCAGAAATCTCCCGCTCTTTACACCTCAACAGCAGCATATGTT
GCTCGAGACATGGCAGCGATAGTCGATGCATTGGATGGGACCTCTGCAAAACTTAACTACTGGGGTTTCTCATATGGAACTATCTTCCTAGCTG
AGTTTATCCAAACTTTCCCAGGCCGCGTGGGAAGAGTTCTTGCCGATGGTGTTTTCGACGCAAAGGCAAATGCACTCACATACGTTAGCCAACT
TCCCAACGATCAACTCAGTGTTCGTGCTTCGTTGAACGATTTTGCAGCTTTCTGCACCACCGCCGGTAGTAAAGGTTGCTCTTTTGCCACCGCC
CCTACTGGAACCTCAGGTACTGTTGCTACCAGACTGGACAACATAATGAAGGATATGTTCCTCAATCCTATTGTTGCTTCGGGCTTAAGCATCA
GCTTAGATATCCTCAGTCCCGTTCTTGCATCTCTTCTCAGAGTTCCAACCACGTGGAAAACGCTTGCATCTGTCTTATCCGGTCTTGAAACTCG
TGACGCAACTGCTCTTATTTCACTCCTTGGATCGCTAGCAGCGAGTGCACCAACCGATGGCTCTGCAGCAGGCGTTGGCACTCTTGCTACTTAC
CCACTTGGTTGTGTGGATAACGCTGCTTCAAATGGAGTTACCTTGGACACTGTCATTTCTCTCACCAAAAGCATCTCAATCTCCGAAGACACCC
CAATACTAAATGCTGGACTTATCCCCATAACATTTTGTCGCAACTTCCCTTCCACGCGTCCGCTTGTTCCAAACGTAGGAGTAAGTTTGATGTC
GAAGACCGATACTCTTCTCGCAACAGCCAAAACACCAATCCTCATTGTCTCGGCCGAGAATGATCCAACAACCCCTCTCAAGTCTGCAAAAGCA
CTCCGAAGCCTCCTCCCTAGTTCATCCACCCTCGTATACCGCGGAGGAAGCGGACACACCACCATCTCACACGCATCTCTCGGAATGGCAAAAG
CGATCTCCAATTTCTTTGTCAGCGGTACCATGCCCACGGACGGAGCTCGATTTGCAGTGGACCAGAATATTTTCCCAACAGCTGCAGCGAGTGG
TTTAGTTACACCAGCTGCTTTCAACGGAACCTATTCTACACAAGATCAAAGTTTCCTGACCGCAACATACAACATTGGCATTGCCTTCTTAGCA
ATCGCATAA

>Bcin15g03910(MLST6), partial sequence [organism=Botrytis cinerea, strain D08_H_8_4]
ATGGCGCCCATTACCAAAATTATCTCGTTGCTCAGCAGCGCATCGCTCATCAACGCTATCGTTATGCCTGAGAGTGTTCTTGAATTTCCCGTCG
CGCATGATGAACATGCCTCTGTCTACGATAAAAACAGTCCTCTTCCAAGTATTTTCAACCTCACCTTGTCCGGAAAGACTCGCCAAAACACAAA
ATCATCCAACGATGAAGATGATACTCCACTTCCCTTGATTATCTGGCATGGACTCGGCGATAATTACAAAGCGGATGGTCTTGCGCAAGTTGGA
AAACTAGCTGAAGCTATTCATCCTGGGACTTTTGTCTACAATATTCATGTAGATGAGGATGCATCTGCAGATAGGACAGCTACCTTCTTTGGAA
ATCTCACTCGTGAGTACATCCCCTATTTTTCCTTTAAATATCATACTAACTCTCTTACCAAGTTCAAATCGAAAAGGTCTGCGAAGACCTCGCC
TCCCATCCTATTCTCTCTACCGCGCCCGCCGTCGACGCAATTGGATTCTCCCAAGGCGGCCAATTCTTGCGTGGTTACATATCCCGCTGCAATG

CTCCACCCATCCGCTCTCTCCTGACCTTTGGTTCCCAACACAACGGCATTTCTGCCTTCCAAGCCTGTGGTCCTGCCGATTTCCTCTGTCGCGG
TGCTCAAACCCTTTTGCGATCCAACACCTGGTCAACCTTTGTCCAATCTCGTCTCGTACCCGCTCAATACTTCAGAGATCCGGAAAACCTAGAC
TCTTACCTTGAATATTCCAATTTCCTTGCCGACATCAATAATGAGCGCGTTCTCAAGAACCAAACATATAAATCCAACATGGAAAAATTGGAAC
GATTCGTAATGTATGTCTTTGAAGACGACACAACTGTCATTCCTAAGGAAAGTGGATGGTGGGCTGAAGTCAACGGCACGGAAGTTACACCACT
GAAAGAAAGAGCCATTTATAAAGAAGATTGGCTAGGTTTAAAGACATTGGATGAGGCCGGAAAATTAGTTTTCGAAACCATTCCAGGGGGACAT
ATGACGTTAGGAGAGGAGATGCTAGAGAAGGCTTTCAAAGAGTATTTTGGTCCAGCAGGGAAGAAATTTGGGGAGAAGCAAGCCCAGATGACGG
GACACGAGGAGGAGTTGTAA

>Bcin16g03460(MLST7), partial sequence [organism=Botrytis cinerea, strain D08_H_8_4]
ATGCTTATTTACGCTTTCGCATTGATACCTCTTTACGTCCTAGTATGGGTGCCTCTTTCTCAAGTAATCTACGGTCGTCAATCTGCTCTTCATG
AACCTCTTGATTTGAACTCTTCATTAATTGCAAGTGATGAACCCCTATTCTGCCCAGAGGATAGTTACAAGACGTATATCATTAGTCGAGAACC
ACTCATGATATATATTGACGGATTTTTGAAAGCGAATGAAAGTAAACATTTGGTTGATGTTAGGTGTGTTATTCATTCTGATGAAATGAACAAG
AGAGACTGATGAGATAGTGAACCGCTTTATGAACCATCTACTGTTTCTCACGGACAGGAAGTTACCATTGATCCTTCAGTTCGGAATTCTGAAG
TGGCGGTTTTAGAGAGGGATGAGGTGGTCAGGTGTATTGAGCATAGAGCGAGGGCATTTCAGGGGTGGAGGGGCGAGATGGGGATTGAGAAGTT
GAGGACGCAGAGGTATGGGGTTGGAGGACATTATGGGATGCATTTGTAAGTTTTGGGGGATTGACGAAGGCTTTTGTCTATTTTCTACCAGTAC
GGATCTTGAAAGAAAGATAGCATGAACACGAGGGCTAATAACTAGGAAATAGCGATTGGAGCGGAGGTAAACGTGGCATAGACCGATTTAGTAC
TTTCATGGTCTATGTCGACGTATCCTCTGATATCGAAGGTGGAGGAACGGAATTCCCACGTATTGTGGGACCAAAAGGAGGAAGGTGGGAGGAC
TTCCTGGAAACTACGGAAGCATTGGATCCAAGAACTGGAGAAAATGTAACAGTAGAAGGGGTGACATTCAAACCAATCAAGGGAAATGCCGTAT
TCTGGGAAAATACTGACAACAACGGGAGGGGCTATGATGAAACATGGCACGCTGGTCTACCGGTGGAAAAAGGCTCGAAAGTAGGGTTGAATAT
TTGGAGTTATGGGAGGACCATTAGATGA

>Bcin12g03020(MLST8), partial sequence [organism=Botrytis cinerea, strain D08_H_8_4]
AGCACGAATAAACCATCGGCAAGGGAAATATTCTCTGAACCACAACATGACACGAGCGCGGAAGAGTATATCGGGCGAGAAGCTTCATCAAGAG
CACCAAAGCGACAACGAGTCGATGATAATTATAACTCTTACGGTGGAAGAAATGAAAATACAGCAGCTTATGCTTCCGGGAAACTTCCATCTGG
AAGTATAAATGTTGGTGGAGGTAGGAAGACCACTTTTCAAGAAGAACCTCGAACGGCATTTGTCGCTGGCAAGTTGCCCCCTGGAAGTATCAAC
ATTGGTGGAAAGAAGGCTACCCAAATCGAGGACGAGGGTAGAGCAGCTTATGCTTCCGGAAAATTGCCCCCAGGAAGTATAAACTCTGGCGCAA
AGAAGGCGATTTCATTCCAAGATGAAACGAGACCGGCTTATGTCTCCGGAAAGCTTCCACATGGTAGTATCGACGGTATGCGAAACCGTGAAAT
GGCTGCCGTCCACCGCGAAATTGCTGAGGGTGGGAGGAAACCAGGCCAGGTTGTCTCTTCTCTATTCACATTCAATCCTACTTCAAAGAAAACT
TTCGATGAACCAGAAGAACAAGCGGAACCGGCAAAACCATCCAATGCGCCTTTGACAGAGGAAATGGCGACTTTCACCAATCTAGGGCTATCGA
GAAGGCTTGCAGCCCATCTATCGACTAAACTCGATATGAAAGCCCCGACGGCCATTCAAAAAGCATCTGTGCAGCAGTTGGTATCGGACGATAG
CGATGCTTTCATACAAGCAGAGACTGGATCTGGAAAAACTTTGGCATATCTACTACCTATAGTCGAGCGAATATAGCATTGAGTGAGATGCTAA
TCGCG

>Bcin02g07770(MLST9), partial sequence [organism=Botrytis cinerea, strain D08_H_8_4]
ATGGTTGCCTTCTCAAAATCATTACAGCTTTCCCTTTCGGTCTTGGCATCTACAGTCATTGCCATCCCTACACCCTCACAACTTGAGTCTCGGG
CCGTTATCGATTCCGATGCCGTTGTAGGATTTGCCGAAACTGTTCCCAGTGGGACCGTAGGAACAGTTTATGAGGCATATAAACCATTCCTTAA
AGTCGTAAATGGATGCGTACCATTCCCTGCCGTCGATGCATCGGGTAACACAGGGTATGTCCTTATACCTTTCTCTTCCACACGATTGCTATTG
AGTCTCTAATATATTTTAGTGGTGGTTTGTCACCAACTGGCAGCAGCAATGGTGAATGCAGCAGCAGTACCGGTCAAGTATATGTTCGAGGAGG
ACAAAGCGGATCAAACTACGCTATCATGTACTCGTGGTAAGTTCTCTTTAAACTTCTCCTTATAGATCCAACCTAACAAAATCTTAGGTACATG
CCAAAGGACGAGCCCTCAACCGGTATTGGTCACCGTCACGATTGGGAAGGTGTAATTGTCTGGCTCTCCAGCGCCACCGCCACAACTGCCGACA
ACATCTTAGCCGTTTGTCCTTCCGCCCACGGAGGCTGGGATTGTTCCACGGATGGCTATTCCCTTTCTGGTACCAGCCCTCTTATCAAGTACGA
AAGTATCTGGCCCGTCGATCACTCAATGGGTCTTACTAGTACTGTTGGTGGAAAACAACCTATGATTGCTTGGGAGTCTTTACCAACTGCTGCT
CAAACTGCTCTTGAGAACACCGATTTCGGTGCTGCGAATGTTCCATTCATTCCGGCTGTTTTCACAGATAATCTTGCGAAGGCTACTTTCTAG

>Bcin04g02090(MLST10), partial sequence [organism=Botrytis cinerea, strain D08_H_8_4]
AATTGTCGAATATGACTATGAATGATGTTTACAAGCCCTACATCCATGTAAGAAATGTAGAATAAAGAGATCAGTAACTGGAACTAATATCGTT
TGCAGGCTTTCAAGTTACTTACGCAGTTCAACCCAATCACTACAGCTATTGCCGAATCCCCACTATTTCAAATGGCTGTCTCAGCAAATACCAT
CGAAAAGTACACACTGCTAGGCCCTTTCTTCAGAATATCTCCTCTGCAACAGGAAGTTACCAGGGAATACTTCAGTGCGCCAAAGACGATAGAT
AGGCGACACATTGCCACATCTCAAGATGCGTTACGATTGACCTTACAAACCCATCAAAAAGATTTACTTGATATCATCAACCACTTTGTTCGAG
CAAGTCCAATCGCAAAAAGCAAAACCCTGGATTGGTTCGCCTACATTGTGAATCAAAATCACAAGCGTCGAGCACTTCAGGTAGACCCGAAAGA
AGTGTCTTCTGATGGCTTTATGCACAATGTCACTGTCGTTCTAGATGGTCTTTGTGAGCCATTCATGGATACCACATTCTCAAAAATTTCGAAG
ATTGATATTGATTATCTAAGACGTGCGCCTCGTGTAGATATCAAGGACGAGACCAAGTTGAACGCTGATGAGAAGGCTTCTGAGAAGTATTATG
AGGACACTGTTCCTGGCACTTCTAATTTCATCTCTGAGGTATTCTTTCTGACATTGGCTGCTCATCATTATG

>Bcin01g07220(MLST1), partial sequence [organism=Botrytis cinerea, strain D08_H_6]
ACGAAGTTTTGGATAGGAACTCTGAGGAAATGCGTAATCTCGTTGGCCGCTTTGCTGGTATTGTATTCACAGCATTTTTTATGCTATTGATATC
AATCATTTGGGCTTTCGTGAATACATGGAAACTGACATTAGTCTCAATGGCAACTGGGCCAGTTATATACGCTGTCACCAAAACGTTCAATCGC

GTGAGTGGAAAATGGGAAAACAAGTGCAACTACGCATCTGAAATGACCACTGGCATATTTTCAGAGACTTTCTCCAACATCAAAGTGGTTCGGG
CTTTTACTCTGGAAACTTACTTTGAGACAAAACACACCAAAGCTACAGAAGAACTCTATAAAGTTGGACTAATACGAGCAAACTACTCGGGATT
GCTGTGGGGATTGACAGATGCGATGTCATTCTTCATCACTGCAACTATCTTTTATTATGCCACGGTTCTCATTACCAAGAGAGAGATCAGTATT
GCGACTGCACTACAGACTGTCAATCTTCTATTATTTGGTATTTCTAATAGTACGAATATGCTGGCTATGATACCACAAATCAACTCTTCTCGCG
TTACAGCTACGCATATGCTTGCATTAGCCAATCTCGATTCATCTTCCTCCCACGAAAATAAAGGAACCGAACGGCTTTCGACAATCTTTCCAAT
CAAATTCAACCGTCTTTCATTCACATACCCTACTCGTCCTGAAAAACGAACGATATCATCCTTTTCTCTTTCCCTGATTCCTAACTCAACAACT
GCACTTGTCGGACCCTCCGGCTCCGGAAAATCTACGATAGCTGCTCTGCTCATTGGTCTCTATCCGCCAGATACTTCAACACC

>Bcin05g07690(MLST2), partial sequence [organism=Botrytis cinerea, strain D08_H_6]
AGGATGCCTTCTCTCTACGACAGCAAGCCTTCGATGATATGTTGTGGGTAGTTCTTGGCTGGCTGGATACTGTCAAATTCATTGATTTACATTC
TGAATTGCACTATTCAAACGACTCTCAGCCAGAATGGTACGGACAACAATATAAACCTGCATTTGCACATCGAGCGCGACTATTTTGGGAATTG
GCTTCACAAGGATGGGATACTACTCTCTGTGGTGGTGGGATGATATGGTCACCATACCTTACTCCATACAAGAATGCAATTACCAATGAACTCT
ATATCGCAGCTTCGATATCGATGTACCTCTATTTCCCCGGAGATGACAATCAATCCCCATTTATGCTTTCCAACCCTTCATATCCACCTCACGA
TCCGAAATATCTACAGGCAGCTGTTGATGCTTACAAATGGCTGAATGGTTCCAACATGACGGATTTACAAGGATTATATGTCGACGGGTATCAT
ATCTCGAATCTTTCTGGCGGTGAAAACACCCATTGCGATTCTAGAAATGAGATGGTATATACCTACAATCAAGGTGTTTTGCTTACTGGACAAC
GTGGTTTGTATGACGCAACCGCCGCACGATCATACCTTGTGGATGGCCACAAACTCATCGCGAATGTTATTAATGCCACAGGCTATGACCTGAA
ACACAATGTTGCCATCTCACCGCCACCCAAAGATGGTTCCGCATTGGCAAAGTGGTTTGGCCTGGGTAGGAATGGAATACTGGAAGAAGGATGC
GATTCAAGTGCTTCGTGTTCTCAAAATGGACAAACTTTCAAAGGCATATTCTTTCACCACTTGATTGCGTTCTGTAGTGATTTGCCAGGGGAGC
CTATTGCAGGGACGAAGGAA

>Bcin06g01710(MLST3), partial sequence [organism=Botrytis cinerea, strain D08_H_6]
CAAGGCAATCACCTAATTTCATGGCGTTCTTCCCTGCATCATCTACCTACCCTGGAATGCTGGGAGAATTATACTCAGCAGCTTTCACAGCTCC
TGCTTTCAATTGGATCTGTTCCCCTGCTGTGACAGAATTGGAGACGGTTGTAATGGATTGGTTGGCCAAGCTTCTCAATCTCCCAGACTGTTAT
TTGTCGTCGACTCATGGTGGTGGTGTTATCCAAGGATCAGCCTCGGAAGCTATCGTTACCGTTATGGTTGCTGCCCGCGATAAGTATCTTCGTG
AAACCACTGAAGGTCTGTCGGGAATTGAACTCGAGGATGCGATTGCATATAAGAGGAGTAAGCTAGTTGCACTAGGAAGCGAAATGGCACACAG
CTCCACGCAGAAAGCAGCGCAGATAGCTGGCGTTAGATTCCGATCGATTCCAGTACTCGCATCCAATGATTTCGCCATGACGGGTGATGATTTA
GAGAAGGTATTGAAAGAATGCAAATCTCAAGGATTGGAACCCTTCTATCTAACTTCGACGTTGGGAACAACATCTACATGCGCAGTTGACGACT
TCGCATCTATTGCAACAGTACTTTCAAAATATGCACCTCCAGATGTTGCAGGCGAGATCTGGGTTCACGTCGATGCTGCTTATGCAGGTGCAGC
TTTGGTTTGCCCTGAATACCATCATCTAACATCGTCCTTCCAGCATTTCCATTCCTTTGATATGAACATGCACAAATGGCTTCTGACAAATTTC
GACGCTTCTTGCCTATATGTCAAGAAACGCAAAGATCTGATCGATGCACTCTCCATAACACCAAGTTATCTTCGCAACGAGTTTTCAGAGAGTA
GACTCGTAACCGACTATCGGGACTGGCAAATTCCTCTCGGAAGACGTTTCCGAAGCT

>Bcin09g03030(MLST4), partial sequence [organism=Botrytis cinerea, strain D08_H_6]
GCAGAGAGAGTCTTGGAGGAGGTCGCCAAGAGTTGGAAGAATAGGAGTGGCGGTGTCATTGTCGAGGGCGAGGGAACGGAATTGAAGGAGATTC
TGAAAGCTTTGGAAGGGAATATGAGTGGTGGAAGGATAGTCATAGGAAGAGAGCTAAGCCGGCAGAATAGTTTAGTACTGGGATCATCACAATA
TGGAGAGGTCAATCATACAAGACTTGGGCTACGGCCAGGGAATATACCCAGAGAGGATAGTCAGTCAAGTTTGGGAATGTCAACGTTGGAGGTC
AATGACGAGGAAGATGAGGATGGCCTGATGGATCCAAGAAGGTGGTTAAAAGTCGTTGATGCATTTGAGCAACCTCGACTGGTGTACAATGTTG
CTAAAAAGCACTTTGATAGGTATGTTTCAATGATAAAATTTTATCTGAATCGACTAACTCAGTACAGAGATACCTCCAAACCTTCATTGTTCCC
ACCTGCGTCTCATAAAACACTCCTCTTCCAAAACCGCTATAATGTTATCCATCAACGTCTCCTTCGCAATGAATCTTTTCAAACGCCCGCTTTT
CAAGGTGGCAAATCTTCCCTTCAACGCAGCACGTCCGCCATTACCACCCAACAACAATCATACAAATTAACGCCGATAGCTAATCTTCTCGGTC
GCAATCGCAGCTCTCATATGCTTCTCGGTCTCCTCAGTATTTCACCCACTGGTACCCTCGCCATCAATGACCTG

>Bcin11g01310(MLST5), partial sequence [organism=Botrytis cinerea, strain D08_H_6]
TCATGTGGAACCGGCGTAGAATGCGCAACGCTTGAAGTTCCGCTCGAATATGGCGATGCAACGTCAACGGGCAAAAGCCAGTGTTGCGCTTGCT
CGTTATCCTGCCACTGTTGCCGCGAGCAAGAAGCTCGGGTCTCTCTTGATAAATCCCGGTGGACCCGGTGCCTCTGGTGTTGGCTTTGTGCAGT
CTGGAGCCGGTGCCGCCGTCTCGACACTGAGTGGTGGATTATACGATATCATCGGATGGGATCCACGTGGAACCGGTGCTTCGGCTCCTATTTT
GGAATGTTTTGCAAATGCCAGTGCGGAGTATGATTTTAACAACGCGTTTCCATCTGCTCCGAATCTCTGGCTCGGACAATTTGCGAATGCCAGC
GCAAATTCTGCTGTTAGCTCTGCTATCACATCCTTTGACACTTCTGTCGCTGCTCTTGCAAAAGCTTGCGTGGCTCAGAAATCTCCCGCTCTTT
ACACCTCAACAGCAGCATATGTTGCTCGAGACATGGCAGCGATAGTCGATGCATTGGATGGGACCTCTGCAAAACTTAACTACTGGGGTTTCTC
ATATGGAACTATCTTCCTAGCTGAGTTTATCCAAACTTTCCCAGGCCGCGTGGGAAGAGTTCTTGCCGATGGTGTTTTCGACGCAAAGGCAAAT
GCACTCACATACGTTAGCCAACTTCCCAACGATCAACTCAGTGTTCGTGCTTCGTTGAACGATTTTGCAGCTTTCTGCACCACCGCCGGTAGTA
AAGGTTGCTCTTTTGCCACCGCCCCTACTGGAACCTCAGGTACTGTTGCTACCAGACTGGACAACATAATGAAGGATATG

>Bcin15g03910(MLST6), partial sequence [organism=Botrytis cinerea, strain D08_H_6]
ATGATACTCCACTTCCCTTGATTATCTGGCATGGACTCGGCGATAATTACAAAGCGGATGGTCTTGCGCAAGTTGGAAAACTAGCTGAAGCTAT
TCATCCTGGGACTTTTGTCTATAATATTCATGTAGATGAGGATGCATCTGCAGATAGGACAGCTACCTTCTTTGGAAATCTCACTCGTGAGTAC
ATCCCCTATTTTTCCTTTAAATACCATACTAACTCTCTTACCAAGTTCAAATCGAAAAGGTCTGCGAAGACCTCGCCTCCCATCCTATTCTCTC

TACCGCGCCCGCCGTCGACGCAATTGGATTCTCCCAAGGCGGCCAATTCTTGCGCGGTTACATATCCCGCTGCAATGCTCCACCCATCCGCTCT
CTCCTGACCTTCGGTTCCCAACACAACGGCATTTCTGCCTTCCAAGCCTGTGGTCCTGCCGATTTCCTCTGTCGCGGTGCTCAAACCCTTTTGC
GATCCAACACCTGGTCAACCTTTGTCCAATCTCGTCTCGTACCTGCTCAATACTTCAGAGATCCGGAAAACCTAGACTCTTACCTTGAATATTC
CAATTTCCTTGCCGACATCAATAATGAGCGCGTTCTCAAGAACCAAACATATAAATCCAACATGGAAAAATTGGAACGATTCGTAATGTATGTC
TTTGAAGACGATACAACTGTCATCCCTAAGGAAAGTGGATGGTGGGCTGAAGTCAATGGCACGGAAGTTACACCACTGAAAGAAAGAGCCATTT
ATAAAGAAGATTGGCTAGGGTTAAAGACATTGGGATGAGGCCGGAAAATTAGTTTTCGAAACCATTCCAGGGGGACATATGACGTTAGGAGAGG
AGATGCTAGAGAAGGCTTTCAAAGA

>Bcin16g03460(MLST7), partial sequence [organism=Botrytis cinerea, strain D08_H_6]
TCTTCATGAACCTCTTAATTTGAACTCTTCATTAATTGCAAGTGATGAACCCCTATTCTGCCCAGAGGATAGTTACAAGACGTATATCATTAGT
CGAGAACCACTCATGATATACATTGACGGATTTTTGAAAGCGAATGAAAGTAAACACTTGGTTGATGTTAGGTGTGTTATTTATTCTGATGAAA
TGAACAAGAGAGACTGATGAGATAGTGAACCGCTTTATGAACCGTCTACTGTTTCTCACGGACAGGAAGTTACCATTGATCCTTCGGTTCGAAA
TTCTGAAGTGGCGATTTTAGAGAGGGATGAGGTGGTCAGGTGTATTGAGCATAGAGCGAGGGCATTTCAGGGGTGGAGGGGCGAGATGGGGATT
GAGAAGCTGAGGACGCAGAGGTATGGGGTTGGAGGACATTATGGGATGCATTTGTAAGTTTTGGGGGATTGACGAAGGCCTCTGTCTATTTTCT
ATCAGTACGGATCTTGAAAGAAAGATAGCATGAACACGAGGGCTAATAACTAGGAAATAGCGATTGGAGCGGAGGTAAACGTGGCATAGACCGA
TTTAGTACTTTCATGGTCTATGTCGACGTATCCTCTGATATCGAAGGTGGAGGAACGGAATTCCCACGTATTGTGGGACCAAAAGGAGGAAGGT
GGGAGGACTTCCTGGAAACTACGGAAGCATTGGATCCAAGAACTGGAGAAAATGTAACAGTAGAAGGGGTGACATTCAAACCAATCAAGGGAAA
TGCCGTATTCT

>Bcin12g03020(MLST8), partial sequence [organism=Botrytis cinerea, strain D08_H_6]
ACTGCGCAGCACCGAGTCACCAAAAGCACGAATAAACCATCGGCAAGGGAAATATTCTCTGAACCACAACATGACACGAGCGCGGAAGAGTATA
TCGGGCGAGAAGCTTCATCAAGAGCACCAAAGCGACAACGAGTCGATGATAATTATAACTCTTACGGTGGAAGAAATGAGAATACAGCAGCTTA
TGCTTCCGGGAAACTTCCATCTGGAAGTATAAATGTTGGTGGAGGTAGGAAGACCACATTTCAAGAAGAACCTCGAACGGCATTTGTCGCTGGC
AAGTTGCCCCCTGGAAGTATCAACATTGGTGGAAAGAAGGCTACCCAAATCGAGGACGAGGGTAGAGCAGCTTATGCTTCCGGAAAATTGCCCC
CAGGAAGTATAAACTCTGGCGCAAAGAAGGCGATTTCATTCCAAGATGAAACGAGACCGGCTTATGTCTCTGGAAAGCTTCCACATGGTAGTAT
CGACGGTATGCGAAACCGTGAAATGGCTGCCGTCCACCGCGAAATTGCTGAGGGTGGGAGGAAACCAGGCCAGGTTGTCTCTTCTCTATTCACA
TTCAATCCTACTTCAAAGAAAACTTTCGATGAACCCGAAGAACAAGCGGAACCGGCAAAACCATCCAATGCACCTTTGACAGAGGAAATGGCGA
CTTTCACCAATCTAGGGTTATCGAGAAGGCTTGCAGCCTATCTATCGACTAAACTCGATATGAAAGCCCCGACCGCTATTCAAAAAGCATCTGT
GCAGCAGTTGGTATCGGACGATAGCGATGCTTTCATACAAGCAGAGACTGGATCTGGAAAAACTTTGGCATATCTACTACCTATAGTCGAGCGA
ATATTAGCATTGAGTGAGAATGGCGT

>Bcin04g02090(MLST10), partial sequence [organism=Botrytis cinerea, strain D08_H_6]
AACTGTCGAATATGACTATGAATGATGTTTACAAGCCCTACATCCATGTAAGAAATGTAGAATAGAGAGATCAGTAACTGGAACTAATATCGTT
TGTAGGCTTTCAAGTTACTTACGCAGTTCAACCCAATCACTACAGCTATTGCCGAATCCCCACTATTTCAAATGGCTGTCTCAGCAAATACCAT
CGAAAAGTACACACTGCTAGGCCCTTTCTTCAGAATATCTCCTCTGCAACAGGAAGTTACCAGGGAATACTTCAGTGCGCCAAAGACGATAGAT
AGACGACACATTGCCACATCTCAAGATGCGTTACGATTAACCTTACAAACCCATCAAAAAGATTTACTTGATATCATCAACCACTTTGTTCGAG
CAAGTCCAATCGCAAAAAGCAAAACCCTGGATTGGTTCGCCTACATTGTGAATCAAAATCACAAACGTCGAGCACTTCAGGTAGACCCGAAAGA
AGTGTCTTCTGATGGCTTTATGCACAATGTCACTGTCGTTCTAGATGGTCTTTGTGAGCCATTCATGGATACCACATTCTCGAAAATTTCGAAG
ATTGATATTGATTATCTAAGACGTGCGCCTCGTGTAGATATCAAGGACGAGACCAAGTTGAACGCTGATGAGAAGGCTTCTGAGAAGTATTATG
AGGACACTGTTCCTGGCACTTCTAATTTCATCTCTGAGGTCTTCTTTCTGACATTGG

>Bcin01g07220(MLST1), partial sequence [organism=Botrytis cinerea, strain D09_Bc11]
ATGACAATATTGAAAAGCAAAGGCTCGAAGAGTCACAAAAAATACAGTCATTCTACTTCTACCTCACGCATCCGATTGCCAGATGATGATCAGA
ATTCGTCCATCGAATTGCGAAATATCAATGACTACCACGCCGATGAAGAAATCAAATCATCTTTCAAATCCCTATTCACATTCACAACAAAAAA
GCACATATCTACCATAGTTCCATGTATAGTATTTGCAATCATTAGCGGCGTTCTAAAACCTATATCGGCAATATTTTACGGAAATATCTTTGGT
ACTCTTACAAATTTTGGGTCCGGTGTTCTCACCGCACAAGAGACCTTACAACATGTCTCGAAATGGTGCATAGCTATAACTGTTCTTGGGGGCG
CAGTATGGTTGTTCGAAGGCTTGTTTCTGTGTTCGTGGATGGTATTTGGAGAGCTCCAGGCTAGAAGCGTGCGCGAGAAAATGTTTGCCGGCAT
GTTGGAGAAGGATTTGGAATGGTTTGATCTGCGTAGAGATGGCATTGGATCATTATTAATTCGAATTGAAACGTGTGTGGAATCCATTTATGGT
CGAAATGAAAACATACTGATTGAACAATAGACAAATTCGAGAGCTCCAATTATCCACATCCCAGCCTCTTGGGTTTCTCCTGTTTGAGACTGCT
AGCGCATGTGCAGCTCTTGGGACTGCCTTCTTTTACTCTTGGAATCTGACTCTTGTCATTATAGCGACATTTCCAATTGCCGGTGGAATACTTT
ATCTAATTTCTAGAAACATGGGCCCAGCCATCGAAGCCCAGAAAAGAGAGCTATCGCAAGCTTCGAAATTTACAAATACAGCCATTACAGCCAT
CGATACTGTCAAAGCATTCAACGGACAGGACCAGGAAGTTTGGCAATACTTCTTAGTAATCAAAAAATCAACAGTTCATTACATGATCCAAGCC
AGGTCAAATGCATTTCAATTTGGGATTACCAAATTTGTCATGGTGGCCATCTTTGTTCAAGGCTTTGGGTATGGTCTCACGTTAGTCGATCATG
GACTAGATGCCGGAAAGGTTCTTACAACTTTCTATGCTTGTTTGACTGGGATGATGGCTATAGAAGTCGTTTTACCACAATGGCTTGTCTTAGC
AAAAGGAATGTCCGCTGGAGCGACTCTCAAGTCTATCATGAACCAAGTGGATAGAGGAGGAGTTGCAAAACACCCAGAAGGGTTAATGGTACCT
AAAACCTGCAACGGTGATATAGAAGTCAATAATGTAAGTAGAGATGGCCACGATAAACCATCAAGACTAATCAAGATCAGGTTACATTTGGATA
TCCTTCCAATCGACAGCACAATGCTCTTATAAAAACAACATTCTTCTTTCCTGCTGGCGAGACTACTTTTGTAGTTGGTAAAAGTGGTTCTGGA

AAGAGTACATTGGGAAACTTGTTGCTCAAATTTTACGAGCCACAGGAGGGAAGTATCTTGATAGATGGCCAACGTATACAGGATCTCGATACCG
ATTGGCTGAGACGCAACATCACTCTCGTACTGCAGCAGAGTGTGGTATTCAACGAGACGGTTAGGAAAAACATAGAATTCGGCAAGGAAGGAAT
AAAAAATGAAGCTGGTATCATAGACGCCTGTTCTACAGCCAATCTAGAACAAGTAATTGCCGATCTTCCTGATGGACTTGATACTATGATTGGC
TCTAAGGAGAGACAACTGAGTGGTGGACAAAAACAACGTGTTGCATTGGCACGAGCTAGATTACGAGATGCACCAATCTTGATTCTTGACGAAG
GAACTAGTGCTCTTGATCTGACCAACCGACTCAAAATCATGGATAATATTCGAGAATGGAGGAAAGGAAAAACGACAATTATTGTGACTCATGA
TATTTCCCAGATATTGGATGATGACTACGTTTACGTTATGGATCAGAGTAAGGTTGTCCAGGAAGGATATCGTAGGAAGCTTTCTGCGAAAGCA
AACGGTACTTTCGCTACATTTTCGCAACTCCCGGGATTGCCCGTTGAACAGTCGACAGACTTGTCCAACATCAGAAGAAACTCGGACCCTGGAA
CACCATTAACTGGAAGCTTTGAAGACTTTGTTCAAGAATTGAAACCTCGTTTCTCTACCAAGTCTACAATCTATGGTCCAAATGCAGCAAATCG
TAGGTCCTTGGATATATCAAACAGACGATTATCTCTTGGATATGCTTCAATAGCTTATGCCAATGACCTACAATCAGATAACATTTGGACATCT
GGAATTGACAGTTCACGTTCAAGGTTTGATGATTTGCAAAGACCTACCTCACAGTTTCAGAAGTTCCAAGAGTCGGCTAGTAGGCAGTCTATAC
ATCCTATCAGGCCACCAAGGTATCAAGACGCAGGATTCCAATCGATCCCAGAACATCCCTCAGACAAGCCAAGCTTTGATAGAAAACCTCTGAT
TCAAAGCCCTCTCAGCTTGGTATCAATCGAAAAGAATCGAGACTCTAAAGTCGCGCCTATGGATTCTTTGTTGGAACACTCAGATGACTCTGCT
GATAATGCGGGCGATTCTGAAGAGAAGAACGAAACGGGTCATCTCAAACCCGATACTTTAACTAAGATCTTAAAGACTGTTTGGCCCATGCTGA
ACAAGAAGGAACGCACAATCCTTTCGGTTGCATTTGCCGCAGCATTTATTGTGGCAGTATCAACCCCAGCTTTTGCTATAATATATACCAAACT
TCTTGACACTTTTTACCAAAAGGAGAACCGCAACTCGAATGCTTTAAAATGGTCACTTGTCTTACTCGGAATCGCCATCATTGATGGCATATCT
TGTTTCTTTTCGCATTATGCTTTGGAACGTGCTGGACAAGCTTGGGTTAGTGCTCTGCGTGTAGAAGCACTAAAGAAGATTCTCGCACAACCGA
AGCCATGGTTTGAGGAATCCAGGAATTCACCTAGCCGGTTGAACGAAGTTTTGGATAGGAACTCTGAGGAAATGCGTAATCTCGTTGGCCGCTT
TGCTGGTATTGTATTCACAGCATTTTTTATGCTATTGATATCAATCATTTGGGCTTTCGTGAATACATGGAAACTGACATTAGTCTCAATGGCA
ACTGGGCCAGTTATATACGCTGTCACCAAAACGTTCAATCGCGTGAGTGGAAAATGGGAAAACAAGTGCAACTACGCATCTGAAATGACCACTG
GCATATTTTCAGAGACTTTCTCCAACATCAAAGTGGTTCGGGCTTTTACTCTGGAAACTTACTTTAAGACAAAACACACCAAAGCTACAGAAGA
ACTCTATAAAGTTGGACTAATACGAGCAAACTACTCGGGATTGCTGTGGGGATTGACAGATGCAATGTCATTCTTCATCACTGCAACTATCTTT
TATTATGCCACGGTTCTCATTACCAAGAGAGAGATCAGTATCGCGGCTGCACTACAGACTGTCAATCTTCTATTATTTGGTATTTCTAATAGTA
CGAATATGCTGGCCATGATACCACAAATCAACTCTTCTCGCGTTACAGCTACGCATATGCTTGCATTAGCCAATCTCGATTCATCTTCCTCCCA
CGAAAATAAAGGAACCGAACGGCTTTCGACAATCTTTCCAATCAAATTCAACCGTCTTTCATTCACATACCCTACTCGTCCTGAAAAACGAACG
ATATCATCCTTTTCTCTTTCCCTGATTCCTAACTCAACAACTGCACTTGTCGGACCCTCCGGCTCCGGAAAATCTACAATAGCTGCTCTGCTCA
TTGGTCTCTATCCGCCAGATACTTCAACACCTCCACCGTTGACATTCAATCGCGTCTCCATAAGTAACTGTCACATTCCGTCTCTCCGGGCTTC
TCTCTCACTCGTTCCACAAATACCGATTCTATTTCCAGCTACCATTCTCCATAACATCATTTATGGTCTCCCAGAATCTTCTCCTTGTGCTAGT
CTTCCATCTGCTATTGCATCAGCAAAAGATGCTGGGATCCATGAATTTATCACATCGCTTCCACAAGGTTATGATACTATGGTAGGAGATGGAG
GACAAGAGTTATCGGGAGGACAGGCCCAGAGAATTGTGATATCGAGAGCACTGGTCAGAAAACCAACTTTATTGATATTAGATGAAGCGACGAG
TGGATTAGATGGTGGTAGTGCAGAGGTGATAAGAGAGACTGTGCAGAAGTTGAAGGAGAGAGAAGGAATGGCCACGCTGGTTGTTAGTCATACG
GCTGAAATGATGAAGATAGCGGGACGAGTCGTGGTGATGGAAGAGGGAGAGATAGTTGAGAGTGGTGGTTTCGATGAGTTGAAGAATAAAGTCG
AGGGGAAATTCAGGACTTTAGTTAGAGATGATTCGGGTGATAAAGTGGTGCTGAAGGAAGAGAATGATGCTGGAGAGGCTAGTAGTAACGCTAG
CAGCGATGCTCACAAAGGTGGGTTGGGTATTAGAATTCCTGGTTACGATGAGGTAGAATTCCATACTGGAAGAGACACGCCGATTCGAGATGCA
AGAAGGAGAGATACTTGGCTGCGGCAAAACAATCATTGA

>Bcin05g07690(MLST2), partial sequence [organism=Botrytis cinerea, strain D09_Bc11]
ATGATTTCGTTCTCAACACAGATCAATCCACGACCGCGTATATCTTTGCTACTGCTGTGCTTATTCTGCGCACTATCTTTCTCAATTGGCGTAG
ATGGGAGGCCCAACGATGTTGTGCGACAGAATGATTTGATTCAACGACCACTTACGAATAATGAAGATATCACAATCAATGGGAGCCCTCAGGA
GCTCATCAAGCCTGGAGACTCAAAGGTTCTACCGGCAATGTTAAGCGCGCTGGACGTGCTACAAGAAGACTACTTTGCGACATGGCAAGGCATC
TATCCAACAGGAATCGATTGGACATCTGCAGTGATTGGTACAATACTTTCTTTGCATGTTGAAACCACTTTAGAGCAAGAGCTAACACCACTCT
CTAAACAGGCACCTATGTTGCTGGTGCACTCACCACTATCACCAAGTCCTTTTCTGCACTTTCCTCATCAAAAACGAACGAGAACATCATCAAT
AAATACTTTGCTGAAGTAATTGGGTTTTATTTTGGCCAGGATGCCTTCTCTCTACGACAGCAAGCCTTCGATGATATGTTGTGGGTAGTTCTTG
GCTGGCTGGATACTGTCAAATTCATTGATTTACATTCTGAATTGCACTATTCAAACGACTCTCAGCCAGAATGGTACGGACAACAATATAAACC
TGCATTTGCACATCGAGCGCGACTATTTTGGGAATTGGCTTCACAAGGATGGGATACTACTCTCTGTGGTGGTGGGATGATATGGTCACCATAC
CTTACTCCATACAAGAACGCAATTACCAATGAACTCTATATCGCAGCTTCGATATCGATGTACCTATATTTCCCCGGAGATGACAATCAATCCC
CATTTATGCTTTCCAACCCTTCATATCCACCTCACGATCCGAAATATCTACAGGCAGCTGTTGATGCTTACAAATGGCTGAATGGTTCCAACAT
GACGGATTTACAAGGATTATATGTCGACGGGTACCATATCTCGAATCTTTCTGGCGGTGAAAACACCCATTGCGATTCTAGAAATGAGATGGTA
TATACCTACAATCAAGGTGTTTTGCTTACTGGACAACGTGGTTTGTATGACGCAACCGCCGCACGATCATACCTTGTAGATGGCCACAAACTCA
TCGCGAATGTTATTAATGCCACAGGCTATGACCTGAAACACAATGTTGTCATCTCACCGCCACCCAAAGATGGTTCCGCATTGGCAAAGTGGTT
TGGCCTGGGTAGGAATGGAATACTGGAAGAAGGATGCGATTCAAGTGCTTCGTGTTCTCAAAATGGACAAACTTTCAAAGGCATATTCTTTCAT
CACTTGATTGCGTTCTGTAGTGATTTGCCAGGGGAGCCTATTGCAGGGACGAAGGAAAGCTTAGAACTCGACAGAGTGTGGCATTCTGACAAAT
GCTCACAGTATACAAAATGGATCAGGCGAAATGCCGAAGCTGCGTTAAGTACCAAGAATGAAGAAGGGAAATTTGGTATGTGGTGGGGTGTACC
GGCTACACAGAGTTCTTTAGCGGACTATACACAGGACCAGACATCGAGAGGCTCTGTAGATTATCGAAATACTGGAGTTCCAAAGAACACCGAA
TGGAGAGGAGAAGAATACCCCAGAGAAGCGAAAGCGAGCAAGACTAAAGAAGGTGCAGATGTATATAGTGGGGTAGAAGATCCCAATGATCGAG
GTAGAGGAAGAACGGTAGAAACTCAGGGTGGAGGATTGTCTGTCTTGAGAGCATTATGGGAGGTAGAGCTGAGATGA

>Bcin06g01710(MLST3), partial sequence [organism=Botrytis cinerea, strain D09_Bc11]

ATGGATTCGAAGCAGTTTAGGGAGGCGGCGACGTCGGCGATTGACGAAAGTAAGTTCCTGCTTCCCAAGATGAAGTACTGTATACTGACCAGCA
TAGTCATCCAATATTATGATAACATTCATGAACGAAGAGTAATTTCAAATGTCGAGCCTGGTTACCTGAAGAAGATTCTTCCAGATGGTCCACC
GGAAGAGGGTGAATCATGGGCAGAGATCCAGAAAGATATCGAATCTAAAATTATGCCTGGTCTGACACATTGGTGAGTCTGACTTTTGTATTTG
AGCGTTAAGATAGACACTGATATACCAAGGCAATCACCTAATTTCATGGCGTTCTTCCCTGCATCATCTACCTACCCTGGAATGCTGGGAGAAT
TATACTCAGCAGCTTTCACAGCACCTGCTTTCAATTGGATCTGTTCCCCTGCTGTGACAGAATTGGAGACGGTTGTAATGGATTGGCTGGCCAA
GCTTCTCAATCTCCCAGACTGTTATTTGTCTTCGACTCATGGTGGTGGTGTCATCCAAGGATCAGCCTCGGAAGCTATCGTTACCGTTATGGTT
GCTGCCCGCGATAAATATCTTCGTGAAACTACTGAAGGTCTGTCGGGCATTGAACTCGAGGATGCGATTGCATATAAGAGGAGTAAGCTAGTTG
CACTAGGAAGCGAAATGGCACACAGCTCCACGCAGAAAGCAGCGCAAATAGCTGGCGTTAGATTCCGATCGATTCCAGTACTCGCATCCAATGA
TTTCGCCATGACGGGTGATGATTTAGAGAAGGTATTGAAAGAATGCAAATCTCAAGGATTGGAACCCTTCTATCTAACTTCGACTTTGGGAACA
ACATCTACATGCGCAGTTGACGACTTCGCATCTATTGCAACAGTACTTTCAAAATATGCACCTCCAGATGTTGCAGGCGAGATCTGGGTTCACG
TCGATGCTGCTTATGCAGGTGCAGCTTTGGTTTGCCCTGAATACCATCATCTAACATCGTCCTTCCAGCATTTCCATTCCTTCGATATGAACAT
GCACAAATGGCTTCTGACAAATTTCGACGCTTCTTGTCTATATGTCAAGAAACGCAAAGATCTGATCGATGCACTCTCCATAACACCAAGTTAT
CTTCGCAACGAGTTTTCAGAGAGTGGACTCGTAACCGACTATCGGGACTGGCAAATTCCTCTCGGAAGACGCTTCCGAAGCTTAAAGATTTGGT
TTGTCCTCAGAACCTACGGAGTCAAGGGCCTACAAGAGCACATCCGAAAGCACGTGAAGCTCGGAGAATTACTTGTCAGCCTGCTCAAGACACG
AGAAGATCTATTCAATATAGTTACAGGGCCCAATTTCGCCCTCACTGTTCTGAACATTGTTCCCAAATCCACAAATGCGGATGCACAGAATAGT
CTCACGAAAGAAGTCTACGAGCTGATTAACAAACGAAGCGAAATCTATCTCACAGCTGGCGTGGTGGCGGGTGCATATGTAATCCGAGTCGTGA
GTGCGAATCCCAAGGCGGAAGAAAGTTATATCCGCAAAGCTTTTGATATCCTAGTTGATACGACTGAAGAGGTCCGGGATGGGAAAGCGAGCAA
GCGGGGAAATCTCAAAGGGGTTGTTATGAATAGGAAGGCGGAGGGTGTGGGTGAAGTCGCTGTTGTTAATGGGAATGGATTACCAAATCAGAGC
TAG

>Bcin09g03030(MLST4), partial sequence [organism=Botrytis cinerea, strain D09_Bc11]
ATGGCTCSCCYCMATCCCACAACGAAATCAAAAGCCCCTCCGCTCTTCAGGCCTCCTGCTACCCCCGCTACAAATTCGAATCCTATCCCCTCAT
CATCGCCTGCATTTGCAACTCCGGTACATCCTATACGACCCTTCAACCCCACCGCAGCTCCTATACCGAAAGCGAGTATCCTACCAATTCTCCT
TCCACCTGCGACTTTAAGACCATTGGCTTTCCGCACTTTCACAAAAAAGCATAGTTTAACATTGACGTCGTCGGCATTACAAGTGTTGGCTACT
TTTATTGGAAAGCATTGTGGGACAGGATGGAGGGAAGAAGGACTGGCAGAGAGAGTCTTGGAGGAGGTCGCCAAGAGTTGGAAGAATAGGAGTG
GCGGTGTCATTGTCGAGGGCGAGGGAACGGAATTGAAGGAGATTCTGAAAGCTTTGGAAGGGAATATGAGTGGTGGAAGGATAGTCATAGGAAG
AGAGCTAAGCCGGCAGAATAGTTTAGTACTGGGATCATCACAATATGGAGAGGTCAATCATACAAGACTTGGGCTACGGCCAGGGAATATACCC
AGAGAGGATAGTCAGTCAAGTTTGGGAATGTCAACGTTGGAGGTCAATGACGAGGAAGATGAGGATGGCCTGATGGATCCAAGAAGGTGGTTAA
AAGTCATTGATGCATTTGAGCAACCTCGACTGGTGTACAATGTTGCTAAAAAGCACTTTGATAGGTATGTTTCAATGATAAAATTTCATCTGAA
TCGACTAACTCAGTACAGAGATACCTCCAAACCTTCATTGTTCCCACCTGCGTCTCATAAAACACTCCTCTTCCAAAACCGCTATAATGTTATC
CATCAACGTCTCCTTCGCAATGAATCTTTTCAAACGCCCGCTTTTCAAGGTGGCAAATCTTCCCTTCAACGCAGCACGTCCGCCATTACCACCC
AACAACAATCATACAAATTAACGCCGATAGCTAATCTTCTCGGTCGCAATCGCAGCTCTCATATGCTTCTCGGTCTCCTCAGTATTTCACCCAC
TGGTACCCTCGCCATCAATGACCTGACGGGCAGTATCGCTCTCGATCTTACACACGCAGCAGCCATTCCCGAAGATAGCGCCTGGTTTGCCCCT
GGGATGATGGTACTCGTAGACGGCACGTACGAGGAAGATGAAACTGGGACGTCATCTCGTCTTGGTGGAAATGGGGGCGTAGGGGGTACTATTT
CTGGAAAATTTGTCGGCTTCTTCATCGGCCACCCTCCCCCCGAACGCCGTCATGTGACTTTAGGCACAGCCGGTGAGGGAGATACCACAGCCGG
CGGTGGTTTCGGCTGGGTAGACTTCCTAGGCGTGGGTAGTTCTCGCGCCCTAGGCAACAAAATGCAACGACTGGAACAAAAGCTTCTCCGACCC
CCTCCCCCTGACACAGATACAGATGATCCCCCGCCTCCATCCAGAGGCCGTGTAGTCATTCTTGGTGATGTACATCTCGACATTCCTCAAACTC
TTCAAGCCCTCAAAAAGATTCTCTCTCTATACTCATCTGAACCAGAAGGCTGCACACCTATGACCTTTATCCTCCTCGGCTCTTTCGTCTCTCA
TGCCGTACTGGCTCGGGGCGGATCGGGAGGCTCTATAGAATATAAAGAGTATTTTGATTCCCTTGCAGCCGTTCTCTCTGAATACCCTACCATC
TTAAGTACAGCAACATTCATATTTATCCCTGGCCCCAACGATGCATGGGTTTCAGCCTTCTCATCCGGGTCTACTGTTCCTCTGCCCAGAAAAC
CCGTACCGGAAATGTTCACATCCCGCATAAAACGTGCCTTTGCAAACGCAAACACTGAAATGGAGAAAGAACACGGTAATAAAGGAGATGGAGA
GGCCATTTGGACGAGTAATCCTGCAAGAGTCAGTTTATTTGGCATGAGCTGTGAACTGGTAGTATTCCGCGACGACGTTAGCGGACGATTGCGT
CGTACAGCTGTTACACTCAAGTCATCTCAAACGTCAAACCCTGAAAACGAAGATGAAGATATAGATATGTCACCACCTCCATCTTCAATTCCCT
CTTCCACCCCTCCTCCAGAAATCGATCCCGATATCCACACCGCGCGTCGTCTTACCAGGACACTCCTCGATCAGGGACATCTTTCACCCTTTCC
TCTAAACATCGCACCCCAGCATTGGGATTTCTCAAATGCGTTGAGCATTTATCCATTGCCAACAGCAATTGTTATGTGCGATGTAGATAGCCCG
GCATTTTGCTTGACGTACGAGGGGTGTCATGTAATGAATCCAGCGAGTGTAGTAGCAAAGGGGAGAAGAGGCGTAGCGAGATGGATAGAGTATG
ATGTTTGGGGACGGGCTGGGAAGGTCAGAGAGGTGGGATTTTAG

>Bcin11g01310(MLST5), partial sequence [organism=Botrytis cinerea, strain D09_Bc11]
ATGCCTTCCATTAGCAGCATTCTAGTCGCCACTGTTAATTTGTCGGTCCTTGTGAATGGACTGACATGGACCTCATGTGGAACCGGCGTAGAAT
GCGCAACGCTTGAAGTTCCGCTCGAATATGGCGATGCAACGTCAACGGCAAAAGCCAGTGTTGCGCTTGCTCGTTATCCTGCCACTGTTGCCGC
GAGCAAGAAGCTCGGGTCTCTCTTGATAAACCCCGGTGGACCCGGTGCCTCTGGTGTTGGCTTTGTGCAGTCTGGAGCCGGTGCCGCCGTCTCG
ACACTGAGTGGTGGATTGTACGATATCATCGGATGGGATCCACGTGGAACCGGTGCTTCGGCTCCTATTTTGGAATGTTTTGCAAATGCCAGTG
CGGAGTATGATTTTAACAACGCGTTTCCATCTGCTCCGAATCTCTGGCTCGGACAATTTGCGAATGCCAGCGCAAATTCTGCTGTTAGCTCTGC
TATCACATCCTTTGACACTTCTGTCGCTGCTCTTGCAAAAGCTTGCGTGGCTCAGAAATCTCCCGCTCTTTACACCTCAACAGCAGCATATGTT
GCTCGAGACATGGCAGCGATAGTCGATGCATTGGATGGGACCTCTGCAAAACTTAACTACTGGGGTTTCTCATATGGAACTATTTTCCTAGCTG
AGTTTATCCAAACTTTCCCAGGCCGTGTGGGAAGAGTTCTTGCCGATGGTGTTTTCGACGCAAAGGCAAATGCACTCACATACGTTAGCCAACT

TCCCAACGATCAACTCAGTGTTCGTGCTTCGTTGAACGATTTTGCAGCTTTCTGCACCACCGCCGGTAGTAAAGGTTGCTCTTTTGCCACCGCC
CCTACTGGAACCTCAGGTACTGTTGCTACCAGACTGGACAACATAATGAAGGATATGTTCCTCAATCCTATTGTTGCTTCGGGCTTAAGCATCA
GCTTAGATATCCTCAGTCCCCTTCTTGCATCTCTTCTTAGAGTTCCAACCACGTGGAAAACGCTTGCATCTGTCTTATCCGGTCTTGAAACTCG
TGACGCAACTGCTCTTATTTCACTCCTTGGATCGCTAGCAGCGAGTGCACCAACCGACGGCTCTGCAGCAGGCGTTGGCACTCTTGCTACTTAC
CCACTTGGTTGTGTTGATAACGCTGCTTCAAATGGAGTTACCTTGGACACTGTCATTTCTCTCACCAAAAGCATCTCAATCTCCGAAGACACCC
CAATACTAAATGCTGGACTTATCCCCATAACATTTTGTCGCAACTTCCCTTCCACGCGTCCGCTTGTTCCAAACGTAGGAGTAAGTTTGATGTC
GAAGACCGATACTCTTCTCGCAACAGCCAAAACACCAATCCTCATTGTCTCGGCCGAGAATGATCCAACAACCCCTCTCAAGTCTGCAAAAGCA
CTCCGAAGCCTACTCCCTAGTTCATCCACCCTCGTATACCGCGGAGGAAGCGGACACACCACCATCTCACACGCATCTCTCGGAATGGCAAAAG
CGATCTCCAATTTCTTTGTCAGCGGTACCATGCCCAAGGACGGAGCTCGATTTGCAGTGGACCAGAATATTTTCCCAACAGCTGCAGCGAGTGG
TTTAGTTACACCAGCTGCTTTCAACGGAACCTATTCTACACAAGATCAAAGTTTCCTAACCGCAACATACAACATTGGCATTGCCTTCTTAGCA
ATCGCATAA

>Bcin15g03910(MLST6), partial sequence [organism=Botrytis cinerea, strain D09_Bc11]
ATGGCGCCCATTACCAAAATTATCTCGTTTCTCAGCAGCGCATCGCTGATCAACGCTATCGTTATGCCTGAGAGTGTTCTTGAATTTCCCGTCG
CGCATGATGAACATGCCTCTGTCTACGATAAAAACAGTCCTCTTCCAAGTATTTTCAACCTCACCTTGTCCGGAAAGACTCGCCAAAACACAAA
ATCATCCAACGATGAAGATGATACTCCACTTCCCTTGATTATCTGGCATGGACTCGGCGATAATTACAAAGCGGATGGTCTTGCGCAAGTTGGA
AAACTAGCTGAAGCTATTCATCCTGGGACTTTTGTCTACAATATTCATGTAGATGAGGATGCATCTGCAGATAGGACAGCTACCTTCTTTGGAA
ATCTCACTCGTGAGTACATCCCCTATTTTTCCTTTAAATATCATACTAACTCTCTTACCAAGTTCAAATCGAAAAGGTCTGCGAAGACCTCGCC
TCCCATCCTATTCTCTCTACCGCGCCCGCCGTCGACGCAATTGGATTCTCCCAAGGCGGCCAATTCTTGCGTGGTTACATATCCCGCTGCAATG
CTCCACCCATCCGCTCTCTCCTGACCTTCGGTTCCCAACACAACGGCATTTCTGCCTTCCAAGCCTGTGGTCCTGCCGATTTCCTCTGTCGCGG
TGCTCAAACCCTTTTGCGATCCAACACCTGGTCAACCTTTGTCCAATCTCGTCTCGTACCCGCTCAATACTTCAGAGATCCGGAAAACCTAGAC
TCTTACCTTGAATATTCCAATTTCCTTGCCGACATCAATAATGAGCGCGTTCTCAAGAACCAAACATATAAATCCAACATGGAAAAATTGGAAC
GATTCGTAATGTATGTCTTTGAAGACGATACAACTGTCATCCCTAAGGAAAGTGGATGGTGGGCTGAAGTCAACGGCACGGAAGTTACACCACT
GAAAGAAAGAGCCATTTATAAAGAAGATTGGCTAGGTTTAAAGACATTGGATGAGGCCGGAAAATTAGTTTTCGAAACCATTCCAGGGGGACAT
ATGACGTTAGGAGAGGAGATGCTAGAGAAGGCTTTCAAAGAGTATTTTGGTCCAGCAGGGAAGAAATTTGGGGAGAAGCAAGCCCAGATGACGG
GACACGAGGAGGAGTTGTAA

>Bcin16g03460(MLST7), partial sequence [organism=Botrytis cinerea, strain D09_Bc11]
ATGCTTATTTACGCTTTCGCATTGATACCTCTTTACGTCCTAGTATGGGTGCCTCTTTCTCAAGTAATCTACGGTCGTCAATCTGCTCTTCATG
AACCTCTTGATTTGAACTCTTCATTAATTGCAAGTGATGAACCCCTATTCTGCCCAGAGGATAGTTACAAGACGTATATCATTAGTCGAGAACC
ACTCATGATATATATTGACGGATTTTTGAAAGCGAATGAAAGTAAACATTTGGTTGATGTTAGGTGTGTTATTTATTCTGATGAAATGAACAAG
AGAGACTGATGAGATAGTGAGCCGCTTTATGAACCGTCAACTGTTTCTCACGGACAGGAAGTTACCATTGATCCTTCAGTTCGGAATTCTGAAG
TGGCGGTTTTAGAGAGGGATGAGGTGGTCAGGTGTATTGAGCATAGAGCGAGGGCATTTCAGGGGTGGAGGGGCGAGATGGGGATTGAGAAGTT
GAGGACGCAGAGGTATGGGGTTGGAGGACATTATGGGATGCATTTGTAAGTTTTGGGGGATTGACGAAGGCTTCTGTCTATTTTCTATCAGCAC
GGATCTTGAAAGAAAGATAGCATGAACACGCGGGCTAATAACTAGGAAATAGCGATTGGAGCGGAGGTAAACGTGGCATAGACCGATTTAGTAC
TTTCATGGTCTATGTCGACGTATCCTCTGATATCGAAGGTGGAGGAACGGAATTCCCACGTATCGTGGGACCAAAAGGAGGAAGGTGGGAGGAC
TTCCTGGAAACTACGGAAGCATTGGATCCAAGAACTGGAGAAAATGTAACAGTAGAAGGGGTGACATTCAAACCAATCAAGGGAAATGCCGTAT
TCTGGGAAAATACTGACAACAACGGGAGGGGCTATGATGAAACATGGCACGCTGGTCTACCGGTGGAAAAAGGCTCGAAAGTAGGGTTGAATAT
TTGGAGTTATGGGAGGACCATTAGATGA

>Bcin12g03020(MLST8), partial sequence [organism=Botrytis cinerea, strain D09_Bc11]
ATGGCCGACGATGGAATGCTGATGAATTTTGAGATTGGGGATGTACCTATTGTTGCGAAACAAGCATTTAAAGGAGGACGTTGGAAGGATCGAT
TGGCTGCGAAGAAAACTGCGCAGCACCGAGTCACCAAAAGCACGAATAAACCATCGGCAAGGGAAATATTCTCTGAACCACAACATGACACGAG
CGCGGAAGAGTATATCGGGCGAGAAGCTTCATCAAGAGCACCAAAGCGACAACGAGTCGATGATAATTATAACTCTTACGGTGGAAGAAATGAA
AATACAGCAGCTTATGCTTCCGGGAAACTTCCATCTGGAAGTATAAATGTTGGTGGAGGTAGGAAGACCACTTTTCAAGAAGAACCACGAACGG
CATTTGTCGCTGGCAAGTTGCCCCCTGGAAGTATCAACATTGGTGGAAAGAAGGCTACCCAAATCGAGGACGAGGGTAGAGCAGCTTATGCTTC
CGGAAAATTGCCTCCAGGAAGTATAAACTCTGGCGCAAAGAAGGCGATTTCATTCCAAGATGAAACGAGACCGGCTTATGTCTCCGGAAAGCTT
CCACATGGTAGTATCGACGGTATGCGAAACCGTGAAATGGCTGCCGTCCACCGCGAAATTGCTGAGGGTGGGAGGAAACCAGGCCAGGTTGTCT
CTTCTCTATTCACATTCAATCCTACTTCAAAGAAAACTTTTGATGAACCCGAAGAACAAGCGGAACCGGCAAAACCATCCAATGCGCCTTTGAC
AGAGGAAATGGCGACTTTCACCAATCTAGGGTTATCGAGAAGGCTTGCAGCCCATCTATCGACTAAACTCGATATGAAAGCTCCGACCGCCATT
CAAAAAGCATCTGTACAGCAGTTGGTATCGGACGATAGCGATGCTTTCATACAAGCAGAGACTGGATCTGGAAAAACTTTGGCATATCTACTAC
CTATAGTCGAGCGAATATTAGCATTGAGTGAGAATGGCGTACAAATACATCGAGATTCTGGACTTTTTGCGATTATTCTTTCACCTACAAGAGA
ACTGTGCAAACAAATCGCGGCTGTGCTGGAGAAAGTTTTACGTTGTGCGCCATGGATTGTTGGTACGACCGTTAACGGAGGAGAGAGTAAGCAA
TCAGAAAAGGCTAGACTTCGAAAGGGTGTCAATATTCTTGTTGCAACACCGGGTCGATTAGCAGATCATCTCGATAACACAGAAGTGTTGAATG
TAGCTACTGTAAGATGGCTAGTGTTGGATGAAGGAGATAGATTAATGGAGCTTGGGTTCGAGGAAGAGATTAAAGGCATCGTTGAGAAGATAGG
TCGAAGATCAGTGGCCAAAGCAAATTCGGACATGGGGTCACTCCCGAAACGAAGAGTTACTATCTTATGTTCTGCTACGATGAAGATGAATGTT
CAGCGTTTGGGAGAAATCAGTTTAAAGGATGCTGTGCACATTCAAGCGGATCCTTCCGAACAAGAGAAACAAGACAAGGAAAATGGCGTTGAAG

CCCAAGATAAAGCTTTCTCCGCCCCTACGCAATTGAAGCAGTCATATGCTATCGTTCCTGCGAAGCTCAGACTTGTTACCCTGACGGCTCTATT
GAAACGTGCTTTTGCACGAAAAGGGTCCGTGATGAAGGCGATCGTTTTCATTTCTTGTGCTGACTCTGTTGACTTTCACTTCTCGCTCTTCTCT
CGTACCCCAGAAGCTTCAGCCGAGGTTGTGGACGAGGAGAAGGTTGATCTTCCCGCATTACCAAAATCAGAATTGGTCAAAGAGACTATTGCGC
ATGGAACTACAATTTCGAATAATTCAAACCCTGTAATTCTACACAAACTTCACGGATCTCTTGCACAAAACATCAGAACAGCAACCCTCAAGGC
ATTTTCCGAATCTGCGGACCCTTGTGTTATGATATGTACTGATGTTGCATCTCGAGGTCTCGATCTTCCAAATGTCGACTTTGTCATCGAGTAT
GACCCGCCTTTTAGCGCGGAAGATCATCTACATCGTGTTGGTCGAACAGCCAGAGCTGGTCGCGAGGGTCGTGCTTTGATCTTCCTTATGCCTG
GCGTCGAGGAGGAATATGTTTCTATCTTAGCATCCGGATACCGTGAAGGAAAGAAAGCTCTGACACGTCACACTGCTGAGGATTTGATACAAAA
GGGTTTCGGTGGTATAGGTCGCGAATGGGAAGAGAGAGCTACGAATTTCCAACTTGAAGTTGAACGATGGAGTTTGGACTCGCCAAAATATCTT
GAGATGGCACGCCGTGGATACCAGAGTCATATCAGGGCTTATGCCACTCACGTCGCAAATGAGAGACACATATTCAATATGCAAGAATTGCACT
TGGGTCATTTGGCCAAAGCATTTGCCTTGCGAGACAAACCTGGAAGCATTAAGGTGCCAGGATTAAGACCAGCGAAGATGACCAAAGCAGATCG
AAGTGTCGCGGCTAGGAAGGCGAAGAGGGGTGAGAAAGCAGAAGATAAAGCTCCCGAGGGAGAAAGAGTGCGGAAACAAAAGAAGATGGAGCTG
GACTTGCCTACTGTGGATGGCAATGAAGCTGCCGCGAGGATGAAGAGGAAGATGAAGGAGCATATGGCTGCTGCGAGCGAGTTCAATATTGGTT
GA

>Bcin02g07770(MLST9), partial sequence [organism=Botrytis cinerea, strain D09_Bc11]
ATGGTTGCCTTCTCAAAATCATTACAGCTTTCCCTTTCGGTCTTGGCATCTACAGTCATTGCCATCCCTACACCATCACAACTTGAGTCTCGGG
CCGTTATCGATTCCGATGCCGTTGTAGGATTTGCCGAAACTGTTCCCAGTGGGACCGTAGGAACAGTTTATGAGGCATATAAACCATTCCTTAA
AGTCGTAAATGGATGCGTACCATTCCCTGCCGTCGATGCATCGGGTAACACAGGGTATGTCCTTATATCTTTCTCTTCCACACGATTGCTATTG
AGTCTCTAACATATTTTAGTGGTGGTTTGTCACCAACTGGCAGTAGCAATGGTGGTTGCAGCAGCAGTACCGGTCAAGTATATGTTCGAGGAGG
ACAAAGCGGATCAAACTACGCCATCATGTACTCCTGGTAAGTTCTCTCTAAACTTCTCCTTATAGATCCAACCTAACAAAATCTTAGGTACATG
CCAAAGGACGAGCCCTCAACCGGTATTGGTCACCGTCACGATTGGGAAGGTGTAATTGTCTGGCTCTCCAGCGCCACCGCCACAACTGCCGACA
ACATCTTAGCCGTTTGTCCTTCCGCCCACGGAGGCTGGGATTGTTCCACGGATGGCTATTCCCTTTCTGGTACCAGCCCTCTTATCAAGTACGA
AAGTATCTGGCCCGTCGATCACTCAATGGGTCTTACTAGTACTGTTGGTGGAAAACAACCTATGATTGCTTGGGAGTCTTTACCAACTGCTGCT
CAAACTGCTCTTGAGAACACCGATTTCGGTGCTGCGAATGTTCCATTCATTCCGGCTGTTTTCACAGACAATCTTGCGAAGGCTACTTTCTAG

>Bcin04g02090(MLST10), partial sequence [organism=Botrytis cinerea, strain D09_Bc11]
AACTGTCGAATATGACTATGAATGATGTTTACAAGCCCTACATCCATGTAAGAAATGTAGAATAGAGAGATCAGTAACTGGAACTAATATCGTT
TGTAGGCTTTCAAGTTACTTACGCAGTTCAACCCAATCACTACAGCTATTGCCGAATCCCCACTGTTTCAAATGGCCGTCTCAGCAAATACCAT
CGAAAAGTACACACTGCTAGGCCCTTTCTTCAGAATATCTCCTCTGCAACAGGAAGTTACCAGGGAATACTTCAGTGCGCCAAAGACGATAGAT
AGGCGACACATTGCCACATCTCAAGATGCGTTACGATTGACCTTACAAACCCATCAAAAAGATTTACTTGATATCATCAACCACTTTGTTCGAG
CAAGTCCAATCGCAAAAAGCAAAACCCTGGATTGGTTCGCCTACATTGTGAATCAAAATCACAAGCGTCGAGCACTTCAGGTAGACCCGAAAGA
AGTGTCTTCTGATGGCTTTATGCACAATGTCACTGTCGTTCTAGATGGTCTTTGTGAGCCATTCATGGATACCACATTCTCGAAAATTTCGAAG
ATTGATATTGATTATCTAAGACGTGCGCCTCGTGTAGATATCAAGGACGAGACCAAGTTGAACGCTGACGAGAAGGCTTCTGAGAAGTATTATG
AGGACACTGTTCCTGGCACTTCTAATTTCATCTCTGAGGTCTTCTTTCTCACATTGG

>Bcin01g07220(MLST1), partial sequence [organism=Botrytis cinerea, strain D13_E_IL4]
CAAGCTTGGGTTAGTGCTCTGCGTGTAGAAGCACTAAAGAAGATTCTCGCACAACCGAAGTCATGGTTTGAGGAATCCAGGAATTCACCTGGCC
GGTTGAACGAAGTTTTGGATAGGAACTCTGAGGAAATGCGTAATCTCGTTGGCCGCTTTGCTGGTATTGTATTCACAGCATTTTTTATGCTATT
GATATCAATCATTTGGGCTTTCGTGAATACATGGAAACTGACATTAGTCTCGATGGCAACTGGGCCAGTTATATACGCTGTCACCAAAACGTTC
AATCGCGTGAGTGGAAAATGGGAAAACAAGTGCAACTACGCCTCTGAAATGACCACTGGCATATTTTCAGAGACTTTCTCCAACATCAAAGTGG
TTCGGGCTTTTACTCTGGAAACTTACTTTGAGACAAAACACACCAAAGCTACAGAAGAACTTTATAAAGTTGGACTAATACGAGCAAACTACTC
GGGATTGCTGTGGGGATTGACAGATGCGATGTCATTCTTCATCACTGCAACTATCTTTTATTATGCCACGGTTCTCATTACCAAGAGAGAGATC
AGTATCGCGACTGCACTACAGACTGTCAATCTTCTATTATTTGGTATTTCTAATAGTACGAATATGCTGGCCATGATACCACAAATCAACTCTT
CTCGCGTTACAGCTACGCATATGCTTGCATTAGCCAATCTCGATTCATCTTCCTCCCACGAAAATAAAGGAACCGAACGGCTTTCGACAATCTT
TCCAATCAAATTCAACCGTCTCTCATTCACATACCCTACTCGTCCTGAAAAACGAACGATATCATCCTTTTCTCTTTCCCTGATTCCTAACTCA
ACAACTGCACTTGTCGGACCCTCCGGCTCCGGAAAATCTACAATAGCTGCTCTGCTCATTGGTCTCTATCCGCCAGATACTTCAACACC

>Bcin05g07690(MLST2), partial sequence [organism=Botrytis cinerea, strain D13_E_IL4]
TGGGTAGTTCTTGGCTGGCTGGATACTGTCAAATTCATTGATTTACATTCTGAATTGCACTATTCAAACGACTCTCAGCCAGAATGGTACGGAC
AACAATATAAACCTGCATTTGCACATCGAGCGCGACTATTTTGGGAATTGGCTTCACAAGGATGGGATACTACTCTCTGTGGTGGTGGGATGAT
ATGGTCACCATACCTTACTCCATACAAGAACGCAATTACCAATGAACTCTATATCGCAGCTTCGATATCGATGTACCTATATTTCCCCGGAGAT
GACAATCAATCCCCATTTATGATTTCCAACCCTTCATATCCACCTCACGATCCGAAATATCTACAGGCAGCTGTTGATGCTTACAAATGGCTGA
ATGGTTCCAACATGACGGATTCACAAGGATTATATGTCGACGGGTACCATATCTCGAATCTTTCTGGCGGTGAAAACACCCATTGCGATTCTAG
AAATGAGATGGTATATACCTACAATCAAGGTGTTTTGCTTACTGGACAACGTGGTTTGTATGACGCAACCGCCGCACGATCATACCTTGTAGAT
GGCCACAAACTCATCGCGAATGTTATTAATGCCACAGGCTATGACCTGAAACACAATGTTGTCATCTCACCGCCACCCAAAGATGGTTCCGCAT
TGGCAAAGTGGTTTGGCCTGGGTAGGAATGGAATACTGGAAGAAGGATGCGATTCAAGTGCTTCGTGTTCTCAAAATGGACAAACTTTCAAAGG
CATATTCTTTCATCACTTGATTGCGTTCTGTAGTGATTTGCCAGGGGAGCCTATTGCAGGGACGAAGGAA

>Bcin06g01710(MLST3), partial sequence [organism=Botrytis cinerea, strain D13_E_IL4]
CAAGGCAATCACCTAATTTCATGGCGTTCTTCCCTGCATCATCTACCTACCCTGGAATGCTGGGAGAATTATACTCAGCAGCTCTCACAGCACC
TGCTTTCAATTGGATCTGTTCCCCTGCTGTGACAGAATTGGAGACGGTTGTAATGGATTGGCTGGCCAAGCTTCTCAATCTCCCAGACTGTTAT
TTGTCTTCGACTCATGGTGGTGGTGTTATCCAAGGATCAGCCTCGGAAGCTGTCGTTACCGTTATGGTTGCTGCCCGCGATAAATATCTTCGTG
AAACTACTGAAGGTCTGTCGGGTATTGAACTCGAGGATGCGATTGCATATAAAGGAGTAAGCTAGTTGCACTAGGAAGCGAAATGGCACACAG
TTCCACGCAGAAAGCAGCGCAGATAGCTGGCGTTAGATTCCGATCGATTCCAGTACTCGCATCCAATGATTTCGCCATGACGGGTGATGATTTA
GAGAAGGTATTGAAAGAATGCAAATCTCAGGGATTGGAACCCTTCTATCTAACTTCGACTTTGGGAACAACATCTACATGCGCAGTTGACGACT
TCGCATCTATTACAACAGTACTTTCAAAATATGCACCTCCAGATGTTGCAGGCGAGATCTGGGTTCACGTCGATGCTGCTTATGCAGGTGCAGC
TTTGGTTTGCCCTGAATACCATCATCTAACATCGTCCTTCCAGCATTTCCATTCCTTCGATATGAACATGCACAAATGGCTTCTGACAAATTTC
GACGCTTCTTGTCTATATGTCAAGAAACGCAAAGATCTGATCGATGCACTATCCATAACACCAAGTTATCTTCGCAACGAGTTTTCAGAGAGTG
GACTCGTAACCGACTATCGTGACTGGCAAATTCCTCTCGGAAGACGCTTCCGAAGCT

>Bcin09g03030(MLST4), partial sequence [organism=Botrytis cinerea, strain D13_E_IL4]
GCAGAGAGAGTCTTGGAGGAGGTCGCCAAGAGTTGGAAGAATAGGAGTGGCGGTGTCATTGTCGAGGGCGAGGGAACGGAATTGAAGGAGATTC
TGAAAGCTTTGGAAGGGAATATGAGTGGTGGAAGGATAGTCATAGGAAGAGAGCTAAGCCGGCAAAATAGTTTAGTACTGGGATCATCACAATA
TGGAGAGATCAATCATACAAGACTTGGGCTACGGCCAGGGAATATACCCAGAGAGGATAGTCAGTCAAGTTTGGGAATGTCAACGTTGGAGGTC
AATGACGAGGAAGATGAGGATGGCCTGATGGATCCAAGAAGGTGGTTAAAAGTCATTGATGCATTTGAGCAACCTCGACTAGTGTACAATGTTG
CTAAAAAGCACTTTGATAGGTATGTTTCAATGATAAAATTTTATCTGAATCGACTAACTCAGTACAGAGATACCTCCAAACCTTCATTGTTCCC
ACCTGCGTCTCATAAAACACTCCTCTTCCAAAACCGCTATAATGTTATCCATCAACGTCTCCTTCGCAATGAATCTTTTCAAACGCCCGCTTTT
CAAGGTGGCAAATCTTCCCTTCAACGCAGCACGTCCGCCATTACCACCCAACAACAATCATACAAATTAACGCCGATAGCTAATCTTCTCGGTC
GCAATCGCAGCTCTCATATGCTTCTCGGTCTCCTCAGTATTTCACCCACTGGTACCCTCGCCATCAATGACCTG

>Bcin11g01310(MLST5), partial sequence [organism=Botrytis cinerea, strain D13_E_IL4]
TCATGTGGAACCGGCGTAGAATGCGCAACGCTTGAAGTTCCGCTCGAATATGGCGATGCAACGTCAACGGCAAAAGCCAGTGTTGCGCTTGCTC
GTTATCCTGCCACTGTTGCCGCGAGCAAGAAGCTCGGGTCTCTCTTGATAAACCCCGGTGGACCCGGTGCCTCTGGTGTTGGCTTTGTGCAGTC
TGGAGCCGGTGCCGCCGTCTCGACACTGAGTGGTGGATTGTACGATATCATCGGATGGGATCCACGTGGAACCGGTGCTTCGGCTCCTATTTTG
GAATGTTTTGCAAATGCCAGTGCGGAGTATGATTTTAACAACGCGTTTCCATCTGCTCCGAATCTCTGGCTCGGACAATTTGCGAATGCCAGCG
CAAATTCTGCTGTTAGCTCTGCTATCACATCCTTTGACACTTCTGTCGCTGCTCTTGCAAAAGCTTGCGTGGCTCAGAAATCTCCCGCTCTTTA
CACCTCAACAGCAGCATATGTTGCTCGAGACATGGCAGCGATAGTCGATGCATTGGATGGGACCTCTGCAAAACTTAACTACTGGGGTTTCTCA
TATGGAACTATTTTCCTAGCTGAGTTTATCCAAACTTTCCCAGGCCGCGTGGGAAGAGTTCTTGCCGATGGTGTTTTCGACGCAAAGGCAAATG
CACTCACATACGTTAGCCAACTTCCCAACGATCAACTCAGTGTTCGTGCTTCGTTGAACGATTTTGCAGCTTTCTGCACCACCGCCGGTAGTAA
AGGTTGCTCTTTTGCCACCGCCCCTACTGGAACCTCAGGTACTGTTGCTACCAGACTGGACAACATAATGAAAGATATG

>Bcin15g03910(MLST6), partial sequence [organism=Botrytis cinerea, strain D13_E_IL4]
ATGATACTCCACTTCCCTTGATTATCTGGCATGGACTCGGCGATAATTACAAAGCGGATGGTCTTGCGCAAGTTGGAAAACTAGCTGAAGCTAT
TCATCCTGGGACTTTTGTCTACAATATTCATGTAGATGAGGATGCATCTGCAGATAGGACAGCTACCTTCTTTGGAAATCTCACTCGTGAGTAC
ATTCCCTATTTTTCCTTTAAATATCATACTAACTCTCTTACCAAGTTCAAATCGAAAAGGTCTGCGAAGACCTCGCCTCCCATCCTATTCTCTC
TACCGCGCCCGCCGTCGACGCAATTGGATTCTCCCAAGGCGGCCAATTCTTGCGTGGTTACATATCCCGCTGCAATGCTCCACCCATCCGCTCT
CTCCTGACCTTCGGTTCCCAACACAACGGCATTTCTGCCTTCCAAGCCTGTGGTCCTGCCGATTTCCTCTGTCGCGGTGCTCAAACCCTTTTGC
GATCCAACACCTGGTCAACCTTTGTCCAATCTCGTCTCGTACCCGCTCAATACTTCAGAGATCCGGAAAACCTAGACTCTTACCTTGAATATTC
CAATTTCCTTGCCGACATCAATAATGAGCGCGTTCTCAAGAACCAAACATATAAATCCAACATGGAAAAATTGGAACGATTCGTAATGTATGTC
TTTGAAGACGACACAACTGTCATCCCTAAGGAAAGTGGATGGTGGGCTGAAGTCAACGGCACGGAAGTTACACCACTGAAAGAAAGAGCCATTT
ATAAAGAAGATTGGCTAGGTTTAAAGACATTGGATGAGGCCGGAAAATTAGTTTTCGAAACCATTCCAGGGGACATATGACGTTAGGAGAGGAG
ATGCTAGAGAAGGCTTTCAAGA

>Bcin16g03460(MLST7), partial sequence [organism=Botrytis cinerea, strain D13_E_IL4]
TCTTCATGAACCTCTTAATTTGAACTCTTCATTATTTGCAAGTGATGAACCCCTATTTCTGCCCAGAGGATAGTTACAAGACGTATATCATTAG
TCGAGAACCACTCATGATATATATTGACGGATTTTTGAAAGCGAATGAAAGTAAACACTTGGTTGATGTTAGGTGTGTTATTTATTCTGATGAA
ATGAACAAGAGAGACTGATGAGATAGTGAACCGCTTTATGAACCGTCTACTGTTTCTCACGGGCAGGAAGTTACCATTGATACTTCAGTTCGAA
ATTCTGAAGTGGCGGTTTTAGAGAGGGATGAGGTGGTCAGGTGTATTGAGCATAGAGCGAGGGCATTTCAGGGGTGGAGGGGCGAGATGGGGAT
TGAGAAGTTGAGGACGCAGAGGTATGGGGTTGGAGGACATTATGGGATGCATTTGTAAGTTTTGGGGGATTGACGAAGGCTTTTGTCTATTTTC
TACCAGTACGGATCTTGAAAGAAAGATAGCATGAACACGAGGGCTAATAACTAGGAAATAGCGATTGGAGTGGAGGTAAACGTGGCATAGACCG
ATTTAGTACTTTCATGGTCTATGTCGACGTATCCTCTGATATCGAAGGTGGAGGAACGGAATTCCCACGTATCGTGGGACCAAAAGGAGGAAGG
TGGGAGGACTTCCTGGAAACTACGGAAGCATTGGATCCAAGAACTGGAGAAAATGTAACAGTAGAAGGGGTGACATTCAAACCAATCAAGGGAA
ATGCCGTATTCT

>Bcin12g03020(MLST8), partial sequence [organism=Botrytis cinerea, strain D13_E_IL4]
ACTGCGCAGCACCGAGTCACCAAAAGCACGAATAAACCATCGGCAAGGGAAATATTCTCTGAACCACAACATGACACGAGCGCGGAAGAGTATA
TCGGGCGAGAAGCTTCATCAAGAGCACCAAAGCGACAACGAGTCGATGATAATTATAACTCTTACGGTGGAAGAAATGAAAATACAGCAGCTTA
TGCTTCCGGGAAACTTCCATCTGGAAGTATAAATGTTGGTGGAGGTAGGAAGACCACTTTTCAAGAAGAACCACGAACGGCATTTGTCGCTGGC
AAGTTGCCCCCTGGAAGTATCAACATTGGTGGAAAGAAGGCTACCCAAATCGAGGACGAGGGTAGAGCAGCTTATGCTTCCGGAAAATTGCCTC
CAGGAAGTATAAACTCTGGCGCAAAGAAGGCGATTTCATTCCAAGATGAAACGAGACCGGCTTATGTCTCCGGAAAGCTTCCACATGGTAGTAT
CGACGGTATGCGAAACCGTGAAATGGCTGCCGTCCACCGCGAAATTGCTGAGGGTGGGAGGAAACCAGGCCAGGTTGTCTCTTCTCTATTCACA
TTCAATCCTACTTCAAAGAAAACTTTTGATGAACCCGAAGAACAAGCGGAACCGGCAAAACCATCCAATGCGCCTTTGACAGAGGAAATGGCGA
CTTTCACCAATCTAGGGTTATCGAGAAGGCTTGCAGCCCATCTATCGACTAAACTCGATATGAAAGCTCCGACCGCCATTCAAAAAGCATCTGT
ACAGCAGTTGGTATCGGACGATAGCGATGCTTTCATACAAGCAGAGACTGGATCTGGAAAAACTTTGGCATATCTACTACCTATAGTCGAGCGA
ATATTAGCATTGAGTGAGAATGGCGTACA

>Bcin02g07770(MLST9), partial sequence [organism=Botrytis cinerea, strain D13_E_IL4]
CGATTCCGATGCCGTTGTAGGATTTGCCGAAACTGTTCCCAGTGGGACCGTAGGAACAGTTTATGAGGCATATAAACCATTCCTTAAAGTCGTA
AATGGATGCGTACCATTCCCTGCCGTCGATGCATCGGGTAACACAGGGTATGTCCTTATACCATTCTCTTCCACACGATTGCTATTGAGTCTCT
AACATATTTTAGTGGTGGTTTGTCACCAACTGGCAGTAGCAATGGTGGTTGCAGCAGCAGTACCGGTCAAGTATATGTTCGAGGAGGACAAAGC
GGATCAAACTACGCCATCATGTACTCCTGGTAAGTTCTCTCTAAACTTCTCCTTATAGATCCAACCTAACAAAATCTTAGGTACATGCCAAAGG
ACGAGCCCTCAACCGGTATTGGTCACCGTCACGATTGGGAAGGTGTAATTGTCTGGCTCTCAAGCGCCACCGCCACAACTGCCGACAACATCTT
AGCCGTTTGTCCTTCCGCCCACGGAGGCTGGGATTGTTCCACCGATGGATATTCCCTTTCTGGTACCAGCCCTCTTATCAAGTACGAAAGTATC
TGGCCCGTCGATCATTCAATGGGTCTTACTAGTACTGTTGGTGGAAAACAACCTATGATTGCTTGGGAGTCTTTACCAACTGCTGCTCAAACTG
CTCTTGAGAACACCGATTTCGGTGCTGCGAATGTTCCATTCATTCCGGCTGTTTTCACAGATAATCTTGCGAAGGCTACTTTCTAG

>Bcin04g02090(MLST10), partial sequence [organism=Botrytis cinerea, strain D13_E_IL4]
AACTGTCGAATATGACTATGAATGATGTTTACAAGCCCTACATCCATGTAAGAAATGTAGAATAGAGAGATCAGTAACTGGAACTAATATCGTT
TGTAGGCTTTCAAGTTACTTACGCAGTTCAACCCAATCACTACAGCTATTGCCGAATCCCCACTATTTCAAATGGCTGTCTCAGCAAATACCAT
CGAAAAGTACACACTGCTAGGCCCTTTCTTCAGAATATCTCCTCTGCAACAGGAAGTTACCAGGGAATACTTCAGTGCGCCAAAGACGATAGAT
AGGCGACACATTGCCACATCTCAAGATGCCTTACGATTGACCTTACAAACCCATCAAAAAGATTTACTTGATATCATCAACCACTTTGTTCGAG
CAAGTCCAATCGCAAAAAGCAAAACCCTGGATTGGTTCGCCTACATTGTGAATCAAAATCACAAGCGTCGAGCACTTCAGGTAGACCCGAAAGA
AGTGTCTTCTGATGGCTTTATGCACAATGTCACTGTCGTTCTAGATGGTCTTTGTGAGCCATTCATGGATACCACATTCTCGAAAATTTCGAAG
ATTGATATTGATTATCTAAGACGTGCGCCTCGTGTAGATATCAAGGACGAGACCAAGTTGAACGCTGACGAGAAGGCTTCTGAGAAGTATTATG
AGGACACTGTTCCTGGCACTTCTAATTTCATCTCTGAGGTCTTCTTTCTCACATTGG

>Bcin01g07220(MLST1), partial sequence [organism=Botrytis cinerea, strain D13_E_IL12]
GGTGTTGAAGTATCTGGCGGATAGAGACCAATGAGCAGAGCAGCTATTGTAGATTTTCCGGAGCCGGAGGGTCCGACAAGTGCAGTTGTTGAGT
TAGGAATCAGGGAAAGAGAAAAGGATGATATCGTTCGTTTTTCAGGACGAGTAGGGTATGTGAATGAGAGACGGTTGAATTTGATTGGAAAGAT
TGTCGAAAGCCGTTCGGTTCCTTTATTTTCGTGGGAGGAAGATGAATCGAGATTGGCTAATGCAAGCATATGCGTAGCTGTAACGCGAGAAGAG
TTGATTTGTGGTATCATGGCCAGCATATTCGTACTATTAGAAATACCAAATAATAGAAGATTGACAGTCTGTAGTGCAGTCGCGATACTGATCT
CTCTCTTGGTAATGAGAACCGTGGCATAATAAAAGATAGTTGCAGTGATGAAGAATGACATCGCATCTGTCAATCCCCACAGCAATCCCGAGTA
GTTTGCTCGTATTAGTCCAACTTTATAAAGTTCTTCTGTAGCTTTGGTGTGTTTTGTCTCAAAGTAAGTTTCCAGAGTAAAAGCCCGAACCACT
TTGATGTTGGAGAAAGTCTCTGAAAATATGCCAGTGGTCATTTCAGAGGCGTAGTTGCACTTGTTTTCCCATTTTCCACTCACGCGATTGAACG
TTTTGGTGACAGCGTATATAACTGGCCCAGTTGCCATCGAGACTAATGTCAGTTTCCATGTATTCACGAAAGCCCAAATGATTGATATCAATAG
CATAAAAAAATGCTGTGAATACAATACCAGCAAAGCGGCCAACGAGATTACGCATTTCCTCAGAGTTCCTATCCAAAACTTCGT

>Bcin05g07690(MLST2), partial sequence [organism=Botrytis cinerea, strain D13_E_IL12]
AGGATGCCTTCTCTCTACGACAGCAAGCCTTCGATGATATGTTGTGGGTAGTTCTTGGCTGGCTGGATACTGTCAAATTCATTGATTTACATTC
TGAATTGCACTATTCAAACGACTCTCAGCCAGAATGGTACGGACAACAATATAAACCTGCATTTGCACATCGAGCGCGACTATTTTGGGAATTG
GCTTCACAAGGATGGGATACTACTCTCTGTGGTGGTGGGATGATATGGTCACCATACCTTACTCCATACAAGAACGCAATTACCAATGAACTCT
ATATCGCAGCTTCGATATCGATGTACCTATATTTCCCCGGAGATGACAATCAATCCCCATTTATGATTTCCAACCCTTCATATCCACCTCACGA
TCCGAAATATCTACAGGCAGCTGTTGATGCTTACAAATGGCTGAATGGTTCCAACATGACGGATTCACAAGGATTATATGTCGACGGGTACCAT
ATCTCGAATCTTTCTGGCGGTGAAAACACCCATTGCGATTCTAGAAATGAGATGGTATATACCTACAATCAAGGTGTTTTGCTTACTGGACAAC
GTGGTTTGTATGACGCAACCGCCGCACGATCATACCTTGTAGATGGCCACAAACTCATCGCGAATGTTATTAATGCCACAGGCTATGACCTGAA
ACACAATGTTGTCATCTCACCGCCACCCAAAGATGGTTCCGCATTGGCAAAGTGGTTTGGCCTGGGTAGGAATGGAATACTGGAAGAAGGATGC
GATTCAAGTGCTTCGTGTTCTCAAAATGGACAAACTTTCAAAGGCATATTCTTTCATCACTTGATTGCGTTCTGTAGTGATTTGCCAGGGGAGC
CTATTGCAGGGACGAAGGAA

>Bcin06g01710(MLST3), partial sequence [organism=Botrytis cinerea, strain D13_E_IL12]
AGCTTCGGAAGCGTCTTCCGAGAGGAATTTGCCAGTCACGATAGTCGGTTACGAGTCCACTCTCTGAAAACTCGTTGCGAAGATAACTTGGTGT

TATGGATAGTGCATCGATCAGATCTTTGCGTTTCTTGACATATAGACAAGAAGCGTCGAAATTTGTCAGAAGCCATTTGTGCATGTTCATATCG
AAGGAATGGAAATGCTGGAAGGACGATGTTAGATGATGGTATTCAGGGCAAACCAAAGCTGCACCTGCATAAGCAGCATCGACGTGAACCCAGA
TCTCGCCTGCAACATCTGGAGGTGCATATTTTGAAAGTACTGTTGTAATAGATGCGAAGTCGTCAACTGCGCATGTAGATGTTGTTCCCAAAGT
CGAAGTTAGATAGAAGGGTTCCAATCCCTGAGATTTGCATTCTTTCAATACCTTCTCTAAATCATCACCCGTCATGGCGAAATCATTGGATGCG
AGTACTGGAATCGATCGGAATCTAACGCCAGCTATCTGCGCTGCTTTCTGCGTGGAACTGTGTGCCATTTCGCTTCCTAGTGCAACTAGCTTAC
TCCTTTTATATGCAATCGCATCCTCGAGTTCAATACCCGACAGACCTTCAGTAGTTTCACGAAGATATTTATCGCGGGCAGCAACCATAACGGT
AACGACAGCTTCCGAGGCTGATCCTTGGATAACACCACCACCATGAGTCGAAGACAAATAACAGTCTGGGAGATTGAGAAGCTTGGCCAGCCAA
TCCATTACAACCGTCTCCAATTCTGTCACAGCAGGGGAACAGATCCAATTGAAAGCAGGTGCTGTGAGAGCTGCTGAGTATAATTCTCCCAGCA
TTCCAGGGTAGGTAGATGATGCAGGGAAGAACGCCATGAAATTAGGTGATTGCCTTG

>Bcin09g03030(MLST4), partial sequence [organism=Botrytis cinerea, strain D13_E_IL12]
GCAGAGAGAGTCTTGGAGGAGGTCGCCAAGAGTTGGAAGAATAGGAGTGGCGGTGTCATTGTCGAGGGCGAGGGAACGGAATTGAAGGAGATTC
TGAAAGCTTTGGAAGGGAATATGAGTGGTGGAAGGATAGTCATAGGAAGAGAGCTAAGCCGGCAGAATAGTTTAGTACTGGGATCATCACAATA
TGGAGAGGTCAATCATACAAGACTTGGGCTACGGCCAGGGAATATACCCAGAGAGGATAGTCAGTCAAGTTTGGGAATGTCAACGTTGGAGGTC
AATGACGAGGAAGATGAGGATGGCCTGATGGATCCAAGAAGGTGGTTAAAAGTCATTGATGCATTTGAGCAACCTCGACTGGTGTACAATGTTG
CTAAAAAGCACTTTGATAGGTATGTTTCAATGATAAGATTTTATCTGAATCGACTAACTCAGTACAGAGATACCTCCAAACCTTCATTGTTCCC
ACCTGCGTCTCATAAAACACTCCTCTTCCAAAACCGCTATAATGTTATCCATCAACGTCTCCTTCGCAATGAATCTTTTCAAACGCCCCGCTTTT
CAAGGTGGCAAATCTTCCCTTCAACGCAGCACGTCCGCCATTACCACCCAACAACAATCATACAAATTAACGCCGATAGCTAATCTTCTCGGTC
GCAATCGCAGCTCTCATATGCTTCTCGGTCTCCTCAGTATTTCACCCACTGGTACCCTCGGCATCAATGACCTG

>Bcin11g01310(MLST5), partial sequence [organism=Botrytis cinerea, strain D13_E_IL12]
TCATGTGGAACCGGCGTAGAATGCGCAACGCTTGAAGTTCCGCTCGAATATGGCGATGCAACGTCAACGGCAAAAGCCAGTGTTGCGCTTGCTC
GTTATCCTGCCACTGTTGCCGCGAGCAAGAAGCTCGGGTCTCTCTTGATAAACCCCGGTGGACCCGGTGCCTCTGGTGTTGGCTTTGTGCAGTC
TGGAGCCGGTGCCGCCGTCTCGACACTGAGTGGTGGATTGTACGATATCATCGGATGGGATCCACGTGGAACCGGTGCTTCGGCTCCTATTTTG
GAATGTTTTGCAAATGCCAGTGCGGAGTATGATTTTAACAACGCGTTTCCATCTGCTCCGAATCTCTGGCTCGGACAATTTGCGAATGCCAGCG
CAAATTCTGCTGTTAGCTCTGCTATCACATCCTTTGACACTTCTGTCGCTGCTCTTGCAAAAGCTTGCGTGGCTCAGAAATCTCCCGCTCTTTA
CACCTCAACAGCAGCATATGTTGCTCGAGACATGGCAGCGATAGTCGATGCATTGGATGGGACCTCTGCAAAACTTAACTACTGGGGTTTCTCA
TATGGAACTATTTTCCTAGCTGAGTTTATCCAAACTTTCCCAGGCCGCGTGGGAAGAGTTCTTGCCGATGGTGTTTTCGACGCAAAGGCAAATG
CACTCACATACGTTAGCCAACTTCCCAACGATCAACTCAGTGTTCGTGCTTCGTTGAACGATTTTGCAGCTTTCTGCACCACCGCCGGTAGTAA
AGGTTGCTCTTTTGCCACCGCCCCTACTGGAACCTCAGGTACTGTTGCTACCAGACTGGACAACATAATGAAAGATATG

>Bcin15g03910(MLST6), partial sequence [organism=Botrytis cinerea, strain D13_E_IL12]
TCTTGAAAGCCTTCTCTAGCATCTCCTCTCCTAACGTCATATGTCCCGTAGAATGGTTTCGAAAACTAATTTTCCGGCCTCATCCAATGTCTTT
AAACCTAGCCAATCTTCTTTATAAATGGCTCTTTCTTTCAGTGGTGTAACTTCCGTGCCGTTGACTTCAGCCCACCATCCACTTTCCTTAGGGA
TGACAGTTGTGTCGTCTTCAAAGACATACATTACGAATCGTTCCAATTTTTCCATGTTGGATTTATATGTTTGGTTCTTGAGAACGCGCTCATT
ATTGATGTCGGCAAGGAAATTGGAATATTCAAGGTAAGAGTCTAGGTTTTCCGGATCTCTGAAGTATTGAGCGGGTACGAGACGAGATTGGACA
AAGGTTGACCAGGTGTTGGATCGCAAAAGGGTTTGAGCACCGCGACAGAGGAAATCGGCAGGACCACAGGCTTGGAAGGCAGAAATGCCGTTGT
GTTGGGAACCGAAGGTCAGGAGAGAGCGGATGGGTGGAGCATTGCAGCGGGATATGTAACCACGCAAGAATTGGCCGCCTTGGGAGAATCCAAT
TGCGTCGACGGCGGGCGCGGTAGAGAGAATAGGATGGGAGGCGAGGTCTTCGCAGACCTTTTCGATTTGAACTTGGTAAGAGAGTTAGTATGAT
ATTTAAAGGAAAAATAGGGAATGTACTCACGAGTGAGATTTCCAAAGAAGGTAGCTGTCCTATCTGCAGATGCATCCTCATCTACATGAATATT
GTAGACAAAAGTCCCAGGATGAATAGCTTCAGCTAGTTTTCCAACTTGCGCAAGACCATCCGCTTTGTAATTATCGCCGAGTCCATGCCAGATA
ATCAAGGGAAGTGGAGTATCAT

>Bcin16g03460(MLST7), partial sequence [organism=Botrytis cinerea, strain D13_E_IL12]
TCTTCATGAACCTCTTAATTTGAACTCTTCATTATTTGCAAGTGATGAACCCCTATTTCTGCCCAGAGGATAGTTACAAGACGTATATCATTAG
TCGAGAACCACTCATGATATATATTGACGGATTTTTGAAAGCGAATGAAAGTAAACACTTGGTTGATGTTAGGTGTGTTATTTATTCTGATGAA
ATGAACAAGAGAGACTGATGAGATAGTGAACCGCTTTATGAACCGTCTACTGTTTCTCACGGGCAGGAAGTTACCATTGATACTTCAGTTCGAA
ATTCTGAAGTGGCGGTTTTAGAGAGGGATGAGGTGGTCAGGTGTATTGAGCATAGAGCGAGGGCATTTCAGGGGTGGAGGGGCGAGATGGGGAT
TGAGAAGTTGAGGACGCAGAGGTATGGGGTTGGAGGACATTATGGGATGCATTTGTAAGTTTTGGGGGATTGACGAAGGCTTTTGTCTATTTTC
TACCAGTACGGATCTTGAAAGAAAGATAGCATGAACACGAGGGCTAATAACTAGGAAATAGCGATTGGAGTGGAGGTAAACGTGGCATAGACCG
ATTTAGTACTTTCATGGTCTATGTCGACGTATCCTCTGATATCGAAGGTGGAGGAACGGAATTCCCACGTATCGTGGGACCAAAAGGAGGAAGG
TGGGAGGACTTCCTGGAAACTACGGAAGCATTGGATCCAAGAACTGGAGAAAATGTAACAGTAGAAGGGGTGACATTCAAACCAATCAAGGGAA
ATGCCGTATTCT

>Bcin12g03020(MLST8), partial sequence [organism=Botrytis cinerea, strain D13_E_IL12]
ACTGCGCAGCACCGAGTCACCAAAAGCACGAATAAACCATCGGCAAGGGAAATATTCTCTGAACCACAACATGACACGAGCGCGGAAGAGTATA
TCGGGCGAGAAGCTTCATCAAGAGCACCAAAGCGACAACGAGTCGATGATAATTATAACTCTTACGGTGGAAGAAATGAAAATACAGCAGCTTA

TGCTTCCGGGAAACTTCCATCTGGAAGTATAAATGTTGGTGGAGGTAGGAAGACCACTTTTCAAGAAGAACCACGAACGGCATTTGTCGCTGGC
AAGTTGCCCCCTGGAAGTATCAACATTGGTGGAAAGAAGGCTACCCAAATCGAGGACGAGGGTAGAGCAGCTTATGCTTCCGGAAAATTGCCTC
CAGGAAGTATAAACTCTGGCGCAAAGAAGGCGATTTCATTCCAAGATGAAACGAGACCGGCTTATGTCTCCGGAAAGCTTCCACATGGTAGTAT
CGACGGTATGCGAAACCGTGAAATGGCTGCCGTCCACCGCGAAATTGCTGAGGGTGGGAGGAAACCAGGCCAGGTTGTCTCTTCTCTATTCACA
TTCAATCCTACTTCAAAGAAAACTTTTGATGAACCCGAAGAACAAGCGGAACCGGCAAAACCATCCAATGCGCCTTTGACAGAGGAAATGGCGA
CTTTCACCAATCTAGGGTTATCGAGAAGGCTTGCAGCCCATCTATCGACTAAACTCGATATGAAAGCTCCGACCGCCATTCAAAAAGCATCTGT
ACAGCAGTTGGTATCGGACGATAGCGATGCTTTCATACAAGCAGAGACTGGATCTGGAAAAACTTTGGCATATCTACTACCTATAGTCGAGCGA
ATATTAGCATTGAGTGAGAATGGCGT

>Bcin02g07770(MLST9), partial sequence [organism=Botrytis cinerea, strain D13_E_IL12]
CGATTCGATGCCGTGTAGGATTGCCGAAACTGTTCCCAGTGGGACCGTAGGAACAGTTTATGAGGCATATAAACCATTCCCTTAAAGTCGTAAA
TGGATGCGTACCATTCCCTGCCGTCGATGCATCGGGTAACACAGGGTATGTCCTTATACCTTTCTCTTCCACACGATTGCTATTGAGTCTCTAA
CATATTTTAGTGGTGGTTTGTCACCAACTGGCAGCAGCAATGGTGAATGCAGCAGCAGTACCGGTCAAGTATATGTTCGAGGAGGACAAAGCGG
ATCAAACTACGCTATCATGTACTCCTGGTAAGTTCTCTCTAAACTTCTCCTTATAGATCCAACCTAACAAAATCTTAGGTACATGCCAAAGGAC
GAGCCCTCAACCGGTATTGGTCACCGTCACGATTGGGAAGGTGTAATTG

>Bcin04g02090(MLST10), partial sequence [organism=Botrytis cinerea, strain D13_E_IL12]
AACTGTCGAATATGACTATGAATGATGTTTACAAGCCCTACATCCATGTAAGAAATGTAGAATAGAGAGATCAGTAACTGGAACTAATATCGTT
TGTAGGCTTTCAAGTTACTTACGCAGTTCAACCCAATCACTACAGCTATTGCCGAATCCCCACTATTTCAAATGGCTGTCTCAGCAAATACCAT
CGAAAAGTACACACTGCTAGGCCCTTTCTTCAGAATATCTCCTCTGCAACAGGAAGTTACCAGGGAATACTTCAGTGCGCCAAAGACGATAGAT
AGGCGACACATTGCCACATCTCAAGATGCCTTACGATTGACCTTACAAACCCATCAAAAAGATTTACTTGATATCATCAACCACTTTGTTCGAG
CAAGTCCAATCGCAAAAAGCAAAACCCTGGATTGGTTCGCCTACATTGTGAATCAAAATCACAAGCGTCGAGCACTTCAGGTAGACCCGAAAGA
AGTGTCTTCTGATGGCTTTATGCACAATGTCACTGTCGTTCTAGATGGTCTTTGTGAGCCATTCATGGATACCACATTCTCGAAAATTTCGAAG
ATTGATATTGATTATCTAAGACGTGCGCCTCGTGTAGATATCAAGGACGAGACCAAGTTGAACGCTGACGAGAAGGCTTCTGAGAAGTATTATG
AGGACACTGTTCCTGGCACTTCTAATTTCATCTCTGAGGTCTTCTTTCTCACATTGG

>Bcin01g07220(MLST1), partial sequence [organism=Botrytis cinerea, strain G11_MG1_E5]
CTGAAATAGAATCGGTATTGTGAACGAGTGAGAGAGTAGCCCGAGAGACGGAATGTGACAGTTACTTATGAGACGCGATTGAATGTCAACGGTG
GAGGTGTTGAAGTATCTGGCGGATAGAGACCAATGAGCAGAGCAGCTATTGTAGATTTTCCGGAGCCGGAGGGTCCGACAAGTGCAGTTGTTGA
GTTAGGAATCAGGGAAAGAGAAAAGGATGATATCGTTCGTTTTTCAGGACGAGTAGGATATGTGAATGAGAGACGGTTGAATTTGATTGGAAAG
ATTGTCGAAAGCCGTTCGGTTCCTTTATTTTCGTGGGAGGAAGATGAATCGAGATTGGCTAATGCAAGCATATGCGTAGCTGTAACGCGAGAAG
AGTTGATTTGTGGTATCATGGCCAGCATATTCGTACTATTAGAAATACCAAATAATAGAAGATTGACAGTCTGTAGTGCAGTCGCGATACTGAT
CTCTCTCTTGGTAATGAGAACCGTGGCATAATAAAAGATAGTTGCAGTGATGAAGAATGACATCGCATCTGTCAATCCCCACAGCAATCCCGAG
TAGTTTGCTCGTATTAGTCCAACTTTATAAAGTTCTTCTGTAGCTTTGGTGTGTTTTGTCTCAAAGTAAGTTTCCAGAGTAAAAGCCCGAACCA
CTTTGATGTTGGAGAAAGTCTCTGAAAATATGCCAGTGGTCATTTCAGAGGCGTAGTTGCACTTGTTTTCCCATTTTCCACTCACGCGATTGAA
CGTTTTGGTGACAGCGTATATAACTGGCCCAGTTGCCATCGAGACTAATGTCAGTTTCCATGTATTCACGAAAGCCCAAATGATTGATATCAAT
AGCATAAAAAATGCTGTGAATACAATACCAGCAAAGCGGCCAACGAGATTACGCATTTCCTCAGAGTTCCTATCCAAAACTTCGT

>Bcin05g07690(MLST2), partial sequence [organism=Botrytis cinerea, strain G11_MG1_E5]
AGGATGCCTTCTCTCTACGACAGCAAGCCTTCGATGATATGTTGTGGGTAGTTCTTGGCTGGCTGGATACTGTCAAATTCATTGATTTACATTC
TGAATTGCACTATTCAAACGACTCTCAGCCAGAATGGTACGGACAACAATATAAACCTGCATTTGCACATCGAGCGCGACTATTTTGGGAATTG
GCTTCACAAGGATGGGATACTACTCTCTGTGGTGGTGGGATGATATGGTCACCATACCTTACTCCATACAAGAACGCAATTACCAATGAACTCT
ATATCGCAGCTTCGATATCGATGTACCTATATTTCCCCGGAGATGACAATCAATCCCCATTTATGCTTTCCAACCCTTCATATCCACCTCACGA
TCCGAAATATCTACAGGCAGCTGTTGATGCTTACAAATGGCTGAATGGTTCCAACATGACGGATTTACAAGGATTATATGTCGACGGGTACCAT
ATCTCGAATCTTTCTGGCGGTGAAAACACCCATTGCGATTCTAGAAATGAGATGGTATATACCTACAATCAAGGTGTTTTGCTTACTGGACAAC
GTGGTTTGTATGACGCAACCGCCGCACGATCATACCTTGTAGATGGCCACAAACTCATCGCGAATGTTATTAATGCCACAGGCTATGACCTGAA
ACACAATGTTGTCATCTCACCGCCACCCAAAGATGGTTCCGCATTGGCAAAGTGGTTTGGCCTGGGTAGGAATGGAATACTGGAAGAAGGATGC
GATTCAAGTGCTTCGTGTTCTCAAAATGGACAAACTTTCAAAGGCATATTCTTTCATCACTTGATTGCGTTCTGTAGTGATTTGCCAGGGGAGC
CTATTGCAGGGACGAAGGAA

>Bcin06g01710(MLST3), partial sequence [organism=Botrytis cinerea, strain G11_MG1_E5]
AGCTTCGGAAGCGTCTTCCGAGAGGAATTTGCCAGTCCCGATAGTCGGTTACGAGTCCACTCTCTGAAAACTCGTTGCGAAGATAACTTGGTGT
TATGGAGAGTGCATCGATCAGATCTTTGCGTTTCTTGACATATAGACAAGAAGCGTCGAAATTTGTCAGAAGCCATTTGTGCATGTTCATATCG
AAGGAATGGAAATGCTGGAAGGACGATGTTAGATGATGGTATTCAGGGCAAACCAAAGCTGCACCTGCATAAGCAGCATCGACGTGAACCCAGA
TCTCGCCTGCAACATCTGGAGGTGCATATTTTGAAAGTACTGTTGCAATAGATGCGAAGTCGTCAACTGCGCATGTAGATGTTGTTCCCAAAGT
CGAAGTTAGATAGAAGGGTTCCAATCCTTGAGATTTGCATTCTTTCAATACCTTCTCTAAATCATCACCCGTCATGGCGAAATCATTGGATGCG
AGTACTGGAATCGATCGGAATCTAACGCCAGCTATCTGCGCTGCTTTCTGCGTGGAGCTGTGTGCCATTTCGCTTCCTAGTGCAACTAGCTTAC

TCCTCTTATATGCAATCGCATCCTCGAGTTCAATGCCCGACAGACCTTCAGTGGTTTCACGAAGATATTTATCGCGGGCAGCAACCATAACGGT
AACGATAGCTTCCGAGGCTGATCCTTGGATAACACCACCACCATGAGTCGAAGACAAATAACAGTCTGGGAGATTGAGAAGCTTGGCCAGCCAA
TCCATTACAACCGTCTCCAATTCTGTCACAGCAGGGGAACAGATCCAATTGAAAGCAGGAGCTGTGAAAGCTGCTGAGTATAATTCTCCCAGCA
TTCCAGGGTAGGTAGATGATGCAGGGAAGAACGCCATGAAATTAGGTGATTGCCTTG

>Bcin09g03030(MLST4), partial sequence [organism=Botrytis cinerea, strain G11_MG1_E5]
GCAGAGAGAGTCTTGGAGGAGGTCGCCAAGAGTTGGAAGAATAGGAGTGGCGGTGTCATTGTCGAGGGCGAGGGAACGGAATTGAAGGAGATTC
TGAAAGCTTTGGAAGGGAATATGAGTGGTGGAAGGATAGTCATAGGAAGAGAGCTAAGCCGGCAGAATAGTTTAGTACTGGGATCATCACAATA
TGGAGAGGTCAATCATACAAGACTTGGGCTACGGCCAGGGAATATACCCAGAGAGGATAGTCAGTCAAGTTTGGGAATGTCAACGTTGGAGGTC
AATGACGAGGAAGATGAGGATGGCCTGATGGATCCAAGAAGGTGGTTAAAAGTCATTGATGCATTTGAGCAACCTCGACTGGTGTACAATGTTG
CTAAAAAGCACTTTGATAGGTATGTTTCAATGATAAGATTTTATCTGAATCGACTAACTCAGTACAGAGATACCTCCAAACCTTCATTGTTCCC
ACCTGCGTCTCATAAAACACTCCTCTTCCAAAACCGCTATAATGTTATCCATCAACGTCTCCTTCGCAATGAATCTTTTCAAACGCCCGCTTTT
CAAGGTGGCAAATCTTCCCTTCAACGCAGCACGTCCGCCATTACCACCCAACAACAATCATACAAATTAACGCCGATAGCTAATCTTCTCGGTC
GCAATCGCAGCTCTCATATGCTTCTCGGTCTCCTCAGTATTTCACCCACTGGTACCCTCGCCATCAATGACCTG

>Bcin11g01310(MLST5), partial sequence [organism=Botrytis cinerea, strain G11_MG1_E5]
TCATGTGGAACCGGCGTAGAATGCGCAACGCTTGAAGTTCCGCTCGAATATGGCGATGCAACGTCAACGGCAAAAGCCAGTGTTGCGCTTGCTC
GTTATCCTGCCACTGTTGCCGCGAGCAAGAAGCTCGGGTCTCTCTTGATAAACCCCGGTGGACCCGGTGCCTCTGGTGTTGGCTTTGTGCAGTC
TGGAGCCGGTGCCGCCGTCTCGACACTGAGTGGTGGATTGTACGATATCATCGGATGGGATCCACGTGGAACCGGTGCTTCGGCTCCTATTTTG
GAATGTTTTGCAAATGCCAGTGCGGAGTATGATTTTAACAACGCGTTTCCATCTGCTCCGAATCTCTGGCTCGGACAATTTGCGAATGCCAGCG
CAAATTCTGCTGTTAGCTCTGCTATCACATCCTTTGACACTTCTGTCGCTGCTCTTGCAAAAGCTTGCGTGGCTCAGAAATCTCCCGCTCTTTA
CACCTCAACAGCAGCATATGTTGCTCGAGACATGGCAGCGATAGTCGATGCATTGGATGGGACCTCTGCAAAACTTAACTACTGGGGTTTCTCA
TATGGAACTATTTTCCTAGCTGAGTTTATCCAAACTTTCCCAGGCCGCGTGGGAAGAGTTCTTGCCGATGGTGTTTTCGACGCAAAGGCAAATG
CACTCACATACGTTAGCCAACTTCCCAACGATCAACTCAGTGTTCGTGCTTCGTTGAACGATTTTGCAGCTTTCTGCACCACCGCCGGTAGTAA
AGGTTGCTCTTTTGCCACCGCCCCTACTGGAACCTCAGGTACTGTTGCTACCAGACTGGACAACATAATGAAGGATATG

>Bcin15g03910(MLST6), partial sequence [organism=Botrytis cinerea, strain G11_MG1_E5]
TCTTTGAAAGCGTTCTCTAGCATCTCCTCTCCTAACGTCATATGTCCCCGTGGAATGGTTTCGAAAACTAATTTTCCGGCCTCATCCAATGTCT
TTAAACCTAACCAATCTTCTTTATAAATGGCTCTTTCTTTCAGTGGTGTAACTTCCGTGCCGTTGACTTCAGCCCACCATCCACTTTCCTTAGG
GATGACAGTTGTATCGTCTTCAAAGACATACATTACGAATCGTTCCAATTTTTCCATGTTGGATTTATATGTTTGGTTCTTGAGAACGCGCTCA
TTATTGATGTCGGCAAGGAAATTGGAATATTCAAGGTAAGAGTCTAGGTTTTCCGGATCTCTGAAGTATTGAGCGGGTACGAGACGAGATTGGA
CAAAGGTTGACCAGGTGTTGGATCGCAAAAGGGTTTGAGCACCGCGACAGAGGAAATCGGCAGGACCACAGGCTTGGAAGGCAGAAATGCCGTT
GTGTTGGGAACCGAAGGTCAGGAGAGAGCGGATGGGTGGAGCATTGCAGCGGGATATGTAACCACGCAAGAATTGGCCGCCTTGGGAGAATCCA
ATTGCGTCGACGGCGGGCGCGGTAGAGAGAATAGGATGGGAGGCGAGGTCTTCGCAGACCTTTTCGATTTGAACTTGGTAAGAGAGTTAGTATG
ATATTTAAAGGAAAAATAGGGGATGTACTCACGAGTGAGATTTCCAAAGAAGGTAGCTGTCCTATCTGCAGATGCATCCTCATCTACATGAATA
TTGTAGACAAAAGTCCCAGGATGAATAGCTTCAGCTAGTTTTCCAACTTGCGCAAGACCATCCGCTTTGTAATTATCGCCGAGTCCATGCCAGA
TAATCAAGGGAAGTGGAGTATCAT

>Bcin16g03460(MLST7), partial sequence [organism=Botrytis cinerea, strain G11_MG1_E5]
TCTTCATGAACCTCTTAATTTGAACTCTTCATTATTTGCAAGTGATGAACCCCTATTTCTGCCCAGAGGATAGTTACAAGACGTATATCATTAG
TCGAGAACCACTCATGATATATATTGACGGATTTTTGAAAGCGAATGAAAGTAAACATTTGGTTGATGTTAGGTGTGTTATTTATTCTGATGAA
ATGAACAAGAGAGACTGATGAGATAGTGAACCGCTTTATGAACCGTCTACTGTTTCTCACGGGCAGGAAGTTACCATTGATACTTCAGTTCGAA
ATTCTGAAGTGGCGGTTTTAGAGAGGGATGAGGTGGTCAGGTGTATTGAGCATAGAGCGAGGGCATTTCAGGGGTGGAGGGGCGAGATGGGGAT
TGAGAAGTTGAGGACGCAGAGGTATGGGGTTGGAGGACATTATGGGATGCATTTGTAAGTTTTGGGGGATTGACGAAGGCTTCTGTCTATTTTC
TACCAGTACGGATCTTGAAAGAAAGATGGCATGAACACGAGGGCTAATAACTAGGAAATAGCGATTGGAGCGGAGGTAAACGTGGCATAGACCG
ATTTAGTACTTTCATGGTCTATGTCGACGTATCCTCTGATATCGAAGGTGGAGGAACGGAATTCCCACGTATTGTGGGACCAAAAGGAGGAAGG
TGGGAGGACTTCCTGGAAACTACGGAAGCATTGGATCCAAGAACTGGAGAAAATGTAACAGTAGAAGGGGTGACATTCAAACCAATCAAGGGAA
ATGCCGTATTCT

>Bcin12g03020(MLST8), partial sequence [organism=Botrytis cinerea, strain G11_MG1_E5]
ACTGCGCAGCACCGAGTCACCAAAAGCACGAATAAACCATCGGCAAGGGAAATATTCTCTGAACCACAACATGACACGAGCGCGGAAGAGTATA
TCGGGCGAGAAGCTTCATCAAGAGCACCAAAGCGACAACGAGTCGATGATAATTATAACTCTTACGGTGGAAGAAATGAAAATACAGCAGCTTA
TGCTTCCGGGAAACTTCCATCTGGAAGTATAAATGTTGGTGGAGGTAGGAAGACCACTTTTCAAGAAGAACCACGAACGGCATTTGTCGCTGGC
AAGTTGCCCCCTGGAAGTATCAACATTGGTGGAAAGAAGGCTACCCAAATCGAGGACGAGGGTAGAGCAGCTTATGCTTCCGGAAAATTGCCTC
CAGGAAGTATAAACTCTGGCGCAAAGAAGGCGATTTCATTCCAAGATGAAACGAGACCGGCTTATGTCTCCGGAAAGCTTCCACATGGTAGTAT
CGACGGTATGCGAAACCGTGAAATGGCTGCCGTCCACCGCGAAATTGCTGAGGGTGGGAGGAAACCAGGCCAGGTTGTCTCTTCTCTATTCACA
TTCAATCCTACTTCAAAGAAAACTTTTGATGAACCCGAAGAACAAGCGGAACCGGCAAAACCATCCAATGCGCCTTTGACAGAGGAAATGGCGA

```
CTTTCACCAATCTAGGGTTATCGAGAAGGCTTGCAGCCCATCTATCGACTAAACTCGATATGAAAGCTCCGACCGCCATTCAAAAAGCATCTGT
ACAGCAGTTGGTATCGGACGATAGCGATGCTTTCATACAAGCAGAGACTGGATCTGGAAAAACTTTGGCATATCTACTACCTATAGTCGAGCGA
ATATTAGCATTGAGTGAGAATGGCGTACA

>Bcin02g07770(MLST9), partial sequence [organism=Botrytis cinerea, strain G11_MG1_E5]
CATCTTCAGCCAGCGAGGATGACGAACTACCGCGGATAGACCCTATTGACAGATTTTAGCCCTAGTGGTGCTGAATCGGAAAAAGATTATTTCC
GAACCCCCACTGACCTCTATTCTAAGTTCAAGTGACGTGAGTGTAGTATATCTCACGCCTTCAACAGATACTAAATATCAAACATTACACAGGC
AAAAGCCAATAGACTCCCAGAATATAGCCCCTTATATTCATACATAAAGACACAAAGTCTAGAAAGTAGCCTTCGCAAGATTGTCTGTGAAAAC
AGCCGGAATGAATGGAACATTCGCAGCACCGAAATCGGTGTTCTCAAGAGCAGTTTGAGCAGCAGTTGGTAAAGACTCCCAAGCAATCATAGGT
TGTTTTCCACCAACAGTACTAGTAAGACCCATTGAGTGATCGACGGGCCAGATACTTTCGTACTTGATAAGAGGGCTGGTACCAGAAAGGGAAT
AGCCATCCGTGGAACAATCCCAGCCTCCGTGGGCGGAAGGACAAACGGCTAAGATGTTGTCGGCAGTTGTGGCGGTGGCGCTGGAGAGCCAGAC
AATTACACCTTCCCAATCGTGACGGTGACCAATACCGGTTGAGGGCTCGTCCTTTGGCATGTACCTAAGATTTTGTTAGGTTGGATCTATAAGG
AGAAGTTTAGAGAGAACTTACCAGAGTACATGATGGCGTAGTTTGATCCGCTTTGTCCTCCTCGAACATATACTTGACCGGTACTGCTGCTGCA
ACCACCATTGCTACTGCCAGTTGGTGACAAACCACCACTAAAATATGTTAGAGACTCATAGCAATCGTGTGGAAGAGAAAGATATAAGGACATA
CCCTGTGTTACCGATGCATCGACGGCAGGGAATGGTACGCATCCCTTTACGACT
TTA

>Bcin04g02090(MLST10), partial sequence [organism=Botrytis cinerea, strain G11_MG1_E5]
AACTGTCGAAAATGACTATGAATGATGTTTACAAGCCCTACATCCATGTAAGAAATGTAGAATAGAGAGATCAGTAACTGGAACTAATATCGTT
TGTAGGCTTTCAAGTTACTTACGCAGTTCAACCCAATCACTACAGCTATTGCCGAATCCCCACTATTTCAAATGGCTGTCTCAGCAAATACCAT
CGAAAAGTACACACTGCTAGGCCCTTTCTTCAGAATATCTCCTCTGCAACAGGAAGTTACCAGGGAATACTTCAGTGCGCCAAAGACGATAGAT
AGGCGACACATTGCCACATCTCAAGATGCCTTACGATTGACCTTACAAACCCATCAAAAAGATTTACTTGATATCATCAACCACTTTGTTCGAG
CAAGTCCAATCGCAAAAAGCAAAACCCTGGATTGGTTCGCCTACATTGTGAATCAAAATCACAAGCGTCGAGCACTTCAGGTAGACCCGAAAGA
AGTGTCTTCTGATGGCTTTATGCACAATGTCACTGTCGTTCTAGATGGTCTTTGTGAGCCATTCATGGATACCACATTCTCGAAAATTTCGAAG
ATTGATATTGATTATCTAAGACGTGCGCCTCGTGTAGATATCAAGGACGAGACCAAGTTGAACGCTGACGAGAAGGCTTCTGAGAAGTATTATG
AGGACACTGTTCCTGGCACTTCTAATTTCATCTCTGAGGTCTTCTTTCTCACATTGG

>Bcin01g07220(MLST1), partial sequence [organism=Botrytis cinerea, strain G11_MG1_E7]
GGTGTTGAAGTATCTGGCGGATAGAGACCAATGAGCAGAGCAGCTATGTAGATTTTCCGGAGCCGGAGGGTCCGACAAGTGCAGTTGTTGAGTT
AGGAATCAGGGAAAGAGAAAAGGATGATATCGTTCGTTTTTCAGGACGAGTAGGGTATGTGAATGAAAGACGGTTGAATTTGATTGGAAAGATT
GTCGAAAGCCGTTCGGTTCCTTTATTTTCGTGGGAGGAAGATGAATCGAGATTGGCTAATGCAAGCATATGCGTAGCTGTAACGCGAGAAGAGT
TGATTTGTGGTATCATGGCCAGCATATTCGTACTATTAGAAATACCAAATAATAGAAGATTGACAGTCTGTAGTGCAGTCGCGATACTGATCTC
TCTCTTGGTAATGAGAACCGTGGCATAATAAAAGATAGTTGCAGTGATGAAGAATGACATCGCATCTGTCAATCCCCACAGCAATCCCGAGTAG
TTTGCTCGTATTAGTCCAACTTTATAAAGTTCTTCTGTAGCTTTGGTGTGTTTTGTCTCAAAGTAAGTTTCCAGAGTAAAAGCCCGAACCACTT
TGATGTTGGAGAAAGTCTCTGAAAATATGCCAGTGGTCATTTCAGAGGCGTAGTTGCACTTGTTTTCCCATTTTCCACTCACGCGATTGAACGT
TTTGGTGACAGCGTATATAACTGGCCCAGTTGCCATCGAGACTAATGTCAGTTTCCATGTATTCACGAAAGCCCAAATGATTGATATCAATAGC
ATAAAAAATGCTGTGAATACAATACCAGCAAAGCGGCCAACGAGATTACGCATTTCCTCAGAGTTCCTATCCAAAACTTCGT

>Bcin05g07690(MLST2), partial sequence [organism=Botrytis cinerea, strain G11_MG1_E7]
AGGATGCCTTCTCTCTACGACAGCAAGCCTTCGATGATATGTTGTGGGTAGTTCTTGGCTGGCTGGATACTGTCAAATTCATTGATTTACATTC
TGAATTGCACTATTCAAACGACTCTCAGCCAGAATGGTACGGACAACAATATAAACCTGCATTTGCACATCGAGCGCGACTATTTTGGGAATTG
GCTTCACAAGGATGGGATACTACTCTCTGTGGTGGTGGGATGATATGGTCACCATACCTTACTCCATACAAGAACGCAATTACCAATGAACTCT
ATATCGCAGCTTCGATATCGATGTACCTATATTTCCCCGGAGATGACAATCAATCCCCATTTATGCTTTCCAACCCTTCATATCCACCTCACAA
TCCGAAATATCTACAGGCAGCTGTTGATGCTTACAAATGGCTGAATGGTTCCAACATGACGGATTTACAAGGATTATATGTCGACGGGTACCAT
ATCTCGAATCTTTCTGGCGGTGAAAACACCCATTGCGATTCTAGAAATGAGATGGTATATACCTACAATCAAGGTGTTTTGCTTACTGGACAAC
GTGGTTTGTATGACGCAACCGCCGCACGATCATACCTTGTAGATGGCCACAAACTCATCGCGAATGTTATTAATGCCACAGGCTATGACCTGAA
ACACAATGTTGTCATCTCACCGCCACCCAAAGATGGTTCCGCATTGGCAAAGTGGTTTGGCCTGGGTAGGAATGGAATACTGGAAGAAGGATGC
GATTCAAGTGCTTCGTGTTCTCAAAATGGACAAACTTTCAAAGGCATATTCTTTCATCACTTGATTGCGTTCTGTAGTGATTTGCCAGGGGAGC
CTATTGCAGGGACGAAGGAA

>Bcin06g01710(MLST3), partial sequence [organism=Botrytis cinerea, strain G11_MG1_E7]
AGCTTCGGAAGCGTCTTCCGAGAGGAATTTGCCAGTCCCGATAGTCGGTTACGAGTCCACTCTCTGAAAACTCGTTGCGAAGATAACTTGGTGT
TATGGAGAGTGCATCGATCAGATCTTTGCGTTTCTTGACATATAGACAAGAAGCGTCGAAATTTGTCAGAAGCCATTTGTGCATGTTCATATCG
AAGGAATGGAAATGCTGGAAGGACGATGTTAGATGATGGTATTCAGGGCAAACCAAAGCTGCACCTGCATAAGCAGCATCGACGTGAACCCAGA
TCTCGCCTGCAACATCTGGAGGTGCATATTTTGAAAGTACTGTTGCAATAGATGCGAAGTCGTCAACTGCGCATGTAGATGTTGTTCCCAAAGT
CGAAGTTAGATAGAAGGGTTCCAATCCTTGAGATTTGCATTCTTCCAATACCTTCTCTAAATCATCACCCGTCATGGCGAAATCATTGGATGCG
AGTACTGGAATCGATCGGAATCTAACGCCAGCTATCTGCGCTGCTTTCTGCGTGGAGCTGTGTGCCATTTCGCTTCCTAGTGCAACTAGCTTAC
```

TCCTCTTATATGCAATCGCATCCTCGAGTTCAATGCCCGACAGACCTTCAGTAGTTTCACGAAGATATTTATCGCGGGCAGCAACCATAACGGT
AACGATAGCTTCCGAGGCTGATCCTTGGATAACACCACCACCATGAGTCGAAGACAAATAACAGTCTGGGAGATTGAGAAGCTTGGCCAGCCAA
TCCATTACAACCGTCTCCAATTCTGTCACGGCAGGGGAACAGATCCAATTGAAAGCAGGAGCTGTGAAAGCTGCTGAGTATAATTCTCCCAGCA
TTCCAGGGTAGGTAGATGATGCAGGGAAGAACGCCATGAAATTAGGTGATTGCCTTG

>Bcin09g03030(MLST4), partial sequence [organism=Botrytis cinerea, strain G11_MG1_E7]
GCAGAGAGAGTCTTGGAGGAGGTCGCCAAGAGTTGGAAGAATAGGAGTGGCGGTGTCATTGTCGAGGGCGAGGGAACGGAATTGAAGGAGATTC
TGAAAGCTTTGGAAGGGAATATGAGTGGTGGAAGGATAGTCATAGGAAGAGAGCTAAGCCGGCAGAATAGTTTAGTACTGGGATCATCACAATA
TGGAGAGGTCAATCATACAAGACTTGGGCTACGGCCAGGGAATATACCCAGAGAGGATAGTCAGTCAAGTTTGGGAATGTCAACGTTGGAGGTC
AATGACGAGGAAGATGAGGATGGCCTGATGGATCCAAGAAGGTGGTTAAAAGTCATTGATGCATTTGAGCAACCTCGACTGGTGTACAATGTTG
CTAAAAAGCACTTTGATAGGTATGTTTCAATGATAAAATTTTATCTGAATCGACTAACTCAGTACAGAGATACCTCCAAACCTTCATTGTTCCC
ACCTGCGTCTCATAAAACACTCCTCTTCCAAAACCGCTATAATGTTATCCATCAACGTCTCCTTCGCAATGAATCTTTTCAAACGCCCGCTTTT
CAAGGTGGCAAATCTTCCCTTCAACGCAGCACGTCCGCCATTACCACCCAACAACAATCATACAAATTAACGCCGATAGCTAATCTTCTCGGTC
GCAATCGCAGCTCTCATATGCTTCTCGGTCTCCTCAGTATTTCACCCACTGGTACCCTCGCCATCAATGACCTG

>Bcin11g01310(MLST5), partial sequence [organism=Botrytis cinerea, strain G11_MG1_E7]
TCATGTGGAACCGGCGTAGAATGCGCAACGCTTGAAGTTCCGCTCGAATATGGCGATGCAACGTCAACGGCAAAAGCCAGTGTTGCGCTTGCTC
GTTATCCTGCCACTGTTGCCGCGAGCAAGAAGCTCGGGTCTCTCTTGATAAACCCCGGTGGACCCGGTGCCTCTGGTGTTGGCTTTGTGCAGTC
TGGAGCCGGTGCCGCCGTCTCGACACTGAGTGGTGGATTGTACGATATCATCGGATGGGATCCACGTGGAACCGGTGCTTCGGCTCCTATTTTG
GAATGTTTTGCAAATGCTAGTGCGGAGTATGATTTTAACAACGCGTTTCCATCTGCTCCGAATCTCTGGCTCGGACAATTTGCGAATGCCAGCG
CAAATTCTGCTGTTAGCTCTGCTATCACATCCTTTGACACTTCTGTCGCTGCTCTTGCAAAAGCTTGCGTGGCTCAGAAATCTCCCGCTCTTTA
CACCTCAACAGCAGCATATGTTGCTCGAGACATGGCAGCGATAGTCGATGCATTGGATGGGACCTCTGCAAAACTTAACTACTGGGGTTTCTCA
TATGGAACTATTTTCCTAGCTGAGTTTATCCAAACTTTCCCAGGCCGCGTGGGAAGAGTTCTTGCCGATGGTGTTTTCGACGCAAAGGCAAATG
CACTCACATACGTTAGCCAACTTCCCAACGATCAACTCAGTGTTCGTGCTTCGTTGAACGATTTTGCAGCTTTCTGCACCACCGCCGGTAGTAA
AGGTTGCTCTTTTGCCACCGCCCCTACTGGAACCTCAGGTACTGTTGCTACCAGACTGGACAACATAATGAAGGATATG

>Bcin15g03910(MLST6), partial sequence [organism=Botrytis cinerea, strain G11_MG1_E7]
TCTTTGAAAGCCTTCTCTAGCATCTCCTCTCCTAACGTCATATGTCCCCCTGGAATGGTTTCGAAAACTAATTTTCCGGCCTCATCCAATGTCT
TTAAACCTAGCCAATCTTCTTTATAAATGGCTCTTTCTTTCAGTGGTGTAACTTCCGTGCCGTTGACTTCAGCCCACCATCCACTTTCCTTAGG
GATGACAGTTGTATCGTCTTCAAAGACATACATTACGAATCGTTCCAATTTTTCCATGTTGGATTTATATGTTTGGTTCTTGAGAACGCGCTCA
TTATTGATGTCGGCAAGGAAATTGGAATATTCAAGGTAAGAGTCTAGGTTTTCCGGATCTCTGAAGTATTGAGCGGGTACGAGACGAGATTGGA
CAAAGGTTGACCAGGTGTTGGATCGCAAAAGGGTTTGAGCACCGCGACAGAGGAAATCGGCAGGACCACAGGCTTGGAAGGCAGAAATGCCGTT
GTGTTGGGAACCGAAGGTCAGGAGAGAGCGGATGGGTGGAGCATTGCAGCGGGATATGTAACCGCGCAAGAATTGGCCGCCTTGGGAGAATCCA
ATTGCGTCGACGGCGGGCGCGGTAGAGAGAATAGGATGGGAGGCGAGGTCTTCGCAGACCTTTTCGATTTGAACTTGGTAAGAGAGTTAGTATG
GTATTTAAAGGGAAAATAGGGGATGTACTCACGAGTGAGATTTCCAAAGAAGGTAGCTGTCCTATCTGCAGATGCATCCTCATCTACATGAATA
TTGTAGACAAAAGTCCCAGGATGAATAGCTTCAGCTAGTTTTCCAACTTGCGCAAGACCATCCGCTTTGTAATTATCGCCGAGTCCATGCCAGA
TAATCAAGGGAAGTGGAGTATCAT

>Bcin16g03460(MLST7), partial sequence [organism=Botrytis cinerea, strain G11_MG1_E7]
TCTTCATGAACCTCTTGATTTGAACTCTTCATTAATTGCAAGTGATGAACCCCTATTCTGCCCAGAGGATAGTTACAAGACGTATATCATTAGT
CGAGAACCACTCATGATATATATTGACGGATTTTTGAAAGCGAATGAAAGTAAACATTTGGTTGATGTTAGGTGTGTTATTCATTCTGATGAAA
TGAACAAGAGAGACTGATGAGATAGTGAACCGCTTTATGAACCATCTACTGTTTCTCACGGACAGGAAGTTACCATTGATCCTTCAGTTCGGAA
TTCTGAAGTGGCGGTTTTAGAGAGGGATGAGGTGGTCAGGTGTATTGAGCATAGAGCGAGGGCATTTCAGGGGTGGAGGGGCGAGATGGGGATT
GAGAAGTTGAGGACGCAGAGGTATGGGGTTGGAGGACATTATGGGATGCATTTGTAAGTTTTGGGGGATTGACGAAGGCTTTTGTCTATTTTCT
ACCAGTACGGATCTTGAAAGAAAGATAGCATGAACACGAGGGCTGATAACTAGGAAATAGCGATTGGAGCGGAGGTAAACGTGGCATAGACCGA
TTTAGTACTTTCATGGTCTATGTCGACGTATCCTCTGATATCGAAGGTGGAGGAACGGAATTCCCACGTATCGTGGGACCAAAAGGAGGAAGGT
GGGAGGACTTCCTGGAAACTACGGAAGCATTGGATCCAAGAACTGGAGAAAATGTAACAGTAGAAGGGGTGACATTCAAACCAATCAAGGGAAA
TGCCGTATTCT

>Bcin12g03020(MLST8), partial sequence [organism=Botrytis cinerea, strain G11_MG1_E7]
ACTGCGCAGCACCGAGTCACCAAAAGCACGAATAAACCATCGGCAAGGGAAATATTCTCTGAACCACAACATGACACGAGCGCGGAAGAGTATA
TCGGGCGAGAAGCTTCATCAAGAGCACCAAAGCGACAACGAGTCGATGATAATTATAACTCTTACGGTGGAAGAAATGAAAATACAGCAGCTTA
TGCTTCCGGGAAACTTCCATCTGGAAGTATAAATGTTGGTGGAGGTAGGAAGACCACTTTTCAAGAAGAACCACGAACGGCATTTGTCGCTGGC
AAGTTGCCCCCTGGAAGTATCAACATTGGTGGAAAGAAGGCTACCCAAATCGAGGACGAGGGTAGAGCAGCTTATGCTTCCGGAAAATTGCCTC
CAGGAAGTATAAACTCTGGCGCAAAGAAGGCGATTTCATTCCAAGATGAAACGAGACCGGCTTATGTCTCCGGAAAGCTTCCGCATGGTAGTAT
CGACGGTATGCGAAACCGTGAAATGGCTGCCGTCCACCGCGAAATTGCTGAGGGTGGGAGGAAACCAGGCCAGGTTGTCTCTTCTCTATTCACA
TTTAATCCTACTTCAAAGAAAACTTTTGATGAACCCGAAGAACAAGCGGAACCGGCAAAACCATCCAATGCGCCTTTGACAGAGGAAATGGCGA

CTTTCACCAATCTAGGGTTATCGAGAAGGCTTGCAGCCCATCTATCGACTAAACTCGATATGAAAGCTCCGACCGCCATTCAAAAAGCATCTGT
ACAGCAGTTGGTATCGGACGATAGCGATGCTTTCATACAAGCAGAGACTGGATCTGGAAAAACTTTGGCATATCTACTACCTATAGTCGAGCGA
ATATTAGCATTGAGTGAGAATGGCGT

>Bcin02g07770(MLST9), partial sequence [organism=Botrytis cinerea, strain G11_MG1_E7]
CGATTCCGATGCCGTGTAGGATTTGCCGAAACTGTTCCCAGTGGGACCGTAGGAACAGTTTATGAGGCATATAAACCATTCCCTTAAAGTCGTA
AATGGATGCGTACCATTCCCTGCCGTCGATGCATCGGGTAACACAGGGTATGTCCTTATACCTTTCTCTTCCACACGATTGCTATTGAGTCTCT
GACATATTTTAGTGGTGGTTTGTCACCAACTGGCAGTAGCAATGGTGGTTGCAGCAGCAGTACCGGTCAAGTATATGTTCGAGGAGGACAAAGC
GGATCAAACTACGCCATCATGTACTCCTGGTAAGTTCTCTCTAAACTTCTCCTTATAGATCCAACCTAACAAAATCTTAGGTACATGCCAAAGG
ACGAGCCTTCAACCGGTATTGGTCACCGTCACGATTGGGAAGGTGTAATTGTCTGGCTCTCCAGCGCCACCGCCACAACTGCCGACAACATCTT
AGCCGTTTGTCCTTCTGCCCACGGAGGCTGGGATTGTTCCACCGATGGCTATTCCCTTTCTGGTACCAGCCCTCTTATCAAGTACGAAAGTATC
TGGCCCGTCGATCATTCAATGGGTCTTACTAGTACTGTTGGTGGAACACAACCTATGATCGCTTGGGAGTCTTTACCTACTGCTGCTCAAACTG
CTCTTGAGAACACCGATTTCGGTGCTGCGAATGTTCCATTCATTCCGGCTGTTTTCACAGATAATCTTGCGAAGGCTACTTTCTAG

>Bcin04g02090(MLST10), partial sequence [organism=Botrytis cinerea, strain G11_MG1_E7]
AACTGTCGAATATGACTATGAATGATGTTTACAAGCCCTACATCCATGTAAGAAATGTAGAATAGAGAGATCAGTAACTGGAACTAATATCGTT
TGTAGGCTTTCAAGTTACTTACGCAGTTCAACCCAATCACTACAGCTATTGCCGAATCCCCACTGTTTCAAATGGCCGTCTCAGCAAATACCAT
CGAAAAGTACACACTGCTAGGCCCTTTCTTCAGAATATCTCCTCTGCAACAGGAAGTTACCAGGGAATACTTCAGTGCGCCAAAGACGATAGAT
AGGCGACACATTGCCACATCTCAAGATGCGTTACGATTGACTCTACAAACCCATCAAAAAGATTTACTTGATATCATCAACCACTTTGTTCGAG
CAAGTCCAATCGCAAAAAGCAAAACCCTGGATTGGTTCGCCTACATTGTGAATCAAAATCACAAGCGTCGAGCACTTCAGGTAGACCCGAAAGA
AGTGTCTTCTGATGGCTTTATGCACAATGTCACTGTCGTTCTAGATGGTCTTTGTGAGCCATTCATGGATACCACATTCTCGAAAATTTCGAAG
ATTGATATTGATTATCTAAGACGTGCGCCTCGTGTAGATATCAAGGACGAGACCAAGTTGAACGCTGACGAGAAGGCTTCTGAGAAGTATTATG
AGGACACTGTTCCTGGCACTTCTAATTTCATCTCTGAGGTCTTCTTTCTCACATTGG

>Bcin01g07220(MLST1), partial sequence [organism=Botrytis cinerea, strain N11_S_E10]
GGTGTGAAGTATCTGGCGGATAGAGAACCAATGAGCAGAGCAGCTATCGTAGATTTTCCGGAGCCGGAGGGTCCGACAAGTGCAGTTGTTGAGT
TAGGAATCAGAGAAAGAGAAAAGGATGATATCGTTCGTTTTTCAGGACGAGTAGGGTATGTGAATGAAAGACGGTTGAATTTGATTGGAAAGAT
TGTCGAAAGCCGTTCGGTTCCTTTATTTTCGTGGGAGGAAGATGAATCGAGATTGGCTAATGCAAGCATATGCGTAGCTGTAACGCGAGAAGAG
TTGATTTGTGGTATCATAGCCAGCATATTCGTACTATTAGAAATACCAAATAATAGAAGATTGACAGTCTGTAGTGCAGTCGCAATACTGATCT
CTCTCTTGGTAATGAGAACCGTGGCATAATAAAAGATAGTTGCAGTGATGAAGAATGACATCGCATCTGTCAATCCCCACAGCAATCCCGAGTA
GTTTGCTCGTATTAGTCCAACTTTATAGAGTTCTTCTGTAGCTTTGGTGTGTTTTGTCTCAAAGTAAGTTTCCAGAGTAAAAGCCCGAACCACT
TTGATGTTGGAGAAAGTCTCTGAAAATATGCCAGTGGTCATTTCAGATGCGTAGTTGCACTTGTTTTCCCATTTTCCACTCACGCGATTGAACG
TTTTGGTGACAGCGTATATAACTGGCCCAGTTGCCATTGAGACTAATGTCAGTTTCCATGTATTCACGAAAGCCCAAATGATTGATATCAATAG
CATAAAAAATGCTGTGAATACAATACCAGCAAAGCGGCCAACGAGATTACGCATTTCCTCAGAGTTCCTATCCAAAACTTCGT

>Bcin05g07690(MLST2), partial sequence [organism=Botrytis cinerea, strain N11_S_E10]
AGGATGCCTTCTCTCTACGACAGCAAGCCTTCGATGATATGTTGTGGGTAGTTCTTGGCTGGCTGGATACTGTCAAATTCATTGATTTACATTC
TGAATTGCACTATTCAAACGACTCTCAGCCAGAATGGTACGGACAACAATATAAACCTGCATTTGCACATCGAGCGCGACTATTTTGGGAATTG
GCTTCACAAGGATGGGATACTACTCTCTGTGGTGGTGGGATGATATGGTCACCATACCTTACTCCATACAAGAATGCAATTACCAATGAACTCT
ATATCGCAGCTTCGATATCGATGTACCTCTATTTCCCCGGAGATGACAATCAATCCCCATTTATGCTTTCCAACCCTTCATATCCACCTCACGA
TCCGAAATATCTACAGGCAGCTGTTGATGCTTACAAATGGCTGAATGGTTCCAACATGACGGATTTACAAGGATTATATGTCGACGGGTATCAT
ATCTCGAATCTTTCTGGCGGTGAAAACACCCATTGCGATTCTAGAAATGAGATGGTATATACCTACAATCAAGGTGTTTTGCTTACTGGACAAC
GTGGTTTGTATGACGCAACCGCCGCACGATCATACCTTGTGGATGGCCACAAACTCATCGCGAATGTTATTAATGCCACAGGCTATGACCTGAA
ACACAATGTTGCCATCTCACCGCCACCCAAAGATGGTTCCGCATTGGCAAAGTGGTTTGGCCTGGGTAGGAATGGAATACTGGAAGAAGGATGC
GATTCAAGTGCTTCGTGTTCTCAAAATGGACAAACTTTCAAAGGCATATTCTTTCACCACTTGATTGCGTTCTGTAGTGATTTGCCAGGGGAGC
CTATTGCAGGGACGAAGGAA

>Bcin06g01710(MLST3), partial sequence [organism=Botrytis cinerea, strain N11_S_E10]
AGCTTCGGAAACGTCTTCCGAGAGGAATTTGCCAGTCCCGATAGTCGGTTACGAGTCCACTCTCTGAAAACTCGTTGCGAAGATAACTTGGTGT
TATGGAGAGTGCATCGATCAGATCTTTGCGTTTCTTGACATATAGGCAAGAAGCGTCGAAATTTGTCAGAAGCCATTTGTGCATGTTCATATCA
AAGGAATGGAAATGCTGGAAGGACGATGTTAGATGATGGTATTCAGGGCAAACCAAAGCTGCACCTGCATAAGCAGCATCGACGTGAACCCAGA
TCTCGCCTGCAACATCTGGAGGTGCATATTTTGAAAGTACTGTTGCAATAGATGCGAAGTCGTCAACTGCGCATGTAGATGTTGTTCCCAACGT
CGAAGTTAGATAGAAGGGTTCCAATCCTTGAGATTTGCATTCTTTCAATACCTTCTCTAAATCATCACCCGTCATGGCGAAATCATTGGATGCG
AGTACTGGAATCGATCGGAATCTAACGCCAGCTATCTGCGCTGCTTTCTGCGTGGAGCTGTGTGCCATTTCGCTTCCTAGTGCAACTAGCTTAC
TCCTCTTATATGCAATCGCATCCTCGAGTTCAATTCCCGACAGACCTTCAGTGGTTTCACGAAGATACTTATCGCGGGCAGCAACCATAACGGT
AACGATAGCTTCCGAGGCTGATCCTTGGATAACACCACCACCATGAGTCGACGACAAATAACAGTCTGGGAGATTGAGAAGCTTGGCCAACCAA
TCCATTACAACCGTCTCCAATTCTGTCACAGCAGGGGAACAGATCCAATTGAAAGCAGGAGCTGTGAAAGCTGCTGAGTATAATTCTCCCAGCA

TTCCAGGGTAGGTAGATGATGCAGGGAAGAACGCCATGAAATTAGGTGATTGCCTTG

>Bcin09g03030(MLST4), partial sequence [organism=Botrytis cinerea, strain N11_S_E10]
GCAGAGAGAGTCTTGGAGGAGGTCGCCAAGAGTTGGAAGAATAGGAGTGGCGGTGTCATTGTCGAGGGCGAGGGAACGGAATTGAAGGAGATTC
TGAAAGCTTTGGAAGGGAATATGAGTGGTGGAAGGATAGTCATAGGAAGAGAGCTAAGCCGGCAGAATAGTTTAGTACTGGGATCATCACAATA
TGGAGAGGTCAATCATACAAGACTTGGGCTACGGCCAGGGAATATACCCAGAGAGGATAGTCAGTCAAGTTTGGGAATGTCAACGTTGGAGGTC
AATGACGAGGAAGATGAGGATGGCCTGATGGATCCAAGAAGGTGGTTAAAAGTCGTTGATGCATTTGAGCAACCTCGACTGGTGTACAATGTTG
CTAAAAAGCACTTTGATAGGTATGTTTCAATGATAAAATTTTATCTGAATCGACTAACTCAGTACAGAGATACCTCCAAACCTTCATTGTTCCC
ACCTGCGTCTCATAAAACACTCCTCTTCCAAAACCGCTATAATGTTATCCATCAACGTCTCCTTCGCAATGAATCTTTTCAAACGCCCGCTTTT
CAAGGTGGCAAATCTTCCCTTCAACGCAGCACGTCCGCCATTACCACCCAACAACAATCATACAAATTAACGCCGATAGCTAATCTTCTCGGTC
GCAATCGCAGCTCTCATATGCTTCTCGGTCTCCTCAGTATTTCACCCACTGGTACCCTCGCCATCAATGACCTG

>Bcin11g01310(MLST5), partial sequence [organism=Botrytis cinerea, strain N11_S_E10]
TCATGTGGAACCGGCGTAGAATGCGCAACGCTTGAAGTTCCGCTCGAATATGGCGATGCAACGTCAACGGCAAAAGCCAGTGTTGCGCTTGCTC
GTTATCCTGCCACTGTTGCCGCGAGCAAGAAGCTCGGGTCTCTCTTGATAAATCCCGGTGGACCCGGTGCCTCTGGTGTTGGCTTTGTGCAGTC
TGGAGCCGGTGCCGCCGTCTCGACACTGAGTGGTGGATTATACGATATCATCGGATGGGATCCACGTGGAACCGGTGCTTCGGCTCCTATTTTG
GAATGTTTTGCAAATGCCAGTGCGGAGTATGATTTTAACAACGCGTTTCCATCTGCTCCGAATCTCTGGCTCGGACAATTTGCGAATGCCAGCG
CAAATTCTGCTGTTAGCTCTGCTATCACATCCTTTGACACTTCTGTCGCTGCTCTTGCAAAAGCTTGCGTGGCTCAGAAATCTCCCGCTCTTTA
CACCTCAACAGCAGCATATGTTGCTCGAGACATGGCAGCGATAGTCGATGCATTGGATGGGACCTCTGCAAAACTTAACTACTGGGGTTTCTCA
TATGGAACTATCTTCCTAGCTGAGTTTATCCAAACTTTCCCAGGCCGCGTGGGAAGAGTTCTTGCCGATGGTGTTTTCGACGCAAAGGCAAATG
CACTCACATACGTTAGCCAACTTCCCAACGATCAACTCAGTGTTCGTGCTTCGTTGAACGATTTTGCAGCTTTCTGCACCACCGCCGGTAGTAA
AGGTTGCTCTTTTGCCACCGCCCCTACTGGAACCTCAGGTACTGTTGCTACCAGACTGGACAACATAATGAAGGATATG

>Bcin15g03910(MLST6), partial sequence [organism=Botrytis cinerea, strain N11_S_E10]
TCTTTGAAAGCCTTCTCTAGCATCTCCTCTCCTAACGTCATATGTCCCCCTGGAATGGTTTCGAAAACTAATTTTCCGGCCTCATCCCAATGTC
TTTAACCCTAGCCAATCTTCTTTATAAATGGCTCTTTCTTTCAGTGGTGTAACTTCCGTGCCATTGACTTCAGCCCACCATCCACTTTCCTTAG
GGATGACAGTTGTATCGTCTTCAAAGACATACATTACGAATCGTTCCAATTTTTCCATGTTGGATTTATATGTTTGGTTCTTGAGAACGCGCTC
ATTATTGATGTCGGCAAGGAAATTGGAATATTCAAGGTAAGAGTCTAGGTTTTCCGGATCTCTGAAGTATTGAGCAGGTACGAGACGAGATTGG
ACAAAGGTTGACCAGGTGTTGGATCGCAAAAGGGTTTGAGCACCGCGACAGAGGAAATCGGCAGGACCACAGGCTTGGAAGGCAGAAATGCCGT
TGTGTTGGGAACCGAAGGTCAGGAGAGAGCGGATGGGTGGAGCATTGCAGCGGGATATGTAACCGCGCAAGAATTGGCCGCCTTGGGAGAATCC
AATTGCGTCGACGGCGGGCGCGGTAGAGAGAATAGGATGGGAGGCGAGGTCTTCGCAGACCTTTTCGATTTGAACTTGGTAAGAGAGTTAGTAT
GGTATTTAAAGGAAAAATAGGGGATGTACTCACGAGTGAGATTTCCAAAGAAGGTAGCTGTCCTATCTGCAGATGCATCCTCATCTACATGAAT
ATTATAGACAAAAGTCCCAGGATGAATAGCTTCAGCTAGTTTTCCAACTTGCGCAAGACCATCCGCTTTGTAATTATCGCCGAGTCCATGCCAG
ATAATCAAGGGAAGTGGAGTATCAT

>Bcin16g03460(MLST7), partial sequence [organism=Botrytis cinerea, strain N11_S_E10]
TCTTCATGAACCTCTTAATTTGAACTCTTCATTAATTGCAAGTGATGAACCCCTATTTCTGCCCAGAGGATAGTTACAAGACGTATATCATTAG
TCGAGAACCACTCATGATATACATTGACGGATTTTTGAAAGCGAATGAAAGTAAACACTTGGTTGATGTTAGGTGTGTTATTTATTCTGATGAA
ATGAACAAGAGAGACTGATGAGATAGTGAACCGCTTTATGAACCGTCTACTGTTTCTCACGGACAGGAAGTTACCATTGATCCTTCGGTTCGAA
ATTCTGAAGTGGCGGTTTTAGAGAGGGATGAGGTGGTCAGGTGTATTGAGCATAGAGCGAGGGCATTTCAGGGGTGGAGGGGCGAGATGGGGAT
TGAGAAGTTGAGGACGCAGAGGTATGGGGTTGGAGGACATTATGGGATGCATTTGTAAGTTTTGGGGGATTGACGAAGGCCTCTGTTTATTTTC
TATCAGTACGGATCTTGAAAGAAAGATAGCATGAACACGAGGGCTAATAACTAGGAAATAGCGATTGGAGCGGAGGTAAACGTGGCATAGACCG
ATTTAGTACTTTCATGGTCTATGTCGACGTATCCTCTGATATCGAAGGTGGAGGAACGGAATTCCCACGTATTGTGGGACCAAAAGGAGGAAGG
TGGGAGGACTTCCTGGAAACTACGGAAGCATTGGATCCAAGAACTGGAGAAAATGTAACAGTAGAAGGGGTGACATTCAAACCAATCAAGGGAA
ATGCCGTATTCT

>Bcin12g03020(MLST8), partial sequence [organism=Botrytis cinerea, strain N11_S_E10]
ACTGCGCAGCACCGAGTCACCAAAAGCACGAATAAACCATCGGCAAGGGAAATATTCTCTGAACCACAACATGACACGAGCGCGGAAGAGTATA
TCGGGCGAGAAGCTTCATCAAGAGCACCAAAGCGACAACGAGTCGATGATAATTATAACTCTTACGGTGGAAGAAATGAGAATACAGCAGCTTA
TGCTTCCGGGAAACTTCCATCTGGAAGTATAAATGTTGGTGGAGGTAGGAAGACCACATTTCAAGAAGAACCTCGAACGGCATTTGTCGCTGGC
AAGTTGCCCCCTGGAAGTATCAACATTGGTGGAAAGAAGGCTACCCAAATCGAGGACGAGGGTAGAGCAGCTTATGCTTCCGGAAAATTGCCCC
CAGGAAGTATAAACTCTGGCGCAAAGAAGGCGATTTCATTCCAAGATGAAACGAGACCGGCTTATGTCTCTGGAAAGCTTCCACATGGTAGTAT
CGACGGTATGCGAAACCGTGAAATGGCTGCCGTCCACCGCGAAATTGCTGAGGGTGGGAGGAAACCAGGCCAGGTTGTCTCTTCTCTATTCACA
TTCAATCCTACTTCAAAGAAAACTTTCGATGAACCCGAAGAACAAGCGGAACCGGCAAAACCATCCAATGCACCTTTGACAGAGGAAATGGCGA
CTTTCACCAATCTAGGGTTATCGAGAAGGCTTGCAGCCCATCTATCGACTAAACTCGATATGAAAGCCCCGACCGCCATTCAAAAAGCATCTGT
GCAGCAGTTGGTATCGGACGATAGCGATGCTTTCATACAAGCAGAGACTGGATCTGGAAAAACTTTGGCATATCTACTACCTATAGTCGAGCGA
ATATTAGCATTGAGTGAGAATGGCGTACA

>Bcin02g07770(MLST9), partial sequence [organism=Botrytis cinerea, strain N11_S_E10]
CGTCACGATTGGGAAGGTGTAATTGTCTGGCTCTCAAGCGCCACCGCCACAACTGCCGACAACATCTTAGCCGTTTGTCCTTCCGCCCACGGAG
GCTGGGATTGTTCCACCGATGGCTATTCCCTTTCTGGTACCAGCCCTCTTATCAAGTACGAAAGTATCTGGCCCGTCGATCATTCAATGGGTCT
TACTAGTACTGTTGGTGGAAAACAACCTATGATTGCTTGGGAGTCTTTACCAACTGCTGCTCAAACTGCTCTTGAGAACACCGATTTCGGTGCT
GCGAATGTTCCATTCATTCCGGCTGTTTTCACAGATAATCTTGCGAAAGCTACTTTCTAG

>Bcin04g02090(MLST10), partial sequence [organism=Botrytis cinerea, strain N11_S_E10]
AACTGTCGAATCTGACTATGAATGATGTTTACAAGCCCTACATCCATGTAAGAAATGTAGAATAGAGAGATCAGTAACTGGAACTAATATCGTT
TGCAGGCTTTCAAGTTACTTACGCAGTTCAACCCAATCACTACAGCTATTGCCGAATCCCCACTATTTCAAATGGCTGTCTCAGCAAATACCAT
CGAAAAGTACACACTGCTAGGCCCTTTCTTCAGAATATCTCCTCTGCAACAGGAAGTTACCAGGGAATACTTCAGTGCGCCAAAGACGATAGAT
AGGCGACACATTGCCACATCTCAAGATGCCTTACGATTGACCTTACAAACCCATCAAAAAGATTTACTTGATATCATCAACCACTTTGTTCGAG
CAAGTCCAATCGCCAAAAGCAAAACCCTGGATTGGTTCGCCTACATTGTGAATCAAAATCACAAGCGTCGAGCACTTCAGGTAGACCCGAAAGA
AGTGTCTTCTGATGGTTTTATGCACAATGTCACTGTCGTTCTAGATGGTCTTTGTGAGCCATTCATGGATACCACATTCTCAAAAATTTCGAAG
ATTGATATTGATTATCTAAGACGTGCGCCTCGTGTAGATATCAAGGACGAGACCAAGTTGAACGCTGATGAGAAGGCTTCTGAGAAGTATTATG
AGGACACTGTTCCTGGCACTTCTAATTTCATCTCTGAGGTCTTCTTTCTGACATTGG

>Bcin01g07220(MLST1), partial sequence [organism=Botrytis cinerea, strain N11_S_E15]
GGTGTGAAGTATCTGGCGGATAGAGACCAATGAGCAGAGCAGCTATTGTAGATTTTCCGGAGCCGGAGGGTCCGACAAGTGCAGTTGTTGAGTT
AGGAATCAGGGAAAGAGAAAAGGATGATATCGTTCGTTTTTCAGGACGAGTAGGGTATGTGAATGAAAGACGGTTGAATTTGATTGGAAAGATT
GTCGAAAGCCGTTCGGTTCCTTTATTTTCGTGGGAGGAAGATGAATCGAGATTGGCTAATGCAAGCATATGCGTAGCTGTAACGCGAGAAGAGT
TGATTTGTGGTATCATGGCCAGCATATTCGTACTATTAGAAATACCAAATAATAGAAGATTGACAGTCTGTAGTGCAGCCGCGATACTGATCTC
TCTCTTGGTAATGAGAACCGTGGCATAATAAAAGATAGTTGCAGTGATGAAGAATGACATTGCATCTGTCAATCCCCACAGCAATCCCGAGTAG
TTTGCTCGTATTAGTCCAACTTTATAGAGTTCTTCTGTAGCTTTGGTGTGTTTTGTCTTAAAGTAAGTTTCCAGAGTAAAAGCCCGAACCACTT
TGATGTTGGAGAAAGTCTCTGAAAATATGCCAGTGGTCATTTCAGATGCGTAGTTGCACTTGTTTTCCCATTTTCCACTCACGCGATTGAACGT
TTTGGTGACAGCGTATATAACTGGCCCAGTTGCCATTGAGACTAATGTCAGTTTCCATGTATTCACGAAAGCCCAAATGATTGATATCAATAGC
ATAAAAAATGCTGTGAATACAATACCAGCAAAGCGGCCAACGAGATTACGCATTTCCTCAGAGTTCCTATCCAAAACTTCG

>Bcin05g07690(MLST2), partial sequence [organism=Botrytis cinerea, strain N11_S_E15]
AGGATGCCTTCTCTCTACGACAGCAAGCCTTCGATGATATGTTGTGGGTAGTTCTTGGCTGGCTGGATACTGTCAAATTCATTGATTTACATTC
TGAATTGCACTATTCAAACGACTCTCAGCCAGAATGGTACGGACAACAATATAAACCTGCATTTGCACATCGAGCGCGACTATTTTGGGAATTG
GCTTCACAAGGATGGGATACTACTCTCTGTGGTGGTGGGATGATATGGTCACCATACCTTACTCCATACAAGAACGCAATTACCAATGAACTCT
ATATCGCAGCTTCGATATCGATGTACCTATATTTCCCCGGAGATGACAATCAATCCCCATTTATGCTTTCCAACCCTTCATATCCACCTCACGA
TCCGAAATATCTACAGGCAGCTGTTGATGCTTACAAATGGCTGAATGGTTCCAACATGACGGATTTACAAGGATTATATGTCGACGGGTACCAT
ATCTCGAATCTTTCTGGCGGTGAAAACACCCATTGCGATTCTAGAAATGAGATGGTATATACCTACAATCAAGGTGTTTTGCTTACTGGACAAC
GTGGTTTGTATGACGCAACCGCCGCACGATCATACCTTGTAGATGGCCACAAACTCATCGCGAATGTTATTAATGCCACAGGCTATGACCTGAA
ACACAATGTTGTCATCTCACCGCCACCCAAAGATGGTTCCGCATTGGCAAAGTGGTTTGGCCTGGGTAGGAATGGAATACTGGAAGAAGGATGC
GATTCAAGTGCTTCGTGTTCTCAAAATGGACAAACTTTCAAAGGCATATTCTTTCATCACTTGATTGCGTTCTGTAGTGATTTGCCAGGGGAGC
CTATTGCAGGGACGAAGGAAT

>Bcin06g01710(MLST3), partial sequence [organism=Botrytis cinerea, strain N11_S_E15]
AGCTTCGGAAGCGTCTTCCGAGAGGAATTTGCCAGTCCCGATAGTCGGTTACGAGTCCACTCTCTGAAAACTCGTTGCGAAGATAACTTGGTGT
TATGGACAGTGCATCGATCAGATCTTTGCGTTTCTTGACATATAGACAAGAAGCGTCGAAATTTGTCAGAAGCCATTTGTGCATGTTCATATCG
AAGGAATGGAAATGCTGGAAAGACGATGTTAGATGATGGTATTCAGGGCAAACCAAAGCTGCACCTGCATAAGCAGCATCGACGTGAACCCAGA
TCTCGCCTGCAACATCTGGAGGTGCATATTTTGAAAGTACTGTTGCAATAGATGCGAAGTCGTCAACTGCGCATGTAGATGTTGTTCCCAAAGT
CGAAGTTAGATAGAAGGGTTCCAATCCTTGAGATTTGCATTCTTTCAATACCTTCTCTAAATCATCACCCGTCATGGCGAAATCATTGGATGCG
AGTACTGGAATCGATCGGAATCTAACGCCAGCTATTTGCGCTGCTTTCTGCGTGGAGCTGTGTGCCATTTCGCTTCCTAGTGCAACTAGCTTAC
TCCTCTTATATGCAATCGCATCCTCGAGTTCAATGCCCGACAGACCTTCAGTAGTTTCACGAAGATATTTATCGCGGGCAGCAACCATAACGGT
AACGATAGCTTCCGAGGCTGATCCTTGGATGACACCACCACCATGAGTCGAAGACAAATAACAGTCTGGGAGATTGAGAAGCTTGGCCAGCCAA
TCCATTACAACCGTCTCCAATTCTGTCACAGCAGGGGAACAGATCCAATTGAAAGCAGGTGCTGTGAAAGCTGCTGAGTATAATTCTCCCAGCA
TTCCAGGGTAGGTAGATGATGCAGGGAAGAACGCCATGAAATTAGGTGATTGCCTTG

>Bcin09g03030(MLST4), partial sequence [organism=Botrytis cinerea, strain N11_S_E15]
GCAGAGAGAGTCTTGGAGGAGGTCGCCAAGAGTTGGAAGAATAGGAGTGGCGGTGTCATTGTCGAGGGCGAGGGAACGGAATTGAAGGAGATTC
TGAAAGCTTTGGAAGGGAATATGAGTGGTGGAAGGATAGTCATAGGAAGAGAGCTAAGCCGGCAGAATAGTTTAGTACTGGGATCATCACAATA
TGGAGAGGTCAATCATACAAGACTTGGGCTACGGCCAGGGAATATACCCAGAGAGGATAGTCAGTCAAGTTTGGGAATGTCAACGTTGGAGGTC
AATGACGAGGAAGATGAGGATGGCCTGATGGATCCAAGAAGGTGGTTAAAAGTCATTGATGCATTTGAGCAACCTCGACTGGTGTACAATGTTG

CTAAAAAGCACTTTGATAGGTATGTTTCAATGATAAAATTTCATCTGAATCGACTAACTCAGTACAGAGATACCTCCAAACCTTCATTGTTCCC
ACCTGCGTCTCATAAAACACTCCTCTTCCAAAACCGCTATAATGTTATCCATCAACGTCTCCTTCGCAATGAATCTTTTCAAACGCCCGCTTTT
CAAGGTGGCAAATCTTCCCTTCAACGCAGCACGTCCGCCATTACCACCCAACAACAATCATACAAATTAACGCCGATAGCTAATCTTCTCGGTC
GCAATCGCAGCTCTCATATGCTTCTCGGTCTCCTCAGTATTTCACCCACTGGTACCCTCGCCATCAATGACCTG

>Bcin11g01310(MLST5), partial sequence [organism=Botrytis cinerea, strain N11_S_E15]
TCATGTGGACCGGCGTAGAATGCGCAACGCTTGAAGTTCCGCTCGAATATGGCGATGCAACGTCAACGGCAAAAGCCAGTGTTGCGCTTGCTCG
TTATCCTGCCACTGTTGCCGCGAGCAAGAAGCTCGGGTCTCTCTTGATAAATCCCGGTGGACCCGGTGCCTCTGGTGTTGGCTTTGTGCAGTCT
GGAGCCGGTGCCGCCGTCTCGACACTGAGTGGTGGATTGTACGATATCATCGGATGGGATCCACGTGGAACCGGTGCTTCGGCTCCTATTTTGG
AATGTTTTGCAAATGCCAGTGCGGAGTATGATTTTAACAACGCGTTTCCATCTGCTCCGAATCTCTGGCTCGGACAATTTGCGAATGCCAGCGC
AAATTCTGCTGTTAGCTCTGCTATCACATCCTTTGACACTTCTGTCGCTGCTCTTGCAAAAGCTTGCGTGGCTCAGAAATCTCCCGCTCTTTAC
ACCTCAACAGCAGCATATGTTGCTCGAGACATGGCAGCGATAGTCGATGCATTGGATGGGACCTCTGCAAAACTTAACTACTGGGGTTTCTCAT
ATGGAACTATTTTCCTAGCTGAGTTTATCCAAACTTTCCCAGGCCGCGTGGGAAGAGTTCTTGCCGATGGTGTTTTCGACGCAAAGGCAAATGC
ACTCACATACGTTAGCCAACTTCCCAACGATCAACTCAGTGTTCGTGCTTCGTTGAACGATTTTGCAGCTTTCTGCACCACCGCCGGTAGTAAA
GGTTGCTCTTTTGCCACCGCCCCTACTGGAACCACAGGTACTGTTGCTACCAGACTGGACAACATAATGAAGGATATG

>Bcin15g03910(MLST6), partial sequence [organism=Botrytis cinerea, strain N11_S_E15]
TCTTTGAAAGCCTTCTCTAGCATCTCCTCTCCTAACGTCATATGTCCCCCTGGAATGGTTTCGAAAACTAATTTTCCGGCCTCATCCCAATGTC
TTTAAACCTAGCCAATCTTCTTTATAAATGGCTCTTTCTTTCAGTGGTGTAACTTCCGTGCCGTTGACTTCAGCCCACCATCCACTTTCCTTAG
GGATGACAGTTGTATCGTCTTCAAAGACATACATTACGAATCGTTCCAATTTTTCCATGTTGGATTTATATGTTTGGTTCTTGAGAACGCGCTC
ATTATTGATGTCGGCAAGGAAATTGGAATATTCAAGGTAAGAGTCTAGGTTTTCCGGATCTCTGAAGTATTGAGCGGGTACGAGACGAGATTGG
ACAAAGGTTGACCAGGTGTTGGATCGCAAAAGGGTTTGAGCACCGCGACAGAGGAAATCGGCAGGACCACAGGCTTGGAAGGCAGAAATGCCGT
TGTGTTGGGAACCGAAGGTCAGGAGAGAGCGGATGGGTGGAGCATTGCAGCGGGATATGTAACCACGCAAGAATTGGCCGCCTTGGGAGAATCC
AATTGCGTCGACGGCGGGCGCGGTAGAGAGAATAGGATGGGAGGCGAGGTCTTCGCAGACCTTTTCGATTTGAACTTGGTAAGAGAGTTAGTAT
GATATTTAAAGGAAAAATAGGGGATGTACTCACGAGTGAGATTTCCAAAGAAGGTAGCTGTCCTATCTGCAGATGCATCCTCATCTACATGAAT
ATTGTAGACAAAAGTCCCAGGATGAATAGCTTCAGCTAGTTTTCCAACTTGCGCAAGACCATCCGCTTTGTAATTATCGCCGAGTCCATGCCAG
ATAATCAAGGGAAGTGGAGTATCAT

>Bcin16g03460(MLST7), partial sequence [organism=Botrytis cinerea, strain N11_S_E15]
TCTTCATGAACCTCTTGATTTGAACTCTTCATTAATTGCAAGTGATGAACCCCTATTTCTGCCCAGAGGATAGTTACAAGACGTATATCATTAG
TCGAGAACCACTCATGATATATATTGACGGATTTTTGAAAGCGAATGAAAGTAAACATTTGGTTGATGTTAGGTGTGTTATTTATTCTGATGAA
ATGAACAAGAGAGACTGATGAGATAGTGAGCCGCTTTATGAACCGTCAACTGTTTCTCACGGACAGGAAGTTACCATTGATCCTTCAGTTCGGA
ATTCTGAAGTGGCGGTTTTAGAGAGGGATGAGGTGGTCAGGTGTATTGAGCATAGAGCGAGGGCATTTCAGGGGTGGAGGGGCGAGATGGGGAT
TGAGAAGTTGAGGACGCAGAGGTATGGGGTTGGAGGACATTATGGGATGCATTTGTAAGTTTTGGGGGATTGACGAAGGCTTCTGTCTATTTTC
TATCAGCACGGATCTTGAAAGAAAGATAGCATGAACACGCGGGCTAATAACTAGGAAATAGCGATTGGAGCGGAGGTAAACGTGGCATAGACCG
ATTTAGTACTTTCATGGTCTATGTCGACGTATCCTCTGATATCGAAGGTGGAGGAACGGAATTCCCACGTATCGTGGGACCAAAAGGAGGAAGG
TGGGAGGACTTCCTGGAAACTACGGAAGCATTGGATCCAAGAACTGGAGAAAATGTAACAGTAGAAGGGGTGACATTCAAACCAATCAAGGGAA
ATGCCGTATTCT

>Bcin12g03020(MLST8), partial sequence [organism=Botrytis cinerea, strain N11_S_E15]
ACTGCGCAGCACCGAGTCACCAAAAGCACGAATAAACCATCGGCAAGGGAAATATTCTCTGAACCACAACATGACACGAGCGCGGAAGAGTATA
TCGGGCGAGAAGCTTCATCAAGAGCACCAAAGCGACAACGAGTCGATGATAATTATAACTCTTACGGTGGAAGAAATGAAAATACAGCAGCTTA
TGCTTCCGGGAAACTTCCATCTGGAAGTATAAATGTTGGTGGAGGTAGGAAGACCACTTTTCAAGAAGAACCACGAACGGCATTTGTCGCTGGC
AAGTTGCCCCCCTGGAAGTATCAACATTGGTGGAAAGAAGGCTACCCAAATCGAGGACGAGGGTAGAGCAGCTTATGCTTCCGGAAAATTGCCTC
CAGGAAGTATAAACTCTGGCGCAAAGAAGGCGATTTCATTCCAAGATGAAACGAGACCGGCTTATGTCTCCGGAAAGCTTCCACATGGTAGTAT
CGACGGTATGCGAAACCGTGAAATGGCTGCCGTCCACCGCGAAATTGCTGAGGGTGGGAGGAAACCAGGCCAGGTTGTCTCTTCTCTATTCACA
TTCAATCCTACTTCAAAGAAAACTTTTGATGAACCCGAAGAACAAGCGGAACCGGCAAAACCATCCAATGCGCCTTTGACAGAGGAAATGGCGA
CTTTCACCAATCTAGGGTTATCGAGAAGGCTTGCAGCTCATCTATCGACTAAACTCGATATGAAAGCTCCGACTGCCATTCAAAAAGCATCTGT
ACAGCAGTTGGTATCGGACGATAGCGATGCTTTCATACAAGCAGAGACTGGATCTGGAAAAACTTTGGCATATCTACTACCTATAGTCGAGCGA
ATATTAGCATTGAGTGAGAATGGCGT

>Bcin04g02090(MLST10), partial sequence [organism=Botrytis cinerea, strain N11_S_E15]
AACTGTCGAATATGACTATGAATGATGTTTACAAGCCCTACATCCATGTAAGAAATGTAGAATAGAGAGATCAGTAACTGGAACTAATATCGTT
TGTAGGCTTTCAAGTTACTTACGCAGTTCAACCCAATCACTACAGCTATTGCCGAATCCCCACTGTTTCAAATGGCCGTCTCAGCAAATACCAT
CGAAAAGTACACACTGCTAGGCCCTTTCTTCAGAATATCTCCTCTGCAACAGGAAGTTACCAGGGAATACTTCAGTGCGCCAAAGACGATAGAT
AGGCGACACATTGCCACATCTCAAGATGCGTTACGATTGACCTTACAAACCCATCAAAAAGATTTACTTGATATCATCAACCACTTTGTTCGAG
CAAGTCCAATCGCAAAAAGCAAAACCCTGGATTGGTTCGCCTACATTGTGAATCAAAATCACAAGCGTCGAGCACTTCAGGTAGACCCGAAAGA

AGTGTCTTCTGATGGCTTTATGCACAATGTCACTGTCGTTCTAGATGGTCTTTGTGAGCCATTCATGGATACCACATTCTCGAAAATTTCGAAG
ATTGATATTGATTATCTAAGACGTGCGCCTCGTGTAGATATCAAGGACGAGACCAAGTTGAACGCTGACGAGAAGGCTTCTGAGAAGTATTATG
AGGACACTGTTCCTGGCACTTCTAATTTCATCTCTGAGGTCTTCTTTCTCACATTGG

>Bcin01g07220(MLST1), partial sequence [organism=Botrytis cinerea, strain SA12_Ro]
GGTGTTGAAGTATCTGGCGGATAGAGACCAACGAGCAGAGCAGCTATTGTAGATTTTCCGGAGCCGGAGGGTCCGACAAGTGCAGTTGTTGAGT
TAGGAATCAGGGAAAGAGAAAAGGATGATATCGTTCGTTTTTCAGGACGAGTAGGATATGTGAATGAGAGACTGTTGAATTTGATTGGAAAGAT
TGTCGAAAGCCGTTCGGCTCCTTTATTTTCGTGGGAGGAAGATGAATCGAGATTGGCTAATGCAAGCATATGCGTAGCTGTAACGCGAGAAGAG
TTGATTTGTGGTATCATGGCCAGCATATTCGTACTATTAGAAATACCAAATAATAGAAGATTGACAGTCTGCAGTGCAGTCGCGATACTGATCT
CTCTCTTGGTAATGAGAACCGTGGCATAATAAAAGATAGTTGCAGTGATGAAGAATGACATCGCATCTGTCAATCCCCACAGCAATCCCGAGTA
GTTTGCTCGTATTAGTCCAATTTTATAGAGTTCTTCTGTAGCTTTGGTGTGTTTTGTCTCAAAGTAAGTTTCCAGAGTAAAAGCCCGAACCACT
TTGATGTTGGAGAAAGTCTCTGAAAATATGCCAGTGGTCATTTCAGATGCGTAGTTGCACTTGTTTTCCCATTTTCCACTCACGCGATTGAACG
TTTTGGTGACAGCGTATATAACTGGCCCAGTCGCCATTGAGACTAATGTCAGTTTCCATGTATTCACGAAAGCCCAAATGATTGATATCAATAG
CATAAAAAATGCTGTGAATACAATACCAGCAAAGCGGCCAACGAGATTACGCATTTCCTCAGAGTTCCTATCCAAAACTTCGT

>Bcin05g07690(MLST2), partial sequence [organism=Botrytis cinerea, strain SA12_Ro]
AGGATGCCTTCTCTCTACGACAGCAAGCCTTCGATGATATGTTGTGGGTAGTTCTTGGCTGGCTGGATACTGTCAAATTCATTGATTTACATTC
TGAATTGCACTATTCAAACGACTCTCAGCCAGAATGGTACGGACAACAATATAAACCTGCATTTGCACATCGAGCGCGACTATTTTGGGAATTG
GCTTCACAAGGATGGGATACTACTCTCTGTGGTGGTGGGATGATATGGTCACCATACCTTACTCCATACAAGAACGCAATTACCAATGAACTCT
ATATCGCAGCTTCGATATCGATGTACCTATATTTCCCCGGAGATGACAATCAATCCCCATTTATGATTTCCAACCCTTCATATCCACCTCACGA
TCCGAAATATCTACAGGCAGCTGTTGATGCTTACAAATGGCTGAATGGTTCCAACATGACGGATTCACAAGGATTATATGTCGACGGGTACCAT
ATCTCGAATCTTTCTGGCGGTGAAAACACCCATTGCGATTCTAGAAATGAGATGGTATATACCTACAATCAAGGTGTTTTGCTTACTGGACAAC
GTGGTTTGTATGACGCAACCGCCGCACGATCATACCTTGTAGATGGCCACAAACTCATCGCGAATGTTATTAATGCCACAGGCTATGACCTGAA
ACACAATGTTGTCATCTCACCGCCACCCAAAGATGGTTCCGCATTGGCAAAGTGGTTTGGCCTGGGTAGGAATGGAATACTGGAAGAAGGATGC
GATTCAAGTGCTTCGTGTTCTCAAAATGGACAAACTTTCAAAGGCATATTCTTTCATCACTTGATTGCGTTCTGTAGTGATTTGCCAGGGGAGC
CTATTGCAGGGACGAAGGAA

>Bcin06g01710(MLST3), partial sequence [organism=Botrytis cinerea, strain SA12_Ro]
AGCTTCGGAAGCGTCTTCCGAGAGGAATTTGCCAGTCCCGATAGTCGGTTACGAGTCCACTCTCTGAAAACTCGTTGCGAAGATAACTTGGTGT
TATGGAGAGTGCATCGATCAGATCTTTGCGTTTCTTGACATATAGACAAGAAGCGTCGAAATTTGTCAGAAGCCATTTGTGCATGTTCATATCG
AAGGAATGGAAATGCTGGAAGGACGATGTTAGATGATGGTATTCAGGGCAAACCAAAGCTGCACCTGCATAAGCAGCATCGACGTGAACCCAGA
TCTCGCCTGCAACATCTGGAGGTGCATATTTTGAAAGTACTGTTGCAATAGATGCGAAGTCGTCAACTGCGCATGTAGATGTTGTTCCCAAAGT
CGAAGTTAGATAGAAGGGTTCCAATCCTTGAGATTTGCATTCTTCCAATACCTTCTCTAAATCATCACCCGTCATGGCGAAATCATTGGATGCG
AGTACTGGAATCGATCGGAATCTAACGCCAGCTATCTGCGCTGCTTTCTGCGTGGAGCTGTGTGCCATTTCGCTTCCTAGTGCAACTAGCTTAC
TCCTCTTATATGCAATCGCATCCTCGAGTTCAATGCCCGACAGACCTTCAGTAGTTTCACGAAGATATTTATCGCGGGCAGCAACCATAACGGT
AACGATAGCTTCCGAGGCTGATCCTTGGATAACACCACCACCATGAGTCGAAGACAAATAACAGTCTGGGAGATTGAGAAGCTTGGCCAGCCAA
TCCATTACAACCGTCTCCAATTCTGTCACGGCAGGGGAACAGATCCAATTGAAAGCAGGAGCTGTGAAAGCTGCTGAGTATAATTCTCCCAGCA
TTCCAGGGTAGGTAGATGATGCAGGGAAGAACGCCATGAAATTAGGTGATTGCCTTG

>Bcin09g03030(MLST4), partial sequence [organism=Botrytis cinerea, strain SA12_Ro]
GCAGAGAGAGTCTTGGAGGAGGTCGCCAAGAGTTGGAAGAATAGGAGTGGCGGTGTCATTGTCGAGGGCGAGGGAACGGAATTGAAGGAGATTC
TGAAAGCTTTGGAAGGGAATATGAGTGGTGGAAGGATAGTCATAGGAAGAGAGCTAAGCCGGCAGAATAGTTTAGTACTGGGATCATCACAATA
TGGAGAGGTCAATCATACAAGACTTGGGCTACGGCCAGGGAATATACCCAGAGAGGATAGTCAGTCAAGTTTGGGAATGTCAACGTTGGAGGTC
AATGACGAGGAGGATGAGGATGGCCTGATGGATCCAAGAAGGTGGTTAAAAGTCATTGATGCATTTGAGCAACCTCGACTGGTGTACAATGTTG
CTAAAAAGCACTTTGATAGGTATGTTTCAATGATAAAATTTTATCTGAATCTACTAACTCAGTACAGAGATACCTCCAAACCTTCATTGTTCCC
ACCTGCGTCTCATAAAACACTCCTCTTCCAAAACCGCTATAATGTTATCCATCAACGTCTCCTTCGCAATGAATCTTTTCAAACGCCCGCTTTT
CAAGGTGGCAAATCTTCCCTTCAACGCAGCACGTCCGCCATTACCACCCAACAACAATCATACAAATTAACGCCGATAGCTAATCTTCTCGGTC
GCAATCGCAGCTCTCATATGCTTCTCGGTCTCCTCAGTATTTCACCCACTGGTACCCTCGCCATCAATGACCTG

>Bcin11g01310(MLST5), partial sequence [organism=Botrytis cinerea, strain SA12_Ro]
TCATGTGGAACCGGCGTAGAATGCGCAACGCTTGAAGTTCCGCTCGAATATGGCGATGCAACGTCAACGGCAAAAGCCAGTGTTGCGCTTGCTC
GTTATCCTGCCACTGTTGCCGCGAGCAAGAAGCTCGGGTCTCTCTTGATAAACCCCGGTGGACCCGGTGCCTCTGGTGTTGGCTTTGTGCAGTC
TGGAGCCGGTGCCGCCGTCTCGACACTGAGTGGTGGATTGTACGATATCATCGGATGGGATCCACGTGGAACCGGTGCTTCGGCTCCTATTTTG
GAATGTTTTGCAAATGCCAGTGCGGAGTATGATTTTAACAACGCGTTTCCATCTGCTCCGAATCTCTGGCTCGGACAATTTGCGAATGCCAGCG
CAAATTCTGCTGTTAGCTCTGCTATCACATCCTTTGACACTTCTGTCGCTGCTCTTGCAAAAGCTTGCGTGGCTCAGAAATCTCCCGCTCTTTA
CACCTCAACAGCAGCATATGTTGCTCGAGACATGGCAGCGATAGTCGATGCATTGGATGGGACCTCTGCAAAACTTAACTACTGGGGTTTCTCA
TATGGAACTATCTTCCTAGCTGAGTTTATCCAAACTTTCCCAGGCCGCGTGGGAAGAGTTCTTGCCGATGGTGTTTTCGACGCAAAGGCAAATG

CACTCACATACGTTAGCCAACTTCCCAACGATCAACTCAGTGTTCGTGCTTCGTTGAACGATTTTGCAGCTTTCTGCACCACCGCCGGTAGTAA
AGGTTGCTCTTTTGCCACCGCCCCTACTGGAACCTCAGGTACTGTTGCTACCAGACTGGACAACATAATGAAGGATATG

>Bcin15g03910(MLST6), partial sequence [organism=Botrytis cinerea, strain SA12_Ro]
TCTTTGAAAGCCTTCTCTAGCATCTCCTCTCCTAACGTCATATGTCCCCCTGGAATGGTTTCGAAAACTAATTTTCCGGCCTCATCCAATGTCT
TTAAACCTAGCCAATCTTCTTTATAAATGGCTCTTTCTTTCAGTGGTGTAACTTCCGTGCCGTTGACTTCAGCCCACCATCCACTTTCCTTAGG
AATGACAGTTGTGTCGTCTTCAAAGACATACATTACGAATCGTTCCAATTTTTCCATGTTGGATTTATATGTTTGGTTCTTGAGAACGCGCTCA
TTATTGATGTCGGCAAGGAAATTGGAATATTCAAGGTAAGAGTCTAGGTTTTCCGGATCTCTGAAGTATTGAGCGGGTACGAGACGAGATTGGA
CAAAGGTTGACCAGGTGTTGGATCGCAAAAGGGTTTGAGCACCGCGACAGAGGAAATCGGCAGGACCACAGGCTTGGAAGGCAGAAATGCCGTT
GTGTTGGGAACCAAAGGTCAGGAGAGAGCGGATGGGTGGAGCATTGCAGCGGGATATGTAACCACGCAAGAATTGGCCGCCTTGGGAGAATCCA
ATTGCGTCGACGGCGGGCGCGGTAGAGAGAATAGGATGGGAGGCGAGGTCTTCGCAGACCTTTTCGATTTGAACTTGGTAAGAGAGTTAGTATG
ATATTTAAAGGAAAAATAGGGGATGTACTCACGAGTGAGATTTCCAAAGAAGGTAGCTGTCCTATCTGCAGATGCATCCTCATCTACATGAATA
TTGTAGACAAAAGTCCCAGGATGAATAGCTTCAGCTAGTTTTCCAACTTGCGCAAGACCATCCGCTTTGTAATTATCGCCGAGTCCATGCCAGA
TAATCAAGGGAAGTGGAGTATCAT

>Bcin16g03460(MLST7), partial sequence [organism=Botrytis cinerea, strain SA12_Ro]
TCTTCATGAACCTCTTGATTTGAACTCTTCATTAATTGCAAGTGATGAACCCCTATTTCTGCCCAGAGGATAGTTACAAGACGTATATCATTAG
TCGAGAACCACTCATGATATATATTGACGGGTTTTTGAAAGCGAATGAAAGTAAACATTTGGTTGATGTTAGGTGTGTTATTTATTCTGATGAA
ATGAACAAGAGAGACTGATGAGATAGTGAGCCGCTTTATGAACCGTCAACTGTTTCTCACGGACAGGAAGTTACCATTGATCCTTCAGTTCGGA
ATTCTGAAGTGGCGGTTTTAGAGAGGGATGAGGTGGTCAGGTGTATTGAGCATAGAGCGAGGGCATTTCAGGGGTGGAGGGGCGAGATGGGGAT
TGAGAAGTTGAGGACGCAGAGGTATGGGGTTGGAGGACATTATGGGATGCATTTGTAAGTTTTGGGGGATTGACGAAGGCTTCTGTCTATTTTC
TATCAGCACGGATCTTGAAAGAAAGATAGCATGAACACGCGGGCTAATAACTAGGAAATAGCGATTGGAGCGGAGGTAAACGTGGCATAGACCG
ATTTAGTACTTTCATGGTCTATGTCGACGTATCCTCTGATATCGAAGGTGGAGGAACGGAATTCCCACGTATTGTGGGACCAAAAGGAGGAAGG
TGGGAGGACTTCCTGGAAACTACGGAAGCATTGGATCCAAGAACTGGAGAAAATGTAACAGTAGAAGGGGTGACATTCAAACCAATCAAGGGAA
ATGCCGTATTCT

>Bcin04g02090(MLST10), partial sequence [organism=Botrytis cinerea, strain SA12_Ro]
AACTGTCAAATATGACTATGAATGATGTTTACAAGCCCTACATTCATGTAAGAAATGTAGAATAGAGAGATCAGTAACTGGAACTAATATCGTT
TGTAGGCTTTCAAGTTACTTACGCAGTTCAACCCAATCACTACAGCTATTGCCGAATCCCCACTGTTTCAAATGGCCGTCTCAGCAAATACCAT
CGAAAAGTACACACTGCTAGGCCCTTTCTTCAGAATATCTCCTCTGCAACAGGAAGTTACCAGGGAATACTTCAGTGCGCCAAAGACGATAGAT
AGGCGACACATTGCCACATCTCAAGATGCGTTACGATTGACCTTACAAACCCATCAAAAAGATTTACTTGATATCATCAACCACTTTGTTCGAG
CAAGTCCAATCGCAAAAAGCAAAACCCTGGATTGGTTCGCCTACATTGTGAATCAAAATCACAAGCGTCGAGCACTTCAGGTAGACCCGAAAGA
AGTGTCTTCTGATGGCTTTATGCACAATGTCACTGTCGTTCTAGATGGTCTTTGTGAGCCATTCATGGATACCACATTCTCGAAAATTTCGAAG
ATTGATATTGATTATCTAAGACGTGCGCCTCGTGTAGATATCAAGGACGAGACCAAGTTGAACGCTGACGAGAAGGCTTCTGAGAAGTATTATG
AGGACACTGTTCCTGGCACTTCTAATTTCATCTCTGAGGTCTTCTTTCTCACATTGG

>Bcin01g07220(MLST1), partial sequence [organism=Botrytis cinerea, strain SAS405]
TCAATGATTGTTTTGCCGCAGCCAAGTATCTCTCCTTCTTGCATCTCGAATCGGCGTGTCTCTTCCAGTATGGAATTCTACCTCATCGTAACCA
GGAATTCTAATACCCAACCCACCTTTGTGAGCATCGCTACTAGCGTTACTACTAGCCTCTCCAGCATCATTCTCTTCCTTCAGCACCACTTTAT
CCCCCGAATCATCTCTAACTAAAGTCCTGAATTTCCCCTCGACTTTATTCTTCAACTCATCGAAACCACCACTCTCAACTATCTCTCCCTCTTC
CATCACCACGACTCGTCCCGCTATCTTCATCATTTCAGCCGTATGACTAACAACCAGCGTGGCCATTCCTTCTCTCTTCTTCAACTTCTGCACA
GTCTCTCTTATCACCTCTGCACTACCACCATCTAATCCACTCGTCGCTTCATCTAATATCAATAAAGTTGGTTTTCTGACCAGTGCTCTTGATA
TCACAATTCTCTGGGCCTGTCCTCCCGATAACTCTTGTCCTCCATCTCCTACCATAGTATCATAACCTTGTGGAAGCGATGTGATAAATTCATG
GATCCCAGCATCTTTTGCTGATGCAATAGCAGATGGAAGACTAGCACAAGGAGAAGATTCTGGGAGACCATAAATGATGTTATGGAGAATGGTA
GCTGGAAATAGAATCGGTATTTGTGGAACGAGTGAGAGAGAAGCCCGGAGAGACGGAATGTGACAGTTACTTATGGAGACGCGATTGAATGTCA
ACGGTGGAGGTGTTGAAGTATCTGGCGGATAGAGACCAATGAGCAGAGCAGCTATTGTAGATTTTCCGGAGCCGGAGGGTCCGACAAGTGCAGT
TGTTGAGTTAGGAATCAGGGAAAGAGAAAAGGATGATATCGTTCGTTTTTCAGGACGAGTAGGGTATGTGAATGAAAGACGGTTGAATTTGATT
GGAAAGATTGTCGAAAGCCGTTCGGTTCCTTTATTTTCGTGGGAGGAAGATGAATCGAGATTGGCTAATGCAAGCATATGCGTAGCTGTAACGC
GAGAAGAGTTGATTTGTGGTATCATGGCCAGCATATTCGTACTATTAGAAATACCAAATAATAGAAGATTGACAGTCTGTAGTGCAGTCGCGAT
ACTGATCTCTCTCTTGGTAATGAGAACCGTGGCATAATAAAAGATAGTTGCAGTGATGAAGAATGACATCGCATCTGTCAATCCCCACAGCAAT
CCCGAGTAGTTTGCTCGTATTAGTCCAACTTTATAAAGTTCTTCTGTAGCTTTGGTGTGTTTTGTCTCAAAGTAAGTTTCCAGAGTAAAAGCCC
GAACCACTTTGATGTTGGAGAACGTCTCTGAAAATATGCCAGTGGTCATTTCAGAGGCGTAGTTGCACTTGTTTTCCCATTTTCCACTCACGCG
ATTGAACGTTTTGGTGACAGCGTATATAACTGGCCCAGTTGCCATCGAGACTAATGTCAGTTTCCATGTATTCACGAAAGCCCAAATGATTGAT
ATCAATAGCATAAAAAATGCTGTGAATACAATACCAGCAAAGCGGCCAACGAGATTACGCATTTCCTCAGAGTTCCTATCCAAAACTTCGTTCA
ACCGGCTAGGTGAATTCCTGGATTCCTCAAACCATGACTTCGGTTGTGCGAGAATCTTCTTTAGTGCTTCTACACGCAGAGCACTAACCCAAGC
TTGTCCAGCACGTTCCAAAGCATAATGCGAAAAGAAACAAGATATGCCATCAATGATGGCGATTCCGAGTAAGACAAGTGACCATTTTAAAGCA
TTCGAGTTGCGGTTCTCCTTTTGGTAAAAAGTGTCAAGAAGTTTGGTATATATTATAGCAAAAGCTGGGGTTGATACTGCCACAATAAATGCTG

CGGCAAATGCAACCGAAAGGATTGTGCGTTCCTTCTTGTTCAGCATGGGCCAAACAGTCTTTAAGATCTTAGTTAAAGTATCGGGTTTGAGATG
ACCCGTTTCGTTCTTCTCTTCAGAATCGCCCGCATTATCAGCAGAGTCATCTGAGTGTTCCAACAAAGAATCCATAGGCGCGACTTTAGAGTCT
CGATTCTTTTCGATTGATACCAAGCTGAGAGGGCTTTGAATCAGAGGTTTTCTATCAAAGCTTGGCTTGTCTGAGGGATGTTCTGGGATCGATT
GGAATCCTGCGTCTTGATACCTTGGTGGCCTGATAGGATGTATAGACTGCCTACTAGCCGACTCTTGGAACTTCTGAAACTGTGAGGTAGGTCT
TTGCAAATCATCAAACCTTGAACGTGAACTGTCAATTCCAGATGTCCAAATGTTGTCTGATTGTAGGTCATTGGCATAGGCTATTGAAGCATAT
CCAAGAGATAATCGTCTGTTTGATATATCCAAGGACCTACGATTTGCTGCATTTGGACCATAGATTGTAGACTTGGTAGAGAAACGAGGTTTCA
ATTCTTGAACAAAGTCTTCAAAGCTTCCAGTTAATGGTGTTCCAGGGTCCGAGTTTCTTCTGATGTTGGACAAGTCTGTCGACTGTTCAACGGG
CAATCCCGGGAGTTGCGAAAATGTAGCGAAAGTACCGTTTGCTTTCGCAGAAAGCTTCCTACGATATCCTTCCTGGACAACCTTACTCTGATCC
ATAACGTAAACGTAGTCATCATCCAATATCTGGGAAATATCATGAGTCACAATAATTGTCGTTTTTCCTTTCCTCCATTCTCGAATATTATCCA
TGATTTTGAGTCGGTTGGTCAGATCAAGAGCACTAGTTCCTTCGTCAAGAATCAAGATTGGTGCATCTCGTAATCTAGCTCGTGCCAATGCAAC
ACGTTGTTTTTGTCCACCACTCAGTTGTCTCTCCTTAGAGCCAATCATAGTATCAAGTCCATCAGGAAGATCGGCAATTACTTGTTCTAGATTG
GCTGTAGAACAGGCGTCTATGATATCAGCTTCATTTTTTATTCCTCCCTTGCCAAATTCTATGTTTTTCCTAACCGTCTCGTTGAATACCACAC
TCTGCTGCAGTACGAGAGTGATGTTGCGTCTCAGCCAATCGGTATCGAGATCCTGTATACGTTGGCCATCTATCAAGATACTTCCCTCCTGTGG
CTCGTAAAATTTGAGCAACAAGTTTCCCAATGTACTCTTTCCAGAACCACTTTTACCAACTACAAAAGTAGTCTCGCCAGCAGGAAAGAAGAAT
GTTGTTTTTGTAAGAGCATTGTGCTGTCGATTGGAAGGATATCCAAATGTAACCTGATCTTGATTAGTCTTGATGGTTTATCGTGGCCATCTCT
ACTTACATTATTGACTTCTATATCACCGTTGCAGGTTTTAGGTACCATTAACCCTTCTGGGTGTTTTGCAACTCCTCCTCTATTCACTTGGTTC
ATGATAGACTTGAGAGTCGCTCCAGCGGACATTCCTTTTGCTAAGACAAGCCATTGTGGTAAAACGACTTCTATAGCCATCATCCCAGTCAAAC
AAGCATAGAAAGTTGTAAGAACCTTTCCGGCATCTAGTCCATGATCGACTAACGTGAGACCATACCCAAAGCCTTGAACAAAGATGGCCACCAT
GACAAATTTGGTAATCCCAAATTGAAATGCATTTGACCTGGCTTGGATCATGTAATGAACTGTTGATTTTTTGATTGCTAAGAAGTATTGCCAA
ACTTCCTGGTCCTGTCCGTTGAATGCTTTGACAGTATCGATGGCTGTAATGGCTGTATTTGTAAATTTCGAAGCTTGCGATAGCTCTCTTTTCT
GGGCTTCGATGGCTGGGCCCATGTTTCTAGAAATTAGATAAAGTATTCCACCGGCAATTGGAAATGTCGCTATAATGACAAGAGTCAGATTCCA
AGAGTAAAAGAAGGCAGTCCCAAGAGCTGCACATGCGCTAGCAGTTTCAAACAAGAGAAACCCAAGAGGCTGGGATGTGGATAATTGGAGCTCT
CGAATTTGTCTATTGTTCAATCAGTATGTTTTCATTTCGACCATAAATGGATTCCACACACGTTTCAATTCGAATTAATAATGATCCAATGCCA
TCTCTACGCAGATCAAACCATTCCAAATCCTTCTCCAACATGCCGGCAAACATTTTCTCGCGCACGCTTCTAGCCTGGAGCTCTCCAAATACCA
TCCACGAACACAGAAACAAGCCTTCGAACAACCATACTGCGCCCCCGAGAACAGTTATAGCTATGCACCATTTCGAGACATGTTGTAAGGTCTC
TTGTGCGGTGAGAACACCGGACCCAAAATTTGTAAGAGTACCAAATATATTTCCGTAAAATATTGCCGATATAGGTTTTAGAACGCCGCTAATG
ATTGCAAATACTATACATGGAACTATGGTAGATATGTGCTTTTTGGTTGTGAATGTGAATAGGGATTTGAAAGATGATTTGATTTCTTCATCGG
CGTGGTAGTCATTGATATTTCGCAATTCGATGGACGAATTCTGATCATCATCTGGCAATCGGATGCGTGAGGTAGAAGTAGAATGACTGTATTT
TTTGTGACTCTTCGAGCCTTTGCTTTTCAATATTGTCAT

>Bcin05g07690(MLST2), partial sequence [organism=Botrytis cinerea, strain SAS405]
ATGATTTCGTTCTCAACACAGATCAATCCACGACCGCGTATATCTTTGCTACTGCTGTGCTTATTCTGCGCACTATCTTTCTCAATTGGCGTAG
ATGGGAGGCCCAACGATGTTGTGCGACAGAATGATTTGATTCAACGACCACTTACGAATAATGAAGATATCACAATCAATGGGAGCCCTCAGGA
GCTCATCAAGCCTGGAGACTCAAAGGTTCTACCGGCAATGTTAAGCGCGCTGGACGTGCTACAAGAAGACTACTTTGCGACATGGCAAGGCATC
TATCCAACAGGAATCGATTGGACATCTGCAGTGATTGGTACAATACTTTCTTTGCATGTTGAAACCACTTTAGAGCAAGAGCTAACACCACTCT
CTAAACAGGCACCTATGTTGCTGGTGCACTCACCACTATCACCAAGTCCTTTTCTGCACTTTCCTCATCAAAAACGAACGAGAACATCATCAAT
AAATACTTTGCTGAAGTAATTGGGTTTTATTTTGGCCAGGATGCCTTCTCTCTACGACAGCAAGCCTTCGATGATATGTTGTGGGTAGTTCTTG
GCTGGCTGGATACTGTCAAATTCATTGATTTACATTCTGAATTGCACTATTCAAACGACTCTCAGCCAGAATGGTACGGACAACAATATAAACC
TGCATTTGCACATCGAGCGCGACTATTTTGGGAATTGGCTTCACAAGGATGGGATACTACTCTCTGTGGTGGTGGGATGATATGGTCACCATAC
CTTACTCCATACAAGAACGCAATTACCAATGAACTCTATATCGCAGCTTCGATATCGATGTACCTATATTTCCCCGGAGATGACAATCAATCCC
CATTTATGCTTTCCAACCCTTCATATCCACCTCACGATCCGAAATATCTACAGGCAGCTGTTGATGCTTACAAATGGCTGAATGATTCCAACAT
GACGGATTTACAAGGATTATATGTCGACGGGTACCATATCTCGAATCTTTCTGGCTGTGAAAACACCCATTGCGATTCTAGAAATGAGATGGTA
TATACCTACAATCAAGGTGTTTTGCTTACTGGACAACGTGGTTTGTATGACGCAACCGCCGCACGATCATACCTTGTAGATGGCCACAAACTCA
TCGCGAATGTTATTAATGCCACAGGCTATGACCTGAAACACAATGTTGTCATCTCACCGCCACCCAAAGATGGTTCCGCATTGGCAAAGTGGTT
TGGCCTGGGTAGGAATGGAATACTGGAAGAAGGATGCGATTCAAGTGCTTCGTGTTCTCAGAATGGACAAACTTTCAAAGGCATATTCTTTCAT
CACTTGATTGCGTTCTGTAGTGATTTGCCAGGGGAGCCTATTGCAGGGACGAAGGAAAGCTTAGAACTCGACAGAGTGTGGCATTCTGACAAAT
GCTCACAGTATACAAAATGGATCAGGCGAAATGCCGAAGCTGCGTTAAGTACCAAGAATGAAGAAGGGAAATTTGGTATGTGGTGGGGTGTACC
GGCTACACAGAGTTCTTTAGCGGACTATACACAGGACCAGACATCGAGAGGCTCTGTAGATTATCGAAATACTGGAGTTCCAAAGAACACCGAA
TGGAGAGGAGAAGAATACCCCAGAGAAGCGAAAGCGAGCAGGACTAAAGAAGGCGCAGATGTATATAGTGGGGTAGAAGATCCCAATGATCGAG
GTAGAGGAAGAACGGTAGAAACTCAGGGTGGAGGATTGTCTGTCTTGAGAGCATTATGGGAGGTAGAGCTGAGATGA

>Bcin06g01710(MLST3), partial sequence [organism=Botrytis cinerea, strain SAS405]
CTAGCTCTGATTTGGTAATCCATTCCCATTAACAACAGCGACTTCACCCACACCCTCCGCCTTCCTATTCATAACAACCCCTTTGAGATTTCCC
CGCTTGCTCGCTTTCCCATCCCGGACCTCTTCAGTCGTATCAACTAGGATATCAAAAGCTTTGCGGATATAACTTTCTTCCGCCTTGGGATTCG
CACTCACGACTCGGATTACATATGCACCCGCCACCACGCCAGCTGTGAGATAGATTTCGCTTCGTTTGTTAATCAGCTCGTAGACTTCTTTCGT
GAGACTATTCTGTGCATCCGCATTTGTGGATTTGGGAACAATGTTCAGAACAGTGAGGGCGAAATTGGGCCCTGTAACTATATTGAATAGATCT
TCTCGTGTCTTGAGCAGGCTGACAAGTAATTCTCCGAGCTTCACGTGCTTTCGGATGTGCTCTTGTAGGCCCTTGACTCCGTAGGTTCTGAGGA

CAAACCAAATCTTTAAGCTTCGGAAGCGTCTTCCGAGAGGAATTTGCCAGTCCCGATAGTCGGTTACGAGTCCACTCTCTGAAAACTCGTTGCG
AAGATAACTTGGTGTTATGGAGAGTGCATCGATCAGATCTTTGCGTTTCTTGACATATAGACAAGAAGCGTCGAAATTTGTCAGAAGCCATTTG
TGCATGTTCATATCGAAGGAATGGAAATGCTGGAAGGACGATGTTAGATGATGGTATTCAGGGCAAACCAAAGCTGCACCTGCATAAGCAGCAT
CGACGTGAACCCAGATCTCGCCTGCAACATCTGGAGGTGCATATTTTGAAAGTACTGTTGCAATAGATGCGAAGTCGTCAACTGCGCATGTAGA
TGTTGTTCCCAAAGTCGAAGTTAGATAGAAGGGTTCCAATCCTTGAGATTTGCATTCTTTCAATACCTTCTCTAAATCATCACCCGTCATGGCG
AAATCATTGGATGCGAGTACTGGAATCGATCGGAATCTAACGCCAGCTATTTGCGCTGCTTTCTGCGTGGAGCTGTGTGCCATTTCGCTTCCTA
GTGCAACTAGCTTACTCCTCTTATATGCAATCGCATCCTCGAGTTCAATGCCCGACAGACCTTCAGTAGTTTCACGAAGATATTTATCGCGGGC
AGCAACCATAACGGTAACGATAGCTTCCGAGGCTGATCCTTGGATGACACCACCACCATGAGTCGAAGACAAATAACAGTCTGGGAGATTGAGA
AGCTKGSCCAGCCAATCCATTACAACCGTCTCCAATTCTGTCACAGCAGGGGAACAGATCCAATTGAAAGCAGGTGCTGTGAAAGCTGCTGAGT
ATAATTCTCCCAGCATTCCAGGGTAGGTAGATGATGCAGGGAAGAACGCCATGAAATTAGGTGATTGCCTTGGTATATCAGTGTCTATCTTAAC
GCTCAAATACAAAAGTCAGACTCACCAATGTGTCAGACCAGGCATAATTTTAGATTCGATATCTTTCTGGATCTCTGCCCATGATTCACCCTCT
TCCGGTGGACCATCTGGAAGAATCTTCTTCAGGTAACCAGGCTCGACATTTGAAATTACTCTTCGTTCATGAATGTTATCATAATATTGGATGA
CTATGCTGGTCAGTATACAGTACTTCATCTTGGGAAGCAGGAACTTACTTTCGTCAATCGCCGACGTCGCCGCCTCCCTAAACTGCTTCGAATC
CAT

>Bcin09g03030(MLST4), partial sequence [organism=Botrytis cinerea, strain SAS405]
ATGGCTCSCCTCAATCCCACAACGAAATCAAAAGCCCCTCCGCTCTTCAGGCCTCCTGCTACCCCCGCTACAAATTCGAATCCTATCCCCTCAT
CATCGCCTGCATTTGCAACTCCGGTACATCCTATACGACCCTTCAACCCCACCGCAGCTCCTATACCGAAAGCGAGTATCCTACCAATTCTCCT
TCCACCTGCGACTTTAAGACCATTGGCTTTCCGCACTTTCACAAAAAAGCATAGTTTAACATTGACGTCGTCGGCATTACAAGTGTTGGCTACT
TTTATTGGAAAGCATTGTGGGACAGGATGGAGGGAAGAAGGACTGGCAGAGAGAGTCTTGGAGGAGGTCGCCAAGAGTTGGAAGAATAGGAGTG
GCGGTGTCATTGTCGAGGGCGAGGGAACGGAATTGAAGGAGATTCTGAAAGCTTTGGAAGGGAATATGAGTGGTGGAAGGATAGTCATAGGAAG
AGAGCTAAGCCGGCAGAATAGTTTAGTACTGGGATCATCACAATATGGAGAGGTCAATCATACAAGACTTGGGCTACGGCCAGGGAATATACCC
AGAGAGGATAGTCAGTCAAGTTTGGGAATGTCAACGTTGGAGGTCAATGACGAGGAAGATGAGGATGGCCTGATGGATCCAAGAAGGTGGTTAA
AAGTCATTGATGCATTTGAGCAACCTCGACTGGTGTACAATGTTGCTAAAAAGCACTTTGATAGGTATGTTTCAATGATAAAATTTTATCTGAA
TCGACTAACTCAGTACAGAGATACCTCCAAACCTTCATTGTTCCCACCTGCGTCTCATAAAACACTCCTCTTCCAAAACCGCTATAATGTTATC
CATCAACGTCTCCTTCGCAATGAATCTTTTCAAACGCCCGCTTTTCAAGGTGGCAAATCTTCCCTTCAACGCAGCACGTCCGCCATTACCACCC
AACAACAATCATACAAATTAACGCCGATAGCTAATCTTCTCGGTCGCAATCGCAGCTCTCATATGCTTCTCGGTCTCCTCAGTATTTCACCCAC
TGGTACCCTCGCCATCAATGACCTGACGGGCAGTATCGCTCTCGATCTTACACACGCAGCAGCCATTCCCGAAGATAGCGCGTGGTTTGCCCCT
GGGATGATGGTACTCGTAGACGGCACGTACGAGGAAGATGAAACTGGGACGTCATCTCGTCTTGGTGGAAATGGGGGCGTAGGGGGTACTATTT
CTGGAAAATTTGTCGGCTTCTTCATCGGCCACCCTCCCCCCGAACGCCGTCATGTGACTTTAGGCACAGCCGGTGAGGGAGATACCACAGCCGG
CGGTGGTTTCGGCTGGGTAGACTTCCTAGGCGTGGGTAGTTCTCGCGCCCTAGGCAACAAAATGCAACGACTGGAACAAAAGCTTCTCCGACCC
CCTCCCCCTGACACAGATACAGATGATCCCCCGCCTCCATCCAGAGGCCGTGTAGTCATTCTTGGTGATGTACATCTCGACATTCCTCAAACTC
TTCAAGCCCTCAAAAAGATTCTCTCTCTATACTCATCTGAACCAGAAGGCTGCACACCTATGACCTTTATCCTCCTCGGCTCTTTCGTCTCTCA
TGCCGTACTGGCTCGGGGCGGRTCGGGAGGCTCTATAGAATATAAAGAGTATTTTGATTCCCTTGCAGCCGTTCTCTCTGAATACCCTACCATC
TTAAGTACAGCAACATTCATATTTATCCCTGGCCCCAACGATGCATGGGTTTCAGCCTTCTCATCCGGGTCTACTGTTCCTCTGCCCAGAAAAC
CCGTACCGGAAATGTTCACATCCCGCATAAAACGTGCCTTTGCAAACGCAAACACTGAAATGGAGAAAGAACACGGTAATAAAGGAGATGGAGA
GGCCATTTGGACGAGTAATCCTGCAAGAGTCAGTTTATTTGGCATGAGCTGTGAACTGGTAGTATTCCGCGACGACGTTAGCGGACGATTGCGT
CGTACAGCTGTTACACTCAAGTCATCTCAAACGTCAAACCCTGAAAACGAAGATGAAGATATAGATATGTCACCACCTCCATCTTCAATTCCCT
CTTCCACCCCTCCTCCAGAAATCGATCCCGATATCCACACCGCGCGTCGTCTTACCAGGACACTCCTCGATCAGGGACATCTTTCACCCTTTCC
TCTAAACATCGCACCCCAGCATTGGGATTTCTCAAATGCGTTGAGCATTTATCCATTGCCAACAGCAATTGTTATGTGCGATGTAGATAGCCCG
GCATTTTGCTTGACGTACGAGGGGTGTCATGTAATGAATCCAGCGAGTGTAGTAGCAAAGGGGAGAAGAGGCGTAGCGAGATGGATAGAGTATG
ATGTTTGGGGACGGGCTGGGAAGGTCAGAGAGGTGGGATTTTAG

>Bcin11g01310(MLST5), partial sequence [organism=Botrytis cinerea, strain SAS405]
ATGCCTTCCATTAGCAGCATTCTAGTCGCCACTGTCAATTTGTCGGTCCTTGTGAATGGACTGACATGGACCTCATGTGGAACCGGCGTAGAAT
GCGCAACGCTTGAAGTTCCGCTCGAATATGGCGATGCAACGTCAACGGCAAAAGCCAGTGTTGCGCTTGCTCGTTATCCTGCCACTGTTGCCGC
GAGCAAGAAGCTCGGGTCTCTCTTGATAAATCCCGGTGGACCCGGTGCCTCTGGTGTTGGCTTTGTGCAGTCTGGAGCCGGTGCCGCCGTCTCG
ACACTGAGTGGTGGATTGTACGATATCATCGGATGGGATCCACGTGGAACCGGTGCTTCGGCTCCTATTTTGGAATGTTTTGCAAATGCCAGTG
CGGAGTATGATTTTAACAACGCGTTTCCATCTGCTCCGAATCTCTGGCTCGGACAATTTGCGAATGCCAGCGCAAATTCTGCTGTTAGCTCTGC
TATCACATCCTTTGACACTTCTGTCGCTGCTCTTGCAAAAGCTTGCGTGGCTCAGAAATCTCCCGCTCTTTACACCTCAACAGCAGCATATGTT
GCTCGAGACATGGCAGCGATAGTCGATGCATTGGATGGGACCTCTGCAAAACTTAACTACTGGGGTTTCTCATATGGAACTATTTTCCTAGCTG
AGTTTATCCAAACTTTCCCAGGCCGCGTGGGAAGAGTTCTTGCCGATGGTGTTTTCGACGCAAAGGCAAATGCACTCACATACGTTAGCCAACT
TCCCAACGATCAACTCAGTGTTCGTGCTTCGTTGAACGATTTTGCAGCTTTCTGCACCACCGCCGGTAGTAAAGGTTGCTCTTTTGCCACCGCC
CCTACTGGAACCACAGGTACTGTTGCTACCAGACTGGACAACATAATGAAGGATATGTTCCTCAATCCTATTGTTGCTTCGGGCTTAAGCATCA
GCTTAGATATCCTCAGTCCCCTTCTTGCATCTCTTCTTAGAGTTCCAACCACGTGGAAAACGCTTGCATCTGTCTTATCCGGTCTTGAAACTCG
TGACGCAACTGCTCTTATTTCACTCCTTGGATCGCTAGCAGCGAGTGCACCAACCGACGGCTCTGCAGCAGGCGTTGGCACTCTTGCTACTTAC
CCACTTGGTTGTGTTGATAACGCTGCTTCAAATGGAGTTACCTTGGACACTGTCATTTCTCTCACCAAAAGCATCTCAATCTCCGAAGACACCC

CAATACTAAATGCTGGACTTATCCCCATAACATTTTGTCGCAACTTCCCTTCCACGCGTCCGCTTGTTCCAAACGTAGGAGCAAGTTTGATGTC
GAAGACCGATACTCTTCTCGCAACAGCCAAAACACCAATCCTCATTGTCTCGGCCGAGAATGATCCAACAACCCCTCTCAAGTCTGCAAAAGCA
CTCCGAAGCCTACTCCCTAGTTCATCCACCCTCGTATACCGCGGAGGAAGCGGACACACCACCATCTCACACGCATCTCTCGGAATGGCAAAAG
CGATCTCTAATTTCTTTGTCAGCGGTACCATGCCCAAGGACGGAGCTCGATTTGCAGTGGACCAGAATATTTTCCCAACAGCTGCAGCGAGTGG
TTTAGTTACACCAGCTGCTTTCAACGGAACCTATTCTACACAAGATCAAAGTTTCCTAACCGCAACATACAACATTGGCATTGCCTTCTTAGCA
ATCGCATAA

>Bcin15g03910(MLST6), partial sequence [organism=Botrytis cinerea, strain SAS405]
TTACAACTCCTCCTCGTGTCCCGTCATCTGGGCTTGCTTCTCCCCAAATTTCTTCCCTGCTGGACCAAAATACTCTTTGAAAGCCTTCTCTAGC
ATCTCCTCTCCTAACGTCATATGTCCCCCTGGAATGGTTTCGAAAACTAATTTTCCGGCCTCATCCAATGTCTTTAAACCTAGCCAATCTTCTT
TATAAATGGCTCTTTCTTTCAGTGGTGTAACTTCCGTGCCGTTGACTTCAGCCCACCATCCACTTTCCTTAGGGATGACAGTTGTATCGTCTTC
AAAGACATACATTACGAATCGTTCCAATTTTTCCATGTTGGATTTATATGTTTGGTTCTTGAGAACGCGCTCATTATTGATGTCGGCAAGGAAA
TTGGAATATTCAAGGTAAGAGTCTAGGTTTTCCGGATCTCTGAAGTATTGAGCGGGTACGAGACGAGATTGGACAAAGGTTGACCAGGTGTTGG
ATCGCAAAAGGGTTTGAGCACCGCGACAGAGGAAATCGGCAGGACCACAGGCTTGGAAGGCAGAAATGCCGTTGTGTTGGGAACCGAAGGTCAG
GAGAGAGCGGATGGGTGGAGCATTGCAGCGGGATATGTAGCCACGCAAGAATTGGCCGCCTTGGGAGAATCCAATTGCGTCGACGGCGGGCGCG
GTAGAGAGAATAGGATGGGAGGCGAGGTCTTCGCAGACCTTTTCGATTTGAACTTGGTAAGAGAGTTAGTATGATATTTAAAGGAAAAATAGGG
GATGTACTCACGAGTGAGATTTCCAAAGAAGGTAGCTGTCCTATCTGCAGATGCATCCTCATCTACATGAATATTGTAGACAAAAGTCCCAGGA
TGAATAGCTTCAGCTAGTTTTCCAACTTGCGCAAGACCATCCGCTTTGTAATTATCGCCGAGTCCATGCCAGATAATCAAGGGAAGTGGAGTAT
CATCTTCATCGTTGGATGATTTTGTGTTTTGGCGAGTCTTTCCGGACAAGGTGAGGTTGAAAATACTTGGAAGAGGACTGTTTTTATCGTAGAC
AGAGGCATGTTCATCATGCGCGACGGGAAATTCAAGAACACTCTCAGGCATAACGATAGCGTTGATGAGCGATGCGCTGCTGAGCAACGAGATA
ATTTTGGTAATGGGCGCCAT

>Bcin16g03460(MLST7), partial sequence [organism=Botrytis cinerea, strain SAS405]
ATGCTTATTTACGCTTTCGCATTGATACCTCTTTACGTCCTAGTATGGGTGCCTCTTTCTCAAGTAATCTACGGTCGTCAATCTGCTCTTCATG
AACCTCTTAATTTGAACTCTTCATTATTTGCAAGTGATGAACCCCTATTCTGCCCAGAGGATAGTTACAAGACGTATATCATTAGTCGAGAACC
ACTCATGATATATATTGACGGATTTTTGAAAGCGAATGAAAGTAAACATTTGGTTGATGTTAGGTGTGTTATTTATTCTGATGAAATGAACAAG
AGAGACTGATGAGATAGTGAGCCGCTTTATGAACCGTCAACTGTTTCTCACGGACAGGAAGTTACCATTGATCCTTCAGTTCGGAATTCTGAAG
TGGCGGTTTTAGAGAGGGATGAGGTGGTCAGGTGTATTGAGCATAGAGCGAGGGCATTTCAGGGGTGGAGGGGCGAGATGGGGATTGAGAAGTT
GAGGACGCAGAGGTATGGGGTTGGAGGACATTATGGGATGCATTTGTAAGTTTTGGGGGATTGACGAAGGCTTTTGTCTATTTTCTACCAGTAC
GGATCTTGAAAGAAAGATAGCATGAACACGAGGGCTAATAACTAGGAAATAGCGATTGGAGCGGAGGTAAACGTGGCATAGACCGATTTAGTAC
TTTCATGGTCTATGTCGACGTATCCTCTGATATCGAAGGTGGAGGAACGGAATTCCCACGTATCGTGGGACCAAAAGGAGGAAGGTGGGAGGAC
TTCCTGGAAACTACGGAAGCATTGGATCCAAGAACTGGAGAAAATGTAACAGTAGAAGGGGTGACATTCAAACCAATCAAGGGAAATGCCGTAT
TCTGGGAAAATACTGACAACAACGGGAGGGGCTATGATGAAACATGGCACGCTGGTCTACCGGTGGAAAAAGGCTCGAAAGTAGGGTTGAATAT
TTGGAGTTATGGGAGGACCATTAGATGA

>Bcin12g03020(MLST8), partial sequence [organism=Botrytis cinerea, strain SAS405]
ATGGCCGACGATGGAATGCTGATGAATTTTGAGATTGGGGATGTACCTATTGTTGCGAAACAAGCATTTAAAGGAGGACGTTGGAAGGATCGAT
TGGCTGCGAAGAAAACTGCGCAGCACCGAGTCACCAAAAGCACGAATAAACCATCGGCAAGGGAAATATTCTCTGAACCACAACATGACACGAG
CGCGGAAGAGTATATCGGGCGAGAAGCTTCATCAAGAGCACCAAAGCGACAACGAGTCGATGATAATTATAACTCTTACGGTGGAAGAAATGAA
AATACAGCAGCTTATGCTTCCGGGAAACTTCCATCTGGAAGTATAAATGTTGGTGGAGGTAGGAAGACCACTTTTCAAGAAGAACCACGAACGG
CATTTGTCGCTGGCAAGTTGCCCCCTGGAAGTATCAACATTGGTGGAAAGAAGGCTACCCAAATCGAGGACGAGGGTAGAGCAGCTTATGCTTC
CGGAAAATTGCCTCCAGGAAGTATAAACTCTGGCGCAAAGAAGGCGATTTCATTCCAAGATGAAACGAGACCGGCTTATGTCTCCGGAAAGCTT
CCACATGGTAGTATCGACGGTATGCGAAACCGTGAAATGGCTGCCGTCCACCGCGAAATTGCTGAGGGTGGGAGGAAACCAGGCCAGGTTGTCT
CTTCTCTATTCACATTCAATCCTACTTCAAAGAAAACTTTTGATGAACCCGAAGAACAAGCGGAACCGGCAAAACCATCCAATGCGCCTTTGAC
AGAGGAAATGGCGACTTTCACCAATCTAGGGTTATCGAGAAGGCTTGCAGCCCATCTATCGACTAAACTCGATATGAAAGCTCCGACCGCCATT
CAAAAAGCATCTGTACAGCAGTTGGTATCGGACGATAGCGATGCTTTCATACAAGCAGAGACTGGATCTGGAAAAACTTTGGCATATCTACTAC
CTATAGTCGAGCGAATATTAGCATTGAGTGAGAATGGCGTACAAATACATCGAGATTCTGGACTTTTTGCGATTATTCTTTCACCTACAAGAGA
ACTGTGCAAACAAATCGCGGCTGTGCTGGAGAAAGTTTTACGTTGTGCGCCATGGATTGTTGGTACGACCGTTAACGGAGGAGAGAGTAAGCAA
TCAGAAAAGGCTAGACTTCGAAAGGGTGTCAATATTCTTGTTGCAACACCTGGTCGATTAGCAGATCATCTCGATAACACAGAAGTGTTGAATG
TAGCTACTGTAAGATGGCTAGTGTTGGATGAAGGAGATAGATTAATGGAGCTTGGGTTCGAGGAAGAGATTAAAGGCATCGTTGAGAAGATAGG
TCGAAGATCAGTGGCCAAAGCAAATTCGGACATGGGGTCACTCCCGAAACGAAGAGTTACTATCTTATGTTCTGCTACGATGAAGATGAATGTT
CAGCGTTTGGGAGAAATCAGTTTAAAGGATGCTGTGCACATTCAAGCGGATCCTTCCGAACAAGAGAAACAAGACAAGGAAAATGGCGTTGAAG
CCCAAGATAAAGCTTTCTCCGCCCCTACGCAATTGAAGCAGTCATATGCTATCGTTCCTGCGAAGCTCAGACTTGTTACCCTGACGGCTCTATT
GAAACGTGCTTTTGCACGAAAAGGGTCCGTGATGAAGGCGATCGTTTTCATTTCTTGTGCTGACTCTGTTGACTTTCACTTCTCGCTCTTCTCT
CGTACCCCAGAAGCTTCAGCCGAGGTTGTGGACGAGGAGAAGGTTGATCTTCCCGCATTACCAAAATCAGAATTGGTCAAAGAGACTATTGCGC
ATGGAACTACAATTTCGAATAATTCAAACCCTGTAATTCTACACAAACTTCACGGATCTCTTGCACAAAACATCAGAACAGCAACCCTCAAGGC
ATTTTCCGAATCTGCGGACCCTTGTGTTATGATATGTACTGATGTTGCATCTCGAGGTCTCGATCTTCCAAATGTCGACTTTGTCATCGAGTAT

GACCCGCCTTTTAGCGCGGAAGATCATCTACATCGTGTTGGTCGAACAGCCAGAGCTGGTCGCGAGGGTCGTGCTTTGATCTTCCTTATGCCTG
GCGTCGAGGAGGAATATGTTTCTATCTTAGCATCCGGATACCGTGAAGGAAAGAAAGCTCTGACACGTCACACTGCTGAGGATTTGATACAAAA
GGGTTTCGGTGGTATAGGTCGCGAATGGGAAGAGAGAGCTACGAATTTCCAACTTGAAGTTGAACGATGGAGTTTGGACTCGCCAAAATATCTT
GAGATGGCACGCCGTGGATACCAGAGTCATATCAGGGCTTATGCCACTCACGTCGCAAATGAGAGACACATATTCAATATGCAAGAATTGCACT
TGGGTCATTTGGCCAAAGCATTTGCCTTGCGAGACAAACCTGGAAGCATTAAGGTGCCAGGATTAAGACCAGCGAAGATGACCAAAGCAGATCG
AAGTGTCGCGGCTAGGAAGGCGAAGAGGGGTGAGAAAGCAGAAGATAAAGCTCCCGAGGGAGAAAGAGTGCGGAAACAAAAGAAGATGGAGCTG
GACTTGCCTACTGTGGATGGCAATGAAGCTGCCGCGAGGATGAAGAGGAAGATGAAGGAGCATATGGCTGCTGCGAGCGAGTTCAATATTGGTT
GA

>Bcin02g07770(MLST9), partial sequence [organism=Botrytis cinerea, strain SAS405]
ATGGTTGCCTTCTCAAAATCATTACAGCTTTCCCTTTCGGTCTTGGCATCTACAGTCATTGCCATCCCTACACCATCACAACTTGAGTCTCGGG
CCGTTATCGATTCCGATGCCGTTGTAGGATTTGCCGAAACTGTTCCCAGTGGGACCGTAGGAACAGTTTATGAGGCATATAAACCATTCCTTAA
AGTCGTAAATGGATGCGTACCATTCCCTGCCGTCGATGCATCGGGTAACACAGGGTATGTCCTTATATCTTTCTCTTCCACACGATTGCTATTG
AGTCTCTAACATATTTTAGTGGTGGTTTGTCACCAACTGGCAGTAGCAATGGTGGTTGCAGCAGCAGTACCGGTCAAGTATATGTTCGAGGAGG
ACAAAGCGGATCAAACTACGCCATCATGTACTCCTGGTAAGTTCTCTCTAAACTTCTCCTTATAGATCCAACCTAACAAAATCTTAGGTACATG
CCAAAGGACGAGCCCTCAACCGGTATTGGTCACCGTCACGATTGGGAAGGTGTAATTGTCTGGCTCTCCAGCGCCACCGCCACAACTGCCGACA
ACATCTTAGCCGTTTGTCCTTCCGCCCACGGAGGCTGGGATTGTTCCACGGATGGCTATTCCCTTTCTGGTACCAGCCCTCTTATCAAGTACGA
AAGTATCTGGCCCGTCGATCACTCAATGGGTCTTACTAGTACTGTTGGTGGAAAACAACCTATGATTGCTTGGGAGTCTTTACCAACTGCTGCT
CAAACTGCTCTTGAGAACACCGATTTCGGTGCTGCGAATGTTCCATTCATTCCGGCTGTTTTCACAGACAATCTTGCGAAGGCTACTTTCTAG

>Bcin04g02090(MLST10), partial sequence [organism=Botrytis cinerea, strain SAS405]
AACTGTCGAATATGACTATGAATGATGTTTACAAGCCCTACATCCATGTAAGAAATGTAGAATAGAGAGATCAGTAACTGGAACTAATATCGTT
TGTAGGCTTTCAAGTTACTTACGCAGTTCAACCCAATCACTACAGCTATTGCCGAATCCCCACTATTTCAAATGGCTGTCTCAGCAAATACCAT
CGAAAAGTACACACTGCTAGGCCCTTTCTTCAGAATATCTCCTCTGCAACAGGAAGTTACCAGGGAATACTTCAGTGCGCCAAAGACGATAGAT
AGGCGACACATTGCCACATCTCAAGATGCCTTACGATTGACCTTACAAACCCATCAAAAAGATTTACTTGATATCATCAACCACTTTGTTCGAG
CAAGTCCAATCGCCAAAAGCAAAACCTTGGATTGGTTCGCCTACATTGTGAATCAAAATCACAAGCGTCGAGCACTTCAGGTAGACCCGAAAGA
AGTGTCTTCTGATGGCTTTATGCACAATGTCACTGTCGTTCTAGATGGTCTTTGTGAGCCATTCATGGATACCACATTCTCGAAAATTTCGAAG
ATTGATATTGATTATCTAAGACGTGCGCCTCGTGTAGATATCAAGGACGAGACCAAGTTGAACGCTGACGAGAAGGCTTCTGAGAAGTATTATG
AGGACACTGTTCCTGGCACTTCTAATTTCATCTCTGAGGTCTTCTTTCTCACATTGG

>Bcin01g07220(MLST1), partial sequence [organism=Botrytis cinerea, strain U10_SC_BRO1]
GGTGTTGAAGTATCTGGCGGATAGAGACCAATGAGCAGAGCAGCTATTGTAGATTTTCCGGAGCCGGAGGGTCCGACAAGTGCAGTTGTTGAGT
TAGGAATCAGGGAAAGAGAAAAGGATGATATCGTTCGTTTTTCAGGACGAGTAGGGTATGTGAATGAAAGACGGTTGAATTTGATTGGAAAGAT
TGTCGAAAGCCGTTCGGTTCCTTTATTTTCGTGGGAGGAAGATGAATCGAGATTGGCTAATGCAAGCATATGCGTAGCTGTAACGCGAGAAGAG
TTGATTTGTGGTATCATGGCCAGCATATTCGTACTATTAGAAATACCAAATAATAGAAGATTGACAGTCTGTAGTGCAGTCGCGATACTGATCT
CTCTCTTGGTAATGAGAACCGTGGCATAATAAAAGATAGTTGCAGTGATGAAGAATGACATCGCATCTGTCAATCCCCACAGCAATCCCGAGTA
GTTTGCTCGTATTAGTCCAACTTTATAAAGTTCTTCTGTAGCTTTGGTGTGTTTTGTCTCAAAGTAAGTTTCCAGAGTAAAAGCCCGAACCACT
TTGATGTTGGAGAAAGTCTCTGAAAATATGCCAGTGGTCATTTCAGAGGCGTAGTTGCACTTGTTTTCCCATTTTCCACTCACGCGATTGAATG
TTTTGGTGACAGCGTATATAACTGGCCCAGTTGCCATCGAGACTAATGTCAGTTTCCATGTATTCACAAAAGCCCAAATGATTGATATCAATAG
CATAAAAAAATGCTGTGAATACAATACCAGCAAAGCGGCCAACGAGATTACGCATTTCCTCAGAGTTCCTATCCAAAACTTCGT

>Bcin05g07690(MLST2), partial sequence [organism=Botrytis cinerea, strain U10_SC_BRO1]
AGGATGCCTTCTCTCTACGACAGCAAGCCTTCGATGATATGTTGTGGGTAGTTCTTGGCTGGCTGGATACTGTCAAATTCATTGATTTACATTC
TGAATTGCACTATTCAAACGACTCTCAGCCAGAATGGTACGGACAACAATATAAACCTGCATTTGCACATCGAGCGCGACTATTTTGGGAATTG
GCTTCACAAGGATGGGATACTACTCTCTGTGGTGGTGGGATGATATGGTCACCATACCTTACTCCATACAAGAACGCAATTACCAATGAACTCT
ATATCGCAGCTTCGATATCGATGTACCTATATTTCCCCGGAGATGACAATCAATCCCCATTTATGCTTTCCAACCCTTCATATCCACCTCACGA
TCCGAAATATCTACAGGCAGCTGTTGATGCTTACAAATGGCTGAATGGTTCCAACATGACGGATTTACAAGGATTATATGTCGACGGGTACCAT
ATCTCGAATCTTTCTGGCGGTGAAAACACCCATTGCGATTCTAGAAATGAGATGGTATATACCTACAATCAAGGTGTTTTGCTTACTGGACAAC
GTGGTTTGTATGACGCAACCGCCGCACGATCATACCTTGTAGATGGCCACAAACTCATCGCGAATGTTATTAATGCCACAGGCTATGACCTGAA
ACACAATGTTGTCATCTCACCGCCACCCAAAGATGGTTCCGCATTGGCAAAGTGGTTTGGCCTGGGTAGGAATGGAATACTGGAAGAAGGATGC
GATTCAAGTGCTTCGTGTTCTCAGAATGGACAAACTTTCAAAGGCATATTCTTTCATCACTTGATTGCGTTCTGTAGTGATTTGCCAGGGGAGC
CTATTGCAGGGACGAAGGAA

>Bcin06g01710(MLST3), partial sequence [organism=Botrytis cinerea, strain U10_SC_BRO1]
AGCTTCGGAAGCGTCTTCCGAGAGGAATTTGCCAGTCCCGATAGTCGGTTACGAGTCCACTCTCTGAAAACTCGTTGCGAAGATAACTTGGTGT
TATGGAGAGTGCATCGATCAGATCTTTGCGTTTCTTGACATATAGACAAGAAGCGTCGAAATTTGTCAGAAGCCATTTGTGCATGTTCATATCG
AAGGAATGGAAATGCTGGAAGGACGATGTTAGATGATGGTATTCAGGGCAAACCAAAGCTGCACCTGCATAAGCAGCATCGACGTGAACCCAGA

TCTCGCCTGCAACATCTGGAGGTGCATATTTTGAAAGTACTGTTGCAATAGATGCGAAGTCGTCAACTGCGCATGTAGATGTTGTTCCCAAAGT
CGAAGTTAGATAGAAGGGTTCCAATCCTTGAGATTTGCATTCTTTCAACACCTTCTCTAAATCATCACCCGTCATGGCGAAATCATTGGATGCG
AGTACTGGAATCGATCGGAATCTAACGCCAGCTATTTGCGCTGCTTTCTGCGTGGAGCTGTGTGCCATTTCGCTTCCTAGTGCAACTAGCTTAC
TCCTCTTATATGCAATCGCATCCTCGAGCTCAATGCCCGACAGACCTTCAGTAGTTTCACGAAGATATTTATCGCGGGCAGCAACCATAACGGT
AACGATAGCTTCCGAGGCTGATCCTTGGATGACACCACCACCATGAGTCGAAGACAAATAACAGTCTGGGAGATTGAGAAGCTTGGCCAGCCAA
TCCATTACAACCGTCTCCAATTCTGTCACAGCAGGGGAACAGATCCAATTGAAAGCAGGTGCTGTGAAAGCTGCTGAGTATAATTCTCCCAGCA
TTCCAGGGTAGGTAGATGATGCAGGGAAGAACGCCATGAAATTAGGTGATTGCCTTG

>Bcin09g03030(MLST4), partial sequence [organism=Botrytis cinerea, strain U10_SC_BRO1]
GCAGAGAGAGTCTTGGAGGAGGTCGCCAAGAGTTGGAAGAATAGGAGTGGCGGTGTCATTGTCGAGGGCGAGGGAACGGAATTGAAGGAGATTC
TGAAAGCTTTGGAAGGGAATATGAGTGGTGGAAGGATAGTCATAGGAAGAGAGCTAAGCCGGCAGAATAGTTTAGTACTGGGATCATCACAATA
TGGAGAGGTCAATCATACAAGACTTGGGCTACGGCCAGGGAATATACCCAGAGAGGATAGTCAGTCAAGTTTGGGAATGTCAACGTTGGAGGTC
AATGACGAAGAAGATGAGGATGGCCTGATGGATCCAAGAAGGTGGTTAAAAGTCATTGATGCATTTGAGCAACCTCGACTGGTGTACAATGTTG
CTAAAAAGCACTTTGATAGGTATGTTTCAATGATAAAATTTTATCTGAATCGACTAACTCAGTACAGAGATGCCTCCAAACCTTCATTGTTCCC
ACCTGCGTCTCATAAAACACTCCTCTTCCAAAACCGCTATAATGTTATCCATCAACGTCTCCTTCGCAATGAATCTTTTCAAACGCCCGCTTTT
CAAGGTGGCAAATCTTCCCTTCAACGCAGCACGTCCGCCATTACCACACAACAACAATCATACAAATTAACGCCGATAGCTAATCTTCTCGGTC
GCAATCGCAGCTCTCATATGCTTCTCGGTCTCCTCAGTATTTCACCCACTGGTACCCTCGCCATCAATGACCTG

>Bcin11g01310(MLST5), partial sequence [organism=Botrytis cinerea, strain U10_SC_BRO1]
TCATGTGGAACCGGCGTAGAATGCGCAACGCTTGAAGTTCCGCTCGAATATGGCGATGCAACGTCAACGGCAAAAGCCAGTGTTGCGCTTGCTC
GTTATCCTGCCACTGTTGCCGCGAGCAAGAAGCTCGGGTCTCTCTTGATAAATCCCGGTGGACCCGGTGCCTCTGGTGTTGGCTTTGTGCAGTC
TGGAGCCGGTGCCGCCGTCTCGACACTGAGTGGTGGATTGTACGATATCATCGGATGGGATCCACGTGGAACCGGTGCTTCGGCTCCTATTTTG
GAATGTTTTGCAAATGCCAGTGCGGAGTATGATTTTAACAACGCGTTTCCATCTGCTCCGAATCTCTGGCTCGGACAATTTGCGAATGCCAGCG
CAAATTCTGCTGTTAGCTCTGCTATCACATCCTTTGACACTTCTGTCGCTGCTCTTGCAAAAGCTTGCGTGGCTCAGAAATCTCCCGCTCTTTA
CACCTCAACAGCAGCATATGTTGCTCGAGACATGGCAGCGATAGTCGATGCATTGGATGGGACCTCTGCAAAACTTAACTACTGGGGTTTCTCA
TATGGAACTATTTTCCTAGCTGAGTTTATCCAAACTTTCCCAGGCCGCGTGGGAAGAGTTCTTGCCGATGGTGTTTTCGACGCAAAGGCAAATG
CACTCACATACGTTAGCCAACTTCCCAACGATCAACTCAGTGTTCGTGCTTCGTTGAACGATTTTGCAGCTTTCTGCACCACCGCCGGTAGTAA
AGGTTGCTCTTTTGCCACCGCCCCTACTGGAACCACAGGTACTGTTGCTACCAGACTGGACAACATAATGAAGGATATG

>Bcin15g03910(MLST6), partial sequence [organism=Botrytis cinerea, strain U10_SC_BRO1]
TCTTTGAAAGCCTTCTCTAGCATCTCCTCTCCTAACGTCATATGTCCCCCTGGAATGGTTTCGAAAACTAATTTTCCGGCCTCATCCCAATGTC
TTTAAACCTAGCCAATCTTCTTTATAAATGGCTCTTTCTTTCAGTGGTGTAACTTCCGTGCCGTTGACTTCAGCCCACCATCCACTTTCCTTAG
GGATGACAGTTGTATCGTCTTCAAAGACATACATTACGAATCGTTCCAATTTTTCCATGTTGGATTTATATGTTTGGTTCTTGAGAACGCGCTC
ATTATTGATGTCGGCAAGGAAATTGGAATATTCAAGGTAAGAGTCTAGGTTTTCCGGATCTCTGAAGTATTGAGCGGGTACGAGACGAGATTGG
ACAAAGGTTGACCAGGTGTTGGATCGCAAAAGGGTTTGAGCACCGCGACAGAGGAAATCGGCAGGACCACAGGCTTGGAAGGCAGAAATGCCGT
TGTGTTGGGAACCGAAGGTCAGGAGAGAGCGGATGGGTGGAGCATTGCAGCGGGATATGTAACCACGCAAGAATTGGCCGCCTTGGGAGAATCC
AATTGCGTCGACGGCGGGCGCGGTAGAGAGAATAGGATGGGAGGCGAGGTCTTCGCAGACCTTTTCGATTTGAACTTGGTAAGAGAGTTAGTAT
GATATTTAAAGGAAAAATAGGGGATGTACTCACGAGTGAGATTTCCAAAGAAGGTAGCTGTCCTATCTGCAGATGCATCCTCATCTACATGAAT
ATTGTAGACAAAAGTCCCAGGATGAATAGCTTCAGCTAGTTTTCCAACTTGCGCAAGACCATCCGCTTTGTAATTATCGCCGAGTCCATGCCAG
ATAATCAAGGGAAGTGGAGTATCAT

>Bcin16g03460(MLST7), partial sequence [organism=Botrytis cinerea, strain U10_SC_BRO1]
TCTTCATGAACCTCTTGATTTGAACTCTTCATTAATTGCAAGTGATGAACCCCTATTTCTGCCCAGAGGATAGTTACAAGACGTATATCATTAG
TCGAGAACCACTCATGATATATATTGACGGGTTTTTGAAAGCGAATGAAAGTAAACATTTGGTTGATGTTAGGTGTGTTATTTATTCTGATGAA
ATGAACAAGAGAGACTGATGAGATAGTGAGCCGCTTTATGAACCGTCAACTGTTTCTCACGGACAGGAAGTTACCATTGATCCTTCAGTTCGGA
ATTCTGAAGTGGCGGTTTTAGAGAGGGATGAGGTGGTCAGGTGTATTGAGCATAGAGCGAGGGCATTTCAGGGGTGGAGGGGCGAGATGGGGAT
TGAGAAGTTGAGGACGCAGAGGTATGGGGTTGGAGGACATTATGGGATGCATTTGTAAGTTTTGGGGGATTGACGAAGGCTTCTGTCTATTTTC
TATCAGCACGGATCTTGAAAGAAAGATAGCATGAACACGCGGGCTAATAACTAGGAAATAGCGATTGGAGCGGAGGTAAACGTGGCATAGACCG
ATTTAGTACTTTCATGGTCTATGTCGACGTATCCTCTGATATCGAAGGTGGAGGAACGGAATTCCCACGTATTGTGGGACCAAAAGGAGGAAGG
TGGGAGGACTTCCTGGAAACTACGGAAGCATTGGATCCAAGAACTGGAGAAAATGTAACAGTAGAAGGGGTGACATTCAAACCAATCAAGGGAA
ATGCCGTTTCT

>Bcin12g03020(MLST8), partial sequence [organism=Botrytis cinerea, strain U10_SC_BRO1]
ACTGCGCAGCACCGAGTCACCAAAAGCACGAATAAACCATCGGCAAGGGAAATATTCTCTGAACCACAACATGACACGAGCGCGGAAGAGTATA
TCGGGCGAGAAGCTTCATCAAGAGCACCAAAGCGACAACGAGTCGATGATAATTATAACTCTTACGGTGGAAGAAATGAAAATACAGCAGCTTA
TGCTTCCGGGAAACTTCCATCTGGAAGTATAAATGTTGGTGGAGGTAGGAAGACCACTTTTCAAGAAGAACCTCGAACGGCATTTGTCGCTGGC
AAGTTGCCCCCTGGAAGTATCAACATTGGTGGAAAGAAGGCTACCCAAATCGAGGACGAGGGTAGAGCAGCTTATGCTTCCGGAAAATTGCCCC

```
CAGGAAGTATAAACTCTGGCGCAAAGAAGGCGATTTCATTCCAAGATGAAACGAGACCGGCTTATGTCTCCGGAAAGCTTCCACATGGTAGTAT
CGACGGTATGCGAAACCGTGAAATGGCTGCCGTCCACCGCGAAATTGCTGAGGGTGGGAGGAAACCAGGCCAGGTTGTCTCTTCTCTATTCACA
TTCAATCCTACTTCAAAGAAAACTTTCGATGAACCAGAAGAACAAGCGGAACCGGCAAAACCATCCAATGCGCCTTTGACAGAGGAAATGGCGA
CTTTCACCAATCTAGGGCTATCGAGAAGGCTTGCAGCCCATCTATCGACTAAACTCGATATGAAAGCCCCGACGGCTATTCAAAAAGCATCTGT
GCAGCAGTTGGTATCGGACGATAGCGATGCTTTCATACAAGCAGAGACCGGATCTGGAAAAACTTTGGCATATCTACTACCTATAGTCGAGCGA
ATATTAGCATTGAGTGAGAATGGCGTACA

>Bcin02g07770(MLST9), partial sequence  [organism=Botrytis cinerea, strain U10_SC_BRO1]
CGATTCCGATGCCGTTGTAGGATTTGCTGAAACTGTTCCCAGTGGGACCGTAGGAACAGTTTATGAGGCATATAAACCATTCCTTAAAGTCGTA
AATGGATGCGTACCATTCCCTGCCGTCGATGCATCGGGTAACACAGGGTATGTCCTTATATCTTTCTCTTCCACACGATTGCTATTGAGTCTCT
AACATATTTTAGTGGTGGTTTGTCACCAACTGGCAGTAGCAATGGTGGTTGCAGCAGCAGTACCGGTCAAGTATATGTTCGAGGAGGACAAAGC
GGATCAAACTACGCCATCATGTACTCCTGGTAAGTTCTCTCTAAACTTCTCCTTATAGATCCAACCTAACAAAATCTTAGGTACATGCCAAAGG
ACGAGCCCTCAACCGGTATTGGTCACCGTCACGATTGGGAAGGTGTAATTGTCTGGCTCTCCAGCGCCACCGCCACAACTGCCGACAACATCTT
AGCCGTTTGTCCTTCCGCCCACGGAGGCTGGGATTGTTCCACGGATGGCTATTCCCTTTCTGGTACCAGCCCTCTTATCAAGTACGAAAGTATC
TGGCCCGTCGATCACTCAATGGGTCTTACTAGTACTGTTGGTGGAAAACAACCTATGATTGCTTGGGAGTCTTTACCAACTGCTGCTCAAACTG
CTCTTGAGAACACCGATTTCGGTGCTGCGAATGTTCCATTCATTCCGGCTGTTTTCACAGACAATCTTGCGAAGGCTACTTTCTAG

>Bcin04g02090(MLST10), partial sequence [organism=Botrytis cinerea, strain U10_SC_BRO1]
AACTGTCGAATATGACTATGAATGATGTTTACAAGCCCTACATCCATGTAAGAAATGTAGAATAGAGAGATCAGTAACTGGAACTAATATCGTT
TGTAGGCTTTCAAGTTACTTACGCAGTTCAACCCAATCACTACAGCTATTGCCGAATCCCCACTGTTTCAAATGGCCGTCTCAGCAAATACCAT
CGAAAAGTACACACTGCTAGGCCCTTTCTTCAGAATATCTCCTCTGCAACAGGAAGTTACCAGGGAATACTTCAGTGCGCCAAAGACGATAGAT
AGGCGACACATTGCCACATCTCAAGATGCGTTACGATTGACCTTACAAACCCATCAAAAAGATTTACTTGATATCATCAACCACTTTGTTCGAG
CAAGTCCAATCGCAAAAAGCAAAACCCTGGATTGGTTCGCCTACATTGTGAATCAAAATCACAAGCGTCGAGCACTTCAGGTAGACCCGAAAGA
AGTGTCTTCTGATGGCTTTATGCACAATGTCACTGTCGTTCTAGATGGTCTTTGTGAGCCATTCATGGATACCACATTCTCGAAAATTTCGAAG
ATTGATATTGATTATCTAAGACGTGCGCCTCGTGTAGATATCAAGGACGAGACCAAGTTGAACGCTGACGAGAAGGCTTCTGAGAAGTATTATG
AGGACACTGTTCCTGGCACTTCTAATTTCATCTCTGAGGTCTTCTTTCTCACATTGG

>Bcin01g07220(MLST1), partial sequence  [organism=Botrytis cinerea, strain U11_M_E1]
GGTGTTGAAGTATCTGGCGGATAGAGACCAATGAGCAGAGCAGCTATTGTAGATTTTCCGGAGCCGGAAGGTCCGACAAGTGCAGTTGTTGAGT
TAGGAATCAGGGAAAGAGAAAAGGATGATATCGTTCGTTTTTCGGGACGAGTAGGATATGTGAATGAGAGACGGTTGAATTTGATTGAAAAGAT
TGTCGAAAGCCGTTCGGTTCCTTTATTTTCGTGGGAGGAAGATGAATCGAGATTGGCTAATGCAAGCATATGCGTAGCTGTAACGCGAGAAGAG
TTGATTTGTGGTATCATGGCCAGCATATTCGTACTATTAGAAATACCAAATAATAGAAGATTGACAGTCTGTAGTGCAGTCGCGATACTGATCT
CTCTCTTGGTAATGAGAACCGTGGCATAATAAAAGATAGTTGCAGTGATGAAGAATGACATCGCATCTGTCAATCCCCACAGCAATCCCGAGTA
GTTTGCTCGTATTAGTCCAACTTTATAAAGTTCTTCTGTAGCTTTGGTGTGTTTTGTCTCAAAGTAAGTTTCCAGAGTAAAAGCCCGAACCACT
TTGATGTTGGAGAAAGTCTCTGAAAATATGCCAGTGGTCATTTCAGAGGCGTAGTTGCACTTGTTTTCCCATTTTCCACTCACGCGATTGAACG
TTTTGGTGACAGCGTATATAACTGGCCCAGTTGCCATCGAGACTAATGTCAGTTTCCATGTATTCACGAAAGCCCAAATGATTGATATCAATAG
CATAAAAAACGCTGTGAATACAATACCAGCAAAGCGGCCAACGAGATTACGCATTTCCTCAGAGTTCCTATCCAAAACTTCGT

>Bcin05g07690(MLST2), partial sequence [organism=Botrytis cinerea, strain U11_M_E1]
AGGATGCCTTCTCTCTACGACAGCAAGCCTTCGATGATATGTTGTGGGTAGTTCTTGGCTGGCTGGATACTGTCAAATTCATTGATTTACATTC
TGAATTGCACTATTCAAACGACTCTCAGCCAGAATGGTACGGACAACAATATAAGCCTGCATTTGCACATCGAGCGCGACTATTTTGGGAATTG
GCTTCACAAGGATGGGATACTACTCTCTGTGGTGGTGGGATGATATGGTCACCATACCTTACTCCATACAAGAATGCAATTACCAATGAACTCT
ATATCGCAGCTTCGATATCGATGTACCTCTATTTCCCCGGAGATGACAATCAATCCCCATTTATGCTTTCCAACCCTTCATATCCACCTCACGA
TCCGAAATATCTACAGGCAGCTGTTGATGCTTACAAATGGCTGAATGGTTCCAACATGACGGATTTACAAGGATTATATGTCGACGGGTATCAT
ATCTCGAATCTTTCTGGCGGTGAAAACACCCATTGCGATTCTAGAAATGAGATGGTATATACCTACAATCAAGGTGTTTTGCTTACTGGACAAC
GTGGTTTGTATGACGCAACCGCCGCACGATCATACCTTGTGGATGGCCACAAACTCATCGCGAATGTTATTAATGCCACAGGCTATGACCTGAA
ACACAATGTTGTCATCTCACCGCCACCCAAAGATGGTTCCGCATTGGCAAAGTGGTTTGGCCTGGGTAGGAATGGAATACTGGAAGAAGGATGC
GATTCAAGTGCTTCGTGTTCTCAAAATGGACAAACTTTCAAAGGCATATTCTTTCATCACTTGATTGCATTCTGTAGTGATTTGCCAGGGGAGC
CTATTGCAGGGACGAAAGAA

>Bcin06g01710(MLST3), partial sequence [organism=Botrytis cinerea, strain U11_M_E1]
AGCTTCGGAAACGTCTTCCGAGAGGAATTTGCCAGTCCCGATAGTCGGTTACGAGTCCACTCTCTGAAAACTCGTTGCGAAGATAACTTGGTGT
TATGGAGAGTGCATCGATCAGATCTTTGCGTTTCTTGACATATAGGCAAGAAGCGTCGAAATTTGTCAGAAGCCATTTGTGCATGTTCATATCA
AAGGAATGGAAATGCTGGAAGGACGATGTTAGATGATGGTATTCAGGGCAAACCAAAGCTGCACCTGCATAAGCAGCATCGACGTGAACCCAGA
TCTCGCCTGCAACATCTGGAGGTGCATATTTTGAAAGTACTGTTGCAATAGATGCGAAGTCGTCAACTGCGCATGTAGATGTTGTTCCCAACGT
CGAAGTTAGATAGAAGGGTTCCAATCCTTGAGATTTGCATTCTTTCAATACCTTCTCTAAATCATCACCCGTCATGGCGAAATCATTGGATGCG
AGTACTGGAATCGATCGGAATCTAACGCCAGCTATCTGCGCTGCTTTCTGCGTGGAGCTGTGTGCCATTTCGCTTCCTAGTGCAACTAGCTTAC
```

TCCTCTTATATGCAATCGCATCCTCGAGTTCAATTCCCGACAGACCTTCAGTGGTTTCACGAAGATACTTATCGCGGCAGCAACCATAACGGT
AACGATAGCTTCCGAGGCTGATCCTTGGATAACACCACCACCATGAGTCGACGACAAATAACAGTCTGGGAGATTGAGAAGCTTGGCCAACCAA
TCCATTACAACCGTCTCCAATTCTGTCACAGCAGGGGAACAGATCCAATTGAAAGCAGGAGCTGTGAAAGCTGCTGAGTATAATTCTCCCAGCA
TTCCAGGGTAGGTAGATGATGCAGGGAAGAACGCCATGAAATTAGGTGATTGCCTTG

>Bcin09g03030(MLST4), partial sequence [organism=Botrytis cinerea, strain U11_M_E1]
GCAGAGAGAGTCTTGGAGGAGGTCGCCAAGAGTTGGAAGAATAGGAGTGGCGGTGTCATTGTCGAGGGCGAGGGAACGGAATTGAAGGAGATTC
TGAAAGCTTTGGAAGGGAATATGAGTGGTGGAAGGATAGTCATAGGAAGAGAGCTAAGCCGGCAGAATAGTTTAGTACTGGGATCATCACAATA
TGGAGAGGTCAATCATACAAGACTTGGGCTACGGCCAGGGAATATACCCAGAGAGGATAGTCAGTCAAGTTTGGGAATGTCAACGTTGGAGGTC
AATGACGAGGAAGATGAGGATGGCCTGATGGATCCAAGAAGGTGGTTAAAAGTCGTTGATGCATTTGAGCAACCTCGACTGGTGTACAATGTTG
CTAAAAAGCACTTTGATAGGTATGTTTCAATGATAAAATTTTATCTGAATCGACTAACTCAGTACAGAGATACCTCCAAACCTTCATTGTTCCC
ACCTGCGTCTCATAAAACACTCCTCTTCCAAAACCGCTATAATGTTATCCATCAACGTCTCCTTCGCAATGAATCTTTTCAAACGCCCGCTTTT
CAAGGTGGCAAATCTTCCCTTCAACGCAGCACGTCCGCCATTACCACCCAACAACAATCATACAAATTAACGCCGATAGCTAATCTTCTCGGTC
GCAATCGCAGCTCTCATATGCTTCTCGGTCTCCTCAGTATTTCACCCACTGGTACCCTCGCCATCAATGACCTG

>Bcin11g01310(MLST5), partial sequence [organism=Botrytis cinerea, strain U11_M_E1]
TCATGTGGAACCGGCGTAGAATGCGCAACGCTTGAAGTTCCGCTCGAATATGGCGATGCAACGTCAACGGCAAAAGCCAGTGTTGCGCTTGCTC
GTTATCCTGCCACTGTTGCCGCGAGCAAGAAGCTCGGGTCTCTCTTGATAAATCCCGGTGGACCCGGTGCCTCTGGTGTTGGCTTTGTGCAGTC
TGGAGCCGGTGCCGCCGTCTCGACACTGAGTGGTGGATTATACGATATCATCGGATGGGATCCACGTGGAACCGGTGCTTCGGCTCCTATTTTG
GAATGTTTTGCAAATGCCAGTGCGGAGTATGATTTTAACAACGCGTTTCCATCTGCTCCGAATCTCTGGCTCGGACAATTTGCGAATGCCAGCG
CAAATTCTGCTGTTAGCTCTGCTATCACATCCTTTGACACTTCTGTCGCTGCTCTTGCAAAAGCTTGCGTGGCTCAGAAATCTCCCGCTCTTTA
CACCTCAACAGCAGCATATGTTGCTCGAGACATGGCAGCGATAGTCGATGCATTGGATGGGACCTCTGCAAAACTTAACTACTGGGGTTTCTCA
TATGGAACTATCTTCCTAGCTGAGTTTATCCAAACTTTCCCAGGCCGCGTGGGAAGAGTTCTTGCCGATGGTGTTTTCGACGCAAAGGCAAATG
CACTCACATACGTTAGCCAACTTCCCAACGATCAACTCAGTGTTCGTGCTTCGTTGAACGATTTTGCAGCTTTCTGCACCACCGCCGGTAGTAA
AGGTTGCTCTTTTGCCACCGCCCCTACTGGAACCTCAGGTACTGTTGCTACCAGACTGGACAACATAATGAAGGATATG

>Bcin15g03910(MLST6), partial sequence [organism=Botrytis cinerea, strain U11_M_E1]
TCTTTGAAAGCCTTCTCTAGCATCTCCTCTCCTAACGTCATATGTCCCCGTGGAATGGTTTCGAAAACTAATTTTCCGGCCTCATCCCAATGTC
TTTAACCCTAGCCAATCTTCTTTATAAATGGCTCTTTCTTTCAGTGGTGTAACTTCCGTGCCGTTGACTTCAGCCCACCATCCACTTTCCTTAG
GGATGACAGTTGTATCGTCTTCAAAGACATACATTACGAATCGTTCCAATTTTTCCATGTTGGATTTATATGTTTGGTTCTTGAGAACGCGCTC
ATTATTGATGTCGGCAAGGAAATTGGAATATTCAAGGTAAGAGTCTAGGTTTTCCGGATCTCTGAAGTATTGAGCGGGTACGAGACGAGATTGG
ACAAAGGTTGACCAGGTGTTGGATCGCAAAAGGGTTTGAGCACCGCGACAGAGGAAATCGGCAGGACCACAGGATTGGAAGGCAGAAATGCCGT
TGTGTTGGGAACCGAAGGTCAGGAGAGAGCGGATGGGTGGAGCATTGCAGCGGGATATGTAACCGCGCAAGAATTGGCCGCCTTGGGAGAATCC
AATTGCGTCGACGGCGGGCGCGGTAGAGAGAATAGGATGGGAGGCGAGGTCTTCGCAGACCTTTTCGATTTGAACTTGGTAAGAGAGTTAGTAT
GGTATTTAAAGGAAAAATAGGGGATGTACTCACGAGTGAGATTTCCAAAGAAGGTAGCTGTCCTATCTGCAGATGCATCCTCATCTACATGAAT
ATTATAGACAAAAGTCCCAGGATGAATAGCTTCAGCTAGTTTTCCAACTTGCGCAAGACCATCCGCTTTGTAATTATCGCCGAGTCCATGCCAG
ATAATCAAGGGAAGTGGAGTATCAT

>Bcin16g03460(MLST7), partial sequence [organism=Botrytis cinerea, strain U11_M_E1]
TCTTCATGAACCTCTTAATTTGAACTCTTCATTAATTGCAAGTGATGAACCCCTATTTCTGCCCAGAGGATAGTTACAAGACGTATATCATTAG
TCGAGAACCACTCATGATATACATTGACGGATTTTTGAAAGCGAATGAAAGTAAACACTTGGTTGATGTTAGGTGTGTTATTTATTCTGATGAA
ATGAACAAGAGAGACTGATGAGATAGTGAACCGCTTTATGAACCGTCTACTGTTTCTCACGGACAGGAAGTTACCATTGATCCTTCGGTTCGAA
ATTCTGAAGTGGCGATTTTAGAGAGGGATGAGGTGGTCAGGTGTATTGAGCATAGAGCGAGGGCATTTCAGGGGTGGAGGGGCGAGATGGGGAT
TGAGAAGCTGAGGACGCAGAGGTATGGGGTTGGAGGACATTATGGGATGCATTTGTAAGTTTTGGGGGATTGACGAAGGCCTCTGTCTATTTTC
TATCAGTACGGATCTTGAAAGAAAGATAGCATGAACACGAGGGCTAATAACTAGGAAATAGCGATTGGAGCGGAGGTAAACGTGGCATAGACCG
ATTTAGTACTTTCATGGTCTATGTCGACGTATCCTCTGATATCGAAGGTGGAGGAACGGAATTCCCACGTATTGTGGGACCAAAAGGAGGAAGG
TGGGAGGACTTCCTGGAAACTACGGAAGCATTGGATCCAAGAACTGGAGAAAATGTAACAGTAGAAGGGGTGACATTCAAACCAATCAAGGGAA
ATGCCGTATTCT

>Bcin12g03020(MLST8), partial sequence [organism=Botrytis cinerea, strain U11_M_E1]
AGTCACCAAAAGCACGAATAAACCATCGGCAAGGGAAATATTCTCTGAACCACAACATGACACGAGCGCGGAAGAGTATATCGGGCGAGAAGCT
TCATCAAGAGCACCAAAGCGACAACGAGTCGATGATAATTATAACTCTTACGGTGGAAGAAATGAGAATACAGCAGCTTATGCTTCCGGGAAAC
TTCCATCTGGAAGTATAAATGTTGGTGGAGGTAGGAAGACCACTTTTCAAGAAGAACCTCGAACGGCATTTGTCGCTGGCAAGTTGCCCCCTGG
AAGTATCAACATTGGTGGAAAGAAGGCTACCCAAATCGAGGACGAGGGTAGAGCAGCTTATGCTTCCGGAAAATTGCCCCCAGGAAGTATAAAC
TCTGGCGCAAAGAAGGCGATTTCATTCCAAGATGAAACGAGACCGGCTTATGTCTCTGGAAAGCTTCCACATGGTAGTATCGACGGTATGCGAA
ACCGTGAAATGGCTGCCGTCCACCGCGAAATTGCTGAGGGTGGGAGGAAACCAGGCCAGGTTGTCTCTTCTCTATTCACATTCAATCCTACTTC
AAAGAAAACTTTCGATGAACCCGAAGAACAAGCGGAACCGGCAAAACCATCCAATGCACCTTTGACAGAGGAAATGGCGACTTTCACCAATCTA

GGGTTATCGAGAAGGCTTGCAGCCCATCTATCGACTAAACTCGATATGAAAGCCCCGACCGCCATTCAAAAAGCATCTGTGCAGCAGTTGGTAT
CGGACGATAGCGATGCTTTCATACAAGCAGAGACTGGATCTGGAAAAACTTTGGCATATCTACTACCTATAGTCGAGCGAATATTAGCATTGAG
TGAGAATGGCGT

>Bcin02g07770(MLST9), partial sequence [organism=Botrytis cinerea, strain U11_M_E1]
ACACGATTGCTATTGAGTCTCTAACATATTTTAGTGGTGGTTTGTCACCAACTGGCAGTAGCAATGGTGGTTGCAGCAGCAGTACCGGTCAAGT
ATATGTTCGAGGAGGACAAAGCGGATCAAACTACGCCATCATGTACTCCTGGTAAGTTCTCTCTAAACTTCTCCTTATAGATCCAACCTAACAA
AATCTTAGGTACATGCCAAAGGACGAGCCCTCAACCGGTATTGGTCACCGTCACGATTGGGAAGGTGTAATTGTCTGGCTCTCAAGCGCCACCG
CCACAACTGCCGACAACATCTTAGCCGTTTGTCCTTCCGCCCACGGAGGCTGGGATTGTTCCACCGATGGATATTCCCTTTCTGGTACCAGCCC
TCTTATCAAGTACGAAAGTATCTGGCCCGTCGATCATTCAATGGGTCTTACTAGTACTGTTGGTGGAAAACAACCTATGATTGCTTGGGAGTCT
TTACCAACTGCTGCTCAAACTGCTCTTGAGAACACCGATTTCGGTGCTGCGAATGTTCCATTCATTCCGGCTGTTTTCACAGATAATCTTGCGA
AGGCTACTTTCTAG

>Bcin04g02090(MLST10), partial sequence [organism=Botrytis cinerea, strain U11_M_E1]
AACTGTCGAATATGACTATGAATGATGTTTACAAGCCCTACATCCATGTAAGAAATGTAGAATAGAGAGATCAGTAACTGGAACTAATATCGTT
TGTAGGCTTTCAAGTTACTTACGCAGTTCAACCCAATCACTACAGCTATTGCCGAATCCCCACTATTTCAAATGGCTGTCTCAGCAAATACCAT
CGAAAAGTACACACTGCTAGGCCCTTTCTTCAGAATATCTCCTCTGCAACAGGAAGTTACCAGGGAATACTTCAGTGCGCCAAAGACGATAGAT
AGACGACACATTGCCACATCTCAAGATGCGTTACGATTAACCTTACAAACCCATCAAAAAGATTTACTTGATATCATCAACCACTTTGTTCGAG
CAAGTCCAATCGCAAAAAGCAAAACCCTGGATTGGTTCGCCTACATTGTGAATCAAAATCACAAACGTCGAGCACTTCAGGTAGACCCGAAAGA
AGTGTCTTCTGATGGCTTTATGCACAATGTCACTGTCGTTCTAGATGGTCTTTGTGAGCCATTCATGGATACCACATTCTCGAAAATTTCGAAG
ATTGATATTGATTATCTAAGACGTGCGCCTCGTGTAGATATCAAGGACGAGACCAAGTTGAACGCTGATGAGAAGGCTTCTGAGAAGTATTATG
AGGACACTGTTCCTGGCACTTCTAATTTCATCTCTGAGGTCTTCTTTCTGACATTGG

>Bcin01g07220(MLST1), partial sequence [organism=Botrytis paeoniae, strain Pae_14]
GGTGTTGAGGTATCTGGTGGATAAAGACCAATGAGGAGAGCAGCTATTGTAGATTTTCCGGAGCCGGAGGGTCCGACAAGTGCGGTTATTGAGT
TAGGGTTTAGGGAAAGAGAAAAGGATGATATCGTTCGTGTTTCAGGACGAGCAGGATATGTGAATGAGAGGCGTTTGAATTTGATTGGAAAAAT
TGTCGAAAGTCGTTCGGTTCCTTTATTTTCGTGGGACGAAGATGAATCGAGATTGGCTAATTCAAGCATATGCGTAGCTGTAACGCGAGAAGAG
TTAATTTGTGGTATCATGGCCAGCATATTCGTACTATTAGAAATACCGAATAGTAGAAGATTGACAGTCTGTAATGCAGTCGCGATACTAATCT
CTCTTTTGGTAGTGAGAACCGTGGCATAATAAAAGATAGTTGCAGTGATGAAGAATGACATCGCATCTGTCAGTCCCCAAAGTAGCCCCGAGTA
GTTTGCTCGTACTCGTCCAACTTTATAAAGCTCTTCTGTAGCTTTGGTGTGCTTCTTCTCGAAATAAGTTTCCAAAGTAAAAGCCCGAACCACT
TTGATGTTGGAGAAAGTCTCTGAAAATATGCCGGTGGTCATTTCAGAAGCGTAGTTGCATTTGTTTTCCCATTTTCCACTCACGCGATTGAATG
TTTTGGTGACAGCGTATATAACTGGCCCAGTTGCCATTGAGACTAGTGTGAGTTTCCATGTATTCACGAACGCCAAGATGATTGATATAAATAG
CATAAAGAATGCTGTAAATACAATACCAGCAAAGCGACCAACGAGATTACGCATTTCCTCAGAATTCCTATCCAAAACTTCG

>Bcin05g07690(MLST2), partial sequence [organism=Botrytis paeoniae, strain Pae_14]
TAGGATGCCTTCTCTCTACGACAGCAAGCCTATGATGATATGTTATGGGTAGTTCTTGGTTGGTTGGATACTGTCAAATTCATTGATTTGCACT
CTGAATTACACTATTCGAACGACTCTCAACCAGAATGGTACGGACAACAATACAAACCTGCATTTGCCCACCGGGCACGACTATTTTGGGAATT
GGCATCGCAAGGATGGGATACTACTCTCTGTGGTGGTGGGATGATATGGTCTCCATACCTTACTCCATACAAGAACGCAATCACCAATGAACTC
TACATCGCAGCTTCGATATCGATGTATCTATATTTCCCTGGAGATGACAATCAATCTCCATTTATTCTTTCCAACCCTACATATTCACCTCGCG
ATCCGAAATATCTACAGGCAGCTGTTGATGCTTACAAATGGCTGAACGGTTCAAATATGACGGATTTGCAAGGATTGTATGTCGACGGATATCA
TATCTCGAATCTTTCTGGCGGTGACAATACCCATTGCGATTCTAGAAATGAGATGGTATATACCTACAATCAAGGCGTTTTACTTACTGGACAA
CGTGGTTTGTATGACGCAACCGCCGCAAGATCATACCTTGTGGATGGCCATAAACTTATCGCGAATGTTATTAATGCCACAGGCTACGATCTTA
AAGACAACGTTGTCATCTCACCGCCACCCAAAGATGGGTCCGCATTGGCAAAATGGTTTGGTCTTGGTAGAAATGGAATACTGGAAGAAGGTTG
CGATTCAAGTGCTTCGTGTTCTCAAAATGGACAAACTTTCAAAGGCATATTCTTTCATCATTTGATTGCATTCTGTAGTGATTTGCCACGGGAG
CCTATTGCAGGGACGAAAGA

>Bcin06g01710(MLST3), partial sequence [organism=Botrytis paeoniae, strain Pae_14]
GCTTCGGAAGCGTCTACCAAGAGAGATTTGCCAGTCCCGATAGTCGGTTACGAGTCCACTTTCTGAGAACTCGTTGCGAAGATAACTTGGTGTT
ATGGAGAGTGCGTCGATCAAATCTTTGCGTTTCTTGACATATAGACATGATGCGTCGAAATTTGTCAGAAGCCATTTGTGCATGTTCATATCGA
ACGAATGGAAATGTTGGAAGGACGATGTTAAATGATGGTATTCGGGGCAAACTAAAGCTGCACCTGCATAAGCAGCATCAACGTGAACCCAGAT
CTCGCCTGCAACATCTGGACGTGCGTATTTTGAAATTATTGACGCGATAGATGCGAAATCGTCAACTGCGCATGTGGATGTTGTTCCCAAAGTC
GAAGTTAGATAGAAGGGTTCCAATCCTTGAGATTTGCATTCTTTCAATACTTTCTCTAGATCATCACCCGTCATGGCGAAATCGTTGGATGCGA
GTACTGGAATGGATCGAAACCTAACACCGGCTATTTGCGCTGCTTTCTGTGTGGAGCTGTGTGCCATTTCGCTTCCTAAGGCAACTAGCTTACT
CCTCTTGTATGCAATCGCATCTTCGAGTTCAACCCCTGATAAACCCGCAGTAGTTTCACGTAGATATTTATCGCGAGCAGCAACCATGACGGTA
ACTATAGCTTCTGAAGCCGATCCTTGGATAACACCACCACCATGAGTCGTTGACAAATAACAGTCTGGGAGATTAAGAAGCTTGGCCAGCCAGT
CCATTACAACAGTCTCCAATTCTGTCACAGCAGGGGAACAGATCCAATTGAAAGCAGGAGCAGTAAAAGCTGCTGAGTATAACTCTCCGAGCAT
TCCAGGATAGGTAGATGATGCGGGGAAGAAAGCCATAAAATTAGGTGATTGCCTTG

>Bcin09g03030(MLST4), partial sequence [organism=Botrytis paeoniae, strain Pae_14]
AAGAAGGACTGGCAGAAAGAGTTCTAGAGGAGGTTGCAAAGAGTTGGAAGAATAGGAGTGGCGGTGTTATTGTCGATGGAGAGGGGACGGAATT
GAAGGAGATTCTGAAAGCTTTGGAAGGGAATATGAGTGGTGGGAGGATAGTCGTAGGACGAGAATTAAGCCGGCAGAACAGTTTAGTATTGGGA
TCATCACAATATGGAGAAGTCAATCATACCAGGCTTGGACTACGGCCAGGGAATATACCTAGAGAGGATAGTCAGTCAAGTTTGGGAATGTCAA
CGTTGGAGGTGAATGACGAGGAAGATGAGGATGGCCTGATGGATCCAAGGAAGTGGTTGAAAGTCATCGATGCTTTTGAGCAACCTCGATTGGT
GTATAATGTTGCTAAGAAGCACTTTGATAGGTATGTTTCAATGATATTCTTTCATCTGAATCGACTAATGCATTACAGAGATACCTCCAAACCT
TCATTATTCCCACCTGCGTCCCATAAAACACTCCTCTTCCAAAACCGCTATAATGTTATCCATCAACGTCTTCTTCGCAATGAGTCTTTTCAAA
CGCCCGCTTTTCAAGGTGGCAAAGCTTCTCTTCAACGTAGCACATCCGCCATTACCACCCAGCAACAATCATACAAATTAACGCCGATAGCTAA
TCTTCTCGGCCGCAATCGCAGCACCCATATGCTTCTCGGCCTCCTCAGCATTTCGCCCACTGGTACCCTCGCCATTAATGACCTG

>Bcin11g01310(MLST5), partial sequence [organism=Botrytis paeoniae, strain Pae_14]
TCATGTGGCACCGGTGTAGAATGCGCAACCCTTGACGTTCCGCTCGAATATGGCGATTCGAAGTCAACAGCGAAAGCAAATGTCGCGCTTGCTC
GTTATCCTGCCACTGTAGCAGCGAGCAAGAAGCTCGGGTCTCTCTTGATCAACCCCGGTGGACCCGGTGCCAGTGGTGTTGGATTTGTGCAGTC
CGGGGCCGGTGCCGCCGTCTCGGCACTTAGCGGTGGATTGTACGATGTCATTGGATGGGATCCGCGTGGAACTGGTGCTTCGGCTCCTATCTTG
GAATGCTTTCCAAATGCCAGTGCGGAGTATGATTTCAACAACGCATTTCCATCCGCTCCAAATCTTTGGCTTGGACAATTTGCGAATGCTAGCG
CCGATGCTGCTGTTAGCTCTGCTATCACATCTTTTGACACCTCTGTCGCTGCTCTTGCAAAAGCTTGTGTAGCTCAGAAATCTCCCGCTCTTTA
CACCTCAACAGCGGCATATGTTGTCCGAGACATGGCAGCGATAGTTGATGCATTGGATGGAACTTCTGCAAAACTCAACTACTGGGGTTTCTCA
TATGGAACTATTTTCCTCGCCGAGTTTATCCAAACCTTTCCAGGCCGCGTAGGAAGAGTTATTGCTGATGGTGTCTTTGACGCAAAGGCAAACG
CCCTTACATACGTCAGCCAACTACCAAACGATCAAGTCAGTGTTCGTGCTTCGTTGAACGATTTTGCAGCTTTCTGCACCACCGCCGGCAGTAA
AGGATGCTCTTTTGCCACCGCACCTACTG

>Bcin15g03910(MLST6), partial sequence [organism=Botrytis paeoniae, strain Pae_14]
TTTTCTAGCATCTCCTCTCCTAATGTCATATGACCCCCTGGAATGGTTTCGAAAACCAATTTTCCGGCTTCATCCAATGTCTTCAAACCTACCC
AATCTTCTTTATAAATGGCTCTTTCTTTCAGTGGTGTAACTTCCGTGCCGTTGACTTCAGCCCACCATCCACTTTCCTTGGGAATGACGGTTGT
ATCGTCTTCGAAGACGTACATGACGAATCGTTCCAATCTTTCCATGTTTGATTTATATGTTTCATTCTTGAGAACACGCTCATTATTGATATCG
GCGAGGAAATTAGAAAATTCAAGGTAAGATTCTAGATTTTCGGGATCTCGGAAGTATTGAGCGGGTACGAGACGAGATTGGACAAATGTTGACC
AGGTGTTGGAACGTAGGAGGGTTTGAGCACCCCGACAGAGAAAATCGGTAGGACCACAGGCTTGGAAGGAAGAAATGCCGTTATGTTGGGAACC
GAATGTCAGGAGGGAGCGGATGGGTGGAGAATTGCAGCGGGATATGTAGCCGCGCAAGAATTGGCCTCCTTGGGAGAATCCGATTGCGTCGACG
GCGGGCGCGGTAGAGAGAATAGGATGGGAGGCTAGGTCTTCGCAGACCTTTTCGATTTGAACTTGGTGAGAGAGTTAGTATGGTGTTTAAAGGA
AAGCTAGAGGATGTACTCACGAGTGAGATTTCCAAAGAAAGTAGCCGTCCTATCTGCAGATGCATCATCATCTACATGAATATTATAGACAAAA
GTCCCGGGGTGAATAGCTTCAGCTAATTCTCCAATTTGCGCAAGACCATCCGCTTTGTAGTTATCACCCAGTCCATGCCAGATAATCAAGGGTA
GTGGAGTATCAT

>Bcin16g03460(MLST7), partial sequence [organism=Botrytis paeoniae, strain Pae_14]
TCTTCATGAACCTCTCAATTTGAACTCTTCATTTATCGCAAGTGATGAACCCCTATTTTGTCCGGAGGATAGCTATAAGACGTTCATTATTAGT
CGAGAACCACTTATGATATATATTGAAGGGTTTTTGAAAGCGAATGAGAGTAAACATTTGGTGGATGTTAGGTATGTTGGTTCTTCTGATGGGA
TGAAGAGGAGAAACTGATGGGATAGTGAACCGCTTTATAAACCGTCTACGGTTTCTCATGGTCAGGAAGTTACAGTTGATACTTCGGTTCGAGA
TTCTGAGGTAGCAGTACTAGAGAGAGAGATGAGGTGGTTAGATGTATTGAGCATAGAGCGAGAGCATTTCAGGGATGGAGAGGTGAGATGGGGATT
GAGAAGTTGAGGACGCAGAGGTATGGGGTGGGGGGACATTATGGGATGCATTTGTAAGTTTTGGGGGATTGACGAAGGCTTTTGTTTATTTTCT
ATCAACATGGAGCTTTCCGAAGAGATATTGTGTACACGAGGGCTGATGATAGAAATAGCGATTGGAGCGGAGGCAAACGTGGCATAGACCGAAT
AAGTACGTTCATGGTCTACGTCGACGTATCCTCCGATATCAAAGGTGGAGGAACGGAATTTCCACGTATCGTGGGACCAAAAGGAGGAAGGTGG
GAGGAATTCCTCGATACTACCGAAGCATTAGACCCAAGAAGCGGAAAAAATGTAACGGTAGAAGGAGTGACATTCAAACCCATCAAGGGAAATG
CCGTATTCT

>Bcin12g03020(MLST8), partial sequence [organism=Botrytis paeoniae, strain Pae_14]
ACTGCGCAGCACCGAGTCACTAAGAGCACGCATAAACCATCGGCAAGAGAAATATTCTCTGAACCACAACATGATACCAGCGCGGAAGAGTATA
TTGGGCGAGGAGCTTCATCAAGAGCACCAAAGCGACAACGAGTCGATGATAATTACAACTCTTACGGCGGAAGAAATGAAAATACAGCAGCTTA
TGCTTCCGGAAAGCTTCCATCTGGAACTATAAATGTTGGAGGAGGTAGAAAGACCACTTTTCAAGAAGAACCTAGAACAGCATTTGTCGCTGGC
AAGTTGCCTCCCGGAAGTATAAATATTGGTGGAAAGAAGGCTACCCAAATCGAGGACGAGGGTAGAGCAGCTTATGCTTCCGGGAAATTGCCTC
CAGGAAGTATAAATTCTGGCGCAAAGAAGGTTATTTCATTCCAGGATGAAACGAGACCGGCTTATGTCTCCGGAAAGCTCCCACATGGTAGTAT
CGAAGGTATGCGAAACCGTGAAATGGCTGCTGTCCACCGCGAAATTGCTGAGGGTGGGAGGAAACCAGGCCAGGTTGTCTCGTCTCTATTCACA
TTCAATCCCACTTCAAAGAAAACTTTCGATGAACCTGAAGAACAAGCGGAACCGGCAAAACCATCCAACGCGCCTTTGACAGAGGAAATGGCGA
CATTCACCAATCTAGGACTATCGAGAAGGCTTGCAGCCCATCTATCGACTAAACTCGATATGAAAGCTCCAACCGCCATCCAAAAGGCATCCGT
ACAGCAGCTGATATCAGACGATAGCGATGCCTTCATACAAGCAGAGACTGGATCTGGAAAAACCTTGGCATATCTACTACCTATAGTTGAGCGG
ATATTGGCATTGAGTGAGAATGGCGTACA

>Bcin02g07770(MLST9), partial sequence [organism=Botrytis paeoniae, strain Pae_14]
ATCTACAGTCGTTGCCATCCCTACACCATCACAGCTTGAGTCTCGGGCCGTTATCAGTTCCGATGCCGTTGTAGGATTTCCCGAAACTGTTCCT
AGCGGGACAGTAGGAACAGTCTATGAGACATATCAACCATATCTTGATGTAGTCAATGGATGCGTACCATTCCCTGCCGTCGATGCATCGGGTA
ACACGGGGTATGTCCTTACATTTTTATCCACATGATTCCTATTGAGTCTAACATATTTTAGTGGTGGTTTGGCACCAACTGGCAGTAGCAACGG
TGGTTGCAGCAGTAGTACTGGTCAAGTATACGTCCGAGGAGCACAAAGCGGATCATACTACGGTATCATGTACTCCTGGTAAGTGCTCTCTAAT
CACCTCCTTACAGATCCAAACTAATAAAATCTCAGGTACATGCCAAAAGACGAGCCCTCAACCGGTATTGGTCACCGTCACGACTGGGAAGGTG
TAATCGTCTGGCTCTCCAGCTCAACCGCCACAACCGCCGACAACATCGTAGCCGTGTGTCCTTCCGCCCATGGAGGCTGGGATTGTTCCACCGA
TGGTTATTCCCTTTCTGGTACCAGTCCTCTCATCAAGTACGAAAGTATCTGGCCTATCGATCACTCAATGGGTCTTACTAGTACTGTTGGTGGA
CAACAACCTATGATTGCTTGGGAATCTTTACCTACTGCTGCTCAAACTGCTCTTGAGACGACTGATTTTGGTTCTGCGAATGTTCCATTCATTC
CATCTGTTTTC

>Bcin04g02090(MLST10), partial sequence [organism=Botrytis paeoniae, strain Pae_14]
AACTCTCAATATGACTATGAATGATGTTTACAAGCCCTACATTCATGTAAGAAATGTAGAATGGAGAGATCAATAACTGGAACTAATGTCGTTT
GTAGGCTTTCAAATTGCTTACGCAGTTCAACCCAATCACTACAGCTATTGCCGAATCTCCACTATTCCAAATGGCTGTTTCAGCAAATACTATC
GAAAAGTACACACTACTTGGCCCTTTCTTCAGAATATCTCCTTTGCAACAAGAAGTTACCAGGGAATACTTCGGTGCGCCAAAGACGATAGATA
GGCGACATATTGCCACATCTCAAGATGCGTTACGATTGACCTTACAAACCCATCAAAAAGATTTACTTGATATCATCAACCACTTTGTTCGAGC
AAGTCCAATAGCTAAAAGCAAAACCTTGGATTGGTTTGCCTACATCGTAAATCAAAATCACAAGCGTCGAGCACTTCAGGTGGATCCAAAAGAA
GTGTCTTCTGATGGCTTTATGCATAACGTCACTGTTGTTTTAGATGGTCTTTGCGAGCCATTCATGGATACCACATTTTCGAAGATCTCGAAGA
TTGATATCGATTATCTACGACGTGCGCCTCGTGTTGATATTAAGGACGAGACCAAGTTGAACGCTGACGAGAAGGCTTCCGAAAAGTATTATGA
GGCCACTGTTGATGGCACTTCTAATTTCATCTCCGAGGTCTTCTTTCTGACGCTGG

>Bcin01g07220(MLST1), partial sequence [organism=Botrytis sinoviticola, strain GB5_5]
GTGTTGAAGCATCTGGCGGATAAAGACCAATGAGCAGAGCAGCTATTGTAGATTTTCCAGAGCCGGAGGGTCCGACAAGTGCAGTTGTTGAGTT
AGGACTCAGGGAAAGAGAAAAGGATGATATCGTTCGTTTTTCAGGTCGAGCAGGGTATGTAAACAAGAGGCGGTCGAATTTGATTGGAAAAATT
GTTGAAAGTCGTTCGGTTCCTTTATTTTCGTGGGAGGAAGATGAATCAAGATTGGCTAATGCAAGCATACGCGTCGCTGTAACGCGAGAAGAGT
TGATTTGTGGTATCATGGCCAGCATGTTCGTACTATTAGATATACCGAATAGTAGAAGATTGACAGTCTGTAGTGCAGTCGCGATACTGATCTC
TCTTTTGGTAATGAGAACCGTGGCATAATAAAAGATAGTTGCAGTGATGAAGAAGGACATCGCATCTGTCAATCCCCACAGTAATCCCGAGTAA
TTTGCTCGTATTAGTCCAACTTTATAAAGTTCTTCTGTAGCTTTGGTGTGTTTTGTCTCAAAATAAGTTTCCAGAGTAAAAGCCCGAACCACTT
TGATGTTGGAGAAAGTCTCTGAAAATATGCCGGTGGTCATTTCAGAGGCGTAGTTGCACTTGTTTTCCCATTTTCCACTCACACGATTAAAAGT
TTTGGTAACAGCGTATATGACTGGCCCAGTTGCCATTGAGACTAATGTTAGTTTCCATGTATTCACGAAAGCCCATATGATTGATATCAATAGC
ATGAAAAATGCCGTGAACACGATACCAGCGAAGCGACCAACGAGATTACGCATTTCCTCGGAGTTCCTATCCAAAACTTCGT

>Bcin05g07690(MLST2), partial sequence [organism=Botrytis sinoviticola, strain GB5_5]
AGGATGCCTTCTCTCTACGACAGCAAGCCTATGATGATATGTTATGGGTAGTTCTTGGTTGGCTGGATACTGTCAAATTCATTGATTTACACTC
TGAATTACACTATTCGAACGATTCTCAACCAGAATGGTACGGACAACAATACAAACCTGCATTTGCACATCGAGCGCGACTATTTTGGGAATTG
GCATCACAAGGATGGGACACTACCCTCTGTGGAGGTGGGATGATATGGTCTCCATACCTTACCCCATACAAGAACGCAATTACCAATGAACTCT
ATATCGCAGCTTCGATCTCGATGTACCTCTATTTCCCTGGAGATGACAATCAATCCCCATTTATGCTTTCCAACCCTTCATATCCACCTCGCGA
TCCGAAATATCTACAAGCAGCTGTTGATGCTTACAGATGGCTGAACGGTTCCAACATGACGGATTTGCAGGGACTATATGTCGACGGATATCAT
ATCTCGAATCTTTCTGGCGGTGAAAACACGCATTGCGATTCTAGAAATGAGATGGTATATACCTACAATCAAGGTGTTTTACTTACTGGACAAC
GCGGTTTGTATGACGCAACCGCCGCACGATCATACCTTCTGGATGGCCACAAACTCATCGCGAATGTTGTTAATGCCACAGGCTATGATCTGAA
ACACAATGTTGTCATCTCACCGCCACCCAAAGATGGTTCCGCATTGGCAAAGTGGTTCGGTCTTGGTAGGAATGGAATACTGGAAGAAGGTTGC
GATTCAAGTGCTTCGTGTTCCCAAAATGGACAAACTTTCAAAGGCATATTCTTTCATCATTTGATTGCATTCTGTAGTGATTTGCCAAGGGAGC
CTCTTGCAGGGACGAAAGAAA

>Bcin06g01710(MLST3), partial sequence [organism=Botrytis sinoviticola, strain GB5_5]
TTTAAGCTTCGGAAGCGTCTTCCGAGAGGAATCTGCCAGTCCCGATAGTCGGTTACGAGTCCACTCTCTGAAAACTCGTTGCGAAGATAACTTG
GTGTTATGGAGAGTGCATCGATCAAATCTTTGCGTTTCTTGACATATAAACAAGAAGCGTCGAAATTTGTAAGAAGCCATTTGTGCATGTTCAT
ATCGAATGAATGGAAATGTTGGAAGGATGATGTTAGATGATGGTATTCAGGGCAAACCAAAGCTGCACCTGCATAGGCAGCATCGACATGAACC
CAGATCTCGCCTGCAACATCTGGAGGTGCATACTTTGAAAGTACTGTTGTGATAGATGCGAAGTCATCAACTGCGCATGTGGATGTTGTCCCCA
AAGTCGAAGTTAGATAGAAGGGTTCCAATCCTTGAGATTTGCATTCTTTCAATACCTTTTCTAAATCATCACCGGTCATGGCAAAATCATTAGA
TGCGAGCACTGGAACCGATCGGAATCTAACGCCAGCTATCTGCGCTGCTTTCTGCGTGGAGCTGTGTGCCATTTCGCTTCCTAGTGCAACTAGC
TTACTCCTCTTATATGCAATCGCATCCTCGAGTTCAATACCTGACAGACCTTCAGTAGTTTCACGAAGATATTTATCGCGGGCAGCGACCATTA
CGGTAACGATAGCTTCCGAAGCTGATCCTTGGATGACACCACCACCATGAGTCGAAGACAAATAACAGTCTGGGAGATTGAGAAGCTTGGCCAG
CCAATCCATTACAACCGTCTCCAATTCTGTCACAGCAGGGGAGCAGATCCAATTGAAAGCCGGAGCTGTGAAAGCTGCTGAGTATAATTCTCCA
AGCATTCCAGGGTAGGTAGATGATGCAGGGAAGAACGCCATGAAATTAGGTGATTGCCTTGG

>Bcin09g03030(MLST4), partial sequence [organism=Botrytis sinoviticola, strain GB5_5]

AAGAAGGACTGGCAGAGAGAGTTTTGGAGGAGGTTGCAAAGAGTTGGAAGAATCGGAGTGGCGGTGTTATTGTCGAGGGCGAGGGGACGGAATT
GAAGGAGATTCTGAAAGCTTTGGAAGGGAATATGAGTGGTGGAAGGATAGTCGTAGGAAGAGAATTAAGCCGGCAGAACAGTTTAGTACTGGGG
TCATCACAATATGGAGAGGTCAATCATACGAGACTTGGACTACGGCCTGGGAATATACCTAGAGAGGATAGTCAGTCAAGTTTGGGGATGTCAA
CATTGGAGGTTAATGACGAGGAAGATGAGGATGGCCTGCTGGATCCAAGGAAATGGTTAAAAGTCATTGATGCTTTCGAGCAACCTCGACTGGT
GTATAACGTTCCTAAGAAGCACTTTGATAGGTATGTTTCAATGATACCATTCCATCTGAATCGACTAATGCAGTACAGAGATACCTCAAAGCCT
TCATTATTCCCACCTGCGTCCCATAAAACACTCCTCTTCCAAAACCGCTATAATGTTATCCATCAACGTCTCCTTCGCAATGAATCTTTTCAAA
CGCCAGCTTTTCAAGGTGGCAAAGCTTCCCTTCAACGCAGCACGTCCGCTATTACCACCCAACAACAATCATACAAATTAACGCCGATAGCTAA
TCTTCTCGGCCGCAATCGCAGCTCTCATATGCTTCTTGGCCTCCTCAGTATTTCGCCTACTGGTACCCTCGCCATTAATGACCTG

>Bcin11g01310(MLST5), partial sequence [organism=Botrytis sinoviticola, strain GB5_5]
TCATGTGGAACCGGCGTAGAATGCGCAACGCTTGAAGTTCCGCTCGAATATGGCGATGCGAAGTCAACAGCAAAAGCCAGTGTCGCGCTTGCTC
GTTATCCTGCCACTGTTGCCGCGAGCAAGAAGCTCGGGTCTCTCTTGATAAATCCCGGTGGACCCGGTGCCAGTGGTGTTGGCTTTGTGCAGTC
TGGAGCCGGTGCCGCGGTGTCAACACTGAGTGGTGGATTATACGATATCATCGGATGGGATCCACGTGGAACTGGTGCTTCGGCTCCTATTTTA
GAATGCTTTCCAAATGCCAGTGCGGAGTATAATTTTAACAACGCATTTCCATCTGCTCCGAACCTCTGGCTCGGACAATTTGCGAATGCCAGCG
CCAATTCCGCTGTTAGCTCTGCTATCACATCTTTTGACACTTCTGTCGCTGCTCTTGCAAAAGCTTGTGTAGCTCAGAAATCTCCCGCTCTTTA
CACATCAACAGCAGCATATGTTGCTCGAGACATGGCAGCGATAGTTGATGCATTGGATGGAACCTCTGCAAAACTCAACTACTGGGGTTTCTCA
TATGGAACTATTTTCCTTGCCGAGTTTATCCAAACTTTCCCAGGCCGCGTGGGAAGAGTTCTTGCCGATGGTGTTTTTGACGCAAAGGCAAATG
CCCTTACATACGTCAGCCAACTTCCAAACGATCAACTCAGTGTTCGTGCTTCATTGAATGATTTTTCAGCTTTCTGCACCACCGCCGGCAGTAA
AGGTTGCTCTTTTGCCACCGCTCCTGCTGGAATCACAGGTACTGTTGCCACCAGACTTGACAACATAATGAAGGATAT

>Bcin15g03910(MLST6), partial sequence [organism=Botrytis sinoviticola, strain GB5_5]
TCTTTGAAAGCCTTTTCTAGCATCTCCTCCCCCTAACGTCATATGACCCCCTGGAATGGTTTCGAAAACTAATTTTCCGGCCTCATCCAATGTC
TTCAGACCTAGCCAATCTTCTTTATAAATGGCTCTTTCTTTCAGTGGTGTAACTTCCGTGCCGTTGACTTCAGCCCACCATCCACTTTCCTTAG
GAATGACGGTTGTGTCGTCTTCAAAGACGTACATTACGAATCGTTCCAATTTTTCCATGTTGGACTTATATGTTTCGTTCTTGAGAACGCGCTC
GTTATTAATGTCGGCGAGGAAATTGGAATATTCAAGGTAAGAGTCTAGGTTTTCAGGATCTCTGAAGTATTGAGCGGGTACGAGACGAGATTGG
ACAAATGTTGACCAGGTGTTGGATCGCAAAAGGGTTTGAGCACCGCGACAGAGGAAATCGGTAGGACCACAGGCTTGGAAGGCAGAAATACCGT
TGTGTTGGGAACCGAAGGTCAGGAGAGAGCGGATGGGTGGAGAATTGCAGCGGGATATGTAACCGCGCAAGAATTGGCCGCCTTGGGAGAATCC
AATTGCATCGACGGCGGGCGCGGTAGAAAGGATAGGATGGGAGGCGAGGTCTTCGCAGACCTTTTCGATTTGGACTTGGTAAGAGAGTTAGTAT
GTCGTTTGAAGGAAAGCTAGGGGATGTACTCACGAGTGAGATTTCCGAAGAAGGTAGCTGTCCTATCTGCAGATGCATCCTCATCTACATGAAT
ATTATAGACGAAAGTCCCGGGATGAATAGCTTCAGCTAATTCTCCAACTTGCGCAAGACCATCCGCTTTGTAATTATCGCCGAGTCCATGCCAG
ATAATCAAGGGTAGTGGAGTATCA

>Bcin16g03460(MLST7), partial sequence [organism=Botrytis sinoviticola, strain GB5_5]
TCTTCATGAACCTCTTAATTTGAACTCTTCATTAATTGCAAGTGATGAACCCCTATTTTGCCCAGAAGATAGTTACAAGACGTATATCATTAGT
CGAGAACCACTCATGATATATATTGAAGGGTTTTTGAAAGCGAATGAAAGCAAACATTTGGTTGGTGTTAGGTATGTTATTTATTCTGGTGAAA
TGAACAGAAGAAACTGATAAGAAAGTGAACCGCTTTATGAACCGTCTACTGTTTCTCATGGACAGGAAGTTACTATTGATACTTCGGTTCGACA
TTCTGAAGTAGCGGTGTTAGAGAGGGATGAGGTGGTCAGGTGTATTGAGCATAGAGCGAGGGCATTTCAGGGGTGGAGGGGTGAGATGGGGATT
GAGAAGTTGAGGACGCAGAGGTATGGGGTTGGAGGACATTATGGGATGCATTTGTAAGTCTTGGGAGATTGACGAAGTCTTTTGCTTATTTTCT
ATCAACTCGCATCTTTACAAAGAAAAATCATGAATACGAGGGCTAATGTCTAGGAATTAGCGATTGGAGCGGAGGCAAACGTGGCATAGACCGA
TTCAGTACTTTCATGGTCTATGTCGACGTATCCTCTGATATCGAAGGTGGAGGAACGGAATTCCCACGTATTGTGGGACCAAAAGGAGGAAAGT
GGGAGGAATTCCTGGATACTACCGAAGAATTAGATCCAAGAACTGGAAAAAATGTAACAGTTGAAGGGGTGACATTCAAACCTATCAAGGGAAA
TGCCGTATTCT

>Bcin12g03020(MLST8), partial sequence [organism=Botrytis sinoviticola, strain GB5_5]
AAGCACGAATAAACCATCGGCAAGAGAAATATTCTCTGAACCACAACATGACACGAGCGCGGAAGAGTATATCGGGCGAGGAGCTTCATCCAGA
GCACCAAAGCGACAACGAGTCGATGATAATTACAACTCTTACGGTGGAAGAAATGAAAATACAGCAGCTTATGCTTCCGGAAAGCTTCCATCTG
GAAGTATAAATGTTGGTGGGGGCAGGAAGACCACTTTTCAAGAAGAACCTAGAACGGCATTTGTCGCTGGCAAGTTGCCCCCTGGAAGCATCAA
CATCGGTGGAAAGAAGGCTACCCAAATCGAGGACGAGGGTAGAGCGGCTTATGCTTCCAGAAAATTGCCCCCAGGAAGTATAAACTCTGGCGCA
AAGAAGGCCACTTCATTCCAGGATGAAACGAGACCGGCCTATGTCTCGGGAAAGCTCCCACATGGTAGTATCGACGGTATGCGAAACCGTGAAA
TGGCTGCTGTCCATCGCGAAATTGCTGAGGGTGGGAGGAAGCCAGGCCAGGTTGTCTCGTCTCTATTCACATTCAATCCCACTTCAAAGAAAAC
TTTCGATGAACCTGAAGAACAAGCCGAACCGACAAAACCATCCAATGCGCCTTTAACAGAGGAAATGGCAACATTCACCAATTTAGGATTATCG
AGAAGGCTTGCAGCCCATCTATCGACTAAACTCGATATGAAAGCTCCAACCGCCATTCAAAAAGCATCTGTGCAGCAGTTGATATCGGACGATA
GCGATGCTTTCATACAAGCAGAGACTGGATCTGGAAAAACCTTAGCATATCTACTACCTATAGTTGAGCGAATATTAGCATTGAGTGAGA

>Bcin02g07770(MLST9), partial sequence [organism=Botrytis sinoviticola, strain GB5_5]
CGATTCCGATGCCGTGTAGGATTTGCCGAAACTGTTCCCAGTGGGACGGTAGGAACAGTTTATGAGGCATATAACCATTCTTCAAAGTCGTAAA
TGGATGCGTACCATTCCCTGCCGTCGATGCATCGGGTAACACAGGGTATGTCCCTTATATCTTTCTCTTCCACACGATTGCTACCGAGTCTCTA

ACATATTTTAGTGGTGGTTTGTCACCAACTGGCAGTAGCAACGGTGGTTGCAGCAGCAGTACCGGTCAAGTATATGTCAGAGGAGCACAAAGCG
GATCAAACTACGCTATCATGTACTCCTGGTAAGTTCTCTCTAAACTTCTCCTTATAAATCCAATCTAACAAAATCTTAGGTACATGCCAAAAGA
CGAGCCCTCAACCGGTATTGGTCACCGTCACGATTGGGAAGGTGTAATCGTCTGGCTCTCCAGCGCCACCGCCACGACTGCCGACAATATCTTA
GCTGTGTGTCCCTCCGCCCACGGAGGCTGGGATTGTTCCACCGATGGTTATTCCCTTTCTGGTACTAGCCCTCTTATCAAGTACGAGAGTGTCT
GGCCCGTCGATCATTCAATGGGTCTCACTAGTACTGTTGGTGGACAACAACCTATGATTGCTTGGGAGTCTTTACCTACTGCTGCTCAAACTGC
TCTTGAGAACACTGATTTCGGTGCTGCGAATGTTCCATTCATTCCAGCTGTTTTCACAGACAATCTTGCAAAGGCTACTTTTTAG

>Bcin04g02090(MLST10), partial sequence [organism=Botrytis sinoviticola, strain GB5_5]
ACTCTCGAACATGACTATGAATGATGTTTACAAGCCCTACATCCATGTAAGAAATGTAGAATGGAGATATCAGTAACTGTAACTAATGTCGTTC
GTAGGCTTTCAAATTACTTACGCAGTTCAACCCAATCACTACAGCTATTGCCGAATCCCCACTATTTCAAATGGCTGTCTCAGCAAATACCATC
GAAAAGTACACACTGCTAGGCCCTTTCTTCAGAATATCTCCTCTGCAACAGGAAGTTACCAGGGAATACTTCAGTGCACCAAAGACGATAGATA
GGCGACATATTGCCACATCTCAAGATGCTTTACGATTGACCTTACAAACCCATCAAAAAGATTTACTTGATATCATCAACCACTTTGTTCGAGC
AAGTCCAATTGCCAAAAGCAAAACCTTGGATTGGTTCGCCTACATTGTGAATCAAAATCACAAGCGCCGAGCACTTCAGGTAGACCCGAAAGAA
GTATCTTCTGATGGTTTCATGCATAATGTCACTGTCGTTCTAGATGGTCTTTGTGAGCCATTCATGGATACCACATTCTCAAAGATTTCGAAGA
TTGATATTGATTATCTAAGACGTGCGCCTCGTGTAGATATCAAGGACGAGACCAAGCTGAACGCTGATGAGAAGGCTTCCGAGAAGTATTATGA
GGACACTGTTCCTGGCACTTCTAATTTCATCTCTGAGGTCTTCTTTCTGACATTGGCTGCTCATCATTATGGTAGTGAAGCTCTTAATGCCACG
CATAAGAGTCTGGAGAAAGACATCAAATATATTCAAAAGCAATTGACTGCCGTTGAAGCAG
